# Transcriptome profiling of osteoclast subsets associated with arthritis: A pathogenic role of CCR2^hi^ osteoclast progenitors

**DOI:** 10.3389/fimmu.2022.994035

**Published:** 2022-12-15

**Authors:** Maša Filipović, Darja Flegar, Sara Aničić, Dino Šisl, Tomislav Kelava, Nataša Kovačić, Alan Šućur, Danka Grčević

**Affiliations:** ^1^ Department of Physiology and Immunology, University of Zagreb School of Medicine, Zagreb, Croatia; ^2^ Laboratory for Molecular Immunology, Croatian Institute for Brain Research, University of Zagreb School of Medicine, Zagreb, Croatia; ^3^ Department of Anatomy, University of Zagreb School of Medicine, Zagreb, Croatia

**Keywords:** RNA sequencing, mouse arthritis, chemokine receptor, osteoclast differentiation, osteoclast progenitor (OCP), gene expression, qPCR, flow cytometry

## Abstract

**Introduction:**

The existence of different osteoclast progenitor (OCP) subsets has been confirmed by numerous studies. However, pathological inflammation-induced osteoclastogenesis remains incompletely understood. Detailed characterization of OCP subsets may elucidate the pathophysiology of increased osteoclast activity causing periarticular and systemic bone resorption in arthritis. In our study, we rely on previously defined OCP subsets categorized by the level of CCR2 expression as circulatory-like committed CCR2^hi^ OCPs, which are substantially expanded in arthritis, and marrow-resident CCR2^lo^ OCPs of immature phenotype and behavior.

**Methods:**

In order to perform transcriptome characterization of those subsets in the context of collagen-induced arthritis (CIA), we sorted CCR2^hi^ and CCR2^lo^ periarticular bone marrow OCPs of control and arthritic mice, and performed next-generation RNA sequencing (n=4 for each group) to evaluate the differential gene expression profile using gene set enrichment analysis with further validation.

**Results:**

A disparity between CCR2^hi^ and CCR2^lo^ subset transcriptomes (863 genes) was detected, with the enrichment of pathways for osteoclast differentiation, chemokine and NOD-like receptor signaling in the CCR2^hi^ OCP subset, and ribosome biogenesis in eukaryotes and ribosome pathways in the CCR2^lo^ OCP subset. The effect of intervention (CIA) within each subset was greater in CCR2^hi^ (92 genes) than in CCR2^lo^ (43 genes) OCPs. Genes associated with the osteoclastogenic pathway (*Fcgr1*, *Socs3*), and several genes involved in cell adhesion and migration (*F11r*, *Cd38*, *Lrg1*) identified the CCR2^hi^ subset and distinguish CIA from control group, as validated by qPCR (n=6 for control mice, n=9 for CIA mice). The latter gene set showed a significant positive correlation with arthritis clinical score and frequency of CCR2^hi^ OCPs. Protein-level validation by flow cytometry showed increased proportion of OCPs expressing F11r/CD321, CD38 and Lrg1 in CIA, indicating that they could be used as disease markers. Moreover, osteoclast pathway-identifying genes remained similarly expressed (*Fcgr1*) or even induced by several fold (*Socs3*) in preosteoclasts differentiated *in vitro* from CIA mice compared to pre-cultured levels, suggesting their importance for enhanced osteoclastogenesis of the CCR2^hi^ OCPs in arthritis.

**Conclusion:**

Our approach detected differentially expressed genes that could identify distinct subset of OCPs associated with arthritis as well as indicate possible therapeutic targets aimed to modulate osteoclast activity.

## Introduction

Osteoclasts are exclusive bone resorbing cells of hematopoietic origin (1–5). The current concept suggests that they arise from two sources of progenitors, the embryonic yolk sac-derived erythromyeloid extramedullary progenitors and hematopoietic stem cell-derived myeloid progenitors. A common myeloid trilineage progenitor for human and murine osteoclasts, with the ability to differentiate into osteoclasts, macrophages and dendritic cells, has been well characterized by several groups (6–12). Recent advances through lineage tracing, RNA sequencing (RNA-seq) and intravital imaging confirmed the existence of multiple osteoclast progenitor (OCP) subsets with high plasticity and the capacity to repopulate bone surfaces from the circulating pool (9, 10, 13–16). Moreover, it has been suggested that multinuclear bone resorbing osteoclasts may undergo recycling through cell fission, forming osteomorphs (as an alternate cell fate to apoptosis), which are released into the circulation as primed OCPs (15). These circulatory progenitors are susceptible to chemoattraction to bone surfaces by chemokine signals, where they may eventually re-fuse to form active osteoclasts. In particular, the inflammatory environment in arthritis is highly supportive for peripheral OCP attraction (17, 18).

Notable osteoclast activity in places distant from the hematopoietically active bone marrow suggests the existence of medullary and extramedullary reservoirs of OCPs in arthritis. The amplified osteoclast activity is triggered by production of proinflammatory cytokines and autoantibodies as well as enhanced differentiation from their increasingly recruited progenitors (17, 19, 20). Augmented osteoclast function and excessive bone resorption in rheumatoid arthritis (RA) result in articular bone erosions, periarticular osteopenia and generalized bone loss in the axial and appendicular skeleton, leading to irreversible joint damage if not treated promptly (21, 22).

Pathological inflammation-induced osteoclastogenesis within the bone marrow compartment and at extramedullary sites remains poorly understood. Transcriptome profiling is a useful technique to precisely characterize actively expressed genes and transcripts under various conditions in a defined cell or population (23, 24). In the case of OCPs, this approach may identify differentially expressed genes associated with distinct arthritis-induced OCP subsets as well as indicate possible therapeutic targets to modulate progenitor migration, proliferation, differentiation or activity. Moreover, OCPs are a frequent focus of therapeutic anti-resorptive interventions to ameliorate bone loss in postmenopausal osteoporosis, inflammation and malignancy (2). However, recent clinical research still suggests shortcomings of existing therapies to suppress osteoclast activity, suggesting that the biology of OCPs is not fully understood.

In our study, we rely on previously defined OCP subsets categorized by the level of CCR2 expression (9). Circulatory-like OCPs, which exhibit a high expression of the chemokine receptor CCR2 (hereafter abbreviated as CCR2^hi^), are substantially expanded in arthritis and present a significant source of highly osteoclastogenic cells attracted by the inflammatory environment to the affected bone. Compared to resident bone marrow OCPs with a low expression of CCR2 (hereafter abbreviated as CCR2^lo^), they are less proliferative, with a more mature phenotype and a potent differentiation activity (9). CCL2 plays a role in immune cell infiltration of tissues during immune-mediated inflammatory diseases and can act in an autocrine manner on osteoclast lineage cells (25–27). We previously showed that preosteoclasts differentiated *in vitro* from CCR2^hi^ OCPs of mice with collagen-induced arthritis (CIA) express high level of CCL2, whereas preventive blockade of CCR2/CCL2 axis decreases osteoclast activity *in vivo* and *in vitro*, contributing to reduced bone resorption (9). Therefore, we sorted CCR2^hi^ and CCR2^lo^ OCP subsets of control (CTRL) and arthritic (CIA) mice, and performed next-generation RNA-seq to evaluate the differential gene expression (DGE) profile. Our results confirmed a disparity between CCR2^hi^ and CCR2^lo^ OCP subsets, and identified enrichment of pathways for osteoclast differentiation, chemokine and NOD-like receptor signaling in the CCR2^hi^, and ribosome biogenesis in eukaryotes and ribosome pathways in the CCR2^lo^ OCP subset. Genes associated with the osteoclastogenic pathway (*Fcgr1, Socs3*), and several genes involved in cell adhesion and migration (*F11r*, *Cd38*, *Lrg1*) could be used to both identify the CCR2^hi^ subset and distinguish CIA from CTRL group. Osteoclast pathway-identifying genes remained similarly expressed (*Fcgr1*) or even induced by several fold (*Socs3*) in differentiating preosteoclasts from CIA mice compared to pre-cultured levels. Protein-level validation by flow cytometry showed increased proportion of OCPs expressing F11r/CD321, CD38 and Lrg1 in CIA, indicating that they could be used as disease markers.

## Materials and methods

### Mice

For the CIA model, we used male C57BL/6 (B6; 10 to 12 weeks old) mice. Mice were maintained at the animal facility of the Croatian Institute for Brain Research, University of Zagreb School of Medicine (Zagreb, Croatia) under standard housing conditions. All the animal experiments in this study were conducted under protocols approved by the national Ethics Committee (EP 182/2018). Relevant guidelines and regulations of use of laboratory animals (EU Directive 2010/63/EU for animal experiments, the National Institutes of Health guide for the care and use of Laboratory animals) and ARRIVE (Animal Research: Reporting in Vivo Experiments) guidelines for reporting animal research (28) were followed.

### Arthritis induction and visual scoring

For CIA induction we used a previously described modified protocol (9, 29, 30). Chicken collagen type II (CII, Sigma-Aldrich, Saint Louis, MO, USA) was prepared as a 4 mg/mL solution in 0.01 M acetic acid and emulsified 1:1 with Freund’s complete adjuvant (CFA, BD Biosciences, San Jose, CA, USA) containing 4 mg/mL of heat-killed *Mycobacterium tuberculosis*, strain H37RA (BD Biosciences). Experimental (CIA) mice were immunized by an intradermal injection (at the base of the tail) of 100 µL of the emulsion, whereas age- and sex-matched CTRL mice were handled in a same manner without treatment. After 21 days, the mice received the same volume of emulsion containing Freund’s incomplete adjuvant (IFA) instead of CFA. Mice were examined in 2-day intervals and clinical signs of arthritis were scored in each paw as previously described (31): 0 = unchanged, 1 = swelling and/or redness limited to one finger/toe, 2 = swelling and/or redness of more than one finger/toe, or slight paw swelling, 3 = moderate paw swelling and redness, 4 = severe paw swelling and redness with ankylosis; with the maximum clinical score of 16 per mouse.

### Flow cytometry and cell sorting

Mice were sacrificed 33-35 days after primary immunization. Single cell suspensions were prepared from bone marrow of distal tibia adjacent to the tibiotalar joint (periarticular bone marrow) by flushing bones with staining medium (phosphate buffered saline containing 2% heat-inactivated fetal bovine serum (PBS/2% FBS); Gibco, Thermo Fisher Scientific). Cells were labeled with commercially available monoclonal antibodies for phenotyping and cell sorting, incubated for 30 minutes at 4°C in the dark and washed with staining medium. For labeling we used a mixture of monoclonal antibodies (detailed in **Supplementary Table 1**) to lymphoid markers (anti-B220, anti-CD3e, anti-NK1.1), myeloid markers (anti-CD11b, anti-Ly6G, anti-CD115, anti-Fcgr1/CD64), pan-leukocyte marker (anti-CD45), chemokine receptor (anti-CCR2), hematopoietic progenitor marker (anti-Kit/CD117) and cell adhesion molecules (anti-F11r/CD321, anti-CD38, anti-Lrg1). Since Lrg1 is mainly secreted by producing cells, we used intracellular staining for Lrg1 detection; cells were first stained for the expression of surface markers, then fixed and permeabilized by Intracellular Staining Buffer Set (eBiosciences San Diego, CA, USA) according to the manufacturers’ instructions and stained with anti-Lrg1 antibody. We used 0.1 μg/mL 4’,6-diamidino-2-phenylindole (DAPI, Sigma) staining to exclude dead cells. Stained cells were acquired on BD FACSAria II (BD Biosciences) instrument, and the data was analyzed using FlowJo software (TreeStar, Ashland, OR, USA).

Sorting of CCR2^hi^ OCPs (CD45^+^Ly6G^−^CD3^−^B220^−^NK1.1^−^CD11b^–/lo^CD115^+^CCR2^hi^) and CCR2^lo^ OCPs (CD45^+^Ly6G^−^CD3^−^B220^−^NK1.1^−^CD11b^–/lo^CD115^+^CCR2^lo^) from periarticular bone marrow was performed on the BD FACSAria II instrument, using gating strategies as described previously (9, 32). Gates were set according to unstained, fluorescence minus one and/or isotype controls. For RNA-seq, cells were collected in PCR-clean tubes containing PBS/2% FBS. For subsequent cell culture, cells were collected in tubes containing α-minimum essential medium (α-MEM, Capricorn MEMA-RXA) with 10% FBS. In all experiments, cells were kept cooled to 5°C while being sorted. Sorting purity, verified by reanalyzing sorted cells, was greater than 99.5% for all experiments.

### Osteoclastogenic cultures

Sorted OCPs were plated into 96-well plates at a density of 6×10^3^ cells/well for RNA extraction (culture day 2) and tartrate-resistant acid phosphatase (TRAP) stain (culture day 3-5), as optimized in the previous study (9). Cells were cultured in 0.2 mL/well of α-MEM/10% FBS supplemented with 30 ng/mL recombinant mouse (rm) macrophage colony-stimulating factor (M-CSF) and 30 ng/mL rm receptor activator of nuclear factor κB ligand (RANKL) (both from R&D Systems, NE Minneapolis, MN, USA). Total RNA from cultured cells (6-8 wells per sample) was extracted using TRIzol reagent (Applied Biosystems, Thermo Fisher Scientific, Waltham, MA, USA) according to the manufacturer’s instructions. TRAP stain (Leukocyte Acid Phosphatase Kit; Sigma-Aldrich) was performed upon cell fixation in 4% paraformaldehyde in PBS, according to the manufacturer’s instructions. Osteoclasts were identified by Axiovert 200 light microscope (Carl Zeiss Microscopy, Jena, Germany), as TRAP^+^ multinucleated cells (cells with more than three nuclei).

### RNA isolation

Periarticular bone marrow was harvested from CIA mice showing clinical signs of arthritis in the disease score range 6/16 to 16/16. In parallel, age- and sex-matched untreated CTRL mice were used for comparison. OCP subsets were sorted based on the level of CCR2 expression (day 0). Total of 4 CTRL samples (each pooled from 3-4 CTRL mice to yield similar number of cells as in a single CIA mouse) and 4 CIA samples (individual mouse) were prepared (each divided into CCR2^lo^ and CCR2^hi^ subsets).

For subsequent RNA-seq, total cell RNA was isolated from 5.5×10^4^ – 1.9×10^5^ sorted cells for each sample using *Quick*-RNA Microprep Kit (Zymo Research, Irvine, CA, USA) according to the manufacturer’s instructions. Briefly, RNA was extracted in lysis buffer and purified using column technology with included DNase I treatment. RNA quantity was determined using a Qubit 3.0 Fluorometer (Thermo Fisher Scientific). Average RNA yield was approximately 20-30 ng/10^4^ sorted cells. RNA quality was assessed using the Eukaryote Total RNA Nano Assay and 2100 Bioanalyzer system (Agilent, Santa Clara, CA, USA). For all samples, RNA integrity number (RIN) was above 8.

### Library preparation and RNA sequencing

The NeoPrep Library System and a TruSeq Stranded mRNA Library Prep Kit (Illumina, NP-202–1001) were used to prepare libraries from 50 ng of total RNA. Collected libraries were analyzed on a 2100 Bioanalyzer, diluted to 1.4 pM and sequenced on an Illumina NextSeq 500 System using NextSeq 500/550 High-Output v2 Kit, with 75 cycles (Illumina, FC-404-2005). Run setup, direct data streaming, demultiplexing and analysis were performed at the BaseSpace Sequence Hub (Illumina) using the RNA Express BaseSpace App with default analysis parameters.

### Bioinformatic analysis

Quality of FASTQ files was assessed using FastQC v0.11.9^1^. In order to filter the raw reads, quality trimming and adapter clipping were performed by fastp v0.20.0 (33), with a sliding window approach cutting options for front and tail, default window size of 4, and default mean quality of 20. The minimum length was 15 bp and the maximal number of N base was 5. PloyX trimming in 3’ ends was enabled. Ribosomal RNA (rRNA) was removed with SortMeRNA v2.1b (34). The 2.1b version was used in order to avoid repeated indexing of the databases. Trimmed and rRNA-cleaned reads were then mapped using HISAT2 v2.1.0 with default parameters (35). Reads were aligned to the reference mouse genome from Ensembl (genome *Mus_musculus*. GRCm38.99, annotation *Mus_musculus*. GRCm38.dna.primary_assembly).

The output was piped into Samtools (36), which was used to create, index and merge BAM files of reads from different lanes belonging to individual samples. The mapped reads based on this processed annotation was quantified using featureCounts v2.0.1 (37). Non-multi-mapped reads on the exon level were counted. TPM (Transcript Per Million) normalization and filter step was performed, as proposed (38). For each gene, the TPM value was calculated, followed by the calculation of the mean TPM in each condition (subset or intervention). A gene needed to have at least 2 TPM to be considered further. Filtered count tables were passed to the DESeq2 module v1.28.0 (39) to assess DGE with applying normalization, as well as regularized log (rlog) transformation for DGE analysis (DGEA).

Genes with absolute log_2_ fold change (|logFC|)≥2 (except the volcano plots, set at |logFC|>1) and Benjamini-Hochberg (BH) correction adjusted p value lower than 0.01 were considered significantly changed. The results were visualized with volcano plots (EnhancedVolcano^2^), principal component analysis (PCA) plots, and sample-to-sample heatmaps on genes with highest logFCs, using ReportingTools R package (40).

To obtain possible functional insight from the high number of differentially expressed genes, gene set enrichment (GSE) analysis (GSEA) was performed by WEB-based GEne SeT AnaLysis Toolkit (WebGestalt) (41) for an overrepresentation analysis on all genes of the pathways in the Kyoto Encyclopedia of Genes and Genomes (KEGG) database, with False Discovery Rate (FDR) threshold for pathways set at <0.05. Weighted set cover redundancy reduction was used to find top gene sets while maximizing gene coverage. Additionally, to investigate whether different classification methods generated consistent results, GSEA with gene ontology (GO) terms was performed by piano R package (42), using consensus scoring approach based on multiple methods. Significant gene sets that had median rank 1-10 in at least one directionality class were selected and displayed. As with DGE, downstream pathway analysis was performed using different sample batches to analyze differences in GSE between the OCP subsets (CCR2^hi^ and CCR2^lo^) as well as interventions (CTRL and CIA groups), and the approach was further iterated by running GSEA on either all of the samples or just on samples from a single OCP subset or intervention group.

Large part of this process was automated in a pipeline fashion using RNAflow (43).

### Quantitative PCR gene expression analysis

For quantitative PCR validation, cDNA was reversely transcribed using High Capacity RNA-to-cDNA Kit (Applied Biosystems). The amount of cDNA corresponding to 20 ng of reversely transcribed RNA was amplified by ABI Prism 7500 system (Applied Biosystems), using TaqMan Gene Expression Master mix and commercially available TaqMan Gene Expression Assays (Applied Biosystems) for mouse genes *Irf7* (interferon-regulatory factor 7; Assay ID: Mm00516788_m1), *Itgam* (integrin subunit alpha M or CD11b; Assay ID: Mm00434455_m1), *Fcgr1* (Fc gamma receptor Ia or CD64; Assay ID: Mm00438874_m1), *Socs3* (suppressor of cytokine signaling 3, Assay ID: Mm00545913_s1), *Cd38* (Assay ID: Mm00483143_m1), *Kit* (or CD117; Assay ID: Mm00445212_m1), *F11r* (F11 receptor, junctional adhesion molecule-A (JAM-A) or CD321; Assay ID: Mm00554113_m1), *Lrg1* (leucine rich alpha-2-glycoprotein 1; Assay ID: Mm01278767_m1), *Gnl3* (G protein nucleolar 3; Assay ID: Mm00463571_m1), *Dctd* (deoxycytidylate deaminase; Assay ID: Mm00618904_m1) and *Hmbs* (hydroxymethylbilane synthase; Assay ID: Mm01143545_m1). Gene expression of each target gene was normalized to the expression level of the housekeeping gene *Hmbs*, using cycle threshold (Ct) values. Level of gene expression was presented as fold-difference relative to the calibrator sample (cDNA from sorted CCR2^lo^ subset of CTRL group, day 0) using ΔΔCt method.

### Statistical analysis

The results were statistically analyzed using MedCalc Statistical Software version 13.1.2 (MedCalc Software bvba, Ostend, Belgium). Group size and number of samples for RNA-seq and qPCR analyses are noted within the figure legends. Kolmogorov–Smirnov test was used to verify normality of data distribution. Results are presented as median with interquartile range (IQR) and data are plotted as individual values and box-and-whisker diagrams, where middle horizontal lines represent medians, boxes represent the IQR, whiskers represent 1.5 times the IQR, and squares represent outliers. Differences between groups were analyzed by Mann–Whitney U-test or by the non-parametric Kruskal-Wallis test followed by Conover test for group-to-group comparisons. Correlations were assessed using rank correlation and Spearman’s coefficient rho (ρ). In all experiments α-level was set at 0.05. Bioinformatic analysis of RNA-seq experiment is described above.

## Results

### Increased frequency of CCR2^hi^ and CCR2^lo^ subsets of OCPs in CIA

**Figure 1:**
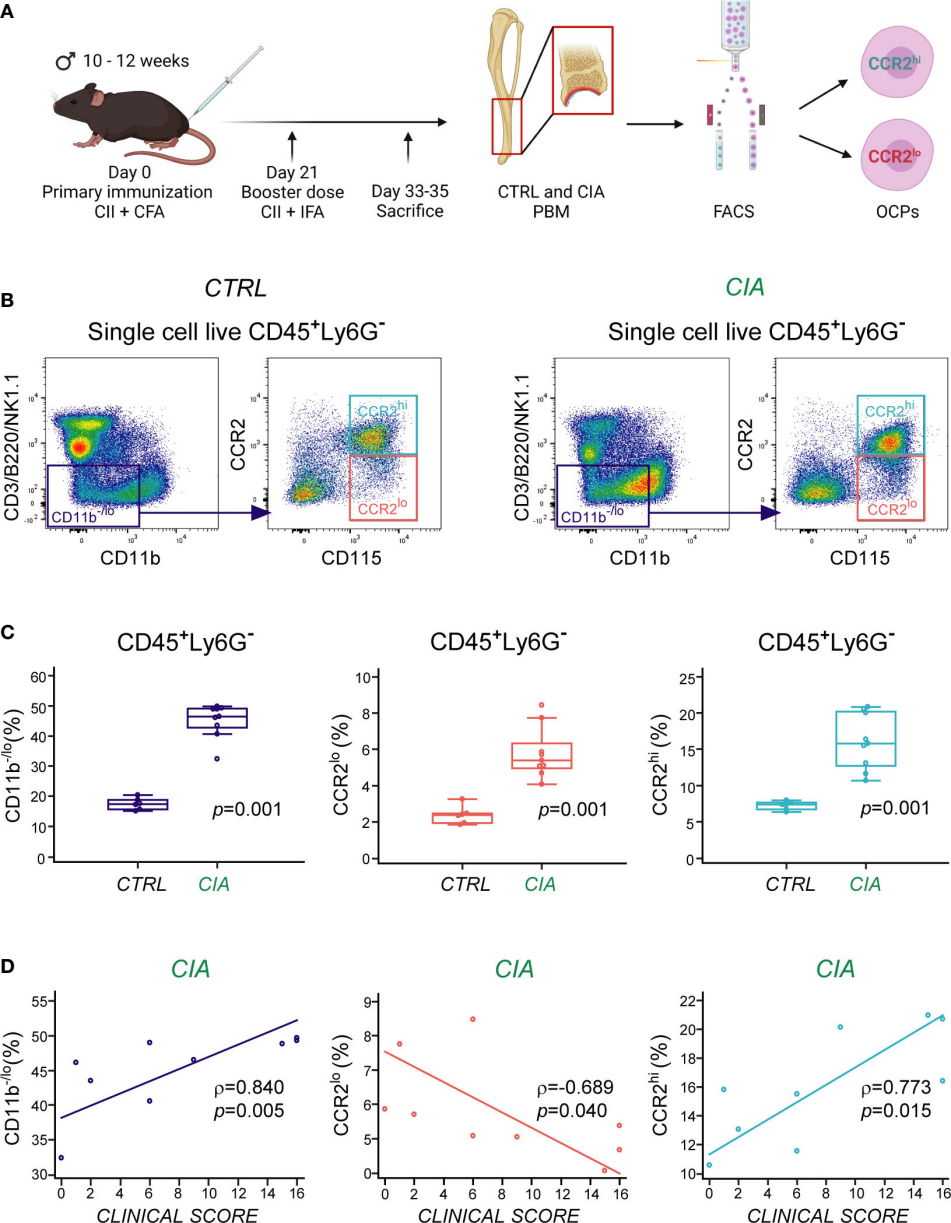
Increased frequency of CCR2^hi^ and CCR2^lo^ subsets of osteoclast progenitors (OCPs) in periarticular bone marrow (PBM) of mice with collagen-induced arthritis (CIA). **(A)** Experimental design of arthritis induction, with subsequent cell phenotyping and sorting by flow cytometry. CIA was induced in B6 mice, whereas the control (CTRL) group was left untreated. OCPs from PBM were identified as CD45^+^Ly6G^−^CD3^−^B220^−^NK1.1^−^CD11b^–/lo^CD115^+^ cells, and further separated based on the expression of chemokine receptor CCR2 (into CCR2^lo^ and CCR2^hi^ subsets). Created with BioRender.com **(B)** Representative dot-plots of CCR2 expression on OCPs from CTRL (*left*) and CIA (*right*) mice. OCPs were gated from non-lymphoid (CD3^−^B220^−^NK1.1^−^) cells with low expression of integrin CD11b (purple rectangle), and further separated to CD115^+^ OCPs with high expression of CCR2 (cyan rectangle) and low expression of CCR2 (red rectangle). **(C)** Frequency of non-lymphoid CD11b^-/lo^ cells and CD115^+^ OCPs (CCR2^lo^ and CCR2^hi^) in CIA compared to CTRL mice (n=6-9). Individual values are presented; horizontal lines represent the median, boxes represent the interquartile range (IQR), and whiskers represent 1.5 times the IQR. Statistically significant difference was determined at p<0.05, Mann–Whitney U-test. **(D)** Association of the frequencies of CD11b^-/lo^ cells and CD115^+^ OCPs (CCR2^lo^ and CCR2^hi^) with the clinical score of arthritis in CIA mice. Individual values and trend lines are presented with the Spearman’s rank correlation coefficient (ρ). Statistically significant difference was determined at p<0.05.

CIA was induced in male B6 mice by a modified protocol as previously described (9), and periarticular bone marrow of distal tibia was collected for flow cytometric analysis and cell sorting 33-35 days following immunization (**Figure 1A**). To identify osteoclastogenic cell subsets in the periarticular area, gating strategy was performed as previously described (9, 32). Bone marrow OCPs were dissected by delineation of live bone marrow single-cells that were further gated for hematopoietic (CD45^+^) agranulocytes (Ly6G^−^), followed by gating of non-lymphoid (CD3^−^B220^−^NK1.1^−^) cells with low expression of CD11b (CD11b^−/lo^) (**Supplementary Figure 1A**). OCPs were identified by the expression of CD115 (M-CSF receptor), and separated based on the level of chemokine receptor CCR2 expression into CCR2^lo^ and CCR2^hi^ OCP subsets (**Figure 1B**). Periarticular area of arthritic mice showed enrichment in hematopoietic cells (approximately 3-4× increase in periarticular hematopoietic absolute cell number per mouse in CIA compared to CTRL, not shown), with a significantly higher percentage of myeloid cells (CD11b^−/lo^), including CD115^+^ OCPs (CCR2^lo^ and CCR2^hi^), among the CD45^+^Ly6G^−^ population (**Figure 1C**). We previously observed that CCR2^hi^ OCPs, contained within the CD11b^−/lo^ population, are specifically associated with arthritis (9). Both of these populations (CD11b^−/lo^ and CCR2^hi^) were significantly enlarged in CIA mice (**Figure 1C**) and positively correlated with the arthritis clinical score (**Figure 1D**). The CCR2^lo^ subset conversely exhibited a negative correlation with the arthritis clinical score (**Figure 1D**).

### Distinctiveness of transcriptome clustering in CCR2^hi^ and CCR2^lo^ OCP subsets

**Figure 2:**
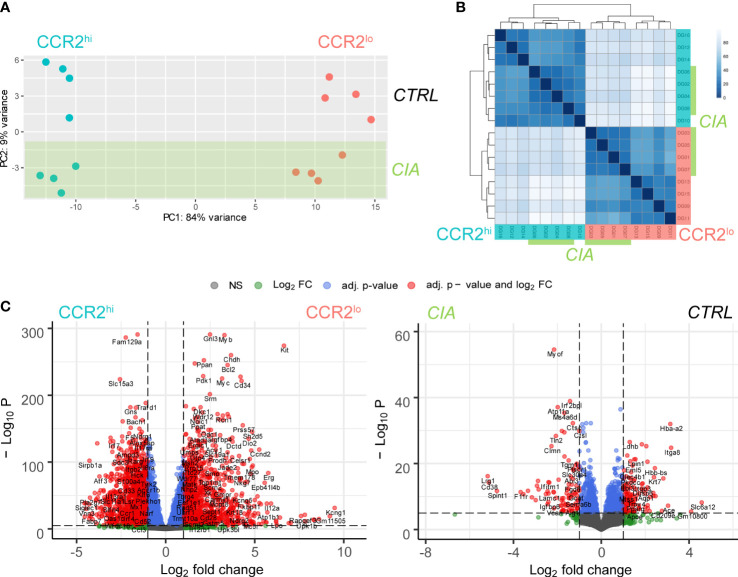
Distinctiveness of transcriptome clustering in CCR2^hi^ and CCR2^lo^ osteoclast progenitor (OCP) subsets. **(A)** Principal component analysis (PCA) performed based on the expression of the top 50 variable genes from RNA sequencing data in all samples. Dots represent individual samples, color denotes the subset: CCR2^hi^ in cyan and CCR2^lo^ in red, while the samples belonging to the intervention group, collagen-induced arthritis (CIA), are shaded in green. **(B)** Sample similarity analysis heatmap where the shade of the color represents similarity in gene expression between samples. Samples labeled 1-8 are derived from CIA mice (marked green), and samples labeled 9-16 from control mice (CTRL). Odd numbered samples represent the CCR2^hi^ subset (cyan), while even numbered samples represent the CCR2^lo^ subset (red) of OCPs. **(C)** Volcano plots showing the comparison of gene expression between CCR2^hi^ and CCR2^lo^ subsets, as well as the comparison between CIA and CTRL groups. Negative logarithm of Benjamini-Hochberg correction adjusted p value (-Log_10_ P) for each gene is shown in relation to the logarithm of fold change (Log_2_ FC) of the gene. Differentially expressed genes are shown as red dots (|logFC|>1, p<0.01).

RNA-seq was performed on CCR2^hi^ and CCR2^lo^ OCP subsets sorted from periarticular bone marrow of both CTRL and CIA mice. As an initial analysis step, sample similarity was assessed using PCA plot and correlation heatmap with hierarchical clustering. Samples clustered closely based on the OCP subset (CCR2^hi^ or CCR2^lo^), delineated along the PC1 axis, which accounted for the majority of variance between the samples (84%), unlike the intervention-based (CTRL or CIA) sample clustering which appeared to have much smaller impact, delineated along PC2 axis (**Figure 2A**). Assessing OCP subsets from only CTRL or only CIA group even further accentuated delineation along the PC1 axis (93 and 95% variance, respectively; **Supplementary Figure 2A**). Interestingly, when comparing intervention (CTRL or CIA) within the same OCP subset, we could observe a distinct impact of CIA, with a resulting delineation along the PC1 axis of roughly 70% compared to CTRL samples of the same OCP subset (**Supplementary Figure 2A**).

By using a heatmap to display the correlation of gene expression for all pairwise combinations of all samples, coupled with a hierarchical tree to indicate sample similarity based on normalized gene expression values, the same trend is obvious, where the CCR2^hi^ OCP subset is clearly distinguished and separated in all samples from the CCR2^lo^ OCP subset (**Figure 2B**). In addition to this strong subset pattern, it was still possible to observe a weaker pattern discriminating CIA from CTRL samples, both by using a heatmap and hierarchical clustering. This, again, coincides with PCA and suggests that CCR2^hi^ and CCR2^lo^ subsets are significantly different by their transcriptomes, with the intervention in the form of CIA having a less pronounced, but still observable impact in each OCP subset. High group uniformity with tight clustering and high correlations suggested there were no significantly outlying samples and that the data was of good quality for further DGEA. The above findings were similarly illustrated by volcano plots (**Figure 2C** and **Supplementary Figure 2B**).

In summary, a total of 15011 genes were found to be expressed analyzing all samples (**Supplementary Data 1**), with significant differential expression of 863 genes between CCR2^hi^ and CCR2^lo^ subsets using criteria of |logFC|≥2 and BH adjusted p<0.01 (384 upregulated, 479 downregulated) (**Supplementary Data 2**). Numbers were similar when analyzing OCP subsets in samples from only CIA or only CTRL group (CTRL: 401 upregulated, 492 downregulated; CIA: 359 upregulated, 412 downregulated) (**Supplementary Data 3**). As with PCA and sample-to-sample correlation heatmap, the intervention had notably less impact on gene expression compared to differences between OCP subsets. There were 68 differentially expressed genes in CIA *vs* CTRL (30 upregulated, 38 downregulated) when comparing all samples with criteria of |logFC|≥2 and BH adjusted p<0.01 (**Supplementary Data 4**). Interestingly, CIA seemed to have more impact on the gene expression in the CCR2^hi^ subset (45 upregulated, 47 downregulated), than in the CCR2^lo^ subset (19 upregulated, 24 downregulated) (**Supplementary Data 5**). In addition, intervention (CTRL or CIA) did not have impact on the expression of CCR2 or CCL2 within each OCP subset (CCR2^hi^ or CCR2^lo^), but the CCR2^hi^ subset had higher expression of both CCR2 and CCL2 compared to the CCR2^lo^ subset (**Supplementary Data 5**).

### Higher expression of osteoclast differentiation, chemokine signaling and NOD-like receptor signaling pathways in CCR2^hi^ OCP subset

**Figure 3:**
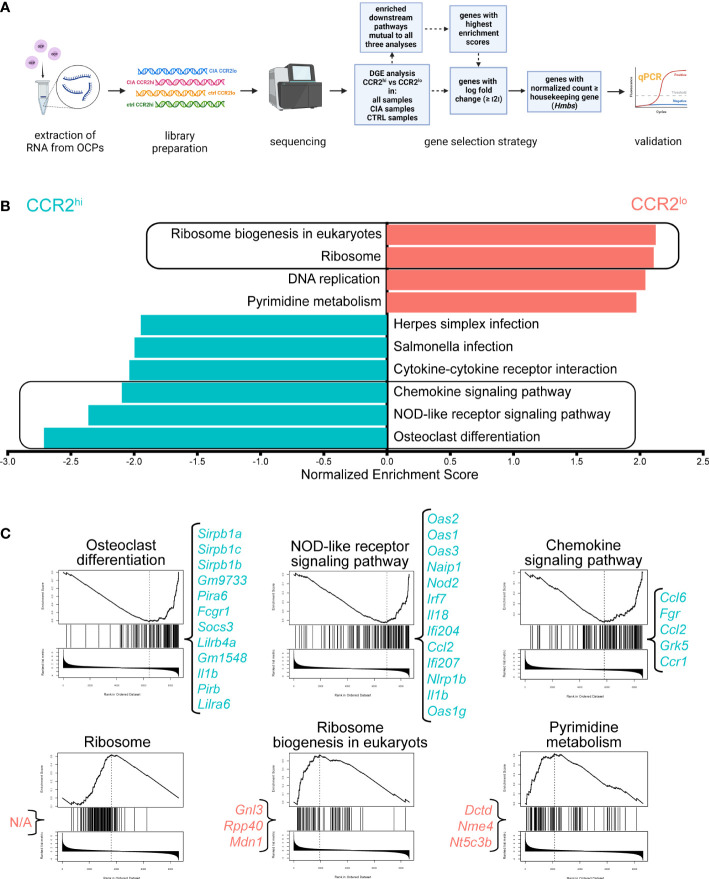
Higher expression of osteoclast differentiation, chemokine signaling and NOD-like receptor signaling pathways in CCR2^hi^ osteoclast progenitor (OCP) subset. **(A)** Experimental design of RNA sequencing. Sorted OCPs from control (CTRL) mice and mice with collagen-induced arthritis (CIA) were subjected to RNA extraction and library preparation. Differential gene expression (DGE) analysis of the OCP subset (CCR2^hi^ *vs* CCR2^lo^) was performed in all samples, only in CIA group, or only in CTRL group, followed by gene set enrichment (GSE) analysis using WebGestalt. The same method was used to compare intervention (CIA *vs* CTRL) in all samples, only in the CCR2^hi^ subset or only in the CCR2^lo^ subset. The criteria for gene selection for qPCR validation is shown in the blue boxes. Created with BioRender.com **(B)** Downstream pathway analysis showing normalized enrichment scores for significantly enriched pathways in CCR2^hi^ (cyan) or CCR2^lo^ (red) OCP subsets in comparison with gene expression in all samples. Redundancy reduction using weighted set cover was applied to minimize the number of pathways while maximizing gene coverage. False discovery rate was set to <0.05. Framed pathways were also enriched when comparing OCP subsets in only CIA or only CTRL group. **(C)** GSE plots for OCP subset-characteristic pathways. The line in the top plot represents the running enrichment score for a given pathway as the analysis goes down the ranked list. The value at the peak denotes the final enrichment score. The middle plot shows where the genes related to the pathway are located in the ranking. Genes that appear before the positive enrichment score, or after the negative enrichment score, represent the leading edge subset. The lower plot shows the distribution of the ranking metric along the list of the ranked genes. Genes in the leading edge with highest enrichment scores that fit the proposed criteria are listed in curly brackets. N/A – non-applicable as none of the genes from this pathway fit the criteria.

To obtain possible functional insight from the high number of differentially expressed genes, GSEA was performed and downstream pathway information was obtained. **Figure 3A** summarizes experimental design of RNA-seq followed by DGEA and GSEA, coupled with gene selection criteria for validation by qPCR.

In general, CCR2^hi^ subset enriched pathways revolved around bone resorption and inflammatory response, while CCR2^lo^ subset enriched pathways tied to cell replication, implying its higher proliferative capacity. As displayed in **Figure 3B**, regardless if analyzing all samples, or samples from only CTRL or only CIA group, five downstream pathways were found to be consistently detected as being significantly enriched (**Supplementary Data 6, 7**). Of those, three were upregulated in CCR2^hi^ OCPs: osteoclast differentiation, NOD-like receptor signaling and chemokine signaling. On the other hand, CCR2^lo^ OCPs had enriched ribosome biogenesis in eukaryotes and ribosome pathways. GSE plots for these OCP subset-characteristic pathways, along with genes with highest enrichment scores, are displayed in **Figure 3C** (and in more detail in **Supplementary Data 6, 7**).

As these five pathways are attribute of OCP subsets and were unaffected by intervention (enriched in both CTRL and CIA), they could be considered key functional pathways of CCR2^hi^ and CCR2^lo^ OCPs, further validating our previous implication regarding characteristics of these OCP subsets (9). Additionally, there were several inflammatory pathways upregulated in CCR2^hi^ OCPs only in CIA: including Staphylococcus aureus infection, Toll-like receptor signaling and TNF signaling (**Supplementary Figure 3A** and **Supplementary Data 7**). Consensus scoring of multiple methods onto GO terms yielded similar results, with CCR2^hi^ GO terms involving both increased reactivity to various mediators of inflammation and their production, as well as cell binding; while CCR2^lo^ GO terms implied cell proliferation (**Supplementary Figure 4** for CCR2^hi^ *vs* CCR2^lo^, **Supplementary Figure 5** for CTRL *vs* CIA).

Since DGEA produced almost nine hundred genes to be different between CCR2^hi^ and CCR2^lo^ subsets despite the stringent criteria of |logFC|≥2 and BH adjusted p<0.01 (**Supplementary Data 2**), it was necessary to employ a systematic filtering approach to narrow down all these potentially important genes to ten final candidates for further analysis and validation (**Figure 3A**). The logic behind a top-down selection approach was to pick genes with several fold changed expression (**Supplementary Data 2–5**) from the above mentioned most significantly enriched pathways (**Supplementary Data 6, 7**), with the final criteria being their level of expression matching or exceeding our chosen housekeeping gene (*Hmbs*) (**Supplementary Data 1**). The goal was to drastically reduce the number of genes to those of very high biological relevance, which could be, subsequently, easily validated by other methods due to high level of expression.

For CCR2^hi^ OCPs, this filtering resulted in four key subset-specific genes (**Supplementary Figure 6A**). From two most enriched pathways three genes were obtained using these criteria: *Fcgr1* (|logFC|=2.59) and *Socs3* (|logFC|=2.65) emerged from osteoclast differentiation pathway, while *Irf7* (|logFC|=2.59) emerged from the NOD-like receptor signaling pathway (**Figure 3C**). Since Staphylococcus aureus infection pathway was specifically upregulated in CCR2^hi^ OCPs of the CIA group, being the second highest enriched pathway (after the osteoclast differentiation pathway), we opted to use the same filtering process from which *Itgam* (|logFC|=2.62) emerged as the fourth signature gene of the subset.

For CCR2^lo^ OCPs, the filtering resulted in two key subset-specific genes. From the most enriched pathway, ribosome biogenesis in eukaryotes, the first obtained gene was *Gnl3* (|logFC|=2.49). Using our filtering criteria, there was no suitable gene from the ribosome pathway, which was the second highest enriched in CCR2^lo^ OCPs (**Figure 3C**). Thus, genes from the pyrimidine metabolism pathway, detected as being enriched in CCR2^lo^ OCPs of the CTRL group, were filtered and *Dctd* (|logFC|=3.77) emerged as the second signature gene of the subset (**Supplementary Figure 6A**).

The same approach for comparing groups based on intervention (CTRL or CIA) yielded different enriched pathways depending on the input OCP subsets (all samples, only CCR2^hi^ or only CCR2^lo^) (**Supplementary Figure 3B** and **Supplementary Data 8**). However, two significantly enriched downstream pathways in CIA, malaria (enriched in CCR2^hi^) and African trypanosomiasis (enriched in CCR2^lo^), did share a set of significantly changed hemoglobin genes (*Hba-a2*, *-bt*, *-b1*, *-bs*, *-a1*). Similar results were also obtained in GO terms by piano consensus scoring (**Supplementary Figure 5**). Regardless, the hemoglobin genes did not match the final selection criteria either due to having |logFC|<2 or due to their normalized gene counts being significantly lower than the *Hmbs* housekeeping gene (**Supplementary Data 1**).

Thus, we decided to choose additional four genes by focusing on the most differentially expressed genes in the proposed pathogenic subset – CCR2^hi^ OCPs. Using the same criteria of |logFC|≥2 and BH adjusted p<0.01, followed by ensuring normalized count of selected gene matched or exceeded *Hmbs* housekeeping gene, we filtered the most differentially expressed genes based on the intervention (CIA *vs* CTRL) only in CCR2^hi^ OCPs (**Supplementary Data 5**) and picked top three upregulated genes: *Lrg1* (|logFC|=5.65), *Cd38* (|logFC|=5.53) and *F11r* (|logFC|=4.02) (**Supplementary Figure 6B**). Finally, *Kit* (|logFC|=-6.86) was selected as the gene with the lowest expression in CCR2^hi^ *vs* CCR2^lo^ OCPs from the CIA group (**Supplementary Figure 6C** and **Supplementary Data 3**).

With this final selection of ten genes – four that are specific for CCR2^hi^ OCPs, two that are specific for CCR2^lo^ OCPs, three with the highest expression in CCR2^hi^ OCPs from CIA *vs* CTRL and one with the lowest expression in CCR2^hi^ *vs* CCR2^lo^ from CIA – we set out for further experimental validation.

### Confirmation of differential gene expression in OCP subsets by qPCR

**Figure 4:**
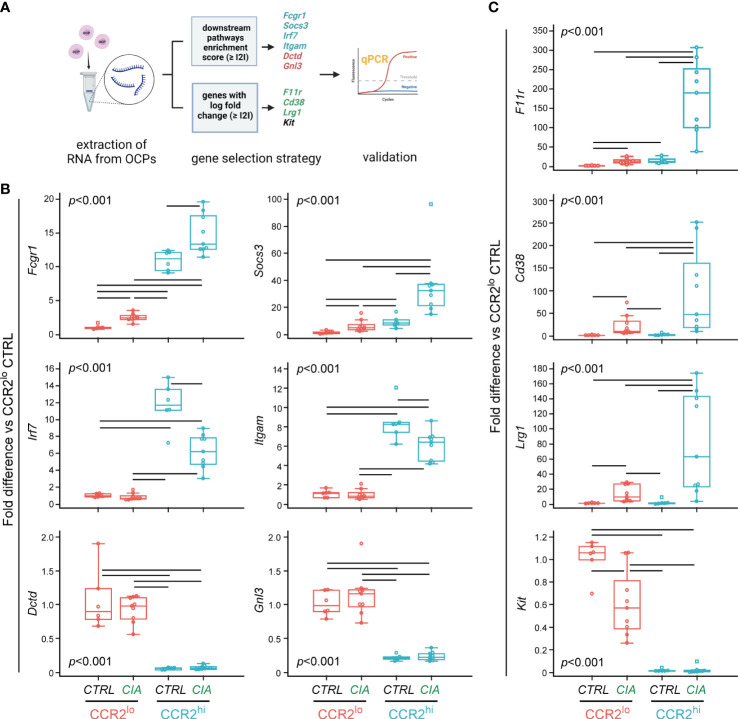
Validation of differentially expressed genes in osteoclast progenitor (OCP) subsets by qPCR. **(A)** Experimental design for qPCR validation of gene expression in sorted OCPs. RNA was extracted from sorted CCR2^lo^ and CCR2^hi^ OCP subsets from control (CTRL) mice and mice with collagen-induced arthritis (CIA), and reversely transcribed to cDNA. Genes were selected based on the enrichment score and based on the log fold change, and validated in CTRL and CIA groups (n=6 for CTRL, n=9 for CIA). Created with BioRender.com **(B)** qPCR analysis of genes within biological pathways significantly different between OCP subsets (CCR2^lo^ and CCR2^hi^), characterized as subset markers. **(C)** qPCR analysis of genes that have the most significant differential expression by intervention (CTRL or CIA), characterized as disease markers. **(B, C)** Gene expression was normalized to the *Hmbs* housekeeping gene and presented as fold difference relative to the calibrator sample (cDNA from CCR2^lo^ subset of CTRL, day 0). Individual values are presented; colored horizontal lines represent the median, boxes represent the interquartile range (IQR), whiskers represent 1.5 times the IQR, and squares represent outliers. Statistically significant difference (represented by black horizontal lines) was determined at p<0.05, Kruskal–Wallis test followed by Conover test for group-to-group comparisons.

Gene expression of the selected ten genes was evaluated in CTRL group (n=6 for each subset, which included remaining RNA samples used for RNA-seq, and two additional samples extracted using the same conditions) and CIA group (n=9 for each subset, which included remaining RNA samples used for RNA-seq, and five additional samples extracted using the same conditions) by qPCR (**Figure 4A**).

Six genes were identified within biological pathways significantly different between OCP subsets (CCR2^hi^ *vs* CCR2^lo^). Expression of *Fcgr1* and *Socs3*, from the osteoclast differentiation pathway, was higher in the CCR2^hi^ compared to the CCR2^lo^ subset, and, in addition, significantly increased in CIA compared to CTRL in both OCP subsets (**Figure 4B**). Next two genes, *Irf7* and *Itgam* are associated with NOD-like receptor signaling pathway and Staphylococcus aureus infection pathway, respectively. Their expression was, again, significantly higher in the CCR2^hi^ compared to the CCR2^lo^ subset, but lower in CIA compared to CTRL within the CCR2^hi^ subset (**Figure 4B**). Finally, *Dctd* and *Gnl3* genes are found within metabolic pathways (pyrimidine metabolism and ribosome biogenesis in eukaryotes, respectively), and were increased in the CCR2^lo^ compared to the CCR2^hi^ subset, with no further effect of CIA (**Figure 4B**).

Three additional genes were used to identify intervention (CIA *vs* CTRL) within the CCR2^hi^ subset: *F11r*, *Cd38* and *Lrg1*. RNA transcripts of these genes were abundant in the CCR2^hi^ OCPs from CIA, reaching several hundred-fold higher expression compared to CCR2^hi^ OCPs from CTRL (**Figure 4C**). These genes were increased with CIA also in the CCR2^lo^ subset, although at a notably lower level of expression. Indeed, CCR2^hi^-specific genes of the osteoclast differentiation pathway (*Fcgr1* and *Socs3*) and CIA-specific genes (*F11r*, *Cd38* and *Lrg1*) followed a similar pattern of expression (**Figures 4B, C**). Finally, *Kit* gene showed a lower expression in the CCR2^hi^ than in the CCR2^lo^ subset with further decrease in CIA compared to CTRL in the latter subset, implying it can be used as CTRL-specific gene.

### Significant correlation of intervention-identifying genes with the disease clinical score and size of CCR2^hi^ OCP subset

**Figure 5:**
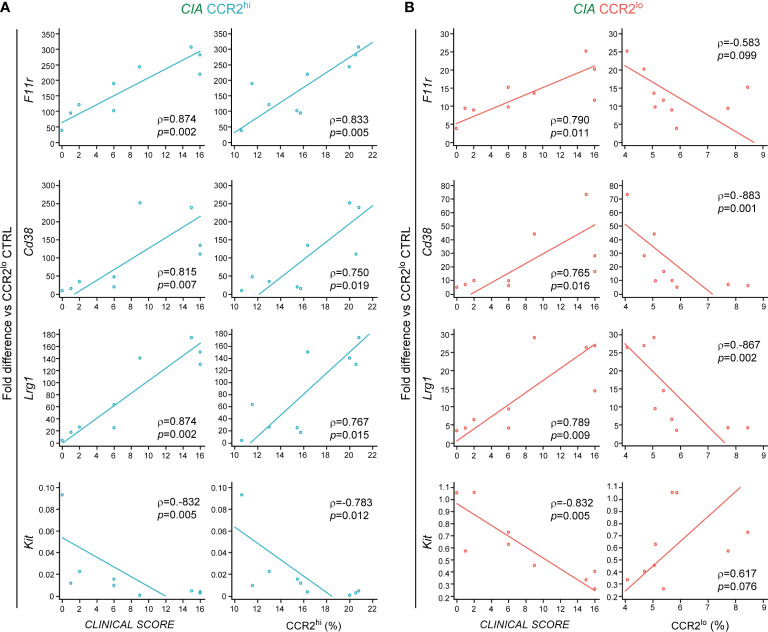
Associations of genes used as disease markers with the clinical score and size of osteoclast progenitor (OCP) subsets in collagen-induced arthritis (CIA). RNA was extracted from sorted CCR2^hi^ and CCR2^lo^ OCP subsets, reversely transcribed to cDNA and validated by qPCR in CIA group (n=9). Gene expression was normalized to the *Hmbs* housekeeping gene and presented as fold difference relative to the calibrator sample (cDNA from CCR2^lo^ subset of the control group, day 0). **(A)** Correlations between relative gene expression and the clinical score of arthritis, and between relative gene expression and frequency of CCR2^hi^ OCPs in CIA. **(B)** Correlations between relative gene expression and the clinical score of arthritis, and between relative gene expression and frequency of CCR2^lo^ OCPs in CIA. Percentages of CCR2^hi^ and CCR2^lo^ subsets are calculated out of CD45^+^Ly6G^-^ population. Individual values and trend lines are presented with the Spearman’s rank correlation coefficient (ρ). Statistically significant difference was determined at p<0.05.

In our previous study we identified the CCR2^hi^ as migratory and more committed subset of OCPs specifically expanded in arthritis (9). Based on the presented results we further concluded that several of selected genes could be used to distinguish the CCR2^hi^ subset from the CCR2^lo^ subset of the CIA group, namely *Fcgr1* and *Socs3* belonging to the osteoclast differentiation pathway, as well as to distinguish CIA from CTRL group within the CCR2^hi^ subset, namely *F11r*, *Cd38* and *Lrg1*, involved in cell migration and adhesion. To confirm the pathogenic role of CCR2^hi^ OCPs, we next explored the association of the gene expression level with the clinical score of arthritis and the size of the CCR2^hi^ subset. Interestingly, expression of *F11r*, *Cd38* and *Lrg1* showed a significant positive correlation with the disease clinical score and the frequency of CCR2^hi^ OCPs (**Figure 5A**). Compared to CCR2^hi^, these genes were 5- to 12-fold less expressed in CCR2^lo^ OCPs, but the difference was still significant for CIA *vs* CTRL. Their expression level in CCR2^lo^ OCPs exhibit a significant positive correlation with the disease clinical score and significant negative correlation with the frequency of CCR2^lo^ OCPs (**Figure 5B**). These findings complement observed association between the size of OCP subsets and disease clinical score presented in **Figure 1**. On the other hand, expression of *Kit* gene was overall higher in the CCR2^lo^ OCP subset, with lower expression in CIA compared to CTRL. Accordingly, the expression level of *Kit* showed a significant negative correlation with the disease clinical score in both subsets, significant negative correlation with the frequency of CCR2^hi^ OCPs and significant positive correlation with the frequency of CCR2^lo^ OCPs (**Figures 5A, B**). We, therefore, concluded that *F11r*, *Cd38* and *Lrg1* could be used as signature genes of the CCR2^hi^ subset in CIA and *Kit* could be used as signature gene of the CCR2^lo^ subset in CTRL. In contrast, the expression of selected pathway-associated genes was not significantly associated with the clinical score of arthritis nor subset size (not shown), possibly indicating that their expression level is more indicative of CCR2^hi^ OCP differentiation potential and less useful as a disease marker.

### Confirmation of differential protein expression in OCP subsets by flow cytometry

**Figure 6:**
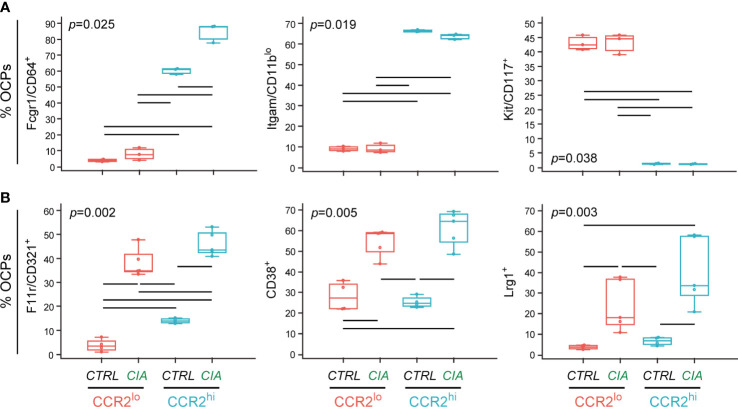
Validation of differentially expressed genes in osteoclast progenitor (OCP) subsets by flow cytometry. OCPs were first identified in control (CTRL) mice and mice with collagen-induced arthritis (CIA) by phenotyping CCR2^hi^ and CCR2^lo^ subsets. Then, the additional markers of OCP subsets and interventions were included into the screening antibody panels for protein-level validation. **(A)** Membrane expression of Fcgr1/CD64, Itgam/CD11b and Kit/CD117, presented as the percentage of immediate parent CD115^+^CCR2^lo^ and immediate parent CD115^+^CCR2^hi^ subsets (n=3 for both CTRL and CIA). **(B)** Membrane expression of F11r/CD321 and CD38, and intracellular expression of Lrg1, presented as the percentage of immediate parent CD115^+^CCR2^lo^ and immediate parent CD115^+^CCR2^hi^ subsets (n=4 for CTRL and n=5 for CIA). Individual values are presented; colored horizontal lines represent the median, boxes represent the interquartile range (IQR) and whiskers represent 1.5 times the IQR. Statistically significant difference (represented by black horizontal lines) was determined at p<0.05, Kruskal–Wallis test followed by Conover test for group-to-group comparisons.

Since RNA-seq and qPCR analyses implicated several genes that could be used either to distinguish OCP subsets or interventions, we further validated the expression of corresponding proteins by flow cytometry (**Figure 6** and **Supplementary Figure 7**).

In the first set of experiments phenotyping was performed in CTRL (n=3) and CIA mice (n=3) for the membrane expression of Fcgr1/CD64, Itgam/CD11b and Kit/CD117 to discriminate OCP subsets (**Figure 6A**). Significantly higher proportion of Fcgr1/CD64^+^ and Itgam/CD11b^lo^ OCPs was observed in the CCR2^hi^ compared to the CCR2^lo^ subset, whereas significantly higher proportion of Kit/CD117^+^ OCPs was observed in the CCR2^lo^ compared to the CCR2^hi^ subset. Additional comparisons between CTRL and CIA groups within the CCR2^hi^ subset indicated that only Fcgr1/CD64^+^ frequency was significantly associated with arthritis (**Figure 6A**).

In the second set of experiments, membrane expression of F11r/CD321 and CD38, and intracellular expression of Lrg1 were assessed in CTRL (n=4) and CIA mice (n=5) to discriminate interventions (**Figure 6B**). Clear difference based on intervention was detected, with these markers being expressed by significantly higher proportion of OCPs in CIA compared to CTRL. In addition, frequency of F11r/CD321^+^ OCPs was significantly higher in the CCR2^hi^ than in the CCR2^lo^ subset of both CTRL and CIA (**Figure 6B**). Besides the size of positive population, we calculated the respective geometric mean fluorescence intensity (MFI) (**Supplementary Figure 7**). Briefly, we can conclude that increased frequency of cells expressing respective marker (% of positive cells) is not accompanied by a significant change in the marker density per cell (MFI value).

### Higher expression of osteoclast differentiation, chemokine and NOD-like receptor signaling pathways in maturing CCR2^hi^ OCPs

**Figure 7:**
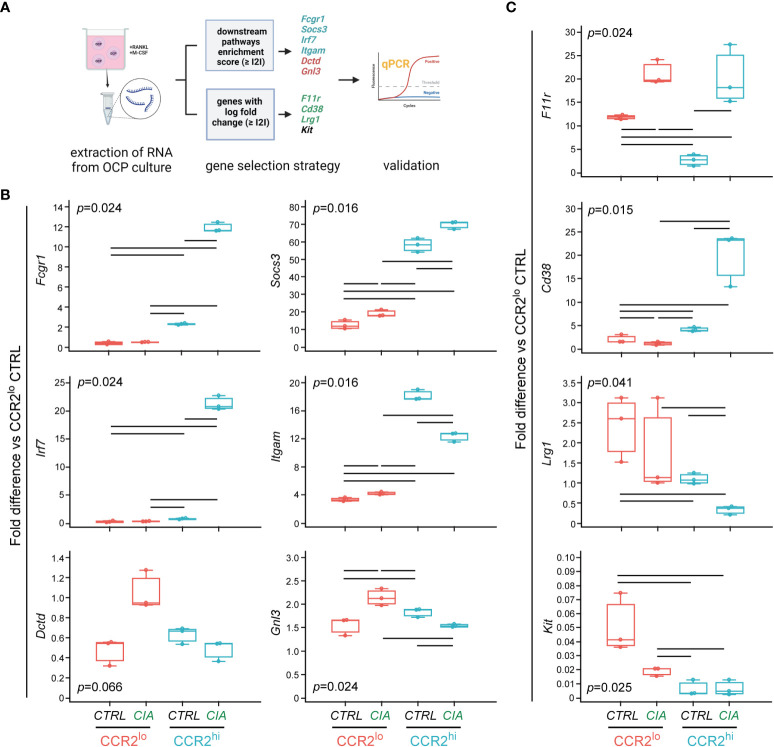
Evaluation of differentially expressed genes in committed preosteoclasts by qPCR. **(A)** Experimental design for qPCR analysis of gene expression in cultured OCPs. Sorted CCR2^lo^ and CCR2^hi^ OCP subsets from control (CTRL) mice and mice with collagen-induced arthritis (CIA) were cultured for two days with RANKL and M-CSF to induce osteoclast differentiation. RNA was extracted from cultured preosteoclasts and reversely transcribed to cDNA. Genes were selected based on the enrichment score and based on the log fold change, and analyzed in CIA and CTRL groups (pooled cells from n=3-5 mice, RNA extracted from n=6-8 wells, data presented as technical replicates n=3). Created with BioRender.com **(B)** qPCR analysis of genes within biological pathways significantly different between OCP subsets (CCR2^lo^ and CCR2^hi^), characterized as subset markers. **(C)** qPCR analysis of genes that have the most significant differential expression by intervention (CTRL or CIA), characterized as disease markers. **(B, C)** Gene expression was normalized to the *Hmbs* housekeeping gene and presented as fold difference relative to the calibrator sample (cDNA from CCR2^lo^ subset of CTRL, day 0). Individual values are presented; colored horizontal lines represent the median, boxes represent the interquartile range (IQR), and whiskers represent 1.5 times the IQR. Statistically significant difference (represented by black horizontal lines) was determined at p<0.05, Kruskal–Wallis test followed by Conover test for group-to-group comparisons.

To further explore the importance of selected genes during osteoclast differentiation, we sorted CCR2^lo^ and CCR2^hi^ OCP subsets from CTRL and CIA mice, and cultured them *in vitro* in osteoclastogenic conditions (M-CSF and RANKL) for two days before RNA extraction (**Figure 7A**). We previously showed that these OCP subsets are phenotypically and functionally different, but both could generate TRAP^+^ osteoclasts *in vitro* (**Supplementary Figure 1B**).

The expression level of genes from the osteoclast differentiation pathway in the CCR2^hi^ subset from CIA group remained high (*Fcgr1*), or even increased (*Socs3*) at day 2 osteoclastogenic culture compared to non-cultured counterparts (day 0), confirming their role in osteoclastogenic potential in arthritis (**Figure 7B**). Similar applies to *Irf7* gene, belonging to the NOD-like receptor signaling pathway, being highly induced by RANKL/M-CSF-stimulation in cultured CCR2^hi^ subset from CIA compared to the same subset from CTRL (**Figure 7B**). *Itgam* gene from Staphylococcus aureus infection pathway, although moderately induced by differentiation, remained lower in RANKL/M-CSF-stimulated CCR2^hi^ OCPs in CIA compared to CTRL. Higher expression of genes associated with biosynthetic pathways (*Dctd* and *Gnl3*) in pre-cultured CCR2^lo^ *vs* CCR2^hi^ OCPs was less apparent in differentiating preosteoclasts, although the highest expression was found in the CCR2^lo^ subset of CIA group (**Figure 7B**). In contrast, genes used as disease markers, whose biological functions are mostly associated with cell adhesion (*F11r*, *Cd38* and *Lrg1*), were expressed at reduced levels in differentiating preosteoclasts (**Figure 7C**). However, expression of *Cd38* was higher in the CCR2^hi^ subset of CIA *vs* CTRL and F11r was higher in CIA *vs* CTRL for both OCP subsets, whereas the expression pattern was completely abolished for *Lrg1* compared to pre-cultured OCPs (**Figure 7C**). Expectedly, *Kit* gene, as a marker of the immature stage, was significantly lower in differentiating compared to pre-cultured OCPs (more than 20-fold) and remained higher in the CCR2^lo^ subset from CTRL compared to CIA (**Figure 7C**).

These results suggest that differential expression of pathway-associated genes between OCP subsets as well as between CTRL and CIA groups remained detectable or even intensified during osteoclast differentiation, in contrast to gene expression pattern of disease-marker genes, used to identify CIA group immediately after sorting.

## Discussion

The aim of our study was to identify differentially expressed genes as indicators of a transcriptional response to arthritis in two OCP subsets, defined by the level of CCR2 expression. Downstream analysis performed using GSEA successfully indicated important biological pathways being consistently changed between subsets, revealing that CCR2^hi^ OCPs had more active chemokine and NOD-like receptor signaling pathways, as well as osteoclast differentiation pathway, while CCR2^lo^ OCPs had enriched ribosome biogenesis in eukaryotes and ribosome pathways. Generally speaking, we found more significant changes in gene set analyses between CCR2^hi^ and CCR2^lo^ subsets than between CTRL and CIA groups, although it was still possible to observe a weaker differential pattern that clearly discriminates these intervention groups. Our stringent analysis criteria confirmed a disparity between CCR2^hi^ and CCR2^lo^ subset transcriptomes (863 genes), with the effect of intervention (CIA) within each OCP subset being greater in CCR2^hi^ (92 genes) than in CCR2^lo^ (43 genes) OCPs. A set of ten genes was further selected for validation by qPCR, confirming that CCR2^hi^ OCPs represent a committed osteoclastogenic migratory subset susceptible to chemokine signals, whereas CCR2^lo^ OCPs are more immature subset of highly proliferative behavior. Moreover, genes associated with osteoclast differentiation pathway remained highly expressed in cultured preosteoclasts from CIA mice, suggesting their importance for enhanced osteoclastogenesis of the CCR2^hi^ OCP subset in arthritis.

Activated osteoclasts have been identified as a pathogenic substrate of bone destruction in arthritis as well as in many other diseases marked by inflammation-induced bone loss. We previously defined the CCR2^hi^ OCP subset (CD45^+^Ly6G^−^CD3^−^B220^−^NK1.1^−^CD11b^–/lo^CD115^+^CCR2^hi^) as specifically associated with arthritis and able to migrate into the affected joint (9). As confirmed by the present study, the CCR2^hi^ subset is highly enriched in periarticular bone marrow, showing significant positive correlation with the disease clinical score. According to the phenotype and functional characterization, we proposed that this subset represents circulatory OCP pool, attracted toward the CCL2 gradient to infiltrate the inflamed joints. Therefore, the enlarged subset of the CCR2^hi^ OCPs in the periarticular area is most probably comprised of these infiltrating cells. Although it is possible that CCR2 expression is downregulated upon arrival to inflamed areas, only a modest enlargement of CCR2^lo^ subset argues against conversion of CCR2^hi^ into CCR2^lo^ phenotype. Our future studies will attempt to verify that the highly osteoclastogenic CCR2^hi^ OCPs readily attach to bone surfaces, causing periarticular bone resorption. Transcriptome profiling attested that this subset includes migratory committed OCPs, with the significant enrichment of pathways related to bone resorption and inflammatory response. GSEA clearly confirmed a high osteoclastogenic potential of the CCR2^hi^ OCP subset not only in arthritic, but also in CTRL mice. More than ten genes were identified to have enrichment scores ≥2 within the osteoclast differentiation pathway gene set compared to the CCR2^lo^ OCP subset. qPCR confirmed that two of those genes (*Fcgr1* and *Socs3*), which were selected for validation from the osteoclast differentiation pathway, showed significant association with the CCR2^hi^ OCP subset and were further increased in arthritis. Moreover, those genes remained highly expressed (*Fcgr1*) or even increased in expression (*Socs3*) in preosteoclasts maturing from CCR2^hi^ OCPs *in vitro* by RANKL/M-CSF stimulation. We have previously shown that such preosteoclasts exhibited enhanced expression of *Rank*, *cFms/CD115* and *cFos* in arthritis compared to control (9). SOCS3 is induced by inflammatory cytokines (i.e. IL-6 and TNF-α) signaling through the Janus kinase (JAK)/signal transducer and activator of transcription (STAT) pathway and, in a feedback loop, acts as a potent negative regulator in immune cells (44). Recent evidence proved SOCS3 activation in different bone cells, including osteoclasts, chondrocytes and osteoblasts, playing critical roles in modulation of the inflammatory response and osteoclastogenesis in pathological states (45–47). However, expression of *Socs* genes can be induced by JAK/STAT-independent activation, including NF-κB and AP-1, by stimuli such as TGF-β and RANKL (48, 49). Therefore, contradictory roles of SOCS3 in osteoclasts have been reported. TGF-β stimulates osteoclast differentiation and survival through transcriptional activation of *Socs3*, and overexpression of *Socs3* mRNA promotes osteoclastogenesis *via* suppression of IFN-β (48, 49). On the other hand, *Socs3* deletion in hematopoietic and endothelial cells increases osteoclast numbers and bone destruction in arthritis (50). In addition to stimulation by RANKL and M-CSF, osteoclastogenesis requires costimulation by the immunoreceptor tyrosine-based activation motif pathway, mediated by the FcR common γ chain (FcRγ) and DNAX-activating protein (DAP)12 (51, 52). Osteoclasts and their progenitors express Fcγ receptors at the level comparable to macrophages and dendritic cells, and both crosslinking of FcγR on murine preosteoclasts and FcγR-ligation by antibodies against citrullinated proteins (ACPA) on human preosteoclasts promote osteoclastogenesis (53, 54). This implies that the expression of FcγRs plays an important role in enhanced osteoclast activity in diseases accompanied with high level of autoantibodies, including RA. Blocking of the FcγRs or deleting the *FcγR* gene reduces osteoclastogenesis stimulated by immune complexes and alleviates disease severity in arthritis models (55, 56). In addition to gene expression, we observed increased frequency of Fcgr1/CD64^+^ CCR2^hi^ OCPs in CIA compared to CTRL by flow cytometry. Expression of CD64 on peripheral monocytes of RA patients correlates with disease severity (57), whereas treatment with anti-TNF agent infliximab and cytostatic drug methotrexate decreases expression of CD64, paralleled by a decrease in inflammatory markers (erythrocyte sedimentation rate and C-reactive protein) (58).

In addition to the osteoclast differentiation pathway, the CCR2^hi^ subset was marked by several other gene set pathways, regardless of intervention, including NOD-like receptor signaling and chemokine signaling pathways; while CIA samples alone had additionally enriched Staphylococcus aureus infection, TNF signaling and Toll-like receptor signaling pathways. Enriched chemokine signaling pathway was expected in CCR2^hi^ OCPs, since this subset was defined based on the level of chemokine receptor (CCR2) expression. Likewise, the inflammatory pathways were enriched in CIA, as it is a systemic immune-mediated inflammatory disease. However, these findings were important to confirm the identity of our samples and the pathogenic role of CCR2^hi^ OCPs in arthritis. IRF7, selected from the NOD-like receptor signaling pathway, belongs to the IRF family proteins responsible for the production of type I interferons downstream of pathogen recognition receptors (59, 60). Several autoimmune diseases, including RA have been found to have a type I IFN signature, and genetic variants or single nucleotide polymorphisms (SNPs) within IRF genes have been detected as risk or protection factors in patients with autoimmune diseases (60, 61). *Irf7*-deficient B6 mice with K/B×N serum transfer arthritis had increased arthritis severity, with augmented systemic and local proinflammatory cytokines, indicating an overall anti-inflammatory role of IRF7 (ascribed to its regulation of IFN production and cytokine gene expression) (61). Indeed, our results showed downregulation of *Irf7* expression in the CCR2^hi^ OCP subset from arthritic mice and no association of its expression with disease clinical score. However, *in vitro* maturing osteoclasts differentiated from the CCR2^hi^ OCP subset of arthritic mice had higher expression of *Irf7* compared to CTRL cultures and pre-cultured OCPs, which may be related to the induction of *Irf7* gene by RANKL, reported in thymic epithelial cells (62). Moreover, there is a regulatory interaction between SOCS proteins and IRF7 in a way that SOCS1 and SOCS3, induced by TLR7 in human plasmacytoid dendritic cells, promote IRF7 proteasomal degradation (63). Interestingly, application of antagonistic human (h)CCR2 small molecule inhibitor after traumatic brain injury to hCCR2 knock-in mice reduced the expression of *Irf7* and mitigated loss of cognitive functions, improving disease outcome (64). A common marker of myeloid cells *Itgam* (CD11b), listed under the Staphylococcus aureus infection pathway gene set, was overexpressed in the CCR2^hi^ compared to the CCR2^lo^ OCP subset. In line with our gating strategy, which included only CD11b^-/lo^ non-lymphoid cells, we could not expect a high degree of CD11b protein expression. However, we showed that CCR2^hi^ OCPs expressed low levels of CD11b, whereas CCR2^lo^ OCPs were mostly CD11b-negative, indirectly confirming the purity and identity of sorted populations. In addition, *Itgam* gene expression was lower in CCR2^hi^ OCPs from CIA compared to the corresponding OCPs from CTRL. The magnitude of expression and the difference between CIA and CTRL was even more evident in preosteoclasts differentiated from CCR2^hi^ OCPs. Jacquin et al. showed dynamic expression of CD11b during murine osteoclastogenesis, with the initial low levels in mouse bone marrow OCPs, upregulation *in vitro* by M-CSF and subsequent downregulation by RANKL (6). Further investigation indicated that CD11b acts as a negative regulator of the earliest stages of osteoclast differentiation, since *Itgam*-deficient mice exhibited increased osteoclast numbers and decreased bone mass, whereas stimulation of CD11b/CD18 signaling by fibrinogen inhibited osteoclast differentiation by suppressing RANK expression and repressing *NFATc1* transcription (65). CD11b/CD18 interacts with a number of inflammatory ligands, such as microbial products, adhesion molecules, fibrinogen and complement components, and could block inflammatory osteoclastogenesis. In addition, it reduces TLR-dependent proinflammatory signaling in leukocytes and suppresses IFN-I signaling *via* the AKT/FOXO3/IFN3/7 pathway (66, 67). TLR-stimulated macrophages from *ITGAM* SNP carriers with systemic lupus erythematosus showed increased basal expression of IRF7 and IFN-β (66), whereas activation of CD11b inhibited the LPS-induced pro-inflammatory response in macrophages of mice with endotoxic shock (67).

Generally, the CCR2^lo^ OCP subset showed enrichment of biosynthetic pathways, such as ribosome biogenesis in eukaryotes, ribosome, DNA replication and pyrimidine metabolism in the CTRL group. This is in line with our previous observation that this subset exhibits increased cycling behavior and immature phenotype (9), suggesting it contains OCPs that recapitulate homeostatic activity of bone marrow osteoclasts. Both of the selected genes, *Gnl3* from the ribosome biogenesis in eukaryotes and *Dctd* from the pyrimidine metabolism pathways, are markers predominantly associated with proliferating stem and progenitor cells as well as immortalized cancer cell lines (68, 69). They were expressed in the CCR2^lo^ subset to a similar extent in CTRL and CIA samples (as validated by qPCR). Functionally related is the *Kit* gene, which was selected as the gene with the lowest expression in CCR2^hi^ *vs* CCR2^lo^ OCPs in CIA. Expectedly, qPCR revealed that *Kit* (receptor for stem cell factor or CD117) was expressed at higher level in the CTRL *vs* CIA group by CCR2^lo^ subset, considered as a source of immature OCPs that are possibly more frequent under physiological conditions. Moreover, proportion of OCPs expressing protein level of Kit/CD117 was higher in CCR2^lo^ than in CCR2^hi^ subset. CD115^+^CD117^+^ double-positive cells, lacking expression of other mature hematopoietic markers, were shown to exhibit characteristics of monocyte progenitors with the multilineage potency to differentiate into osteoclasts, dendritic cells and macrophages (8, 32). Further dissection of the bone marrow using CD115 and CD117 markers showed that, as OCPs mature, they downregulate CD117 but remain CD115^+^ (6), which is in line with our observation of lower *Kit* expression in OCPs stimulated by RANKL and M-CSF *in vitro* compared to pre-cultured OCPs. Moreover, *Kit* expression inversely correlated with arthritis clinical score suggesting its negative association with inflammation. It was shown that mobilization of immature mouse bone marrow cells (in response to granulocyte colony-stimulating factor) was associated with decreased CD117 expression (70), whereas pulmonary and vascular inflammation was diminished in a rat model of asthma by CD117^+^ bone marrow cell systemic administration (71).

Since our aim was to propose disease markers that could potentially indicate arthritis severity, we selected the top three upregulated genes in CIA, namely *Lrg1*, *Cd38* and *F11r*. As expected, qPCR showed that these genes were significantly overexpressed in the CCR2^hi^ OCP subset of CIA compared to CTRL mice, but, in addition, they were also increased in the CCR2^lo^ OCP subset of CIA compared to CTRL mice, although at a much lower level of expression. Expression was positively associated with arthritis severity and size of the CCR2^hi^ OCP subset. Functionally, these molecules are related to cell migration, adhesion properties and tissue homing, suggesting that CCR2^hi^ OCPs are attracted from the circulatory pool to the site of inflammation. Our previous study revealed that the highly osteoclastogenic arthritis-associated CCR2^hi^ OCP subset could be detected among infiltrating cells within the bone marrow periarticular compartment, but also in the spleen and the peripheral blood (9). Moreover, phenotyping showed increased frequency of OCPs expressing F11r/CD321, CD38 and Lrg1 in CIA compared to CTRL mice. Apart from F11r/CD321, we could not detect a significant difference in the proportion of cells expressing CD38 and Lrg1 between CCR2^lo^ and CCR2^hi^ OCP subsets within CIA group by flow cytometry. This could be explained by reported internalization, shedding and/or secretion of these molecules under inflammatory conditions, so detected membrane (F11r/CD321 and CD38) or intracellular (Lrg1) proteins may not completely correspond to their total production (72–74). LRG1 is associated with several human diseases, including cancer, inflammatory disorders, autoimmunity and neurological diseases (75). It is a secreted member of the family of leucine-rich repeat (LRR) proteins that serves as an acute phase protein and could be, therefore, used as a disease marker in RA and inflammatory bowel diseases (76). In pathological conditions, LRG1 is produced not only in the liver but also at lesion sites, i.e. in proliferative synovial fibroblasts, the macrophage lineage and endothelial cells. CD38 acts as an enzyme, with NAD-depleting and intracellular signaling activity, or as a receptor with adhesive functions (77). *Cd38*-deficient mice exhibit marked attenuation of arthritis as well as inhibition of proinflammatory cytokines, paralleled with decreased phosphorylation of NF-κB (78). Expression of CD38 was observed on peripheral blood plasma cells/plasmablasts and T lymphocytes as well as in synovial tissue biopsies from RA patients (79). F11r plays a key role in leukocyte transmigration and inflammatory extravasation (80, 81). The pathogenic role of F11r has been associated with human RA (82) as well as the K/B×N serum transfer model of mouse arthritis (83). Treatment with anti-F11r/JAM-A monoclonal antibodies delayed arthritis onset and partially ameliorated overall disease severity. Although the described genes were associated with the pathophysiology of arthritis, to the best of our knowledge, this is the first study which confirmed specific overexpression of these genes in arthritic OCPs. However, their expression largely diminished with osteoclast maturation, suggesting that their association is only with the progenitor stage of the osteoclast lineage.

## Conclusions

**Figure 8:**
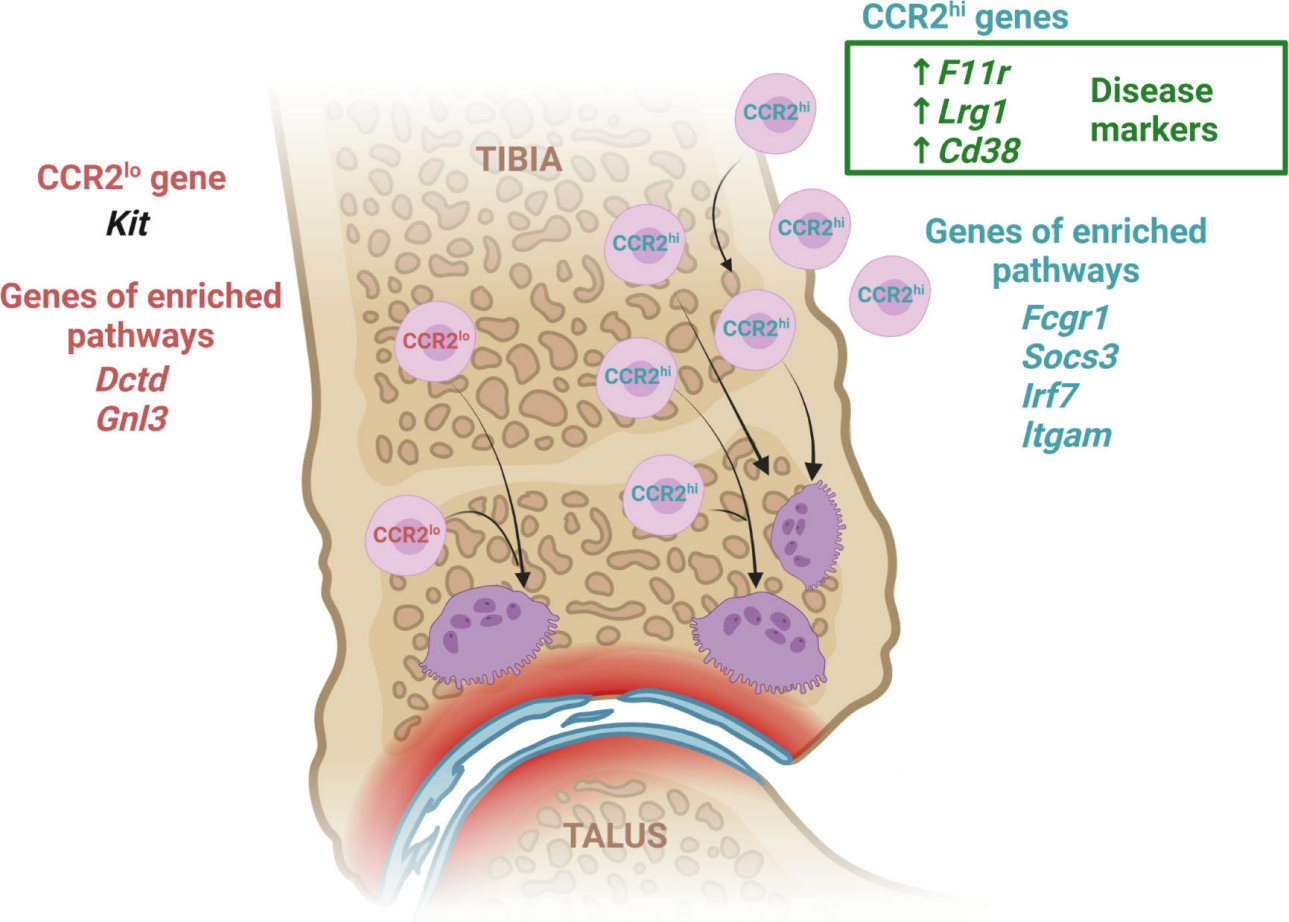
Transcriptome response of CCR2^hi^ and CCR2^lo^ osteoclast progenitor (OCP) subsets to arthritis. To better reveal the biology of inflammatory osteoresorption, we assessed the transcriptome profile of two distinct subsets of OCPs categorized by the level of CCR2 expression. We identified several genes involved in cell adhesion and migration (*F11r*, *Cd38*, *Lrg1*), which could be considered arthritis-specific genes of CCR2^hi^ subset due to being more expressed in arthritic compared to control mice and exhibiting a positive correlation between expression level and disease clinical score. Arthritic mice had an increased frequency of OCPs expressing corresponding proteins compared to controls, indicating that they could be used as disease markers. In addition, expression of *Kit* was higher in CCR2^lo^ subset and inversely correlated with arthritis, implicating its negative role in inflammation. OCP transcriptomes also revealed enrichment of pathways for osteoclast differentiation, chemokine signaling and inflammatory response in the CCR2^hi^ subset (*Fcgr1*, *Socs3*, *Irf7*, *Itgam*), and pathways associated with biosynthetic processes in the CCR2^lo^ subset (*Gnl3*, *Dctd*). Generally, our results indicate that the CCR2^lo^ subset most probably includes bone marrow resident OCPs of immature phenotype and behavior, physiologically maintaining a homeostatic level of osteoclast activity. On the other hand, the CCR2^hi^ subset represents a circulatory pool of committed OCPs, which are substantially expanded in arthritis. They are marked by a high osteoclastogenic potential as well as susceptibility to inflammatory and chemoattractant signals, thus infiltrating the periarticular compartment. Created with BioRender.com.

Although the sequence of osteoclast differentiation within the bone marrow compartment under physiological conditions is well described, the origin and precise differentiation trajectory of inflammatory osteoclasts still needs to be fully revealed. Transcriptome profiling is a useful technique to provide reliable insight into actively expressed genes and transcripts under a specific intervention, thus uncovering disease biology. We believe that our study contributes to the understanding of increased osteoclast activity associated with arthritis, by identifying two distinct OCP subsets according to the level of CCR2 expression (**Figure 8**). These subsets differ largely by pathway gene set expression, indicating high osteoclastogenic potential and chemokine signaling in CCR2^hi^ OCPs, in both control and arthritic conditions, and, in addition, increased inflammation-associated pathways in arthritis. Moreover, selected genes from the osteoclast differentiation pathway were highly expressed in arthritic osteoclasts maturing from the CCR2^hi^ subset *in vitro*. CCR2^lo^ OCPs were enriched in biosynthetic pathways and less diverse between CTRL and CIA than CCR2^hi^ OCPs. Moreover, we identified several genes and their corresponding proteins, involved in cell adhesion and tissue homing, that could be used as markers of arthritis severity. Although they were already associated with autoimmune pathology and proposed as potential therapeutic targets, our study revealed their novel role in inflammatory OCP biology.

## Data availability statement

The datasets presented in this study can be found in online repositories. The name of the repository and accession number can be found below: NCBI Sequence Read Archive; PRJNA858276.

## Ethics statement

The animal study was reviewed and approved by Ministarstvo poljoprivrede (Ministry of Agriculture), Uprava za veterinarstvo i sigurnost hrane, Zagreb, Croatia and the Ethics Committee of the University of Zagreb School of Medicine, Zagreb, Croatia.

## Author contributions

MF, AŠ, DG and DF designed the study. MF, AŠ, DG, DF, DŠ and TK performed the experiments. MF, AŠ, DG, DF, SA, DŠ, TK and NK acquired and analyzed data. MF, AŠ, DG, DF, SA and NK interpreted the results. MF, AŠ, DG and DF prepared the manuscript. All authors critically revised the manuscript and approved the final version. All authors contributed to the article and approved the submitted version.

## Footnotes

1
https://www.bioinformatics.babraham.ac.uk/projects/fastqc/.

2
https://github.com/kevinblighe/EnhancedVolcano.
