# Supplementary material for: Transcriptome profiling of osteoclast subsets associated with arthritis: A pathogenic role of CCR2hi osteoclast progenitors
Source: Front Immunol. 2022 Dec 15;13:994035. doi: 10.3389/fimmu.2022.994035 (PMC9797520; doi:10.3389/fimmu.2022.994035)
Supplement: Supplementary file 1 [file Table_1.pdf]

Supplementary table 1

List of primary conjugated monoclonal antibodies for flow cytometry and cell sorting				
Cell marker	Clone	Fluorochrome	Manufacturer	Catalogue number
CD3	145-2C11	FITC	BioLegend (San Diego, CA, USA)	100306
B220 (CD45R)	RA3-6B2	FITC	eBiosciences (San Diego, CA, USA)	11-0452-86
NK1.1	PK136	FITC	BioLegend (San Diego, CA, USA)	108706
CD11b	M1/70	APC/Fire™750	BioLegend (San Diego, CA, USA)	101262
CD11b	M1/70	PE Dazzle™594	BioLegend (San Diego, CA, USA)	101255
Ly6G	1A8	PerCP-eFluor™710	eBiosciences (San Diego, CA, USA)	46-9668-82
CD115	AFS98	PE-Cy7	BioLegend (San Diego, CA, USA)	135524
CD45	30-F11	APC	eBiosciences (San Diego, CA, USA)	17-0451-83
CD45	30-F11	Brilliant Violet 510™	BioLegend (San Diego, CA, USA)	103138
CCR2 (CD192)	SA203G11	PE	R&D Systems (Bio-Techne, Abingdon, UK)	FAB5538P
CCR2 (CD192)	SA203G11	APC/Fire™750	BioLegend (San Diego, CA, USA)	150630
F11r (CD321)	27-9	PE	BioLegend (San Diego, CA, USA)	107803
CD38	90	PE	BioLegend (San Diego, CA, USA)	102707
Lrg1	C-4	Alexa Fluor® 647	Santa Cruz Biotechnology, Inc. (Dallas, TX, USA)	sc-390920 AF-647
Kit (CD117)	2B8	APC	BD Pharmingen (Franklin Lakes, NJ, USA)	553356
Fcgr1 (CD64)	X54-5/7.1	PerCP/Cyanine5.5	BioLegend (San Diego, CA, USA)	139307
