# Supplementary material for: Transcriptome profiling of osteoclast subsets associated with arthritis: A pathogenic role of CCR2hi osteoclast progenitors
Source: Front Immunol. 2022 Dec 15;13:994035. doi: 10.3389/fimmu.2022.994035 (PMC9797520; doi:10.3389/fimmu.2022.994035)
Supplement: Supplementary file 2 [file Image_1.pdf]

Supplementary figure 1

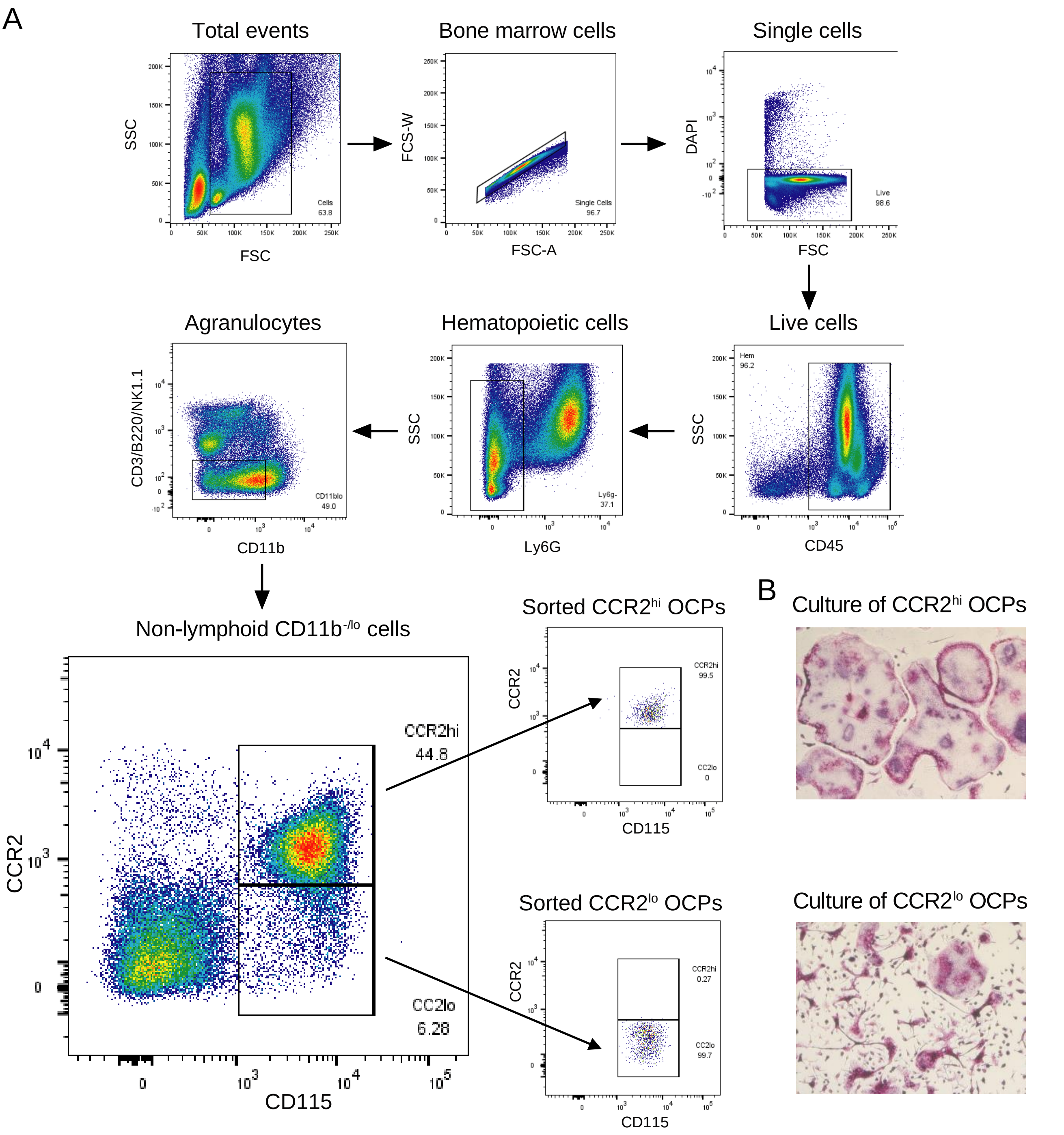

Supplementary figure 1. Gating strategy to identify and isolate bone marrow osteoclast progenitors (OCPs). Periarticular bone marrow cells were flushed from hind limb long bones of male mice and OCPs were identified as $CD45^{+}Ly6G^{-}CD3^{-}B220^{-}NK1.1^{-}CD11b^{-/lo}CD115^{+}$ cells using flow-cytometry. (**A**) Applied gating strategy first included delineation of live bone marrow single-cells, then comprised hematopoietic ($CD45^{+}$) agranulocytes ($Ly6G^{-}$), followed by gating of non-lymphoid ($CD3^{-}B220^{-}NK1.1^{-}$) cells with low expression of CD11b ($CD11b^{-/lo}$). OCPs were further delineated upon CD115 expression and sorted, based on the level of CCR2 expression, as $CCR2^{hi}$ and $CCR2^{lo}$ subsets. Sorting efficiency was above 99.5%. (**B**) Both OCP subsets were able to generate osteoclasts upon *in vitro* stimulation with M-CSF and RANKL. At the culture endpoint (3-5 days), cells were stained for tartrate-resistant acid phosphatase (TRAP) expression. Osteoclasts were identified as $TRAP^{+}$ multinucleated cells (≥3 nuclei), magnification 200×.
