# Supplementary material for: Transcriptome profiling of osteoclast subsets associated with arthritis: A pathogenic role of CCR2hi osteoclast progenitors
Source: Front Immunol. 2022 Dec 15;13:994035. doi: 10.3389/fimmu.2022.994035 (PMC9797520; doi:10.3389/fimmu.2022.994035)
Supplement: Supplementary file 3 [file Image_2.pdf]

Supplementary figure 2

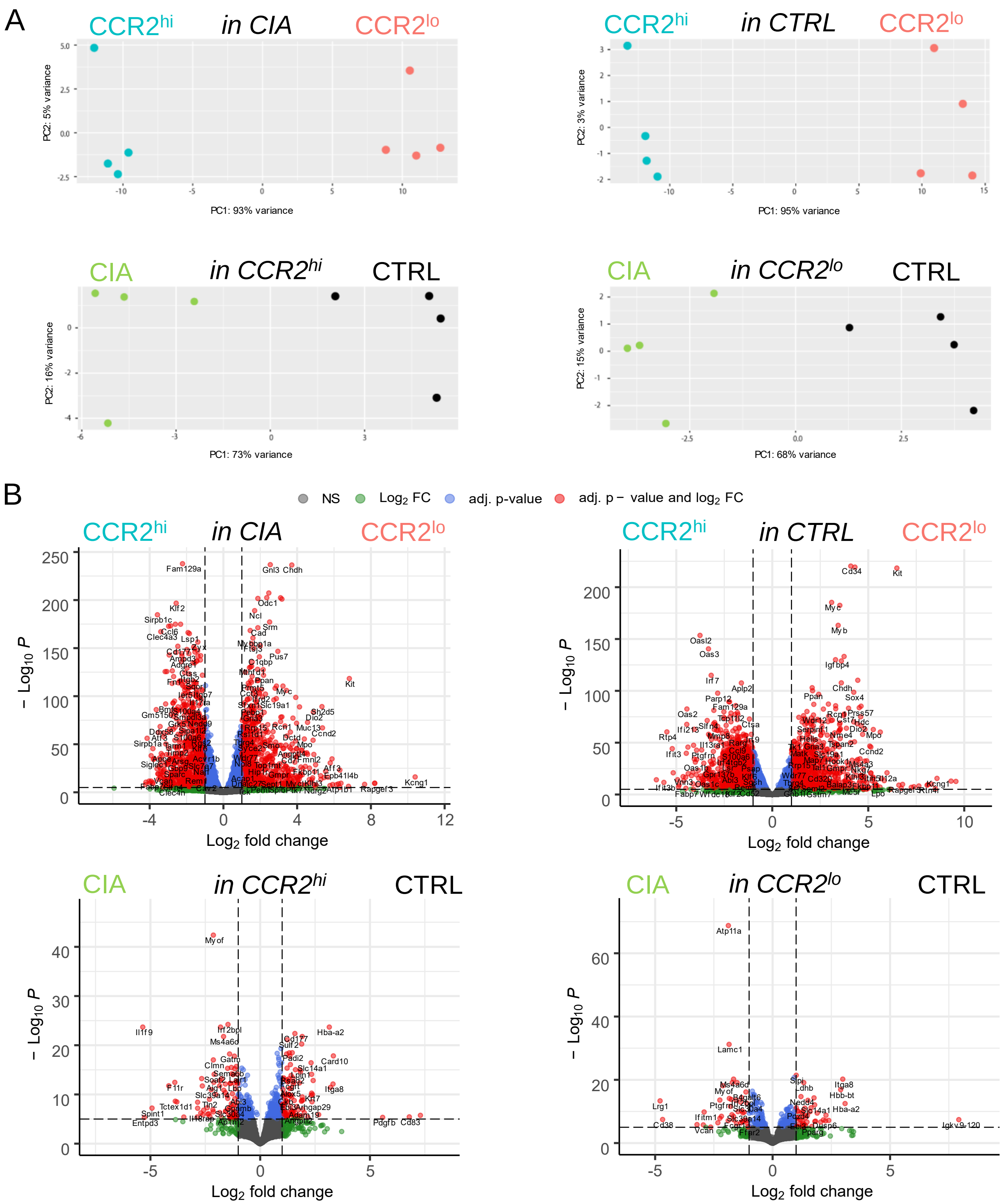

Supplementary figure 2. Sample clustering and similarity analysis. (A) Principal component analysis (PCA) performed based on expression of top 50 variable genes from RNA sequencing data in sample subsets. Upper two plots illustrate clustering based on OCP subset (CCR2^{lo} and CCR2^{hi}) in only collagen induced-arthritis (CIA) group or control (CTRL) group. Lower two plots illustrate clustering based on intervention (CIA or CTRL) in samples containing only CCR2^{lo} or CCR2^{hi} OCPs. Dots represent individual samples and are color coded to source (CCR2^{hi} cyan, CCR2^{lo} red) or intervention (CIA green, CTRL black). **(B)** Volcano plots showing comparison of gene expression in sample subsets. Upper two plots compare CCR2^{hi} and CCR2^{lo} subsets in only CIA group or CTRL group, while two lower plots compare the effect of intervention (CIA or CTRL) in samples containing only CCR2^{lo} OCPs or CCR2^{hi} OCPs. Negative logarithm of Benjamini-Hochberg correction adjusted p value (-Log_{10} P) for each gene is shown in relation to logarithm of fold change (Log_2 FC) of that gene. Differentially expressed genes are shown as red dots (|logFC|>1, p <0.01).
