# Supplementary material for: Transcriptome profiling of osteoclast subsets associated with arthritis: A pathogenic role of CCR2hi osteoclast progenitors
Source: Front Immunol. 2022 Dec 15;13:994035. doi: 10.3389/fimmu.2022.994035 (PMC9797520; doi:10.3389/fimmu.2022.994035)
Supplement: Supplementary file 4 [file Image_3.pdf]

Supplementary figure 3

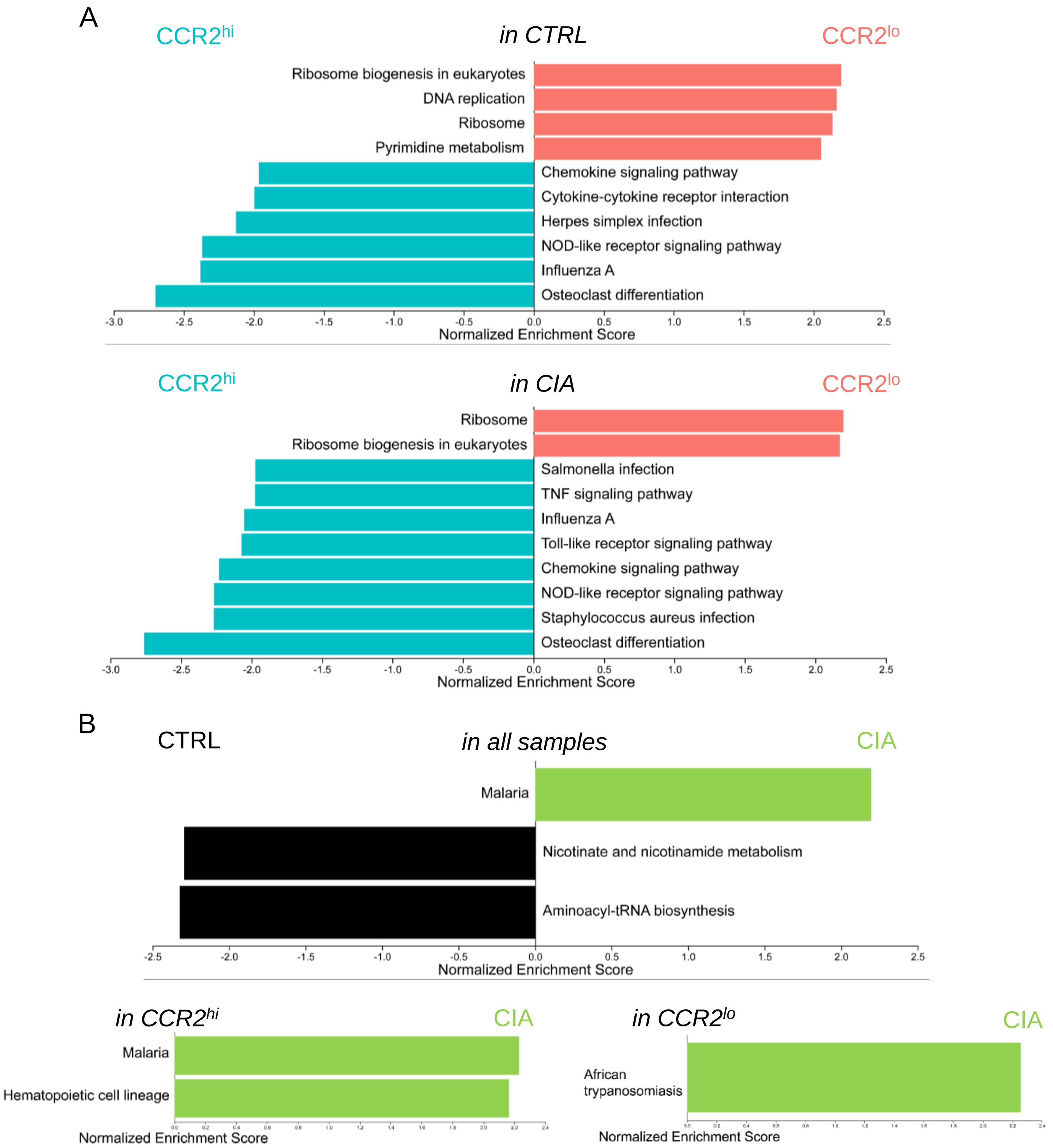

Supplementary figure 3. Gene set enrichment analysis (GSEA). (**A**) Downstream pathway analysis bar charts showing normalized enrichment scores (NESs) for pathways significantly enriched in either CCR2^{hi} (cyan) or CCR2^{lo} (red) subset when comparing gene expression in only control (CTRL) group or collagen induced arthritis (CIA) group. (**B**) Downstream pathway analysis bar charts showing NESs for significantly enriched pathways based on intervention (CIA or CTRL) in both OCP subsets (top chart), only in CCR2^{hi} subset (left bottom chart) or CCR2^{lo} subset (right bottom chart). Redundancy reduction using weighted set cover was used to minimize number of pathways while maximizing gene coverage. False discovery rate was set to <0.05. Downstream pathway analysis was carried out using WebGestalt.
