# Supplementary material for: Transcriptome profiling of osteoclast subsets associated with arthritis: A pathogenic role of CCR2hi osteoclast progenitors
Source: Front Immunol. 2022 Dec 15;13:994035. doi: 10.3389/fimmu.2022.994035 (PMC9797520; doi:10.3389/fimmu.2022.994035)
Supplement: Supplementary file 5 [file Image_4.pdf]

Supplementary figure 4

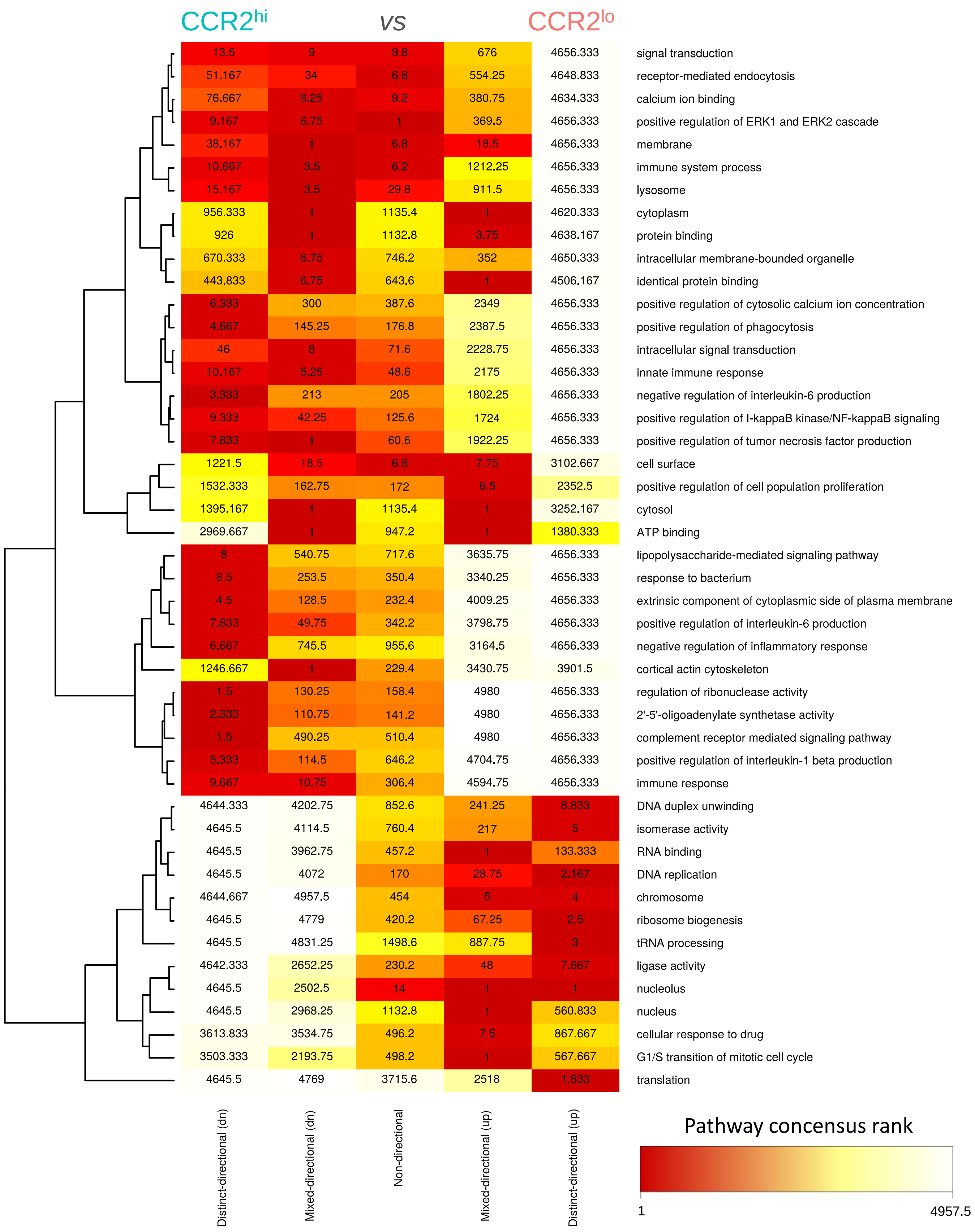

Supplementary figure 3. Heatmap of downstream pathways' concensus scores. Scores for each of the directionality classes comparing CCR2lo with CCR2hi cell subset. Significant gene sets that have median rank 1-10 in at least one class are displayed. Intensity of color visually denotes higher ranks. Created using piano R package.
