# Supplementary material for: Transcriptome profiling of osteoclast subsets associated with arthritis: A pathogenic role of CCR2hi osteoclast progenitors
Source: Front Immunol. 2022 Dec 15;13:994035. doi: 10.3389/fimmu.2022.994035 (PMC9797520; doi:10.3389/fimmu.2022.994035)
Supplement: Supplementary file 6 [file Image_5.pdf]

Supplementary figure 5

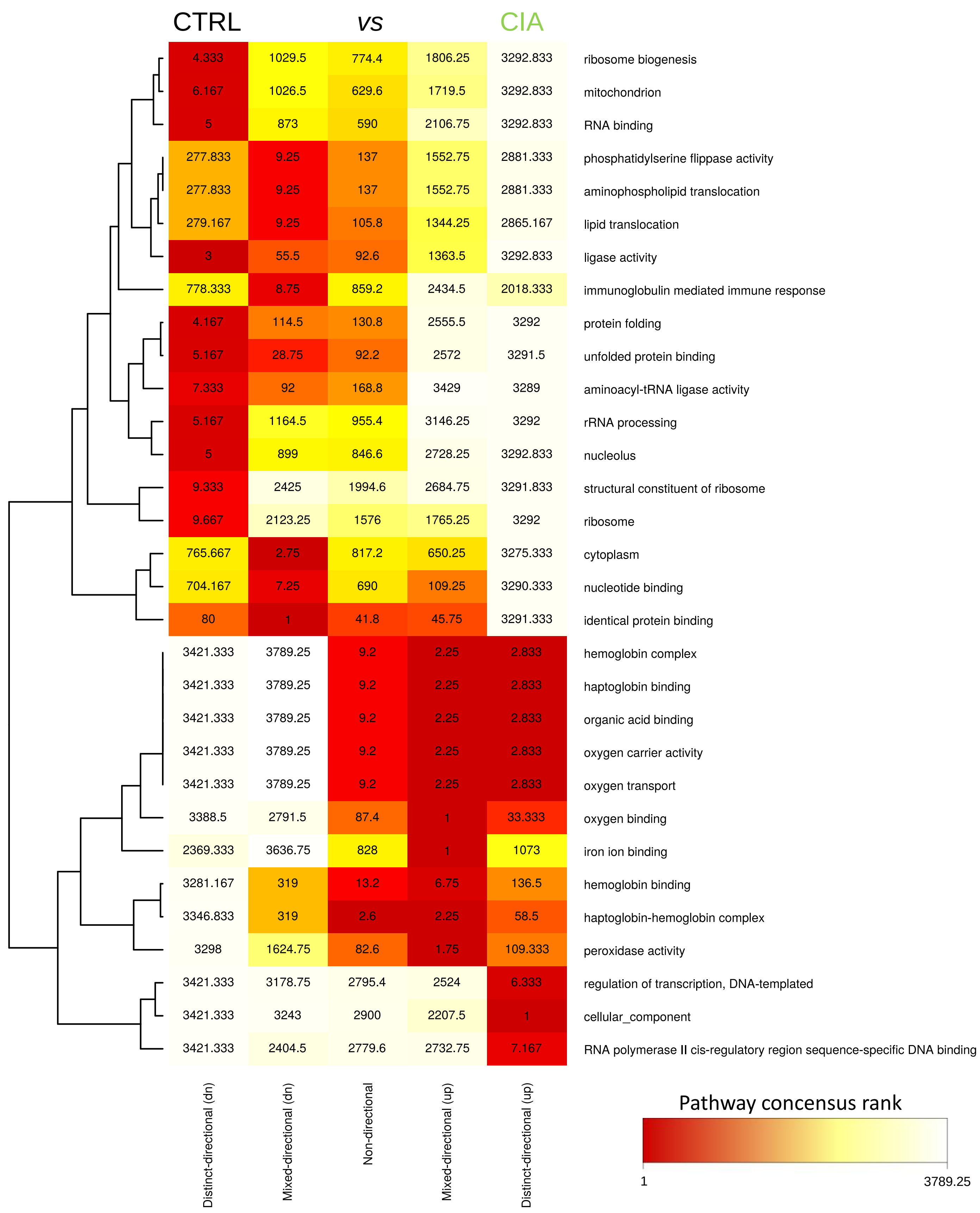

Supplementary figure 5. Heatmap of downstream pathways' concensus scores. Scores for each of the directionality classes comparing samples from collagen induced arthritis group (CIA) with control (CTRL) group. Significant gene sets that have median rank 1-10 in at least one class are displayed. Intensity of color visually denotes higher ranks. Created using piano R package.
