# Supplementary material for: Transcriptome profiling of osteoclast subsets associated with arthritis: A pathogenic role of CCR2hi osteoclast progenitors
Source: Front Immunol. 2022 Dec 15;13:994035. doi: 10.3389/fimmu.2022.994035 (PMC9797520; doi:10.3389/fimmu.2022.994035)
Supplement: Supplementary file 7 [file Image_6.pdf]

Supplementary figure 6

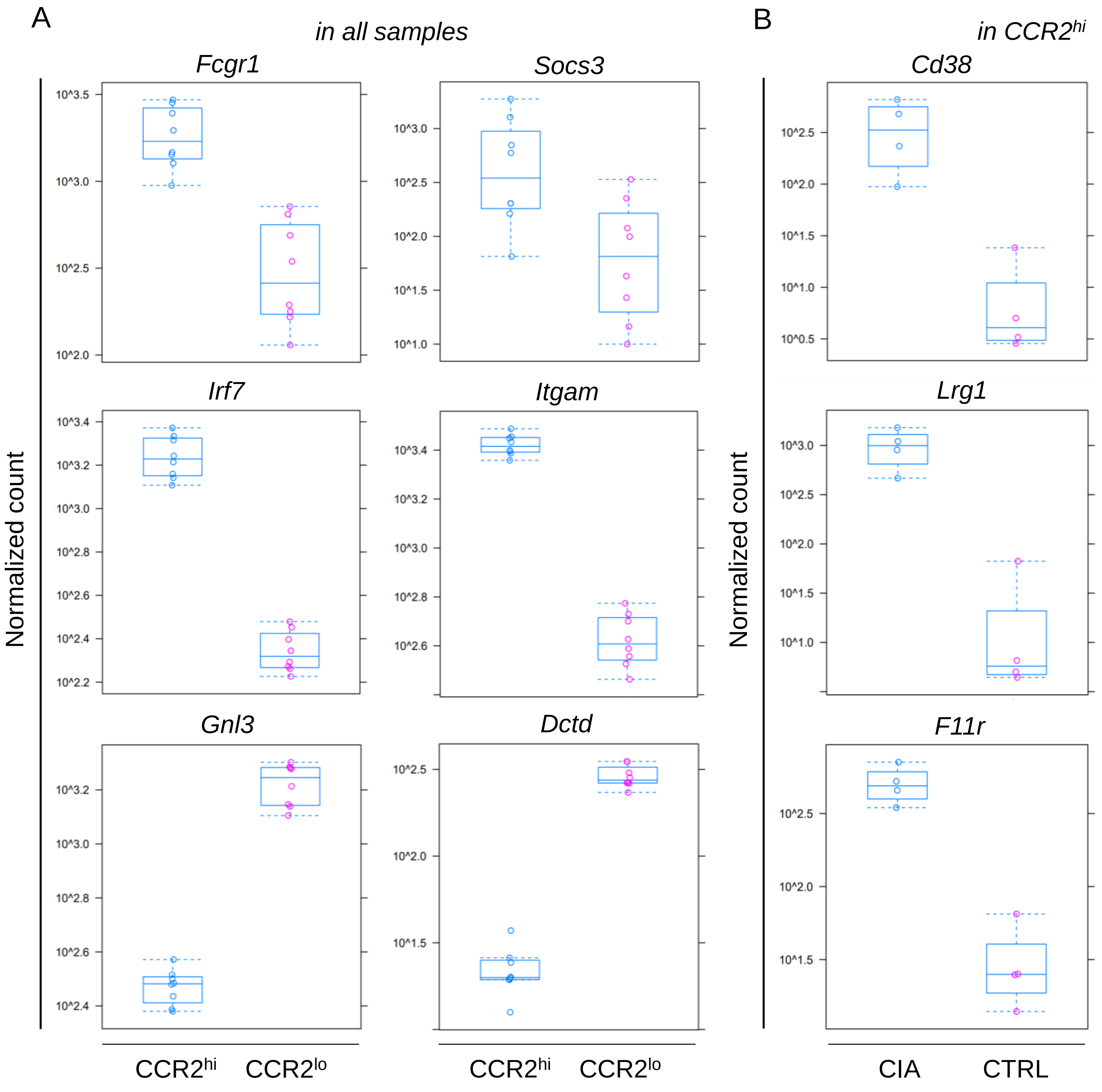

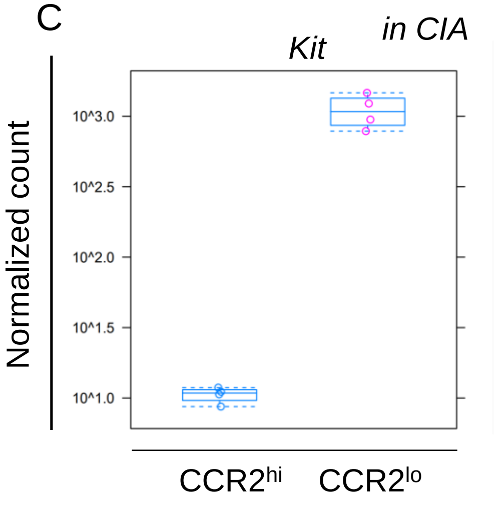

Supplementary figure 6. Normalized counts for selected genes. (**A**) Comparison of normalized gene counts between CCR2^{lo} and CCR2^{hi} osteoclast progenitor (OCP) subsets from all samples, regardless of intervention. (**B**) Comparison of normalized gene counts in CCR2^{hi} OCPs based on intervention - collagen induced arthritis (CIA) group or control (CTRL) groups. (**C**) Comparison of normalized gene counts for *Kit* gene between CCR2^{lo} and CCR2^{hi} OCPs from CIA group. All results are presented as median with interquartile range (IQR), where middle horizontal lines represent medians, boxes represent the IQR and whiskers represent 1.5 times the IQR. For all comparisons, Benjamini-Hochberg corrected p values are <0.01.
