# Supplementary material for: Transcriptome profiling of osteoclast subsets associated with arthritis: A pathogenic role of CCR2hi osteoclast progenitors
Source: Front Immunol. 2022 Dec 15;13:994035. doi: 10.3389/fimmu.2022.994035 (PMC9797520; doi:10.3389/fimmu.2022.994035)
Supplement: Supplementary file 8 [file Image_7.pdf]

Supplementary figure 7

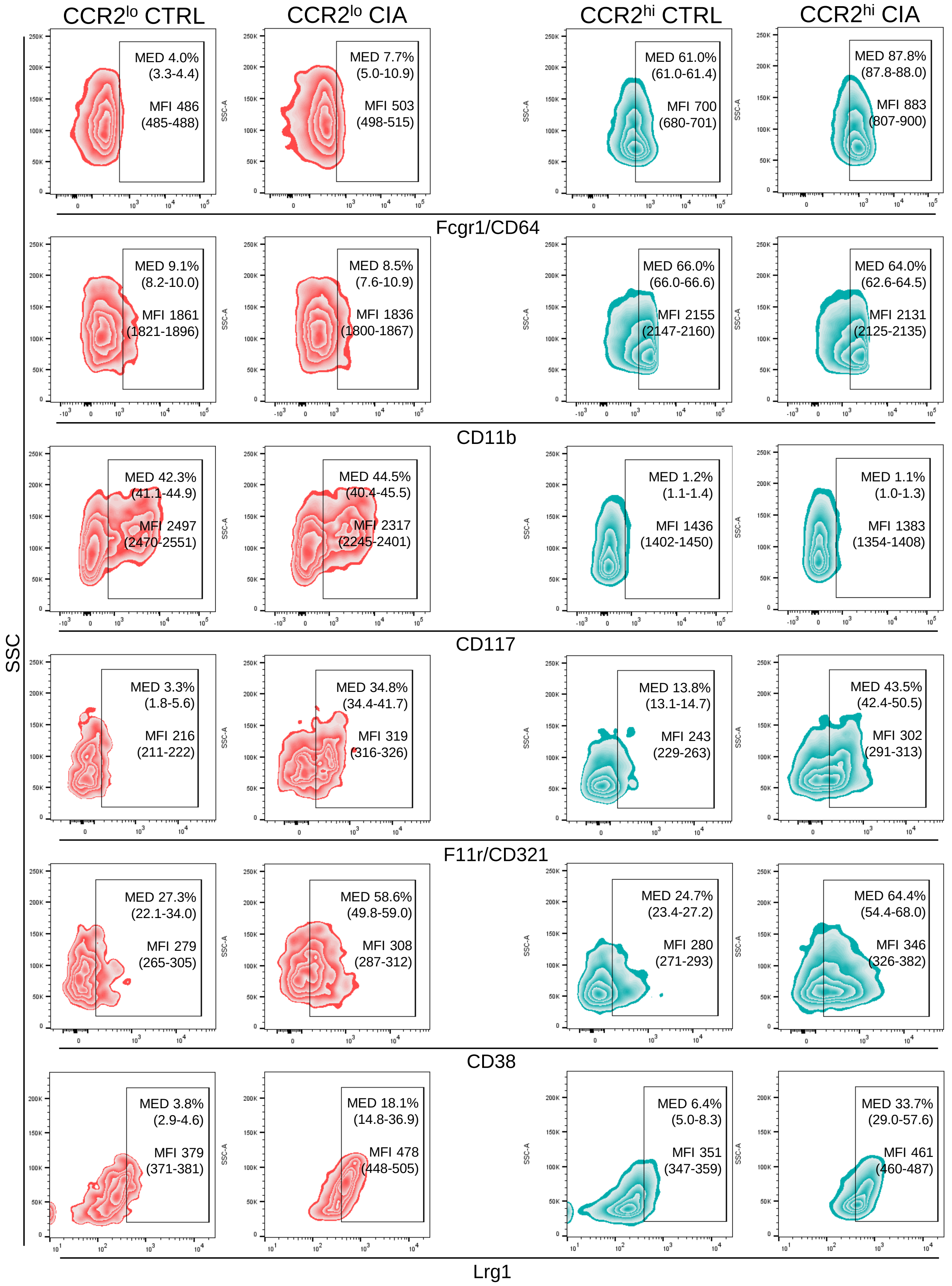

Supplementary figure 7. Phenotype of CCR2^{lo} and CCR2^{hi} subset of osteoclast progenitors in control mice (CTRL) and mice with collagen-induced arthritis (CIA). Positive populations are presented as median (MED) percentage and geometric mean fluorescence intensity (MFI), both with interquartile range (in brackets) for CTRL (n=3-4) and CIA (n=3-5). Values are calculated out of CD115^{+} CCR2^{lo} or CD115^{+} CCR2^{hi} subsets. Data were analyzed using FlowJo software.
