# Supplementary material for: Transcriptome profiling of osteoclast subsets associated with arthritis: A pathogenic role of CCR2hi osteoclast progenitors
Source: Front Immunol. 2022 Dec 15;13:994035. doi: 10.3389/fimmu.2022.994035 (PMC9797520; doi:10.3389/fimmu.2022.994035)
Supplement: Supplementary file 9 [file DataSheet_1.zip › Supplementary data 1 GENE COUNTS/Supplementary data 1 legend.docx]

Supplementary data 1. Gene count tables obtained from differential expression of genes (DEG) analysis using DESeq2. DEG was performed on a total of 15011 genes obtained after the TPM (Transcript per Million) filtering of featureCounts quantified mapped reads. The counts are presented with applied normalization (normalized_counts.xlsx), as well as regularized log (rlog) transformation (transformed_counts_rld.xlsx).

The first column denotes Ensemble gene ID, with the following columns denoting counts of that particular gene in a specific sample, where first 8 samples (DG1-8) are from CIA (collagen-induced arthritis) mica, while the latter 8 samples (DG9-16) are from control mice. Even-numbered samples contained sorted CCR2^hi^ osteoclast progenitor cells, while odd-numbered samples contained sorted CCR2^lo^ osteoclast progenitor cells.
