# Supplementary material for: Transcriptome profiling of osteoclast subsets associated with arthritis: A pathogenic role of CCR2hi osteoclast progenitors
Source: Front Immunol. 2022 Dec 15;13:994035. doi: 10.3389/fimmu.2022.994035 (PMC9797520; doi:10.3389/fimmu.2022.994035)
Supplement: Supplementary file 10 [file DataSheet_2.zip › Supplementary data 2 DGE CCR2lo vs CCR2hi all samples/RNAseq_analysis_with_DESeq2_p0.01_extended.html]

RNA-seq analysis of differential expression using DESeq2, P value cutoff 0.01


## RNA-seq analysis of differential expression using DESeq2, P value cutoff 0.01

| ID | Name | Type | Position | Image | logFC | p-Value | Adjusted p-Value |
| --- | --- | --- | --- | --- | --- | --- | --- |
| ID | Name | Type | Position | Image | logFC | p-Value | Adjusted p-Value |
| ENSMUSG00000042354 | Gnl3 | protein\_coding | 14:31012433-31019152 (-) |  | 2.4900 | 0.00e+00 | 0.00e+00 |
| ENSMUSG00000031996 | Aplp2 | protein\_coding | 9:31149557-31211815 (-) |  | -1.5800 | 0.00e+00 | 0.00e+00 |
| ENSMUSG00000019982 | Myb | protein\_coding | 10:21124935-21160984 (-) |  | 3.3100 | 2.30e-294 | 1.15e-290 |
| ENSMUSG00000026483 | Fam129a | protein\_coding | 1:151571186-151721939 (+) |  | -2.2400 | 1.19e-290 | 4.47e-287 |
| ENSMUSG00000005672 | Kit | protein\_coding | 5:75574916-75656722 (+) |  | 6.6500 | 2.31e-278 | 6.91e-275 |
| ENSMUSG00000015970 | Chdh | protein\_coding | 14:30009023-30040527 (+) |  | 3.6700 | 4.70e-264 | 1.17e-260 |
| ENSMUSG00000004100 | Ppan | protein\_coding | 9:20888175-20892178 (+) |  | 2.1500 | 2.50e-256 | 5.34e-253 |
| ENSMUSG00000027405 | Nop56 | protein\_coding | 2:130274430-130279313 (+) |  | 1.6400 | 1.55e-251 | 2.91e-248 |
| ENSMUSG00000057329 | Bcl2 | protein\_coding | 1:106538178-106714274 (-) |  | 3.4800 | 4.12e-249 | 6.85e-246 |
| ENSMUSG00000006494 | Pdk1 | protein\_coding | 2:71873224-71903858 (+) |  | 2.1100 | 1.85e-232 | 2.77e-229 |
| ENSMUSG00000060470 | Adgrg3 | protein\_coding | 8:95017692-95045250 (+) |  | 4.2100 | 1.31e-231 | 1.79e-228 |
| ENSMUSG00000022346 | Myc | protein\_coding | 15:61985391-61990374 (+) |  | 3.1700 | 7.38e-229 | 9.21e-226 |
| ENSMUSG00000024737 | Slc15a3 | protein\_coding | 19:10839727-10859362 (+) |  | -2.5700 | 1.12e-227 | 1.29e-224 |
| ENSMUSG00000016494 | Cd34 | protein\_coding | 1:194938819-194961279 (+) |  | 4.2700 | 3.08e-225 | 3.30e-222 |
| ENSMUSG00000006442 | Srm | protein\_coding | 4:148591503-148594993 (+) |  | 2.4900 | 2.70e-205 | 2.69e-202 |
| ENSMUSG00000042726 | Trafd1 | protein\_coding | 5:121371725-121385632 (-) |  | -1.1200 | 4.73e-192 | 4.43e-189 |
| ENSMUSG00000036402 | Gng12 | protein\_coding | 6:66896397-67021350 (+) |  | 1.1400 | 3.21e-187 | 2.82e-184 |
| ENSMUSG00000034707 | Gns | protein\_coding | 10:121365090-121397249 (+) |  | -1.9800 | 1.40e-185 | 1.12e-182 |
| ENSMUSG00000031403 | Dkc1 | protein\_coding | X:75095854-75109777 (+) |  | 1.9600 | 1.42e-185 | 1.12e-182 |
| ENSMUSG00000050721 | Plekho2 | protein\_coding | 9:65552698-65580040 (-) |  | -1.6700 | 5.98e-185 | 4.48e-182 |
| ENSMUSG00000061024 | Rrs1 | protein\_coding | 1:9545408-9547455 (+) |  | 1.5800 | 4.99e-183 | 3.56e-180 |
| ENSMUSG00000026019 | Wdr12 | protein\_coding | 1:60069785-60098645 (-) |  | 2.0600 | 9.04e-178 | 6.15e-175 |
| ENSMUSG00000040314 | Ctsg | protein\_coding | 14:56099881-56102574 (-) |  | 3.4800 | 2.36e-175 | 1.54e-172 |
| ENSMUSG00000057541 | Pus7 | protein\_coding | 5:23740648-23783711 (-) |  | 2.9300 | 2.78e-175 | 1.74e-172 |
| ENSMUSG00000005973 | Rcn1 | protein\_coding | 2:105386291-105399319 (-) |  | 3.2200 | 4.25e-173 | 2.55e-170 |
| ENSMUSG00000042345 | Ubash3a | protein\_coding | 17:31207873-31246892 (+) |  | 3.5400 | 1.77e-171 | 1.02e-168 |
| ENSMUSG00000015176 | Nolc1 | protein\_coding | 19:46075863-46085530 (+) |  | 1.8200 | 7.71e-171 | 4.28e-168 |
| ENSMUSG00000025612 | Bach1 | protein\_coding | 16:87698945-87733346 (+) |  | -1.7000 | 2.16e-170 | 1.16e-167 |
| ENSMUSG00000064147 | Rab44 | protein\_coding | 17:29114145-29148980 (+) |  | 1.6500 | 6.14e-169 | 3.17e-166 |
| ENSMUSG00000022686 | B3gnt5 | protein\_coding | 16:19760208-19772753 (+) |  | 3.0100 | 2.04e-167 | 1.02e-164 |
| ENSMUSG00000040274 | Cdk6 | protein\_coding | 5:3341485-3531008 (+) |  | 3.2300 | 1.44e-166 | 6.94e-164 |
| ENSMUSG00000026480 | Ncf2 | protein\_coding | 1:152800194-152836991 (+) |  | -1.3100 | 5.12e-166 | 2.40e-163 |
| ENSMUSG00000031709 | Tbc1d9 | protein\_coding | 8:83165352-83272934 (+) |  | -2.4200 | 2.67e-164 | 1.21e-161 |
| ENSMUSG00000029246 | Ppat | protein\_coding | 5:76913249-76951578 (-) |  | 1.8000 | 2.54e-163 | 1.12e-160 |
| ENSMUSG00000020323 | Prss57 | protein\_coding | 10:79781474-79790961 (-) |  | 4.3300 | 2.32e-158 | 9.95e-156 |
| ENSMUSG00000062585 | Cnr2 | protein\_coding | 4:135895394-135920207 (+) |  | -1.9700 | 1.07e-154 | 4.46e-152 |
| ENSMUSG00000108414 | Snhg1 | lncRNA | 19:8723475-8726443 (+) |  | 1.7800 | 7.57e-154 | 3.06e-151 |
| ENSMUSG00000025647 | Shisa5 | protein\_coding | 9:109038565-109057777 (+) |  | -1.4800 | 3.54e-153 | 1.39e-150 |
| ENSMUSG00000011179 | Odc1 | protein\_coding | 12:17544794-17551505 (+) |  | 2.4100 | 1.85e-151 | 7.03e-149 |
| ENSMUSG00000054942 | Miga1 | protein\_coding | 3:152273849-152340407 (-) |  | 2.5500 | 1.88e-151 | 7.03e-149 |
| ENSMUSG00000026181 | Ppm1f | protein\_coding | 16:16896469-16927364 (+) |  | 1.8700 | 4.73e-150 | 1.73e-147 |
| ENSMUSG00000024785 | Rcl1 | protein\_coding | 19:29101375-29143929 (+) |  | 1.8000 | 3.91e-149 | 1.39e-146 |
| ENSMUSG00000045349 | Sh2d5 | protein\_coding | 4:138250403-138261332 (+) |  | 4.8600 | 1.31e-148 | 4.58e-146 |
| ENSMUSG00000005125 | Ndrg1 | protein\_coding | 15:66929318-66969640 (-) |  | -1.3400 | 1.97e-148 | 6.71e-146 |
| ENSMUSG00000092203 | 1110038B12Rik | lncRNA | 17:34950238-34952471 (-) |  | 2.1400 | 2.49e-146 | 8.13e-144 |
| ENSMUSG00000026579 | F5 | protein\_coding | 1:164151838-164220277 (+) |  | -2.0500 | 2.50e-146 | 8.13e-144 |
| ENSMUSG00000037902 | Sirpa | protein\_coding | 2:129592835-129632228 (+) |  | -1.4200 | 1.85e-145 | 5.89e-143 |
| ENSMUSG00000030753 | Thap12 | protein\_coding | 7:98703103-98718062 (+) |  | 1.1500 | 4.39e-145 | 1.37e-142 |
| ENSMUSG00000031506 | Ptpn7 | protein\_coding | 1:135132700-135145317 (+) |  | 1.1100 | 1.89e-144 | 5.77e-142 |
| ENSMUSG00000022797 | Tfrc | protein\_coding | 16:32608920-32632794 (+) |  | 2.7200 | 6.96e-144 | 2.08e-141 |
| ENSMUSG00000017493 | Igfbp4 | protein\_coding | 11:99041244-99054392 (+) |  | 3.0700 | 1.90e-143 | 5.57e-141 |
| ENSMUSG00000029036 | Atad3a | protein\_coding | 4:155740641-155761093 (-) |  | 1.9100 | 4.35e-143 | 1.25e-140 |
| ENSMUSG00000037999 | Arap2 | protein\_coding | 5:62602445-62766159 (-) |  | 2.4500 | 7.36e-142 | 2.08e-139 |
| ENSMUSG00000028497 | Hacd4 | protein\_coding | 4:88396144-88438928 (-) |  | -2.4300 | 1.99e-141 | 5.53e-139 |
| ENSMUSG00000034614 | Pik3ip1 | protein\_coding | 11:3330401-3342971 (+) |  | 3.7500 | 1.07e-139 | 2.92e-137 |
| ENSMUSG00000032661 | Oas3 | protein\_coding | 5:120753098-120777661 (-) |  | -3.1000 | 1.59e-139 | 4.24e-137 |
| ENSMUSG00000060063 | Alox5ap | protein\_coding | 5:149264767-149288153 (+) |  | -1.3800 | 1.90e-138 | 4.99e-136 |
| ENSMUSG00000007682 | Dio2 | protein\_coding | 12:90724552-90738438 (-) |  | 4.6800 | 6.00e-138 | 1.55e-135 |
| ENSMUSG00000029484 | Anxa3 | protein\_coding | 5:96793339-96845966 (+) |  | 1.3400 | 9.47e-138 | 2.40e-135 |
| ENSMUSG00000034850 | Tmem127 | protein\_coding | 2:127247908-127261107 (+) |  | -0.9000 | 1.12e-137 | 2.79e-135 |
| ENSMUSG00000034612 | Chst11 | protein\_coding | 10:82985498-83195900 (+) |  | 3.6000 | 3.60e-137 | 8.84e-135 |
| ENSMUSG00000021457 | Syk | protein\_coding | 13:52583173-52648792 (+) |  | -1.1500 | 7.80e-137 | 1.88e-134 |
| ENSMUSG00000021190 | Lgmn | protein\_coding | 12:102394084-102439813 (-) |  | -1.8300 | 9.68e-137 | 2.30e-134 |
| ENSMUSG00000003518 | Dusp3 | protein\_coding | 11:101971143-101987013 (-) |  | -1.6900 | 4.42e-136 | 1.03e-133 |
| ENSMUSG00000072964 | Bhlhb9 | protein\_coding | X:135797594-135891081 (+) |  | 2.6900 | 1.10e-135 | 2.53e-133 |
| ENSMUSG00000001576 | Ergic1 | protein\_coding | 17:26561489-26656934 (+) |  | 1.7800 | 4.72e-135 | 1.07e-132 |
| ENSMUSG00000026020 | Nop58 | protein\_coding | 1:59684971-59719044 (+) |  | 1.8200 | 5.70e-135 | 1.27e-132 |
| ENSMUSG00000017760 | Ctsa | protein\_coding | 2:164832873-164841032 (+) |  | -1.0900 | 1.07e-134 | 2.36e-132 |
| ENSMUSG00000025498 | Irf7 | protein\_coding | 7:141262706-141266481 (-) |  | -2.9600 | 3.44e-134 | 7.48e-132 |
| ENSMUSG00000026879 | Gsn | protein\_coding | 2:35256380-35307892 (+) |  | -2.4400 | 3.93e-134 | 8.41e-132 |
| ENSMUSG00000020534 | Shmt1 | protein\_coding | 11:60788104-60811718 (-) |  | 2.1400 | 4.88e-134 | 1.03e-131 |
| ENSMUSG00000040785 | Ttc3 | protein\_coding | 16:94370618-94469343 (+) |  | 2.2200 | 9.19e-134 | 1.91e-131 |
| ENSMUSG00000020706 | Ftsj3 | protein\_coding | 11:106249142-106256079 (-) |  | 1.5200 | 1.39e-133 | 2.85e-131 |
| ENSMUSG00000031562 | Dctd | protein\_coding | 8:48099092-48153233 (+) |  | 3.7700 | 1.51e-133 | 3.05e-131 |
| ENSMUSG00000040093 | Bmf | protein\_coding | 2:118528757-118549687 (-) |  | -3.1100 | 3.25e-133 | 6.50e-131 |
| ENSMUSG00000025648 | Pfkfb4 | protein\_coding | 9:108991778-109032228 (+) |  | -2.0300 | 4.12e-133 | 8.13e-131 |
| ENSMUSG00000031627 | Irf2 | protein\_coding | 8:46739732-46847458 (+) |  | -1.1900 | 2.29e-132 | 4.45e-130 |
| ENSMUSG00000020034 | Tcp11l2 | protein\_coding | 10:84576626-84614359 (+) |  | -2.0700 | 1.17e-131 | 2.24e-129 |
| ENSMUSG00000078780 | Gm5150 | protein\_coding | 3:15946870-16006437 (-) |  | -3.8500 | 4.73e-131 | 8.96e-129 |
| ENSMUSG00000036046 | 5031439G07Rik | protein\_coding | 15:84943936-84988551 (-) |  | -1.0900 | 4.88e-131 | 9.14e-129 |
| ENSMUSG00000046711 | Hmga1 | protein\_coding | 17:27556497-27563674 (+) |  | 1.3600 | 7.86e-131 | 1.45e-128 |
| ENSMUSG00000002897 | Il17ra | protein\_coding | 6:120463247-120487559 (+) |  | -1.3100 | 1.24e-130 | 2.26e-128 |
| ENSMUSG00000026509 | Capn2 | protein\_coding | 1:182467260-182517608 (-) |  | -1.8500 | 1.43e-130 | 2.58e-128 |
| ENSMUSG00000020547 | Bzw2 | protein\_coding | 12:36091835-36158080 (-) |  | 1.9000 | 3.89e-130 | 6.93e-128 |
| ENSMUSG00000058099 | Nfam1 | protein\_coding | 15:82992973-83033306 (-) |  | -1.2300 | 8.63e-130 | 1.52e-127 |
| ENSMUSG00000028010 | Gar1 | protein\_coding | 3:129824912-129831396 (-) |  | 1.9600 | 1.84e-129 | 3.21e-127 |
| ENSMUSG00000046805 | Mpeg1 | protein\_coding | 19:12460779-12465283 (+) |  | -1.6200 | 5.89e-129 | 1.01e-126 |
| ENSMUSG00000038335 | Tsr1 | protein\_coding | 11:74898071-74909342 (+) |  | 1.7900 | 3.49e-128 | 5.93e-126 |
| ENSMUSG00000027395 | Polr1b | protein\_coding | 2:129100995-129126594 (+) |  | 1.8800 | 4.15e-128 | 6.98e-126 |
| ENSMUSG00000032089 | Il10ra | protein\_coding | 9:45253837-45269149 (-) |  | -1.6000 | 4.22e-128 | 7.01e-126 |
| ENSMUSG00000023262 | Acy1 | protein\_coding | 9:106432981-106438319 (-) |  | 2.6600 | 6.89e-128 | 1.13e-125 |
| ENSMUSG00000041057 | Wdr43 | protein\_coding | 17:71615895-71659031 (+) |  | 1.3600 | 9.51e-128 | 1.55e-125 |
| ENSMUSG00000035064 | Eef2k | protein\_coding | 7:120842831-120907450 (+) |  | 2.8300 | 5.30e-127 | 8.54e-125 |
| ENSMUSG00000048865 | Arhgap30 | protein\_coding | 1:171388954-171410298 (+) |  | -0.9120 | 1.16e-126 | 1.85e-124 |
| ENSMUSG00000026365 | Cfh | protein\_coding | 1:140084708-140183764 (-) |  | -2.9800 | 6.72e-126 | 1.06e-123 |
| ENSMUSG00000023048 | Prr13 | protein\_coding | 15:102459028-102462806 (+) |  | -1.2800 | 2.20e-125 | 3.43e-123 |
| ENSMUSG00000039982 | Dtx4 | protein\_coding | 19:12466338-12501454 (-) |  | 1.5700 | 2.27e-125 | 3.50e-123 |
| ENSMUSG00000039770 | Ypel5 | protein\_coding | 17:72836453-72851195 (+) |  | -1.3800 | 4.32e-125 | 6.60e-123 |
| ENSMUSG00000036002 | Fam214b | protein\_coding | 4:43032414-43046220 (-) |  | -1.9800 | 6.55e-125 | 9.90e-123 |
| ENSMUSG00000042228 | Lyn | protein\_coding | 4:3678115-3813122 (+) |  | -1.3600 | 1.40e-124 | 2.10e-122 |
| ENSMUSG00000022814 | Umps | protein\_coding | 16:33954782-33967038 (-) |  | 1.2700 | 1.97e-124 | 2.91e-122 |
| ENSMUSG00000004929 | Thop1 | protein\_coding | 10:81070035-81082559 (+) |  | 2.0400 | 2.59e-124 | 3.81e-122 |
| ENSMUSG00000024644 | Cndp2 | protein\_coding | 18:84667465-84685702 (-) |  | -0.8640 | 4.58e-124 | 6.65e-122 |
| ENSMUSG00000032570 | Atp2c1 | protein\_coding | 9:105403539-105527319 (-) |  | -0.9980 | 6.13e-124 | 8.83e-122 |
| ENSMUSG00000023110 | Prmt5 | protein\_coding | 14:54507187-54517525 (-) |  | 1.5600 | 1.12e-123 | 1.59e-121 |
| ENSMUSG00000030786 | Itgam | protein\_coding | 7:128062640-128118491 (+) |  | -2.6200 | 3.41e-123 | 4.81e-121 |
| ENSMUSG00000030530 | Furin | protein\_coding | 7:80388585-80405436 (-) |  | -1.3100 | 1.52e-122 | 2.12e-120 |
| ENSMUSG00000031508 | Ankrd10 | protein\_coding | 8:11611583-11635757 (-) |  | 1.0200 | 1.77e-122 | 2.45e-120 |
| ENSMUSG00000024011 | Pi16 | protein\_coding | 17:29317680-29330593 (+) |  | -1.9500 | 2.75e-122 | 3.78e-120 |
| ENSMUSG00000000184 | Ccnd2 | protein\_coding | 6:127125162-127152193 (-) |  | 5.2700 | 1.69e-121 | 2.30e-119 |
| ENSMUSG00000028211 | Trp53inp1 | protein\_coding | 4:11156431-11174379 (+) |  | -2.5100 | 2.70e-121 | 3.65e-119 |
| ENSMUSG00000022564 | Grina | protein\_coding | 15:76246764-76249904 (+) |  | -1.6600 | 3.19e-121 | 4.27e-119 |
| ENSMUSG00000001436 | Slc19a1 | protein\_coding | 10:77032241-77061002 (+) |  | 2.8200 | 7.82e-121 | 1.04e-118 |
| ENSMUSG00000075269 | Bex6 | protein\_coding | 16:32179823-32186972 (+) |  | 2.0300 | 3.17e-120 | 4.16e-118 |
| ENSMUSG00000028599 | Tnfrsf1b | protein\_coding | 4:145213463-145246870 (-) |  | -1.5600 | 3.22e-120 | 4.20e-118 |
| ENSMUSG00000029275 | Gfi1 | protein\_coding | 5:107716657-107726036 (-) |  | 4.8200 | 3.41e-120 | 4.40e-118 |
| ENSMUSG00000005686 | Ampd3 | protein\_coding | 7:110768206-110812405 (+) |  | -2.1700 | 4.47e-120 | 5.72e-118 |
| ENSMUSG00000030126 | Tmcc1 | protein\_coding | 6:116018611-116193486 (-) |  | -1.6300 | 2.40e-118 | 3.05e-116 |
| ENSMUSG00000040681 | Hmgn1 | protein\_coding | 16:96120618-96127729 (-) |  | 1.5800 | 3.24e-118 | 4.07e-116 |
| ENSMUSG00000055994 | Nod2 | protein\_coding | 8:88647315-88688474 (+) |  | -3.0000 | 3.41e-118 | 4.25e-116 |
| ENSMUSG00000018774 | Cd68 | protein\_coding | 11:69664213-69666153 (-) |  | -1.8700 | 3.57e-118 | 4.41e-116 |
| ENSMUSG00000027203 | Dut | protein\_coding | 2:125247190-125258608 (+) |  | 1.4300 | 4.94e-118 | 6.07e-116 |
| ENSMUSG00000055612 | Cdca7 | protein\_coding | 2:72476159-72486893 (+) |  | 1.8700 | 8.41e-118 | 1.02e-115 |
| ENSMUSG00000029994 | Anxa4 | protein\_coding | 6:86736840-86793584 (-) |  | -1.6500 | 8.89e-118 | 1.07e-115 |
| ENSMUSG00000029178 | Klf3 | protein\_coding | 5:64803388-64832901 (+) |  | -1.9100 | 1.00e-117 | 1.20e-115 |
| ENSMUSG00000027639 | Samhd1 | protein\_coding | 2:157097533-157135265 (-) |  | -1.7500 | 1.80e-117 | 2.14e-115 |
| ENSMUSG00000024177 | Nme4 | protein\_coding | 17:26091734-26095602 (-) |  | 3.4900 | 2.37e-117 | 2.79e-115 |
| ENSMUSG00000049823 | Zbtb12 | protein\_coding | 17:34879483-34896867 (+) |  | 2.2100 | 5.71e-117 | 6.68e-115 |
| ENSMUSG00000040249 | Lrp1 | protein\_coding | 10:127538161-127621148 (-) |  | -2.3400 | 9.78e-117 | 1.14e-114 |
| ENSMUSG00000024456 | Diaph1 | protein\_coding | 18:37843601-37935476 (-) |  | -0.9800 | 9.93e-117 | 1.14e-114 |
| ENSMUSG00000054640 | Slc8a1 | protein\_coding | 17:81373105-81738377 (-) |  | -1.2600 | 8.67e-116 | 9.91e-114 |
| ENSMUSG00000041438 | Utp4 | protein\_coding | 8:106893636-106923088 (+) |  | 1.7400 | 8.97e-116 | 1.02e-113 |
| ENSMUSG00000040964 | Arhgef10l | protein\_coding | 4:140514485-140666012 (-) |  | -1.5200 | 2.91e-114 | 3.28e-112 |
| ENSMUSG00000034667 | Xpot | protein\_coding | 10:121587380-121626332 (-) |  | 1.2600 | 7.75e-114 | 8.66e-112 |
| ENSMUSG00000002325 | Irf9 | protein\_coding | 14:55603571-55610030 (+) |  | -1.0500 | 1.70e-113 | 1.88e-111 |
| ENSMUSG00000022148 | Fyb | protein\_coding | 15:6522853-6663313 (+) |  | -2.2800 | 2.26e-113 | 2.49e-111 |
| ENSMUSG00000038642 | Ctss | protein\_coding | 3:95526786-95556403 (+) |  | -2.0300 | 2.32e-113 | 2.53e-111 |
| ENSMUSG00000061838 | Suclg2 | protein\_coding | 6:95473009-95718800 (-) |  | 1.4600 | 2.88e-113 | 3.13e-111 |
| ENSMUSG00000038178 | Slc43a2 | protein\_coding | 11:75531694-75577575 (+) |  | -1.3100 | 2.06e-112 | 2.21e-110 |
| ENSMUSG00000032436 | Cmtm7 | protein\_coding | 9:114756836-114781856 (-) |  | 1.0500 | 2.97e-112 | 3.18e-110 |
| ENSMUSG00000003526 | Prodh | protein\_coding | 16:18060357-18090203 (-) |  | 2.8600 | 5.20e-112 | 5.53e-110 |
| ENSMUSG00000018446 | C1qbp | protein\_coding | 11:70977836-70983026 (-) |  | 2.0000 | 6.00e-112 | 6.33e-110 |
| ENSMUSG00000016028 | Celsr1 | protein\_coding | 15:85898929-86033777 (-) |  | 4.1400 | 6.33e-112 | 6.63e-110 |
| ENSMUSG00000017781 | Pitpna | protein\_coding | 11:75588097-75628804 (+) |  | -0.9780 | 8.18e-112 | 8.51e-110 |
| ENSMUSG00000026426 | Arl8a | protein\_coding | 1:135146824-135156269 (+) |  | -0.8200 | 2.63e-111 | 2.71e-109 |
| ENSMUSG00000025330 | Padi4 | protein\_coding | 4:140745865-140774236 (-) |  | -2.2300 | 3.03e-111 | 3.11e-109 |
| ENSMUSG00000047180 | Neurl3 | protein\_coding | 1:36264597-36274679 (-) |  | -2.7400 | 4.07e-111 | 4.14e-109 |
| ENSMUSG00000025161 | Slc16a3 | protein\_coding | 11:120948480-120960868 (+) |  | -1.5200 | 4.14e-111 | 4.18e-109 |
| ENSMUSG00000032959 | Pebp1 | protein\_coding | 5:117282654-117287625 (-) |  | 1.6500 | 7.54e-111 | 7.58e-109 |
| ENSMUSG00000029591 | Ung | protein\_coding | 5:114130386-114139323 (+) |  | 2.0000 | 9.03e-111 | 9.02e-109 |
| ENSMUSG00000001288 | Rarg | protein\_coding | 15:102234938-102257517 (-) |  | -1.7400 | 9.41e-111 | 9.33e-109 |
| ENSMUSG00000060098 | Prmt7 | protein\_coding | 8:106210936-106252794 (+) |  | 1.5000 | 1.36e-110 | 1.34e-108 |
| ENSMUSG00000025742 | Prps2 | protein\_coding | X:167346322-167382749 (-) |  | 0.9830 | 1.51e-110 | 1.48e-108 |
| ENSMUSG00000039167 | Adgrl4 | protein\_coding | 3:151437887-151545086 (+) |  | 2.5500 | 2.56e-110 | 2.48e-108 |
| ENSMUSG00000027858 | Tspan2 | protein\_coding | 3:102734529-102801513 (+) |  | 3.7400 | 6.45e-110 | 6.23e-108 |
| ENSMUSG00000021384 | Susd3 | protein\_coding | 13:49230690-49248706 (-) |  | -2.1000 | 2.35e-109 | 2.26e-107 |
| ENSMUSG00000025743 | Sdc3 | protein\_coding | 4:130792537-130826319 (+) |  | -2.5900 | 4.69e-109 | 4.48e-107 |
| ENSMUSG00000042155 | Klhl23 | protein\_coding | 2:69821944-69836651 (+) |  | 1.8000 | 6.60e-109 | 6.25e-107 |
| ENSMUSG00000026271 | Gpr35 | protein\_coding | 1:92950865-92986391 (+) |  | -2.0100 | 6.72e-109 | 6.32e-107 |
| ENSMUSG00000035105 | Egln3 | protein\_coding | 12:54178981-54203860 (-) |  | 3.4100 | 1.04e-108 | 9.69e-107 |
| ENSMUSG00000024151 | Msh2 | protein\_coding | 17:87672330-87723713 (+) |  | 1.3300 | 3.28e-108 | 3.05e-106 |
| ENSMUSG00000042608 | Stk40 | protein\_coding | 4:126103957-126141029 (+) |  | -1.3700 | 5.01e-108 | 4.62e-106 |
| ENSMUSG00000037992 | Rara | protein\_coding | 11:98927818-98974942 (+) |  | -1.7200 | 5.03e-108 | 4.62e-106 |
| ENSMUSG00000025795 | Rassf3 | protein\_coding | 10:121410350-121476347 (-) |  | -1.1000 | 8.87e-108 | 8.10e-106 |
| ENSMUSG00000022824 | Muc13 | protein\_coding | 16:33794037-33819934 (+) |  | 4.7400 | 2.41e-107 | 2.19e-105 |
| ENSMUSG00000010048 | Ifrd2 | protein\_coding | 9:107587642-107593385 (+) |  | 1.8900 | 3.94e-107 | 3.56e-105 |
| ENSMUSG00000015340 | Cybb | protein\_coding | X:9435252-9487771 (-) |  | -1.9900 | 4.14e-107 | 3.72e-105 |
| ENSMUSG00000029860 | Zyx | protein\_coding | 6:42349630-42360213 (+) |  | -1.3100 | 5.34e-107 | 4.76e-105 |
| ENSMUSG00000028633 | Ctps | protein\_coding | 4:120539868-120570276 (-) |  | 1.7200 | 1.23e-106 | 1.09e-104 |
| ENSMUSG00000069874 | Irgm2 | protein\_coding | 11:58199618-58222782 (+) |  | -1.7100 | 3.10e-106 | 2.73e-104 |
| ENSMUSG00000014907 | Naf1 | protein\_coding | 8:66860217-66890564 (+) |  | 1.5800 | 3.20e-106 | 2.80e-104 |
| ENSMUSG00000036944 | Tmem71 | protein\_coding | 15:66526212-66561103 (-) |  | -1.9900 | 7.00e-106 | 6.10e-104 |
| ENSMUSG00000044066 | Cep68 | protein\_coding | 11:20227037-20249429 (-) |  | 1.6900 | 9.74e-106 | 8.43e-104 |
| ENSMUSG00000026915 | Strbp | protein\_coding | 2:37483228-37703859 (-) |  | 1.3400 | 2.21e-105 | 1.90e-103 |
| ENSMUSG00000031824 | 6430548M08Rik | protein\_coding | 8:120114152-120165306 (+) |  | -2.2600 | 2.34e-105 | 2.00e-103 |
| ENSMUSG00000025511 | Tspan4 | protein\_coding | 7:141475240-141493427 (+) |  | 2.5700 | 4.58e-105 | 3.90e-103 |
| ENSMUSG00000069662 | Marcks | protein\_coding | 10:37133375-37138920 (-) |  | -2.3700 | 5.56e-105 | 4.70e-103 |
| ENSMUSG00000054321 | Taf4b | protein\_coding | 18:14783245-14900359 (+) |  | 2.4000 | 8.13e-105 | 6.84e-103 |
| ENSMUSG00000095788 | Sirpb1a | protein\_coding | 3:15371653-15426520 (-) |  | -4.3000 | 1.05e-104 | 8.75e-103 |
| ENSMUSG00000037185 | Krt80 | protein\_coding | 15:101347444-101370162 (-) |  | -2.6900 | 3.74e-104 | 3.11e-102 |
| ENSMUSG00000070348 | Ccnd1 | protein\_coding | 7:144929931-144939925 (-) |  | 3.6400 | 5.01e-104 | 4.15e-102 |
| ENSMUSG00000036371 | Serbp1 | protein\_coding | 6:67238176-67297736 (+) |  | 0.9840 | 5.20e-104 | 4.28e-102 |
| ENSMUSG00000075284 | Wipf1 | protein\_coding | 2:73429610-73529734 (-) |  | -1.0100 | 6.37e-104 | 5.21e-102 |
| ENSMUSG00000049988 | Lrrc25 | protein\_coding | 8:70616155-70621483 (+) |  | -1.7300 | 1.14e-103 | 9.32e-102 |
| ENSMUSG00000026641 | Usf1 | protein\_coding | 1:171411313-171419142 (+) |  | -0.7710 | 1.26e-103 | 1.02e-101 |
| ENSMUSG00000021108 | Prkch | protein\_coding | 12:73584796-73778185 (+) |  | -2.0000 | 1.54e-103 | 1.24e-101 |
| ENSMUSG00000035208 | Slfn8 | protein\_coding | 11:83002158-83020810 (-) |  | -2.0900 | 2.44e-103 | 1.96e-101 |
| ENSMUSG00000026457 | Adipor1 | protein\_coding | 1:134415378-134433351 (+) |  | -0.7380 | 2.61e-103 | 2.08e-101 |
| ENSMUSG00000021069 | Pygl | protein\_coding | 12:70190811-70231488 (-) |  | -1.3700 | 2.73e-103 | 2.16e-101 |
| ENSMUSG00000018428 | Akap1 | protein\_coding | 11:88830792-88864586 (-) |  | 1.9100 | 2.91e-103 | 2.30e-101 |
| ENSMUSG00000023915 | Tnfrsf21 | protein\_coding | 17:43016555-43089189 (+) |  | -1.2500 | 4.11e-103 | 3.22e-101 |
| ENSMUSG00000030036 | Mogs | protein\_coding | 6:83115496-83118898 (+) |  | 1.2500 | 4.96e-103 | 3.87e-101 |
| ENSMUSG00000024270 | Slc39a6 | protein\_coding | 18:24579881-24603817 (-) |  | 1.0300 | 7.80e-103 | 6.05e-101 |
| ENSMUSG00000022962 | Gart | protein\_coding | 16:91621186-91646952 (-) |  | 1.4300 | 8.47e-103 | 6.53e-101 |
| ENSMUSG00000026603 | Smyd2 | protein\_coding | 1:189880492-189922363 (-) |  | 2.1700 | 1.04e-102 | 7.99e-101 |
| ENSMUSG00000002147 | Stat6 | protein\_coding | 10:127642986-127660957 (+) |  | -1.1200 | 1.58e-102 | 1.21e-100 |
| ENSMUSG00000025429 | Pstpip2 | protein\_coding | 18:77789914-77882879 (+) |  | -1.3500 | 1.62e-102 | 1.23e-100 |
| ENSMUSG00000021794 | Glud1 | protein\_coding | 14:34310727-34345265 (+) |  | -0.9220 | 3.02e-102 | 2.29e-100 |
| ENSMUSG00000054520 | Sh3bp2 | protein\_coding | 5:34525838-34563641 (+) |  | -1.2800 | 3.92e-102 | 2.95e-100 |
| ENSMUSG00000032434 | Cmtm6 | protein\_coding | 9:114731116-114749344 (+) |  | -0.9480 | 4.05e-102 | 3.03e-100 |
| ENSMUSG00000025001 | Hells | protein\_coding | 19:38930915-38971051 (+) |  | 1.7200 | 1.52e-101 | 1.13e-99 |
| ENSMUSG00000033985 | Tesk2 | protein\_coding | 4:116720948-116805956 (+) |  | -1.3500 | 1.92e-101 | 1.42e-99 |
| ENSMUSG00000020387 | Jade2 | protein\_coding | 11:51813455-51857653 (-) |  | 3.4200 | 2.12e-101 | 1.56e-99 |
| ENSMUSG00000022016 | Akap11 | protein\_coding | 14:78492246-78536808 (-) |  | 1.0900 | 5.40e-101 | 3.96e-99 |
| ENSMUSG00000041688 | Amot | protein\_coding | X:145446425-145505181 (-) |  | 2.1700 | 5.81e-101 | 4.25e-99 |
| ENSMUSG00000021458 | Aopep | protein\_coding | 13:62964893-63326096 (+) |  | 1.3500 | 6.14e-101 | 4.46e-99 |
| ENSMUSG00000027947 | Il6ra | protein\_coding | 3:89864059-89913196 (-) |  | -1.0500 | 6.70e-101 | 4.85e-99 |
| ENSMUSG00000064120 | Mocs1 | protein\_coding | 17:49428362-49455435 (+) |  | -0.9570 | 8.70e-101 | 6.26e-99 |
| ENSMUSG00000038507 | Parp12 | protein\_coding | 6:39086410-39118349 (-) |  | -2.6100 | 1.09e-100 | 7.81e-99 |
| ENSMUSG00000031904 | Slc7a6 | protein\_coding | 8:106168857-106198706 (+) |  | 1.5100 | 1.35e-100 | 9.59e-99 |
| ENSMUSG00000001305 | Rrp15 | protein\_coding | 1:186720978-186749358 (-) |  | 1.5400 | 1.70e-100 | 1.21e-98 |
| ENSMUSG00000031453 | Rasa3 | protein\_coding | 8:13566948-13677603 (-) |  | -1.1300 | 1.97e-100 | 1.39e-98 |
| ENSMUSG00000020823 | Sec14l1 | protein\_coding | 11:117115168-117159268 (+) |  | -1.2400 | 2.35e-100 | 1.65e-98 |
| ENSMUSG00000020717 | Pecam1 | protein\_coding | 11:106654217-106750628 (-) |  | 2.0200 | 3.07e-100 | 2.15e-98 |
| ENSMUSG00000027360 | Hdc | protein\_coding | 2:126593667-126619299 (-) |  | 4.3200 | 3.87e-100 | 2.70e-98 |
| ENSMUSG00000028656 | Cap1 | protein\_coding | 4:122859047-122886056 (-) |  | -1.0600 | 4.24e-100 | 2.94e-98 |
| ENSMUSG00000009566 | Fpgs | protein\_coding | 2:32682609-32704145 (-) |  | 2.1000 | 4.47e-100 | 3.09e-98 |
| ENSMUSG00000045636 | Mtus1 | protein\_coding | 8:40990914-41133726 (-) |  | -0.8420 | 6.20e-100 | 4.26e-98 |
| ENSMUSG00000076431 | Sox4 | protein\_coding | 13:28948919-28953713 (-) |  | 4.4100 | 7.67e-100 | 5.25e-98 |
| ENSMUSG00000027995 | Tlr2 | protein\_coding | 3:83836272-83841767 (-) |  | -1.7300 | 9.80e-100 | 6.67e-98 |
| ENSMUSG00000038279 | Nop2 | protein\_coding | 6:125131909-125144753 (+) |  | 1.4400 | 1.17e-99 | 7.95e-98 |
| ENSMUSG00000068566 | Myadm | protein\_coding | 7:3289080-3300442 (+) |  | -1.6600 | 1.78e-99 | 1.20e-97 |
| ENSMUSG00000090665 | Gad1-ps | processed\_pseudogene | 10:99444044-99445819 (+) |  | -2.9200 | 1.90e-99 | 1.27e-97 |
| ENSMUSG00000002957 | Ap2a2 | protein\_coding | 7:141562173-141633011 (+) |  | -1.0800 | 2.15e-99 | 1.43e-97 |
| ENSMUSG00000020692 | Nle1 | protein\_coding | 11:82900768-82908411 (-) |  | 1.7900 | 2.56e-99 | 1.70e-97 |
| ENSMUSG00000020988 | L2hgdh | protein\_coding | 12:69690433-69724873 (-) |  | 2.4500 | 3.19e-99 | 2.11e-97 |
| ENSMUSG00000001687 | Ubl3 | protein\_coding | 5:148504635-148552789 (-) |  | -1.1300 | 3.25e-99 | 2.14e-97 |
| ENSMUSG00000020101 | Vsir | protein\_coding | 10:60346851-60372684 (+) |  | -1.4500 | 3.42e-99 | 2.24e-97 |
| ENSMUSG00000030880 | Polr3e | protein\_coding | 7:120917744-120947432 (+) |  | 1.0900 | 3.57e-99 | 2.34e-97 |
| ENSMUSG00000004730 | Adgre1 | protein\_coding | 17:57358691-57483527 (+) |  | -2.2100 | 3.90e-99 | 2.54e-97 |
| ENSMUSG00000024164 | C3 | protein\_coding | 17:57203970-57228136 (-) |  | -1.6400 | 5.39e-99 | 3.49e-97 |
| ENSMUSG00000020097 | Sgpl1 | protein\_coding | 10:61098642-61147703 (-) |  | -0.6860 | 1.08e-98 | 7.00e-97 |
| ENSMUSG00000041439 | Mfsd6 | protein\_coding | 1:52656286-52727462 (-) |  | -1.6600 | 1.81e-98 | 1.16e-96 |
| ENSMUSG00000000290 | Itgb2 | protein\_coding | 10:77530252-77565708 (+) |  | -1.8700 | 1.94e-98 | 1.24e-96 |
| ENSMUSG00000038482 | Tfdp1 | protein\_coding | 8:13338751-13378448 (+) |  | 1.0300 | 2.05e-98 | 1.31e-96 |
| ENSMUSG00000000134 | Tfe3 | protein\_coding | X:7762560-7775202 (+) |  | -0.9660 | 3.19e-98 | 2.02e-96 |
| ENSMUSG00000018819 | Lsp1 | protein\_coding | 7:142460809-142494867 (+) |  | -1.7600 | 3.66e-98 | 2.31e-96 |
| ENSMUSG00000037946 | Fgd3 | protein\_coding | 13:49261554-49320311 (-) |  | -1.4900 | 4.59e-98 | 2.89e-96 |
| ENSMUSG00000059878 | Zfp422 | protein\_coding | 6:116624016-116628999 (-) |  | 1.3400 | 7.82e-98 | 4.90e-96 |
| ENSMUSG00000035517 | Tdrd7 | protein\_coding | 4:45965334-46034761 (+) |  | -1.2600 | 1.57e-97 | 9.77e-96 |
| ENSMUSG00000027540 | Ptpn1 | protein\_coding | 2:167932057-167979385 (+) |  | -1.3900 | 1.87e-97 | 1.16e-95 |
| ENSMUSG00000021939 | Ctsb | protein\_coding | 14:63122462-63145923 (+) |  | -1.1600 | 1.96e-97 | 1.21e-95 |
| ENSMUSG00000054766 | Set | protein\_coding | 2:30057378-30072577 (+) |  | 1.2600 | 2.33e-97 | 1.44e-95 |
| ENSMUSG00000049804 | Armcx4 | protein\_coding | X:134686519-134696757 (+) |  | 4.9000 | 2.73e-97 | 1.67e-95 |
| ENSMUSG00000074227 | Spint2 | protein\_coding | 7:29256323-29281912 (-) |  | 3.1900 | 5.93e-97 | 3.62e-95 |
| ENSMUSG00000018583 | G3bp1 | protein\_coding | 11:55469685-55504838 (+) |  | 0.9470 | 6.65e-97 | 4.05e-95 |
| ENSMUSG00000025086 | Trub1 | protein\_coding | 19:57452912-57491005 (+) |  | 1.8700 | 1.43e-96 | 8.65e-95 |
| ENSMUSG00000022664 | Slc35a5 | protein\_coding | 16:45139573-45158706 (-) |  | -1.2300 | 1.45e-96 | 8.76e-95 |
| ENSMUSG00000025026 | Add3 | protein\_coding | 19:53140443-53247399 (+) |  | -1.3900 | 1.75e-96 | 1.05e-94 |
| ENSMUSG00000026974 | Zmynd19 | protein\_coding | 2:24949792-24962075 (+) |  | 1.5300 | 1.80e-96 | 1.08e-94 |
| ENSMUSG00000031788 | Kifc3 | protein\_coding | 8:95099828-95202812 (-) |  | -2.0800 | 2.41e-96 | 1.44e-94 |
| ENSMUSG00000079225 | Gm9531 | transcribed\_processed\_pseudogene | 9:81677598-81678677 (+) |  | 1.2900 | 2.99e-96 | 1.78e-94 |
| ENSMUSG00000022476 | Polr3h | protein\_coding | 15:81888228-81926240 (-) |  | 1.7300 | 5.48e-96 | 3.24e-94 |
| ENSMUSG00000037275 | Gemin5 | protein\_coding | 11:58120002-58168539 (-) |  | 1.4800 | 6.79e-96 | 4.01e-94 |
| ENSMUSG00000004207 | Psap | protein\_coding | 10:60277627-60302597 (+) |  | -1.2000 | 7.22e-96 | 4.24e-94 |
| ENSMUSG00000005823 | Gpr108 | protein\_coding | 17:57234635-57248446 (-) |  | -0.8990 | 8.58e-96 | 5.02e-94 |
| ENSMUSG00000027082 | Tfpi | protein\_coding | 2:84432855-84476775 (-) |  | 1.9700 | 1.00e-95 | 5.85e-94 |
| ENSMUSG00000020075 | Ddx21 | protein\_coding | 10:62580251-62602281 (-) |  | 1.1200 | 1.91e-95 | 1.11e-93 |
| ENSMUSG00000020629 | Adi1 | protein\_coding | 12:28675231-28682175 (+) |  | 1.4900 | 2.12e-95 | 1.22e-93 |
| ENSMUSG00000017929 | B4galt5 | protein\_coding | 2:167298444-167349183 (-) |  | -1.0600 | 2.35e-95 | 1.35e-93 |
| ENSMUSG00000055538 | Zcchc24 | protein\_coding | 14:25711642-25769039 (-) |  | -2.1100 | 2.73e-95 | 1.57e-93 |
| ENSMUSG00000058006 | Mdn1 | protein\_coding | 4:32657119-32775217 (+) |  | 2.0100 | 6.61e-95 | 3.78e-93 |
| ENSMUSG00000022973 | Synj1 | protein\_coding | 16:90936092-91011308 (-) |  | -1.1300 | 7.54e-95 | 4.29e-93 |
| ENSMUSG00000009350 | Mpo | protein\_coding | 11:87793581-87804413 (+) |  | 4.8600 | 9.27e-95 | 5.26e-93 |
| ENSMUSG00000022014 | Epsti1 | protein\_coding | 14:77904239-78002657 (+) |  | -1.7800 | 1.01e-94 | 5.72e-93 |
| ENSMUSG00000042759 | Apobr | protein\_coding | 7:126584942-126589112 (+) |  | -1.8200 | 1.91e-94 | 1.07e-92 |
| ENSMUSG00000095567 | Noc2l | protein\_coding | 4:156235919-156247616 (+) |  | 1.1800 | 2.09e-94 | 1.17e-92 |
| ENSMUSG00000024851 | Pitpnm1 | protein\_coding | 19:4099998-4113965 (+) |  | -1.5800 | 2.84e-94 | 1.59e-92 |
| ENSMUSG00000045658 | Pid1 | protein\_coding | 1:84036296-84364180 (-) |  | -2.4800 | 3.83e-94 | 2.13e-92 |
| ENSMUSG00000017830 | Dhx58 | protein\_coding | 11:100694884-100704271 (-) |  | -1.6400 | 4.27e-94 | 2.37e-92 |
| ENSMUSG00000003484 | Cyp4f18 | protein\_coding | 8:71988482-72009626 (-) |  | -1.5600 | 5.42e-94 | 2.99e-92 |
| ENSMUSG00000026107 | Nabp1 | protein\_coding | 1:51465862-51478425 (-) |  | -2.1400 | 5.76e-94 | 3.17e-92 |
| ENSMUSG00000037926 | Ssh2 | protein\_coding | 11:77216287-77460220 (+) |  | -1.9400 | 8.55e-94 | 4.69e-92 |
| ENSMUSG00000005803 | Sqor | protein\_coding | 2:122765237-122809569 (+) |  | -1.6300 | 8.92e-94 | 4.87e-92 |
| ENSMUSG00000020589 | Fam49a | protein\_coding | 12:12262139-12380965 (+) |  | -1.9200 | 9.29e-94 | 5.06e-92 |
| ENSMUSG00000024078 | Ttc27 | protein\_coding | 17:74717732-74863570 (+) |  | 1.4600 | 1.22e-93 | 6.61e-92 |
| ENSMUSG00000040624 | Plekhg1 | protein\_coding | 10:3740364-3967303 (+) |  | -2.0700 | 1.28e-93 | 6.92e-92 |
| ENSMUSG00000030872 | Gga2 | protein\_coding | 7:121986722-122021222 (-) |  | 1.4400 | 1.51e-93 | 8.15e-92 |
| ENSMUSG00000054408 | Spcs3 | protein\_coding | 8:54520433-54529998 (-) |  | 0.7190 | 2.01e-93 | 1.08e-91 |
| ENSMUSG00000030314 | Atg7 | protein\_coding | 6:114643097-114860614 (+) |  | -0.9630 | 2.95e-93 | 1.58e-91 |
| ENSMUSG00000030223 | Ptpro | protein\_coding | 6:137252319-137463233 (+) |  | -1.8400 | 2.99e-93 | 1.59e-91 |
| ENSMUSG00000024097 | Srsf7 | protein\_coding | 17:80200080-80207307 (-) |  | 0.8570 | 3.48e-93 | 1.85e-91 |
| ENSMUSG00000116957 | Gm34680 | lncRNA | 16:32550211-32553708 (+) |  | 4.0700 | 5.85e-93 | 3.09e-91 |
| ENSMUSG00000058818 | Pirb | protein\_coding | 7:3711409-3720391 (-) |  | -2.1700 | 6.18e-93 | 3.26e-91 |
| ENSMUSG00000044700 | Tmem201 | protein\_coding | 4:149715375-149738044 (-) |  | 2.3000 | 7.11e-93 | 3.73e-91 |
| ENSMUSG00000048779 | P2ry6 | protein\_coding | 7:100937630-100974649 (-) |  | -2.6600 | 9.65e-93 | 5.05e-91 |
| ENSMUSG00000034401 | Spata6 | protein\_coding | 4:111719984-111829184 (+) |  | -1.2300 | 1.32e-92 | 6.87e-91 |
| ENSMUSG00000033316 | Galnt9 | protein\_coding | 5:110544355-110621380 (+) |  | -2.7500 | 3.94e-92 | 2.05e-90 |
| ENSMUSG00000034557 | Zfyve9 | protein\_coding | 4:108637466-108780798 (-) |  | -2.4100 | 5.36e-92 | 2.78e-90 |
| ENSMUSG00000028859 | Csf3r | protein\_coding | 4:126024550-126044440 (+) |  | -1.1500 | 5.40e-92 | 2.79e-90 |
| ENSMUSG00000001761 | Smo | protein\_coding | 6:29735503-29761365 (+) |  | 2.5800 | 6.48e-92 | 3.34e-90 |
| ENSMUSG00000002289 | Angptl4 | protein\_coding | 17:33773750-33781830 (-) |  | 3.5200 | 7.43e-92 | 3.81e-90 |
| ENSMUSG00000028069 | Gpatch4 | protein\_coding | 3:88043108-88055993 (+) |  | 1.7100 | 1.02e-91 | 5.22e-90 |
| ENSMUSG00000060261 | Gtf2i | protein\_coding | 5:134237834-134314760 (-) |  | 1.1900 | 2.08e-91 | 1.06e-89 |
| ENSMUSG00000026234 | Ncl | protein\_coding | 1:86344719-86359400 (-) |  | 1.5500 | 2.79e-91 | 1.42e-89 |
| ENSMUSG00000033147 | Slc22a15 | protein\_coding | 3:101855776-101924453 (-) |  | -1.1300 | 3.03e-91 | 1.53e-89 |
| ENSMUSG00000056529 | Ptafr | protein\_coding | 4:132564067-132582683 (+) |  | -1.8800 | 3.14e-91 | 1.58e-89 |
| ENSMUSG00000072653 | Zfp783 | transcribed\_unprocessed\_pseudogene | 6:47943171-47955913 (+) |  | 3.5300 | 3.16e-91 | 1.59e-89 |
| ENSMUSG00000042129 | Rassf4 | protein\_coding | 6:116633008-116673952 (-) |  | -0.9240 | 3.61e-91 | 1.81e-89 |
| ENSMUSG00000022901 | Cd86 | protein\_coding | 16:36603869-36666081 (-) |  | -1.9200 | 4.00e-91 | 1.99e-89 |
| ENSMUSG00000058793 | Cds2 | protein\_coding | 2:132263148-132312050 (+) |  | -1.1800 | 4.10e-91 | 2.04e-89 |
| ENSMUSG00000021959 | Lats2 | protein\_coding | 14:57689662-57758388 (-) |  | -1.1600 | 4.28e-91 | 2.12e-89 |
| ENSMUSG00000017176 | Nt5c3b | protein\_coding | 11:100422321-100441808 (-) |  | 2.5400 | 5.05e-91 | 2.50e-89 |
| ENSMUSG00000026074 | Map4k4 | protein\_coding | 1:39900913-40026310 (+) |  | -1.0600 | 5.27e-91 | 2.59e-89 |
| ENSMUSG00000067367 | Lyar | protein\_coding | 5:38220470-38234306 (+) |  | 1.4500 | 5.28e-91 | 2.59e-89 |
| ENSMUSG00000033436 | Armcx2 | protein\_coding | X:134804145-134809221 (-) |  | 2.7700 | 1.19e-90 | 5.81e-89 |
| ENSMUSG00000033066 | Gas7 | protein\_coding | 11:67455437-67688990 (+) |  | -1.7200 | 2.82e-90 | 1.37e-88 |
| ENSMUSG00000036469 | March1 | protein\_coding | 8:65617900-66471637 (+) |  | -2.1400 | 3.03e-90 | 1.47e-88 |
| ENSMUSG00000005981 | Trap1 | protein\_coding | 16:4039971-4077827 (-) |  | 1.2000 | 4.10e-90 | 1.99e-88 |
| ENSMUSG00000031467 | Agpat5 | protein\_coding | 8:18846277-18891361 (+) |  | 1.2100 | 4.72e-90 | 2.28e-88 |
| ENSMUSG00000024187 | Fam234a | protein\_coding | 17:26211822-26244242 (-) |  | -1.6800 | 5.97e-90 | 2.88e-88 |
| ENSMUSG00000032846 | Zswim6 | protein\_coding | 13:107724617-107890064 (-) |  | -1.5600 | 8.31e-90 | 3.99e-88 |
| ENSMUSG00000053801 | Grwd1 | protein\_coding | 7:45825223-45830944 (-) |  | 2.0800 | 1.52e-89 | 7.27e-88 |
| ENSMUSG00000003283 | Hck | protein\_coding | 2:153108468-153151441 (+) |  | -1.6500 | 1.54e-89 | 7.35e-88 |
| ENSMUSG00000028261 | Ndufaf4 | protein\_coding | 4:24898083-24905001 (+) |  | 1.6600 | 1.76e-89 | 8.37e-88 |
| ENSMUSG00000031976 | Urb2 | protein\_coding | 8:124021508-124048505 (+) |  | 1.3300 | 2.27e-89 | 1.08e-87 |
| ENSMUSG00000021175 | Cdca7l | protein\_coding | 12:117804289-117878706 (+) |  | 1.7100 | 2.35e-89 | 1.11e-87 |
| ENSMUSG00000090124 | Ugt1a7c | protein\_coding | 1:88095062-88220002 (+) |  | -1.1400 | 2.49e-89 | 1.17e-87 |
| ENSMUSG00000031353 | Rbbp7 | protein\_coding | X:162760402-162779092 (+) |  | 0.9840 | 3.60e-89 | 1.69e-87 |
| ENSMUSG00000039089 | L3mbtl3 | protein\_coding | 10:26274468-26375971 (-) |  | 1.7400 | 3.92e-89 | 1.84e-87 |
| ENSMUSG00000051498 | Tlr6 | protein\_coding | 5:64952031-64960097 (-) |  | -1.5300 | 3.98e-89 | 1.86e-87 |
| ENSMUSG00000029438 | Bcl7a | protein\_coding | 5:123343834-123374992 (+) |  | 1.9800 | 4.57e-89 | 2.13e-87 |
| ENSMUSG00000057113 | Npm1 | protein\_coding | 11:33152287-33163206 (-) |  | 1.4800 | 5.19e-89 | 2.41e-87 |
| ENSMUSG00000025364 | Pa2g4 | protein\_coding | 10:128557766-128565987 (-) |  | 1.5600 | 5.81e-89 | 2.68e-87 |
| ENSMUSG00000036943 | Rab8b | protein\_coding | 9:66843664-66919687 (-) |  | -0.7080 | 8.76e-89 | 4.04e-87 |
| ENSMUSG00000044477 | Zfand3 | protein\_coding | 17:30004733-30210828 (+) |  | -1.1100 | 1.03e-88 | 4.72e-87 |
| ENSMUSG00000015149 | Sirt2 | protein\_coding | 7:28766735-28788661 (+) |  | -0.9910 | 1.86e-88 | 8.52e-87 |
| ENSMUSG00000024885 | Aldh3b1 | protein\_coding | 19:3913491-3929808 (-) |  | -1.5800 | 1.96e-88 | 8.94e-87 |
| ENSMUSG00000037474 | Dtl | protein\_coding | 1:191537356-191575544 (-) |  | 1.6000 | 2.58e-88 | 1.18e-86 |
| ENSMUSG00000059325 | Hopx | protein\_coding | 5:77086988-77115121 (-) |  | -2.0500 | 2.60e-88 | 1.18e-86 |
| ENSMUSG00000034947 | Tmem106a | protein\_coding | 11:101582242-101591788 (+) |  | -2.2300 | 3.43e-88 | 1.55e-86 |
| ENSMUSG00000040613 | Apobec1 | protein\_coding | 6:122577792-122602444 (-) |  | -2.2500 | 4.59e-88 | 2.07e-86 |
| ENSMUSG00000066357 | Wdr6 | protein\_coding | 9:108572311-108578739 (-) |  | 1.8200 | 5.06e-88 | 2.27e-86 |
| ENSMUSG00000117613 | Gm2629 | lncRNA | 18:15194782-15214696 (+) |  | 2.2100 | 6.04e-88 | 2.71e-86 |
| ENSMUSG00000035493 | Tgfbi | protein\_coding | 13:56609523-56639562 (+) |  | -1.7600 | 6.66e-88 | 2.98e-86 |
| ENSMUSG00000019943 | Atp2b1 | protein\_coding | 10:98914406-99026143 (+) |  | -1.2300 | 8.45e-88 | 3.77e-86 |
| ENSMUSG00000090290 | Tarbp1 | protein\_coding | 8:126425329-126475065 (-) |  | 1.8000 | 2.43e-87 | 1.08e-85 |
| ENSMUSG00000006273 | Atp6v1b2 | protein\_coding | 8:69088646-69113711 (+) |  | -0.7160 | 3.00e-87 | 1.33e-85 |
| ENSMUSG00000051817 | Sox12 | protein\_coding | 2:152393611-152398063 (-) |  | 3.7300 | 3.59e-87 | 1.59e-85 |
| ENSMUSG00000027185 | Nat10 | protein\_coding | 2:103721256-103761270 (-) |  | 1.6000 | 3.61e-87 | 1.59e-85 |
| ENSMUSG00000025007 | Aldh18a1 | protein\_coding | 19:40550257-40588463 (-) |  | 1.8400 | 4.48e-87 | 1.97e-85 |
| ENSMUSG00000028708 | Mknk1 | protein\_coding | 4:115839198-115879250 (+) |  | -0.9180 | 4.56e-87 | 1.99e-85 |
| ENSMUSG00000025995 | Wdr75 | protein\_coding | 1:45795166-45823619 (+) |  | 1.3500 | 1.97e-86 | 8.61e-85 |
| ENSMUSG00000031827 | Cotl1 | protein\_coding | 8:119809222-119840544 (-) |  | -1.3300 | 2.02e-86 | 8.80e-85 |
| ENSMUSG00000051177 | Plcb1 | protein\_coding | 2:134786067-135475258 (+) |  | -1.5400 | 2.32e-86 | 1.01e-84 |
| ENSMUSG00000030156 | Cd69 | protein\_coding | 6:129267325-129275436 (-) |  | 2.8900 | 2.67e-86 | 1.15e-84 |
| ENSMUSG00000024245 | Tmem178 | protein\_coding | 17:80944632-81001816 (+) |  | 4.1000 | 3.25e-86 | 1.40e-84 |
| ENSMUSG00000030589 | Rasgrp4 | protein\_coding | 7:29134851-29153961 (+) |  | -2.1500 | 3.70e-86 | 1.59e-84 |
| ENSMUSG00000046080 | Clec9a | protein\_coding | 6:129408862-129424763 (+) |  | 3.3400 | 4.13e-86 | 1.77e-84 |
| ENSMUSG00000024236 | Svil | protein\_coding | 18:4920540-5119299 (+) |  | -1.3600 | 4.86e-86 | 2.08e-84 |
| ENSMUSG00000040732 | Erg | protein\_coding | 16:95359169-95586593 (-) |  | 5.7900 | 5.06e-86 | 2.16e-84 |
| ENSMUSG00000021365 | Nedd9 | protein\_coding | 13:41309581-41487362 (-) |  | -1.2200 | 5.27e-86 | 2.24e-84 |
| ENSMUSG00000057554 | Lgals8 | protein\_coding | 13:12439415-12464944 (-) |  | -1.1100 | 5.88e-86 | 2.49e-84 |
| ENSMUSG00000023150 | Ivns1abp | protein\_coding | 1:151344477-151364422 (+) |  | 1.2900 | 6.17e-86 | 2.61e-84 |
| ENSMUSG00000042210 | Abhd14a | protein\_coding | 9:106440051-106447678 (-) |  | 2.7600 | 7.28e-86 | 3.07e-84 |
| ENSMUSG00000050377 | Il31ra | protein\_coding | 13:112519898-112594360 (-) |  | 2.7100 | 7.81e-86 | 3.29e-84 |
| ENSMUSG00000040522 | Tlr8 | protein\_coding | X:167242696-167264329 (-) |  | -2.1800 | 9.20e-86 | 3.86e-84 |
| ENSMUSG00000039232 | Stx11 | protein\_coding | 10:12938209-12964298 (-) |  | -1.2100 | 1.14e-85 | 4.76e-84 |
| ENSMUSG00000033706 | Smyd5 | protein\_coding | 6:85431989-85446435 (+) |  | 1.6100 | 1.65e-85 | 6.88e-84 |
| ENSMUSG00000001025 | S100a6 | protein\_coding | 3:90612882-90624181 (+) |  | -1.9700 | 2.71e-85 | 1.13e-83 |
| ENSMUSG00000029771 | Irf5 | protein\_coding | 6:29526625-29541871 (+) |  | -1.4300 | 3.32e-85 | 1.38e-83 |
| ENSMUSG00000053332 | Gas5 | lncRNA | 1:161034422-161038539 (+) |  | 1.4500 | 4.30e-85 | 1.78e-83 |
| ENSMUSG00000045094 | Arhgef37 | protein\_coding | 18:61491657-61536594 (-) |  | -3.0600 | 4.40e-85 | 1.81e-83 |
| ENSMUSG00000030681 | Mvp | protein\_coding | 7:126986860-127014621 (-) |  | -1.1800 | 4.84e-85 | 1.99e-83 |
| ENSMUSG00000037029 | Zfp146 | protein\_coding | 7:30161269-30169750 (-) |  | 0.8340 | 7.54e-85 | 3.09e-83 |
| ENSMUSG00000024841 | Eif1ad | protein\_coding | 19:5366741-5371526 (+) |  | 0.9350 | 8.58e-85 | 3.51e-83 |
| ENSMUSG00000060275 | Nrg2 | protein\_coding | 18:36017652-36197380 (-) |  | 1.7000 | 1.13e-84 | 4.59e-83 |
| ENSMUSG00000036067 | Slc2a6 | protein\_coding | 2:27021363-27027998 (-) |  | -1.3400 | 1.15e-84 | 4.66e-83 |
| ENSMUSG00000043439 | Epop | protein\_coding | 11:97627389-97629702 (-) |  | 2.9300 | 1.24e-84 | 5.04e-83 |
| ENSMUSG00000008393 | Carhsp1 | protein\_coding | 16:8658580-8672155 (-) |  | -1.4200 | 1.25e-84 | 5.07e-83 |
| ENSMUSG00000095609 | Gm21188 | protein\_coding | 13:120034605-120052194 (-) |  | -1.4800 | 1.67e-84 | 6.73e-83 |
| ENSMUSG00000033192 | Lpcat2 | protein\_coding | 8:92855339-92919279 (+) |  | -3.0400 | 1.69e-84 | 6.79e-83 |
| ENSMUSG00000079056 | Kcnip3 | protein\_coding | 2:127456498-127522094 (-) |  | 4.8100 | 1.95e-84 | 7.82e-83 |
| ENSMUSG00000058163 | Gm5431 | protein\_coding | 11:48887422-48902214 (-) |  | -2.7300 | 2.50e-84 | 1.00e-82 |
| ENSMUSG00000031657 | Heatr3 | protein\_coding | 8:88137855-88172027 (+) |  | 1.4300 | 3.13e-84 | 1.25e-82 |
| ENSMUSG00000000561 | Wdr77 | protein\_coding | 3:105959369-105970037 (+) |  | 1.1300 | 3.14e-84 | 1.25e-82 |
| ENSMUSG00000019122 | Ccl9 | protein\_coding | 11:83572919-83578636 (-) |  | -1.6800 | 3.63e-84 | 1.44e-82 |
| ENSMUSG00000027184 | Caprin1 | protein\_coding | 2:103762941-103797649 (-) |  | 0.6190 | 3.91e-84 | 1.55e-82 |
| ENSMUSG00000024937 | Ehbp1l1 | protein\_coding | 19:5707376-5726317 (-) |  | -1.0400 | 5.49e-84 | 2.17e-82 |
| ENSMUSG00000028572 | Hook1 | protein\_coding | 4:95967240-96025413 (+) |  | 3.0900 | 5.69e-84 | 2.24e-82 |
| ENSMUSG00000035697 | Arhgap45 | protein\_coding | 10:80016653-80031472 (+) |  | -0.9150 | 5.73e-84 | 2.25e-82 |
| ENSMUSG00000074794 | Arrdc3 | protein\_coding | 13:80883384-80896042 (+) |  | -1.3000 | 6.09e-84 | 2.39e-82 |
| ENSMUSG00000085148 | Mir22hg | lncRNA | 11:75461539-75466676 (+) |  | -2.2100 | 8.69e-84 | 3.40e-82 |
| ENSMUSG00000021585 | Cast | protein\_coding | 13:74692368-74808810 (-) |  | -1.0200 | 1.12e-83 | 4.35e-82 |
| ENSMUSG00000028300 | C9orf72 | protein\_coding | 4:35191285-35226175 (-) |  | -1.4400 | 1.17e-83 | 4.57e-82 |
| ENSMUSG00000053931 | Cnn3 | protein\_coding | 3:121426497-121458207 (+) |  | 2.8300 | 1.27e-83 | 4.91e-82 |
| ENSMUSG00000026222 | Sp100 | protein\_coding | 1:85649988-85709998 (+) |  | -1.2400 | 1.64e-83 | 6.34e-82 |
| ENSMUSG00000067586 | S1pr3 | protein\_coding | 13:51408639-51422797 (+) |  | 2.9200 | 1.75e-83 | 6.74e-82 |
| ENSMUSG00000030427 | Lilra6 | protein\_coding | 7:3908280-3915503 (-) |  | -2.0600 | 2.29e-83 | 8.81e-82 |
| ENSMUSG00000048612 | Myof | protein\_coding | 19:37899036-38043577 (-) |  | -2.5800 | 2.33e-83 | 8.95e-82 |
| ENSMUSG00000038121 | Fam210a | protein\_coding | 18:68260187-68300333 (-) |  | 1.0400 | 2.34e-83 | 8.98e-82 |
| ENSMUSG00000022831 | Hcls1 | protein\_coding | 16:36934983-36963212 (+) |  | -0.9220 | 2.60e-83 | 9.93e-82 |
| ENSMUSG00000021270 | Hsp90aa1 | protein\_coding | 12:110690605-110702728 (-) |  | 1.5300 | 3.16e-83 | 1.20e-81 |
| ENSMUSG00000026730 | Pter | protein\_coding | 2:12924041-13003455 (+) |  | 2.4700 | 3.36e-83 | 1.28e-81 |
| ENSMUSG00000028559 | Osbpl9 | protein\_coding | 4:109061145-109202272 (-) |  | -1.0300 | 3.62e-83 | 1.37e-81 |
| ENSMUSG00000000753 | Serpinf1 | protein\_coding | 11:75409769-75422701 (-) |  | 2.0900 | 5.06e-83 | 1.91e-81 |
| ENSMUSG00000002006 | Pdzd4 | protein\_coding | X:73793359-73824969 (-) |  | 3.2800 | 5.83e-83 | 2.20e-81 |
| ENSMUSG00000006728 | Cdk4 | protein\_coding | 10:127063534-127067920 (+) |  | 1.3200 | 9.31e-83 | 3.50e-81 |
| ENSMUSG00000037376 | Trmt6 | protein\_coding | 2:132804207-132816055 (-) |  | 0.9690 | 1.02e-82 | 3.82e-81 |
| ENSMUSG00000028273 | Pdlim5 | protein\_coding | 3:142239590-142395696 (-) |  | -0.9990 | 1.10e-82 | 4.12e-81 |
| ENSMUSG00000074419 | Gm15448 | protein\_coding | 7:3816781-3825687 (-) |  | -2.2900 | 1.23e-82 | 4.59e-81 |
| ENSMUSG00000063268 | Parp10 | protein\_coding | 15:76231174-76243441 (-) |  | -1.2200 | 1.35e-82 | 5.05e-81 |
| ENSMUSG00000022793 | B4galt4 | protein\_coding | 16:38742264-38769049 (+) |  | 2.1800 | 1.44e-82 | 5.35e-81 |
| ENSMUSG00000050335 | Lgals3 | protein\_coding | 14:47367751-47386160 (+) |  | -1.8300 | 1.51e-82 | 5.58e-81 |
| ENSMUSG00000032109 | Nlrx1 | protein\_coding | 9:44252717-44268599 (-) |  | -1.0700 | 1.66e-82 | 6.12e-81 |
| ENSMUSG00000040234 | Tm7sf3 | protein\_coding | 6:146602352-146642824 (-) |  | 1.0300 | 2.11e-82 | 7.77e-81 |
| ENSMUSG00000029233 | Srd5a3 | protein\_coding | 5:76140271-76155504 (+) |  | -0.8880 | 2.35e-82 | 8.66e-81 |
| ENSMUSG00000019818 | Cd164 | protein\_coding | 10:41519414-41531048 (+) |  | 0.8410 | 2.59e-82 | 9.51e-81 |
| ENSMUSG00000024006 | Stk38 | protein\_coding | 17:28970880-29007995 (-) |  | -1.4100 | 2.91e-82 | 1.07e-80 |
| ENSMUSG00000042659 | Arrdc4 | protein\_coding | 7:68736995-68749241 (-) |  | -1.6100 | 3.03e-82 | 1.11e-80 |
| ENSMUSG00000002332 | Dhrs1 | protein\_coding | 14:55739020-55745690 (-) |  | -1.0400 | 3.67e-82 | 1.34e-80 |
| ENSMUSG00000029561 | Oasl2 | protein\_coding | 5:114896936-114912234 (+) |  | -3.3900 | 4.43e-82 | 1.61e-80 |
| ENSMUSG00000038612 | Mcl1 | protein\_coding | 3:95658788-95663176 (+) |  | -1.3400 | 8.00e-82 | 2.90e-80 |
| ENSMUSG00000024300 | Myo1f | protein\_coding | 17:33555707-33607764 (+) |  | -1.0200 | 1.12e-81 | 4.05e-80 |
| ENSMUSG00000024778 | Fas | protein\_coding | 19:34290666-34327772 (+) |  | -2.4500 | 1.15e-81 | 4.16e-80 |
| ENSMUSG00000039153 | Runx2 | protein\_coding | 17:44495987-44814797 (-) |  | 2.0000 | 1.31e-81 | 4.73e-80 |
| ENSMUSG00000024053 | Emilin2 | protein\_coding | 17:71252172-71311978 (-) |  | -1.6200 | 1.61e-81 | 5.79e-80 |
| ENSMUSG00000032870 | Smap2 | protein\_coding | 4:120968317-121017247 (-) |  | -0.8250 | 1.99e-81 | 7.13e-80 |
| ENSMUSG00000025050 | Pcgf6 | protein\_coding | 19:47033617-47050906 (-) |  | 2.3900 | 2.22e-81 | 7.95e-80 |
| ENSMUSG00000032431 | Crtap | protein\_coding | 9:114375134-114390675 (-) |  | 1.4400 | 3.08e-81 | 1.10e-79 |
| ENSMUSG00000024066 | Xdh | protein\_coding | 17:73883895-73950196 (-) |  | -1.6000 | 3.40e-81 | 1.21e-79 |
| ENSMUSG00000032508 | Myd88 | protein\_coding | 9:119335934-119341411 (-) |  | -1.0500 | 3.68e-81 | 1.31e-79 |
| ENSMUSG00000047879 | Usp14 | protein\_coding | 18:9993066-10045119 (-) |  | 0.9720 | 3.75e-81 | 1.33e-79 |
| ENSMUSG00000033335 | Dnm2 | protein\_coding | 9:21424908-21507759 (+) |  | -0.6180 | 4.17e-81 | 1.47e-79 |
| ENSMUSG00000029490 | Mfsd7a | protein\_coding | 5:108441054-108449100 (-) |  | -1.6800 | 5.17e-81 | 1.82e-79 |
| ENSMUSG00000061731 | Ext1 | protein\_coding | 15:53064038-53346159 (-) |  | -1.2200 | 5.36e-81 | 1.89e-79 |
| ENSMUSG00000059883 | Irak4 | protein\_coding | 15:94543643-94581815 (+) |  | -0.9030 | 7.40e-81 | 2.59e-79 |
| ENSMUSG00000019996 | Map7 | protein\_coding | 10:20148471-20281590 (+) |  | 2.2100 | 7.85e-81 | 2.75e-79 |
| ENSMUSG00000038147 | Cd84 | protein\_coding | 1:171839697-171890718 (+) |  | -1.8200 | 7.89e-81 | 2.76e-79 |
| ENSMUSG00000025980 | Hspd1 | protein\_coding | 1:55077835-55088243 (-) |  | 1.7100 | 8.50e-81 | 2.96e-79 |
| ENSMUSG00000026628 | Atf3 | protein\_coding | 1:191170296-191218039 (-) |  | -3.7400 | 9.52e-81 | 3.31e-79 |
| ENSMUSG00000017466 | Timp2 | protein\_coding | 11:118301069-118355740 (-) |  | -2.9200 | 1.11e-80 | 3.85e-79 |
| ENSMUSG00000033166 | Dis3 | protein\_coding | 14:99075206-99099770 (-) |  | 1.2600 | 1.24e-80 | 4.30e-79 |
| ENSMUSG00000056498 | Tmem154 | protein\_coding | 3:84666192-84704575 (+) |  | -1.8400 | 1.69e-80 | 5.84e-79 |
| ENSMUSG00000025357 | Dgka | protein\_coding | 10:128720134-128744855 (-) |  | -1.3900 | 1.82e-80 | 6.26e-79 |
| ENSMUSG00000022885 | St6gal1 | protein\_coding | 16:23224740-23360350 (+) |  | -3.2700 | 1.90e-80 | 6.52e-79 |
| ENSMUSG00000030079 | Ruvbl1 | protein\_coding | 6:88465409-88497572 (+) |  | 1.2300 | 2.04e-80 | 6.97e-79 |
| ENSMUSG00000035725 | Prkx | protein\_coding | X:77761411-77796278 (-) |  | -1.2400 | 2.13e-80 | 7.28e-79 |
| ENSMUSG00000020741 | Cluh | protein\_coding | 11:74649495-74670847 (+) |  | 1.3500 | 3.73e-80 | 1.27e-78 |
| ENSMUSG00000034652 | Cd300a | protein\_coding | 11:114890041-114904654 (+) |  | -1.5200 | 4.06e-80 | 1.38e-78 |
| ENSMUSG00000027695 | Pld1 | protein\_coding | 3:27938695-28133362 (+) |  | -1.4600 | 6.28e-80 | 2.13e-78 |
| ENSMUSG00000068036 | Afdn | protein\_coding | 17:13760539-13906150 (+) |  | -1.5000 | 7.57e-80 | 2.57e-78 |
| ENSMUSG00000021048 | Mthfd1 | protein\_coding | 12:76255298-76319803 (+) |  | 1.5400 | 8.84e-80 | 2.99e-78 |
| ENSMUSG00000001020 | S100a4 | protein\_coding | 3:90603771-90606045 (+) |  | -2.1200 | 9.69e-80 | 3.27e-78 |
| ENSMUSG00000025869 | Nop16 | protein\_coding | 13:54584185-54590090 (-) |  | 1.5000 | 1.23e-79 | 4.13e-78 |
| ENSMUSG00000068587 | Mgam | protein\_coding | 6:40628831-40769123 (+) |  | 3.6200 | 1.31e-79 | 4.41e-78 |
| ENSMUSG00000021595 | Nsun2 | protein\_coding | 13:69533746-69635780 (+) |  | 1.2300 | 1.38e-79 | 4.62e-78 |
| ENSMUSG00000040209 | Zfp704 | protein\_coding | 3:9427020-9610085 (-) |  | 1.6400 | 1.39e-79 | 4.63e-78 |
| ENSMUSG00000011257 | Pabpc4 | protein\_coding | 4:123262351-123298925 (+) |  | 1.6700 | 1.58e-79 | 5.26e-78 |
| ENSMUSG00000033032 | Afap1l1 | protein\_coding | 18:61730261-61786702 (-) |  | 2.3800 | 1.58e-79 | 5.27e-78 |
| ENSMUSG00000021392 | Nol8 | protein\_coding | 13:49653078-49679016 (+) |  | 0.9770 | 1.69e-79 | 5.61e-78 |
| ENSMUSG00000021133 | Susd6 | protein\_coding | 12:80790510-80880835 (+) |  | -0.8990 | 2.02e-79 | 6.70e-78 |
| ENSMUSG00000028717 | Tal1 | protein\_coding | 4:115056426-115071755 (+) |  | 2.4900 | 2.43e-79 | 8.03e-78 |
| ENSMUSG00000029772 | Ahcyl2 | protein\_coding | 6:29768011-29912310 (+) |  | -1.1700 | 2.75e-79 | 9.07e-78 |
| ENSMUSG00000020798 | Spns3 | protein\_coding | 11:72494919-72550506 (-) |  | 2.5100 | 4.04e-79 | 1.33e-77 |
| ENSMUSG00000027864 | Ptgfrn | protein\_coding | 3:101040232-101110278 (-) |  | -3.1200 | 4.66e-79 | 1.53e-77 |
| ENSMUSG00000058799 | Nap1l1 | protein\_coding | 10:111473223-111498150 (+) |  | 0.8140 | 4.76e-79 | 1.56e-77 |
| ENSMUSG00000042978 | Sbk1 | protein\_coding | 7:126248862-126295016 (+) |  | -2.0000 | 5.76e-79 | 1.88e-77 |
| ENSMUSG00000037278 | Tmem97 | protein\_coding | 11:78541817-78550777 (-) |  | 1.5400 | 6.08e-79 | 1.98e-77 |
| ENSMUSG00000003154 | Foxj2 | protein\_coding | 6:122819914-122845366 (+) |  | -1.7000 | 8.76e-79 | 2.85e-77 |
| ENSMUSG00000038578 | Susd1 | protein\_coding | 4:59314683-59438633 (-) |  | 1.1800 | 8.85e-79 | 2.87e-77 |
| ENSMUSG00000024170 | Telo2 | protein\_coding | 17:25099570-25115967 (-) |  | 1.3200 | 8.92e-79 | 2.89e-77 |
| ENSMUSG00000042675 | Ypel3 | protein\_coding | 7:126776955-126780514 (+) |  | -2.1300 | 9.40e-79 | 3.04e-77 |
| ENSMUSG00000031662 | Snx20 | protein\_coding | 8:88626563-88636128 (-) |  | -1.3000 | 9.41e-79 | 3.04e-77 |
| ENSMUSG00000002885 | Adgre5 | protein\_coding | 8:83723251-83741326 (-) |  | -1.9200 | 1.14e-78 | 3.67e-77 |
| ENSMUSG00000025351 | Cd63 | protein\_coding | 10:128900989-128912822 (+) |  | 3.4000 | 1.22e-78 | 3.90e-77 |
| ENSMUSG00000000934 | Top1mt | protein\_coding | 15:75657035-75678800 (-) |  | 2.4700 | 1.66e-78 | 5.32e-77 |
| ENSMUSG00000023942 | Slc29a1 | protein\_coding | 17:45585200-45599606 (-) |  | 1.2600 | 1.71e-78 | 5.46e-77 |
| ENSMUSG00000028550 | Atg4c | protein\_coding | 4:99193934-99259787 (+) |  | -1.3600 | 2.56e-78 | 8.19e-77 |
| ENSMUSG00000000594 | Gm2a | protein\_coding | 11:55098115-55113029 (+) |  | -0.7820 | 2.74e-78 | 8.73e-77 |
| ENSMUSG00000021193 | Pitrm1 | protein\_coding | 13:6548149-6580515 (+) |  | 1.2300 | 3.15e-78 | 1.00e-76 |
| ENSMUSG00000032086 | Bace1 | protein\_coding | 9:45838580-45864399 (+) |  | 1.5300 | 3.64e-78 | 1.16e-76 |
| ENSMUSG00000024675 | Ms4a4c | protein\_coding | 19:11404770-11427246 (+) |  | -2.1400 | 4.50e-78 | 1.43e-76 |
| ENSMUSG00000030083 | Abtb1 | protein\_coding | 6:88835914-88841984 (-) |  | -1.2000 | 4.90e-78 | 1.55e-76 |
| ENSMUSG00000020766 | Galk1 | protein\_coding | 11:116008457-116012719 (-) |  | 1.7200 | 6.44e-78 | 2.03e-76 |
| ENSMUSG00000053338 | Tarm1 | protein\_coding | 7:3486500-3502624 (-) |  | -2.5900 | 7.01e-78 | 2.20e-76 |
| ENSMUSG00000006411 | Nectin4 | protein\_coding | 1:171370099-171388598 (+) |  | -2.3300 | 7.07e-78 | 2.22e-76 |
| ENSMUSG00000015837 | Sqstm1 | protein\_coding | 11:50199366-50210827 (-) |  | -0.7230 | 7.08e-78 | 2.22e-76 |
| ENSMUSG00000004612 | Nkg7 | protein\_coding | 7:43437073-43438249 (+) |  | 4.2600 | 7.53e-78 | 2.35e-76 |
| ENSMUSG00000005370 | Msh6 | protein\_coding | 17:87975062-87990892 (+) |  | 1.2600 | 9.87e-78 | 3.08e-76 |
| ENSMUSG00000030541 | Idh2 | protein\_coding | 7:80094846-80115392 (-) |  | 1.2300 | 1.12e-77 | 3.48e-76 |
| ENSMUSG00000026558 | Uck2 | protein\_coding | 1:167222883-167285320 (-) |  | 1.3200 | 1.16e-77 | 3.59e-76 |
| ENSMUSG00000031939 | Taf1d | protein\_coding | 9:15306214-15316991 (+) |  | 1.0200 | 1.22e-77 | 3.77e-76 |
| ENSMUSG00000042215 | Bag2 | protein\_coding | 1:33745484-33757795 (-) |  | 4.1800 | 1.42e-77 | 4.39e-76 |
| ENSMUSG00000025591 | Tma16 | protein\_coding | 8:66473118-66486530 (-) |  | 1.6900 | 1.56e-77 | 4.81e-76 |
| ENSMUSG00000039616 | Mocos | protein\_coding | 18:24653691-24701575 (+) |  | -1.7700 | 1.86e-77 | 5.72e-76 |
| ENSMUSG00000022471 | Xrcc6 | protein\_coding | 15:81987835-82040085 (+) |  | 1.0100 | 2.79e-77 | 8.57e-76 |
| ENSMUSG00000027994 | Mcub | protein\_coding | 3:129914960-129970206 (-) |  | -2.0900 | 2.94e-77 | 9.01e-76 |
| ENSMUSG00000027843 | Ptpn22 | protein\_coding | 3:103859795-103912247 (+) |  | -1.2000 | 2.97e-77 | 9.10e-76 |
| ENSMUSG00000047798 | Cd300lf | protein\_coding | 11:115116214-115133992 (-) |  | -2.2600 | 3.00e-77 | 9.17e-76 |
| ENSMUSG00000031823 | Zdhhc7 | protein\_coding | 8:120080890-120101482 (-) |  | -0.7550 | 3.72e-77 | 1.13e-75 |
| ENSMUSG00000023961 | Enpp4 | protein\_coding | 17:44096308-44105809 (-) |  | 2.8600 | 5.25e-77 | 1.60e-75 |
| ENSMUSG00000050075 | Gpr171 | protein\_coding | 3:59096448-59101821 (-) |  | 3.0300 | 5.31e-77 | 1.61e-75 |
| ENSMUSG00000098112 | Bin2 | protein\_coding | 15:100641077-100669553 (-) |  | -0.9720 | 6.21e-77 | 1.88e-75 |
| ENSMUSG00000052160 | Pld4 | protein\_coding | 12:112760655-112768990 (+) |  | -1.3100 | 6.48e-77 | 1.96e-75 |
| ENSMUSG00000029413 | Naaa | protein\_coding | 5:92257659-92278170 (-) |  | -1.6200 | 8.11e-77 | 2.45e-75 |
| ENSMUSG00000071708 | Sms | protein\_coding | X:157443855-157492287 (-) |  | 0.9880 | 9.01e-77 | 2.71e-75 |
| ENSMUSG00000058624 | Gda | protein\_coding | 19:21391307-21473445 (-) |  | -1.4700 | 1.12e-76 | 3.37e-75 |
| ENSMUSG00000036155 | Mgat5 | protein\_coding | 1:127205015-127488336 (+) |  | 1.4700 | 1.21e-76 | 3.64e-75 |
| ENSMUSG00000078771 | Evi2a | protein\_coding | 11:79526560-79530609 (-) |  | -2.3100 | 1.45e-76 | 4.35e-75 |
| ENSMUSG00000052102 | Gnpda1 | protein\_coding | 18:38327535-38339003 (-) |  | -1.2100 | 1.70e-76 | 5.09e-75 |
| ENSMUSG00000046879 | Irgm1 | protein\_coding | 11:48861968-48871683 (-) |  | -1.2300 | 1.97e-76 | 5.87e-75 |
| ENSMUSG00000035711 | Dok3 | protein\_coding | 13:55523231-55529296 (-) |  | -0.9840 | 2.11e-76 | 6.29e-75 |
| ENSMUSG00000030214 | Plbd1 | protein\_coding | 6:136612070-136661928 (-) |  | -3.0200 | 2.26e-76 | 6.73e-75 |
| ENSMUSG00000047749 | Zc3hav1l | protein\_coding | 6:38287396-38299259 (-) |  | 2.0300 | 2.51e-76 | 7.44e-75 |
| ENSMUSG00000070390 | Nlrp1b | protein\_coding | 11:71153102-71230733 (-) |  | -2.4000 | 3.02e-76 | 8.93e-75 |
| ENSMUSG00000005800 | Mmp8 | protein\_coding | 9:7558456-7568485 (+) |  | -2.7700 | 4.38e-76 | 1.30e-74 |
| ENSMUSG00000032175 | Tyk2 | protein\_coding | 9:21104068-21131243 (-) |  | -1.0200 | 6.68e-76 | 1.97e-74 |
| ENSMUSG00000004933 | Matk | protein\_coding | 10:81252935-81263365 (+) |  | 1.3100 | 9.21e-76 | 2.71e-74 |
| ENSMUSG00000033307 | Mif | protein\_coding | 10:75859353-75860240 (-) |  | 2.3000 | 9.95e-76 | 2.92e-74 |
| ENSMUSG00000032724 | Abtb2 | protein\_coding | 2:103566310-103718423 (+) |  | -2.6500 | 1.12e-75 | 3.27e-74 |
| ENSMUSG00000073678 | Pgap1 | protein\_coding | 1:54472994-54557684 (-) |  | -2.5100 | 1.45e-75 | 4.24e-74 |
| ENSMUSG00000015846 | Rxra | protein\_coding | 2:27676440-27762957 (+) |  | -1.8100 | 2.38e-75 | 6.92e-74 |
| ENSMUSG00000055322 | Tns1 | protein\_coding | 1:73910231-74124449 (-) |  | 3.2500 | 2.38e-75 | 6.92e-74 |
| ENSMUSG00000040658 | Dnph1 | protein\_coding | 17:46496711-46499624 (+) |  | 1.8600 | 2.67e-75 | 7.77e-74 |
| ENSMUSG00000027506 | Tpd52 | protein\_coding | 3:8925593-9004723 (-) |  | -1.2100 | 3.12e-75 | 9.05e-74 |
| ENSMUSG00000018500 | Adora2b | protein\_coding | 11:62248984-62266453 (+) |  | -1.5100 | 3.50e-75 | 1.02e-73 |
| ENSMUSG00000042462 | Dctpp1 | protein\_coding | 7:127256959-127260709 (-) |  | 1.2500 | 4.24e-75 | 1.23e-73 |
| ENSMUSG00000035960 | Apex1 | protein\_coding | 14:50924968-50927139 (+) |  | 1.6400 | 4.94e-75 | 1.42e-73 |
| ENSMUSG00000014496 | Ankrd28 | protein\_coding | 14:31698768-31830651 (-) |  | 1.2000 | 5.34e-75 | 1.54e-73 |
| ENSMUSG00000018548 | Trim37 | protein\_coding | 11:87127077-87220683 (+) |  | 1.0800 | 7.13e-75 | 2.05e-73 |
| ENSMUSG00000039960 | Rhou | protein\_coding | 8:123653929-123663884 (+) |  | -1.8600 | 7.81e-75 | 2.24e-73 |
| ENSMUSG00000071644 | Eef1g | protein\_coding | 19:8967041-8978479 (+) |  | 1.3600 | 9.13e-75 | 2.62e-73 |
| ENSMUSG00000031444 | F10 | protein\_coding | 8:13037308-13056676 (+) |  | -1.4700 | 9.60e-75 | 2.74e-73 |
| ENSMUSG00000003824 | Syce2 | protein\_coding | 8:84872111-84888221 (+) |  | 1.5800 | 1.01e-74 | 2.87e-73 |
| ENSMUSG00000034220 | Gpc1 | protein\_coding | 1:92831645-92860779 (+) |  | 1.5800 | 1.01e-74 | 2.89e-73 |
| ENSMUSG00000021876 | Rnase4 | protein\_coding | 14:51091077-51106151 (+) |  | -2.1800 | 1.17e-74 | 3.34e-73 |
| ENSMUSG00000027075 | Slc43a1 | protein\_coding | 2:84838850-84863594 (+) |  | 4.1900 | 1.25e-74 | 3.55e-73 |
| ENSMUSG00000019866 | Crybg1 | protein\_coding | 10:43950636-44148853 (-) |  | -2.0300 | 1.30e-74 | 3.68e-73 |
| ENSMUSG00000022438 | Parvb | protein\_coding | 15:84232043-84315688 (+) |  | -1.6200 | 1.40e-74 | 3.94e-73 |
| ENSMUSG00000040945 | Rcc2 | protein\_coding | 4:140700541-140723220 (+) |  | 1.2100 | 1.46e-74 | 4.12e-73 |
| ENSMUSG00000026921 | Egfl7 | protein\_coding | 2:26580014-26593120 (+) |  | 3.5900 | 1.56e-74 | 4.39e-73 |
| ENSMUSG00000029364 | Wsb2 | protein\_coding | 5:117357304-117378601 (+) |  | -1.0800 | 1.88e-74 | 5.28e-73 |
| ENSMUSG00000034786 | Gpsm3 | protein\_coding | 17:34589806-34591754 (+) |  | -1.0200 | 2.33e-74 | 6.54e-73 |
| ENSMUSG00000032353 | Tmed3 | protein\_coding | 9:89699206-89705068 (-) |  | 1.2300 | 2.37e-74 | 6.64e-73 |
| ENSMUSG00000037580 | Gch1 | protein\_coding | 14:47153895-47189413 (-) |  | -1.7700 | 2.65e-74 | 7.41e-73 |
| ENSMUSG00000055805 | Fmnl1 | protein\_coding | 11:103171107-103198901 (+) |  | -0.9810 | 2.76e-74 | 7.70e-73 |
| ENSMUSG00000068039 | Tcp1 | protein\_coding | 17:12915701-12925067 (+) |  | 0.9000 | 3.10e-74 | 8.62e-73 |
| ENSMUSG00000031207 | Msn | protein\_coding | X:96096042-96168552 (+) |  | -0.8190 | 3.14e-74 | 8.72e-73 |
| ENSMUSG00000035783 | Acta2 | protein\_coding | 19:34241090-34255590 (-) |  | -3.1100 | 3.46e-74 | 9.60e-73 |
| ENSMUSG00000019256 | Ahr | protein\_coding | 12:35497974-35535038 (-) |  | -1.5800 | 3.92e-74 | 1.08e-72 |
| ENSMUSG00000020918 | Kat2a | protein\_coding | 11:100704746-100712465 (-) |  | 1.2200 | 7.09e-74 | 1.96e-72 |
| ENSMUSG00000021703 | Serinc5 | protein\_coding | 13:92611091-92711947 (+) |  | -1.3000 | 7.12e-74 | 1.96e-72 |
| ENSMUSG00000024677 | Ms4a6b | protein\_coding | 19:11516512-11531256 (+) |  | -1.6200 | 8.09e-74 | 2.23e-72 |
| ENSMUSG00000032440 | Tgfbr2 | protein\_coding | 9:116084293-116175360 (-) |  | -1.7000 | 9.26e-74 | 2.54e-72 |
| ENSMUSG00000022018 | Rgcc | protein\_coding | 14:79288756-79301645 (-) |  | 3.7300 | 9.66e-74 | 2.65e-72 |
| ENSMUSG00000001768 | Rin2 | protein\_coding | 2:145675215-145887616 (+) |  | -1.8800 | 9.76e-74 | 2.67e-72 |
| ENSMUSG00000013707 | Tnfaip8l2 | protein\_coding | 3:95139521-95142360 (-) |  | -1.2500 | 1.03e-73 | 2.81e-72 |
| ENSMUSG00000057497 | Fam136a | protein\_coding | 6:86365646-86370058 (+) |  | 1.9800 | 1.06e-73 | 2.88e-72 |
| ENSMUSG00000027007 | Itprid2 | protein\_coding | 2:79635352-79672966 (+) |  | -0.8880 | 1.16e-73 | 3.16e-72 |
| ENSMUSG00000058355 | Abce1 | protein\_coding | 8:79683462-79711740 (-) |  | 1.1000 | 1.34e-73 | 3.64e-72 |
| ENSMUSG00000093765 | Gm20658 | lncRNA | 12:116102769-116138648 (-) |  | -1.8100 | 1.35e-73 | 3.65e-72 |
| ENSMUSG00000081603 | Gm14681 | processed\_pseudogene | X:66778442-66778852 (-) |  | 1.4400 | 1.65e-73 | 4.46e-72 |
| ENSMUSG00000030557 | Mef2a | protein\_coding | 7:67231163-67372858 (-) |  | -1.4000 | 1.94e-73 | 5.25e-72 |
| ENSMUSG00000036606 | Plxnb2 | protein\_coding | 15:89155549-89180788 (-) |  | -1.2900 | 2.85e-73 | 7.70e-72 |
| ENSMUSG00000021306 | Gpr137b | protein\_coding | 13:13357620-13394014 (-) |  | -2.7400 | 2.96e-73 | 7.96e-72 |
| ENSMUSG00000044149 | Nkrf | protein\_coding | X:36887540-36903513 (-) |  | 1.4700 | 6.11e-73 | 1.64e-71 |
| ENSMUSG00000063193 | Cd300lb | protein\_coding | 11:114922781-114934386 (-) |  | -1.4200 | 7.56e-73 | 2.03e-71 |
| ENSMUSG00000024835 | Coro1b | protein\_coding | 19:4148619-4154035 (+) |  | -0.9490 | 8.50e-73 | 2.28e-71 |
| ENSMUSG00000022755 | Adgrg7 | protein\_coding | 16:56724609-56795855 (-) |  | 4.6000 | 1.11e-72 | 2.97e-71 |
| ENSMUSG00000004040 | Stat3 | protein\_coding | 11:100885098-100939540 (-) |  | -1.1100 | 1.29e-72 | 3.45e-71 |
| ENSMUSG00000001995 | Sipa1l2 | protein\_coding | 8:125418063-125569808 (-) |  | -1.6100 | 2.13e-72 | 5.68e-71 |
| ENSMUSG00000083307 | AA414768 | processed\_pseudogene | X:12936872-12938128 (+) |  | -2.3500 | 2.15e-72 | 5.73e-71 |
| ENSMUSG00000025225 | Nfkb2 | protein\_coding | 19:46304320-46312385 (+) |  | -1.2300 | 2.46e-72 | 6.53e-71 |
| ENSMUSG00000036478 | Btg1 | protein\_coding | 10:96617006-96622809 (+) |  | -1.5600 | 2.53e-72 | 6.69e-71 |
| ENSMUSG00000028657 | Ppt1 | protein\_coding | 4:122836242-122859175 (+) |  | -0.8570 | 3.10e-72 | 8.21e-71 |
| ENSMUSG00000032812 | Arap1 | protein\_coding | 7:101348067-101412586 (+) |  | -1.1400 | 3.13e-72 | 8.27e-71 |
| ENSMUSG00000027478 | Dnmt3b | protein\_coding | 2:153649450-153687730 (+) |  | 2.0100 | 3.25e-72 | 8.58e-71 |
| ENSMUSG00000039145 | Camk1d | protein\_coding | 2:5293457-5714515 (-) |  | -2.0700 | 3.67e-72 | 9.65e-71 |
| ENSMUSG00000024413 | Npc1 | protein\_coding | 18:12189692-12236400 (-) |  | -1.1000 | 4.63e-72 | 1.22e-70 |
| ENSMUSG00000037070 | Rbmxl1 | protein\_coding | 8:78505269-78508898 (-) |  | 0.9440 | 6.90e-72 | 1.81e-70 |
| ENSMUSG00000031256 | Cstf2 | protein\_coding | X:134059187-134086819 (+) |  | 0.8560 | 9.68e-72 | 2.53e-70 |
| ENSMUSG00000001100 | Poldip2 | protein\_coding | 11:78512193-78522736 (+) |  | 1.0100 | 9.87e-72 | 2.58e-70 |
| ENSMUSG00000035772 | Mrps2 | protein\_coding | 2:28468066-28471178 (+) |  | 0.8670 | 1.00e-71 | 2.62e-70 |
| ENSMUSG00000024359 | Hspa9 | protein\_coding | 18:34937414-34954357 (-) |  | 1.4000 | 1.08e-71 | 2.81e-70 |
| ENSMUSG00000031785 | Adgrg1 | protein\_coding | 8:94974751-95014217 (+) |  | 4.4700 | 1.14e-71 | 2.97e-70 |
| ENSMUSG00000030189 | Ybx3 | protein\_coding | 6:131364855-131388476 (-) |  | 1.2600 | 1.50e-71 | 3.90e-70 |
| ENSMUSG00000027660 | Skil | protein\_coding | 3:31095058-31122577 (+) |  | -1.3000 | 1.90e-71 | 4.91e-70 |
| ENSMUSG00000029922 | Mkrn1 | protein\_coding | 6:39397804-39420462 (-) |  | -0.9250 | 2.10e-71 | 5.44e-70 |
| ENSMUSG00000024220 | Zfp523 | protein\_coding | 17:28177129-28205886 (+) |  | 1.6600 | 2.16e-71 | 5.59e-70 |
| ENSMUSG00000001281 | Itgb7 | protein\_coding | 15:102215995-102231944 (-) |  | -1.1000 | 2.33e-71 | 6.00e-70 |
| ENSMUSG00000027035 | Cers6 | protein\_coding | 2:68861441-69114282 (+) |  | -1.3600 | 2.44e-71 | 6.28e-70 |
| ENSMUSG00000046908 | Ltb4r1 | protein\_coding | 14:55765962-55768494 (+) |  | -1.2100 | 2.60e-71 | 6.67e-70 |
| ENSMUSG00000056612 | Ppp1r14b | protein\_coding | 19:6974968-6977324 (+) |  | 1.3600 | 2.98e-71 | 7.64e-70 |
| ENSMUSG00000046861 | Hectd3 | protein\_coding | 4:116995317-117005277 (+) |  | -0.7380 | 3.56e-71 | 9.12e-70 |
| ENSMUSG00000001416 | Cct3 | protein\_coding | 3:88297116-88321767 (+) |  | 1.2800 | 3.61e-71 | 9.23e-70 |
| ENSMUSG00000020877 | Scrn2 | protein\_coding | 11:97029938-97033958 (+) |  | 2.5900 | 3.63e-71 | 9.25e-70 |
| ENSMUSG00000002699 | Lcp2 | protein\_coding | 11:34046920-34092295 (+) |  | -1.1100 | 3.69e-71 | 9.40e-70 |
| ENSMUSG00000022500 | Litaf | protein\_coding | 16:10959275-11066157 (-) |  | -0.8960 | 4.12e-71 | 1.05e-69 |
| ENSMUSG00000028434 | Epb41l4b | protein\_coding | 4:56991972-57143437 (-) |  | 5.5900 | 4.15e-71 | 1.05e-69 |
| ENSMUSG00000015889 | Lta4h | protein\_coding | 10:93453411-93484875 (+) |  | 1.2600 | 5.28e-71 | 1.34e-69 |
| ENSMUSG00000039976 | Tbc1d16 | protein\_coding | 11:119143045-119228499 (-) |  | 2.4500 | 7.39e-71 | 1.87e-69 |
| ENSMUSG00000007815 | Rhoa | protein\_coding | 9:108306129-108337934 (+) |  | -0.6210 | 7.61e-71 | 1.92e-69 |
| ENSMUSG00000042599 | Kdm7a | protein\_coding | 6:39136623-39206789 (-) |  | -1.7800 | 8.23e-71 | 2.07e-69 |
| ENSMUSG00000042444 | Mindy2 | protein\_coding | 9:70599014-70657174 (-) |  | -1.0500 | 9.93e-71 | 2.50e-69 |
| ENSMUSG00000037062 | Sh3glb1 | protein\_coding | 3:144683678-144720335 (-) |  | -1.0300 | 1.23e-70 | 3.09e-69 |
| ENSMUSG00000030047 | Arhgap25 | protein\_coding | 6:87458545-87533259 (-) |  | -1.3000 | 1.54e-70 | 3.87e-69 |
| ENSMUSG00000027475 | Kif3b | protein\_coding | 2:153291413-153333390 (+) |  | -1.1700 | 1.57e-70 | 3.94e-69 |
| ENSMUSG00000043587 | Pxylp1 | protein\_coding | 9:96823336-96892669 (-) |  | 0.9500 | 1.58e-70 | 3.94e-69 |
| ENSMUSG00000022324 | Matn2 | protein\_coding | 15:34306677-34436273 (+) |  | 1.6700 | 1.61e-70 | 4.01e-69 |
| ENSMUSG00000021474 | Sfxn1 | protein\_coding | 13:54071869-54108342 (+) |  | 1.3900 | 1.68e-70 | 4.18e-69 |
| ENSMUSG00000041360 | Pum3 | protein\_coding | 19:27388698-27429825 (-) |  | 0.8700 | 1.78e-70 | 4.42e-69 |
| ENSMUSG00000000194 | Gpr107 | protein\_coding | 2:31152316-31218775 (+) |  | -0.8870 | 2.25e-70 | 5.59e-69 |
| ENSMUSG00000034422 | Parp14 | protein\_coding | 16:35832874-35871544 (-) |  | -1.6700 | 2.46e-70 | 6.10e-69 |
| ENSMUSG00000021326 | Trim27 | protein\_coding | 13:21179445-21194724 (+) |  | 0.9210 | 2.49e-70 | 6.15e-69 |
| ENSMUSG00000032667 | Pon2 | protein\_coding | 6:5264147-5298455 (-) |  | -1.0900 | 2.81e-70 | 6.94e-69 |
| ENSMUSG00000042613 | Pbxip1 | protein\_coding | 3:89436706-89450952 (+) |  | -0.9060 | 2.91e-70 | 7.17e-69 |
| ENSMUSG00000005846 | Rsl1d1 | protein\_coding | 16:11192970-11203331 (-) |  | 1.2400 | 3.19e-70 | 7.87e-69 |
| ENSMUSG00000064373 | Selenop | protein\_coding | 15:3268547-3280508 (+) |  | -1.8700 | 3.28e-70 | 8.07e-69 |
| ENSMUSG00000051790 | Nlgn2 | protein\_coding | 11:69823122-69837784 (-) |  | 2.8200 | 3.58e-70 | 8.80e-69 |
| ENSMUSG00000006519 | Cyba | protein\_coding | 8:122424776-122432930 (-) |  | -1.2600 | 3.72e-70 | 9.11e-69 |
| ENSMUSG00000030096 | Slc6a6 | protein\_coding | 6:91684053-91759066 (+) |  | -0.9170 | 4.44e-70 | 1.09e-68 |
| ENSMUSG00000040463 | Mybbp1a | protein\_coding | 11:72441355-72451768 (+) |  | 1.5000 | 4.47e-70 | 1.09e-68 |
| ENSMUSG00000035969 | Rusc2 | protein\_coding | 4:43381979-43427088 (+) |  | -1.8700 | 5.11e-70 | 1.25e-68 |
| ENSMUSG00000001674 | Ddx18 | protein\_coding | 1:121553835-121567989 (-) |  | 1.1300 | 9.63e-70 | 2.34e-68 |
| ENSMUSG00000045411 | 2410002F23Rik | protein\_coding | 7:44246722-44252319 (+) |  | 1.4900 | 9.93e-70 | 2.41e-68 |
| ENSMUSG00000058715 | Fcer1g | protein\_coding | 1:171229572-171234365 (-) |  | -1.0400 | 1.14e-69 | 2.77e-68 |
| ENSMUSG00000025785 | Exosc7 | protein\_coding | 9:123113215-123136129 (+) |  | 1.0200 | 1.19e-69 | 2.89e-68 |
| ENSMUSG00000040483 | Xaf1 | protein\_coding | 11:72301629-72313733 (+) |  | -2.2200 | 1.47e-69 | 3.57e-68 |
| ENSMUSG00000051579 | Tceal8 | protein\_coding | X:136168984-136172342 (-) |  | 1.3000 | 1.64e-69 | 3.96e-68 |
| ENSMUSG00000005566 | Trim28 | protein\_coding | 7:12999114-13031035 (+) |  | 1.0400 | 1.64e-69 | 3.96e-68 |
| ENSMUSG00000036985 | Zdhhc9 | protein\_coding | X:48171969-48208878 (-) |  | -1.2200 | 1.65e-69 | 3.96e-68 |
| ENSMUSG00000029580 | Actb | protein\_coding | 5:142903115-142906754 (-) |  | -0.9200 | 1.67e-69 | 4.01e-68 |
| ENSMUSG00000015937 | Macroh2a1 | protein\_coding | 13:56073619-56136361 (-) |  | 0.9700 | 2.18e-69 | 5.24e-68 |
| ENSMUSG00000017309 | Cd300lg | protein\_coding | 11:102041509-102055620 (+) |  | -1.4000 | 2.23e-69 | 5.35e-68 |
| ENSMUSG00000029014 | Dnajc2 | protein\_coding | 5:21757267-21785251 (-) |  | 0.9240 | 2.57e-69 | 6.16e-68 |
| ENSMUSG00000074417 | Gm14548 | protein\_coding | 7:3884242-3898120 (-) |  | -1.8400 | 2.58e-69 | 6.16e-68 |
| ENSMUSG00000052609 | Plekhg3 | protein\_coding | 12:76530891-76580488 (+) |  | -0.9870 | 2.58e-69 | 6.16e-68 |
| ENSMUSG00000037697 | Ddhd1 | protein\_coding | 14:45588467-45658143 (-) |  | -1.2800 | 2.93e-69 | 6.97e-68 |
| ENSMUSG00000028849 | Map7d1 | protein\_coding | 4:126232167-126256343 (-) |  | -0.6880 | 3.56e-69 | 8.47e-68 |
| ENSMUSG00000039981 | Zc3h12d | protein\_coding | 10:7832470-7870396 (+) |  | -2.0800 | 5.14e-69 | 1.22e-67 |
| ENSMUSG00000022407 | Adsl | protein\_coding | 15:80948490-80970946 (+) |  | 1.2100 | 9.31e-69 | 2.21e-67 |
| ENSMUSG00000027287 | Snap23 | protein\_coding | 2:120567671-120601255 (+) |  | -1.0800 | 9.82e-69 | 2.32e-67 |
| ENSMUSG00000036989 | Trim3 | protein\_coding | 7:105604463-105633571 (-) |  | -1.0100 | 1.25e-68 | 2.96e-67 |
| ENSMUSG00000024037 | Wdr4 | protein\_coding | 17:31494322-31519980 (-) |  | 1.2800 | 1.40e-68 | 3.30e-67 |
| ENSMUSG00000056737 | Capg | protein\_coding | 6:72544391-72562983 (+) |  | -1.5600 | 1.41e-68 | 3.32e-67 |
| ENSMUSG00000020650 | Bcap29 | protein\_coding | 12:31590967-31634658 (-) |  | 1.3400 | 1.47e-68 | 3.46e-67 |
| ENSMUSG00000026094 | Stk17b | protein\_coding | 1:53755506-53785224 (-) |  | -1.5900 | 1.56e-68 | 3.65e-67 |
| ENSMUSG00000028530 | Jak1 | protein\_coding | 4:101152367-101265282 (-) |  | -0.7110 | 1.61e-68 | 3.76e-67 |
| ENSMUSG00000001986 | Gria3 | protein\_coding | X:41400854-41678601 (+) |  | 1.8600 | 1.90e-68 | 4.44e-67 |
| ENSMUSG00000019872 | Smpdl3a | protein\_coding | 10:57794335-57811830 (+) |  | -1.9500 | 1.98e-68 | 4.64e-67 |
| ENSMUSG00000044026 | Slc35g1 | protein\_coding | 19:38395980-38405610 (+) |  | 1.6400 | 2.17e-68 | 5.05e-67 |
| ENSMUSG00000040616 | Tmem51 | protein\_coding | 4:142030992-142084304 (-) |  | -2.3400 | 2.89e-68 | 6.73e-67 |
| ENSMUSG00000032589 | Bsn | protein\_coding | 9:108096022-108190384 (-) |  | 2.5200 | 3.07e-68 | 7.14e-67 |
| ENSMUSG00000015766 | Eps8 | protein\_coding | 6:137477245-137654876 (-) |  | -1.7900 | 3.68e-68 | 8.54e-67 |
| ENSMUSG00000067150 | Xpo5 | protein\_coding | 17:46202782-46243598 (+) |  | 0.9920 | 4.10e-68 | 9.50e-67 |
| ENSMUSG00000068129 | Cst7 | protein\_coding | 2:150570415-150578944 (+) |  | 3.3200 | 4.33e-68 | 1.00e-66 |
| ENSMUSG00000079481 | Nhsl2 | protein\_coding | X:101849385-102092055 (+) |  | -1.5000 | 4.67e-68 | 1.08e-66 |
| ENSMUSG00000001056 | Nhp2 | protein\_coding | 11:51619735-51623714 (+) |  | 1.2800 | 5.04e-68 | 1.16e-66 |
| ENSMUSG00000030720 | Cln3 | protein\_coding | 7:126571207-126585817 (-) |  | -0.9210 | 5.53e-68 | 1.27e-66 |
| ENSMUSG00000027381 | Bcl2l11 | protein\_coding | 2:128126038-128162547 (+) |  | -1.0800 | 5.77e-68 | 1.33e-66 |
| ENSMUSG00000022822 | Abcc5 | protein\_coding | 16:20331303-20426394 (-) |  | -1.2300 | 6.42e-68 | 1.48e-66 |
| ENSMUSG00000038145 | Snrk | protein\_coding | 9:122117266-122169702 (+) |  | -0.9090 | 6.79e-68 | 1.56e-66 |
| ENSMUSG00000022466 | Rpap3 | protein\_coding | 15:97675097-97705825 (-) |  | 1.2200 | 7.99e-68 | 1.83e-66 |
| ENSMUSG00000031838 | Ifi30 | lncRNA | 8:70762774-70766663 (-) |  | -1.2800 | 1.04e-67 | 2.37e-66 |
| ENSMUSG00000006418 | Rnf114 | protein\_coding | 2:167492645-167516173 (+) |  | -0.9450 | 1.86e-67 | 4.25e-66 |
| ENSMUSG00000040451 | Sgms1 | protein\_coding | 19:32122727-32389714 (-) |  | -1.3100 | 2.14e-67 | 4.88e-66 |
| ENSMUSG00000048234 | Rnf149 | protein\_coding | 1:39551296-39577405 (-) |  | -1.2000 | 2.21e-67 | 5.04e-66 |
| ENSMUSG00000079523 | Tmsb10 | protein\_coding | 6:72957347-72958748 (-) |  | -1.1900 | 2.48e-67 | 5.63e-66 |
| ENSMUSG00000027367 | Stard7 | protein\_coding | 2:127270218-127298932 (+) |  | 0.7740 | 2.48e-67 | 5.63e-66 |
| ENSMUSG00000067212 | H2-T23 | protein\_coding | 17:36029773-36032855 (-) |  | -1.2700 | 2.78e-67 | 6.29e-66 |
| ENSMUSG00000032786 | Alas1 | protein\_coding | 9:106233455-106248654 (-) |  | 1.5700 | 2.85e-67 | 6.46e-66 |
| ENSMUSG00000022704 | Qtrt2 | protein\_coding | 16:43861407-43926809 (-) |  | 1.4000 | 2.93e-67 | 6.62e-66 |
| ENSMUSG00000053040 | Aph1c | protein\_coding | 9:66814994-66834726 (-) |  | -1.5100 | 3.23e-67 | 7.29e-66 |
| ENSMUSG00000000532 | Acvr1b | protein\_coding | 15:101174067-101213684 (+) |  | -1.0500 | 4.67e-67 | 1.05e-65 |
| ENSMUSG00000013629 | Cad | protein\_coding | 5:31054780-31078479 (+) |  | 1.9900 | 5.26e-67 | 1.18e-65 |
| ENSMUSG00000090164 | BC035044 | protein\_coding | 6:128849090-128891126 (-) |  | 1.4100 | 5.74e-67 | 1.29e-65 |
| ENSMUSG00000045679 | Pqlc3 | protein\_coding | 12:16988648-17000408 (-) |  | -1.1000 | 7.16e-67 | 1.60e-65 |
| ENSMUSG00000021242 | Npc2 | protein\_coding | 12:84754562-84773152 (-) |  | -0.9250 | 7.40e-67 | 1.66e-65 |
| ENSMUSG00000040620 | Dhx33 | protein\_coding | 11:70984091-71004437 (-) |  | 1.1000 | 8.43e-67 | 1.88e-65 |
| ENSMUSG00000021224 | Numb | protein\_coding | 12:83794034-83921934 (-) |  | -0.8810 | 8.72e-67 | 1.95e-65 |
| ENSMUSG00000074361 | C5ar2 | protein\_coding | 7:16234585-16244154 (-) |  | -2.7900 | 9.76e-67 | 2.17e-65 |
| ENSMUSG00000024999 | Noc3l | protein\_coding | 19:38788128-38819237 (-) |  | 1.1800 | 1.00e-66 | 2.23e-65 |
| ENSMUSG00000032902 | Slc16a1 | protein\_coding | 3:104638668-104658462 (+) |  | 2.2700 | 1.08e-66 | 2.40e-65 |
| ENSMUSG00000040325 | Dcaf1 | protein\_coding | 9:106821874-106880992 (+) |  | 1.7100 | 1.57e-66 | 3.49e-65 |
| ENSMUSG00000073489 | Ifi204 | protein\_coding | 1:173747293-173766943 (-) |  | -2.7700 | 1.65e-66 | 3.65e-65 |
| ENSMUSG00000061458 | Nol10 | protein\_coding | 12:17348458-17430095 (+) |  | 1.0100 | 1.67e-66 | 3.69e-65 |
| ENSMUSG00000026395 | Ptprc | protein\_coding | 1:138062861-138175708 (-) |  | -1.6600 | 1.90e-66 | 4.19e-65 |
| ENSMUSG00000048440 | Cyp4f16 | protein\_coding | 17:32536558-32551798 (+) |  | -1.0300 | 2.13e-66 | 4.70e-65 |
| ENSMUSG00000033705 | Stard9 | protein\_coding | 2:120629121-120731895 (+) |  | -1.8300 | 2.20e-66 | 4.84e-65 |
| ENSMUSG00000037260 | Hgsnat | protein\_coding | 8:25944453-25976753 (-) |  | -1.3100 | 3.67e-66 | 8.06e-65 |
| ENSMUSG00000033416 | Gucd1 | protein\_coding | 10:75351111-75517972 (-) |  | -0.9830 | 3.93e-66 | 8.63e-65 |
| ENSMUSG00000022508 | Bcl6 | protein\_coding | 16:23965052-23988852 (-) |  | -1.6800 | 3.96e-66 | 8.68e-65 |
| ENSMUSG00000004609 | Cd33 | protein\_coding | 7:43524216-43544428 (-) |  | -2.2500 | 5.20e-66 | 1.14e-64 |
| ENSMUSG00000024527 | Afg3l2 | protein\_coding | 18:67404764-67449172 (-) |  | 0.7650 | 8.60e-66 | 1.88e-64 |
| ENSMUSG00000039985 | Sinhcaf | protein\_coding | 6:148921035-148946467 (-) |  | 1.3500 | 9.77e-66 | 2.13e-64 |
| ENSMUSG00000026930 | Gpsm1 | protein\_coding | 2:26315515-26348237 (+) |  | 2.1600 | 1.53e-65 | 3.33e-64 |
| ENSMUSG00000029528 | Pxn | protein\_coding | 5:115506676-115555987 (+) |  | -0.6930 | 1.65e-65 | 3.60e-64 |
| ENSMUSG00000032231 | Anxa2 | protein\_coding | 9:69453620-69491795 (+) |  | -1.2200 | 1.84e-65 | 4.00e-64 |
| ENSMUSG00000066151 | Fkbp15 | protein\_coding | 4:62300342-62360548 (-) |  | -0.6550 | 2.10e-65 | 4.55e-64 |
| ENSMUSG00000039497 | Dse | protein\_coding | 10:34151393-34207715 (-) |  | -1.8500 | 2.11e-65 | 4.57e-64 |
| ENSMUSG00000019303 | Psmc3ip | protein\_coding | 11:101091823-101095436 (-) |  | 1.4300 | 2.96e-65 | 6.41e-64 |
| ENSMUSG00000062203 | Gspt1 | protein\_coding | 16:11219292-11254325 (-) |  | 0.9400 | 4.00e-65 | 8.64e-64 |
| ENSMUSG00000029175 | Slc35f6 | protein\_coding | 5:30647933-30659729 (+) |  | -1.0500 | 5.87e-65 | 1.27e-63 |
| ENSMUSG00000030357 | Fkbp4 | protein\_coding | 6:128429735-128438677 (-) |  | 1.4400 | 7.00e-65 | 1.51e-63 |
| ENSMUSG00000002984 | Tomm40 | protein\_coding | 7:19701313-19715438 (-) |  | 1.0900 | 7.39e-65 | 1.59e-63 |
| ENSMUSG00000014077 | Chp1 | protein\_coding | 2:119547697-119587027 (+) |  | -0.7160 | 7.79e-65 | 1.67e-63 |
| ENSMUSG00000009687 | Fxyd5 | protein\_coding | 7:31032722-31042481 (-) |  | -1.0500 | 8.26e-65 | 1.77e-63 |
| ENSMUSG00000017697 | Ada | protein\_coding | 2:163726584-163750239 (-) |  | 2.1600 | 9.53e-65 | 2.04e-63 |
| ENSMUSG00000032122 | Slc37a2 | protein\_coding | 9:37227585-37255738 (-) |  | -1.7800 | 1.11e-64 | 2.37e-63 |
| ENSMUSG00000017802 | Retreg3 | protein\_coding | 11:101096322-101119893 (-) |  | -0.6800 | 1.14e-64 | 2.43e-63 |
| ENSMUSG00000032280 | Tle3 | protein\_coding | 9:61372366-61418497 (+) |  | -0.8760 | 1.34e-64 | 2.87e-63 |
| ENSMUSG00000035093 | Secisbp2l | protein\_coding | 2:125736986-125782870 (-) |  | -0.9090 | 1.37e-64 | 2.92e-63 |
| ENSMUSG00000039715 | Wdr34 | protein\_coding | 2:30031546-30048881 (-) |  | 2.1100 | 1.44e-64 | 3.07e-63 |
| ENSMUSG00000030107 | Usp18 | protein\_coding | 6:121245906-121270917 (+) |  | -2.1700 | 1.56e-64 | 3.31e-63 |
| ENSMUSG00000049313 | Sorl1 | protein\_coding | 9:41964720-42124297 (-) |  | -1.0700 | 1.80e-64 | 3.82e-63 |
| ENSMUSG00000008475 | Arpc5 | protein\_coding | 1:152766542-152775597 (+) |  | -0.8450 | 1.85e-64 | 3.92e-63 |
| ENSMUSG00000024143 | Rhoq | protein\_coding | 17:86963082-87000069 (+) |  | -1.4800 | 3.01e-64 | 6.37e-63 |
| ENSMUSG00000038456 | Dennd2a | protein\_coding | 6:39462378-39557867 (-) |  | -2.2600 | 4.13e-64 | 8.73e-63 |
| ENSMUSG00000021149 | Gtpbp4 | protein\_coding | 13:8966331-8996083 (-) |  | 1.0400 | 4.14e-64 | 8.73e-63 |
| ENSMUSG00000026896 | Ifih1 | protein\_coding | 2:62595798-62646255 (-) |  | -1.6600 | 6.36e-64 | 1.34e-62 |
| ENSMUSG00000032185 | Carm1 | protein\_coding | 9:21546894-21592623 (+) |  | 1.0000 | 7.72e-64 | 1.62e-62 |
| ENSMUSG00000029406 | Pitpnm2 | protein\_coding | 5:124118690-124249760 (-) |  | 1.9100 | 7.97e-64 | 1.67e-62 |
| ENSMUSG00000046223 | Plaur | protein\_coding | 7:24462484-24475968 (+) |  | -1.1400 | 8.53e-64 | 1.79e-62 |
| ENSMUSG00000051344 | Plekhm3 | protein\_coding | 1:64785983-64956824 (-) |  | -1.4600 | 9.49e-64 | 1.99e-62 |
| ENSMUSG00000025764 | Jade1 | protein\_coding | 3:41555731-41616864 (+) |  | 0.7380 | 1.04e-63 | 2.18e-62 |
| ENSMUSG00000044092 | C130050O18Rik | protein\_coding | 5:139405280-139415623 (+) |  | -1.5800 | 1.63e-63 | 3.40e-62 |
| ENSMUSG00000041040 | Fam117b | protein\_coding | 1:59913006-59985346 (+) |  | -0.8570 | 1.69e-63 | 3.52e-62 |
| ENSMUSG00000035673 | Sbno2 | protein\_coding | 10:80056992-80105571 (-) |  | -1.0000 | 1.82e-63 | 3.79e-62 |
| ENSMUSG00000052040 | Klf13 | protein\_coding | 7:63886351-63938915 (-) |  | -1.3400 | 1.83e-63 | 3.80e-62 |
| ENSMUSG00000036894 | Rap2b | protein\_coding | 3:61361638-61368430 (+) |  | -0.9640 | 2.29e-63 | 4.77e-62 |
| ENSMUSG00000031432 | Prps1 | protein\_coding | X:140456613-140476140 (+) |  | 1.0700 | 2.54e-63 | 5.27e-62 |
| ENSMUSG00000020272 | Stk10 | protein\_coding | 11:32533305-32624587 (+) |  | -1.2500 | 2.62e-63 | 5.43e-62 |
| ENSMUSG00000057191 | AB124611 | protein\_coding | 9:21526176-21545333 (+) |  | -1.4600 | 2.87e-63 | 5.94e-62 |
| ENSMUSG00000028042 | Zbtb7b | protein\_coding | 3:89377644-89394776 (-) |  | -1.0800 | 3.19e-63 | 6.58e-62 |
| ENSMUSG00000056708 | Ier5 | protein\_coding | 1:155096361-155099636 (-) |  | -2.1800 | 3.54e-63 | 7.30e-62 |
| ENSMUSG00000052593 | Adam17 | protein\_coding | 12:21323509-21373632 (-) |  | -0.7340 | 4.76e-63 | 9.81e-62 |
| ENSMUSG00000035901 | Dennd5a | protein\_coding | 7:109893780-109960470 (-) |  | -0.6580 | 5.11e-63 | 1.05e-61 |
| ENSMUSG00000026796 | Fam129b | protein\_coding | 2:32876114-32925254 (+) |  | -1.0400 | 5.79e-63 | 1.19e-61 |
| ENSMUSG00000022390 | Zc3h7b | protein\_coding | 15:81745057-81796260 (+) |  | 1.1500 | 6.00e-63 | 1.23e-61 |
| ENSMUSG00000036202 | Rif1 | protein\_coding | 2:52072832-52122383 (+) |  | 0.8260 | 6.22e-63 | 1.27e-61 |
| ENSMUSG00000117869 | Snhg4 | lncRNA | 18:35553371-35558316 (+) |  | 2.3400 | 7.02e-63 | 1.44e-61 |
| ENSMUSG00000022892 | App | protein\_coding | 16:84949685-85173766 (-) |  | -0.9220 | 7.20e-63 | 1.47e-61 |
| ENSMUSG00000022108 | Itm2b | protein\_coding | 14:73362226-73385289 (-) |  | -0.8470 | 8.03e-63 | 1.64e-61 |
| ENSMUSG00000015342 | Xk | protein\_coding | X:9272756-9313250 (+) |  | 2.3800 | 9.12e-63 | 1.86e-61 |
| ENSMUSG00000018008 | Cyth4 | protein\_coding | 15:78597047-78622019 (+) |  | -0.8450 | 9.66e-63 | 1.97e-61 |
| ENSMUSG00000032113 | Chek1 | protein\_coding | 9:36708482-36727065 (-) |  | 1.3300 | 1.04e-62 | 2.12e-61 |
| ENSMUSG00000070031 | Sp140 | protein\_coding | 1:85600378-85645037 (+) |  | -0.9890 | 1.14e-62 | 2.30e-61 |
| ENSMUSG00000017837 | Nkiras2 | protein\_coding | 11:100619244-100627607 (+) |  | -0.9440 | 1.17e-62 | 2.37e-61 |
| ENSMUSG00000033159 | Cnppd1 | protein\_coding | 1:75134554-75142711 (-) |  | -0.7330 | 1.77e-62 | 3.58e-61 |
| ENSMUSG00000025403 | Shmt2 | protein\_coding | 10:127517123-127522444 (-) |  | 1.7300 | 2.17e-62 | 4.38e-61 |
| ENSMUSG00000006304 | Arpc2 | protein\_coding | 1:74236084-74268209 (+) |  | -0.5860 | 2.24e-62 | 4.52e-61 |
| ENSMUSG00000040213 | Kyat3 | protein\_coding | 3:142701051-142746870 (+) |  | 1.7300 | 2.29e-62 | 4.63e-61 |
| ENSMUSG00000021281 | Tnfaip2 | protein\_coding | 12:111442469-111455018 (+) |  | -1.0600 | 2.69e-62 | 5.42e-61 |
| ENSMUSG00000025314 | Ptprj | protein\_coding | 2:90429754-90580647 (-) |  | -1.5400 | 2.88e-62 | 5.79e-61 |
| ENSMUSG00000032624 | Eml4 | protein\_coding | 17:83350931-83480361 (+) |  | 0.7410 | 2.93e-62 | 5.88e-61 |
| ENSMUSG00000006732 | Mettl1 | protein\_coding | 10:127041414-127046365 (+) |  | 1.9500 | 3.12e-62 | 6.26e-61 |
| ENSMUSG00000039934 | Gsap | protein\_coding | 5:21186255-21315132 (+) |  | -1.8100 | 3.61e-62 | 7.23e-61 |
| ENSMUSG00000096210 | H1f0 | protein\_coding | 15:79028450-79030504 (+) |  | -1.1500 | 3.64e-62 | 7.29e-61 |
| ENSMUSG00000109324 | Prmt1 | protein\_coding | 7:44975989-44986568 (-) |  | 1.0500 | 4.43e-62 | 8.84e-61 |
| ENSMUSG00000026202 | Tuba4a | protein\_coding | 1:75214228-75219865 (-) |  | 0.9550 | 4.45e-62 | 8.87e-61 |
| ENSMUSG00000022913 | Psmg1 | protein\_coding | 16:95979933-95990960 (-) |  | 1.2600 | 4.49e-62 | 8.95e-61 |
| ENSMUSG00000023032 | Slc4a8 | protein\_coding | 15:100761747-100823968 (+) |  | 2.4800 | 5.11e-62 | 1.02e-60 |
| ENSMUSG00000003848 | Nob1 | protein\_coding | 8:107412486-107425051 (-) |  | 1.1800 | 5.42e-62 | 1.08e-60 |
| ENSMUSG00000079104 | Prps1l3 | protein\_coding | 12:57230412-57239882 (+) |  | 0.8440 | 6.01e-62 | 1.19e-60 |
| ENSMUSG00000039361 | Picalm | protein\_coding | 7:90130213-90213465 (+) |  | -1.3100 | 6.36e-62 | 1.26e-60 |
| ENSMUSG00000040907 | Atp1a3 | protein\_coding | 7:24978167-25005958 (-) |  | -1.2500 | 7.99e-62 | 1.58e-60 |
| ENSMUSG00000020077 | Srgn | protein\_coding | 10:62493833-62527451 (-) |  | 1.7200 | 9.52e-62 | 1.88e-60 |
| ENSMUSG00000021892 | Sh3bp5 | protein\_coding | 14:31359880-31436078 (-) |  | -1.4100 | 1.17e-61 | 2.30e-60 |
| ENSMUSG00000035513 | Ntng2 | protein\_coding | 2:29194541-29253005 (-) |  | -1.6400 | 1.19e-61 | 2.34e-60 |
| ENSMUSG00000028851 | Nudc | protein\_coding | 4:133532542-133545996 (-) |  | 0.8930 | 1.23e-61 | 2.43e-60 |
| ENSMUSG00000032000 | Birc3 | protein\_coding | 9:7848699-7873186 (-) |  | -1.2200 | 1.28e-61 | 2.52e-60 |
| ENSMUSG00000052087 | Rgs14 | protein\_coding | 13:55369732-55384687 (+) |  | -1.1900 | 1.46e-61 | 2.86e-60 |
| ENSMUSG00000027368 | Dusp2 | protein\_coding | 2:127336159-127338376 (+) |  | 3.0800 | 1.50e-61 | 2.94e-60 |
| ENSMUSG00000000346 | Dazap2 | protein\_coding | 15:100615349-100620761 (+) |  | -0.5570 | 1.54e-61 | 3.01e-60 |
| ENSMUSG00000002308 | Cd320 | protein\_coding | 17:33843091-33849774 (+) |  | 2.4200 | 1.54e-61 | 3.02e-60 |
| ENSMUSG00000025492 | Ifitm3 | protein\_coding | 7:141009586-141010770 (-) |  | -2.0200 | 1.68e-61 | 3.28e-60 |
| ENSMUSG00000005237 | Dnah2 | protein\_coding | 11:69420809-69549110 (-) |  | -2.3100 | 1.90e-61 | 3.70e-60 |
| ENSMUSG00000024736 | Tmem132a | protein\_coding | 19:10857822-10869940 (-) |  | -2.0400 | 1.92e-61 | 3.74e-60 |
| ENSMUSG00000000804 | Usp32 | protein\_coding | 11:84984442-85140161 (-) |  | -1.0000 | 2.09e-61 | 4.07e-60 |
| ENSMUSG00000034168 | Irf2bpl | protein\_coding | 12:86880701-86884798 (-) |  | -1.7200 | 3.14e-61 | 6.10e-60 |
| ENSMUSG00000025037 | Maoa | protein\_coding | X:16619698-16687818 (+) |  | 1.9600 | 3.15e-61 | 6.11e-60 |
| ENSMUSG00000004473 | Clec11a | protein\_coding | 7:44302687-44306902 (-) |  | 2.6000 | 3.20e-61 | 6.21e-60 |
| ENSMUSG00000028057 | Rit1 | protein\_coding | 3:88716838-88731049 (+) |  | -1.0600 | 3.32e-61 | 6.42e-60 |
| ENSMUSG00000034330 | Plcg2 | protein\_coding | 8:117498291-117635142 (+) |  | -0.8380 | 3.34e-61 | 6.45e-60 |
| ENSMUSG00000013974 | Mcemp1 | protein\_coding | 8:3665754-3669259 (+) |  | -1.8000 | 3.43e-61 | 6.62e-60 |
| ENSMUSG00000038387 | Rras | protein\_coding | 7:45017961-45021647 (+) |  | -2.2000 | 3.75e-61 | 7.23e-60 |
| ENSMUSG00000018983 | E2f2 | protein\_coding | 4:136172394-136196057 (+) |  | -1.1500 | 3.84e-61 | 7.39e-60 |
| ENSMUSG00000037638 | Zbtb42 | protein\_coding | 12:112678828-112682747 (+) |  | -1.6500 | 3.91e-61 | 7.52e-60 |
| ENSMUSG00000059456 | Ptk2b | protein\_coding | 14:66153257-66281052 (-) |  | -0.8580 | 3.96e-61 | 7.61e-60 |
| ENSMUSG00000035004 | Igsf6 | protein\_coding | 7:121064067-121074572 (-) |  | -0.7940 | 4.16e-61 | 7.97e-60 |
| ENSMUSG00000034377 | Tulp4 | protein\_coding | 17:6106437-6251128 (+) |  | -1.8800 | 4.56e-61 | 8.72e-60 |
| ENSMUSG00000022031 | Elp3 | protein\_coding | 14:65530449-65593075 (-) |  | 1.0500 | 4.59e-61 | 8.79e-60 |
| ENSMUSG00000017843 | Ppp2r5c | protein\_coding | 12:110447120-110583062 (+) |  | -0.5740 | 4.78e-61 | 9.13e-60 |
| ENSMUSG00000020899 | Pfas | protein\_coding | 11:68985697-69008460 (-) |  | 1.4800 | 5.06e-61 | 9.65e-60 |
| ENSMUSG00000031309 | Rps6ka3 | protein\_coding | X:159210307-159368244 (+) |  | -0.7590 | 5.31e-61 | 1.01e-59 |
| ENSMUSG00000030789 | Itgax | protein\_coding | 7:128129547-128150657 (+) |  | 2.6500 | 5.41e-61 | 1.03e-59 |
| ENSMUSG00000040345 | Arhgap9 | protein\_coding | 10:127321964-127329943 (+) |  | -0.9660 | 5.48e-61 | 1.04e-59 |
| ENSMUSG00000009035 | Tmem184b | protein\_coding | 15:79360684-79403569 (-) |  | -1.0200 | 5.94e-61 | 1.13e-59 |
| ENSMUSG00000050138 | Kcnk12 | protein\_coding | 17:87745801-87797994 (-) |  | 2.3200 | 6.12e-61 | 1.16e-59 |
| ENSMUSG00000022946 | Dop1b | protein\_coding | 16:93711904-93810590 (+) |  | -1.3800 | 6.16e-61 | 1.17e-59 |
| ENSMUSG00000059895 | Ptp4a3 | protein\_coding | 15:73723145-73758766 (+) |  | 1.2600 | 7.50e-61 | 1.42e-59 |
| ENSMUSG00000026814 | Eng | protein\_coding | 2:32646595-32682669 (+) |  | 1.4900 | 9.15e-61 | 1.73e-59 |
| ENSMUSG00000027189 | Trim44 | protein\_coding | 2:102300119-102407828 (-) |  | 0.8080 | 1.06e-60 | 2.01e-59 |
| ENSMUSG00000026317 | Cln8 | protein\_coding | 8:14881335-14901720 (+) |  | -1.2300 | 1.23e-60 | 2.31e-59 |
| ENSMUSG00000017144 | Rnd3 | protein\_coding | 2:51130438-51149111 (-) |  | -1.4900 | 1.28e-60 | 2.41e-59 |
| ENSMUSG00000072620 | Slfn2 | protein\_coding | 11:83065112-83070678 (+) |  | -1.6200 | 1.31e-60 | 2.46e-59 |
| ENSMUSG00000097195 | Snhg5 | lncRNA | 9:88495268-88523562 (-) |  | 1.5100 | 1.61e-60 | 3.01e-59 |
| ENSMUSG00000029507 | Pus1 | protein\_coding | 5:110773667-110780659 (-) |  | 0.9940 | 1.63e-60 | 3.06e-59 |
| ENSMUSG00000004393 | Ddx56 | protein\_coding | 11:6258919-6267772 (-) |  | 1.2000 | 1.88e-60 | 3.52e-59 |
| ENSMUSG00000038807 | Rap1gap2 | protein\_coding | 11:74383356-74610915 (-) |  | -1.7900 | 2.19e-60 | 4.09e-59 |
| ENSMUSG00000057596 | Trim30d | protein\_coding | 7:104470014-104507849 (-) |  | -1.3300 | 2.48e-60 | 4.63e-59 |
| ENSMUSG00000000253 | Gmpr | protein\_coding | 13:45507444-45553800 (+) |  | 3.1700 | 3.19e-60 | 5.94e-59 |
| ENSMUSG00000020901 | Pik3r5 | protein\_coding | 11:68432121-68497849 (+) |  | -1.2600 | 3.34e-60 | 6.23e-59 |
| ENSMUSG00000037674 | Rfx7 | protein\_coding | 9:72532240-72622937 (+) |  | 1.0400 | 3.51e-60 | 6.54e-59 |
| ENSMUSG00000074182 | Znhit6 | protein\_coding | 3:145576205-145604795 (+) |  | 1.4500 | 3.83e-60 | 7.11e-59 |
| ENSMUSG00000035248 | Tut7 | protein\_coding | 13:59771561-59823147 (-) |  | -0.6420 | 4.11e-60 | 7.63e-59 |
| ENSMUSG00000045980 | Tmem104 | protein\_coding | 11:115187487-115247023 (+) |  | -1.2100 | 4.13e-60 | 7.65e-59 |
| ENSMUSG00000057147 | Dph6 | protein\_coding | 2:114516416-114654964 (-) |  | 1.2100 | 4.27e-60 | 7.90e-59 |
| ENSMUSG00000023809 | Rps6ka2 | protein\_coding | 17:7170115-7303315 (+) |  | 2.1000 | 4.36e-60 | 8.06e-59 |
| ENSMUSG00000033792 | Atp7a | protein\_coding | X:106027276-106124926 (+) |  | -1.2000 | 4.52e-60 | 8.34e-59 |
| ENSMUSG00000032712 | Resf1 | protein\_coding | 6:149309414-149335663 (+) |  | -0.9770 | 4.69e-60 | 8.66e-59 |
| ENSMUSG00000000078 | Klf6 | protein\_coding | 13:5861482-5870394 (+) |  | -1.3100 | 5.02e-60 | 9.24e-59 |
| ENSMUSG00000027067 | Ssrp1 | protein\_coding | 2:85037234-85047109 (+) |  | 0.7190 | 5.21e-60 | 9.58e-59 |
| ENSMUSG00000019087 | Atp6ap1 | protein\_coding | X:74297097-74304721 (+) |  | -0.7050 | 5.43e-60 | 9.99e-59 |
| ENSMUSG00000043832 | Clec4a3 | protein\_coding | 6:122952515-122969875 (+) |  | -3.5100 | 5.70e-60 | 1.05e-58 |
| ENSMUSG00000041685 | Fcho2 | protein\_coding | 13:98723403-98815449 (-) |  | -1.3200 | 6.18e-60 | 1.13e-58 |
| ENSMUSG00000022861 | Dgkg | protein\_coding | 16:22468461-22657221 (-) |  | -1.2700 | 6.66e-60 | 1.22e-58 |
| ENSMUSG00000036053 | Fmnl2 | protein\_coding | 2:52857860-53133804 (+) |  | 3.7600 | 6.87e-60 | 1.26e-58 |
| ENSMUSG00000064065 | Ipcef1 | protein\_coding | 10:6885796-7052478 (-) |  | -2.0500 | 6.94e-60 | 1.27e-58 |
| ENSMUSG00000024308 | Tapbp | protein\_coding | 17:33915899-33929292 (+) |  | -0.6700 | 7.88e-60 | 1.44e-58 |
| ENSMUSG00000031066 | Usp11 | protein\_coding | X:20703906-20720539 (+) |  | 2.0400 | 8.30e-60 | 1.51e-58 |
| ENSMUSG00000025283 | Sat1 | protein\_coding | X:155213132-155216449 (-) |  | -1.4700 | 9.02e-60 | 1.64e-58 |
| ENSMUSG00000026739 | Bmi1 | protein\_coding | 2:18677018-18686629 (+) |  | 0.8910 | 9.19e-60 | 1.67e-58 |
| ENSMUSG00000040296 | Ddx58 | protein\_coding | 4:40203773-40239828 (-) |  | -3.6800 | 1.06e-59 | 1.92e-58 |
| ENSMUSG00000020423 | Btg2 | protein\_coding | 1:134075170-134079120 (-) |  | -2.1000 | 1.07e-59 | 1.95e-58 |
| ENSMUSG00000035478 | Mbd3 | protein\_coding | 10:80392539-80399550 (-) |  | 0.8280 | 1.54e-59 | 2.78e-58 |
| ENSMUSG00000032477 | Cdc25a | protein\_coding | 9:109875579-109893895 (+) |  | 1.2800 | 1.56e-59 | 2.82e-58 |
| ENSMUSG00000060733 | Ipmk | protein\_coding | 10:71347763-71433327 (+) |  | -0.7180 | 1.60e-59 | 2.88e-58 |
| ENSMUSG00000022325 | Pop1 | protein\_coding | 15:34495304-34530648 (+) |  | 1.4200 | 1.66e-59 | 3.00e-58 |
| ENSMUSG00000061689 | Dlgap4 | protein\_coding | 2:156613705-156764363 (+) |  | -1.3400 | 1.82e-59 | 3.28e-58 |
| ENSMUSG00000004266 | Ptpn6 | protein\_coding | 6:124720707-124738714 (-) |  | -0.7240 | 1.88e-59 | 3.39e-58 |
| ENSMUSG00000073910 | Mob3b | protein\_coding | 4:34949074-35157484 (-) |  | -1.3000 | 1.96e-59 | 3.53e-58 |
| ENSMUSG00000005142 | Man2b1 | protein\_coding | 8:85083270-85098282 (+) |  | -0.9140 | 2.15e-59 | 3.86e-58 |
| ENSMUSG00000112023 | Lilr4b | protein\_coding | 10:51480632-51486703 (+) |  | -2.9400 | 2.68e-59 | 4.80e-58 |
| ENSMUSG00000024383 | Map3k2 | protein\_coding | 18:32163089-32236751 (+) |  | -0.7370 | 3.10e-59 | 5.56e-58 |
| ENSMUSG00000020821 | Kif1c | protein\_coding | 11:70700548-70731964 (+) |  | -0.9100 | 3.33e-59 | 5.96e-58 |
| ENSMUSG00000034120 | Srsf2 | protein\_coding | 11:116849901-116853094 (-) |  | 0.6250 | 4.08e-59 | 7.30e-58 |
| ENSMUSG00000024525 | Impa2 | protein\_coding | 18:67289186-67322472 (+) |  | 0.7690 | 4.72e-59 | 8.42e-58 |
| ENSMUSG00000029192 | Tbc1d14 | protein\_coding | 5:36490604-36593276 (-) |  | -0.9130 | 4.76e-59 | 8.48e-58 |
| ENSMUSG00000002870 | Mcm2 | protein\_coding | 6:88883474-88898780 (-) |  | 1.2600 | 5.03e-59 | 8.96e-58 |
| ENSMUSG00000026737 | Pip4k2a | protein\_coding | 2:18842255-18998126 (-) |  | -0.9940 | 5.30e-59 | 9.43e-58 |
| ENSMUSG00000059108 | Ifitm6 | protein\_coding | 7:141015699-141017924 (-) |  | -2.6000 | 6.17e-59 | 1.10e-57 |
| ENSMUSG00000026466 | Tor1aip1 | protein\_coding | 1:156004599-156036480 (-) |  | -0.6770 | 7.97e-59 | 1.41e-57 |
| ENSMUSG00000039308 | Ndst2 | protein\_coding | 14:20723730-20734562 (-) |  | -0.9720 | 9.10e-59 | 1.61e-57 |
| ENSMUSG00000038893 | Fam117a | protein\_coding | 11:95337018-95381872 (+) |  | 0.9000 | 9.61e-59 | 1.70e-57 |
| ENSMUSG00000054843 | Atrnl1 | protein\_coding | 19:57611034-58133343 (+) |  | -1.0000 | 1.14e-58 | 2.01e-57 |
| ENSMUSG00000041774 | Ydjc | protein\_coding | 16:17144621-17160701 (+) |  | 1.9200 | 1.20e-58 | 2.12e-57 |
| ENSMUSG00000039512 | Uhrf1bp1 | protein\_coding | 17:27856441-27900041 (+) |  | 0.9260 | 1.66e-58 | 2.94e-57 |
| ENSMUSG00000034723 | Tmx4 | protein\_coding | 2:134594185-134644145 (-) |  | 0.9120 | 1.69e-58 | 2.97e-57 |
| ENSMUSG00000022139 | Mbnl2 | protein\_coding | 14:120275669-120431697 (+) |  | -0.7800 | 1.87e-58 | 3.28e-57 |
| ENSMUSG00000039936 | Pik3cd | protein\_coding | 4:149649168-149702571 (-) |  | -0.6230 | 2.16e-58 | 3.80e-57 |
| ENSMUSG00000046865 | Fbl | protein\_coding | 7:28169710-28179269 (+) |  | 1.1300 | 2.20e-58 | 3.86e-57 |
| ENSMUSG00000047146 | Tet1 | protein\_coding | 10:62804570-62908996 (-) |  | 3.2700 | 2.56e-58 | 4.50e-57 |
| ENSMUSG00000031948 | Kars | protein\_coding | 8:111993443-112011323 (-) |  | 0.7620 | 2.73e-58 | 4.78e-57 |
| ENSMUSG00000031812 | Map1lc3b | protein\_coding | 8:121590361-121598760 (+) |  | -0.8850 | 2.79e-58 | 4.87e-57 |
| ENSMUSG00000033128 | Gga1 | protein\_coding | 15:78877190-78894585 (+) |  | -0.6290 | 3.22e-58 | 5.63e-57 |
| ENSMUSG00000010609 | Psen2 | protein\_coding | 1:180227004-180263438 (-) |  | -0.9210 | 3.26e-58 | 5.69e-57 |
| ENSMUSG00000054404 | Slfn5 | protein\_coding | 11:82951349-82964840 (+) |  | -3.5100 | 3.40e-58 | 5.92e-57 |
| ENSMUSG00000027009 | Itga4 | protein\_coding | 2:79255426-79333123 (+) |  | -1.1800 | 3.85e-58 | 6.70e-57 |
| ENSMUSG00000003500 | Impdh1 | protein\_coding | 6:29200434-29216364 (-) |  | 1.5100 | 3.92e-58 | 6.82e-57 |
| ENSMUSG00000018476 | Kdm6b | protein\_coding | 11:69398508-69413675 (-) |  | -1.1600 | 4.50e-58 | 7.82e-57 |
| ENSMUSG00000029781 | Fkbp9 | protein\_coding | 6:56832059-56879358 (+) |  | 2.1300 | 5.47e-58 | 9.49e-57 |
| ENSMUSG00000030657 | Xylt1 | protein\_coding | 7:117380979-117673580 (+) |  | -1.0800 | 5.53e-58 | 9.59e-57 |
| ENSMUSG00000052369 | Tmem106c | protein\_coding | 15:97964200-97970275 (+) |  | 1.3300 | 5.71e-58 | 9.89e-57 |
| ENSMUSG00000029267 | Mtf2 | protein\_coding | 5:108065674-108109004 (+) |  | 0.7490 | 5.90e-58 | 1.02e-56 |
| ENSMUSG00000046245 | Pilra | protein\_coding | 5:137821952-137836281 (-) |  | -2.2000 | 5.97e-58 | 1.03e-56 |
| ENSMUSG00000057219 | Armc7 | protein\_coding | 11:115475667-115490467 (+) |  | -1.1600 | 6.03e-58 | 1.04e-56 |
| ENSMUSG00000020125 | Elane | protein\_coding | 10:79886247-79888215 (+) |  | 3.0900 | 6.24e-58 | 1.08e-56 |
| ENSMUSG00000031826 | Usp10 | protein\_coding | 8:119910360-119957560 (+) |  | 1.0300 | 6.47e-58 | 1.11e-56 |
| ENSMUSG00000020471 | Pold2 | protein\_coding | 11:5872180-5878292 (-) |  | 1.2000 | 6.83e-58 | 1.17e-56 |
| ENSMUSG00000023156 | Rpp14 | protein\_coding | 14:8080367-8091834 (+) |  | 1.1600 | 6.92e-58 | 1.19e-56 |
| ENSMUSG00000022111 | Uchl3 | protein\_coding | 14:101653967-101696125 (+) |  | 1.1900 | 7.41e-58 | 1.27e-56 |
| ENSMUSG00000026193 | Fn1 | protein\_coding | 1:71585520-71653200 (-) |  | -2.6600 | 7.68e-58 | 1.32e-56 |
| ENSMUSG00000062867 | Impdh2 | protein\_coding | 9:108560286-108565584 (+) |  | 1.2900 | 7.77e-58 | 1.33e-56 |
| ENSMUSG00000053398 | Phgdh | protein\_coding | 3:98313170-98339990 (-) |  | 2.7800 | 8.60e-58 | 1.47e-56 |
| ENSMUSG00000026496 | Parp1 | protein\_coding | 1:180568924-180601254 (+) |  | 0.8110 | 9.22e-58 | 1.57e-56 |
| ENSMUSG00000005087 | Cd44 | protein\_coding | 2:102811141-102901665 (-) |  | -0.7200 | 9.66e-58 | 1.65e-56 |
| ENSMUSG00000027514 | Zbp1 | protein\_coding | 2:173206612-173218923 (-) |  | -2.8600 | 1.10e-57 | 1.88e-56 |
| ENSMUSG00000023473 | Celsr3 | protein\_coding | 9:108826320-108852969 (+) |  | -1.2100 | 1.10e-57 | 1.88e-56 |
| ENSMUSG00000008604 | Ubqln4 | protein\_coding | 3:88553758-88569725 (+) |  | 0.9680 | 1.22e-57 | 2.08e-56 |
| ENSMUSG00000052889 | Prkcb | protein\_coding | 7:122288751-122634402 (+) |  | -1.9400 | 1.32e-57 | 2.24e-56 |
| ENSMUSG00000028466 | Creb3 | protein\_coding | 4:43562332-43567060 (+) |  | -0.8740 | 1.37e-57 | 2.32e-56 |
| ENSMUSG00000029447 | Cct6a | protein\_coding | 5:129786998-129846371 (+) |  | 1.1300 | 1.44e-57 | 2.43e-56 |
| ENSMUSG00000048027 | Rgmb | protein\_coding | 17:15803188-15831039 (-) |  | -2.2000 | 1.44e-57 | 2.44e-56 |
| ENSMUSG00000006517 | Mvd | protein\_coding | 8:122433601-122443422 (-) |  | -1.0900 | 1.49e-57 | 2.52e-56 |
| ENSMUSG00000030101 | Sumf1 | protein\_coding | 6:108107028-108185582 (-) |  | -0.8020 | 1.68e-57 | 2.84e-56 |
| ENSMUSG00000027430 | Dtd1 | protein\_coding | 2:144599897-144768758 (+) |  | 1.6600 | 1.74e-57 | 2.94e-56 |
| ENSMUSG00000016477 | E2f3 | protein\_coding | 13:29906575-29986063 (-) |  | 1.1000 | 1.75e-57 | 2.95e-56 |
| ENSMUSG00000032221 | Mns1 | protein\_coding | 9:72438011-72462025 (+) |  | 1.0700 | 1.79e-57 | 3.02e-56 |
| ENSMUSG00000026833 | Olfm1 | protein\_coding | 2:28192992-28230736 (+) |  | -1.4800 | 2.09e-57 | 3.51e-56 |
| ENSMUSG00000060703 | Cd302 | protein\_coding | 2:60251993-60284488 (-) |  | -1.8800 | 2.16e-57 | 3.62e-56 |
| ENSMUSG00000030942 | Thumpd1 | protein\_coding | 7:119715093-119720798 (-) |  | 0.8800 | 2.51e-57 | 4.20e-56 |
| ENSMUSG00000013160 | Atp6v0d1 | protein\_coding | 8:105524465-105566047 (-) |  | -0.7490 | 2.63e-57 | 4.40e-56 |
| ENSMUSG00000038214 | Bend3 | protein\_coding | 10:43478831-43515396 (+) |  | 1.9400 | 2.83e-57 | 4.74e-56 |
| ENSMUSG00000061286 | Exosc5 | protein\_coding | 7:25659164-25671368 (+) |  | 0.9110 | 3.28e-57 | 5.48e-56 |
| ENSMUSG00000004264 | Phb2 | protein\_coding | 6:124712336-124716950 (+) |  | 0.8620 | 3.47e-57 | 5.80e-56 |
| ENSMUSG00000022792 | Yars2 | protein\_coding | 16:16302965-16309640 (+) |  | 1.3100 | 3.54e-57 | 5.90e-56 |
| ENSMUSG00000026355 | Mcm6 | protein\_coding | 1:128331590-128359664 (-) |  | 1.3000 | 3.54e-57 | 5.90e-56 |
| ENSMUSG00000078783 | Gm9733 | protein\_coding | 3:15296551-15332302 (-) |  | -3.0900 | 3.85e-57 | 6.40e-56 |
| ENSMUSG00000017057 | Il13ra1 | protein\_coding | X:36112110-36171259 (+) |  | -2.8600 | 3.96e-57 | 6.58e-56 |
| ENSMUSG00000027878 | Notch2 | protein\_coding | 3:98013527-98150361 (+) |  | -1.1300 | 4.25e-57 | 7.05e-56 |
| ENSMUSG00000020941 | Map3k14 | protein\_coding | 11:103219762-103267472 (-) |  | -1.1800 | 4.25e-57 | 7.05e-56 |
| ENSMUSG00000079442 | St6galnac4 | protein\_coding | 2:32587095-32599698 (+) |  | 1.4300 | 4.30e-57 | 7.12e-56 |
| ENSMUSG00000026786 | Apbb1ip | protein\_coding | 2:22774094-22875653 (+) |  | -0.7730 | 4.71e-57 | 7.79e-56 |
| ENSMUSG00000000384 | Tbrg4 | protein\_coding | 11:6615598-6626067 (-) |  | 1.0900 | 5.17e-57 | 8.54e-56 |
| ENSMUSG00000018593 | Sparc | protein\_coding | 11:55394500-55423183 (-) |  | -2.5700 | 5.58e-57 | 9.21e-56 |
| ENSMUSG00000047881 | Rell1 | protein\_coding | 5:63908897-63968897 (-) |  | -0.9380 | 6.61e-57 | 1.09e-55 |
| ENSMUSG00000003868 | Ruvbl2 | protein\_coding | 7:45421760-45438096 (-) |  | 1.1000 | 6.91e-57 | 1.14e-55 |
| ENSMUSG00000020641 | Rsad2 | protein\_coding | 12:26442746-26456452 (-) |  | -2.8700 | 7.45e-57 | 1.23e-55 |
| ENSMUSG00000024962 | Vegfb | protein\_coding | 19:6982473-6987651 (-) |  | 1.6300 | 9.16e-57 | 1.51e-55 |
| ENSMUSG00000026047 | Poglut2 | protein\_coding | 1:44106546-44118808 (-) |  | 1.2700 | 1.18e-56 | 1.95e-55 |
| ENSMUSG00000069833 | Ahnak | protein\_coding | 19:8989284-9076914 (+) |  | -1.6100 | 1.35e-56 | 2.21e-55 |
| ENSMUSG00000053897 | Slc39a8 | protein\_coding | 3:135825279-135888572 (+) |  | 1.9600 | 1.49e-56 | 2.44e-55 |
| ENSMUSG00000039356 | Exosc2 | protein\_coding | 2:31670715-31681349 (+) |  | 1.1300 | 1.65e-56 | 2.70e-55 |
| ENSMUSG00000026469 | Xpr1 | protein\_coding | 1:155275701-155417415 (-) |  | -0.9690 | 1.65e-56 | 2.71e-55 |
| ENSMUSG00000011831 | Evi5 | protein\_coding | 5:107744795-107875107 (-) |  | -1.0200 | 2.40e-56 | 3.92e-55 |
| ENSMUSG00000117183 | Gm20008 | processed\_pseudogene | 17:22713782-22716937 (-) |  | 2.1900 | 2.55e-56 | 4.15e-55 |
| ENSMUSG00000069255 | Dusp22 | protein\_coding | 13:30659999-30711231 (+) |  | -1.5300 | 2.57e-56 | 4.19e-55 |
| ENSMUSG00000003559 | As3mt | protein\_coding | 19:46707458-46741099 (+) |  | 1.2100 | 2.65e-56 | 4.32e-55 |
| ENSMUSG00000020009 | Ifngr1 | protein\_coding | 10:19591949-19610229 (+) |  | -0.7510 | 2.95e-56 | 4.79e-55 |
| ENSMUSG00000021279 | Cdc42bpb | protein\_coding | 12:111292976-111377718 (-) |  | -1.4500 | 2.97e-56 | 4.82e-55 |
| ENSMUSG00000038393 | Txnip | protein\_coding | 3:96557957-96561883 (+) |  | -1.7100 | 2.99e-56 | 4.86e-55 |
| ENSMUSG00000024696 | Lpxn | protein\_coding | 19:12796193-12833807 (+) |  | -1.0900 | 3.23e-56 | 5.24e-55 |
| ENSMUSG00000029247 | Paics | protein\_coding | 5:76951307-76967509 (+) |  | 0.8670 | 3.58e-56 | 5.79e-55 |
| ENSMUSG00000025044 | Msr1 | protein\_coding | 8:39581685-39642673 (-) |  | -3.1600 | 4.22e-56 | 6.83e-55 |
| ENSMUSG00000038963 | Slco4a1 | protein\_coding | 2:180456245-180474867 (+) |  | 2.7500 | 4.61e-56 | 7.45e-55 |
| ENSMUSG00000075705 | Msrb1 | protein\_coding | 17:24736642-24742778 (+) |  | -1.1600 | 4.92e-56 | 7.94e-55 |
| ENSMUSG00000003808 | Farsa | protein\_coding | 8:84856989-84869257 (+) |  | 0.7810 | 5.35e-56 | 8.63e-55 |
| ENSMUSG00000013236 | Ptprs | protein\_coding | 17:56412426-56476483 (-) |  | 1.3500 | 5.76e-56 | 9.28e-55 |
| ENSMUSG00000026281 | Dtymk | protein\_coding | 1:93792576-93801934 (-) |  | 1.1100 | 6.32e-56 | 1.02e-54 |
| ENSMUSG00000035834 | Polr3g | protein\_coding | 13:81673843-81711013 (-) |  | 1.9200 | 6.49e-56 | 1.04e-54 |
| ENSMUSG00000031537 | Ikbkb | protein\_coding | 8:22659212-22706589 (-) |  | -0.9400 | 6.63e-56 | 1.06e-54 |
| ENSMUSG00000032594 | Ip6k1 | protein\_coding | 9:108002501-108048782 (+) |  | -0.7600 | 8.92e-56 | 1.43e-54 |
| ENSMUSG00000038028 | Tigar | protein\_coding | 6:127085116-127109557 (-) |  | 1.1900 | 9.18e-56 | 1.47e-54 |
| ENSMUSG00000074677 | Sirpb1c | protein\_coding | 3:15795145-15848528 (-) |  | -4.0200 | 1.05e-55 | 1.68e-54 |
| ENSMUSG00000085156 | Snhg15 | lncRNA | 11:6525591-6528779 (-) |  | 1.8900 | 1.17e-55 | 1.87e-54 |
| ENSMUSG00000016664 | Pacsin2 | protein\_coding | 15:83375607-83464606 (-) |  | -0.6360 | 1.20e-55 | 1.92e-54 |
| ENSMUSG00000055817 | Mta3 | protein\_coding | 17:83706163-83821516 (+) |  | -0.8920 | 1.41e-55 | 2.26e-54 |
| ENSMUSG00000097715 | Gpr137b-ps | transcribed\_unprocessed\_pseudogene | 13:12615057-12650388 (-) |  | -1.8400 | 1.46e-55 | 2.32e-54 |
| ENSMUSG00000021987 | Mtmr6 | protein\_coding | 14:60265228-60302370 (+) |  | -0.8340 | 1.58e-55 | 2.52e-54 |
| ENSMUSG00000032324 | Tspan3 | protein\_coding | 9:56131725-56165477 (-) |  | 0.9820 | 1.59e-55 | 2.52e-54 |
| ENSMUSG00000030559 | Rab38 | protein\_coding | 7:88430273-88491572 (+) |  | 2.8300 | 1.79e-55 | 2.84e-54 |
| ENSMUSG00000049866 | Arl4c | protein\_coding | 1:88673125-88702221 (-) |  | -1.6000 | 1.80e-55 | 2.86e-54 |
| ENSMUSG00000049103 | Ccr2 | protein\_coding | 9:124101950-124113557 (+) |  | -1.5700 | 1.99e-55 | 3.16e-54 |
| ENSMUSG00000075254 | Heg1 | protein\_coding | 16:33684370-33771576 (+) |  | 1.7300 | 2.13e-55 | 3.37e-54 |
| ENSMUSG00000074622 | Mafb | protein\_coding | 2:160363703-160367065 (-) |  | -2.9600 | 2.15e-55 | 3.40e-54 |
| ENSMUSG00000041488 | Stx3 | protein\_coding | 19:11775118-11819403 (-) |  | -1.2100 | 2.20e-55 | 3.48e-54 |
| ENSMUSG00000027330 | Cdc25b | protein\_coding | 2:131186949-131198497 (+) |  | -1.1800 | 2.25e-55 | 3.54e-54 |
| ENSMUSG00000029924 | Slc37a3 | protein\_coding | 6:39334773-39377675 (-) |  | -2.3100 | 2.46e-55 | 3.88e-54 |
| ENSMUSG00000101389 | Ms4a4a | protein\_coding | 19:11375523-11392790 (+) |  | -3.7700 | 2.57e-55 | 4.05e-54 |
| ENSMUSG00000053101 | Gpr141 | protein\_coding | 13:19749682-19824257 (-) |  | -1.4100 | 2.77e-55 | 4.35e-54 |
| ENSMUSG00000024856 | Cdk2ap2 | protein\_coding | 19:4097182-4099019 (+) |  | -1.1500 | 3.03e-55 | 4.76e-54 |
| ENSMUSG00000030662 | Ipo5 | protein\_coding | 14:120911224-120947999 (+) |  | 1.0700 | 3.38e-55 | 5.30e-54 |
| ENSMUSG00000029063 | Nadk | protein\_coding | 4:155562378-155591001 (+) |  | -0.7980 | 4.25e-55 | 6.66e-54 |
| ENSMUSG00000022120 | Rnf219 | protein\_coding | 14:104477536-104522645 (-) |  | 1.0200 | 4.30e-55 | 6.74e-54 |
| ENSMUSG00000020812 | Snhg16 | lncRNA | 11:116671604-116685072 (+) |  | 1.5300 | 4.66e-55 | 7.29e-54 |
| ENSMUSG00000026853 | Crat | protein\_coding | 2:30400471-30415813 (-) |  | -0.9270 | 5.42e-55 | 8.47e-54 |
| ENSMUSG00000019970 | Sgk1 | protein\_coding | 10:21882184-21999903 (+) |  | -1.7900 | 5.55e-55 | 8.67e-54 |
| ENSMUSG00000005514 | Por | protein\_coding | 5:135670033-135735326 (+) |  | -0.8740 | 5.68e-55 | 8.86e-54 |
| ENSMUSG00000025153 | Fasn | protein\_coding | 11:120805846-120824547 (-) |  | 1.6500 | 6.02e-55 | 9.39e-54 |
| ENSMUSG00000020437 | Myo1g | protein\_coding | 11:6506548-6520965 (-) |  | -0.7950 | 7.48e-55 | 1.16e-53 |
| ENSMUSG00000028758 | Kif17 | protein\_coding | 4:138250435-138301967 (+) |  | 4.3200 | 7.52e-55 | 1.17e-53 |
| ENSMUSG00000027804 | Ppid | protein\_coding | 3:79591342-79603650 (+) |  | 1.0600 | 7.97e-55 | 1.24e-53 |
| ENSMUSG00000027074 | Slc43a3 | protein\_coding | 2:84936579-84958509 (+) |  | 1.0200 | 8.07e-55 | 1.25e-53 |
| ENSMUSG00000057421 | Las1l | protein\_coding | X:95935335-95956962 (-) |  | 0.9840 | 8.95e-55 | 1.39e-53 |
| ENSMUSG00000019873 | Reep3 | protein\_coding | 10:67009189-67096945 (-) |  | -1.0800 | 1.02e-54 | 1.57e-53 |
| ENSMUSG00000000811 | Txnrd3 | protein\_coding | 6:89643988-89675529 (+) |  | 2.5600 | 1.02e-54 | 1.58e-53 |
| ENSMUSG00000028693 | Nasp | protein\_coding | 4:116601052-116627941 (-) |  | 0.8510 | 1.28e-54 | 1.98e-53 |
| ENSMUSG00000043017 | Ptgir | protein\_coding | 7:16906490-16910905 (+) |  | -1.9900 | 1.31e-54 | 2.02e-53 |
| ENSMUSG00000006678 | Pola1 | protein\_coding | X:93304767-93632155 (-) |  | 0.8780 | 1.41e-54 | 2.18e-53 |
| ENSMUSG00000005413 | Hmox1 | protein\_coding | 8:75093621-75100589 (+) |  | -2.7200 | 1.60e-54 | 2.47e-53 |
| ENSMUSG00000038481 | Cdk19 | protein\_coding | 10:40339564-40483818 (+) |  | -0.6080 | 1.84e-54 | 2.83e-53 |
| ENSMUSG00000056749 | Nfil3 | protein\_coding | 13:52967209-52981073 (-) |  | -1.1900 | 1.98e-54 | 3.04e-53 |
| ENSMUSG00000074578 | Zfas1 | lncRNA | 2:167062934-167065862 (+) |  | 1.0700 | 2.26e-54 | 3.47e-53 |
| ENSMUSG00000030220 | Arhgdib | protein\_coding | 6:136923655-136941899 (-) |  | -0.5440 | 3.09e-54 | 4.74e-53 |
| ENSMUSG00000004317 | Clcn5 | protein\_coding | X:7153810-7319358 (-) |  | -0.9910 | 3.51e-54 | 5.39e-53 |
| ENSMUSG00000049037 | Clec4a1 | protein\_coding | 6:122921848-122934619 (+) |  | -3.1400 | 4.77e-54 | 7.31e-53 |
| ENSMUSG00000046722 | Cdc42se1 | protein\_coding | 3:95228732-95236409 (+) |  | -0.6600 | 4.90e-54 | 7.49e-53 |
| ENSMUSG00000031101 | Sash3 | protein\_coding | X:48146436-48161565 (+) |  | -0.6620 | 5.50e-54 | 8.40e-53 |
| ENSMUSG00000060950 | Trmt61a | protein\_coding | 12:111678105-111683902 (+) |  | 1.6600 | 5.80e-54 | 8.85e-53 |
| ENSMUSG00000034203 | Chchd4 | protein\_coding | 6:91462172-91473546 (-) |  | 1.4400 | 9.14e-54 | 1.39e-52 |
| ENSMUSG00000020064 | Herc4 | protein\_coding | 10:63243810-63317878 (+) |  | -1.0300 | 9.23e-54 | 1.41e-52 |
| ENSMUSG00000022575 | Gsdmd | protein\_coding | 15:75862327-75867408 (+) |  | -0.8190 | 9.24e-54 | 1.41e-52 |
| ENSMUSG00000073490 | Ifi207 | protein\_coding | 1:173723427-173741747 (-) |  | -2.6600 | 1.14e-53 | 1.73e-52 |
| ENSMUSG00000074272 | Ceacam1 | protein\_coding | 7:25461707-25477603 (-) |  | -1.3800 | 1.22e-53 | 1.85e-52 |
| ENSMUSG00000030536 | Iqgap1 | protein\_coding | 7:80711583-80825974 (-) |  | -0.8550 | 1.75e-53 | 2.65e-52 |
| ENSMUSG00000029470 | P2rx4 | protein\_coding | 5:122707544-122729738 (+) |  | -0.8820 | 1.83e-53 | 2.77e-52 |
| ENSMUSG00000003228 | Grk5 | protein\_coding | 19:60889749-61095968 (+) |  | -2.4500 | 2.02e-53 | 3.06e-52 |
| ENSMUSG00000008318 | Relt | protein\_coding | 7:100845847-100863446 (-) |  | -1.0100 | 2.27e-53 | 3.44e-52 |
| ENSMUSG00000037493 | Cib2 | protein\_coding | 9:54544794-54560218 (-) |  | -1.9400 | 2.32e-53 | 3.51e-52 |
| ENSMUSG00000030847 | Bag3 | protein\_coding | 7:128523616-128546981 (+) |  | -1.0600 | 2.33e-53 | 3.51e-52 |
| ENSMUSG00000026170 | Cyp27a1 | protein\_coding | 1:74713574-74737892 (+) |  | -1.9800 | 2.35e-53 | 3.55e-52 |
| ENSMUSG00000027353 | Mcm8 | protein\_coding | 2:132816141-132844197 (+) |  | 1.2600 | 2.37e-53 | 3.57e-52 |
| ENSMUSG00000062397 | Zfp706 | protein\_coding | 15:36997027-37007773 (-) |  | 0.6300 | 2.59e-53 | 3.90e-52 |
| ENSMUSG00000038736 | Nudcd1 | protein\_coding | 15:44373163-44428307 (-) |  | 1.2000 | 2.65e-53 | 3.99e-52 |
| ENSMUSG00000027366 | Sppl2a | protein\_coding | 2:126890391-126933235 (-) |  | -0.9040 | 2.73e-53 | 4.11e-52 |
| ENSMUSG00000004069 | Dnaja3 | protein\_coding | 16:4639989-4707695 (+) |  | 0.7600 | 2.75e-53 | 4.12e-52 |
| ENSMUSG00000027199 | Gatm | protein\_coding | 2:122594467-122611303 (-) |  | 1.7600 | 3.26e-53 | 4.88e-52 |
| ENSMUSG00000027490 | E2f1 | protein\_coding | 2:154559407-154569892 (-) |  | 0.9040 | 3.54e-53 | 5.30e-52 |
| ENSMUSG00000026648 | Dclre1c | protein\_coding | 2:3424131-3464130 (+) |  | -1.5200 | 4.25e-53 | 6.35e-52 |
| ENSMUSG00000026192 | Atic | protein\_coding | 1:71557150-71579631 (+) |  | 1.1600 | 4.29e-53 | 6.41e-52 |
| ENSMUSG00000056666 | Retsat | protein\_coding | 6:72598475-72608425 (+) |  | 1.2400 | 4.41e-53 | 6.59e-52 |
| ENSMUSG00000039208 | Metrnl | protein\_coding | 11:121701544-121716306 (+) |  | -2.9700 | 4.56e-53 | 6.80e-52 |
| ENSMUSG00000000915 | Hip1r | protein\_coding | 5:123973628-124005558 (+) |  | 1.9600 | 4.62e-53 | 6.89e-52 |
| ENSMUSG00000029208 | Guf1 | protein\_coding | 5:69556923-69575973 (+) |  | 0.8930 | 5.01e-53 | 7.46e-52 |
| ENSMUSG00000021338 | Carmil1 | protein\_coding | 13:24012344-24280795 (-) |  | 2.8400 | 5.49e-53 | 8.17e-52 |
| ENSMUSG00000032350 | Gclc | protein\_coding | 9:77754535-77794485 (+) |  | 0.7750 | 5.67e-53 | 8.43e-52 |
| ENSMUSG00000021418 | Rpp40 | protein\_coding | 13:35893472-35906359 (-) |  | 2.3500 | 6.11e-53 | 9.07e-52 |
| ENSMUSG00000035954 | Dock4 | protein\_coding | 12:40445952-40846874 (+) |  | -1.6700 | 7.18e-53 | 1.06e-51 |
| ENSMUSG00000045410 | Akr1e1 | protein\_coding | 13:4590750-4609174 (-) |  | 1.6100 | 8.02e-53 | 1.19e-51 |
| ENSMUSG00000035354 | Uvrag | protein\_coding | 7:98885021-99141141 (-) |  | -0.6760 | 8.08e-53 | 1.20e-51 |
| ENSMUSG00000024681 | Ms4a3 | protein\_coding | 19:11629496-11640851 (-) |  | 3.9900 | 8.81e-53 | 1.30e-51 |
| ENSMUSG00000002307 | Daxx | protein\_coding | 17:33909414-33915590 (+) |  | -0.7330 | 8.95e-53 | 1.32e-51 |
| ENSMUSG00000028191 | Bcl10 | protein\_coding | 3:145922804-145934356 (+) |  | -0.8210 | 9.03e-53 | 1.33e-51 |
| ENSMUSG00000005732 | Ranbp1 | protein\_coding | 16:18239784-18248732 (-) |  | 1.1300 | 9.43e-53 | 1.39e-51 |
| ENSMUSG00000021868 | Ppif | protein\_coding | 14:25694154-25700468 (+) |  | 1.2400 | 1.00e-52 | 1.48e-51 |
| ENSMUSG00000038388 | Mpp6 | protein\_coding | 6:50110241-50198939 (+) |  | 0.6620 | 1.05e-52 | 1.55e-51 |
| ENSMUSG00000025732 | Mcrip2 | protein\_coding | 17:25863698-25868764 (-) |  | 2.1300 | 1.10e-52 | 1.62e-51 |
| ENSMUSG00000022906 | Parp9 | protein\_coding | 16:35938470-35972605 (+) |  | -1.0900 | 1.19e-52 | 1.75e-51 |
| ENSMUSG00000038527 | C1rl | protein\_coding | 6:124493113-124510643 (+) |  | -1.4200 | 1.21e-52 | 1.77e-51 |
| ENSMUSG00000040521 | Tsfm | protein\_coding | 10:127011572-127030840 (-) |  | 0.9260 | 1.27e-52 | 1.87e-51 |
| ENSMUSG00000061979 | Rcc1l | protein\_coding | 5:134148054-134176774 (-) |  | 1.3300 | 1.37e-52 | 2.00e-51 |
| ENSMUSG00000026970 | Rbms1 | protein\_coding | 2:60750193-60963192 (-) |  | -0.8460 | 1.41e-52 | 2.06e-51 |
| ENSMUSG00000038936 | Sccpdh | protein\_coding | 1:179668210-179687189 (+) |  | 2.2000 | 1.88e-52 | 2.75e-51 |
| ENSMUSG00000006179 | Prss16 | protein\_coding | 13:22002173-22009742 (-) |  | 1.9300 | 1.90e-52 | 2.77e-51 |
| ENSMUSG00000021079 | Timm9 | protein\_coding | 12:71123173-71136684 (-) |  | 1.4700 | 2.39e-52 | 3.48e-51 |
| ENSMUSG00000000149 | Gna12 | protein\_coding | 5:140758408-140830431 (-) |  | 0.6840 | 2.39e-52 | 3.48e-51 |
| ENSMUSG00000053158 | Fes | protein\_coding | 7:80377756-80387946 (-) |  | -0.6260 | 2.53e-52 | 3.68e-51 |
| ENSMUSG00000020175 | Rab36 | protein\_coding | 10:75037058-75054748 (+) |  | -1.8600 | 2.91e-52 | 4.23e-51 |
| ENSMUSG00000059326 | Csf2ra | protein\_coding | 19:61223957-61228429 (-) |  | -0.9760 | 3.16e-52 | 4.59e-51 |
| ENSMUSG00000071041 | Impdh2-ps | processed\_pseudogene | 8:100030558-100032102 (+) |  | 1.2200 | 3.45e-52 | 5.01e-51 |
| ENSMUSG00000109511 | Nup62 | protein\_coding | 7:44816088-44830812 (+) |  | 0.8130 | 3.61e-52 | 5.24e-51 |
| ENSMUSG00000025278 | Flnb | protein\_coding | 14:7817957-7951588 (+) |  | 1.6100 | 3.90e-52 | 5.65e-51 |
| ENSMUSG00000019088 | Dnase1l1 | protein\_coding | X:74273217-74282337 (-) |  | -1.2200 | 4.05e-52 | 5.86e-51 |
| ENSMUSG00000036452 | Arhgap26 | protein\_coding | 18:38993145-39376284 (+) |  | -1.0800 | 4.26e-52 | 6.15e-51 |
| ENSMUSG00000026116 | Tmem131 | protein\_coding | 1:36792191-36943666 (-) |  | -0.9090 | 4.39e-52 | 6.34e-51 |
| ENSMUSG00000031216 | Stard8 | protein\_coding | X:99003248-99074728 (+) |  | -1.2300 | 4.91e-52 | 7.09e-51 |
| ENSMUSG00000026433 | Rab29 | protein\_coding | 1:131867224-131872887 (+) |  | -1.1200 | 5.32e-52 | 7.67e-51 |
| ENSMUSG00000029925 | Tbxas1 | protein\_coding | 6:38875404-39084585 (+) |  | -1.5300 | 5.38e-52 | 7.75e-51 |
| ENSMUSG00000043939 | A530064D06Rik | protein\_coding | 17:48149126-48167275 (-) |  | -1.9800 | 5.59e-52 | 8.05e-51 |
| ENSMUSG00000073409 | H2-Q6 | protein\_coding | 17:35424850-35430055 (+) |  | 1.8200 | 6.23e-52 | 8.95e-51 |
| ENSMUSG00000018927 | Ccl6 | protein\_coding | 11:83587882-83593087 (-) |  | -3.2900 | 6.24e-52 | 8.96e-51 |
| ENSMUSG00000038299 | Wdr36 | protein\_coding | 18:32837225-32867594 (+) |  | 0.9890 | 6.30e-52 | 9.04e-51 |
| ENSMUSG00000063275 | Hacd1 | protein\_coding | 2:13850282-14056135 (-) |  | 1.6300 | 6.51e-52 | 9.33e-51 |
| ENSMUSG00000023827 | Agpat4 | protein\_coding | 17:12118704-12219645 (+) |  | -2.3900 | 6.66e-52 | 9.53e-51 |
| ENSMUSG00000004952 | Rasa4 | protein\_coding | 5:136083916-136111860 (+) |  | 0.8630 | 6.80e-52 | 9.72e-51 |
| ENSMUSG00000045045 | Lrfn4 | protein\_coding | 19:4611785-4615667 (-) |  | 2.6700 | 7.26e-52 | 1.04e-50 |
| ENSMUSG00000021018 | Polr2h | protein\_coding | 16:20717665-20722267 (+) |  | 1.1100 | 8.06e-52 | 1.15e-50 |
| ENSMUSG00000028136 | Snx27 | protein\_coding | 3:94497544-94582716 (-) |  | -0.6760 | 8.89e-52 | 1.27e-50 |
| ENSMUSG00000030447 | Cyfip1 | protein\_coding | 7:55841745-55932602 (+) |  | -0.7350 | 9.11e-52 | 1.30e-50 |
| ENSMUSG00000043336 | Filip1l | protein\_coding | 16:57353093-57573126 (+) |  | -1.7000 | 9.18e-52 | 1.31e-50 |
| ENSMUSG00000049502 | Dtx3l | protein\_coding | 16:35926511-35939151 (-) |  | -0.9150 | 1.01e-51 | 1.44e-50 |
| ENSMUSG00000039158 | Akna | protein\_coding | 4:63367125-63403354 (-) |  | -0.8200 | 1.09e-51 | 1.55e-50 |
| ENSMUSG00000028923 | Necap2 | protein\_coding | 4:141066512-141078357 (-) |  | -0.7810 | 1.09e-51 | 1.55e-50 |
| ENSMUSG00000025266 | Gnl3l | protein\_coding | X:150983141-151017322 (-) |  | 0.6550 | 1.09e-51 | 1.55e-50 |
| ENSMUSG00000001098 | Kctd10 | protein\_coding | 5:114363567-114380508 (-) |  | -0.7770 | 1.16e-51 | 1.65e-50 |
| ENSMUSG00000040990 | Sh3kbp1 | protein\_coding | X:159627272-159978069 (+) |  | -0.6550 | 1.18e-51 | 1.67e-50 |
| ENSMUSG00000020227 | Irak3 | protein\_coding | 10:120141648-120202130 (-) |  | -0.9480 | 1.19e-51 | 1.68e-50 |
| ENSMUSG00000020475 | Pgam2 | protein\_coding | 11:5801640-5803733 (-) |  | -1.4100 | 1.31e-51 | 1.85e-50 |
| ENSMUSG00000025354 | Dnajc14 | protein\_coding | 10:128804062-128819446 (+) |  | -0.5640 | 1.33e-51 | 1.88e-50 |
| ENSMUSG00000021477 | Ctsl | protein\_coding | 13:64359337-64370890 (-) |  | -1.2700 | 1.54e-51 | 2.17e-50 |
| ENSMUSG00000028033 | Kcnq5 | protein\_coding | 1:21398403-21961942 (-) |  | 4.3000 | 1.71e-51 | 2.40e-50 |
| ENSMUSG00000038976 | Ppp1r9b | protein\_coding | 11:94991035-95006899 (+) |  | -0.7400 | 1.91e-51 | 2.68e-50 |
| ENSMUSG00000025163 | Cd7 | protein\_coding | 11:121036747-121039418 (-) |  | 3.1500 | 2.09e-51 | 2.94e-50 |
| ENSMUSG00000003123 | Lipe | protein\_coding | 7:25379527-25398710 (-) |  | -0.7830 | 2.46e-51 | 3.46e-50 |
| ENSMUSG00000037731 | Themis2 | protein\_coding | 4:132781843-132796387 (-) |  | -0.8180 | 2.55e-51 | 3.57e-50 |
| ENSMUSG00000030341 | Tnfrsf1a | protein\_coding | 6:125349362-125362484 (+) |  | -0.5460 | 3.22e-51 | 4.51e-50 |
| ENSMUSG00000029082 | Bst1 | protein\_coding | 5:43818885-43843986 (+) |  | -3.1200 | 3.27e-51 | 4.58e-50 |
| ENSMUSG00000021116 | Eif2s1 | protein\_coding | 12:78861819-78887010 (+) |  | 0.8690 | 3.52e-51 | 4.93e-50 |
| ENSMUSG00000050708 | Ftl1 | protein\_coding | 7:45457944-45459884 (-) |  | -0.6960 | 3.59e-51 | 5.02e-50 |
| ENSMUSG00000019990 | Pde7b | protein\_coding | 10:20398004-20725078 (-) |  | -1.9800 | 4.22e-51 | 5.89e-50 |
| ENSMUSG00000018209 | Stk4 | protein\_coding | 2:164070322-164155524 (+) |  | -0.4480 | 4.34e-51 | 6.05e-50 |
| ENSMUSG00000010307 | Tmem86a | protein\_coding | 7:47050601-47054777 (+) |  | -1.6100 | 4.34e-51 | 6.05e-50 |
| ENSMUSG00000053835 | H2-T24 | protein\_coding | 17:36005695-36020560 (-) |  | -2.1000 | 4.58e-51 | 6.38e-50 |
| ENSMUSG00000010025 | Aldh3a2 | protein\_coding | 11:61223417-61267464 (-) |  | -0.7570 | 4.90e-51 | 6.83e-50 |
| ENSMUSG00000069892 | 9930111J21Rik2 | protein\_coding | 11:49015874-49051242 (-) |  | -1.2500 | 4.93e-51 | 6.85e-50 |
| ENSMUSG00000038679 | Trps1 | protein\_coding | 15:50654752-50890463 (-) |  | -1.2600 | 5.06e-51 | 7.03e-50 |
| ENSMUSG00000050244 | Heatr1 | protein\_coding | 13:12395027-12440289 (+) |  | 0.9410 | 5.08e-51 | 7.04e-50 |
| ENSMUSG00000114608 | Gm36161 | lncRNA | 13:120010390-120018931 (+) |  | -1.7200 | 5.08e-51 | 7.04e-50 |
| ENSMUSG00000021707 | Dhfr | protein\_coding | 13:92354726-92389053 (+) |  | 1.2500 | 5.21e-51 | 7.22e-50 |
| ENSMUSG00000042684 | Npl | protein\_coding | 1:153503015-153550045 (-) |  | 1.7300 | 5.47e-51 | 7.58e-50 |
| ENSMUSG00000036693 | Nop14 | protein\_coding | 5:34638536-34660148 (-) |  | 1.1600 | 6.33e-51 | 8.75e-50 |
| ENSMUSG00000030835 | Nomo1 | protein\_coding | 7:46033698-46084212 (+) |  | 1.2200 | 6.61e-51 | 9.13e-50 |
| ENSMUSG00000026430 | Rassf5 | protein\_coding | 1:131176410-131245258 (-) |  | -0.7820 | 6.97e-51 | 9.62e-50 |
| ENSMUSG00000019528 | Gyg | protein\_coding | 3:20122084-20155317 (-) |  | -0.9530 | 7.73e-51 | 1.07e-49 |
| ENSMUSG00000056076 | Eif3b | protein\_coding | 5:140419328-140443360 (+) |  | 0.9520 | 8.28e-51 | 1.14e-49 |
| ENSMUSG00000048578 | Mlec | protein\_coding | 5:115142981-115158179 (-) |  | 0.7550 | 8.49e-51 | 1.17e-49 |
| ENSMUSG00000035847 | Ids | protein\_coding | X:70343069-70365084 (-) |  | -1.1100 | 9.33e-51 | 1.28e-49 |
| ENSMUSG00000004054 | Map3k11 | protein\_coding | 19:5688742-5702865 (+) |  | -0.6300 | 9.78e-51 | 1.34e-49 |
| ENSMUSG00000022965 | Ifngr2 | protein\_coding | 16:91547072-91565623 (+) |  | -0.9860 | 9.79e-51 | 1.34e-49 |
| ENSMUSG00000003099 | Ppp5c | protein\_coding | 7:17004640-17027924 (-) |  | 0.7600 | 1.06e-50 | 1.45e-49 |
| ENSMUSG00000026491 | Ahctf1 | protein\_coding | 1:179744894-179803680 (-) |  | 0.7760 | 1.09e-50 | 1.50e-49 |
| ENSMUSG00000026600 | Soat1 | protein\_coding | 1:156424525-156474331 (-) |  | -0.7200 | 1.16e-50 | 1.59e-49 |
| ENSMUSG00000027293 | Ehd4 | protein\_coding | 2:120089175-120154606 (-) |  | -0.7300 | 1.30e-50 | 1.78e-49 |
| ENSMUSG00000051343 | Rab11fip5 | protein\_coding | 6:85334962-85374634 (-) |  | -1.1300 | 1.34e-50 | 1.83e-49 |
| ENSMUSG00000004865 | Srpk1 | protein\_coding | 17:28587648-28622709 (-) |  | 0.8910 | 1.40e-50 | 1.91e-49 |
| ENSMUSG00000032397 | Tipin | protein\_coding | 9:64281581-64305424 (+) |  | 1.0200 | 1.53e-50 | 2.09e-49 |
| ENSMUSG00000031328 | Flna | protein\_coding | X:74223461-74249820 (-) |  | -0.8270 | 1.58e-50 | 2.15e-49 |
| ENSMUSG00000032216 | Nedd4 | protein\_coding | 9:72662346-72749852 (+) |  | 5.2000 | 1.64e-50 | 2.24e-49 |
| ENSMUSG00000019066 | Rab3d | protein\_coding | 9:21907491-21918192 (-) |  | -0.7860 | 1.69e-50 | 2.30e-49 |
| ENSMUSG00000054676 | 1600014C10Rik | protein\_coding | 7:38183217-38197568 (+) |  | -0.8590 | 1.89e-50 | 2.57e-49 |
| ENSMUSG00000022488 | Nckap1l | protein\_coding | 15:103453794-103498810 (+) |  | -0.6030 | 1.90e-50 | 2.58e-49 |
| ENSMUSG00000041147 | Brca2 | protein\_coding | 5:150522630-150570329 (+) |  | 1.3700 | 2.22e-50 | 3.01e-49 |
| ENSMUSG00000035722 | Abca7 | protein\_coding | 10:79996494-80015572 (+) |  | -0.8170 | 2.34e-50 | 3.17e-49 |
| ENSMUSG00000048007 | Timm8a1 | protein\_coding | X:134537256-134541865 (-) |  | 1.1600 | 2.38e-50 | 3.23e-49 |
| ENSMUSG00000039748 | Exo1 | protein\_coding | 1:175880581-175913489 (+) |  | 1.4100 | 2.60e-50 | 3.52e-49 |
| ENSMUSG00000037706 | Cd81 | protein\_coding | 7:143052739-143067934 (+) |  | 2.6700 | 2.65e-50 | 3.59e-49 |
| ENSMUSG00000028869 | Gnl2 | protein\_coding | 4:125016585-125055380 (+) |  | 0.6760 | 3.29e-50 | 4.44e-49 |
| ENSMUSG00000052056 | Zfp217 | protein\_coding | 2:170108643-170148103 (-) |  | -0.7020 | 3.32e-50 | 4.48e-49 |
| ENSMUSG00000028587 | Orc1 | protein\_coding | 4:108579423-108614833 (+) |  | 1.4300 | 3.58e-50 | 4.82e-49 |
| ENSMUSG00000042106 | Inka1 | protein\_coding | 9:107984223-107985879 (-) |  | 2.7800 | 3.75e-50 | 5.05e-49 |
| ENSMUSG00000003348 | Mob3a | protein\_coding | 10:80685253-80701977 (-) |  | -0.6750 | 3.79e-50 | 5.09e-49 |
| ENSMUSG00000040212 | Emp3 | protein\_coding | 7:45918023-45921404 (-) |  | -0.7130 | 3.88e-50 | 5.22e-49 |
| ENSMUSG00000028480 | Glipr2 | protein\_coding | 4:43957401-43979118 (+) |  | -1.3900 | 4.18e-50 | 5.62e-49 |
| ENSMUSG00000029009 | Mthfr | protein\_coding | 4:148039077-148059551 (+) |  | -1.1800 | 4.21e-50 | 5.65e-49 |
| ENSMUSG00000015745 | Plekho1 | protein\_coding | 3:95988429-95996001 (-) |  | -1.0400 | 4.41e-50 | 5.92e-49 |
| ENSMUSG00000058672 | Tubb2a | protein\_coding | 13:34074274-34078007 (-) |  | -1.0300 | 4.59e-50 | 6.14e-49 |
| ENSMUSG00000030691 | Fchsd2 | protein\_coding | 7:101092863-101284405 (+) |  | 0.9180 | 4.72e-50 | 6.32e-49 |
| ENSMUSG00000027889 | Ampd2 | protein\_coding | 3:108074062-108086651 (-) |  | 0.8110 | 5.38e-50 | 7.20e-49 |
| ENSMUSG00000034875 | Nudt19 | protein\_coding | 7:35547185-35556304 (-) |  | 1.1500 | 5.68e-50 | 7.59e-49 |
| ENSMUSG00000038975 | Rabggtb | protein\_coding | 3:153907287-153913009 (-) |  | 0.9410 | 5.72e-50 | 7.64e-49 |
| ENSMUSG00000009291 | Pttg1ip | protein\_coding | 10:77581720-77598732 (+) |  | -0.6420 | 5.83e-50 | 7.78e-49 |
| ENSMUSG00000002204 | Napsa | protein\_coding | 7:44572380-44586862 (+) |  | -0.6330 | 6.28e-50 | 8.37e-49 |
| ENSMUSG00000001247 | Lsr | protein\_coding | 7:30957770-30973464 (-) |  | -2.1000 | 6.31e-50 | 8.41e-49 |
| ENSMUSG00000048249 | Crebrf | protein\_coding | 17:26715650-26776635 (+) |  | -1.4600 | 6.33e-50 | 8.41e-49 |
| ENSMUSG00000024515 | Smad4 | protein\_coding | 18:73639009-73703780 (-) |  | -0.6220 | 6.39e-50 | 8.50e-49 |
| ENSMUSG00000006641 | Slc5a6 | protein\_coding | 5:31036036-31048924 (-) |  | 1.4600 | 6.53e-50 | 8.67e-49 |
| ENSMUSG00000022967 | Ifnar1 | protein\_coding | 16:91485238-91507441 (+) |  | -0.6490 | 6.73e-50 | 8.93e-49 |
| ENSMUSG00000022721 | Trmt2a | protein\_coding | 16:18248679-18254772 (+) |  | 0.7440 | 6.83e-50 | 9.05e-49 |
| ENSMUSG00000036879 | Phkb | protein\_coding | 8:85840959-86061376 (+) |  | 0.7220 | 7.53e-50 | 9.97e-49 |
| ENSMUSG00000049999 | Ppp1r3d | protein\_coding | 2:178411206-178414472 (-) |  | -1.6100 | 7.72e-50 | 1.02e-48 |
| ENSMUSG00000027339 | Rassf2 | protein\_coding | 2:131989415-132030258 (-) |  | -0.6140 | 8.55e-50 | 1.13e-48 |
| ENSMUSG00000030127 | Cops7a | protein\_coding | 6:124958413-124965538 (-) |  | 0.7520 | 8.73e-50 | 1.15e-48 |
| ENSMUSG00000035754 | Wdr18 | protein\_coding | 10:79960152-79970203 (+) |  | 1.2100 | 1.09e-49 | 1.44e-48 |
| ENSMUSG00000040033 | Stat2 | protein\_coding | 10:128270559-128292849 (+) |  | -1.4800 | 1.28e-49 | 1.68e-48 |
| ENSMUSG00000019777 | Hdac2 | protein\_coding | 10:36974544-37001889 (+) |  | 0.7220 | 1.47e-49 | 1.94e-48 |
| ENSMUSG00000048897 | Zfp710 | protein\_coding | 7:80024814-80094173 (+) |  | -0.8440 | 1.53e-49 | 2.02e-48 |
| ENSMUSG00000026177 | Slc11a1 | protein\_coding | 1:74375195-74386062 (+) |  | -3.0300 | 1.56e-49 | 2.05e-48 |
| ENSMUSG00000017670 | Elmo2 | protein\_coding | 2:165288031-165326479 (-) |  | -0.6930 | 1.65e-49 | 2.17e-48 |
| ENSMUSG00000067336 | Bmpr2 | protein\_coding | 1:59763400-59879014 (+) |  | -0.9800 | 1.79e-49 | 2.35e-48 |
| ENSMUSG00000024312 | Wdr46 | protein\_coding | 17:33940660-33949697 (+) |  | 1.0500 | 2.18e-49 | 2.86e-48 |
| ENSMUSG00000033720 | Sfxn5 | protein\_coding | 6:85213049-85333422 (-) |  | -1.6600 | 2.32e-49 | 3.04e-48 |
| ENSMUSG00000029730 | Mcm7 | protein\_coding | 5:138164583-138172422 (-) |  | 1.1100 | 2.65e-49 | 3.47e-48 |
| ENSMUSG00000041444 | Arhgap32 | protein\_coding | 9:32116136-32268446 (+) |  | 1.7300 | 2.83e-49 | 3.71e-48 |
| ENSMUSG00000028159 | Dapp1 | protein\_coding | 3:137931007-137981545 (-) |  | -0.7610 | 3.32e-49 | 4.34e-48 |
| ENSMUSG00000024781 | Lipa | protein\_coding | 19:34492318-34527474 (-) |  | -0.7950 | 3.36e-49 | 4.38e-48 |
| ENSMUSG00000096917 | 2500002B13Rik | lncRNA | 8:57488054-57508877 (+) |  | -2.0900 | 3.59e-49 | 4.68e-48 |
| ENSMUSG00000056592 | Zfp658 | protein\_coding | 7:43562256-43575461 (+) |  | -1.7700 | 4.15e-49 | 5.41e-48 |
| ENSMUSG00000045322 | Tlr9 | protein\_coding | 9:106222598-106226883 (+) |  | -1.1700 | 4.31e-49 | 5.62e-48 |
| ENSMUSG00000031668 | Eif2ak3 | protein\_coding | 6:70844515-70905245 (+) |  | -0.7700 | 4.42e-49 | 5.75e-48 |
| ENSMUSG00000042389 | Tsen2 | protein\_coding | 6:115544664-115578628 (+) |  | 1.7500 | 4.44e-49 | 5.78e-48 |
| ENSMUSG00000032841 | Prr5l | protein\_coding | 2:101714285-101883027 (-) |  | -3.4700 | 5.02e-49 | 6.51e-48 |
| ENSMUSG00000094392 | Gm3788 | processed\_pseudogene | 3:86672033-86672168 (-) |  | -1.2100 | 5.19e-49 | 6.74e-48 |
| ENSMUSG00000041506 | Rrp9 | protein\_coding | 9:106475963-106485424 (+) |  | 1.1900 | 5.25e-49 | 6.81e-48 |
| ENSMUSG00000024579 | Pcyox1l | protein\_coding | 18:61696837-61707635 (-) |  | 1.5100 | 5.43e-49 | 7.03e-48 |
| ENSMUSG00000040675 | Mthfd1l | protein\_coding | 10:3973118-4167081 (+) |  | 0.7620 | 5.96e-49 | 7.72e-48 |
| ENSMUSG00000015806 | Qdpr | protein\_coding | 5:45434021-45450236 (-) |  | 1.0500 | 6.07e-49 | 7.86e-48 |
| ENSMUSG00000034459 | Ifit1 | protein\_coding | 19:34640871-34650009 (+) |  | -3.2700 | 6.21e-49 | 8.03e-48 |
| ENSMUSG00000056069 | Otulinl | protein\_coding | 15:27655069-27681579 (-) |  | -0.7810 | 7.73e-49 | 9.98e-48 |
| ENSMUSG00000078763 | Slfn1 | protein\_coding | 11:83116849-83122670 (+) |  | -3.8600 | 8.44e-49 | 1.09e-47 |
| ENSMUSG00000086290 | Snhg12 | lncRNA | 4:132308623-132311024 (+) |  | 1.4800 | 8.84e-49 | 1.14e-47 |
| ENSMUSG00000035575 | Utp6 | protein\_coding | 11:79932321-79962390 (-) |  | 0.6380 | 9.56e-49 | 1.23e-47 |
| ENSMUSG00000029713 | Gnb2 | protein\_coding | 5:137528127-137533510 (-) |  | -0.5680 | 9.69e-49 | 1.25e-47 |
| ENSMUSG00000020143 | Dock2 | protein\_coding | 11:34226815-34783892 (-) |  | -0.6310 | 9.72e-49 | 1.25e-47 |
| ENSMUSG00000029298 | Gbp9 | protein\_coding | 5:105077630-105139539 (-) |  | -2.6100 | 1.10e-48 | 1.41e-47 |
| ENSMUSG00000027883 | Gpsm2 | protein\_coding | 3:108678638-108722309 (-) |  | -1.1100 | 1.16e-48 | 1.49e-47 |
| ENSMUSG00000027615 | Hps3 | protein\_coding | 3:19995945-20035315 (-) |  | -1.1100 | 1.20e-48 | 1.53e-47 |
| ENSMUSG00000027519 | Rab22a | protein\_coding | 2:173659760-173707343 (+) |  | -0.6380 | 1.35e-48 | 1.73e-47 |
| ENSMUSG00000034354 | Mtmr3 | protein\_coding | 11:4480868-4594863 (-) |  | -0.5780 | 1.45e-48 | 1.85e-47 |
| ENSMUSG00000004677 | Myo9b | protein\_coding | 8:71272714-71360713 (+) |  | -0.6570 | 1.46e-48 | 1.86e-47 |
| ENSMUSG00000037344 | Slc12a9 | protein\_coding | 5:137314558-137333597 (-) |  | -0.7960 | 1.68e-48 | 2.15e-47 |
| ENSMUSG00000117771 | Gm25432 | processed\_pseudogene | 19:5435998-5436725 (+) |  | 1.8700 | 1.71e-48 | 2.19e-47 |
| ENSMUSG00000030287 | Itpr2 | protein\_coding | 6:146108299-146502223 (-) |  | -1.3800 | 1.72e-48 | 2.19e-47 |
| ENSMUSG00000037960 | Card19 | protein\_coding | 13:49202950-49216037 (-) |  | -1.1200 | 1.82e-48 | 2.32e-47 |
| ENSMUSG00000023913 | Pla2g7 | protein\_coding | 17:43568098-43612201 (+) |  | -4.2900 | 1.86e-48 | 2.37e-47 |
| ENSMUSG00000055782 | Abcd2 | protein\_coding | 15:91145871-91191799 (-) |  | -0.8680 | 2.02e-48 | 2.57e-47 |
| ENSMUSG00000020647 | Ncoa1 | protein\_coding | 12:4247362-4477182 (-) |  | -1.0500 | 2.07e-48 | 2.63e-47 |
| ENSMUSG00000003031 | Cdkn1b | protein\_coding | 6:134920401-134925513 (+) |  | -0.9310 | 2.07e-48 | 2.63e-47 |
| ENSMUSG00000078249 | Hmga1b | protein\_coding | 11:120762794-120764419 (+) |  | 1.3300 | 2.31e-48 | 2.93e-47 |
| ENSMUSG00000020700 | Map3k3 | protein\_coding | 11:106084613-106155446 (+) |  | -0.7940 | 2.31e-48 | 2.93e-47 |
| ENSMUSG00000032497 | Lrrfip2 | protein\_coding | 9:111117592-111225668 (+) |  | -0.8510 | 2.34e-48 | 2.96e-47 |
| ENSMUSG00000025439 | Clns1a | protein\_coding | 7:97696634-97720796 (+) |  | 0.6810 | 2.58e-48 | 3.27e-47 |
| ENSMUSG00000107355 | AI839979 | lncRNA | 5:31569595-31571397 (-) |  | -1.5300 | 2.67e-48 | 3.38e-47 |
| ENSMUSG00000074212 | Dnajb14 | protein\_coding | 3:137867675-137916557 (+) |  | -1.3600 | 2.95e-48 | 3.73e-47 |
| ENSMUSG00000006281 | Tep1 | protein\_coding | 14:50824059-50870560 (-) |  | -0.8970 | 3.17e-48 | 4.01e-47 |
| ENSMUSG00000014547 | Wdfy2 | protein\_coding | 14:62837678-62961509 (+) |  | -0.7340 | 3.74e-48 | 4.72e-47 |
| ENSMUSG00000025212 | Sfxn3 | protein\_coding | 19:45047503-45056383 (+) |  | -1.1300 | 3.79e-48 | 4.77e-47 |
| ENSMUSG00000021482 | Prxl2c | protein\_coding | 13:64275280-64312710 (-) |  | -0.9960 | 3.94e-48 | 4.96e-47 |
| ENSMUSG00000001707 | Eef1e1 | protein\_coding | 13:38644207-38659058 (-) |  | 1.2500 | 4.87e-48 | 6.13e-47 |
| ENSMUSG00000021597 | Slf1 | protein\_coding | 13:77043088-77135473 (-) |  | 0.9440 | 4.89e-48 | 6.15e-47 |
| ENSMUSG00000040950 | Mgl2 | protein\_coding | 11:70130329-70137550 (+) |  | 2.8200 | 5.10e-48 | 6.40e-47 |
| ENSMUSG00000004552 | Ctse | protein\_coding | 1:131638306-131675505 (+) |  | -1.2500 | 5.10e-48 | 6.40e-47 |
| ENSMUSG00000029135 | Fosl2 | protein\_coding | 5:32135801-32157842 (+) |  | -1.1300 | 5.18e-48 | 6.50e-47 |
| ENSMUSG00000024098 | Twsg1 | protein\_coding | 17:65921972-65951226 (-) |  | 1.0700 | 5.32e-48 | 6.66e-47 |
| ENSMUSG00000020167 | Tcf3 | protein\_coding | 10:80409514-80433647 (-) |  | 0.5790 | 5.52e-48 | 6.91e-47 |
| ENSMUSG00000026360 | Rgs2 | protein\_coding | 1:143999338-144004161 (-) |  | -1.3800 | 5.57e-48 | 6.97e-47 |
| ENSMUSG00000017774 | Myo1c | protein\_coding | 11:75650504-75673910 (+) |  | -0.7130 | 6.23e-48 | 7.79e-47 |
| ENSMUSG00000020644 | Id2 | protein\_coding | 12:25093799-25097140 (-) |  | -2.3000 | 6.78e-48 | 8.47e-47 |
| ENSMUSG00000054115 | Skp2 | protein\_coding | 15:9111985-9155425 (-) |  | 0.9420 | 7.23e-48 | 9.03e-47 |
| ENSMUSG00000025138 | Sirt7 | protein\_coding | 11:120618372-120625240 (-) |  | -0.6300 | 7.62e-48 | 9.50e-47 |
| ENSMUSG00000041231 | Ublcp1 | protein\_coding | 11:44454571-44470498 (-) |  | -1.6300 | 8.68e-48 | 1.08e-46 |
| ENSMUSG00000046207 | Pik3r6 | protein\_coding | 11:68503019-68552698 (+) |  | -0.8910 | 1.12e-47 | 1.40e-46 |
| ENSMUSG00000033955 | Tnks1bp1 | protein\_coding | 2:85048022-85073048 (+) |  | -1.3400 | 1.13e-47 | 1.41e-46 |
| ENSMUSG00000031984 | 2810004N23Rik | protein\_coding | 8:124836945-124863120 (-) |  | 0.8030 | 1.16e-47 | 1.44e-46 |
| ENSMUSG00000002983 | Relb | protein\_coding | 7:19606217-19629438 (-) |  | -1.9200 | 1.17e-47 | 1.46e-46 |
| ENSMUSG00000057315 | Arhgap24 | protein\_coding | 5:102481391-102897937 (+) |  | -1.3900 | 1.33e-47 | 1.65e-46 |
| ENSMUSG00000052212 | Cd177 | protein\_coding | 7:24743983-24760311 (-) |  | -2.5700 | 1.34e-47 | 1.66e-46 |
| ENSMUSG00000031931 | Ankrd49 | protein\_coding | 9:14779618-14784856 (-) |  | 0.7550 | 1.35e-47 | 1.67e-46 |
| ENSMUSG00000032691 | Nlrp3 | protein\_coding | 11:59541568-59566956 (+) |  | -1.4000 | 1.40e-47 | 1.73e-46 |
| ENSMUSG00000036273 | Lrrk2 | protein\_coding | 15:91673175-91816120 (+) |  | -1.1200 | 1.41e-47 | 1.74e-46 |
| ENSMUSG00000024212 | Mllt1 | protein\_coding | 17:56892611-56935415 (-) |  | 0.6230 | 1.46e-47 | 1.80e-46 |
| ENSMUSG00000033916 | Chmp2a | protein\_coding | 7:13032010-13034807 (-) |  | -0.8180 | 1.69e-47 | 2.09e-46 |
| ENSMUSG00000110682 | A530010L16Rik | lncRNA | 8:122760045-122763021 (-) |  | -2.0900 | 2.24e-47 | 2.76e-46 |
| ENSMUSG00000027580 | Helz2 | protein\_coding | 2:181227615-181242027 (-) |  | -1.5200 | 2.32e-47 | 2.85e-46 |
| ENSMUSG00000074039 | 4930520O04Rik | lncRNA | 9:114368303-114377229 (+) |  | 3.0500 | 2.50e-47 | 3.08e-46 |
| ENSMUSG00000079557 | March2 | protein\_coding | 17:33685692-33718670 (-) |  | -0.8170 | 2.53e-47 | 3.12e-46 |
| ENSMUSG00000027907 | S100a11 | protein\_coding | 3:93520488-93526287 (+) |  | -1.0300 | 2.63e-47 | 3.23e-46 |
| ENSMUSG00000038046 | Mrm3 | protein\_coding | 11:76243715-76250619 (+) |  | 1.4600 | 2.69e-47 | 3.30e-46 |
| ENSMUSG00000025558 | Dock9 | protein\_coding | 14:121542046-121797837 (-) |  | 3.5100 | 2.80e-47 | 3.44e-46 |
| ENSMUSG00000003062 | Stard3nl | protein\_coding | 13:19357676-19395795 (-) |  | -0.9030 | 2.88e-47 | 3.54e-46 |
| ENSMUSG00000006731 | B4galnt1 | protein\_coding | 10:127165225-127172330 (+) |  | 0.6770 | 2.96e-47 | 3.63e-46 |
| ENSMUSG00000022779 | Top3b | protein\_coding | 16:16870736-16892990 (+) |  | 0.9970 | 3.35e-47 | 4.10e-46 |
| ENSMUSG00000025209 | Twnk | protein\_coding | 19:45005663-45012762 (+) |  | 1.2400 | 3.56e-47 | 4.36e-46 |
| ENSMUSG00000002660 | Clpp | protein\_coding | 17:56990305-56996188 (+) |  | 1.0100 | 3.63e-47 | 4.43e-46 |
| ENSMUSG00000029363 | Rfc5 | protein\_coding | 5:117378103-117389047 (-) |  | 0.8090 | 3.66e-47 | 4.47e-46 |
| ENSMUSG00000033294 | Noc4l | protein\_coding | 5:110648418-110653417 (-) |  | 0.9790 | 3.82e-47 | 4.66e-46 |
| ENSMUSG00000038733 | Wdr26 | protein\_coding | 1:181173228-181212001 (-) |  | -0.5920 | 4.13e-47 | 5.03e-46 |
| ENSMUSG00000020739 | Nup85 | protein\_coding | 11:115564434-115583985 (+) |  | 0.8710 | 4.17e-47 | 5.08e-46 |
| ENSMUSG00000071657 | Bscl2 | protein\_coding | 19:8837467-8848683 (+) |  | -0.7000 | 4.36e-47 | 5.31e-46 |
| ENSMUSG00000005802 | Slc30a4 | protein\_coding | 2:122681233-122702663 (-) |  | 2.0000 | 4.98e-47 | 6.06e-46 |
| ENSMUSG00000062421 | Arf2 | protein\_coding | 11:103966739-103985337 (+) |  | -0.8020 | 5.17e-47 | 6.29e-46 |
| ENSMUSG00000079036 | Alkbh1 | protein\_coding | 12:87425840-87444017 (-) |  | 0.9820 | 5.24e-47 | 6.36e-46 |
| ENSMUSG00000020806 | Rhbdf2 | protein\_coding | 11:116598165-116627019 (-) |  | -1.0800 | 5.51e-47 | 6.68e-46 |
| ENSMUSG00000046410 | Kcnk6 | protein\_coding | 7:29221926-29232515 (-) |  | -0.6600 | 5.56e-47 | 6.74e-46 |
| ENSMUSG00000078700 | D030028A08Rik | lncRNA | 11:96916270-96965060 (+) |  | 1.6500 | 5.61e-47 | 6.80e-46 |
| ENSMUSG00000022554 | Hgh1 | protein\_coding | 15:76368898-76371437 (+) |  | 1.5000 | 6.22e-47 | 7.54e-46 |
| ENSMUSG00000017999 | Ddx27 | protein\_coding | 2:167015193-167034947 (+) |  | 0.8020 | 6.28e-47 | 7.59e-46 |
| ENSMUSG00000044229 | Nxpe4 | protein\_coding | 9:48162023-48400025 (+) |  | -2.4800 | 6.38e-47 | 7.72e-46 |
| ENSMUSG00000042105 | Inpp5f | protein\_coding | 7:128611328-128696425 (+) |  | -1.0200 | 7.51e-47 | 9.06e-46 |
| ENSMUSG00000018379 | Srsf1 | protein\_coding | 11:88047373-88053755 (+) |  | 0.5840 | 7.56e-47 | 9.12e-46 |
| ENSMUSG00000069516 | Lyz2 | protein\_coding | 10:117277331-117282321 (-) |  | -2.5000 | 7.79e-47 | 9.39e-46 |
| ENSMUSG00000026519 | Tmem63a | protein\_coding | 1:180942344-180975112 (+) |  | -0.8000 | 8.71e-47 | 1.05e-45 |
| ENSMUSG00000044452 | Zfp507 | protein\_coding | 7:35772343-35803003 (-) |  | 3.8500 | 8.83e-47 | 1.06e-45 |
| ENSMUSG00000020358 | Hnrnpab | protein\_coding | 11:51600100-51606847 (-) |  | 0.7910 | 9.25e-47 | 1.11e-45 |
| ENSMUSG00000030123 | Plxnd1 | protein\_coding | 6:115954811-115995005 (-) |  | -1.0500 | 9.45e-47 | 1.14e-45 |
| ENSMUSG00000022748 | Cmss1 | protein\_coding | 16:57302000-57606864 (-) |  | 1.6300 | 1.02e-46 | 1.22e-45 |
| ENSMUSG00000034487 | Poglut3 | protein\_coding | 9:53384025-53401867 (+) |  | 1.2600 | 1.04e-46 | 1.25e-45 |
| ENSMUSG00000041308 | Sntb2 | protein\_coding | 8:106935750-107019714 (+) |  | -0.7170 | 1.15e-46 | 1.38e-45 |
| ENSMUSG00000037922 | Bank1 | protein\_coding | 3:136053363-136326066 (-) |  | -2.3900 | 1.28e-46 | 1.53e-45 |
| ENSMUSG00000051314 | Ffar2 | protein\_coding | 7:30818348-30823775 (-) |  | 2.9700 | 1.36e-46 | 1.63e-45 |
| ENSMUSG00000026627 | Pacc1 | protein\_coding | 1:191325912-191350914 (+) |  | 1.0900 | 1.47e-46 | 1.76e-45 |
| ENSMUSG00000028995 | Fam126a | protein\_coding | 5:23915276-24030690 (-) |  | -0.7920 | 1.49e-46 | 1.78e-45 |
| ENSMUSG00000025486 | Sirt3 | protein\_coding | 7:140863666-140882309 (-) |  | 1.1400 | 1.51e-46 | 1.80e-45 |
| ENSMUSG00000023963 | Cyp39a1 | protein\_coding | 17:43667425-43751431 (+) |  | -1.4600 | 1.54e-46 | 1.84e-45 |
| ENSMUSG00000049553 | Polr1a | protein\_coding | 6:71909053-71984935 (+) |  | 1.0200 | 1.75e-46 | 2.08e-45 |
| ENSMUSG00000030966 | Trim21 | protein\_coding | 7:102557921-102565486 (-) |  | -0.8050 | 1.90e-46 | 2.27e-45 |
| ENSMUSG00000069792 | Wfdc17 | protein\_coding | 11:83703991-83706268 (+) |  | -3.1400 | 2.61e-46 | 3.11e-45 |
| ENSMUSG00000032014 | Oaf | protein\_coding | 9:43221235-43239911 (-) |  | 1.4400 | 2.64e-46 | 3.14e-45 |
| ENSMUSG00000018507 | Trpv2 | protein\_coding | 11:62574486-62600515 (+) |  | -0.8240 | 2.93e-46 | 3.48e-45 |
| ENSMUSG00000028948 | Nol9 | protein\_coding | 4:152039321-152061494 (+) |  | 1.0000 | 2.94e-46 | 3.49e-45 |
| ENSMUSG00000034321 | Exosc1 | protein\_coding | 19:41922292-41933423 (-) |  | 0.9080 | 3.10e-46 | 3.68e-45 |
| ENSMUSG00000062995 | Ica1 | protein\_coding | 6:8630527-8778488 (-) |  | 3.9500 | 3.26e-46 | 3.87e-45 |
| ENSMUSG00000022788 | Fgd4 | protein\_coding | 16:16416917-16600549 (-) |  | -1.1100 | 3.30e-46 | 3.91e-45 |
| ENSMUSG00000031575 | Ash2l | protein\_coding | 8:25815996-25847694 (-) |  | 0.6010 | 3.32e-46 | 3.93e-45 |
| ENSMUSG00000051832 | E230016K23Rik | lncRNA | 11:83582056-83623693 (+) |  | -2.4600 | 3.35e-46 | 3.96e-45 |
| ENSMUSG00000112825 | Gm9118 | processed\_pseudogene | 10:56497341-56498094 (+) |  | 1.4200 | 3.68e-46 | 4.35e-45 |
| ENSMUSG00000054051 | Ercc6 | protein\_coding | 14:32513521-32580990 (+) |  | 0.8370 | 3.87e-46 | 4.57e-45 |
| ENSMUSG00000039193 | Nlrc4 | protein\_coding | 17:74425438-74459142 (-) |  | -0.7700 | 4.83e-46 | 5.69e-45 |
| ENSMUSG00000019978 | Epb41l2 | protein\_coding | 10:25359798-25523519 (+) |  | -0.6990 | 4.98e-46 | 5.87e-45 |
| ENSMUSG00000032690 | Oas2 | protein\_coding | 5:120730333-120749853 (-) |  | -3.9400 | 5.03e-46 | 5.92e-45 |
| ENSMUSG00000028064 | Sema4a | protein\_coding | 3:88435959-88461182 (-) |  | -0.6750 | 5.20e-46 | 6.12e-45 |
| ENSMUSG00000035443 | Thyn1 | protein\_coding | 9:26999710-27007336 (+) |  | 0.7410 | 5.42e-46 | 6.38e-45 |
| ENSMUSG00000026615 | Eprs | protein\_coding | 1:185363044-185428360 (+) |  | 1.0500 | 5.57e-46 | 6.55e-45 |
| ENSMUSG00000032834 | Pwp2 | protein\_coding | 10:78170909-78185149 (-) |  | 1.0400 | 6.11e-46 | 7.17e-45 |
| ENSMUSG00000028479 | Gne | protein\_coding | 4:44034075-44084177 (-) |  | 0.9550 | 6.15e-46 | 7.22e-45 |
| ENSMUSG00000033777 | Tlr13 | protein\_coding | X:106143204-106160493 (+) |  | -2.6400 | 6.70e-46 | 7.85e-45 |
| ENSMUSG00000058392 | Rrp1b | protein\_coding | 17:32036100-32062865 (+) |  | 0.9960 | 6.81e-46 | 7.98e-45 |
| ENSMUSG00000044583 | Tlr7 | protein\_coding | X:167304929-167330558 (-) |  | -3.2600 | 6.90e-46 | 8.07e-45 |
| ENSMUSG00000028874 | Fgr | protein\_coding | 4:132974095-133001910 (+) |  | -3.2600 | 6.96e-46 | 8.14e-45 |
| ENSMUSG00000044749 | Abca6 | protein\_coding | 11:110176820-110251776 (-) |  | -3.4400 | 6.96e-46 | 8.14e-45 |
| ENSMUSG00000038368 | Focad | protein\_coding | 4:88094629-88411011 (+) |  | 1.5100 | 7.23e-46 | 8.44e-45 |
| ENSMUSG00000024952 | Rps6ka4 | protein\_coding | 19:6829085-6840636 (-) |  | -0.7850 | 7.89e-46 | 9.20e-45 |
| ENSMUSG00000022517 | Mgrn1 | protein\_coding | 16:4886249-4938296 (+) |  | -0.7780 | 7.99e-46 | 9.32e-45 |
| ENSMUSG00000045664 | Cdc42ep2 | protein\_coding | 19:5915636-5924816 (-) |  | -3.2900 | 9.04e-46 | 1.05e-44 |
| ENSMUSG00000025810 | Nrp1 | protein\_coding | 8:128358604-128503363 (+) |  | -0.9980 | 1.07e-45 | 1.24e-44 |
| ENSMUSG00000020453 | Patz1 | protein\_coding | 11:3288874-3309083 (+) |  | 0.8380 | 1.09e-45 | 1.27e-44 |
| ENSMUSG00000024944 | Arl2 | protein\_coding | 19:6134374-6141548 (-) |  | 0.9860 | 1.12e-45 | 1.31e-44 |
| ENSMUSG00000027712 | Anxa5 | protein\_coding | 3:36448923-36475894 (-) |  | -0.9660 | 1.18e-45 | 1.38e-44 |
| ENSMUSG00000020089 | Ppa1 | protein\_coding | 10:61648552-61674168 (+) |  | 1.6100 | 1.20e-45 | 1.40e-44 |
| ENSMUSG00000038467 | Chmp4b | protein\_coding | 2:154651705-154694785 (+) |  | -0.5890 | 1.24e-45 | 1.44e-44 |
| ENSMUSG00000028218 | Fam92a | protein\_coding | 4:12153409-12172015 (-) |  | 1.7700 | 1.49e-45 | 1.73e-44 |
| ENSMUSG00000034177 | Rnf43 | protein\_coding | 11:87662722-87735539 (+) |  | 2.0700 | 1.52e-45 | 1.76e-44 |
| ENSMUSG00000031016 | Wee1 | protein\_coding | 7:110122046-110143286 (+) |  | 0.9290 | 1.61e-45 | 1.87e-44 |
| ENSMUSG00000038633 | Degs1 | protein\_coding | 1:182275772-182282804 (-) |  | -0.9390 | 1.78e-45 | 2.06e-44 |
| ENSMUSG00000022157 | Mcpt8 | protein\_coding | 14:56082166-56085273 (-) |  | 2.9600 | 1.79e-45 | 2.07e-44 |
| ENSMUSG00000026504 | Sdccag8 | protein\_coding | 1:176814660-177020437 (+) |  | -0.9890 | 2.00e-45 | 2.31e-44 |
| ENSMUSG00000039217 | Il18 | protein\_coding | 9:50554827-50581840 (+) |  | -2.9700 | 2.11e-45 | 2.44e-44 |
| ENSMUSG00000070319 | Eif3g | protein\_coding | 9:20894349-20898623 (-) |  | 0.6520 | 2.12e-45 | 2.44e-44 |
| ENSMUSG00000002996 | Hbp1 | protein\_coding | 12:31926254-31950535 (-) |  | -0.9920 | 2.67e-45 | 3.07e-44 |
| ENSMUSG00000031697 | Orc6 | protein\_coding | 8:85299632-85308278 (+) |  | 0.9100 | 2.69e-45 | 3.10e-44 |
| ENSMUSG00000040859 | Bsdc1 | protein\_coding | 4:129461581-129488498 (+) |  | -0.8050 | 2.70e-45 | 3.11e-44 |
| ENSMUSG00000059552 | Trp53 | protein\_coding | 11:69580359-69591873 (+) |  | 0.9140 | 2.75e-45 | 3.17e-44 |
| ENSMUSG00000024165 | Jpt2 | protein\_coding | 17:24937419-24960689 (-) |  | 0.9560 | 2.82e-45 | 3.24e-44 |
| ENSMUSG00000066975 | Cryba4 | protein\_coding | 5:112246493-112252518 (-) |  | -3.4000 | 2.88e-45 | 3.31e-44 |
| ENSMUSG00000024498 | Tcerg1 | protein\_coding | 18:42511487-42575793 (+) |  | 0.7960 | 3.26e-45 | 3.73e-44 |
| ENSMUSG00000038712 | Mindy1 | protein\_coding | 3:95281345-95296166 (+) |  | -0.7320 | 3.36e-45 | 3.85e-44 |
| ENSMUSG00000032562 | Gnai2 | protein\_coding | 9:107614125-107635367 (-) |  | -0.6110 | 3.41e-45 | 3.90e-44 |
| ENSMUSG00000028034 | Fubp1 | protein\_coding | 3:152210422-152236826 (+) |  | 0.6950 | 3.45e-45 | 3.95e-44 |
| ENSMUSG00000026981 | Il1rn | protein\_coding | 2:24336853-24351494 (+) |  | -2.1000 | 3.47e-45 | 3.97e-44 |
| ENSMUSG00000037149 | Ddx1 | protein\_coding | 12:13216973-13249213 (-) |  | 1.0400 | 3.57e-45 | 4.08e-44 |
| ENSMUSG00000018167 | Stard3 | protein\_coding | 11:98358368-98381112 (+) |  | -0.9040 | 3.60e-45 | 4.11e-44 |
| ENSMUSG00000052749 | Trim30b | protein\_coding | 7:104355382-104369884 (-) |  | -2.0800 | 3.68e-45 | 4.19e-44 |
| ENSMUSG00000041836 | Ptpre | protein\_coding | 7:135537481-135686293 (+) |  | -0.8410 | 4.04e-45 | 4.61e-44 |
| ENSMUSG00000024013 | Fgd2 | protein\_coding | 17:29360914-29379661 (+) |  | 0.7710 | 4.76e-45 | 5.42e-44 |
| ENSMUSG00000038930 | Rccd1 | protein\_coding | 7:80293229-80324454 (-) |  | 1.7000 | 4.99e-45 | 5.67e-44 |
| ENSMUSG00000095115 | Itpripl2 | protein\_coding | 7:118485111-118491975 (-) |  | -0.6090 | 5.41e-45 | 6.15e-44 |
| ENSMUSG00000053907 | Mat2a | protein\_coding | 6:72432799-72439558 (-) |  | 0.8560 | 5.55e-45 | 6.31e-44 |
| ENSMUSG00000018415 | Gid4 | protein\_coding | 11:60417145-60450927 (+) |  | 0.9500 | 5.97e-45 | 6.77e-44 |
| ENSMUSG00000063273 | Naa15 | protein\_coding | 3:51415148-51476507 (+) |  | 0.6940 | 5.97e-45 | 6.77e-44 |
| ENSMUSG00000047098 | Rnf31 | protein\_coding | 14:55591708-55603693 (+) |  | -0.6410 | 5.99e-45 | 6.79e-44 |
| ENSMUSG00000024617 | Camk2a | protein\_coding | 18:60925618-60988152 (+) |  | 3.4500 | 6.02e-45 | 6.82e-44 |
| ENSMUSG00000037138 | Aff3 | protein\_coding | 1:38177326-38664955 (-) |  | 5.0500 | 6.26e-45 | 7.08e-44 |
| ENSMUSG00000023994 | Nfya | protein\_coding | 17:48386885-48409906 (-) |  | 0.4680 | 6.33e-45 | 7.16e-44 |
| ENSMUSG00000031683 | Lsm6 | protein\_coding | 8:78804865-78821140 (-) |  | 0.7210 | 7.53e-45 | 8.51e-44 |
| ENSMUSG00000024067 | Dpy30 | protein\_coding | 17:74299474-74323944 (-) |  | 0.9010 | 7.81e-45 | 8.82e-44 |
| ENSMUSG00000034793 | G6pc3 | protein\_coding | 11:102189620-102194081 (+) |  | 1.2000 | 7.83e-45 | 8.83e-44 |
| ENSMUSG00000024457 | Trim26 | protein\_coding | 17:36837134-36859398 (+) |  | -0.9090 | 8.12e-45 | 9.16e-44 |
| ENSMUSG00000061981 | Flot2 | protein\_coding | 11:78037931-78060434 (+) |  | -0.7510 | 8.49e-45 | 9.57e-44 |
| ENSMUSG00000021374 | Nup153 | protein\_coding | 13:46679905-46727940 (-) |  | 0.6090 | 9.85e-45 | 1.11e-43 |
| ENSMUSG00000036553 | Sh3tc1 | protein\_coding | 5:35697180-35739987 (-) |  | -0.8210 | 1.10e-44 | 1.23e-43 |
| ENSMUSG00000027605 | Acss2 | protein\_coding | 2:155517948-155585724 (+) |  | -1.3000 | 1.10e-44 | 1.23e-43 |
| ENSMUSG00000063659 | Zbtb18 | protein\_coding | 1:177442351-177450764 (+) |  | 0.6300 | 1.15e-44 | 1.29e-43 |
| ENSMUSG00000036225 | Kctd1 | protein\_coding | 18:14968685-15151446 (-) |  | 1.7100 | 1.17e-44 | 1.31e-43 |
| ENSMUSG00000059810 | Rgs3 | protein\_coding | 4:62559847-62704001 (+) |  | -1.2500 | 1.29e-44 | 1.45e-43 |
| ENSMUSG00000042507 | Elmsan1 | protein\_coding | 12:84149176-84218881 (-) |  | -0.8150 | 1.36e-44 | 1.52e-43 |
| ENSMUSG00000039501 | Znfx1 | protein\_coding | 2:167035793-167063015 (-) |  | -0.9090 | 1.50e-44 | 1.68e-43 |
| ENSMUSG00000052776 | Oas1a | protein\_coding | 5:120896256-120907521 (-) |  | -2.0200 | 1.54e-44 | 1.72e-43 |
| ENSMUSG00000060036 | Rpl3 | protein\_coding | 15:80077791-80091868 (-) |  | 0.6050 | 1.72e-44 | 1.92e-43 |
| ENSMUSG00000026321 | Tnfrsf11a | protein\_coding | 1:105780718-105847981 (+) |  | -1.7700 | 1.82e-44 | 2.04e-43 |
| ENSMUSG00000073676 | Hspe1 | protein\_coding | 1:55088132-55091307 (+) |  | 1.2700 | 1.83e-44 | 2.04e-43 |
| ENSMUSG00000039640 | Mrpl12 | protein\_coding | 11:120484613-120489065 (+) |  | 1.2400 | 1.85e-44 | 2.06e-43 |
| ENSMUSG00000039735 | Fnbp1l | protein\_coding | 3:122538719-122619715 (-) |  | 1.8200 | 2.00e-44 | 2.23e-43 |
| ENSMUSG00000004996 | Mri1 | protein\_coding | 8:84249906-84257326 (-) |  | 1.0000 | 2.26e-44 | 2.52e-43 |
| ENSMUSG00000033685 | Ucp2 | protein\_coding | 7:100493337-100502020 (+) |  | -0.7380 | 2.34e-44 | 2.60e-43 |
| ENSMUSG00000035692 | Isg15 | protein\_coding | 4:156199455-156200796 (-) |  | -2.6500 | 2.48e-44 | 2.76e-43 |
| ENSMUSG00000001418 | Glmp | protein\_coding | 3:88325023-88331313 (+) |  | -0.6080 | 2.57e-44 | 2.86e-43 |
| ENSMUSG00000052821 | Cysltr1 | protein\_coding | X:106574346-106603679 (-) |  | -1.6900 | 2.94e-44 | 3.26e-43 |
| ENSMUSG00000036353 | P2ry12 | protein\_coding | 3:59216272-59262871 (-) |  | -2.8500 | 2.98e-44 | 3.30e-43 |
| ENSMUSG00000021143 | Pacs2 | protein\_coding | 12:113014508-113074401 (+) |  | -0.9230 | 3.11e-44 | 3.45e-43 |
| ENSMUSG00000022557 | Bop1 | protein\_coding | 15:76452989-76477277 (-) |  | 0.8750 | 3.30e-44 | 3.66e-43 |
| ENSMUSG00000019920 | Lims1 | protein\_coding | 10:58323466-58424691 (+) |  | -0.8910 | 3.55e-44 | 3.93e-43 |
| ENSMUSG00000096472 | Cdkn2d | protein\_coding | 9:21288410-21291407 (-) |  | -1.1600 | 3.83e-44 | 4.24e-43 |
| ENSMUSG00000027349 | Fam98b | protein\_coding | 2:117249739-117271540 (+) |  | 0.8250 | 4.01e-44 | 4.43e-43 |
| ENSMUSG00000106734 | Gm20559 | lncRNA | 6:3333194-3346128 (-) |  | -2.0700 | 4.08e-44 | 4.51e-43 |
| ENSMUSG00000004709 | Cd244a | protein\_coding | 1:171559193-171609746 (+) |  | -1.5700 | 4.18e-44 | 4.61e-43 |
| ENSMUSG00000073491 | Ifi213 | protein\_coding | 1:173566283-173599274 (-) |  | -3.9600 | 4.20e-44 | 4.64e-43 |
| ENSMUSG00000027678 | Ncoa3 | protein\_coding | 2:165992636-166073242 (+) |  | -0.8800 | 4.41e-44 | 4.86e-43 |
| ENSMUSG00000020527 | Myo19 | protein\_coding | 11:84880148-84911226 (+) |  | 1.5800 | 5.44e-44 | 5.99e-43 |
| ENSMUSG00000024833 | Pola2 | protein\_coding | 19:5940542-5964202 (-) |  | 1.0500 | 5.86e-44 | 6.45e-43 |
| ENSMUSG00000079139 | Gm4204 | processed\_pseudogene | 1:135231995-135233225 (+) |  | 0.6720 | 5.99e-44 | 6.59e-43 |
| ENSMUSG00000089901 | Gm8113 | protein\_coding | 14:43925390-43933411 (+) |  | 3.8200 | 6.20e-44 | 6.82e-43 |
| ENSMUSG00000031765 | Mt1 | protein\_coding | 8:94179082-94180327 (+) |  | 2.0200 | 6.37e-44 | 7.00e-43 |
| ENSMUSG00000002319 | Ipo4 | protein\_coding | 14:55625400-55635957 (-) |  | 1.3400 | 6.58e-44 | 7.23e-43 |
| ENSMUSG00000043671 | Dpy19l3 | protein\_coding | 7:35685165-35754454 (-) |  | 2.3400 | 7.03e-44 | 7.71e-43 |
| ENSMUSG00000022752 | Tomm70a | protein\_coding | 16:57121703-57156705 (+) |  | 0.8420 | 7.17e-44 | 7.86e-43 |
| ENSMUSG00000027165 | B230118H07Rik | protein\_coding | 2:101560781-101649532 (-) |  | 1.5800 | 7.50e-44 | 8.22e-43 |
| ENSMUSG00000020132 | Rab21 | protein\_coding | 10:115287084-115315594 (-) |  | -0.4580 | 8.41e-44 | 9.21e-43 |
| ENSMUSG00000020573 | Pik3cg | protein\_coding | 12:32173473-32208659 (-) |  | -0.6980 | 9.05e-44 | 9.90e-43 |
| ENSMUSG00000026104 | Stat1 | protein\_coding | 1:52119440-52161865 (+) |  | -1.0300 | 9.22e-44 | 1.01e-42 |
| ENSMUSG00000078429 | Ctdsp2 | protein\_coding | 10:126978717-126999975 (+) |  | -0.7630 | 9.57e-44 | 1.05e-42 |
| ENSMUSG00000002274 | Metrn | protein\_coding | 17:25793221-25797136 (-) |  | 1.0200 | 1.03e-43 | 1.12e-42 |
| ENSMUSG00000023944 | Hsp90ab1 | protein\_coding | 17:45567775-45573271 (-) |  | 1.1700 | 1.03e-43 | 1.12e-42 |
| ENSMUSG00000000530 | Acvrl1 | protein\_coding | 15:101128522-101145336 (+) |  | -1.5700 | 1.06e-43 | 1.15e-42 |
| ENSMUSG00000038910 | Plcl2 | protein\_coding | 17:50509403-50688484 (+) |  | -0.5830 | 1.08e-43 | 1.18e-42 |
| ENSMUSG00000013846 | St3gal1 | protein\_coding | 15:67102875-67176830 (-) |  | -1.1800 | 1.12e-43 | 1.22e-42 |
| ENSMUSG00000003531 | Dgcr6 | protein\_coding | 16:18052860-18071632 (+) |  | 1.3500 | 1.17e-43 | 1.27e-42 |
| ENSMUSG00000024858 | Grk2 | protein\_coding | 19:4286001-4306222 (-) |  | -0.4060 | 1.31e-43 | 1.42e-42 |
| ENSMUSG00000020328 | Nudcd2 | protein\_coding | 11:40733667-40740046 (+) |  | 0.9690 | 1.36e-43 | 1.48e-42 |
| ENSMUSG00000029570 | Lfng | protein\_coding | 5:140607320-140615545 (+) |  | -1.7400 | 1.39e-43 | 1.51e-42 |
| ENSMUSG00000003955 | Fam162a | protein\_coding | 16:36043761-36071594 (-) |  | 1.1600 | 1.53e-43 | 1.66e-42 |
| ENSMUSG00000066595 | Flvcr1 | protein\_coding | 1:191005847-191026158 (-) |  | -0.8640 | 1.54e-43 | 1.67e-42 |
| ENSMUSG00000038644 | Pold1 | protein\_coding | 7:44532746-44548849 (-) |  | 0.9700 | 1.56e-43 | 1.69e-42 |
| ENSMUSG00000050567 | Maml1 | protein\_coding | 11:50255634-50292311 (-) |  | -0.8200 | 1.93e-43 | 2.09e-42 |
| ENSMUSG00000055491 | Pprc1 | protein\_coding | 19:46044886-46072915 (+) |  | 1.1500 | 1.94e-43 | 2.10e-42 |
| ENSMUSG00000073412 | Lst1 | protein\_coding | 17:35185095-35188439 (-) |  | -1.8200 | 2.11e-43 | 2.28e-42 |
| ENSMUSG00000021298 | Gpr132 | protein\_coding | 12:112850873-112868228 (-) |  | -1.4400 | 2.15e-43 | 2.32e-42 |
| ENSMUSG00000062661 | Ncs1 | protein\_coding | 2:31245823-31295989 (+) |  | 3.2800 | 2.34e-43 | 2.53e-42 |
| ENSMUSG00000017132 | Cyth1 | protein\_coding | 11:118132019-118248592 (-) |  | -0.8260 | 2.69e-43 | 2.90e-42 |
| ENSMUSG00000029622 | Arpc1b | protein\_coding | 5:145114215-145130705 (+) |  | -0.5590 | 2.71e-43 | 2.92e-42 |
| ENSMUSG00000107383 | Gm4366 | processed\_pseudogene | 7:116824510-116825851 (-) |  | 1.3400 | 2.87e-43 | 3.08e-42 |
| ENSMUSG00000042363 | Lgalsl | protein\_coding | 11:20823576-20831056 (-) |  | 2.0400 | 2.87e-43 | 3.08e-42 |
| ENSMUSG00000030980 | Knop1 | protein\_coding | 7:118842222-118856254 (-) |  | 0.7610 | 2.87e-43 | 3.08e-42 |
| ENSMUSG00000020376 | Rnf130 | protein\_coding | 11:50025346-50125719 (+) |  | -0.7670 | 2.90e-43 | 3.11e-42 |
| ENSMUSG00000029998 | Pcyox1 | protein\_coding | 6:86386006-86397154 (-) |  | -0.5340 | 3.16e-43 | 3.40e-42 |
| ENSMUSG00000029610 | Aimp2 | protein\_coding | 5:143902704-143909847 (-) |  | 1.1200 | 3.54e-43 | 3.80e-42 |
| ENSMUSG00000035049 | Rrp12 | protein\_coding | 19:41862851-41896173 (-) |  | 1.2700 | 3.58e-43 | 3.84e-42 |
| ENSMUSG00000006360 | Crip1 | protein\_coding | 12:113146316-113153879 (+) |  | -1.4100 | 3.66e-43 | 3.92e-42 |
| ENSMUSG00000063480 | Snu13 | protein\_coding | 15:82040525-82047598 (-) |  | 0.9470 | 4.08e-43 | 4.36e-42 |
| ENSMUSG00000028884 | Rpa2 | protein\_coding | 4:132768332-132778752 (+) |  | 1.0800 | 4.22e-43 | 4.51e-42 |
| ENSMUSG00000042694 | Stn1 | protein\_coding | 19:47501033-47537507 (-) |  | -1.1400 | 5.07e-43 | 5.42e-42 |
| ENSMUSG00000001588 | Acap1 | protein\_coding | 11:69881567-69895539 (-) |  | 0.9720 | 5.19e-43 | 5.55e-42 |
| ENSMUSG00000032235 | Ice2 | protein\_coding | 9:69397906-69433122 (+) |  | 0.9630 | 5.28e-43 | 5.64e-42 |
| ENSMUSG00000025899 | Alkbh8 | protein\_coding | 9:3335140-3391154 (+) |  | 0.9160 | 5.37e-43 | 5.73e-42 |
| ENSMUSG00000039621 | Prex1 | protein\_coding | 2:166566342-166713832 (-) |  | -0.8660 | 5.44e-43 | 5.80e-42 |
| ENSMUSG00000054293 | P2ry10b | protein\_coding | X:107148927-107173661 (+) |  | -2.3800 | 5.69e-43 | 6.06e-42 |
| ENSMUSG00000008540 | Mgst1 | protein\_coding | 6:138140316-138156755 (+) |  | -1.2800 | 6.29e-43 | 6.69e-42 |
| ENSMUSG00000038151 | Prdm1 | protein\_coding | 10:44437177-44528501 (-) |  | -3.1500 | 6.36e-43 | 6.77e-42 |
| ENSMUSG00000035835 | Plppr3 | protein\_coding | 10:79860475-79874634 (-) |  | 1.6200 | 6.72e-43 | 7.14e-42 |
| ENSMUSG00000010554 | Mettl16 | protein\_coding | 11:74770830-74828525 (+) |  | 0.9670 | 7.17e-43 | 7.61e-42 |
| ENSMUSG00000026249 | Serpine2 | protein\_coding | 1:79794197-79861180 (-) |  | 3.2600 | 7.51e-43 | 7.97e-42 |
| ENSMUSG00000085385 | Snhg17 | lncRNA | 2:158353700-158361580 (-) |  | 1.3000 | 8.22e-43 | 8.72e-42 |
| ENSMUSG00000026928 | Card9 | protein\_coding | 2:26352176-26360918 (-) |  | -0.5110 | 8.62e-43 | 9.14e-42 |
| ENSMUSG00000024730 | Ms4a8a | protein\_coding | 19:11067471-11081102 (-) |  | -3.7200 | 9.09e-43 | 9.62e-42 |
| ENSMUSG00000025574 | Tk1 | protein\_coding | 11:117815526-117826092 (-) |  | 0.9620 | 9.15e-43 | 9.69e-42 |
| ENSMUSG00000035203 | Epn1 | protein\_coding | 7:5080235-5098178 (+) |  | -0.7890 | 9.96e-43 | 1.05e-41 |
| ENSMUSG00000026305 | Lrrfip1 | protein\_coding | 1:90998737-91128944 (+) |  | -0.5180 | 1.00e-42 | 1.06e-41 |
| ENSMUSG00000005718 | Tfap4 | protein\_coding | 16:4544661-4559854 (-) |  | 1.5300 | 1.08e-42 | 1.14e-41 |
| ENSMUSG00000048120 | Entpd1 | protein\_coding | 19:40612366-40741602 (+) |  | -1.6300 | 1.18e-42 | 1.25e-41 |
| ENSMUSG00000024735 | Prpf19 | protein\_coding | 19:10895231-10909559 (+) |  | 0.7690 | 1.19e-42 | 1.26e-41 |
| ENSMUSG00000032322 | Pstpip1 | protein\_coding | 9:56089962-56128888 (+) |  | -0.7230 | 1.19e-42 | 1.26e-41 |
| ENSMUSG00000026245 | Farsb | protein\_coding | 1:78417975-78488897 (-) |  | 0.8900 | 1.20e-42 | 1.26e-41 |
| ENSMUSG00000032279 | Idh3a | protein\_coding | 9:54586334-54604661 (+) |  | 1.0900 | 1.21e-42 | 1.27e-41 |
| ENSMUSG00000049916 | 2610318N02Rik | protein\_coding | 16:17113398-17125167 (-) |  | 1.9500 | 1.24e-42 | 1.31e-41 |
| ENSMUSG00000060601 | Nr1h2 | protein\_coding | 7:44549616-44553951 (-) |  | -0.4830 | 1.32e-42 | 1.38e-41 |
| ENSMUSG00000029621 | Arpc1a | protein\_coding | 5:145083830-145108761 (+) |  | -0.5490 | 1.36e-42 | 1.43e-41 |
| ENSMUSG00000015522 | Arnt | protein\_coding | 3:95434388-95497240 (+) |  | -0.6370 | 1.44e-42 | 1.51e-41 |
| ENSMUSG00000039096 | Rsad1 | protein\_coding | 11:94539798-94549255 (-) |  | 2.6800 | 1.50e-42 | 1.57e-41 |
| ENSMUSG00000094796 | BC147527 | protein\_coding | 13:120300392-120308779 (+) |  | -1.5200 | 1.57e-42 | 1.65e-41 |
| ENSMUSG00000062825 | Actg1 | protein\_coding | 11:120345690-120348542 (-) |  | -0.5490 | 1.61e-42 | 1.68e-41 |
| ENSMUSG00000049130 | C5ar1 | protein\_coding | 7:16246743-16259540 (-) |  | -2.6700 | 1.64e-42 | 1.72e-41 |
| ENSMUSG00000049401 | Ogfr | protein\_coding | 2:180589245-180595836 (+) |  | -0.6620 | 1.93e-42 | 2.01e-41 |
| ENSMUSG00000028318 | Polr1e | protein\_coding | 4:45018583-45036565 (+) |  | 1.0500 | 2.16e-42 | 2.26e-41 |
| ENSMUSG00000022526 | Zfp251 | protein\_coding | 15:76851609-76871435 (-) |  | 1.0900 | 2.38e-42 | 2.49e-41 |
| ENSMUSG00000073529 | F830208F22Rik | lncRNA | 18:77793259-77796743 (-) |  | -1.9800 | 2.39e-42 | 2.50e-41 |
| ENSMUSG00000032020 | Ubash3b | protein\_coding | 9:41011098-41161697 (-) |  | -1.0800 | 2.44e-42 | 2.55e-41 |
| ENSMUSG00000052534 | Pbx1 | protein\_coding | 1:168119364-168432270 (-) |  | -1.8600 | 2.52e-42 | 2.62e-41 |
| ENSMUSG00000024034 | Tmprss3 | protein\_coding | 17:31179265-31198977 (-) |  | 3.7000 | 2.58e-42 | 2.68e-41 |
| ENSMUSG00000090602 | Gm5611 | processed\_pseudogene | 9:17030045-17030896 (+) |  | 1.6300 | 2.58e-42 | 2.69e-41 |
| ENSMUSG00000039963 | Ccdc40 | protein\_coding | 11:119228572-119265238 (+) |  | 3.5700 | 2.67e-42 | 2.78e-41 |
| ENSMUSG00000028672 | Hmgcl | protein\_coding | 4:135946448-135962617 (+) |  | -0.6750 | 2.71e-42 | 2.82e-41 |
| ENSMUSG00000031488 | Rab11fip1 | protein\_coding | 8:27138773-27174646 (-) |  | -1.0400 | 2.73e-42 | 2.84e-41 |
| ENSMUSG00000030717 | Nupr1 | protein\_coding | 7:126623249-126630861 (-) |  | -1.9200 | 2.80e-42 | 2.90e-41 |
| ENSMUSG00000032300 | 1700017B05Rik | protein\_coding | 9:57253117-57262612 (-) |  | 1.1900 | 3.01e-42 | 3.12e-41 |
| ENSMUSG00000046808 | Atp10d | polymorphic\_pseudogene | 5:72203329-72298775 (+) |  | -1.1500 | 3.03e-42 | 3.14e-41 |
| ENSMUSG00000024679 | Ms4a6d | protein\_coding | 19:11586604-11604849 (-) |  | -1.7700 | 3.09e-42 | 3.20e-41 |
| ENSMUSG00000053318 | Slamf8 | protein\_coding | 1:172581758-172590568 (-) |  | -2.3300 | 3.16e-42 | 3.27e-41 |
| ENSMUSG00000016487 | Ppfibp1 | protein\_coding | 6:146888487-147032025 (+) |  | 1.0100 | 3.25e-42 | 3.36e-41 |
| ENSMUSG00000060131 | Atp8b4 | protein\_coding | 2:126320973-126500674 (-) |  | 0.5200 | 3.68e-42 | 3.80e-41 |
| ENSMUSG00000071203 | Naip5 | protein\_coding | 13:100211739-100246323 (-) |  | -1.0800 | 3.82e-42 | 3.95e-41 |
| ENSMUSG00000079477 | Rab7 | protein\_coding | 6:87999106-88045270 (-) |  | -0.5750 | 3.82e-42 | 3.95e-41 |
| ENSMUSG00000066406 | Akap13 | protein\_coding | 7:75455534-75754609 (+) |  | -0.9290 | 4.32e-42 | 4.45e-41 |
| ENSMUSG00000070327 | Rnf213 | protein\_coding | 11:119393100-119487418 (+) |  | -1.4300 | 5.10e-42 | 5.26e-41 |
| ENSMUSG00000079293 | Clec7a | polymorphic\_pseudogene | 6:129461591-129472779 (-) |  | -1.4200 | 5.11e-42 | 5.26e-41 |
| ENSMUSG00000029802 | Abcg2 | protein\_coding | 6:58584523-58695676 (+) |  | 1.4600 | 5.27e-42 | 5.42e-41 |
| ENSMUSG00000016921 | Srsf6 | protein\_coding | 2:162931528-162937121 (+) |  | 0.5560 | 5.75e-42 | 5.91e-41 |
| ENSMUSG00000024845 | Tmem134 | protein\_coding | 19:4125934-4132307 (+) |  | -0.6690 | 6.16e-42 | 6.33e-41 |
| ENSMUSG00000038623 | Tm6sf1 | protein\_coding | 7:81859001-81884434 (+) |  | -0.7970 | 6.24e-42 | 6.41e-41 |
| ENSMUSG00000014164 | Klhl3 | protein\_coding | 13:58000228-58113592 (-) |  | 4.5900 | 6.24e-42 | 6.41e-41 |
| ENSMUSG00000032584 | Mst1r | protein\_coding | 9:107906873-107920383 (+) |  | -1.8200 | 6.38e-42 | 6.55e-41 |
| ENSMUSG00000026917 | Wdr5 | protein\_coding | 2:27515157-27536535 (+) |  | 0.6700 | 6.52e-42 | 6.69e-41 |
| ENSMUSG00000005873 | Reep5 | protein\_coding | 18:34344885-34374070 (-) |  | -0.6080 | 6.54e-42 | 6.70e-41 |
| ENSMUSG00000034285 | Nipsnap1 | protein\_coding | 11:4873951-4894200 (+) |  | 1.4200 | 6.74e-42 | 6.90e-41 |
| ENSMUSG00000019969 | Psen1 | protein\_coding | 12:83688152-83735199 (+) |  | -0.5100 | 7.32e-42 | 7.48e-41 |
| ENSMUSG00000029050 | Ski | protein\_coding | 4:155154075-155222592 (-) |  | -0.7880 | 7.33e-42 | 7.49e-41 |
| ENSMUSG00000038236 | Hoxa7 | protein\_coding | 6:52214491-52221854 (-) |  | 4.0000 | 7.59e-42 | 7.75e-41 |
| ENSMUSG00000078652 | Psme3 | protein\_coding | 11:101316213-101323537 (+) |  | 0.6960 | 7.81e-42 | 7.97e-41 |
| ENSMUSG00000041747 | Utp15 | protein\_coding | 13:98246845-98263041 (-) |  | 0.7900 | 7.94e-42 | 8.10e-41 |
| ENSMUSG00000032596 | Uba7 | protein\_coding | 9:107975505-107984060 (+) |  | -1.0500 | 8.65e-42 | 8.82e-41 |
| ENSMUSG00000079017 | Ifi27l2a | protein\_coding | 12:103442167-103443680 (-) |  | -3.1400 | 9.56e-42 | 9.74e-41 |
| ENSMUSG00000034848 | Ttc21b | protein\_coding | 2:66184327-66256617 (-) |  | 1.7700 | 9.87e-42 | 1.00e-40 |
| ENSMUSG00000032456 | Nmnat3 | protein\_coding | 9:98287435-98420438 (+) |  | 1.7200 | 1.06e-41 | 1.08e-40 |
| ENSMUSG00000024235 | Map3k8 | protein\_coding | 18:4331327-4353015 (-) |  | -1.4100 | 1.13e-41 | 1.15e-40 |
| ENSMUSG00000032754 | Slc8b1 | protein\_coding | 5:120511168-120534024 (+) |  | -0.6210 | 1.14e-41 | 1.16e-40 |
| ENSMUSG00000030339 | Ltbr | protein\_coding | 6:125306571-125313885 (-) |  | -0.5560 | 1.15e-41 | 1.17e-40 |
| ENSMUSG00000044167 | Foxo1 | protein\_coding | 3:52268336-52353221 (+) |  | -1.1300 | 1.37e-41 | 1.39e-40 |
| ENSMUSG00000000386 | Mx1 | polymorphic\_pseudogene | 16:97447035-97462907 (-) |  | -1.6700 | 1.41e-41 | 1.43e-40 |
| ENSMUSG00000003355 | Fkbp11 | protein\_coding | 15:98724366-98728198 (-) |  | 4.6800 | 1.48e-41 | 1.50e-40 |
| ENSMUSG00000000278 | Scpep1 | protein\_coding | 11:88924020-88955465 (-) |  | -0.7340 | 1.53e-41 | 1.55e-40 |
| ENSMUSG00000023088 | Abcc1 | protein\_coding | 16:14361558-14475737 (+) |  | 0.8600 | 1.57e-41 | 1.59e-40 |
| ENSMUSG00000020134 | Peli1 | protein\_coding | 11:21091291-21150323 (+) |  | -0.9540 | 1.60e-41 | 1.62e-40 |
| ENSMUSG00000031995 | St14 | protein\_coding | 9:31089402-31131853 (-) |  | 1.4200 | 1.65e-41 | 1.67e-40 |
| ENSMUSG00000034595 | Ppp1r18 | protein\_coding | 17:35865593-35875596 (+) |  | -0.6210 | 1.67e-41 | 1.69e-40 |
| ENSMUSG00000062937 | Mtap | protein\_coding | 4:89137122-89181081 (+) |  | 0.8770 | 1.72e-41 | 1.73e-40 |
| ENSMUSG00000004356 | Utp20 | protein\_coding | 10:88746607-88826804 (-) |  | 1.3600 | 1.77e-41 | 1.79e-40 |
| ENSMUSG00000078945 | Naip2 | protein\_coding | 13:100144063-100202092 (-) |  | -0.8570 | 1.87e-41 | 1.88e-40 |
| ENSMUSG00000034616 | Ssh3 | protein\_coding | 19:4261668-4269180 (-) |  | -1.0000 | 1.96e-41 | 1.97e-40 |
| ENSMUSG00000087196 | Gm13373 | lncRNA | 2:28315016-28324050 (-) |  | -3.1900 | 2.08e-41 | 2.10e-40 |
| ENSMUSG00000024621 | Csf1r | protein\_coding | 18:61100598-61132149 (+) |  | -0.7570 | 2.39e-41 | 2.40e-40 |
| ENSMUSG00000020300 | Cpeb4 | protein\_coding | 11:31872211-31935634 (+) |  | -0.9900 | 2.59e-41 | 2.60e-40 |
| ENSMUSG00000021147 | Wdr37 | protein\_coding | 13:8802968-8871909 (-) |  | -0.6410 | 2.60e-41 | 2.61e-40 |
| ENSMUSG00000015947 | Fcgr1 | protein\_coding | 3:96282909-96293969 (-) |  | -2.5900 | 2.75e-41 | 2.76e-40 |
| ENSMUSG00000020483 | Dynll2 | protein\_coding | 11:87979525-87987533 (-) |  | 0.9060 | 3.21e-41 | 3.22e-40 |
| ENSMUSG00000041633 | Kctd12b | protein\_coding | X:153685154-153696391 (-) |  | -1.6200 | 3.51e-41 | 3.52e-40 |
| ENSMUSG00000027420 | Bfsp1 | protein\_coding | 2:143826528-143863173 (-) |  | -2.7200 | 3.59e-41 | 3.59e-40 |
| ENSMUSG00000112148 | Lilrb4a | protein\_coding | 10:51490956-51496611 (+) |  | -2.5000 | 3.71e-41 | 3.71e-40 |
| ENSMUSG00000041642 | Kif21b | protein\_coding | 1:136131389-136177998 (+) |  | -1.1200 | 3.81e-41 | 3.81e-40 |
| ENSMUSG00000034758 | Tle6 | protein\_coding | 10:81590904-81601073 (-) |  | 2.9600 | 4.04e-41 | 4.04e-40 |
| ENSMUSG00000039109 | F13a1 | protein\_coding | 13:36867178-37050244 (-) |  | -0.7440 | 4.10e-41 | 4.10e-40 |
| ENSMUSG00000091955 | Gm9844 | protein\_coding | 7:24862213-24862697 (+) |  | -1.2300 | 4.33e-41 | 4.32e-40 |
| ENSMUSG00000003438 | Timm50 | protein\_coding | 7:28305516-28312072 (-) |  | 0.7380 | 4.55e-41 | 4.54e-40 |
| ENSMUSG00000028745 | Capzb | protein\_coding | 4:139192899-139291818 (+) |  | -0.6020 | 4.64e-41 | 4.62e-40 |
| ENSMUSG00000028683 | Eif2b3 | protein\_coding | 4:117019402-117087306 (+) |  | 1.1300 | 4.87e-41 | 4.85e-40 |
| ENSMUSG00000049422 | Chchd10 | protein\_coding | 10:75933130-75937747 (+) |  | 2.1900 | 5.04e-41 | 5.02e-40 |
| ENSMUSG00000002820 | Atg4d | protein\_coding | 9:21265293-21277772 (+) |  | -0.8160 | 5.12e-41 | 5.09e-40 |
| ENSMUSG00000022263 | Trio | protein\_coding | 15:27730651-28025848 (-) |  | -1.0200 | 5.93e-41 | 5.90e-40 |
| ENSMUSG00000078853 | Igtp | protein\_coding | 11:58199556-58207591 (+) |  | -1.3700 | 5.96e-41 | 5.92e-40 |
| ENSMUSG00000039899 | Fgl2 | protein\_coding | 5:21372642-21378374 (+) |  | -0.9920 | 6.45e-41 | 6.41e-40 |
| ENSMUSG00000041313 | Slc7a1 | protein\_coding | 5:148327410-148399904 (-) |  | 1.0800 | 6.84e-41 | 6.79e-40 |
| ENSMUSG00000059363 | Fxn | protein\_coding | 19:24261453-24280605 (-) |  | 1.3100 | 7.59e-41 | 7.52e-40 |
| ENSMUSG00000058655 | Eif4b | protein\_coding | 15:102073773-102097173 (+) |  | 0.5440 | 8.17e-41 | 8.09e-40 |
| ENSMUSG00000031377 | Bmx | protein\_coding | X:164192842-164258193 (-) |  | -0.9730 | 9.34e-41 | 9.26e-40 |
| ENSMUSG00000039206 | Daglb | protein\_coding | 5:143464584-143505942 (+) |  | -0.7140 | 1.07e-40 | 1.06e-39 |
| ENSMUSG00000030871 | Ears2 | protein\_coding | 7:122037213-122067263 (-) |  | 1.5900 | 1.07e-40 | 1.06e-39 |
| ENSMUSG00000018567 | Gabarap | protein\_coding | 11:69991143-69994951 (+) |  | -0.8500 | 1.18e-40 | 1.16e-39 |
| ENSMUSG00000027374 | Mrps5 | protein\_coding | 2:127587222-127606829 (+) |  | 0.9830 | 1.19e-40 | 1.17e-39 |
| ENSMUSG00000029461 | Fam168a | protein\_coding | 7:100706635-100841656 (+) |  | -0.7760 | 1.27e-40 | 1.25e-39 |
| ENSMUSG00000028651 | Ppie | protein\_coding | 4:123127115-123139951 (-) |  | 0.9430 | 1.27e-40 | 1.25e-39 |
| ENSMUSG00000026878 | Rab14 | protein\_coding | 2:35180205-35201120 (-) |  | -0.4750 | 1.28e-40 | 1.26e-39 |
| ENSMUSG00000056917 | Sipa1 | protein\_coding | 19:5651185-5663707 (-) |  | -0.6570 | 1.31e-40 | 1.30e-39 |
| ENSMUSG00000026458 | Ppfia4 | protein\_coding | 1:134296783-134332928 (-) |  | -1.0800 | 1.33e-40 | 1.31e-39 |
| ENSMUSG00000025780 | Itih5 | protein\_coding | 2:10153571-10256529 (+) |  | 5.3200 | 1.44e-40 | 1.42e-39 |
| ENSMUSG00000002458 | Rgs19 | protein\_coding | 2:181688419-181693977 (-) |  | -0.4870 | 1.59e-40 | 1.57e-39 |
| ENSMUSG00000017652 | Cd40 | protein\_coding | 2:165055627-165072948 (+) |  | -1.9300 | 1.62e-40 | 1.59e-39 |
| ENSMUSG00000027323 | Rad51 | protein\_coding | 2:119112793-119147445 (+) |  | 1.1400 | 1.63e-40 | 1.60e-39 |
| ENSMUSG00000028811 | Yars | protein\_coding | 4:129189760-129219607 (+) |  | 0.8670 | 1.80e-40 | 1.77e-39 |
| ENSMUSG00000066952 | Myo1h | protein\_coding | 5:114289166-114365357 (+) |  | -2.3900 | 1.90e-40 | 1.87e-39 |
| ENSMUSG00000059796 | Eif4a1 | protein\_coding | 11:69666936-69672423 (-) |  | 0.7030 | 2.05e-40 | 2.01e-39 |
| ENSMUSG00000033233 | Trim45 | protein\_coding | 3:100922202-100936920 (+) |  | 1.1600 | 2.07e-40 | 2.03e-39 |
| ENSMUSG00000074519 | Zfp971 | protein\_coding | 2:178023284-178034022 (+) |  | -1.6800 | 2.19e-40 | 2.14e-39 |
| ENSMUSG00000046897 | Zfp740 | protein\_coding | 15:102203249-102215606 (+) |  | -0.5780 | 2.59e-40 | 2.53e-39 |
| ENSMUSG00000034300 | Fam53c | protein\_coding | 18:34758906-34773760 (+) |  | -0.6990 | 2.68e-40 | 2.62e-39 |
| ENSMUSG00000001440 | Kpnb1 | protein\_coding | 11:97159714-97187881 (-) |  | 0.7430 | 2.69e-40 | 2.63e-39 |
| ENSMUSG00000027322 | Siglec1 | protein\_coding | 2:131069220-131086765 (-) |  | -4.5400 | 2.69e-40 | 2.63e-39 |
| ENSMUSG00000057469 | E2f6 | protein\_coding | 12:16810963-16839742 (+) |  | 0.6040 | 2.83e-40 | 2.76e-39 |
| ENSMUSG00000025034 | Trim8 | protein\_coding | 19:46501702-46516848 (+) |  | -0.6940 | 2.88e-40 | 2.81e-39 |
| ENSMUSG00000036138 | Acaa1a | protein\_coding | 9:119339676-119350299 (+) |  | -0.7670 | 3.05e-40 | 2.98e-39 |
| ENSMUSG00000022858 | Tra2b | protein\_coding | 16:22244549-22266005 (-) |  | 0.5400 | 3.19e-40 | 3.10e-39 |
| ENSMUSG00000002058 | Unc119 | protein\_coding | 11:78343482-78349164 (+) |  | -1.3900 | 3.33e-40 | 3.24e-39 |
| ENSMUSG00000070354 | Evi2 | protein\_coding | 11:79513385-79530589 (-) |  | -1.3100 | 3.37e-40 | 3.27e-39 |
| ENSMUSG00000040010 | Slc7a5 | protein\_coding | 8:121881150-121907694 (-) |  | 1.6000 | 3.46e-40 | 3.36e-39 |
| ENSMUSG00000056234 | Ncoa4 | protein\_coding | 14:32159865-32179855 (+) |  | 0.8490 | 3.60e-40 | 3.49e-39 |
| ENSMUSG00000066148 | Prpf4 | protein\_coding | 4:62408797-62426990 (+) |  | 0.6710 | 3.66e-40 | 3.55e-39 |
| ENSMUSG00000023572 | Ccndbp1 | protein\_coding | 2:121008403-121016904 (+) |  | -0.8400 | 4.24e-40 | 4.11e-39 |
| ENSMUSG00000041891 | Lman1 | protein\_coding | 18:65980738-66022580 (-) |  | 0.7390 | 4.41e-40 | 4.28e-39 |
| ENSMUSG00000030257 | Srgap3 | protein\_coding | 6:112717971-112947266 (-) |  | 1.8200 | 4.63e-40 | 4.48e-39 |
| ENSMUSG00000033355 | Rtp4 | protein\_coding | 16:23520291-23614222 (+) |  | -4.6200 | 4.88e-40 | 4.73e-39 |
| ENSMUSG00000027765 | P2ry1 | protein\_coding | 3:61002795-61008982 (+) |  | -1.1900 | 4.94e-40 | 4.78e-39 |
| ENSMUSG00000026536 | Ifi211 | protein\_coding | 1:173896345-173913046 (-) |  | -1.5200 | 5.25e-40 | 5.07e-39 |
| ENSMUSG00000022571 | Pycrl | protein\_coding | 15:75916477-75921560 (-) |  | 1.0700 | 5.35e-40 | 5.17e-39 |
| ENSMUSG00000017548 | Suz12 | protein\_coding | 11:79993106-80034123 (+) |  | 0.6390 | 6.79e-40 | 6.55e-39 |
| ENSMUSG00000000958 | Slc7a7 | protein\_coding | 14:54369442-54417780 (-) |  | -1.1500 | 6.86e-40 | 6.62e-39 |
| ENSMUSG00000066551 | Hmgb1 | protein\_coding | 5:149046702-149184489 (-) |  | 0.5780 | 6.87e-40 | 6.62e-39 |
| ENSMUSG00000026377 | Nifk | protein\_coding | 1:118321839-118333822 (+) |  | 0.9770 | 7.21e-40 | 6.95e-39 |
| ENSMUSG00000061175 | Fnip2 | protein\_coding | 3:79455974-79567796 (-) |  | -1.2500 | 7.23e-40 | 6.96e-39 |
| ENSMUSG00000014074 | Rnf168 | protein\_coding | 16:32277459-32301434 (+) |  | 0.7380 | 7.80e-40 | 7.51e-39 |
| ENSMUSG00000028944 | Prkag2 | protein\_coding | 5:24862744-25100642 (-) |  | -0.6530 | 7.91e-40 | 7.61e-39 |
| ENSMUSG00000025473 | Adam8 | protein\_coding | 7:139978932-139992562 (-) |  | -2.2300 | 8.00e-40 | 7.69e-39 |
| ENSMUSG00000025503 | Taldo1 | protein\_coding | 7:141392199-141402968 (+) |  | -0.7070 | 8.18e-40 | 7.85e-39 |
| ENSMUSG00000027900 | Dram2 | protein\_coding | 3:106547798-106575890 (+) |  | -1.3200 | 8.18e-40 | 7.85e-39 |
| ENSMUSG00000039033 | Tasp1 | protein\_coding | 2:139833480-140066805 (-) |  | 1.2500 | 8.44e-40 | 8.10e-39 |
| ENSMUSG00000030122 | Ptms | protein\_coding | 6:124913681-124920103 (-) |  | -1.1800 | 8.97e-40 | 8.60e-39 |
| ENSMUSG00000040455 | Usp45 | protein\_coding | 4:21767156-21837872 (+) |  | -0.8610 | 1.03e-39 | 9.85e-39 |
| ENSMUSG00000020919 | Stat5b | protein\_coding | 11:100780731-100850724 (-) |  | -0.5280 | 1.04e-39 | 9.95e-39 |
| ENSMUSG00000034613 | Ppm1h | protein\_coding | 10:122678762-122945795 (+) |  | -0.6510 | 1.07e-39 | 1.03e-38 |
| ENSMUSG00000006127 | Inpp5k | protein\_coding | 11:75630988-75648871 (+) |  | -0.7680 | 1.08e-39 | 1.03e-38 |
| ENSMUSG00000020780 | Srp68 | protein\_coding | 11:116245166-116274217 (-) |  | 0.7270 | 1.08e-39 | 1.04e-38 |
| ENSMUSG00000039159 | Ube2h | protein\_coding | 6:30211289-30304539 (-) |  | -1.0300 | 1.10e-39 | 1.06e-38 |
| ENSMUSG00000032312 | Csk | protein\_coding | 9:57626646-57653631 (-) |  | -0.5860 | 1.13e-39 | 1.07e-38 |
| ENSMUSG00000027776 | Il12a | protein\_coding | 3:68690644-68698547 (+) |  | 5.9800 | 1.18e-39 | 1.13e-38 |
| ENSMUSG00000019852 | Arfgef3 | protein\_coding | 10:18581839-18743949 (-) |  | 2.3300 | 1.20e-39 | 1.14e-38 |
| ENSMUSG00000028527 | Ak4 | protein\_coding | 4:101419277-101466995 (+) |  | 5.7400 | 1.26e-39 | 1.20e-38 |
| ENSMUSG00000008373 | Prpf31 | protein\_coding | 7:3629985-3642486 (+) |  | 0.7260 | 1.31e-39 | 1.25e-38 |
| ENSMUSG00000030187 | Klra2 | protein\_coding | 6:131219223-131247362 (-) |  | -3.4200 | 1.37e-39 | 1.30e-38 |
| ENSMUSG00000020190 | Mknk2 | protein\_coding | 10:80665327-80678112 (-) |  | -0.5660 | 1.40e-39 | 1.33e-38 |
| ENSMUSG00000041859 | Mcm3 | protein\_coding | 1:20802968-20820312 (-) |  | 1.1200 | 1.49e-39 | 1.41e-38 |
| ENSMUSG00000023010 | Tmbim6 | protein\_coding | 15:99392882-99410049 (+) |  | -0.5010 | 1.56e-39 | 1.48e-38 |
| ENSMUSG00000034201 | Gas2l1 | protein\_coding | 11:5054132-5065327 (-) |  | -1.8900 | 1.56e-39 | 1.48e-38 |
| ENSMUSG00000039294 | Cybc1 | protein\_coding | 11:121222588-121229322 (-) |  | -0.6320 | 1.59e-39 | 1.51e-38 |
| ENSMUSG00000001739 | Cldn15 | protein\_coding | 5:136966616-136975858 (+) |  | 1.1500 | 1.59e-39 | 1.51e-38 |
| ENSMUSG00000023341 | Mx2 | polymorphic\_pseudogene | 16:97535308-97560900 (+) |  | -2.0300 | 1.63e-39 | 1.54e-38 |
| ENSMUSG00000000605 | Clcn4 | protein\_coding | 7:7282309-7300851 (-) |  | -0.5510 | 1.73e-39 | 1.64e-38 |
| ENSMUSG00000021665 | Hexb | protein\_coding | 13:97176331-97198357 (-) |  | -1.0400 | 1.81e-39 | 1.71e-38 |
| ENSMUSG00000089942 | Pira2 | protein\_coding | 7:3836812-3845051 (-) |  | -1.9000 | 1.83e-39 | 1.73e-38 |
| ENSMUSG00000030254 | Rad18 | protein\_coding | 6:112619850-112696686 (-) |  | 1.0900 | 1.89e-39 | 1.79e-38 |
| ENSMUSG00000029504 | Ddx51 | protein\_coding | 5:110653451-110660496 (+) |  | 0.8400 | 1.91e-39 | 1.80e-38 |
| ENSMUSG00000028792 | Ak2 | protein\_coding | 4:128991958-129011529 (+) |  | 0.7210 | 1.93e-39 | 1.82e-38 |
| ENSMUSG00000052533 | Nup188 | protein\_coding | 2:30286397-30344266 (+) |  | 0.8310 | 1.93e-39 | 1.82e-38 |
| ENSMUSG00000022365 | Derl1 | protein\_coding | 15:57869502-57892448 (-) |  | -0.4300 | 1.94e-39 | 1.83e-38 |
| ENSMUSG00000028552 | Eps15 | protein\_coding | 4:109280268-109387817 (+) |  | -0.7090 | 2.00e-39 | 1.88e-38 |
| ENSMUSG00000085396 | Firre | lncRNA | X:50555744-50635321 (-) |  | 1.6100 | 2.03e-39 | 1.91e-38 |
| ENSMUSG00000009292 | Trpm2 | protein\_coding | 10:77907722-77970563 (-) |  | -1.5000 | 2.06e-39 | 1.94e-38 |
| ENSMUSG00000023800 | Tiam2 | protein\_coding | 17:3326573-3531344 (+) |  | -0.8410 | 2.21e-39 | 2.07e-38 |
| ENSMUSG00000021262 | Evl | protein\_coding | 12:108554720-108688516 (+) |  | -0.8860 | 2.24e-39 | 2.10e-38 |
| ENSMUSG00000057335 | Cep170 | protein\_coding | 1:176733653-176814067 (-) |  | -0.8360 | 2.28e-39 | 2.14e-38 |
| ENSMUSG00000042724 | Map3k9 | protein\_coding | 12:81721010-81781175 (-) |  | -1.4600 | 2.31e-39 | 2.17e-38 |
| ENSMUSG00000079057 | Cyp4v3 | protein\_coding | 8:45304944-45333216 (-) |  | -1.2000 | 2.36e-39 | 2.21e-38 |
| ENSMUSG00000019373 | Cops3 | protein\_coding | 11:59817795-59839838 (-) |  | 0.8580 | 2.39e-39 | 2.24e-38 |
| ENSMUSG00000018736 | Ndel1 | protein\_coding | 11:68821434-68871858 (-) |  | -0.6980 | 2.46e-39 | 2.30e-38 |
| ENSMUSG00000020585 | Laptm4a | protein\_coding | 12:8921664-8938742 (+) |  | -0.9150 | 2.48e-39 | 2.32e-38 |
| ENSMUSG00000056209 | Npm3 | protein\_coding | 19:45747734-45749591 (-) |  | 1.0500 | 2.75e-39 | 2.57e-38 |
| ENSMUSG00000062545 | Tlr12 | protein\_coding | 4:128615443-128618619 (-) |  | 3.7000 | 2.92e-39 | 2.73e-38 |
| ENSMUSG00000033467 | Crlf2 | protein\_coding | 5:109554709-109558993 (-) |  | -0.8270 | 3.35e-39 | 3.13e-38 |
| ENSMUSG00000051864 | Tbc1d22a | protein\_coding | 15:86214459-86498503 (+) |  | -0.5800 | 3.36e-39 | 3.14e-38 |
| ENSMUSG00000021023 | Prorp | protein\_coding | 12:55299577-55382533 (+) |  | 1.3500 | 3.58e-39 | 3.34e-38 |
| ENSMUSG00000038370 | Pcp4l1 | protein\_coding | 1:171173262-171196268 (-) |  | 3.0800 | 3.65e-39 | 3.40e-38 |
| ENSMUSG00000036106 | Prr5 | protein\_coding | 15:84669620-84703673 (+) |  | 1.4200 | 4.27e-39 | 3.98e-38 |
| ENSMUSG00000032737 | Inppl1 | protein\_coding | 7:101822632-101838229 (-) |  | -1.6300 | 4.67e-39 | 4.35e-38 |
| ENSMUSG00000039682 | Lap3 | protein\_coding | 5:45493374-45512691 (+) |  | 1.0200 | 4.70e-39 | 4.37e-38 |
| ENSMUSG00000029213 | Commd8 | protein\_coding | 5:72156575-72168189 (-) |  | -0.8540 | 4.92e-39 | 4.57e-38 |
| ENSMUSG00000028613 | Lrp8 | protein\_coding | 4:107801869-107876840 (+) |  | 1.3200 | 4.94e-39 | 4.60e-38 |
| ENSMUSG00000028465 | Tln1 | protein\_coding | 4:43531519-43562691 (-) |  | -0.6880 | 5.73e-39 | 5.33e-38 |
| ENSMUSG00000027808 | Serp1 | protein\_coding | 3:58519817-58525892 (-) |  | -0.8600 | 5.78e-39 | 5.37e-38 |
| ENSMUSG00000040322 | Slc25a24 | protein\_coding | 3:109123149-109168457 (+) |  | -0.9930 | 5.82e-39 | 5.40e-38 |
| ENSMUSG00000001909 | Trmt1 | protein\_coding | 8:84686307-84699808 (+) |  | 0.8340 | 6.11e-39 | 5.67e-38 |
| ENSMUSG00000035165 | Kcne3 | protein\_coding | 7:100176502-100184869 (+) |  | -1.9500 | 6.23e-39 | 5.77e-38 |
| ENSMUSG00000021880 | Rnase6 | protein\_coding | 14:51123908-51132187 (+) |  | -1.6600 | 6.26e-39 | 5.80e-38 |
| ENSMUSG00000029410 | Ppef2 | protein\_coding | 5:92226679-92256278 (-) |  | -2.7500 | 6.41e-39 | 5.93e-38 |
| ENSMUSG00000025950 | Idh1 | protein\_coding | 1:65158616-65186500 (-) |  | -0.9910 | 7.09e-39 | 6.56e-38 |
| ENSMUSG00000034902 | Pip5k1c | protein\_coding | 10:81292963-81319973 (+) |  | -0.5570 | 7.14e-39 | 6.60e-38 |
| ENSMUSG00000027465 | Tbc1d20 | protein\_coding | 2:152293828-152313996 (+) |  | -0.6220 | 7.22e-39 | 6.67e-38 |
| ENSMUSG00000058230 | Arhgap35 | protein\_coding | 7:16493719-16614993 (-) |  | 1.0200 | 7.72e-39 | 7.13e-38 |
| ENSMUSG00000028826 | Maco1 | protein\_coding | 4:134802759-134853345 (-) |  | 0.7630 | 7.77e-39 | 7.17e-38 |
| ENSMUSG00000020413 | Hus1 | protein\_coding | 11:8993137-9011191 (-) |  | 0.7900 | 7.90e-39 | 7.29e-38 |
| ENSMUSG00000041390 | Mdfic | protein\_coding | 6:15720661-15802169 (+) |  | -0.7950 | 9.00e-39 | 8.30e-38 |
| ENSMUSG00000028933 | Xrcc2 | protein\_coding | 5:25689812-25705825 (-) |  | 0.9400 | 9.55e-39 | 8.80e-38 |
| ENSMUSG00000049076 | Acap2 | protein\_coding | 16:31092412-31201245 (-) |  | -0.7510 | 1.05e-38 | 9.64e-38 |
| ENSMUSG00000026037 | Orc2 | protein\_coding | 1:58462771-58505109 (-) |  | 0.8710 | 1.05e-38 | 9.65e-38 |
| ENSMUSG00000024357 | Sil1 | protein\_coding | 18:35266396-35499780 (-) |  | 0.9010 | 1.05e-38 | 9.65e-38 |
| ENSMUSG00000097392 | Thoc2l | protein\_coding | 5:104508352-104554207 (+) |  | 1.0600 | 1.07e-38 | 9.80e-38 |
| ENSMUSG00000030707 | Coro1a | protein\_coding | 7:126699773-126707787 (-) |  | -0.8370 | 1.08e-38 | 9.91e-38 |
| ENSMUSG00000020430 | Pes1 | protein\_coding | 11:3963975-3980004 (+) |  | 0.7390 | 1.10e-38 | 1.00e-37 |
| ENSMUSG00000030232 | Aebp2 | protein\_coding | 6:140622663-140678472 (+) |  | 0.4450 | 1.10e-38 | 1.01e-37 |
| ENSMUSG00000021895 | Arhgef3 | protein\_coding | 14:27114899-27403911 (+) |  | -1.4100 | 1.18e-38 | 1.08e-37 |
| ENSMUSG00000039005 | Tlr4 | protein\_coding | 4:66827584-66930284 (+) |  | -1.3000 | 1.27e-38 | 1.16e-37 |
| ENSMUSG00000033752 | Mnd1 | protein\_coding | 3:84087933-84155786 (-) |  | 1.9900 | 1.38e-38 | 1.26e-37 |
| ENSMUSG00000028664 | Ephb2 | protein\_coding | 4:136647539-136835988 (-) |  | 5.4100 | 1.43e-38 | 1.31e-37 |
| ENSMUSG00000066037 | Hnrnpr | protein\_coding | 4:136310942-136359447 (+) |  | 0.4870 | 1.52e-38 | 1.39e-37 |
| ENSMUSG00000074896 | Ifit3 | protein\_coding | 19:34583531-34588731 (+) |  | -4.3400 | 1.67e-38 | 1.52e-37 |
| ENSMUSG00000020114 | Cand1 | protein\_coding | 10:119199255-119240055 (-) |  | 0.7400 | 1.71e-38 | 1.56e-37 |
| ENSMUSG00000069830 | Nlrp1a | protein\_coding | 11:71092236-71144704 (-) |  | -1.0300 | 1.80e-38 | 1.65e-37 |
| ENSMUSG00000032776 | Mctp2 | protein\_coding | 7:72077830-72306608 (-) |  | -1.9100 | 1.86e-38 | 1.70e-37 |
| ENSMUSG00000032481 | Smarcc1 | protein\_coding | 9:110117708-110240178 (+) |  | 0.7610 | 1.95e-38 | 1.77e-37 |
| ENSMUSG00000023951 | Vegfa | protein\_coding | 17:46016993-46032369 (-) |  | 4.0700 | 2.01e-38 | 1.84e-37 |
| ENSMUSG00000097908 | 4933404O12Rik | lncRNA | 5:136919146-136937112 (+) |  | 1.3000 | 2.13e-38 | 1.94e-37 |
| ENSMUSG00000022945 | Chaf1b | protein\_coding | 16:93883901-93906115 (+) |  | 1.0500 | 2.19e-38 | 1.99e-37 |
| ENSMUSG00000024725 | Ostf1 | protein\_coding | 19:18516137-18631823 (-) |  | -0.8140 | 2.22e-38 | 2.02e-37 |
| ENSMUSG00000028614 | Ndc1 | protein\_coding | 4:107367784-107416346 (+) |  | 0.7710 | 2.55e-38 | 2.32e-37 |
| ENSMUSG00000039987 | Phtf2 | protein\_coding | 5:20758663-20882124 (-) |  | 0.8810 | 2.76e-38 | 2.51e-37 |
| ENSMUSG00000089832 | Shkbp1 | protein\_coding | 7:27342133-27356019 (-) |  | -0.8110 | 2.95e-38 | 2.67e-37 |
| ENSMUSG00000028953 | Abcf2 | protein\_coding | 5:24565345-24577467 (-) |  | 0.6510 | 3.15e-38 | 2.86e-37 |
| ENSMUSG00000039660 | Spout1 | protein\_coding | 2:30173453-30178459 (-) |  | 1.0200 | 3.25e-38 | 2.94e-37 |
| ENSMUSG00000058835 | Abi1 | protein\_coding | 2:22940073-23040241 (-) |  | -0.7450 | 3.58e-38 | 3.24e-37 |
| ENSMUSG00000068922 | Msto1 | protein\_coding | 3:88905107-88913999 (-) |  | 0.9690 | 3.59e-38 | 3.25e-37 |
| ENSMUSG00000031165 | Was | protein\_coding | X:8081453-8090498 (-) |  | -0.4280 | 3.84e-38 | 3.47e-37 |
| ENSMUSG00000005533 | Igf1r | protein\_coding | 7:67952827-68233668 (+) |  | 1.1100 | 3.86e-38 | 3.49e-37 |
| ENSMUSG00000041995 | Zbed3 | protein\_coding | 13:95323612-95337841 (+) |  | 1.1600 | 3.98e-38 | 3.59e-37 |
| ENSMUSG00000111535 | Gm35154 | lncRNA | 10:44598720-44689081 (+) |  | -3.4100 | 4.08e-38 | 3.69e-37 |
| ENSMUSG00000001918 | Slc1a5 | protein\_coding | 7:16781340-16798274 (+) |  | 0.6620 | 4.17e-38 | 3.77e-37 |
| ENSMUSG00000031555 | Adam9 | protein\_coding | 8:24949611-25016927 (-) |  | -0.9900 | 4.38e-38 | 3.95e-37 |
| ENSMUSG00000025860 | Xiap | protein\_coding | X:42059679-42109656 (+) |  | -0.5800 | 4.56e-38 | 4.11e-37 |
| ENSMUSG00000001785 | Pwp1 | protein\_coding | 10:85829494-85889096 (+) |  | 0.7640 | 4.83e-38 | 4.36e-37 |
| ENSMUSG00000005103 | Wdr1 | protein\_coding | 5:38526813-38563221 (-) |  | -0.4880 | 5.08e-38 | 4.58e-37 |
| ENSMUSG00000029270 | Dipk1a | protein\_coding | 5:107908053-107987085 (-) |  | -0.8160 | 5.36e-38 | 4.82e-37 |
| ENSMUSG00000022408 | Fam83f | protein\_coding | 15:80671847-80700425 (+) |  | -1.9600 | 5.78e-38 | 5.20e-37 |
| ENSMUSG00000071713 | Csf2rb | protein\_coding | 15:78325752-78353847 (+) |  | -0.6540 | 5.84e-38 | 5.25e-37 |
| ENSMUSG00000003161 | Sri | protein\_coding | 5:8046078-8069379 (+) |  | -0.4580 | 5.94e-38 | 5.34e-37 |
| ENSMUSG00000028690 | Mmachc | protein\_coding | 4:116702279-116708406 (-) |  | 1.4300 | 5.97e-38 | 5.36e-37 |
| ENSMUSG00000004665 | Cnn2 | protein\_coding | 10:79988584-79996062 (+) |  | -1.0100 | 6.10e-38 | 5.48e-37 |
| ENSMUSG00000030852 | Tacc2 | protein\_coding | 7:130577438-130764785 (+) |  | 3.9000 | 6.11e-38 | 5.48e-37 |
| ENSMUSG00000066278 | Vps37b | protein\_coding | 5:124004641-124032270 (-) |  | -1.0100 | 6.25e-38 | 5.60e-37 |
| ENSMUSG00000057729 | Prtn3 | protein\_coding | 10:79874476-79883174 (+) |  | 2.4100 | 6.25e-38 | 5.60e-37 |
| ENSMUSG00000033554 | Dph5 | protein\_coding | 3:115887837-115934361 (+) |  | 1.0000 | 6.34e-38 | 5.67e-37 |
| ENSMUSG00000038058 | Nod1 | protein\_coding | 6:54923949-54972612 (-) |  | -0.9760 | 6.80e-38 | 6.08e-37 |
| ENSMUSG00000029319 | Coq2 | protein\_coding | 5:100654723-100675140 (-) |  | 0.7000 | 6.81e-38 | 6.09e-37 |
| ENSMUSG00000038451 | Spsb2 | protein\_coding | 6:124808661-124810619 (+) |  | -1.0500 | 7.32e-38 | 6.54e-37 |
| ENSMUSG00000071379 | Hpcal1 | protein\_coding | 12:17690856-17791933 (+) |  | -0.8580 | 7.63e-38 | 6.81e-37 |
| ENSMUSG00000054823 | Nsd3 | protein\_coding | 8:25601601-25719667 (+) |  | -0.6850 | 7.67e-38 | 6.84e-37 |
| ENSMUSG00000030091 | Nup210 | protein\_coding | 6:91013068-91116829 (-) |  | 1.0800 | 8.65e-38 | 7.71e-37 |
| ENSMUSG00000066232 | Ipo7 | protein\_coding | 7:110018274-110056609 (+) |  | 0.5470 | 8.82e-38 | 7.86e-37 |
| ENSMUSG00000031828 | Klhl36 | protein\_coding | 8:119862266-119876995 (+) |  | -0.8890 | 9.33e-38 | 8.31e-37 |
| ENSMUSG00000037434 | Slc30a1 | protein\_coding | 1:191906767-191913247 (+) |  | -0.8810 | 9.41e-38 | 8.37e-37 |
| ENSMUSG00000043909 | Trp53bp1 | protein\_coding | 2:121193281-121271407 (-) |  | 1.2800 | 1.00e-37 | 8.94e-37 |
| ENSMUSG00000037851 | Iars | protein\_coding | 13:49682100-49734267 (+) |  | 0.9400 | 1.11e-37 | 9.85e-37 |
| ENSMUSG00000047735 | Samd9l | protein\_coding | 6:3372257-3399572 (-) |  | -2.2000 | 1.15e-37 | 1.02e-36 |
| ENSMUSG00000040269 | Mrps28 | protein\_coding | 3:8802146-8923918 (-) |  | 1.0100 | 1.15e-37 | 1.02e-36 |
| ENSMUSG00000023022 | Lima1 | protein\_coding | 15:99778470-99875456 (-) |  | -1.4000 | 1.23e-37 | 1.09e-36 |
| ENSMUSG00000032382 | Snx1 | protein\_coding | 9:66088133-66126587 (-) |  | -0.7460 | 1.33e-37 | 1.18e-36 |
| ENSMUSG00000005583 | Mef2c | protein\_coding | 13:83504034-83667080 (+) |  | 0.8530 | 1.33e-37 | 1.18e-36 |
| ENSMUSG00000025264 | Tsr2 | protein\_coding | X:151087094-151096543 (-) |  | 1.3000 | 1.34e-37 | 1.19e-36 |
| ENSMUSG00000029605 | Oas1b | polymorphic\_pseudogene | 5:120812635-120824163 (+) |  | -3.2300 | 1.35e-37 | 1.19e-36 |
| ENSMUSG00000029468 | P2rx7 | protein\_coding | 5:122643911-122691432 (+) |  | -0.7850 | 1.49e-37 | 1.32e-36 |
| ENSMUSG00000000876 | Pxmp4 | protein\_coding | 2:154585758-154603708 (-) |  | -0.7430 | 1.54e-37 | 1.36e-36 |
| ENSMUSG00000041607 | Mbp | protein\_coding | 18:82475146-82585637 (+) |  | -1.2700 | 1.63e-37 | 1.44e-36 |
| ENSMUSG00000025410 | Dctn2 | protein\_coding | 10:127266368-127281950 (+) |  | -0.4480 | 1.71e-37 | 1.51e-36 |
| ENSMUSG00000040596 | Pogk | protein\_coding | 1:166384622-166409863 (-) |  | 0.9980 | 1.72e-37 | 1.52e-36 |
| ENSMUSG00000030579 | Tyrobp | protein\_coding | 7:30413760-30417585 (+) |  | -1.1400 | 1.78e-37 | 1.57e-36 |
| ENSMUSG00000052298 | Cdc42se2 | protein\_coding | 11:54717456-54787675 (-) |  | -0.5460 | 1.83e-37 | 1.61e-36 |
| ENSMUSG00000029430 | Ran | protein\_coding | 5:129020069-129024323 (+) |  | 0.8860 | 2.02e-37 | 1.78e-36 |
| ENSMUSG00000062098 | Btbd3 | protein\_coding | 2:138256565-138589292 (+) |  | 1.8300 | 2.03e-37 | 1.79e-36 |
| ENSMUSG00000031722 | Hp | protein\_coding | 8:109575128-109579172 (-) |  | -0.9600 | 2.10e-37 | 1.84e-36 |
| ENSMUSG00000036257 | Pnpla8 | protein\_coding | 12:44221370-44322532 (+) |  | -0.6790 | 2.12e-37 | 1.86e-36 |
| ENSMUSG00000035455 | Fignl1 | protein\_coding | 11:11787431-11808962 (-) |  | 0.8300 | 2.15e-37 | 1.89e-36 |
| ENSMUSG00000035342 | Lzts2 | protein\_coding | 19:45014414-45027111 (+) |  | 4.0700 | 2.17e-37 | 1.91e-36 |
| ENSMUSG00000032423 | Syncrip | protein\_coding | 9:88447009-88482574 (-) |  | 0.7010 | 2.24e-37 | 1.96e-36 |
| ENSMUSG00000020120 | Plek | protein\_coding | 11:16971206-17052381 (-) |  | -1.3600 | 2.28e-37 | 2.00e-36 |
| ENSMUSG00000025794 | Rpl14 | protein\_coding | 9:120571444-120574654 (+) |  | 0.7490 | 2.36e-37 | 2.07e-36 |
| ENSMUSG00000041895 | Wipi1 | protein\_coding | 11:109573331-109611967 (-) |  | -1.1400 | 2.56e-37 | 2.25e-36 |
| ENSMUSG00000040712 | Camta2 | protein\_coding | 11:70669463-70688105 (-) |  | -1.0200 | 2.69e-37 | 2.36e-36 |
| ENSMUSG00000066235 | Pomgnt2 | protein\_coding | 9:121981606-121997110 (-) |  | 3.2300 | 2.77e-37 | 2.42e-36 |
| ENSMUSG00000000204 | Slfn4 | protein\_coding | 11:83175186-83190216 (+) |  | -3.1500 | 2.89e-37 | 2.53e-36 |
| ENSMUSG00000002718 | Cse1l | protein\_coding | 2:166906040-166946389 (+) |  | 0.6340 | 3.02e-37 | 2.64e-36 |
| ENSMUSG00000020128 | Vps54 | protein\_coding | 11:21239281-21321136 (+) |  | -0.8280 | 3.43e-37 | 3.00e-36 |
| ENSMUSG00000022550 | Adck5 | protein\_coding | 15:76576358-76595816 (+) |  | 1.3900 | 3.61e-37 | 3.16e-36 |
| ENSMUSG00000023079 | Gtf2ird1 | protein\_coding | 5:134357656-134456716 (-) |  | 1.6100 | 3.69e-37 | 3.22e-36 |
| ENSMUSG00000029923 | Rab19 | protein\_coding | 6:39381175-39390380 (+) |  | -2.1900 | 3.90e-37 | 3.40e-36 |
| ENSMUSG00000032485 | Scap | protein\_coding | 9:110333288-110384950 (+) |  | 0.8930 | 4.15e-37 | 3.62e-36 |
| ENSMUSG00000001062 | Vps9d1 | protein\_coding | 8:123242356-123254348 (-) |  | -1.0300 | 4.57e-37 | 3.99e-36 |
| ENSMUSG00000025239 | Limd1 | protein\_coding | 9:123478706-123521552 (+) |  | -0.5360 | 4.62e-37 | 4.02e-36 |
| ENSMUSG00000032252 | Glce | protein\_coding | 9:62057248-62122655 (-) |  | 0.7090 | 4.82e-37 | 4.20e-36 |
| ENSMUSG00000022180 | Slc7a8 | protein\_coding | 14:54722209-54781946 (-) |  | -1.8300 | 4.95e-37 | 4.30e-36 |
| ENSMUSG00000027378 | Nphp1 | protein\_coding | 2:127740732-127788897 (-) |  | 2.6900 | 5.07e-37 | 4.41e-36 |
| ENSMUSG00000024251 | Thada | protein\_coding | 17:84190076-84466205 (-) |  | 0.8050 | 5.12e-37 | 4.45e-36 |
| ENSMUSG00000117404 | Gm50035 | lncRNA | 18:15201112-15207791 (-) |  | 2.2400 | 5.16e-37 | 4.48e-36 |
| ENSMUSG00000066258 | Trim12a | protein\_coding | 7:104299894-104315466 (-) |  | -0.8130 | 5.58e-37 | 4.84e-36 |
| ENSMUSG00000053080 | 2700081O15Rik | protein\_coding | 19:7417625-7425904 (+) |  | 1.9200 | 5.76e-37 | 5.00e-36 |
| ENSMUSG00000020642 | Rnf144a | protein\_coding | 12:26300964-26415254 (-) |  | -0.6320 | 5.77e-37 | 5.00e-36 |
| ENSMUSG00000038518 | Jarid2 | protein\_coding | 13:44729474-44921643 (+) |  | -0.6810 | 5.78e-37 | 5.01e-36 |
| ENSMUSG00000024537 | Psmg2 | protein\_coding | 18:67641599-67654162 (+) |  | 0.8030 | 5.84e-37 | 5.05e-36 |
| ENSMUSG00000042660 | Wdr55 | protein\_coding | 18:36760220-36763810 (+) |  | 0.8580 | 5.86e-37 | 5.07e-36 |
| ENSMUSG00000022698 | Naa50 | protein\_coding | 16:44139830-44163366 (+) |  | 0.7790 | 6.69e-37 | 5.79e-36 |
| ENSMUSG00000044345 | Marveld1 | protein\_coding | 19:42147400-42151703 (+) |  | 0.9150 | 6.81e-37 | 5.89e-36 |
| ENSMUSG00000026463 | Atp2b4 | protein\_coding | 1:133699457-133801041 (-) |  | 1.2700 | 6.82e-37 | 5.89e-36 |
| ENSMUSG00000020949 | Fkbp3 | protein\_coding | 12:65062424-65074007 (-) |  | 1.0300 | 6.82e-37 | 5.89e-36 |
| ENSMUSG00000041268 | Dmxl2 | protein\_coding | 9:54365158-54501626 (-) |  | -0.9360 | 7.37e-37 | 6.36e-36 |
| ENSMUSG00000069769 | Msi2 | protein\_coding | 11:88339382-88718513 (-) |  | 1.3700 | 7.39e-37 | 6.38e-36 |
| ENSMUSG00000033906 | Zdhhc15 | protein\_coding | X:104536969-104671064 (-) |  | 2.0900 | 7.49e-37 | 6.46e-36 |
| ENSMUSG00000079227 | Ccr5 | protein\_coding | 9:124121543-124147699 (+) |  | -1.8000 | 7.51e-37 | 6.47e-36 |
| ENSMUSG00000020657 | Dnajc27 | protein\_coding | 12:4082583-4110606 (+) |  | 1.1200 | 7.90e-37 | 6.81e-36 |
| ENSMUSG00000062373 | Tmem65 | protein\_coding | 15:58782269-58823638 (-) |  | -0.8140 | 1.01e-36 | 8.66e-36 |
| ENSMUSG00000022191 | Drosha | protein\_coding | 15:12824815-12935291 (+) |  | 0.8380 | 1.04e-36 | 8.93e-36 |
| ENSMUSG00000026709 | Dars2 | protein\_coding | 1:161040601-161070658 (-) |  | 0.8130 | 1.12e-36 | 9.64e-36 |
| ENSMUSG00000000686 | Abhd15 | protein\_coding | 11:77515121-77538607 (+) |  | -0.8970 | 1.22e-36 | 1.05e-35 |
| ENSMUSG00000024965 | Fermt3 | protein\_coding | 19:6998958-7019469 (-) |  | -0.4520 | 1.27e-36 | 1.09e-35 |
| ENSMUSG00000020733 | Slc9a3r1 | protein\_coding | 11:115163341-115181181 (+) |  | -0.5940 | 1.27e-36 | 1.09e-35 |
| ENSMUSG00000029426 | Scarb2 | protein\_coding | 5:92441314-92506833 (-) |  | -0.6290 | 1.52e-36 | 1.30e-35 |
| ENSMUSG00000030057 | Cnbp | protein\_coding | 6:87842615-87851106 (-) |  | 0.9790 | 1.53e-36 | 1.31e-35 |
| ENSMUSG00000056459 | Zbtb25 | protein\_coding | 12:76347782-76369602 (-) |  | 1.0300 | 1.54e-36 | 1.32e-35 |
| ENSMUSG00000044768 | D1Ertd622e | protein\_coding | 1:97606318-97662074 (-) |  | -0.7950 | 1.54e-36 | 1.32e-35 |
| ENSMUSG00000025579 | Gaa | protein\_coding | 11:119267887-119285454 (+) |  | 0.8420 | 1.55e-36 | 1.33e-35 |
| ENSMUSG00000071714 | Csf2rb2 | protein\_coding | 15:78282507-78305721 (-) |  | -0.8630 | 1.62e-36 | 1.38e-35 |
| ENSMUSG00000026832 | Cytip | protein\_coding | 2:58129137-58195532 (-) |  | -1.4000 | 1.62e-36 | 1.38e-35 |
| ENSMUSG00000018433 | Nol11 | protein\_coding | 11:107166663-107189381 (-) |  | 0.7020 | 1.74e-36 | 1.49e-35 |
| ENSMUSG00000102418 | Sh2d1b1 | protein\_coding | 1:170277320-170286769 (+) |  | -1.2200 | 1.77e-36 | 1.51e-35 |
| ENSMUSG00000024900 | Cpt1a | protein\_coding | 19:3322334-3385733 (+) |  | -0.9210 | 1.90e-36 | 1.62e-35 |
| ENSMUSG00000031591 | Asah1 | protein\_coding | 8:41340197-41374773 (-) |  | -0.8750 | 1.93e-36 | 1.64e-35 |
| ENSMUSG00000025791 | Pgm1 | protein\_coding | 4:99929414-99987294 (+) |  | 0.8550 | 1.97e-36 | 1.68e-35 |
| ENSMUSG00000030161 | Gabarapl1 | protein\_coding | 6:129533160-129542346 (+) |  | 0.8300 | 2.05e-36 | 1.75e-35 |
| ENSMUSG00000021532 | Fastkd3 | protein\_coding | 13:68582234-68592338 (+) |  | 1.2000 | 2.17e-36 | 1.85e-35 |
| ENSMUSG00000029640 | Usp12 | protein\_coding | 5:146734809-146795006 (-) |  | -0.5110 | 2.20e-36 | 1.87e-35 |
| ENSMUSG00000027823 | Gmps | protein\_coding | 3:63976106-64022579 (+) |  | 0.6040 | 2.47e-36 | 2.10e-35 |
| ENSMUSG00000020432 | Tcn2 | protein\_coding | 11:3917192-3932159 (-) |  | -0.6860 | 2.49e-36 | 2.11e-35 |
| ENSMUSG00000055866 | Per2 | protein\_coding | 1:91415982-91459324 (-) |  | 2.6900 | 2.78e-36 | 2.37e-35 |
| ENSMUSG00000061665 | Cd2ap | protein\_coding | 17:42792951-42876665 (-) |  | -0.9120 | 2.79e-36 | 2.37e-35 |
| ENSMUSG00000028330 | Ncbp1 | protein\_coding | 4:46138613-46172403 (+) |  | 0.5250 | 2.84e-36 | 2.41e-35 |
| ENSMUSG00000064254 | Ethe1 | protein\_coding | 7:24587543-24608925 (+) |  | -0.6950 | 3.19e-36 | 2.70e-35 |
| ENSMUSG00000035666 | Gtf3c4 | protein\_coding | 2:28822299-28840360 (-) |  | 0.7010 | 3.19e-36 | 2.71e-35 |
| ENSMUSG00000018326 | Ywhab | protein\_coding | 2:163994960-164018588 (+) |  | -0.4500 | 3.35e-36 | 2.83e-35 |
| ENSMUSG00000033356 | Pus7l | protein\_coding | 15:94522688-94543547 (-) |  | 0.9600 | 3.41e-36 | 2.89e-35 |
| ENSMUSG00000004562 | Arhgef40 | protein\_coding | 14:51984719-52006251 (+) |  | 2.3400 | 3.48e-36 | 2.94e-35 |
| ENSMUSG00000020399 | Havcr2 | protein\_coding | 11:46454935-46481255 (+) |  | -2.6400 | 3.90e-36 | 3.30e-35 |
| ENSMUSG00000033016 | Nfatc1 | protein\_coding | 18:80606205-80713071 (-) |  | -0.7940 | 4.02e-36 | 3.40e-35 |
| ENSMUSG00000024429 | Gnl1 | protein\_coding | 17:35979851-35989462 (+) |  | 0.8350 | 4.34e-36 | 3.67e-35 |
| ENSMUSG00000041132 | N4bp2l1 | protein\_coding | 5:150571644-150597188 (-) |  | -0.9680 | 4.65e-36 | 3.93e-35 |
| ENSMUSG00000029098 | Acox3 | protein\_coding | 5:35583040-35615352 (+) |  | -0.8560 | 4.68e-36 | 3.95e-35 |
| ENSMUSG00000089715 | Cbx6 | protein\_coding | 15:79823896-79834688 (-) |  | 1.2800 | 4.77e-36 | 4.03e-35 |
| ENSMUSG00000024030 | Abcg1 | protein\_coding | 17:31057675-31117988 (+) |  | -1.4800 | 4.82e-36 | 4.07e-35 |
| ENSMUSG00000025190 | Got1 | protein\_coding | 19:43499752-43524605 (-) |  | 1.2000 | 4.86e-36 | 4.09e-35 |
| ENSMUSG00000018666 | Cbx1 | protein\_coding | 11:96789127-96808640 (+) |  | 0.5440 | 4.96e-36 | 4.18e-35 |
| ENSMUSG00000033107 | Rnf125 | protein\_coding | 18:20944625-20983862 (+) |  | 2.5500 | 5.12e-36 | 4.31e-35 |
| ENSMUSG00000023106 | Denr | protein\_coding | 5:123907175-123928835 (+) |  | 0.9210 | 5.32e-36 | 4.47e-35 |
| ENSMUSG00000073775 | Kti12 | protein\_coding | 4:108847785-108849413 (+) |  | 0.7200 | 5.37e-36 | 4.51e-35 |
| ENSMUSG00000000711 | Rab5b | protein\_coding | 10:128677175-128696264 (-) |  | -0.5200 | 5.49e-36 | 4.61e-35 |
| ENSMUSG00000040034 | Nup43 | protein\_coding | 10:7667503-7678881 (+) |  | 1.2100 | 5.91e-36 | 4.96e-35 |
| ENSMUSG00000024620 | Pdgfrb | protein\_coding | 18:61045150-61085061 (+) |  | 3.7400 | 6.02e-36 | 5.05e-35 |
| ENSMUSG00000038517 | Tbkbp1 | protein\_coding | 11:97136171-97151495 (-) |  | -0.9590 | 6.16e-36 | 5.16e-35 |
| ENSMUSG00000058173 | Smco4 | protein\_coding | 9:15493432-15545260 (+) |  | 1.5700 | 6.41e-36 | 5.37e-35 |
| ENSMUSG00000046668 | Cxxc5 | protein\_coding | 18:35829397-35861688 (+) |  | 2.5400 | 6.42e-36 | 5.38e-35 |
| ENSMUSG00000034282 | Evpl | protein\_coding | 11:116220559-116238077 (-) |  | 4.7500 | 6.75e-36 | 5.65e-35 |
| ENSMUSG00000030089 | Slc41a3 | protein\_coding | 6:90604725-90646412 (+) |  | -1.3700 | 6.81e-36 | 5.70e-35 |
| ENSMUSG00000031652 | N4bp1 | protein\_coding | 8:86808160-86885258 (-) |  | -0.6230 | 6.86e-36 | 5.74e-35 |
| ENSMUSG00000059714 | Flot1 | protein\_coding | 17:35823230-35832791 (+) |  | -0.8450 | 7.02e-36 | 5.86e-35 |
| ENSMUSG00000025142 | Aspscr1 | protein\_coding | 11:120672973-120709447 (+) |  | -0.5500 | 7.08e-36 | 5.92e-35 |
| ENSMUSG00000059498 | Fcgr3 | protein\_coding | 1:171051174-171064935 (-) |  | -0.8610 | 7.31e-36 | 6.10e-35 |
| ENSMUSG00000021948 | Prkcd | protein\_coding | 14:30595354-30626210 (-) |  | -0.7510 | 7.54e-36 | 6.29e-35 |
| ENSMUSG00000029534 | St7 | protein\_coding | 6:17692933-17943025 (+) |  | 1.2300 | 7.69e-36 | 6.41e-35 |
| ENSMUSG00000048148 | Nwd1 | protein\_coding | 8:72646711-72717876 (+) |  | 5.0300 | 7.72e-36 | 6.43e-35 |
| ENSMUSG00000024654 | Asrgl1 | protein\_coding | 19:9109868-9135636 (-) |  | 0.8180 | 7.75e-36 | 6.45e-35 |
| ENSMUSG00000057143 | Trim12c | protein\_coding | 7:104338754-104353362 (-) |  | -0.7710 | 8.44e-36 | 7.03e-35 |
| ENSMUSG00000039747 | Orai2 | protein\_coding | 5:136147459-136170713 (-) |  | -0.7890 | 8.84e-36 | 7.35e-35 |
| ENSMUSG00000044465 | Fam160a2 | protein\_coding | 7:105371211-105400054 (-) |  | -1.0600 | 9.13e-36 | 7.59e-35 |
| ENSMUSG00000026626 | Ppp2r5a | protein\_coding | 1:191351975-191403272 (-) |  | -0.8750 | 9.32e-36 | 7.74e-35 |
| ENSMUSG00000062078 | Qk | protein\_coding | 17:10202601-10319854 (-) |  | -0.5210 | 9.37e-36 | 7.78e-35 |
| ENSMUSG00000030512 | Snrpa1 | protein\_coding | 7:66059003-66074587 (+) |  | 0.7320 | 1.03e-35 | 8.58e-35 |
| ENSMUSG00000047710 | Champ1 | protein\_coding | 8:13869641-13881639 (+) |  | 0.7350 | 1.08e-35 | 8.95e-35 |
| ENSMUSG00000038172 | Ttc39b | protein\_coding | 4:83220300-83324255 (-) |  | -3.2000 | 1.16e-35 | 9.63e-35 |
| ENSMUSG00000021699 | Pde4d | protein\_coding | 13:108449948-109953461 (+) |  | 2.1300 | 1.18e-35 | 9.77e-35 |
| ENSMUSG00000027722 | Spata5 | protein\_coding | 3:37419896-37579096 (+) |  | 0.7950 | 1.33e-35 | 1.10e-34 |
| ENSMUSG00000039908 | Slc26a11 | protein\_coding | 11:119355557-119381079 (+) |  | -1.9900 | 1.36e-35 | 1.12e-34 |
| ENSMUSG00000024451 | Arap3 | protein\_coding | 18:37972628-37998969 (-) |  | -1.3500 | 1.39e-35 | 1.15e-34 |
| ENSMUSG00000022403 | St13 | protein\_coding | 15:81363669-81400077 (-) |  | 0.7110 | 1.40e-35 | 1.16e-34 |
| ENSMUSG00000028394 | Pole3 | protein\_coding | 4:62522649-62525068 (-) |  | 0.7930 | 1.43e-35 | 1.18e-34 |
| ENSMUSG00000039929 | Urb1 | protein\_coding | 16:90751527-90810413 (-) |  | 0.8540 | 1.53e-35 | 1.27e-34 |
| ENSMUSG00000021846 | Peli2 | protein\_coding | 14:48120823-48281575 (+) |  | -0.8930 | 1.58e-35 | 1.31e-34 |
| ENSMUSG00000020638 | Cmpk2 | protein\_coding | 12:26469204-26479837 (+) |  | -0.8740 | 1.62e-35 | 1.33e-34 |
| ENSMUSG00000023927 | Satb1 | protein\_coding | 17:51736187-51833290 (-) |  | 1.4000 | 1.65e-35 | 1.36e-34 |
| ENSMUSG00000042446 | Zmym4 | protein\_coding | 4:126861939-126968135 (-) |  | 0.7120 | 1.67e-35 | 1.38e-34 |
| ENSMUSG00000026269 | Rnpepl1 | protein\_coding | 1:92910783-92924384 (+) |  | -0.7680 | 1.74e-35 | 1.43e-34 |
| ENSMUSG00000078521 | Aunip | protein\_coding | 4:134510999-134523927 (+) |  | 1.1800 | 2.16e-35 | 1.78e-34 |
| ENSMUSG00000019790 | Stxbp5 | protein\_coding | 10:9755547-9901079 (-) |  | 0.9040 | 2.18e-35 | 1.79e-34 |
| ENSMUSG00000028896 | Rcc1 | protein\_coding | 4:132331919-132353605 (-) |  | 0.8020 | 2.33e-35 | 1.92e-34 |
| ENSMUSG00000036918 | Ttc7 | protein\_coding | 17:87282886-87381769 (+) |  | -0.5480 | 2.43e-35 | 2.00e-34 |
| ENSMUSG00000040940 | Arhgef1 | protein\_coding | 7:24902912-24926594 (+) |  | -0.7260 | 2.44e-35 | 2.01e-34 |
| ENSMUSG00000022124 | Fbxl3 | protein\_coding | 14:103080239-103099566 (-) |  | -0.6250 | 2.53e-35 | 2.08e-34 |
| ENSMUSG00000068735 | Trp53i11 | protein\_coding | 2:93187548-93201759 (+) |  | 4.3300 | 2.66e-35 | 2.19e-34 |
| ENSMUSG00000030814 | Bcl7c | protein\_coding | 7:127661456-127708933 (-) |  | 0.8690 | 2.72e-35 | 2.23e-34 |
| ENSMUSG00000004508 | Gab2 | protein\_coding | 7:97081586-97308946 (+) |  | -0.6700 | 2.96e-35 | 2.43e-34 |
| ENSMUSG00000024640 | Psat1 | protein\_coding | 19:15904678-15947337 (-) |  | 0.7610 | 2.97e-35 | 2.43e-34 |
| ENSMUSG00000027597 | Ahcy | protein\_coding | 2:155059310-155074497 (-) |  | 1.0400 | 3.07e-35 | 2.51e-34 |
| ENSMUSG00000025150 | Cbr2 | protein\_coding | 11:120729489-120732114 (-) |  | -2.8700 | 3.41e-35 | 2.79e-34 |
| ENSMUSG00000020570 | Sypl | protein\_coding | 12:32953891-32979860 (+) |  | 0.4040 | 3.49e-35 | 2.85e-34 |
| ENSMUSG00000028970 | Abcb1b | protein\_coding | 5:8798147-8866315 (+) |  | 0.9830 | 3.60e-35 | 2.94e-34 |
| ENSMUSG00000067995 | Gtf2f2 | protein\_coding | 14:75896937-76010865 (-) |  | 0.7470 | 3.61e-35 | 2.95e-34 |
| ENSMUSG00000075033 | Nxpe3 | protein\_coding | 16:55839953-55895285 (-) |  | 0.9920 | 3.63e-35 | 2.97e-34 |
| ENSMUSG00000036432 | Siah2 | protein\_coding | 3:58674938-58692400 (-) |  | -0.9480 | 3.66e-35 | 2.99e-34 |
| ENSMUSG00000087598 | Zfp111 | protein\_coding | 7:24193947-24211443 (-) |  | 1.0300 | 3.69e-35 | 3.01e-34 |
| ENSMUSG00000029614 | Rpl6 | protein\_coding | 5:121204481-121209241 (+) |  | 0.4370 | 3.73e-35 | 3.04e-34 |
| ENSMUSG00000031633 | Slc25a4 | protein\_coding | 8:46206797-46211284 (-) |  | 0.5880 | 3.83e-35 | 3.12e-34 |
| ENSMUSG00000025140 | Pycr1 | protein\_coding | 11:120635712-120643769 (-) |  | 1.5300 | 3.89e-35 | 3.16e-34 |
| ENSMUSG00000063065 | Mapk3 | protein\_coding | 7:126759601-126765819 (+) |  | -0.6240 | 4.20e-35 | 3.42e-34 |
| ENSMUSG00000042677 | Zc3h12a | protein\_coding | 4:125118423-125127840 (-) |  | -1.0100 | 4.23e-35 | 3.44e-34 |
| ENSMUSG00000029179 | Zcchc4 | protein\_coding | 5:52775409-52824665 (+) |  | 0.7960 | 4.26e-35 | 3.47e-34 |
| ENSMUSG00000029311 | Hsd17b11 | protein\_coding | 5:103989762-104021919 (-) |  | -0.5720 | 4.48e-35 | 3.64e-34 |
| ENSMUSG00000062093 | Gm10110 | transcribed\_processed\_pseudogene | 14:89896228-89899447 (-) |  | 1.6100 | 4.86e-35 | 3.95e-34 |
| ENSMUSG00000003814 | Calr | protein\_coding | 8:84841850-84846934 (-) |  | 1.3900 | 4.90e-35 | 3.98e-34 |
| ENSMUSG00000022439 | Parvg | protein\_coding | 15:84324026-84342978 (+) |  | 0.7070 | 4.95e-35 | 4.02e-34 |
| ENSMUSG00000075415 | Fnbp1 | protein\_coding | 2:31026206-31142008 (-) |  | -0.5910 | 4.99e-35 | 4.05e-34 |
| ENSMUSG00000060090 | Rp2 | protein\_coding | X:20364481-20405653 (+) |  | -0.8630 | 5.43e-35 | 4.40e-34 |
| ENSMUSG00000031781 | Ciapin1 | protein\_coding | 8:94819804-94838358 (-) |  | 0.7180 | 5.57e-35 | 4.51e-34 |
| ENSMUSG00000040078 | Ptges3-ps | processed\_pseudogene | 6:85843980-85844459 (+) |  | 0.8960 | 5.79e-35 | 4.69e-34 |
| ENSMUSG00000021706 | Zfyve16 | protein\_coding | 13:92487108-92530868 (-) |  | -0.8900 | 6.64e-35 | 5.37e-34 |
| ENSMUSG00000040688 | Tbl3 | protein\_coding | 17:24697949-24707660 (-) |  | 0.7700 | 7.08e-35 | 5.72e-34 |
| ENSMUSG00000040659 | Efhd2 | protein\_coding | 4:141858142-141874920 (-) |  | -0.5210 | 7.30e-35 | 5.90e-34 |
| ENSMUSG00000026880 | Stom | protein\_coding | 2:35313986-35336976 (-) |  | -0.4000 | 7.77e-35 | 6.28e-34 |
| ENSMUSG00000028641 | P3h1 | protein\_coding | 4:119232915-119248975 (+) |  | 1.2400 | 8.14e-35 | 6.57e-34 |
| ENSMUSG00000031402 | Mpp1 | protein\_coding | X:75109733-75131016 (-) |  | -0.6430 | 8.15e-35 | 6.57e-34 |
| ENSMUSG00000060477 | Irak2 | protein\_coding | 6:113638467-113695026 (+) |  | -1.1100 | 9.59e-35 | 7.73e-34 |
| ENSMUSG00000071645 | Tut1 | protein\_coding | 19:8953847-8966210 (+) |  | 0.7760 | 9.74e-35 | 7.85e-34 |
| ENSMUSG00000008730 | Hipk1 | protein\_coding | 3:103739815-103791563 (-) |  | -0.7970 | 1.03e-34 | 8.31e-34 |
| ENSMUSG00000032359 | Ctsh | protein\_coding | 9:90054152-90076089 (+) |  | -0.9780 | 1.13e-34 | 9.06e-34 |
| ENSMUSG00000032178 | Ilf3 | protein\_coding | 9:21367871-21405361 (+) |  | 0.7590 | 1.14e-34 | 9.16e-34 |
| ENSMUSG00000024953 | Prdx5 | protein\_coding | 19:6906697-6910106 (-) |  | -1.1800 | 1.17e-34 | 9.42e-34 |
| ENSMUSG00000008398 | Elk3 | protein\_coding | 10:93247414-93311135 (-) |  | -0.7230 | 1.21e-34 | 9.75e-34 |
| ENSMUSG00000030688 | Stard10 | protein\_coding | 7:101317086-101346626 (+) |  | 1.6000 | 1.28e-34 | 1.03e-33 |
| ENSMUSG00000029816 | Gpnmb | protein\_coding | 6:49036546-49070929 (+) |  | -1.9200 | 1.28e-34 | 1.03e-33 |
| ENSMUSG00000028744 | Pqlc2 | protein\_coding | 4:139294029-139310708 (-) |  | -1.1000 | 1.48e-34 | 1.18e-33 |
| ENSMUSG00000044986 | Tst | protein\_coding | 15:78399556-78405907 (-) |  | 1.6200 | 1.50e-34 | 1.20e-33 |
| ENSMUSG00000046822 | Slc39a3 | protein\_coding | 10:81028538-81037426 (-) |  | -0.7760 | 1.52e-34 | 1.22e-33 |
| ENSMUSG00000040722 | Scamp5 | protein\_coding | 9:57441327-57468060 (-) |  | 2.9400 | 1.59e-34 | 1.28e-33 |
| ENSMUSG00000054263 | Lifr | protein\_coding | 15:7090614-7197489 (+) |  | -2.5900 | 1.65e-34 | 1.32e-33 |
| ENSMUSG00000025962 | Fastkd2 | protein\_coding | 1:63730614-63754655 (+) |  | 1.0700 | 1.67e-34 | 1.33e-33 |
| ENSMUSG00000035900 | Gramd4 | protein\_coding | 15:86057695-86137634 (+) |  | -0.7320 | 1.68e-34 | 1.34e-33 |
| ENSMUSG00000044811 | Cd300c2 | protein\_coding | 11:114996721-115001880 (-) |  | -2.6000 | 1.71e-34 | 1.37e-33 |
| ENSMUSG00000091649 | Phf11b | protein\_coding | 14:59320964-59341351 (-) |  | -1.8800 | 1.74e-34 | 1.39e-33 |
| ENSMUSG00000030365 | Clec2i | protein\_coding | 6:128887588-128898167 (+) |  | -2.4000 | 1.79e-34 | 1.43e-33 |
| ENSMUSG00000022142 | Nup155 | protein\_coding | 15:8109273-8161247 (+) |  | 0.6690 | 1.82e-34 | 1.45e-33 |
| ENSMUSG00000078789 | Dph1 | protein\_coding | 11:75177643-75191241 (-) |  | 1.1800 | 1.97e-34 | 1.57e-33 |
| ENSMUSG00000047649 | Cd3eap | protein\_coding | 7:19356014-19359483 (-) |  | 0.8420 | 2.05e-34 | 1.64e-33 |
| ENSMUSG00000047539 | Fbxo28 | protein\_coding | 1:182313102-182341629 (-) |  | -0.5860 | 2.07e-34 | 1.65e-33 |
| ENSMUSG00000022604 | Cep97 | protein\_coding | 16:55899888-55934855 (-) |  | 1.2600 | 2.11e-34 | 1.68e-33 |
| ENSMUSG00000041712 | Ubr7 | protein\_coding | 12:102757967-102777707 (+) |  | 0.6360 | 2.37e-34 | 1.88e-33 |
| ENSMUSG00000029344 | Tpst2 | protein\_coding | 5:112276691-112315361 (+) |  | -0.8040 | 2.37e-34 | 1.88e-33 |
| ENSMUSG00000041459 | Tardbp | protein\_coding | 4:148612382-148627019 (-) |  | 0.5570 | 2.39e-34 | 1.90e-33 |
| ENSMUSG00000042729 | Wdr74 | protein\_coding | 19:8735827-8740624 (+) |  | 0.9210 | 2.64e-34 | 2.10e-33 |
| ENSMUSG00000028108 | Ecm1 | protein\_coding | 3:95734147-95739569 (-) |  | -2.1400 | 2.69e-34 | 2.14e-33 |
| ENSMUSG00000030660 | Pik3c2a | protein\_coding | 7:116337265-116443449 (-) |  | -0.9920 | 2.91e-34 | 2.31e-33 |
| ENSMUSG00000078812 | Eif5a | protein\_coding | 11:69916714-69921958 (-) |  | 0.7590 | 2.94e-34 | 2.33e-33 |
| ENSMUSG00000037461 | Ints7 | protein\_coding | 1:191575636-191623688 (+) |  | 0.9850 | 2.95e-34 | 2.34e-33 |
| ENSMUSG00000056602 | Fry | protein\_coding | 5:150118645-150497753 (+) |  | -1.1300 | 2.97e-34 | 2.36e-33 |
| ENSMUSG00000034424 | Gcsh | protein\_coding | 8:116981810-116993537 (-) |  | 1.1000 | 3.00e-34 | 2.38e-33 |
| ENSMUSG00000000486 | Sept1 | protein\_coding | 7:127214447-127233130 (-) |  | 2.5600 | 3.06e-34 | 2.42e-33 |
| ENSMUSG00000028101 | Pias3 | protein\_coding | 3:96696384-96706070 (+) |  | -0.9050 | 3.15e-34 | 2.50e-33 |
| ENSMUSG00000061119 | Prcp | protein\_coding | 7:92874470-92934583 (+) |  | -0.7060 | 3.17e-34 | 2.50e-33 |
| ENSMUSG00000024493 | Lars | protein\_coding | 18:42202298-42262194 (-) |  | 0.6780 | 3.25e-34 | 2.57e-33 |
| ENSMUSG00000057672 | Pkn1 | protein\_coding | 8:83666536-83699179 (-) |  | -0.5920 | 3.26e-34 | 2.58e-33 |
| ENSMUSG00000022175 | Lrp10 | protein\_coding | 14:54464137-54471497 (+) |  | -0.5410 | 3.28e-34 | 2.59e-33 |
| ENSMUSG00000031730 | Dhodh | protein\_coding | 8:109591343-109608673 (-) |  | 0.9790 | 3.29e-34 | 2.60e-33 |
| ENSMUSG00000041827 | Oasl1 | protein\_coding | 5:114923240-114937915 (+) |  | -2.2700 | 3.40e-34 | 2.68e-33 |
| ENSMUSG00000015755 | Tab2 | protein\_coding | 10:7905653-7956230 (-) |  | -0.5590 | 3.48e-34 | 2.75e-33 |
| ENSMUSG00000015850 | Adamtsl4 | protein\_coding | 3:95676201-95687917 (-) |  | -2.5000 | 3.56e-34 | 2.81e-33 |
| ENSMUSG00000039745 | Htatip2 | protein\_coding | 7:49759115-49773975 (+) |  | -0.7340 | 3.59e-34 | 2.83e-33 |
| ENSMUSG00000026341 | Actr3 | protein\_coding | 1:125392905-125435727 (-) |  | -0.6860 | 3.62e-34 | 2.85e-33 |
| ENSMUSG00000038375 | Trp53inp2 | protein\_coding | 2:155381059-155389850 (+) |  | -1.7000 | 3.72e-34 | 2.93e-33 |
| ENSMUSG00000041977 | Arhgef11 | protein\_coding | 3:87617559-87738034 (+) |  | -0.8170 | 4.14e-34 | 3.26e-33 |
| ENSMUSG00000000552 | Zfp385a | protein\_coding | 15:103313895-103340093 (-) |  | -0.8370 | 4.32e-34 | 3.40e-33 |
| ENSMUSG00000035958 | Tdp2 | protein\_coding | 13:24831679-24842153 (+) |  | 0.6180 | 4.50e-34 | 3.54e-33 |
| ENSMUSG00000034024 | Cct2 | protein\_coding | 10:117051001-117063814 (-) |  | 0.6520 | 4.53e-34 | 3.56e-33 |
| ENSMUSG00000098557 | Kctd12 | protein\_coding | 14:102976581-102982637 (-) |  | -0.6500 | 4.78e-34 | 3.75e-33 |
| ENSMUSG00000035173 | Ccdc186 | protein\_coding | 19:56787481-56822190 (-) |  | -0.8290 | 5.24e-34 | 4.11e-33 |
| ENSMUSG00000026749 | Nek6 | protein\_coding | 2:38511643-38594606 (+) |  | 1.4000 | 5.25e-34 | 4.12e-33 |
| ENSMUSG00000032409 | Atr | protein\_coding | 9:95857597-95951781 (+) |  | 1.0300 | 5.34e-34 | 4.18e-33 |
| ENSMUSG00000044934 | Zfp367 | protein\_coding | 13:64133018-64153202 (-) |  | 0.7980 | 5.44e-34 | 4.26e-33 |
| ENSMUSG00000046721 | Rpl14-ps1 | processed\_pseudogene | 7:45324965-45325617 (+) |  | 0.7660 | 5.50e-34 | 4.30e-33 |
| ENSMUSG00000042190 | Cmklr1 | protein\_coding | 5:113612354-113650426 (-) |  | -3.6600 | 5.65e-34 | 4.42e-33 |
| ENSMUSG00000081752 | Sms-ps | processed\_pseudogene | X:62775585-62776685 (-) |  | 0.8970 | 5.81e-34 | 4.54e-33 |
| ENSMUSG00000037465 | Klf10 | protein\_coding | 15:38291463-38300706 (-) |  | -1.0900 | 6.00e-34 | 4.69e-33 |
| ENSMUSG00000090213 | Tmem189 | protein\_coding | 2:167642608-167689822 (-) |  | -0.6780 | 6.07e-34 | 4.74e-33 |
| ENSMUSG00000028251 | Tstd3 | protein\_coding | 4:21757382-21767212 (-) |  | -1.0200 | 6.67e-34 | 5.21e-33 |
| ENSMUSG00000028673 | Fuca1 | protein\_coding | 4:135920735-135940311 (+) |  | -0.7370 | 6.78e-34 | 5.29e-33 |
| ENSMUSG00000037824 | Tspan14 | protein\_coding | 14:40906445-40966807 (-) |  | -0.5350 | 6.96e-34 | 5.43e-33 |
| ENSMUSG00000035311 | Gnptab | protein\_coding | 10:88379132-88447329 (+) |  | 0.7690 | 7.11e-34 | 5.54e-33 |
| ENSMUSG00000024667 | Tmem216 | protein\_coding | 19:10533865-10556238 (-) |  | 1.5100 | 7.13e-34 | 5.55e-33 |
| ENSMUSG00000047213 | Ythdf3 | protein\_coding | 3:16183212-16217037 (+) |  | -0.5880 | 7.30e-34 | 5.68e-33 |
| ENSMUSG00000021000 | Mia2 | protein\_coding | 12:59095799-59191583 (+) |  | -0.7790 | 7.31e-34 | 5.69e-33 |
| ENSMUSG00000004626 | Stxbp2 | protein\_coding | 8:3630955-3643644 (+) |  | -0.4090 | 7.50e-34 | 5.83e-33 |
| ENSMUSG00000033970 | Rfc3 | protein\_coding | 5:151642756-151651242 (-) |  | 0.9470 | 7.58e-34 | 5.89e-33 |
| ENSMUSG00000039943 | Plcb4 | protein\_coding | 2:135659011-136014593 (+) |  | 1.9200 | 7.77e-34 | 6.04e-33 |
| ENSMUSG00000039959 | Hip1 | protein\_coding | 5:135406531-135545120 (-) |  | -1.0600 | 8.06e-34 | 6.26e-33 |
| ENSMUSG00000023990 | Tfeb | protein\_coding | 17:47737030-47792419 (+) |  | -0.9350 | 8.40e-34 | 6.52e-33 |
| ENSMUSG00000046006 | Gapt | protein\_coding | 13:110352616-110357199 (-) |  | -0.8400 | 8.78e-34 | 6.81e-33 |
| ENSMUSG00000024966 | Stip1 | protein\_coding | 19:7020702-7039967 (-) |  | 0.7380 | 8.81e-34 | 6.83e-33 |
| ENSMUSG00000042590 | Ipo11 | protein\_coding | 13:106794439-106936958 (-) |  | 0.7290 | 9.06e-34 | 7.02e-33 |
| ENSMUSG00000063412 | Gm10131 | protein\_coding | 8:34111002-34111737 (-) |  | 1.1100 | 9.14e-34 | 7.08e-33 |
| ENSMUSG00000046079 | Lrrc8d | protein\_coding | 5:105699969-105832436 (+) |  | -0.6180 | 9.34e-34 | 7.23e-33 |
| ENSMUSG00000031422 | Morf4l2 | protein\_coding | X:136732942-136743690 (-) |  | 0.6550 | 9.48e-34 | 7.34e-33 |
| ENSMUSG00000026123 | Plekhb2 | protein\_coding | 1:34849976-34879580 (+) |  | -0.4970 | 1.03e-33 | 7.93e-33 |
| ENSMUSG00000004451 | Ralb | protein\_coding | 1:119470305-119504794 (-) |  | -0.8030 | 1.18e-33 | 9.15e-33 |
| ENSMUSG00000031927 | 1700012B09Rik | protein\_coding | 9:14756587-14771030 (-) |  | 2.0300 | 1.20e-33 | 9.24e-33 |
| ENSMUSG00000015291 | Gdi1 | protein\_coding | X:74304998-74311862 (+) |  | -0.7530 | 1.23e-33 | 9.51e-33 |
| ENSMUSG00000043740 | B430306N03Rik | protein\_coding | 17:48316141-48327024 (+) |  | -1.1800 | 1.26e-33 | 9.74e-33 |
| ENSMUSG00000028484 | Psip1 | protein\_coding | 4:83455680-83486459 (-) |  | 0.6130 | 1.30e-33 | 1.01e-32 |
| ENSMUSG00000037849 | Ifi206 | protein\_coding | 1:173468485-173491041 (-) |  | -3.9100 | 1.37e-33 | 1.06e-32 |
| ENSMUSG00000020661 | Dnmt3a | protein\_coding | 12:3806007-3914443 (+) |  | 1.1500 | 1.38e-33 | 1.07e-32 |
| ENSMUSG00000002835 | Chaf1a | protein\_coding | 17:56040439-56072289 (+) |  | 0.9040 | 1.44e-33 | 1.11e-32 |
| ENSMUSG00000028082 | Sh3d19 | protein\_coding | 3:85971109-86130526 (+) |  | 1.8600 | 1.48e-33 | 1.14e-32 |
| ENSMUSG00000005102 | Eif2ak4 | protein\_coding | 2:118388618-118475234 (+) |  | 0.8110 | 1.51e-33 | 1.16e-32 |
| ENSMUSG00000034187 | Nsf | protein\_coding | 11:103821782-103954056 (-) |  | -0.6960 | 1.52e-33 | 1.17e-32 |
| ENSMUSG00000008855 | Hdac5 | protein\_coding | 11:102194432-102230166 (-) |  | -0.7080 | 1.54e-33 | 1.18e-32 |
| ENSMUSG00000089726 | Mir17hg | lncRNA | 14:115042879-115046727 (+) |  | 1.5600 | 1.64e-33 | 1.26e-32 |
| ENSMUSG00000024847 | Aip | protein\_coding | 19:4114446-4125858 (-) |  | -0.9510 | 1.72e-33 | 1.32e-32 |
| ENSMUSG00000036533 | Cdc42ep3 | protein\_coding | 17:79333727-79355091 (-) |  | -0.7180 | 1.80e-33 | 1.38e-32 |
| ENSMUSG00000027324 | Rpusd2 | protein\_coding | 2:119034790-119039769 (+) |  | 1.3200 | 1.87e-33 | 1.44e-32 |
| ENSMUSG00000071072 | Ptges3 | protein\_coding | 10:128058954-128077272 (+) |  | 0.9450 | 1.98e-33 | 1.52e-32 |
| ENSMUSG00000036902 | Neto2 | protein\_coding | 8:85636588-85700924 (-) |  | 1.4500 | 2.06e-33 | 1.58e-32 |
| ENSMUSG00000030319 | Cand2 | protein\_coding | 6:115774538-115805557 (+) |  | 3.8700 | 2.08e-33 | 1.59e-32 |
| ENSMUSG00000077450 | Rab11b | protein\_coding | 17:33742484-33760530 (-) |  | -0.5020 | 2.16e-33 | 1.65e-32 |
| ENSMUSG00000039630 | Hnrnpu | protein\_coding | 1:178321108-178337797 (-) |  | 0.4470 | 2.31e-33 | 1.77e-32 |
| ENSMUSG00000001228 | Uhrf1 | protein\_coding | 17:56303321-56323486 (+) |  | 1.0500 | 2.34e-33 | 1.79e-32 |
| ENSMUSG00000023043 | Krt18 | protein\_coding | 15:102028180-102032027 (+) |  | 3.8600 | 2.37e-33 | 1.81e-32 |
| ENSMUSG00000073411 | H2-D1 | protein\_coding | 17:35262730-35267499 (+) |  | -0.8050 | 2.43e-33 | 1.86e-32 |
| ENSMUSG00000018189 | Uchl5 | protein\_coding | 1:143777272-143807466 (+) |  | 0.8440 | 2.62e-33 | 2.00e-32 |
| ENSMUSG00000025823 | Pdia4 | protein\_coding | 6:47796141-47813430 (-) |  | 0.9250 | 2.77e-33 | 2.12e-32 |
| ENSMUSG00000028822 | Tmem50a | protein\_coding | 4:134897849-134915024 (-) |  | -0.6290 | 2.78e-33 | 2.12e-32 |
| ENSMUSG00000050147 | F2rl3 | protein\_coding | 8:72761880-72763874 (+) |  | 3.2500 | 2.84e-33 | 2.17e-32 |
| ENSMUSG00000032263 | Bckdhb | protein\_coding | 9:83925145-84124240 (+) |  | 1.6000 | 2.87e-33 | 2.18e-32 |
| ENSMUSG00000020275 | Rel | protein\_coding | 11:23736847-23770970 (-) |  | -0.8340 | 2.98e-33 | 2.27e-32 |
| ENSMUSG00000029090 | Adgra3 | protein\_coding | 5:49959956-50059006 (-) |  | 3.7100 | 3.04e-33 | 2.31e-32 |
| ENSMUSG00000045038 | Prkce | protein\_coding | 17:86167785-86657919 (+) |  | -0.9010 | 3.05e-33 | 2.32e-32 |
| ENSMUSG00000097471 | 5830432E09Rik | lncRNA | 7:135635931-135652314 (-) |  | -1.6400 | 3.15e-33 | 2.40e-32 |
| ENSMUSG00000028173 | Wls | protein\_coding | 3:159839672-159938664 (+) |  | 0.5470 | 3.25e-33 | 2.47e-32 |
| ENSMUSG00000005043 | Sgsh | protein\_coding | 11:119343425-119355536 (-) |  | -1.0100 | 3.38e-33 | 2.57e-32 |
| ENSMUSG00000031482 | Slc25a15 | protein\_coding | 8:22375551-22398597 (-) |  | 0.9350 | 3.57e-33 | 2.71e-32 |
| ENSMUSG00000033594 | Spata2l | protein\_coding | 8:123229801-123236233 (-) |  | -1.3000 | 4.00e-33 | 3.04e-32 |
| ENSMUSG00000028709 | Mob3c | protein\_coding | 4:115828092-115836185 (+) |  | -1.0900 | 4.01e-33 | 3.04e-32 |
| ENSMUSG00000022422 | Dscc1 | protein\_coding | 15:55076099-55090491 (-) |  | 1.0500 | 4.05e-33 | 3.07e-32 |
| ENSMUSG00000026201 | Stk16 | protein\_coding | 1:75210838-75215606 (+) |  | 0.5630 | 4.10e-33 | 3.11e-32 |
| ENSMUSG00000040548 | Tex2 | protein\_coding | 11:106502147-106613423 (-) |  | 0.6440 | 4.11e-33 | 3.11e-32 |
| ENSMUSG00000011884 | Gltp | protein\_coding | 5:114669398-114690984 (-) |  | -0.6300 | 4.36e-33 | 3.30e-32 |
| ENSMUSG00000049969 | Plekhf2 | protein\_coding | 4:10988662-11007927 (-) |  | -0.6240 | 4.61e-33 | 3.49e-32 |
| ENSMUSG00000054814 | Usp46 | protein\_coding | 5:73998453-74068431 (-) |  | 0.9110 | 4.88e-33 | 3.69e-32 |
| ENSMUSG00000027509 | Rae1 | protein\_coding | 2:173000117-173015739 (+) |  | 0.6590 | 5.15e-33 | 3.89e-32 |
| ENSMUSG00000023972 | Ptk7 | protein\_coding | 17:46564471-46629504 (-) |  | 3.3000 | 5.42e-33 | 4.10e-32 |
| ENSMUSG00000001847 | Rac1 | protein\_coding | 5:143503634-143528036 (-) |  | -0.5720 | 5.60e-33 | 4.23e-32 |
| ENSMUSG00000027130 | Slc12a6 | protein\_coding | 2:112265825-112363163 (+) |  | -0.8240 | 5.70e-33 | 4.30e-32 |
| ENSMUSG00000074575 | Kcng1 | protein\_coding | 2:168260117-168281736 (-) |  | 8.9300 | 5.74e-33 | 4.33e-32 |
| ENSMUSG00000022090 | Pdlim2 | protein\_coding | 14:70164218-70177681 (-) |  | -1.1800 | 5.82e-33 | 4.39e-32 |
| ENSMUSG00000026176 | Ctdsp1 | protein\_coding | 1:74391509-74397285 (+) |  | -0.5220 | 5.83e-33 | 4.39e-32 |
| ENSMUSG00000020255 | D10Wsu102e | protein\_coding | 10:83360221-83488505 (+) |  | 0.6170 | 6.02e-33 | 4.53e-32 |
| ENSMUSG00000024855 | Pacs1 | protein\_coding | 19:5133158-5273119 (-) |  | -0.8100 | 6.36e-33 | 4.79e-32 |
| ENSMUSG00000020476 | Dbnl | protein\_coding | 11:5788488-5800962 (+) |  | -0.4660 | 6.79e-33 | 5.11e-32 |
| ENSMUSG00000033318 | Gstt2 | protein\_coding | 10:75831114-75837424 (-) |  | 1.5600 | 6.88e-33 | 5.18e-32 |
| ENSMUSG00000031917 | Nip7 | protein\_coding | 8:107056877-107060931 (+) |  | 0.9390 | 7.01e-33 | 5.27e-32 |
| ENSMUSG00000019297 | Nop9 | protein\_coding | 14:55745693-55755500 (+) |  | 0.5760 | 7.12e-33 | 5.35e-32 |
| ENSMUSG00000001794 | Capns1 | protein\_coding | 7:30186936-30198811 (-) |  | -0.6890 | 7.26e-33 | 5.45e-32 |
| ENSMUSG00000021583 | Erap1 | protein\_coding | 13:74639568-74693201 (+) |  | 0.5140 | 7.38e-33 | 5.54e-32 |
| ENSMUSG00000021569 | Trip13 | protein\_coding | 13:73911347-73937784 (-) |  | 0.7720 | 7.46e-33 | 5.60e-32 |
| ENSMUSG00000028771 | Ptpn12 | protein\_coding | 5:20986645-21055911 (-) |  | -0.9880 | 7.52e-33 | 5.64e-32 |
| ENSMUSG00000028107 | Tars2 | protein\_coding | 3:95739976-95760206 (-) |  | 0.7520 | 7.58e-33 | 5.68e-32 |
| ENSMUSG00000004056 | Akt2 | protein\_coding | 7:27591552-27640826 (+) |  | -0.4900 | 7.62e-33 | 5.71e-32 |
| ENSMUSG00000022817 | Itgb5 | protein\_coding | 16:33829665-33949338 (+) |  | -0.7950 | 7.98e-33 | 5.98e-32 |
| ENSMUSG00000074203 | G430095P16Rik | protein\_coding | 8:84723007-84726844 (+) |  | -1.3400 | 9.51e-33 | 7.12e-32 |
| ENSMUSG00000052085 | Dock8 | protein\_coding | 19:24999534-25202432 (+) |  | -0.7420 | 1.01e-32 | 7.57e-32 |
| ENSMUSG00000021451 | Sema4d | protein\_coding | 13:51685529-51793747 (-) |  | -1.0100 | 1.08e-32 | 8.05e-32 |
| ENSMUSG00000070780 | Rbm47 | protein\_coding | 5:66016549-66173118 (-) |  | -0.8700 | 1.08e-32 | 8.07e-32 |
| ENSMUSG00000026174 | Cnot9 | protein\_coding | 1:74506058-74530842 (+) |  | 0.6200 | 1.16e-32 | 8.64e-32 |
| ENSMUSG00000034731 | Dgkh | protein\_coding | 14:78558750-78732776 (-) |  | -1.1800 | 1.18e-32 | 8.82e-32 |
| ENSMUSG00000035378 | Shq1 | protein\_coding | 6:100568256-100671157 (-) |  | 1.1300 | 1.18e-32 | 8.83e-32 |
| ENSMUSG00000022019 | Tdrd3 | protein\_coding | 14:87416639-87545504 (+) |  | 0.7320 | 1.19e-32 | 8.85e-32 |
| ENSMUSG00000075028 | Prdm11 | protein\_coding | 2:92972018-93046167 (-) |  | 1.4000 | 1.19e-32 | 8.87e-32 |
| ENSMUSG00000026728 | Vim | protein\_coding | 2:13573927-13582826 (+) |  | -0.5550 | 1.39e-32 | 1.04e-31 |
| ENSMUSG00000028433 | Ubap2 | protein\_coding | 4:41194313-41275144 (-) |  | 0.8830 | 1.41e-32 | 1.05e-31 |
| ENSMUSG00000002111 | Spi1 | protein\_coding | 2:91082390-91115756 (+) |  | -0.5460 | 1.41e-32 | 1.05e-31 |
| ENSMUSG00000002489 | Tiam1 | protein\_coding | 16:89787111-90143769 (-) |  | -0.7180 | 1.41e-32 | 1.05e-31 |
| ENSMUSG00000038775 | Vill | protein\_coding | 9:119052778-119071525 (+) |  | -1.0400 | 1.52e-32 | 1.13e-31 |
| ENSMUSG00000055760 | Gemin6 | protein\_coding | 17:80224441-80228497 (+) |  | 1.1200 | 1.68e-32 | 1.25e-31 |
| ENSMUSG00000028902 | Sf3a3 | protein\_coding | 4:124714776-124732460 (+) |  | 0.5670 | 1.68e-32 | 1.25e-31 |
| ENSMUSG00000031954 | Cfdp1 | protein\_coding | 8:111768491-111854291 (-) |  | 0.6580 | 1.79e-32 | 1.33e-31 |
| ENSMUSG00000000916 | Nsun5 | protein\_coding | 5:135369953-135376805 (+) |  | 0.9070 | 1.80e-32 | 1.33e-31 |
| ENSMUSG00000035215 | Lsm7 | protein\_coding | 10:80852821-80855209 (-) |  | 0.8800 | 1.88e-32 | 1.39e-31 |
| ENSMUSG00000039741 | Bahcc1 | protein\_coding | 11:120232947-120292296 (+) |  | 1.3200 | 1.92e-32 | 1.42e-31 |
| ENSMUSG00000039263 | Npepl1 | protein\_coding | 2:174110349-174123070 (+) |  | 0.7650 | 2.07e-32 | 1.53e-31 |
| ENSMUSG00000007836 | Hnrnpa0 | protein\_coding | 13:58125879-58128556 (-) |  | 0.6110 | 2.15e-32 | 1.59e-31 |
| ENSMUSG00000074682 | Zcchc3 | protein\_coding | 2:152411955-152415027 (-) |  | 1.2700 | 2.16e-32 | 1.60e-31 |
| ENSMUSG00000027346 | Gpcpd1 | protein\_coding | 2:132529082-132587729 (-) |  | -1.1400 | 2.68e-32 | 1.98e-31 |
| ENSMUSG00000074102 | Rbm15b | protein\_coding | 9:106880918-106887428 (-) |  | 0.7040 | 2.70e-32 | 2.00e-31 |
| ENSMUSG00000059900 | Tmem40 | protein\_coding | 6:115729131-115762410 (-) |  | 3.8400 | 2.72e-32 | 2.01e-31 |
| ENSMUSG00000005813 | Metap1 | protein\_coding | 3:138458956-138489515 (-) |  | 0.5700 | 2.78e-32 | 2.06e-31 |
| ENSMUSG00000024130 | Abca3 | protein\_coding | 17:24351950-24410201 (+) |  | -0.8140 | 2.80e-32 | 2.07e-31 |
| ENSMUSG00000030421 | Uri1 | protein\_coding | 7:37959992-38023551 (-) |  | 0.6190 | 2.88e-32 | 2.13e-31 |
| ENSMUSG00000081665 | Gm15922 | polymorphic\_pseudogene | 7:3731630-3739889 (-) |  | -1.9500 | 2.99e-32 | 2.21e-31 |
| ENSMUSG00000068184 | Ndufaf2 | protein\_coding | 13:108002715-108158623 (-) |  | 1.1500 | 3.01e-32 | 2.22e-31 |
| ENSMUSG00000029086 | Prom1 | protein\_coding | 5:43993620-44102032 (-) |  | 3.6700 | 3.08e-32 | 2.27e-31 |
| ENSMUSG00000066682 | Pilrb2 | protein\_coding | 5:137865827-137871815 (-) |  | -2.0400 | 3.13e-32 | 2.30e-31 |
| ENSMUSG00000028675 | Pnrc2 | protein\_coding | 4:135870918-135873850 (-) |  | -0.7540 | 3.27e-32 | 2.41e-31 |
| ENSMUSG00000026288 | Inpp5d | protein\_coding | 1:87620312-87720507 (+) |  | -0.5420 | 3.40e-32 | 2.50e-31 |
| ENSMUSG00000030148 | Clec4a2 | protein\_coding | 6:123106428-123143999 (+) |  | -1.2700 | 3.43e-32 | 2.52e-31 |
| ENSMUSG00000030921 | Trim30a | protein\_coding | 7:104409025-104465193 (-) |  | -0.8520 | 3.46e-32 | 2.54e-31 |
| ENSMUSG00000035726 | Supt16 | protein\_coding | 14:52160414-52197416 (-) |  | 0.5540 | 3.53e-32 | 2.59e-31 |
| ENSMUSG00000022957 | Itsn1 | protein\_coding | 16:91729281-91920597 (+) |  | -0.9730 | 3.69e-32 | 2.71e-31 |
| ENSMUSG00000033960 | Jcad | protein\_coding | 18:4634878-4682869 (+) |  | 1.4400 | 3.81e-32 | 2.80e-31 |
| ENSMUSG00000029174 | Tbc1d1 | protein\_coding | 5:64156305-64351486 (+) |  | -0.6880 | 3.91e-32 | 2.87e-31 |
| ENSMUSG00000030869 | Ndufab1 | protein\_coding | 7:122085403-122101886 (-) |  | 0.7470 | 4.00e-32 | 2.93e-31 |
| ENSMUSG00000003546 | Klc4 | protein\_coding | 17:46630624-46646022 (-) |  | -0.9690 | 4.06e-32 | 2.98e-31 |
| ENSMUSG00000038425 | Poli | protein\_coding | 18:70508680-70530620 (-) |  | 1.1700 | 4.06e-32 | 2.98e-31 |
| ENSMUSG00000045551 | Fpr1 | protein\_coding | 17:17876471-17883940 (-) |  | -3.7100 | 4.10e-32 | 3.01e-31 |
| ENSMUSG00000031729 | Ist1 | protein\_coding | 8:109671325-109693260 (-) |  | -0.4410 | 4.16e-32 | 3.05e-31 |
| ENSMUSG00000038023 | Atp6v0a2 | protein\_coding | 5:124628576-124724455 (+) |  | -0.5050 | 4.23e-32 | 3.09e-31 |
| ENSMUSG00000041949 | Tango6 | protein\_coding | 8:106683068-106851439 (+) |  | 1.0900 | 4.34e-32 | 3.18e-31 |
| ENSMUSG00000048039 | Isg20l2 | protein\_coding | 3:87930314-87940686 (+) |  | 0.5070 | 4.59e-32 | 3.35e-31 |
| ENSMUSG00000059923 | Grb2 | protein\_coding | 11:115644045-115708597 (-) |  | -0.4360 | 4.65e-32 | 3.40e-31 |
| ENSMUSG00000001741 | Il16 | protein\_coding | 7:83642825-83745726 (-) |  | -0.5300 | 5.09e-32 | 3.72e-31 |
| ENSMUSG00000028639 | Ybx1 | protein\_coding | 4:119277981-119294604 (-) |  | 0.6520 | 5.20e-32 | 3.80e-31 |
| ENSMUSG00000019907 | Ppp1r12a | protein\_coding | 10:108162193-108284475 (+) |  | -0.5570 | 5.43e-32 | 3.96e-31 |
| ENSMUSG00000029512 | Ulk1 | protein\_coding | 5:110784488-110810097 (-) |  | -0.9460 | 5.48e-32 | 3.99e-31 |
| ENSMUSG00000031557 | Plekha2 | protein\_coding | 8:25039144-25102376 (-) |  | -0.6850 | 5.52e-32 | 4.02e-31 |
| ENSMUSG00000109244 | Gm44751 | lncRNA | 7:88311478-88315864 (+) |  | -1.7100 | 5.63e-32 | 4.10e-31 |
| ENSMUSG00000027133 | Nop10 | protein\_coding | 2:112261926-112263269 (+) |  | 1.0900 | 6.03e-32 | 4.39e-31 |
| ENSMUSG00000025804 | Ccr1 | protein\_coding | 9:123962124-123968692 (-) |  | -2.2300 | 6.08e-32 | 4.43e-31 |
| ENSMUSG00000038845 | Phb | protein\_coding | 11:95666957-95680773 (+) |  | 0.9850 | 6.16e-32 | 4.48e-31 |
| ENSMUSG00000028423 | Nfx1 | protein\_coding | 4:40970906-41025993 (+) |  | 0.5280 | 6.62e-32 | 4.81e-31 |
| ENSMUSG00000034617 | Mtrr | protein\_coding | 13:68560780-68582149 (-) |  | 1.1000 | 6.95e-32 | 5.05e-31 |
| ENSMUSG00000064023 | Klk8 | protein\_coding | 7:43797577-43803826 (+) |  | 2.6100 | 7.00e-32 | 5.09e-31 |
| ENSMUSG00000021998 | Lcp1 | protein\_coding | 14:75131101-75230842 (+) |  | -0.5790 | 7.56e-32 | 5.49e-31 |
| ENSMUSG00000026866 | Kynu | protein\_coding | 2:43555329-43682715 (+) |  | -1.8900 | 7.67e-32 | 5.56e-31 |
| ENSMUSG00000030609 | Aen | protein\_coding | 7:78895854-78911209 (+) |  | 0.8780 | 8.22e-32 | 5.96e-31 |
| ENSMUSG00000021929 | Kpna3 | protein\_coding | 14:61365211-61439874 (-) |  | 0.6190 | 8.91e-32 | 6.46e-31 |
| ENSMUSG00000046329 | Slc25a23 | protein\_coding | 17:57043711-57059863 (-) |  | 1.6200 | 9.40e-32 | 6.81e-31 |
| ENSMUSG00000018909 | Arrb1 | protein\_coding | 7:99535466-99606771 (+) |  | -0.8200 | 9.57e-32 | 6.93e-31 |
| ENSMUSG00000022443 | Myh9 | protein\_coding | 15:77760587-77842175 (-) |  | -0.6320 | 1.01e-31 | 7.32e-31 |
| ENSMUSG00000001542 | Ell2 | protein\_coding | 13:75706757-75772364 (+) |  | -1.5600 | 1.06e-31 | 7.68e-31 |
| ENSMUSG00000030104 | Edem1 | protein\_coding | 6:108828641-108859356 (+) |  | -0.6740 | 1.10e-31 | 7.98e-31 |
| ENSMUSG00000000056 | Narf | protein\_coding | 11:121237253-121255856 (+) |  | -1.1300 | 1.11e-31 | 8.01e-31 |
| ENSMUSG00000036622 | Atp13a2 | protein\_coding | 4:140986873-141007330 (+) |  | -0.8020 | 1.12e-31 | 8.10e-31 |
| ENSMUSG00000027752 | Exosc8 | protein\_coding | 3:54728678-54735393 (-) |  | 0.5720 | 1.12e-31 | 8.10e-31 |
| ENSMUSG00000023104 | Rfc2 | protein\_coding | 5:134581366-134601805 (+) |  | 0.4950 | 1.12e-31 | 8.11e-31 |
| ENSMUSG00000049791 | Fzd4 | protein\_coding | 7:89404355-89413134 (+) |  | -1.3700 | 1.15e-31 | 8.28e-31 |
| ENSMUSG00000052681 | Rap1b | protein\_coding | 10:117813871-117846035 (-) |  | -0.8730 | 1.23e-31 | 8.85e-31 |
| ENSMUSG00000027775 | Mfsd1 | protein\_coding | 3:67582741-67604237 (+) |  | -0.4640 | 1.25e-31 | 8.99e-31 |
| ENSMUSG00000056515 | Rab31 | protein\_coding | 17:65651729-65772752 (-) |  | -0.6040 | 1.31e-31 | 9.47e-31 |
| ENSMUSG00000020010 | Vnn3 | protein\_coding | 10:23851462-23869843 (+) |  | -4.5200 | 1.33e-31 | 9.55e-31 |
| ENSMUSG00000020923 | Ubtf | protein\_coding | 11:102304560-102319742 (-) |  | 0.5470 | 1.42e-31 | 1.02e-30 |
| ENSMUSG00000072115 | Ang | protein\_coding | 14:51091150-51102009 (+) |  | -1.7600 | 1.43e-31 | 1.03e-30 |
| ENSMUSG00000025817 | Nudt5 | protein\_coding | 2:5845019-5871895 (+) |  | 0.7670 | 1.43e-31 | 1.03e-30 |
| ENSMUSG00000035671 | Zswim4 | protein\_coding | 8:84210678-84237055 (-) |  | -0.8870 | 1.45e-31 | 1.04e-30 |
| ENSMUSG00000109036 | 2210406H18Rik | TEC | 7:121062852-121063959 (-) |  | -1.2000 | 1.45e-31 | 1.04e-30 |
| ENSMUSG00000075703 | Selenoi | protein\_coding | 5:30232581-30272427 (+) |  | 0.6010 | 1.46e-31 | 1.05e-30 |
| ENSMUSG00000027463 | Slc52a3 | protein\_coding | 2:151996511-152009258 (+) |  | -2.7300 | 1.46e-31 | 1.05e-30 |
| ENSMUSG00000015474 | Ppt2 | protein\_coding | 17:34616662-34628510 (-) |  | -0.8990 | 1.48e-31 | 1.06e-30 |
| ENSMUSG00000027454 | Gins1 | protein\_coding | 2:150905400-150931280 (+) |  | 0.9590 | 1.49e-31 | 1.07e-30 |
| ENSMUSG00000027078 | Ube2l6 | protein\_coding | 2:84798828-84810335 (+) |  | -1.4000 | 1.49e-31 | 1.07e-30 |
| ENSMUSG00000021003 | Galc | protein\_coding | 12:98202294-98259459 (-) |  | -0.7110 | 1.62e-31 | 1.16e-30 |
| ENSMUSG00000032050 | Rdx | protein\_coding | 9:52047173-52100463 (+) |  | 0.4630 | 1.71e-31 | 1.22e-30 |
| ENSMUSG00000032265 | Tent5a | protein\_coding | 9:85320439-85327348 (-) |  | -0.7910 | 1.74e-31 | 1.24e-30 |
| ENSMUSG00000006699 | Cdc42 | protein\_coding | 4:137319696-137357720 (-) |  | -0.5000 | 1.80e-31 | 1.29e-30 |
| ENSMUSG00000033102 | Cdc14b | protein\_coding | 13:64189268-64275290 (-) |  | -1.3100 | 1.84e-31 | 1.32e-30 |
| ENSMUSG00000027227 | Sord | protein\_coding | 2:122234749-122265340 (+) |  | 0.6330 | 1.96e-31 | 1.40e-30 |
| ENSMUSG00000074513 | Arfip1 | protein\_coding | 3:84496093-84582625 (-) |  | -0.9440 | 1.97e-31 | 1.40e-30 |
| ENSMUSG00000049848 | Ceacam19 | protein\_coding | 7:19875742-19887965 (-) |  | -2.9000 | 1.98e-31 | 1.42e-30 |
| ENSMUSG00000069515 | Lyz1 | protein\_coding | 10:117287797-117292868 (-) |  | -3.5100 | 2.09e-31 | 1.49e-30 |
| ENSMUSG00000011752 | Pgam1 | protein\_coding | 19:41911923-41918660 (+) |  | 0.7510 | 2.10e-31 | 1.50e-30 |
| ENSMUSG00000001604 | Tcea3 | protein\_coding | 4:136247729-136274898 (+) |  | -2.2900 | 2.12e-31 | 1.51e-30 |
| ENSMUSG00000047293 | Gpr15 | protein\_coding | 16:58717433-58719070 (-) |  | -1.8100 | 2.25e-31 | 1.60e-30 |
| ENSMUSG00000034681 | Rnps1 | protein\_coding | 17:24414565-24425901 (+) |  | 0.5240 | 2.39e-31 | 1.70e-30 |
| ENSMUSG00000033373 | Fntb | protein\_coding | 12:76765589-76921412 (+) |  | 1.2900 | 2.51e-31 | 1.78e-30 |
| ENSMUSG00000021276 | Cinp | protein\_coding | 12:110872610-110889145 (-) |  | 0.6860 | 2.51e-31 | 1.79e-30 |
| ENSMUSG00000070939 | Tgfbrap1 | protein\_coding | 1:43047200-43098637 (-) |  | -0.4850 | 2.55e-31 | 1.81e-30 |
| ENSMUSG00000026393 | Nek7 | protein\_coding | 1:138482875-138620141 (-) |  | -0.5860 | 2.58e-31 | 1.83e-30 |
| ENSMUSG00000027076 | Timm10 | protein\_coding | 2:84826997-84830213 (+) |  | 1.0400 | 2.59e-31 | 1.84e-30 |
| ENSMUSG00000033114 | Slc35d2 | protein\_coding | 13:64096308-64129368 (-) |  | -1.3600 | 2.69e-31 | 1.91e-30 |
| ENSMUSG00000090946 | Ccdc71l | protein\_coding | 12:32378704-32382943 (+) |  | -0.6610 | 2.72e-31 | 1.93e-30 |
| ENSMUSG00000026279 | Thap4 | protein\_coding | 1:93701935-93754864 (-) |  | 0.8830 | 2.85e-31 | 2.02e-30 |
| ENSMUSG00000020484 | Xbp1 | protein\_coding | 11:5520659-5525893 (+) |  | -0.5100 | 2.90e-31 | 2.06e-30 |
| ENSMUSG00000026637 | Traf5 | protein\_coding | 1:191997205-192092559 (-) |  | -1.0300 | 2.91e-31 | 2.06e-30 |
| ENSMUSG00000020361 | Hspa4 | protein\_coding | 11:53259814-53300457 (-) |  | 0.6080 | 3.18e-31 | 2.25e-30 |
| ENSMUSG00000002812 | Flii | protein\_coding | 11:60714123-60727263 (-) |  | -0.5180 | 3.27e-31 | 2.32e-30 |
| ENSMUSG00000017861 | Mybl2 | protein\_coding | 2:163054687-163084688 (+) |  | 1.0700 | 3.31e-31 | 2.34e-30 |
| ENSMUSG00000005882 | Uqcc1 | protein\_coding | 2:155846894-155930310 (-) |  | 0.7210 | 3.37e-31 | 2.38e-30 |
| ENSMUSG00000020974 | Pole2 | protein\_coding | 12:69201773-69228195 (-) |  | 0.8720 | 3.58e-31 | 2.53e-30 |
| ENSMUSG00000031592 | Pcm1 | protein\_coding | 8:41239752-41332344 (+) |  | 0.4990 | 3.76e-31 | 2.66e-30 |
| ENSMUSG00000022844 | Pdia5 | protein\_coding | 16:35397312-35490873 (-) |  | 1.9500 | 3.88e-31 | 2.74e-30 |
| ENSMUSG00000059182 | Skap2 | protein\_coding | 6:51857422-52012549 (-) |  | -0.6610 | 3.89e-31 | 2.75e-30 |
| ENSMUSG00000011254 | Thg1l | protein\_coding | 11:45946843-45955494 (-) |  | 0.8520 | 3.95e-31 | 2.78e-30 |
| ENSMUSG00000031357 | Syap1 | protein\_coding | X:162857057-162888447 (-) |  | -0.8580 | 3.97e-31 | 2.80e-30 |
| ENSMUSG00000060591 | Ifitm2 | protein\_coding | 7:140954837-140955987 (-) |  | -0.6650 | 4.00e-31 | 2.82e-30 |
| ENSMUSG00000073889 | Il11ra1 | protein\_coding | 4:41699989-41769474 (+) |  | 0.8000 | 4.00e-31 | 2.82e-30 |
| ENSMUSG00000024571 | Gm16286 | protein\_coding | 18:80206765-80212741 (+) |  | 0.5160 | 4.40e-31 | 3.10e-30 |
| ENSMUSG00000008859 | Rala | protein\_coding | 13:17880571-17944239 (-) |  | -0.7800 | 4.47e-31 | 3.15e-30 |
| ENSMUSG00000026946 | Nmi | protein\_coding | 2:51948487-51973494 (-) |  | -0.8410 | 4.68e-31 | 3.30e-30 |
| ENSMUSG00000036636 | Clcn7 | protein\_coding | 17:25133391-25162104 (+) |  | -0.6580 | 5.01e-31 | 3.52e-30 |
| ENSMUSG00000025584 | Pde8a | protein\_coding | 7:81213596-81334533 (+) |  | -0.9440 | 5.16e-31 | 3.63e-30 |
| ENSMUSG00000026872 | Zeb2 | protein\_coding | 2:44983632-45117395 (-) |  | -0.8150 | 5.30e-31 | 3.73e-30 |
| ENSMUSG00000019254 | Ppp1r12c | protein\_coding | 7:4481520-4501680 (-) |  | -0.5530 | 5.38e-31 | 3.78e-30 |
| ENSMUSG00000059119 | Nap1l4 | protein\_coding | 7:143513579-143549106 (-) |  | 0.4080 | 5.46e-31 | 3.83e-30 |
| ENSMUSG00000026696 | Vamp4 | protein\_coding | 1:162570515-162599084 (+) |  | -1.0500 | 5.47e-31 | 3.84e-30 |
| ENSMUSG00000070476 | Fam217b | protein\_coding | 2:178414524-178424428 (+) |  | -0.9000 | 5.56e-31 | 3.90e-30 |
| ENSMUSG00000067148 | Polr1c | protein\_coding | 17:46243920-46248054 (-) |  | 0.6320 | 5.80e-31 | 4.06e-30 |
| ENSMUSG00000083899 | Gm12346 | transcribed\_processed\_pseudogene | 11:77293444-77295898 (+) |  | 1.3300 | 5.80e-31 | 4.06e-30 |
| ENSMUSG00000033739 | Fkbpl | protein\_coding | 17:34644764-34646324 (+) |  | -1.0800 | 6.11e-31 | 4.28e-30 |
| ENSMUSG00000102155 | Gm37468 | lncRNA | 3:15547479-15548599 (+) |  | -3.0700 | 6.18e-31 | 4.32e-30 |
| ENSMUSG00000000708 | Kat2b | protein\_coding | 17:53566861-53672720 (+) |  | -0.7490 | 6.34e-31 | 4.44e-30 |
| ENSMUSG00000034341 | Wbp2 | protein\_coding | 11:116078573-116086995 (-) |  | -0.7460 | 6.44e-31 | 4.50e-30 |
| ENSMUSG00000056130 | Ticam2 | protein\_coding | 18:46557291-46574533 (-) |  | -0.5700 | 6.51e-31 | 4.55e-30 |
| ENSMUSG00000000826 | Dnajc5 | protein\_coding | 2:181520485-181555133 (+) |  | -0.3330 | 6.59e-31 | 4.61e-30 |
| ENSMUSG00000071369 | Map3k5 | protein\_coding | 10:19934472-20142753 (+) |  | -0.8800 | 6.60e-31 | 4.61e-30 |
| ENSMUSG00000062901 | Klhl24 | protein\_coding | 16:20097542-20129221 (+) |  | -0.8040 | 7.27e-31 | 5.08e-30 |
| ENSMUSG00000028645 | Slc2a1 | protein\_coding | 4:119108711-119137983 (+) |  | -0.8580 | 7.29e-31 | 5.08e-30 |
| ENSMUSG00000004610 | Etfb | protein\_coding | 7:43444083-43457800 (+) |  | 0.8120 | 7.53e-31 | 5.25e-30 |
| ENSMUSG00000019055 | Plod1 | protein\_coding | 4:147909753-147936767 (-) |  | -0.8840 | 7.65e-31 | 5.33e-30 |
| ENSMUSG00000026516 | Nvl | protein\_coding | 1:181087138-181144204 (-) |  | 0.5600 | 7.95e-31 | 5.54e-30 |
| ENSMUSG00000028018 | Gstcd | protein\_coding | 3:132981752-133092033 (-) |  | 1.1100 | 8.06e-31 | 5.61e-30 |
| ENSMUSG00000035000 | Dpp4 | protein\_coding | 2:62330073-62412231 (-) |  | 1.1300 | 8.11e-31 | 5.65e-30 |
| ENSMUSG00000008384 | Sertad1 | protein\_coding | 7:27486910-27490316 (+) |  | -1.1800 | 8.46e-31 | 5.88e-30 |
| ENSMUSG00000092416 | Zfp141 | protein\_coding | 7:42473386-42505740 (-) |  | 0.8530 | 8.86e-31 | 6.17e-30 |
| ENSMUSG00000043940 | Wdfy3 | protein\_coding | 5:101832956-102069921 (-) |  | -0.9690 | 9.40e-31 | 6.53e-30 |
| ENSMUSG00000038759 | Nup205 | protein\_coding | 6:35177421-35247596 (+) |  | 0.6600 | 9.48e-31 | 6.59e-30 |
| ENSMUSG00000040354 | Mars | protein\_coding | 10:127296221-127311786 (-) |  | 0.6960 | 9.69e-31 | 6.73e-30 |
| ENSMUSG00000021215 | Net1 | protein\_coding | 13:3882018-3918220 (-) |  | 0.7360 | 9.77e-31 | 6.78e-30 |
| ENSMUSG00000031539 | Ap3m2 | protein\_coding | 8:22787354-22805622 (-) |  | -1.3300 | 1.04e-30 | 7.19e-30 |
| ENSMUSG00000028156 | Eif4e | protein\_coding | 3:138526179-138559696 (+) |  | 0.6430 | 1.07e-30 | 7.42e-30 |
| ENSMUSG00000021427 | Ssr1 | protein\_coding | 13:37966605-37994217 (-) |  | 0.5790 | 1.07e-30 | 7.45e-30 |
| ENSMUSG00000084349 | Rpl3-ps1 | processed\_pseudogene | X:13202571-13203780 (+) |  | 0.5700 | 1.11e-30 | 7.71e-30 |
| ENSMUSG00000059060 | Rad51b | protein\_coding | 12:79297282-79814690 (+) |  | 1.4900 | 1.21e-30 | 8.36e-30 |
| ENSMUSG00000006057 | Atp5g1 | protein\_coding | 11:96068852-96075670 (-) |  | 0.8640 | 1.23e-30 | 8.52e-30 |
| ENSMUSG00000020340 | Cyfip2 | protein\_coding | 11:46193850-46312859 (-) |  | 1.0300 | 1.34e-30 | 9.27e-30 |
| ENSMUSG00000110644 | Gm7390 | processed\_pseudogene | 9:4977959-4979174 (+) |  | 1.0700 | 1.52e-30 | 1.05e-29 |
| ENSMUSG00000024941 | Scyl1 | protein\_coding | 19:5758351-5771419 (-) |  | -0.4660 | 1.58e-30 | 1.10e-29 |
| ENSMUSG00000028063 | Lmna | protein\_coding | 3:88480147-88509956 (-) |  | -2.4800 | 1.61e-30 | 1.11e-29 |
| ENSMUSG00000054150 | Syne3 | protein\_coding | 12:104929933-105009809 (-) |  | -0.6120 | 1.66e-30 | 1.15e-29 |
| ENSMUSG00000036639 | Nudt1 | protein\_coding | 5:140321656-140338137 (+) |  | 0.9190 | 1.68e-30 | 1.16e-29 |
| ENSMUSG00000101188 | Eif4a-ps4 | processed\_pseudogene | 1:60703932-60705149 (+) |  | 0.7380 | 1.71e-30 | 1.18e-29 |
| ENSMUSG00000014668 | Chfr | protein\_coding | 5:110135842-110171972 (+) |  | -0.5570 | 1.74e-30 | 1.20e-29 |
| ENSMUSG00000048924 | Ccdc125 | protein\_coding | 13:100669717-100697240 (+) |  | -0.7900 | 1.89e-30 | 1.30e-29 |
| ENSMUSG00000024082 | Ndufaf7 | protein\_coding | 17:78937136-78948052 (+) |  | -0.4970 | 1.93e-30 | 1.33e-29 |
| ENSMUSG00000002428 | Hltf | protein\_coding | 3:20057811-20118490 (+) |  | -0.6760 | 2.06e-30 | 1.42e-29 |
| ENSMUSG00000037126 | Psd | protein\_coding | 19:46312087-46327156 (-) |  | -1.4400 | 2.17e-30 | 1.49e-29 |
| ENSMUSG00000025613 | Cct8 | protein\_coding | 16:87483326-87495873 (-) |  | 0.7180 | 2.22e-30 | 1.53e-29 |
| ENSMUSG00000021773 | Comtd1 | protein\_coding | 14:21845855-21848977 (-) |  | 1.8100 | 2.22e-30 | 1.53e-29 |
| ENSMUSG00000020648 | Dus4l | protein\_coding | 12:31640050-31654826 (-) |  | 1.4800 | 2.26e-30 | 1.55e-29 |
| ENSMUSG00000112843 | Gm46224 | lncRNA | 10:51526340-51531161 (+) |  | -1.6100 | 2.76e-30 | 1.90e-29 |
| ENSMUSG00000020474 | Polm | protein\_coding | 11:5827860-5838016 (-) |  | -1.2600 | 2.78e-30 | 1.91e-29 |
| ENSMUSG00000018381 | Abi3 | protein\_coding | 11:95830074-95842476 (-) |  | -1.8200 | 2.83e-30 | 1.94e-29 |
| ENSMUSG00000000085 | Scmh1 | protein\_coding | 4:120405281-120530186 (+) |  | 0.8490 | 2.88e-30 | 1.98e-29 |
| ENSMUSG00000045098 | Kmt5b | protein\_coding | 19:3767421-3818303 (+) |  | -0.5890 | 2.92e-30 | 2.00e-29 |
| ENSMUSG00000043131 | Mob1a | protein\_coding | 6:83326016-83343776 (+) |  | -0.6780 | 2.93e-30 | 2.01e-29 |
| ENSMUSG00000009575 | Cbx5 | protein\_coding | 15:103191544-103239816 (-) |  | 0.8630 | 2.99e-30 | 2.05e-29 |
| ENSMUSG00000015839 | Nfe2l2 | protein\_coding | 2:75675513-75704641 (-) |  | -0.5570 | 3.24e-30 | 2.22e-29 |
| ENSMUSG00000078942 | Naip6 | protein\_coding | 13:100281121-100317674 (-) |  | -1.0300 | 3.36e-30 | 2.30e-29 |
| ENSMUSG00000024940 | Ltbp3 | protein\_coding | 19:5740904-5758532 (+) |  | 2.3000 | 3.52e-30 | 2.41e-29 |
| ENSMUSG00000037315 | Jade3 | protein\_coding | X:20425688-20519939 (+) |  | 1.0900 | 3.62e-30 | 2.47e-29 |
| ENSMUSG00000073982 | Rhog | protein\_coding | 7:102239123-102256054 (-) |  | -0.5160 | 3.69e-30 | 2.52e-29 |
| ENSMUSG00000029147 | Ppm1g | protein\_coding | 5:31202664-31220687 (-) |  | 0.5790 | 3.79e-30 | 2.59e-29 |
| ENSMUSG00000027774 | Gfm1 | protein\_coding | 3:67430096-67476529 (+) |  | 0.7200 | 3.93e-30 | 2.69e-29 |
| ENSMUSG00000020994 | Pnn | protein\_coding | 12:59066884-59073998 (+) |  | 0.5560 | 3.99e-30 | 2.73e-29 |
| ENSMUSG00000020781 | Tsen54 | protein\_coding | 11:115814724-115823094 (+) |  | 0.7670 | 4.11e-30 | 2.81e-29 |
| ENSMUSG00000018417 | Myo1b | protein\_coding | 1:51749765-51916071 (-) |  | 3.1800 | 4.22e-30 | 2.88e-29 |
| ENSMUSG00000028973 | Abcb8 | protein\_coding | 5:24393663-24410054 (+) |  | 0.8810 | 4.26e-30 | 2.91e-29 |
| ENSMUSG00000049957 | Ccdc137 | protein\_coding | 11:120458115-120464358 (+) |  | 0.8230 | 4.31e-30 | 2.94e-29 |
| ENSMUSG00000031644 | Nek1 | protein\_coding | 8:60993195-61131346 (+) |  | 1.1100 | 4.33e-30 | 2.95e-29 |
| ENSMUSG00000025578 | Cbx8 | protein\_coding | 11:119036305-119040969 (-) |  | -0.7530 | 4.42e-30 | 3.01e-29 |
| ENSMUSG00000028189 | Ctbs | protein\_coding | 3:146449795-146465849 (+) |  | -0.8150 | 4.49e-30 | 3.06e-29 |
| ENSMUSG00000073725 | Lmbrd1 | protein\_coding | 1:24678630-24766301 (+) |  | -0.7890 | 4.53e-30 | 3.08e-29 |
| ENSMUSG00000117975 | Itprip | protein\_coding | 19:47894602-47919299 (-) |  | -0.5260 | 4.75e-30 | 3.23e-29 |
| ENSMUSG00000030474 | Siglece | protein\_coding | 7:43651070-43660161 (-) |  | -0.7610 | 5.07e-30 | 3.45e-29 |
| ENSMUSG00000032939 | Nup93 | protein\_coding | 8:94214564-94317227 (+) |  | 0.6790 | 5.20e-30 | 3.53e-29 |
| ENSMUSG00000019301 | Hsd17b1 | protein\_coding | 11:101078411-101080527 (+) |  | 3.6300 | 5.36e-30 | 3.64e-29 |
| ENSMUSG00000021996 | Esd | protein\_coding | 14:74732297-74750765 (+) |  | -0.7300 | 5.70e-30 | 3.87e-29 |
| ENSMUSG00000022682 | Rrn3 | protein\_coding | 16:13780708-13814839 (+) |  | 0.5620 | 5.79e-30 | 3.93e-29 |
| ENSMUSG00000035199 | Arl6ip5 | protein\_coding | 6:97210689-97233315 (+) |  | -0.5620 | 5.99e-30 | 4.06e-29 |
| ENSMUSG00000034998 | Foxn2 | protein\_coding | 17:88440711-88490533 (+) |  | -0.5030 | 6.07e-30 | 4.12e-29 |
| ENSMUSG00000021624 | Cd180 | protein\_coding | 13:102693558-102739629 (+) |  | -1.0900 | 6.59e-30 | 4.46e-29 |
| ENSMUSG00000020961 | Ston2 | protein\_coding | 12:91633009-91788387 (-) |  | 1.6400 | 6.82e-30 | 4.62e-29 |
| ENSMUSG00000050088 | 1600012H06Rik | protein\_coding | 17:14943184-14959570 (+) |  | -0.8490 | 6.93e-30 | 4.69e-29 |
| ENSMUSG00000001569 | Nom1 | protein\_coding | 5:29434664-29457843 (+) |  | 0.4910 | 7.80e-30 | 5.28e-29 |
| ENSMUSG00000111118 | Gm6545 | processed\_pseudogene | 19:12528755-12530540 (-) |  | -2.0100 | 8.78e-30 | 5.94e-29 |
| ENSMUSG00000052798 | Nup107 | protein\_coding | 10:117750621-117792705 (-) |  | 0.5700 | 8.98e-30 | 6.07e-29 |
| ENSMUSG00000046062 | Ppp1r15b | protein\_coding | 1:133131143-133139783 (+) |  | -0.5460 | 9.12e-30 | 6.16e-29 |
| ENSMUSG00000071847 | Apcdd1 | protein\_coding | 18:62922231-62961179 (+) |  | -1.3100 | 9.17e-30 | 6.20e-29 |
| ENSMUSG00000033423 | Eri3 | protein\_coding | 4:117550365-117674297 (+) |  | 0.6700 | 9.25e-30 | 6.24e-29 |
| ENSMUSG00000102644 | Thap6 | transcribed\_unitary\_pseudogene | 5:91962389-91972066 (+) |  | -0.8140 | 9.49e-30 | 6.40e-29 |
| ENSMUSG00000032489 | Kif9 | protein\_coding | 9:110476958-110525179 (+) |  | -1.8400 | 1.00e-29 | 6.74e-29 |
| ENSMUSG00000027665 | Pik3ca | protein\_coding | 3:32397671-32468486 (+) |  | -0.4590 | 1.01e-29 | 6.81e-29 |
| ENSMUSG00000022253 | Nadk2 | protein\_coding | 15:9071260-9110891 (+) |  | 0.5810 | 1.02e-29 | 6.90e-29 |
| ENSMUSG00000002825 | Qtrt1 | protein\_coding | 9:21411837-21420274 (+) |  | 1.0600 | 1.08e-29 | 7.24e-29 |
| ENSMUSG00000111877 | Gm6477 | processed\_pseudogene | 10:39198538-39199301 (+) |  | 1.3900 | 1.09e-29 | 7.35e-29 |
| ENSMUSG00000036442 | Thap11 | protein\_coding | 8:105855132-105856950 (+) |  | -0.5950 | 1.13e-29 | 7.62e-29 |
| ENSMUSG00000020380 | Rad50 | protein\_coding | 11:53649519-53707319 (-) |  | 0.7950 | 1.19e-29 | 8.02e-29 |
| ENSMUSG00000024104 | Washc2 | protein\_coding | 6:116208038-116262686 (+) |  | -0.6390 | 1.21e-29 | 8.17e-29 |
| ENSMUSG00000046432 | Bex3 | protein\_coding | X:136270253-136271978 (+) |  | 1.1500 | 1.22e-29 | 8.17e-29 |
| ENSMUSG00000029106 | Add1 | protein\_coding | 5:34573664-34632308 (+) |  | -0.5050 | 1.23e-29 | 8.26e-29 |
| ENSMUSG00000031304 | Il2rg | protein\_coding | X:101264378-101268255 (-) |  | -1.1700 | 1.25e-29 | 8.39e-29 |
| ENSMUSG00000032478 | Nme6 | protein\_coding | 9:109832749-109843116 (+) |  | 1.3600 | 1.26e-29 | 8.47e-29 |
| ENSMUSG00000037331 | Larp1 | protein\_coding | 11:58009064-58062034 (+) |  | 0.6760 | 1.28e-29 | 8.60e-29 |
| ENSMUSG00000073492 | Gm10521 | protein\_coding | 1:171895664-171898237 (+) |  | -2.3600 | 1.30e-29 | 8.72e-29 |
| ENSMUSG00000115869 | Gm31814 | lncRNA | 16:24186029-24263159 (-) |  | -2.2500 | 1.36e-29 | 9.14e-29 |
| ENSMUSG00000021254 | Gpatch2l | protein\_coding | 12:86241858-86291784 (+) |  | -0.6180 | 1.38e-29 | 9.25e-29 |
| ENSMUSG00000030946 | Lhpp | protein\_coding | 7:132610638-132706420 (+) |  | 1.0700 | 1.38e-29 | 9.25e-29 |
| ENSMUSG00000052331 | Ankrd44 | protein\_coding | 1:54645340-54926387 (-) |  | -0.6740 | 1.51e-29 | 1.01e-28 |
| ENSMUSG00000018697 | Aatf | protein\_coding | 11:84422855-84513522 (-) |  | 0.7780 | 1.64e-29 | 1.09e-28 |
| ENSMUSG00000026932 | Nacc2 | protein\_coding | 2:26055535-26123220 (-) |  | -1.3600 | 1.72e-29 | 1.15e-28 |
| ENSMUSG00000028980 | H6pd | protein\_coding | 4:149979475-150009023 (-) |  | -0.5180 | 1.80e-29 | 1.20e-28 |
| ENSMUSG00000024193 | Phf1 | protein\_coding | 17:26933052-26937908 (+) |  | -0.9470 | 1.88e-29 | 1.25e-28 |
| ENSMUSG00000041598 | Cdc42ep4 | protein\_coding | 11:113726850-113751881 (-) |  | -0.9950 | 1.89e-29 | 1.26e-28 |
| ENSMUSG00000034165 | Ccnd3 | protein\_coding | 17:47505051-47599691 (+) |  | -0.6120 | 1.95e-29 | 1.30e-28 |
| ENSMUSG00000021548 | Ccnh | protein\_coding | 13:85189408-85223469 (+) |  | 0.6150 | 1.98e-29 | 1.32e-28 |
| ENSMUSG00000028393 | Alad | protein\_coding | 4:62509169-62519918 (-) |  | 1.3700 | 2.08e-29 | 1.39e-28 |
| ENSMUSG00000034908 | Sidt2 | protein\_coding | 9:45937857-45955258 (-) |  | -0.7690 | 2.10e-29 | 1.40e-28 |
| ENSMUSG00000031367 | Ap1s2 | protein\_coding | X:163909017-163933666 (+) |  | -0.9830 | 2.11e-29 | 1.41e-28 |
| ENSMUSG00000057667 | Bloc1s3 | protein\_coding | 7:19504486-19508367 (-) |  | -0.9570 | 2.13e-29 | 1.42e-28 |
| ENSMUSG00000057375 | Yipf1 | protein\_coding | 4:107314363-107359823 (+) |  | -0.5940 | 2.17e-29 | 1.44e-28 |
| ENSMUSG00000022257 | Laptm4b | protein\_coding | 15:34238028-34284302 (+) |  | 1.5300 | 2.22e-29 | 1.48e-28 |
| ENSMUSG00000028093 | Acp6 | protein\_coding | 3:97158777-97177299 (+) |  | 0.8450 | 2.28e-29 | 1.51e-28 |
| ENSMUSG00000020393 | Kremen1 | protein\_coding | 11:5191552-5261558 (-) |  | -0.7590 | 2.38e-29 | 1.58e-28 |
| ENSMUSG00000030811 | Fbxl19 | protein\_coding | 7:127744543-127769483 (+) |  | 0.9410 | 2.41e-29 | 1.60e-28 |
| ENSMUSG00000031949 | Adat1 | protein\_coding | 8:111966908-111992302 (-) |  | 1.5400 | 2.44e-29 | 1.62e-28 |
| ENSMUSG00000020458 | Rtn4 | protein\_coding | 11:29692947-29744331 (+) |  | -0.7050 | 2.46e-29 | 1.64e-28 |
| ENSMUSG00000005410 | Mcm5 | protein\_coding | 8:75109569-75128439 (+) |  | 0.8520 | 2.49e-29 | 1.65e-28 |
| ENSMUSG00000029186 | Pi4k2b | protein\_coding | 5:52741574-52769340 (+) |  | 0.9180 | 2.53e-29 | 1.68e-28 |
| ENSMUSG00000073838 | Tufm | protein\_coding | 7:126487361-126490731 (+) |  | 0.7260 | 2.61e-29 | 1.73e-28 |
| ENSMUSG00000074886 | Grk6 | protein\_coding | 13:55445072-55460927 (+) |  | -0.5650 | 2.61e-29 | 1.73e-28 |
| ENSMUSG00000046157 | Tmem229b | protein\_coding | 12:78961795-79007627 (-) |  | 0.8450 | 2.67e-29 | 1.77e-28 |
| ENSMUSG00000027550 | Lrrcc1 | protein\_coding | 3:14533788-14572658 (+) |  | 0.6550 | 2.90e-29 | 1.92e-28 |
| ENSMUSG00000028676 | Srsf10 | protein\_coding | 4:135855747-135869908 (+) |  | 0.5560 | 3.09e-29 | 2.05e-28 |
| ENSMUSG00000098234 | Snhg6 | lncRNA | 1:9941959-9944118 (-) |  | 1.2100 | 3.11e-29 | 2.06e-28 |
| ENSMUSG00000026082 | Rev1 | protein\_coding | 1:38052786-38129801 (-) |  | 0.6610 | 3.29e-29 | 2.18e-28 |
| ENSMUSG00000047731 | Wbp1l | protein\_coding | 19:46599084-46657389 (+) |  | -0.4810 | 3.83e-29 | 2.53e-28 |
| ENSMUSG00000025907 | Rb1cc1 | protein\_coding | 1:6206197-6276648 (+) |  | -0.6630 | 3.88e-29 | 2.56e-28 |
| ENSMUSG00000024120 | Lrpprc | protein\_coding | 17:84705247-84790789 (-) |  | 0.7420 | 3.95e-29 | 2.61e-28 |
| ENSMUSG00000037683 | Armc3 | protein\_coding | 2:19199302-19310241 (+) |  | -3.1900 | 4.11e-29 | 2.71e-28 |
| ENSMUSG00000063568 | Jazf1 | protein\_coding | 6:52768797-53068631 (-) |  | -2.5100 | 4.41e-29 | 2.91e-28 |
| ENSMUSG00000026248 | Mrpl44 | protein\_coding | 1:79776018-79781445 (+) |  | 0.6880 | 4.46e-29 | 2.95e-28 |
| ENSMUSG00000032399 | Rpl4 | protein\_coding | 9:64173375-64178666 (+) |  | 0.4910 | 4.93e-29 | 3.25e-28 |
| ENSMUSG00000047139 | Cd24a | protein\_coding | 10:43578284-43584265 (+) |  | 0.5040 | 4.95e-29 | 3.26e-28 |
| ENSMUSG00000034708 | Grn | protein\_coding | 11:102430315-102437048 (+) |  | -0.9110 | 5.01e-29 | 3.30e-28 |
| ENSMUSG00000042628 | Zfyve1 | protein\_coding | 12:83546558-83597222 (-) |  | -0.7700 | 5.30e-29 | 3.49e-28 |
| ENSMUSG00000079555 | Haus3 | protein\_coding | 5:34153880-34169527 (-) |  | 0.7580 | 5.34e-29 | 3.51e-28 |
| ENSMUSG00000025155 | Dus1l | protein\_coding | 11:120789201-120796403 (-) |  | 0.4390 | 5.53e-29 | 3.64e-28 |
| ENSMUSG00000058728 | Cd300c | protein\_coding | 11:114956116-114969157 (-) |  | 3.6300 | 5.58e-29 | 3.67e-28 |
| ENSMUSG00000027327 | 1700037H04Rik | protein\_coding | 2:131146324-131160081 (-) |  | 0.9750 | 5.78e-29 | 3.80e-28 |
| ENSMUSG00000038545 | Cul7 | protein\_coding | 17:46650337-46664364 (+) |  | 1.5700 | 6.60e-29 | 4.34e-28 |
| ENSMUSG00000000318 | Clec10a | protein\_coding | 11:70156197-70170834 (+) |  | -0.8700 | 6.71e-29 | 4.41e-28 |
| ENSMUSG00000068335 | Dok1 | protein\_coding | 6:83030934-83033471 (-) |  | -0.8680 | 6.79e-29 | 4.46e-28 |
| ENSMUSG00000001783 | Rtcb | protein\_coding | 10:85938637-85957823 (-) |  | 0.5950 | 6.80e-29 | 4.46e-28 |
| ENSMUSG00000030291 | Med21 | protein\_coding | 6:146642547-146650732 (+) |  | 1.0700 | 6.89e-29 | 4.52e-28 |
| ENSMUSG00000023952 | Gtpbp2 | protein\_coding | 17:46161032-46169370 (+) |  | -0.7260 | 6.98e-29 | 4.58e-28 |
| ENSMUSG00000034427 | Myo15b | protein\_coding | 11:115858406-115892603 (+) |  | 3.0900 | 7.07e-29 | 4.63e-28 |
| ENSMUSG00000025875 | Tspan17 | protein\_coding | 13:54789377-54796776 (+) |  | -1.2900 | 7.19e-29 | 4.71e-28 |
| ENSMUSG00000029916 | Agk | protein\_coding | 6:40325172-40396762 (+) |  | 1.1300 | 7.26e-29 | 4.75e-28 |
| ENSMUSG00000004936 | Map2k1 | protein\_coding | 9:64185770-64253631 (-) |  | -0.5490 | 7.48e-29 | 4.90e-28 |
| ENSMUSG00000035011 | Zbtb7a | protein\_coding | 10:81135220-81152995 (+) |  | -0.4750 | 7.71e-29 | 5.04e-28 |
| ENSMUSG00000060216 | Arrb2 | protein\_coding | 11:70432635-70440828 (+) |  | -0.6170 | 7.81e-29 | 5.11e-28 |
| ENSMUSG00000005034 | Prkacb | protein\_coding | 3:146729574-146812990 (-) |  | 0.5160 | 8.34e-29 | 5.45e-28 |
| ENSMUSG00000031447 | Lamp1 | protein\_coding | 8:13159161-13175338 (+) |  | -0.5890 | 8.36e-29 | 5.46e-28 |
| ENSMUSG00000074158 | Zfp976 | protein\_coding | 7:42609526-42642588 (-) |  | 2.6400 | 8.71e-29 | 5.69e-28 |
| ENSMUSG00000015944 | Castor2 | protein\_coding | 5:134099711-134144343 (+) |  | -1.0400 | 9.02e-29 | 5.89e-28 |
| ENSMUSG00000045763 | Basp1 | protein\_coding | 15:25363277-25413764 (-) |  | 2.6600 | 9.22e-29 | 6.01e-28 |
| ENSMUSG00000027997 | Casp6 | protein\_coding | 3:129901425-129914103 (+) |  | -1.1000 | 9.54e-29 | 6.22e-28 |
| ENSMUSG00000037419 | Endod1 | protein\_coding | 9:14353990-14381507 (-) |  | 0.7230 | 9.72e-29 | 6.34e-28 |
| ENSMUSG00000025395 | Prim1 | protein\_coding | 10:128015168-128030037 (+) |  | 0.9250 | 1.01e-28 | 6.56e-28 |
| ENSMUSG00000027951 | Adar | protein\_coding | 3:89715022-89753446 (+) |  | -0.6590 | 1.08e-28 | 7.02e-28 |
| ENSMUSG00000024927 | Rela | protein\_coding | 19:5637483-5648130 (+) |  | -0.4670 | 1.15e-28 | 7.48e-28 |
| ENSMUSG00000000732 | Icosl | protein\_coding | 10:78069302-78083913 (+) |  | -0.8750 | 1.15e-28 | 7.51e-28 |
| ENSMUSG00000010095 | Slc3a2 | protein\_coding | 19:8706882-8723369 (-) |  | 0.7770 | 1.20e-28 | 7.83e-28 |
| ENSMUSG00000000359 | Rem1 | protein\_coding | 2:152626951-152635198 (+) |  | -1.5100 | 1.23e-28 | 7.99e-28 |
| ENSMUSG00000022940 | Pigp | protein\_coding | 16:94358763-94371842 (-) |  | 1.7300 | 1.24e-28 | 8.07e-28 |
| ENSMUSG00000042626 | Shc1 | protein\_coding | 3:89418443-89430027 (+) |  | -0.5100 | 1.31e-28 | 8.54e-28 |
| ENSMUSG00000004661 | Arid3b | protein\_coding | 9:57790353-57836793 (-) |  | -1.1200 | 1.35e-28 | 8.76e-28 |
| ENSMUSG00000022394 | L3mbtl2 | protein\_coding | 15:81663889-81688315 (+) |  | 0.6330 | 1.36e-28 | 8.80e-28 |
| ENSMUSG00000028420 | Tmem38b | protein\_coding | 4:53826045-53862019 (+) |  | -0.8660 | 1.45e-28 | 9.38e-28 |
| ENSMUSG00000020536 | Llgl1 | protein\_coding | 11:60699723-60714186 (+) |  | -0.5720 | 1.48e-28 | 9.61e-28 |
| ENSMUSG00000046994 | Mars2 | protein\_coding | 1:55237177-55248470 (+) |  | 0.7100 | 1.48e-28 | 9.62e-28 |
| ENSMUSG00000027984 | Hadh | protein\_coding | 3:131233419-131272101 (-) |  | 0.6130 | 1.52e-28 | 9.85e-28 |
| ENSMUSG00000015966 | Il17rb | protein\_coding | 14:29996135-30008896 (-) |  | 2.9000 | 1.63e-28 | 1.06e-27 |
| ENSMUSG00000037656 | Slc20a2 | protein\_coding | 8:22476788-22569612 (+) |  | 0.8630 | 1.68e-28 | 1.09e-27 |
| ENSMUSG00000031303 | Map3k15 | protein\_coding | X:159988433-160123351 (+) |  | -1.0700 | 1.72e-28 | 1.12e-27 |
| ENSMUSG00000035596 | Mboat7 | protein\_coding | 7:3677789-3693523 (-) |  | -0.5000 | 1.73e-28 | 1.12e-27 |
| ENSMUSG00000021952 | Xpo4 | protein\_coding | 14:57577521-57665430 (-) |  | 0.6700 | 1.73e-28 | 1.12e-27 |
| ENSMUSG00000021131 | Erh | protein\_coding | 12:80634022-80644341 (-) |  | 0.9030 | 1.76e-28 | 1.14e-27 |
| ENSMUSG00000028041 | Adam15 | protein\_coding | 3:89338542-89349996 (-) |  | -0.5540 | 1.78e-28 | 1.15e-27 |
| ENSMUSG00000045639 | Zfp629 | protein\_coding | 7:127607031-127615797 (-) |  | 0.6850 | 1.86e-28 | 1.20e-27 |
| ENSMUSG00000076435 | Acsf2 | protein\_coding | 11:94557102-94601871 (-) |  | 1.3200 | 1.92e-28 | 1.24e-27 |
| ENSMUSG00000115026 | Gm49041 | lncRNA | 6:39207068-39207611 (+) |  | -2.1300 | 1.94e-28 | 1.25e-27 |
| ENSMUSG00000032507 | Fbxl2 | protein\_coding | 9:113963637-114046191 (-) |  | -1.0900 | 1.95e-28 | 1.26e-27 |
| ENSMUSG00000058388 | Phtf1 | protein\_coding | 3:103968110-104024598 (+) |  | 0.6160 | 2.15e-28 | 1.39e-27 |
| ENSMUSG00000036504 | Phpt1 | protein\_coding | 2:25573430-25575224 (-) |  | 1.4800 | 2.20e-28 | 1.42e-27 |
| ENSMUSG00000035397 | Klf16 | protein\_coding | 10:80567124-80577321 (-) |  | 0.7950 | 2.28e-28 | 1.47e-27 |
| ENSMUSG00000014846 | Tppp3 | protein\_coding | 8:105467493-105471526 (-) |  | -2.6100 | 2.29e-28 | 1.47e-27 |
| ENSMUSG00000058135 | Gstm1 | protein\_coding | 3:108012255-108017973 (-) |  | 0.7310 | 2.32e-28 | 1.49e-27 |
| ENSMUSG00000020520 | Galnt10 | protein\_coding | 11:57645442-57787514 (+) |  | -0.7430 | 2.34e-28 | 1.51e-27 |
| ENSMUSG00000028080 | Lrba | protein\_coding | 3:86224680-86782692 (+) |  | 0.9280 | 2.41e-28 | 1.55e-27 |
| ENSMUSG00000022673 | Mcm4 | protein\_coding | 16:15623897-15637400 (-) |  | 0.9970 | 2.41e-28 | 1.55e-27 |
| ENSMUSG00000028581 | Laptm5 | protein\_coding | 4:130913125-130936141 (+) |  | -0.6190 | 2.47e-28 | 1.59e-27 |
| ENSMUSG00000015214 | Mtmr1 | protein\_coding | X:71364760-71419196 (+) |  | 0.5440 | 2.49e-28 | 1.60e-27 |
| ENSMUSG00000020709 | Adap2 | protein\_coding | 11:80154105-80178958 (+) |  | -1.8800 | 2.53e-28 | 1.62e-27 |
| ENSMUSG00000039067 | Psmd7 | protein\_coding | 8:107580381-107588464 (-) |  | 0.6390 | 2.59e-28 | 1.66e-27 |
| ENSMUSG00000026972 | Arrdc1 | protein\_coding | 2:24925352-24935252 (-) |  | -0.6370 | 2.63e-28 | 1.69e-27 |
| ENSMUSG00000037235 | Mxd4 | protein\_coding | 5:34173883-34187720 (-) |  | -0.9020 | 2.71e-28 | 1.74e-27 |
| ENSMUSG00000029913 | Prdm5 | protein\_coding | 6:65778988-65937010 (+) |  | 3.8800 | 2.79e-28 | 1.79e-27 |
| ENSMUSG00000028528 | Dnajc6 | protein\_coding | 4:101496631-101642799 (+) |  | 4.7700 | 2.80e-28 | 1.80e-27 |
| ENSMUSG00000038212 | Mfsd14b | protein\_coding | 13:65064663-65112975 (-) |  | -0.7760 | 2.97e-28 | 1.90e-27 |
| ENSMUSG00000024790 | Sac3d1 | protein\_coding | 19:6116000-6118650 (-) |  | 1.0200 | 3.31e-28 | 2.12e-27 |
| ENSMUSG00000037921 | Ddx60 | protein\_coding | 8:61928087-62038244 (+) |  | -2.2200 | 3.39e-28 | 2.17e-27 |
| ENSMUSG00000025436 | Atp23 | protein\_coding | 10:126868427-126901355 (-) |  | 1.3000 | 3.53e-28 | 2.26e-27 |
| ENSMUSG00000006315 | Tmem147 | protein\_coding | 7:30727701-30729540 (-) |  | 0.8210 | 3.61e-28 | 2.31e-27 |
| ENSMUSG00000032898 | Fbxo21 | protein\_coding | 5:117976730-118010201 (+) |  | 1.0800 | 3.65e-28 | 2.33e-27 |
| ENSMUSG00000032480 | Dhx30 | protein\_coding | 9:110084320-110117830 (-) |  | 0.5020 | 3.68e-28 | 2.35e-27 |
| ENSMUSG00000097101 | 1810034E14Rik | lncRNA | 13:64248700-64271852 (+) |  | -1.4000 | 3.77e-28 | 2.41e-27 |
| ENSMUSG00000030086 | Chchd6 | protein\_coding | 6:89383146-89595652 (-) |  | 1.3700 | 3.78e-28 | 2.41e-27 |
| ENSMUSG00000000441 | Raf1 | protein\_coding | 6:115618067-115676635 (-) |  | -0.4030 | 3.85e-28 | 2.46e-27 |
| ENSMUSG00000050350 | Gpr18 | protein\_coding | 14:121911253-121915781 (-) |  | -2.1000 | 3.89e-28 | 2.48e-27 |
| ENSMUSG00000021097 | Clmn | protein\_coding | 12:104763117-104865076 (-) |  | -2.2500 | 3.94e-28 | 2.51e-27 |
| ENSMUSG00000029385 | Ccng2 | protein\_coding | 5:93267257-93276231 (+) |  | -0.8180 | 3.99e-28 | 2.54e-27 |
| ENSMUSG00000021540 | Smad5 | protein\_coding | 13:56703010-56742377 (+) |  | 0.6800 | 3.99e-28 | 2.54e-27 |
| ENSMUSG00000039509 | Nup133 | protein\_coding | 8:123897123-123949265 (-) |  | 0.6670 | 4.00e-28 | 2.55e-27 |
| ENSMUSG00000021140 | Pcnx | protein\_coding | 12:81860023-82000924 (+) |  | -0.8170 | 4.04e-28 | 2.57e-27 |
| ENSMUSG00000053617 | Sh3pxd2a | protein\_coding | 19:47260174-47464401 (-) |  | 1.4800 | 4.06e-28 | 2.58e-27 |
| ENSMUSG00000025477 | Inpp5a | protein\_coding | 7:139389109-139579652 (+) |  | -0.8010 | 4.08e-28 | 2.60e-27 |
| ENSMUSG00000033488 | Cryzl2 | protein\_coding | 1:157458577-157492638 (+) |  | 1.2300 | 4.26e-28 | 2.70e-27 |
| ENSMUSG00000032407 | U2surp | protein\_coding | 9:95456898-95511996 (-) |  | 0.5060 | 4.33e-28 | 2.75e-27 |
| ENSMUSG00000051335 | Gfod1 | protein\_coding | 13:43195245-43304172 (-) |  | 0.9170 | 4.37e-28 | 2.78e-27 |
| ENSMUSG00000056692 | Ilrun | protein\_coding | 17:27751235-27820648 (-) |  | -0.4060 | 4.61e-28 | 2.93e-27 |
| ENSMUSG00000016181 | Utp25 | protein\_coding | 1:193091104-193130272 (-) |  | 0.8480 | 4.71e-28 | 2.99e-27 |
| ENSMUSG00000046434 | Hnrnpa1 | protein\_coding | 15:103240432-103246692 (+) |  | 0.7520 | 4.93e-28 | 3.13e-27 |
| ENSMUSG00000034833 | Tespa1 | protein\_coding | 10:130322870-130364111 (+) |  | 1.7200 | 5.00e-28 | 3.17e-27 |
| ENSMUSG00000052270 | Fpr2 | protein\_coding | 17:17887824-17893952 (+) |  | -3.5700 | 5.04e-28 | 3.20e-27 |
| ENSMUSG00000018677 | Slc25a39 | protein\_coding | 11:102402985-102407946 (-) |  | 0.5740 | 5.33e-28 | 3.38e-27 |
| ENSMUSG00000049775 | Tmsb4x | protein\_coding | X:167207093-167209315 (-) |  | -1.1200 | 5.41e-28 | 3.42e-27 |
| ENSMUSG00000028207 | Asph | protein\_coding | 4:9448069-9669344 (-) |  | -0.8950 | 5.45e-28 | 3.45e-27 |
| ENSMUSG00000071547 | Nt5dc2 | protein\_coding | 14:31131053-31139124 (+) |  | 1.1900 | 5.45e-28 | 3.45e-27 |
| ENSMUSG00000022685 | Parn | protein\_coding | 16:13537960-13668170 (-) |  | 0.7190 | 5.60e-28 | 3.54e-27 |
| ENSMUSG00000041025 | Iffo2 | protein\_coding | 4:139530548-139620382 (+) |  | -1.1700 | 5.63e-28 | 3.56e-27 |
| ENSMUSG00000026031 | Cflar | protein\_coding | 1:58711508-58758884 (+) |  | -0.5850 | 5.76e-28 | 3.64e-27 |
| ENSMUSG00000030629 | Zfand6 | protein\_coding | 7:84613766-84689959 (-) |  | -0.6740 | 5.98e-28 | 3.78e-27 |
| ENSMUSG00000035356 | Nfkbiz | protein\_coding | 16:55811375-55838899 (-) |  | -1.5700 | 6.18e-28 | 3.90e-27 |
| ENSMUSG00000068245 | Phf11d | protein\_coding | 14:59347407-59365470 (-) |  | -2.6400 | 6.34e-28 | 4.00e-27 |
| ENSMUSG00000006800 | Sulf2 | protein\_coding | 2:166073089-166155663 (-) |  | -1.7000 | 6.56e-28 | 4.14e-27 |
| ENSMUSG00000041000 | Trim62 | protein\_coding | 4:128883580-128911328 (+) |  | -1.6000 | 7.19e-28 | 4.53e-27 |
| ENSMUSG00000024063 | Lbh | protein\_coding | 17:72918305-72941947 (+) |  | -0.7170 | 7.35e-28 | 4.63e-27 |
| ENSMUSG00000074088 | Snrnp40 | protein\_coding | 4:130360132-130390026 (+) |  | 0.4330 | 7.61e-28 | 4.79e-27 |
| ENSMUSG00000036875 | Dna2 | protein\_coding | 10:62947026-62974185 (+) |  | -0.6220 | 7.63e-28 | 4.80e-27 |
| ENSMUSG00000023845 | Lnpep | protein\_coding | 17:17521410-17625050 (-) |  | -0.6560 | 7.75e-28 | 4.88e-27 |
| ENSMUSG00000044018 | Mrpl50 | protein\_coding | 4:49512596-49521093 (-) |  | 0.6860 | 7.89e-28 | 4.96e-27 |
| ENSMUSG00000038286 | Bphl | protein\_coding | 13:34037597-34074074 (+) |  | 0.8600 | 8.31e-28 | 5.22e-27 |
| ENSMUSG00000090626 | Tex9 | protein\_coding | 9:72450394-72492212 (-) |  | 1.9600 | 8.41e-28 | 5.29e-27 |
| ENSMUSG00000060538 | Tmem219 | protein\_coding | 7:126886171-126922917 (-) |  | -0.8680 | 8.45e-28 | 5.31e-27 |
| ENSMUSG00000027999 | Pla2g12a | protein\_coding | 3:129878606-129895825 (+) |  | 1.6600 | 8.46e-28 | 5.31e-27 |
| ENSMUSG00000005982 | Naa60 | protein\_coding | 16:3872375-3904770 (+) |  | -0.4710 | 8.61e-28 | 5.40e-27 |
| ENSMUSG00000024797 | Vps51 | protein\_coding | 19:6067842-6077231 (-) |  | -0.5780 | 8.82e-28 | 5.53e-27 |
| ENSMUSG00000057406 | Nsd2 | protein\_coding | 5:33820725-33897975 (+) |  | 0.6730 | 8.91e-28 | 5.59e-27 |
| ENSMUSG00000034709 | Ppp1r21 | protein\_coding | 17:88530118-88588367 (+) |  | -0.5510 | 8.92e-28 | 5.59e-27 |
| ENSMUSG00000034218 | Atm | protein\_coding | 9:53439149-53536740 (-) |  | 0.8720 | 9.36e-28 | 5.87e-27 |
| ENSMUSG00000030528 | Blm | protein\_coding | 7:80454733-80535119 (-) |  | 0.6550 | 9.87e-28 | 6.18e-27 |
| ENSMUSG00000063160 | Numbl | protein\_coding | 7:27258433-27282144 (+) |  | -1.2300 | 9.92e-28 | 6.21e-27 |
| ENSMUSG00000033732 | Sf3b3 | protein\_coding | 8:110810239-110846787 (-) |  | 0.7760 | 1.00e-27 | 6.26e-27 |
| ENSMUSG00000021947 | Cryl1 | protein\_coding | 14:57274993-57398529 (-) |  | -0.8000 | 1.00e-27 | 6.27e-27 |
| ENSMUSG00000032376 | Usp3 | protein\_coding | 9:66514637-66593142 (-) |  | -0.5910 | 1.05e-27 | 6.54e-27 |
| ENSMUSG00000039680 | Mrps6 | protein\_coding | 16:92058270-92112227 (+) |  | 1.3800 | 1.08e-27 | 6.76e-27 |
| ENSMUSG00000029330 | Cds1 | protein\_coding | 5:101765130-101823858 (+) |  | 0.8050 | 1.08e-27 | 6.76e-27 |
| ENSMUSG00000021831 | Ero1l | protein\_coding | 14:45283087-45318771 (-) |  | 0.7150 | 1.09e-27 | 6.81e-27 |
| ENSMUSG00000030714 | Sgf29 | protein\_coding | 7:126649309-126672925 (+) |  | 0.7290 | 1.09e-27 | 6.83e-27 |
| ENSMUSG00000058794 | Nfe2 | protein\_coding | 15:103248212-103258403 (-) |  | -0.6210 | 1.12e-27 | 7.01e-27 |
| ENSMUSG00000054792 | Klhl18 | protein\_coding | 9:110425926-110476694 (-) |  | -0.4920 | 1.33e-27 | 8.30e-27 |
| ENSMUSG00000030336 | Cd27 | protein\_coding | 6:125232622-125237010 (-) |  | 2.4000 | 1.34e-27 | 8.34e-27 |
| ENSMUSG00000029191 | Rfc1 | protein\_coding | 5:65261850-65335670 (-) |  | 0.5860 | 1.35e-27 | 8.43e-27 |
| ENSMUSG00000026049 | Tex30 | protein\_coding | 1:44086613-44102441 (-) |  | 0.9160 | 1.36e-27 | 8.45e-27 |
| ENSMUSG00000069743 | Zfp820 | protein\_coding | 17:21816885-21845772 (-) |  | 2.5800 | 1.38e-27 | 8.62e-27 |
| ENSMUSG00000021908 | Gm6768 | processed\_pseudogene | 12:119260978-119262855 (+) |  | 0.9090 | 1.41e-27 | 8.78e-27 |
| ENSMUSG00000004748 | Mtfp1 | protein\_coding | 11:4091480-4095445 (-) |  | 1.9200 | 1.41e-27 | 8.78e-27 |
| ENSMUSG00000021814 | Anxa7 | protein\_coding | 14:20455260-20480133 (-) |  | -0.5500 | 1.44e-27 | 8.96e-27 |
| ENSMUSG00000054342 | Kcnn4 | protein\_coding | 7:24370263-24386690 (+) |  | 1.9800 | 1.46e-27 | 9.10e-27 |
| ENSMUSG00000062981 | Mrpl42 | protein\_coding | 10:95480805-95501940 (-) |  | 0.6840 | 1.50e-27 | 9.30e-27 |
| ENSMUSG00000024764 | Naa40 | protein\_coding | 19:7225668-7241183 (-) |  | 0.6160 | 1.55e-27 | 9.65e-27 |
| ENSMUSG00000022818 | Cyp2ab1 | protein\_coding | 16:20308387-20325404 (-) |  | -3.1400 | 1.59e-27 | 9.90e-27 |
| ENSMUSG00000064037 | Gpn1 | protein\_coding | 5:31494741-31512904 (+) |  | 0.7070 | 1.80e-27 | 1.12e-26 |
| ENSMUSG00000028266 | Lmo4 | protein\_coding | 3:144188530-144205220 (-) |  | -0.7190 | 2.00e-27 | 1.24e-26 |
| ENSMUSG00000031378 | Abcd1 | protein\_coding | X:73716597-73738534 (+) |  | -0.4700 | 2.03e-27 | 1.26e-26 |
| ENSMUSG00000040820 | Hlcs | protein\_coding | 16:94128882-94313571 (-) |  | -0.9240 | 2.10e-27 | 1.30e-26 |
| ENSMUSG00000034333 | Zbed4 | protein\_coding | 15:88751660-88784516 (+) |  | 0.7180 | 2.11e-27 | 1.31e-26 |
| ENSMUSG00000022519 | Srl | protein\_coding | 16:4480216-4541816 (-) |  | 1.4700 | 2.14e-27 | 1.33e-26 |
| ENSMUSG00000029397 | Rchy1 | protein\_coding | 5:91948904-91963068 (-) |  | -0.6230 | 2.17e-27 | 1.34e-26 |
| ENSMUSG00000034006 | Pqlc1 | protein\_coding | 18:80253292-80292725 (+) |  | -0.7690 | 2.19e-27 | 1.36e-26 |
| ENSMUSG00000020280 | Pus10 | protein\_coding | 11:23665674-23732876 (+) |  | 0.6120 | 2.28e-27 | 1.41e-26 |
| ENSMUSG00000057236 | Rbbp4 | protein\_coding | 4:129307100-129335370 (-) |  | 0.4580 | 2.34e-27 | 1.44e-26 |
| ENSMUSG00000075229 | Ccdc58 | protein\_coding | 16:36071685-36092120 (+) |  | 0.9920 | 2.36e-27 | 1.46e-26 |
| ENSMUSG00000075122 | Cd80 | protein\_coding | 16:38455561-38496335 (+) |  | -1.8300 | 2.37e-27 | 1.46e-26 |
| ENSMUSG00000022999 | Lmbr1l | protein\_coding | 15:98903917-98918231 (-) |  | -0.9000 | 2.39e-27 | 1.47e-26 |
| ENSMUSG00000022765 | Snap29 | protein\_coding | 16:17405986-17430827 (+) |  | -0.5910 | 2.47e-27 | 1.52e-26 |
| ENSMUSG00000024991 | Eif3a | protein\_coding | 19:60761117-60790658 (-) |  | 0.4930 | 2.61e-27 | 1.61e-26 |
| ENSMUSG00000031563 | Wwc2 | protein\_coding | 8:47823959-47990924 (-) |  | 0.8270 | 2.64e-27 | 1.63e-26 |
| ENSMUSG00000040824 | Snrpd2 | protein\_coding | 7:19149722-19153542 (+) |  | 0.7610 | 2.73e-27 | 1.69e-26 |
| ENSMUSG00000001143 | Lman2l | protein\_coding | 1:36419871-36445271 (-) |  | -0.5950 | 2.75e-27 | 1.69e-26 |
| ENSMUSG00000033762 | Recql4 | protein\_coding | 15:76703553-76710548 (-) |  | 1.1000 | 2.85e-27 | 1.75e-26 |
| ENSMUSG00000095028 | Sirpb1b | protein\_coding | 3:15495751-15575065 (-) |  | -3.5900 | 3.02e-27 | 1.86e-26 |
| ENSMUSG00000018398 | Sept8 | protein\_coding | 11:53519257-53549565 (+) |  | -0.9110 | 3.09e-27 | 1.90e-26 |
| ENSMUSG00000072720 | Myo18b | protein\_coding | 5:112688876-112896362 (-) |  | 3.5100 | 3.10e-27 | 1.91e-26 |
| ENSMUSG00000019726 | Lyst | protein\_coding | 13:13590397-13778803 (+) |  | -0.8100 | 3.17e-27 | 1.95e-26 |
| ENSMUSG00000091021 | Gm17300 | lncRNA | 4:132351772-132353382 (+) |  | 1.6600 | 3.41e-27 | 2.10e-26 |
| ENSMUSG00000074994 | Qser1 | protein\_coding | 2:104754795-104816760 (-) |  | 0.7190 | 3.77e-27 | 2.32e-26 |
| ENSMUSG00000041638 | Gcn1 | protein\_coding | 5:115565254-115622654 (+) |  | 0.6460 | 3.82e-27 | 2.34e-26 |
| ENSMUSG00000021182 | Ccdc88c | protein\_coding | 12:100911523-101029056 (-) |  | -0.8150 | 3.90e-27 | 2.40e-26 |
| ENSMUSG00000069893 | 9930111J21Rik1 | protein\_coding | 11:48946150-48979398 (-) |  | -1.3200 | 3.92e-27 | 2.41e-26 |
| ENSMUSG00000022283 | Pabpc1 | protein\_coding | 15:36595661-36609668 (-) |  | 0.6530 | 4.11e-27 | 2.52e-26 |
| ENSMUSG00000038227 | Hoxa9 | protein\_coding | 6:52223100-52231089 (-) |  | 4.0900 | 4.13e-27 | 2.53e-26 |
| ENSMUSG00000028525 | Pde4b | protein\_coding | 4:102087543-102607259 (+) |  | -1.3200 | 4.25e-27 | 2.60e-26 |
| ENSMUSG00000056144 | Trim34a | protein\_coding | 7:104244457-104262234 (+) |  | -1.1900 | 4.25e-27 | 2.60e-26 |
| ENSMUSG00000052942 | Glis3 | protein\_coding | 19:28258851-28680077 (-) |  | 2.0500 | 4.31e-27 | 2.64e-26 |
| ENSMUSG00000034194 | R3hcc1 | protein\_coding | 14:69697307-69707584 (-) |  | 0.9260 | 4.44e-27 | 2.72e-26 |
| ENSMUSG00000058318 | Phf21a | protein\_coding | 2:92093117-92364666 (+) |  | -0.7800 | 4.54e-27 | 2.78e-26 |
| ENSMUSG00000028871 | Rspo1 | protein\_coding | 4:124986430-125009099 (+) |  | -2.0800 | 4.54e-27 | 2.78e-26 |
| ENSMUSG00000052459 | Atp6v1a | protein\_coding | 16:44085402-44139705 (-) |  | -0.5500 | 4.73e-27 | 2.89e-26 |
| ENSMUSG00000041654 | Slc39a11 | protein\_coding | 11:113244853-113650079 (-) |  | 0.8040 | 4.98e-27 | 3.04e-26 |
| ENSMUSG00000045287 | Rtn4rl1 | protein\_coding | 11:75193783-75267769 (+) |  | 2.6900 | 4.99e-27 | 3.05e-26 |
| ENSMUSG00000032860 | P2ry2 | protein\_coding | 7:100996568-101012866 (-) |  | 1.3600 | 5.09e-27 | 3.11e-26 |
| ENSMUSG00000024404 | Riok3 | protein\_coding | 18:12128741-12157387 (+) |  | -0.4990 | 5.17e-27 | 3.16e-26 |
| ENSMUSG00000058809 | Hspd1-ps3 | processed\_pseudogene | 11:41498737-41500458 (+) |  | 1.3400 | 5.28e-27 | 3.22e-26 |
| ENSMUSG00000085786 | Gm15987 | lncRNA | 6:128951204-128975029 (-) |  | -0.7990 | 5.68e-27 | 3.46e-26 |
| ENSMUSG00000069805 | Fbp1 | protein\_coding | 13:62864753-62888282 (-) |  | 3.7600 | 5.75e-27 | 3.50e-26 |
| ENSMUSG00000042097 | Zfp239 | protein\_coding | 6:117862100-117873291 (+) |  | 3.2600 | 5.90e-27 | 3.60e-26 |
| ENSMUSG00000024581 | Napg | protein\_coding | 18:62977831-62999450 (+) |  | -0.5670 | 6.01e-27 | 3.66e-26 |
| ENSMUSG00000038827 | Abitram | protein\_coding | 4:56802345-56809601 (+) |  | 0.6530 | 6.37e-27 | 3.88e-26 |
| ENSMUSG00000101585 | 1600010M07Rik | lncRNA | 7:109998376-110151202 (-) |  | -2.1800 | 6.50e-27 | 3.96e-26 |
| ENSMUSG00000026315 | Serpinb8 | protein\_coding | 1:107590006-107610484 (+) |  | -3.0400 | 6.58e-27 | 4.00e-26 |
| ENSMUSG00000021094 | Dhrs7 | protein\_coding | 12:72650353-72664909 (-) |  | -0.6950 | 6.89e-27 | 4.19e-26 |
| ENSMUSG00000021993 | Mipep | protein\_coding | 14:60784573-60905478 (+) |  | 0.9330 | 6.90e-27 | 4.19e-26 |
| ENSMUSG00000024975 | Pdcd4 | protein\_coding | 19:53892231-53929860 (+) |  | 0.9940 | 7.08e-27 | 4.30e-26 |
| ENSMUSG00000027108 | Ola1 | protein\_coding | 2:73092801-73218924 (-) |  | 0.6280 | 7.17e-27 | 4.35e-26 |
| ENSMUSG00000029185 | Fam114a1 | protein\_coding | 5:64970071-65041886 (+) |  | -0.8810 | 7.24e-27 | 4.40e-26 |
| ENSMUSG00000059555 | Tor4a | protein\_coding | 2:25192966-25196886 (-) |  | -0.5950 | 7.66e-27 | 4.65e-26 |
| ENSMUSG00000106943 | Dancr | lncRNA | 5:74093060-74095406 (+) |  | 2.0400 | 8.09e-27 | 4.91e-26 |
| ENSMUSG00000027134 | Lpcat4 | protein\_coding | 2:112239468-112247111 (+) |  | -0.7010 | 8.66e-27 | 5.25e-26 |
| ENSMUSG00000025133 | Ints4 | protein\_coding | 7:97480956-97541395 (+) |  | 0.5910 | 8.69e-27 | 5.27e-26 |
| ENSMUSG00000052738 | Suclg1 | protein\_coding | 6:73248382-73276911 (+) |  | 0.5420 | 8.71e-27 | 5.28e-26 |
| ENSMUSG00000026975 | Dph7 | protein\_coding | 2:24962400-24972163 (+) |  | 0.8970 | 8.88e-27 | 5.38e-26 |
| ENSMUSG00000103367 | Gm38158 | processed\_pseudogene | 1:176835724-176836062 (+) |  | -1.6600 | 8.97e-27 | 5.43e-26 |
| ENSMUSG00000040260 | Daam2 | protein\_coding | 17:49456022-49564343 (-) |  | -1.6400 | 9.08e-27 | 5.49e-26 |
| ENSMUSG00000028270 | Gbp2 | protein\_coding | 3:142620602-142638008 (+) |  | -1.3900 | 9.15e-27 | 5.53e-26 |
| ENSMUSG00000028967 | Errfi1 | protein\_coding | 4:150853919-150868892 (+) |  | -1.7200 | 9.21e-27 | 5.57e-26 |
| ENSMUSG00000024661 | Fth1 | protein\_coding | 19:9980598-9985098 (+) |  | -0.4880 | 9.22e-27 | 5.58e-26 |
| ENSMUSG00000031360 | Ctps2 | protein\_coding | X:162901238-163032508 (+) |  | -0.6030 | 1.02e-26 | 6.16e-26 |
| ENSMUSG00000102051 | Ly6a2 | transcribed\_unprocessed\_pseudogene | 15:75131377-75135128 (-) |  | -0.8620 | 1.03e-26 | 6.25e-26 |
| ENSMUSG00000021322 | Aoah | protein\_coding | 13:20794113-21036617 (+) |  | -0.6350 | 1.07e-26 | 6.49e-26 |
| ENSMUSG00000042447 | Mios | protein\_coding | 6:8209222-8236274 (+) |  | 0.6770 | 1.08e-26 | 6.55e-26 |
| ENSMUSG00000042594 | Sh2b3 | protein\_coding | 5:121815488-121837646 (-) |  | -0.6030 | 1.19e-26 | 7.20e-26 |
| ENSMUSG00000032373 | Car12 | protein\_coding | 9:66713686-66766845 (+) |  | 4.7400 | 1.32e-26 | 7.98e-26 |
| ENSMUSG00000042121 | Ssh1 | protein\_coding | 5:113937094-113993894 (-) |  | -0.9060 | 1.34e-26 | 8.07e-26 |
| ENSMUSG00000025047 | Pdcd11 | protein\_coding | 19:47090768-47131865 (+) |  | 0.6950 | 1.34e-26 | 8.07e-26 |
| ENSMUSG00000036992 | Nxt1 | protein\_coding | 2:148672601-148676027 (+) |  | 0.6550 | 1.34e-26 | 8.10e-26 |
| ENSMUSG00000032555 | Topbp1 | protein\_coding | 9:103305215-103350428 (+) |  | 0.6590 | 1.41e-26 | 8.51e-26 |
| ENSMUSG00000049764 | Zfp280b | protein\_coding | 10:76032650-76043234 (+) |  | 0.7570 | 1.46e-26 | 8.80e-26 |
| ENSMUSG00000022969 | Il10rb | protein\_coding | 16:91406164-91425834 (+) |  | -0.6270 | 1.47e-26 | 8.84e-26 |
| ENSMUSG00000000378 | Ccm2 | protein\_coding | 11:6546887-6596744 (+) |  | -0.5790 | 1.51e-26 | 9.07e-26 |
| ENSMUSG00000116673 | A630089N07Rik | protein\_coding | 16:98001310-98082450 (-) |  | 1.3700 | 1.60e-26 | 9.60e-26 |
| ENSMUSG00000034247 | Plekhm1 | protein\_coding | 11:103364275-103412687 (-) |  | -0.7560 | 1.62e-26 | 9.74e-26 |
| ENSMUSG00000093402 | Gm18588 | processed\_pseudogene | 3:68902267-68902758 (-) |  | -0.9640 | 1.66e-26 | 9.99e-26 |
| ENSMUSG00000018899 | Irf1 | protein\_coding | 11:53770014-53778374 (+) |  | -1.1400 | 1.74e-26 | 1.04e-25 |
| ENSMUSG00000022051 | Bnip3l | protein\_coding | 14:66985239-67008877 (-) |  | -0.6540 | 1.80e-26 | 1.08e-25 |
| ENSMUSG00000019838 | Slc16a10 | protein\_coding | 10:40033532-40142258 (-) |  | -0.6490 | 1.94e-26 | 1.17e-25 |
| ENSMUSG00000085711 | Gm15163 | processed\_pseudogene | X:157016229-157017530 (-) |  | 1.1500 | 2.09e-26 | 1.25e-25 |
| ENSMUSG00000024048 | Myl12a | protein\_coding | 17:70993656-71002878 (-) |  | -0.5920 | 2.13e-26 | 1.28e-25 |
| ENSMUSG00000052949 | Rnf157 | protein\_coding | 11:116336353-116413032 (-) |  | 1.0200 | 2.21e-26 | 1.32e-25 |
| ENSMUSG00000066684 | Pilrb1 | protein\_coding | 5:137852147-137858106 (-) |  | -1.9200 | 2.21e-26 | 1.32e-25 |
| ENSMUSG00000039753 | Fbxl5 | protein\_coding | 5:43744615-43821638 (-) |  | -1.1600 | 2.22e-26 | 1.33e-25 |
| ENSMUSG00000034570 | Inpp5j | protein\_coding | 11:3494375-3504821 (-) |  | -1.4000 | 2.24e-26 | 1.34e-25 |
| ENSMUSG00000074129 | Rpl13a | protein\_coding | 7:45125558-45128761 (-) |  | 0.8400 | 2.29e-26 | 1.37e-25 |
| ENSMUSG00000031807 | Pgls | protein\_coding | 8:71592176-71601092 (+) |  | 0.6690 | 2.34e-26 | 1.40e-25 |
| ENSMUSG00000032120 | C2cd2l | protein\_coding | 9:44309237-44320285 (-) |  | -0.7420 | 2.37e-26 | 1.42e-25 |
| ENSMUSG00000001065 | Zfp276 | protein\_coding | 8:123254195-123269745 (+) |  | -0.6350 | 2.37e-26 | 1.42e-25 |
| ENSMUSG00000028028 | Alpk1 | protein\_coding | 3:127670310-127780527 (-) |  | -0.7310 | 2.41e-26 | 1.44e-25 |
| ENSMUSG00000001016 | Ilf2 | protein\_coding | 3:90476126-90488379 (+) |  | 0.4710 | 2.43e-26 | 1.45e-25 |
| ENSMUSG00000024410 | Rmc1 | protein\_coding | 18:12168717-12190658 (+) |  | -0.6800 | 2.45e-26 | 1.46e-25 |
| ENSMUSG00000074221 | Zfp568 | protein\_coding | 7:29983955-30028282 (+) |  | 0.6800 | 2.47e-26 | 1.47e-25 |
| ENSMUSG00000055675 | Kbtbd11 | protein\_coding | 8:15011025-15033333 (+) |  | -1.2300 | 2.86e-26 | 1.70e-25 |
| ENSMUSG00000027242 | Wdr76 | protein\_coding | 2:121506723-121544860 (+) |  | 0.7480 | 3.01e-26 | 1.80e-25 |
| ENSMUSG00000044952 | Kctd21 | protein\_coding | 7:97332327-97350213 (+) |  | -1.3900 | 3.05e-26 | 1.82e-25 |
| ENSMUSG00000025507 | Pidd1 | protein\_coding | 7:141438113-141444025 (-) |  | 1.1000 | 3.05e-26 | 1.82e-25 |
| ENSMUSG00000038900 | Rpl12 | protein\_coding | 2:32961559-32965345 (+) |  | 0.8120 | 3.18e-26 | 1.89e-25 |
| ENSMUSG00000026576 | Atp1b1 | protein\_coding | 1:164437109-164458355 (-) |  | 5.8100 | 3.31e-26 | 1.97e-25 |
| ENSMUSG00000021036 | Sptlc2 | protein\_coding | 12:87305058-87388355 (-) |  | -0.4510 | 3.31e-26 | 1.97e-25 |
| ENSMUSG00000036712 | Cyld | protein\_coding | 8:88697028-88751945 (+) |  | -0.4850 | 3.34e-26 | 1.99e-25 |
| ENSMUSG00000004642 | Slbp | protein\_coding | 5:33634952-33652574 (-) |  | 0.5350 | 3.49e-26 | 2.07e-25 |
| ENSMUSG00000026798 | Coq4 | protein\_coding | 2:29787493-29797935 (+) |  | 1.5300 | 3.49e-26 | 2.07e-25 |
| ENSMUSG00000097328 | Tnfsf12 | protein\_coding | 11:69686250-69695849 (-) |  | -1.4800 | 3.63e-26 | 2.16e-25 |
| ENSMUSG00000022489 | Pde1b | protein\_coding | 15:103503034-103530052 (+) |  | -1.2500 | 3.80e-26 | 2.26e-25 |
| ENSMUSG00000100975 | Gm28875 | lncRNA | 12:104925501-104926689 (-) |  | -0.7510 | 3.88e-26 | 2.31e-25 |
| ENSMUSG00000050144 | Slc25a44 | protein\_coding | 3:88410498-88425139 (-) |  | -0.5100 | 3.97e-26 | 2.35e-25 |
| ENSMUSG00000028654 | Mycl | protein\_coding | 4:122995652-123002485 (+) |  | 2.8900 | 4.08e-26 | 2.42e-25 |
| ENSMUSG00000002477 | Snrpd1 | protein\_coding | 18:10617775-10642079 (+) |  | 0.7290 | 4.26e-26 | 2.53e-25 |
| ENSMUSG00000046687 | Gm5424 | processed\_pseudogene | 10:62071123-62072362 (+) |  | 0.7290 | 4.38e-26 | 2.60e-25 |
| ENSMUSG00000043157 | Arl11 | protein\_coding | 14:61309753-61311936 (+) |  | -0.8110 | 4.60e-26 | 2.73e-25 |
| ENSMUSG00000026110 | Mgat4a | protein\_coding | 1:37439340-37541016 (-) |  | 1.2400 | 4.71e-26 | 2.79e-25 |
| ENSMUSG00000048755 | Mcat | protein\_coding | 15:83546797-83563787 (-) |  | 0.8990 | 4.86e-26 | 2.88e-25 |
| ENSMUSG00000078816 | Prkcg | protein\_coding | 7:3289179-3331099 (+) |  | -2.3200 | 4.95e-26 | 2.93e-25 |
| ENSMUSG00000003226 | Ranbp2 | protein\_coding | 10:58446920-58494356 (+) |  | 0.4960 | 5.07e-26 | 3.00e-25 |
| ENSMUSG00000007827 | Ankrd26 | protein\_coding | 6:118501308-118562226 (-) |  | 1.0100 | 5.35e-26 | 3.16e-25 |
| ENSMUSG00000027363 | Usp8 | protein\_coding | 2:126707328-126759297 (+) |  | -0.3850 | 5.51e-26 | 3.25e-25 |
| ENSMUSG00000022184 | Fbxo4 | protein\_coding | 15:3965445-3979573 (-) |  | -0.8710 | 5.59e-26 | 3.30e-25 |
| ENSMUSG00000116165 | Pdxp | protein\_coding | 15:78913919-78919517 (+) |  | 1.3900 | 5.79e-26 | 3.42e-25 |
| ENSMUSG00000024246 | Thumpd2 | protein\_coding | 17:81026332-81065082 (-) |  | 1.2500 | 5.96e-26 | 3.52e-25 |
| ENSMUSG00000032041 | Tirap | protein\_coding | 9:35184551-35200291 (-) |  | -0.6470 | 6.05e-26 | 3.57e-25 |
| ENSMUSG00000050373 | Snx21 | protein\_coding | 2:164785823-164793816 (+) |  | -1.0600 | 6.29e-26 | 3.71e-25 |
| ENSMUSG00000028893 | Sesn2 | protein\_coding | 4:132492032-132510501 (-) |  | -0.7970 | 6.66e-26 | 3.93e-25 |
| ENSMUSG00000027472 | Pdrg1 | protein\_coding | 2:153008890-153015427 (-) |  | 0.6500 | 6.69e-26 | 3.94e-25 |
| ENSMUSG00000028099 | Polr3c | protein\_coding | 3:96711490-96727628 (-) |  | -0.3950 | 6.79e-26 | 4.00e-25 |
| ENSMUSG00000006019 | Dhx34 | protein\_coding | 7:16197147-16222037 (-) |  | -0.6880 | 6.94e-26 | 4.08e-25 |
| ENSMUSG00000041632 | Mrps27 | protein\_coding | 13:99344786-99415562 (+) |  | 0.8690 | 7.42e-26 | 4.37e-25 |
| ENSMUSG00000024271 | Elp2 | protein\_coding | 18:24602866-24639507 (+) |  | 0.4500 | 8.30e-26 | 4.88e-25 |
| ENSMUSG00000086564 | Cd101 | protein\_coding | 3:100993529-101029556 (-) |  | -2.1700 | 8.86e-26 | 5.21e-25 |
| ENSMUSG00000034807 | Colgalt1 | protein\_coding | 8:71610998-71624911 (+) |  | 0.4440 | 8.92e-26 | 5.25e-25 |
| ENSMUSG00000010358 | Ifi35 | protein\_coding | 11:101448407-101458698 (+) |  | -0.6900 | 9.15e-26 | 5.38e-25 |
| ENSMUSG00000038295 | Atg9b | protein\_coding | 5:24384181-24392143 (-) |  | 1.5600 | 9.21e-26 | 5.41e-25 |
| ENSMUSG00000053289 | Ddx10 | protein\_coding | 9:53098635-53248053 (-) |  | 0.6720 | 9.75e-26 | 5.73e-25 |
| ENSMUSG00000028954 | Nub1 | protein\_coding | 5:24685532-24710378 (+) |  | -0.5600 | 9.76e-26 | 5.73e-25 |
| ENSMUSG00000042271 | Nxt2 | protein\_coding | X:142226770-142239692 (+) |  | -0.9520 | 9.76e-26 | 5.73e-25 |
| ENSMUSG00000021589 | Rhobtb3 | protein\_coding | 13:75869537-75943925 (-) |  | 3.4400 | 9.78e-26 | 5.74e-25 |
| ENSMUSG00000021109 | Hif1a | protein\_coding | 12:73901375-73947530 (+) |  | -0.8890 | 9.81e-26 | 5.75e-25 |
| ENSMUSG00000045751 | Mms22l | protein\_coding | 4:24496451-24602950 (+) |  | 0.9030 | 1.08e-25 | 6.34e-25 |
| ENSMUSG00000021340 | Gpld1 | protein\_coding | 13:24943152-24992501 (+) |  | -2.5300 | 1.17e-25 | 6.83e-25 |
| ENSMUSG00000071723 | Gspt2 | protein\_coding | X:94636069-94643244 (+) |  | 0.7970 | 1.18e-25 | 6.89e-25 |
| ENSMUSG00000029148 | Nrbp1 | protein\_coding | 5:31240864-31251566 (+) |  | -0.4180 | 1.18e-25 | 6.92e-25 |
| ENSMUSG00000022105 | Rb1 | protein\_coding | 14:73183673-73325822 (-) |  | -0.7870 | 1.22e-25 | 7.11e-25 |
| ENSMUSG00000038884 | Shfl | protein\_coding | 9:20868642-20874307 (+) |  | 2.5100 | 1.28e-25 | 7.48e-25 |
| ENSMUSG00000026883 | Dab2ip | protein\_coding | 2:35558266-35730994 (+) |  | 2.5100 | 1.30e-25 | 7.60e-25 |
| ENSMUSG00000003039 | Fam32a | protein\_coding | 8:72219730-72224418 (+) |  | -0.7520 | 1.33e-25 | 7.78e-25 |
| ENSMUSG00000027247 | Arhgap1 | protein\_coding | 2:91649860-91672326 (+) |  | -0.5340 | 1.34e-25 | 7.83e-25 |
| ENSMUSG00000042272 | Sestd1 | protein\_coding | 2:77180340-77280592 (-) |  | -0.7130 | 1.36e-25 | 7.94e-25 |
| ENSMUSG00000020865 | Abcc3 | protein\_coding | 11:94343295-94392997 (-) |  | -2.1100 | 1.37e-25 | 7.99e-25 |
| ENSMUSG00000055148 | Klf2 | protein\_coding | 8:72319033-72321656 (+) |  | -2.4100 | 1.50e-25 | 8.78e-25 |
| ENSMUSG00000059743 | Fdps | protein\_coding | 3:89093588-89101959 (-) |  | 0.9150 | 1.52e-25 | 8.87e-25 |
| ENSMUSG00000056091 | St3gal5 | protein\_coding | 6:72097592-72154571 (+) |  | -1.5600 | 1.52e-25 | 8.89e-25 |
| ENSMUSG00000005899 | Smpd4 | protein\_coding | 16:17619354-17644828 (+) |  | 0.7480 | 1.57e-25 | 9.16e-25 |
| ENSMUSG00000022636 | Alcam | protein\_coding | 16:52248996-52454074 (-) |  | -0.6580 | 1.65e-25 | 9.61e-25 |
| ENSMUSG00000036327 | Qsox2 | protein\_coding | 2:26208637-26237525 (-) |  | 0.7320 | 1.66e-25 | 9.66e-25 |
| ENSMUSG00000021754 | Map3k1 | protein\_coding | 13:111746428-111808993 (-) |  | -0.7550 | 1.67e-25 | 9.74e-25 |
| ENSMUSG00000027583 | Zbtb46 | protein\_coding | 2:181387762-181459426 (-) |  | 2.5100 | 1.69e-25 | 9.85e-25 |
| ENSMUSG00000021696 | Elovl7 | protein\_coding | 13:108214404-108285683 (+) |  | 1.5500 | 1.73e-25 | 1.00e-24 |
| ENSMUSG00000030062 | Rpn1 | protein\_coding | 6:88084482-88105304 (+) |  | 0.6890 | 1.76e-25 | 1.02e-24 |
| ENSMUSG00000030560 | Ctsc | protein\_coding | 7:88278085-88310888 (+) |  | -1.0900 | 1.80e-25 | 1.05e-24 |
| ENSMUSG00000041849 | Card6 | protein\_coding | 15:5095981-5108539 (-) |  | -1.1900 | 1.81e-25 | 1.05e-24 |
| ENSMUSG00000041961 | Znrf3 | protein\_coding | 11:5276324-5444847 (-) |  | 1.7600 | 1.88e-25 | 1.09e-24 |
| ENSMUSG00000033538 | Casp4 | protein\_coding | 9:5308828-5336783 (+) |  | -1.4300 | 1.89e-25 | 1.10e-24 |
| ENSMUSG00000003037 | Rab8a | protein\_coding | 8:72161200-72183904 (+) |  | -0.5280 | 1.90e-25 | 1.10e-24 |
| ENSMUSG00000058492 | Scp2-ps2 | transcribed\_unprocessed\_pseudogene | 9:123306600-123317998 (-) |  | -0.7690 | 1.95e-25 | 1.13e-24 |
| ENSMUSG00000054582 | Pabpc1l | protein\_coding | 2:164025450-164050538 (+) |  | -2.1200 | 2.00e-25 | 1.16e-24 |
| ENSMUSG00000063334 | Krr1 | protein\_coding | 10:111972664-111988432 (+) |  | 0.4810 | 2.11e-25 | 1.22e-24 |
| ENSMUSG00000032352 | Lrrc1 | protein\_coding | 9:77430823-77544870 (-) |  | 2.3000 | 2.13e-25 | 1.23e-24 |
| ENSMUSG00000024769 | Cdc42bpg | protein\_coding | 19:6306456-6325652 (+) |  | 1.6400 | 2.14e-25 | 1.24e-24 |
| ENSMUSG00000071715 | Ncf4 | protein\_coding | 15:78244801-78262580 (+) |  | -0.7960 | 2.18e-25 | 1.26e-24 |
| ENSMUSG00000031497 | Tnfsf13b | protein\_coding | 8:10006467-10039072 (+) |  | -2.0700 | 2.21e-25 | 1.28e-24 |
| ENSMUSG00000028495 | Rps6 | protein\_coding | 4:86854660-86857412 (-) |  | 0.4810 | 2.25e-25 | 1.30e-24 |
| ENSMUSG00000052310 | Slc39a1 | protein\_coding | 3:90248172-90253612 (+) |  | -0.5170 | 2.31e-25 | 1.34e-24 |
| ENSMUSG00000031154 | Otud5 | protein\_coding | X:7841364-7876626 (+) |  | -0.4160 | 2.46e-25 | 1.42e-24 |
| ENSMUSG00000104960 | Snhg8 | lncRNA | 3:123507552-123508404 (-) |  | 0.9490 | 2.54e-25 | 1.47e-24 |
| ENSMUSG00000079419 | Ms4a6c | protein\_coding | 19:11469366-11482192 (+) |  | -0.7820 | 2.59e-25 | 1.50e-24 |
| ENSMUSG00000082292 | Gm12250 | processed\_pseudogene | 11:58187739-58189012 (+) |  | -1.1600 | 2.62e-25 | 1.52e-24 |
| ENSMUSG00000022881 | Rfc4 | protein\_coding | 16:23113943-23127737 (-) |  | 0.8220 | 2.76e-25 | 1.59e-24 |
| ENSMUSG00000038894 | Irs2 | protein\_coding | 8:10984681-11008458 (-) |  | -0.9250 | 2.76e-25 | 1.59e-24 |
| ENSMUSG00000028540 | Dph2 | protein\_coding | 4:117888643-117892032 (-) |  | 0.7730 | 2.79e-25 | 1.61e-24 |
| ENSMUSG00000048796 | Cyb561d1 | protein\_coding | 3:108195687-108201212 (-) |  | -0.7740 | 2.87e-25 | 1.66e-24 |
| ENSMUSG00000023988 | Bysl | protein\_coding | 17:47599331-47611492 (-) |  | 0.6570 | 2.90e-25 | 1.67e-24 |
| ENSMUSG00000027598 | Itch | protein\_coding | 2:155133509-155226855 (+) |  | -0.5430 | 3.07e-25 | 1.77e-24 |
| ENSMUSG00000040356 | Skiv2l | protein\_coding | 17:34839228-34850210 (-) |  | -0.3990 | 3.08e-25 | 1.77e-24 |
| ENSMUSG00000034390 | Cmip | protein\_coding | 8:117257064-117459430 (+) |  | -0.6770 | 3.15e-25 | 1.81e-24 |
| ENSMUSG00000064267 | Hvcn1 | protein\_coding | 5:122206804-122242297 (+) |  | 1.0000 | 3.18e-25 | 1.83e-24 |
| ENSMUSG00000091474 | 2610021A01Rik | protein\_coding | 7:41599230-41628533 (+) |  | 0.7930 | 3.22e-25 | 1.85e-24 |
| ENSMUSG00000026694 | Eef1aknmt | protein\_coding | 1:162532127-162548551 (-) |  | 0.8120 | 3.26e-25 | 1.87e-24 |
| ENSMUSG00000023025 | Larp4 | protein\_coding | 15:99970065-100016358 (+) |  | 0.5610 | 3.35e-25 | 1.93e-24 |
| ENSMUSG00000025068 | Gsto1 | protein\_coding | 19:47854970-47864790 (+) |  | 0.6360 | 3.48e-25 | 2.00e-24 |
| ENSMUSG00000042275 | Pelo | protein\_coding | 13:115088355-115090186 (-) |  | 1.0100 | 3.62e-25 | 2.08e-24 |
| ENSMUSG00000026821 | Ralgds | protein\_coding | 2:28513125-28553081 (+) |  | 1.4600 | 3.65e-25 | 2.10e-24 |
| ENSMUSG00000054545 | Ugt1a6a | protein\_coding | 1:88134809-88218997 (+) |  | -1.3400 | 3.66e-25 | 2.10e-24 |
| ENSMUSG00000026655 | Fam107b | protein\_coding | 2:3570488-3782142 (+) |  | 0.4040 | 3.71e-25 | 2.13e-24 |
| ENSMUSG00000095332 | Gm9821 | lncRNA | 2:91945703-91948688 (+) |  | 1.5300 | 3.92e-25 | 2.25e-24 |
| ENSMUSG00000116138 | C030006K11Rik | protein\_coding | 15:76721465-76723845 (-) |  | 1.2000 | 3.93e-25 | 2.25e-24 |
| ENSMUSG00000037601 | Nme1 | protein\_coding | 11:93956979-93968521 (-) |  | 0.8160 | 3.94e-25 | 2.26e-24 |
| ENSMUSG00000053175 | Bcl3 | protein\_coding | 7:19808462-19822770 (-) |  | -1.0200 | 3.95e-25 | 2.26e-24 |
| ENSMUSG00000030045 | Mrpl19 | protein\_coding | 6:81957851-81965958 (-) |  | 0.7030 | 4.00e-25 | 2.29e-24 |
| ENSMUSG00000022553 | Maf1 | protein\_coding | 15:76351294-76354380 (+) |  | -0.6170 | 4.01e-25 | 2.30e-24 |
| ENSMUSG00000024533 | Spire1 | protein\_coding | 18:67488209-67610790 (-) |  | 1.2000 | 4.22e-25 | 2.42e-24 |
| ENSMUSG00000093904 | Tomm20 | protein\_coding | 8:126930667-126945844 (-) |  | 0.7310 | 4.30e-25 | 2.46e-24 |
| ENSMUSG00000035890 | Rnf126 | protein\_coding | 10:79758515-79766952 (-) |  | 0.5300 | 4.33e-25 | 2.47e-24 |
| ENSMUSG00000024963 | Dnajc4 | protein\_coding | 19:6987911-6992572 (-) |  | -0.9100 | 4.40e-25 | 2.51e-24 |
| ENSMUSG00000039994 | Timeless | protein\_coding | 10:128232065-128252941 (+) |  | 0.9380 | 4.59e-25 | 2.62e-24 |
| ENSMUSG00000019944 | Rhobtb1 | protein\_coding | 10:69151434-69291791 (+) |  | 3.0900 | 4.61e-25 | 2.63e-24 |
| ENSMUSG00000058997 | Vwa8 | protein\_coding | 14:78849052-79202310 (+) |  | 0.8580 | 4.70e-25 | 2.68e-24 |
| ENSMUSG00000032193 | Ldlr | protein\_coding | 9:21723483-21749919 (+) |  | -1.0900 | 5.20e-25 | 2.97e-24 |
| ENSMUSG00000026012 | Cd28 | protein\_coding | 1:60716800-60773359 (+) |  | 2.3900 | 5.28e-25 | 3.01e-24 |
| ENSMUSG00000018651 | Tada2a | protein\_coding | 11:84078920-84129600 (-) |  | 0.9020 | 5.41e-25 | 3.08e-24 |
| ENSMUSG00000048787 | Dcun1d3 | protein\_coding | 7:119852796-119896298 (-) |  | -0.9200 | 5.46e-25 | 3.11e-24 |
| ENSMUSG00000090272 | Mndal | protein\_coding | 1:173849126-173880881 (-) |  | -0.9270 | 5.46e-25 | 3.11e-24 |
| ENSMUSG00000031791 | Tmem38a | protein\_coding | 8:72572055-72587282 (+) |  | -1.8200 | 5.48e-25 | 3.12e-24 |
| ENSMUSG00000063628 | Gm7665 | processed\_pseudogene | 18:16274648-16274944 (-) |  | -1.0300 | 5.49e-25 | 3.13e-24 |
| ENSMUSG00000051504 | Siglech | protein\_coding | 7:55768178-55778925 (+) |  | 5.0900 | 5.58e-25 | 3.17e-24 |
| ENSMUSG00000025227 | Mfsd13a | protein\_coding | 19:46341121-46375252 (+) |  | 1.1200 | 5.59e-25 | 3.18e-24 |
| ENSMUSG00000002409 | Dyrk1b | protein\_coding | 7:28179469-28187294 (+) |  | -1.2500 | 5.90e-25 | 3.35e-24 |
| ENSMUSG00000021102 | Glrx5 | protein\_coding | 12:105032688-105042906 (+) |  | 0.6310 | 6.38e-25 | 3.63e-24 |
| ENSMUSG00000058503 | Fam133b | protein\_coding | 5:3543833-3570238 (+) |  | 0.7080 | 6.43e-25 | 3.65e-24 |
| ENSMUSG00000028282 | Casp8ap2 | protein\_coding | 4:32615451-32653265 (+) |  | 0.5150 | 6.61e-25 | 3.76e-24 |
| ENSMUSG00000036769 | Wdr44 | protein\_coding | X:23693051-23806025 (+) |  | -0.7470 | 6.73e-25 | 3.82e-24 |
| ENSMUSG00000037907 | Ankrd13b | protein\_coding | 11:77470485-77489678 (-) |  | 1.2700 | 6.86e-25 | 3.89e-24 |
| ENSMUSG00000079834 | Tmlhe | protein\_coding | GL456233.1:159647-334183 (+) |  | 2.8700 | 6.98e-25 | 3.96e-24 |
| ENSMUSG00000028907 | Utp11 | protein\_coding | 4:124678160-124693600 (-) |  | 0.6160 | 7.02e-25 | 3.98e-24 |
| ENSMUSG00000029030 | Tprgl | protein\_coding | 4:154157485-154160666 (-) |  | -0.7700 | 7.19e-25 | 4.08e-24 |
| ENSMUSG00000032215 | Rsl24d1 | protein\_coding | 9:73113426-73123333 (+) |  | 0.5420 | 7.68e-25 | 4.35e-24 |
| ENSMUSG00000045312 | Lhfpl2 | protein\_coding | 13:94057796-94195409 (+) |  | 2.2100 | 7.74e-25 | 4.39e-24 |
| ENSMUSG00000005442 | Cic | protein\_coding | 7:25267704-25294159 (+) |  | -0.6410 | 8.08e-25 | 4.57e-24 |
| ENSMUSG00000029446 | Psph | protein\_coding | 5:129765558-129787449 (-) |  | 0.8760 | 8.09e-25 | 4.58e-24 |
| ENSMUSG00000010110 | Stx5a | protein\_coding | 19:8741413-8756069 (+) |  | -0.5340 | 8.13e-25 | 4.60e-24 |
| ENSMUSG00000056153 | Socs6 | protein\_coding | 18:88665224-88927481 (-) |  | -0.7050 | 8.43e-25 | 4.77e-24 |
| ENSMUSG00000020775 | Mrpl38 | protein\_coding | 11:116131817-116138868 (-) |  | 0.7030 | 8.83e-25 | 4.99e-24 |
| ENSMUSG00000025262 | Fam120c | protein\_coding | X:151344175-151471842 (+) |  | 2.0100 | 9.02e-25 | 5.10e-24 |
| ENSMUSG00000021311 | Mtr | protein\_coding | 13:12182712-12258113 (-) |  | 0.6440 | 9.09e-25 | 5.14e-24 |
| ENSMUSG00000034729 | Mrps10 | protein\_coding | 17:47368887-47381417 (+) |  | 0.7500 | 9.51e-25 | 5.37e-24 |
| ENSMUSG00000037805 | Rpl10a | protein\_coding | 17:28328471-28331033 (+) |  | 0.5760 | 9.68e-25 | 5.46e-24 |
| ENSMUSG00000075232 | Amd1 | protein\_coding | 10:40287458-40302188 (-) |  | 0.5120 | 9.76e-25 | 5.51e-24 |
| ENSMUSG00000027710 | Acad9 | protein\_coding | 3:36065979-36092853 (+) |  | 0.5690 | 1.00e-24 | 5.66e-24 |
| ENSMUSG00000066861 | Oas1g | protein\_coding | 5:120876142-120887613 (-) |  | -2.2700 | 1.01e-24 | 5.67e-24 |
| ENSMUSG00000002985 | Apoe | protein\_coding | 7:19696109-19699188 (-) |  | -3.3300 | 1.01e-24 | 5.68e-24 |
| ENSMUSG00000063410 | Stk24 | protein\_coding | 14:121286343-121380011 (-) |  | -0.5320 | 1.07e-24 | 6.01e-24 |
| ENSMUSG00000046314 | Stxbp6 | protein\_coding | 12:44852484-45074709 (-) |  | -1.1800 | 1.10e-24 | 6.18e-24 |
| ENSMUSG00000038459 | Abhd17c | protein\_coding | 7:84109356-84151893 (-) |  | 0.5770 | 1.11e-24 | 6.27e-24 |
| ENSMUSG00000042292 | Mrtfa | protein\_coding | 15:81012281-81190757 (-) |  | -0.7500 | 1.15e-24 | 6.46e-24 |
| ENSMUSG00000064341 | mt-Nd1 | protein\_coding | MT:2751-3707 (+) |  | 0.5690 | 1.15e-24 | 6.49e-24 |
| ENSMUSG00000033033 | Calhm2 | protein\_coding | 19:47105353-47138294 (-) |  | -0.6480 | 1.18e-24 | 6.63e-24 |
| ENSMUSG00000024978 | Gpam | protein\_coding | 19:55067268-55127238 (-) |  | 0.7360 | 1.22e-24 | 6.84e-24 |
| ENSMUSG00000027854 | Sike1 | protein\_coding | 3:102995708-103008459 (+) |  | -0.5190 | 1.22e-24 | 6.88e-24 |
| ENSMUSG00000033075 | Senp1 | protein\_coding | 15:98038744-98093744 (-) |  | 0.4210 | 1.24e-24 | 6.95e-24 |
| ENSMUSG00000021819 | Zswim8 | protein\_coding | 14:20707552-20723619 (+) |  | -0.6460 | 1.26e-24 | 7.06e-24 |
| ENSMUSG00000025790 | Slco3a1 | protein\_coding | 7:74275419-74554780 (-) |  | 1.2600 | 1.27e-24 | 7.13e-24 |
| ENSMUSG00000055762 | Eef1d | protein\_coding | 15:75894205-75909556 (-) |  | 0.6370 | 1.28e-24 | 7.20e-24 |
| ENSMUSG00000029518 | Rab35 | protein\_coding | 5:115631908-115647736 (+) |  | -0.4520 | 1.37e-24 | 7.71e-24 |
| ENSMUSG00000006498 | Ptbp1 | protein\_coding | 10:79854427-79864771 (+) |  | 0.5330 | 1.39e-24 | 7.82e-24 |
| ENSMUSG00000039285 | Azi2 | protein\_coding | 9:118040499-118069794 (+) |  | -0.5170 | 1.42e-24 | 7.96e-24 |
| ENSMUSG00000029992 | Gfpt1 | protein\_coding | 6:87042846-87092197 (+) |  | -0.7060 | 1.42e-24 | 7.97e-24 |
| ENSMUSG00000022241 | Tars | protein\_coding | 15:11382301-11399665 (-) |  | 0.6190 | 1.44e-24 | 8.06e-24 |
| ENSMUSG00000021906 | Oxnad1 | protein\_coding | 14:32085374-32103202 (+) |  | 1.1200 | 1.56e-24 | 8.74e-24 |
| ENSMUSG00000012114 | Med15 | protein\_coding | 16:17651208-17732891 (-) |  | -0.4700 | 1.57e-24 | 8.78e-24 |
| ENSMUSG00000017119 | Nbr1 | protein\_coding | 11:101552149-101581951 (+) |  | -0.5340 | 1.57e-24 | 8.79e-24 |
| ENSMUSG00000001156 | Mxd1 | protein\_coding | 6:86647042-86669161 (-) |  | -0.8190 | 1.60e-24 | 8.93e-24 |
| ENSMUSG00000036572 | Upf3b | protein\_coding | X:37091678-37110322 (-) |  | 0.6570 | 1.60e-24 | 8.96e-24 |
| ENSMUSG00000039081 | Zfp503 | protein\_coding | 14:21983959-21989601 (-) |  | -1.5700 | 1.61e-24 | 9.00e-24 |
| ENSMUSG00000037972 | Snn | protein\_coding | 16:11060945-11074985 (+) |  | 3.4000 | 1.63e-24 | 9.11e-24 |
| ENSMUSG00000034342 | Cbl | protein\_coding | 9:44142976-44234049 (-) |  | -0.6610 | 1.75e-24 | 9.76e-24 |
| ENSMUSG00000059866 | Tnip2 | protein\_coding | 5:34496087-34513991 (-) |  | -0.5580 | 1.76e-24 | 9.83e-24 |
| ENSMUSG00000046826 | Fam187b | protein\_coding | 7:30973790-30989726 (+) |  | -2.1200 | 1.78e-24 | 9.94e-24 |
| ENSMUSG00000075470 | Alg10b | protein\_coding | 15:90224311-90233471 (+) |  | 0.3610 | 1.81e-24 | 1.01e-23 |
| ENSMUSG00000116504 | I730030J21Rik | lncRNA | 15:100730481-100732737 (-) |  | 5.3000 | 1.85e-24 | 1.03e-23 |
| ENSMUSG00000060166 | Zdhhc8 | protein\_coding | 16:18220753-18235136 (-) |  | 0.9280 | 1.88e-24 | 1.05e-23 |
| ENSMUSG00000074151 | Nlrc5 | protein\_coding | 8:94434356-94527272 (+) |  | -0.7110 | 1.93e-24 | 1.08e-23 |
| ENSMUSG00000026887 | Mrrf | protein\_coding | 2:36136389-36190647 (+) |  | 0.8600 | 1.94e-24 | 1.08e-23 |
| ENSMUSG00000039458 | Mtmr12 | protein\_coding | 15:12205028-12274496 (+) |  | -0.4770 | 1.95e-24 | 1.09e-23 |
| ENSMUSG00000017715 | Pgs1 | protein\_coding | 11:117986292-118024011 (+) |  | -0.4880 | 2.01e-24 | 1.12e-23 |
| ENSMUSG00000029343 | Crybb1 | protein\_coding | 5:112255815-112269585 (+) |  | -3.8000 | 2.03e-24 | 1.13e-23 |
| ENSMUSG00000113669 | Gm36723 | lncRNA | 12:25303940-25471979 (+) |  | -3.0900 | 2.20e-24 | 1.22e-23 |
| ENSMUSG00000035842 | Ddx11 | protein\_coding | 17:66123520-66152174 (+) |  | 0.9310 | 2.21e-24 | 1.23e-23 |
| ENSMUSG00000097365 | C030034L19Rik | lncRNA | 3:9403064-9437233 (+) |  | 4.4900 | 2.24e-24 | 1.25e-23 |
| ENSMUSG00000000275 | Trim25 | protein\_coding | 11:88999376-89020293 (+) |  | -0.7160 | 2.30e-24 | 1.28e-23 |
| ENSMUSG00000104435 | Gm37422 | lncRNA | 1:189879935-189884652 (+) |  | 2.0400 | 2.32e-24 | 1.29e-23 |
| ENSMUSG00000000743 | Chmp1a | protein\_coding | 8:123204264-123212763 (-) |  | -0.5350 | 2.34e-24 | 1.30e-23 |
| ENSMUSG00000034757 | Tmub2 | protein\_coding | 11:102284931-102289237 (+) |  | -0.4770 | 2.38e-24 | 1.32e-23 |
| ENSMUSG00000059606 | Rnase2b | protein\_coding | 14:51162260-51163018 (+) |  | -2.6000 | 2.49e-24 | 1.38e-23 |
| ENSMUSG00000061613 | U2af1 | protein\_coding | 17:31647081-31658892 (-) |  | 0.6100 | 2.51e-24 | 1.39e-23 |
| ENSMUSG00000027508 | Pag1 | protein\_coding | 3:9687479-9833679 (-) |  | -0.7860 | 2.52e-24 | 1.40e-23 |
| ENSMUSG00000028821 | Syf2 | protein\_coding | 4:134930898-134937548 (+) |  | -0.6560 | 2.58e-24 | 1.43e-23 |
| ENSMUSG00000019960 | Dusp6 | protein\_coding | 10:99263231-99267489 (+) |  | -2.4600 | 2.64e-24 | 1.46e-23 |
| ENSMUSG00000042699 | Dhx9 | protein\_coding | 1:153455758-153487660 (-) |  | 0.6430 | 2.91e-24 | 1.61e-23 |
| ENSMUSG00000073902 | Gm1966 | unprocessed\_pseudogene | 7:106596743-106604035 (-) |  | -1.0300 | 2.95e-24 | 1.63e-23 |
| ENSMUSG00000031153 | Gripap1 | protein\_coding | X:7789765-7820567 (+) |  | -0.6650 | 2.96e-24 | 1.64e-23 |
| ENSMUSG00000027342 | Pcna | protein\_coding | 2:132249162-132253314 (-) |  | 0.6920 | 3.07e-24 | 1.70e-23 |
| ENSMUSG00000001403 | Ube2c | protein\_coding | 2:164769898-164778822 (+) |  | -0.5610 | 3.26e-24 | 1.80e-23 |
| ENSMUSG00000051166 | Eml5 | protein\_coding | 12:98786805-98901484 (-) |  | 1.5100 | 3.42e-24 | 1.89e-23 |
| ENSMUSG00000041225 | Arhgap12 | protein\_coding | 18:6024427-6136098 (-) |  | -0.8510 | 3.43e-24 | 1.90e-23 |
| ENSMUSG00000035891 | Cerk | protein\_coding | 15:86139128-86186141 (-) |  | -0.6110 | 3.49e-24 | 1.93e-23 |
| ENSMUSG00000033991 | Ttc37 | protein\_coding | 13:76098734-76190316 (+) |  | 0.5940 | 3.57e-24 | 1.97e-23 |
| ENSMUSG00000066800 | Rnasel | protein\_coding | 1:153749426-153764221 (+) |  | -0.6660 | 3.58e-24 | 1.98e-23 |
| ENSMUSG00000036966 | Spryd3 | protein\_coding | 15:102116528-102136234 (-) |  | -0.5500 | 3.62e-24 | 2.00e-23 |
| ENSMUSG00000030870 | Ubfd1 | protein\_coding | 7:122067175-122082194 (+) |  | 0.7170 | 3.67e-24 | 2.02e-23 |
| ENSMUSG00000073988 | Ttpa | protein\_coding | 4:20007938-20030785 (+) |  | 1.6300 | 3.69e-24 | 2.04e-23 |
| ENSMUSG00000047789 | Slc38a9 | protein\_coding | 13:112660751-112738749 (+) |  | -0.9600 | 3.80e-24 | 2.10e-23 |
| ENSMUSG00000018171 | Vmp1 | protein\_coding | 11:86583865-86683836 (-) |  | -0.9570 | 3.94e-24 | 2.17e-23 |
| ENSMUSG00000029094 | Afap1 | protein\_coding | 5:35893319-36003923 (+) |  | 1.4100 | 3.98e-24 | 2.19e-23 |
| ENSMUSG00000029674 | Limk1 | protein\_coding | 5:134656039-134688598 (-) |  | 0.9200 | 4.04e-24 | 2.22e-23 |
| ENSMUSG00000030393 | Zik1 | protein\_coding | 7:10487229-10495394 (-) |  | 1.5200 | 4.16e-24 | 2.29e-23 |
| ENSMUSG00000063229 | Ldha | protein\_coding | 7:46841475-46855627 (+) |  | 0.8520 | 4.22e-24 | 2.32e-23 |
| ENSMUSG00000021614 | Vcan | protein\_coding | 13:89655312-89742509 (-) |  | -4.2300 | 4.23e-24 | 2.33e-23 |
| ENSMUSG00000025877 | Hk3 | protein\_coding | 13:55005985-55021385 (-) |  | 0.7190 | 4.59e-24 | 2.52e-23 |
| ENSMUSG00000091478 | Gm10039 | processed\_pseudogene | 11:100361716-100362126 (-) |  | 0.7510 | 4.69e-24 | 2.58e-23 |
| ENSMUSG00000062115 | Rai1 | protein\_coding | 11:60105013-60199197 (+) |  | 0.7410 | 4.78e-24 | 2.62e-23 |
| ENSMUSG00000064210 | Ano6 | protein\_coding | 15:95790843-95974751 (+) |  | -0.5780 | 4.90e-24 | 2.69e-23 |
| ENSMUSG00000030780 | BC017158 | protein\_coding | 7:128271379-128298170 (-) |  | 0.7930 | 5.06e-24 | 2.78e-23 |
| ENSMUSG00000034109 | Golim4 | protein\_coding | 3:75875084-75956949 (-) |  | 0.4570 | 5.08e-24 | 2.79e-23 |
| ENSMUSG00000030137 | Tuba8 | protein\_coding | 6:121210696-121226854 (+) |  | 3.5800 | 5.17e-24 | 2.84e-23 |
| ENSMUSG00000033257 | Ttll4 | protein\_coding | 1:74661745-74703730 (+) |  | 0.7320 | 5.17e-24 | 2.84e-23 |
| ENSMUSG00000041650 | Pcca | protein\_coding | 14:122534324-122891100 (+) |  | 0.7210 | 5.29e-24 | 2.90e-23 |
| ENSMUSG00000073418 | C4b | protein\_coding | 17:34728380-34743882 (-) |  | -2.9400 | 5.32e-24 | 2.92e-23 |
| ENSMUSG00000053436 | Mapk14 | protein\_coding | 17:28691329-28748406 (+) |  | -0.3730 | 5.53e-24 | 3.03e-23 |
| ENSMUSG00000051615 | Rap2a | protein\_coding | 14:120478444-120507194 (+) |  | -0.5760 | 5.60e-24 | 3.07e-23 |
| ENSMUSG00000024480 | Ap3s1 | protein\_coding | 18:46741876-46790826 (+) |  | 0.9410 | 5.77e-24 | 3.16e-23 |
| ENSMUSG00000036186 | Dipk1b | protein\_coding | 2:26628457-26636497 (+) |  | 3.6200 | 5.82e-24 | 3.18e-23 |
| ENSMUSG00000019979 | Apaf1 | protein\_coding | 10:90989311-91082770 (-) |  | -0.5550 | 5.84e-24 | 3.20e-23 |
| ENSMUSG00000026473 | Glul | protein\_coding | 1:153899944-153909723 (+) |  | -0.9010 | 5.85e-24 | 3.20e-23 |
| ENSMUSG00000001166 | Oas1c | protein\_coding | 5:120800194-120812514 (-) |  | -3.0000 | 5.92e-24 | 3.23e-23 |
| ENSMUSG00000015943 | Bola1 | protein\_coding | 3:96196588-96219709 (-) |  | 1.0900 | 5.93e-24 | 3.24e-23 |
| ENSMUSG00000018377 | Vezf1 | protein\_coding | 11:88068279-88084729 (+) |  | -0.5480 | 5.94e-24 | 3.24e-23 |
| ENSMUSG00000028741 | Mrto4 | protein\_coding | 4:139347435-139352576 (-) |  | 0.7920 | 5.95e-24 | 3.25e-23 |
| ENSMUSG00000027236 | Eif3j1 | protein\_coding | 2:122028546-122056598 (+) |  | 0.6940 | 6.00e-24 | 3.28e-23 |
| ENSMUSG00000038816 | Ctnnal1 | protein\_coding | 4:56810935-56865188 (-) |  | 1.9300 | 6.18e-24 | 3.37e-23 |
| ENSMUSG00000039108 | Lsm14b | protein\_coding | 2:180024987-180035465 (+) |  | -0.5400 | 6.59e-24 | 3.60e-23 |
| ENSMUSG00000026669 | Mcm10 | protein\_coding | 2:4989714-5012791 (-) |  | 0.8180 | 6.62e-24 | 3.61e-23 |
| ENSMUSG00000027893 | Ahcyl1 | protein\_coding | 3:107663118-107696560 (-) |  | 0.4110 | 7.09e-24 | 3.87e-23 |
| ENSMUSG00000020549 | Elac2 | protein\_coding | 11:64979038-65002069 (+) |  | 0.6100 | 7.20e-24 | 3.92e-23 |
| ENSMUSG00000021559 | Dapk1 | protein\_coding | 13:60601947-60763191 (+) |  | -1.1800 | 7.41e-24 | 4.04e-23 |
| ENSMUSG00000026238 | Ptma | protein\_coding | 1:86526726-86530712 (+) |  | 0.5390 | 7.62e-24 | 4.15e-23 |
| ENSMUSG00000021476 | Habp4 | protein\_coding | 13:64161824-64186537 (+) |  | -0.9200 | 7.69e-24 | 4.18e-23 |
| ENSMUSG00000067203 | H2-K2 | transcribed\_unprocessed\_pseudogene | 17:33974659-33978827 (-) |  | -1.8100 | 7.75e-24 | 4.21e-23 |
| ENSMUSG00000038383 | Pigu | protein\_coding | 2:155278243-155357430 (-) |  | 0.6350 | 7.89e-24 | 4.29e-23 |
| ENSMUSG00000050796 | B3galt6 | protein\_coding | 4:155989466-155992649 (-) |  | 1.0300 | 8.14e-24 | 4.42e-23 |
| ENSMUSG00000059248 | Sept9 | protein\_coding | 11:117199661-117362325 (+) |  | -0.5080 | 8.24e-24 | 4.48e-23 |
| ENSMUSG00000059834 | Sclt1 | protein\_coding | 3:41626720-41742514 (-) |  | 0.8270 | 8.24e-24 | 4.48e-23 |
| ENSMUSG00000098188 | Sowahc | protein\_coding | 10:59221953-59226434 (+) |  | -0.8900 | 8.36e-24 | 4.54e-23 |
| ENSMUSG00000030315 | Vgll4 | protein\_coding | 6:114860628-114969994 (-) |  | -0.7260 | 8.37e-24 | 4.54e-23 |
| ENSMUSG00000028089 | Chd1l | protein\_coding | 3:97560742-97610203 (-) |  | 0.6240 | 8.58e-24 | 4.65e-23 |
| ENSMUSG00000031596 | Slc7a2 | protein\_coding | 8:40862396-40922308 (+) |  | 1.9200 | 8.62e-24 | 4.68e-23 |
| ENSMUSG00000000028 | Cdc45 | protein\_coding | 16:18780447-18811987 (-) |  | 0.7880 | 8.70e-24 | 4.72e-23 |
| ENSMUSG00000030200 | Bcl2l14 | protein\_coding | 6:134396318-134438736 (+) |  | 2.7800 | 8.74e-24 | 4.74e-23 |
| ENSMUSG00000073147 | 5031425E22Rik | lncRNA | 5:23382308-23434269 (-) |  | -0.8920 | 8.81e-24 | 4.77e-23 |
| ENSMUSG00000070691 | Runx3 | protein\_coding | 4:135120652-135177990 (+) |  | 0.9360 | 8.83e-24 | 4.78e-23 |
| ENSMUSG00000029203 | Ube2k | protein\_coding | 5:65537233-65598988 (+) |  | 0.3800 | 8.89e-24 | 4.81e-23 |
| ENSMUSG00000073468 | Sft2d1 | protein\_coding | 17:8311102-8327442 (+) |  | -0.6610 | 9.01e-24 | 4.88e-23 |
| ENSMUSG00000014470 | Rnf166 | protein\_coding | 8:122466147-122476064 (-) |  | -0.6570 | 9.03e-24 | 4.88e-23 |
| ENSMUSG00000024740 | Ddb1 | protein\_coding | 19:10605327-10629819 (+) |  | 0.6390 | 9.65e-24 | 5.22e-23 |
| ENSMUSG00000054428 | Atpif1 | protein\_coding | 4:132530555-132533659 (-) |  | 0.7320 | 9.69e-24 | 5.24e-23 |
| ENSMUSG00000037685 | Atp8a1 | protein\_coding | 5:67618140-67847434 (-) |  | -0.6770 | 9.89e-24 | 5.35e-23 |
| ENSMUSG00000040747 | Cd53 | protein\_coding | 3:106759921-106790149 (-) |  | -0.8970 | 1.03e-23 | 5.57e-23 |
| ENSMUSG00000028964 | Park7 | protein\_coding | 4:150897133-150914437 (-) |  | 0.7930 | 1.04e-23 | 5.62e-23 |
| ENSMUSG00000034175 | Rhbdd3 | protein\_coding | 11:5098926-5106093 (+) |  | 0.8810 | 1.05e-23 | 5.69e-23 |
| ENSMUSG00000026274 | Pask | protein\_coding | 1:93308770-93343482 (-) |  | 1.2600 | 1.08e-23 | 5.81e-23 |
| ENSMUSG00000004127 | Trmt10a | protein\_coding | 3:138143448-138159821 (+) |  | 1.0300 | 1.08e-23 | 5.82e-23 |
| ENSMUSG00000054509 | Parp4 | protein\_coding | 14:56575619-56659794 (+) |  | -0.7850 | 1.09e-23 | 5.85e-23 |
| ENSMUSG00000017631 | Abr | protein\_coding | 11:76416734-76623558 (-) |  | -0.4260 | 1.09e-23 | 5.89e-23 |
| ENSMUSG00000045466 | Zfp956 | protein\_coding | 6:47953390-47965300 (+) |  | 1.2600 | 1.12e-23 | 6.02e-23 |
| ENSMUSG00000028439 | Fam219a | protein\_coding | 4:41517691-41569538 (-) |  | -0.9780 | 1.12e-23 | 6.04e-23 |
| ENSMUSG00000028016 | Ints12 | protein\_coding | 3:133091840-133110988 (+) |  | -0.6780 | 1.15e-23 | 6.18e-23 |
| ENSMUSG00000032038 | St3gal4 | protein\_coding | 9:35046576-35119268 (-) |  | -0.7520 | 1.15e-23 | 6.19e-23 |
| ENSMUSG00000016534 | Lamp2 | protein\_coding | X:38401357-38456454 (-) |  | -0.7200 | 1.15e-23 | 6.21e-23 |
| ENSMUSG00000050002 | Idnk | protein\_coding | 13:58157649-58164693 (+) |  | -0.5790 | 1.16e-23 | 6.23e-23 |
| ENSMUSG00000001054 | Rmnd5b | protein\_coding | 11:51623671-51635896 (-) |  | -0.5870 | 1.16e-23 | 6.26e-23 |
| ENSMUSG00000026113 | Inpp4a | protein\_coding | 1:37299865-37410736 (+) |  | -0.6580 | 1.18e-23 | 6.35e-23 |
| ENSMUSG00000024896 | Minpp1 | protein\_coding | 19:32485769-32515364 (+) |  | 0.5230 | 1.20e-23 | 6.47e-23 |
| ENSMUSG00000037679 | Inf2 | protein\_coding | 12:112588784-112615557 (+) |  | -0.6130 | 1.23e-23 | 6.60e-23 |
| ENSMUSG00000002250 | Ppard | protein\_coding | 17:28232700-28301474 (+) |  | -0.8470 | 1.24e-23 | 6.67e-23 |
| ENSMUSG00000030538 | Cib1 | protein\_coding | 7:80227147-80232813 (-) |  | -0.5590 | 1.27e-23 | 6.82e-23 |
| ENSMUSG00000030521 | Mphosph10 | protein\_coding | 7:64376527-64392268 (-) |  | 0.8060 | 1.28e-23 | 6.89e-23 |
| ENSMUSG00000019877 | Serinc1 | protein\_coding | 10:57515774-57532530 (-) |  | -0.5600 | 1.30e-23 | 6.96e-23 |
| ENSMUSG00000089774 | Slc5a3 | protein\_coding | 16:92058322-92087473 (+) |  | 1.5900 | 1.36e-23 | 7.31e-23 |
| ENSMUSG00000026083 | Eif5b | protein\_coding | 1:37998010-38055579 (+) |  | 0.5300 | 1.38e-23 | 7.41e-23 |
| ENSMUSG00000021356 | Irf4 | protein\_coding | 13:30749226-30766976 (+) |  | -2.1500 | 1.43e-23 | 7.68e-23 |
| ENSMUSG00000029238 | Clock | protein\_coding | 5:76209868-76304792 (-) |  | -0.6970 | 1.44e-23 | 7.70e-23 |
| ENSMUSG00000028426 | Rad23b | protein\_coding | 4:55350043-55392237 (+) |  | 0.4940 | 1.46e-23 | 7.84e-23 |
| ENSMUSG00000042426 | Dhx29 | protein\_coding | 13:112927454-112969432 (+) |  | 0.5200 | 1.51e-23 | 8.07e-23 |
| ENSMUSG00000028729 | Ebna1bp2 | protein\_coding | 4:118620799-118627776 (+) |  | 0.7650 | 1.56e-23 | 8.32e-23 |
| ENSMUSG00000030303 | Far2 | protein\_coding | 6:148047259-148182758 (+) |  | 1.3200 | 1.56e-23 | 8.33e-23 |
| ENSMUSG00000023007 | Prpf40b | protein\_coding | 15:99295087-99317018 (+) |  | 2.4500 | 1.61e-23 | 8.61e-23 |
| ENSMUSG00000028560 | Usp1 | protein\_coding | 4:98923810-98935543 (+) |  | 0.5060 | 1.62e-23 | 8.64e-23 |
| ENSMUSG00000110331 | Nudc-ps1 | processed\_pseudogene | 8:29286431-29286981 (-) |  | 0.7850 | 1.65e-23 | 8.83e-23 |
| ENSMUSG00000097412 | 1810014B01Rik | transcribed\_unprocessed\_pseudogene | 10:86685525-86694806 (+) |  | 1.3900 | 1.69e-23 | 9.03e-23 |
| ENSMUSG00000026384 | Ptpn4 | protein\_coding | 1:119652467-119837613 (-) |  | 0.8690 | 1.71e-23 | 9.14e-23 |
| ENSMUSG00000035273 | Hpse | protein\_coding | 5:100679484-100719716 (-) |  | -0.6090 | 1.71e-23 | 9.14e-23 |
| ENSMUSG00000034826 | Nup54 | protein\_coding | 5:92415540-92435219 (-) |  | 0.5340 | 1.81e-23 | 9.68e-23 |
| ENSMUSG00000020402 | Vdac1 | protein\_coding | 11:52360860-52389397 (+) |  | 0.5440 | 1.82e-23 | 9.73e-23 |
| ENSMUSG00000031792 | Usb1 | protein\_coding | 8:95332284-95347507 (+) |  | -0.5880 | 1.88e-23 | 1.00e-22 |
| ENSMUSG00000037583 | Nr0b2 | protein\_coding | 4:133553376-133556536 (+) |  | 3.1300 | 1.94e-23 | 1.03e-22 |
| ENSMUSG00000044447 | Dock5 | protein\_coding | 14:67752135-67933442 (-) |  | -0.8530 | 2.00e-23 | 1.07e-22 |
| ENSMUSG00000020697 | Lig3 | protein\_coding | 11:82781108-82804274 (+) |  | 0.6050 | 2.04e-23 | 1.09e-22 |
| ENSMUSG00000022234 | Cct5 | protein\_coding | 15:31590800-31601804 (-) |  | 0.5510 | 2.08e-23 | 1.11e-22 |
| ENSMUSG00000045983 | Eif4g1 | protein\_coding | 16:20668313-20692884 (+) |  | 0.5710 | 2.20e-23 | 1.17e-22 |
| ENSMUSG00000020712 | Tcam1 | protein\_coding | 11:106276672-106288745 (+) |  | 4.7100 | 2.33e-23 | 1.24e-22 |
| ENSMUSG00000049807 | Arhgap23 | protein\_coding | 11:97415533-97502402 (+) |  | -0.7510 | 2.51e-23 | 1.34e-22 |
| ENSMUSG00000040552 | C3ar1 | protein\_coding | 6:122847138-122856161 (-) |  | -3.3000 | 2.53e-23 | 1.34e-22 |
| ENSMUSG00000037573 | Tob1 | protein\_coding | 11:94211454-94215495 (+) |  | -1.1700 | 2.53e-23 | 1.34e-22 |
| ENSMUSG00000027845 | Dclre1b | protein\_coding | 3:103800605-103809444 (-) |  | -0.5130 | 2.62e-23 | 1.39e-22 |
| ENSMUSG00000026781 | Acbd5 | protein\_coding | 2:23068167-23115558 (+) |  | -0.7260 | 2.63e-23 | 1.40e-22 |
| ENSMUSG00000069682 | Gm10275 | transcribed\_processed\_pseudogene | 10:29698746-29699380 (-) |  | 0.7330 | 2.69e-23 | 1.43e-22 |
| ENSMUSG00000023147 | Wrb | protein\_coding | 16:96145407-96157852 (+) |  | 0.8780 | 2.76e-23 | 1.46e-22 |
| ENSMUSG00000049686 | Orai1 | protein\_coding | 5:123015074-123030456 (+) |  | -0.4990 | 2.76e-23 | 1.47e-22 |
| ENSMUSG00000031214 | Ophn1 | protein\_coding | X:98554277-98891025 (-) |  | -1.5300 | 2.79e-23 | 1.48e-22 |
| ENSMUSG00000051220 | Ercc6l | protein\_coding | X:102141716-102157091 (-) |  | 0.6080 | 2.82e-23 | 1.50e-22 |
| ENSMUSG00000018995 | Nars2 | protein\_coding | 7:96951505-97064758 (+) |  | 1.1000 | 2.84e-23 | 1.51e-22 |
| ENSMUSG00000024055 | Cyp4f13 | protein\_coding | 17:32924688-32947402 (-) |  | -1.0100 | 2.90e-23 | 1.53e-22 |
| ENSMUSG00000022323 | Rida | protein\_coding | 15:34484021-34495255 (-) |  | 1.3900 | 2.92e-23 | 1.55e-22 |
| ENSMUSG00000079109 | Pms2 | protein\_coding | 5:143909964-143933968 (+) |  | 0.6450 | 2.99e-23 | 1.58e-22 |
| ENSMUSG00000041797 | Abca9 | protein\_coding | 11:110100749-110168196 (-) |  | -1.7400 | 3.01e-23 | 1.59e-22 |
| ENSMUSG00000037300 | Ttc13 | protein\_coding | 8:124671332-124721983 (-) |  | 0.3730 | 3.03e-23 | 1.60e-22 |
| ENSMUSG00000058704 | Memo1 | protein\_coding | 17:74199036-74295521 (-) |  | 0.6020 | 3.07e-23 | 1.63e-22 |
| ENSMUSG00000097729 | 2310015A10Rik | lncRNA | 12:80120546-80132844 (-) |  | -1.5300 | 3.17e-23 | 1.67e-22 |
| ENSMUSG00000033220 | Rac2 | protein\_coding | 15:78559167-78572783 (-) |  | -0.3810 | 3.23e-23 | 1.71e-22 |
| ENSMUSG00000027086 | Fastkd1 | protein\_coding | 2:69686815-69713516 (-) |  | 1.0600 | 3.23e-23 | 1.71e-22 |
| ENSMUSG00000078616 | Trim30c | protein\_coding | 7:104382065-104400837 (-) |  | -1.7200 | 3.31e-23 | 1.75e-22 |
| ENSMUSG00000019998 | Stx7 | protein\_coding | 10:24149302-24190222 (+) |  | -0.6780 | 3.32e-23 | 1.75e-22 |
| ENSMUSG00000024007 | Ppil1 | protein\_coding | 17:29250803-29264186 (-) |  | 0.7480 | 3.43e-23 | 1.81e-22 |
| ENSMUSG00000025872 | Thoc3 | protein\_coding | 13:54458837-54468849 (-) |  | 0.4660 | 3.46e-23 | 1.82e-22 |
| ENSMUSG00000008429 | Herpud2 | protein\_coding | 9:25108132-25151820 (-) |  | -0.5030 | 3.48e-23 | 1.84e-22 |
| ENSMUSG00000022828 | Gtf2e1 | protein\_coding | 16:37509790-37539789 (-) |  | 0.6430 | 3.49e-23 | 1.84e-22 |
| ENSMUSG00000030830 | Itgal | protein\_coding | 7:127296260-127335138 (+) |  | -0.4340 | 3.65e-23 | 1.92e-22 |
| ENSMUSG00000050029 | Rap2c | protein\_coding | X:51003912-51018018 (-) |  | -0.5310 | 3.77e-23 | 1.99e-22 |
| ENSMUSG00000021770 | Samd8 | protein\_coding | 14:21750531-21798726 (+) |  | -0.6700 | 3.99e-23 | 2.10e-22 |
| ENSMUSG00000026526 | Fh1 | protein\_coding | 1:175600374-175625635 (-) |  | 0.5450 | 4.04e-23 | 2.12e-22 |
| ENSMUSG00000031021 | Tmem9b | protein\_coding | 7:109735834-109752875 (-) |  | -0.5730 | 4.06e-23 | 2.13e-22 |
| ENSMUSG00000032575 | Manf | protein\_coding | 9:106838312-106891979 (-) |  | 0.6750 | 4.06e-23 | 2.14e-22 |
| ENSMUSG00000032883 | Acsl3 | protein\_coding | 1:78657825-78707743 (+) |  | 0.9040 | 4.30e-23 | 2.26e-22 |
| ENSMUSG00000061778 | Mospd2 | protein\_coding | X:164936169-164980375 (-) |  | -0.9040 | 4.89e-23 | 2.57e-22 |
| ENSMUSG00000005054 | Cstb | protein\_coding | 10:78425669-78427622 (+) |  | -0.8480 | 4.97e-23 | 2.61e-22 |
| ENSMUSG00000032051 | Fdx1 | protein\_coding | 9:51943306-51963556 (-) |  | 0.9560 | 5.11e-23 | 2.68e-22 |
| ENSMUSG00000032012 | Nectin1 | protein\_coding | 9:43743984-43832658 (+) |  | -1.3200 | 5.20e-23 | 2.73e-22 |
| ENSMUSG00000042050 | Wdr60 | protein\_coding | 12:116206262-116263022 (-) |  | 3.2900 | 5.41e-23 | 2.84e-22 |
| ENSMUSG00000040749 | Siah1b | protein\_coding | X:164070705-164076493 (-) |  | 1.1600 | 5.46e-23 | 2.86e-22 |
| ENSMUSG00000001891 | Ugp2 | protein\_coding | 11:21321138-21371201 (-) |  | -0.7800 | 5.65e-23 | 2.96e-22 |
| ENSMUSG00000045817 | Zfp36l2 | protein\_coding | 17:84183931-84187947 (-) |  | -0.7270 | 5.72e-23 | 3.00e-22 |
| ENSMUSG00000021012 | Zc3h14 | protein\_coding | 12:98746964-98787753 (+) |  | 0.4650 | 6.07e-23 | 3.18e-22 |
| ENSMUSG00000008450 | Nutf2 | protein\_coding | 8:105860580-105879330 (+) |  | 0.7890 | 6.09e-23 | 3.19e-22 |
| ENSMUSG00000027087 | Itgav | protein\_coding | 2:83724397-83806916 (+) |  | -0.6080 | 6.11e-23 | 3.20e-22 |
| ENSMUSG00000041992 | Rapgef5 | protein\_coding | 12:117516479-117759737 (+) |  | 2.0200 | 6.19e-23 | 3.24e-22 |
| ENSMUSG00000046836 | Brox | protein\_coding | 1:183276352-183297269 (-) |  | -0.5010 | 6.27e-23 | 3.28e-22 |
| ENSMUSG00000028961 | Pgd | protein\_coding | 4:149149991-149166771 (-) |  | -0.4350 | 6.30e-23 | 3.30e-22 |
| ENSMUSG00000030842 | Lamtor1 | protein\_coding | 7:101905904-101926675 (+) |  | -0.4390 | 6.32e-23 | 3.30e-22 |
| ENSMUSG00000005824 | Tnfsf14 | protein\_coding | 17:57189492-57194177 (-) |  | -0.8530 | 6.51e-23 | 3.41e-22 |
| ENSMUSG00000063884 | Ptcd3 | protein\_coding | 6:71880638-71908750 (-) |  | 0.7470 | 6.53e-23 | 3.41e-22 |
| ENSMUSG00000020085 | Aifm2 | protein\_coding | 10:61715263-61739260 (+) |  | -1.1400 | 6.67e-23 | 3.49e-22 |
| ENSMUSG00000021810 | Ecd | protein\_coding | 14:20319852-20348121 (-) |  | 0.5210 | 6.72e-23 | 3.51e-22 |
| ENSMUSG00000056999 | Ide | protein\_coding | 19:37268743-37337852 (-) |  | 0.8310 | 6.75e-23 | 3.53e-22 |
| ENSMUSG00000035150 | Eif2s3x | protein\_coding | X:94188707-94212862 (-) |  | 0.6710 | 6.91e-23 | 3.60e-22 |
| ENSMUSG00000074604 | Mgst2 | protein\_coding | 3:51660360-51682677 (+) |  | 1.2500 | 7.09e-23 | 3.70e-22 |
| ENSMUSG00000107215 | Gm43197 | TEC | 6:3336772-3339354 (-) |  | -1.8100 | 7.22e-23 | 3.77e-22 |
| ENSMUSG00000055435 | Maf | protein\_coding | 8:115682942-115707794 (-) |  | -3.9600 | 7.50e-23 | 3.91e-22 |
| ENSMUSG00000028909 | Ptpru | protein\_coding | 4:131768457-131838288 (-) |  | 1.3000 | 7.65e-23 | 3.99e-22 |
| ENSMUSG00000031388 | Naa10 | protein\_coding | X:73916873-73921944 (-) |  | 0.6940 | 7.83e-23 | 4.08e-22 |
| ENSMUSG00000034158 | Lrrc58 | protein\_coding | 16:37868389-37888858 (+) |  | 0.4880 | 7.87e-23 | 4.10e-22 |
| ENSMUSG00000021866 | Anxa11 | protein\_coding | 14:25842156-25886804 (+) |  | -0.6280 | 7.99e-23 | 4.16e-22 |
| ENSMUSG00000022479 | Vdr | protein\_coding | 15:97854425-97910630 (-) |  | 1.3500 | 8.00e-23 | 4.16e-22 |
| ENSMUSG00000098985 | Gm27219 | processed\_pseudogene | 9:36813133-36813874 (-) |  | 1.7200 | 8.23e-23 | 4.28e-22 |
| ENSMUSG00000033191 | Tie1 | protein\_coding | 4:118471191-118490061 (-) |  | 3.7500 | 8.26e-23 | 4.29e-22 |
| ENSMUSG00000079164 | Tlr5 | protein\_coding | 1:182954788-182976044 (+) |  | -1.3800 | 8.30e-23 | 4.32e-22 |
| ENSMUSG00000021646 | Mccc2 | protein\_coding | 13:99948530-100015639 (-) |  | 0.9950 | 8.80e-23 | 4.58e-22 |
| ENSMUSG00000029500 | Pgam5 | protein\_coding | 5:110259130-110269913 (-) |  | 0.4910 | 8.97e-23 | 4.66e-22 |
| ENSMUSG00000008435 | Rdh13 | protein\_coding | 7:4424770-4445649 (-) |  | 0.8480 | 9.06e-23 | 4.71e-22 |
| ENSMUSG00000019832 | Rab32 | protein\_coding | 10:10545002-10558265 (-) |  | -0.5120 | 9.15e-23 | 4.75e-22 |
| ENSMUSG00000024732 | Ccdc86 | protein\_coding | 19:10941481-10949266 (-) |  | 0.7990 | 9.54e-23 | 4.95e-22 |
| ENSMUSG00000022507 | 1810013L24Rik | protein\_coding | 16:8830100-8858922 (+) |  | -0.5930 | 9.55e-23 | 4.95e-22 |
| ENSMUSG00000031666 | Rbl2 | protein\_coding | 8:91070057-91123844 (+) |  | -0.5240 | 9.59e-23 | 4.97e-22 |
| ENSMUSG00000042719 | Naa25 | protein\_coding | 5:121397936-121444378 (+) |  | 0.5450 | 9.81e-23 | 5.09e-22 |
| ENSMUSG00000031715 | Smarca5 | protein\_coding | 8:80698507-80739497 (-) |  | 0.5000 | 9.96e-23 | 5.16e-22 |
| ENSMUSG00000024079 | Eif2ak2 | protein\_coding | 17:78852564-78882573 (-) |  | -0.7340 | 1.02e-22 | 5.26e-22 |
| ENSMUSG00000056290 | Ms4a4b | protein\_coding | 19:11443553-11467055 (+) |  | -0.9250 | 1.05e-22 | 5.41e-22 |
| ENSMUSG00000017499 | Cdc6 | protein\_coding | 11:98907801-98923940 (+) |  | 1.1700 | 1.07e-22 | 5.56e-22 |
| ENSMUSG00000111034 | Gm48066 | lncRNA | 10:44647518-44662496 (+) |  | -3.4800 | 1.09e-22 | 5.62e-22 |
| ENSMUSG00000001128 | Cfp | protein\_coding | X:20925454-20931555 (-) |  | -0.6380 | 1.10e-22 | 5.69e-22 |
| ENSMUSG00000070461 | 9230112E08Rik | lncRNA | 2:180983924-180987275 (+) |  | -0.9900 | 1.12e-22 | 5.78e-22 |
| ENSMUSG00000047507 | Baiap3 | protein\_coding | 17:25242659-25256364 (-) |  | 3.2300 | 1.12e-22 | 5.78e-22 |
| ENSMUSG00000038872 | Zfhx3 | protein\_coding | 8:107942644-108961630 (+) |  | -1.2600 | 1.13e-22 | 5.86e-22 |
| ENSMUSG00000027806 | Tsc22d2 | protein\_coding | 3:58414715-58466783 (+) |  | -0.6050 | 1.18e-22 | 6.10e-22 |
| ENSMUSG00000047434 | Xxylt1 | protein\_coding | 16:30955144-31081432 (-) |  | 0.6050 | 1.22e-22 | 6.30e-22 |
| ENSMUSG00000036006 | Ripor2 | protein\_coding | 13:24501525-24733816 (+) |  | -0.6180 | 1.22e-22 | 6.31e-22 |
| ENSMUSG00000006585 | Cdt1 | protein\_coding | 8:122568015-122573554 (+) |  | 0.8530 | 1.23e-22 | 6.36e-22 |
| ENSMUSG00000000751 | Rpa1 | protein\_coding | 11:75298166-75348324 (-) |  | 0.6470 | 1.24e-22 | 6.42e-22 |
| ENSMUSG00000038604 | Ripor1 | protein\_coding | 8:105605255-105622219 (+) |  | 0.8380 | 1.26e-22 | 6.52e-22 |
| ENSMUSG00000022964 | Tmem50b | protein\_coding | 16:91574503-91597800 (-) |  | -0.4960 | 1.32e-22 | 6.81e-22 |
| ENSMUSG00000031023 | Akip1 | protein\_coding | 7:109703690-109712189 (+) |  | 0.8650 | 1.35e-22 | 6.98e-22 |
| ENSMUSG00000044461 | Shisa2 | protein\_coding | 14:59625308-59631660 (+) |  | 3.7600 | 1.38e-22 | 7.13e-22 |
| ENSMUSG00000024424 | Ttc39c | protein\_coding | 18:12599896-12738863 (+) |  | -0.6250 | 1.40e-22 | 7.23e-22 |
| ENSMUSG00000032519 | Slc25a38 | protein\_coding | 9:120110374-120124504 (+) |  | -0.7990 | 1.42e-22 | 7.29e-22 |
| ENSMUSG00000026566 | Mpzl1 | protein\_coding | 1:165592240-165634538 (-) |  | 2.5800 | 1.44e-22 | 7.39e-22 |
| ENSMUSG00000031985 | Gnpat | protein\_coding | 8:124863033-124890057 (+) |  | 0.5240 | 1.46e-22 | 7.52e-22 |
| ENSMUSG00000055200 | Sertad3 | protein\_coding | 7:27473768-27477364 (+) |  | -1.0500 | 1.47e-22 | 7.54e-22 |
| ENSMUSG00000030007 | Cct7 | protein\_coding | 6:85451514-85468475 (+) |  | 0.5190 | 1.47e-22 | 7.58e-22 |
| ENSMUSG00000028702 | Rad54l | protein\_coding | 4:116094264-116123690 (-) |  | 0.9380 | 1.52e-22 | 7.79e-22 |
| ENSMUSG00000070730 | Rmdn3 | protein\_coding | 2:119137001-119157034 (-) |  | 0.5100 | 1.52e-22 | 7.79e-22 |
| ENSMUSG00000044338 | Aplnr | protein\_coding | 2:85136225-85139923 (+) |  | -3.1000 | 1.53e-22 | 7.84e-22 |
| ENSMUSG00000044197 | Gpr146 | protein\_coding | 5:139377697-139396415 (+) |  | -0.9510 | 1.53e-22 | 7.85e-22 |
| ENSMUSG00000045928 | 4933440M02Rik | lncRNA | 7:125284025-125349788 (-) |  | 7.2900 | 1.58e-22 | 8.09e-22 |
| ENSMUSG00000039231 | Suv39h1 | protein\_coding | X:8061171-8074760 (-) |  | 0.6310 | 1.62e-22 | 8.32e-22 |
| ENSMUSG00000052155 | Acvr2a | protein\_coding | 2:48814109-48903269 (+) |  | -0.9720 | 1.63e-22 | 8.36e-22 |
| ENSMUSG00000018848 | Rars | protein\_coding | 11:35808381-35834506 (-) |  | 0.4900 | 1.63e-22 | 8.37e-22 |
| ENSMUSG00000027221 | Chst1 | protein\_coding | 2:92599707-92615250 (+) |  | -2.2200 | 1.66e-22 | 8.51e-22 |
| ENSMUSG00000007891 | Ctsd | protein\_coding | 7:142375911-142388038 (-) |  | -0.6260 | 1.77e-22 | 9.05e-22 |
| ENSMUSG00000002396 | Ocel1 | protein\_coding | 8:71371298-71379361 (+) |  | -0.6330 | 1.77e-22 | 9.08e-22 |
| ENSMUSG00000002844 | Adprh | protein\_coding | 16:38444030-38452703 (-) |  | -0.5290 | 1.81e-22 | 9.26e-22 |
| ENSMUSG00000033364 | Usp37 | protein\_coding | 1:74435511-74544284 (-) |  | 0.7390 | 1.81e-22 | 9.27e-22 |
| ENSMUSG00000024902 | Mrpl11 | protein\_coding | 19:4962147-4966999 (+) |  | 0.7000 | 1.91e-22 | 9.77e-22 |
| ENSMUSG00000026356 | Dars | protein\_coding | 1:128363707-128417368 (-) |  | 0.7060 | 1.96e-22 | 1.00e-21 |
| ENSMUSG00000019768 | Esr1 | protein\_coding | 10:4611593-5005614 (+) |  | 0.9490 | 1.98e-22 | 1.01e-21 |
| ENSMUSG00000024993 | Fam45a | protein\_coding | 19:60811585-60836227 (+) |  | -0.7790 | 1.98e-22 | 1.01e-21 |
| ENSMUSG00000024665 | Fads2 | protein\_coding | 19:10062765-10101746 (-) |  | 2.7000 | 2.01e-22 | 1.03e-21 |
| ENSMUSG00000060568 | Fam78b | protein\_coding | 1:167001417-167091302 (+) |  | -2.5600 | 2.07e-22 | 1.06e-21 |
| ENSMUSG00000024197 | Plin3 | protein\_coding | 17:56277476-56292873 (-) |  | -0.5130 | 2.09e-22 | 1.07e-21 |
| ENSMUSG00000020571 | Pdia6 | protein\_coding | 12:17266545-17284770 (+) |  | 0.7060 | 2.12e-22 | 1.08e-21 |
| ENSMUSG00000028289 | Epha7 | protein\_coding | 4:28813131-28967499 (+) |  | 2.7000 | 2.14e-22 | 1.09e-21 |
| ENSMUSG00000028284 | Map3k7 | protein\_coding | 4:31964097-32023467 (+) |  | -0.3730 | 2.18e-22 | 1.11e-21 |
| ENSMUSG00000020422 | Tns3 | protein\_coding | 11:8431652-8664681 (-) |  | -0.5460 | 2.23e-22 | 1.14e-21 |
| ENSMUSG00000013155 | Enkd1 | protein\_coding | 8:105703651-105708210 (-) |  | 0.8160 | 2.32e-22 | 1.18e-21 |
| ENSMUSG00000024726 | Carnmt1 | protein\_coding | 19:18670764-18707200 (+) |  | 0.8250 | 2.33e-22 | 1.19e-21 |
| ENSMUSG00000052997 | Uba2 | protein\_coding | 7:34140688-34169599 (-) |  | 0.4210 | 2.35e-22 | 1.20e-21 |
| ENSMUSG00000020788 | Atp2a3 | protein\_coding | 11:72961169-72993044 (+) |  | -0.6650 | 2.36e-22 | 1.20e-21 |
| ENSMUSG00000026425 | Srgap2 | protein\_coding | 1:131285251-131527352 (-) |  | -0.7800 | 2.37e-22 | 1.21e-21 |
| ENSMUSG00000027502 | Rtf2 | protein\_coding | 2:172440556-172469908 (+) |  | -0.4220 | 2.42e-22 | 1.23e-21 |
| ENSMUSG00000030431 | Tmem238 | protein\_coding | 7:4784557-4789656 (-) |  | 1.2500 | 2.48e-22 | 1.26e-21 |
| ENSMUSG00000078238 | Gm12854 | processed\_pseudogene | 4:116067268-116067564 (+) |  | -0.9730 | 2.51e-22 | 1.28e-21 |
| ENSMUSG00000033326 | Kdm4a | protein\_coding | 4:118136957-118180043 (-) |  | -0.5230 | 2.55e-22 | 1.30e-21 |
| ENSMUSG00000028015 | Ctso | protein\_coding | 3:81932601-81956725 (+) |  | -0.8570 | 2.58e-22 | 1.31e-21 |
| ENSMUSG00000073987 | Ggh | protein\_coding | 4:20042052-20066750 (+) |  | -1.0500 | 2.60e-22 | 1.32e-21 |
| ENSMUSG00000025060 | Slk | protein\_coding | 19:47579678-47645246 (+) |  | -0.4890 | 2.63e-22 | 1.34e-21 |
| ENSMUSG00000034382 | AI661453 | protein\_coding | 17:47436615-47470638 (+) |  | 2.0300 | 2.67e-22 | 1.36e-21 |
| ENSMUSG00000042148 | Cox10 | protein\_coding | 11:63962627-64079468 (-) |  | 0.7140 | 2.71e-22 | 1.38e-21 |
| ENSMUSG00000024818 | Slc25a45 | protein\_coding | 19:5877808-5885878 (+) |  | -0.5960 | 2.76e-22 | 1.40e-21 |
| ENSMUSG00000030042 | Pole4 | protein\_coding | 6:82618992-82705365 (-) |  | 0.6450 | 2.86e-22 | 1.45e-21 |
| ENSMUSG00000045273 | Cenph | protein\_coding | 13:100759674-100775899 (-) |  | 0.8710 | 2.89e-22 | 1.47e-21 |
| ENSMUSG00000026656 | Fcgr2b | protein\_coding | 1:170958617-170976547 (-) |  | -0.7770 | 3.01e-22 | 1.53e-21 |
| ENSMUSG00000042312 | S100a13 | protein\_coding | 3:90514435-90524581 (+) |  | -0.7570 | 3.05e-22 | 1.54e-21 |
| ENSMUSG00000031659 | Adcy7 | protein\_coding | 8:88272403-88329962 (+) |  | -0.5040 | 3.22e-22 | 1.63e-21 |
| ENSMUSG00000022102 | Dok2 | protein\_coding | 14:70766036-70778495 (+) |  | -1.2400 | 3.35e-22 | 1.70e-21 |
| ENSMUSG00000019961 | Tmpo | protein\_coding | 10:91147571-91181315 (-) |  | -0.4850 | 3.48e-22 | 1.76e-21 |
| ENSMUSG00000041912 | Tdrkh | protein\_coding | 3:94413273-94434668 (+) |  | 1.3500 | 3.70e-22 | 1.87e-21 |
| ENSMUSG00000020894 | Vamp2 | protein\_coding | 11:69088490-69092384 (+) |  | -0.7290 | 3.78e-22 | 1.91e-21 |
| ENSMUSG00000024597 | Slc12a2 | protein\_coding | 18:57878678-57946821 (+) |  | 1.5700 | 3.80e-22 | 1.92e-21 |
| ENSMUSG00000019362 | D8Ertd738e | protein\_coding | 8:84246238-84249719 (-) |  | -0.4830 | 3.81e-22 | 1.93e-21 |
| ENSMUSG00000036743 | Psma8 | protein\_coding | 18:14706151-14762301 (+) |  | 1.6400 | 4.04e-22 | 2.04e-21 |
| ENSMUSG00000031540 | Kat6a | protein\_coding | 8:22859535-22943259 (+) |  | -0.5560 | 4.14e-22 | 2.09e-21 |
| ENSMUSG00000040723 | Rcsd1 | protein\_coding | 1:165646516-165709757 (-) |  | -0.3120 | 4.18e-22 | 2.11e-21 |
| ENSMUSG00000044252 | Osbpl1a | protein\_coding | 18:12755314-12941841 (-) |  | 0.7650 | 4.22e-22 | 2.13e-21 |
| ENSMUSG00000063320 | 1190007I07Rik | protein\_coding | 10:82619851-82623228 (-) |  | 1.3500 | 4.38e-22 | 2.21e-21 |
| ENSMUSG00000006720 | Zfp184 | protein\_coding | 13:21945094-21960779 (+) |  | 4.9800 | 4.40e-22 | 2.22e-21 |
| ENSMUSG00000024213 | Nudt3 | protein\_coding | 17:27579382-27623495 (-) |  | 0.6060 | 4.59e-22 | 2.31e-21 |
| ENSMUSG00000068264 | Ap5s1 | protein\_coding | 2:131207078-131213514 (+) |  | -1.0300 | 4.82e-22 | 2.43e-21 |
| ENSMUSG00000025466 | Fuom | protein\_coding | 7:140096770-140102441 (-) |  | -1.1900 | 4.89e-22 | 2.47e-21 |
| ENSMUSG00000018425 | Dhx40 | protein\_coding | 11:86768846-86807746 (-) |  | -0.5720 | 4.90e-22 | 2.47e-21 |
| ENSMUSG00000023393 | Slc17a9 | protein\_coding | 2:180725263-180742280 (+) |  | 1.0900 | 5.05e-22 | 2.54e-21 |
| ENSMUSG00000042410 | Agps | protein\_coding | 2:75832177-75931350 (+) |  | 0.4180 | 5.12e-22 | 2.58e-21 |
| ENSMUSG00000036990 | Otud4 | protein\_coding | 8:79639618-79677724 (+) |  | 0.6610 | 5.12e-22 | 2.58e-21 |
| ENSMUSG00000056665 | Them6 | protein\_coding | 15:74721204-74724639 (+) |  | 1.1500 | 5.21e-22 | 2.62e-21 |
| ENSMUSG00000043059 | Zfp513 | protein\_coding | 5:31198981-31202303 (-) |  | -0.5800 | 5.24e-22 | 2.64e-21 |
| ENSMUSG00000101431 | Gm7901 | processed\_pseudogene | 8:75711600-75713196 (-) |  | 2.4100 | 5.36e-22 | 2.69e-21 |
| ENSMUSG00000073405 | H2-T-ps | unprocessed\_pseudogene | 17:36109234-36111658 (-) |  | -1.2000 | 5.40e-22 | 2.71e-21 |
| ENSMUSG00000017550 | Atad5 | protein\_coding | 11:80089400-80135794 (+) |  | 0.8790 | 5.60e-22 | 2.82e-21 |
| ENSMUSG00000022972 | Cfap298 | protein\_coding | 16:90925809-90935114 (-) |  | 1.3900 | 5.70e-22 | 2.86e-21 |
| ENSMUSG00000037306 | Man1c1 | protein\_coding | 4:134561690-134704290 (-) |  | -0.6860 | 5.75e-22 | 2.89e-21 |
| ENSMUSG00000035062 | Zc4h2 | protein\_coding | X:95639193-95658509 (-) |  | 1.4700 | 5.84e-22 | 2.93e-21 |
| ENSMUSG00000038811 | Gngt2 | protein\_coding | 11:95837216-95845734 (+) |  | -2.0000 | 6.10e-22 | 3.06e-21 |
| ENSMUSG00000038500 | Prr3 | protein\_coding | 17:35972541-35980236 (-) |  | 0.8810 | 6.12e-22 | 3.07e-21 |
| ENSMUSG00000040848 | Sft2d2 | protein\_coding | 1:165174337-165194438 (-) |  | -0.5690 | 6.21e-22 | 3.11e-21 |
| ENSMUSG00000045671 | Spred2 | protein\_coding | 11:19924375-20024026 (+) |  | 1.1300 | 6.22e-22 | 3.12e-21 |
| ENSMUSG00000042606 | Hirip3 | protein\_coding | 7:126861972-126865377 (+) |  | 0.6100 | 6.26e-22 | 3.14e-21 |
| ENSMUSG00000051727 | Kctd14 | protein\_coding | 7:97451323-97459557 (+) |  | 1.5500 | 6.28e-22 | 3.15e-21 |
| ENSMUSG00000034021 | Pds5b | protein\_coding | 5:150673739-150810690 (+) |  | 0.4370 | 6.37e-22 | 3.19e-21 |
| ENSMUSG00000097705 | Gm26740 | lncRNA | 10:22187013-22302835 (-) |  | -1.1400 | 6.39e-22 | 3.20e-21 |
| ENSMUSG00000020440 | Arf5 | protein\_coding | 6:28423560-28426602 (+) |  | -0.7390 | 6.45e-22 | 3.23e-21 |
| ENSMUSG00000001829 | Clpb | protein\_coding | 7:101663633-101795506 (+) |  | 0.6860 | 6.53e-22 | 3.26e-21 |
| ENSMUSG00000078670 | Fam174b | protein\_coding | 7:73740302-73776919 (+) |  | 4.1200 | 7.20e-22 | 3.60e-21 |
| ENSMUSG00000116380 | Gm39556 | lncRNA | 15:74929437-74936316 (+) |  | -2.1100 | 7.34e-22 | 3.67e-21 |
| ENSMUSG00000031490 | Eif4ebp1 | protein\_coding | 8:27260329-27276674 (+) |  | -0.5900 | 7.34e-22 | 3.67e-21 |
| ENSMUSG00000025967 | Eef1b2 | protein\_coding | 1:63176825-63180486 (+) |  | 0.6360 | 7.54e-22 | 3.77e-21 |
| ENSMUSG00000022558 | Mroh1 | protein\_coding | 15:76380261-76453038 (+) |  | -0.6280 | 7.58e-22 | 3.79e-21 |
| ENSMUSG00000029394 | Cdk2ap1 | protein\_coding | 5:124345417-124363082 (-) |  | 0.5410 | 7.80e-22 | 3.90e-21 |
| ENSMUSG00000021371 | Mcur1 | protein\_coding | 13:43538393-43560191 (-) |  | -0.5550 | 7.89e-22 | 3.94e-21 |
| ENSMUSG00000020592 | Sdc1 | protein\_coding | 12:8771323-8793715 (+) |  | 1.9700 | 7.99e-22 | 3.98e-21 |
| ENSMUSG00000028603 | Scp2 | protein\_coding | 4:108043839-108144998 (-) |  | -0.6630 | 8.02e-22 | 4.00e-21 |
| ENSMUSG00000031864 | Ints10 | protein\_coding | 8:68793929-68831667 (+) |  | 0.4370 | 8.05e-22 | 4.01e-21 |
| ENSMUSG00000068328 | Aup1 | protein\_coding | 6:83054521-83057808 (+) |  | -0.3630 | 8.31e-22 | 4.14e-21 |
| ENSMUSG00000033610 | Pank1 | protein\_coding | 19:34806940-34879455 (-) |  | 0.8620 | 8.47e-22 | 4.22e-21 |
| ENSMUSG00000002997 | Prkar2b | protein\_coding | 12:31958476-32061296 (-) |  | 0.4070 | 8.61e-22 | 4.29e-21 |
| ENSMUSG00000022667 | Cd200r1 | protein\_coding | 16:44765736-44794978 (+) |  | -1.2500 | 8.67e-22 | 4.32e-21 |
| ENSMUSG00000020743 | Mif4gd | protein\_coding | 11:115607918-115612969 (-) |  | -0.5490 | 9.03e-22 | 4.50e-21 |
| ENSMUSG00000024867 | Pip5k1b | protein\_coding | 19:24294794-24555872 (-) |  | 2.6900 | 9.24e-22 | 4.60e-21 |
| ENSMUSG00000033272 | Slc35a4 | protein\_coding | 18:36679215-36683861 (+) |  | 0.6190 | 9.29e-22 | 4.62e-21 |
| ENSMUSG00000044533 | Rps2 | protein\_coding | 17:24718116-24721929 (+) |  | 0.7580 | 9.36e-22 | 4.66e-21 |
| ENSMUSG00000042570 | Mier2 | protein\_coding | 10:79540245-79555199 (-) |  | 0.6500 | 9.78e-22 | 4.86e-21 |
| ENSMUSG00000027198 | Ext2 | protein\_coding | 2:93661028-93822568 (-) |  | 0.4720 | 1.01e-21 | 5.02e-21 |
| ENSMUSG00000013698 | Pea15a | protein\_coding | 1:172196728-172206804 (-) |  | -0.7270 | 1.02e-21 | 5.08e-21 |
| ENSMUSG00000047514 | Tspyl1 | protein\_coding | 10:34282190-34285275 (+) |  | -0.4720 | 1.03e-21 | 5.12e-21 |
| ENSMUSG00000002668 | Dennd1c | protein\_coding | 17:57065905-57078514 (-) |  | -1.1900 | 1.04e-21 | 5.19e-21 |
| ENSMUSG00000036561 | Ppp6r2 | protein\_coding | 15:89211553-89287010 (+) |  | -0.7910 | 1.08e-21 | 5.34e-21 |
| ENSMUSG00000113836 | Gm3325 | transcribed\_processed\_pseudogene | 13:62015605-62050986 (+) |  | 1.0600 | 1.08e-21 | 5.34e-21 |
| ENSMUSG00000002608 | Ccdc97 | protein\_coding | 7:25711106-25719088 (-) |  | -0.6170 | 1.08e-21 | 5.38e-21 |
| ENSMUSG00000034928 | Rnf44 | protein\_coding | 13:54679399-54693907 (-) |  | -0.4740 | 1.09e-21 | 5.40e-21 |
| ENSMUSG00000108393 | Gm32633 | lncRNA | 7:64001885-64020959 (+) |  | -1.9100 | 1.09e-21 | 5.42e-21 |
| ENSMUSG00000046688 | Tifa | protein\_coding | 3:127789805-127832164 (+) |  | -0.6370 | 1.11e-21 | 5.52e-21 |
| ENSMUSG00000036111 | Lmo1 | protein\_coding | 7:109138572-109175207 (-) |  | 0.8020 | 1.21e-21 | 5.98e-21 |
| ENSMUSG00000042870 | Tom1 | protein\_coding | 8:75033705-75070121 (+) |  | -0.5310 | 1.21e-21 | 6.01e-21 |
| ENSMUSG00000037608 | Bclaf1 | protein\_coding | 10:20312469-20344613 (+) |  | 0.5380 | 1.23e-21 | 6.08e-21 |
| ENSMUSG00000038780 | Smurf1 | protein\_coding | 5:144876495-144965847 (-) |  | -0.7470 | 1.29e-21 | 6.38e-21 |
| ENSMUSG00000024664 | Fads3 | protein\_coding | 19:10041569-10060110 (+) |  | 2.0900 | 1.30e-21 | 6.41e-21 |
| ENSMUSG00000031821 | Gins2 | protein\_coding | 8:120578633-120589304 (-) |  | 0.9420 | 1.30e-21 | 6.42e-21 |
| ENSMUSG00000059316 | Slc27a4 | protein\_coding | 2:29802634-29817522 (+) |  | -0.5000 | 1.31e-21 | 6.46e-21 |
| ENSMUSG00000039236 | Isg20 | protein\_coding | 7:78913424-78920396 (+) |  | -2.0100 | 1.47e-21 | 7.27e-21 |
| ENSMUSG00000076441 | Ass1 | protein\_coding | 2:31470207-31520672 (+) |  | 0.7670 | 1.48e-21 | 7.30e-21 |
| ENSMUSG00000029217 | Tec | protein\_coding | 5:72755716-72868483 (-) |  | -0.7790 | 1.53e-21 | 7.57e-21 |
| ENSMUSG00000030452 | Nipa2 | protein\_coding | 7:55931287-55962476 (-) |  | -0.4200 | 1.56e-21 | 7.72e-21 |
| ENSMUSG00000040025 | Ythdf2 | protein\_coding | 4:132184912-132212303 (-) |  | 0.3540 | 1.57e-21 | 7.76e-21 |
| ENSMUSG00000019139 | Isyna1 | protein\_coding | 8:70594373-70597290 (+) |  | 0.6410 | 1.62e-21 | 7.98e-21 |
| ENSMUSG00000038843 | Gcnt1 | protein\_coding | 19:17326141-17372844 (-) |  | -0.6530 | 1.64e-21 | 8.09e-21 |
| ENSMUSG00000032612 | Usp4 | protein\_coding | 9:108347853-108392545 (+) |  | -0.3270 | 1.65e-21 | 8.12e-21 |
| ENSMUSG00000028523 | Tctex1d1 | protein\_coding | 4:102978606-103005594 (+) |  | 3.4700 | 1.66e-21 | 8.19e-21 |
| ENSMUSG00000007613 | Tgfbr1 | protein\_coding | 4:47353222-47414931 (+) |  | -0.5070 | 1.71e-21 | 8.43e-21 |
| ENSMUSG00000003458 | Ncstn | protein\_coding | 1:172066013-172082795 (-) |  | -0.3580 | 1.73e-21 | 8.52e-21 |
| ENSMUSG00000063317 | Usp31 | protein\_coding | 7:121642021-121707253 (-) |  | 0.6480 | 1.76e-21 | 8.66e-21 |
| ENSMUSG00000067194 | Eif1ax | protein\_coding | X:159372178-159389928 (+) |  | 0.6710 | 1.77e-21 | 8.72e-21 |
| ENSMUSG00000028430 | Nol6 | protein\_coding | 4:41114427-41124455 (-) |  | 0.7370 | 1.78e-21 | 8.74e-21 |
| ENSMUSG00000042073 | Abhd14b | protein\_coding | 9:106447459-106452923 (+) |  | 1.5000 | 1.78e-21 | 8.76e-21 |
| ENSMUSG00000056116 | H2-T22 | protein\_coding | 17:36037128-36042747 (-) |  | -0.6260 | 1.84e-21 | 9.05e-21 |
| ENSMUSG00000029859 | Epha1 | protein\_coding | 6:42358487-42373268 (-) |  | -1.9700 | 1.88e-21 | 9.23e-21 |
| ENSMUSG00000036246 | Gmip | protein\_coding | 8:69808679-69821870 (+) |  | -0.5300 | 1.88e-21 | 9.23e-21 |
| ENSMUSG00000026581 | Sell | protein\_coding | 1:164061982-164084181 (+) |  | -0.6630 | 2.00e-21 | 9.84e-21 |
| ENSMUSG00000028524 | Sgip1 | protein\_coding | 4:102741297-102973628 (+) |  | 2.5400 | 2.00e-21 | 9.84e-21 |
| ENSMUSG00000028088 | Fmo5 | protein\_coding | 3:97628804-97655282 (+) |  | -1.3800 | 2.02e-21 | 9.91e-21 |
| ENSMUSG00000026072 | Il1r1 | protein\_coding | 1:40225080-40317257 (+) |  | 2.6500 | 2.06e-21 | 1.01e-20 |
| ENSMUSG00000039849 | Pcif1 | protein\_coding | 2:164879304-164894454 (+) |  | -0.3840 | 2.10e-21 | 1.03e-20 |
| ENSMUSG00000034714 | Ttyh2 | protein\_coding | 11:114675431-114720977 (+) |  | 1.8100 | 2.13e-21 | 1.05e-20 |
| ENSMUSG00000071632 | 2510002D24Rik | protein\_coding | 16:18836578-18840160 (+) |  | 1.3400 | 2.24e-21 | 1.10e-20 |
| ENSMUSG00000070002 | Ell | protein\_coding | 8:70539457-70592858 (+) |  | -0.5800 | 2.24e-21 | 1.10e-20 |
| ENSMUSG00000049971 | Glt1d1 | protein\_coding | 5:127632262-127709374 (+) |  | 1.8300 | 2.32e-21 | 1.14e-20 |
| ENSMUSG00000047675 | Rps8 | protein\_coding | 4:117153827-117156243 (-) |  | 0.5100 | 2.33e-21 | 1.14e-20 |
| ENSMUSG00000035949 | Fbxw2 | protein\_coding | 2:34804514-34826311 (-) |  | -0.3630 | 2.35e-21 | 1.15e-20 |
| ENSMUSG00000002949 | Timm44 | protein\_coding | 8:4259731-4275913 (-) |  | 0.4350 | 2.40e-21 | 1.17e-20 |
| ENSMUSG00000035385 | Ccl2 | protein\_coding | 11:82035571-82037453 (+) |  | -2.7900 | 2.44e-21 | 1.19e-20 |
| ENSMUSG00000031858 | Mau2 | protein\_coding | 8:70016123-70042734 (-) |  | -0.4520 | 2.52e-21 | 1.23e-20 |
| ENSMUSG00000061759 | Armt1 | protein\_coding | 10:4432467-4455141 (+) |  | 0.4390 | 2.66e-21 | 1.30e-20 |
| ENSMUSG00000001082 | Mfsd10 | protein\_coding | 5:34633642-34637212 (-) |  | 0.6460 | 2.66e-21 | 1.30e-20 |
| ENSMUSG00000027001 | Dusp19 | protein\_coding | 2:80617045-80632361 (+) |  | 1.2200 | 2.73e-21 | 1.33e-20 |
| ENSMUSG00000022378 | Fam49b | protein\_coding | 15:63929097-64060478 (-) |  | -0.7260 | 2.83e-21 | 1.38e-20 |
| ENSMUSG00000056050 | Mia3 | protein\_coding | 1:183326725-183369553 (-) |  | -0.4900 | 2.99e-21 | 1.46e-20 |
| ENSMUSG00000106820 | D5Ertd605e | lncRNA | 5:147418601-147423044 (+) |  | 1.8500 | 3.02e-21 | 1.48e-20 |
| ENSMUSG00000031513 | Leprotl1 | protein\_coding | 8:34135572-34147033 (-) |  | -0.4160 | 3.14e-21 | 1.53e-20 |
| ENSMUSG00000037287 | Tbcel | protein\_coding | 9:42412316-42507809 (-) |  | -0.5580 | 3.16e-21 | 1.54e-20 |
| ENSMUSG00000017776 | Crk | protein\_coding | 11:75679259-75706908 (+) |  | -0.5710 | 3.20e-21 | 1.56e-20 |
| ENSMUSG00000085761 | 4930455G09Rik | lncRNA | 4:142017898-142028996 (+) |  | -2.2300 | 3.22e-21 | 1.57e-20 |
| ENSMUSG00000049659 | Aftph | protein\_coding | 11:20685084-20741589 (-) |  | -0.4280 | 3.29e-21 | 1.60e-20 |
| ENSMUSG00000016319 | Slc25a5 | protein\_coding | X:36795651-36798807 (+) |  | 0.4400 | 3.31e-21 | 1.61e-20 |
| ENSMUSG00000041117 | Ccdc8 | protein\_coding | 7:16992708-16997516 (+) |  | 4.6800 | 3.37e-21 | 1.64e-20 |
| ENSMUSG00000050930 | Map10 | protein\_coding | 8:125669818-125673359 (+) |  | -1.0700 | 3.43e-21 | 1.67e-20 |
| ENSMUSG00000032514 | Ttc21a | protein\_coding | 9:119937606-119967793 (+) |  | 2.8300 | 3.44e-21 | 1.67e-20 |
| ENSMUSG00000048895 | Cdk5r1 | protein\_coding | 11:80477023-80481184 (+) |  | 1.1200 | 3.50e-21 | 1.70e-20 |
| ENSMUSG00000041354 | Rgl2 | protein\_coding | 17:33929543-33937687 (+) |  | -0.4320 | 3.51e-21 | 1.71e-20 |
| ENSMUSG00000029822 | Osbpl3 | protein\_coding | 6:50293330-50456201 (-) |  | 0.6610 | 3.69e-21 | 1.79e-20 |
| ENSMUSG00000080845 | Gm9115 | processed\_pseudogene | X:102724402-102725749 (-) |  | 2.6100 | 3.74e-21 | 1.82e-20 |
| ENSMUSG00000030142 | Clec4e | protein\_coding | 6:123281789-123289870 (-) |  | -2.1300 | 3.78e-21 | 1.84e-20 |
| ENSMUSG00000032417 | Rwdd2a | protein\_coding | 9:86571991-86574899 (+) |  | -1.0800 | 3.88e-21 | 1.88e-20 |
| ENSMUSG00000021643 | Serf1 | protein\_coding | 13:100108012-100114571 (+) |  | 2.3600 | 3.91e-21 | 1.90e-20 |
| ENSMUSG00000032058 | Ppp2r1b | protein\_coding | 9:50845301-50894229 (+) |  | 0.4930 | 3.92e-21 | 1.90e-20 |
| ENSMUSG00000017607 | Tns4 | protein\_coding | 11:99065678-99089306 (-) |  | -0.7400 | 3.98e-21 | 1.93e-20 |
| ENSMUSG00000056214 | Pard6g | protein\_coding | 18:80046890-80119639 (+) |  | 2.6200 | 4.07e-21 | 1.98e-20 |
| ENSMUSG00000042286 | Stab1 | protein\_coding | 14:31139013-31168641 (-) |  | -3.0600 | 4.11e-21 | 2.00e-20 |
| ENSMUSG00000045868 | Gvin1 | protein\_coding | 7:106156556-106215326 (-) |  | -1.1100 | 4.17e-21 | 2.02e-20 |
| ENSMUSG00000020116 | Pno1 | protein\_coding | 11:17203198-17211568 (-) |  | 0.9520 | 4.25e-21 | 2.06e-20 |
| ENSMUSG00000033020 | Polr2f | protein\_coding | 15:79141009-79151774 (+) |  | 0.5920 | 4.28e-21 | 2.07e-20 |
| ENSMUSG00000042817 | Flt3 | protein\_coding | 5:147330741-147400489 (-) |  | 2.0700 | 4.28e-21 | 2.07e-20 |
| ENSMUSG00000034329 | Brip1 | protein\_coding | 11:86058138-86201193 (-) |  | 0.5230 | 4.41e-21 | 2.14e-20 |
| ENSMUSG00000039195 | 1110008P14Rik | protein\_coding | 2:32377097-32381938 (-) |  | -0.9510 | 4.59e-21 | 2.22e-20 |
| ENSMUSG00000090307 | 1700071M16Rik | lncRNA | 17:43588340-43592442 (-) |  | -4.3700 | 4.76e-21 | 2.30e-20 |
| ENSMUSG00000066000 | Zfp979 | protein\_coding | 4:147611937-147642513 (-) |  | 2.6600 | 4.81e-21 | 2.33e-20 |
| ENSMUSG00000006050 | Sra1 | protein\_coding | 18:36666681-36670815 (-) |  | -0.5130 | 4.89e-21 | 2.37e-20 |
| ENSMUSG00000028854 | Slc9a1 | protein\_coding | 4:133369706-133423702 (+) |  | -0.5110 | 5.01e-21 | 2.42e-20 |
| ENSMUSG00000021694 | Ercc8 | protein\_coding | 13:108158731-108195364 (+) |  | 0.7370 | 5.01e-21 | 2.42e-20 |
| ENSMUSG00000032621 | Srek1 | protein\_coding | 13:103739348-103774608 (-) |  | 0.5250 | 5.10e-21 | 2.47e-20 |
| ENSMUSG00000032218 | Ccnb2 | protein\_coding | 9:70407692-70421547 (-) |  | -0.5900 | 5.17e-21 | 2.50e-20 |
| ENSMUSG00000020079 | Supv3l1 | protein\_coding | 10:62429209-62449738 (-) |  | 0.7710 | 5.24e-21 | 2.53e-20 |
| ENSMUSG00000020883 | Fbxl20 | protein\_coding | 11:98082556-98150403 (-) |  | -0.9430 | 5.25e-21 | 2.53e-20 |
| ENSMUSG00000030577 | Cd22 | protein\_coding | 7:30865402-30880342 (-) |  | 2.2700 | 5.30e-21 | 2.56e-20 |
| ENSMUSG00000037349 | Nudt22 | protein\_coding | 19:6993018-6996117 (-) |  | -0.7510 | 5.76e-21 | 2.78e-20 |
| ENSMUSG00000078606 | Gm4070 | protein\_coding | 7:105895139-105953967 (-) |  | -1.1000 | 6.05e-21 | 2.92e-20 |
| ENSMUSG00000045690 | Wdr89 | protein\_coding | 12:75630596-75669537 (-) |  | 1.5500 | 6.08e-21 | 2.93e-20 |
| ENSMUSG00000052833 | Sae1 | protein\_coding | 7:16320234-16387806 (-) |  | 0.5760 | 6.14e-21 | 2.96e-20 |
| ENSMUSG00000028793 | Rnf19b | protein\_coding | 4:129058271-129085886 (+) |  | -0.5320 | 6.33e-21 | 3.05e-20 |
| ENSMUSG00000070000 | Fcho1 | protein\_coding | 8:71708387-71725716 (-) |  | 0.5590 | 6.57e-21 | 3.17e-20 |
| ENSMUSG00000024050 | Wiz | protein\_coding | 17:32354055-32389439 (-) |  | 0.6210 | 6.61e-21 | 3.18e-20 |
| ENSMUSG00000025793 | Hgs | protein\_coding | 11:120467635-120483979 (+) |  | -0.4750 | 6.73e-21 | 3.24e-20 |
| ENSMUSG00000031112 | Stk26 | protein\_coding | X:50841047-50893097 (+) |  | 0.5100 | 6.74e-21 | 3.24e-20 |
| ENSMUSG00000024174 | Pot1b | protein\_coding | 17:55651951-55712628 (-) |  | -0.9120 | 6.77e-21 | 3.25e-20 |
| ENSMUSG00000031570 | Plpp5 | protein\_coding | 8:25720037-25724887 (+) |  | 0.6230 | 6.97e-21 | 3.35e-20 |
| ENSMUSG00000022377 | Asap1 | protein\_coding | 15:64086857-64382919 (-) |  | -0.4820 | 7.17e-21 | 3.45e-20 |
| ENSMUSG00000032411 | Tfdp2 | protein\_coding | 9:96196275-96323646 (+) |  | 0.6450 | 7.18e-21 | 3.45e-20 |
| ENSMUSG00000020444 | Guk1 | protein\_coding | 11:59183875-59192212 (-) |  | 0.7260 | 7.36e-21 | 3.54e-20 |
| ENSMUSG00000073705 | Cenps | protein\_coding | 4:149127121-149137629 (-) |  | 1.0200 | 7.49e-21 | 3.60e-20 |
| ENSMUSG00000004558 | Ndrg2 | protein\_coding | 14:51905271-51914158 (-) |  | 4.1600 | 7.59e-21 | 3.64e-20 |
| ENSMUSG00000050106 | Tmc8 | protein\_coding | 11:117782076-117793110 (+) |  | -0.9580 | 7.75e-21 | 3.72e-20 |
| ENSMUSG00000084106 | Gm6136 | processed\_pseudogene | 1:86908793-86909683 (-) |  | 0.4590 | 7.87e-21 | 3.78e-20 |
| ENSMUSG00000027787 | Nmd3 | protein\_coding | 3:69721985-69756373 (+) |  | 0.7700 | 7.94e-21 | 3.81e-20 |
| ENSMUSG00000030276 | Ttll3 | protein\_coding | 6:113389260-113414587 (+) |  | -1.1700 | 7.97e-21 | 3.82e-20 |
| ENSMUSG00000028868 | Wasf2 | protein\_coding | 4:133130505-133199756 (+) |  | -0.4800 | 8.01e-21 | 3.84e-20 |
| ENSMUSG00000022864 | D16Ertd472e | protein\_coding | 16:78540338-78576708 (-) |  | 0.8330 | 8.08e-21 | 3.87e-20 |
| ENSMUSG00000030272 | Camk1 | protein\_coding | 6:113334124-113343984 (-) |  | -0.9770 | 8.11e-21 | 3.88e-20 |
| ENSMUSG00000039652 | Cpeb3 | protein\_coding | 19:37021291-37208601 (-) |  | -2.5900 | 8.42e-21 | 4.03e-20 |
| ENSMUSG00000020392 | Cdkn2aipnl | protein\_coding | 11:51967661-51977334 (+) |  | 0.6630 | 8.55e-21 | 4.09e-20 |
| ENSMUSG00000022895 | Ets2 | protein\_coding | 16:95702075-95721051 (+) |  | 1.4600 | 8.55e-21 | 4.09e-20 |
| ENSMUSG00000070803 | Cited4 | protein\_coding | 4:120666572-120667820 (+) |  | 2.7300 | 8.85e-21 | 4.23e-20 |
| ENSMUSG00000022876 | Samsn1 | protein\_coding | 16:75858793-76022281 (-) |  | -1.0500 | 8.89e-21 | 4.25e-20 |
| ENSMUSG00000028937 | Acot7 | protein\_coding | 4:152178134-152271855 (+) |  | 0.4950 | 8.89e-21 | 4.25e-20 |
| ENSMUSG00000032217 | Rnf111 | protein\_coding | 9:70425424-70503725 (-) |  | -0.5100 | 8.98e-21 | 4.29e-20 |
| ENSMUSG00000106743 | Gm42847 | TEC | 5:33990510-33993062 (-) |  | -2.6200 | 9.11e-21 | 4.35e-20 |
| ENSMUSG00000024672 | Ms4a7 | protein\_coding | 19:11321039-11336146 (-) |  | -3.6600 | 9.48e-21 | 4.53e-20 |
| ENSMUSG00000026991 | Pkp4 | protein\_coding | 2:59160850-59355208 (+) |  | 0.6560 | 9.49e-21 | 4.53e-20 |
| ENSMUSG00000054021 | Sirt5 | protein\_coding | 13:43365496-43395203 (+) |  | 1.9400 | 9.55e-21 | 4.56e-20 |
| ENSMUSG00000021809 | Nudt13 | protein\_coding | 14:20294697-20317578 (+) |  | -0.7170 | 9.66e-21 | 4.61e-20 |
| ENSMUSG00000018068 | Ints2 | protein\_coding | 11:86210681-86257575 (-) |  | 0.6310 | 1.03e-20 | 4.90e-20 |
| ENSMUSG00000032803 | Cdv3 | protein\_coding | 9:103353094-103365840 (-) |  | 0.5040 | 1.04e-20 | 4.96e-20 |
| ENSMUSG00000022469 | Rapgef3 | protein\_coding | 15:97744770-97767972 (-) |  | 7.1300 | 1.11e-20 | 5.27e-20 |
| ENSMUSG00000039768 | Dnajc11 | protein\_coding | 4:151933691-151982137 (+) |  | 0.4780 | 1.12e-20 | 5.35e-20 |
| ENSMUSG00000047221 | Fam185a | protein\_coding | 5:21424958-21482124 (+) |  | 1.3700 | 1.12e-20 | 5.35e-20 |
| ENSMUSG00000046324 | Ermp1 | protein\_coding | 19:29608214-29648415 (-) |  | 0.4230 | 1.12e-20 | 5.35e-20 |
| ENSMUSG00000113769 | 5033406O09Rik | lncRNA | 12:111941991-111944484 (-) |  | 2.2800 | 1.17e-20 | 5.56e-20 |
| ENSMUSG00000085939 | Cd63-ps | transcribed\_processed\_pseudogene | 18:50134726-50135600 (+) |  | 3.5200 | 1.21e-20 | 5.75e-20 |
| ENSMUSG00000022474 | Pmm1 | protein\_coding | 15:81951108-81960930 (-) |  | 1.0200 | 1.22e-20 | 5.82e-20 |
| ENSMUSG00000031799 | Tpm4 | protein\_coding | 8:72130174-72153142 (+) |  | -0.5910 | 1.28e-20 | 6.08e-20 |
| ENSMUSG00000030774 | Pak1 | protein\_coding | 7:97788541-97912381 (+) |  | 0.6780 | 1.28e-20 | 6.09e-20 |
| ENSMUSG00000041870 | Ankrd13a | protein\_coding | 5:114774677-114806200 (+) |  | 0.3720 | 1.29e-20 | 6.11e-20 |
| ENSMUSG00000051319 | Mtln | protein\_coding | 2:127791388-127792488 (-) |  | 0.9810 | 1.30e-20 | 6.19e-20 |
| ENSMUSG00000041765 | Ubac2 | protein\_coding | 14:121878620-122021034 (+) |  | -0.4520 | 1.34e-20 | 6.35e-20 |
| ENSMUSG00000074480 | Mex3a | protein\_coding | 3:88532395-88541396 (+) |  | 5.0800 | 1.36e-20 | 6.44e-20 |
| ENSMUSG00000068747 | Sort1 | protein\_coding | 3:108284082-108361511 (+) |  | -0.4810 | 1.36e-20 | 6.47e-20 |
| ENSMUSG00000036278 | Macrod1 | protein\_coding | 19:7056810-7198057 (+) |  | 0.9120 | 1.39e-20 | 6.59e-20 |
| ENSMUSG00000028798 | Eif3i | protein\_coding | 4:129591960-129600648 (-) |  | 0.4280 | 1.40e-20 | 6.63e-20 |
| ENSMUSG00000025151 | Maged1 | protein\_coding | X:94535474-94542143 (-) |  | 1.8500 | 1.42e-20 | 6.71e-20 |
| ENSMUSG00000002017 | Fam98a | protein\_coding | 17:75537086-75551946 (-) |  | 0.5380 | 1.44e-20 | 6.81e-20 |
| ENSMUSG00000033701 | Acbd6 | protein\_coding | 1:155558120-155691330 (+) |  | 0.5870 | 1.45e-20 | 6.88e-20 |
| ENSMUSG00000052271 | Bhlha15 | protein\_coding | 5:144190286-144194441 (+) |  | 3.1700 | 1.47e-20 | 6.96e-20 |
| ENSMUSG00000045509 | Gpr150 | protein\_coding | 13:76054851-76056996 (-) |  | 2.2100 | 1.48e-20 | 7.02e-20 |
| ENSMUSG00000020790 | Ankfy1 | protein\_coding | 11:72690006-72772146 (+) |  | -0.4140 | 1.49e-20 | 7.06e-20 |
| ENSMUSG00000066456 | Hmgn3 | protein\_coding | 9:83109948-83146685 (-) |  | 1.5200 | 1.52e-20 | 7.20e-20 |
| ENSMUSG00000030059 | Tmf1 | protein\_coding | 6:97152997-97179122 (-) |  | -0.4480 | 1.52e-20 | 7.20e-20 |
| ENSMUSG00000020198 | Ap3d1 | protein\_coding | 10:80706956-80742264 (-) |  | -0.4500 | 1.54e-20 | 7.26e-20 |
| ENSMUSG00000020149 | Rab1a | protein\_coding | 11:20201432-20226856 (+) |  | -0.4250 | 1.58e-20 | 7.49e-20 |
| ENSMUSG00000030822 | Prr14 | protein\_coding | 7:127459611-127476759 (+) |  | -0.3730 | 1.65e-20 | 7.78e-20 |
| ENSMUSG00000086291 | Gm15513 | lncRNA | 5:34211810-34213802 (-) |  | -2.2300 | 1.65e-20 | 7.80e-20 |
| ENSMUSG00000020152 | Actr2 | protein\_coding | 11:20062304-20112913 (-) |  | -0.4520 | 1.72e-20 | 8.15e-20 |
| ENSMUSG00000070868 | Skint3 | protein\_coding | 4:112232245-112300468 (+) |  | -3.3300 | 1.76e-20 | 8.32e-20 |
| ENSMUSG00000029033 | Acap3 | protein\_coding | 4:155891822-155907251 (+) |  | -0.5360 | 1.82e-20 | 8.61e-20 |
| ENSMUSG00000026134 | Prim2 | protein\_coding | 1:33453810-33669795 (-) |  | 0.7300 | 1.86e-20 | 8.80e-20 |
| ENSMUSG00000085872 | Gm11505 | lncRNA | 11:87795784-87801144 (-) |  | 7.0100 | 1.94e-20 | 9.14e-20 |
| ENSMUSG00000097990 | Gm19557 | lncRNA | 19:47512982-47516578 (+) |  | -1.8000 | 1.98e-20 | 9.34e-20 |
| ENSMUSG00000058756 | Thra | protein\_coding | 11:98740638-98769006 (+) |  | 0.7430 | 2.11e-20 | 9.93e-20 |
| ENSMUSG00000021257 | Angel1 | protein\_coding | 12:86700502-86726460 (-) |  | 2.0100 | 2.11e-20 | 9.95e-20 |
| ENSMUSG00000032740 | Ccdc88a | protein\_coding | 11:29373658-29510808 (+) |  | -0.6580 | 2.11e-20 | 9.95e-20 |
| ENSMUSG00000037991 | Rmi2 | protein\_coding | 16:10835059-10892966 (+) |  | 1.2100 | 2.19e-20 | 1.03e-19 |
| ENSMUSG00000038535 | Zfp280d | protein\_coding | 9:72274860-72363777 (+) |  | 0.6250 | 2.20e-20 | 1.04e-19 |
| ENSMUSG00000020929 | Eftud2 | protein\_coding | 11:102838473-102880985 (-) |  | 0.5220 | 2.21e-20 | 1.04e-19 |
| ENSMUSG00000046756 | Mrps7 | protein\_coding | 11:115603925-115608036 (+) |  | 0.5980 | 2.25e-20 | 1.06e-19 |
| ENSMUSG00000036959 | Bcorl1 | protein\_coding | X:48341358-48408049 (+) |  | 0.9950 | 2.28e-20 | 1.07e-19 |
| ENSMUSG00000030888 | Rrp8 | protein\_coding | 7:105731730-105737385 (-) |  | 0.5330 | 2.29e-20 | 1.08e-19 |
| ENSMUSG00000027474 | Ccm2l | protein\_coding | 2:153065955-153081735 (+) |  | 1.7600 | 2.37e-20 | 1.12e-19 |
| ENSMUSG00000021958 | Pinx1 | protein\_coding | 14:63860364-63919859 (+) |  | 0.7050 | 2.41e-20 | 1.13e-19 |
| ENSMUSG00000060681 | Slc9a6 | protein\_coding | X:56609757-56664230 (+) |  | -0.4890 | 2.48e-20 | 1.16e-19 |
| ENSMUSG00000023348 | Trip6 | protein\_coding | 5:137309654-137314404 (-) |  | -0.7470 | 2.55e-20 | 1.20e-19 |
| ENSMUSG00000020604 | Arsg | protein\_coding | 11:109473374-109573330 (+) |  | -1.7800 | 2.67e-20 | 1.25e-19 |
| ENSMUSG00000034789 | Rab24 | protein\_coding | 13:55319743-55321946 (-) |  | -0.5350 | 2.69e-20 | 1.26e-19 |
| ENSMUSG00000036112 | Metap2 | protein\_coding | 10:93858489-93897093 (-) |  | 0.5150 | 2.74e-20 | 1.29e-19 |
| ENSMUSG00000032400 | Zwilch | protein\_coding | 9:64137144-64173104 (-) |  | 0.5880 | 2.87e-20 | 1.34e-19 |
| ENSMUSG00000015112 | Slc25a13 | protein\_coding | 6:6041218-6217173 (-) |  | 0.4750 | 2.88e-20 | 1.35e-19 |
| ENSMUSG00000030748 | Il4ra | protein\_coding | 7:125552120-125579474 (+) |  | -0.8380 | 2.90e-20 | 1.36e-19 |
| ENSMUSG00000117621 | Hspe1-rs1 | protein\_coding | 18:47112139-47112653 (+) |  | 1.2500 | 2.90e-20 | 1.36e-19 |
| ENSMUSG00000050043 | Tmx2 | protein\_coding | 2:84671316-84679140 (-) |  | 0.4670 | 2.98e-20 | 1.40e-19 |
| ENSMUSG00000056493 | Foxk1 | protein\_coding | 5:142401497-142462011 (+) |  | -0.5480 | 3.02e-20 | 1.42e-19 |
| ENSMUSG00000038764 | Ptpn3 | protein\_coding | 4:57190841-57301837 (-) |  | 2.2900 | 3.07e-20 | 1.44e-19 |
| ENSMUSG00000029634 | Rnf6 | protein\_coding | 5:146209192-146221555 (-) |  | -0.4490 | 3.08e-20 | 1.44e-19 |
| ENSMUSG00000032563 | Mrpl3 | protein\_coding | 9:105053239-105079888 (+) |  | 0.6580 | 3.11e-20 | 1.46e-19 |
| ENSMUSG00000021811 | Dnajc9 | protein\_coding | 14:20384638-20388910 (-) |  | 0.6350 | 3.12e-20 | 1.46e-19 |
| ENSMUSG00000023259 | Slc26a6 | protein\_coding | 9:108853283-108913049 (+) |  | -0.8410 | 3.15e-20 | 1.47e-19 |
| ENSMUSG00000021823 | Vcl | protein\_coding | 14:20929398-21033676 (+) |  | -0.6950 | 3.19e-20 | 1.49e-19 |
| ENSMUSG00000021288 | Klc1 | protein\_coding | 12:111758849-111807844 (+) |  | -0.5050 | 3.20e-20 | 1.50e-19 |
| ENSMUSG00000044701 | Il27 | protein\_coding | 7:126589010-126594941 (-) |  | -1.9100 | 3.34e-20 | 1.56e-19 |
| ENSMUSG00000046186 | Cd109 | protein\_coding | 9:78615546-78716253 (+) |  | -1.7800 | 3.36e-20 | 1.57e-19 |
| ENSMUSG00000024085 | Man2a1 | protein\_coding | 17:64600736-64755110 (+) |  | -0.4460 | 3.42e-20 | 1.60e-19 |
| ENSMUSG00000030726 | Pold3 | protein\_coding | 7:100082111-100121565 (-) |  | 0.4910 | 3.56e-20 | 1.66e-19 |
| ENSMUSG00000036109 | Mbnl3 | protein\_coding | X:51117269-51206532 (-) |  | 0.8430 | 3.63e-20 | 1.70e-19 |
| ENSMUSG00000027012 | Dync1i2 | protein\_coding | 2:71211706-71263303 (+) |  | -0.4320 | 3.70e-20 | 1.73e-19 |
| ENSMUSG00000113010 | Gm34084 | lncRNA | 13:44472426-44504548 (-) |  | -1.9800 | 4.11e-20 | 1.92e-19 |
| ENSMUSG00000029826 | Zc3hav1 | protein\_coding | 6:38305286-38354603 (-) |  | -0.4700 | 4.12e-20 | 1.92e-19 |
| ENSMUSG00000070934 | Rraga | protein\_coding | 4:86575668-86577285 (+) |  | -0.5950 | 4.30e-20 | 2.00e-19 |
| ENSMUSG00000029276 | Glmn | protein\_coding | 5:107548967-107597888 (-) |  | 1.2700 | 4.39e-20 | 2.04e-19 |
| ENSMUSG00000040661 | Rad54l2 | protein\_coding | 9:106688082-106789194 (-) |  | 0.7000 | 4.74e-20 | 2.21e-19 |
| ENSMUSG00000029836 | Cbx3 | protein\_coding | 6:51470360-51483704 (+) |  | 0.4540 | 4.76e-20 | 2.22e-19 |
| ENSMUSG00000027589 | Pcmtd2 | protein\_coding | 2:181837854-181857461 (+) |  | -0.6240 | 4.80e-20 | 2.23e-19 |
| ENSMUSG00000074063 | Osgin1 | protein\_coding | 8:119434124-119446256 (+) |  | -1.4000 | 4.83e-20 | 2.25e-19 |
| ENSMUSG00000058569 | Tmed9 | protein\_coding | 13:55573732-55597697 (+) |  | 0.3970 | 5.10e-20 | 2.38e-19 |
| ENSMUSG00000008193 | Spib | protein\_coding | 7:44525993-44532071 (-) |  | 2.5300 | 5.22e-20 | 2.43e-19 |
| ENSMUSG00000054455 | Vapb | protein\_coding | 2:173737511-173784339 (+) |  | -0.5290 | 5.36e-20 | 2.49e-19 |
| ENSMUSG00000025156 | Gps1 | protein\_coding | 11:120784272-120789102 (+) |  | 0.4390 | 5.36e-20 | 2.49e-19 |
| ENSMUSG00000041168 | Lonp1 | protein\_coding | 17:56614297-56626887 (-) |  | 0.6730 | 5.47e-20 | 2.54e-19 |
| ENSMUSG00000020264 | Slc36a2 | protein\_coding | 11:55158470-55185077 (-) |  | 1.7000 | 5.50e-20 | 2.55e-19 |
| ENSMUSG00000047843 | Bri3 | protein\_coding | 5:144244437-144446757 (+) |  | -0.9710 | 5.50e-20 | 2.56e-19 |
| ENSMUSG00000019874 | Fabp7 | protein\_coding | 10:57784881-57788450 (+) |  | -4.3000 | 5.60e-20 | 2.60e-19 |
| ENSMUSG00000099757 | BE692007 | lncRNA | 19:11470166-11484578 (-) |  | -0.7180 | 5.61e-20 | 2.61e-19 |
| ENSMUSG00000026172 | Bcs1l | protein\_coding | 1:74588289-74592443 (+) |  | 0.6700 | 5.62e-20 | 2.61e-19 |
| ENSMUSG00000023186 | Vwa5a | protein\_coding | 9:38718268-38743337 (+) |  | -0.7260 | 5.73e-20 | 2.66e-19 |
| ENSMUSG00000086425 | F730016J06Rik | lncRNA | 2:28095477-28127731 (+) |  | -1.4900 | 5.73e-20 | 2.66e-19 |
| ENSMUSG00000102918 | Pcdhgc3 | protein\_coding | 18:37806364-37841873 (+) |  | 2.4600 | 5.82e-20 | 2.70e-19 |
| ENSMUSG00000111116 | Gm48065 | lncRNA | 10:44597889-44602174 (-) |  | -3.6700 | 5.86e-20 | 2.71e-19 |
| ENSMUSG00000038650 | Rnh1 | protein\_coding | 7:141160326-141172857 (-) |  | -0.6740 | 6.03e-20 | 2.79e-19 |
| ENSMUSG00000024891 | Slc29a2 | protein\_coding | 19:5023860-5031972 (+) |  | 1.6200 | 6.06e-20 | 2.81e-19 |
| ENSMUSG00000031584 | Gsr | protein\_coding | 8:33652523-33698163 (+) |  | -0.6080 | 6.12e-20 | 2.83e-19 |
| ENSMUSG00000019813 | Cep57l1 | protein\_coding | 10:41718840-41809871 (-) |  | 0.9040 | 6.24e-20 | 2.89e-19 |
| ENSMUSG00000028096 | Gpr89 | protein\_coding | 3:96868281-96905346 (-) |  | 0.6190 | 6.26e-20 | 2.90e-19 |
| ENSMUSG00000005986 | Ankrd13d | protein\_coding | 19:4270180-4283137 (-) |  | -0.8840 | 6.34e-20 | 2.93e-19 |
| ENSMUSG00000032946 | Rasgrp2 | protein\_coding | 19:6399340-6415216 (+) |  | 0.4520 | 6.37e-20 | 2.95e-19 |
| ENSMUSG00000044231 | Nhlrc1 | protein\_coding | 13:47012557-47014850 (-) |  | 2.7700 | 6.54e-20 | 3.02e-19 |
| ENSMUSG00000003382 | Etv3 | protein\_coding | 3:87525407-87540156 (+) |  | -0.4700 | 6.58e-20 | 3.04e-19 |
| ENSMUSG00000024683 | Mrpl16 | protein\_coding | 19:11770391-11774960 (+) |  | 0.5590 | 6.71e-20 | 3.10e-19 |
| ENSMUSG00000033885 | Pxk | protein\_coding | 14:8098211-8165117 (+) |  | -0.5150 | 7.10e-20 | 3.28e-19 |
| ENSMUSG00000014158 | Trpv4 | protein\_coding | 5:114622152-114658421 (-) |  | -2.2500 | 7.31e-20 | 3.38e-19 |
| ENSMUSG00000107760 | Gm44401 | TEC | 6:136620632-136623006 (+) |  | -2.7800 | 7.44e-20 | 3.43e-19 |
| ENSMUSG00000052415 | Tchh | protein\_coding | 3:93442330-93449077 (+) |  | -1.3500 | 7.65e-20 | 3.53e-19 |
| ENSMUSG00000038619 | Ensa | protein\_coding | 3:95624993-95632102 (+) |  | -0.5150 | 7.73e-20 | 3.57e-19 |
| ENSMUSG00000063235 | Ptpmt1 | protein\_coding | 2:90908716-90918258 (-) |  | 0.7550 | 7.77e-20 | 3.58e-19 |
| ENSMUSG00000034659 | Tmem109 | protein\_coding | 19:10870660-10882001 (-) |  | -0.5100 | 7.92e-20 | 3.65e-19 |
| ENSMUSG00000031093 | Dock11 | protein\_coding | X:35888832-36076562 (+) |  | -0.5420 | 7.94e-20 | 3.66e-19 |
| ENSMUSG00000050394 | Armcx6 | protein\_coding | X:134748461-134751417 (-) |  | 3.9200 | 8.00e-20 | 3.69e-19 |
| ENSMUSG00000051212 | Gpr183 | protein\_coding | 14:121952551-121965195 (-) |  | -0.7730 | 8.05e-20 | 3.71e-19 |
| ENSMUSG00000005378 | Bud23 | protein\_coding | 5:135052957-135064959 (-) |  | 0.4800 | 8.14e-20 | 3.75e-19 |
| ENSMUSG00000061950 | Ppp4r1 | protein\_coding | 17:65782573-65841926 (+) |  | -0.4480 | 8.22e-20 | 3.79e-19 |
| ENSMUSG00000009376 | Met | protein\_coding | 6:17463800-17573980 (+) |  | 0.8060 | 8.24e-20 | 3.79e-19 |
| ENSMUSG00000049881 | 2810025M15Rik | transcribed\_processed\_pseudogene | 1:157412352-157420236 (+) |  | 0.9970 | 8.29e-20 | 3.81e-19 |
| ENSMUSG00000067370 | B3galt4 | protein\_coding | 17:33949909-33951488 (-) |  | -0.8900 | 8.46e-20 | 3.89e-19 |
| ENSMUSG00000079339 | Ifit1bl1 | protein\_coding | 19:34592891-34601956 (-) |  | -4.6600 | 8.49e-20 | 3.90e-19 |
| ENSMUSG00000049299 | Trappc1 | protein\_coding | 11:69323980-69325793 (+) |  | -0.6460 | 8.60e-20 | 3.95e-19 |
| ENSMUSG00000062382 | Ftl1-ps1 | protein\_coding | 13:74399694-74407346 (+) |  | -0.6960 | 8.66e-20 | 3.98e-19 |
| ENSMUSG00000027111 | Itga6 | protein\_coding | 2:71745616-71858416 (+) |  | 0.9780 | 9.20e-20 | 4.22e-19 |
| ENSMUSG00000020869 | Lrrc59 | protein\_coding | 11:94629767-94645216 (+) |  | 0.6790 | 9.81e-20 | 4.50e-19 |
| ENSMUSG00000048058 | Ldlrad3 | protein\_coding | 2:101950203-102186385 (-) |  | -0.9610 | 9.99e-20 | 4.59e-19 |
| ENSMUSG00000037236 | Matr3 | protein\_coding | 18:35562138-35593835 (+) |  | 0.4980 | 1.00e-19 | 4.61e-19 |
| ENSMUSG00000045160 | Bola3 | protein\_coding | 6:83349147-83360136 (+) |  | 0.9540 | 1.01e-19 | 4.61e-19 |
| ENSMUSG00000036461 | Elf1 | protein\_coding | 14:79481194-79582494 (+) |  | 0.3280 | 1.01e-19 | 4.62e-19 |
| ENSMUSG00000026858 | Miga2 | protein\_coding | 2:30364233-30385521 (+) |  | 0.8200 | 1.07e-19 | 4.91e-19 |
| ENSMUSG00000028098 | Rnf115 | protein\_coding | 3:96727664-96791638 (+) |  | -0.5320 | 1.09e-19 | 4.98e-19 |
| ENSMUSG00000031950 | Gabarapl2 | protein\_coding | 8:111940703-111953612 (+) |  | -0.5290 | 1.10e-19 | 5.03e-19 |
| ENSMUSG00000026807 | Ak8 | protein\_coding | 2:28700164-28813165 (+) |  | -1.4100 | 1.11e-19 | 5.08e-19 |
| ENSMUSG00000045598 | Zfp553 | protein\_coding | 7:127233061-127238179 (+) |  | 0.6630 | 1.17e-19 | 5.38e-19 |
| ENSMUSG00000037318 | Traf3ip3 | protein\_coding | 1:193175453-193201703 (-) |  | -0.5470 | 1.26e-19 | 5.76e-19 |
| ENSMUSG00000014837 | 4931428F04Rik | protein\_coding | 8:105280409-105289864 (-) |  | 1.6800 | 1.30e-19 | 5.94e-19 |
| ENSMUSG00000038510 | Rpf2 | protein\_coding | 10:40223246-40247036 (-) |  | 0.7570 | 1.35e-19 | 6.18e-19 |
| ENSMUSG00000028795 | Ccdc28b | protein\_coding | 4:129619274-129623947 (-) |  | -0.5610 | 1.36e-19 | 6.20e-19 |
| ENSMUSG00000034353 | Ramp1 | protein\_coding | 1:91179822-91225196 (+) |  | 0.9020 | 1.39e-19 | 6.34e-19 |
| ENSMUSG00000117713 | Gm46637 | lncRNA | 18:61484766-61489220 (+) |  | -3.8400 | 1.42e-19 | 6.48e-19 |
| ENSMUSG00000036390 | Gadd45a | protein\_coding | 6:67035096-67037457 (-) |  | -0.9150 | 1.43e-19 | 6.52e-19 |
| ENSMUSG00000035133 | Arhgap5 | protein\_coding | 12:52503972-52571975 (+) |  | 0.7140 | 1.48e-19 | 6.77e-19 |
| ENSMUSG00000049940 | Pgrmc2 | protein\_coding | 3:41066326-41083046 (-) |  | 0.4660 | 1.51e-19 | 6.88e-19 |
| ENSMUSG00000029338 | Antxr2 | protein\_coding | 5:97882783-98031043 (-) |  | -0.7200 | 1.54e-19 | 7.02e-19 |
| ENSMUSG00000037266 | Rsrp1 | protein\_coding | 4:134923592-134927671 (+) |  | -0.7270 | 1.55e-19 | 7.06e-19 |
| ENSMUSG00000110062 | B430319F04Rik | TEC | 7:110766677-110769345 (-) |  | -1.8300 | 1.55e-19 | 7.06e-19 |
| ENSMUSG00000086688 | Gm11560 | processed\_pseudogene | 11:99932162-99933109 (+) |  | 0.6960 | 1.56e-19 | 7.11e-19 |
| ENSMUSG00000067713 | Prkag1 | protein\_coding | 15:98812797-98831521 (-) |  | -0.4260 | 1.59e-19 | 7.27e-19 |
| ENSMUSG00000031103 | Elf4 | protein\_coding | X:48411046-48463132 (-) |  | -0.5900 | 1.59e-19 | 7.27e-19 |
| ENSMUSG00000101599 | Gm20342 | lncRNA | 1:63117118-63126837 (+) |  | 1.2700 | 1.66e-19 | 7.57e-19 |
| ENSMUSG00000032966 | Fkbp1a | protein\_coding | 2:151542483-151561692 (+) |  | 0.4820 | 1.69e-19 | 7.68e-19 |
| ENSMUSG00000037572 | Wdhd1 | protein\_coding | 14:47240944-47276857 (-) |  | 0.7530 | 1.69e-19 | 7.68e-19 |
| ENSMUSG00000059713 | Rcan3 | protein\_coding | 4:135412308-135433853 (-) |  | -0.6880 | 1.76e-19 | 8.02e-19 |
| ENSMUSG00000051457 | Spn | protein\_coding | 7:127132232-127137823 (-) |  | 0.4440 | 1.77e-19 | 8.04e-19 |
| ENSMUSG00000020532 | Acaca | protein\_coding | 11:84129672-84401651 (+) |  | 0.5780 | 1.79e-19 | 8.13e-19 |
| ENSMUSG00000039013 | Siglecf | protein\_coding | 7:43351341-43359531 (+) |  | 1.3300 | 1.80e-19 | 8.19e-19 |
| ENSMUSG00000074656 | Eif2s2 | protein\_coding | 2:154871410-154892935 (-) |  | 0.6470 | 1.82e-19 | 8.29e-19 |
| ENSMUSG00000033943 | Mga | protein\_coding | 2:119897228-119969581 (+) |  | 0.5330 | 1.83e-19 | 8.31e-19 |
| ENSMUSG00000013701 | Timm23 | protein\_coding | 14:32180162-32201898 (-) |  | 0.6930 | 1.83e-19 | 8.32e-19 |
| ENSMUSG00000040560 | Wdr7 | protein\_coding | 18:63708685-63989776 (+) |  | -0.6310 | 1.84e-19 | 8.36e-19 |
| ENSMUSG00000109005 | Gm45221 | TEC | 7:90185927-90188198 (+) |  | -1.6000 | 1.84e-19 | 8.36e-19 |
| ENSMUSG00000024381 | Bin1 | protein\_coding | 18:32377230-32435737 (+) |  | 0.5840 | 1.84e-19 | 8.37e-19 |
| ENSMUSG00000027109 | Sp3 | protein\_coding | 2:72936427-72980446 (-) |  | -0.5420 | 1.86e-19 | 8.43e-19 |
| ENSMUSG00000104350 | Gm38244 | TEC | 3:106478283-106480868 (+) |  | -1.9000 | 1.86e-19 | 8.45e-19 |
| ENSMUSG00000029366 | Dck | protein\_coding | 5:88764996-88783281 (+) |  | -0.4790 | 1.87e-19 | 8.49e-19 |
| ENSMUSG00000045176 | Borcs6 | protein\_coding | 11:69059717-69061578 (+) |  | -0.8030 | 1.89e-19 | 8.56e-19 |
| ENSMUSG00000023067 | Cdkn1a | protein\_coding | 17:29090976-29100727 (+) |  | -0.7940 | 1.95e-19 | 8.84e-19 |
| ENSMUSG00000034656 | Cacna1a | protein\_coding | 8:84338639-84640246 (+) |  | 1.8000 | 1.97e-19 | 8.94e-19 |
| ENSMUSG00000047669 | Msl3l2 | protein\_coding | 10:56106917-56116880 (+) |  | 1.0600 | 1.98e-19 | 8.95e-19 |
| ENSMUSG00000071637 | Cebpd | protein\_coding | 16:15887286-15891031 (+) |  | -0.8680 | 2.02e-19 | 9.17e-19 |
| ENSMUSG00000023015 | Racgap1 | protein\_coding | 15:99620496-99651656 (-) |  | -0.3450 | 2.03e-19 | 9.19e-19 |
| ENSMUSG00000000682 | Cd52 | protein\_coding | 4:134082448-134095082 (-) |  | -1.3500 | 2.06e-19 | 9.34e-19 |
| ENSMUSG00000029458 | Brap | protein\_coding | 5:121660563-121687256 (+) |  | -0.3990 | 2.10e-19 | 9.51e-19 |
| ENSMUSG00000024317 | Rnf138 | protein\_coding | 18:21001341-21028224 (+) |  | 0.6820 | 2.13e-19 | 9.66e-19 |
| ENSMUSG00000023904 | Hcfc1r1 | protein\_coding | 17:23673596-23675227 (+) |  | -0.9020 | 2.14e-19 | 9.69e-19 |
| ENSMUSG00000038503 | Mesd | protein\_coding | 7:83884466-83901532 (+) |  | 0.5130 | 2.21e-19 | 9.97e-19 |
| ENSMUSG00000022048 | Dpysl2 | protein\_coding | 14:66802864-66868688 (-) |  | -0.7880 | 2.28e-19 | 1.03e-18 |
| ENSMUSG00000074570 | Cass4 | protein\_coding | 2:172393794-172433757 (+) |  | -1.6600 | 2.30e-19 | 1.04e-18 |
| ENSMUSG00000026158 | Ogfrl1 | protein\_coding | 1:23366424-23397772 (-) |  | -0.4950 | 2.35e-19 | 1.06e-18 |
| ENSMUSG00000024436 | Mrps18b | protein\_coding | 17:35910379-35916389 (-) |  | 0.9300 | 2.41e-19 | 1.09e-18 |
| ENSMUSG00000028437 | Ubap1 | protein\_coding | 4:41348996-41390525 (+) |  | -0.4870 | 2.49e-19 | 1.12e-18 |
| ENSMUSG00000048087 | Gm4737 | protein\_coding | 16:46152985-46155077 (-) |  | 1.0500 | 2.52e-19 | 1.14e-18 |
| ENSMUSG00000055024 | Ep300 | protein\_coding | 15:81585351-81652077 (+) |  | -0.6390 | 2.53e-19 | 1.14e-18 |
| ENSMUSG00000085795 | Zfp703 | protein\_coding | 8:26977325-26981461 (+) |  | -0.6570 | 2.56e-19 | 1.15e-18 |
| ENSMUSG00000039879 | Heca | protein\_coding | 10:17868612-17948067 (-) |  | -0.5970 | 2.58e-19 | 1.16e-18 |
| ENSMUSG00000043391 | 2510009E07Rik | protein\_coding | 16:21649045-21694665 (-) |  | -1.7800 | 2.60e-19 | 1.17e-18 |
| ENSMUSG00000029463 | Fam216a | protein\_coding | 5:122364580-122372364 (-) |  | 1.2300 | 2.60e-19 | 1.17e-18 |
| ENSMUSG00000030793 | Pycard | protein\_coding | 7:127989708-127993867 (-) |  | -0.7930 | 2.64e-19 | 1.19e-18 |
| ENSMUSG00000040592 | Cd79b | protein\_coding | 11:106311341-106314762 (-) |  | 1.2300 | 2.66e-19 | 1.20e-18 |
| ENSMUSG00000028737 | Aldh4a1 | protein\_coding | 4:139622866-139649690 (+) |  | 0.7120 | 2.69e-19 | 1.21e-18 |
| ENSMUSG00000023832 | Acat2 | protein\_coding | 17:12942890-12960747 (-) |  | 0.6410 | 2.72e-19 | 1.22e-18 |
| ENSMUSG00000019878 | Hsf2 | protein\_coding | 10:57486385-57513135 (+) |  | -0.5730 | 2.74e-19 | 1.23e-18 |
| ENSMUSG00000063382 | Bcl9l | protein\_coding | 9:44482825-44511896 (+) |  | -0.8660 | 2.75e-19 | 1.24e-18 |
| ENSMUSG00000043024 | Gm16433 | processed\_pseudogene | 6:108563605-108565588 (-) |  | 1.2300 | 2.87e-19 | 1.29e-18 |
| ENSMUSG00000045165 | AI467606 | protein\_coding | 7:127091359-127093986 (+) |  | -0.7070 | 2.89e-19 | 1.30e-18 |
| ENSMUSG00000028138 | Adh5 | protein\_coding | 3:138443093-138455499 (+) |  | 0.4460 | 3.01e-19 | 1.35e-18 |
| ENSMUSG00000031536 | Polb | protein\_coding | 8:22628126-22653435 (-) |  | -0.7110 | 3.02e-19 | 1.36e-18 |
| ENSMUSG00000049624 | Slc17a5 | protein\_coding | 9:78536488-78588041 (-) |  | -0.7080 | 3.03e-19 | 1.36e-18 |
| ENSMUSG00000038352 | Arl5c | protein\_coding | 11:97989578-97996181 (-) |  | -0.9130 | 3.03e-19 | 1.36e-18 |
| ENSMUSG00000067577 | A430093F15Rik | lncRNA | 19:10740947-10786043 (+) |  | -2.6900 | 3.03e-19 | 1.36e-18 |
| ENSMUSG00000005936 | Kctd20 | protein\_coding | 17:28951950-28969549 (+) |  | -0.2720 | 3.04e-19 | 1.36e-18 |
| ENSMUSG00000028494 | Plin2 | protein\_coding | 4:86648386-86670060 (-) |  | -1.0500 | 3.21e-19 | 1.44e-18 |
| ENSMUSG00000023452 | Pisd | protein\_coding | 5:32736301-32785646 (-) |  | -0.4670 | 3.22e-19 | 1.44e-18 |
| ENSMUSG00000039886 | Tmem120a | protein\_coding | 5:135735485-135744448 (-) |  | -0.6960 | 3.23e-19 | 1.45e-18 |
| ENSMUSG00000081111 | Gm5913 | processed\_pseudogene | 8:97427712-97429110 (-) |  | 0.9490 | 3.29e-19 | 1.48e-18 |
| ENSMUSG00000085894 | Gm15832 | lncRNA | 1:39547570-39552002 (-) |  | -1.4000 | 3.31e-19 | 1.48e-18 |
| ENSMUSG00000033530 | Ttc7b | protein\_coding | 12:100300770-100520826 (-) |  | 0.5520 | 3.31e-19 | 1.48e-18 |
| ENSMUSG00000109904 | Gm45819 | TEC | 7:110119990-110121740 (+) |  | 1.8100 | 3.32e-19 | 1.49e-18 |
| ENSMUSG00000063810 | Alms1 | protein\_coding | 6:85587531-85702753 (+) |  | 0.9460 | 3.35e-19 | 1.50e-18 |
| ENSMUSG00000030002 | Dusp11 | protein\_coding | 6:85942268-85961667 (-) |  | -0.3670 | 3.41e-19 | 1.52e-18 |
| ENSMUSG00000047123 | Ticam1 | protein\_coding | 17:56269319-56276786 (-) |  | -0.7140 | 3.44e-19 | 1.54e-18 |
| ENSMUSG00000091575 | 2010016I18Rik | lncRNA | 3:106481982-106485913 (-) |  | -1.5500 | 3.46e-19 | 1.55e-18 |
| ENSMUSG00000062028 | Irgc1 | protein\_coding | 7:24431922-24452297 (-) |  | -2.4600 | 3.54e-19 | 1.58e-18 |
| ENSMUSG00000030316 | Tamm41 | protein\_coding | 6:115004381-115037876 (-) |  | 0.6470 | 3.57e-19 | 1.60e-18 |
| ENSMUSG00000029416 | Slc15a4 | protein\_coding | 5:127595664-127632897 (-) |  | -0.5090 | 3.58e-19 | 1.60e-18 |
| ENSMUSG00000043635 | Adamts3 | protein\_coding | 5:89677087-89883334 (-) |  | 1.1200 | 3.65e-19 | 1.63e-18 |
| ENSMUSG00000031928 | Mre11a | protein\_coding | 9:14784654-14837123 (+) |  | 0.4370 | 3.73e-19 | 1.67e-18 |
| ENSMUSG00000047153 | Khnyn | protein\_coding | 14:55884947-55898775 (+) |  | -0.6780 | 3.73e-19 | 1.67e-18 |
| ENSMUSG00000040524 | Zfp609 | protein\_coding | 9:65692391-65827564 (-) |  | 0.5970 | 3.82e-19 | 1.71e-18 |
| ENSMUSG00000032299 | Commd4 | protein\_coding | 9:57155035-57158331 (-) |  | -0.4470 | 3.85e-19 | 1.72e-18 |
| ENSMUSG00000107352 | Gm43660 | lncRNA | 5:31560812-31569072 (-) |  | -1.1600 | 3.90e-19 | 1.74e-18 |
| ENSMUSG00000047821 | Trim16 | protein\_coding | 11:62820231-62850808 (+) |  | -0.4990 | 3.92e-19 | 1.75e-18 |
| ENSMUSG00000019054 | Fis1 | protein\_coding | 5:136953275-136966234 (+) |  | -0.5630 | 3.98e-19 | 1.78e-18 |
| ENSMUSG00000022372 | Sla | protein\_coding | 15:66780819-66831829 (-) |  | -0.9450 | 4.18e-19 | 1.86e-18 |
| ENSMUSG00000033088 | Triobp | protein\_coding | 15:78947724-79005869 (+) |  | -0.4930 | 4.24e-19 | 1.89e-18 |
| ENSMUSG00000038718 | Pbx3 | protein\_coding | 2:34171457-34373142 (-) |  | -0.9660 | 4.30e-19 | 1.92e-18 |
| ENSMUSG00000002602 | Axl | protein\_coding | 7:25757273-25788705 (-) |  | -1.1500 | 4.32e-19 | 1.92e-18 |
| ENSMUSG00000038543 | BC028528 | protein\_coding | 3:95883954-95892005 (-) |  | -0.9410 | 4.34e-19 | 1.93e-18 |
| ENSMUSG00000039531 | Zup1 | protein\_coding | 10:33919142-33951269 (-) |  | -0.7840 | 4.48e-19 | 2.00e-18 |
| ENSMUSG00000004099 | Dnmt1 | protein\_coding | 9:20907209-20959888 (-) |  | 0.6920 | 4.58e-19 | 2.04e-18 |
| ENSMUSG00000039787 | Cercam | protein\_coding | 2:29869164-29882840 (+) |  | 1.1400 | 4.63e-19 | 2.06e-18 |
| ENSMUSG00000009535 | Rnmt | protein\_coding | 18:68300355-68324852 (+) |  | 0.3910 | 4.78e-19 | 2.13e-18 |
| ENSMUSG00000031389 | Arhgap4 | protein\_coding | X:73891442-73921870 (-) |  | -0.5120 | 4.86e-19 | 2.16e-18 |
| ENSMUSG00000005374 | Tbl2 | protein\_coding | 5:135149657-135165760 (+) |  | 0.4260 | 4.89e-19 | 2.17e-18 |
| ENSMUSG00000024349 | Tmem173 | protein\_coding | 18:35733679-35740554 (-) |  | -0.3430 | 4.90e-19 | 2.18e-18 |
| ENSMUSG00000022247 | Brix1 | protein\_coding | 15:10474779-10485947 (-) |  | 0.6950 | 5.04e-19 | 2.24e-18 |
| ENSMUSG00000092627 | D130058E05Rik | lncRNA | 1:89930234-89933290 (+) |  | 2.0500 | 5.24e-19 | 2.32e-18 |
| ENSMUSG00000035824 | Tk2 | protein\_coding | 8:104226685-104248558 (-) |  | -0.7020 | 5.44e-19 | 2.41e-18 |
| ENSMUSG00000027882 | Stxbp3 | protein\_coding | 3:108793176-108840526 (-) |  | -0.6540 | 5.58e-19 | 2.48e-18 |
| ENSMUSG00000097493 | 9930014A18Rik | lncRNA | 15:60822271-60831793 (+) |  | 1.6300 | 5.67e-19 | 2.52e-18 |
| ENSMUSG00000029699 | Ssc4d | protein\_coding | 5:135960211-135974531 (-) |  | -0.9320 | 5.74e-19 | 2.54e-18 |
| ENSMUSG00000028885 | Smpdl3b | protein\_coding | 4:132732966-132757252 (-) |  | -2.3300 | 5.75e-19 | 2.55e-18 |
| ENSMUSG00000022314 | Rad21 | protein\_coding | 15:51962240-51991747 (-) |  | -0.3980 | 5.77e-19 | 2.56e-18 |
| ENSMUSG00000024188 | Luc7l | protein\_coding | 17:26252896-26285504 (+) |  | 0.4730 | 5.88e-19 | 2.60e-18 |
| ENSMUSG00000026618 | Iars2 | protein\_coding | 1:185284726-185329396 (-) |  | 0.5080 | 6.06e-19 | 2.68e-18 |
| ENSMUSG00000078502 | Gm13212 | protein\_coding | 4:145585166-145625345 (+) |  | -0.9990 | 6.24e-19 | 2.76e-18 |
| ENSMUSG00000041515 | Irf8 | protein\_coding | 8:120736358-120756694 (+) |  | 0.8780 | 6.26e-19 | 2.77e-18 |
| ENSMUSG00000007050 | Lsm2 | protein\_coding | 17:34981862-34985891 (+) |  | 0.6520 | 6.29e-19 | 2.78e-18 |
| ENSMUSG00000022370 | Mrpl13 | protein\_coding | 15:55534094-55557748 (-) |  | 0.6410 | 7.05e-19 | 3.12e-18 |
| ENSMUSG00000042197 | Zfp451 | protein\_coding | 1:33761545-33814595 (-) |  | 0.4850 | 7.06e-19 | 3.12e-18 |
| ENSMUSG00000061455 | Stx17 | protein\_coding | 4:48124915-48186507 (+) |  | -0.6580 | 7.11e-19 | 3.14e-18 |
| ENSMUSG00000033751 | Gadd45gip1 | protein\_coding | 8:84831522-84835482 (+) |  | 0.8460 | 7.24e-19 | 3.20e-18 |
| ENSMUSG00000025268 | Maged2 | protein\_coding | X:150806370-150814345 (-) |  | 2.0500 | 7.40e-19 | 3.27e-18 |
| ENSMUSG00000027544 | Nfatc2 | protein\_coding | 2:168476410-168601657 (-) |  | -1.1200 | 7.41e-19 | 3.27e-18 |
| ENSMUSG00000033379 | Atp6v0b | protein\_coding | 4:117884326-117887333 (-) |  | -0.5260 | 7.46e-19 | 3.29e-18 |
| ENSMUSG00000039178 | Tbc1d19 | protein\_coding | 5:53809606-53903965 (+) |  | 1.4200 | 7.47e-19 | 3.30e-18 |
| ENSMUSG00000078796 | Zfp541 | protein\_coding | 7:16061814-16096334 (+) |  | -3.1300 | 7.61e-19 | 3.36e-18 |
| ENSMUSG00000020634 | Ubxn2a | protein\_coding | 12:4879032-4907705 (-) |  | 0.6480 | 7.63e-19 | 3.37e-18 |
| ENSMUSG00000068854 | H2bc21 | protein\_coding | 3:96221119-96223738 (+) |  | -0.9900 | 7.72e-19 | 3.40e-18 |
| ENSMUSG00000023039 | Krt7 | protein\_coding | 15:101411043-101430313 (+) |  | -2.4500 | 8.01e-19 | 3.53e-18 |
| ENSMUSG00000025722 | Wdr73 | protein\_coding | 7:80890723-80901269 (-) |  | 0.7320 | 8.06e-19 | 3.55e-18 |
| ENSMUSG00000039449 | Prpf18 | protein\_coding | 2:4622058-4652113 (-) |  | -0.3590 | 8.16e-19 | 3.59e-18 |
| ENSMUSG00000074342 | I830077J02Rik | protein\_coding | 3:105924358-105932664 (-) |  | -0.5160 | 8.38e-19 | 3.69e-18 |
| ENSMUSG00000024610 | Cd74 | protein\_coding | 18:60803848-60812652 (+) |  | -1.7500 | 8.56e-19 | 3.77e-18 |
| ENSMUSG00000083567 | Gm11451 | processed\_pseudogene | 2:162898894-162899523 (-) |  | 1.4000 | 8.64e-19 | 3.81e-18 |
| ENSMUSG00000004530 | Coro1c | protein\_coding | 5:113842436-113908758 (-) |  | -0.3260 | 8.91e-19 | 3.92e-18 |
| ENSMUSG00000037234 | Hook3 | protein\_coding | 8:26021421-26119224 (-) |  | -0.5380 | 9.32e-19 | 4.10e-18 |
| ENSMUSG00000025580 | Eif4a3 | protein\_coding | 11:119288363-119300089 (-) |  | 0.4400 | 9.86e-19 | 4.34e-18 |
| ENSMUSG00000028914 | Casp9 | protein\_coding | 4:141793612-141815976 (+) |  | -0.4480 | 9.97e-19 | 4.39e-18 |
| ENSMUSG00000053644 | Aldh7a1 | protein\_coding | 18:56509687-56572951 (-) |  | 1.4300 | 1.02e-18 | 4.48e-18 |
| ENSMUSG00000028862 | Map3k6 | protein\_coding | 4:133240818-133252929 (+) |  | -1.0200 | 1.04e-18 | 4.58e-18 |
| ENSMUSG00000020691 | Mettl2 | protein\_coding | 11:105126425-105140394 (+) |  | 0.6250 | 1.07e-18 | 4.72e-18 |
| ENSMUSG00000041135 | Ripk2 | protein\_coding | 4:16122733-16163647 (-) |  | -0.9200 | 1.14e-18 | 4.99e-18 |
| ENSMUSG00000022768 | Ccdc116 | protein\_coding | 16:17139064-17147229 (-) |  | 2.6200 | 1.14e-18 | 4.99e-18 |
| ENSMUSG00000028882 | Ppp1r8 | protein\_coding | 4:132826929-132843169 (-) |  | 0.4320 | 1.14e-18 | 4.99e-18 |
| ENSMUSG00000038351 | Sgsm2 | protein\_coding | 11:74849261-74897060 (-) |  | 1.0100 | 1.14e-18 | 5.00e-18 |
| ENSMUSG00000026197 | Zfand2b | protein\_coding | 1:75168646-75171629 (+) |  | -0.6660 | 1.17e-18 | 5.12e-18 |
| ENSMUSG00000038740 | Mvb12b | protein\_coding | 2:33729953-33887946 (-) |  | 0.6810 | 1.17e-18 | 5.15e-18 |
| ENSMUSG00000024960 | Plcb3 | protein\_coding | 19:6952325-6976470 (-) |  | -0.5020 | 1.18e-18 | 5.18e-18 |
| ENSMUSG00000028719 | Cmpk1 | protein\_coding | 4:114959336-114987241 (-) |  | -0.4830 | 1.19e-18 | 5.23e-18 |
| ENSMUSG00000033952 | Aspm | protein\_coding | 1:139454772-139494091 (+) |  | -0.6370 | 1.20e-18 | 5.25e-18 |
| ENSMUSG00000033047 | Eif3l | protein\_coding | 15:79075179-79094405 (+) |  | 0.4290 | 1.22e-18 | 5.33e-18 |
| ENSMUSG00000021061 | Sptb | protein\_coding | 12:76580488-76710547 (-) |  | -1.4500 | 1.24e-18 | 5.44e-18 |
| ENSMUSG00000034252 | Senp6 | protein\_coding | 9:80066903-80144953 (+) |  | 0.3820 | 1.25e-18 | 5.47e-18 |
| ENSMUSG00000020948 | Klhl28 | protein\_coding | 12:64938833-64965534 (-) |  | -0.6420 | 1.34e-18 | 5.87e-18 |
| ENSMUSG00000029022 | Miip | protein\_coding | 4:147860778-147868816 (-) |  | -0.4250 | 1.35e-18 | 5.89e-18 |
| ENSMUSG00000039244 | E130309D02Rik | protein\_coding | 5:143301195-143315360 (-) |  | 0.5030 | 1.35e-18 | 5.89e-18 |
| ENSMUSG00000038180 | Spag4 | protein\_coding | 2:156065175-156070617 (+) |  | 3.5000 | 1.36e-18 | 5.94e-18 |
| ENSMUSG00000026663 | Atf6 | protein\_coding | 1:170704674-170867771 (-) |  | -0.4960 | 1.37e-18 | 5.98e-18 |
| ENSMUSG00000007038 | Neu1 | protein\_coding | 17:34931253-34935953 (+) |  | -0.5420 | 1.37e-18 | 5.98e-18 |
| ENSMUSG00000069520 | Tmem19 | protein\_coding | 10:115339674-115362501 (-) |  | -0.4380 | 1.40e-18 | 6.11e-18 |
| ENSMUSG00000043004 | Gng2 | protein\_coding | 14:19872559-19977627 (-) |  | -0.4600 | 1.44e-18 | 6.28e-18 |
| ENSMUSG00000097534 | Gm16675 | lncRNA | 8:46668808-46739515 (-) |  | -1.5800 | 1.45e-18 | 6.33e-18 |
| ENSMUSG00000029790 | Cep41 | protein\_coding | 6:30653457-30693749 (-) |  | 1.0900 | 1.53e-18 | 6.67e-18 |
| ENSMUSG00000031392 | Irak1 | protein\_coding | X:74013914-74023918 (-) |  | 0.3110 | 1.61e-18 | 7.04e-18 |
| ENSMUSG00000036352 | Ubac1 | protein\_coding | 2:25998543-26021747 (-) |  | 0.5560 | 1.62e-18 | 7.07e-18 |
| ENSMUSG00000025894 | Aasdhppt | protein\_coding | 9:4294793-4309471 (-) |  | 0.9990 | 1.63e-18 | 7.13e-18 |
| ENSMUSG00000055371 | Stam2 | protein\_coding | 2:52691664-52742281 (-) |  | -0.4610 | 1.78e-18 | 7.77e-18 |
| ENSMUSG00000072582 | Ptrh2 | protein\_coding | 11:86683985-86692457 (+) |  | 0.5760 | 1.96e-18 | 8.56e-18 |
| ENSMUSG00000001424 | Snd1 | protein\_coding | 6:28475139-28935162 (+) |  | 0.5040 | 2.01e-18 | 8.74e-18 |
| ENSMUSG00000029471 | Camkk2 | protein\_coding | 5:122731170-122779409 (-) |  | -0.8150 | 2.02e-18 | 8.80e-18 |
| ENSMUSG00000034126 | Pomt2 | protein\_coding | 12:87106861-87147968 (-) |  | 0.7590 | 2.04e-18 | 8.87e-18 |
| ENSMUSG00000029687 | Ezh2 | protein\_coding | 6:47530139-47595341 (-) |  | 0.5100 | 2.04e-18 | 8.87e-18 |
| ENSMUSG00000020018 | Snrpf | protein\_coding | 10:93583029-93589706 (-) |  | 0.7810 | 2.06e-18 | 8.99e-18 |
| ENSMUSG00000027671 | Actl6a | protein\_coding | 3:32706298-32726973 (+) |  | 0.4190 | 2.09e-18 | 9.08e-18 |
| ENSMUSG00000038855 | Itpkb | protein\_coding | 1:180330485-180424802 (+) |  | -0.5630 | 2.13e-18 | 9.27e-18 |
| ENSMUSG00000029657 | Hsph1 | protein\_coding | 5:149614287-149636376 (-) |  | 0.9580 | 2.13e-18 | 9.27e-18 |
| ENSMUSG00000053819 | Camk2d | protein\_coding | 3:126596302-126846326 (+) |  | -1.6100 | 2.17e-18 | 9.45e-18 |
| ENSMUSG00000039789 | Zfp597 | protein\_coding | 16:3858321-3884561 (-) |  | -0.5910 | 2.17e-18 | 9.45e-18 |
| ENSMUSG00000032470 | Mras | protein\_coding | 9:99385420-99437381 (-) |  | 2.5300 | 2.23e-18 | 9.67e-18 |
| ENSMUSG00000036975 | Tmem177 | protein\_coding | 1:119907890-119913208 (-) |  | 1.0100 | 2.31e-18 | 1.00e-17 |
| ENSMUSG00000032251 | Irak1bp1 | protein\_coding | 9:82829540-82847687 (+) |  | 3.7000 | 2.33e-18 | 1.01e-17 |
| ENSMUSG00000036587 | Fut7 | protein\_coding | 2:25423267-25426374 (+) |  | 1.1700 | 2.36e-18 | 1.03e-17 |
| ENSMUSG00000051675 | Trim32 | protein\_coding | 4:65604986-65616238 (+) |  | 0.8410 | 2.37e-18 | 1.03e-17 |
| ENSMUSG00000004897 | Hdgf | protein\_coding | 3:87906321-87916132 (+) |  | 0.4750 | 2.39e-18 | 1.04e-17 |
| ENSMUSG00000029004 | Kmt2e | protein\_coding | 5:23434441-23504235 (+) |  | -0.5090 | 2.39e-18 | 1.04e-17 |
| ENSMUSG00000032513 | Gorasp1 | protein\_coding | 9:119925257-119937565 (-) |  | 0.5970 | 2.45e-18 | 1.06e-17 |
| ENSMUSG00000054836 | Elp6 | protein\_coding | 9:110305173-110322102 (+) |  | 0.8580 | 2.45e-18 | 1.06e-17 |
| ENSMUSG00000032688 | Malt1 | protein\_coding | 18:65430938-65479067 (+) |  | -0.5530 | 2.46e-18 | 1.07e-17 |
| ENSMUSG00000084883 | Ccdc85c | protein\_coding | 12:108203602-108275425 (-) |  | 1.9200 | 2.50e-18 | 1.08e-17 |
| ENSMUSG00000052926 | Rnaseh2a | protein\_coding | 8:84956610-84969767 (-) |  | 0.5910 | 2.57e-18 | 1.11e-17 |
| ENSMUSG00000065126 | Snord104 | snoRNA | 11:106500993-106501062 (+) |  | 1.3500 | 2.62e-18 | 1.14e-17 |
| ENSMUSG00000028653 | Trit1 | protein\_coding | 4:123016597-123054949 (+) |  | 0.6540 | 2.71e-18 | 1.18e-17 |
| ENSMUSG00000029146 | Snx17 | protein\_coding | 5:31193227-31199143 (+) |  | -0.3450 | 2.74e-18 | 1.18e-17 |
| ENSMUSG00000031786 | Drc7 | protein\_coding | 8:95055103-95078141 (+) |  | 6.5600 | 2.82e-18 | 1.22e-17 |
| ENSMUSG00000112302 | Gm48226 | lncRNA | 12:78222286-78226525 (-) |  | 1.1900 | 2.87e-18 | 1.24e-17 |
| ENSMUSG00000052337 | Immt | protein\_coding | 6:71831331-71877388 (+) |  | 0.3580 | 2.87e-18 | 1.24e-17 |
| ENSMUSG00000051278 | Zgrf1 | protein\_coding | 3:127553489-127618023 (+) |  | 0.9890 | 2.88e-18 | 1.25e-17 |
| ENSMUSG00000039997 | Ifi203 | protein\_coding | 1:173920407-173942672 (-) |  | -0.7640 | 2.89e-18 | 1.25e-17 |
| ENSMUSG00000034640 | Tiparp | protein\_coding | 3:65528410-65555518 (+) |  | -0.5220 | 2.91e-18 | 1.26e-17 |
| ENSMUSG00000033068 | Entpd6 | protein\_coding | 2:150749042-150771675 (+) |  | 0.6750 | 2.97e-18 | 1.28e-17 |
| ENSMUSG00000034485 | Uaca | protein\_coding | 9:60794542-60880370 (+) |  | 3.2300 | 2.99e-18 | 1.29e-17 |
| ENSMUSG00000037552 | Plekhg2 | protein\_coding | 7:28359604-28372599 (-) |  | -0.9120 | 3.02e-18 | 1.30e-17 |
| ENSMUSG00000034442 | Trmt5 | protein\_coding | 12:73280011-73286710 (-) |  | 0.7500 | 3.08e-18 | 1.33e-17 |
| ENSMUSG00000050737 | Ptges | protein\_coding | 2:30889471-30929863 (-) |  | -1.3200 | 3.09e-18 | 1.33e-17 |
| ENSMUSG00000041570 | Camsap2 | protein\_coding | 1:136268123-136346104 (-) |  | -0.5050 | 3.12e-18 | 1.35e-17 |
| ENSMUSG00000036817 | Sun1 | protein\_coding | 5:139200637-139249840 (+) |  | -0.4950 | 3.13e-18 | 1.35e-17 |
| ENSMUSG00000054612 | Mgmt | protein\_coding | 7:136894614-137130266 (+) |  | 1.9900 | 3.13e-18 | 1.35e-17 |
| ENSMUSG00000028927 | Padi2 | protein\_coding | 4:140906344-140952586 (+) |  | -1.4200 | 3.21e-18 | 1.38e-17 |
| ENSMUSG00000000058 | Cav2 | protein\_coding | 6:17281185-17289115 (+) |  | -0.9180 | 3.21e-18 | 1.38e-17 |
| ENSMUSG00000032024 | Clmp | protein\_coding | 9:40685962-40785319 (+) |  | -1.7400 | 3.24e-18 | 1.40e-17 |
| ENSMUSG00000066621 | Tecpr1 | protein\_coding | 5:144194442-144223615 (-) |  | -0.7910 | 3.25e-18 | 1.40e-17 |
| ENSMUSG00000028161 | Ppp3ca | protein\_coding | 3:136670124-136937727 (+) |  | -0.3570 | 3.31e-18 | 1.43e-17 |
| ENSMUSG00000016984 | Etaa1 | protein\_coding | 11:17938756-17953875 (-) |  | 0.4900 | 3.37e-18 | 1.45e-17 |
| ENSMUSG00000070643 | Sox13 | protein\_coding | 1:133382303-133424377 (-) |  | 2.9100 | 3.46e-18 | 1.49e-17 |
| ENSMUSG00000028383 | Hsdl2 | protein\_coding | 4:59581563-59618689 (+) |  | 0.5480 | 3.65e-18 | 1.57e-17 |
| ENSMUSG00000030613 | Ccdc90b | protein\_coding | 7:92561149-92582294 (+) |  | 0.7450 | 3.68e-18 | 1.58e-17 |
| ENSMUSG00000032046 | Abhd12 | protein\_coding | 2:150832493-150904741 (-) |  | -0.4490 | 3.72e-18 | 1.60e-17 |
| ENSMUSG00000046519 | Golph3l | protein\_coding | 3:95588934-95619247 (+) |  | -0.4480 | 3.77e-18 | 1.62e-17 |
| ENSMUSG00000017561 | Crlf3 | protein\_coding | 11:80046493-80080991 (-) |  | -0.4030 | 3.79e-18 | 1.63e-17 |
| ENSMUSG00000022867 | Usp25 | protein\_coding | 16:77013706-77116780 (+) |  | -0.4050 | 3.81e-18 | 1.64e-17 |
| ENSMUSG00000049800 | Sertad2 | protein\_coding | 11:20543253-20653021 (+) |  | -0.5890 | 3.96e-18 | 1.70e-17 |
| ENSMUSG00000052724 | Gm9888 | lncRNA | 9:114780100-114782257 (+) |  | 1.3400 | 4.22e-18 | 1.81e-17 |
| ENSMUSG00000116114 | Gm35853 | lncRNA | 15:101322888-101405834 (+) |  | -1.8500 | 4.22e-18 | 1.81e-17 |
| ENSMUSG00000021180 | Rps6ka5 | protein\_coding | 12:100548439-100726983 (-) |  | 0.3500 | 4.24e-18 | 1.82e-17 |
| ENSMUSG00000066839 | Ecsit | protein\_coding | 9:22072246-22085438 (-) |  | 0.7910 | 4.28e-18 | 1.84e-17 |
| ENSMUSG00000070923 | Klhl9 | protein\_coding | 4:88718292-88722465 (-) |  | -0.4790 | 4.34e-18 | 1.86e-17 |
| ENSMUSG00000110206 | Flt3l | protein\_coding | 7:45129788-45136432 (-) |  | -1.2600 | 4.52e-18 | 1.94e-17 |
| ENSMUSG00000060743 | H3f3a | protein\_coding | 1:180800832-180813943 (-) |  | -0.6830 | 4.53e-18 | 1.94e-17 |
| ENSMUSG00000029480 | Dhx37 | protein\_coding | 5:125413858-125434121 (-) |  | 0.6900 | 4.58e-18 | 1.96e-17 |
| ENSMUSG00000029475 | Kdm2b | protein\_coding | 5:122870665-122989823 (-) |  | 0.5790 | 4.77e-18 | 2.04e-17 |
| ENSMUSG00000007080 | Pole | protein\_coding | 5:110286306-110337474 (+) |  | 1.0100 | 4.78e-18 | 2.05e-17 |
| ENSMUSG00000020946 | Gosr2 | protein\_coding | 11:103676849-103697898 (-) |  | -0.3260 | 4.82e-18 | 2.06e-17 |
| ENSMUSG00000039697 | Ncoa7 | protein\_coding | 10:30628999-30803326 (-) |  | -0.5500 | 4.84e-18 | 2.07e-17 |
| ENSMUSG00000046229 | Scand1 | protein\_coding | 2:156311846-156312747 (-) |  | -0.7140 | 5.00e-18 | 2.14e-17 |
| ENSMUSG00000031012 | Cask | protein\_coding | X:13517080-13851367 (-) |  | -0.5360 | 5.17e-18 | 2.21e-17 |
| ENSMUSG00000049904 | Tmem17 | protein\_coding | 11:22512088-22519234 (+) |  | 2.0300 | 5.24e-18 | 2.24e-17 |
| ENSMUSG00000031133 | Arhgef6 | protein\_coding | X:57231485-57338729 (-) |  | -0.3350 | 5.26e-18 | 2.25e-17 |
| ENSMUSG00000024968 | Rcor2 | protein\_coding | 19:7267325-7275225 (+) |  | 3.0800 | 5.33e-18 | 2.28e-17 |
| ENSMUSG00000029759 | Pon3 | protein\_coding | 6:5220852-5256286 (-) |  | 0.9210 | 5.42e-18 | 2.31e-17 |
| ENSMUSG00000027698 | Nceh1 | protein\_coding | 3:27182965-27284608 (+) |  | -0.5630 | 5.51e-18 | 2.35e-17 |
| ENSMUSG00000090231 | Cfb | protein\_coding | 17:34856374-34862518 (-) |  | -3.2100 | 5.65e-18 | 2.41e-17 |
| ENSMUSG00000066877 | Nck2 | protein\_coding | 1:43444579-43570515 (+) |  | 1.3700 | 5.84e-18 | 2.49e-17 |
| ENSMUSG00000030978 | Rrm1 | protein\_coding | 7:102441695-102469771 (+) |  | 0.6270 | 5.86e-18 | 2.50e-17 |
| ENSMUSG00000058587 | Tmod3 | protein\_coding | 9:75497707-75559657 (-) |  | -0.4070 | 6.02e-18 | 2.57e-17 |
| ENSMUSG00000067071 | Hes6 | protein\_coding | 1:91411483-91414038 (-) |  | 0.5220 | 6.10e-18 | 2.60e-17 |
| ENSMUSG00000022223 | Sdr39u1 | protein\_coding | 14:55897283-55900293 (-) |  | 1.4500 | 6.14e-18 | 2.62e-17 |
| ENSMUSG00000026944 | Abca2 | protein\_coding | 2:25428703-25448540 (+) |  | 0.7380 | 6.28e-18 | 2.68e-17 |
| ENSMUSG00000031320 | Rps4x | protein\_coding | X:102184941-102189394 (-) |  | 0.3940 | 6.52e-18 | 2.78e-17 |
| ENSMUSG00000022214 | Dcaf11 | protein\_coding | 14:55560006-55570065 (+) |  | -0.4450 | 6.57e-18 | 2.80e-17 |
| ENSMUSG00000021466 | Ptch1 | protein\_coding | 13:63508328-63573598 (-) |  | 1.1200 | 6.63e-18 | 2.82e-17 |
| ENSMUSG00000030515 | Tarsl2 | protein\_coding | 7:65644898-65692091 (+) |  | 0.7550 | 6.64e-18 | 2.83e-17 |
| ENSMUSG00000044827 | Tlr1 | protein\_coding | 5:64924679-64933563 (-) |  | -0.8880 | 6.64e-18 | 2.83e-17 |
| ENSMUSG00000036591 | Arhgap21 | protein\_coding | 2:20847919-20968881 (-) |  | 0.8520 | 6.65e-18 | 2.83e-17 |
| ENSMUSG00000021774 | Ube2e1 | protein\_coding | 14:18282722-18331859 (-) |  | 0.6580 | 6.70e-18 | 2.85e-17 |
| ENSMUSG00000028223 | Decr1 | protein\_coding | 4:15917240-15945507 (-) |  | 0.7540 | 6.73e-18 | 2.86e-17 |
| ENSMUSG00000029407 | Uso1 | protein\_coding | 5:92137938-92202798 (+) |  | -0.3980 | 6.76e-18 | 2.87e-17 |
| ENSMUSG00000059263 | Usp47 | protein\_coding | 7:112023504-112111661 (+) |  | -0.2870 | 6.83e-18 | 2.90e-17 |
| ENSMUSG00000037622 | Wdtc1 | protein\_coding | 4:133292459-133353481 (-) |  | -0.5580 | 6.95e-18 | 2.95e-17 |
| ENSMUSG00000013150 | Gfod2 | protein\_coding | 8:105713854-105758664 (-) |  | 2.4900 | 7.05e-18 | 2.99e-17 |
| ENSMUSG00000053841 | Txlna | protein\_coding | 4:129626078-129641065 (-) |  | 0.4150 | 7.16e-18 | 3.04e-17 |
| ENSMUSG00000052914 | Cyp2j6 | protein\_coding | 4:96516138-96553661 (-) |  | 4.1500 | 7.49e-18 | 3.18e-17 |
| ENSMUSG00000029465 | Arpc3 | protein\_coding | 5:122391878-122414184 (+) |  | -0.5220 | 7.55e-18 | 3.20e-17 |
| ENSMUSG00000056938 | Acbd4 | protein\_coding | 11:103101682-103112200 (+) |  | -0.9990 | 7.91e-18 | 3.35e-17 |
| ENSMUSG00000047045 | Tmem164 | protein\_coding | X:142681406-142843494 (+) |  | -0.4250 | 7.98e-18 | 3.39e-17 |
| ENSMUSG00000027340 | Slc23a2 | protein\_coding | 2:132052496-132145108 (-) |  | -0.5730 | 8.27e-18 | 3.51e-17 |
| ENSMUSG00000026189 | Pecr | protein\_coding | 1:72259167-72284314 (-) |  | 2.7800 | 8.48e-18 | 3.59e-17 |
| ENSMUSG00000045503 | Sys1 | protein\_coding | 2:164456964-164479638 (+) |  | -0.4920 | 8.53e-18 | 3.61e-17 |
| ENSMUSG00000020652 | Cenpo | protein\_coding | 12:4196004-4234294 (-) |  | 0.5230 | 8.81e-18 | 3.73e-17 |
| ENSMUSG00000032232 | Cgnl1 | protein\_coding | 9:71626509-71771602 (-) |  | -1.7600 | 8.93e-18 | 3.78e-17 |
| ENSMUSG00000009876 | Cox4i2 | protein\_coding | 2:152754173-152765037 (+) |  | 2.9500 | 9.10e-18 | 3.85e-17 |
| ENSMUSG00000094594 | Tpd52-ps | processed\_pseudogene | 8:79680284-79680806 (-) |  | -1.0600 | 9.11e-18 | 3.86e-17 |
| ENSMUSG00000039804 | Ncoa5 | protein\_coding | 2:165000357-165034867 (-) |  | 0.4120 | 9.19e-18 | 3.89e-17 |
| ENSMUSG00000037364 | Srrt | protein\_coding | 5:137295704-137307674 (-) |  | 0.3970 | 9.31e-18 | 3.94e-17 |
| ENSMUSG00000030990 | Pgap2 | protein\_coding | 7:102210208-102238567 (+) |  | 0.5570 | 9.61e-18 | 4.06e-17 |
| ENSMUSG00000027663 | Zmat3 | protein\_coding | 3:32334792-32366014 (-) |  | -1.1100 | 9.75e-18 | 4.12e-17 |
| ENSMUSG00000068580 | Zfyve19 | protein\_coding | 2:119208617-119217049 (+) |  | 0.6070 | 9.86e-18 | 4.17e-17 |
| ENSMUSG00000032498 | Mlh1 | protein\_coding | 9:111228228-111271791 (-) |  | 0.5460 | 1.00e-17 | 4.24e-17 |
| ENSMUSG00000025766 | D3Ertd751e | protein\_coding | 3:41742611-41803320 (+) |  | 1.0600 | 1.05e-17 | 4.46e-17 |
| ENSMUSG00000048118 | Arid4a | protein\_coding | 12:71015990-71098592 (+) |  | -0.4940 | 1.07e-17 | 4.52e-17 |
| ENSMUSG00000059013 | Sh2d3c | protein\_coding | 2:32721055-32755512 (+) |  | -0.8180 | 1.08e-17 | 4.54e-17 |
| ENSMUSG00000026585 | Kifap3 | protein\_coding | 1:163779583-163917109 (+) |  | 0.6530 | 1.09e-17 | 4.60e-17 |
| ENSMUSG00000028412 | Slc44a1 | protein\_coding | 4:53440413-53622478 (+) |  | -0.9240 | 1.11e-17 | 4.67e-17 |
| ENSMUSG00000030826 | Bcat2 | protein\_coding | 7:45570153-45589711 (+) |  | 0.4650 | 1.11e-17 | 4.67e-17 |
| ENSMUSG00000064090 | Vrk2 | protein\_coding | 11:26471322-26593999 (-) |  | -0.5780 | 1.14e-17 | 4.80e-17 |
| ENSMUSG00000022620 | Arsa | protein\_coding | 15:89472476-89477425 (-) |  | -0.5750 | 1.16e-17 | 4.88e-17 |
| ENSMUSG00000024228 | Nudt12 | protein\_coding | 17:58999618-59013372 (-) |  | 1.8700 | 1.17e-17 | 4.92e-17 |
| ENSMUSG00000056004 | 9330182L06Rik | protein\_coding | 5:9266118-9481825 (+) |  | 1.7700 | 1.22e-17 | 5.13e-17 |
| ENSMUSG00000074807 | Gm10762 | lncRNA | 2:128965505-128968044 (-) |  | 1.2600 | 1.24e-17 | 5.21e-17 |
| ENSMUSG00000032366 | Tpm1 | protein\_coding | 9:67022590-67049406 (-) |  | 0.5700 | 1.24e-17 | 5.23e-17 |
| ENSMUSG00000039740 | Alg2 | protein\_coding | 4:47465067-47474333 (-) |  | 0.6400 | 1.25e-17 | 5.25e-17 |
| ENSMUSG00000025878 | Uimc1 | protein\_coding | 13:55027880-55100300 (-) |  | -0.4290 | 1.35e-17 | 5.68e-17 |
| ENSMUSG00000056394 | Lig1 | protein\_coding | 7:13277283-13311433 (+) |  | 0.8160 | 1.36e-17 | 5.72e-17 |
| ENSMUSG00000011958 | Bnip2 | protein\_coding | 9:69989466-70008317 (+) |  | -0.3450 | 1.44e-17 | 6.08e-17 |
| ENSMUSG00000039765 | Cc2d2a | protein\_coding | 5:43662346-43740972 (+) |  | 2.3700 | 1.45e-17 | 6.09e-17 |
| ENSMUSG00000036249 | Rbm43 | protein\_coding | 2:51924448-51935163 (-) |  | -0.5400 | 1.46e-17 | 6.16e-17 |
| ENSMUSG00000030055 | Rab43 | protein\_coding | 6:87788853-87812164 (-) |  | -0.8330 | 1.51e-17 | 6.35e-17 |
| ENSMUSG00000087107 | AI662270 | lncRNA | 11:83223576-83226604 (+) |  | 0.4310 | 1.51e-17 | 6.36e-17 |
| ENSMUSG00000015053 | Gata2 | protein\_coding | 6:88193891-88207032 (+) |  | 2.9700 | 1.57e-17 | 6.60e-17 |
| ENSMUSG00000075266 | Cenpw | protein\_coding | 10:30194569-30200560 (-) |  | 0.8730 | 1.57e-17 | 6.61e-17 |
| ENSMUSG00000016024 | Lbp | protein\_coding | 2:158306493-158332852 (+) |  | 1.1100 | 1.64e-17 | 6.89e-17 |
| ENSMUSG00000033487 | Fndc3a | protein\_coding | 14:72537946-72710003 (-) |  | -0.4470 | 1.71e-17 | 7.17e-17 |
| ENSMUSG00000036086 | Zranb3 | protein\_coding | 1:127954184-128103047 (-) |  | 1.0300 | 1.76e-17 | 7.38e-17 |
| ENSMUSG00000031198 | Fundc2 | protein\_coding | X:75382399-75396459 (+) |  | 0.5800 | 1.85e-17 | 7.76e-17 |
| ENSMUSG00000055717 | Slain1 | protein\_coding | 14:103650228-103704907 (+) |  | 3.2600 | 1.87e-17 | 7.86e-17 |
| ENSMUSG00000068732 | Tmem167b | protein\_coding | 3:108556425-108562466 (-) |  | -0.4230 | 1.88e-17 | 7.90e-17 |
| ENSMUSG00000050323 | Ndufaf6 | protein\_coding | 4:11051045-11076205 (-) |  | 1.0200 | 2.01e-17 | 8.42e-17 |
| ENSMUSG00000033871 | Ppargc1b | protein\_coding | 18:61298136-61400431 (-) |  | 1.1800 | 2.05e-17 | 8.59e-17 |
| ENSMUSG00000021485 | Mxd3 | protein\_coding | 13:55325168-55329823 (-) |  | -0.7250 | 2.07e-17 | 8.69e-17 |
| ENSMUSG00000071477 | Zfp777 | protein\_coding | 6:48024188-48048911 (-) |  | 0.6670 | 2.12e-17 | 8.89e-17 |
| ENSMUSG00000029314 | Gpat3 | protein\_coding | 5:100845713-100899102 (+) |  | -2.2900 | 2.20e-17 | 9.21e-17 |
| ENSMUSG00000020235 | Fzr1 | protein\_coding | 10:81366222-81378516 (-) |  | -0.3560 | 2.27e-17 | 9.52e-17 |
| ENSMUSG00000015488 | Cacfd1 | protein\_coding | 2:27009926-27021089 (+) |  | -0.5660 | 2.30e-17 | 9.62e-17 |
| ENSMUSG00000000301 | Pemt | protein\_coding | 11:59970614-60046489 (-) |  | 1.8800 | 2.33e-17 | 9.74e-17 |
| ENSMUSG00000020021 | Fgd6 | protein\_coding | 10:94036001-94145339 (+) |  | -1.4300 | 2.38e-17 | 9.93e-17 |
| ENSMUSG00000022718 | Dgcr8 | protein\_coding | 16:18253948-18289246 (-) |  | 0.4700 | 2.47e-17 | 1.03e-16 |
| ENSMUSG00000097585 | E230029C05Rik | lncRNA | 7:89980723-90049069 (+) |  | -1.8800 | 2.49e-17 | 1.04e-16 |
| ENSMUSG00000007739 | Cct4 | protein\_coding | 11:22990519-23003780 (+) |  | 0.4710 | 2.52e-17 | 1.05e-16 |
| ENSMUSG00000045071 | E130308A19Rik | protein\_coding | 4:59626211-59761439 (+) |  | 0.8520 | 2.62e-17 | 1.09e-16 |
| ENSMUSG00000075268 | Gm10819 | processed\_pseudogene | 19:16267061-16267621 (-) |  | 1.4800 | 2.63e-17 | 1.10e-16 |
| ENSMUSG00000021144 | Mta1 | protein\_coding | 12:113098278-113137206 (+) |  | 0.5470 | 2.64e-17 | 1.10e-16 |
| ENSMUSG00000004771 | Rab11a | protein\_coding | 9:64715299-64737758 (-) |  | -0.6910 | 2.69e-17 | 1.12e-16 |
| ENSMUSG00000043323 | Fbrsl1 | protein\_coding | 5:110361754-110448602 (-) |  | -0.5560 | 2.72e-17 | 1.13e-16 |
| ENSMUSG00000032316 | Clk3 | protein\_coding | 9:57750624-57765860 (-) |  | -0.4710 | 2.76e-17 | 1.15e-16 |
| ENSMUSG00000002767 | Mrpl2 | protein\_coding | 17:46646229-46650139 (+) |  | 0.4050 | 2.82e-17 | 1.17e-16 |
| ENSMUSG00000097993 | Ptprv | polymorphic\_pseudogene | 1:135108497-135132594 (-) |  | 1.0600 | 2.88e-17 | 1.20e-16 |
| ENSMUSG00000026275 | Ppp1r7 | protein\_coding | 1:93342854-93373489 (+) |  | 0.4020 | 2.90e-17 | 1.21e-16 |
| ENSMUSG00000049878 | Rlf | protein\_coding | 4:121145373-121215084 (-) |  | -0.6010 | 2.92e-17 | 1.22e-16 |
| ENSMUSG00000003865 | Gys1 | protein\_coding | 7:45434844-45456619 (+) |  | 0.7990 | 3.05e-17 | 1.27e-16 |
| ENSMUSG00000022248 | Rad1 | protein\_coding | 15:10486018-10499063 (+) |  | 0.7110 | 3.10e-17 | 1.29e-16 |
| ENSMUSG00000097488 | 4732487G21Rik | lncRNA | 12:86290761-86306006 (-) |  | -1.0000 | 3.12e-17 | 1.30e-16 |
| ENSMUSG00000026727 | Rsu1 | protein\_coding | 2:13076821-13271415 (-) |  | -0.4300 | 3.15e-17 | 1.31e-16 |
| ENSMUSG00000084806 | Gm15232 | lncRNA | X:167088093-167102090 (-) |  | -2.3800 | 3.22e-17 | 1.34e-16 |
[truncated: 600,528 more chars]
