# Supplementary material for: Transcriptome profiling of osteoclast subsets associated with arthritis: A pathogenic role of CCR2hi osteoclast progenitors
Source: Front Immunol. 2022 Dec 15;13:994035. doi: 10.3389/fimmu.2022.994035 (PMC9797520; doi:10.3389/fimmu.2022.994035)
Supplement: Supplementary file 10 [file DataSheet_2.zip › Supplementary data 2 DGE CCR2lo vs CCR2hi all samples/Supplementary data 2 legend.docx]

Supplementary data 2. Differential gene expression analysis of CCR2^lo^ and CCR2^hi^ osteoclast progenitor cells from all samples (both collagen-induced arthritis (DG1-8) and control mice (DG9-16)). CCR2^lo^ osteoclast progenitor subsets were compared to CCR2^hi^ subset using DESeq 2 and a total of 9005 genes with a Benjamini-Hochberg (BH) correction adjusted p value lower than 0.01 were listed.

First column denotes Ensembl gene ID, followed by gene name in second column, gene type in third column and chromosomal location in the fourth column. Comparison graphs in the fifth column were ommited as to meet the file size constraints.

Sixth column denoted the difference in expression of genes between the osteoclast progenitor subsets using log_2_ fold change comparison of CCR2^lo^ gene count compared to CCR2^hi^ gene count (more negative numbers equal higher expression in CCR2^hi^ subset and vice-versa).

p values obtained using Wald test are noted, as well as BH-correction adjusted p values in the last column. The columns are sortable by clicking on column heading, and all results are searchable by inputting text into the search box in the upper right corner.

The data is accessed using the provided .html file, while the folders contain accessory files needed for .html functionality.
