# Supplementary figures and images for: Transcriptome profiling of osteoclast subsets associated with arthritis: A pathogenic role of CCR2hi osteoclast progenitors (part 1 of 2)
Source: Front Immunol. 2022 Dec 15;13:994035. doi: 10.3389/fimmu.2022.994035 (PMC9797520; doi:10.3389/fimmu.2022.994035)

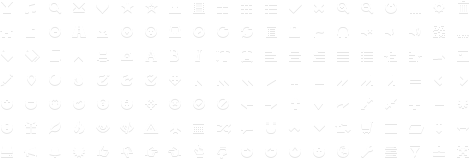

Supplement: Supplementary file 10 [file DataSheet_2.zip › Supplementary data 2 DGE CCR2lo vs CCR2hi all samples/csslib/images/glyphicons-halflings-white.png]

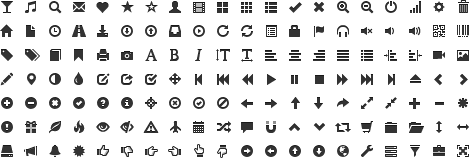

Supplement: Supplementary file 10 [file DataSheet_2.zip › Supplementary data 2 DGE CCR2lo vs CCR2hi all samples/csslib/images/glyphicons-halflings.png]

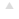

Supplement: Supplementary file 10 [file DataSheet_2.zip › Supplementary data 2 DGE CCR2lo vs CCR2hi all samples/csslib/images/sort_asc.png]

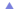

Supplement: Supplementary file 10 [file DataSheet_2.zip › Supplementary data 2 DGE CCR2lo vs CCR2hi all samples/csslib/images/sort_asc_disabled.png]

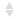

Supplement: Supplementary file 10 [file DataSheet_2.zip › Supplementary data 2 DGE CCR2lo vs CCR2hi all samples/csslib/images/sort_both.png]

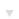

Supplement: Supplementary file 10 [file DataSheet_2.zip › Supplementary data 2 DGE CCR2lo vs CCR2hi all samples/csslib/images/sort_desc.png]

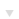

Supplement: Supplementary file 10 [file DataSheet_2.zip › Supplementary data 2 DGE CCR2lo vs CCR2hi all samples/csslib/images/sort_desc_disabled.png]

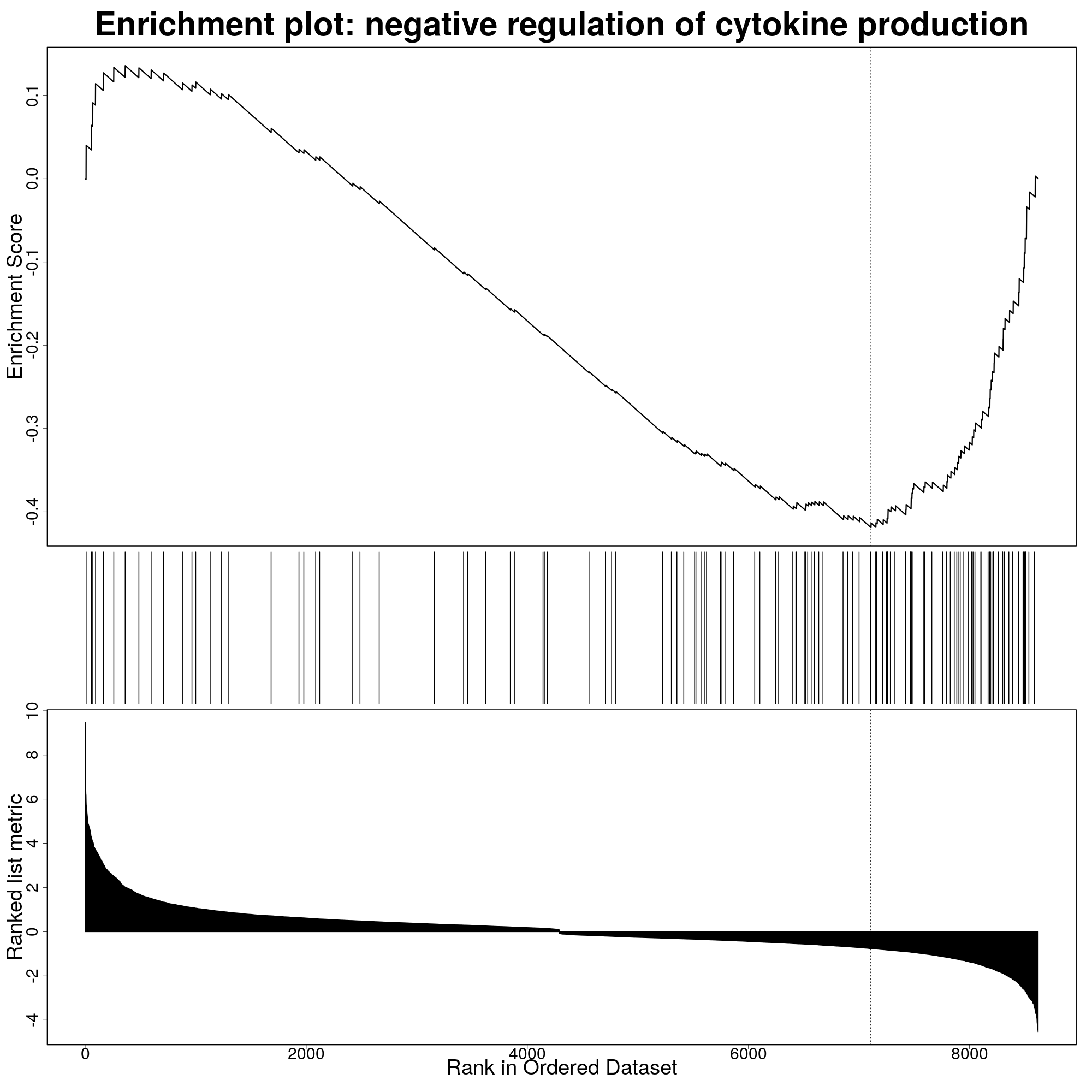

Supplement: Supplementary file 14 [file DataSheet_6.zip › Supplementary data 6 GSEA CCR2lo vs CCR2hi all samples/Project_high_vs_low_GSEA/GO_0001818.png]

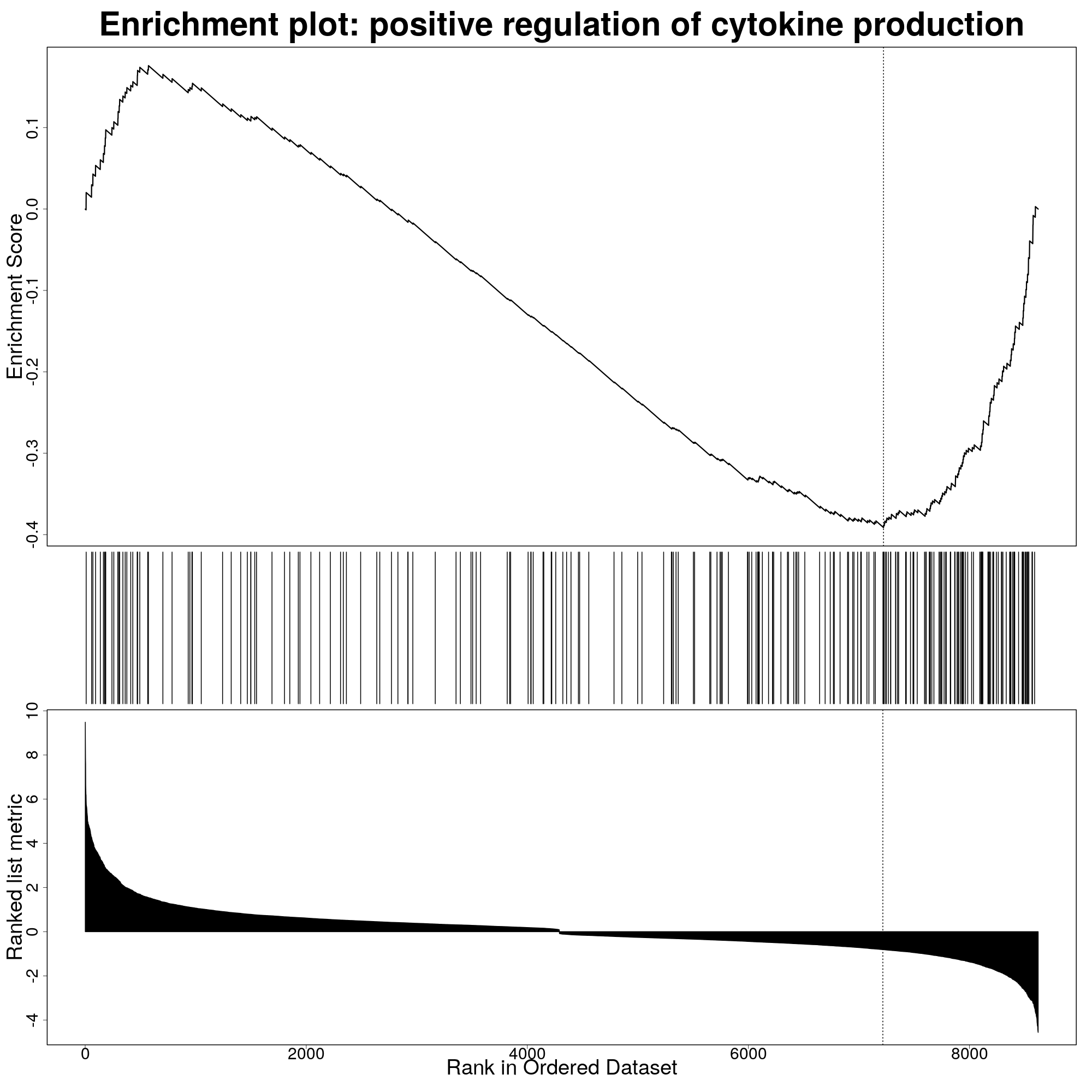

Supplement: Supplementary file 14 [file DataSheet_6.zip › Supplementary data 6 GSEA CCR2lo vs CCR2hi all samples/Project_high_vs_low_GSEA/GO_0001819.png]

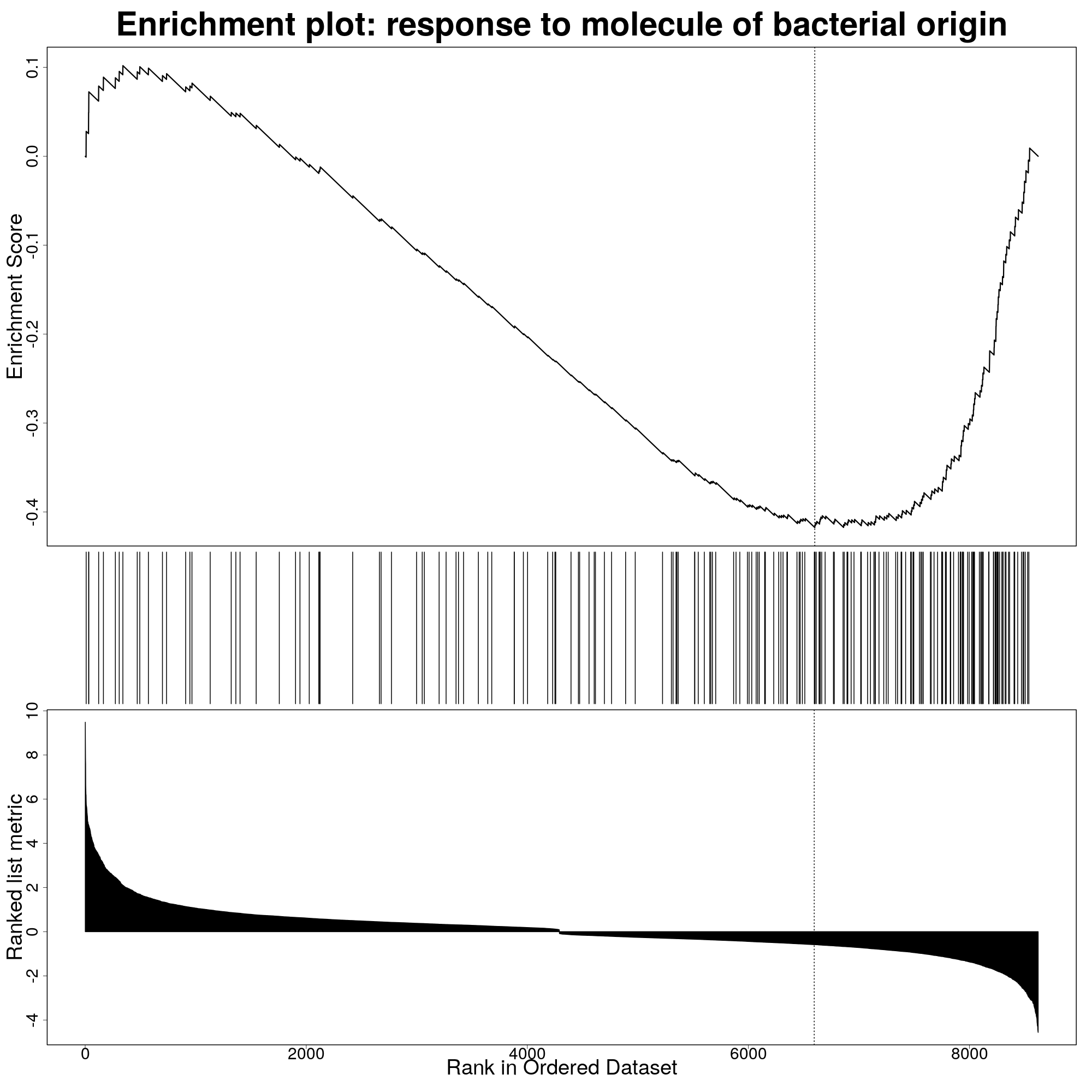

Supplement: Supplementary file 14 [file DataSheet_6.zip › Supplementary data 6 GSEA CCR2lo vs CCR2hi all samples/Project_high_vs_low_GSEA/GO_0002237.png]

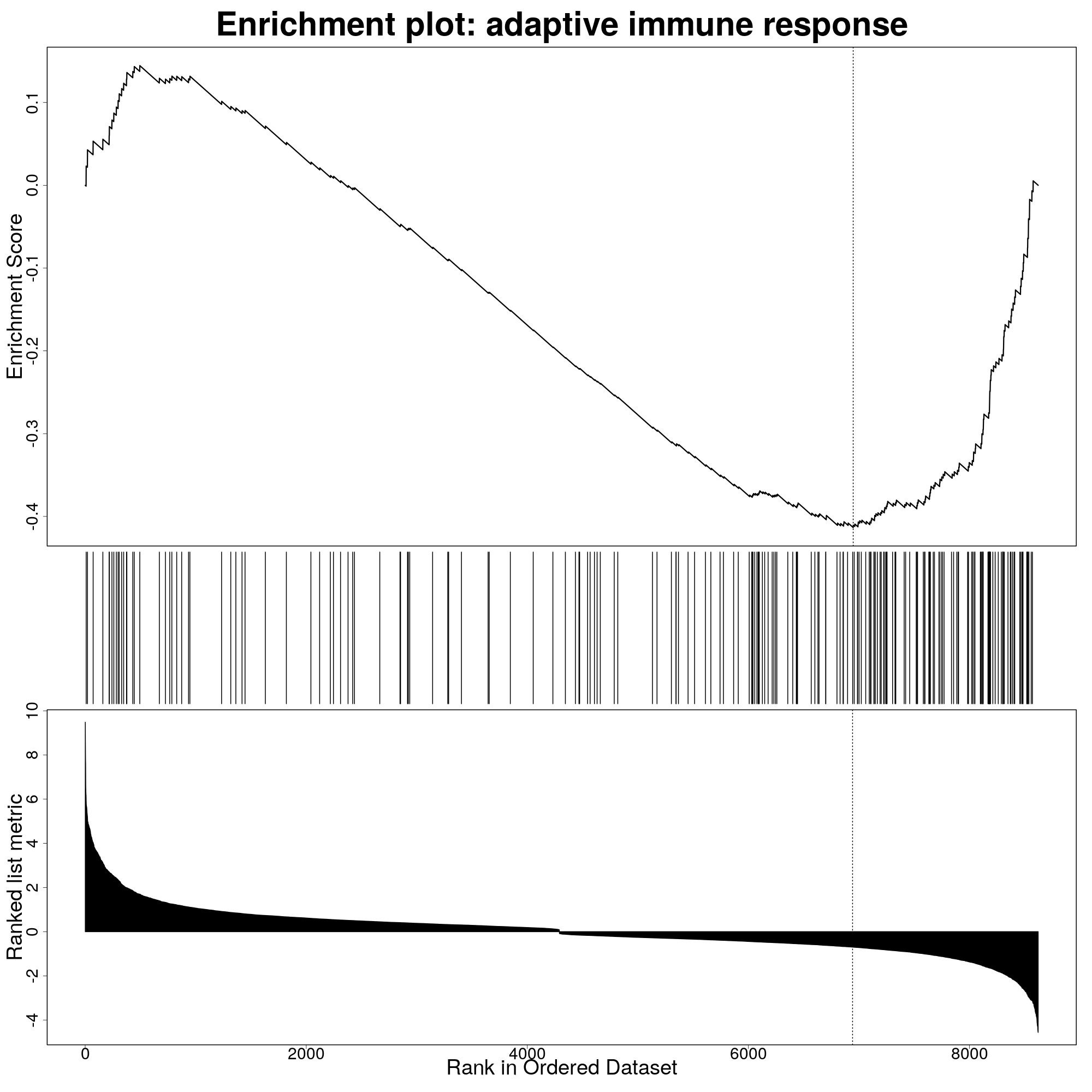

Supplement: Supplementary file 14 [file DataSheet_6.zip › Supplementary data 6 GSEA CCR2lo vs CCR2hi all samples/Project_high_vs_low_GSEA/GO_0002250.png]

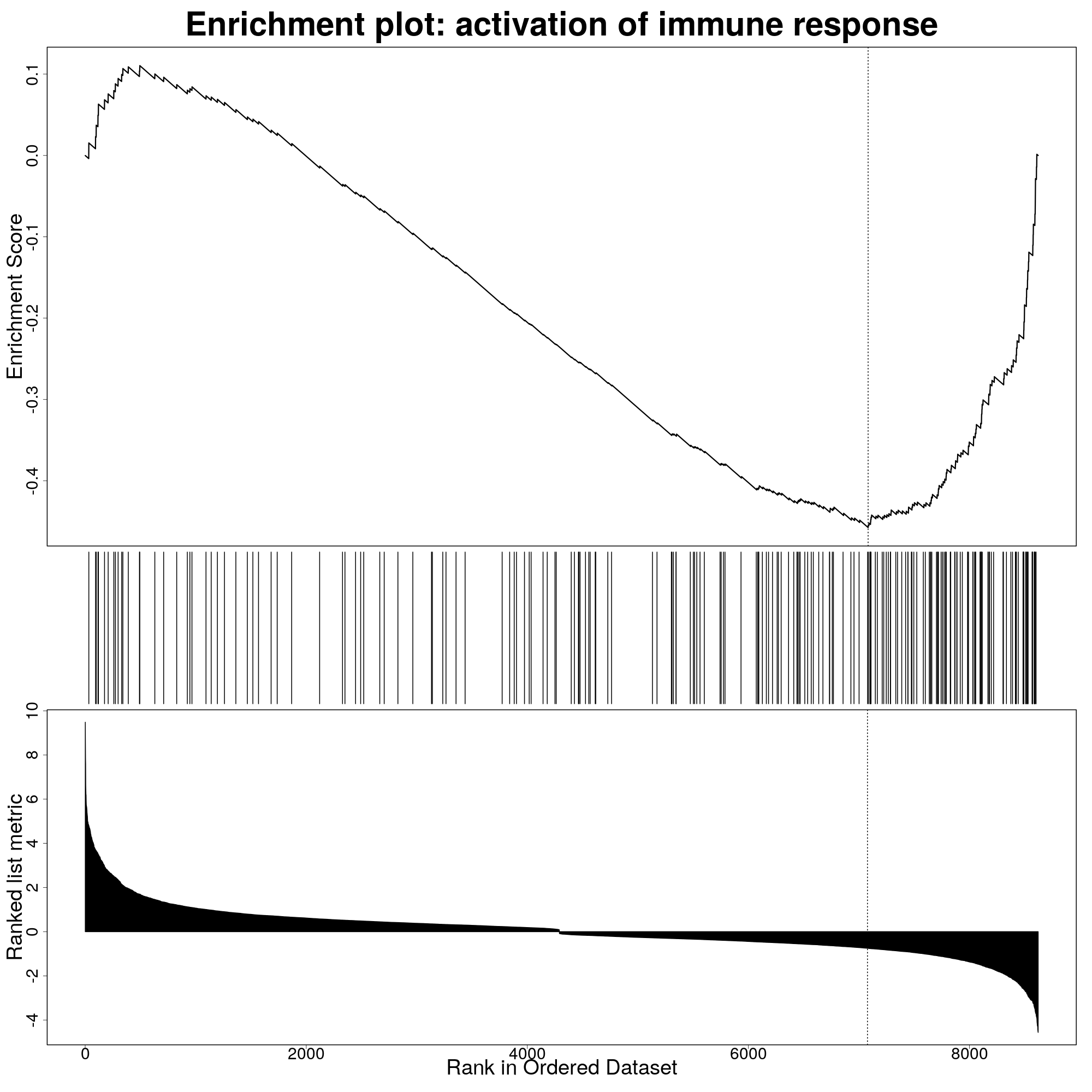

Supplement: Supplementary file 14 [file DataSheet_6.zip › Supplementary data 6 GSEA CCR2lo vs CCR2hi all samples/Project_high_vs_low_GSEA/GO_0002253.png]

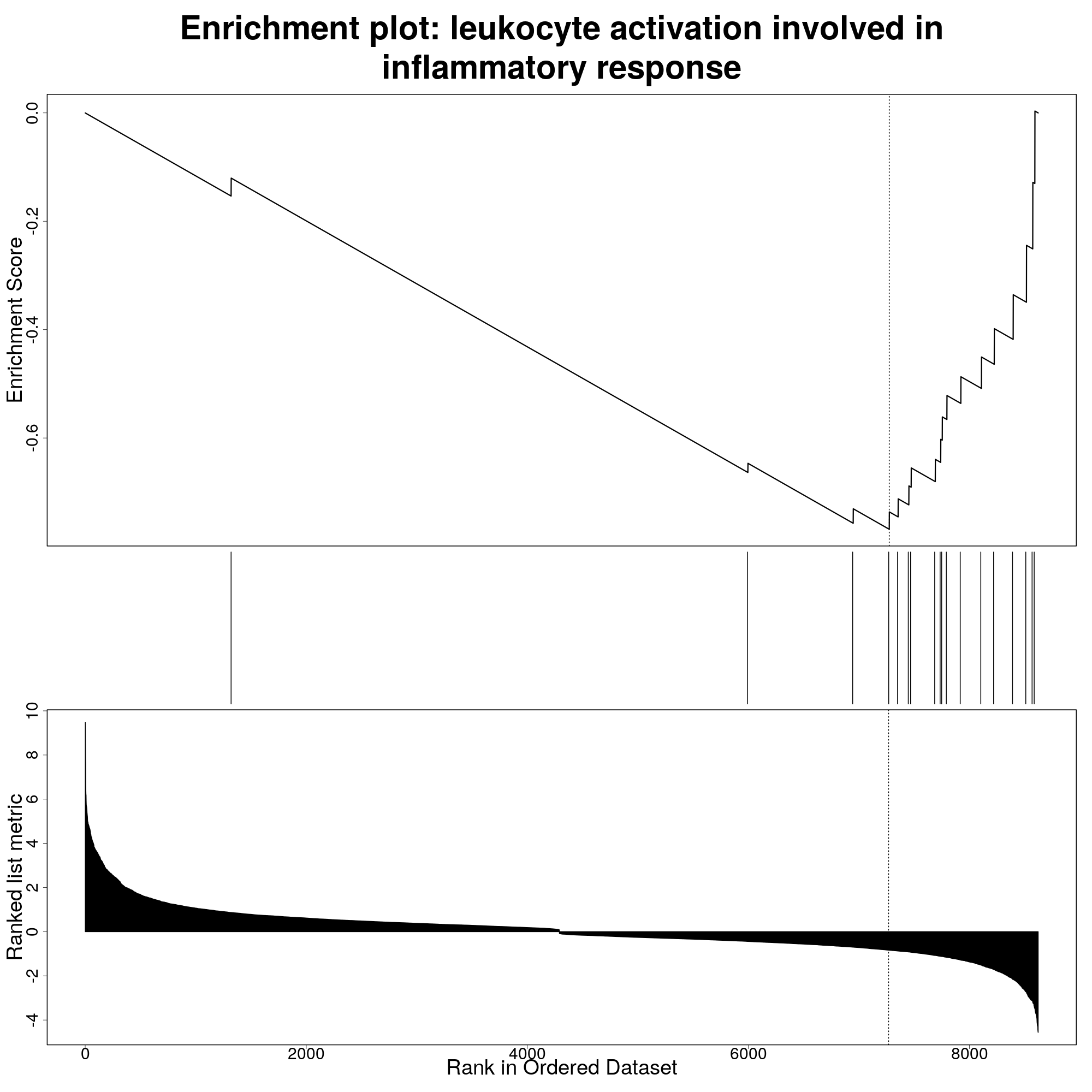

Supplement: Supplementary file 14 [file DataSheet_6.zip › Supplementary data 6 GSEA CCR2lo vs CCR2hi all samples/Project_high_vs_low_GSEA/GO_0002269.png]

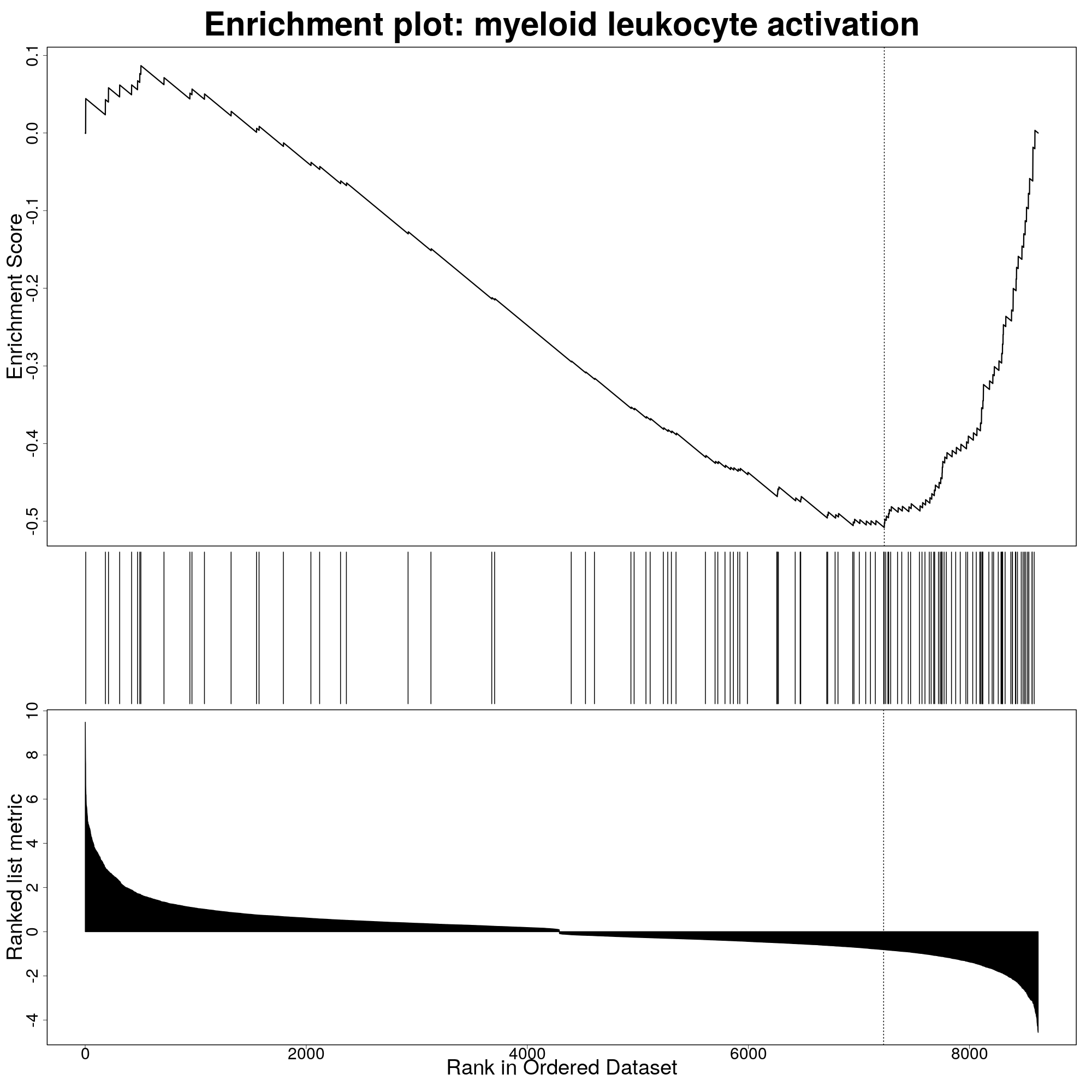

Supplement: Supplementary file 14 [file DataSheet_6.zip › Supplementary data 6 GSEA CCR2lo vs CCR2hi all samples/Project_high_vs_low_GSEA/GO_0002274.png]

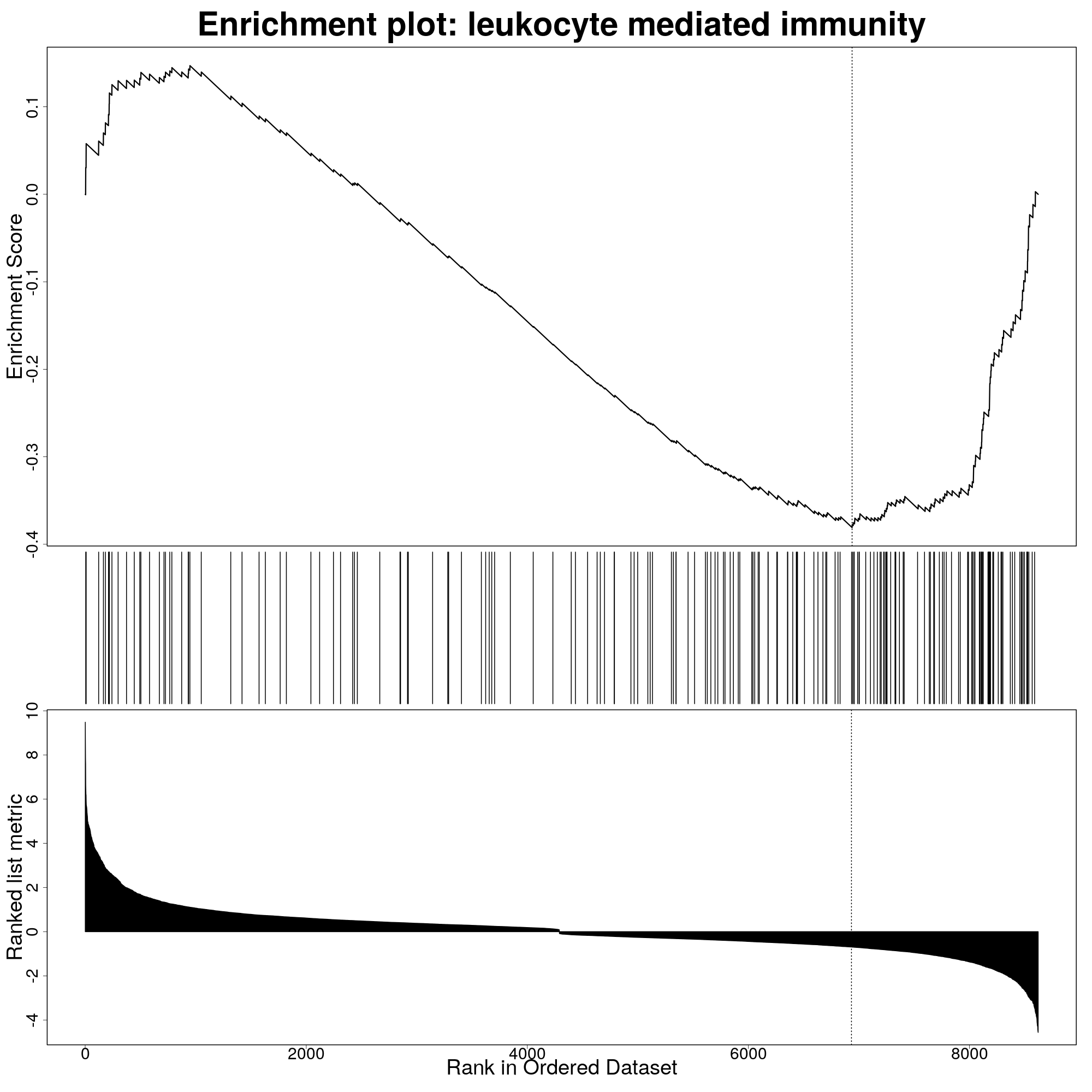

Supplement: Supplementary file 14 [file DataSheet_6.zip › Supplementary data 6 GSEA CCR2lo vs CCR2hi all samples/Project_high_vs_low_GSEA/GO_0002443.png]

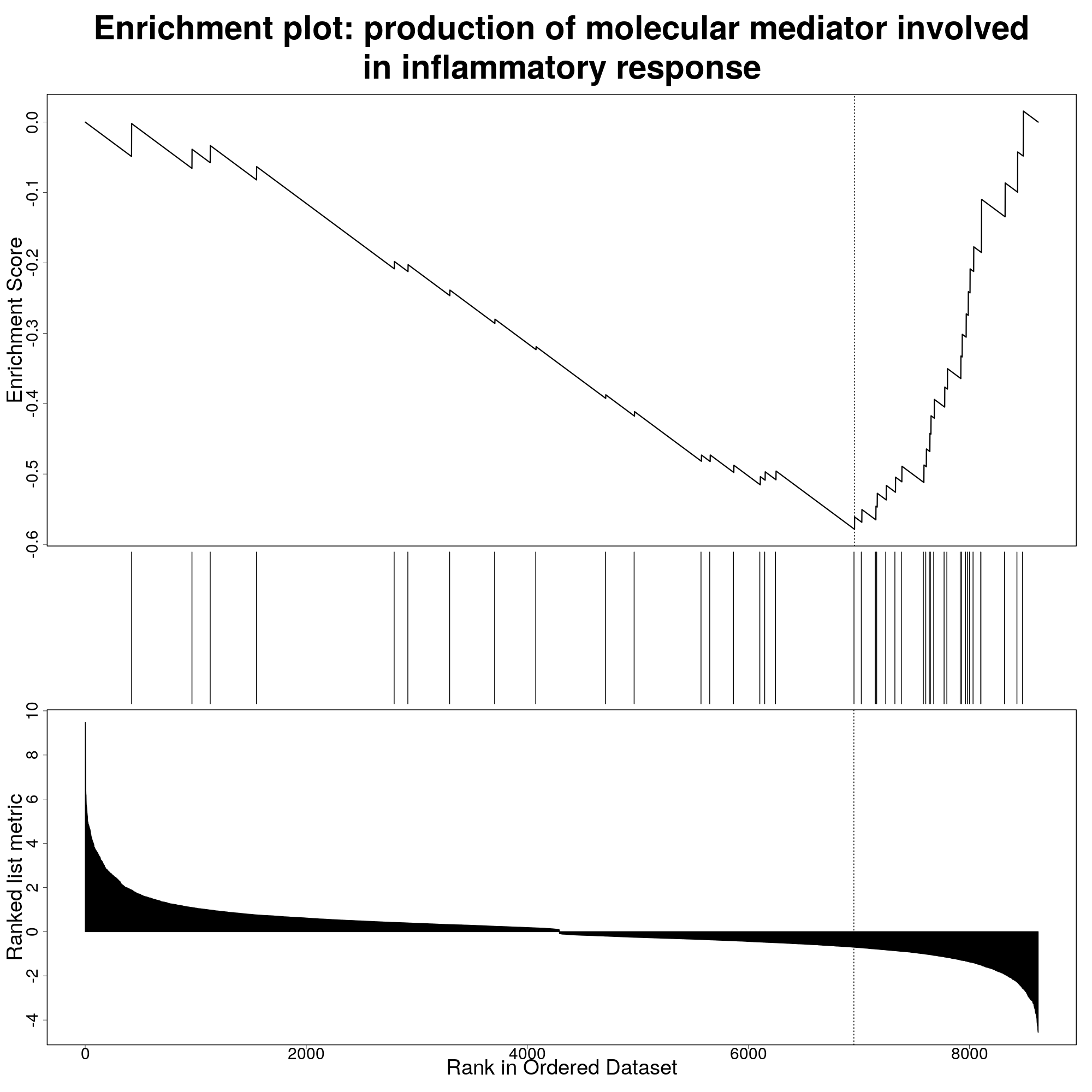

Supplement: Supplementary file 14 [file DataSheet_6.zip › Supplementary data 6 GSEA CCR2lo vs CCR2hi all samples/Project_high_vs_low_GSEA/GO_0002532.png]

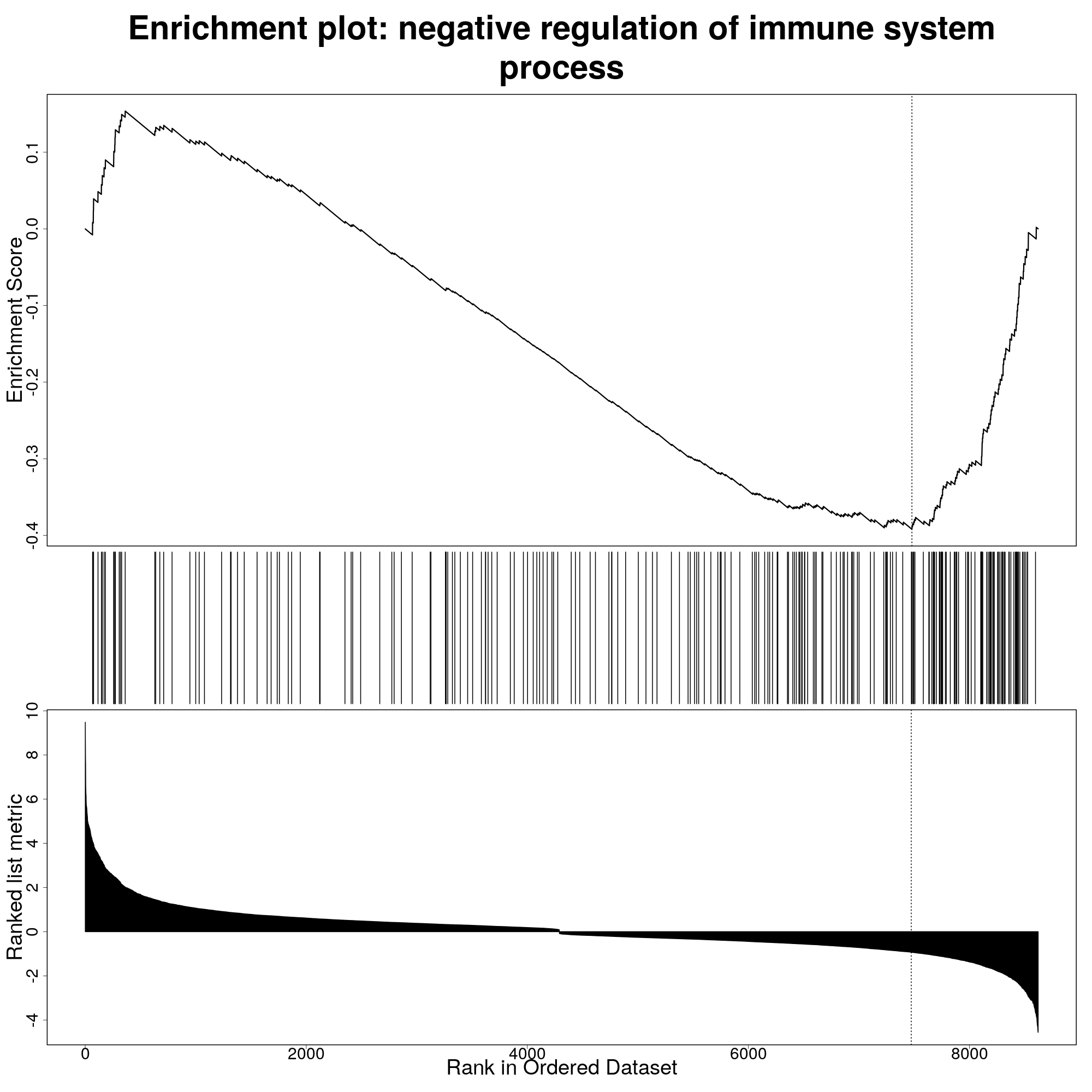

Supplement: Supplementary file 14 [file DataSheet_6.zip › Supplementary data 6 GSEA CCR2lo vs CCR2hi all samples/Project_high_vs_low_GSEA/GO_0002683.png]

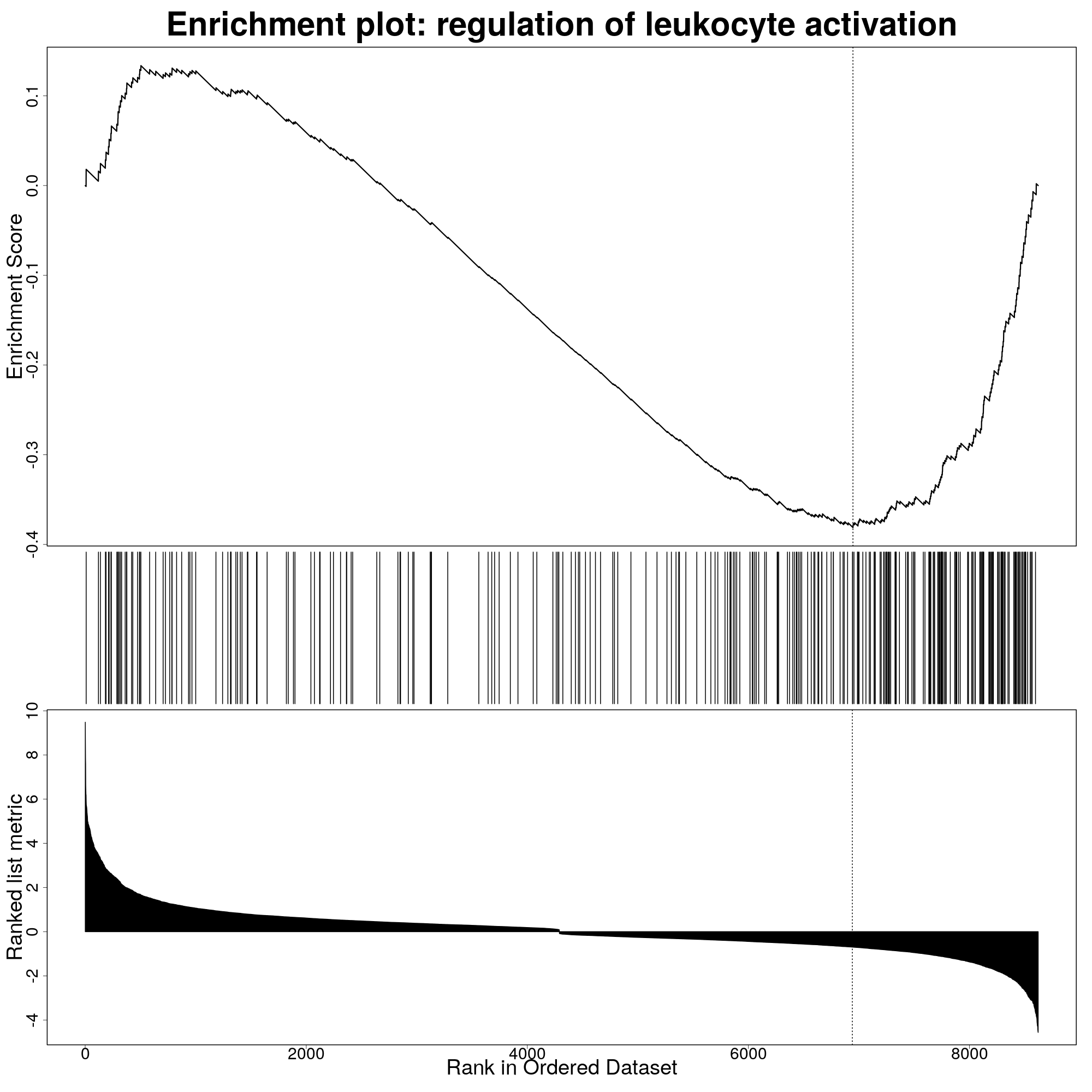

Supplement: Supplementary file 14 [file DataSheet_6.zip › Supplementary data 6 GSEA CCR2lo vs CCR2hi all samples/Project_high_vs_low_GSEA/GO_0002694.png]

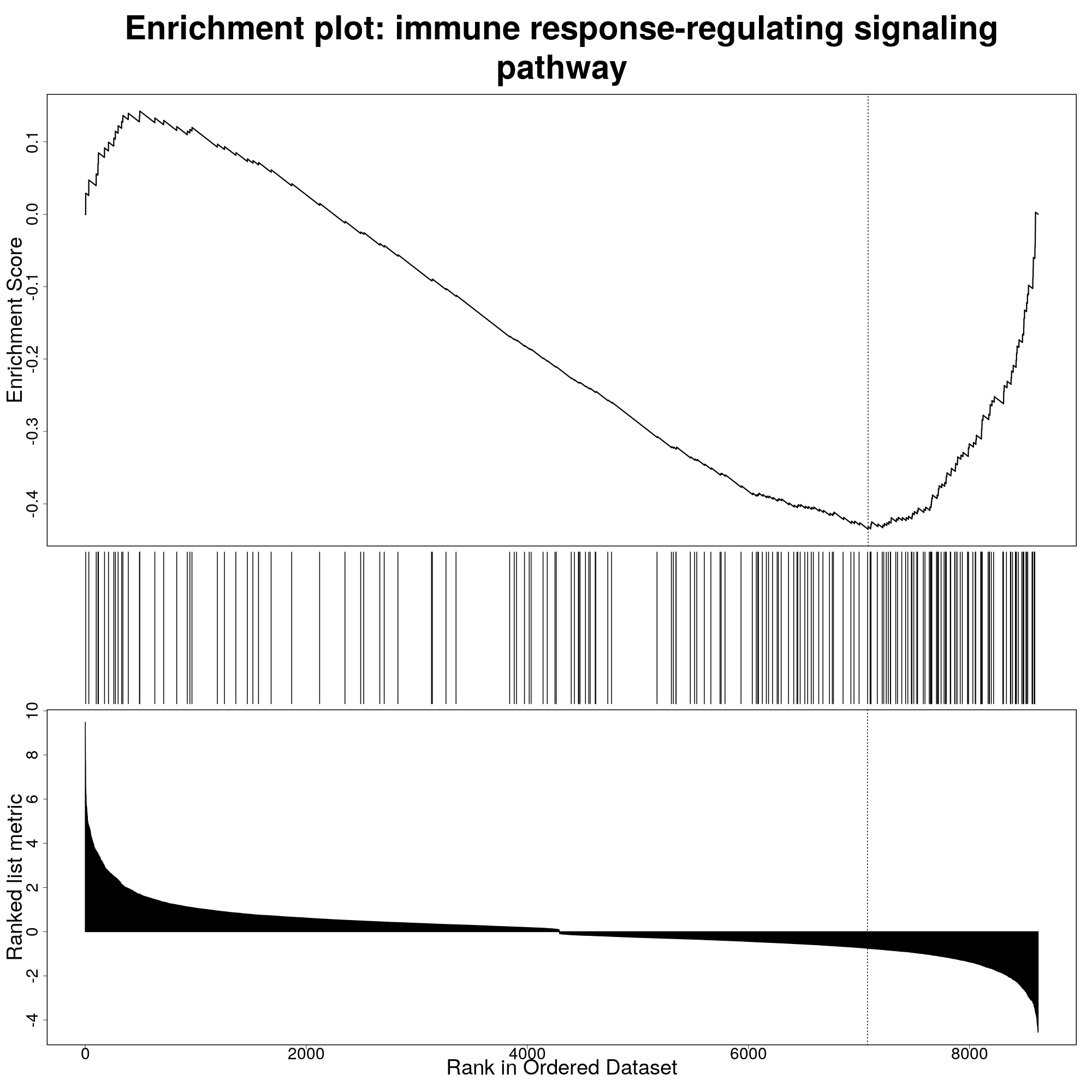

Supplement: Supplementary file 14 [file DataSheet_6.zip › Supplementary data 6 GSEA CCR2lo vs CCR2hi all samples/Project_high_vs_low_GSEA/GO_0002764.png]

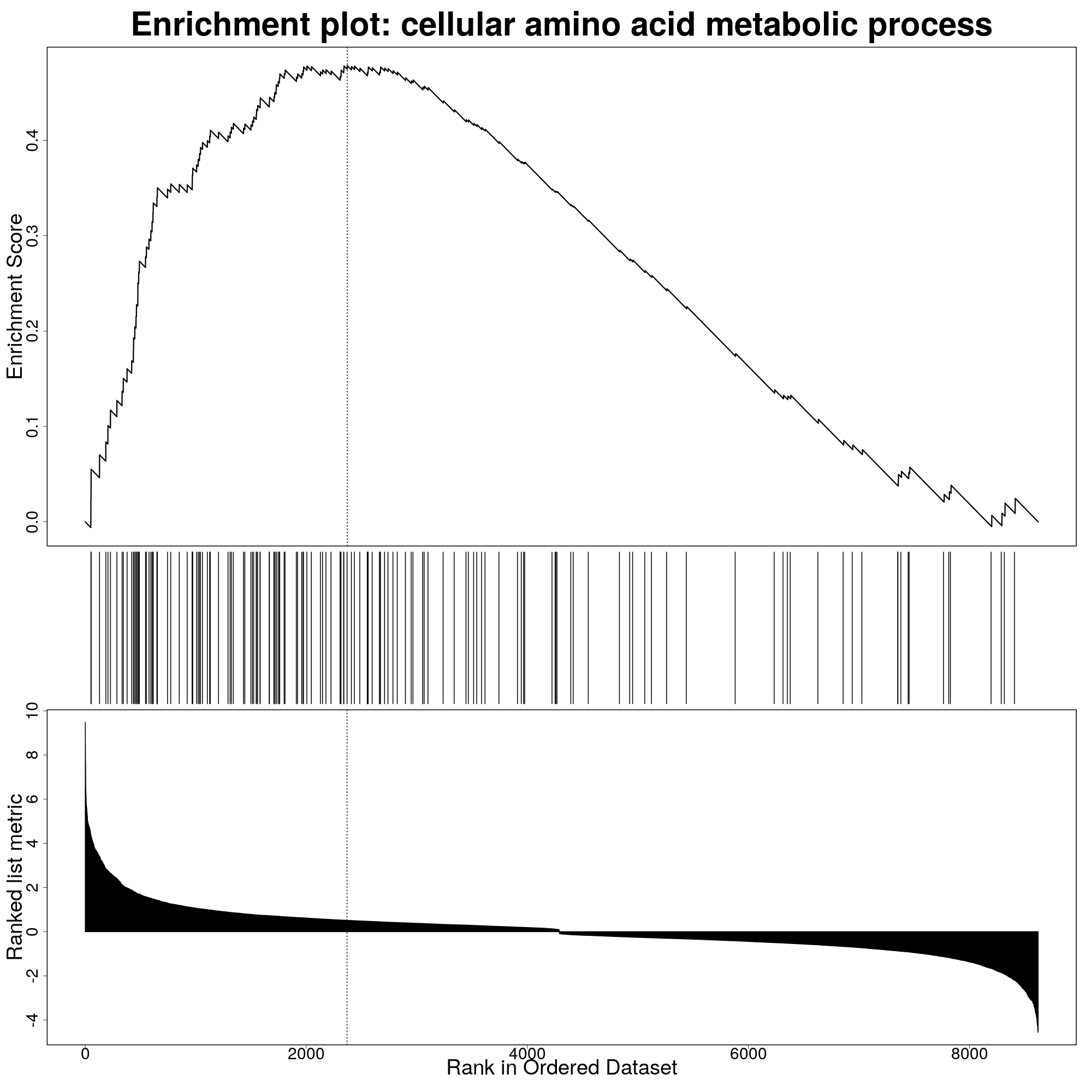

Supplement: Supplementary file 14 [file DataSheet_6.zip › Supplementary data 6 GSEA CCR2lo vs CCR2hi all samples/Project_high_vs_low_GSEA/GO_0006520.png]

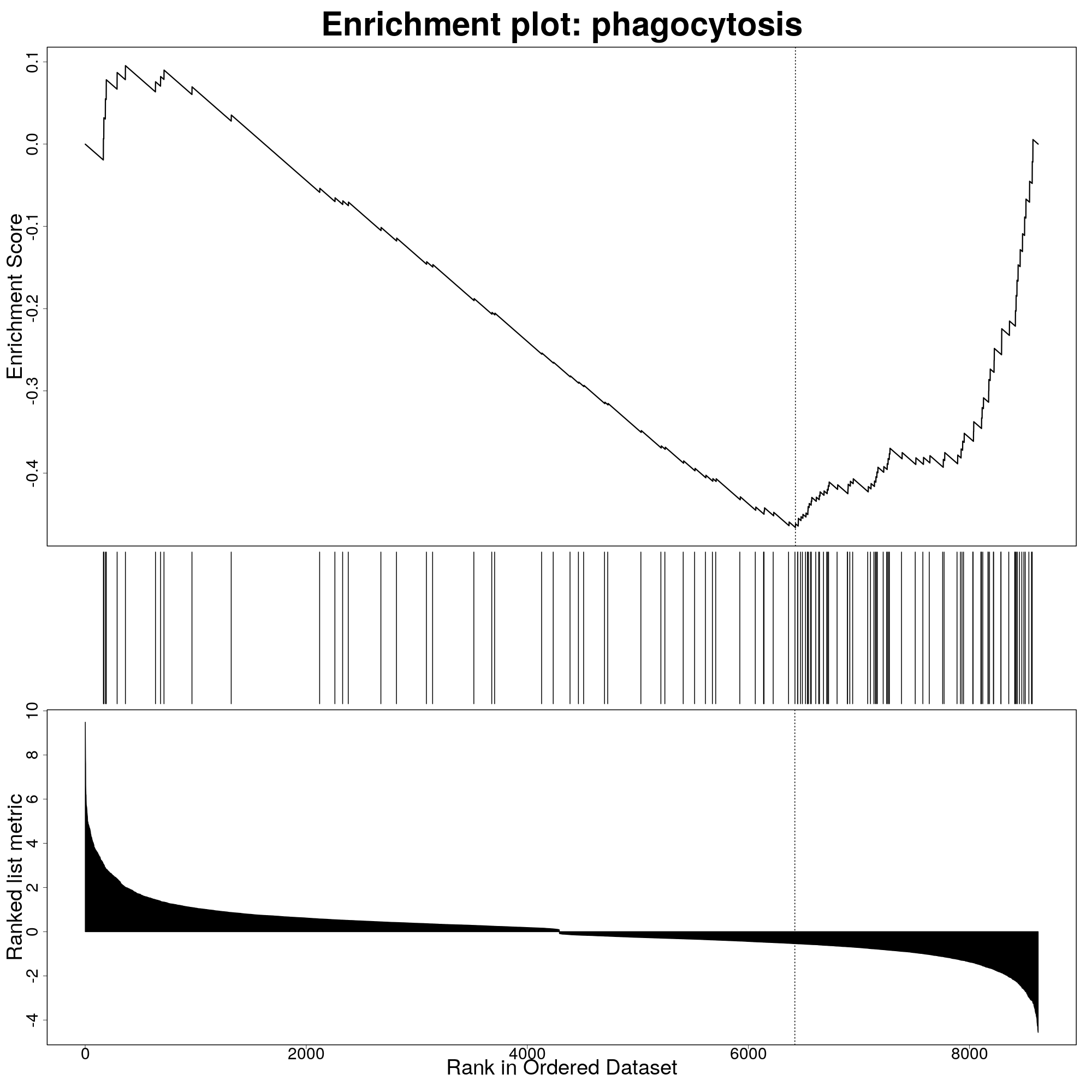

Supplement: Supplementary file 14 [file DataSheet_6.zip › Supplementary data 6 GSEA CCR2lo vs CCR2hi all samples/Project_high_vs_low_GSEA/GO_0006909.png]

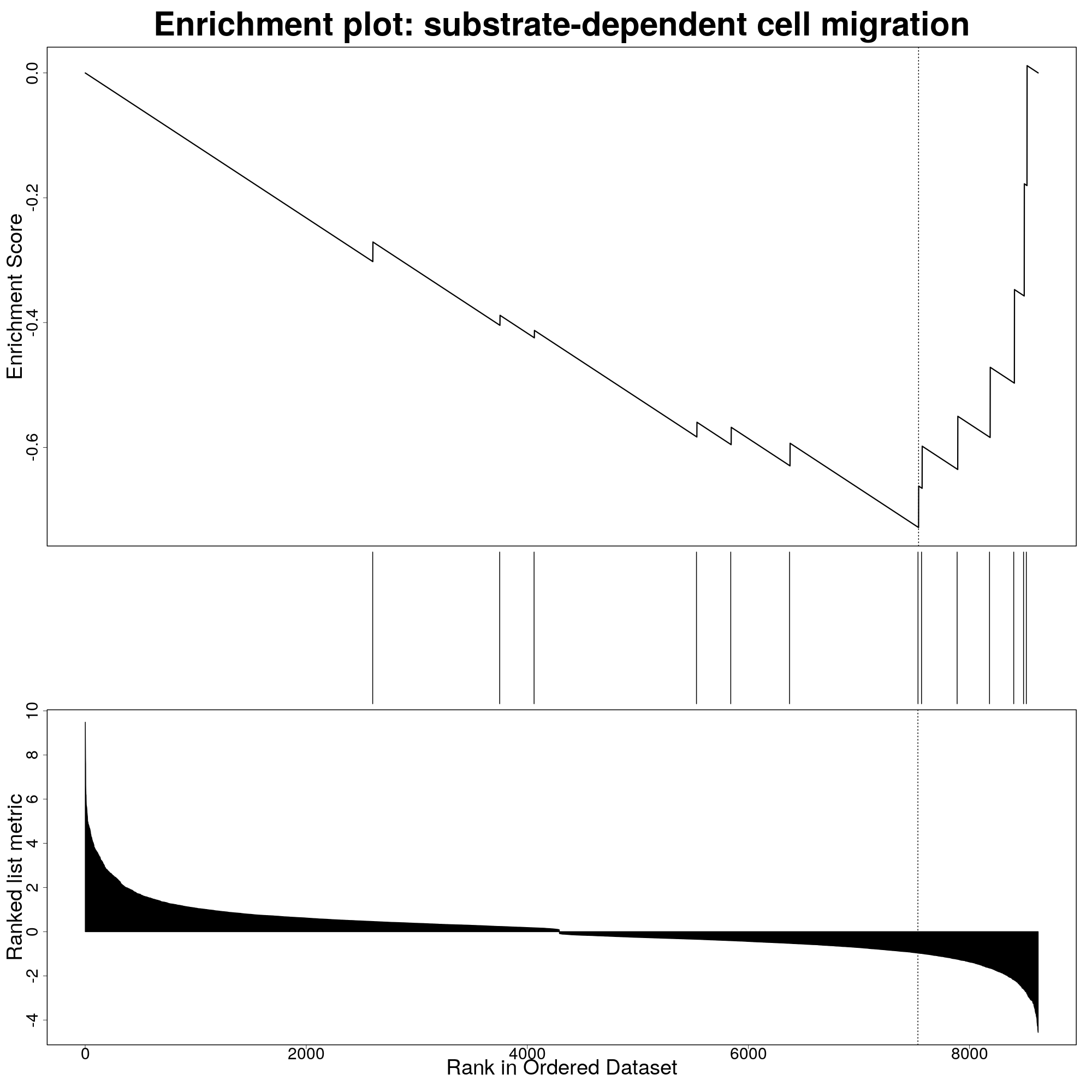

Supplement: Supplementary file 14 [file DataSheet_6.zip › Supplementary data 6 GSEA CCR2lo vs CCR2hi all samples/Project_high_vs_low_GSEA/GO_0006929.png]

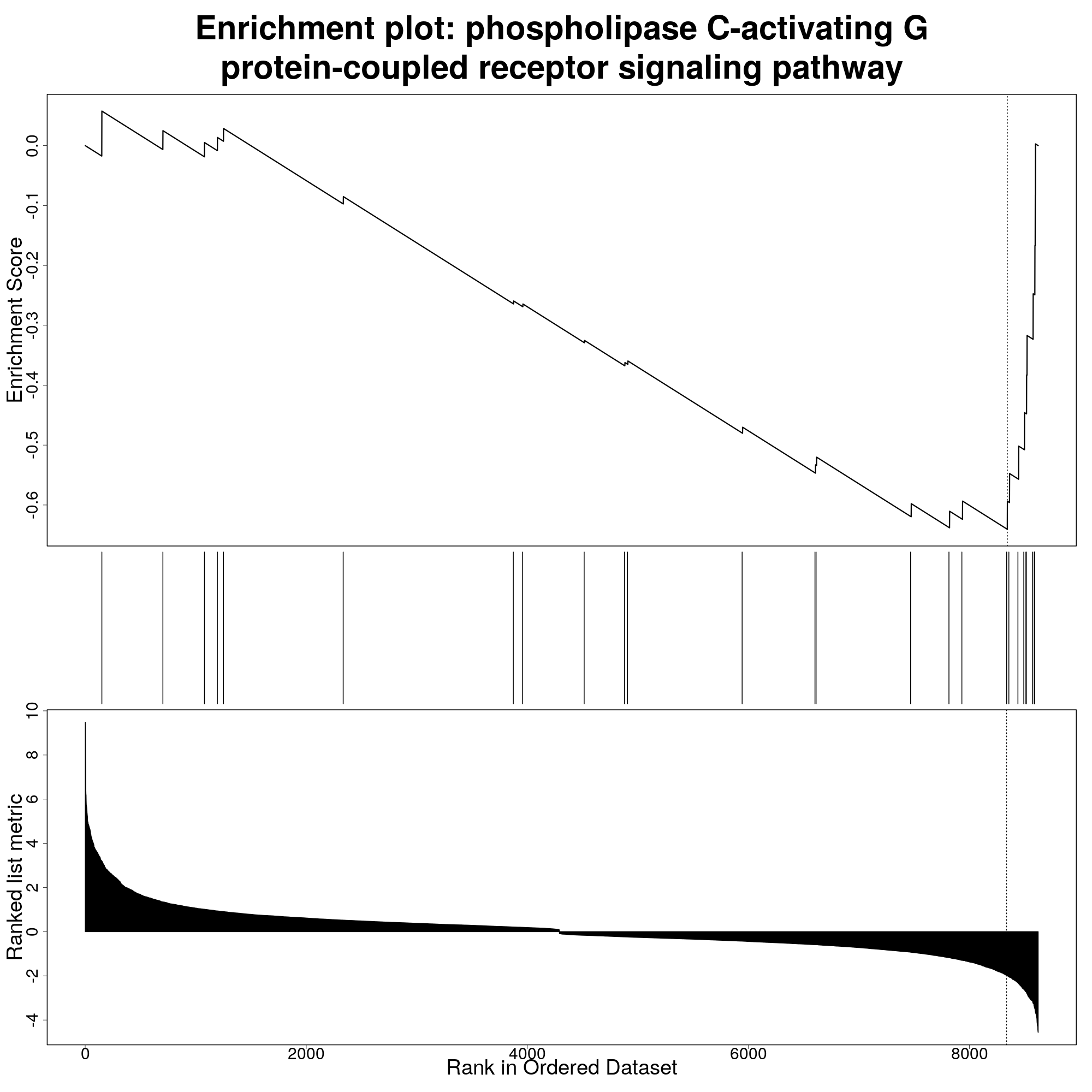

Supplement: Supplementary file 14 [file DataSheet_6.zip › Supplementary data 6 GSEA CCR2lo vs CCR2hi all samples/Project_high_vs_low_GSEA/GO_0007200.png]

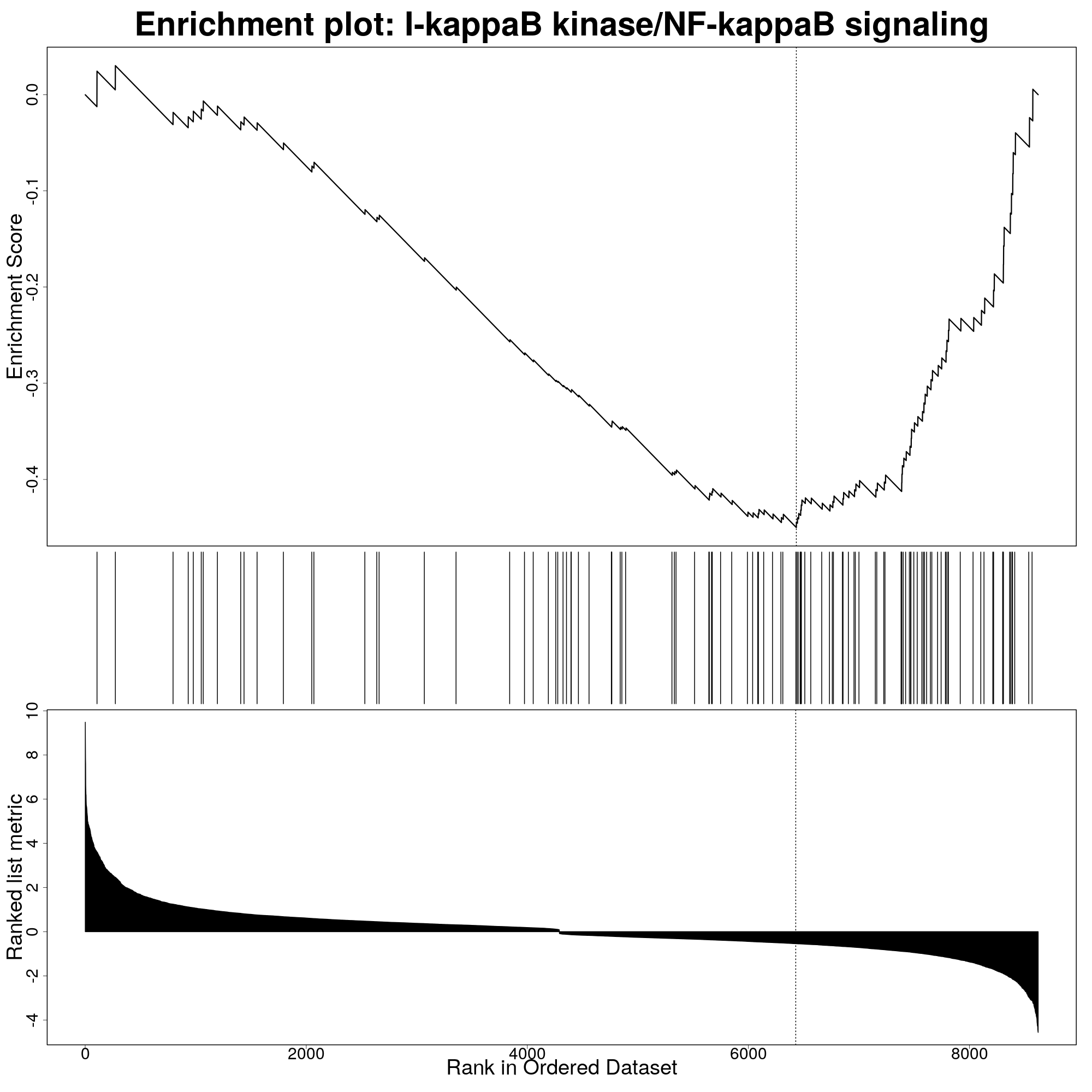

Supplement: Supplementary file 14 [file DataSheet_6.zip › Supplementary data 6 GSEA CCR2lo vs CCR2hi all samples/Project_high_vs_low_GSEA/GO_0007249.png]

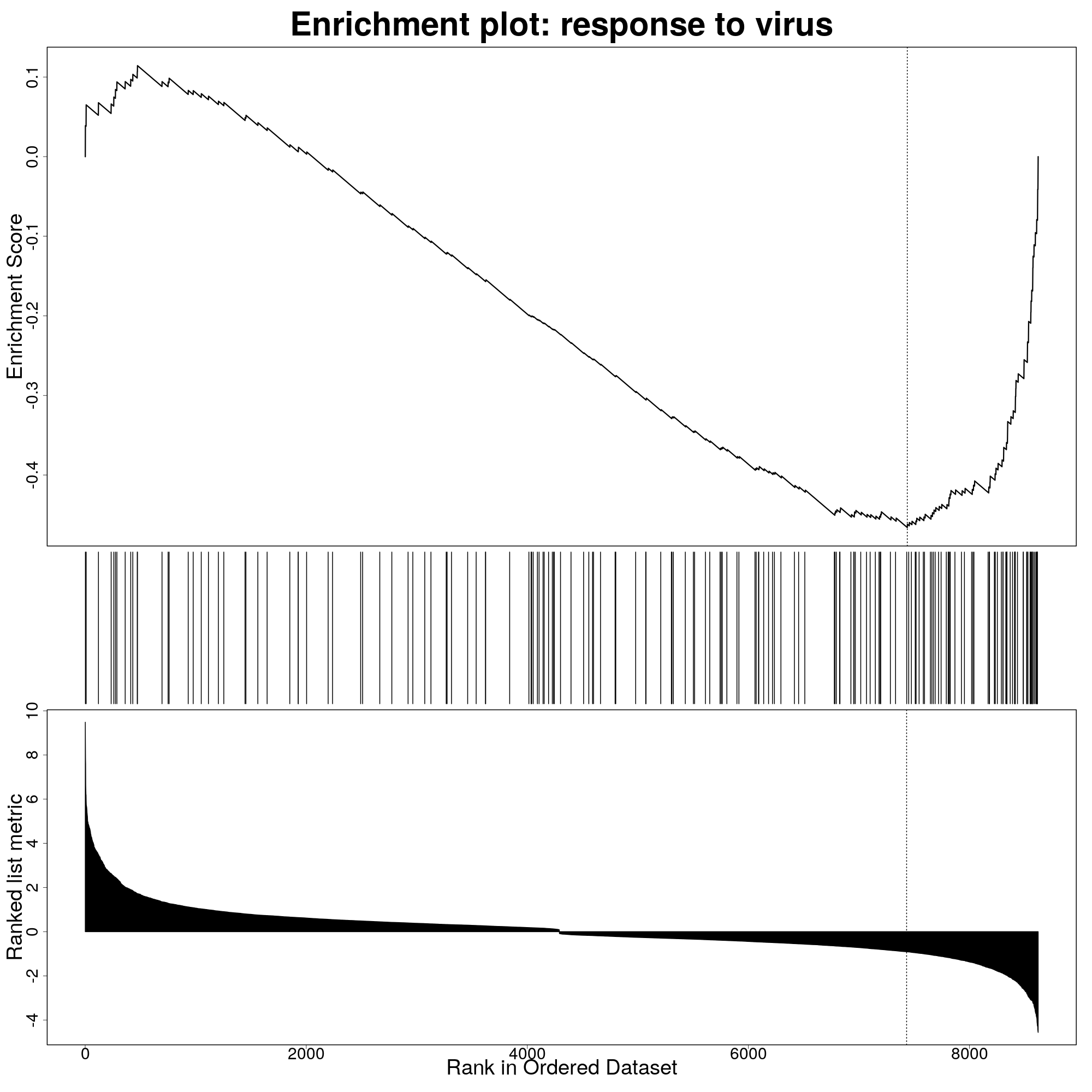

Supplement: Supplementary file 14 [file DataSheet_6.zip › Supplementary data 6 GSEA CCR2lo vs CCR2hi all samples/Project_high_vs_low_GSEA/GO_0009615.png]

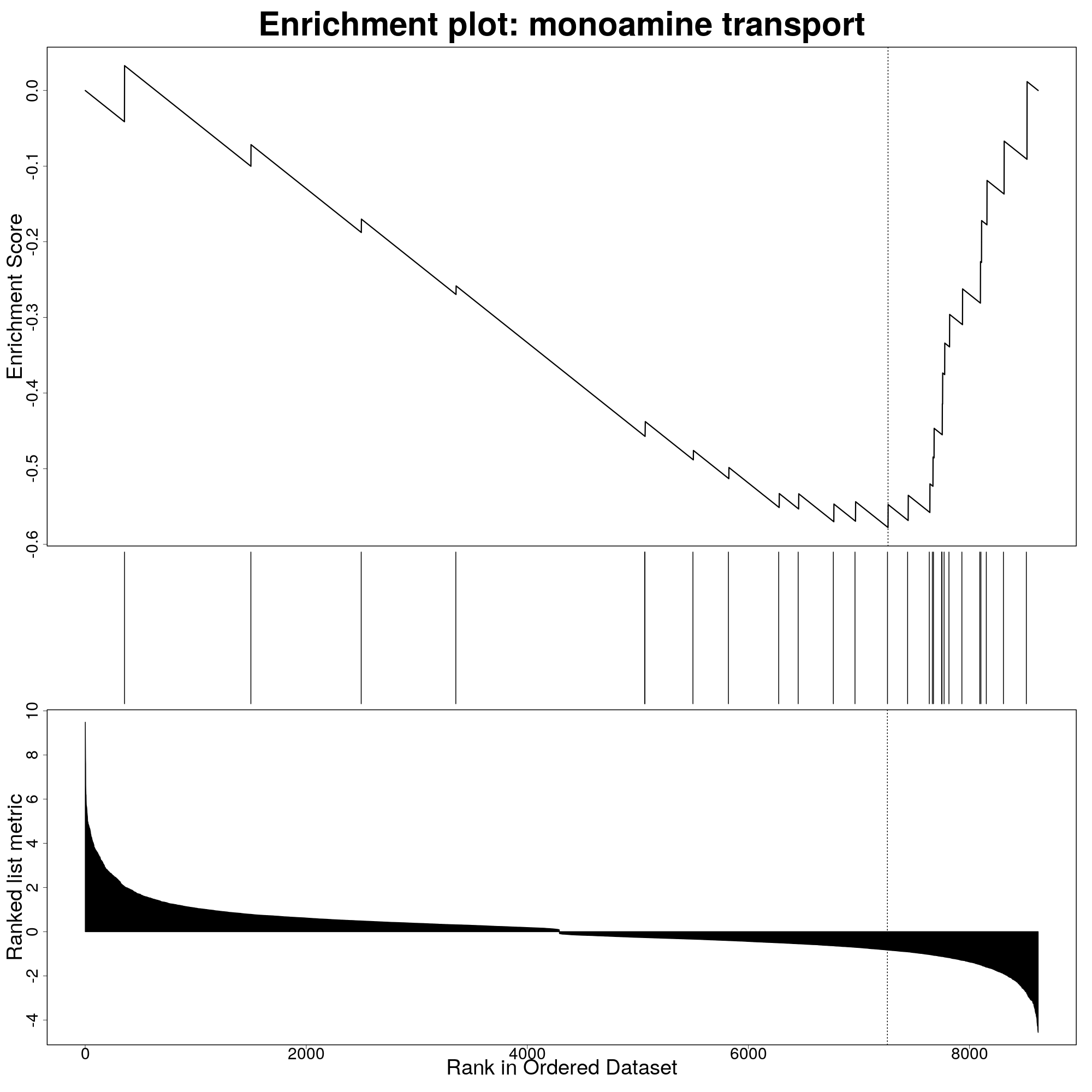

Supplement: Supplementary file 14 [file DataSheet_6.zip › Supplementary data 6 GSEA CCR2lo vs CCR2hi all samples/Project_high_vs_low_GSEA/GO_0015844.png]

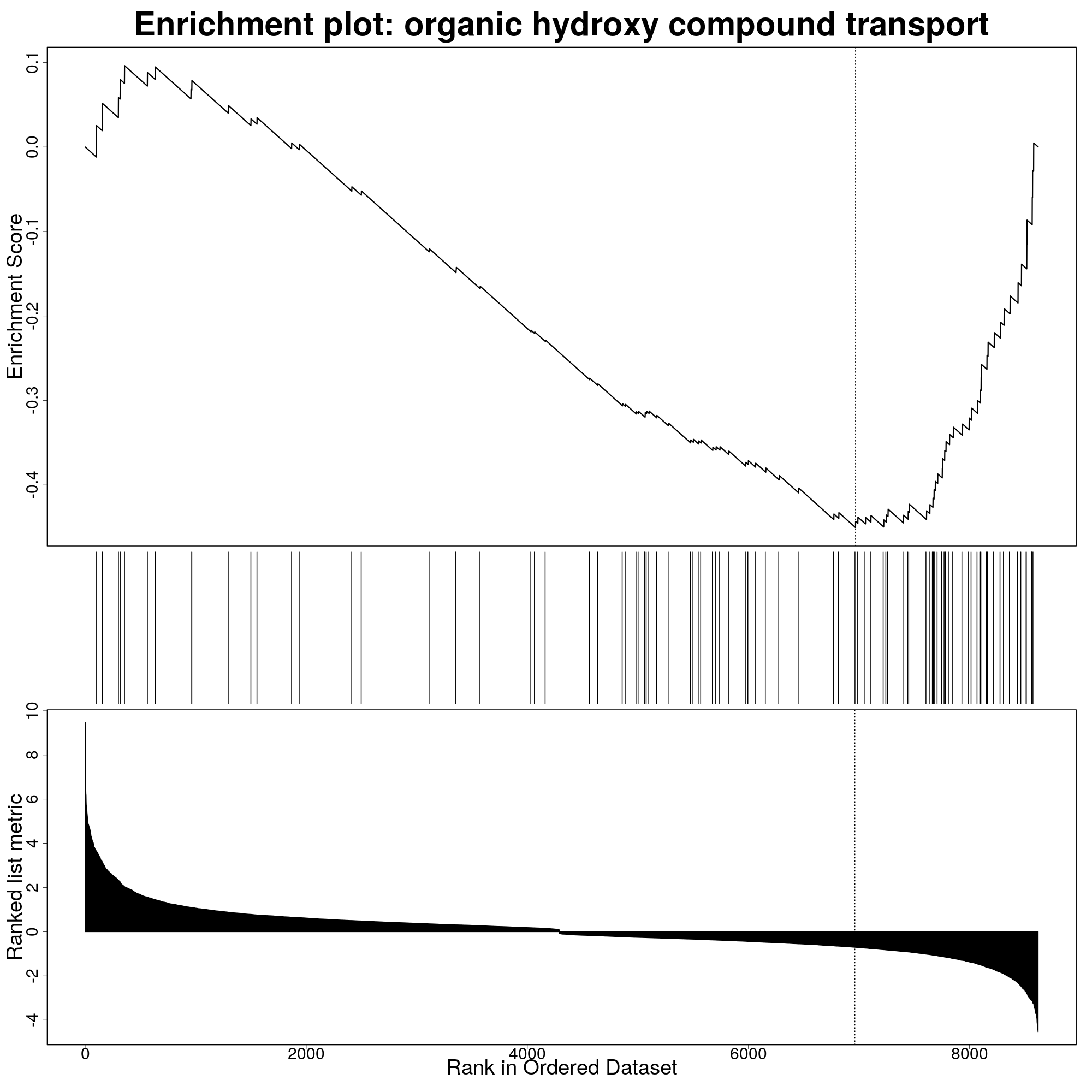

Supplement: Supplementary file 14 [file DataSheet_6.zip › Supplementary data 6 GSEA CCR2lo vs CCR2hi all samples/Project_high_vs_low_GSEA/GO_0015850.png]

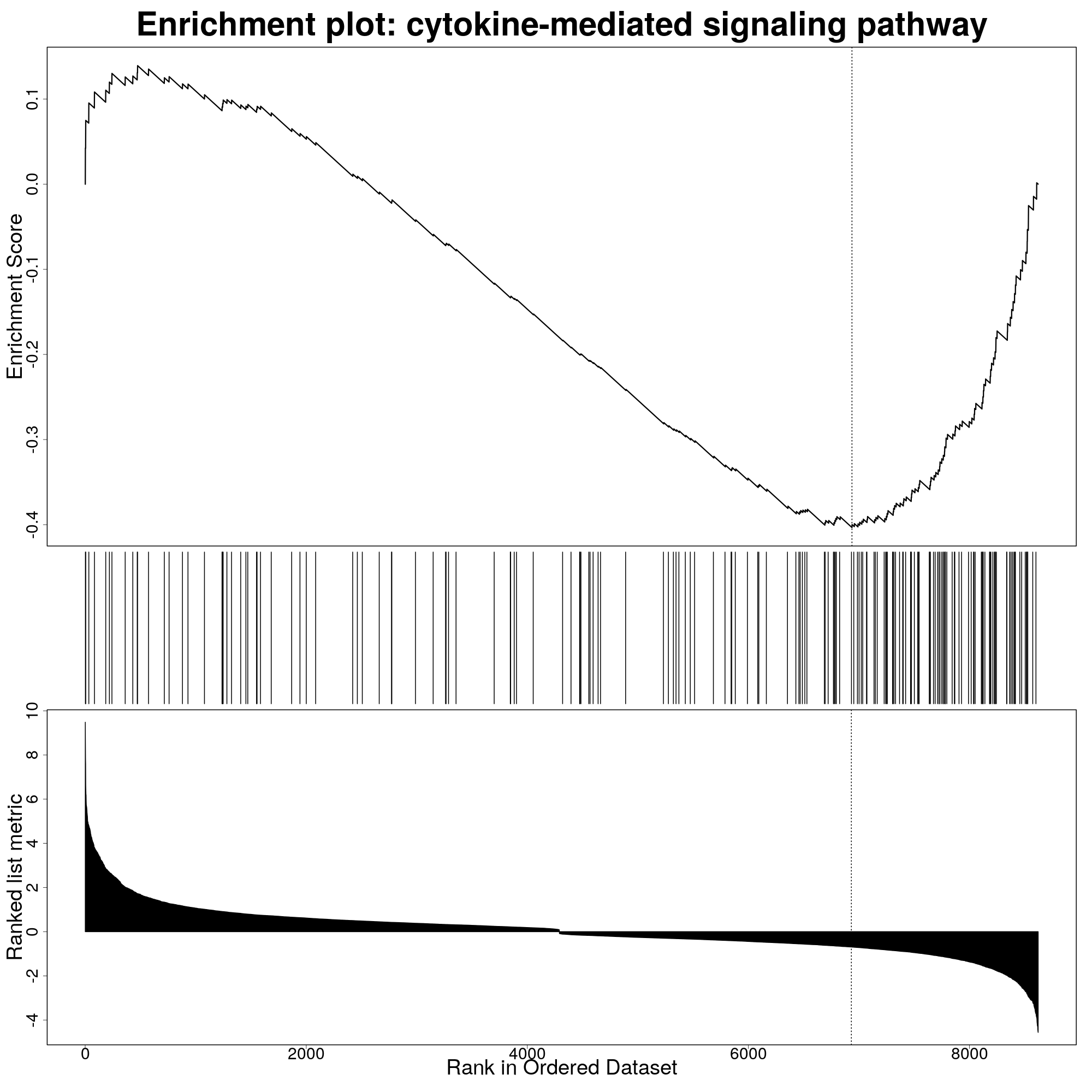

Supplement: Supplementary file 14 [file DataSheet_6.zip › Supplementary data 6 GSEA CCR2lo vs CCR2hi all samples/Project_high_vs_low_GSEA/GO_0019221.png]

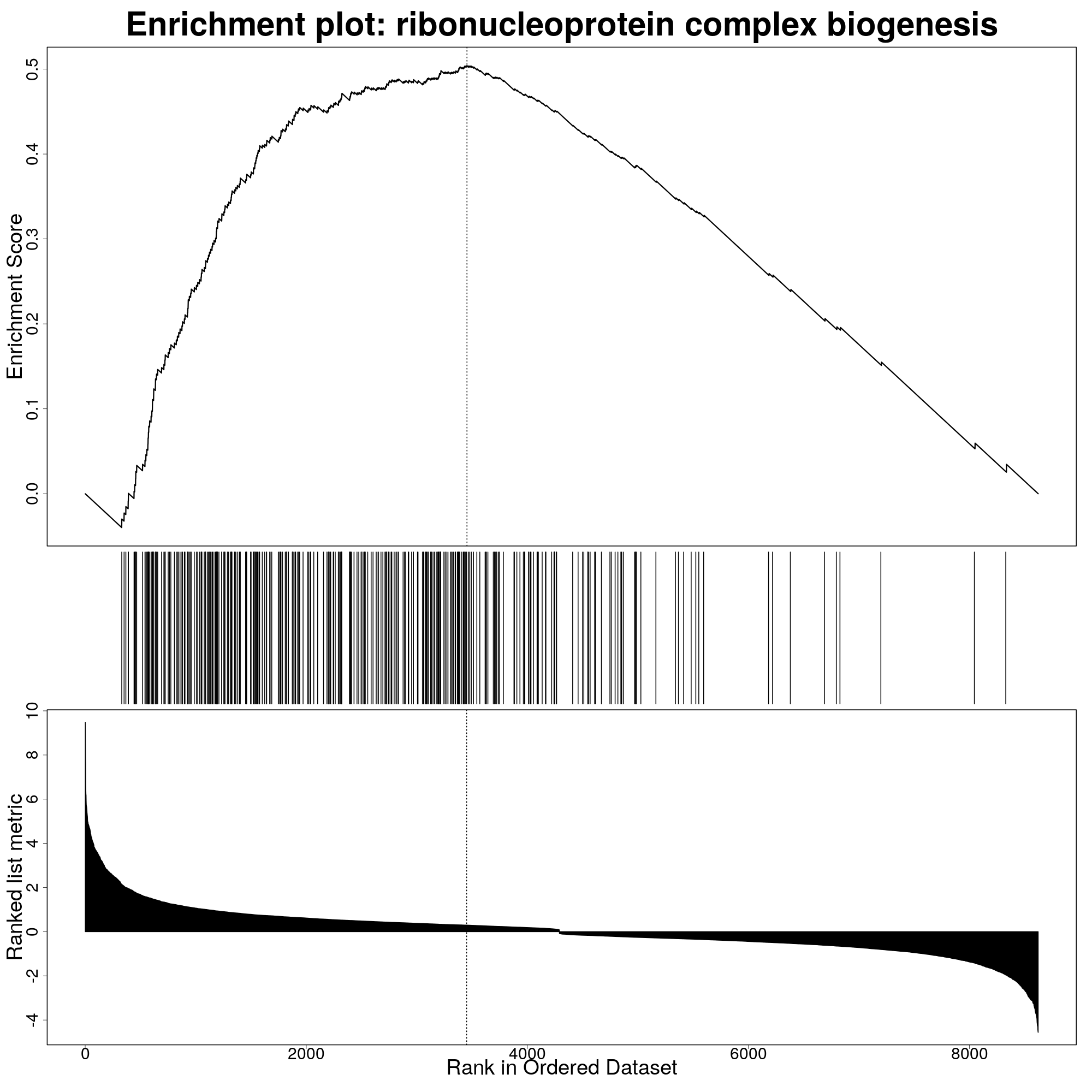

Supplement: Supplementary file 14 [file DataSheet_6.zip › Supplementary data 6 GSEA CCR2lo vs CCR2hi all samples/Project_high_vs_low_GSEA/GO_0022613.png]

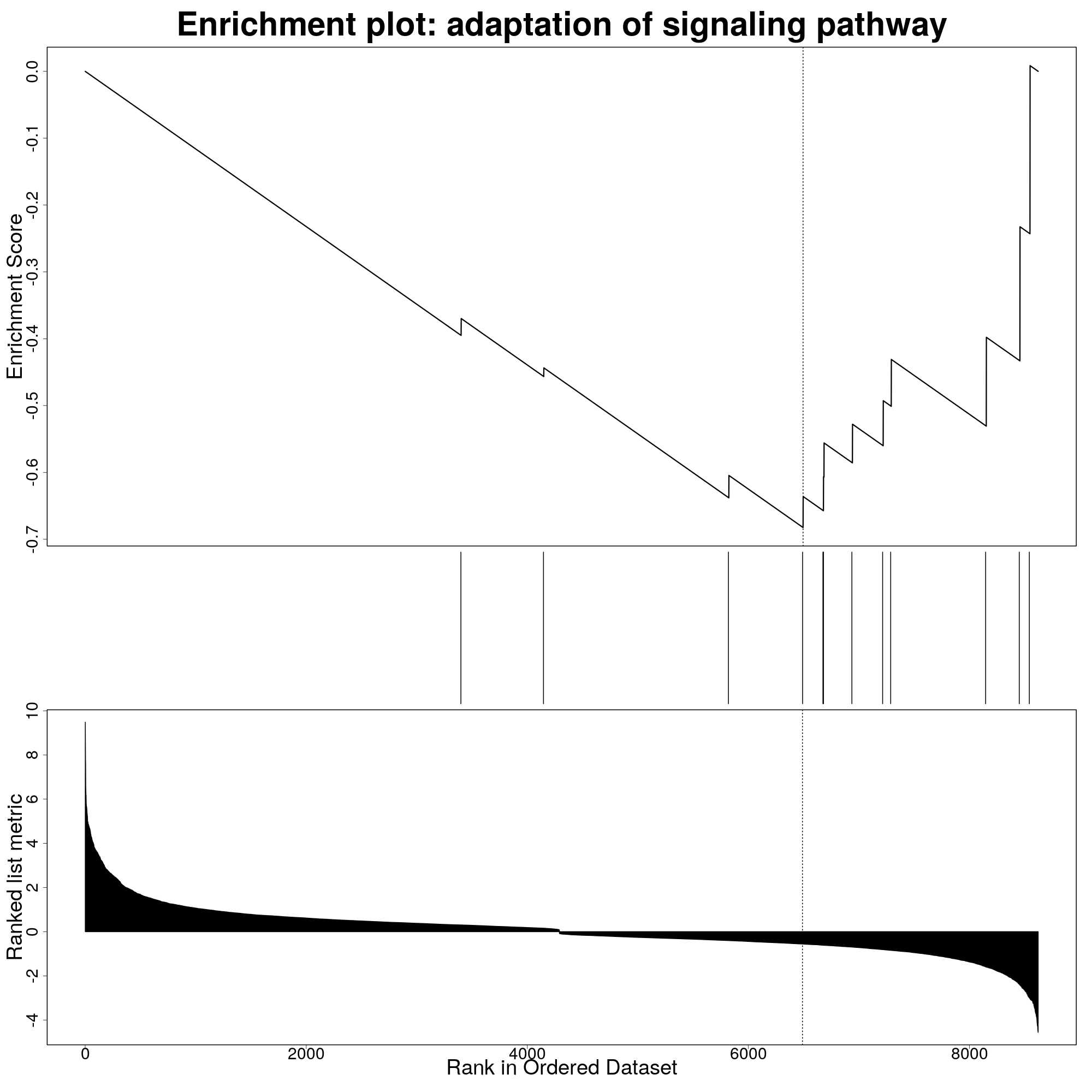

Supplement: Supplementary file 14 [file DataSheet_6.zip › Supplementary data 6 GSEA CCR2lo vs CCR2hi all samples/Project_high_vs_low_GSEA/GO_0023058.png]

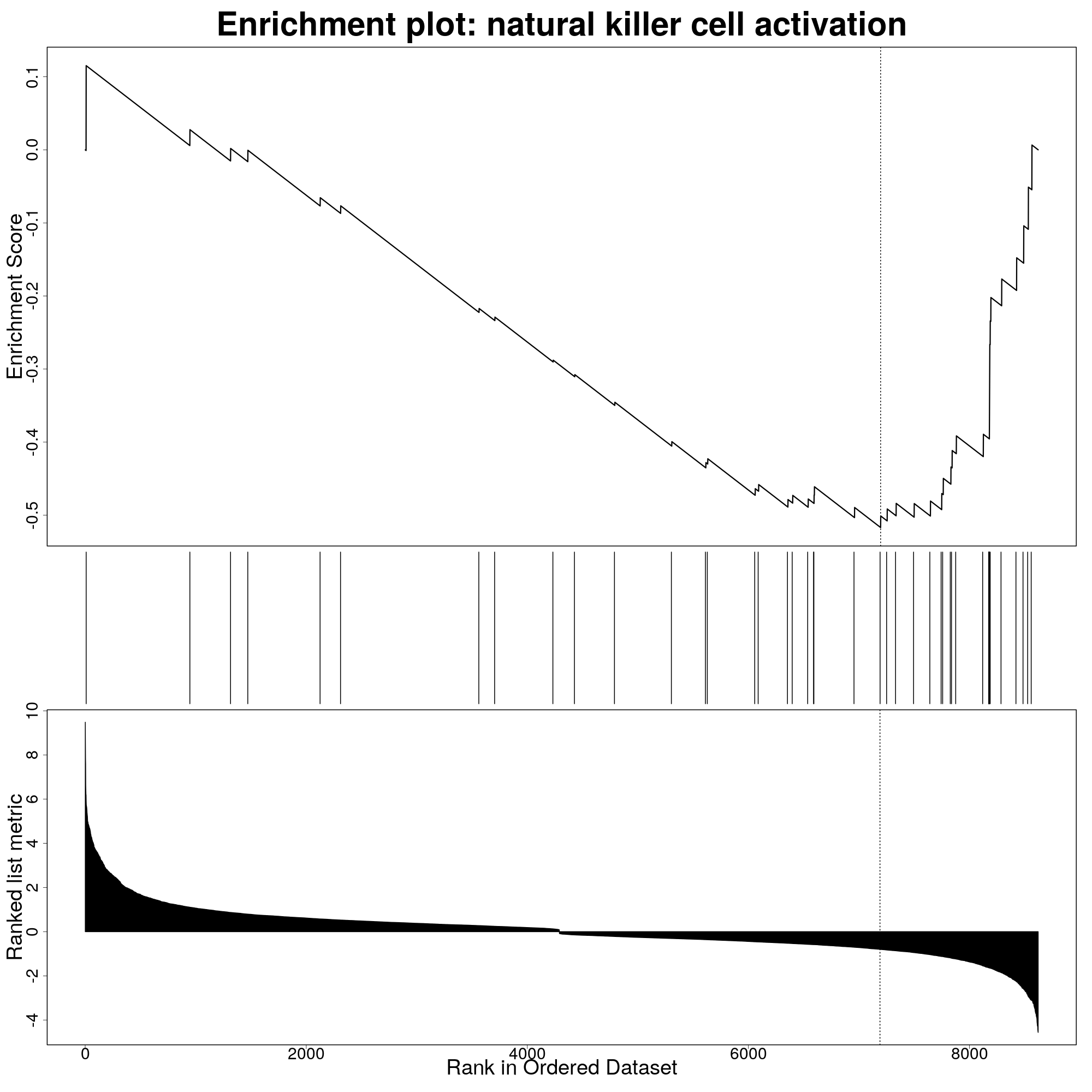

Supplement: Supplementary file 14 [file DataSheet_6.zip › Supplementary data 6 GSEA CCR2lo vs CCR2hi all samples/Project_high_vs_low_GSEA/GO_0030101.png]

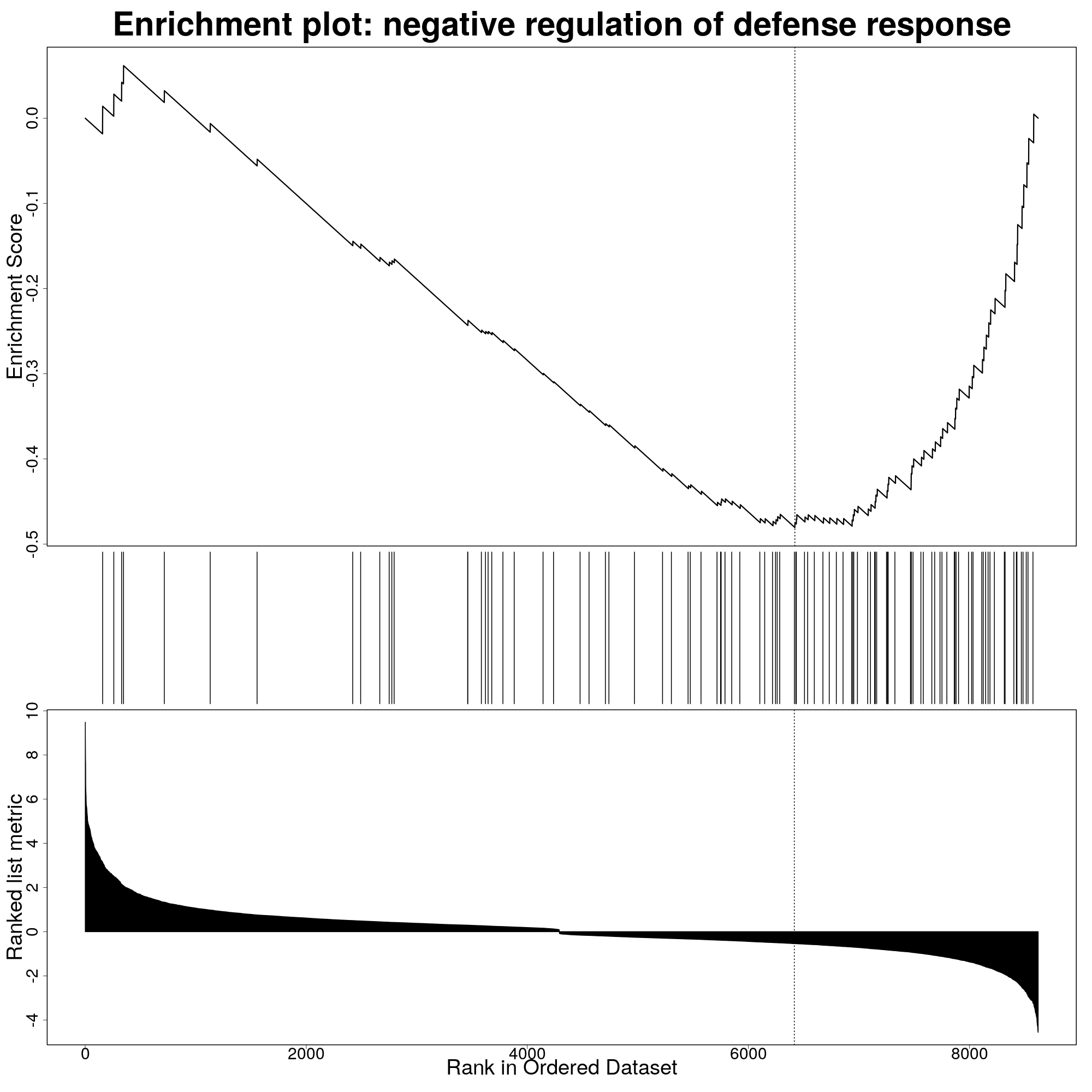

Supplement: Supplementary file 14 [file DataSheet_6.zip › Supplementary data 6 GSEA CCR2lo vs CCR2hi all samples/Project_high_vs_low_GSEA/GO_0031348.png]

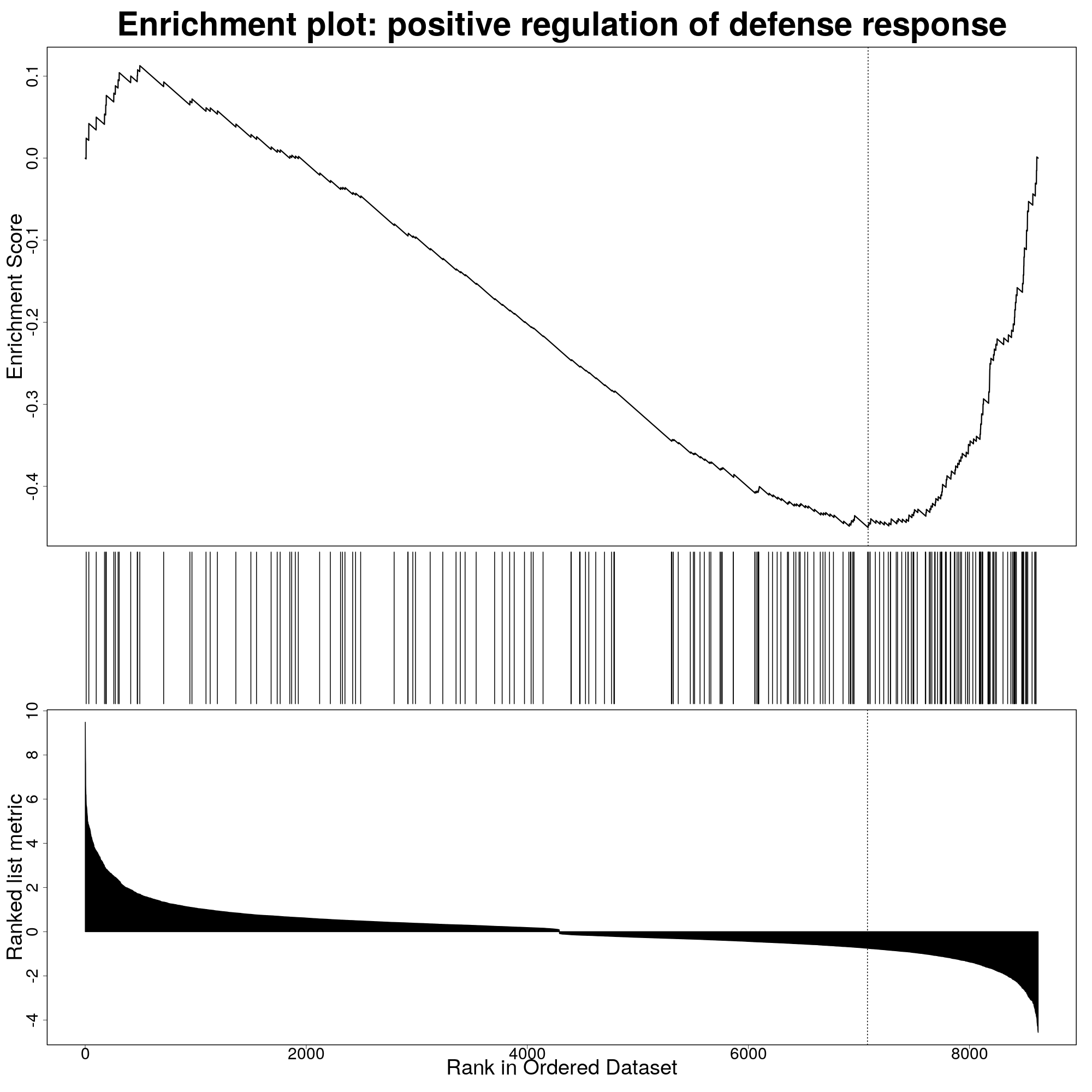

Supplement: Supplementary file 14 [file DataSheet_6.zip › Supplementary data 6 GSEA CCR2lo vs CCR2hi all samples/Project_high_vs_low_GSEA/GO_0031349.png]

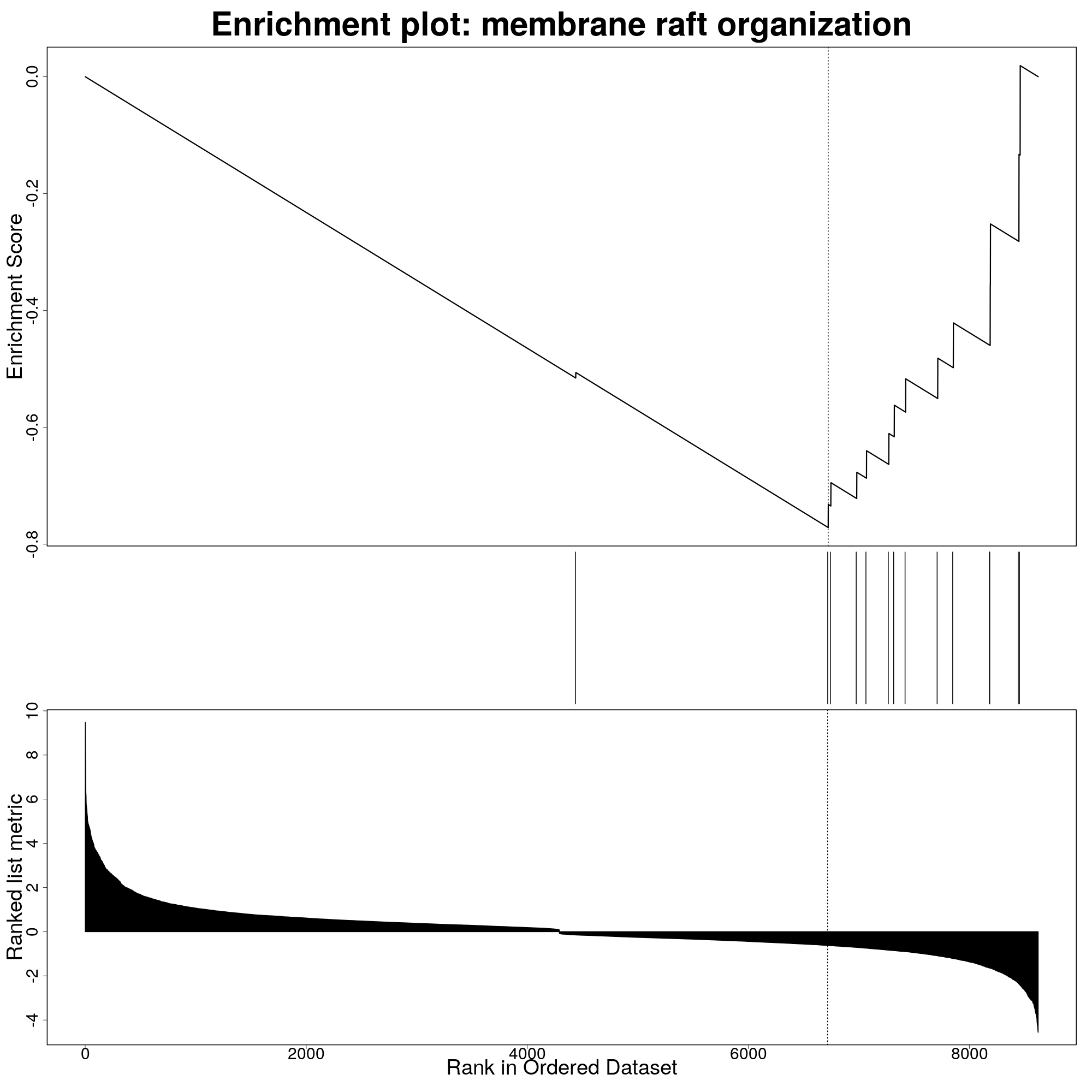

Supplement: Supplementary file 14 [file DataSheet_6.zip › Supplementary data 6 GSEA CCR2lo vs CCR2hi all samples/Project_high_vs_low_GSEA/GO_0031579.png]

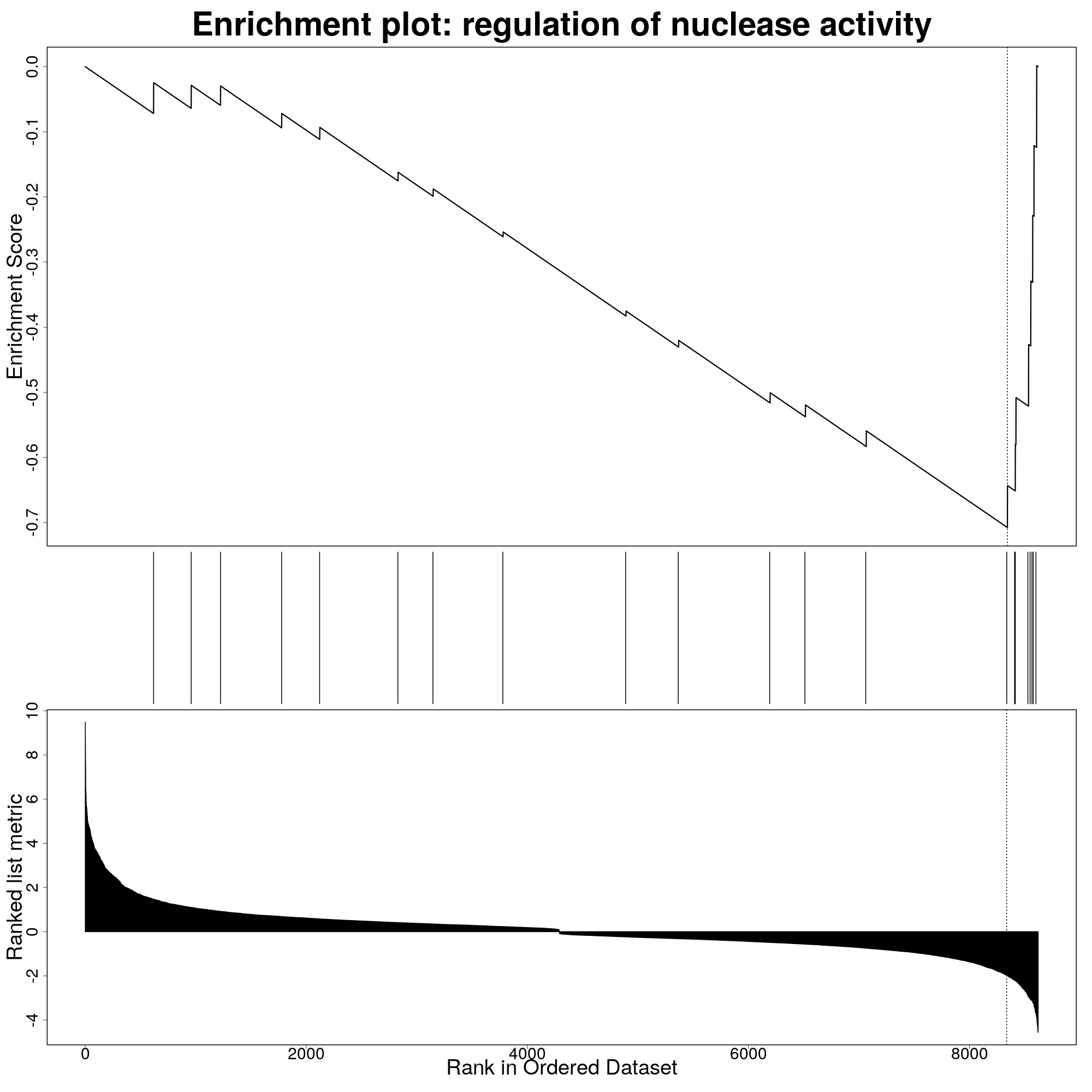

Supplement: Supplementary file 14 [file DataSheet_6.zip › Supplementary data 6 GSEA CCR2lo vs CCR2hi all samples/Project_high_vs_low_GSEA/GO_0032069.png]

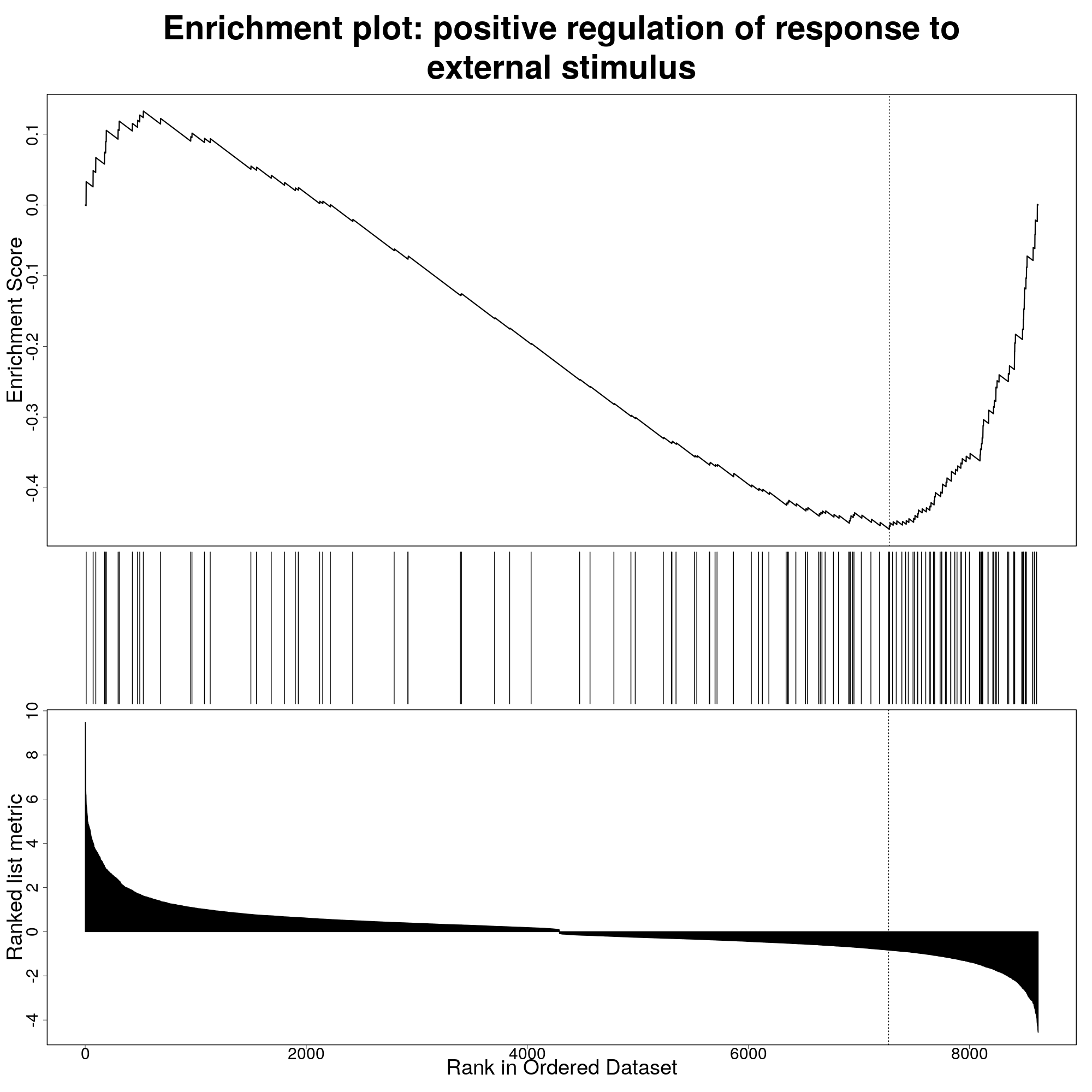

Supplement: Supplementary file 14 [file DataSheet_6.zip › Supplementary data 6 GSEA CCR2lo vs CCR2hi all samples/Project_high_vs_low_GSEA/GO_0032103.png]

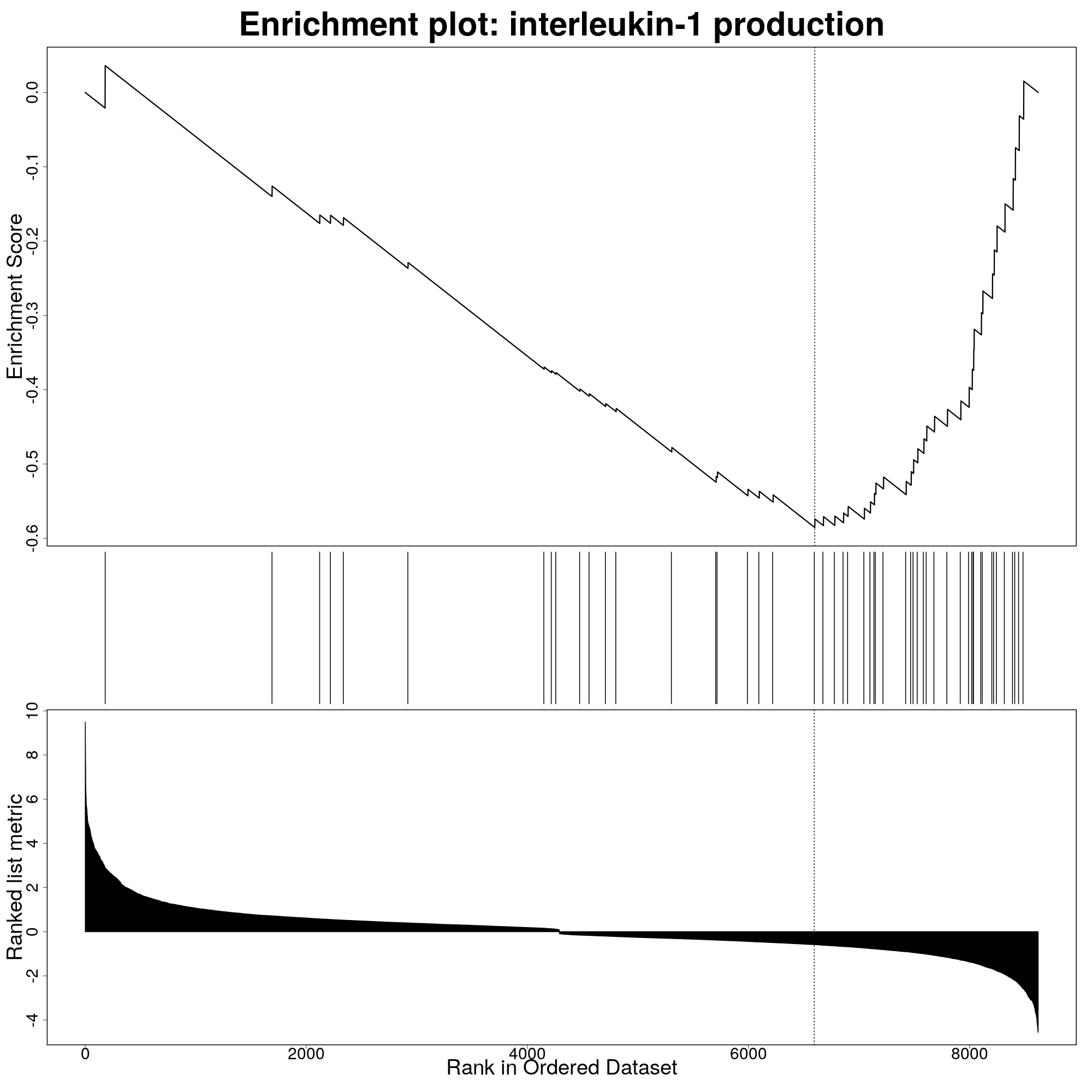

Supplement: Supplementary file 14 [file DataSheet_6.zip › Supplementary data 6 GSEA CCR2lo vs CCR2hi all samples/Project_high_vs_low_GSEA/GO_0032612.png]

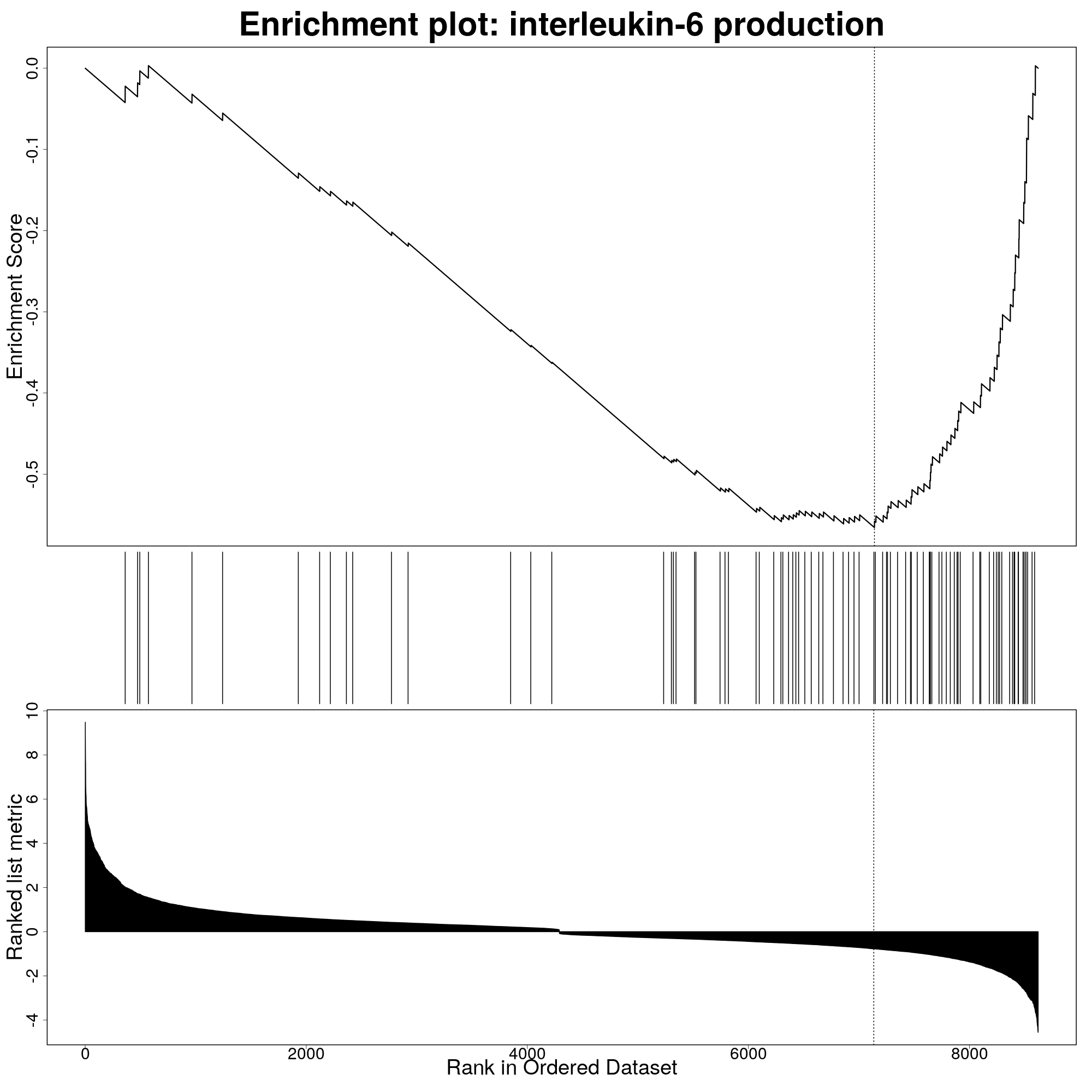

Supplement: Supplementary file 14 [file DataSheet_6.zip › Supplementary data 6 GSEA CCR2lo vs CCR2hi all samples/Project_high_vs_low_GSEA/GO_0032635.png]

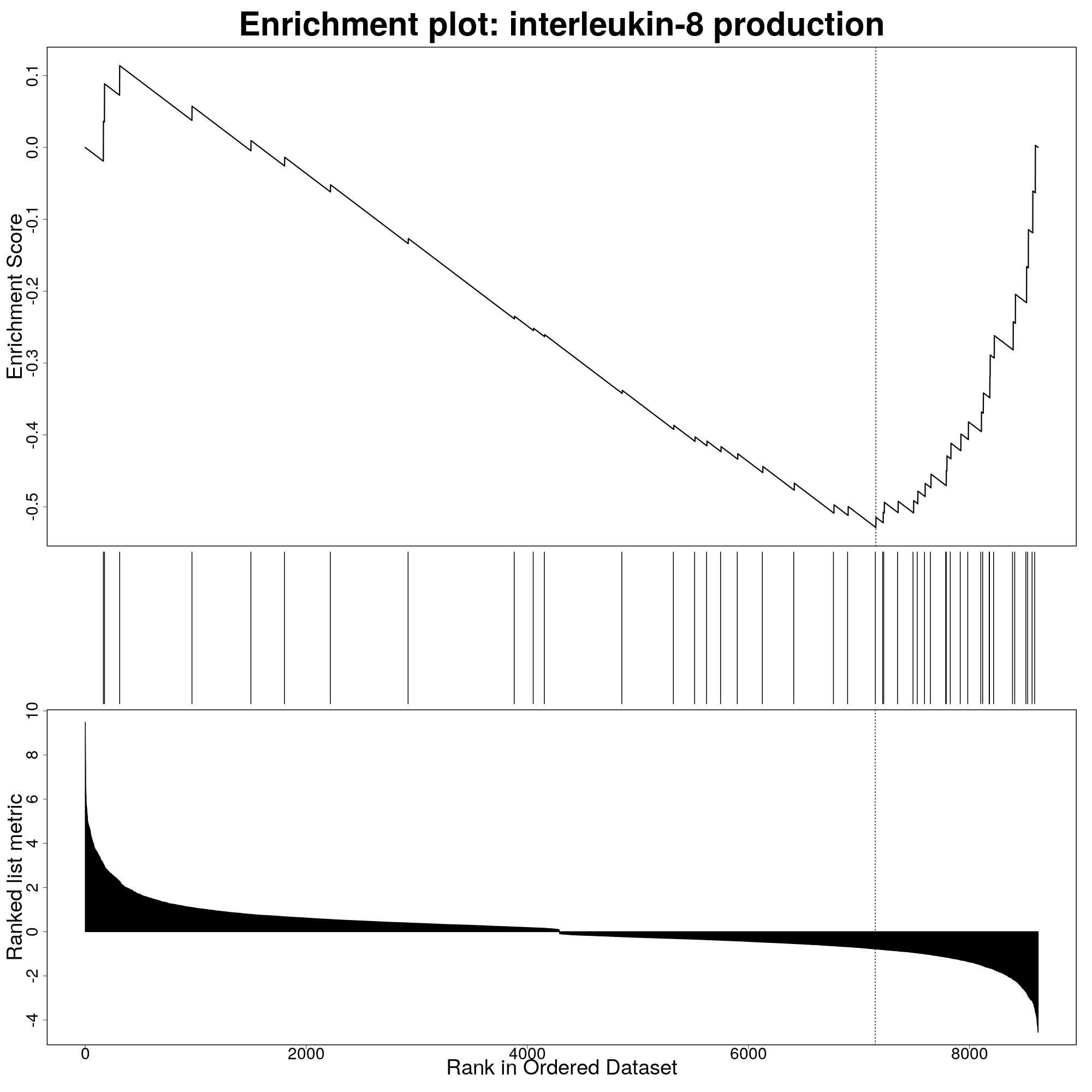

Supplement: Supplementary file 14 [file DataSheet_6.zip › Supplementary data 6 GSEA CCR2lo vs CCR2hi all samples/Project_high_vs_low_GSEA/GO_0032637.png]

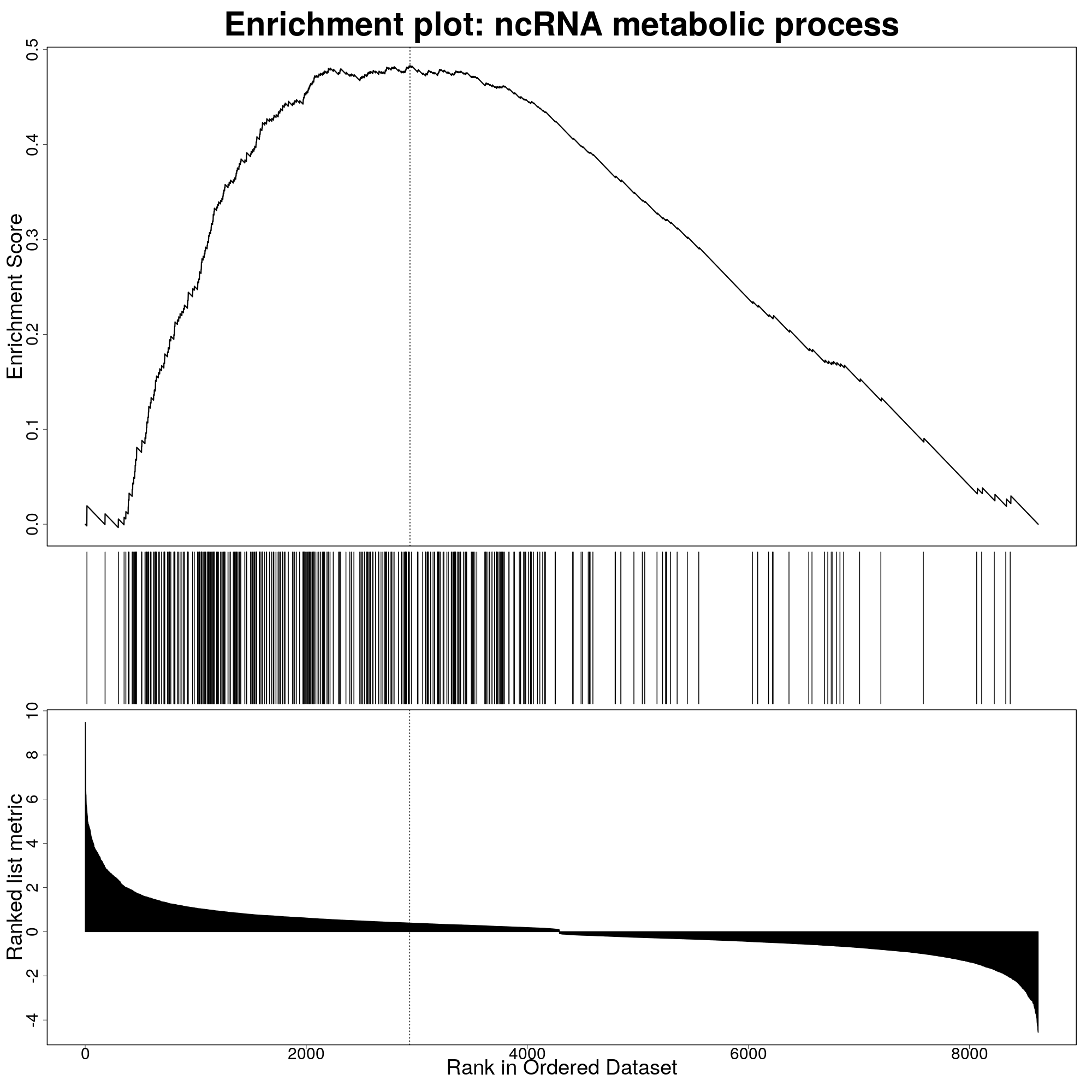

Supplement: Supplementary file 14 [file DataSheet_6.zip › Supplementary data 6 GSEA CCR2lo vs CCR2hi all samples/Project_high_vs_low_GSEA/GO_0034660.png]

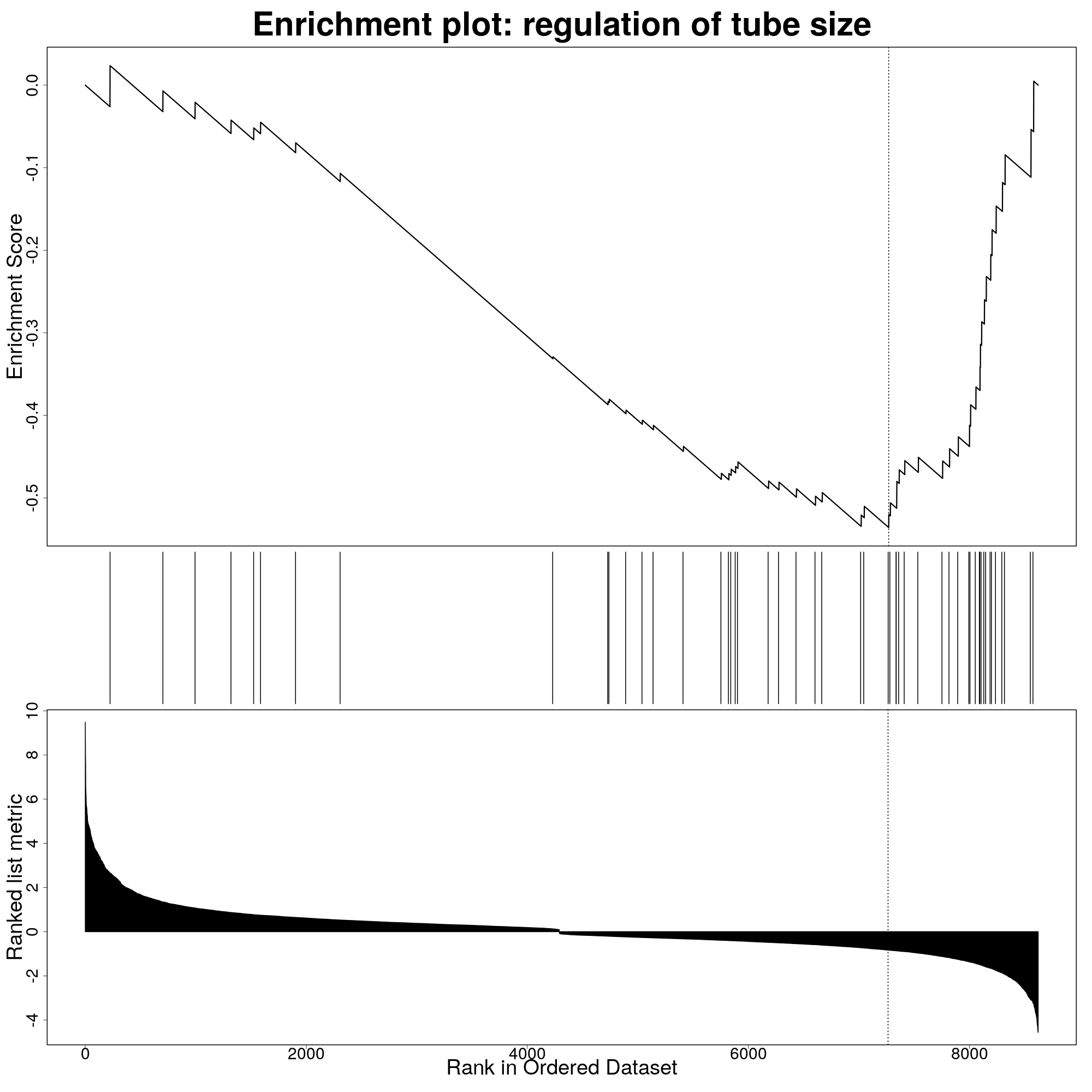

Supplement: Supplementary file 14 [file DataSheet_6.zip › Supplementary data 6 GSEA CCR2lo vs CCR2hi all samples/Project_high_vs_low_GSEA/GO_0035150.png]

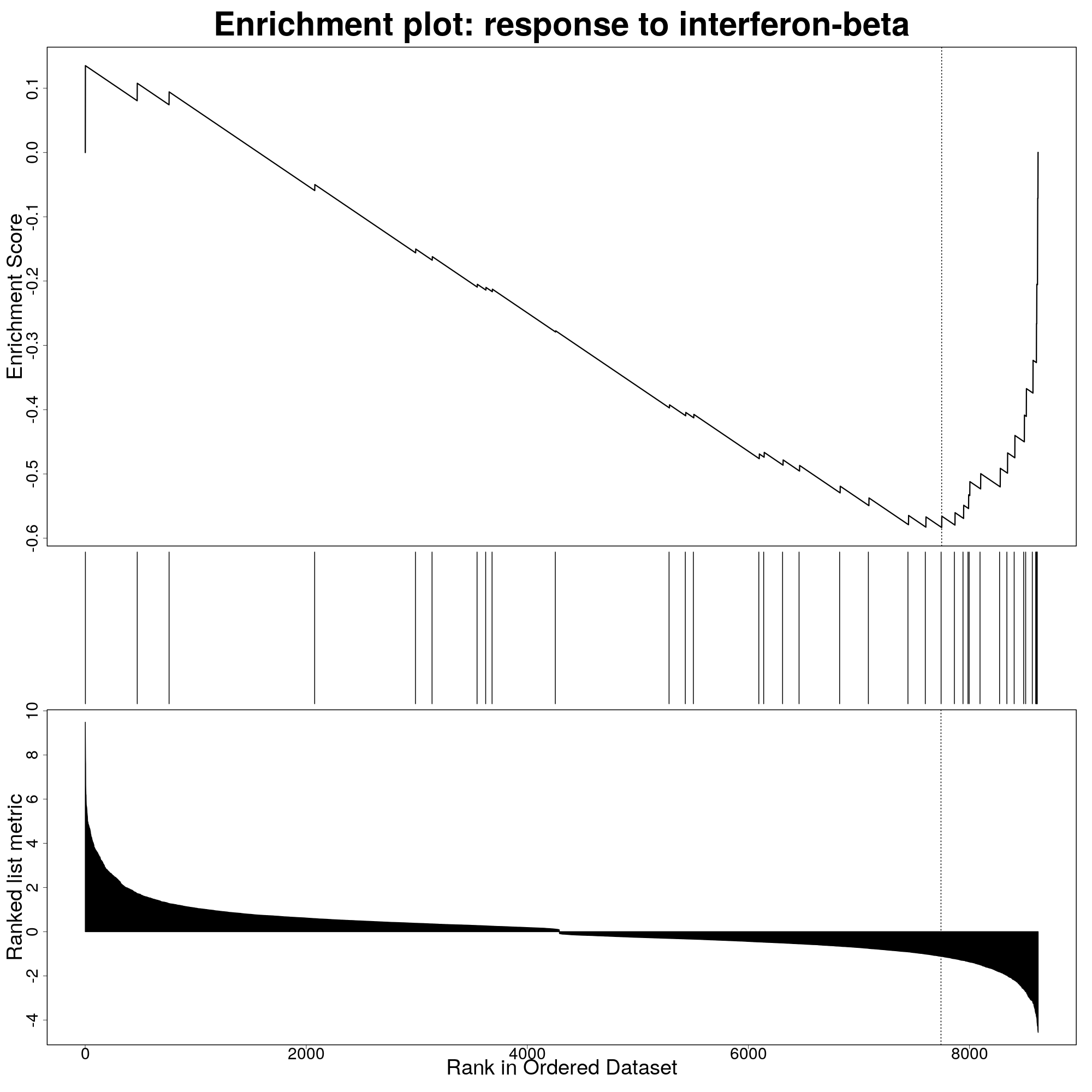

Supplement: Supplementary file 14 [file DataSheet_6.zip › Supplementary data 6 GSEA CCR2lo vs CCR2hi all samples/Project_high_vs_low_GSEA/GO_0035456.png]

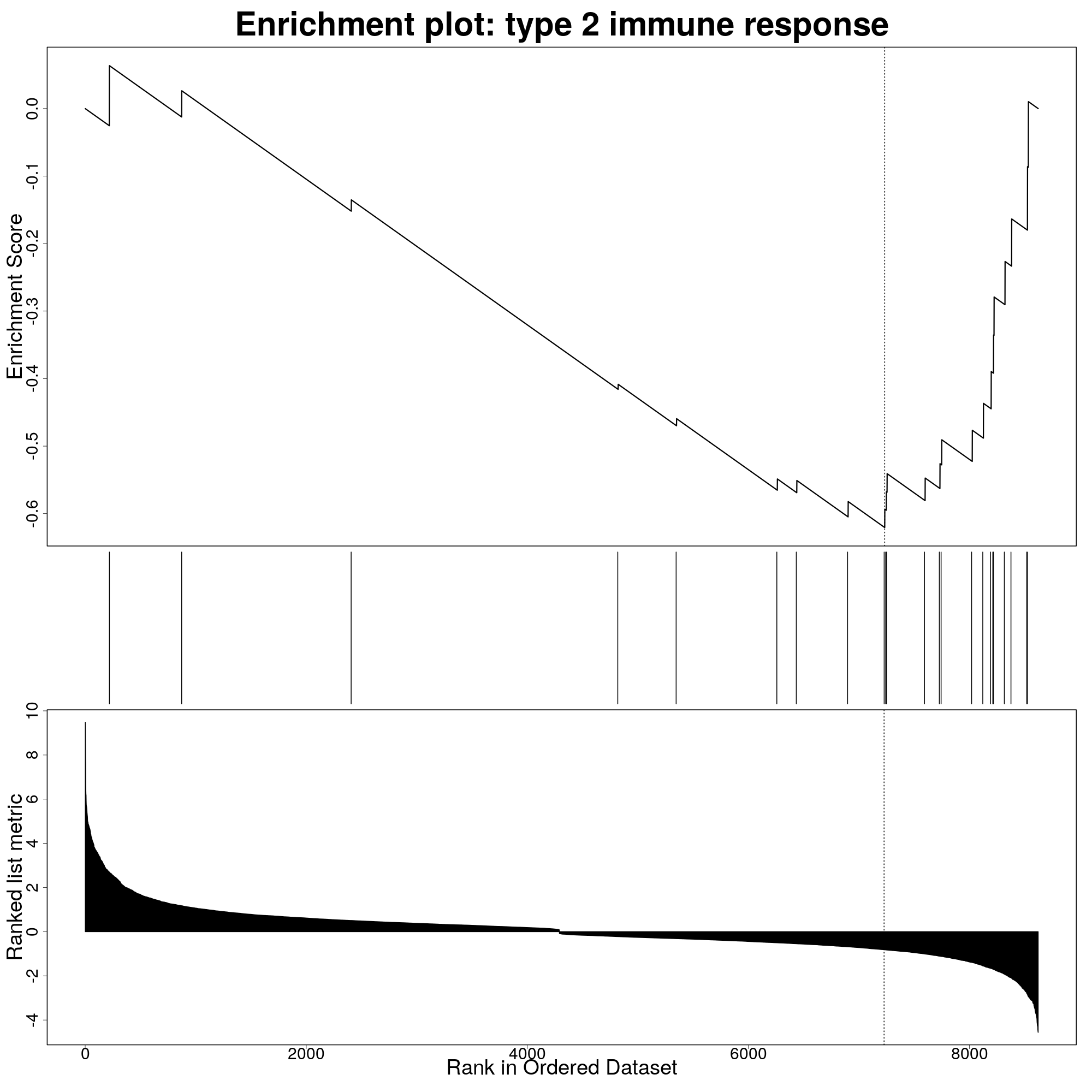

Supplement: Supplementary file 14 [file DataSheet_6.zip › Supplementary data 6 GSEA CCR2lo vs CCR2hi all samples/Project_high_vs_low_GSEA/GO_0042092.png]

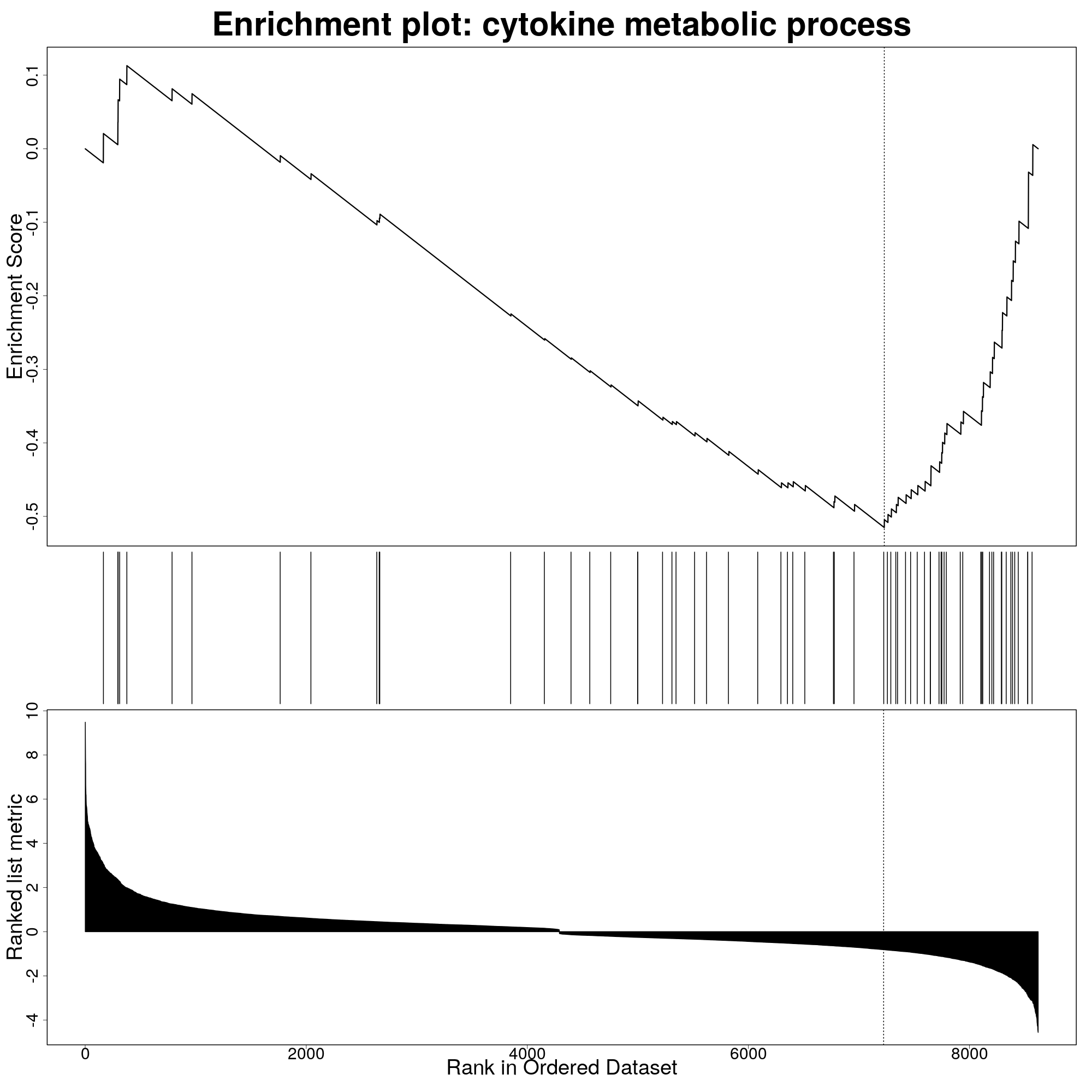

Supplement: Supplementary file 14 [file DataSheet_6.zip › Supplementary data 6 GSEA CCR2lo vs CCR2hi all samples/Project_high_vs_low_GSEA/GO_0042107.png]

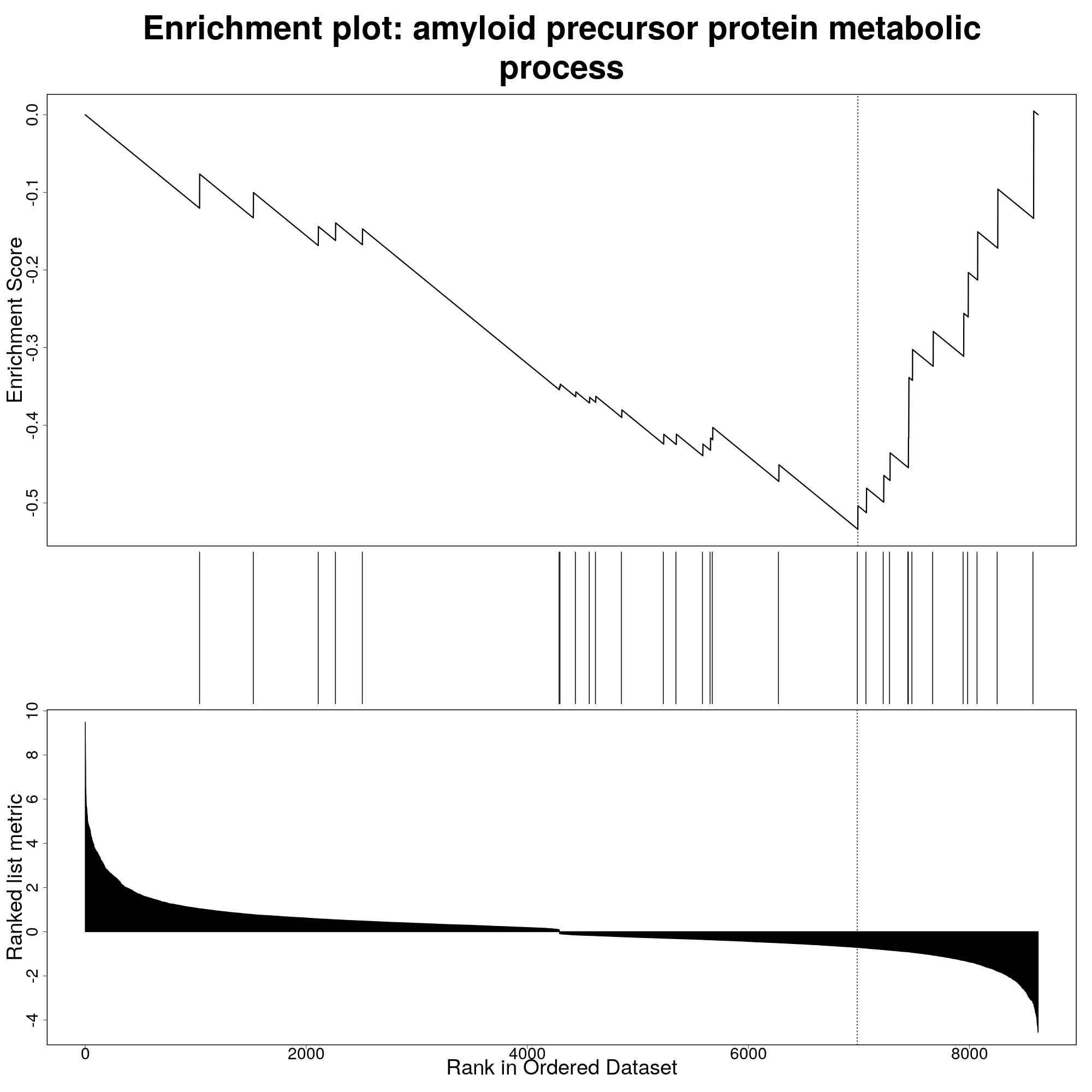

Supplement: Supplementary file 14 [file DataSheet_6.zip › Supplementary data 6 GSEA CCR2lo vs CCR2hi all samples/Project_high_vs_low_GSEA/GO_0042982.png]

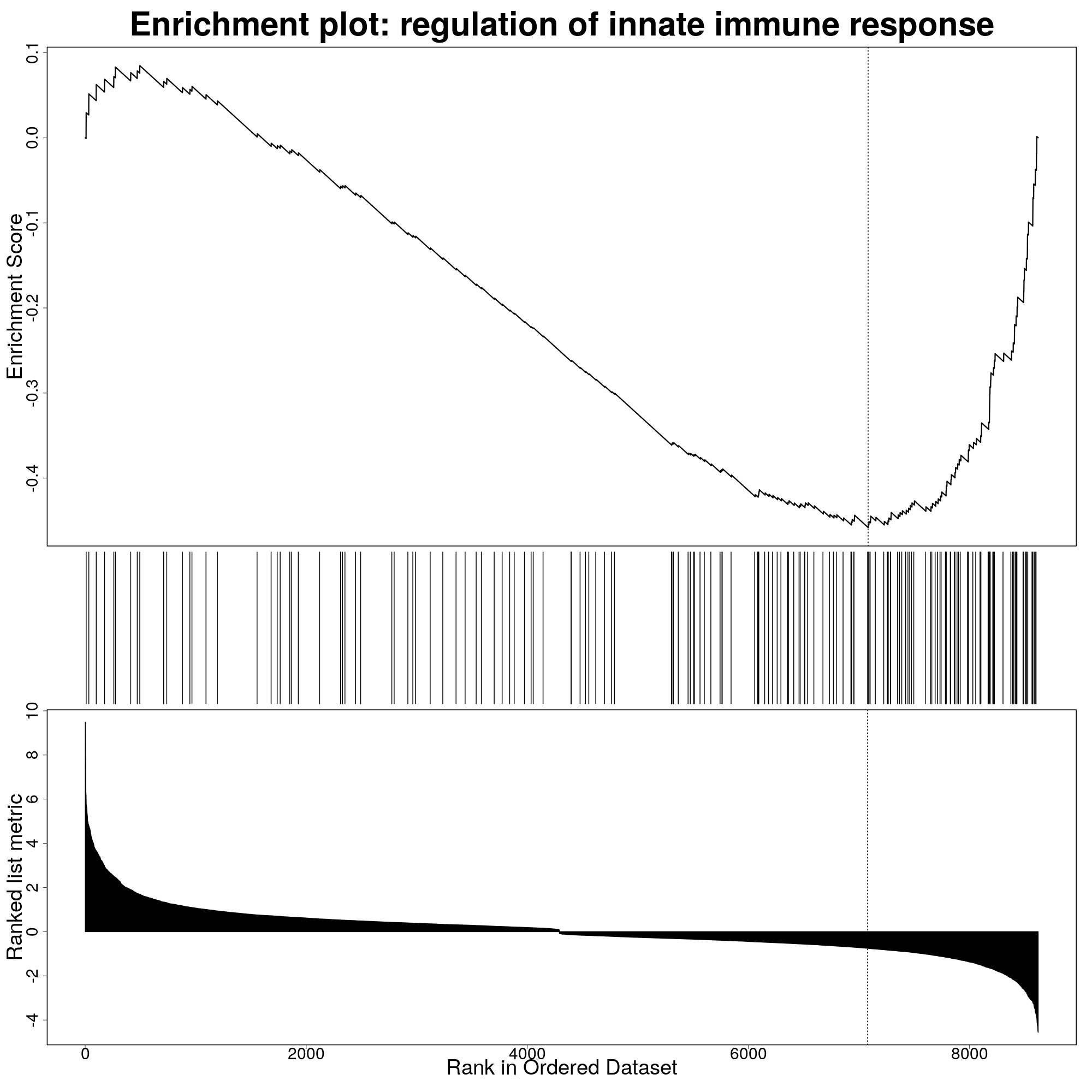

Supplement: Supplementary file 14 [file DataSheet_6.zip › Supplementary data 6 GSEA CCR2lo vs CCR2hi all samples/Project_high_vs_low_GSEA/GO_0045088.png]

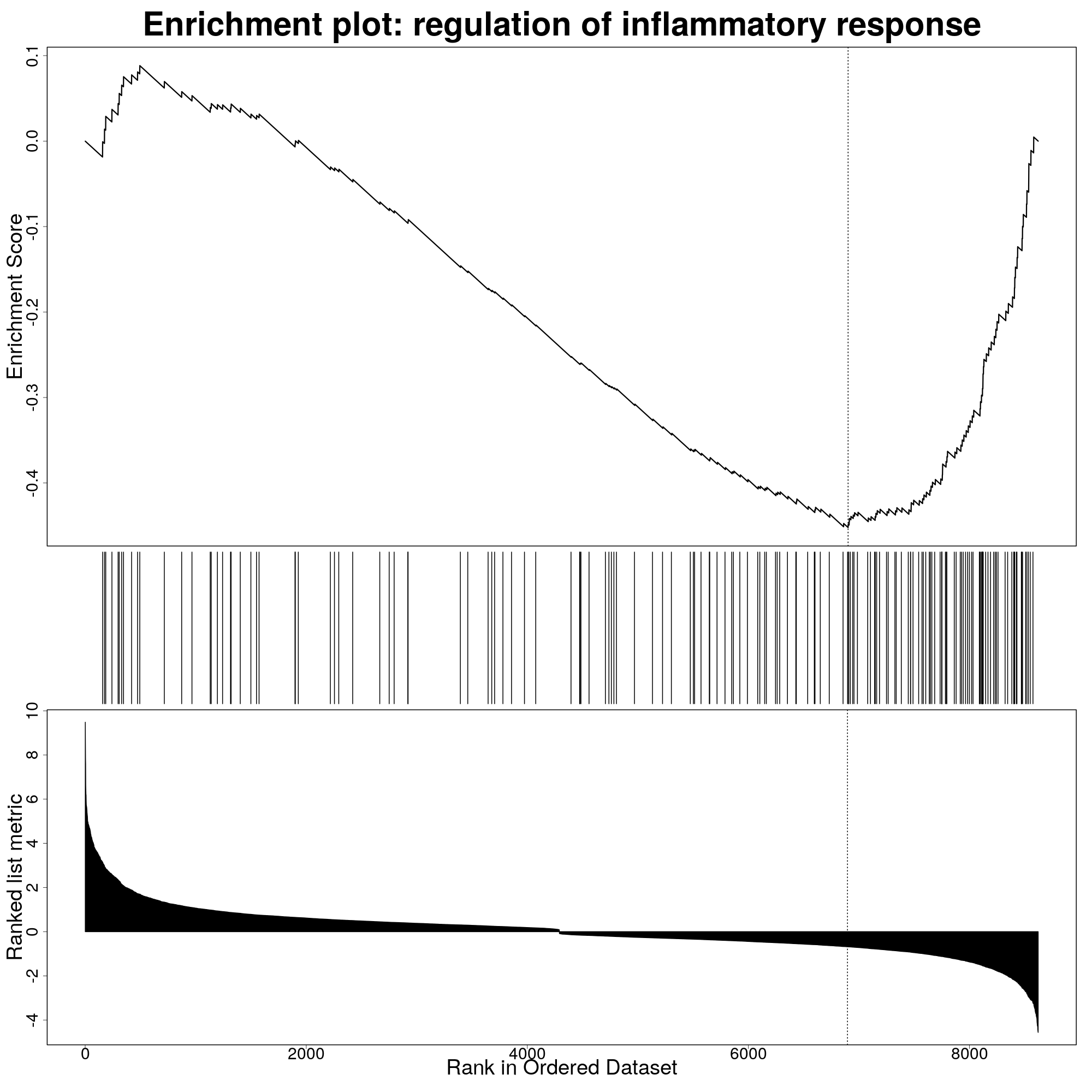

Supplement: Supplementary file 14 [file DataSheet_6.zip › Supplementary data 6 GSEA CCR2lo vs CCR2hi all samples/Project_high_vs_low_GSEA/GO_0050727.png]

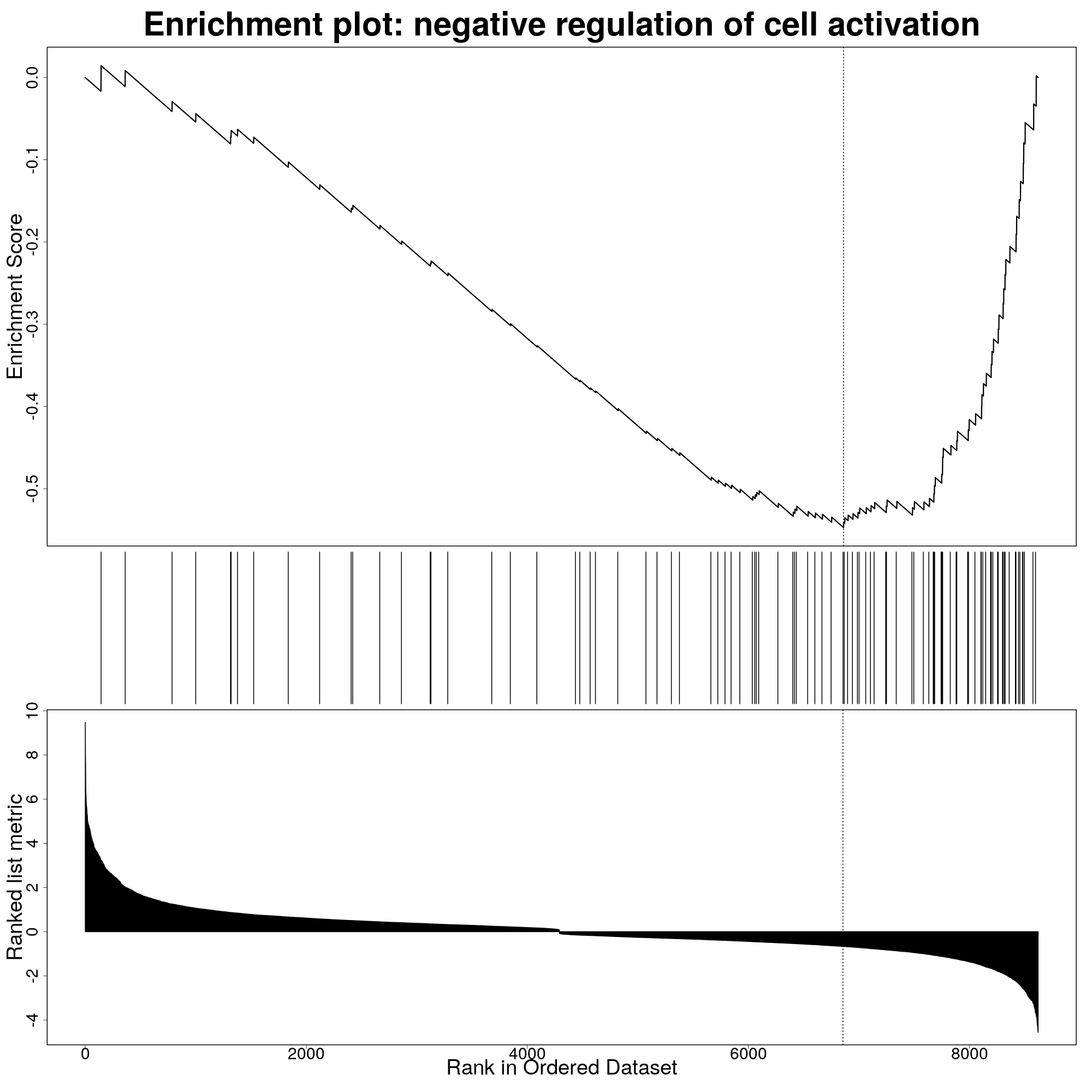

Supplement: Supplementary file 14 [file DataSheet_6.zip › Supplementary data 6 GSEA CCR2lo vs CCR2hi all samples/Project_high_vs_low_GSEA/GO_0050866.png]

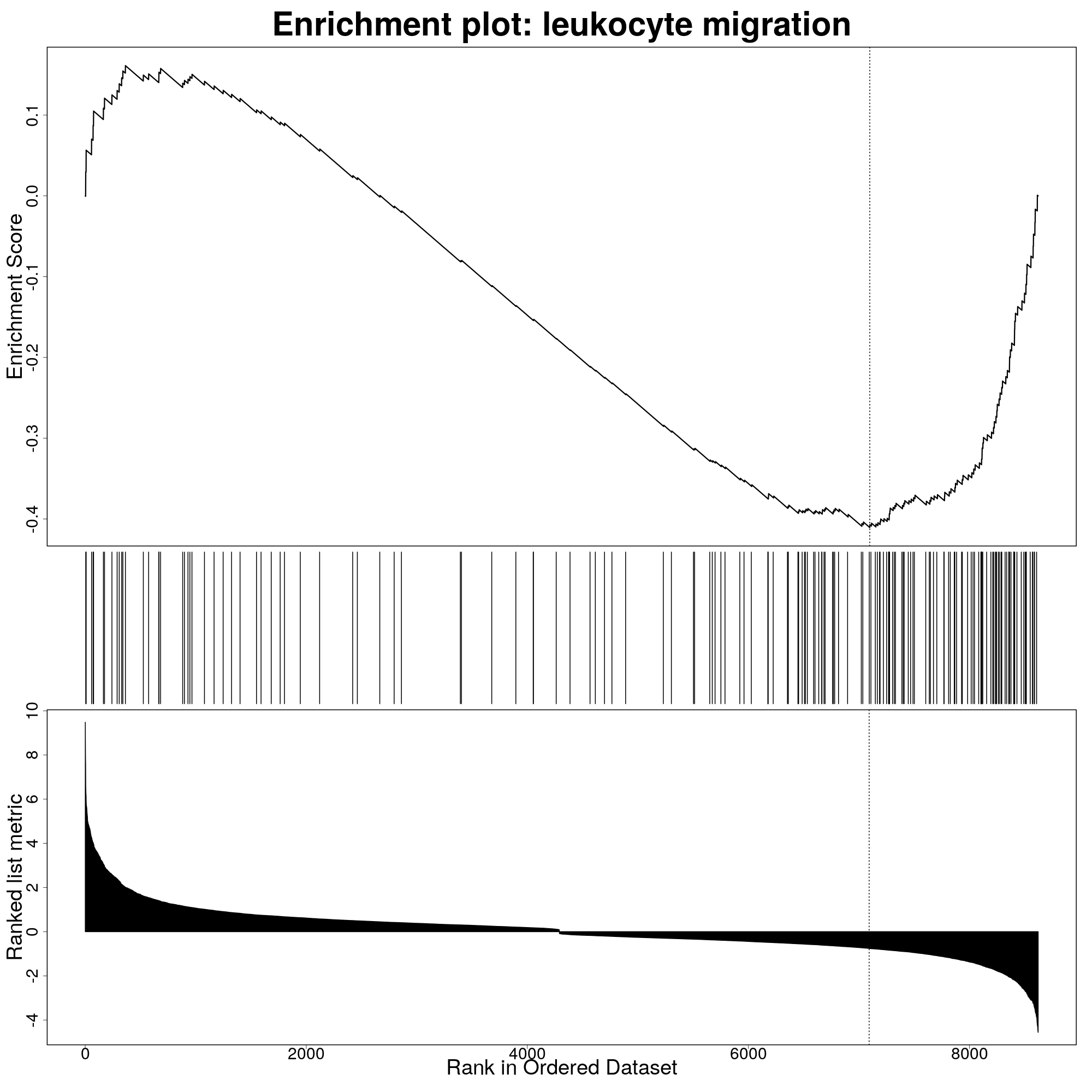

Supplement: Supplementary file 14 [file DataSheet_6.zip › Supplementary data 6 GSEA CCR2lo vs CCR2hi all samples/Project_high_vs_low_GSEA/GO_0050900.png]

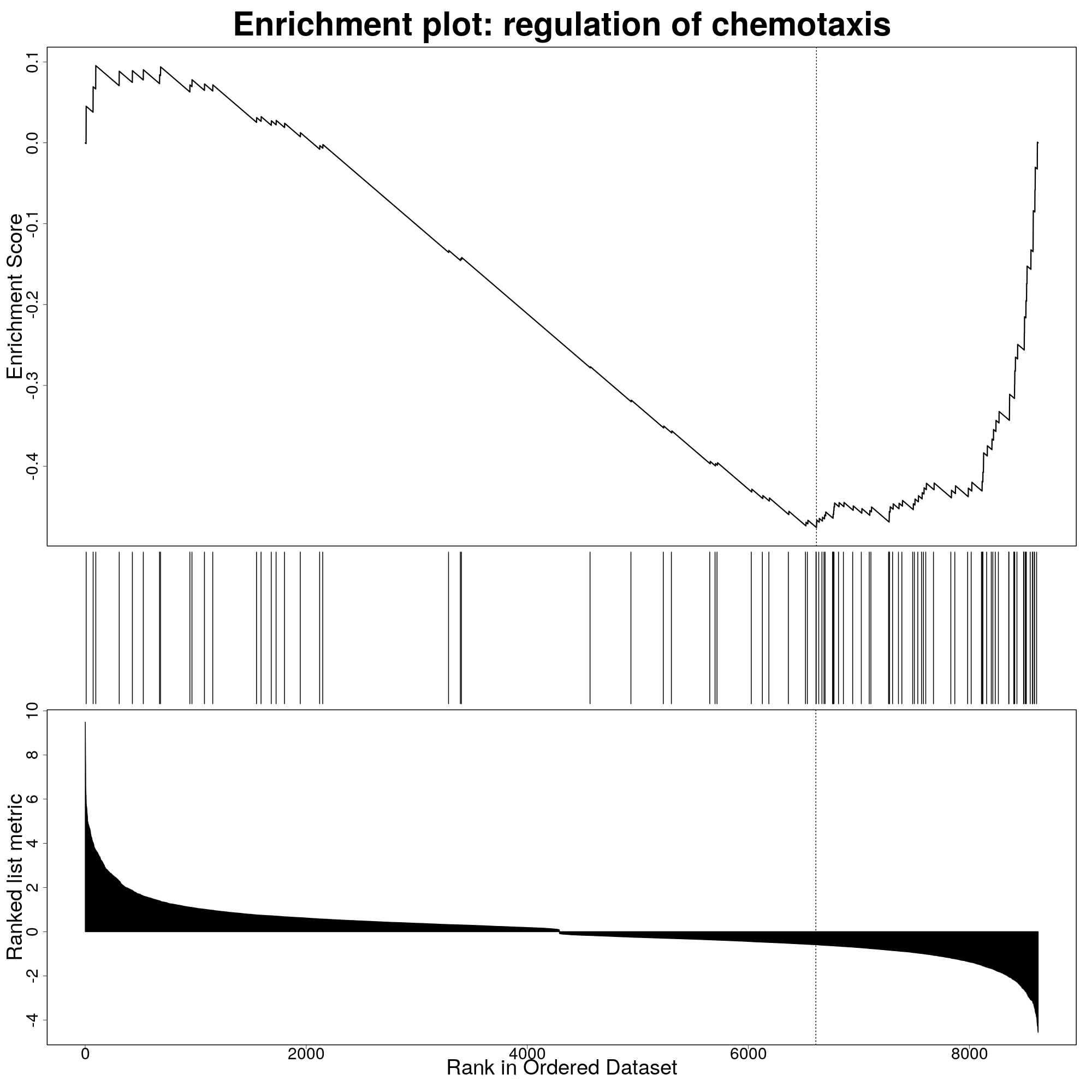

Supplement: Supplementary file 14 [file DataSheet_6.zip › Supplementary data 6 GSEA CCR2lo vs CCR2hi all samples/Project_high_vs_low_GSEA/GO_0050920.png]

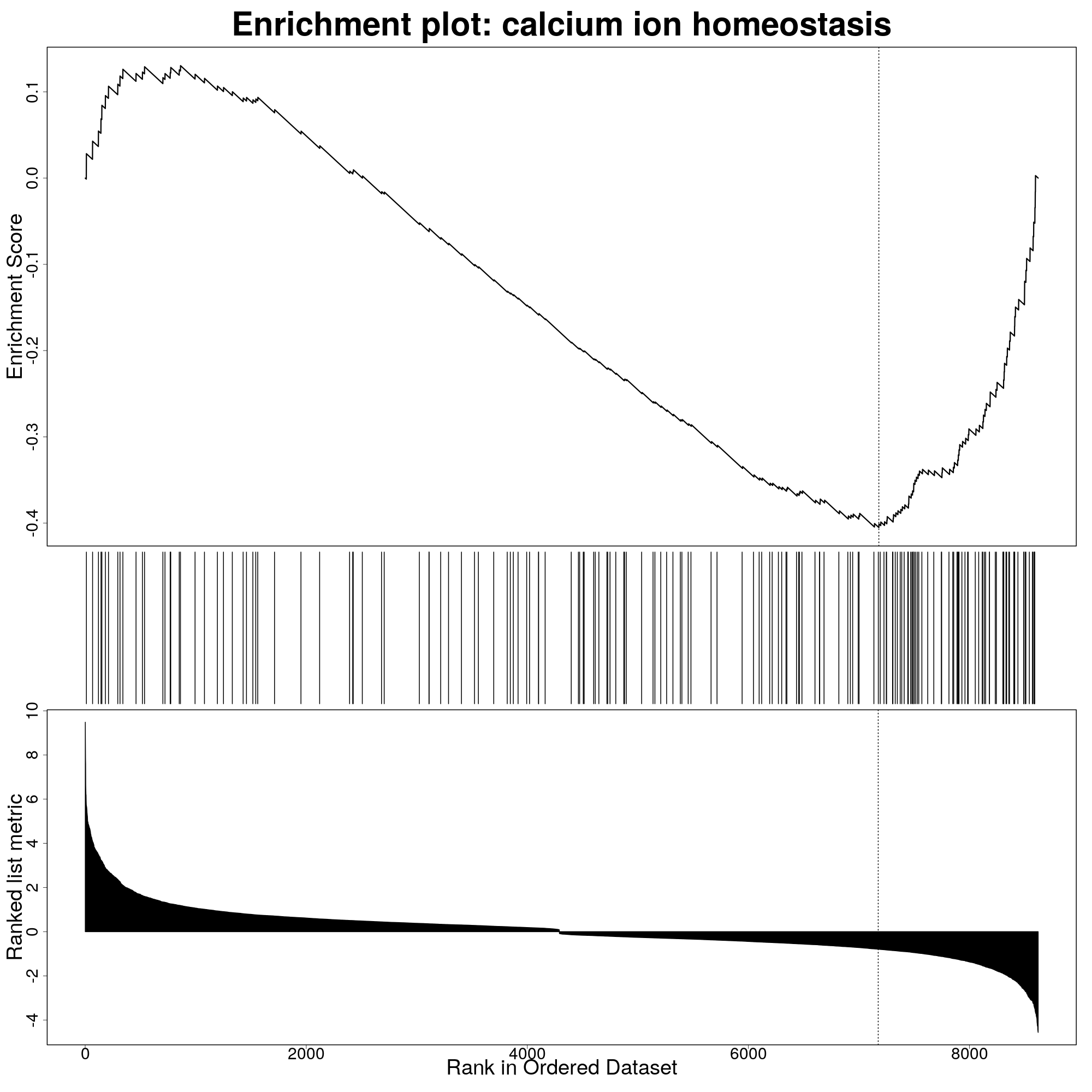

Supplement: Supplementary file 14 [file DataSheet_6.zip › Supplementary data 6 GSEA CCR2lo vs CCR2hi all samples/Project_high_vs_low_GSEA/GO_0055074.png]

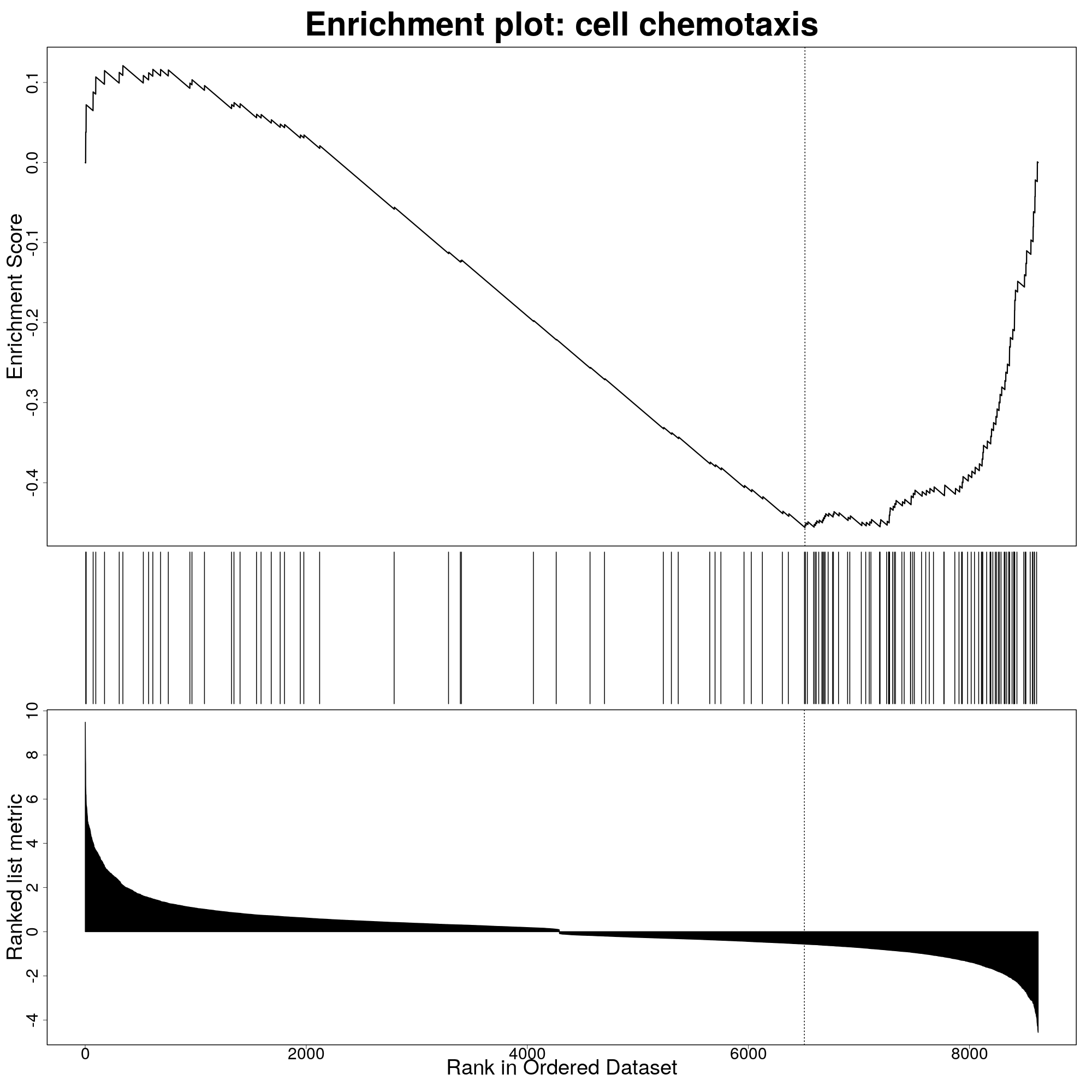

Supplement: Supplementary file 14 [file DataSheet_6.zip › Supplementary data 6 GSEA CCR2lo vs CCR2hi all samples/Project_high_vs_low_GSEA/GO_0060326.png]

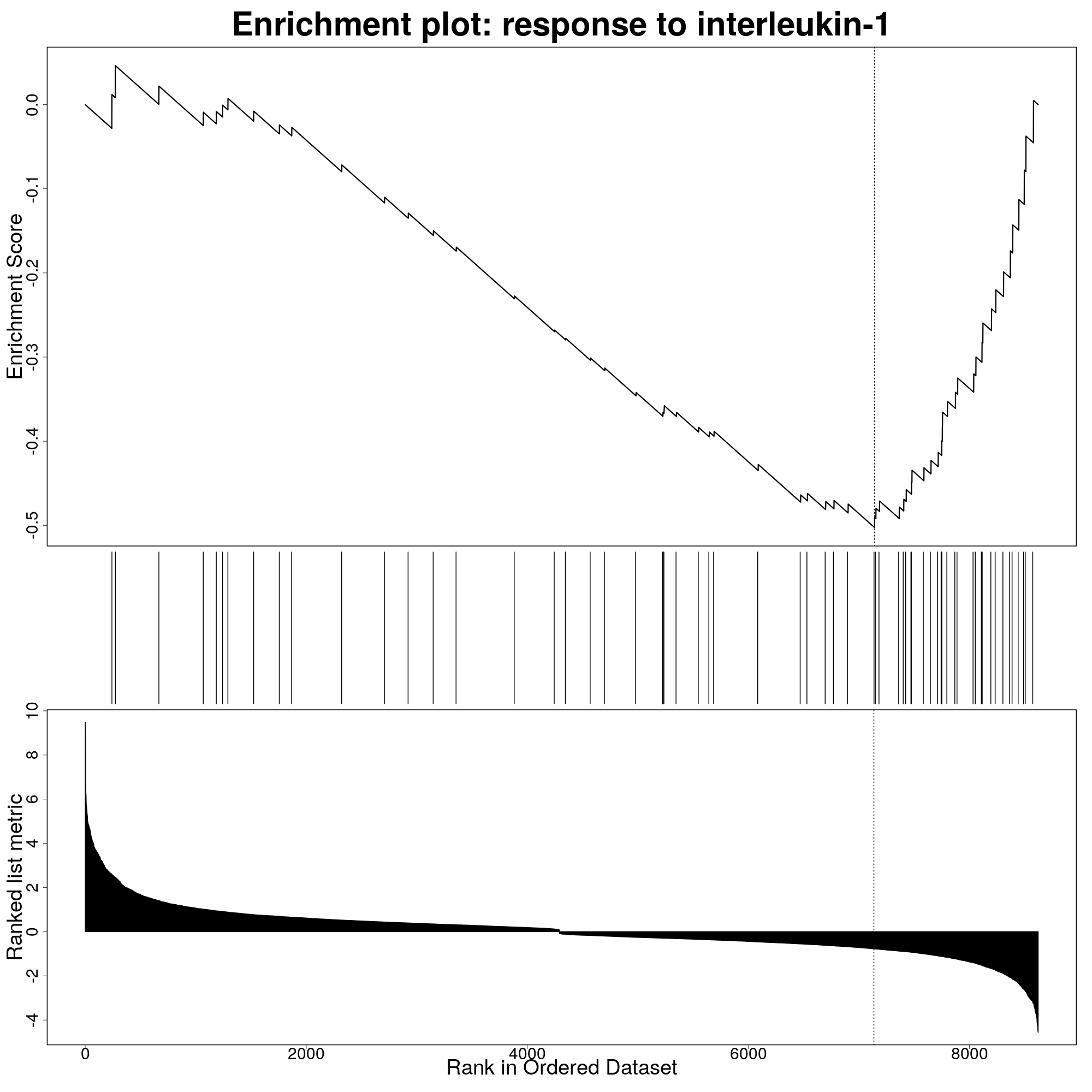

Supplement: Supplementary file 14 [file DataSheet_6.zip › Supplementary data 6 GSEA CCR2lo vs CCR2hi all samples/Project_high_vs_low_GSEA/GO_0070555.png]

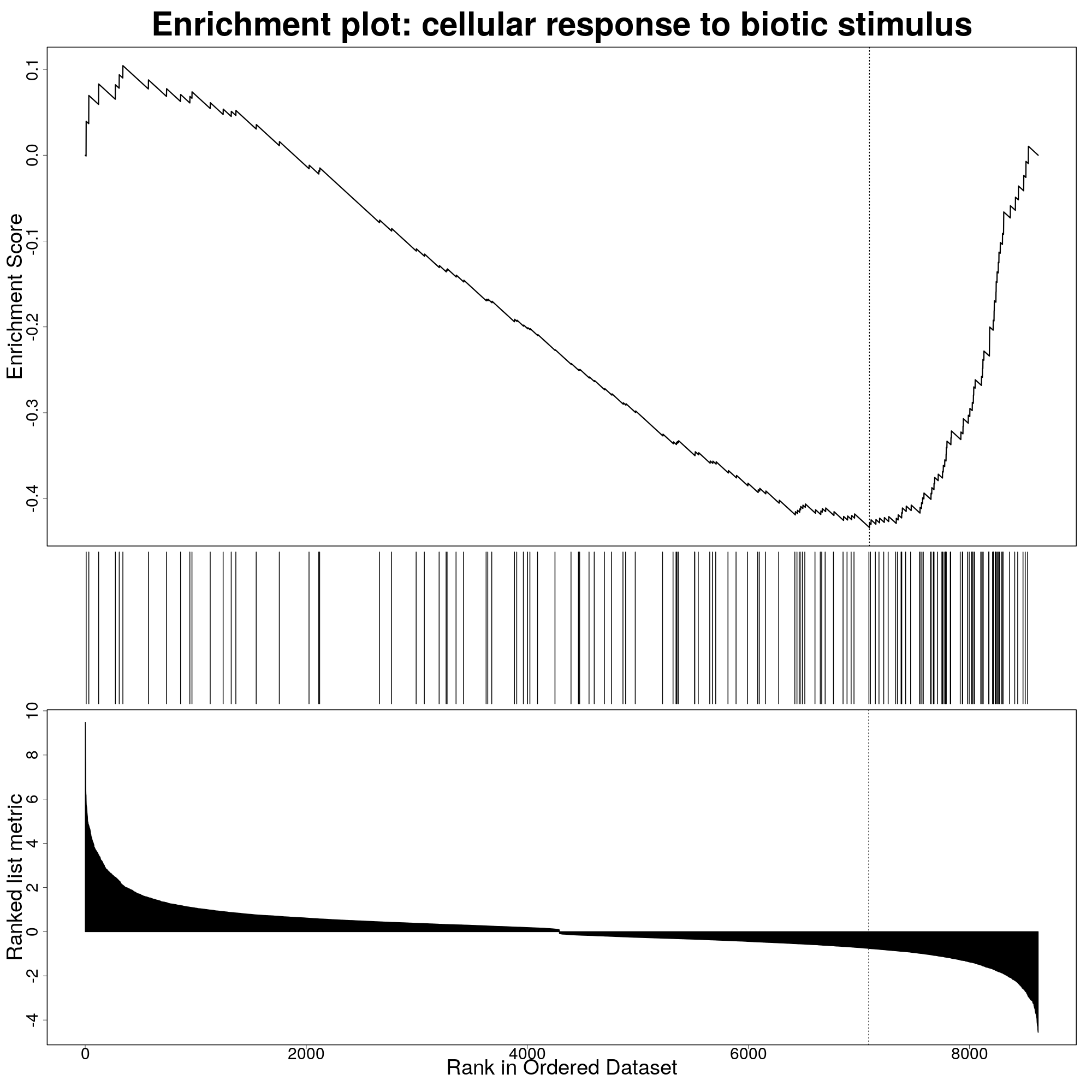

Supplement: Supplementary file 14 [file DataSheet_6.zip › Supplementary data 6 GSEA CCR2lo vs CCR2hi all samples/Project_high_vs_low_GSEA/GO_0071216.png]

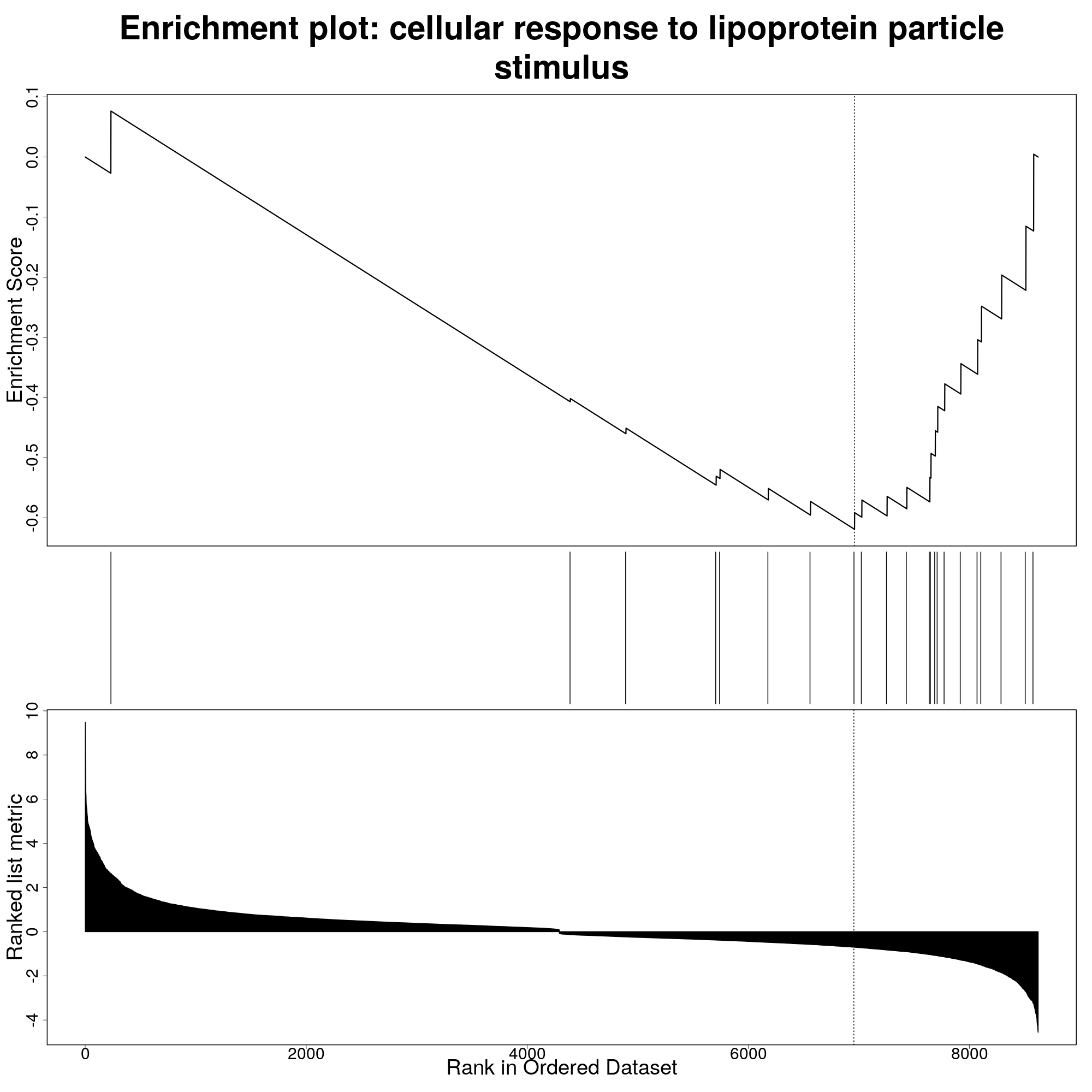

Supplement: Supplementary file 14 [file DataSheet_6.zip › Supplementary data 6 GSEA CCR2lo vs CCR2hi all samples/Project_high_vs_low_GSEA/GO_0071402.png]

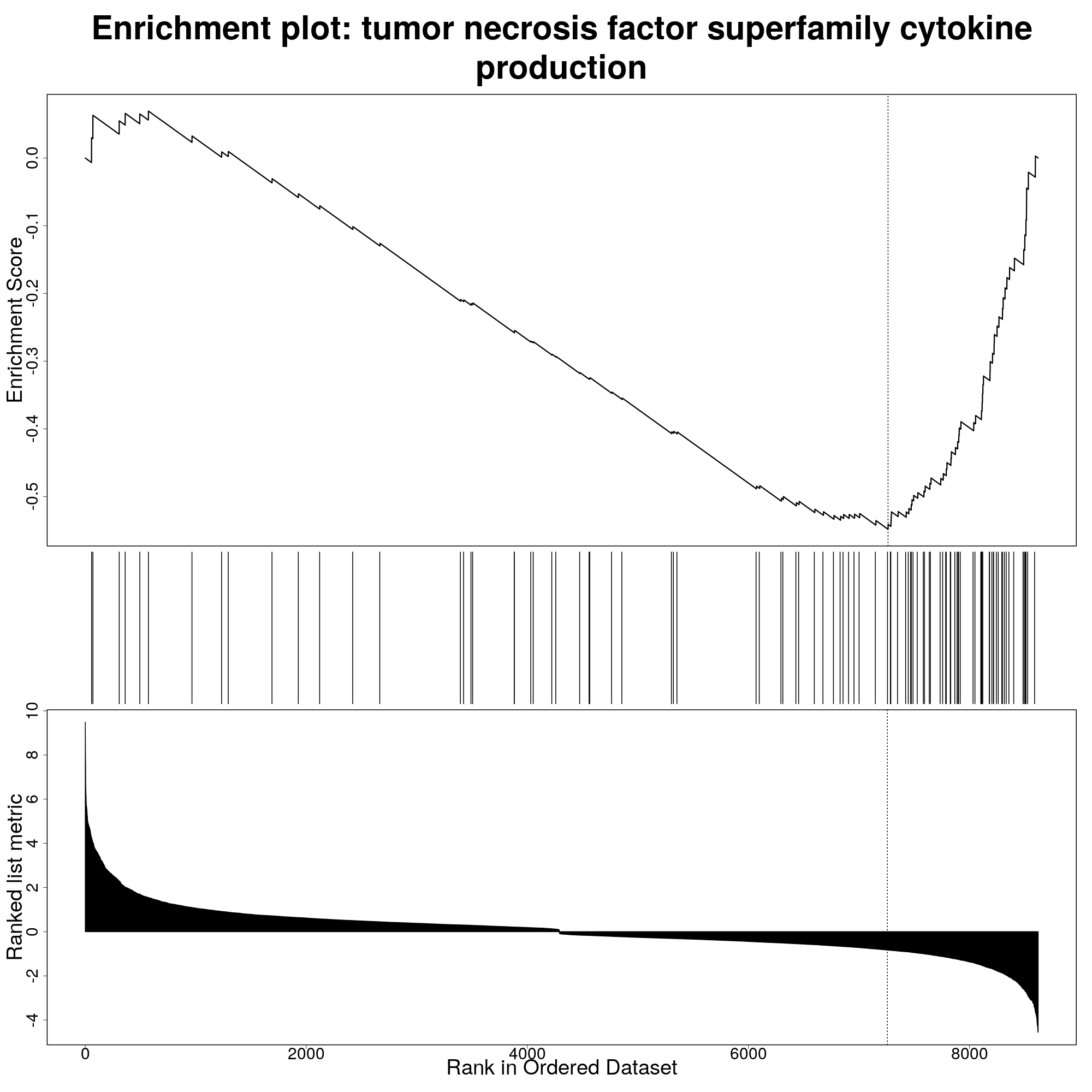

Supplement: Supplementary file 14 [file DataSheet_6.zip › Supplementary data 6 GSEA CCR2lo vs CCR2hi all samples/Project_high_vs_low_GSEA/GO_0071706.png]

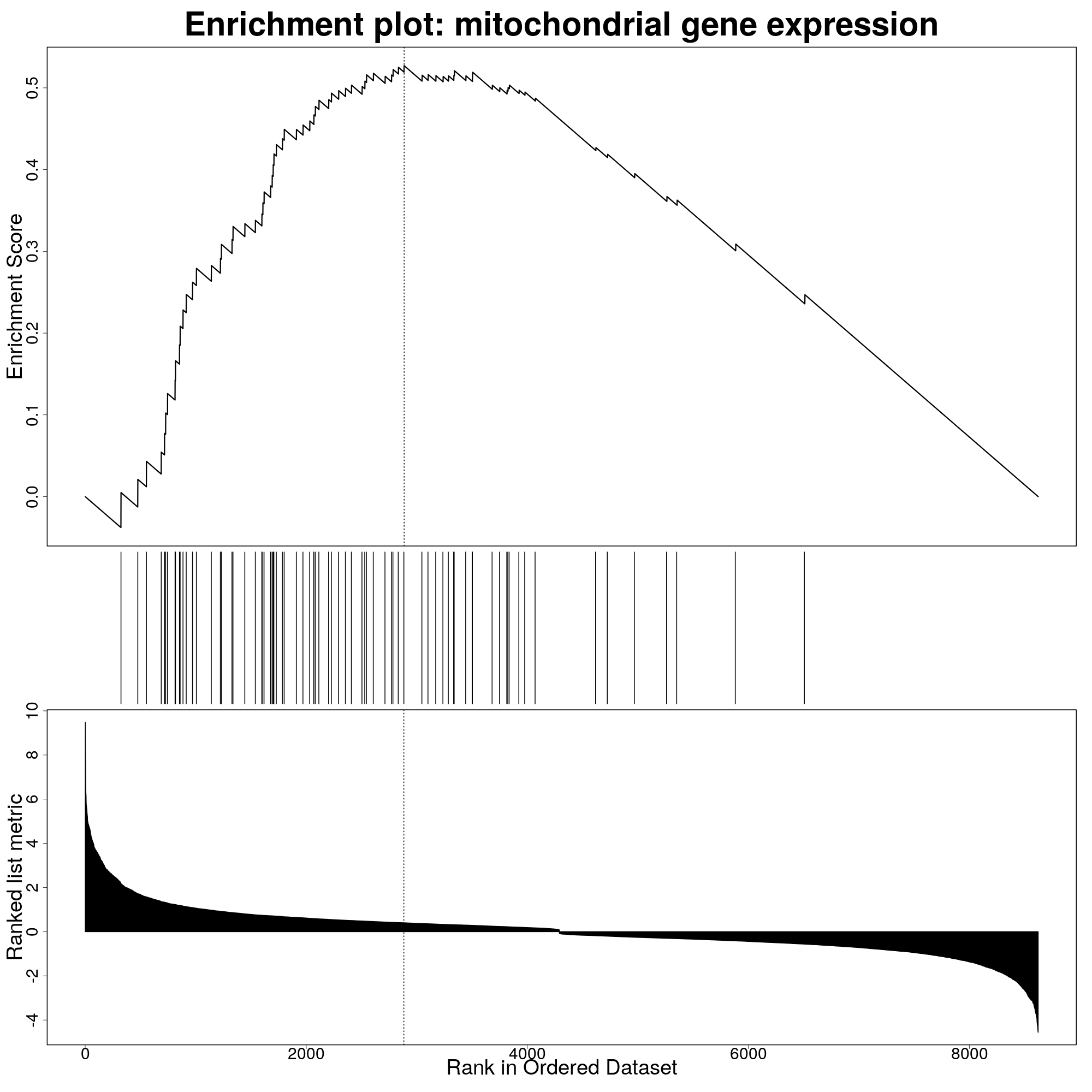

Supplement: Supplementary file 14 [file DataSheet_6.zip › Supplementary data 6 GSEA CCR2lo vs CCR2hi all samples/Project_high_vs_low_GSEA/GO_0140053.png]

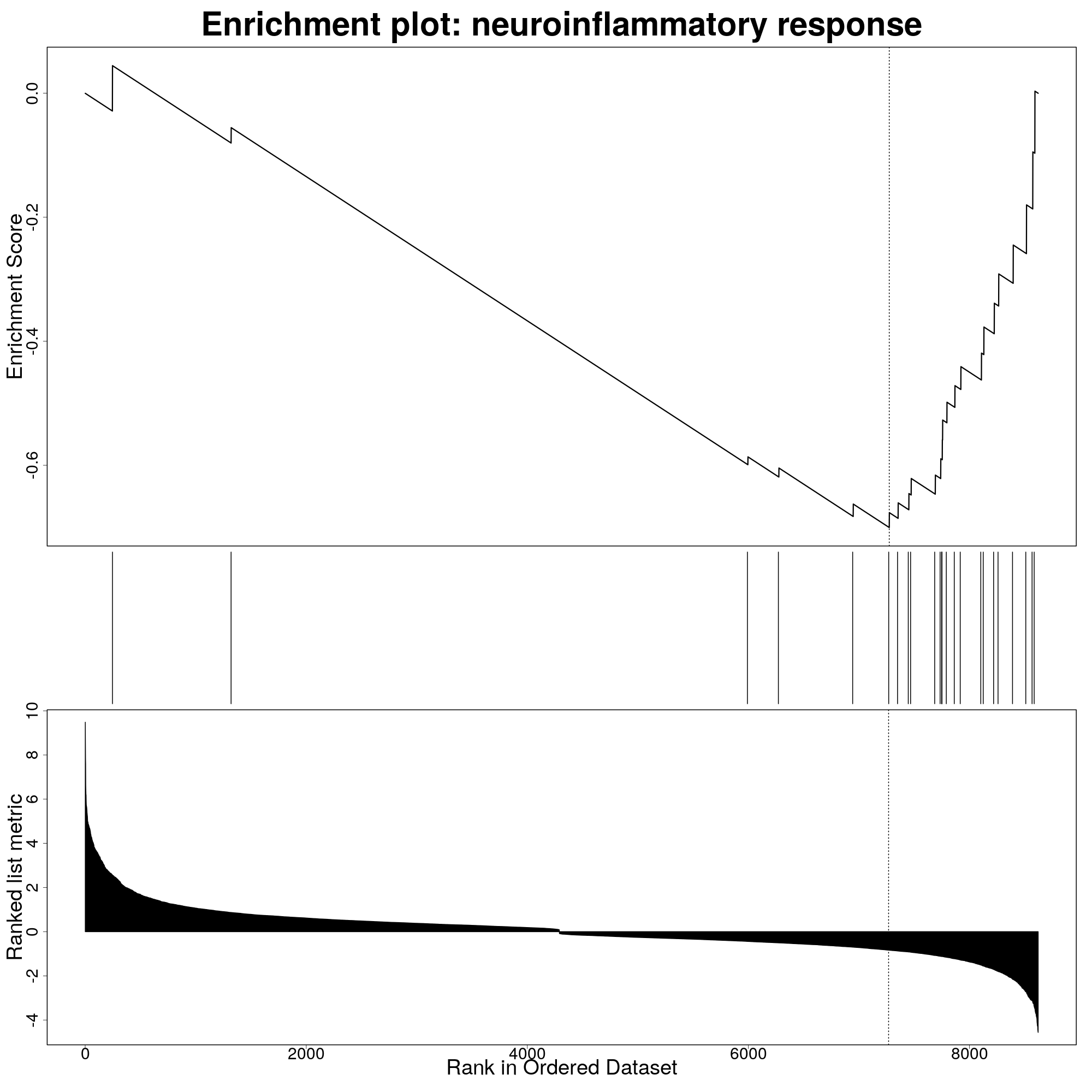

Supplement: Supplementary file 14 [file DataSheet_6.zip › Supplementary data 6 GSEA CCR2lo vs CCR2hi all samples/Project_high_vs_low_GSEA/GO_0150076.png]

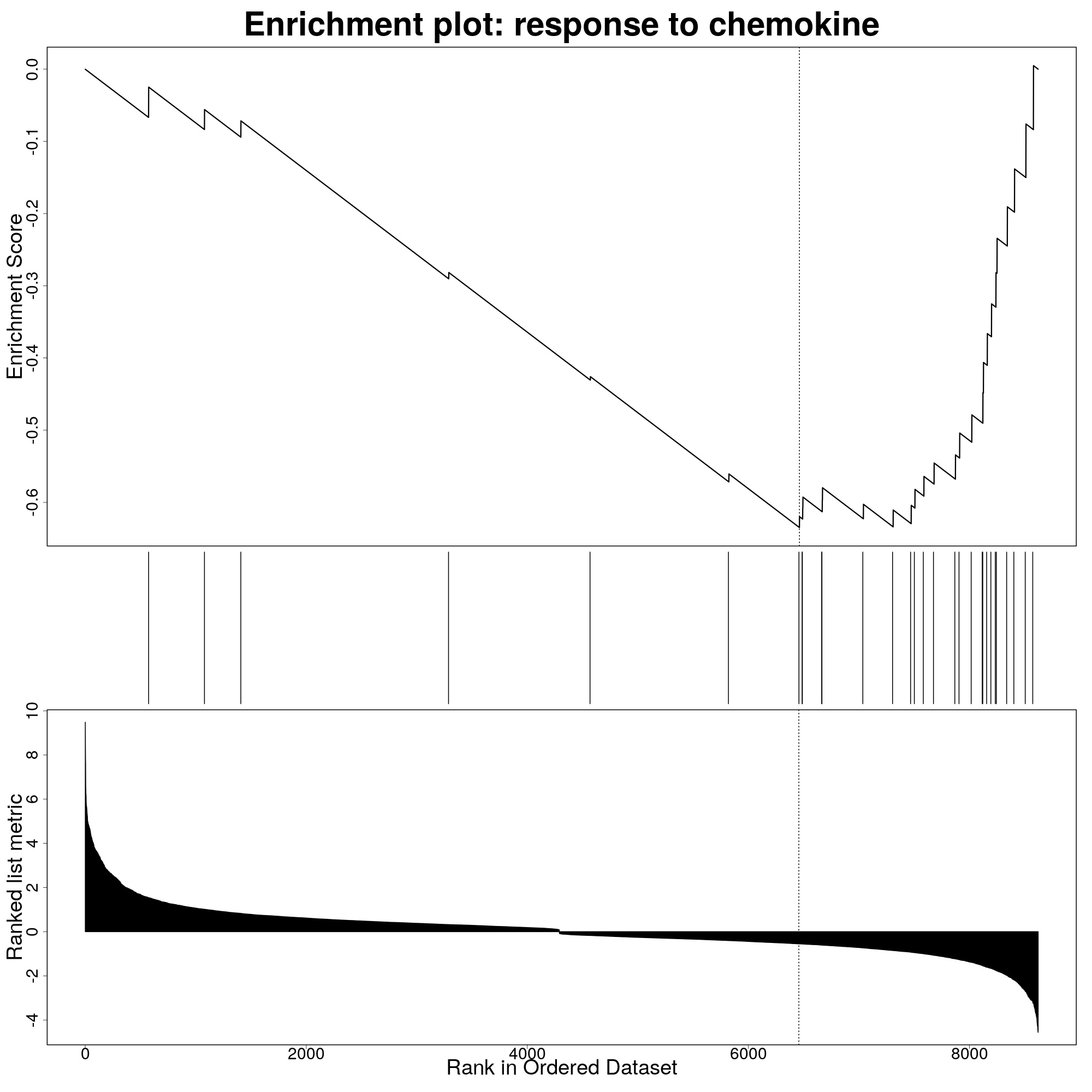

Supplement: Supplementary file 14 [file DataSheet_6.zip › Supplementary data 6 GSEA CCR2lo vs CCR2hi all samples/Project_high_vs_low_GSEA/GO_1990868.png]

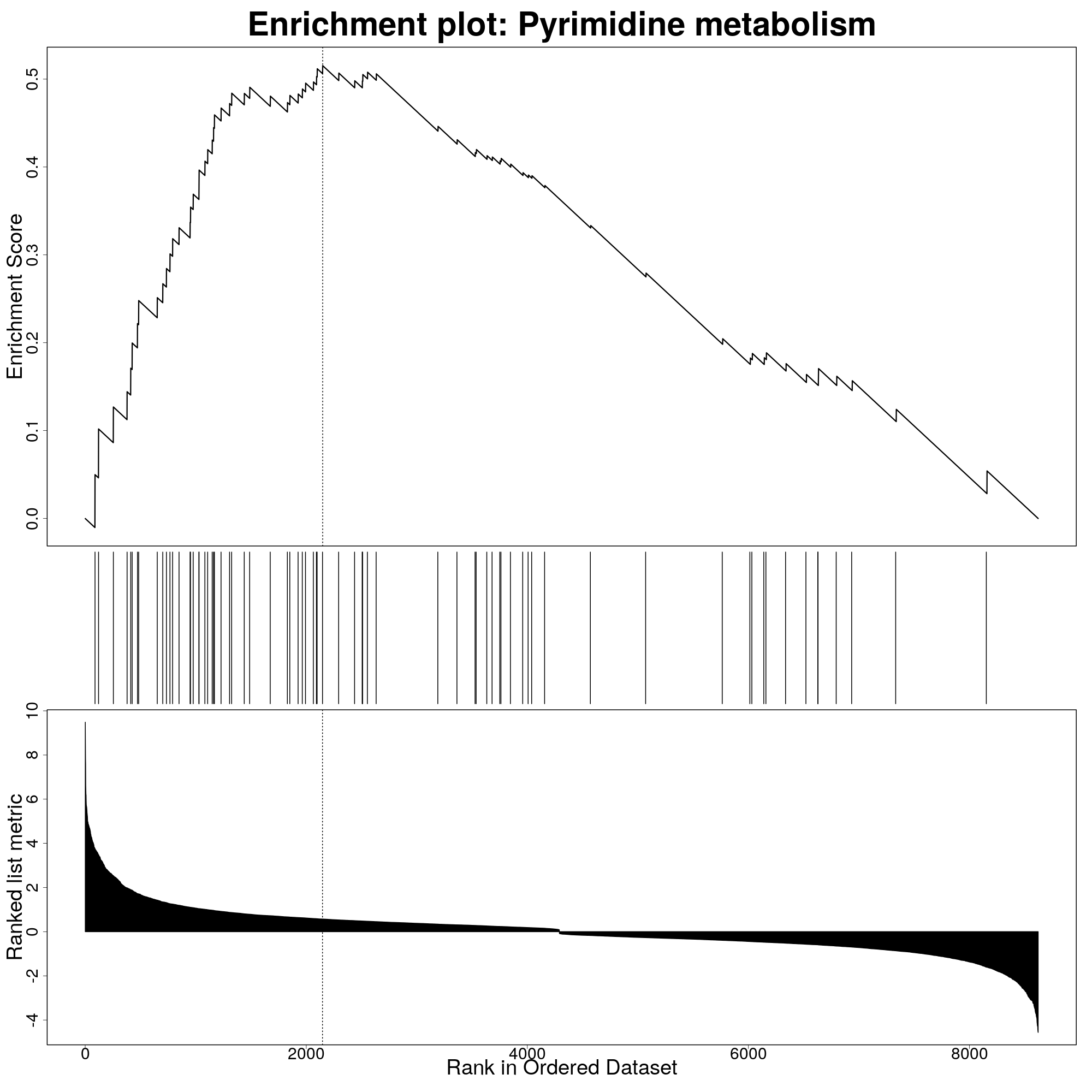

Supplement: Supplementary file 14 [file DataSheet_6.zip › Supplementary data 6 GSEA CCR2lo vs CCR2hi all samples/Project_high_vs_low_GSEA/mmu00240.png]

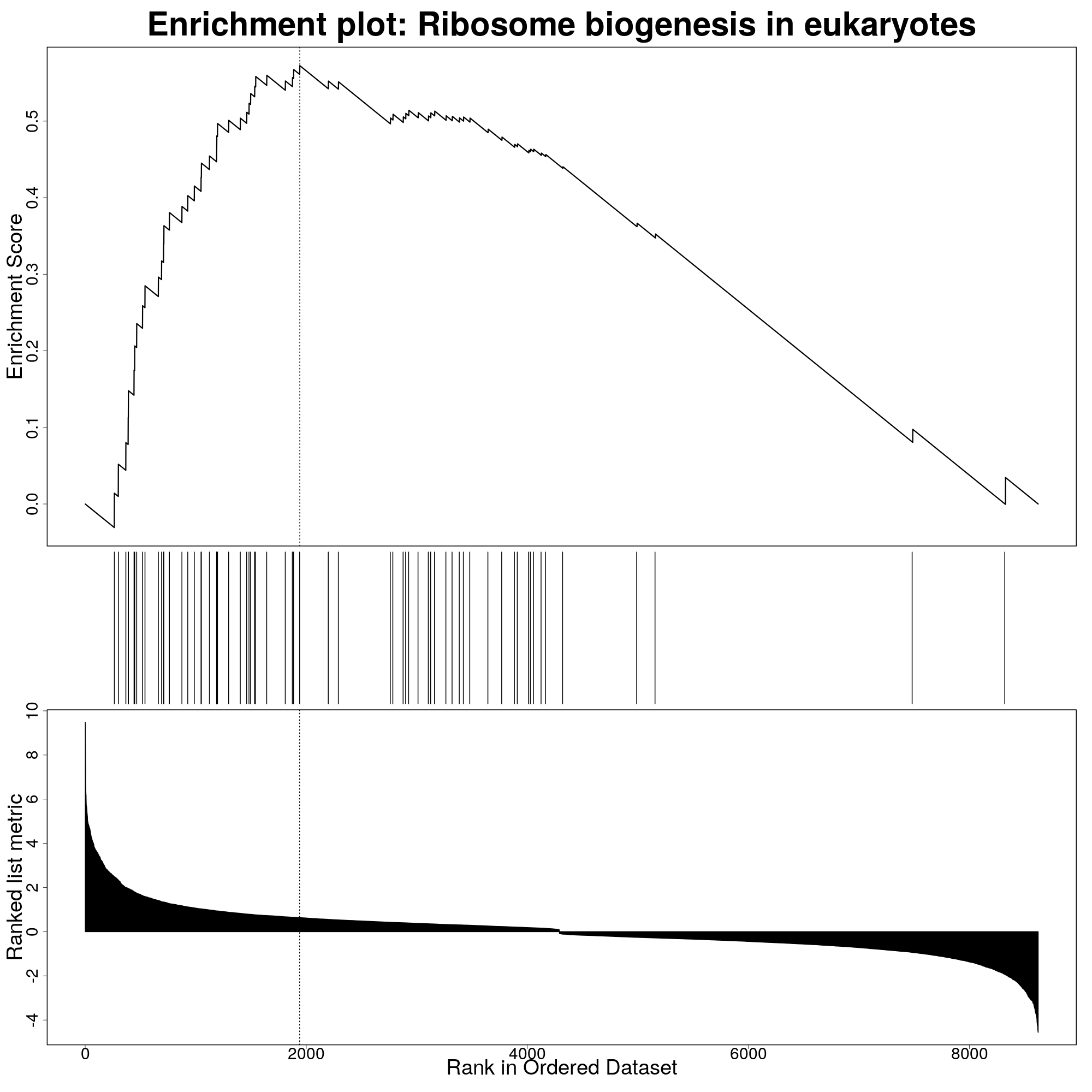

Supplement: Supplementary file 14 [file DataSheet_6.zip › Supplementary data 6 GSEA CCR2lo vs CCR2hi all samples/Project_high_vs_low_GSEA/mmu03008.png]

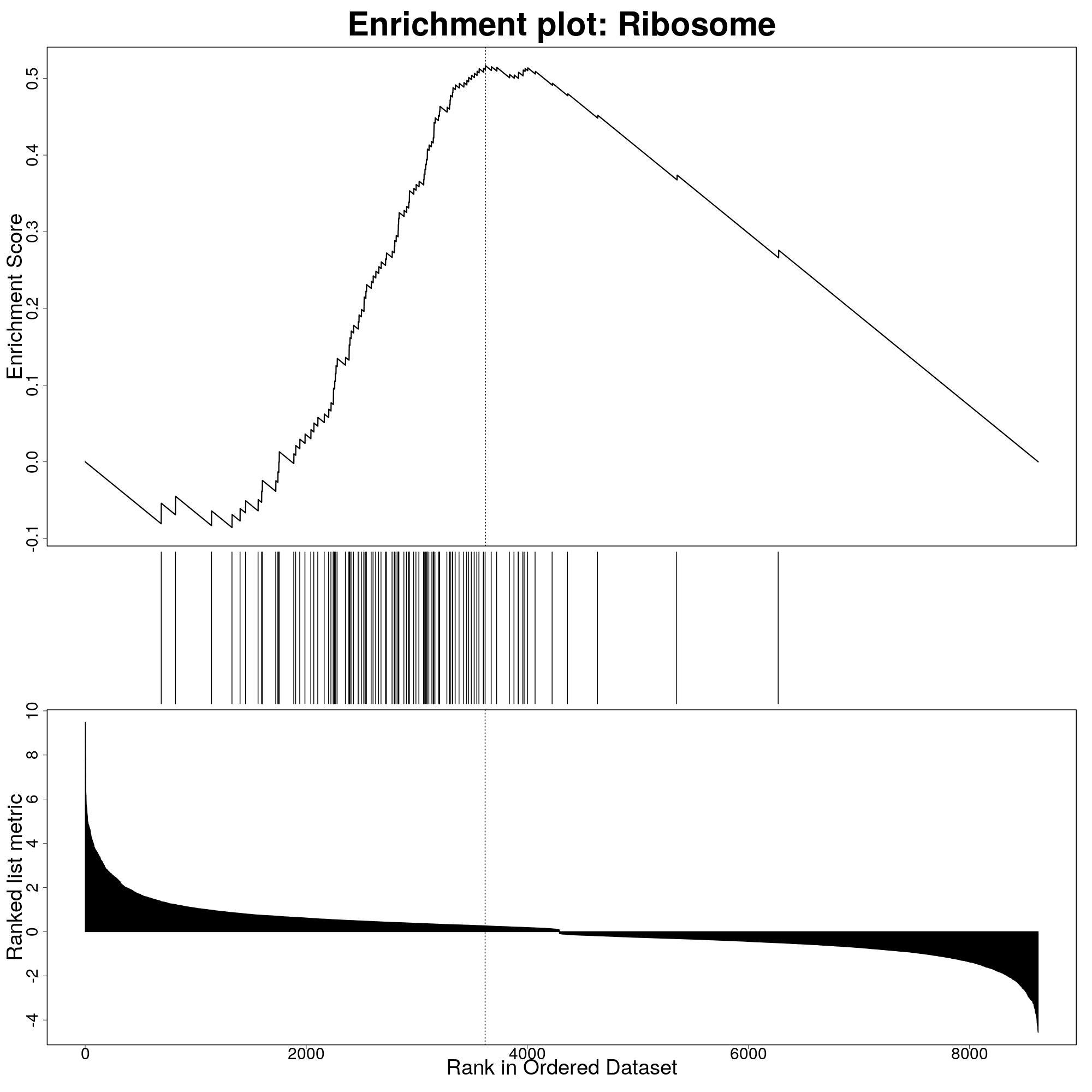

Supplement: Supplementary file 14 [file DataSheet_6.zip › Supplementary data 6 GSEA CCR2lo vs CCR2hi all samples/Project_high_vs_low_GSEA/mmu03010.png]

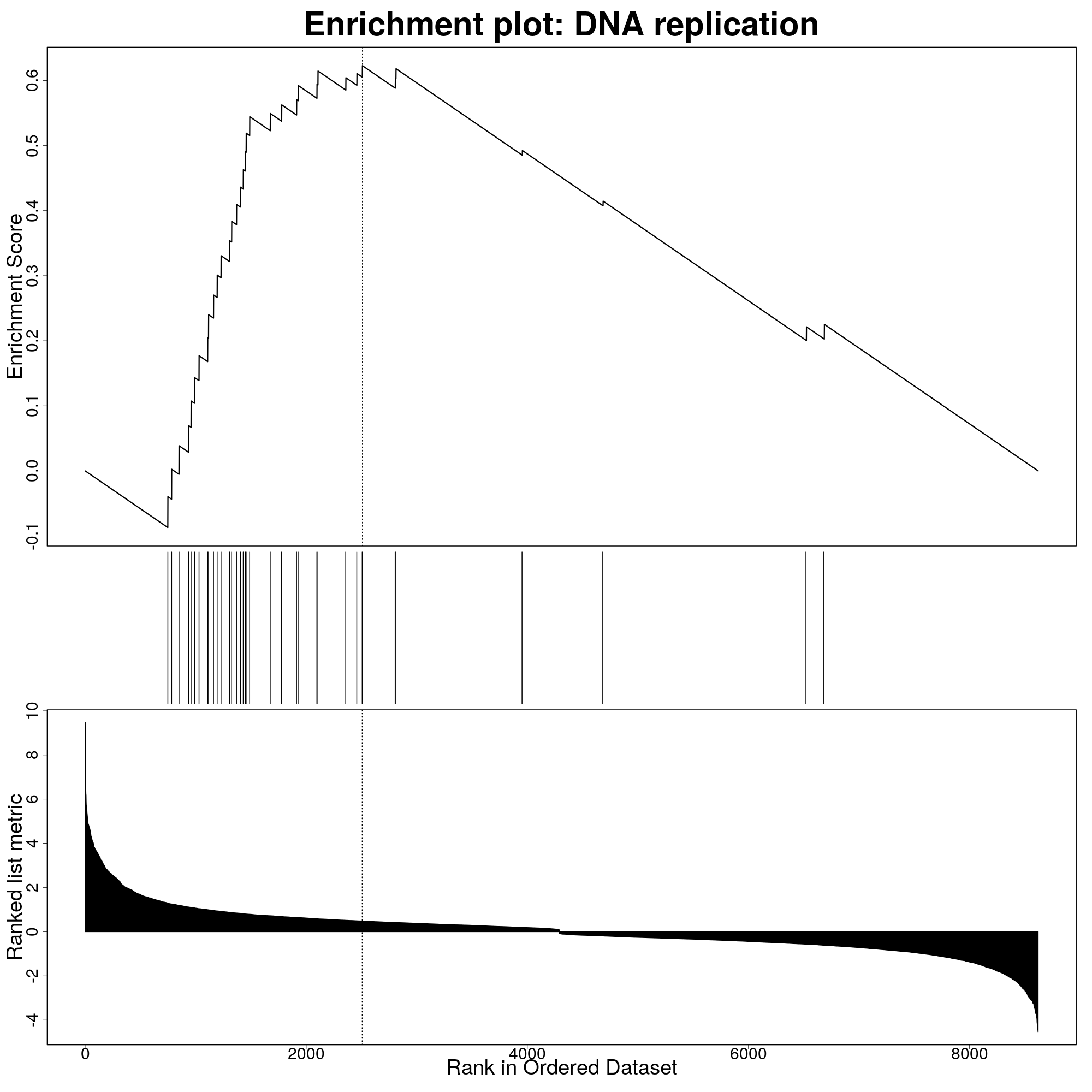

Supplement: Supplementary file 14 [file DataSheet_6.zip › Supplementary data 6 GSEA CCR2lo vs CCR2hi all samples/Project_high_vs_low_GSEA/mmu03030.png]

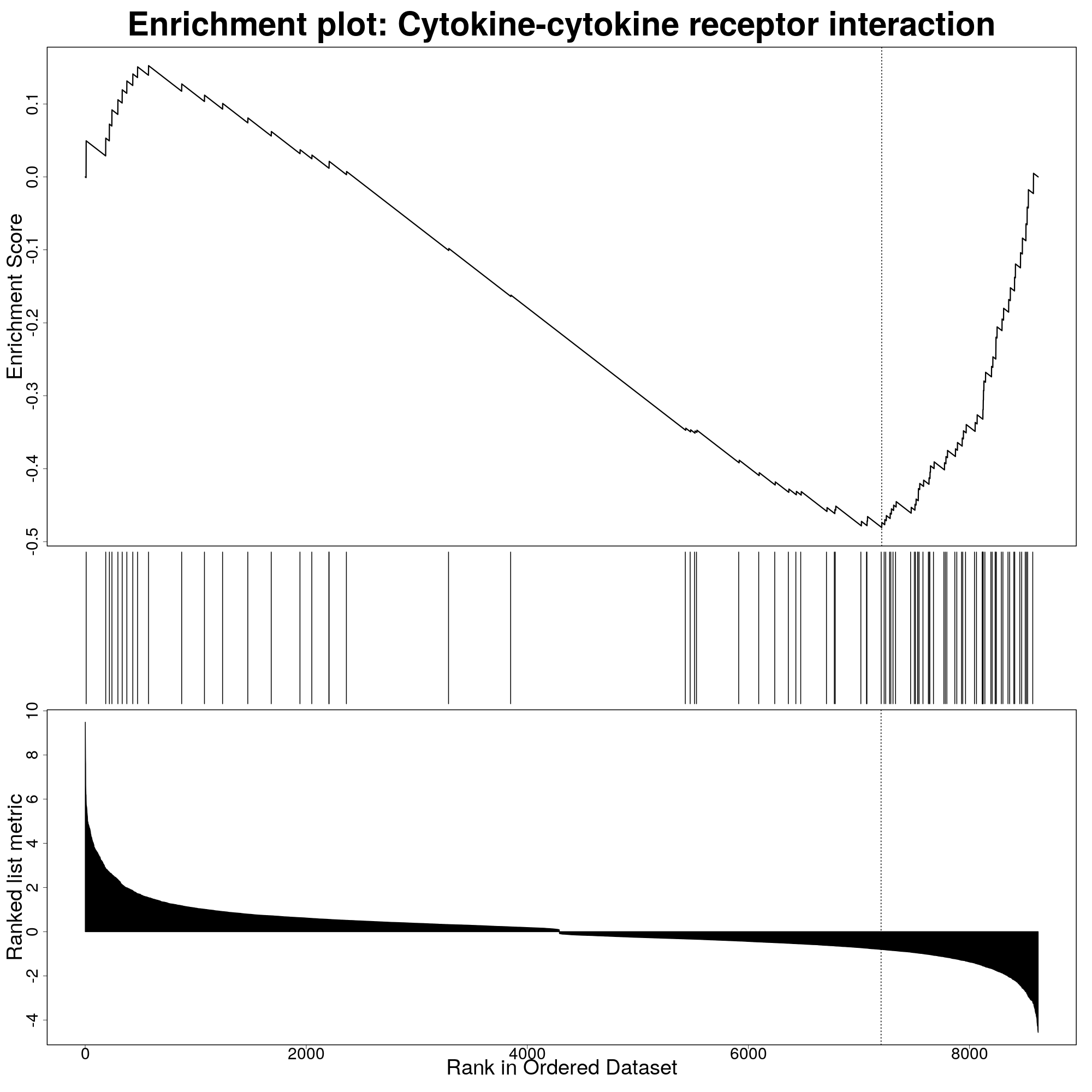

Supplement: Supplementary file 14 [file DataSheet_6.zip › Supplementary data 6 GSEA CCR2lo vs CCR2hi all samples/Project_high_vs_low_GSEA/mmu04060.png]

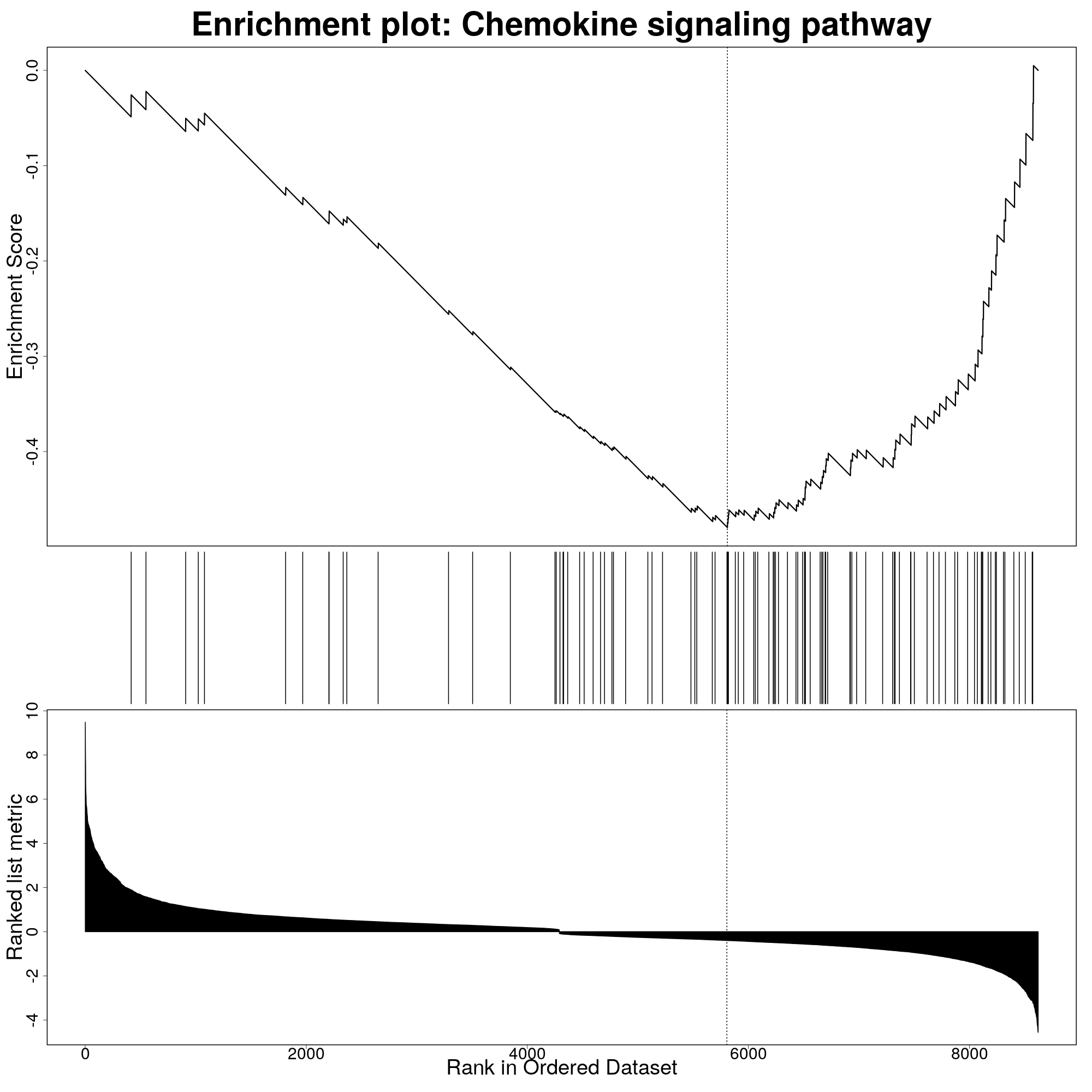

Supplement: Supplementary file 14 [file DataSheet_6.zip › Supplementary data 6 GSEA CCR2lo vs CCR2hi all samples/Project_high_vs_low_GSEA/mmu04062.png]

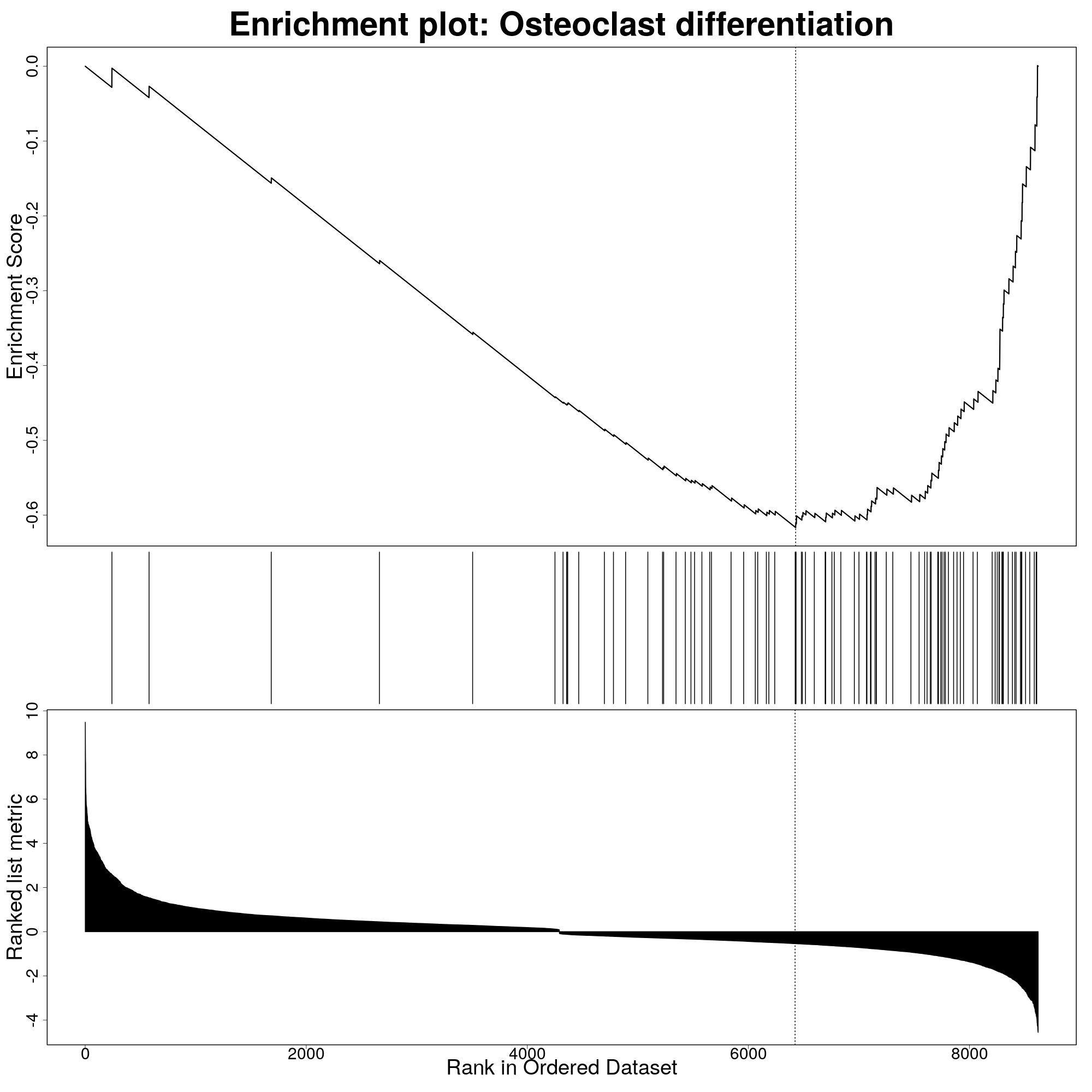

Supplement: Supplementary file 14 [file DataSheet_6.zip › Supplementary data 6 GSEA CCR2lo vs CCR2hi all samples/Project_high_vs_low_GSEA/mmu04380.png]

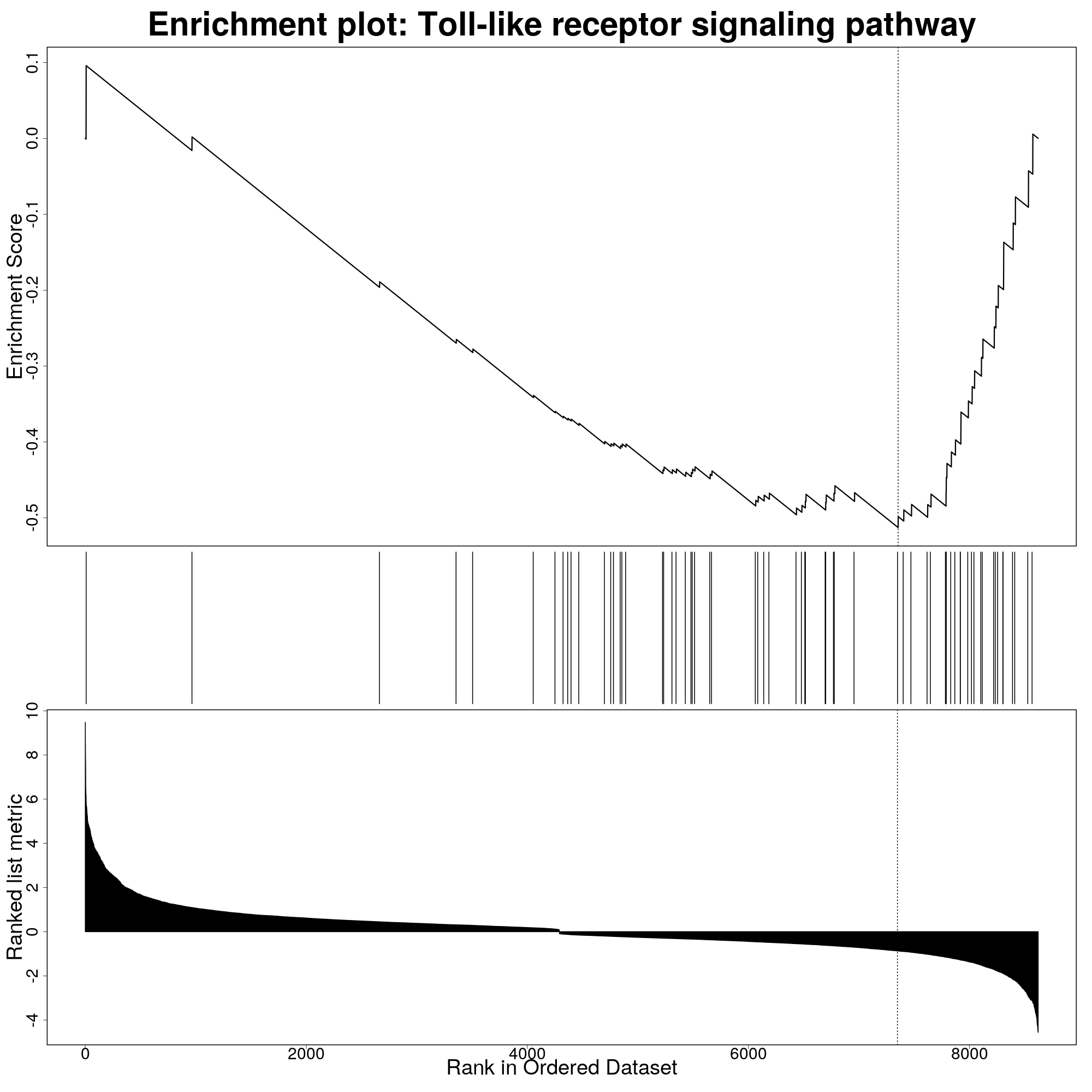

Supplement: Supplementary file 14 [file DataSheet_6.zip › Supplementary data 6 GSEA CCR2lo vs CCR2hi all samples/Project_high_vs_low_GSEA/mmu04620.png]

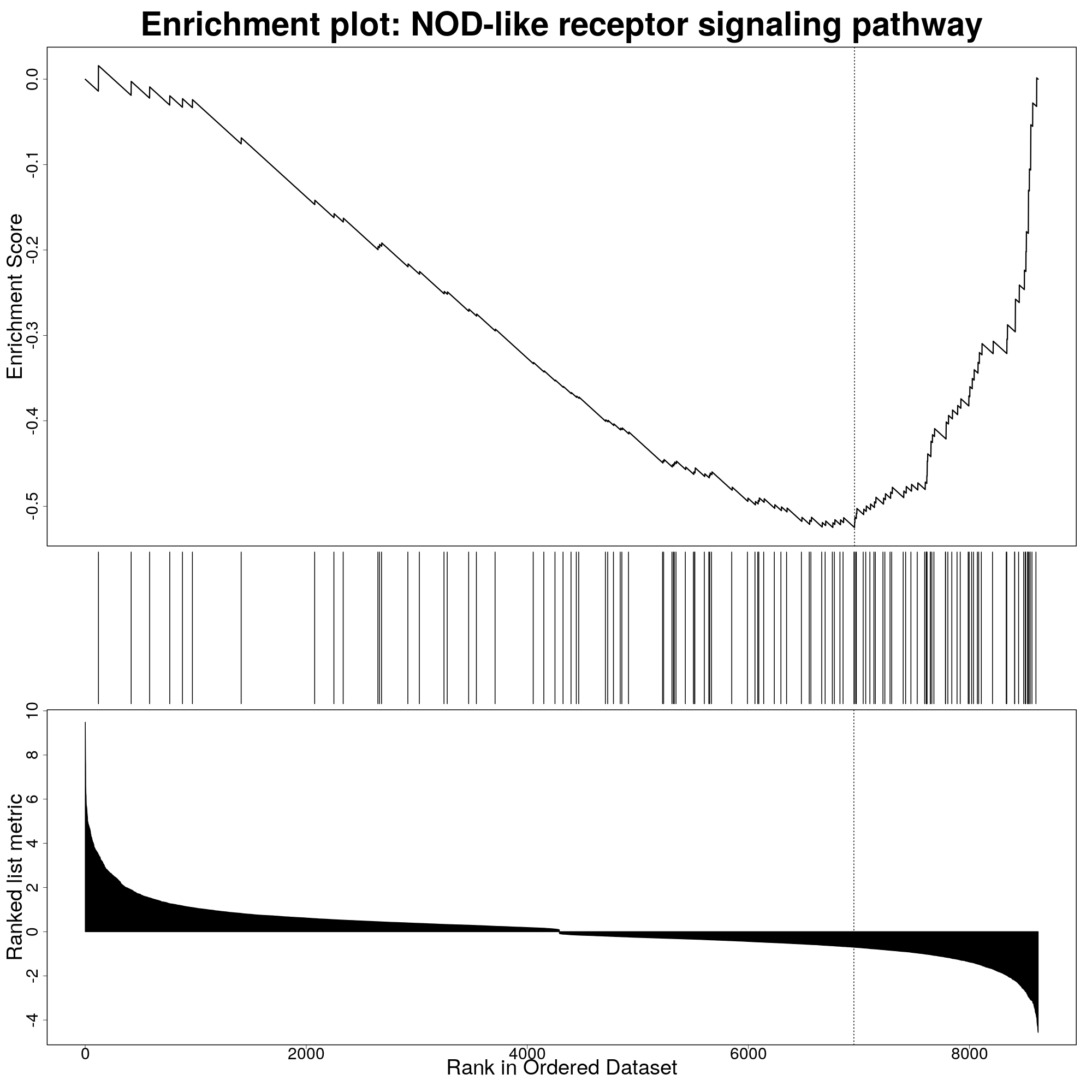

Supplement: Supplementary file 14 [file DataSheet_6.zip › Supplementary data 6 GSEA CCR2lo vs CCR2hi all samples/Project_high_vs_low_GSEA/mmu04621.png]

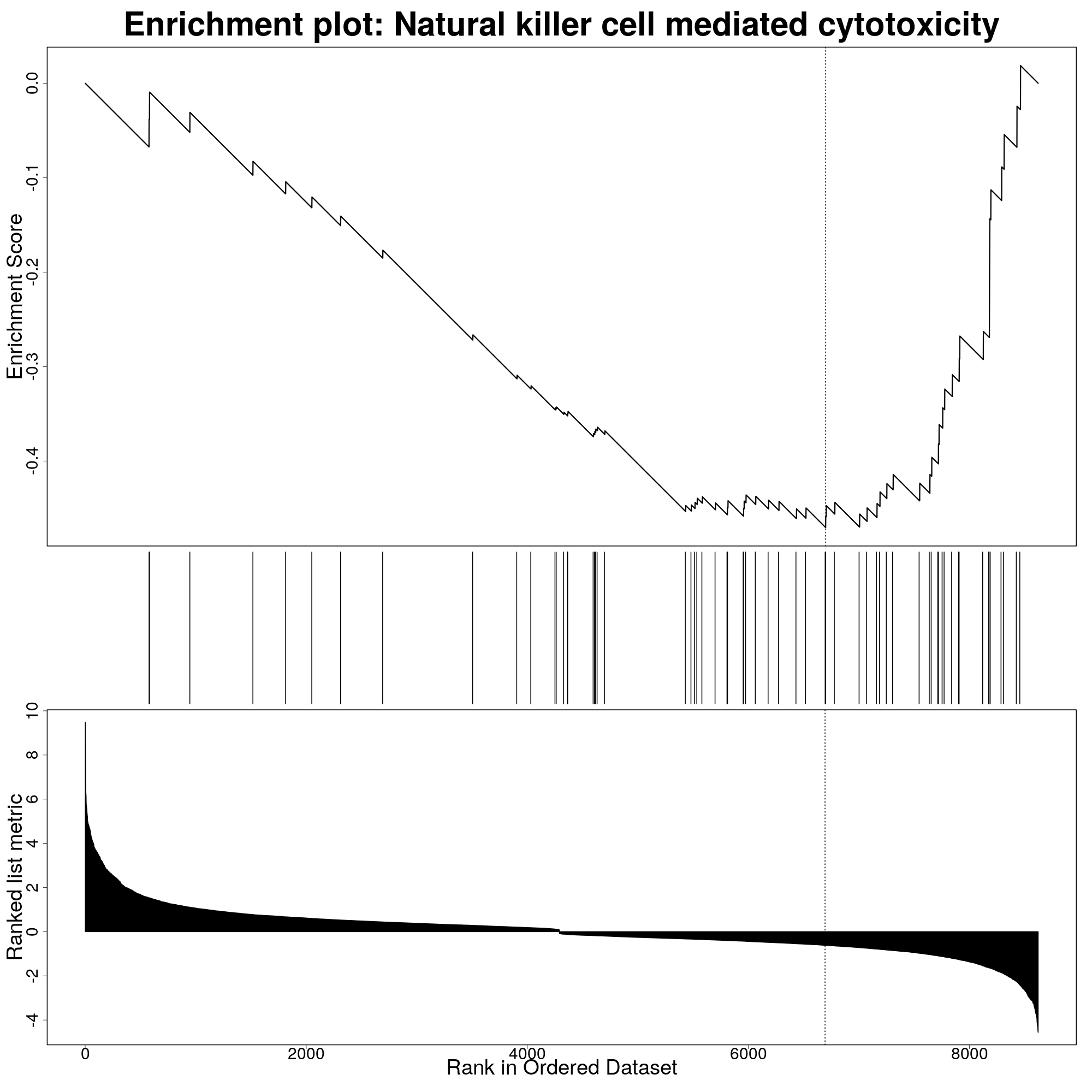

Supplement: Supplementary file 14 [file DataSheet_6.zip › Supplementary data 6 GSEA CCR2lo vs CCR2hi all samples/Project_high_vs_low_GSEA/mmu04650.png]

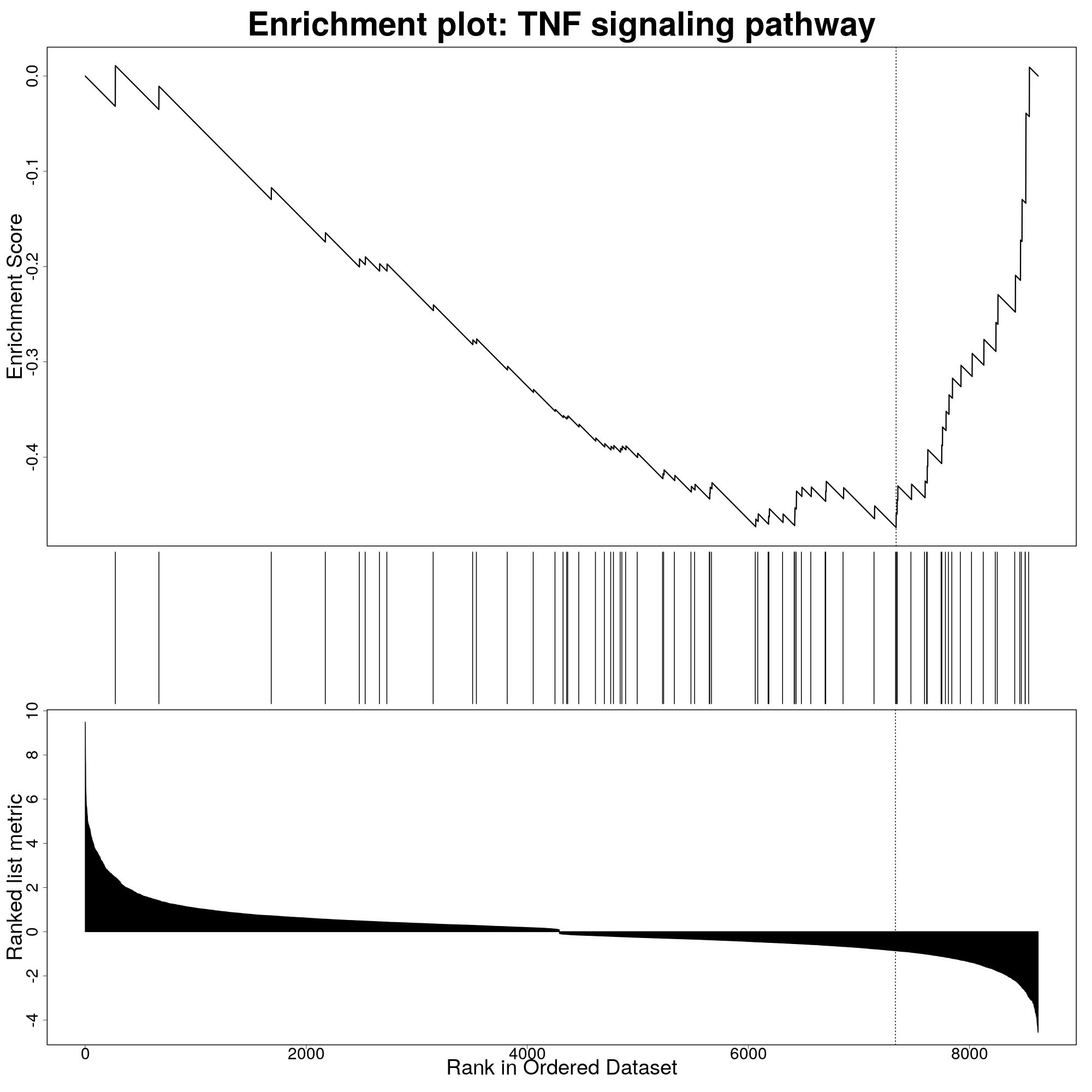

Supplement: Supplementary file 14 [file DataSheet_6.zip › Supplementary data 6 GSEA CCR2lo vs CCR2hi all samples/Project_high_vs_low_GSEA/mmu04668.png]

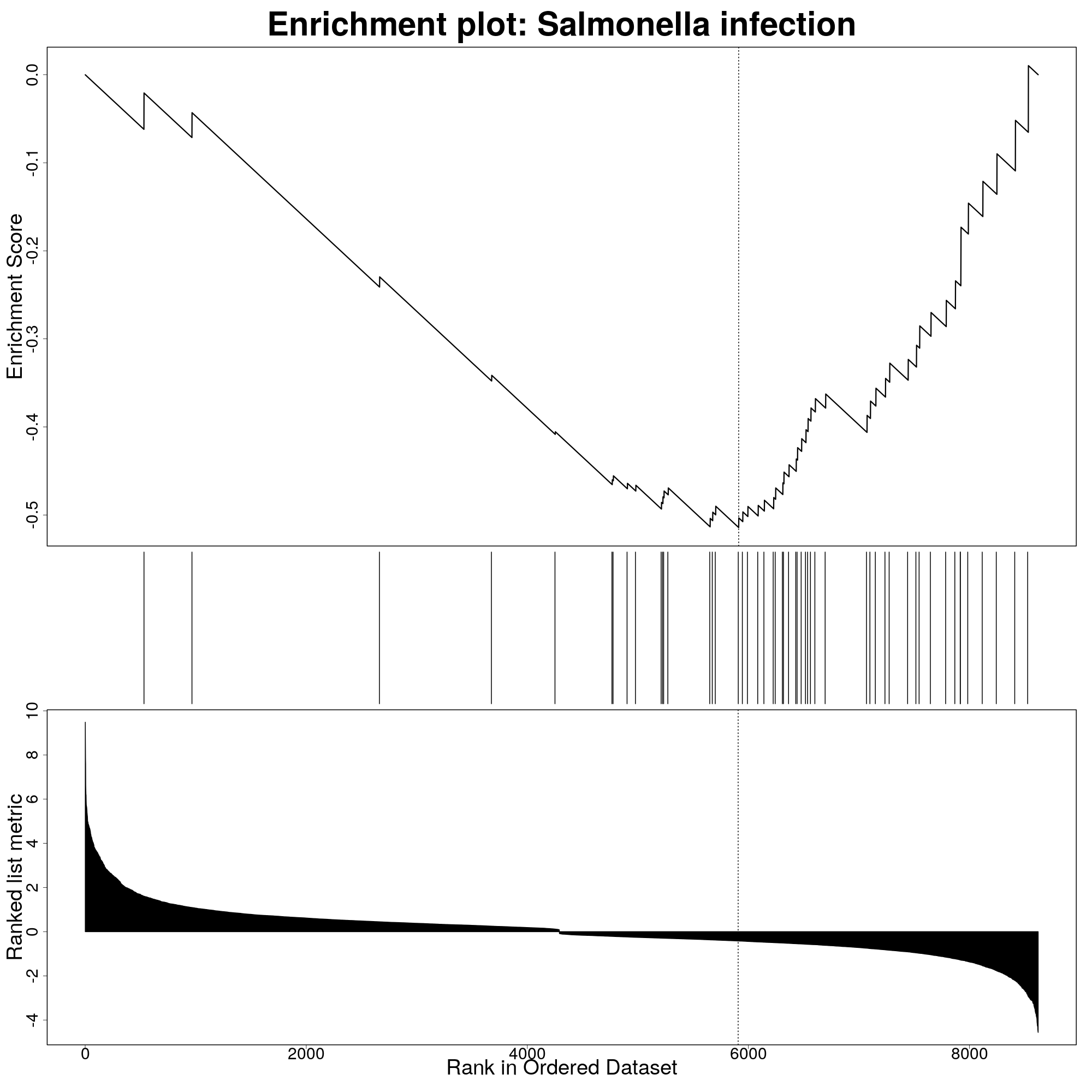

Supplement: Supplementary file 14 [file DataSheet_6.zip › Supplementary data 6 GSEA CCR2lo vs CCR2hi all samples/Project_high_vs_low_GSEA/mmu05132.png]

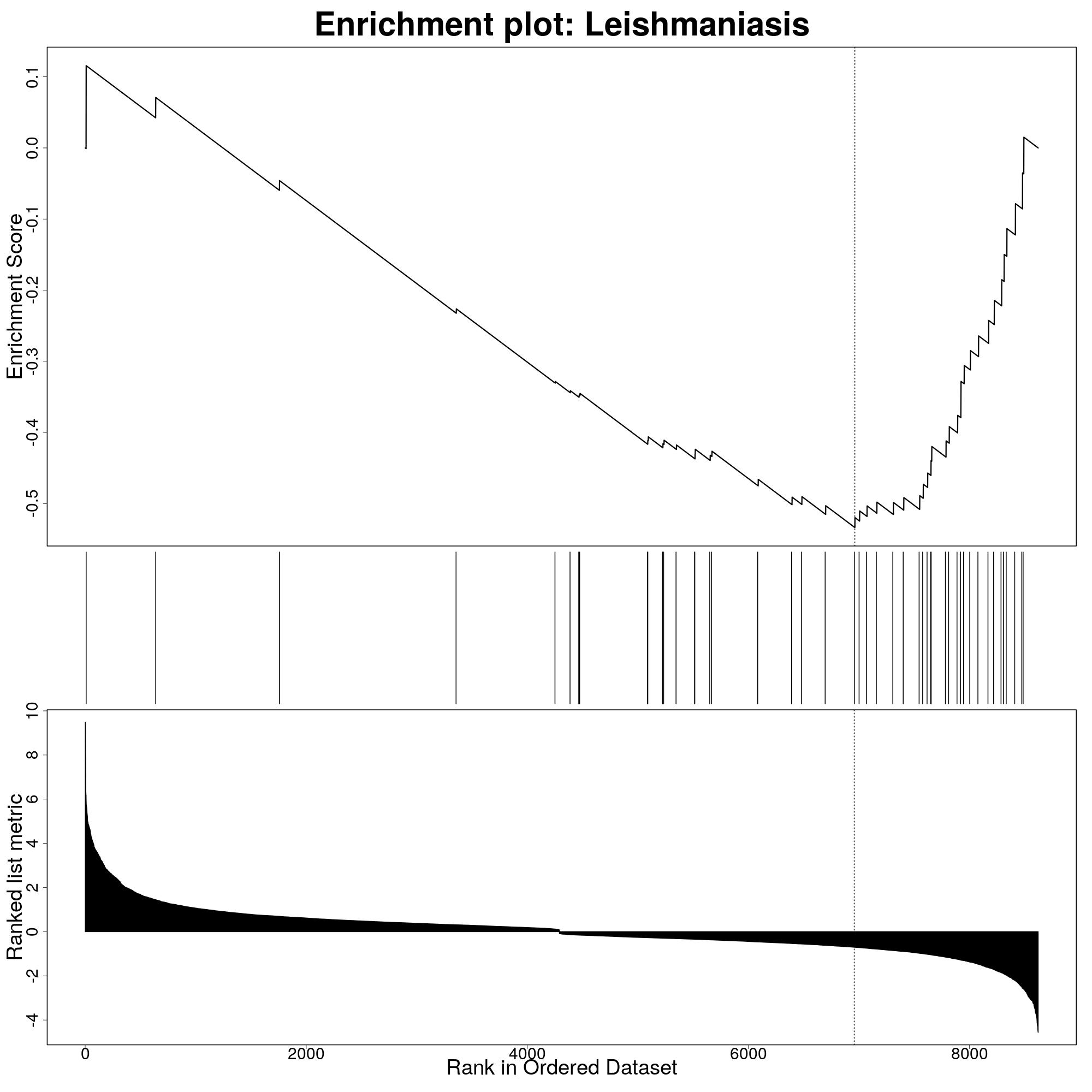

Supplement: Supplementary file 14 [file DataSheet_6.zip › Supplementary data 6 GSEA CCR2lo vs CCR2hi all samples/Project_high_vs_low_GSEA/mmu05140.png]

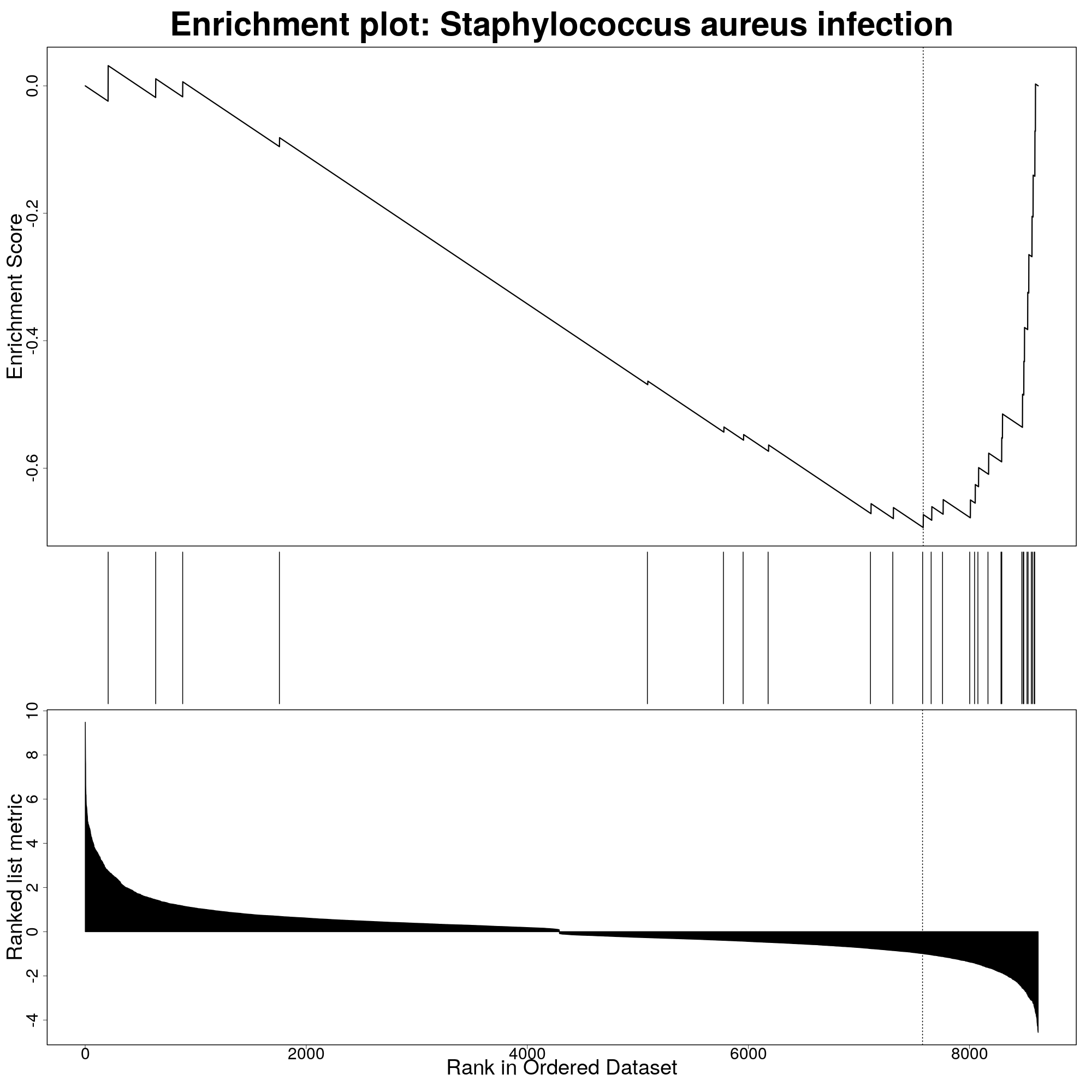

Supplement: Supplementary file 14 [file DataSheet_6.zip › Supplementary data 6 GSEA CCR2lo vs CCR2hi all samples/Project_high_vs_low_GSEA/mmu05150.png]

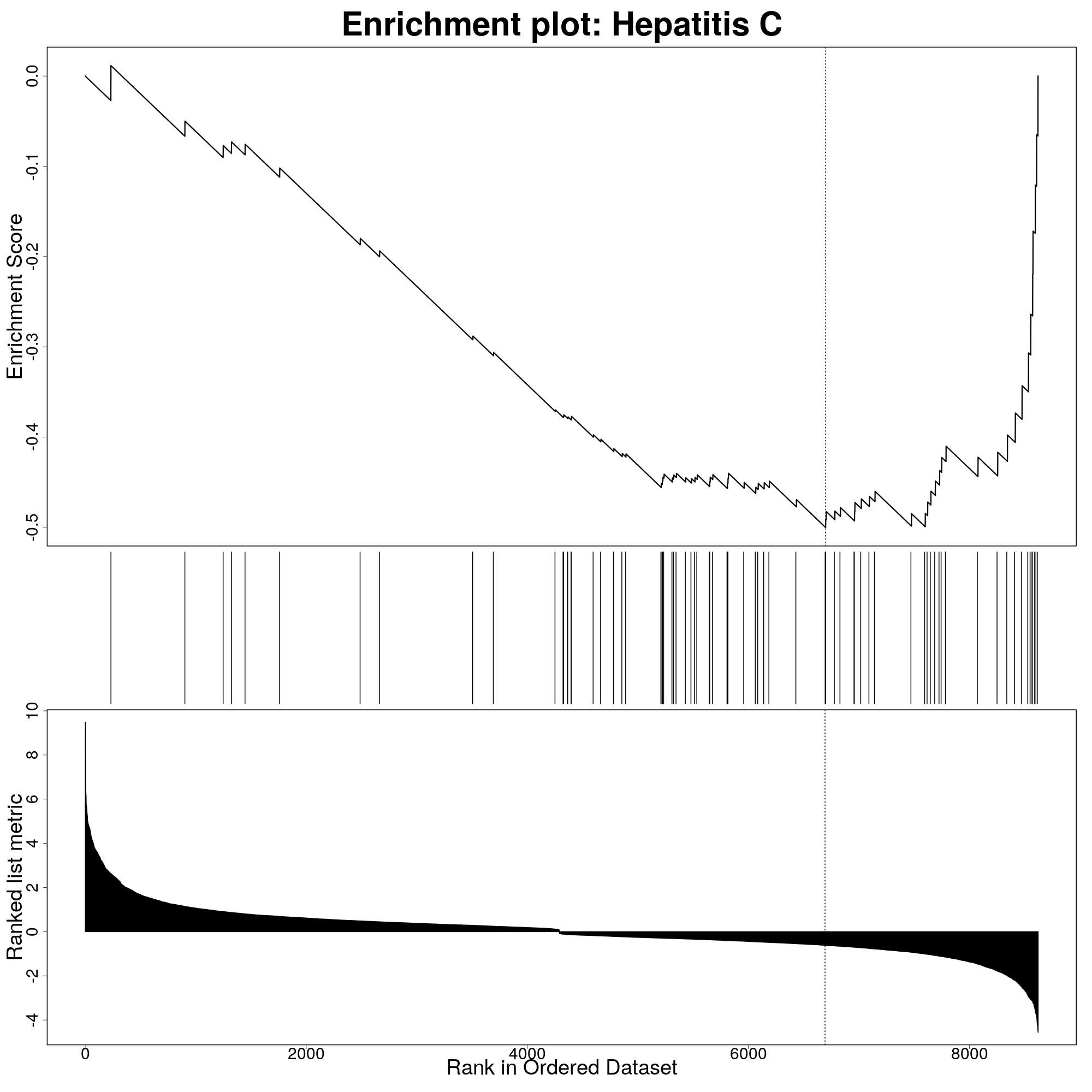

Supplement: Supplementary file 14 [file DataSheet_6.zip › Supplementary data 6 GSEA CCR2lo vs CCR2hi all samples/Project_high_vs_low_GSEA/mmu05160.png]

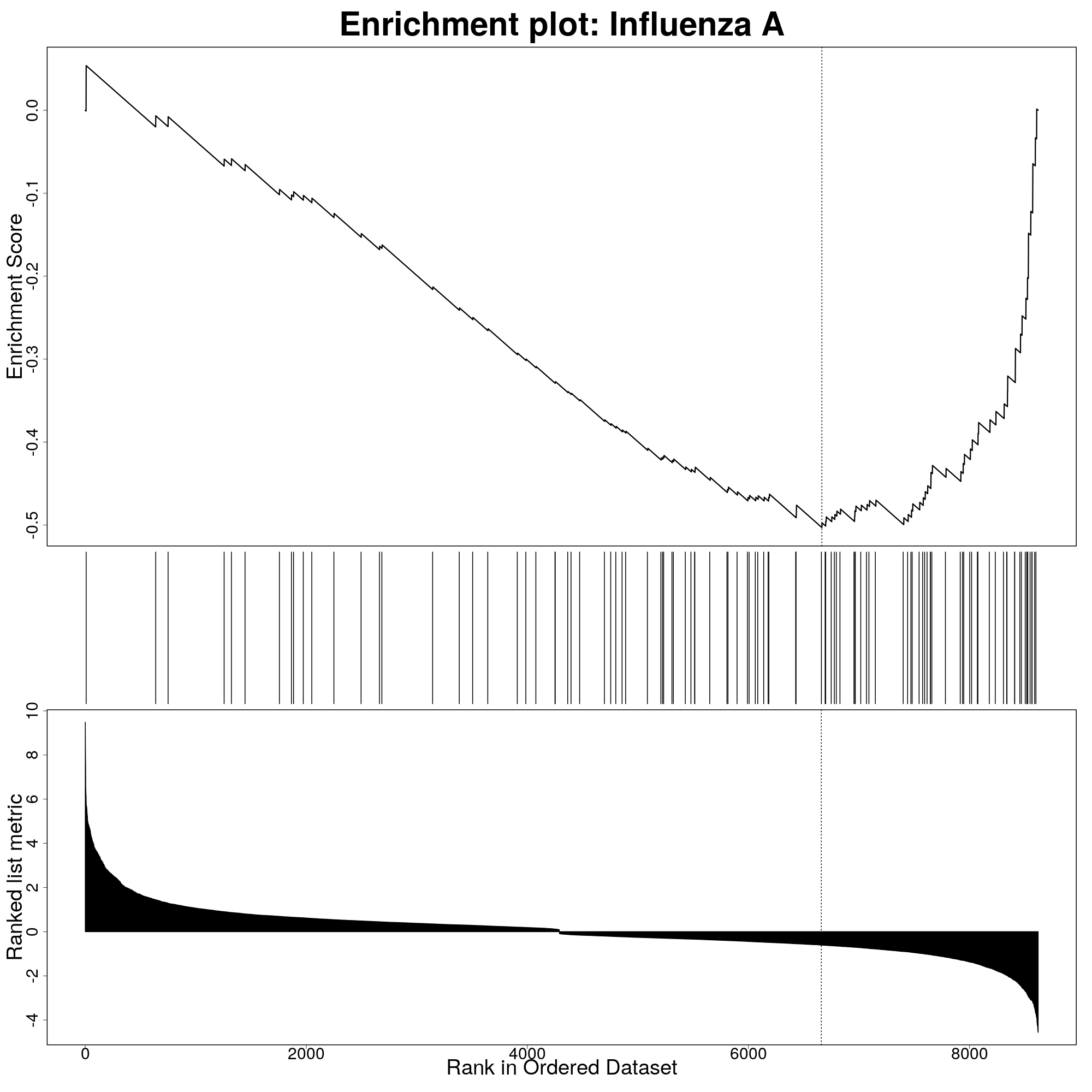

Supplement: Supplementary file 14 [file DataSheet_6.zip › Supplementary data 6 GSEA CCR2lo vs CCR2hi all samples/Project_high_vs_low_GSEA/mmu05164.png]

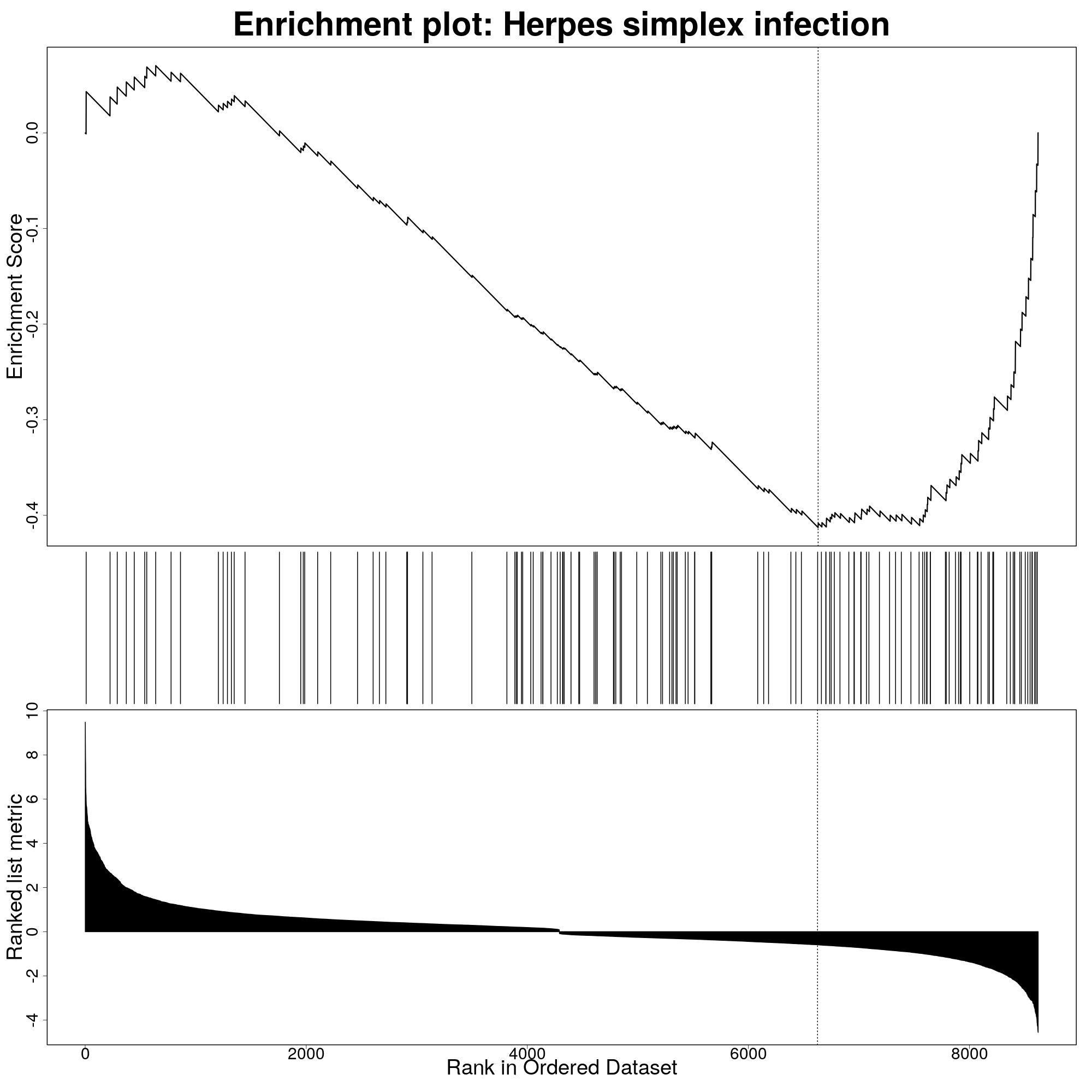

Supplement: Supplementary file 14 [file DataSheet_6.zip › Supplementary data 6 GSEA CCR2lo vs CCR2hi all samples/Project_high_vs_low_GSEA/mmu05168.png]

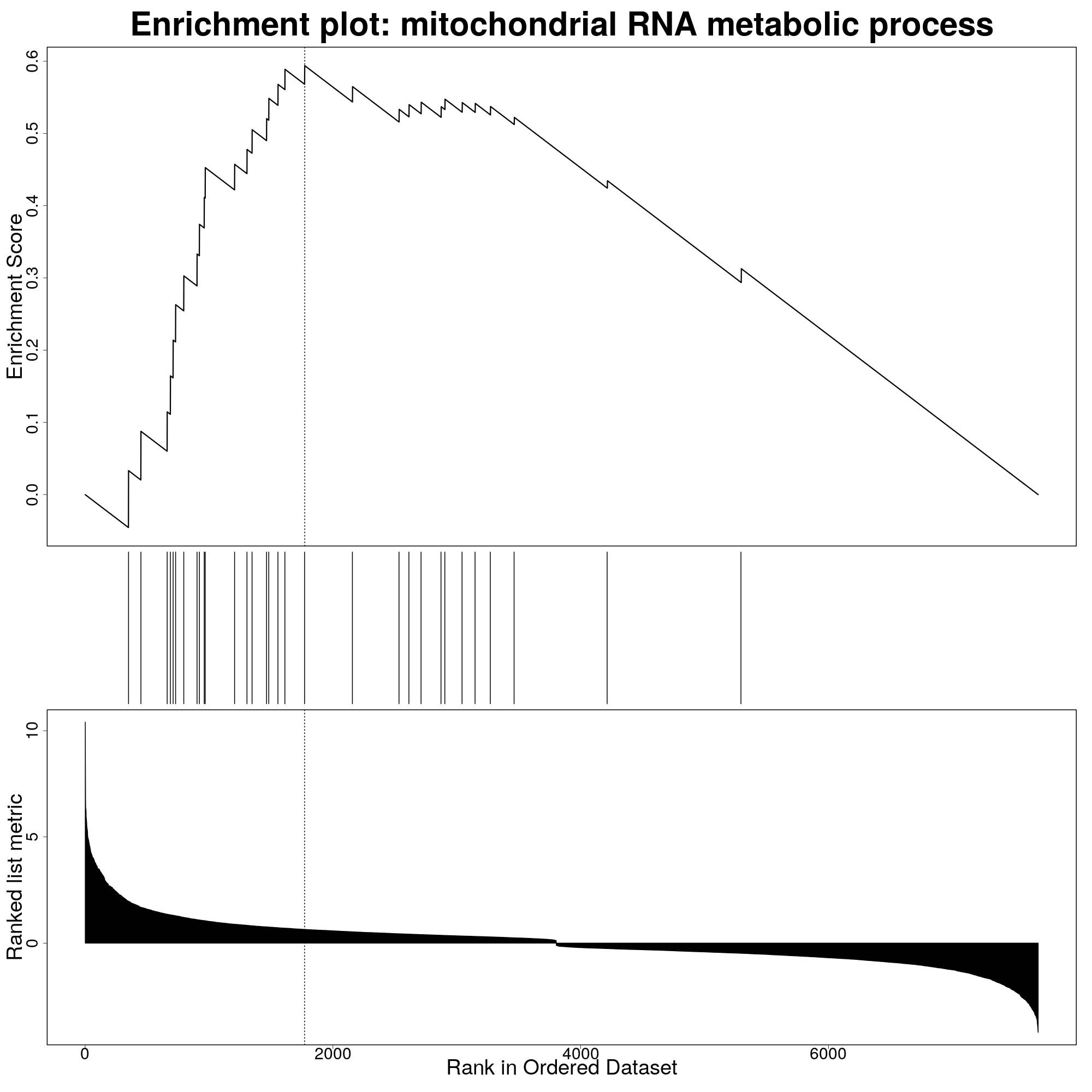

Supplement: Supplementary file 15 [file DataSheet_7.zip › Supplementary data 7 GSEA CCR2lo vs CCR2hi in CIA/Project_high_vs_low_GSEA/GO_0000959.png]

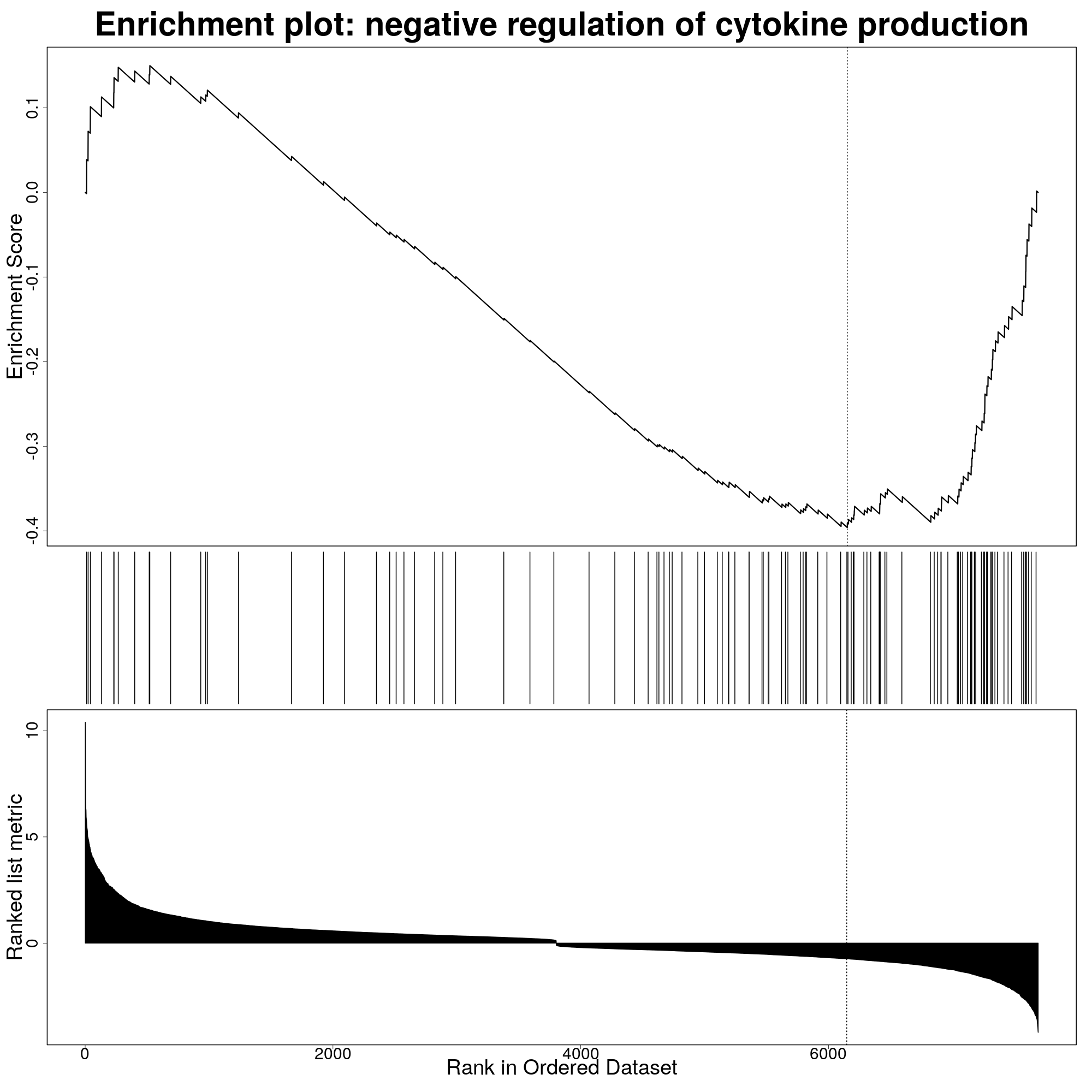

Supplement: Supplementary file 15 [file DataSheet_7.zip › Supplementary data 7 GSEA CCR2lo vs CCR2hi in CIA/Project_high_vs_low_GSEA/GO_0001818.png]

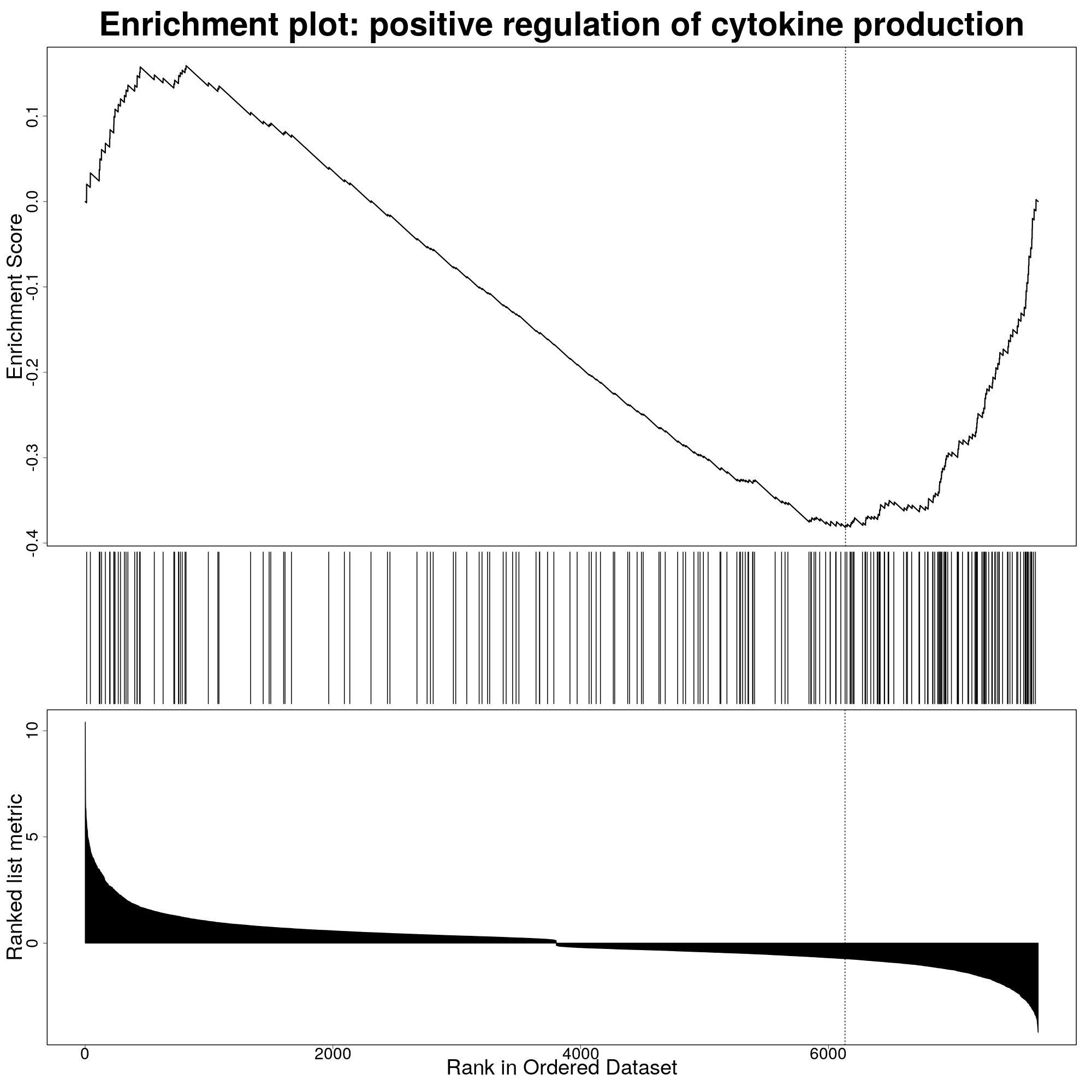

Supplement: Supplementary file 15 [file DataSheet_7.zip › Supplementary data 7 GSEA CCR2lo vs CCR2hi in CIA/Project_high_vs_low_GSEA/GO_0001819.png]

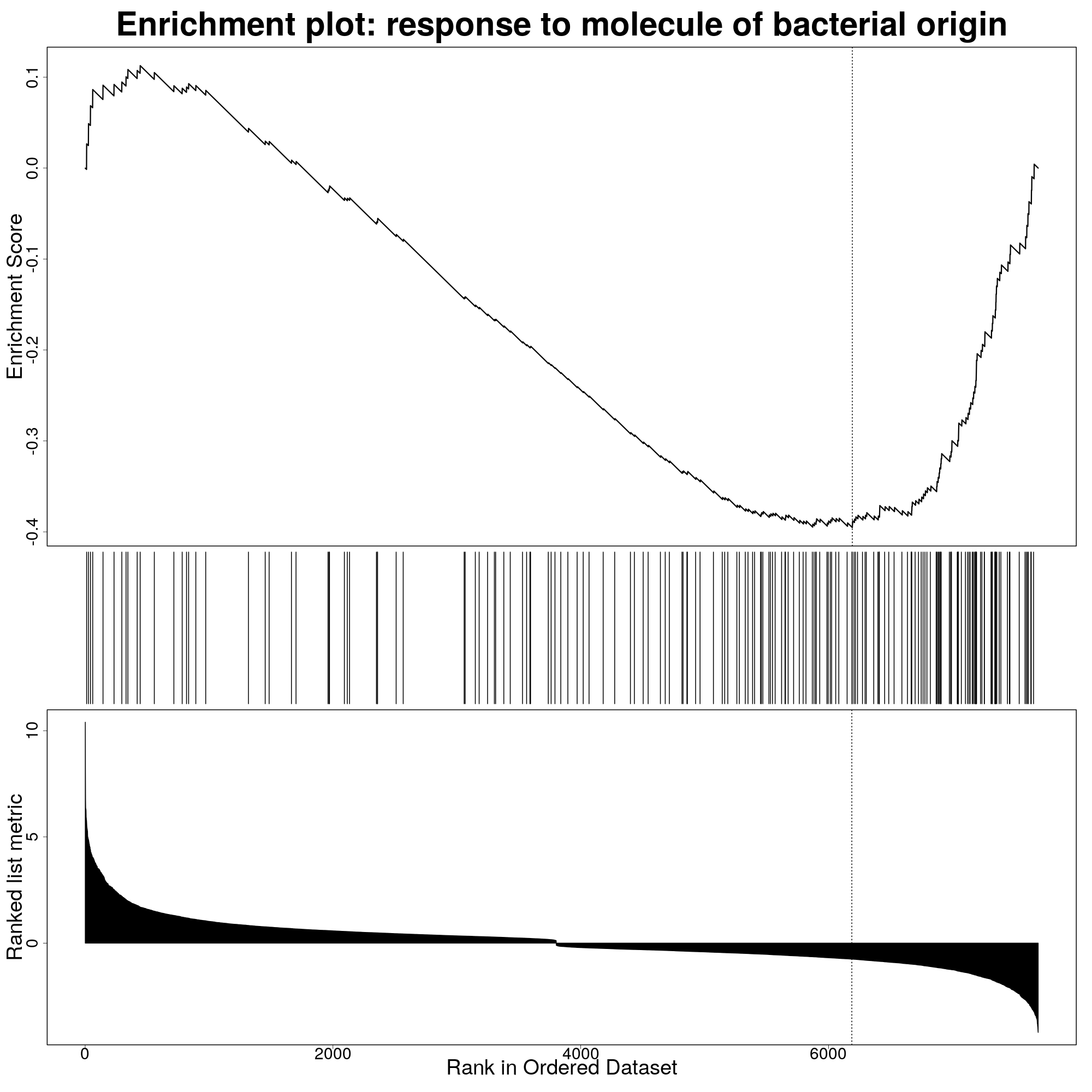

Supplement: Supplementary file 15 [file DataSheet_7.zip › Supplementary data 7 GSEA CCR2lo vs CCR2hi in CIA/Project_high_vs_low_GSEA/GO_0002237.png]

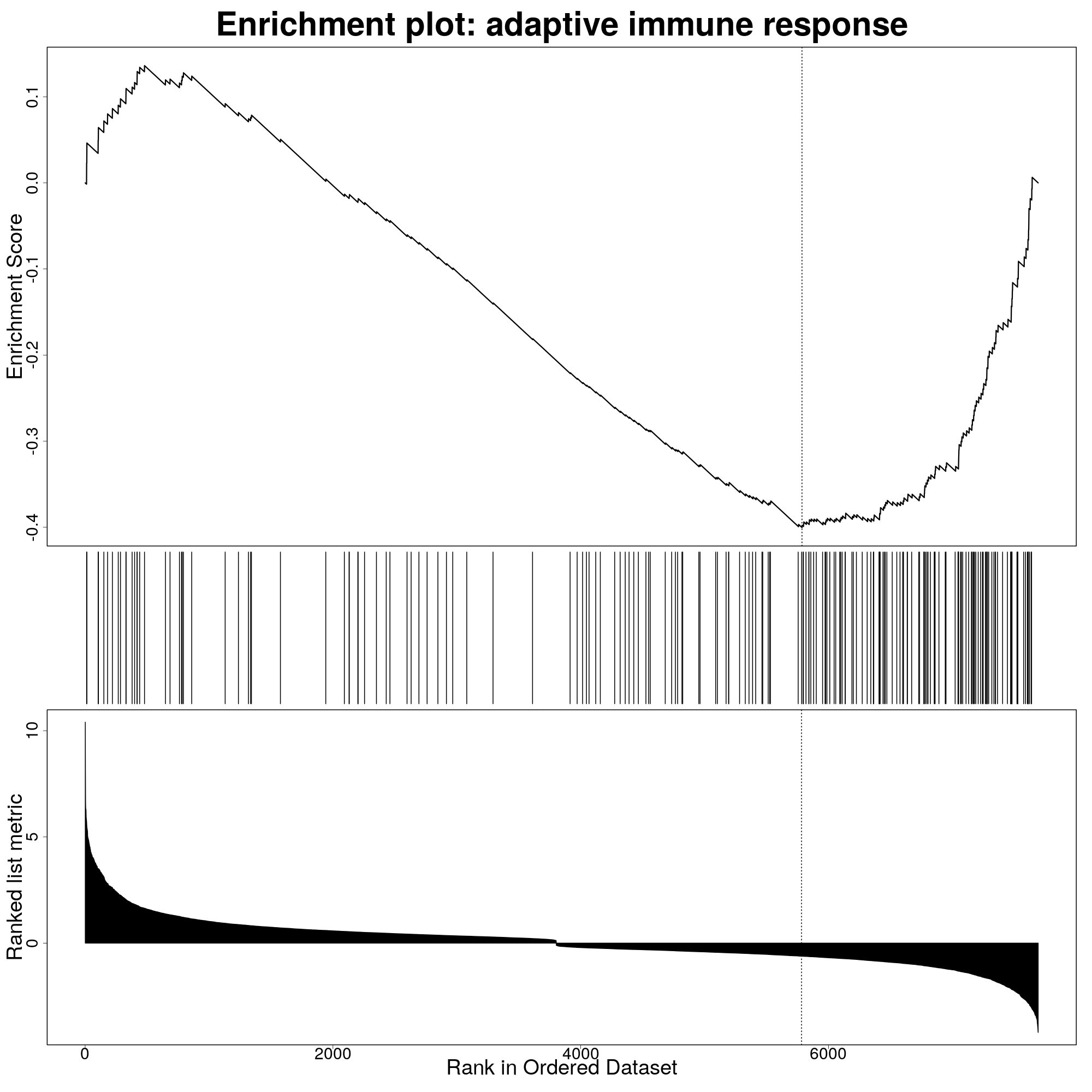

Supplement: Supplementary file 15 [file DataSheet_7.zip › Supplementary data 7 GSEA CCR2lo vs CCR2hi in CIA/Project_high_vs_low_GSEA/GO_0002250.png]

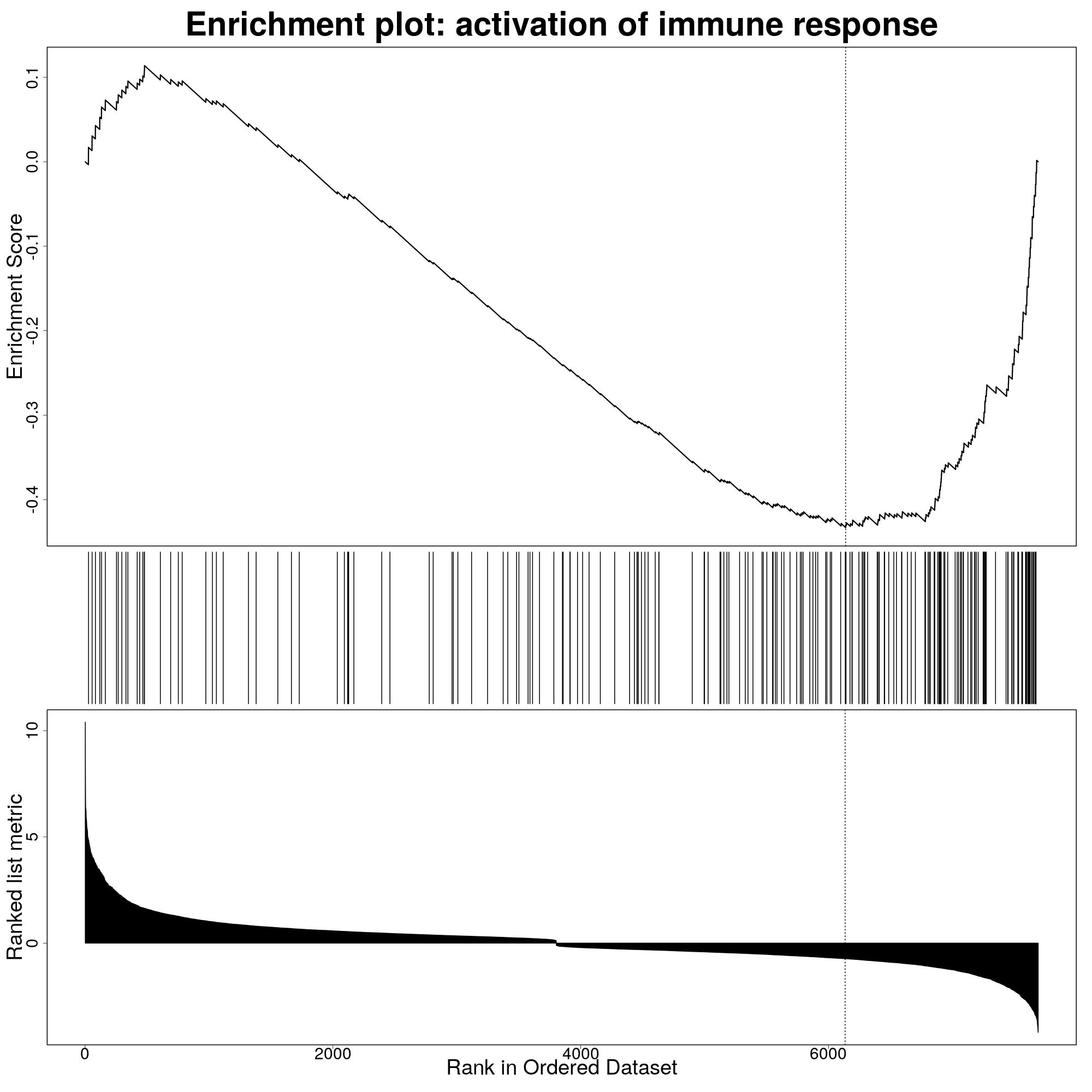

Supplement: Supplementary file 15 [file DataSheet_7.zip › Supplementary data 7 GSEA CCR2lo vs CCR2hi in CIA/Project_high_vs_low_GSEA/GO_0002253.png]

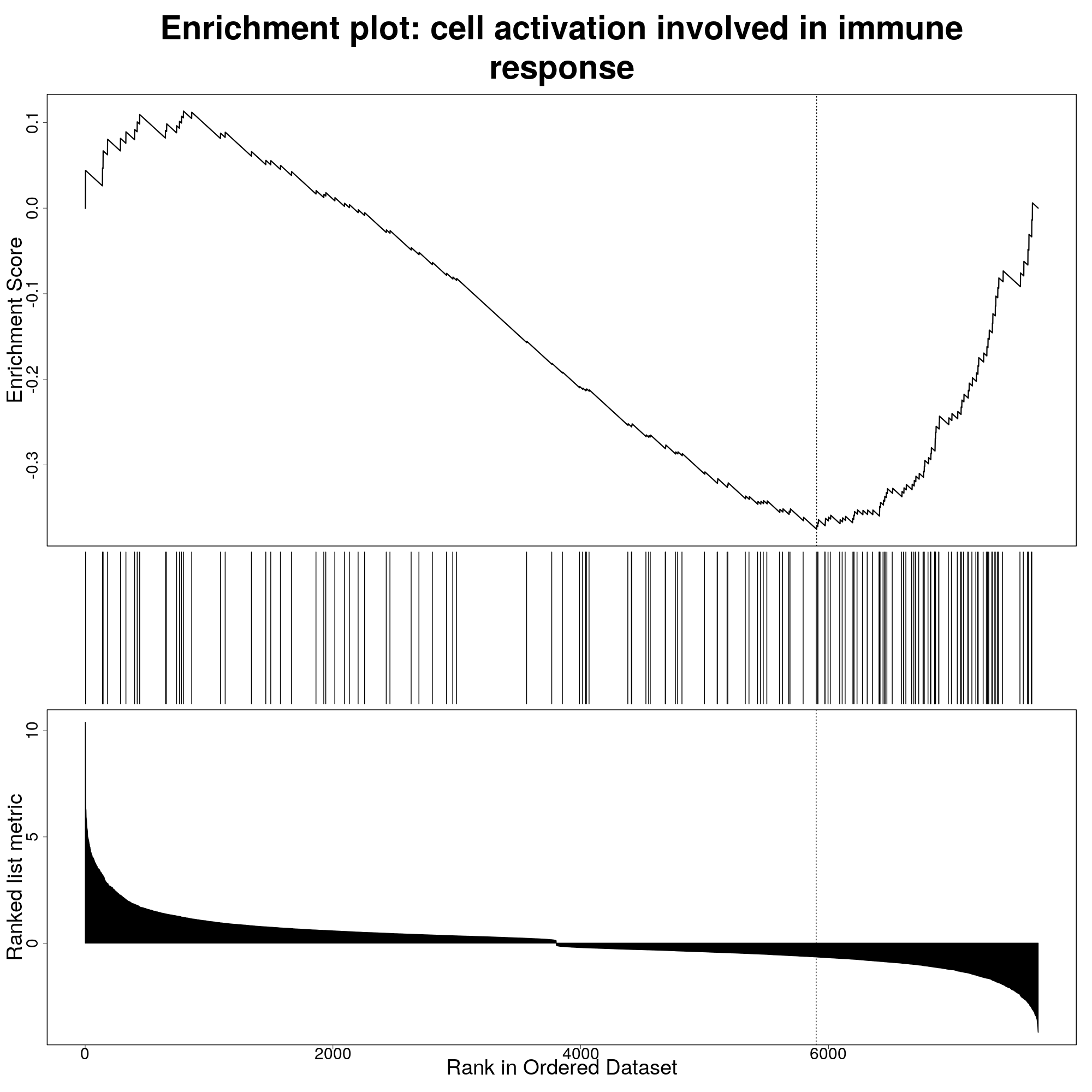

Supplement: Supplementary file 15 [file DataSheet_7.zip › Supplementary data 7 GSEA CCR2lo vs CCR2hi in CIA/Project_high_vs_low_GSEA/GO_0002263.png]

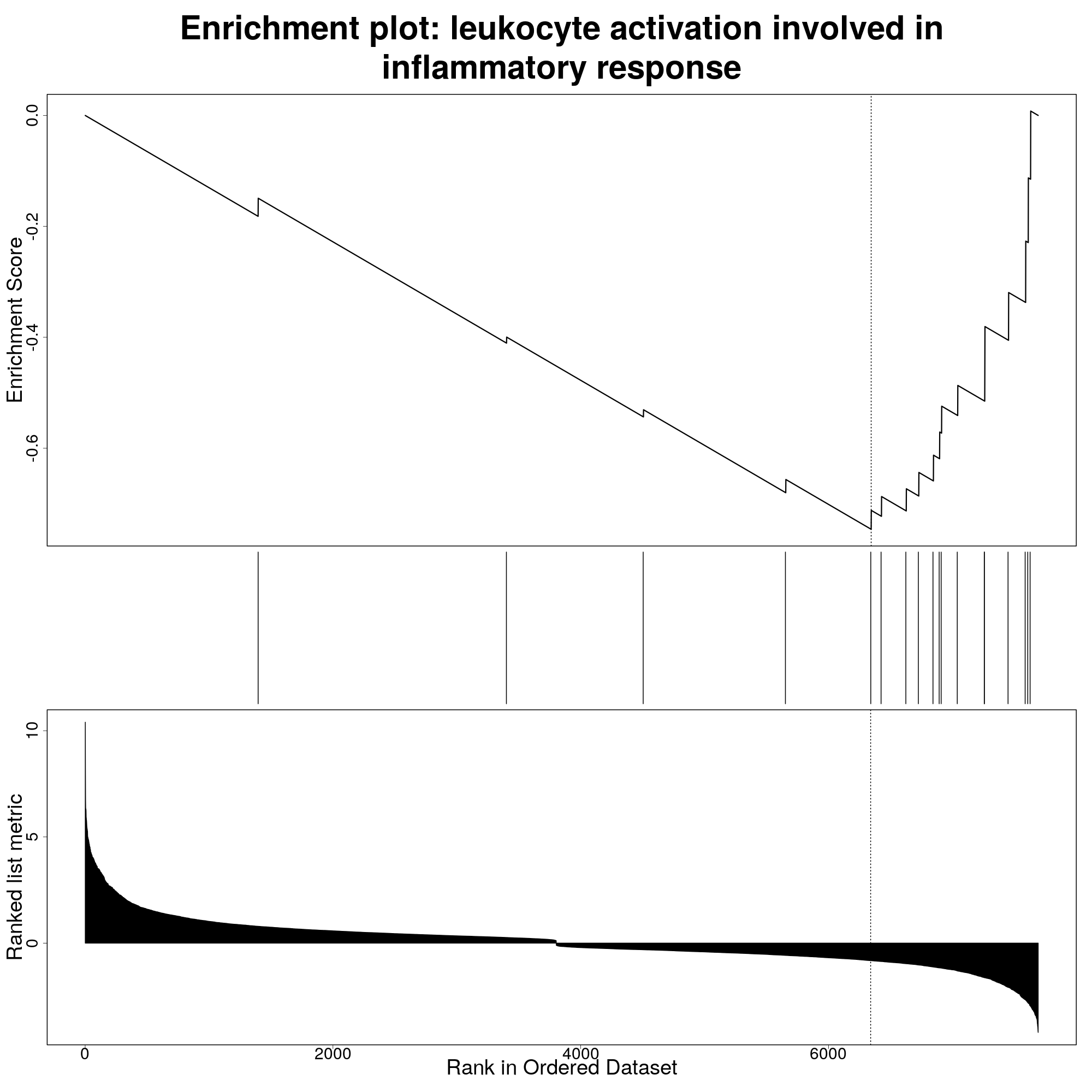

Supplement: Supplementary file 15 [file DataSheet_7.zip › Supplementary data 7 GSEA CCR2lo vs CCR2hi in CIA/Project_high_vs_low_GSEA/GO_0002269.png]

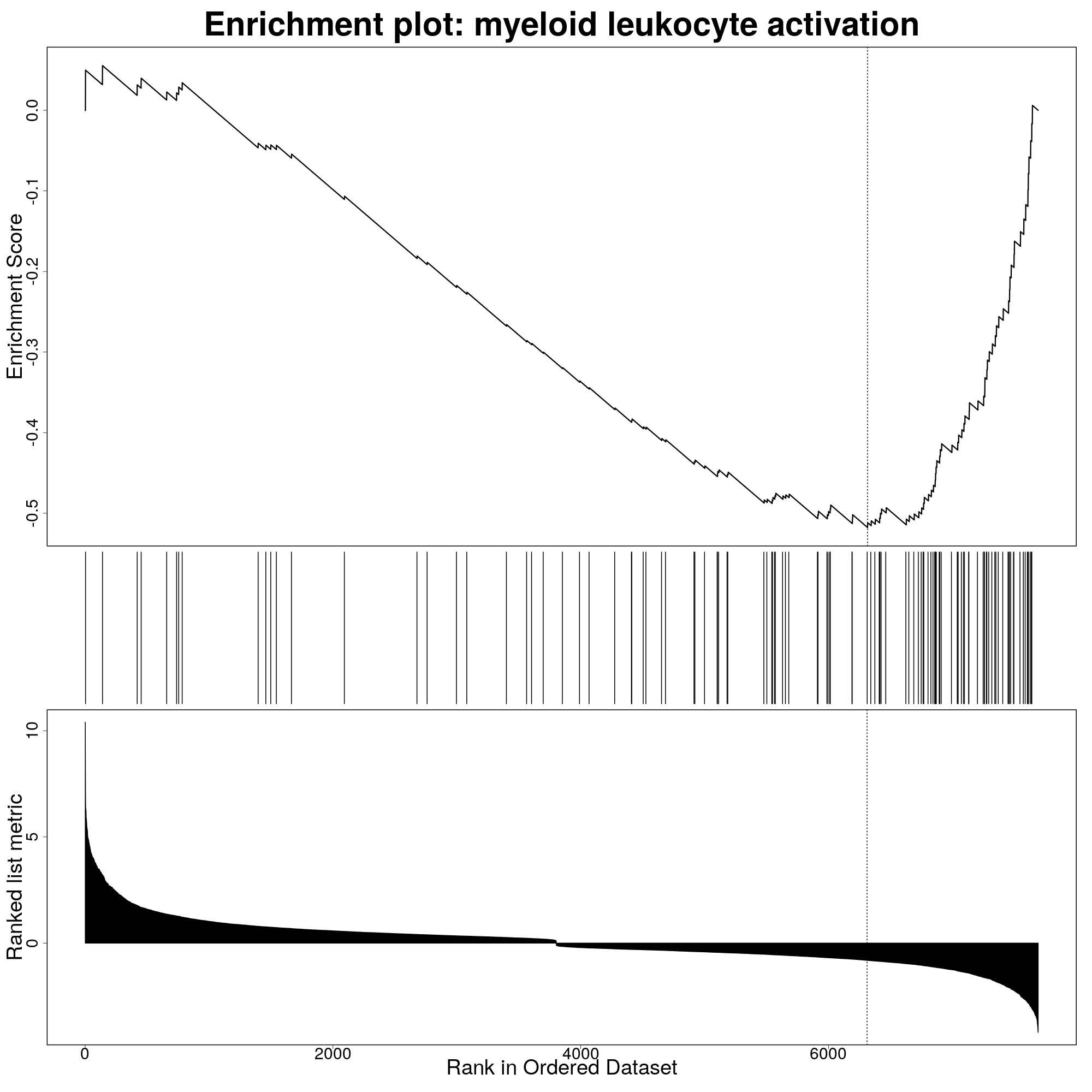

Supplement: Supplementary file 15 [file DataSheet_7.zip › Supplementary data 7 GSEA CCR2lo vs CCR2hi in CIA/Project_high_vs_low_GSEA/GO_0002274.png]

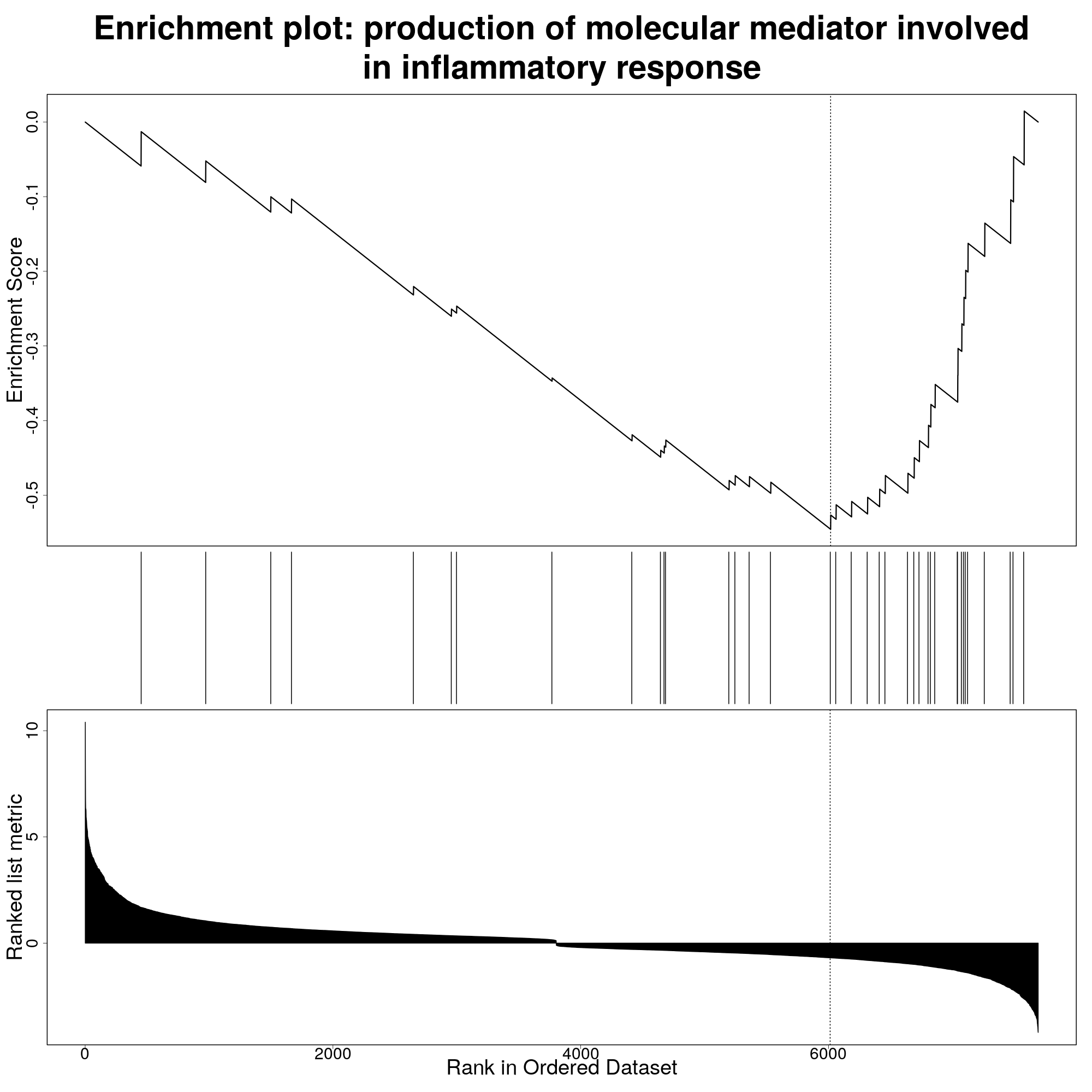

Supplement: Supplementary file 15 [file DataSheet_7.zip › Supplementary data 7 GSEA CCR2lo vs CCR2hi in CIA/Project_high_vs_low_GSEA/GO_0002532.png]

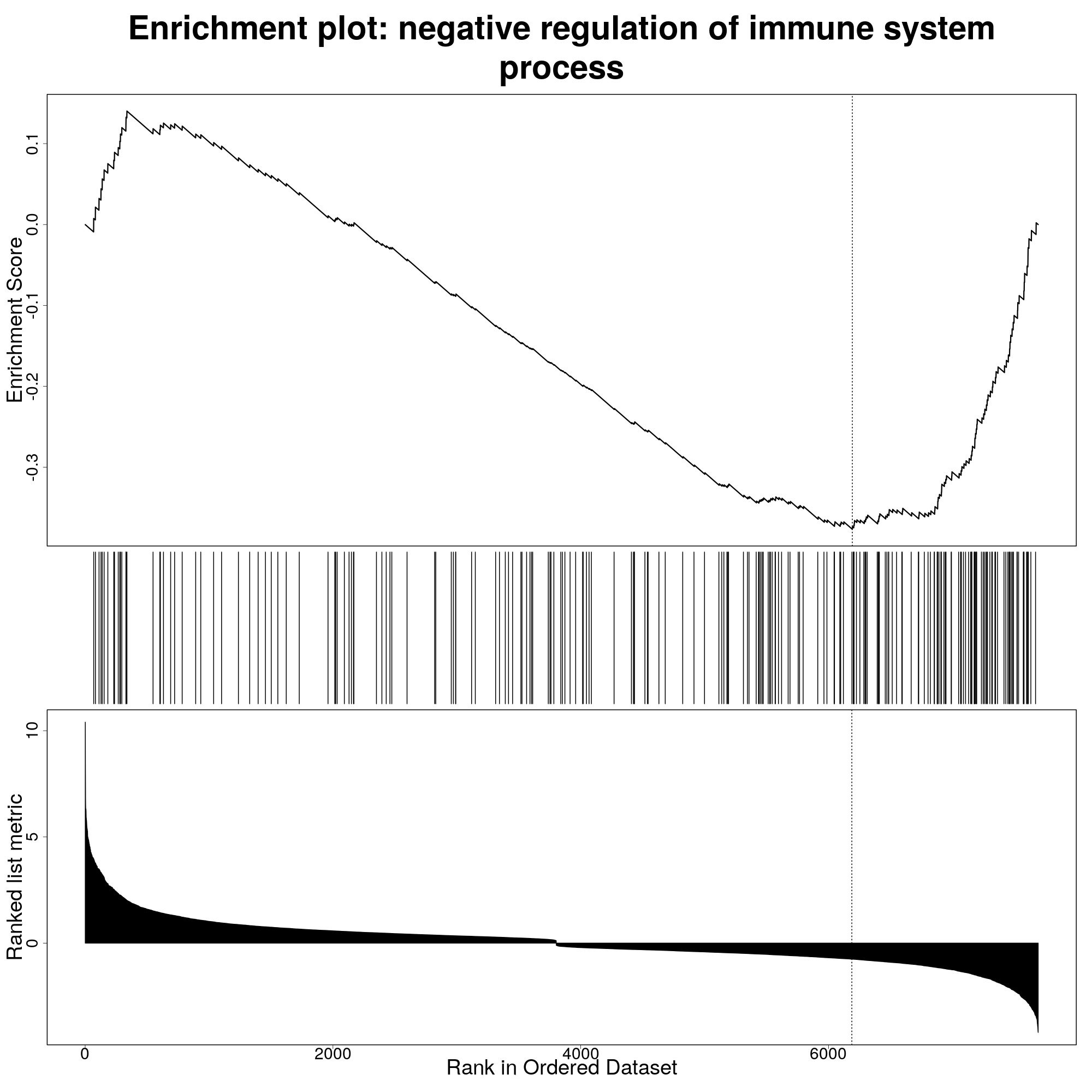

Supplement: Supplementary file 15 [file DataSheet_7.zip › Supplementary data 7 GSEA CCR2lo vs CCR2hi in CIA/Project_high_vs_low_GSEA/GO_0002683.png]

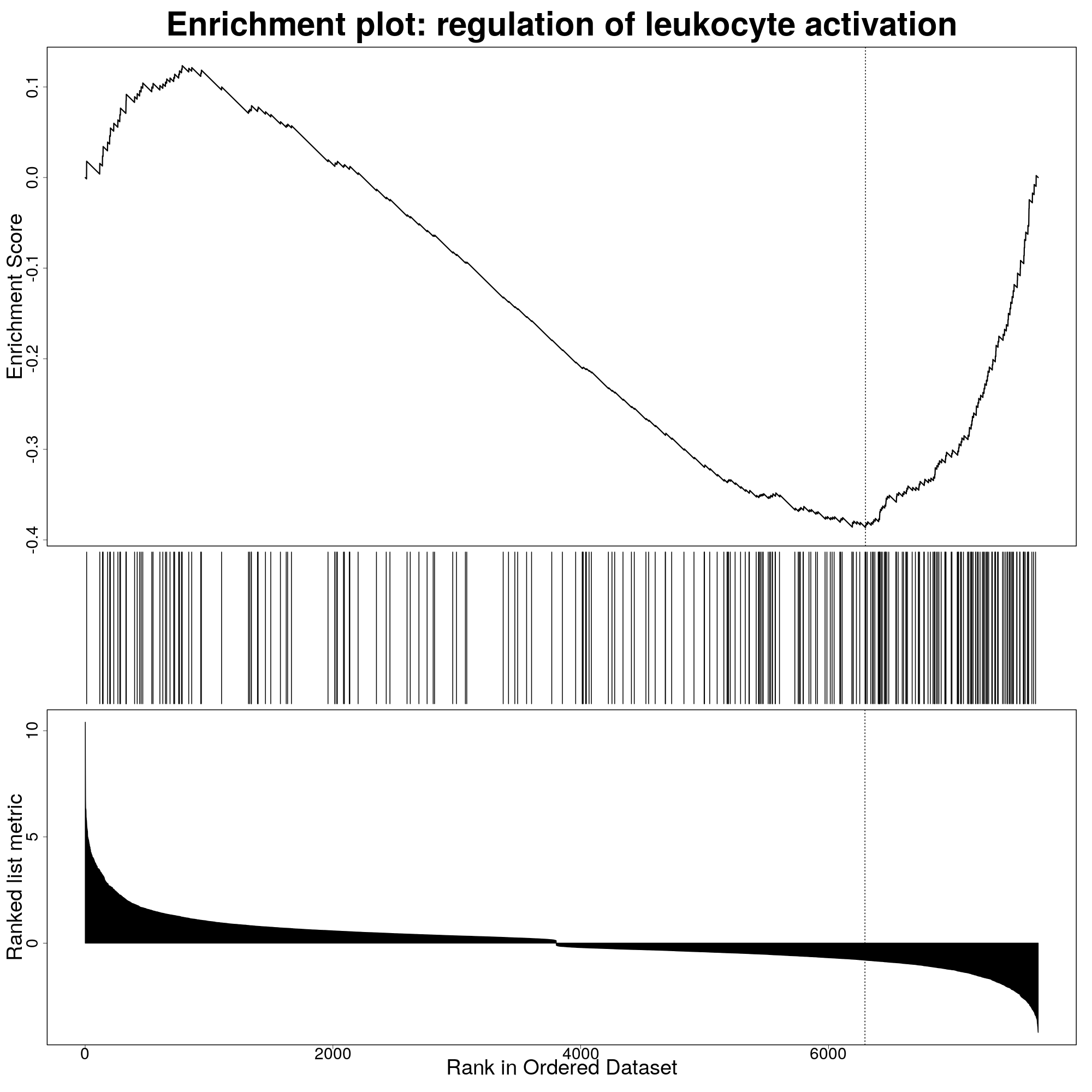

Supplement: Supplementary file 15 [file DataSheet_7.zip › Supplementary data 7 GSEA CCR2lo vs CCR2hi in CIA/Project_high_vs_low_GSEA/GO_0002694.png]

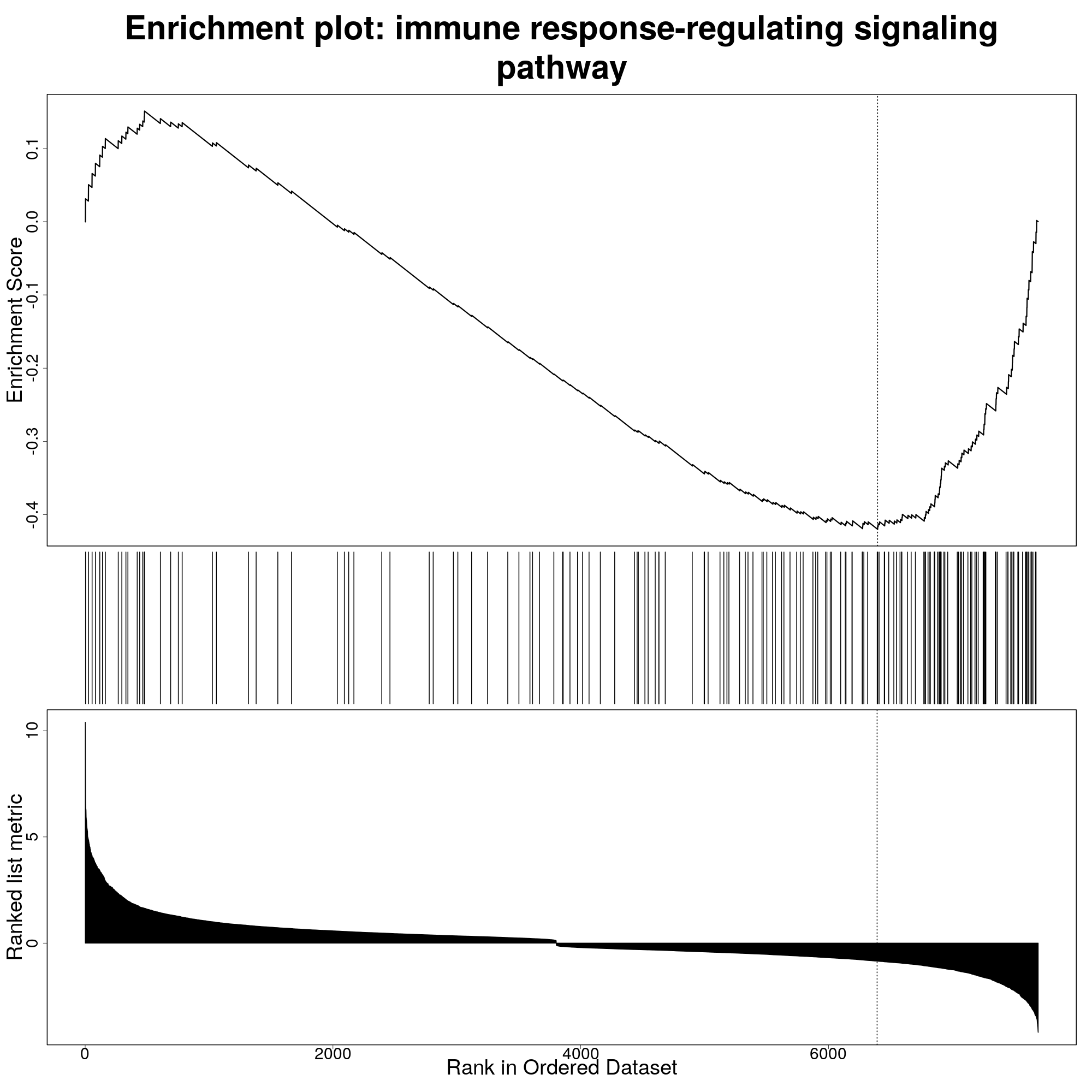

Supplement: Supplementary file 15 [file DataSheet_7.zip › Supplementary data 7 GSEA CCR2lo vs CCR2hi in CIA/Project_high_vs_low_GSEA/GO_0002764.png]

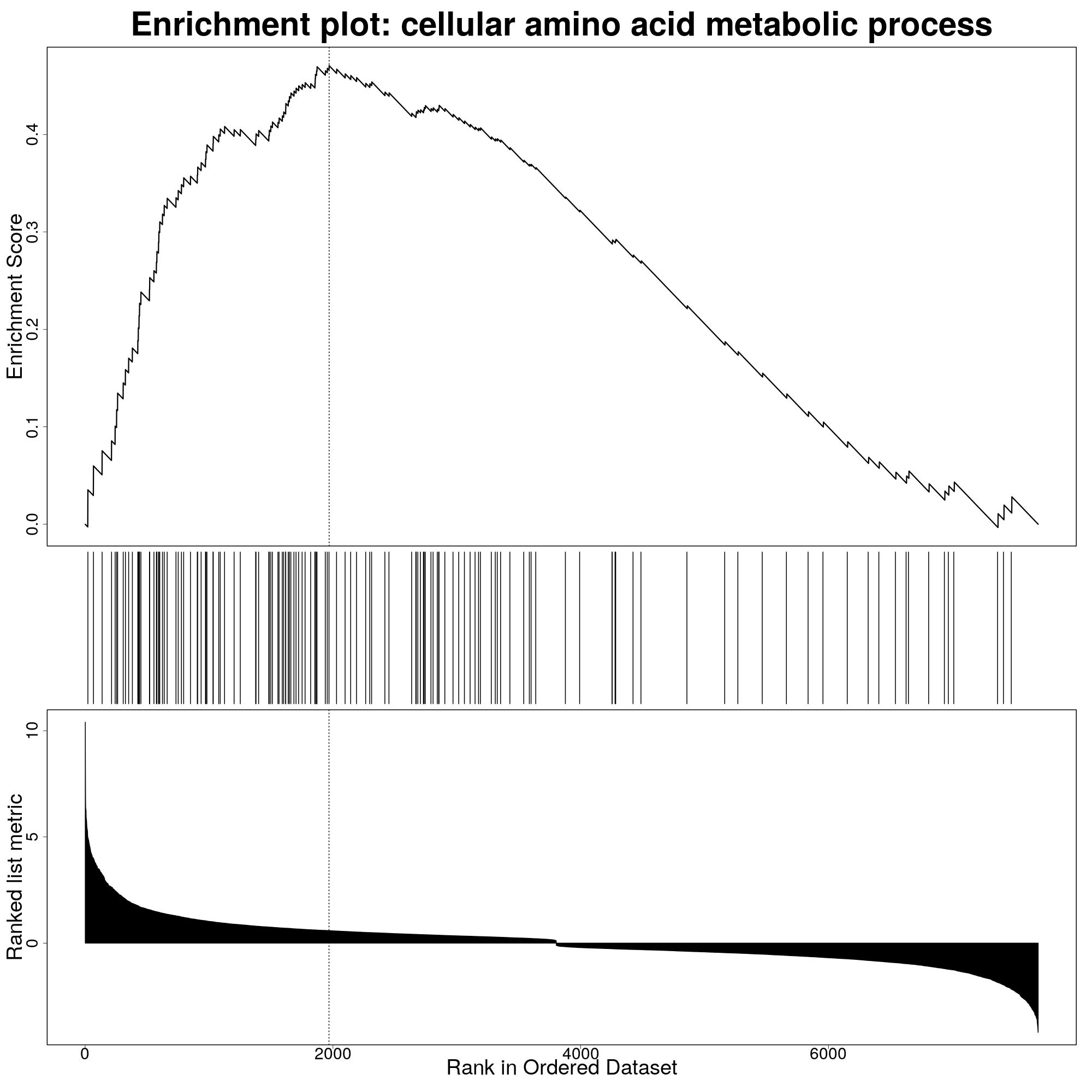

Supplement: Supplementary file 15 [file DataSheet_7.zip › Supplementary data 7 GSEA CCR2lo vs CCR2hi in CIA/Project_high_vs_low_GSEA/GO_0006520.png]

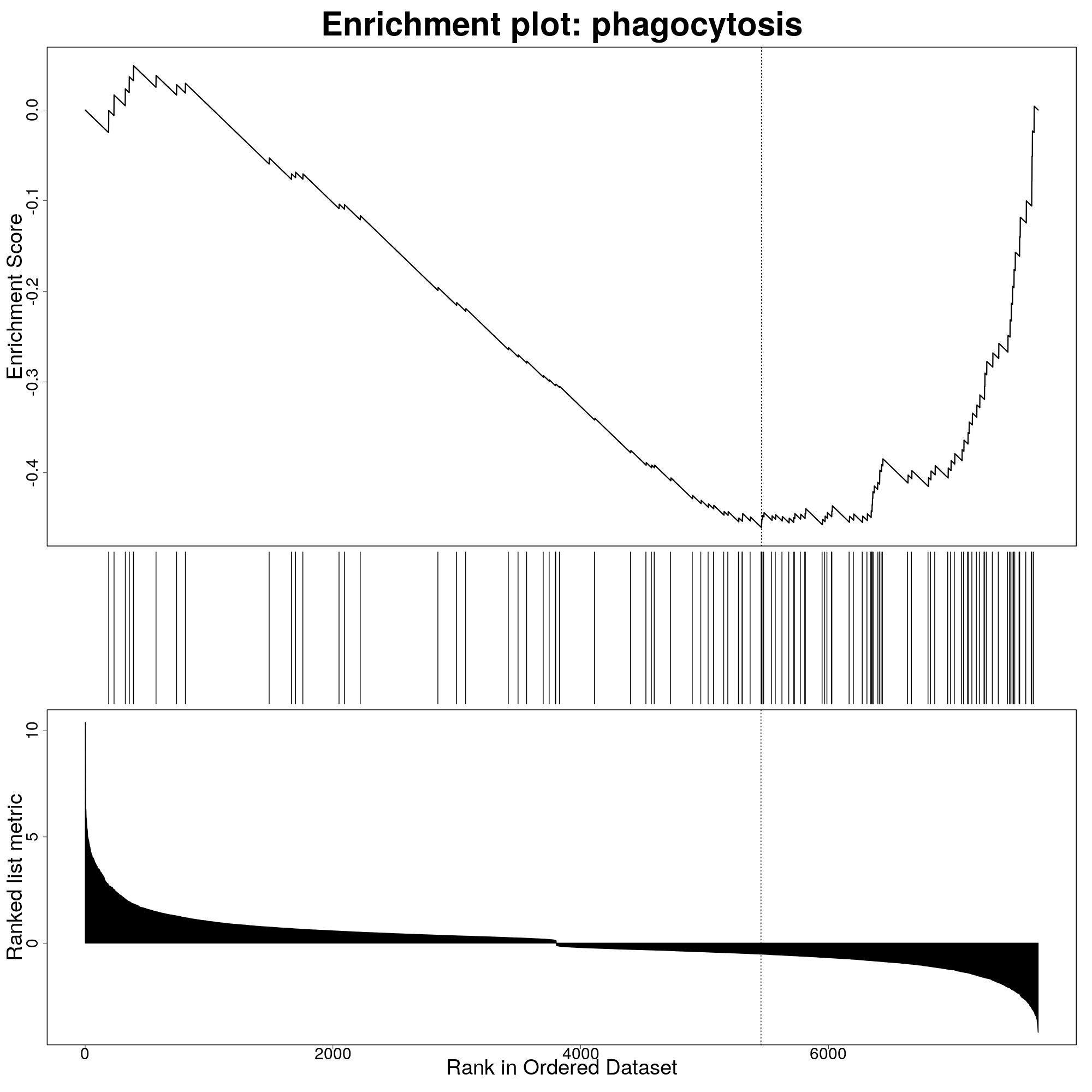

Supplement: Supplementary file 15 [file DataSheet_7.zip › Supplementary data 7 GSEA CCR2lo vs CCR2hi in CIA/Project_high_vs_low_GSEA/GO_0006909.png]

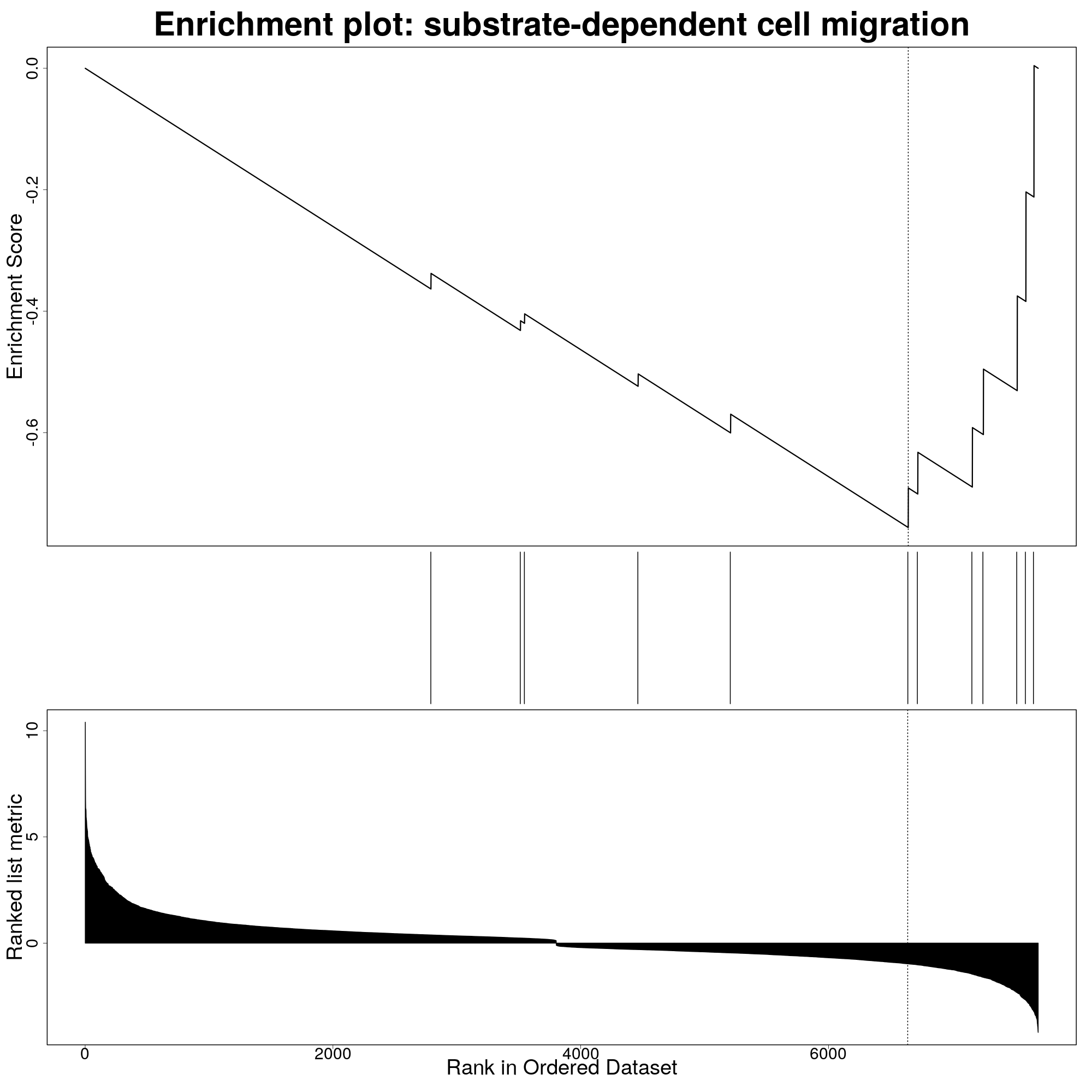

Supplement: Supplementary file 15 [file DataSheet_7.zip › Supplementary data 7 GSEA CCR2lo vs CCR2hi in CIA/Project_high_vs_low_GSEA/GO_0006929.png]

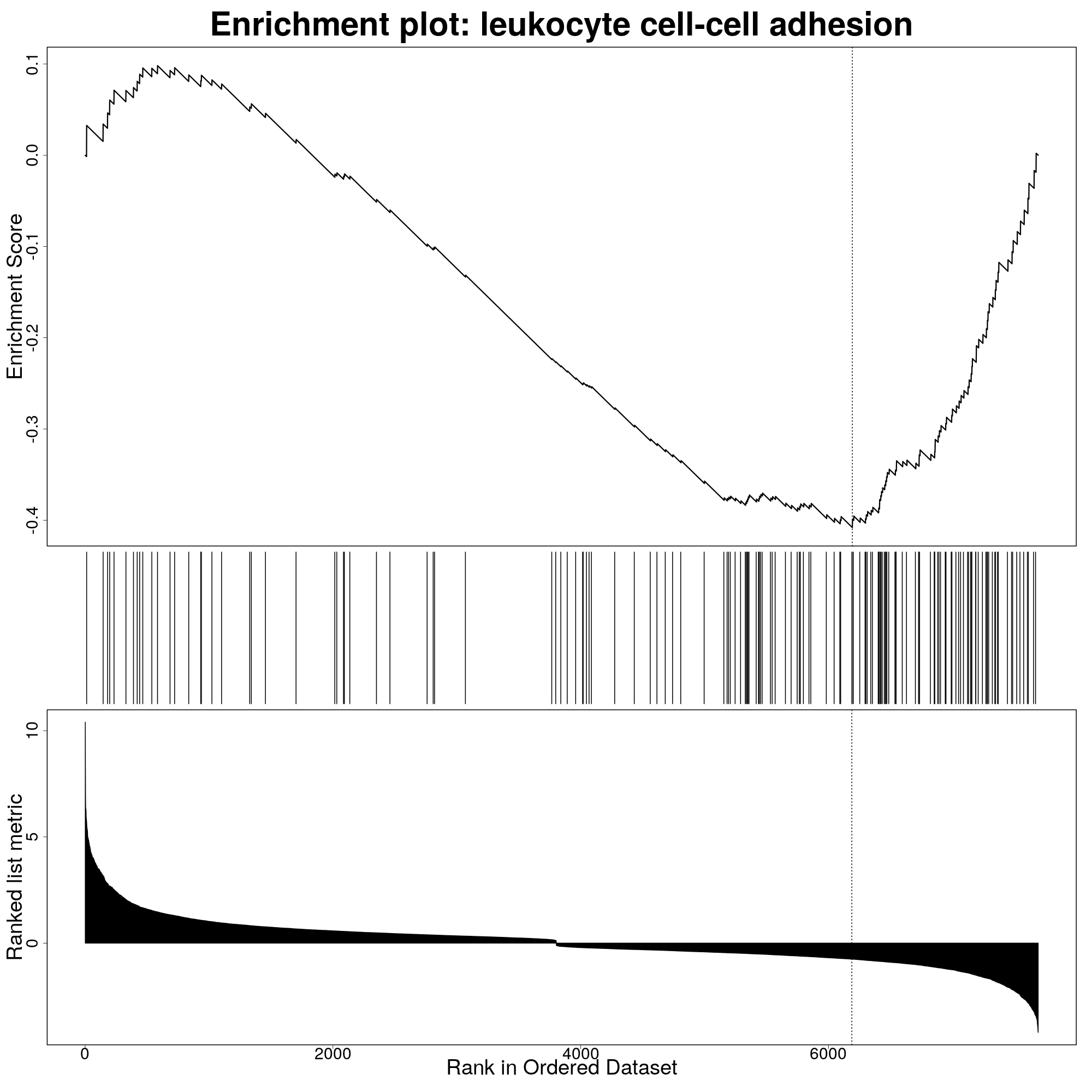

Supplement: Supplementary file 15 [file DataSheet_7.zip › Supplementary data 7 GSEA CCR2lo vs CCR2hi in CIA/Project_high_vs_low_GSEA/GO_0007159.png]

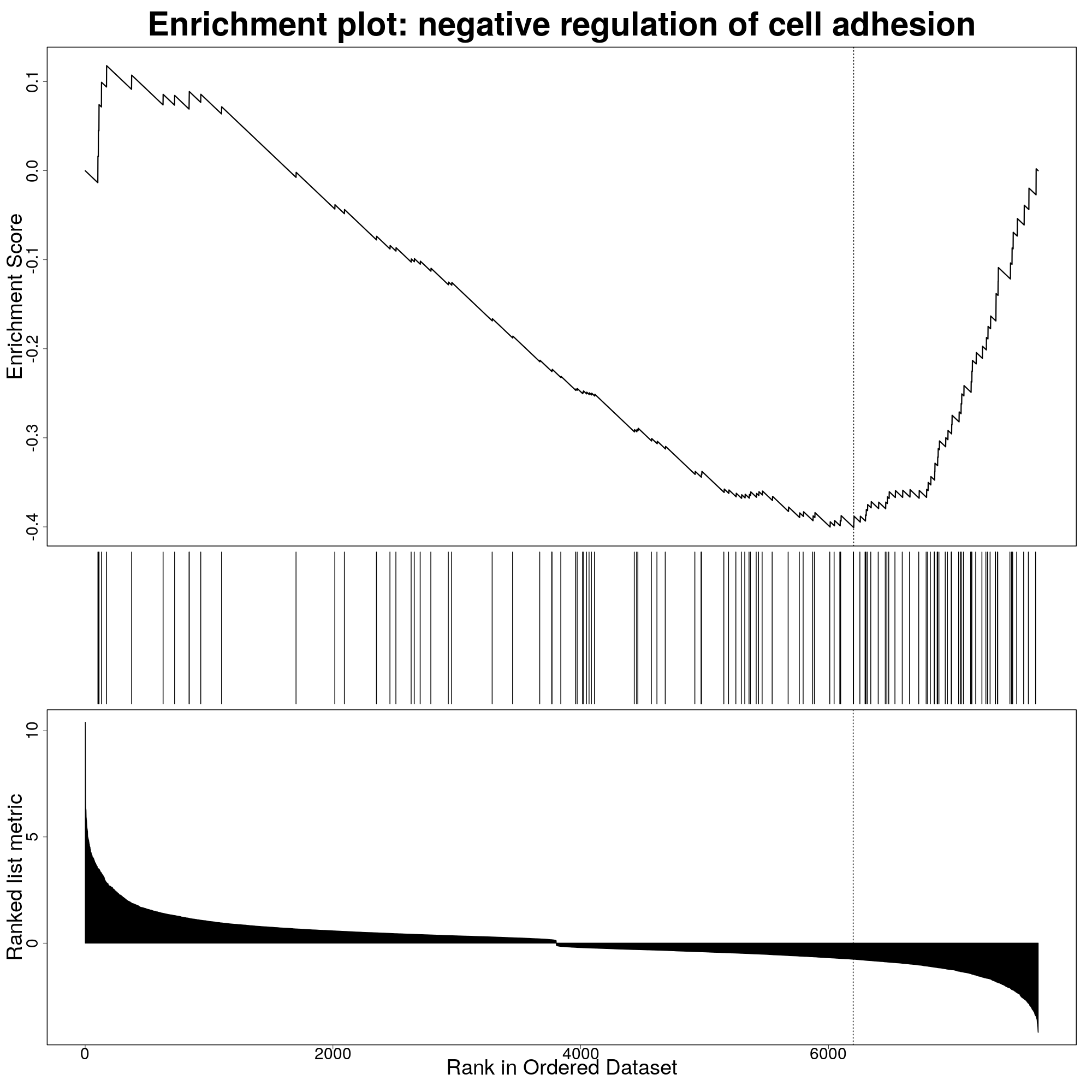

Supplement: Supplementary file 15 [file DataSheet_7.zip › Supplementary data 7 GSEA CCR2lo vs CCR2hi in CIA/Project_high_vs_low_GSEA/GO_0007162.png]

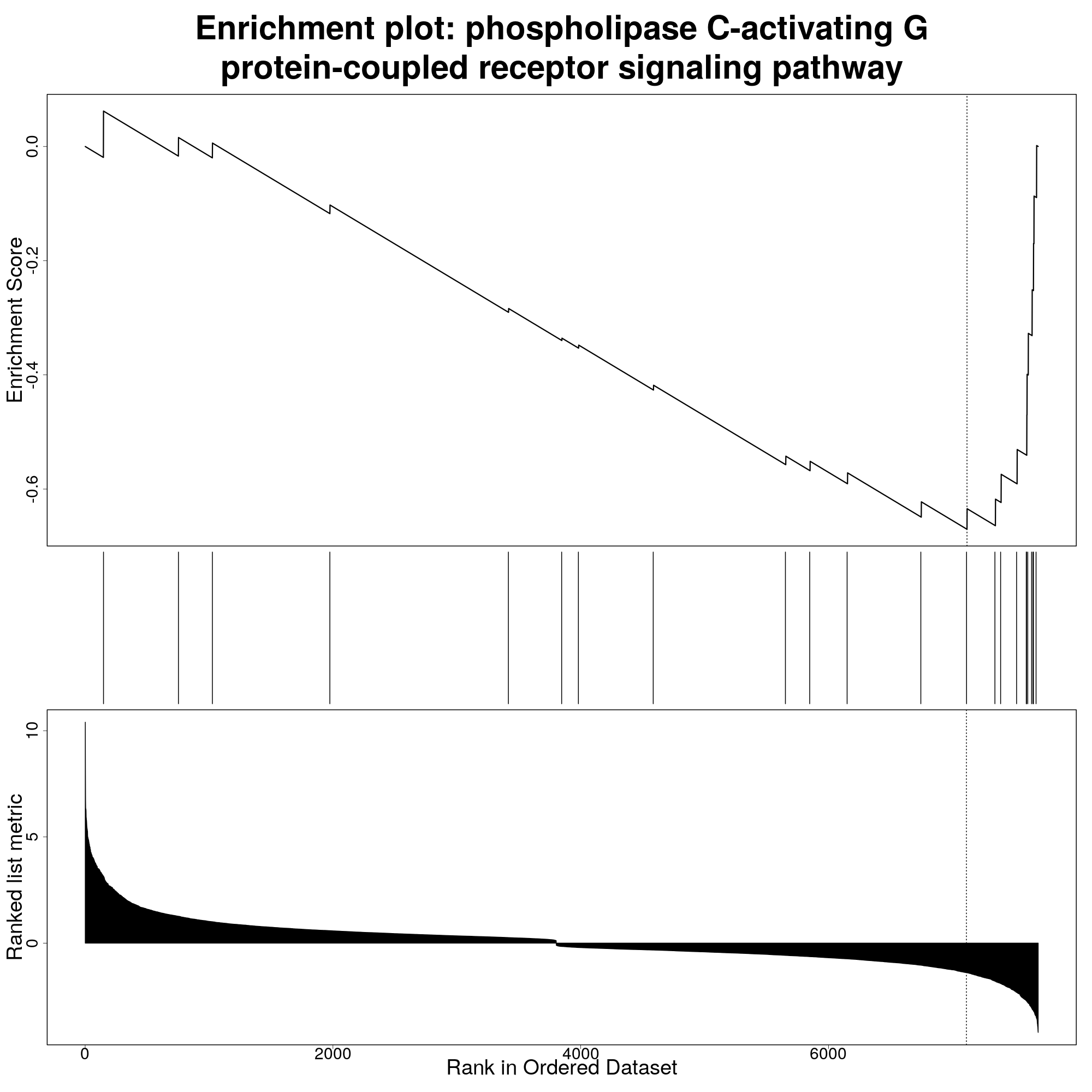

Supplement: Supplementary file 15 [file DataSheet_7.zip › Supplementary data 7 GSEA CCR2lo vs CCR2hi in CIA/Project_high_vs_low_GSEA/GO_0007200.png]

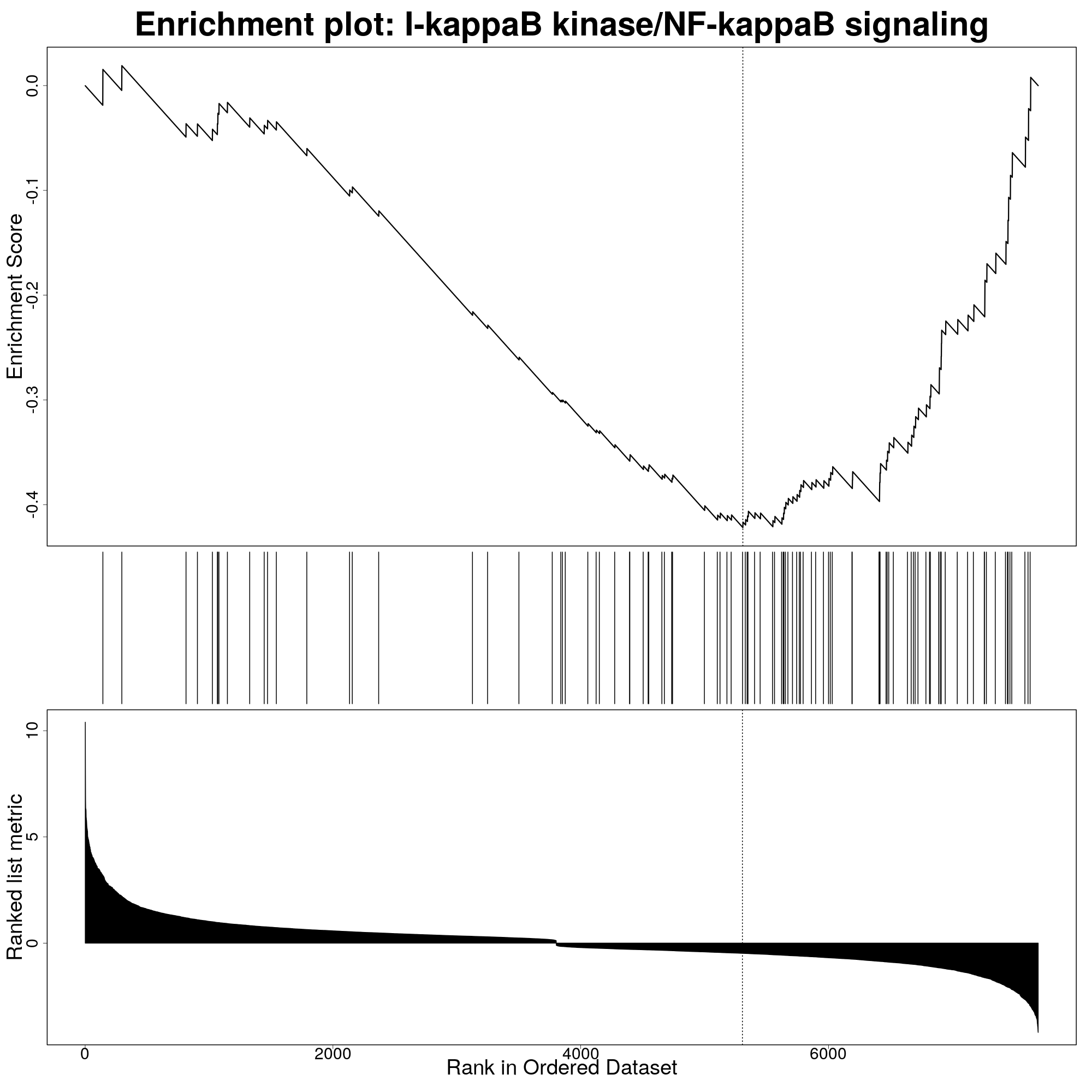

Supplement: Supplementary file 15 [file DataSheet_7.zip › Supplementary data 7 GSEA CCR2lo vs CCR2hi in CIA/Project_high_vs_low_GSEA/GO_0007249.png]

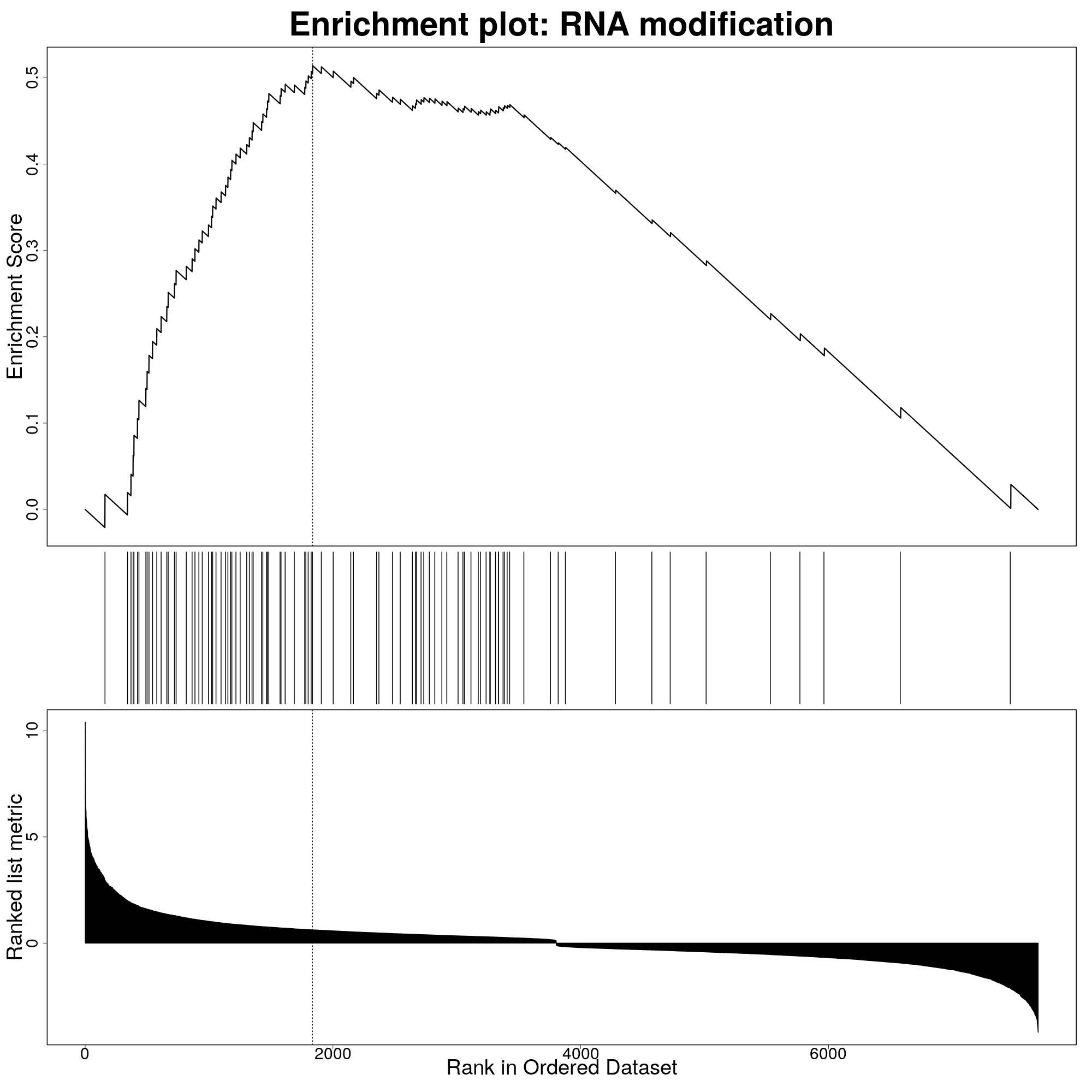

Supplement: Supplementary file 15 [file DataSheet_7.zip › Supplementary data 7 GSEA CCR2lo vs CCR2hi in CIA/Project_high_vs_low_GSEA/GO_0009451.png]

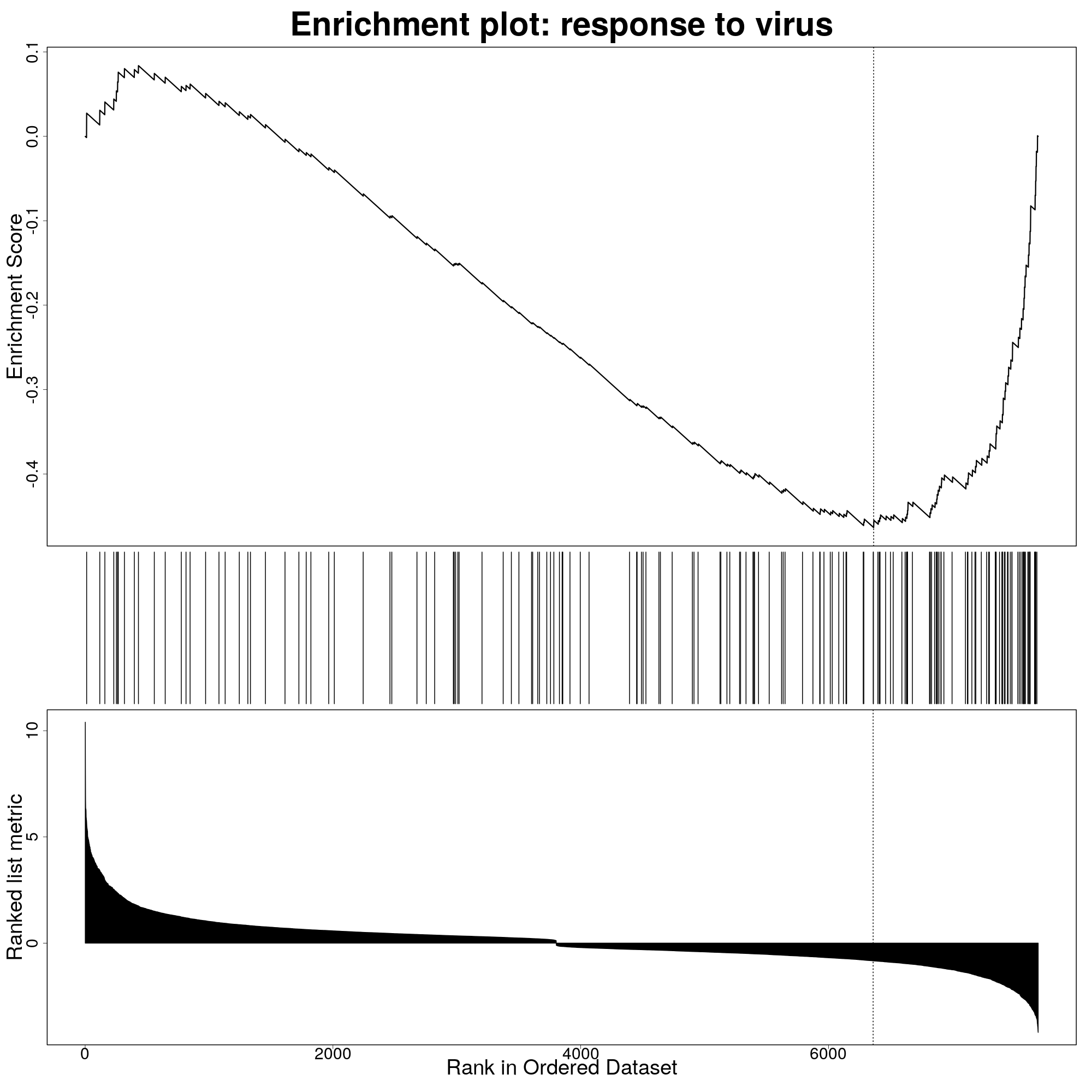

Supplement: Supplementary file 15 [file DataSheet_7.zip › Supplementary data 7 GSEA CCR2lo vs CCR2hi in CIA/Project_high_vs_low_GSEA/GO_0009615.png]

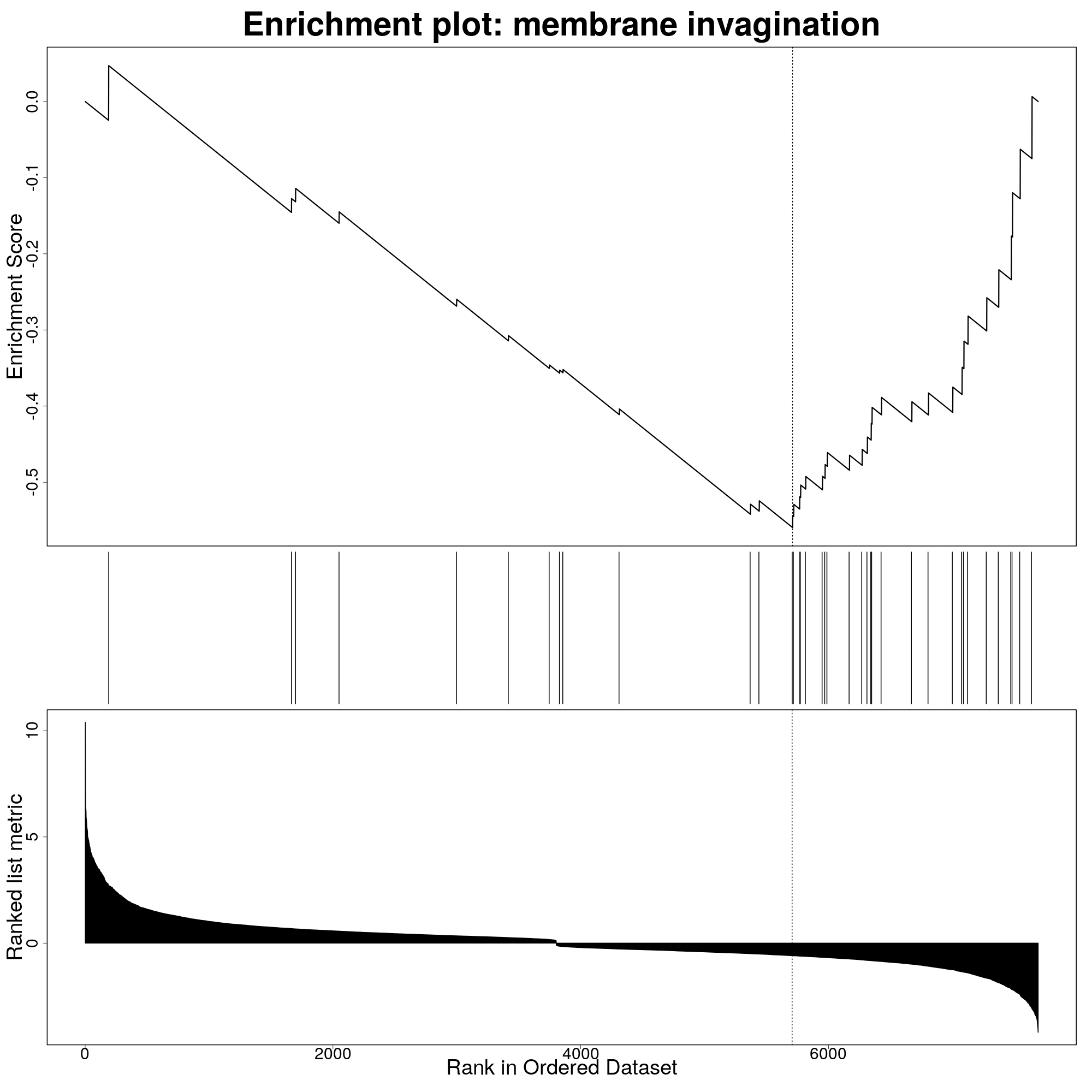

Supplement: Supplementary file 15 [file DataSheet_7.zip › Supplementary data 7 GSEA CCR2lo vs CCR2hi in CIA/Project_high_vs_low_GSEA/GO_0010324.png]

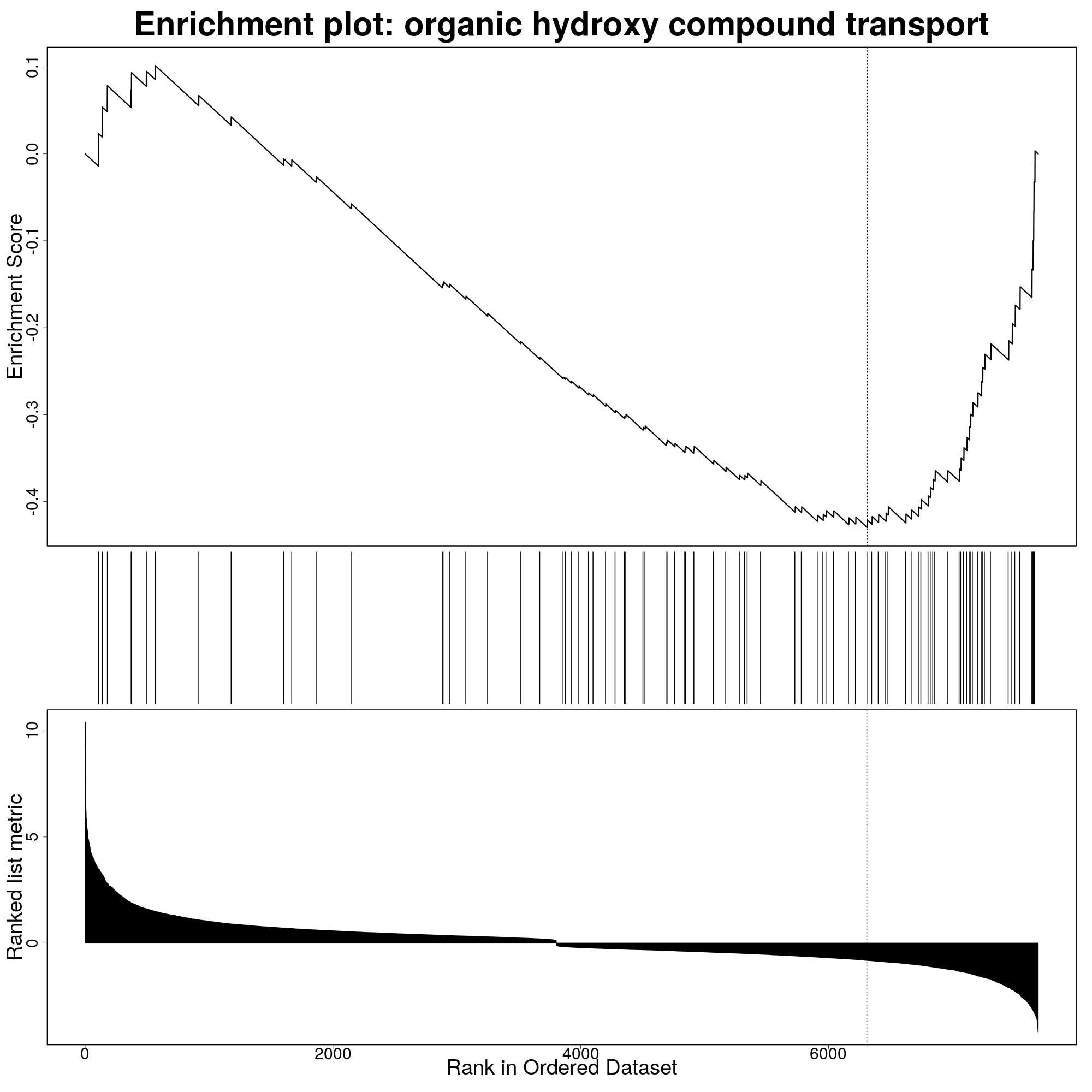

Supplement: Supplementary file 15 [file DataSheet_7.zip › Supplementary data 7 GSEA CCR2lo vs CCR2hi in CIA/Project_high_vs_low_GSEA/GO_0015850.png]
