# Supplementary material for: Transcriptome profiling of osteoclast subsets associated with arthritis: A pathogenic role of CCR2hi osteoclast progenitors
Source: Front Immunol. 2022 Dec 15;13:994035. doi: 10.3389/fimmu.2022.994035 (PMC9797520; doi:10.3389/fimmu.2022.994035)
Supplement: Supplementary file 12 [file DataSheet_4.zip › Supplementary data 4 DGE CTRL vs CIA all samples/RNAseq_analysis_with_DESeq2_p0.01_extended.html]

RNA-seq analysis of differential expression using DESeq2, P value cutoff 0.01


## RNA-seq analysis of differential expression using DESeq2, P value cutoff 0.01

| ID | Name | Type | Position | Image | logFC | p-Value | Adjusted p-Value |
| --- | --- | --- | --- | --- | --- | --- | --- |
| ID | Name | Type | Position | Image | logFC | p-Value | Adjusted p-Value |
| ENSMUSG00000048612 | Myof | protein\_coding | 19:37899036-38043577 (-) |  | -2.1700 | 1.95e-59 | 2.84e-55 |
| ENSMUSG00000034168 | Irf2bpl | protein\_coding | 12:86880701-86884798 (-) |  | -1.4400 | 1.66e-43 | 1.21e-39 |
| ENSMUSG00000031441 | Atp11a | protein\_coding | 8:12757014-12868728 (+) |  | -2.0100 | 1.69e-41 | 8.19e-38 |
| ENSMUSG00000042082 | Arsb | protein\_coding | 13:93771630-93943016 (+) |  | 0.8840 | 1.03e-40 | 3.74e-37 |
| ENSMUSG00000024679 | Ms4a6d | protein\_coding | 19:11586604-11604849 (-) |  | -1.7100 | 1.55e-39 | 4.50e-36 |
| ENSMUSG00000030560 | Ctsc | protein\_coding | 7:88278085-88310888 (+) |  | -1.3100 | 2.65e-36 | 5.83e-33 |
| ENSMUSG00000024349 | Tmem173 | protein\_coding | 18:35733679-35740554 (-) |  | -0.4840 | 3.17e-36 | 5.83e-33 |
| ENSMUSG00000042719 | Naa25 | protein\_coding | 5:121397936-121444378 (+) |  | -0.7000 | 3.22e-36 | 5.83e-33 |
| ENSMUSG00000069917 | Hba-a2 | protein\_coding | 11:32296489-32297298 (+) |  | 3.1900 | 6.40e-36 | 1.03e-32 |
| ENSMUSG00000022912 | Pros1 | protein\_coding | 16:62854307-62929346 (+) |  | -0.8770 | 8.09e-36 | 1.17e-32 |
| ENSMUSG00000037685 | Atp8a1 | protein\_coding | 5:67618140-67847434 (-) |  | -0.8290 | 1.16e-34 | 1.52e-31 |
| ENSMUSG00000062127 | Cttnbp2nl | protein\_coding | 3:105001915-105053146 (-) |  | -1.8200 | 1.17e-33 | 1.32e-30 |
| ENSMUSG00000021477 | Ctsl | protein\_coding | 13:64359337-64370890 (-) |  | -1.0100 | 1.18e-33 | 1.32e-30 |
| ENSMUSG00000074151 | Nlrc5 | protein\_coding | 8:94434356-94527272 (+) |  | -0.8390 | 2.85e-33 | 2.95e-30 |
| ENSMUSG00000079227 | Ccr5 | protein\_coding | 9:124121543-124147699 (+) |  | -1.7100 | 3.55e-33 | 3.44e-30 |
| ENSMUSG00000052698 | Tln2 | protein\_coding | 9:67217087-67559703 (-) |  | -2.1000 | 4.76e-32 | 4.31e-29 |
| ENSMUSG00000030246 | Ldhb | protein\_coding | 6:142490249-142507957 (-) |  | 1.3100 | 3.51e-30 | 3.00e-27 |
| ENSMUSG00000021458 | Aopep | protein\_coding | 13:62964893-63326096 (+) |  | -0.7110 | 4.43e-30 | 3.57e-27 |
| ENSMUSG00000019528 | Gyg | protein\_coding | 3:20122084-20155317 (-) |  | -0.7220 | 6.30e-30 | 4.81e-27 |
| ENSMUSG00000039713 | Plekhg5 | protein\_coding | 4:152072498-152115400 (+) |  | 1.8800 | 2.03e-29 | 1.48e-26 |
| ENSMUSG00000021097 | Clmn | protein\_coding | 12:104763117-104865076 (-) |  | -2.3100 | 7.69e-29 | 5.31e-26 |
| ENSMUSG00000059336 | Slc14a1 | protein\_coding | 18:78100091-78142119 (-) |  | 2.0900 | 8.26e-29 | 5.45e-26 |
| ENSMUSG00000026768 | Itga8 | protein\_coding | 2:12106632-12301922 (-) |  | 3.2300 | 2.64e-28 | 1.67e-25 |
| ENSMUSG00000032508 | Myd88 | protein\_coding | 9:119335934-119341411 (-) |  | -0.5990 | 1.89e-27 | 1.10e-24 |
| ENSMUSG00000030083 | Abtb1 | protein\_coding | 6:88835914-88841984 (-) |  | 0.6930 | 1.89e-27 | 1.10e-24 |
| ENSMUSG00000060512 | 0610040J01Rik | protein\_coding | 5:63812363-63899625 (+) |  | -1.0100 | 3.46e-27 | 1.93e-24 |
| ENSMUSG00000046314 | Stxbp6 | protein\_coding | 12:44852484-45074709 (-) |  | 1.2000 | 6.80e-26 | 3.65e-23 |
| ENSMUSG00000027864 | Ptgfrn | protein\_coding | 3:101040232-101110278 (-) |  | -1.7100 | 1.42e-25 | 7.38e-23 |
| ENSMUSG00000035847 | Ids | protein\_coding | X:70343069-70365084 (-) |  | 0.7680 | 4.54e-25 | 2.27e-22 |
| ENSMUSG00000037235 | Mxd4 | protein\_coding | 5:34173883-34187720 (-) |  | 0.8410 | 8.58e-25 | 4.15e-22 |
| ENSMUSG00000020593 | Lpin1 | protein\_coding | 12:16535669-16646966 (-) |  | 1.5900 | 1.02e-24 | 4.76e-22 |
| ENSMUSG00000056091 | St3gal5 | protein\_coding | 6:72097592-72154571 (+) |  | 1.5300 | 1.16e-24 | 5.26e-22 |
| ENSMUSG00000028228 | Cpne3 | protein\_coding | 4:19519254-19570108 (-) |  | -0.5080 | 2.15e-24 | 9.43e-22 |
| ENSMUSG00000045038 | Prkce | protein\_coding | 17:86167785-86657919 (+) |  | 0.7630 | 2.45e-24 | 1.04e-21 |
| ENSMUSG00000022218 | Tgm1 | protein\_coding | 14:55700009-55713926 (-) |  | -1.5200 | 2.78e-24 | 1.15e-21 |
| ENSMUSG00000048442 | Smim5 | protein\_coding | 11:115899966-115906269 (+) |  | 1.3500 | 4.92e-24 | 1.98e-21 |
| ENSMUSG00000116114 | Gm35853 | lncRNA | 15:101322888-101405834 (+) |  | 2.1500 | 7.10e-24 | 2.79e-21 |
| ENSMUSG00000027175 | Tcp11l1 | protein\_coding | 2:104657288-104712169 (-) |  | -0.8080 | 1.85e-23 | 7.07e-21 |
| ENSMUSG00000027199 | Gatm | protein\_coding | 2:122594467-122611303 (-) |  | -1.1400 | 3.08e-23 | 1.14e-20 |
| ENSMUSG00000032369 | Plscr1 | protein\_coding | 9:92249750-92272278 (+) |  | -1.1700 | 7.57e-23 | 2.75e-20 |
| ENSMUSG00000051166 | Eml5 | protein\_coding | 12:98786805-98901484 (-) |  | 1.4500 | 1.13e-22 | 4.00e-20 |
| ENSMUSG00000031805 | Jak3 | protein\_coding | 8:71676296-71690575 (+) |  | -1.0100 | 1.60e-22 | 5.53e-20 |
| ENSMUSG00000028124 | Gclm | protein\_coding | 3:122245557-122270732 (+) |  | -1.0200 | 3.10e-22 | 1.05e-19 |
| ENSMUSG00000054676 | 1600014C10Rik | protein\_coding | 7:38183217-38197568 (+) |  | -0.5570 | 3.22e-22 | 1.06e-19 |
| ENSMUSG00000038352 | Arl5c | protein\_coding | 11:97989578-97996181 (-) |  | 0.9820 | 4.61e-22 | 1.49e-19 |
| ENSMUSG00000071713 | Csf2rb | protein\_coding | 15:78325752-78353847 (+) |  | -0.4890 | 5.73e-22 | 1.81e-19 |
| ENSMUSG00000024180 | Tmem8 | protein\_coding | 17:26113299-26123254 (+) |  | 0.7620 | 7.36e-22 | 2.27e-19 |
| ENSMUSG00000052305 | Hbb-bs | protein\_coding | 7:103826534-103828096 (-) |  | 2.4900 | 8.32e-22 | 2.51e-19 |
| ENSMUSG00000022636 | Alcam | protein\_coding | 16:52248996-52454074 (-) |  | 0.6030 | 1.17e-21 | 3.47e-19 |
| ENSMUSG00000052534 | Pbx1 | protein\_coding | 1:168119364-168432270 (-) |  | -1.3000 | 1.43e-21 | 4.16e-19 |
| ENSMUSG00000050379 | Sept6 | protein\_coding | X:36911326-36991794 (-) |  | 0.6340 | 1.46e-21 | 4.17e-19 |
| ENSMUSG00000105504 | Gbp5 | protein\_coding | 3:142493978-142522344 (+) |  | -1.0600 | 1.55e-21 | 4.31e-19 |
| ENSMUSG00000040253 | Gbp7 | protein\_coding | 3:142530342-142550149 (+) |  | -0.9150 | 1.61e-21 | 4.41e-19 |
| ENSMUSG00000074794 | Arrdc3 | protein\_coding | 13:80883384-80896042 (+) |  | 0.6330 | 1.73e-21 | 4.66e-19 |
| ENSMUSG00000005802 | Slc30a4 | protein\_coding | 2:122681233-122702663 (-) |  | -1.3000 | 3.95e-21 | 1.04e-18 |
| ENSMUSG00000039542 | Ncam1 | protein\_coding | 9:49502136-49798925 (-) |  | 1.0600 | 4.61e-21 | 1.19e-18 |
| ENSMUSG00000030748 | Il4ra | protein\_coding | 7:125552120-125579474 (+) |  | -0.8490 | 8.57e-21 | 2.18e-18 |
| ENSMUSG00000058624 | Gda | protein\_coding | 19:21391307-21473445 (-) |  | -0.7420 | 9.13e-21 | 2.28e-18 |
| ENSMUSG00000030147 | Clec4b1 | protein\_coding | 6:123049962-123071555 (+) |  | 1.3900 | 1.77e-20 | 4.35e-18 |
| ENSMUSG00000041926 | Rnpep | protein\_coding | 1:135262712-135284084 (-) |  | -0.5290 | 2.28e-20 | 5.51e-18 |
| ENSMUSG00000000686 | Abhd15 | protein\_coding | 11:77515121-77538607 (+) |  | 0.6540 | 2.73e-20 | 6.50e-18 |
| ENSMUSG00000021830 | Txndc16 | protein\_coding | 14:45133465-45220328 (-) |  | 0.6400 | 3.63e-20 | 8.49e-18 |
| ENSMUSG00000026245 | Farsb | protein\_coding | 1:78417975-78488897 (-) |  | -0.5960 | 4.41e-20 | 1.02e-17 |
| ENSMUSG00000040078 | Ptges3-ps | processed\_pseudogene | 6:85843980-85844459 (+) |  | -0.6590 | 1.10e-19 | 2.49e-17 |
| ENSMUSG00000030427 | Lilra6 | protein\_coding | 7:3908280-3915503 (-) |  | 0.9640 | 1.16e-19 | 2.60e-17 |
| ENSMUSG00000021037 | Ahsa1 | protein\_coding | 12:87266479-87273998 (+) |  | -0.5030 | 1.24e-19 | 2.73e-17 |
| ENSMUSG00000067714 | Lpar5 | protein\_coding | 6:125067920-125082472 (+) |  | 0.7070 | 1.59e-19 | 3.43e-17 |
| ENSMUSG00000033720 | Sfxn5 | protein\_coding | 6:85213049-85333422 (-) |  | -1.0100 | 2.57e-19 | 5.48e-17 |
| ENSMUSG00000028811 | Yars | protein\_coding | 4:129189760-129219607 (+) |  | -0.5850 | 2.64e-19 | 5.55e-17 |
| ENSMUSG00000031391 | L1cam | protein\_coding | X:73853778-73896105 (-) |  | 0.8900 | 3.05e-19 | 6.31e-17 |
| ENSMUSG00000037095 | Lrg1 | protein\_coding | 17:56119678-56122001 (-) |  | -5.3200 | 3.33e-19 | 6.80e-17 |
| ENSMUSG00000043939 | A530064D06Rik | protein\_coding | 17:48149126-48167275 (-) |  | 1.1600 | 3.64e-19 | 7.33e-17 |
| ENSMUSG00000018381 | Abi3 | protein\_coding | 11:95830074-95842476 (-) |  | -1.4200 | 4.51e-19 | 8.97e-17 |
| ENSMUSG00000031555 | Adam9 | protein\_coding | 8:24949611-25016927 (-) |  | -0.6820 | 6.20e-19 | 1.22e-16 |
| ENSMUSG00000074203 | G430095P16Rik | protein\_coding | 8:84723007-84726844 (+) |  | 0.9910 | 7.27e-19 | 1.41e-16 |
| ENSMUSG00000023039 | Krt7 | protein\_coding | 15:101411043-101430313 (+) |  | 2.4600 | 7.64e-19 | 1.46e-16 |
| ENSMUSG00000024247 | Pkdcc | protein\_coding | 17:83215292-83225070 (+) |  | 1.3000 | 1.15e-18 | 2.17e-16 |
| ENSMUSG00000024905 | Tesmin | protein\_coding | 19:3388857-3407823 (+) |  | 1.3700 | 1.41e-18 | 2.63e-16 |
| ENSMUSG00000041633 | Kctd12b | protein\_coding | X:153685154-153696391 (-) |  | 1.0500 | 1.69e-18 | 3.10e-16 |
| ENSMUSG00000037331 | Larp1 | protein\_coding | 11:58009064-58062034 (+) |  | -0.5250 | 1.77e-18 | 3.22e-16 |
| ENSMUSG00000073940 | Hbb-bt | protein\_coding | 7:103812524-103813996 (-) |  | 2.9000 | 1.86e-18 | 3.33e-16 |
| ENSMUSG00000039899 | Fgl2 | protein\_coding | 5:21372642-21378374 (+) |  | -0.6480 | 1.88e-18 | 3.33e-16 |
| ENSMUSG00000039145 | Camk1d | protein\_coding | 2:5293457-5714515 (-) |  | 1.0100 | 2.28e-18 | 3.98e-16 |
| ENSMUSG00000024014 | Pim1 | protein\_coding | 17:29490753-29496112 (+) |  | -1.3300 | 2.51e-18 | 4.33e-16 |
| ENSMUSG00000034667 | Xpot | protein\_coding | 10:121587380-121626332 (-) |  | -0.4840 | 2.83e-18 | 4.84e-16 |
| ENSMUSG00000037242 | Clic4 | protein\_coding | 4:135213969-135272814 (-) |  | -0.3520 | 3.36e-18 | 5.62e-16 |
| ENSMUSG00000097077 | Gm16712 | lncRNA | 17:55954771-55959381 (-) |  | 1.5600 | 3.37e-18 | 5.62e-16 |
| ENSMUSG00000027463 | Slc52a3 | protein\_coding | 2:151996511-152009258 (+) |  | -2.0000 | 4.88e-18 | 8.04e-16 |
| ENSMUSG00000021270 | Hsp90aa1 | protein\_coding | 12:110690605-110702728 (-) |  | -0.6820 | 8.37e-18 | 1.36e-15 |
| ENSMUSG00000001380 | Hars | protein\_coding | 18:36766528-36783205 (-) |  | -0.4050 | 1.03e-17 | 1.66e-15 |
| ENSMUSG00000070031 | Sp140 | protein\_coding | 1:85600378-85645037 (+) |  | -0.5060 | 1.05e-17 | 1.67e-15 |
| ENSMUSG00000032271 | Nnmt | protein\_coding | 9:48591877-48605153 (-) |  | -2.9700 | 1.16e-17 | 1.83e-15 |
| ENSMUSG00000052212 | Cd177 | protein\_coding | 7:24743983-24760311 (-) |  | 1.5100 | 1.21e-17 | 1.89e-15 |
| ENSMUSG00000018848 | Rars | protein\_coding | 11:35808381-35834506 (-) |  | -0.4290 | 1.42e-17 | 2.19e-15 |
| ENSMUSG00000042066 | Tmcc2 | protein\_coding | 1:132356315-132391281 (-) |  | 0.6460 | 1.58e-17 | 2.42e-15 |
| ENSMUSG00000002083 | Bbc3 | protein\_coding | 7:16308393-16318205 (+) |  | 1.0000 | 1.87e-17 | 2.82e-15 |
| ENSMUSG00000047879 | Usp14 | protein\_coding | 18:9993066-10045119 (-) |  | -0.4320 | 2.13e-17 | 3.18e-15 |
| ENSMUSG00000029162 | Khk | protein\_coding | 5:30921431-30931248 (+) |  | 0.7780 | 2.23e-17 | 3.31e-15 |
| ENSMUSG00000025491 | Ifitm1 | protein\_coding | 7:140967221-140969825 (+) |  | -2.4800 | 3.08e-17 | 4.52e-15 |
| ENSMUSG00000019850 | Tnfaip3 | protein\_coding | 10:19000910-19015657 (-) |  | 0.8400 | 3.45e-17 | 5.00e-15 |
| ENSMUSG00000073418 | C4b | protein\_coding | 17:34728380-34743882 (-) |  | -2.4400 | 4.08e-17 | 5.85e-15 |
| ENSMUSG00000056643 | Chst13 | protein\_coding | 6:90308349-90325185 (-) |  | 1.3800 | 4.18e-17 | 5.92e-15 |
| ENSMUSG00000021948 | Prkcd | protein\_coding | 14:30595354-30626210 (-) |  | 0.5050 | 4.22e-17 | 5.92e-15 |
| ENSMUSG00000029084 | Cd38 | protein\_coding | 5:43868553-43912375 (+) |  | -5.2800 | 4.26e-17 | 5.92e-15 |
| ENSMUSG00000006442 | Srm | protein\_coding | 4:148591503-148594993 (+) |  | -0.6800 | 4.29e-17 | 5.92e-15 |
| ENSMUSG00000004929 | Thop1 | protein\_coding | 10:81070035-81082559 (+) |  | -0.7140 | 4.43e-17 | 6.06e-15 |
| ENSMUSG00000070730 | Rmdn3 | protein\_coding | 2:119137001-119157034 (-) |  | -0.4380 | 5.25e-17 | 7.11e-15 |
| ENSMUSG00000015176 | Nolc1 | protein\_coding | 19:46075863-46085530 (+) |  | -0.5480 | 5.30e-17 | 7.12e-15 |
| ENSMUSG00000032802 | Srxn1 | protein\_coding | 2:152105516-152111376 (+) |  | -1.0300 | 6.63e-17 | 8.82e-15 |
| ENSMUSG00000006574 | Slc4a1 | protein\_coding | 11:102348824-102366203 (-) |  | 1.9400 | 7.67e-17 | 1.01e-14 |
| ENSMUSG00000029925 | Tbxas1 | protein\_coding | 6:38875404-39084585 (+) |  | 0.8400 | 8.34e-17 | 1.09e-14 |
| ENSMUSG00000003206 | Ebi3 | protein\_coding | 17:55952640-55957022 (+) |  | 1.2000 | 9.54e-17 | 1.24e-14 |
| ENSMUSG00000020844 | Nxn | protein\_coding | 11:76257198-76399140 (-) |  | -0.7570 | 9.83e-17 | 1.26e-14 |
| ENSMUSG00000020869 | Lrrc59 | protein\_coding | 11:94629767-94645216 (+) |  | -0.6190 | 1.08e-16 | 1.37e-14 |
| ENSMUSG00000026721 | Rabgap1l | protein\_coding | 1:160219174-160793211 (-) |  | 0.8280 | 1.20e-16 | 1.51e-14 |
| ENSMUSG00000033294 | Noc4l | protein\_coding | 5:110648418-110653417 (-) |  | -0.5620 | 1.22e-16 | 1.53e-14 |
| ENSMUSG00000045817 | Zfp36l2 | protein\_coding | 17:84183931-84187947 (-) |  | 0.6080 | 1.29e-16 | 1.60e-14 |
| ENSMUSG00000025980 | Hspd1 | protein\_coding | 1:55077835-55088243 (-) |  | -0.7400 | 1.44e-16 | 1.76e-14 |
| ENSMUSG00000020689 | Itgb3 | protein\_coding | 11:104608000-104670476 (+) |  | 1.9100 | 1.44e-16 | 1.76e-14 |
| ENSMUSG00000053819 | Camk2d | protein\_coding | 3:126596302-126846326 (+) |  | -1.5200 | 1.56e-16 | 1.88e-14 |
| ENSMUSG00000016552 | Foxred2 | protein\_coding | 15:77940522-77956722 (-) |  | 0.8100 | 1.68e-16 | 2.01e-14 |
| ENSMUSG00000020021 | Fgd6 | protein\_coding | 10:94036001-94145339 (+) |  | -1.3900 | 2.04e-16 | 2.43e-14 |
| ENSMUSG00000021116 | Eif2s1 | protein\_coding | 12:78861819-78887010 (+) |  | -0.4730 | 2.46e-16 | 2.90e-14 |
| ENSMUSG00000098557 | Kctd12 | protein\_coding | 14:102976581-102982637 (-) |  | 0.4370 | 2.53e-16 | 2.96e-14 |
| ENSMUSG00000029447 | Cct6a | protein\_coding | 5:129786998-129846371 (+) |  | -0.5800 | 2.70e-16 | 3.14e-14 |
| ENSMUSG00000025007 | Aldh18a1 | protein\_coding | 19:40550257-40588463 (-) |  | -0.7580 | 2.87e-16 | 3.31e-14 |
| ENSMUSG00000021708 | Rasgrf2 | protein\_coding | 13:91880400-92131656 (-) |  | 1.1500 | 3.11e-16 | 3.55e-14 |
| ENSMUSG00000028851 | Nudc | protein\_coding | 4:133532542-133545996 (-) |  | -0.4390 | 4.20e-16 | 4.77e-14 |
| ENSMUSG00000026893 | Gca | protein\_coding | 2:62664285-62694109 (+) |  | -1.1700 | 4.54e-16 | 5.11e-14 |
| ENSMUSG00000003420 | Fcgrt | protein\_coding | 7:45092990-45103851 (-) |  | 1.0500 | 4.59e-16 | 5.12e-14 |
| ENSMUSG00000042688 | Mapk6 | protein\_coding | 9:75369062-75410005 (-) |  | -0.5550 | 4.66e-16 | 5.16e-14 |
| ENSMUSG00000020238 | Ncln | protein\_coding | 10:81486249-81496392 (-) |  | -0.3460 | 4.82e-16 | 5.30e-14 |
| ENSMUSG00000041324 | Inhba | protein\_coding | 13:16011851-16031621 (+) |  | -3.0300 | 5.07e-16 | 5.51e-14 |
| ENSMUSG00000033287 | Kctd17 | protein\_coding | 15:78428564-78439303 (+) |  | -0.6090 | 5.08e-16 | 5.51e-14 |
| ENSMUSG00000054766 | Set | protein\_coding | 2:30057378-30072577 (+) |  | -0.4880 | 5.28e-16 | 5.67e-14 |
| ENSMUSG00000036528 | Ppfibp2 | protein\_coding | 7:107595207-107748583 (+) |  | 0.8500 | 8.43e-16 | 8.99e-14 |
| ENSMUSG00000015766 | Eps8 | protein\_coding | 6:137477245-137654876 (-) |  | -0.8230 | 9.20e-16 | 9.74e-14 |
| ENSMUSG00000052459 | Atp6v1a | protein\_coding | 16:44085402-44139705 (-) |  | -0.4100 | 9.44e-16 | 9.93e-14 |
| ENSMUSG00000024855 | Pacs1 | protein\_coding | 19:5133158-5273119 (-) |  | 0.5440 | 1.02e-15 | 1.07e-13 |
| ENSMUSG00000045193 | Cirbp | protein\_coding | 10:80165985-80172786 (+) |  | 0.5840 | 1.06e-15 | 1.10e-13 |
| ENSMUSG00000028927 | Padi2 | protein\_coding | 4:140906344-140952586 (+) |  | 1.3000 | 1.20e-15 | 1.23e-13 |
| ENSMUSG00000029922 | Mkrn1 | protein\_coding | 6:39397804-39420462 (-) |  | 0.4140 | 1.21e-15 | 1.23e-13 |
| ENSMUSG00000040820 | Hlcs | protein\_coding | 16:94128882-94313571 (-) |  | 0.6810 | 1.22e-15 | 1.23e-13 |
| ENSMUSG00000071714 | Csf2rb2 | protein\_coding | 15:78282507-78305721 (-) |  | -0.5470 | 1.23e-15 | 1.24e-13 |
| ENSMUSG00000002997 | Prkar2b | protein\_coding | 12:31958476-32061296 (-) |  | 0.3390 | 1.26e-15 | 1.26e-13 |
| ENSMUSG00000001229 | Dpp9 | protein\_coding | 17:56186807-56218905 (-) |  | -0.5790 | 1.52e-15 | 1.51e-13 |
| ENSMUSG00000021190 | Lgmn | protein\_coding | 12:102394084-102439813 (-) |  | -0.5810 | 1.55e-15 | 1.53e-13 |
| ENSMUSG00000026121 | Sema4c | protein\_coding | 1:36548639-36558349 (-) |  | 1.5100 | 1.57e-15 | 1.54e-13 |
| ENSMUSG00000023571 | C1qtnf12 | protein\_coding | 4:155962318-155966629 (+) |  | 0.6780 | 1.81e-15 | 1.76e-13 |
| ENSMUSG00000037989 | Wnk2 | protein\_coding | 13:49036303-49148014 (-) |  | 2.1700 | 1.82e-15 | 1.76e-13 |
| ENSMUSG00000014470 | Rnf166 | protein\_coding | 8:122466147-122476064 (-) |  | 0.5190 | 1.91e-15 | 1.83e-13 |
| ENSMUSG00000038970 | Lmtk2 | protein\_coding | 5:144100436-144188204 (+) |  | 0.7240 | 1.93e-15 | 1.85e-13 |
| ENSMUSG00000044468 | Tent5c | protein\_coding | 3:100451628-100489324 (-) |  | 1.3600 | 2.18e-15 | 2.07e-13 |
| ENSMUSG00000019823 | Mical1 | protein\_coding | 10:41476314-41487032 (+) |  | 0.4800 | 2.21e-15 | 2.08e-13 |
| ENSMUSG00000038831 | Ralgps1 | protein\_coding | 2:33133417-33371486 (-) |  | 1.0300 | 2.22e-15 | 2.08e-13 |
| ENSMUSG00000030123 | Plxnd1 | protein\_coding | 6:115954811-115995005 (-) |  | 0.5800 | 2.48e-15 | 2.31e-13 |
| ENSMUSG00000036112 | Metap2 | protein\_coding | 10:93858489-93897093 (-) |  | -0.4410 | 2.75e-15 | 2.54e-13 |
| ENSMUSG00000049999 | Ppp1r3d | protein\_coding | 2:178411206-178414472 (-) |  | -0.8510 | 2.96e-15 | 2.71e-13 |
| ENSMUSG00000039005 | Tlr4 | protein\_coding | 4:66827584-66930284 (+) |  | -0.7900 | 2.97e-15 | 2.71e-13 |
| ENSMUSG00000032902 | Slc16a1 | protein\_coding | 3:104638668-104658462 (+) |  | -1.0400 | 3.06e-15 | 2.77e-13 |
| ENSMUSG00000036304 | Zdhhc23 | protein\_coding | 16:43965033-43979791 (-) |  | -0.8300 | 3.13e-15 | 2.82e-13 |
| ENSMUSG00000024966 | Stip1 | protein\_coding | 19:7020702-7039967 (-) |  | -0.4800 | 3.47e-15 | 3.11e-13 |
| ENSMUSG00000027775 | Mfsd1 | protein\_coding | 3:67582741-67604237 (+) |  | -0.3120 | 3.49e-15 | 3.11e-13 |
| ENSMUSG00000026275 | Ppp1r7 | protein\_coding | 1:93342854-93373489 (+) |  | -0.3750 | 3.60e-15 | 3.19e-13 |
| ENSMUSG00000019960 | Dusp6 | protein\_coding | 10:99263231-99267489 (+) |  | 1.9000 | 4.53e-15 | 3.98e-13 |
| ENSMUSG00000025151 | Maged1 | protein\_coding | X:94535474-94542143 (-) |  | -1.5500 | 4.62e-15 | 4.04e-13 |
| ENSMUSG00000020787 | P2rx1 | protein\_coding | 11:72999103-73015200 (+) |  | 1.0900 | 5.93e-15 | 5.15e-13 |
| ENSMUSG00000029430 | Ran | protein\_coding | 5:129020069-129024323 (+) |  | -0.5410 | 6.20e-15 | 5.36e-13 |
| ENSMUSG00000020682 | Mmp28 | protein\_coding | 11:83440768-83463071 (-) |  | 1.0500 | 6.77e-15 | 5.81e-13 |
| ENSMUSG00000035901 | Dennd5a | protein\_coding | 7:109893780-109960470 (-) |  | 0.3050 | 6.93e-15 | 5.92e-13 |
| ENSMUSG00000032575 | Manf | protein\_coding | 9:106838312-106891979 (-) |  | -0.5300 | 7.36e-15 | 6.24e-13 |
| ENSMUSG00000025150 | Cbr2 | protein\_coding | 11:120729489-120732114 (-) |  | 1.6200 | 7.50e-15 | 6.33e-13 |
| ENSMUSG00000032946 | Rasgrp2 | protein\_coding | 19:6399340-6415216 (+) |  | 0.3840 | 7.64e-15 | 6.41e-13 |
| ENSMUSG00000011884 | Gltp | protein\_coding | 5:114669398-114690984 (-) |  | 0.4080 | 7.89e-15 | 6.58e-13 |
| ENSMUSG00000035165 | Kcne3 | protein\_coding | 7:100176502-100184869 (+) |  | 1.1400 | 8.30e-15 | 6.88e-13 |
| ENSMUSG00000053113 | Socs3 | protein\_coding | 11:117966079-117970047 (-) |  | -2.9600 | 8.78e-15 | 7.24e-13 |
| ENSMUSG00000061353 | Cxcl12 | protein\_coding | 6:117168535-117181367 (+) |  | 2.1200 | 8.95e-15 | 7.34e-13 |
| ENSMUSG00000026170 | Cyp27a1 | protein\_coding | 1:74713574-74737892 (+) |  | 0.9970 | 9.03e-15 | 7.36e-13 |
| ENSMUSG00000020250 | Txnrd1 | protein\_coding | 10:82833951-82897712 (+) |  | -0.5120 | 9.28e-15 | 7.52e-13 |
| ENSMUSG00000050244 | Heatr1 | protein\_coding | 13:12395027-12440289 (+) |  | -0.4850 | 9.33e-15 | 7.52e-13 |
| ENSMUSG00000029082 | Bst1 | protein\_coding | 5:43818885-43843986 (+) |  | -1.6000 | 1.08e-14 | 8.68e-13 |
| ENSMUSG00000018583 | G3bp1 | protein\_coding | 11:55469685-55504838 (+) |  | -0.3500 | 1.09e-14 | 8.69e-13 |
| ENSMUSG00000091811 | Inafm1 | protein\_coding | 7:16272013-16273617 (-) |  | 0.6930 | 1.11e-14 | 8.81e-13 |
| ENSMUSG00000117613 | Gm2629 | lncRNA | 18:15194782-15214696 (+) |  | 0.8510 | 1.29e-14 | 1.02e-12 |
| ENSMUSG00000032411 | Tfdp2 | protein\_coding | 9:96196275-96323646 (+) |  | 0.5300 | 1.38e-14 | 1.08e-12 |
| ENSMUSG00000035673 | Sbno2 | protein\_coding | 10:80056992-80105571 (-) |  | -0.4580 | 1.40e-14 | 1.09e-12 |
| ENSMUSG00000063193 | Cd300lb | protein\_coding | 11:114922781-114934386 (-) |  | 0.6030 | 1.42e-14 | 1.10e-12 |
| ENSMUSG00000029657 | Hsph1 | protein\_coding | 5:149614287-149636376 (-) |  | -0.8410 | 1.67e-14 | 1.29e-12 |
| ENSMUSG00000025155 | Dus1l | protein\_coding | 11:120789201-120796403 (-) |  | -0.3020 | 1.68e-14 | 1.29e-12 |
| ENSMUSG00000062585 | Cnr2 | protein\_coding | 4:135895394-135920207 (+) |  | 0.5630 | 2.03e-14 | 1.55e-12 |
| ENSMUSG00000032724 | Abtb2 | protein\_coding | 2:103566310-103718423 (+) |  | -1.0400 | 2.06e-14 | 1.57e-12 |
| ENSMUSG00000021486 | Prelid1 | protein\_coding | 13:55320500-55325272 (+) |  | -0.4640 | 2.07e-14 | 1.57e-12 |
| ENSMUSG00000011256 | Adam19 | protein\_coding | 11:46055992-46147343 (+) |  | 1.9500 | 2.10e-14 | 1.58e-12 |
| ENSMUSG00000051748 | Wfdc21 | protein\_coding | 11:83746940-83752642 (+) |  | -3.4800 | 2.53e-14 | 1.89e-12 |
| ENSMUSG00000000673 | Haao | protein\_coding | 17:83831156-83847963 (-) |  | 0.6740 | 2.66e-14 | 1.98e-12 |
| ENSMUSG00000027315 | Spint1 | protein\_coding | 2:119237362-119249527 (+) |  | -4.9100 | 2.76e-14 | 2.04e-12 |
| ENSMUSG00000039159 | Ube2h | protein\_coding | 6:30211289-30304539 (-) |  | -0.5920 | 2.81e-14 | 2.07e-12 |
| ENSMUSG00000038070 | Cntln | protein\_coding | 4:84884309-85131921 (+) |  | 0.5760 | 2.84e-14 | 2.08e-12 |
| ENSMUSG00000038481 | Cdk19 | protein\_coding | 10:40339564-40483818 (+) |  | 0.2970 | 3.04e-14 | 2.22e-12 |
| ENSMUSG00000051811 | Cox6b2 | protein\_coding | 7:4751792-4753094 (-) |  | 0.8860 | 3.13e-14 | 2.27e-12 |
| ENSMUSG00000017756 | Slc12a7 | protein\_coding | 13:73733094-73816754 (+) |  | -0.8460 | 3.56e-14 | 2.57e-12 |
| ENSMUSG00000067367 | Lyar | protein\_coding | 5:38220470-38234306 (+) |  | -0.5430 | 3.62e-14 | 2.60e-12 |
| ENSMUSG00000038463 | Olfml2b | protein\_coding | 1:170644532-170682789 (+) |  | -2.5400 | 3.78e-14 | 2.70e-12 |
| ENSMUSG00000011179 | Odc1 | protein\_coding | 12:17544794-17551505 (+) |  | -0.6930 | 4.01e-14 | 2.85e-12 |
| ENSMUSG00000057789 | Bak1 | protein\_coding | 17:27019810-27029009 (-) |  | -0.4250 | 4.84e-14 | 3.42e-12 |
| ENSMUSG00000051832 | E230016K23Rik | lncRNA | 11:83582056-83623693 (+) |  | 1.2600 | 4.86e-14 | 3.42e-12 |
| ENSMUSG00000032531 | Amotl2 | protein\_coding | 9:102716672-102733418 (+) |  | -1.1700 | 4.88e-14 | 3.42e-12 |
| ENSMUSG00000068039 | Tcp1 | protein\_coding | 17:12915701-12925067 (+) |  | -0.3720 | 5.00e-14 | 3.48e-12 |
| ENSMUSG00000024063 | Lbh | protein\_coding | 17:72918305-72941947 (+) |  | 0.4930 | 5.02e-14 | 3.48e-12 |
| ENSMUSG00000042757 | Tmem108 | protein\_coding | 9:103482947-103761837 (-) |  | -2.2600 | 5.27e-14 | 3.64e-12 |
| ENSMUSG00000047866 | Lonp2 | protein\_coding | 8:86624043-86723873 (+) |  | -0.4500 | 5.54e-14 | 3.81e-12 |
| ENSMUSG00000024732 | Ccdc86 | protein\_coding | 19:10941481-10949266 (-) |  | -0.6120 | 5.81e-14 | 3.98e-12 |
| ENSMUSG00000030605 | Mfge8 | protein\_coding | 7:79133768-79149060 (-) |  | 0.6450 | 6.19e-14 | 4.22e-12 |
| ENSMUSG00000021831 | Ero1l | protein\_coding | 14:45283087-45318771 (-) |  | -0.4920 | 6.41e-14 | 4.34e-12 |
| ENSMUSG00000027184 | Caprin1 | protein\_coding | 2:103762941-103797649 (-) |  | -0.2390 | 6.49e-14 | 4.38e-12 |
| ENSMUSG00000038235 | F11r | protein\_coding | 1:171437535-171464603 (+) |  | -3.8200 | 6.65e-14 | 4.46e-12 |
| ENSMUSG00000028614 | Ndc1 | protein\_coding | 4:107367784-107416346 (+) |  | -0.4470 | 7.07e-14 | 4.73e-12 |
| ENSMUSG00000056124 | B4galt6 | protein\_coding | 18:20684599-20746404 (-) |  | -1.3200 | 7.22e-14 | 4.80e-12 |
| ENSMUSG00000024238 | Zeb1 | protein\_coding | 18:5591860-5775467 (+) |  | 1.1800 | 7.41e-14 | 4.91e-12 |
| ENSMUSG00000036138 | Acaa1a | protein\_coding | 9:119339676-119350299 (+) |  | -0.4310 | 7.60e-14 | 5.01e-12 |
| ENSMUSG00000040327 | Cul9 | protein\_coding | 17:46500572-46546388 (-) |  | 0.7440 | 8.23e-14 | 5.40e-12 |
| ENSMUSG00000072694 | 1500011B03Rik | protein\_coding | 5:114808196-114823468 (-) |  | 0.5460 | 8.62e-14 | 5.64e-12 |
| ENSMUSG00000060962 | Dmkn | protein\_coding | 7:30763756-30781063 (+) |  | -2.2500 | 8.71e-14 | 5.64e-12 |
| ENSMUSG00000057948 | Unc13d | protein\_coding | 11:116062095-116077961 (-) |  | 0.3970 | 8.71e-14 | 5.64e-12 |
| ENSMUSG00000001054 | Rmnd5b | protein\_coding | 11:51623671-51635896 (-) |  | 0.4330 | 1.23e-13 | 7.95e-12 |
| ENSMUSG00000031494 | Cd209a | protein\_coding | 8:3743397-3748984 (-) |  | 2.4700 | 1.30e-13 | 8.37e-12 |
| ENSMUSG00000001440 | Kpnb1 | protein\_coding | 11:97159714-97187881 (-) |  | -0.4140 | 1.33e-13 | 8.52e-12 |
| ENSMUSG00000038271 | Iffo1 | protein\_coding | 6:125145241-125161782 (+) |  | 0.6170 | 1.34e-13 | 8.53e-12 |
| ENSMUSG00000030493 | Faap24 | protein\_coding | 7:35392152-35396836 (-) |  | -0.5800 | 1.48e-13 | 9.41e-12 |
| ENSMUSG00000009585 | Apobec3 | protein\_coding | 15:79891659-79915906 (+) |  | -0.4360 | 1.59e-13 | 9.98e-12 |
| ENSMUSG00000031722 | Hp | protein\_coding | 8:109575128-109579172 (-) |  | -0.5540 | 1.59e-13 | 9.98e-12 |
| ENSMUSG00000035891 | Cerk | protein\_coding | 15:86139128-86186141 (-) |  | 0.4430 | 1.73e-13 | 1.08e-11 |
| ENSMUSG00000066687 | Zbtb16 | protein\_coding | 9:48654297-48836222 (-) |  | 1.7800 | 1.73e-13 | 1.08e-11 |
| ENSMUSG00000039682 | Lap3 | protein\_coding | 5:45493374-45512691 (+) |  | -0.5720 | 1.74e-13 | 1.08e-11 |
| ENSMUSG00000022574 | Naprt | protein\_coding | 15:75890956-75894481 (-) |  | -1.6400 | 1.76e-13 | 1.09e-11 |
| ENSMUSG00000006378 | Gcat | protein\_coding | 15:79030874-79038353 (+) |  | -1.2400 | 1.79e-13 | 1.10e-11 |
| ENSMUSG00000031838 | Ifi30 | lncRNA | 8:70762774-70766663 (-) |  | -0.5430 | 1.88e-13 | 1.15e-11 |
| ENSMUSG00000009291 | Pttg1ip | protein\_coding | 10:77581720-77598732 (+) |  | 0.3170 | 2.00e-13 | 1.22e-11 |
| ENSMUSG00000026478 | Lamc1 | protein\_coding | 1:153218922-153332786 (-) |  | -2.5000 | 2.02e-13 | 1.23e-11 |
| ENSMUSG00000031948 | Kars | protein\_coding | 8:111993443-112011323 (-) |  | -0.3480 | 2.08e-13 | 1.26e-11 |
| ENSMUSG00000004655 | Aqp1 | protein\_coding | 6:55336432-55348555 (+) |  | 2.0800 | 2.18e-13 | 1.31e-11 |
| ENSMUSG00000030681 | Mvp | protein\_coding | 7:126986860-127014621 (-) |  | -0.4410 | 2.36e-13 | 1.41e-11 |
| ENSMUSG00000022389 | Tef | protein\_coding | 15:81802421-81826863 (+) |  | 1.2000 | 2.43e-13 | 1.45e-11 |
| ENSMUSG00000005824 | Tnfsf14 | protein\_coding | 17:57189492-57194177 (-) |  | -0.6330 | 2.65e-13 | 1.58e-11 |
| ENSMUSG00000048924 | Ccdc125 | protein\_coding | 13:100669717-100697240 (+) |  | 0.5030 | 2.68e-13 | 1.59e-11 |
| ENSMUSG00000008206 | Cers4 | protein\_coding | 8:4493026-4531680 (+) |  | 1.3300 | 2.69e-13 | 1.59e-11 |
| ENSMUSG00000023947 | Nfkbie | protein\_coding | 17:45555703-45563169 (+) |  | 0.8360 | 2.96e-13 | 1.74e-11 |
| ENSMUSG00000074656 | Eif2s2 | protein\_coding | 2:154871410-154892935 (-) |  | -0.5220 | 3.07e-13 | 1.80e-11 |
| ENSMUSG00000028523 | Tctex1d1 | protein\_coding | 4:102978606-103005594 (+) |  | -2.6400 | 3.14e-13 | 1.83e-11 |
| ENSMUSG00000024070 | Prkd3 | protein\_coding | 17:78949405-79020816 (-) |  | 0.2990 | 3.15e-13 | 1.83e-11 |
| ENSMUSG00000042524 | Sun2 | protein\_coding | 15:79724070-79742536 (-) |  | 0.3860 | 3.19e-13 | 1.84e-11 |
| ENSMUSG00000021178 | Psmc1 | protein\_coding | 12:100110154-100123405 (+) |  | -0.4390 | 3.19e-13 | 1.84e-11 |
| ENSMUSG00000022003 | Slc25a30 | protein\_coding | 14:75760117-75787037 (-) |  | -0.4150 | 3.21e-13 | 1.84e-11 |
| ENSMUSG00000004609 | Cd33 | protein\_coding | 7:43524216-43544428 (-) |  | 0.9520 | 3.35e-13 | 1.91e-11 |
| ENSMUSG00000044103 | Il1f9 | protein\_coding | 2:24186476-24193568 (+) |  | -3.7200 | 3.46e-13 | 1.97e-11 |
| ENSMUSG00000030413 | Pglyrp1 | protein\_coding | 7:18871331-18890459 (+) |  | 1.1100 | 3.48e-13 | 1.97e-11 |
| ENSMUSG00000005142 | Man2b1 | protein\_coding | 8:85083270-85098282 (+) |  | 0.4080 | 3.75e-13 | 2.12e-11 |
| ENSMUSG00000018340 | Anxa6 | protein\_coding | 11:54979108-55033445 (-) |  | 0.3670 | 3.84e-13 | 2.16e-11 |
| ENSMUSG00000030711 | Sult1a1 | protein\_coding | 7:126672865-126676432 (-) |  | 1.4700 | 4.35e-13 | 2.43e-11 |
| ENSMUSG00000062203 | Gspt1 | protein\_coding | 16:11219292-11254325 (-) |  | -0.3990 | 4.62e-13 | 2.58e-11 |
| ENSMUSG00000040532 | Abhd11 | protein\_coding | 5:135009152-135012175 (+) |  | -0.5540 | 4.65e-13 | 2.59e-11 |
| ENSMUSG00000022353 | Mtss1 | protein\_coding | 15:58941234-59082005 (-) |  | 1.2400 | 4.75e-13 | 2.63e-11 |
| ENSMUSG00000062906 | Hdac10 | protein\_coding | 15:89123307-89128700 (-) |  | 0.6860 | 4.89e-13 | 2.70e-11 |
| ENSMUSG00000029304 | Spp1 | protein\_coding | 5:104435118-104441050 (+) |  | -0.9460 | 5.06e-13 | 2.77e-11 |
| ENSMUSG00000020089 | Ppa1 | protein\_coding | 10:61648552-61674168 (+) |  | -0.8210 | 5.08e-13 | 2.77e-11 |
| ENSMUSG00000016206 | H2-M3 | protein\_coding | 17:37270220-37274484 (+) |  | -0.4240 | 5.09e-13 | 2.77e-11 |
| ENSMUSG00000032089 | Il10ra | protein\_coding | 9:45253837-45269149 (-) |  | 0.4790 | 5.31e-13 | 2.88e-11 |
| ENSMUSG00000023055 | Calcoco1 | protein\_coding | 15:102706777-102722178 (-) |  | 0.8210 | 5.69e-13 | 3.08e-11 |
| ENSMUSG00000020592 | Sdc1 | protein\_coding | 12:8771323-8793715 (+) |  | -1.4300 | 6.41e-13 | 3.46e-11 |
| ENSMUSG00000001763 | Tspan33 | protein\_coding | 6:29694222-29718559 (+) |  | 0.9810 | 6.53e-13 | 3.51e-11 |
| ENSMUSG00000022564 | Grina | protein\_coding | 15:76246764-76249904 (+) |  | -0.5070 | 6.94e-13 | 3.71e-11 |
| ENSMUSG00000008450 | Nutf2 | protein\_coding | 8:105860580-105879330 (+) |  | -0.5740 | 7.05e-13 | 3.76e-11 |
| ENSMUSG00000058355 | Abce1 | protein\_coding | 8:79683462-79711740 (-) |  | -0.4360 | 7.25e-13 | 3.85e-11 |
| ENSMUSG00000115338 | Pnp | protein\_coding | 14:50931082-50965237 (+) |  | -0.5340 | 7.26e-13 | 3.85e-11 |
| ENSMUSG00000082099 | Gm12013 | processed\_pseudogene | 11:17095490-17095644 (-) |  | 1.4200 | 7.35e-13 | 3.88e-11 |
| ENSMUSG00000079547 | H2-DMb1 | protein\_coding | 17:34153072-34160230 (+) |  | 0.6290 | 7.81e-13 | 4.11e-11 |
| ENSMUSG00000079225 | Gm9531 | transcribed\_processed\_pseudogene | 9:81677598-81678677 (+) |  | -0.4380 | 7.86e-13 | 4.12e-11 |
| ENSMUSG00000061458 | Nol10 | protein\_coding | 12:17348458-17430095 (+) |  | -0.4180 | 8.35e-13 | 4.36e-11 |
| ENSMUSG00000003541 | Ier3 | protein\_coding | 17:35821684-35822923 (+) |  | -1.1600 | 8.78e-13 | 4.56e-11 |
| ENSMUSG00000028524 | Sgip1 | protein\_coding | 4:102741297-102973628 (+) |  | -1.5400 | 8.91e-13 | 4.60e-11 |
| ENSMUSG00000086291 | Gm15513 | lncRNA | 5:34211810-34213802 (-) |  | 1.6800 | 8.92e-13 | 4.60e-11 |
| ENSMUSG00000030468 | Siglecg | protein\_coding | 7:43408204-43418358 (+) |  | 0.7870 | 9.14e-13 | 4.70e-11 |
| ENSMUSG00000002984 | Tomm40 | protein\_coding | 7:19701313-19715438 (-) |  | -0.4580 | 9.50e-13 | 4.87e-11 |
| ENSMUSG00000000753 | Serpinf1 | protein\_coding | 11:75409769-75422701 (-) |  | 0.7690 | 9.63e-13 | 4.92e-11 |
| ENSMUSG00000056592 | Zfp658 | protein\_coding | 7:43562256-43575461 (+) |  | 0.8450 | 1.00e-12 | 5.11e-11 |
| ENSMUSG00000015947 | Fcgr1 | protein\_coding | 3:96282909-96293969 (-) |  | -1.3700 | 1.07e-12 | 5.45e-11 |
| ENSMUSG00000052310 | Slc39a1 | protein\_coding | 3:90248172-90253612 (+) |  | -0.3540 | 1.08e-12 | 5.47e-11 |
| ENSMUSG00000053063 | Clec12a | protein\_coding | 6:129342691-129365303 (+) |  | 0.6170 | 1.12e-12 | 5.62e-11 |
| ENSMUSG00000046562 | Unc119b | protein\_coding | 5:115122550-115134975 (-) |  | 0.4590 | 1.14e-12 | 5.74e-11 |
| ENSMUSG00000042660 | Wdr55 | protein\_coding | 18:36760220-36763810 (+) |  | -0.4790 | 1.16e-12 | 5.79e-11 |
| ENSMUSG00000020834 | Dhrs13 | protein\_coding | 11:78032280-78037866 (+) |  | -0.7750 | 1.17e-12 | 5.82e-11 |
| ENSMUSG00000028270 | Gbp2 | protein\_coding | 3:142620602-142638008 (+) |  | -0.9230 | 1.17e-12 | 5.82e-11 |
| ENSMUSG00000000759 | Tubgcp3 | protein\_coding | 8:12614277-12672248 (-) |  | -0.3350 | 1.18e-12 | 5.85e-11 |
| ENSMUSG00000027496 | Aurka | protein\_coding | 2:172356190-172370535 (-) |  | -0.3340 | 1.20e-12 | 5.93e-11 |
| ENSMUSG00000070319 | Eif3g | protein\_coding | 9:20894349-20898623 (-) |  | -0.3270 | 1.21e-12 | 5.95e-11 |
| ENSMUSG00000031495 | Cd209d | protein\_coding | 8:3871824-3878555 (-) |  | 2.4800 | 1.27e-12 | 6.23e-11 |
| ENSMUSG00000018377 | Vezf1 | protein\_coding | 11:88068279-88084729 (+) |  | 0.3850 | 1.28e-12 | 6.28e-11 |
| ENSMUSG00000025817 | Nudt5 | protein\_coding | 2:5845019-5871895 (+) |  | -0.4650 | 1.39e-12 | 6.75e-11 |
| ENSMUSG00000028617 | Lrrc42 | protein\_coding | 4:107233514-107253532 (-) |  | -0.5750 | 1.40e-12 | 6.81e-11 |
| ENSMUSG00000028268 | Gbp3 | protein\_coding | 3:142560026-142573209 (+) |  | -0.6200 | 1.47e-12 | 7.12e-11 |
| ENSMUSG00000040521 | Tsfm | protein\_coding | 10:127011572-127030840 (-) |  | -0.4270 | 1.60e-12 | 7.70e-11 |
| ENSMUSG00000063354 | Slc39a4 | protein\_coding | 15:76612383-76617384 (-) |  | 2.5800 | 1.60e-12 | 7.70e-11 |
| ENSMUSG00000021048 | Mthfd1 | protein\_coding | 12:76255298-76319803 (+) |  | -0.5750 | 1.74e-12 | 8.34e-11 |
| ENSMUSG00000027778 | Ift80 | protein\_coding | 3:68892499-69004570 (-) |  | -0.6000 | 1.77e-12 | 8.43e-11 |
| ENSMUSG00000005667 | Mthfd2 | protein\_coding | 6:83305691-83325908 (-) |  | -0.4630 | 1.82e-12 | 8.66e-11 |
| ENSMUSG00000019820 | Utrn | protein\_coding | 10:12382188-12869365 (-) |  | 0.6220 | 1.89e-12 | 8.96e-11 |
| ENSMUSG00000046080 | Clec9a | protein\_coding | 6:129408862-129424763 (+) |  | 0.9910 | 2.17e-12 | 1.03e-10 |
| ENSMUSG00000011752 | Pgam1 | protein\_coding | 19:41911923-41918660 (+) |  | -0.4520 | 2.36e-12 | 1.11e-10 |
| ENSMUSG00000031486 | Adgra2 | protein\_coding | 8:27085583-27123436 (+) |  | 1.4700 | 2.38e-12 | 1.12e-10 |
| ENSMUSG00000074129 | Rpl13a | protein\_coding | 7:45125558-45128761 (-) |  | -0.5540 | 2.43e-12 | 1.14e-10 |
| ENSMUSG00000066952 | Myo1h | protein\_coding | 5:114289166-114365357 (+) |  | 1.2300 | 2.44e-12 | 1.14e-10 |
| ENSMUSG00000008373 | Prpf31 | protein\_coding | 7:3629985-3642486 (+) |  | -0.3860 | 2.47e-12 | 1.15e-10 |
| ENSMUSG00000003955 | Fam162a | protein\_coding | 16:36043761-36071594 (-) |  | -0.5870 | 2.50e-12 | 1.16e-10 |
| ENSMUSG00000040263 | Klhdc4 | protein\_coding | 8:121796313-121829569 (-) |  | -0.3770 | 2.63e-12 | 1.22e-10 |
| ENSMUSG00000028156 | Eif4e | protein\_coding | 3:138526179-138559696 (+) |  | -0.3900 | 2.73e-12 | 1.26e-10 |
| ENSMUSG00000062991 | Nrg1 | protein\_coding | 8:31814551-32884797 (-) |  | -0.5350 | 2.75e-12 | 1.26e-10 |
| ENSMUSG00000027248 | Pdia3 | protein\_coding | 2:121413775-121438687 (+) |  | -0.4550 | 2.77e-12 | 1.27e-10 |
| ENSMUSG00000010755 | Cars | protein\_coding | 7:143557230-143600090 (-) |  | -0.4550 | 2.83e-12 | 1.29e-10 |
| ENSMUSG00000032554 | Trf | protein\_coding | 9:103204001-103230444 (-) |  | 0.8060 | 2.99e-12 | 1.36e-10 |
| ENSMUSG00000026020 | Nop58 | protein\_coding | 1:59684971-59719044 (+) |  | -0.5130 | 3.02e-12 | 1.37e-10 |
| ENSMUSG00000034024 | Cct2 | protein\_coding | 10:117051001-117063814 (-) |  | -0.3740 | 3.13e-12 | 1.41e-10 |
| ENSMUSG00000005732 | Ranbp1 | protein\_coding | 16:18239784-18248732 (-) |  | -0.5150 | 3.21e-12 | 1.45e-10 |
| ENSMUSG00000038872 | Zfhx3 | protein\_coding | 8:107942644-108961630 (+) |  | 0.8980 | 3.31e-12 | 1.49e-10 |
| ENSMUSG00000020423 | Btg2 | protein\_coding | 1:134075170-134079120 (-) |  | 0.8940 | 3.42e-12 | 1.53e-10 |
| ENSMUSG00000024948 | Map4k2 | protein\_coding | 19:6341135-6355615 (+) |  | 0.5250 | 3.49e-12 | 1.56e-10 |
| ENSMUSG00000024587 | Nars | protein\_coding | 18:64499647-64516652 (-) |  | -0.4140 | 3.50e-12 | 1.56e-10 |
| ENSMUSG00000025270 | Alas2 | protein\_coding | X:150547375-150570638 (+) |  | 2.3200 | 3.55e-12 | 1.57e-10 |
| ENSMUSG00000037321 | Tap1 | protein\_coding | 17:34187553-34197225 (+) |  | -0.3730 | 3.63e-12 | 1.61e-10 |
| ENSMUSG00000003235 | Eif2b5 | protein\_coding | 16:20498817-20509323 (+) |  | -0.3400 | 3.72e-12 | 1.64e-10 |
| ENSMUSG00000021087 | Rtn1 | protein\_coding | 12:72211752-72409054 (-) |  | 1.3600 | 3.80e-12 | 1.67e-10 |
| ENSMUSG00000070476 | Fam217b | protein\_coding | 2:178414524-178424428 (+) |  | -0.5400 | 3.84e-12 | 1.68e-10 |
| ENSMUSG00000018363 | Smurf2 | protein\_coding | 11:106820066-106920715 (-) |  | 0.3360 | 3.90e-12 | 1.70e-10 |
| ENSMUSG00000038482 | Tfdp1 | protein\_coding | 8:13338751-13378448 (+) |  | -0.3400 | 3.91e-12 | 1.70e-10 |
| ENSMUSG00000032123 | Dpagt1 | protein\_coding | 9:44326019-44333900 (+) |  | -0.3690 | 3.94e-12 | 1.71e-10 |
| ENSMUSG00000024841 | Eif1ad | protein\_coding | 19:5366741-5371526 (+) |  | -0.3310 | 4.02e-12 | 1.74e-10 |
| ENSMUSG00000034226 | Rhov | protein\_coding | 2:119269201-119271272 (-) |  | -3.3100 | 4.04e-12 | 1.75e-10 |
| ENSMUSG00000030867 | Plk1 | protein\_coding | 7:122159439-122169873 (+) |  | -0.4420 | 4.11e-12 | 1.77e-10 |
| ENSMUSG00000063273 | Naa15 | protein\_coding | 3:51415148-51476507 (+) |  | -0.3420 | 4.15e-12 | 1.78e-10 |
| ENSMUSG00000024986 | Hhex | protein\_coding | 19:37434810-37440731 (+) |  | 0.3770 | 4.46e-12 | 1.91e-10 |
| ENSMUSG00000020075 | Ddx21 | protein\_coding | 10:62580251-62602281 (-) |  | -0.3750 | 4.74e-12 | 2.02e-10 |
| ENSMUSG00000044788 | Fads6 | protein\_coding | 11:115279622-115297663 (-) |  | 0.8940 | 4.91e-12 | 2.09e-10 |
| ENSMUSG00000002017 | Fam98a | protein\_coding | 17:75537086-75551946 (-) |  | -0.4010 | 4.96e-12 | 2.10e-10 |
| ENSMUSG00000031960 | Aars | protein\_coding | 8:111033144-111057664 (+) |  | -0.3990 | 4.97e-12 | 2.10e-10 |
| ENSMUSG00000017466 | Timp2 | protein\_coding | 11:118301069-118355740 (-) |  | 1.0500 | 4.99e-12 | 2.11e-10 |
| ENSMUSG00000041360 | Pum3 | protein\_coding | 19:27388698-27429825 (-) |  | -0.3380 | 5.03e-12 | 2.12e-10 |
| ENSMUSG00000030089 | Slc41a3 | protein\_coding | 6:90604725-90646412 (+) |  | 0.7530 | 5.13e-12 | 2.15e-10 |
| ENSMUSG00000018446 | C1qbp | protein\_coding | 11:70977836-70983026 (-) |  | -0.6140 | 5.31e-12 | 2.22e-10 |
| ENSMUSG00000037601 | Nme1 | protein\_coding | 11:93956979-93968521 (-) |  | -0.5430 | 5.40e-12 | 2.25e-10 |
| ENSMUSG00000099775 | Gm5960 | unprocessed\_pseudogene | 15:75071112-75074638 (+) |  | 0.8110 | 5.60e-12 | 2.33e-10 |
| ENSMUSG00000056069 | Otulinl | protein\_coding | 15:27655069-27681579 (-) |  | 0.3650 | 5.65e-12 | 2.34e-10 |
| ENSMUSG00000045658 | Pid1 | protein\_coding | 1:84036296-84364180 (-) |  | 0.8290 | 5.73e-12 | 2.37e-10 |
| ENSMUSG00000051329 | Nup160 | protein\_coding | 2:90677215-90736328 (+) |  | -0.3820 | 6.10e-12 | 2.51e-10 |
| ENSMUSG00000079037 | Prnp | protein\_coding | 2:131909928-131938429 (+) |  | -2.1500 | 6.19e-12 | 2.55e-10 |
| ENSMUSG00000045322 | Tlr9 | protein\_coding | 9:106222598-106226883 (+) |  | 0.5460 | 6.26e-12 | 2.56e-10 |
| ENSMUSG00000047798 | Cd300lf | protein\_coding | 11:115116214-115133992 (-) |  | -0.8340 | 6.39e-12 | 2.61e-10 |
| ENSMUSG00000020272 | Stk10 | protein\_coding | 11:32533305-32624587 (+) |  | 0.5120 | 6.51e-12 | 2.65e-10 |
| ENSMUSG00000034075 | Zdhhc5 | protein\_coding | 2:84687970-84715180 (-) |  | -0.2630 | 6.98e-12 | 2.84e-10 |
| ENSMUSG00000023045 | Soat2 | protein\_coding | 15:102150526-102163469 (+) |  | -1.5500 | 7.07e-12 | 2.87e-10 |
| ENSMUSG00000000561 | Wdr77 | protein\_coding | 3:105959369-105970037 (+) |  | -0.3950 | 7.29e-12 | 2.94e-10 |
| ENSMUSG00000030223 | Ptpro | protein\_coding | 6:137252319-137463233 (+) |  | 0.6130 | 7.33e-12 | 2.95e-10 |
| ENSMUSG00000034858 | Fam214a | protein\_coding | 9:74952884-75032468 (+) |  | 1.1300 | 7.45e-12 | 2.99e-10 |
| ENSMUSG00000053219 | Raet1e | protein\_coding | 10:22158569-22374139 (+) |  | -1.5200 | 7.61e-12 | 3.05e-10 |
| ENSMUSG00000038456 | Dennd2a | protein\_coding | 6:39462378-39557867 (-) |  | 0.8870 | 7.85e-12 | 3.14e-10 |
| ENSMUSG00000030512 | Snrpa1 | protein\_coding | 7:66059003-66074587 (+) |  | -0.4010 | 8.00e-12 | 3.19e-10 |
| ENSMUSG00000022094 | Slc39a14 | protein\_coding | 14:70303469-70351425 (-) |  | -1.8100 | 8.06e-12 | 3.21e-10 |
| ENSMUSG00000031432 | Prps1 | protein\_coding | X:140456613-140476140 (+) |  | -0.4360 | 8.15e-12 | 3.23e-10 |
| ENSMUSG00000016757 | Ttll12 | protein\_coding | 15:83575090-83595157 (-) |  | -0.7200 | 8.18e-12 | 3.23e-10 |
| ENSMUSG00000035824 | Tk2 | protein\_coding | 8:104226685-104248558 (-) |  | 0.5380 | 8.30e-12 | 3.27e-10 |
| ENSMUSG00000052997 | Uba2 | protein\_coding | 7:34140688-34169599 (-) |  | -0.2960 | 8.68e-12 | 3.40e-10 |
| ENSMUSG00000022391 | Rangap1 | protein\_coding | 15:81704248-81745530 (-) |  | -0.3300 | 8.68e-12 | 3.40e-10 |
| ENSMUSG00000001674 | Ddx18 | protein\_coding | 1:121553835-121567989 (-) |  | -0.4350 | 8.78e-12 | 3.43e-10 |
| ENSMUSG00000025868 | Higd2a | protein\_coding | 13:54590207-54591158 (+) |  | 0.4420 | 9.10e-12 | 3.55e-10 |
| ENSMUSG00000020936 | Nmt1 | protein\_coding | 11:103028190-103068912 (+) |  | -0.2640 | 9.13e-12 | 3.55e-10 |
| ENSMUSG00000022913 | Psmg1 | protein\_coding | 16:95979933-95990960 (-) |  | -0.5120 | 9.26e-12 | 3.59e-10 |
| ENSMUSG00000035150 | Eif2s3x | protein\_coding | X:94188707-94212862 (-) |  | -0.4650 | 9.29e-12 | 3.59e-10 |
| ENSMUSG00000032803 | Cdv3 | protein\_coding | 9:103353094-103365840 (-) |  | -0.3680 | 9.42e-12 | 3.63e-10 |
| ENSMUSG00000020785 | Camkk1 | protein\_coding | 11:73019008-73042073 (+) |  | -1.8800 | 9.60e-12 | 3.70e-10 |
| ENSMUSG00000028378 | Ptgr1 | protein\_coding | 4:58965439-58987119 (-) |  | -0.3880 | 1.06e-11 | 4.08e-10 |
| ENSMUSG00000026594 | Ralgps2 | protein\_coding | 1:156804166-156939626 (-) |  | 0.6950 | 1.14e-11 | 4.37e-10 |
| ENSMUSG00000025701 | Alox5 | protein\_coding | 6:116410077-116461178 (-) |  | 1.6300 | 1.14e-11 | 4.37e-10 |
| ENSMUSG00000032232 | Cgnl1 | protein\_coding | 9:71626509-71771602 (-) |  | 1.3900 | 1.19e-11 | 4.53e-10 |
| ENSMUSG00000037149 | Ddx1 | protein\_coding | 12:13216973-13249213 (-) |  | -0.4980 | 1.22e-11 | 4.63e-10 |
| ENSMUSG00000003072 | Atp5d | protein\_coding | 10:80138632-80145818 (+) |  | -0.3670 | 1.24e-11 | 4.71e-10 |
| ENSMUSG00000049971 | Glt1d1 | protein\_coding | 5:127632262-127709374 (+) |  | 1.3000 | 1.26e-11 | 4.75e-10 |
| ENSMUSG00000022241 | Tars | protein\_coding | 15:11382301-11399665 (-) |  | -0.4100 | 1.30e-11 | 4.88e-10 |
| ENSMUSG00000032740 | Ccdc88a | protein\_coding | 11:29373658-29510808 (+) |  | 0.4810 | 1.34e-11 | 5.05e-10 |
| ENSMUSG00000002068 | Ccne1 | protein\_coding | 7:38097984-38107534 (-) |  | -0.6230 | 1.38e-11 | 5.17e-10 |
| ENSMUSG00000031765 | Mt1 | protein\_coding | 8:94179082-94180327 (+) |  | -0.9780 | 1.44e-11 | 5.38e-10 |
| ENSMUSG00000032594 | Ip6k1 | protein\_coding | 9:108002501-108048782 (+) |  | 0.3260 | 1.57e-11 | 5.85e-10 |
| ENSMUSG00000019873 | Reep3 | protein\_coding | 10:67009189-67096945 (-) |  | 0.4680 | 1.60e-11 | 5.97e-10 |
| ENSMUSG00000079057 | Cyp4v3 | protein\_coding | 8:45304944-45333216 (-) |  | 0.6150 | 1.64e-11 | 6.08e-10 |
| ENSMUSG00000047180 | Neurl3 | protein\_coding | 1:36264597-36274679 (-) |  | 0.8210 | 1.66e-11 | 6.16e-10 |
| ENSMUSG00000055013 | Agap1 | protein\_coding | 1:89454806-89897617 (+) |  | 0.6750 | 1.73e-11 | 6.40e-10 |
| ENSMUSG00000023367 | Tmem176a | protein\_coding | 6:48840919-48847071 (+) |  | -0.6240 | 1.75e-11 | 6.46e-10 |
| ENSMUSG00000024812 | Tjp2 | protein\_coding | 19:24094505-24225030 (-) |  | -0.6910 | 1.76e-11 | 6.47e-10 |
| ENSMUSG00000038524 | Fchsd1 | protein\_coding | 18:37957431-37969774 (-) |  | 0.8380 | 1.80e-11 | 6.60e-10 |
| ENSMUSG00000030231 | Plekha5 | protein\_coding | 6:140424054-140597110 (+) |  | -0.9420 | 1.90e-11 | 6.94e-10 |
| ENSMUSG00000029623 | Pdap1 | protein\_coding | 5:145128769-145140238 (-) |  | -0.4490 | 1.90e-11 | 6.94e-10 |
| ENSMUSG00000001288 | Rarg | protein\_coding | 15:102234938-102257517 (-) |  | 0.5200 | 1.94e-11 | 7.07e-10 |
| ENSMUSG00000071072 | Ptges3 | protein\_coding | 10:128058954-128077272 (+) |  | -0.5260 | 1.98e-11 | 7.17e-10 |
| ENSMUSG00000024981 | Acsl5 | protein\_coding | 19:55251938-55297720 (+) |  | -0.3730 | 2.03e-11 | 7.33e-10 |
| ENSMUSG00000022010 | Tsc22d1 | protein\_coding | 14:76414961-76507765 (+) |  | 0.7110 | 2.10e-11 | 7.59e-10 |
| ENSMUSG00000017221 | Psmd3 | protein\_coding | 11:98682554-98695979 (+) |  | -0.4200 | 2.19e-11 | 7.89e-10 |
| ENSMUSG00000053411 | Cbx7 | protein\_coding | 15:79915807-79971119 (-) |  | 0.9140 | 2.21e-11 | 7.94e-10 |
| ENSMUSG00000019494 | Cops6 | protein\_coding | 5:138161071-138164646 (+) |  | -0.3170 | 2.28e-11 | 8.16e-10 |
| ENSMUSG00000031586 | Rbpms | protein\_coding | 8:33782643-33929863 (-) |  | 1.2400 | 2.32e-11 | 8.29e-10 |
| ENSMUSG00000020368 | Canx | protein\_coding | 11:50293961-50325673 (-) |  | -0.4200 | 2.34e-11 | 8.32e-10 |
| ENSMUSG00000021196 | Pfkp | protein\_coding | 13:6579768-6648777 (-) |  | -0.3940 | 2.34e-11 | 8.32e-10 |
| ENSMUSG00000014158 | Trpv4 | protein\_coding | 5:114622152-114658421 (-) |  | 1.6300 | 2.42e-11 | 8.59e-10 |
| ENSMUSG00000046808 | Atp10d | polymorphic\_pseudogene | 5:72203329-72298775 (+) |  | 0.5610 | 2.59e-11 | 9.15e-10 |
| ENSMUSG00000029804 | Herc3 | protein\_coding | 6:58831465-58920398 (+) |  | 0.5320 | 2.59e-11 | 9.15e-10 |
| ENSMUSG00000031657 | Heatr3 | protein\_coding | 8:88137855-88172027 (+) |  | -0.4880 | 2.70e-11 | 9.51e-10 |
| ENSMUSG00000038147 | Cd84 | protein\_coding | 1:171839697-171890718 (+) |  | 0.6370 | 2.80e-11 | 9.82e-10 |
| ENSMUSG00000032279 | Idh3a | protein\_coding | 9:54586334-54604661 (+) |  | -0.5280 | 2.81e-11 | 9.82e-10 |
| ENSMUSG00000001227 | Sema6b | protein\_coding | 17:56123085-56140343 (-) |  | -1.1300 | 2.81e-11 | 9.82e-10 |
| ENSMUSG00000036777 | Anln | protein\_coding | 9:22332012-22389188 (-) |  | -0.3660 | 2.84e-11 | 9.92e-10 |
| ENSMUSG00000031712 | Il15 | protein\_coding | 8:82331632-82403222 (-) |  | 0.8060 | 2.87e-11 | 9.95e-10 |
| ENSMUSG00000025190 | Got1 | protein\_coding | 19:43499752-43524605 (-) |  | -0.6380 | 2.87e-11 | 9.95e-10 |
| ENSMUSG00000085611 | Ap3s1-ps1 | processed\_pseudogene | X:38685592-38686170 (-) |  | -0.6750 | 2.90e-11 | 1.00e-09 |
| ENSMUSG00000103865 | Gm37416 | TEC | 2:16023108-16023425 (-) |  | -0.6470 | 3.02e-11 | 1.04e-09 |
| ENSMUSG00000024991 | Eif3a | protein\_coding | 19:60761117-60790658 (-) |  | -0.3030 | 3.16e-11 | 1.09e-09 |
| ENSMUSG00000036752 | Tubb4b | protein\_coding | 2:25222160-25224702 (-) |  | -0.3700 | 3.18e-11 | 1.09e-09 |
| ENSMUSG00000034041 | Lyl1 | protein\_coding | 8:84701449-84704940 (+) |  | 0.4620 | 3.36e-11 | 1.15e-09 |
| ENSMUSG00000052738 | Suclg1 | protein\_coding | 6:73248382-73276911 (+) |  | -0.3350 | 3.44e-11 | 1.18e-09 |
| ENSMUSG00000016256 | Ctsz | protein\_coding | 2:174427493-174439039 (-) |  | -0.3370 | 3.48e-11 | 1.19e-09 |
| ENSMUSG00000003348 | Mob3a | protein\_coding | 10:80685253-80701977 (-) |  | 0.2990 | 3.59e-11 | 1.22e-09 |
| ENSMUSG00000040026 | Saa3 | protein\_coding | 7:46711998-46715700 (-) |  | -3.1400 | 3.59e-11 | 1.22e-09 |
| ENSMUSG00000004069 | Dnaja3 | protein\_coding | 16:4639989-4707695 (+) |  | -0.3270 | 3.67e-11 | 1.24e-09 |
| ENSMUSG00000087129 | Gm16316 | lncRNA | 2:163685004-163692279 (-) |  | 1.2400 | 3.69e-11 | 1.25e-09 |
| ENSMUSG00000046456 | Tmem150b | protein\_coding | 7:4706832-4725249 (-) |  | 0.4290 | 3.71e-11 | 1.25e-09 |
| ENSMUSG00000030148 | Clec4a2 | protein\_coding | 6:123106428-123143999 (+) |  | 0.7130 | 3.74e-11 | 1.26e-09 |
| ENSMUSG00000004709 | Cd244a | protein\_coding | 1:171559193-171609746 (+) |  | -0.7450 | 3.75e-11 | 1.26e-09 |
| ENSMUSG00000020706 | Ftsj3 | protein\_coding | 11:106249142-106256079 (-) |  | -0.4070 | 3.96e-11 | 1.33e-09 |
| ENSMUSG00000020635 | Fkbp1b | protein\_coding | 12:4833174-4841591 (-) |  | 0.5580 | 4.38e-11 | 1.46e-09 |
| ENSMUSG00000087177 | E130307A14Rik | lncRNA | 10:39621412-39732007 (-) |  | 0.7070 | 4.44e-11 | 1.48e-09 |
| ENSMUSG00000020532 | Acaca | protein\_coding | 11:84129672-84401651 (+) |  | -0.4220 | 4.44e-11 | 1.48e-09 |
| ENSMUSG00000024360 | Etf1 | protein\_coding | 18:34902785-34932007 (-) |  | -0.3960 | 4.47e-11 | 1.48e-09 |
| ENSMUSG00000028633 | Ctps | protein\_coding | 4:120539868-120570276 (-) |  | -0.5140 | 4.48e-11 | 1.48e-09 |
| ENSMUSG00000041707 | Tmem273 | protein\_coding | 14:32785963-32817984 (+) |  | -1.0900 | 4.64e-11 | 1.53e-09 |
| ENSMUSG00000031907 | Zfp90 | protein\_coding | 8:106415327-106426598 (+) |  | 0.5810 | 4.70e-11 | 1.55e-09 |
| ENSMUSG00000019297 | Nop9 | protein\_coding | 14:55745693-55755500 (+) |  | -0.3170 | 4.73e-11 | 1.55e-09 |
| ENSMUSG00000045404 | Kcnk13 | protein\_coding | 12:99964499-100062682 (+) |  | -1.9000 | 4.88e-11 | 1.60e-09 |
| ENSMUSG00000062866 | Phactr2 | protein\_coding | 10:13207717-13474412 (-) |  | 0.5830 | 4.95e-11 | 1.62e-09 |
| ENSMUSG00000066232 | Ipo7 | protein\_coding | 7:110018274-110056609 (+) |  | -0.2800 | 5.05e-11 | 1.65e-09 |
| ENSMUSG00000033545 | Znrf1 | protein\_coding | 8:111536097-111626030 (+) |  | 0.3550 | 5.05e-11 | 1.65e-09 |
| ENSMUSG00000020642 | Rnf144a | protein\_coding | 12:26300964-26415254 (-) |  | 0.3260 | 5.08e-11 | 1.65e-09 |
| ENSMUSG00000025512 | Chid1 | protein\_coding | 7:141493136-141539857 (-) |  | -0.4020 | 5.14e-11 | 1.67e-09 |
| ENSMUSG00000005237 | Dnah2 | protein\_coding | 11:69420809-69549110 (-) |  | 0.8760 | 5.38e-11 | 1.74e-09 |
| ENSMUSG00000063229 | Ldha | protein\_coding | 7:46841475-46855627 (+) |  | -0.5520 | 5.57e-11 | 1.80e-09 |
| ENSMUSG00000005610 | Eif4g2 | protein\_coding | 7:111067750-111083030 (-) |  | -0.3470 | 5.68e-11 | 1.83e-09 |
| ENSMUSG00000030007 | Cct7 | protein\_coding | 6:85451514-85468475 (+) |  | -0.3480 | 5.70e-11 | 1.83e-09 |
| ENSMUSG00000023272 | Creld2 | protein\_coding | 15:88819646-88826683 (+) |  | -0.4710 | 5.85e-11 | 1.88e-09 |
| ENSMUSG00000024299 | Adamts10 | protein\_coding | 17:33524204-33553782 (+) |  | 0.5900 | 6.01e-11 | 1.92e-09 |
| ENSMUSG00000025648 | Pfkfb4 | protein\_coding | 9:108991778-109032228 (+) |  | 0.5380 | 6.09e-11 | 1.95e-09 |
| ENSMUSG00000019179 | Mdh2 | protein\_coding | 5:135778480-135790398 (+) |  | -0.4830 | 6.21e-11 | 1.98e-09 |
| ENSMUSG00000027405 | Nop56 | protein\_coding | 2:130274430-130279313 (+) |  | -0.3150 | 6.29e-11 | 2.00e-09 |
| ENSMUSG00000079111 | Kdelr2 | protein\_coding | 5:143403838-143421901 (+) |  | -0.3810 | 6.51e-11 | 2.07e-09 |
| ENSMUSG00000042851 | Zc3h6 | protein\_coding | 2:128967402-129018563 (+) |  | 1.0500 | 6.82e-11 | 2.16e-09 |
| ENSMUSG00000060550 | H2-Q7 | protein\_coding | 17:35439155-35443773 (+) |  | -0.8930 | 7.09e-11 | 2.24e-09 |
| ENSMUSG00000025823 | Pdia4 | protein\_coding | 6:47796141-47813430 (-) |  | -0.5010 | 7.21e-11 | 2.27e-09 |
| ENSMUSG00000057133 | Chd6 | protein\_coding | 2:160946978-161109075 (-) |  | 0.5030 | 7.59e-11 | 2.39e-09 |
| ENSMUSG00000030036 | Mogs | protein\_coding | 6:83115496-83118898 (+) |  | -0.3760 | 7.63e-11 | 2.39e-09 |
| ENSMUSG00000062937 | Mtap | protein\_coding | 4:89137122-89181081 (+) |  | -0.4220 | 7.68e-11 | 2.41e-09 |
| ENSMUSG00000078606 | Gm4070 | protein\_coding | 7:105895139-105953967 (-) |  | -0.7640 | 7.83e-11 | 2.45e-09 |
| ENSMUSG00000047963 | Stbd1 | protein\_coding | 5:92603041-92606579 (+) |  | 1.1000 | 7.92e-11 | 2.47e-09 |
| ENSMUSG00000015656 | Hspa8 | protein\_coding | 9:40800984-40810087 (+) |  | -0.3560 | 8.03e-11 | 2.50e-09 |
| ENSMUSG00000041607 | Mbp | protein\_coding | 18:82475146-82585637 (+) |  | 0.6440 | 8.25e-11 | 2.56e-09 |
| ENSMUSG00000022471 | Xrcc6 | protein\_coding | 15:81987835-82040085 (+) |  | -0.3510 | 8.35e-11 | 2.59e-09 |
| ENSMUSG00000040430 | Pitpnc1 | protein\_coding | 11:107207892-107470699 (-) |  | 0.5230 | 8.38e-11 | 2.59e-09 |
| ENSMUSG00000001741 | Il16 | protein\_coding | 7:83642825-83745726 (-) |  | 0.2920 | 8.73e-11 | 2.69e-09 |
| ENSMUSG00000022769 | Sdf2l1 | protein\_coding | 16:17130138-17132383 (-) |  | -0.4760 | 8.86e-11 | 2.73e-09 |
| ENSMUSG00000002006 | Pdzd4 | protein\_coding | X:73793359-73824969 (-) |  | 1.0000 | 9.20e-11 | 2.83e-09 |
| ENSMUSG00000031129 | Slc9a9 | protein\_coding | 9:94669909-95230445 (+) |  | 0.6960 | 9.27e-11 | 2.84e-09 |
| ENSMUSG00000028587 | Orc1 | protein\_coding | 4:108579423-108614833 (+) |  | -0.6160 | 9.66e-11 | 2.96e-09 |
| ENSMUSG00000020277 | Pfkl | protein\_coding | 10:77986947-78010083 (-) |  | -0.5210 | 1.06e-10 | 3.24e-09 |
| ENSMUSG00000036167 | Pphln1 | protein\_coding | 15:93398350-93491510 (+) |  | -0.3610 | 1.06e-10 | 3.24e-09 |
| ENSMUSG00000021213 | Akr1c13 | protein\_coding | 13:4191150-4205596 (+) |  | 0.7550 | 1.06e-10 | 3.24e-09 |
| ENSMUSG00000030168 | Adipor2 | protein\_coding | 6:119353150-119417704 (-) |  | 0.2720 | 1.07e-10 | 3.25e-09 |
| ENSMUSG00000021583 | Erap1 | protein\_coding | 13:74639568-74693201 (+) |  | -0.2780 | 1.08e-10 | 3.26e-09 |
| ENSMUSG00000032966 | Fkbp1a | protein\_coding | 2:151542483-151561692 (+) |  | -0.3440 | 1.11e-10 | 3.36e-09 |
| ENSMUSG00000055762 | Eef1d | protein\_coding | 15:75894205-75909556 (-) |  | -0.4000 | 1.13e-10 | 3.41e-09 |
| ENSMUSG00000033444 | Specc1l | protein\_coding | 10:75212073-75312743 (+) |  | 0.3540 | 1.14e-10 | 3.41e-09 |
| ENSMUSG00000002733 | Plekha3 | protein\_coding | 2:76675281-76696828 (+) |  | -0.5740 | 1.14e-10 | 3.41e-09 |
| ENSMUSG00000041695 | Kcnj2 | protein\_coding | 11:111066164-111076821 (+) |  | -0.9340 | 1.14e-10 | 3.41e-09 |
| ENSMUSG00000109556 | Gm38843 | lncRNA | 6:82803769-82805083 (-) |  | 1.5200 | 1.20e-10 | 3.59e-09 |
| ENSMUSG00000022774 | Ncbp2 | protein\_coding | 16:31948513-31961781 (+) |  | -0.3290 | 1.21e-10 | 3.61e-09 |
| ENSMUSG00000052336 | Cx3cr1 | protein\_coding | 9:119901616-120069879 (-) |  | 1.3000 | 1.23e-10 | 3.66e-09 |
| ENSMUSG00000011832 | Evi5l | protein\_coding | 8:4166567-4211257 (+) |  | 0.7920 | 1.23e-10 | 3.67e-09 |
| ENSMUSG00000022429 | Dmc1 | protein\_coding | 15:79561497-79605109 (-) |  | 1.0600 | 1.24e-10 | 3.67e-09 |
| ENSMUSG00000058881 | Zfp516 | protein\_coding | 18:82910663-83005314 (+) |  | 0.4070 | 1.28e-10 | 3.79e-09 |
| ENSMUSG00000032417 | Rwdd2a | protein\_coding | 9:86571991-86574899 (+) |  | 0.7380 | 1.29e-10 | 3.82e-09 |
| ENSMUSG00000095742 | CAAA01147332.1 | protein\_coding | JH584295.1:66-1479 (-) |  | 0.8200 | 1.30e-10 | 3.82e-09 |
| ENSMUSG00000037509 | Arhgef4 | protein\_coding | 1:34678188-34813309 (+) |  | 0.9310 | 1.30e-10 | 3.83e-09 |
| ENSMUSG00000036928 | Stag3 | protein\_coding | 5:138280240-138312393 (+) |  | 0.9520 | 1.32e-10 | 3.86e-09 |
| ENSMUSG00000025875 | Tspan17 | protein\_coding | 13:54789377-54796776 (+) |  | 0.7420 | 1.32e-10 | 3.86e-09 |
| ENSMUSG00000040354 | Mars | protein\_coding | 10:127296221-127311786 (-) |  | -0.3880 | 1.33e-10 | 3.90e-09 |
| ENSMUSG00000029014 | Dnajc2 | protein\_coding | 5:21757267-21785251 (-) |  | -0.3360 | 1.34e-10 | 3.92e-09 |
| ENSMUSG00000027597 | Ahcy | protein\_coding | 2:155059310-155074497 (-) |  | -0.5380 | 1.37e-10 | 4.00e-09 |
| ENSMUSG00000029060 | Mib2 | protein\_coding | 4:155654677-155669198 (-) |  | 0.5760 | 1.40e-10 | 4.07e-09 |
| ENSMUSG00000001089 | Luzp1 | protein\_coding | 4:136469761-136554780 (+) |  | -0.4640 | 1.41e-10 | 4.10e-09 |
| ENSMUSG00000027854 | Sike1 | protein\_coding | 3:102995708-103008459 (+) |  | 0.3240 | 1.45e-10 | 4.21e-09 |
| ENSMUSG00000030094 | Xpc | protein\_coding | 6:91489305-91515888 (-) |  | 0.5010 | 1.46e-10 | 4.21e-09 |
| ENSMUSG00000039067 | Psmd7 | protein\_coding | 8:107580381-107588464 (-) |  | -0.3710 | 1.48e-10 | 4.25e-09 |
| ENSMUSG00000004460 | Dnajb11 | protein\_coding | 16:22857845-22879634 (+) |  | -0.3270 | 1.53e-10 | 4.40e-09 |
| ENSMUSG00000030108 | Slc6a13 | protein\_coding | 6:121300227-121337733 (+) |  | 1.4500 | 1.63e-10 | 4.67e-09 |
| ENSMUSG00000029217 | Tec | protein\_coding | 5:72755716-72868483 (-) |  | 0.5220 | 1.66e-10 | 4.77e-09 |
| ENSMUSG00000023046 | Igfbp6 | protein\_coding | 15:102144362-102149511 (+) |  | -2.5400 | 1.72e-10 | 4.91e-09 |
| ENSMUSG00000039770 | Ypel5 | protein\_coding | 17:72836453-72851195 (+) |  | 0.3690 | 1.73e-10 | 4.94e-09 |
| ENSMUSG00000002718 | Cse1l | protein\_coding | 2:166906040-166946389 (+) |  | -0.3170 | 1.76e-10 | 5.03e-09 |
| ENSMUSG00000036825 | Ssx2ip | protein\_coding | 3:146404642-146440144 (+) |  | -0.5260 | 1.92e-10 | 5.46e-09 |
| ENSMUSG00000039126 | Prune2 | protein\_coding | 19:16956118-17223932 (+) |  | -1.0100 | 1.92e-10 | 5.46e-09 |
| ENSMUSG00000024359 | Hspa9 | protein\_coding | 18:34937414-34954357 (-) |  | -0.4980 | 1.94e-10 | 5.49e-09 |
| ENSMUSG00000017309 | Cd300lg | protein\_coding | 11:102041509-102055620 (+) |  | 0.5050 | 1.96e-10 | 5.54e-09 |
| ENSMUSG00000000359 | Rem1 | protein\_coding | 2:152626951-152635198 (+) |  | 0.8650 | 2.10e-10 | 5.93e-09 |
| ENSMUSG00000096727 | Psmb9 | protein\_coding | 17:34181987-34187764 (-) |  | -0.4170 | 2.16e-10 | 6.10e-09 |
| ENSMUSG00000020413 | Hus1 | protein\_coding | 11:8993137-9011191 (-) |  | -0.3840 | 2.21e-10 | 6.21e-09 |
| ENSMUSG00000024663 | Rab3il1 | protein\_coding | 19:10001669-10038380 (+) |  | -0.7660 | 2.21e-10 | 6.21e-09 |
| ENSMUSG00000060044 | Tmem26 | protein\_coding | 10:68723646-68782650 (+) |  | 1.5100 | 2.23e-10 | 6.26e-09 |
| ENSMUSG00000030109 | Slc6a12 | protein\_coding | 6:121343076-121365775 (+) |  | 4.8100 | 2.26e-10 | 6.31e-09 |
| ENSMUSG00000031875 | Cmtm3 | protein\_coding | 8:104339410-104347672 (+) |  | 0.4340 | 2.26e-10 | 6.31e-09 |
| ENSMUSG00000032192 | Gnb5 | protein\_coding | 9:75306288-75345876 (+) |  | -1.8200 | 2.29e-10 | 6.39e-09 |
| ENSMUSG00000040699 | Limd2 | protein\_coding | 11:106156256-106160860 (-) |  | 0.4060 | 2.35e-10 | 6.54e-09 |
| ENSMUSG00000032336 | Nptn | protein\_coding | 9:58582240-58657955 (+) |  | 0.2410 | 2.52e-10 | 6.99e-09 |
| ENSMUSG00000042042 | Csgalnact2 | protein\_coding | 6:118107452-118139140 (-) |  | 0.3580 | 2.54e-10 | 7.03e-09 |
| ENSMUSG00000032565 | Nudt16 | protein\_coding | 9:105128903-105131824 (-) |  | 0.5450 | 2.55e-10 | 7.03e-09 |
| ENSMUSG00000092203 | 1110038B12Rik | lncRNA | 17:34950238-34952471 (-) |  | -0.5170 | 2.57e-10 | 7.08e-09 |
| ENSMUSG00000038736 | Nudcd1 | protein\_coding | 15:44373163-44428307 (-) |  | -0.4910 | 2.64e-10 | 7.28e-09 |
| ENSMUSG00000026615 | Eprs | protein\_coding | 1:185363044-185428360 (+) |  | -0.4650 | 2.68e-10 | 7.37e-09 |
| ENSMUSG00000027236 | Eif3j1 | protein\_coding | 2:122028546-122056598 (+) |  | -0.4340 | 2.74e-10 | 7.51e-09 |
| ENSMUSG00000029471 | Camkk2 | protein\_coding | 5:122731170-122779409 (-) |  | 0.5870 | 2.74e-10 | 7.51e-09 |
| ENSMUSG00000042659 | Arrdc4 | protein\_coding | 7:68736995-68749241 (-) |  | 0.5260 | 2.92e-10 | 7.96e-09 |
| ENSMUSG00000020641 | Rsad2 | protein\_coding | 12:26442746-26456452 (-) |  | 1.1100 | 2.92e-10 | 7.96e-09 |
| ENSMUSG00000032382 | Snx1 | protein\_coding | 9:66088133-66126587 (-) |  | -0.3660 | 2.94e-10 | 8.01e-09 |
| ENSMUSG00000056529 | Ptafr | protein\_coding | 4:132564067-132582683 (+) |  | 0.5830 | 2.95e-10 | 8.02e-09 |
| ENSMUSG00000025001 | Hells | protein\_coding | 19:38930915-38971051 (+) |  | -0.5070 | 2.99e-10 | 8.10e-09 |
| ENSMUSG00000029390 | Tmed2 | protein\_coding | 5:124540695-124550506 (+) |  | -0.3410 | 2.99e-10 | 8.10e-09 |
| ENSMUSG00000015291 | Gdi1 | protein\_coding | X:74304998-74311862 (+) |  | 0.3920 | 3.01e-10 | 8.13e-09 |
| ENSMUSG00000020680 | Taf15 | protein\_coding | 11:83473086-83506743 (+) |  | -0.3770 | 3.16e-10 | 8.51e-09 |
| ENSMUSG00000040028 | Elavl1 | protein\_coding | 8:4285382-4325413 (-) |  | -0.3280 | 3.25e-10 | 8.75e-09 |
| ENSMUSG00000032763 | Ilvbl | protein\_coding | 10:78574346-78584502 (+) |  | 0.3780 | 3.28e-10 | 8.81e-09 |
| ENSMUSG00000030149 | Klrk1 | protein\_coding | 6:129610323-129623864 (-) |  | 0.9340 | 3.36e-10 | 9.00e-09 |
| ENSMUSG00000037278 | Tmem97 | protein\_coding | 11:78541817-78550777 (-) |  | -0.5100 | 3.59e-10 | 9.61e-09 |
| ENSMUSG00000068749 | Psma5 | protein\_coding | 3:108256926-108279974 (+) |  | -0.3730 | 3.60e-10 | 9.61e-09 |
| ENSMUSG00000042500 | Ago4 | protein\_coding | 4:126489541-126533472 (-) |  | 0.6660 | 3.68e-10 | 9.82e-09 |
| ENSMUSG00000040848 | Sft2d2 | protein\_coding | 1:165174337-165194438 (-) |  | 0.3700 | 3.76e-10 | 1.00e-08 |
| ENSMUSG00000063952 | Brpf3 | protein\_coding | 17:28801090-28839949 (+) |  | 0.5240 | 3.91e-10 | 1.04e-08 |
| ENSMUSG00000020023 | Tmcc3 | protein\_coding | 10:94311949-94590956 (+) |  | 1.3500 | 3.93e-10 | 1.04e-08 |
| ENSMUSG00000036587 | Fut7 | protein\_coding | 2:25423267-25426374 (+) |  | 0.8350 | 3.95e-10 | 1.05e-08 |
| ENSMUSG00000037548 | H2-DMb2 | protein\_coding | 17:34143307-34151555 (+) |  | 0.6170 | 4.00e-10 | 1.06e-08 |
| ENSMUSG00000074093 | Svip | protein\_coding | 7:51997171-52006018 (-) |  | 0.4450 | 4.03e-10 | 1.06e-08 |
| ENSMUSG00000020547 | Bzw2 | protein\_coding | 12:36091835-36158080 (-) |  | -0.4890 | 4.07e-10 | 1.07e-08 |
| ENSMUSG00000015968 | Cacna1d | protein\_coding | 14:30039939-30491455 (-) |  | 0.8570 | 4.09e-10 | 1.07e-08 |
| ENSMUSG00000041491 | Cep78 | protein\_coding | 19:15955773-15984989 (-) |  | -0.3260 | 4.16e-10 | 1.09e-08 |
| ENSMUSG00000037275 | Gemin5 | protein\_coding | 11:58120002-58168539 (-) |  | -0.4430 | 4.35e-10 | 1.14e-08 |
| ENSMUSG00000018770 | Atp5g3 | protein\_coding | 2:73908447-73911326 (-) |  | -0.4190 | 4.38e-10 | 1.14e-08 |
| ENSMUSG00000045176 | Borcs6 | protein\_coding | 11:69059717-69061578 (+) |  | 0.5550 | 4.38e-10 | 1.14e-08 |
| ENSMUSG00000041736 | Tspo | protein\_coding | 15:83563592-83574203 (+) |  | -0.5200 | 4.39e-10 | 1.14e-08 |
| ENSMUSG00000037151 | Lrrc20 | protein\_coding | 10:61475801-61582791 (+) |  | 0.5600 | 4.43e-10 | 1.15e-08 |
| ENSMUSG00000031422 | Morf4l2 | protein\_coding | X:136732942-136743690 (-) |  | -0.3370 | 4.44e-10 | 1.15e-08 |
| ENSMUSG00000040283 | Btnl9 | protein\_coding | 11:49165585-49187159 (-) |  | 1.0600 | 4.47e-10 | 1.16e-08 |
| ENSMUSG00000038871 | Bpgm | protein\_coding | 6:34476207-34505613 (+) |  | 0.6190 | 4.51e-10 | 1.17e-08 |
| ENSMUSG00000001016 | Ilf2 | protein\_coding | 3:90476126-90488379 (+) |  | -0.2770 | 4.59e-10 | 1.19e-08 |
| ENSMUSG00000029777 | Gars | protein\_coding | 6:55038007-55079500 (+) |  | -0.3820 | 4.62e-10 | 1.19e-08 |
| ENSMUSG00000033047 | Eif3l | protein\_coding | 15:79075179-79094405 (+) |  | -0.3030 | 4.66e-10 | 1.20e-08 |
| ENSMUSG00000071644 | Eef1g | protein\_coding | 19:8967041-8978479 (+) |  | -0.4620 | 4.70e-10 | 1.21e-08 |
| ENSMUSG00000020402 | Vdac1 | protein\_coding | 11:52360860-52389397 (+) |  | -0.3400 | 4.83e-10 | 1.24e-08 |
| ENSMUSG00000021474 | Sfxn1 | protein\_coding | 13:54071869-54108342 (+) |  | -0.4880 | 4.85e-10 | 1.24e-08 |
| ENSMUSG00000001588 | Acap1 | protein\_coding | 11:69881567-69895539 (-) |  | 0.4390 | 4.96e-10 | 1.27e-08 |
| ENSMUSG00000006058 | Snf8 | protein\_coding | 11:96034885-96047430 (+) |  | -0.2630 | 5.05e-10 | 1.29e-08 |
| ENSMUSG00000041763 | Tpp2 | protein\_coding | 1:43933647-44003000 (+) |  | -0.3150 | 5.17e-10 | 1.31e-08 |
| ENSMUSG00000062372 | Otof | protein\_coding | 5:30367062-30461932 (-) |  | 1.4700 | 5.18e-10 | 1.32e-08 |
| ENSMUSG00000036353 | P2ry12 | protein\_coding | 3:59216272-59262871 (-) |  | -1.2400 | 5.28e-10 | 1.34e-08 |
| ENSMUSG00000036817 | Sun1 | protein\_coding | 5:139200637-139249840 (+) |  | 0.3530 | 5.35e-10 | 1.36e-08 |
| ENSMUSG00000019579 | Mydgf | protein\_coding | 17:56175744-56183920 (-) |  | -0.4380 | 5.36e-10 | 1.36e-08 |
| ENSMUSG00000073147 | 5031425E22Rik | lncRNA | 5:23382308-23434269 (-) |  | 0.5500 | 5.48e-10 | 1.38e-08 |
| ENSMUSG00000081603 | Gm14681 | processed\_pseudogene | X:66778442-66778852 (-) |  | -0.4900 | 5.58e-10 | 1.40e-08 |
| ENSMUSG00000026458 | Ppfia4 | protein\_coding | 1:134296783-134332928 (-) |  | 0.5010 | 5.59e-10 | 1.41e-08 |
| ENSMUSG00000030224 | Strap | protein\_coding | 6:137735078-137751932 (+) |  | -0.4000 | 5.69e-10 | 1.43e-08 |
| ENSMUSG00000036737 | Oxsr1 | protein\_coding | 9:119238432-119322427 (-) |  | -0.2940 | 5.70e-10 | 1.43e-08 |
| ENSMUSG00000046006 | Gapt | protein\_coding | 13:110352616-110357199 (-) |  | 0.4290 | 5.74e-10 | 1.43e-08 |
| ENSMUSG00000025364 | Pa2g4 | protein\_coding | 10:128557766-128565987 (-) |  | -0.4830 | 5.77e-10 | 1.44e-08 |
| ENSMUSG00000037197 | Rbm17 | protein\_coding | 2:11585437-11604153 (-) |  | -0.2700 | 5.78e-10 | 1.44e-08 |
| ENSMUSG00000042249 | Grk3 | protein\_coding | 5:112910482-113015791 (-) |  | 0.6580 | 5.94e-10 | 1.48e-08 |
| ENSMUSG00000071547 | Nt5dc2 | protein\_coding | 14:31131053-31139124 (+) |  | -0.6690 | 6.01e-10 | 1.49e-08 |
| ENSMUSG00000045636 | Mtus1 | protein\_coding | 8:40990914-41133726 (-) |  | -0.2460 | 6.03e-10 | 1.50e-08 |
| ENSMUSG00000067071 | Hes6 | protein\_coding | 1:91411483-91414038 (-) |  | 0.3740 | 6.19e-10 | 1.53e-08 |
| ENSMUSG00000026641 | Usf1 | protein\_coding | 1:171411313-171419142 (+) |  | -0.2200 | 6.25e-10 | 1.55e-08 |
| ENSMUSG00000027968 | Larp7 | protein\_coding | 3:127536714-127553349 (-) |  | -0.2840 | 6.58e-10 | 1.62e-08 |
| ENSMUSG00000070056 | Mfhas1 | protein\_coding | 8:35587798-35679449 (+) |  | 0.6040 | 6.61e-10 | 1.63e-08 |
| ENSMUSG00000022555 | Dgat1 | protein\_coding | 15:76502015-76511953 (-) |  | 0.4530 | 6.68e-10 | 1.64e-08 |
| ENSMUSG00000025969 | Nrp2 | protein\_coding | 1:62703285-62818695 (+) |  | -0.4530 | 6.82e-10 | 1.67e-08 |
| ENSMUSG00000029836 | Cbx3 | protein\_coding | 6:51470360-51483704 (+) |  | -0.3050 | 7.10e-10 | 1.74e-08 |
| ENSMUSG00000032011 | Thy1 | protein\_coding | 9:44043384-44048579 (+) |  | 1.3900 | 7.29e-10 | 1.78e-08 |
| ENSMUSG00000020048 | Hsp90b1 | protein\_coding | 10:86690209-86705509 (-) |  | -0.4260 | 7.49e-10 | 1.83e-08 |
| ENSMUSG00000056612 | Ppp1r14b | protein\_coding | 19:6974968-6977324 (+) |  | -0.4670 | 7.58e-10 | 1.85e-08 |
| ENSMUSG00000045427 | Hnrnph2 | protein\_coding | X:134601179-134607060 (+) |  | -0.3290 | 7.76e-10 | 1.89e-08 |
| ENSMUSG00000027804 | Ppid | protein\_coding | 3:79591342-79603650 (+) |  | -0.4190 | 7.81e-10 | 1.90e-08 |
| ENSMUSG00000015305 | Sash1 | protein\_coding | 10:8722219-8886070 (-) |  | 0.6140 | 7.85e-10 | 1.91e-08 |
| ENSMUSG00000052833 | Sae1 | protein\_coding | 7:16320234-16387806 (-) |  | -0.3770 | 7.87e-10 | 1.91e-08 |
| ENSMUSG00000028657 | Ppt1 | protein\_coding | 4:122836242-122859175 (+) |  | -0.2930 | 7.97e-10 | 1.93e-08 |
| ENSMUSG00000040811 | Eml2 | protein\_coding | 7:19176421-19206482 (+) |  | 0.3980 | 8.06e-10 | 1.94e-08 |
| ENSMUSG00000033307 | Mif | protein\_coding | 10:75859353-75860240 (-) |  | -0.7680 | 8.11e-10 | 1.95e-08 |
| ENSMUSG00000031444 | F10 | protein\_coding | 8:13037308-13056676 (+) |  | -0.4930 | 8.26e-10 | 1.99e-08 |
| ENSMUSG00000109244 | Gm44751 | lncRNA | 7:88311478-88315864 (+) |  | -0.8930 | 8.26e-10 | 1.99e-08 |
| ENSMUSG00000033427 | Upb1 | protein\_coding | 10:75401115-75441679 (+) |  | 1.0600 | 8.41e-10 | 2.02e-08 |
| ENSMUSG00000027108 | Ola1 | protein\_coding | 2:73092801-73218924 (-) |  | -0.3590 | 8.45e-10 | 2.02e-08 |
| ENSMUSG00000010048 | Ifrd2 | protein\_coding | 9:107587642-107593385 (+) |  | -0.5240 | 8.59e-10 | 2.05e-08 |
| ENSMUSG00000066491 | Cox6c2 | processed\_pseudogene | 12:56373593-56373823 (-) |  | 1.2700 | 8.67e-10 | 2.07e-08 |
| ENSMUSG00000020178 | Adora2a | protein\_coding | 10:75316877-75334784 (+) |  | 1.5600 | 8.71e-10 | 2.07e-08 |
| ENSMUSG00000042354 | Gnl3 | protein\_coding | 14:31012433-31019152 (-) |  | -0.3970 | 8.71e-10 | 2.07e-08 |
| ENSMUSG00000021365 | Nedd9 | protein\_coding | 13:41309581-41487362 (-) |  | 0.3810 | 8.84e-10 | 2.10e-08 |
| ENSMUSG00000016024 | Lbp | protein\_coding | 2:158306493-158332852 (+) |  | -0.8010 | 8.93e-10 | 2.12e-08 |
| ENSMUSG00000042784 | Muc1 | protein\_coding | 3:89229057-89233381 (+) |  | -2.5900 | 8.95e-10 | 2.12e-08 |
| ENSMUSG00000027774 | Gfm1 | protein\_coding | 3:67430096-67476529 (+) |  | -0.3870 | 8.97e-10 | 2.12e-08 |
| ENSMUSG00000097164 | Cep83os | lncRNA | 10:94671025-94688576 (-) |  | 0.6570 | 8.99e-10 | 2.12e-08 |
| ENSMUSG00000014859 | E2f4 | protein\_coding | 8:105297663-105305370 (+) |  | -0.3050 | 9.05e-10 | 2.13e-08 |
| ENSMUSG00000029314 | Gpat3 | protein\_coding | 5:100845713-100899102 (+) |  | -1.6300 | 9.13e-10 | 2.15e-08 |
| ENSMUSG00000027944 | Hax1 | protein\_coding | 3:89995446-89998780 (-) |  | -0.4200 | 9.27e-10 | 2.18e-08 |
| ENSMUSG00000000440 | Pparg | protein\_coding | 6:115360951-115490399 (+) |  | 1.6100 | 9.31e-10 | 2.18e-08 |
| ENSMUSG00000054675 | Tmem119 | protein\_coding | 5:113793729-113800516 (-) |  | 0.8480 | 9.47e-10 | 2.22e-08 |
| ENSMUSG00000085465 | Gm15347 | lncRNA | 8:12860358-12877344 (-) |  | -1.7100 | 1.00e-09 | 2.35e-08 |
| ENSMUSG00000051444 | Bbs12 | protein\_coding | 3:37312554-37321453 (+) |  | -0.7850 | 1.01e-09 | 2.35e-08 |
| ENSMUSG00000026864 | Hspa5 | protein\_coding | 2:34771970-34777547 (+) |  | -0.5050 | 1.01e-09 | 2.36e-08 |
| ENSMUSG00000041268 | Dmxl2 | protein\_coding | 9:54365158-54501626 (-) |  | -0.4510 | 1.02e-09 | 2.36e-08 |
| ENSMUSG00000054400 | Cklf | protein\_coding | 8:104250861-104264938 (+) |  | 0.4950 | 1.02e-09 | 2.37e-08 |
| ENSMUSG00000068329 | Htra2 | protein\_coding | 6:83051266-83055273 (-) |  | -0.3400 | 1.04e-09 | 2.41e-08 |
| ENSMUSG00000071068 | Treml2 | protein\_coding | 17:48299498-48312533 (+) |  | -0.4160 | 1.05e-09 | 2.43e-08 |
| ENSMUSG00000059714 | Flot1 | protein\_coding | 17:35823230-35832791 (+) |  | -0.4120 | 1.05e-09 | 2.43e-08 |
| ENSMUSG00000030789 | Itgax | protein\_coding | 7:128129547-128150657 (+) |  | 0.9510 | 1.07e-09 | 2.47e-08 |
| ENSMUSG00000029617 | Ccz1 | protein\_coding | 5:143987909-144014877 (-) |  | -0.3500 | 1.08e-09 | 2.49e-08 |
| ENSMUSG00000025330 | Padi4 | protein\_coding | 4:140745865-140774236 (-) |  | 0.6040 | 1.08e-09 | 2.49e-08 |
| ENSMUSG00000070544 | Top1 | protein\_coding | 2:160645888-160722764 (+) |  | -0.3030 | 1.11e-09 | 2.55e-08 |
| ENSMUSG00000021738 | Atxn7 | protein\_coding | 14:13961440-14107302 (+) |  | 0.4090 | 1.12e-09 | 2.56e-08 |
| ENSMUSG00000041235 | Chd7 | protein\_coding | 4:8690406-8867659 (+) |  | -0.5570 | 1.16e-09 | 2.67e-08 |
| ENSMUSG00000024579 | Pcyox1l | protein\_coding | 18:61696837-61707635 (-) |  | -0.6220 | 1.18e-09 | 2.69e-08 |
| ENSMUSG00000039630 | Hnrnpu | protein\_coding | 1:178321108-178337797 (-) |  | -0.2260 | 1.18e-09 | 2.70e-08 |
| ENSMUSG00000026766 | Mmadhc | protein\_coding | 2:50279881-50296801 (-) |  | -0.3590 | 1.24e-09 | 2.81e-08 |
| ENSMUSG00000001416 | Cct3 | protein\_coding | 3:88297116-88321767 (+) |  | -0.4350 | 1.25e-09 | 2.84e-08 |
| ENSMUSG00000003299 | Mrpl4 | protein\_coding | 9:21002738-21008839 (+) |  | -0.2600 | 1.29e-09 | 2.93e-08 |
| ENSMUSG00000017417 | Plxdc1 | protein\_coding | 11:97923238-97986444 (-) |  | 0.6210 | 1.31e-09 | 2.96e-08 |
| ENSMUSG00000064267 | Hvcn1 | protein\_coding | 5:122206804-122242297 (+) |  | 0.5860 | 1.34e-09 | 3.03e-08 |
| ENSMUSG00000045983 | Eif4g1 | protein\_coding | 16:20668313-20692884 (+) |  | -0.3480 | 1.38e-09 | 3.12e-08 |
| ENSMUSG00000025613 | Cct8 | protein\_coding | 16:87483326-87495873 (-) |  | -0.3800 | 1.40e-09 | 3.16e-08 |
| ENSMUSG00000003868 | Ruvbl2 | protein\_coding | 7:45421760-45438096 (-) |  | -0.4200 | 1.45e-09 | 3.27e-08 |
| ENSMUSG00000037104 | Socs5 | protein\_coding | 17:87107679-87137839 (+) |  | -0.6110 | 1.45e-09 | 3.27e-08 |
| ENSMUSG00000010554 | Mettl16 | protein\_coding | 11:74770830-74828525 (+) |  | -0.4250 | 1.47e-09 | 3.30e-08 |
| ENSMUSG00000036561 | Ppp6r2 | protein\_coding | 15:89211553-89287010 (+) |  | 0.5000 | 1.48e-09 | 3.33e-08 |
| ENSMUSG00000034653 | Ythdc2 | protein\_coding | 18:44827746-44889724 (+) |  | -0.3230 | 1.53e-09 | 3.43e-08 |
| ENSMUSG00000024785 | Rcl1 | protein\_coding | 19:29101375-29143929 (+) |  | -0.4040 | 1.54e-09 | 3.45e-08 |
| ENSMUSG00000029388 | Eif2b1 | protein\_coding | 5:124570213-124579131 (-) |  | -0.4220 | 1.56e-09 | 3.48e-08 |
| ENSMUSG00000021880 | Rnase6 | protein\_coding | 14:51123908-51132187 (+) |  | 0.7670 | 1.56e-09 | 3.49e-08 |
| ENSMUSG00000056999 | Ide | protein\_coding | 19:37268743-37337852 (-) |  | -0.5100 | 1.57e-09 | 3.50e-08 |
| ENSMUSG00000034377 | Tulp4 | protein\_coding | 17:6106437-6251128 (+) |  | -0.6810 | 1.57e-09 | 3.50e-08 |
| ENSMUSG00000006800 | Sulf2 | protein\_coding | 2:166073089-166155663 (-) |  | 0.9340 | 1.58e-09 | 3.52e-08 |
| ENSMUSG00000040663 | Clcf1 | protein\_coding | 19:4214238-4223490 (+) |  | 1.2900 | 1.61e-09 | 3.55e-08 |
| ENSMUSG00000071203 | Naip5 | protein\_coding | 13:100211739-100246323 (-) |  | 0.4800 | 1.61e-09 | 3.55e-08 |
| ENSMUSG00000020653 | Klf11 | protein\_coding | 12:24651274-24662789 (+) |  | 0.5950 | 1.62e-09 | 3.57e-08 |
| ENSMUSG00000006932 | Ctnnb1 | protein\_coding | 9:120929216-120960507 (+) |  | -0.2230 | 1.73e-09 | 3.81e-08 |
| ENSMUSG00000023988 | Bysl | protein\_coding | 17:47599331-47611492 (-) |  | -0.3810 | 1.73e-09 | 3.81e-08 |
| ENSMUSG00000024193 | Phf1 | protein\_coding | 17:26933052-26937908 (+) |  | 0.5030 | 1.76e-09 | 3.87e-08 |
| ENSMUSG00000040746 | Rnf167 | protein\_coding | 11:70647235-70651421 (+) |  | 0.4000 | 1.78e-09 | 3.90e-08 |
| ENSMUSG00000026914 | Psmd14 | protein\_coding | 2:61711694-61800376 (+) |  | -0.3450 | 1.78e-09 | 3.90e-08 |
| ENSMUSG00000038393 | Txnip | protein\_coding | 3:96557957-96561883 (+) |  | 0.6510 | 1.79e-09 | 3.91e-08 |
| ENSMUSG00000056536 | Pign | protein\_coding | 1:105518422-105663677 (-) |  | -0.3070 | 1.80e-09 | 3.93e-08 |
| ENSMUSG00000066150 | Slc31a1 | protein\_coding | 4:62360727-62391769 (+) |  | -0.2970 | 1.83e-09 | 4.00e-08 |
| ENSMUSG00000036898 | Zfp157 | protein\_coding | 5:138441468-138460694 (+) |  | 0.5070 | 1.85e-09 | 4.03e-08 |
| ENSMUSG00000060098 | Prmt7 | protein\_coding | 8:106210936-106252794 (+) |  | -0.4010 | 1.89e-09 | 4.11e-08 |
| ENSMUSG00000028159 | Dapp1 | protein\_coding | 3:137931007-137981545 (-) |  | 0.3100 | 1.96e-09 | 4.27e-08 |
| ENSMUSG00000051314 | Ffar2 | protein\_coding | 7:30818348-30823775 (-) |  | -1.1400 | 1.99e-09 | 4.32e-08 |
| ENSMUSG00000020149 | Rab1a | protein\_coding | 11:20201432-20226856 (+) |  | -0.2740 | 2.07e-09 | 4.48e-08 |
| ENSMUSG00000021340 | Gpld1 | protein\_coding | 13:24943152-24992501 (+) |  | -1.4400 | 2.08e-09 | 4.50e-08 |
| ENSMUSG00000028550 | Atg4c | protein\_coding | 4:99193934-99259787 (+) |  | 0.4320 | 2.12e-09 | 4.57e-08 |
| ENSMUSG00000057113 | Npm1 | protein\_coding | 11:33152287-33163206 (-) |  | -0.4420 | 2.12e-09 | 4.57e-08 |
| ENSMUSG00000032915 | Adgre4 | protein\_coding | 17:55749984-55853662 (+) |  | 2.2400 | 2.13e-09 | 4.59e-08 |
| ENSMUSG00000020846 | Rflnb | protein\_coding | 11:76019194-76027782 (-) |  | 0.4260 | 2.19e-09 | 4.72e-08 |
| ENSMUSG00000028330 | Ncbp1 | protein\_coding | 4:46138613-46172403 (+) |  | -0.2500 | 2.22e-09 | 4.77e-08 |
| ENSMUSG00000046688 | Tifa | protein\_coding | 3:127789805-127832164 (+) |  | 0.3970 | 2.25e-09 | 4.82e-08 |
| ENSMUSG00000021109 | Hif1a | protein\_coding | 12:73901375-73947530 (+) |  | -0.5060 | 2.34e-09 | 5.00e-08 |
| ENSMUSG00000020788 | Atp2a3 | protein\_coding | 11:72961169-72993044 (+) |  | 0.4080 | 2.34e-09 | 5.00e-08 |
| ENSMUSG00000025059 | Gk | protein\_coding | X:85701937-85776819 (-) |  | -0.5180 | 2.34e-09 | 5.00e-08 |
| ENSMUSG00000052798 | Nup107 | protein\_coding | 10:117750621-117792705 (-) |  | -0.3000 | 2.37e-09 | 5.05e-08 |
| ENSMUSG00000078515 | Ddi2 | protein\_coding | 4:141677549-141723419 (-) |  | -0.4910 | 2.40e-09 | 5.11e-08 |
| ENSMUSG00000018042 | Cyb5r3 | protein\_coding | 15:83153494-83172592 (-) |  | 0.3510 | 2.47e-09 | 5.24e-08 |
| ENSMUSG00000070407 | Hs3st3b1 | protein\_coding | 11:63885792-63922290 (-) |  | -1.7400 | 2.47e-09 | 5.25e-08 |
| ENSMUSG00000073676 | Hspe1 | protein\_coding | 1:55088132-55091307 (+) |  | -0.5420 | 2.52e-09 | 5.33e-08 |
| ENSMUSG00000034485 | Uaca | protein\_coding | 9:60794542-60880370 (+) |  | -1.9100 | 2.57e-09 | 5.44e-08 |
| ENSMUSG00000046756 | Mrps7 | protein\_coding | 11:115603925-115608036 (+) |  | -0.3850 | 2.58e-09 | 5.45e-08 |
| ENSMUSG00000021127 | Zfp36l1 | protein\_coding | 12:80107754-80113013 (-) |  | -1.9800 | 2.61e-09 | 5.51e-08 |
| ENSMUSG00000022075 | Rhobtb2 | protein\_coding | 14:69784990-69805636 (-) |  | -0.5380 | 2.63e-09 | 5.53e-08 |
| ENSMUSG00000035711 | Dok3 | protein\_coding | 13:55523231-55529296 (-) |  | 0.3160 | 2.63e-09 | 5.53e-08 |
| ENSMUSG00000031613 | Hpgd | protein\_coding | 8:56294585-56321043 (+) |  | 1.8200 | 2.67e-09 | 5.61e-08 |
| ENSMUSG00000020361 | Hspa4 | protein\_coding | 11:53259814-53300457 (-) |  | -0.3110 | 2.69e-09 | 5.64e-08 |
| ENSMUSG00000051727 | Kctd14 | protein\_coding | 7:97451323-97459557 (+) |  | 0.9600 | 2.72e-09 | 5.70e-08 |
| ENSMUSG00000027219 | Slc28a2 | protein\_coding | 2:122426477-122461137 (+) |  | -0.6790 | 2.76e-09 | 5.77e-08 |
| ENSMUSG00000018433 | Nol11 | protein\_coding | 11:107166663-107189381 (-) |  | -0.3300 | 2.76e-09 | 5.77e-08 |
| ENSMUSG00000034349 | Smc4 | protein\_coding | 3:69004738-69034623 (+) |  | -0.2690 | 2.82e-09 | 5.88e-08 |
| ENSMUSG00000010517 | Faf1 | protein\_coding | 4:109676588-109963960 (+) |  | -0.3310 | 2.83e-09 | 5.90e-08 |
| ENSMUSG00000021814 | Anxa7 | protein\_coding | 14:20455260-20480133 (-) |  | -0.3000 | 2.88e-09 | 5.98e-08 |
| ENSMUSG00000020681 | Ace | protein\_coding | 11:105967945-105989964 (+) |  | 3.2400 | 2.92e-09 | 6.05e-08 |
| ENSMUSG00000020455 | Trim11 | protein\_coding | 11:58978093-58991458 (+) |  | -0.3360 | 2.92e-09 | 6.05e-08 |
| ENSMUSG00000006498 | Ptbp1 | protein\_coding | 10:79854427-79864771 (+) |  | -0.3090 | 2.94e-09 | 6.10e-08 |
| ENSMUSG00000032010 | Usp2 | protein\_coding | 9:44067021-44095627 (+) |  | -0.7650 | 3.00e-09 | 6.20e-08 |
| ENSMUSG00000030272 | Camk1 | protein\_coding | 6:113334124-113343984 (-) |  | 0.6190 | 3.01e-09 | 6.21e-08 |
| ENSMUSG00000031633 | Slc25a4 | protein\_coding | 8:46206797-46211284 (-) |  | -0.2820 | 3.04e-09 | 6.26e-08 |
| ENSMUSG00000026489 | Coq8a | protein\_coding | 1:180165238-180199602 (-) |  | 0.8640 | 3.09e-09 | 6.37e-08 |
| ENSMUSG00000029246 | Ppat | protein\_coding | 5:76913249-76951578 (-) |  | -0.3890 | 3.14e-09 | 6.44e-08 |
| ENSMUSG00000030423 | Pop4 | protein\_coding | 7:38261996-38271423 (-) |  | -0.3730 | 3.16e-09 | 6.48e-08 |
| ENSMUSG00000066861 | Oas1g | protein\_coding | 5:120876142-120887613 (-) |  | -1.2900 | 3.27e-09 | 6.69e-08 |
| ENSMUSG00000035297 | Cops4 | protein\_coding | 5:100518309-100547803 (+) |  | -0.2950 | 3.36e-09 | 6.88e-08 |
| ENSMUSG00000068551 | Zfp467 | protein\_coding | 6:48427697-48445825 (-) |  | 0.6730 | 3.37e-09 | 6.88e-08 |
| ENSMUSG00000027589 | Pcmtd2 | protein\_coding | 2:181837854-181857461 (+) |  | 0.4020 | 3.40e-09 | 6.93e-08 |
| ENSMUSG00000043207 | Zmpste24 | protein\_coding | 4:121059237-121098241 (-) |  | -0.3260 | 3.49e-09 | 7.11e-08 |
| ENSMUSG00000029086 | Prom1 | protein\_coding | 5:43993620-44102032 (-) |  | -1.6200 | 3.51e-09 | 7.14e-08 |
| ENSMUSG00000028902 | Sf3a3 | protein\_coding | 4:124714776-124732460 (+) |  | -0.2820 | 3.52e-09 | 7.15e-08 |
| ENSMUSG00000111171 | Gm47815 | lncRNA | 10:43034993-43050219 (+) |  | 1.0200 | 3.55e-09 | 7.21e-08 |
| ENSMUSG00000030105 | Arl8b | protein\_coding | 6:108783099-108825278 (+) |  | -0.2390 | 3.58e-09 | 7.24e-08 |
| ENSMUSG00000038332 | Sesn1 | protein\_coding | 10:41809935-41908424 (+) |  | 0.4310 | 3.58e-09 | 7.24e-08 |
| ENSMUSG00000040723 | Rcsd1 | protein\_coding | 1:165646516-165709757 (-) |  | 0.1900 | 3.68e-09 | 7.44e-08 |
| ENSMUSG00000028101 | Pias3 | protein\_coding | 3:96696384-96706070 (+) |  | 0.4370 | 3.73e-09 | 7.52e-08 |
| ENSMUSG00000015745 | Plekho1 | protein\_coding | 3:95988429-95996001 (-) |  | 0.4100 | 3.83e-09 | 7.71e-08 |
| ENSMUSG00000022265 | Ank | protein\_coding | 15:27466677-27594909 (+) |  | 0.4260 | 3.86e-09 | 7.76e-08 |
| ENSMUSG00000042487 | Leo1 | protein\_coding | 9:75441524-75466432 (+) |  | -0.3810 | 3.91e-09 | 7.86e-08 |
| ENSMUSG00000062070 | Pgk1 | protein\_coding | X:106187100-106203699 (+) |  | -0.4450 | 3.95e-09 | 7.93e-08 |
| ENSMUSG00000050350 | Gpr18 | protein\_coding | 14:121911253-121915781 (-) |  | 1.1100 | 4.01e-09 | 8.03e-08 |
| ENSMUSG00000030498 | Gas2 | protein\_coding | 7:51862015-51994975 (+) |  | 0.5870 | 4.07e-09 | 8.14e-08 |
| ENSMUSG00000024800 | Rpp30 | protein\_coding | 19:36083716-36104777 (+) |  | -0.4510 | 4.09e-09 | 8.17e-08 |
| ENSMUSG00000024982 | Zdhhc6 | protein\_coding | 19:55271291-55316032 (-) |  | -0.3030 | 4.09e-09 | 8.17e-08 |
| ENSMUSG00000022106 | Rcbtb2 | protein\_coding | 14:73123037-73207843 (+) |  | 0.4000 | 4.10e-09 | 8.18e-08 |
| ENSMUSG00000048007 | Timm8a1 | protein\_coding | X:134537256-134541865 (-) |  | -0.4510 | 4.20e-09 | 8.36e-08 |
| ENSMUSG00000005107 | Slc2a9 | protein\_coding | 5:38349273-38503143 (-) |  | 0.5480 | 4.21e-09 | 8.36e-08 |
| ENSMUSG00000022122 | Ednrb | protein\_coding | 14:103814625-103844402 (-) |  | -0.9950 | 4.27e-09 | 8.48e-08 |
| ENSMUSG00000017428 | Psmd11 | protein\_coding | 11:80428615-80473248 (+) |  | -0.3210 | 4.29e-09 | 8.49e-08 |
| ENSMUSG00000066621 | Tecpr1 | protein\_coding | 5:144194442-144223615 (-) |  | 0.5330 | 4.36e-09 | 8.62e-08 |
| ENSMUSG00000024084 | Qpct | protein\_coding | 17:79051906-79090378 (+) |  | 0.6690 | 4.48e-09 | 8.86e-08 |
| ENSMUSG00000110647 | Gm17745 | lncRNA | 10:93335393-93348877 (+) |  | 1.2100 | 4.51e-09 | 8.90e-08 |
| ENSMUSG00000028161 | Ppp3ca | protein\_coding | 3:136670124-136937727 (+) |  | 0.2400 | 4.53e-09 | 8.94e-08 |
| ENSMUSG00000020328 | Nudcd2 | protein\_coding | 11:40733667-40740046 (+) |  | -0.4100 | 4.55e-09 | 8.95e-08 |
| ENSMUSG00000023143 | Nagpa | protein\_coding | 16:5195289-5204012 (-) |  | -0.3440 | 4.55e-09 | 8.95e-08 |
| ENSMUSG00000042590 | Ipo11 | protein\_coding | 13:106794439-106936958 (-) |  | -0.3520 | 4.56e-09 | 8.96e-08 |
| ENSMUSG00000024436 | Mrps18b | protein\_coding | 17:35910379-35916389 (-) |  | -0.6050 | 4.65e-09 | 9.11e-08 |
| ENSMUSG00000030082 | Sec61a1 | protein\_coding | 6:88503579-88518905 (-) |  | -0.2950 | 4.92e-09 | 9.63e-08 |
| ENSMUSG00000073838 | Tufm | protein\_coding | 7:126487361-126490731 (+) |  | -0.3780 | 5.15e-09 | 1.01e-07 |
| ENSMUSG00000022014 | Epsti1 | protein\_coding | 14:77904239-78002657 (+) |  | 0.5020 | 5.19e-09 | 1.01e-07 |
| ENSMUSG00000022387 | Brd1 | protein\_coding | 15:88687034-88734233 (-) |  | 0.2310 | 5.20e-09 | 1.01e-07 |
| ENSMUSG00000034187 | Nsf | protein\_coding | 11:103821782-103954056 (-) |  | -0.3370 | 5.23e-09 | 1.02e-07 |
| ENSMUSG00000021427 | Ssr1 | protein\_coding | 13:37966605-37994217 (-) |  | -0.2940 | 5.29e-09 | 1.03e-07 |
| ENSMUSG00000027495 | Fam210b | protein\_coding | 2:172345565-172355749 (+) |  | 0.7240 | 5.29e-09 | 1.03e-07 |
| ENSMUSG00000022682 | Rrn3 | protein\_coding | 16:13780708-13814839 (+) |  | -0.2880 | 5.40e-09 | 1.05e-07 |
| ENSMUSG00000047264 | Zfp358 | protein\_coding | 8:3493138-3497208 (+) |  | 0.6160 | 5.61e-09 | 1.09e-07 |
| ENSMUSG00000020883 | Fbxl20 | protein\_coding | 11:98082556-98150403 (-) |  | 0.5840 | 5.66e-09 | 1.09e-07 |
| ENSMUSG00000030869 | Ndufab1 | protein\_coding | 7:122085403-122101886 (-) |  | -0.3680 | 5.73e-09 | 1.11e-07 |
| ENSMUSG00000024240 | Epc1 | protein\_coding | 18:6435951-6516108 (-) |  | 0.3010 | 5.77e-09 | 1.11e-07 |
| ENSMUSG00000005575 | Ube2m | protein\_coding | 7:13035120-13038275 (-) |  | -0.2980 | 5.79e-09 | 1.12e-07 |
| ENSMUSG00000052749 | Trim30b | protein\_coding | 7:104355382-104369884 (-) |  | -0.8540 | 5.85e-09 | 1.13e-07 |
| ENSMUSG00000033933 | Vhl | protein\_coding | 6:113623959-113631633 (+) |  | 0.3560 | 6.03e-09 | 1.16e-07 |
| ENSMUSG00000002477 | Snrpd1 | protein\_coding | 18:10617775-10642079 (+) |  | -0.4000 | 6.21e-09 | 1.19e-07 |
| ENSMUSG00000042046 | Dstyk | protein\_coding | 1:132417555-132466958 (+) |  | 0.4060 | 6.27e-09 | 1.20e-07 |
| ENSMUSG00000039697 | Ncoa7 | protein\_coding | 10:30628999-30803326 (-) |  | 0.3680 | 6.31e-09 | 1.21e-07 |
| ENSMUSG00000060860 | Ube2s | protein\_coding | 7:4794546-4812590 (-) |  | -0.4030 | 6.38e-09 | 1.22e-07 |
| ENSMUSG00000031149 | Praf2 | protein\_coding | X:7728439-7731064 (+) |  | -0.8250 | 6.41e-09 | 1.22e-07 |
| ENSMUSG00000020571 | Pdia6 | protein\_coding | 12:17266545-17284770 (+) |  | -0.4210 | 6.43e-09 | 1.23e-07 |
| ENSMUSG00000049295 | Zfp219 | protein\_coding | 14:52006077-52020733 (-) |  | 0.6020 | 6.50e-09 | 1.24e-07 |
| ENSMUSG00000023572 | Ccndbp1 | protein\_coding | 2:121008403-121016904 (+) |  | 0.3670 | 6.52e-09 | 1.24e-07 |
| ENSMUSG00000044072 | Eml6 | protein\_coding | 11:29743048-30026033 (-) |  | 1.1500 | 6.57e-09 | 1.25e-07 |
| ENSMUSG00000030930 | Chst15 | protein\_coding | 7:132235780-132317228 (-) |  | 1.0400 | 6.74e-09 | 1.28e-07 |
| ENSMUSG00000002668 | Dennd1c | protein\_coding | 17:57065905-57078514 (-) |  | 0.7220 | 6.75e-09 | 1.28e-07 |
| ENSMUSG00000037148 | Arhgap10 | protein\_coding | 8:77250366-77517953 (-) |  | -0.6900 | 6.75e-09 | 1.28e-07 |
| ENSMUSG00000032333 | Stoml1 | protein\_coding | 9:58253164-58262520 (+) |  | 0.5320 | 6.89e-09 | 1.30e-07 |
| ENSMUSG00000004665 | Cnn2 | protein\_coding | 10:79988584-79996062 (+) |  | 0.4530 | 6.90e-09 | 1.30e-07 |
| ENSMUSG00000021987 | Mtmr6 | protein\_coding | 14:60265228-60302370 (+) |  | 0.3070 | 6.94e-09 | 1.31e-07 |
| ENSMUSG00000027997 | Casp6 | protein\_coding | 3:129901425-129914103 (+) |  | -0.5740 | 6.96e-09 | 1.31e-07 |
| ENSMUSG00000026112 | Coa5 | protein\_coding | 1:37417084-37430103 (-) |  | -0.2510 | 7.10e-09 | 1.33e-07 |
| ENSMUSG00000027379 | Bub1 | protein\_coding | 2:127801122-127831865 (-) |  | -0.4090 | 7.20e-09 | 1.35e-07 |
| ENSMUSG00000002329 | Mdp1 | protein\_coding | 14:55657879-55660508 (-) |  | 0.3360 | 7.24e-09 | 1.36e-07 |
| ENSMUSG00000020720 | Psmd12 | protein\_coding | 11:107479484-107504362 (+) |  | -0.3720 | 7.27e-09 | 1.36e-07 |
| ENSMUSG00000030189 | Ybx3 | protein\_coding | 6:131364855-131388476 (-) |  | -0.4060 | 7.30e-09 | 1.37e-07 |
| ENSMUSG00000026972 | Arrdc1 | protein\_coding | 2:24925352-24935252 (-) |  | 0.3340 | 7.34e-09 | 1.37e-07 |
| ENSMUSG00000029599 | Ddx54 | protein\_coding | 5:120612739-120628592 (+) |  | -0.2550 | 7.41e-09 | 1.38e-07 |
| ENSMUSG00000037260 | Hgsnat | protein\_coding | 8:25944453-25976753 (-) |  | 0.4400 | 7.43e-09 | 1.38e-07 |
| ENSMUSG00000037465 | Klf10 | protein\_coding | 15:38291463-38300706 (-) |  | 0.5170 | 7.49e-09 | 1.39e-07 |
| ENSMUSG00000000384 | Tbrg4 | protein\_coding | 11:6615598-6626067 (-) |  | -0.3970 | 7.70e-09 | 1.43e-07 |
| ENSMUSG00000023452 | Pisd | protein\_coding | 5:32736301-32785646 (-) |  | 0.3010 | 7.72e-09 | 1.43e-07 |
| ENSMUSG00000078942 | Naip6 | protein\_coding | 13:100281121-100317674 (-) |  | 0.5220 | 7.85e-09 | 1.45e-07 |
| ENSMUSG00000052609 | Plekhg3 | protein\_coding | 12:76530891-76580488 (+) |  | 0.3230 | 7.86e-09 | 1.46e-07 |
| ENSMUSG00000034334 | Fam151b | protein\_coding | 13:92449625-92484015 (-) |  | 0.7990 | 7.93e-09 | 1.47e-07 |
| ENSMUSG00000028953 | Abcf2 | protein\_coding | 5:24565345-24577467 (-) |  | -0.2910 | 7.96e-09 | 1.47e-07 |
| ENSMUSG00000032691 | Nlrp3 | protein\_coding | 11:59541568-59566956 (+) |  | 0.5560 | 8.02e-09 | 1.48e-07 |
| ENSMUSG00000071723 | Gspt2 | protein\_coding | X:94636069-94643244 (+) |  | 0.4370 | 8.23e-09 | 1.52e-07 |
| ENSMUSG00000024078 | Ttc27 | protein\_coding | 17:74717732-74863570 (+) |  | -0.4050 | 8.50e-09 | 1.56e-07 |
| ENSMUSG00000044197 | Gpr146 | protein\_coding | 5:139377697-139396415 (+) |  | -0.5610 | 8.57e-09 | 1.57e-07 |
| ENSMUSG00000026229 | Psmd1 | protein\_coding | 1:86064387-86139151 (+) |  | -0.3440 | 8.62e-09 | 1.58e-07 |
| ENSMUSG00000024480 | Ap3s1 | protein\_coding | 18:46741876-46790826 (+) |  | -0.5360 | 8.66e-09 | 1.59e-07 |
| ENSMUSG00000041891 | Lman1 | protein\_coding | 18:65980738-66022580 (-) |  | -0.3210 | 8.76e-09 | 1.60e-07 |
| ENSMUSG00000036078 | Sigmar1 | protein\_coding | 4:41738493-41756157 (-) |  | -0.3150 | 9.10e-09 | 1.66e-07 |
| ENSMUSG00000060477 | Irak2 | protein\_coding | 6:113638467-113695026 (+) |  | 0.5180 | 9.13e-09 | 1.67e-07 |
| ENSMUSG00000038732 | Mboat1 | protein\_coding | 13:30136489-30246717 (+) |  | -0.6780 | 9.63e-09 | 1.76e-07 |
| ENSMUSG00000069539 | Scyl2 | protein\_coding | 10:89638721-89686285 (-) |  | -0.3330 | 9.76e-09 | 1.78e-07 |
| ENSMUSG00000064147 | Rab44 | protein\_coding | 17:29114145-29148980 (+) |  | 0.3420 | 9.78e-09 | 1.78e-07 |
| ENSMUSG00000025889 | Snca | protein\_coding | 6:60731575-60829855 (-) |  | 2.1400 | 9.78e-09 | 1.78e-07 |
| ENSMUSG00000052533 | Nup188 | protein\_coding | 2:30286397-30344266 (+) |  | -0.3630 | 9.89e-09 | 1.79e-07 |
| ENSMUSG00000048329 | Mfsd6l | protein\_coding | 11:68556186-68558245 (+) |  | 0.8940 | 9.89e-09 | 1.79e-07 |
| ENSMUSG00000031834 | Pik3r2 | protein\_coding | 8:70768176-70776713 (-) |  | -0.3070 | 1.01e-08 | 1.83e-07 |
| ENSMUSG00000027715 | Ccna2 | protein\_coding | 3:36564865-36572150 (-) |  | -0.3530 | 1.03e-08 | 1.86e-07 |
| ENSMUSG00000079499 | 6530402F18Rik | lncRNA | 2:29245107-29253006 (-) |  | -0.4520 | 1.04e-08 | 1.87e-07 |
| ENSMUSG00000027698 | Nceh1 | protein\_coding | 3:27182965-27284608 (+) |  | 0.3720 | 1.04e-08 | 1.87e-07 |
| ENSMUSG00000035725 | Prkx | protein\_coding | X:77761411-77796278 (-) |  | -0.3720 | 1.08e-08 | 1.94e-07 |
| ENSMUSG00000022111 | Uchl3 | protein\_coding | 14:101653967-101696125 (+) |  | -0.4180 | 1.09e-08 | 1.96e-07 |
| ENSMUSG00000020649 | Rrm2 | protein\_coding | 12:24708241-24714146 (+) |  | -0.4610 | 1.10e-08 | 1.97e-07 |
| ENSMUSG00000021038 | Vipas39 | protein\_coding | 12:87238868-87266256 (-) |  | -0.3610 | 1.10e-08 | 1.98e-07 |
| ENSMUSG00000037458 | Azin1 | protein\_coding | 15:38487427-38519266 (-) |  | -0.2320 | 1.11e-08 | 2.00e-07 |
| ENSMUSG00000025995 | Wdr75 | protein\_coding | 1:45795166-45823619 (+) |  | -0.3880 | 1.12e-08 | 2.00e-07 |
| ENSMUSG00000107761 | 2010008C14Rik | TEC | 6:125083437-125084029 (+) |  | 0.6580 | 1.12e-08 | 2.00e-07 |
| ENSMUSG00000027823 | Gmps | protein\_coding | 3:63976106-64022579 (+) |  | -0.2740 | 1.12e-08 | 2.01e-07 |
| ENSMUSG00000031232 | Magt1 | protein\_coding | X:105968084-106011906 (-) |  | -0.2670 | 1.14e-08 | 2.03e-07 |
| ENSMUSG00000047676 | Rpsa-ps10 | processed\_pseudogene | 3:150072658-150073542 (-) |  | -0.3960 | 1.14e-08 | 2.04e-07 |
| ENSMUSG00000025212 | Sfxn3 | protein\_coding | 19:45047503-45056383 (+) |  | 0.4430 | 1.16e-08 | 2.06e-07 |
| ENSMUSG00000028789 | Azin2 | protein\_coding | 4:128930233-128962442 (-) |  | 1.5100 | 1.17e-08 | 2.07e-07 |
| ENSMUSG00000003134 | Tbc1d8 | protein\_coding | 1:39371492-39478755 (-) |  | 0.5070 | 1.17e-08 | 2.08e-07 |
| ENSMUSG00000004552 | Ctse | protein\_coding | 1:131638306-131675505 (+) |  | 0.4860 | 1.18e-08 | 2.08e-07 |
| ENSMUSG00000029416 | Slc15a4 | protein\_coding | 5:127595664-127632897 (-) |  | 0.3240 | 1.22e-08 | 2.15e-07 |
| ENSMUSG00000020692 | Nle1 | protein\_coding | 11:82900768-82908411 (-) |  | -0.4740 | 1.28e-08 | 2.26e-07 |
| ENSMUSG00000023067 | Cdkn1a | protein\_coding | 17:29090976-29100727 (+) |  | -0.5010 | 1.28e-08 | 2.27e-07 |
| ENSMUSG00000029468 | P2rx7 | protein\_coding | 5:122643911-122691432 (+) |  | 0.3480 | 1.29e-08 | 2.28e-07 |
| ENSMUSG00000029171 | Pgm2 | protein\_coding | 5:64092950-64128351 (+) |  | -0.2910 | 1.29e-08 | 2.28e-07 |
| ENSMUSG00000082292 | Gm12250 | processed\_pseudogene | 11:58187739-58189012 (+) |  | -0.6310 | 1.32e-08 | 2.33e-07 |
| ENSMUSG00000028599 | Tnfrsf1b | protein\_coding | 4:145213463-145246870 (-) |  | 0.3810 | 1.33e-08 | 2.33e-07 |
| ENSMUSG00000075010 | AW112010 | lncRNA | 19:11047612-11055808 (-) |  | -0.8810 | 1.33e-08 | 2.33e-07 |
| ENSMUSG00000048758 | Rpl29 | protein\_coding | 9:106429454-106431568 (+) |  | -0.3260 | 1.34e-08 | 2.34e-07 |
| ENSMUSG00000021660 | Btf3 | protein\_coding | 13:98309896-98317006 (-) |  | -0.3110 | 1.35e-08 | 2.36e-07 |
| ENSMUSG00000052825 | Gm9892 | processed\_pseudogene | 8:52196065-52197056 (-) |  | -0.5160 | 1.37e-08 | 2.40e-07 |
| ENSMUSG00000018548 | Trim37 | protein\_coding | 11:87127077-87220683 (+) |  | -0.3340 | 1.38e-08 | 2.40e-07 |
| ENSMUSG00000021710 | Nln | protein\_coding | 13:104023057-104109614 (-) |  | -0.2670 | 1.38e-08 | 2.41e-07 |
| ENSMUSG00000068959 | Zfp619 | protein\_coding | 7:39517766-39540420 (+) |  | 0.6780 | 1.39e-08 | 2.41e-07 |
| ENSMUSG00000048058 | Ldlrad3 | protein\_coding | 2:101950203-102186385 (-) |  | 0.6000 | 1.39e-08 | 2.42e-07 |
| ENSMUSG00000028134 | Ptbp2 | protein\_coding | 3:119718742-119784466 (-) |  | 0.5590 | 1.40e-08 | 2.42e-07 |
| ENSMUSG00000021771 | Vdac2 | protein\_coding | 14:21825238-21845879 (+) |  | -0.2450 | 1.40e-08 | 2.42e-07 |
| ENSMUSG00000039234 | Sec24d | protein\_coding | 3:123267455-123365641 (+) |  | -0.4970 | 1.42e-08 | 2.45e-07 |
| ENSMUSG00000035953 | Pip4p1 | protein\_coding | 14:50926068-50930856 (-) |  | 0.3550 | 1.43e-08 | 2.47e-07 |
| ENSMUSG00000028683 | Eif2b3 | protein\_coding | 4:117019402-117087306 (+) |  | -0.4760 | 1.45e-08 | 2.51e-07 |
| ENSMUSG00000022698 | Naa50 | protein\_coding | 16:44139830-44163366 (+) |  | -0.3480 | 1.46e-08 | 2.53e-07 |
| ENSMUSG00000067787 | Blcap | protein\_coding | 2:157556362-157571274 (-) |  | 0.4330 | 1.47e-08 | 2.53e-07 |
| ENSMUSG00000070348 | Ccnd1 | protein\_coding | 7:144929931-144939925 (-) |  | 0.8640 | 1.47e-08 | 2.53e-07 |
| ENSMUSG00000024896 | Minpp1 | protein\_coding | 19:32485769-32515364 (+) |  | -0.2960 | 1.47e-08 | 2.53e-07 |
| ENSMUSG00000063480 | Snu13 | protein\_coding | 15:82040525-82047598 (-) |  | -0.3860 | 1.48e-08 | 2.54e-07 |
| ENSMUSG00000027452 | Acss1 | protein\_coding | 2:150618105-150668500 (-) |  | 0.8190 | 1.50e-08 | 2.58e-07 |
| ENSMUSG00000056220 | Pla2g4a | protein\_coding | 1:149829618-149961290 (-) |  | -0.4320 | 1.53e-08 | 2.62e-07 |
| ENSMUSG00000046687 | Gm5424 | processed\_pseudogene | 10:62071123-62072362 (+) |  | -0.3900 | 1.54e-08 | 2.64e-07 |
| ENSMUSG00000018819 | Lsp1 | protein\_coding | 7:142460809-142494867 (+) |  | 0.4740 | 1.55e-08 | 2.64e-07 |
| ENSMUSG00000043017 | Ptgir | protein\_coding | 7:16906490-16910905 (+) |  | 0.7220 | 1.58e-08 | 2.71e-07 |
| ENSMUSG00000027901 | Dennd2d | protein\_coding | 3:106482405-106503030 (+) |  | -0.4320 | 1.59e-08 | 2.72e-07 |
| ENSMUSG00000099413 | Gm17767 | lncRNA | 1:51507086-51596718 (-) |  | -1.1500 | 1.65e-08 | 2.81e-07 |
| ENSMUSG00000026526 | Fh1 | protein\_coding | 1:175600374-175625635 (-) |  | -0.3100 | 1.65e-08 | 2.81e-07 |
| ENSMUSG00000030335 | Mrpl51 | protein\_coding | 6:125191801-125196269 (+) |  | -0.3340 | 1.66e-08 | 2.82e-07 |
| ENSMUSG00000032507 | Fbxl2 | protein\_coding | 9:113963637-114046191 (-) |  | -0.5540 | 1.67e-08 | 2.84e-07 |
| ENSMUSG00000028861 | Mrps15 | protein\_coding | 4:126046925-126055532 (+) |  | -0.3550 | 1.68e-08 | 2.85e-07 |
| ENSMUSG00000020358 | Hnrnpab | protein\_coding | 11:51600100-51606847 (-) |  | -0.3100 | 1.71e-08 | 2.90e-07 |
| ENSMUSG00000020775 | Mrpl38 | protein\_coding | 11:116131817-116138868 (-) |  | -0.3850 | 1.72e-08 | 2.92e-07 |
| ENSMUSG00000020475 | Pgam2 | protein\_coding | 11:5801640-5803733 (-) |  | 0.5180 | 1.76e-08 | 2.97e-07 |
| ENSMUSG00000025134 | Alyref | protein\_coding | 11:120592121-120598365 (-) |  | -0.3990 | 1.76e-08 | 2.98e-07 |
| ENSMUSG00000017002 | Slpi | protein\_coding | 2:164354070-164389095 (-) |  | 0.6250 | 1.78e-08 | 3.00e-07 |
| ENSMUSG00000021645 | Smn1 | protein\_coding | 13:100124852-100137690 (+) |  | -0.3350 | 1.80e-08 | 3.04e-07 |
| ENSMUSG00000059796 | Eif4a1 | protein\_coding | 11:69666936-69672423 (-) |  | -0.2970 | 1.81e-08 | 3.04e-07 |
| ENSMUSG00000029816 | Gpnmb | protein\_coding | 6:49036546-49070929 (+) |  | -0.8640 | 1.82e-08 | 3.06e-07 |
| ENSMUSG00000040010 | Slc7a5 | protein\_coding | 8:121881150-121907694 (-) |  | -0.6770 | 1.83e-08 | 3.08e-07 |
| ENSMUSG00000027583 | Zbtb46 | protein\_coding | 2:181387762-181459426 (-) |  | 1.2400 | 1.84e-08 | 3.08e-07 |
| ENSMUSG00000020849 | Ywhae | protein\_coding | 11:75732869-75765845 (+) |  | -0.2640 | 1.88e-08 | 3.15e-07 |
| ENSMUSG00000030983 | Bccip | protein\_coding | 7:133709333-133721145 (+) |  | -0.2720 | 1.90e-08 | 3.19e-07 |
| ENSMUSG00000003154 | Foxj2 | protein\_coding | 6:122819914-122845366 (+) |  | 0.5060 | 1.91e-08 | 3.19e-07 |
| ENSMUSG00000027203 | Dut | protein\_coding | 2:125247190-125258608 (+) |  | -0.3480 | 1.93e-08 | 3.22e-07 |
| ENSMUSG00000037224 | Zfyve28 | protein\_coding | 5:34194893-34288449 (-) |  | 1.8400 | 1.93e-08 | 3.22e-07 |
| ENSMUSG00000007050 | Lsm2 | protein\_coding | 17:34981862-34985891 (+) |  | -0.4130 | 1.94e-08 | 3.22e-07 |
| ENSMUSG00000018068 | Ints2 | protein\_coding | 11:86210681-86257575 (-) |  | -0.3800 | 1.97e-08 | 3.27e-07 |
| ENSMUSG00000031672 | Got2 | protein\_coding | 8:95864134-95888547 (-) |  | -0.4330 | 1.97e-08 | 3.27e-07 |
| ENSMUSG00000047293 | Gpr15 | protein\_coding | 16:58717433-58719070 (-) |  | -0.8660 | 1.98e-08 | 3.28e-07 |
| ENSMUSG00000021156 | Zmynd11 | protein\_coding | 13:9684833-9765330 (-) |  | 0.3090 | 2.02e-08 | 3.34e-07 |
| ENSMUSG00000019806 | Aig1 | protein\_coding | 10:13647054-13868980 (-) |  | -1.4500 | 2.04e-08 | 3.37e-07 |
| ENSMUSG00000001436 | Slc19a1 | protein\_coding | 10:77032241-77061002 (+) |  | -0.6650 | 2.04e-08 | 3.38e-07 |
| ENSMUSG00000027253 | Lrp4 | protein\_coding | 2:91457511-91513779 (+) |  | 0.5520 | 2.05e-08 | 3.39e-07 |
| ENSMUSG00000030216 | Wbp11 | protein\_coding | 6:136813654-136828233 (-) |  | -0.3470 | 2.09e-08 | 3.44e-07 |
| ENSMUSG00000058886 | Deaf1 | protein\_coding | 7:141297180-141327690 (-) |  | 0.3510 | 2.09e-08 | 3.44e-07 |
| ENSMUSG00000026974 | Zmynd19 | protein\_coding | 2:24949792-24962075 (+) |  | -0.4100 | 2.10e-08 | 3.45e-07 |
| ENSMUSG00000104350 | Gm38244 | TEC | 3:106478283-106480868 (+) |  | -1.1700 | 2.11e-08 | 3.48e-07 |
| ENSMUSG00000069919 | Hba-a1 | protein\_coding | 11:32283511-32284465 (+) |  | 2.2700 | 2.14e-08 | 3.51e-07 |
| ENSMUSG00000005683 | Cs | protein\_coding | 10:128337734-128362479 (+) |  | -0.2900 | 2.19e-08 | 3.60e-07 |
| ENSMUSG00000025324 | Atp10a | protein\_coding | 7:58656166-58829420 (+) |  | -0.3240 | 2.21e-08 | 3.62e-07 |
| ENSMUSG00000026019 | Wdr12 | protein\_coding | 1:60069785-60098645 (-) |  | -0.4000 | 2.24e-08 | 3.66e-07 |
| ENSMUSG00000063524 | Eno1 | protein\_coding | 4:150236721-150248879 (+) |  | -0.3700 | 2.25e-08 | 3.68e-07 |
| ENSMUSG00000014907 | Naf1 | protein\_coding | 8:66860217-66890564 (+) |  | -0.3910 | 2.28e-08 | 3.72e-07 |
| ENSMUSG00000021614 | Vcan | protein\_coding | 13:89655312-89742509 (-) |  | -2.3300 | 2.33e-08 | 3.80e-07 |
| ENSMUSG00000071711 | Mpst | protein\_coding | 15:78406416-78414013 (+) |  | -0.3480 | 2.35e-08 | 3.84e-07 |
| ENSMUSG00000038539 | Atf5 | protein\_coding | 7:44812256-44816658 (-) |  | -0.5980 | 2.37e-08 | 3.86e-07 |
| ENSMUSG00000028221 | Pip4p2 | protein\_coding | 4:14864076-14915176 (+) |  | -0.3830 | 2.38e-08 | 3.87e-07 |
| ENSMUSG00000005161 | Prdx2 | protein\_coding | 8:84969587-84974834 (+) |  | -0.3250 | 2.39e-08 | 3.88e-07 |
| ENSMUSG00000040111 | Gramd1b | protein\_coding | 9:40293233-40531383 (-) |  | 0.3430 | 2.45e-08 | 3.98e-07 |
| ENSMUSG00000095567 | Noc2l | protein\_coding | 4:156235919-156247616 (+) |  | -0.3200 | 2.51e-08 | 4.07e-07 |
| ENSMUSG00000018909 | Arrb1 | protein\_coding | 7:99535466-99606771 (+) |  | 0.3900 | 2.51e-08 | 4.07e-07 |
| ENSMUSG00000029923 | Rab19 | protein\_coding | 6:39381175-39390380 (+) |  | 0.9030 | 2.54e-08 | 4.11e-07 |
| ENSMUSG00000073678 | Pgap1 | protein\_coding | 1:54472994-54557684 (-) |  | 0.7570 | 2.62e-08 | 4.23e-07 |
| ENSMUSG00000047250 | Ptgs1 | protein\_coding | 2:36230426-36252272 (+) |  | 0.8010 | 2.63e-08 | 4.24e-07 |
| ENSMUSG00000029614 | Rpl6 | protein\_coding | 5:121204481-121209241 (+) |  | -0.1960 | 2.71e-08 | 4.38e-07 |
| ENSMUSG00000047547 | Cltb | protein\_coding | 13:54592401-54611344 (-) |  | -0.3880 | 2.72e-08 | 4.38e-07 |
| ENSMUSG00000054263 | Lifr | protein\_coding | 15:7090614-7197489 (+) |  | -1.1700 | 2.85e-08 | 4.58e-07 |
| ENSMUSG00000004040 | Stat3 | protein\_coding | 11:100885098-100939540 (-) |  | -0.3410 | 2.88e-08 | 4.63e-07 |
| ENSMUSG00000022419 | Deptor | protein\_coding | 15:55112317-55259271 (+) |  | 0.3440 | 2.88e-08 | 4.63e-07 |
| ENSMUSG00000034795 | Ccdc122 | protein\_coding | 14:77036772-77112257 (+) |  | 0.7910 | 2.89e-08 | 4.63e-07 |
| ENSMUSG00000009563 | Tor2a | protein\_coding | 2:32757234-32762244 (+) |  | -0.3410 | 2.93e-08 | 4.69e-07 |
| ENSMUSG00000031995 | St14 | protein\_coding | 9:31089402-31131853 (-) |  | 0.5740 | 2.95e-08 | 4.72e-07 |
| ENSMUSG00000024007 | Ppil1 | protein\_coding | 17:29250803-29264186 (-) |  | -0.4180 | 2.96e-08 | 4.72e-07 |
| ENSMUSG00000037822 | Smim14 | protein\_coding | 5:65446844-65537184 (-) |  | 0.4060 | 2.96e-08 | 4.72e-07 |
| ENSMUSG00000032939 | Nup93 | protein\_coding | 8:94214564-94317227 (+) |  | -0.3310 | 2.96e-08 | 4.72e-07 |
| ENSMUSG00000044456 | Rin3 | protein\_coding | 12:102283048-102390855 (+) |  | 0.3840 | 2.96e-08 | 4.72e-07 |
| ENSMUSG00000019916 | P4ha1 | protein\_coding | 10:59323296-59373304 (+) |  | -0.3900 | 3.13e-08 | 4.99e-07 |
| ENSMUSG00000034792 | Gna15 | protein\_coding | 10:81502306-81524225 (-) |  | 0.4550 | 3.15e-08 | 5.00e-07 |
| ENSMUSG00000041836 | Ptpre | protein\_coding | 7:135537481-135686293 (+) |  | 0.3300 | 3.17e-08 | 5.03e-07 |
| ENSMUSG00000034850 | Tmem127 | protein\_coding | 2:127247908-127261107 (+) |  | 0.1990 | 3.21e-08 | 5.09e-07 |
| ENSMUSG00000054619 | Mettl7a1 | protein\_coding | 15:100304140-100328662 (+) |  | 0.4090 | 3.23e-08 | 5.12e-07 |
| ENSMUSG00000032349 | Elovl5 | protein\_coding | 9:77917364-77984519 (+) |  | -0.2330 | 3.25e-08 | 5.14e-07 |
| ENSMUSG00000038205 | Prkab2 | protein\_coding | 3:97658193-97673812 (+) |  | 0.5650 | 3.26e-08 | 5.15e-07 |
| ENSMUSG00000029147 | Ppm1g | protein\_coding | 5:31202664-31220687 (-) |  | -0.2800 | 3.30e-08 | 5.20e-07 |
| ENSMUSG00000022136 | Dnajc3 | protein\_coding | 14:118937976-118981697 (+) |  | -0.2500 | 3.30e-08 | 5.20e-07 |
| ENSMUSG00000043157 | Arl11 | protein\_coding | 14:61309753-61311936 (+) |  | 0.4230 | 3.38e-08 | 5.33e-07 |
| ENSMUSG00000110331 | Nudc-ps1 | processed\_pseudogene | 8:29286431-29286981 (-) |  | -0.4320 | 3.48e-08 | 5.48e-07 |
| ENSMUSG00000049090 | Zadh2 | protein\_coding | 18:84088063-84097528 (+) |  | 0.3290 | 3.50e-08 | 5.50e-07 |
| ENSMUSG00000020120 | Plek | protein\_coding | 11:16971206-17052381 (-) |  | 0.5860 | 3.52e-08 | 5.53e-07 |
| ENSMUSG00000038437 | Mllt6 | protein\_coding | 11:97663414-97685463 (+) |  | 0.5850 | 3.52e-08 | 5.53e-07 |
| ENSMUSG00000037295 | Ldlrap1 | protein\_coding | 4:134741554-134768024 (-) |  | 0.3620 | 3.54e-08 | 5.54e-07 |
| ENSMUSG00000037300 | Ttc13 | protein\_coding | 8:124671332-124721983 (-) |  | -0.2070 | 3.57e-08 | 5.58e-07 |
| ENSMUSG00000068856 | Sf3b4 | protein\_coding | 3:96172332-96177564 (+) |  | -0.3400 | 3.61e-08 | 5.64e-07 |
| ENSMUSG00000030257 | Srgap3 | protein\_coding | 6:112717971-112947266 (-) |  | 0.7240 | 3.63e-08 | 5.67e-07 |
| ENSMUSG00000028837 | Psmb2 | protein\_coding | 4:126677630-126709714 (+) |  | -0.2630 | 3.72e-08 | 5.80e-07 |
| ENSMUSG00000030047 | Arhgap25 | protein\_coding | 6:87458545-87533259 (-) |  | 0.4010 | 3.80e-08 | 5.92e-07 |
| ENSMUSG00000070858 | Gm1673 | protein\_coding | 5:33983433-33985013 (+) |  | 1.8600 | 3.85e-08 | 5.99e-07 |
| ENSMUSG00000056888 | Glipr1 | protein\_coding | 10:111985448-112002631 (-) |  | 0.4070 | 3.92e-08 | 6.10e-07 |
| ENSMUSG00000035890 | Rnf126 | protein\_coding | 10:79758515-79766952 (-) |  | -0.2810 | 4.00e-08 | 6.21e-07 |
| ENSMUSG00000030045 | Mrpl19 | protein\_coding | 6:81957851-81965958 (-) |  | -0.3730 | 4.04e-08 | 6.26e-07 |
| ENSMUSG00000012535 | Tnpo3 | protein\_coding | 6:29540827-29609887 (-) |  | -0.2420 | 4.07e-08 | 6.30e-07 |
| ENSMUSG00000027374 | Mrps5 | protein\_coding | 2:127587222-127606829 (+) |  | -0.4030 | 4.26e-08 | 6.59e-07 |
| ENSMUSG00000031634 | Ufsp2 | protein\_coding | 8:45975528-45996958 (+) |  | -0.3330 | 4.28e-08 | 6.62e-07 |
| ENSMUSG00000101188 | Eif4a-ps4 | processed\_pseudogene | 1:60703932-60705149 (+) |  | -0.3520 | 4.30e-08 | 6.64e-07 |
| ENSMUSG00000060594 | Layn | protein\_coding | 9:51054640-51077094 (-) |  | 0.7440 | 4.35e-08 | 6.71e-07 |
| ENSMUSG00000058794 | Nfe2 | protein\_coding | 15:103248212-103258403 (-) |  | 0.3110 | 4.38e-08 | 6.75e-07 |
| ENSMUSG00000031167 | Rbm3 | protein\_coding | X:8138975-8145880 (-) |  | 0.3680 | 4.40e-08 | 6.77e-07 |
| ENSMUSG00000048142 | Nat8l | protein\_coding | 5:33995984-34005916 (+) |  | 0.9610 | 4.40e-08 | 6.77e-07 |
| ENSMUSG00000022285 | Ywhaz | protein\_coding | 15:36770770-36796929 (-) |  | -0.2130 | 4.42e-08 | 6.80e-07 |
| ENSMUSG00000073968 | Trim68 | protein\_coding | 7:102677582-102687327 (-) |  | 0.6940 | 4.47e-08 | 6.86e-07 |
| ENSMUSG00000026434 | Nucks1 | protein\_coding | 1:131910534-131936321 (+) |  | -0.3900 | 4.49e-08 | 6.88e-07 |
| ENSMUSG00000026709 | Dars2 | protein\_coding | 1:161040601-161070658 (-) |  | -0.3510 | 4.49e-08 | 6.88e-07 |
| ENSMUSG00000033161 | Atp1a1 | protein\_coding | 3:101576219-101604684 (-) |  | -0.3140 | 4.54e-08 | 6.94e-07 |
| ENSMUSG00000021356 | Irf4 | protein\_coding | 13:30749226-30766976 (+) |  | 1.1700 | 4.54e-08 | 6.94e-07 |
| ENSMUSG00000024338 | Psmb8 | protein\_coding | 17:34197721-34201454 (+) |  | -0.3110 | 4.66e-08 | 7.11e-07 |
| ENSMUSG00000022403 | St13 | protein\_coding | 15:81363669-81400077 (-) |  | -0.3120 | 4.67e-08 | 7.12e-07 |
| ENSMUSG00000032727 | Mier3 | protein\_coding | 13:111680979-111718596 (+) |  | 0.2900 | 4.69e-08 | 7.15e-07 |
| ENSMUSG00000030357 | Fkbp4 | protein\_coding | 6:128429735-128438677 (-) |  | -0.4630 | 4.70e-08 | 7.16e-07 |
| ENSMUSG00000063160 | Numbl | protein\_coding | 7:27258433-27282144 (+) |  | 0.6140 | 4.72e-08 | 7.18e-07 |
| ENSMUSG00000057666 | Gapdh | protein\_coding | 6:125161715-125166467 (-) |  | -0.4530 | 4.72e-08 | 7.18e-07 |
| ENSMUSG00000021709 | Erbin | protein\_coding | 13:103818787-103920514 (-) |  | 0.2400 | 4.80e-08 | 7.28e-07 |
| ENSMUSG00000053716 | Dusp7 | protein\_coding | 9:106368632-106375724 (+) |  | 0.5110 | 4.85e-08 | 7.35e-07 |
| ENSMUSG00000022512 | Cldn1 | protein\_coding | 16:26356642-26371841 (-) |  | 1.5300 | 4.86e-08 | 7.35e-07 |
| ENSMUSG00000071337 | Tia1 | protein\_coding | 6:86404219-86433405 (+) |  | 0.3030 | 4.86e-08 | 7.35e-07 |
| ENSMUSG00000067038 | Rps12-ps3 | processed\_pseudogene | 19:59322371-59322766 (+) |  | -0.5660 | 4.91e-08 | 7.42e-07 |
| ENSMUSG00000022817 | Itgb5 | protein\_coding | 16:33829665-33949338 (+) |  | -0.3630 | 4.93e-08 | 7.44e-07 |
| ENSMUSG00000030689 | Ino80e | protein\_coding | 7:126850960-126862377 (-) |  | -0.2610 | 5.07e-08 | 7.64e-07 |
| ENSMUSG00000002409 | Dyrk1b | protein\_coding | 7:28179469-28187294 (+) |  | 0.6550 | 5.12e-08 | 7.71e-07 |
| ENSMUSG00000026142 | Rhbdd1 | protein\_coding | 1:82316452-82445366 (+) |  | 0.2980 | 5.18e-08 | 7.79e-07 |
| ENSMUSG00000029030 | Tprgl | protein\_coding | 4:154157485-154160666 (-) |  | 0.4070 | 5.18e-08 | 7.79e-07 |
| ENSMUSG00000038762 | Abcf1 | protein\_coding | 17:35956819-35969761 (-) |  | -0.2790 | 5.18e-08 | 7.79e-07 |
| ENSMUSG00000035199 | Arl6ip5 | protein\_coding | 6:97210689-97233315 (+) |  | 0.2680 | 5.24e-08 | 7.86e-07 |
| ENSMUSG00000016319 | Slc25a5 | protein\_coding | X:36795651-36798807 (+) |  | -0.2530 | 5.40e-08 | 8.09e-07 |
| ENSMUSG00000043671 | Dpy19l3 | protein\_coding | 7:35685165-35754454 (-) |  | 0.8910 | 5.42e-08 | 8.12e-07 |
| ENSMUSG00000038759 | Nup205 | protein\_coding | 6:35177421-35247596 (+) |  | -0.3120 | 5.43e-08 | 8.13e-07 |
| ENSMUSG00000036948 | Map11 | protein\_coding | 5:138259656-138264033 (-) |  | 0.2450 | 5.51e-08 | 8.24e-07 |
| ENSMUSG00000000594 | Gm2a | protein\_coding | 11:55098115-55113029 (+) |  | 0.2260 | 5.52e-08 | 8.25e-07 |
| ENSMUSG00000030055 | Rab43 | protein\_coding | 6:87788853-87812164 (-) |  | 0.5290 | 5.56e-08 | 8.29e-07 |
| ENSMUSG00000028333 | Anp32b | protein\_coding | 4:46450902-46472657 (+) |  | -0.3480 | 5.88e-08 | 8.77e-07 |
| ENSMUSG00000074657 | Kif5a | protein\_coding | 10:127225696-127263348 (-) |  | 1.0900 | 5.91e-08 | 8.79e-07 |
| ENSMUSG00000108621 | Gm37494 | lncRNA | 7:39544402-39580589 (+) |  | 0.3920 | 6.01e-08 | 8.93e-07 |
| ENSMUSG00000078652 | Psme3 | protein\_coding | 11:101316213-101323537 (+) |  | -0.2780 | 6.02e-08 | 8.93e-07 |
| ENSMUSG00000022587 | Ly6e | protein\_coding | 15:74955051-74959905 (+) |  | -0.3120 | 6.02e-08 | 8.93e-07 |
| ENSMUSG00000024270 | Slc39a6 | protein\_coding | 18:24579881-24603817 (-) |  | -0.2570 | 6.03e-08 | 8.94e-07 |
| ENSMUSG00000033703 | Fcsk | protein\_coding | 8:110882456-110902488 (-) |  | 0.5030 | 6.12e-08 | 9.06e-07 |
| ENSMUSG00000049866 | Arl4c | protein\_coding | 1:88673125-88702221 (-) |  | 0.5510 | 6.14e-08 | 9.08e-07 |
| ENSMUSG00000021595 | Nsun2 | protein\_coding | 13:69533746-69635780 (+) |  | -0.3540 | 6.19e-08 | 9.14e-07 |
| ENSMUSG00000024583 | Txnl1 | protein\_coding | 18:63661094-63708801 (-) |  | -0.3600 | 6.20e-08 | 9.15e-07 |
| ENSMUSG00000032185 | Carm1 | protein\_coding | 9:21546894-21592623 (+) |  | -0.3210 | 6.21e-08 | 9.16e-07 |
| ENSMUSG00000017132 | Cyth1 | protein\_coding | 11:118132019-118248592 (-) |  | 0.3240 | 6.22e-08 | 9.17e-07 |
| ENSMUSG00000040213 | Kyat3 | protein\_coding | 3:142701051-142746870 (+) |  | -0.5550 | 6.35e-08 | 9.35e-07 |
| ENSMUSG00000032042 | Srpr | protein\_coding | 9:35200175-35247973 (+) |  | -0.2380 | 6.42e-08 | 9.44e-07 |
| ENSMUSG00000031266 | Gla | protein\_coding | X:134588149-134601125 (-) |  | -0.4010 | 6.50e-08 | 9.54e-07 |
| ENSMUSG00000044533 | Rps2 | protein\_coding | 17:24718116-24721929 (+) |  | -0.4270 | 6.62e-08 | 9.71e-07 |
| ENSMUSG00000040907 | Atp1a3 | protein\_coding | 7:24978167-25005958 (-) |  | 0.4060 | 6.64e-08 | 9.73e-07 |
| ENSMUSG00000038175 | Mylip | protein\_coding | 13:45389742-45412022 (+) |  | 0.5670 | 6.73e-08 | 9.85e-07 |
| ENSMUSG00000033906 | Zdhhc15 | protein\_coding | X:104536969-104671064 (-) |  | 0.8250 | 6.76e-08 | 9.89e-07 |
| ENSMUSG00000020638 | Cmpk2 | protein\_coding | 12:26469204-26479837 (+) |  | 0.3790 | 6.85e-08 | 1.00e-06 |
| ENSMUSG00000002797 | Ggct | protein\_coding | 6:54982580-54992950 (-) |  | -0.6170 | 6.98e-08 | 1.02e-06 |
| ENSMUSG00000001128 | Cfp | protein\_coding | X:20925454-20931555 (-) |  | 0.3500 | 6.99e-08 | 1.02e-06 |
| ENSMUSG00000030662 | Ipo5 | protein\_coding | 14:120911224-120947999 (+) |  | -0.3700 | 7.12e-08 | 1.04e-06 |
| ENSMUSG00000032115 | Hyou1 | protein\_coding | 9:44379490-44392369 (+) |  | -0.4310 | 7.14e-08 | 1.04e-06 |
| ENSMUSG00000000318 | Clec10a | protein\_coding | 11:70156197-70170834 (+) |  | 0.4200 | 7.18e-08 | 1.04e-06 |
| ENSMUSG00000028937 | Acot7 | protein\_coding | 4:152178134-152271855 (+) |  | -0.2850 | 7.21e-08 | 1.05e-06 |
| ENSMUSG00000041459 | Tardbp | protein\_coding | 4:148612382-148627019 (-) |  | -0.2460 | 7.26e-08 | 1.05e-06 |
| ENSMUSG00000078429 | Ctdsp2 | protein\_coding | 10:126978717-126999975 (+) |  | 0.2960 | 7.28e-08 | 1.06e-06 |
| ENSMUSG00000024769 | Cdc42bpg | protein\_coding | 19:6306456-6325652 (+) |  | 0.8460 | 7.40e-08 | 1.07e-06 |
| ENSMUSG00000057729 | Prtn3 | protein\_coding | 10:79874476-79883174 (+) |  | -1.0100 | 7.44e-08 | 1.08e-06 |
| ENSMUSG00000024997 | Prdx3 | protein\_coding | 19:60864051-60874556 (-) |  | -0.2790 | 7.57e-08 | 1.09e-06 |
| ENSMUSG00000020859 | Spag9 | protein\_coding | 11:93996091-94126085 (+) |  | 0.2880 | 7.58e-08 | 1.09e-06 |
| ENSMUSG00000020696 | Rffl | protein\_coding | 11:82802449-82871210 (-) |  | 0.3830 | 7.89e-08 | 1.14e-06 |
| ENSMUSG00000044037 | Als2cl | protein\_coding | 9:110879870-110900530 (+) |  | 0.9820 | 7.93e-08 | 1.14e-06 |
| ENSMUSG00000026580 | Selp | protein\_coding | 1:164115264-164150026 (+) |  | -1.8900 | 7.93e-08 | 1.14e-06 |
| ENSMUSG00000016494 | Cd34 | protein\_coding | 1:194938819-194961279 (+) |  | 0.8580 | 7.96e-08 | 1.14e-06 |
| ENSMUSG00000024187 | Fam234a | protein\_coding | 17:26211822-26244242 (-) |  | 0.4480 | 8.11e-08 | 1.16e-06 |
| ENSMUSG00000029587 | Zfp12 | protein\_coding | 5:143235163-143248834 (+) |  | 0.4110 | 8.17e-08 | 1.17e-06 |
| ENSMUSG00000087150 | BC064078 | transcribed\_unprocessed\_pseudogene | 6:128992952-129008040 (+) |  | 0.6650 | 8.22e-08 | 1.18e-06 |
| ENSMUSG00000024067 | Dpy30 | protein\_coding | 17:74299474-74323944 (-) |  | -0.3430 | 8.25e-08 | 1.18e-06 |
| ENSMUSG00000020941 | Map3k14 | protein\_coding | 11:103219762-103267472 (-) |  | 0.3980 | 8.25e-08 | 1.18e-06 |
| ENSMUSG00000015568 | Lpl | protein\_coding | 8:68880491-68907448 (+) |  | 1.1300 | 8.37e-08 | 1.20e-06 |
| ENSMUSG00000053931 | Cnn3 | protein\_coding | 3:121426497-121458207 (+) |  | 0.7680 | 8.43e-08 | 1.20e-06 |
| ENSMUSG00000067878 | Map7d3 | protein\_coding | X:56797858-56822326 (-) |  | 0.4840 | 8.55e-08 | 1.22e-06 |
| ENSMUSG00000046062 | Ppp1r15b | protein\_coding | 1:133131143-133139783 (+) |  | -0.2580 | 8.59e-08 | 1.22e-06 |
| ENSMUSG00000056153 | Socs6 | protein\_coding | 18:88665224-88927481 (-) |  | 0.3670 | 8.62e-08 | 1.23e-06 |
| ENSMUSG00000069805 | Fbp1 | protein\_coding | 13:62864753-62888282 (-) |  | 1.6400 | 8.64e-08 | 1.23e-06 |
| ENSMUSG00000037649 | H2-DMa | protein\_coding | 17:34119541-34139101 (+) |  | 0.4020 | 8.85e-08 | 1.26e-06 |
| ENSMUSG00000021254 | Gpatch2l | protein\_coding | 12:86241858-86291784 (+) |  | 0.2920 | 9.23e-08 | 1.31e-06 |
| ENSMUSG00000024397 | Aif1 | protein\_coding | 17:35170991-35176068 (-) |  | 0.5720 | 9.26e-08 | 1.31e-06 |
| ENSMUSG00000040016 | Ptger3 | protein\_coding | 3:157566892-157645888 (+) |  | -1.8500 | 9.28e-08 | 1.31e-06 |
| ENSMUSG00000040688 | Tbl3 | protein\_coding | 17:24697949-24707660 (-) |  | -0.3340 | 9.35e-08 | 1.32e-06 |
| ENSMUSG00000043740 | B430306N03Rik | protein\_coding | 17:48316141-48327024 (+) |  | 0.5230 | 9.37e-08 | 1.32e-06 |
| ENSMUSG00000048087 | Gm4737 | protein\_coding | 16:46152985-46155077 (-) |  | -0.6220 | 9.37e-08 | 1.32e-06 |
| ENSMUSG00000026192 | Atic | protein\_coding | 1:71557150-71579631 (+) |  | -0.4050 | 9.46e-08 | 1.34e-06 |
| ENSMUSG00000032126 | Hmbs | protein\_coding | 9:44336339-44344228 (-) |  | -0.3410 | 9.49e-08 | 1.34e-06 |
| ENSMUSG00000021929 | Kpna3 | protein\_coding | 14:61365211-61439874 (-) |  | -0.2810 | 9.82e-08 | 1.38e-06 |
| ENSMUSG00000081534 | Slc48a1 | protein\_coding | 15:97778520-97792692 (+) |  | 0.3110 | 9.88e-08 | 1.39e-06 |
| ENSMUSG00000035772 | Mrps2 | protein\_coding | 2:28468066-28471178 (+) |  | -0.2560 | 9.89e-08 | 1.39e-06 |
| ENSMUSG00000005481 | Ddx39 | protein\_coding | 8:83715177-83726892 (+) |  | -0.3480 | 9.90e-08 | 1.39e-06 |
| ENSMUSG00000019768 | Esr1 | protein\_coding | 10:4611593-5005614 (+) |  | 0.5160 | 9.97e-08 | 1.40e-06 |
| ENSMUSG00000024544 | Ldlrad4 | protein\_coding | 18:67933257-68268630 (+) |  | 1.0300 | 1.00e-07 | 1.41e-06 |
| ENSMUSG00000029247 | Paics | protein\_coding | 5:76951307-76967509 (+) |  | -0.2930 | 1.00e-07 | 1.41e-06 |
| ENSMUSG00000029534 | St7 | protein\_coding | 6:17692933-17943025 (+) |  | -0.5200 | 1.01e-07 | 1.41e-06 |
| ENSMUSG00000001281 | Itgb7 | protein\_coding | 15:102215995-102231944 (-) |  | 0.3260 | 1.02e-07 | 1.43e-06 |
| ENSMUSG00000022792 | Yars2 | protein\_coding | 16:16302965-16309640 (+) |  | -0.4320 | 1.06e-07 | 1.47e-06 |
| ENSMUSG00000032932 | Hspa13 | protein\_coding | 16:75745431-75767104 (-) |  | -0.2800 | 1.06e-07 | 1.48e-06 |
| ENSMUSG00000002658 | Gtf2f1 | protein\_coding | 17:57003405-57011288 (-) |  | -0.2680 | 1.06e-07 | 1.48e-06 |
| ENSMUSG00000060568 | Fam78b | protein\_coding | 1:167001417-167091302 (+) |  | 1.3800 | 1.06e-07 | 1.48e-06 |
| ENSMUSG00000000628 | Hk2 | protein\_coding | 6:82725025-82774454 (-) |  | 0.3450 | 1.06e-07 | 1.48e-06 |
| ENSMUSG00000021131 | Erh | protein\_coding | 12:80634022-80644341 (-) |  | -0.4330 | 1.07e-07 | 1.49e-06 |
| ENSMUSG00000021111 | Papola | protein\_coding | 12:105784694-105838944 (+) |  | -0.2200 | 1.09e-07 | 1.51e-06 |
| ENSMUSG00000023110 | Prmt5 | protein\_coding | 14:54507187-54517525 (-) |  | -0.3480 | 1.12e-07 | 1.55e-06 |
| ENSMUSG00000022797 | Tfrc | protein\_coding | 16:32608920-32632794 (+) |  | -0.5630 | 1.14e-07 | 1.57e-06 |
| ENSMUSG00000075602 | Ly6a | protein\_coding | 15:74994877-74998031 (-) |  | -1.6000 | 1.14e-07 | 1.58e-06 |
| ENSMUSG00000021069 | Pygl | protein\_coding | 12:70190811-70231488 (-) |  | 0.3370 | 1.14e-07 | 1.58e-06 |
| ENSMUSG00000039640 | Mrpl12 | protein\_coding | 11:120484613-120489065 (+) |  | -0.4690 | 1.15e-07 | 1.59e-06 |
| ENSMUSG00000000711 | Rab5b | protein\_coding | 10:128677175-128696264 (-) |  | 0.2190 | 1.17e-07 | 1.62e-06 |
| ENSMUSG00000042426 | Dhx29 | protein\_coding | 13:112927454-112969432 (+) |  | -0.2750 | 1.17e-07 | 1.62e-06 |
| ENSMUSG00000034210 | Efcab14 | protein\_coding | 4:115737744-115777327 (+) |  | -0.3380 | 1.19e-07 | 1.64e-06 |
| ENSMUSG00000042770 | Hebp1 | protein\_coding | 6:135137522-135198022 (-) |  | -0.7860 | 1.21e-07 | 1.66e-06 |
| ENSMUSG00000021969 | Zdhhc20 | protein\_coding | 14:57832703-57890276 (-) |  | 0.3410 | 1.21e-07 | 1.66e-06 |
| ENSMUSG00000022557 | Bop1 | protein\_coding | 15:76452989-76477277 (-) |  | -0.3320 | 1.22e-07 | 1.68e-06 |
| ENSMUSG00000031171 | Ftsj1 | protein\_coding | X:8238668-8252406 (-) |  | -0.3710 | 1.22e-07 | 1.68e-06 |
| ENSMUSG00000103041 | Gm37305 | lncRNA | 3:65957758-65962036 (+) |  | 0.5370 | 1.23e-07 | 1.69e-06 |
| ENSMUSG00000024683 | Mrpl16 | protein\_coding | 19:11770391-11774960 (+) |  | -0.3230 | 1.24e-07 | 1.69e-06 |
| ENSMUSG00000037851 | Iars | protein\_coding | 13:49682100-49734267 (+) |  | -0.3880 | 1.24e-07 | 1.70e-06 |
| ENSMUSG00000022477 | Aco2 | protein\_coding | 15:81872309-81915133 (+) |  | -0.2860 | 1.27e-07 | 1.74e-06 |
| ENSMUSG00000046897 | Zfp740 | protein\_coding | 15:102203249-102215606 (+) |  | 0.2290 | 1.28e-07 | 1.75e-06 |
| ENSMUSG00000041797 | Abca9 | protein\_coding | 11:110100749-110168196 (-) |  | 0.9240 | 1.29e-07 | 1.76e-06 |
| ENSMUSG00000076441 | Ass1 | protein\_coding | 2:31470207-31520672 (+) |  | -0.4240 | 1.30e-07 | 1.77e-06 |
| ENSMUSG00000003131 | Pafah1b2 | protein\_coding | 9:45962859-46012690 (-) |  | -0.2100 | 1.30e-07 | 1.78e-06 |
| ENSMUSG00000000386 | Mx1 | polymorphic\_pseudogene | 16:97447035-97462907 (-) |  | 0.6540 | 1.32e-07 | 1.79e-06 |
| ENSMUSG00000031539 | Ap3m2 | protein\_coding | 8:22787354-22805622 (-) |  | 0.6070 | 1.33e-07 | 1.81e-06 |
| ENSMUSG00000020549 | Elac2 | protein\_coding | 11:64979038-65002069 (+) |  | -0.3190 | 1.34e-07 | 1.82e-06 |
| ENSMUSG00000027714 | Exosc9 | protein\_coding | 3:36552606-36565727 (+) |  | -0.3530 | 1.40e-07 | 1.90e-06 |
| ENSMUSG00000033446 | Lpar6 | protein\_coding | 14:73237895-73243294 (+) |  | 0.5080 | 1.41e-07 | 1.91e-06 |
| ENSMUSG00000037447 | Arid5a | protein\_coding | 1:36307733-36324029 (+) |  | -0.2940 | 1.42e-07 | 1.93e-06 |
| ENSMUSG00000015937 | Macroh2a1 | protein\_coding | 13:56073619-56136361 (-) |  | -0.2900 | 1.42e-07 | 1.93e-06 |
| ENSMUSG00000031659 | Adcy7 | protein\_coding | 8:88272403-88329962 (+) |  | 0.2730 | 1.46e-07 | 1.98e-06 |
| ENSMUSG00000061979 | Rcc1l | protein\_coding | 5:134148054-134176774 (-) |  | -0.4550 | 1.47e-07 | 1.98e-06 |
| ENSMUSG00000041920 | Slc16a6 | protein\_coding | 11:109450855-109473598 (-) |  | -0.4090 | 1.48e-07 | 2.00e-06 |
| ENSMUSG00000030298 | Sec13 | protein\_coding | 6:113728062-113740743 (-) |  | -0.2150 | 1.49e-07 | 2.01e-06 |
| ENSMUSG00000030088 | Aldh1l1 | protein\_coding | 6:90486427-90600203 (+) |  | -1.4400 | 1.51e-07 | 2.03e-06 |
| ENSMUSG00000038807 | Rap1gap2 | protein\_coding | 11:74383356-74610915 (-) |  | 0.5730 | 1.52e-07 | 2.05e-06 |
| ENSMUSG00000047454 | Gphn | protein\_coding | 12:78226379-78684772 (+) |  | -0.4060 | 1.54e-07 | 2.07e-06 |
| ENSMUSG00000043091 | Tuba1c | protein\_coding | 15:99029891-99038110 (+) |  | -0.3240 | 1.56e-07 | 2.09e-06 |
| ENSMUSG00000055053 | Nfic | protein\_coding | 10:81396186-81455635 (-) |  | 0.3850 | 1.59e-07 | 2.13e-06 |
| ENSMUSG00000022967 | Ifnar1 | protein\_coding | 16:91485238-91507441 (+) |  | 0.2290 | 1.59e-07 | 2.13e-06 |
| ENSMUSG00000027309 | 4930402H24Rik | protein\_coding | 2:130706200-130906406 (-) |  | 0.4230 | 1.60e-07 | 2.14e-06 |
| ENSMUSG00000033629 | Hacd3 | protein\_coding | 9:64986983-65021693 (-) |  | -0.3550 | 1.60e-07 | 2.15e-06 |
| ENSMUSG00000026926 | Pmpca | protein\_coding | 2:26389339-26397122 (+) |  | -0.2680 | 1.61e-07 | 2.15e-06 |
| ENSMUSG00000106734 | Gm20559 | lncRNA | 6:3333194-3346128 (-) |  | 0.7770 | 1.61e-07 | 2.15e-06 |
| ENSMUSG00000112825 | Gm9118 | processed\_pseudogene | 10:56497341-56498094 (+) |  | -0.5200 | 1.62e-07 | 2.16e-06 |
| ENSMUSG00000057388 | Mrpl18 | protein\_coding | 17:12911349-12916345 (-) |  | -0.3640 | 1.63e-07 | 2.18e-06 |
| ENSMUSG00000004952 | Rasa4 | protein\_coding | 5:136083916-136111860 (+) |  | 0.2980 | 1.65e-07 | 2.19e-06 |
| ENSMUSG00000022453 | Naga | protein\_coding | 15:82329532-82338925 (-) |  | 0.2990 | 1.66e-07 | 2.21e-06 |
| ENSMUSG00000027333 | Smox | protein\_coding | 2:131491496-131525922 (+) |  | 0.5570 | 1.68e-07 | 2.23e-06 |
| ENSMUSG00000040197 | Cd209e | protein\_coding | 8:3847965-3854309 (-) |  | 2.9800 | 1.68e-07 | 2.23e-06 |
| ENSMUSG00000033632 | AW554918 | protein\_coding | 18:25168999-25467321 (+) |  | 0.4270 | 1.69e-07 | 2.25e-06 |
| ENSMUSG00000020708 | Psmc5 | protein\_coding | 11:106256154-106263120 (+) |  | -0.3340 | 1.70e-07 | 2.25e-06 |
| ENSMUSG00000068141 | Gm10232 | processed\_pseudogene | 17:3044014-3044733 (-) |  | -0.5060 | 1.71e-07 | 2.27e-06 |
| ENSMUSG00000003380 | Rabac1 | protein\_coding | 7:24969752-24972754 (-) |  | 0.4630 | 1.72e-07 | 2.28e-06 |
| ENSMUSG00000021892 | Sh3bp5 | protein\_coding | 14:31359880-31436078 (-) |  | 0.4430 | 1.74e-07 | 2.30e-06 |
| ENSMUSG00000078616 | Trim30c | protein\_coding | 7:104382065-104400837 (-) |  | -0.9010 | 1.74e-07 | 2.30e-06 |
| ENSMUSG00000033713 | Foxn3 | protein\_coding | 12:99190078-99563582 (-) |  | 0.4690 | 1.75e-07 | 2.31e-06 |
| ENSMUSG00000026822 | Lcn2 | protein\_coding | 2:32384633-32388252 (-) |  | -1.5100 | 1.76e-07 | 2.32e-06 |
| ENSMUSG00000033949 | Trim36 | protein\_coding | 18:46165300-46212607 (-) |  | 0.5080 | 1.76e-07 | 2.32e-06 |
| ENSMUSG00000034430 | Zxdc | protein\_coding | 6:90369492-90403490 (+) |  | 0.3580 | 1.80e-07 | 2.37e-06 |
| ENSMUSG00000024909 | Efemp2 | protein\_coding | 19:5473954-5482517 (+) |  | -1.3000 | 1.80e-07 | 2.37e-06 |
| ENSMUSG00000033685 | Ucp2 | protein\_coding | 7:100493337-100502020 (+) |  | 0.2750 | 1.82e-07 | 2.39e-06 |
| ENSMUSG00000002732 | Fkbp7 | protein\_coding | 2:76663044-76673116 (-) |  | 0.8290 | 1.83e-07 | 2.40e-06 |
| ENSMUSG00000032040 | Dcps | protein\_coding | 9:35124408-35176061 (-) |  | -0.2840 | 1.83e-07 | 2.40e-06 |
| ENSMUSG00000021947 | Cryl1 | protein\_coding | 14:57274993-57398529 (-) |  | 0.3820 | 1.86e-07 | 2.44e-06 |
| ENSMUSG00000018697 | Aatf | protein\_coding | 11:84422855-84513522 (-) |  | -0.3600 | 1.87e-07 | 2.44e-06 |
| ENSMUSG00000071669 | Snx29 | protein\_coding | 16:11322908-11755472 (+) |  | 0.6430 | 1.87e-07 | 2.45e-06 |
| ENSMUSG00000097328 | Tnfsf12 | protein\_coding | 11:69686250-69695849 (-) |  | 0.7260 | 1.88e-07 | 2.46e-06 |
| ENSMUSG00000031715 | Smarca5 | protein\_coding | 8:80698507-80739497 (-) |  | -0.2650 | 1.89e-07 | 2.47e-06 |
| ENSMUSG00000028088 | Fmo5 | protein\_coding | 3:97628804-97655282 (+) |  | 0.7550 | 1.90e-07 | 2.48e-06 |
| ENSMUSG00000055491 | Pprc1 | protein\_coding | 19:46044886-46072915 (+) |  | -0.4350 | 1.90e-07 | 2.48e-06 |
| ENSMUSG00000034413 | Neurl1b | protein\_coding | 17:26414829-26446349 (+) |  | 0.5370 | 1.91e-07 | 2.49e-06 |
| ENSMUSG00000026833 | Olfm1 | protein\_coding | 2:28192992-28230736 (+) |  | -0.4830 | 1.92e-07 | 2.49e-06 |
| ENSMUSG00000037992 | Rara | protein\_coding | 11:98927818-98974942 (+) |  | 0.4060 | 1.92e-07 | 2.50e-06 |
| ENSMUSG00000026068 | Il18rap | protein\_coding | 1:40515362-40551705 (+) |  | -1.9300 | 1.95e-07 | 2.53e-06 |
| ENSMUSG00000026807 | Ak8 | protein\_coding | 2:28700164-28813165 (+) |  | 0.8040 | 2.00e-07 | 2.59e-06 |
| ENSMUSG00000028648 | Ndufs5 | protein\_coding | 4:123712710-123718202 (-) |  | -0.3640 | 2.02e-07 | 2.62e-06 |
| ENSMUSG00000022708 | Zbtb20 | protein\_coding | 16:42875881-43642602 (+) |  | 0.8530 | 2.04e-07 | 2.64e-06 |
| ENSMUSG00000028907 | Utp11 | protein\_coding | 4:124678160-124693600 (-) |  | -0.3100 | 2.07e-07 | 2.68e-06 |
| ENSMUSG00000048234 | Rnf149 | protein\_coding | 1:39551296-39577405 (-) |  | -0.3580 | 2.09e-07 | 2.70e-06 |
| ENSMUSG00000040219 | Ttc12 | protein\_coding | 9:49436963-49486225 (-) |  | 0.9930 | 2.10e-07 | 2.71e-06 |
| ENSMUSG00000030287 | Itpr2 | protein\_coding | 6:146108299-146502223 (-) |  | 0.4860 | 2.11e-07 | 2.73e-06 |
| ENSMUSG00000046598 | Bdh1 | protein\_coding | 16:31422280-31458901 (+) |  | -1.7400 | 2.12e-07 | 2.74e-06 |
| ENSMUSG00000085181 | Gm12709 | lncRNA | 4:102967266-102989755 (-) |  | -1.9600 | 2.13e-07 | 2.74e-06 |
| ENSMUSG00000002910 | Arrdc2 | protein\_coding | 8:70835129-70839720 (-) |  | 0.4890 | 2.16e-07 | 2.79e-06 |
| ENSMUSG00000026385 | Dbi | protein\_coding | 1:120113280-120121078 (-) |  | -0.3550 | 2.18e-07 | 2.80e-06 |
| ENSMUSG00000030980 | Knop1 | protein\_coding | 7:118842222-118856254 (-) |  | -0.2860 | 2.19e-07 | 2.81e-06 |
| ENSMUSG00000062647 | Rpl7a | protein\_coding | 2:26910764-26913318 (+) |  | -0.2990 | 2.19e-07 | 2.81e-06 |
| ENSMUSG00000000085 | Scmh1 | protein\_coding | 4:120405281-120530186 (+) |  | 0.3830 | 2.20e-07 | 2.82e-06 |
| ENSMUSG00000033170 | Card10 | protein\_coding | 15:78775138-78803042 (-) |  | 2.4100 | 2.20e-07 | 2.82e-06 |
| ENSMUSG00000067194 | Eif1ax | protein\_coding | X:159372178-159389928 (+) |  | -0.3650 | 2.22e-07 | 2.85e-06 |
| ENSMUSG00000005378 | Bud23 | protein\_coding | 5:135052957-135064959 (-) |  | -0.2720 | 2.26e-07 | 2.89e-06 |
| ENSMUSG00000026365 | Cfh | protein\_coding | 1:140084708-140183764 (-) |  | -0.6460 | 2.27e-07 | 2.90e-06 |
| ENSMUSG00000022540 | Rogdi | protein\_coding | 16:5008730-5013553 (-) |  | 0.4970 | 2.29e-07 | 2.93e-06 |
| ENSMUSG00000031216 | Stard8 | protein\_coding | X:99003248-99074728 (+) |  | 0.4180 | 2.31e-07 | 2.94e-06 |
| ENSMUSG00000020929 | Eftud2 | protein\_coding | 11:102838473-102880985 (-) |  | -0.2920 | 2.31e-07 | 2.95e-06 |
| ENSMUSG00000032883 | Acsl3 | protein\_coding | 1:78657825-78707743 (+) |  | -0.4720 | 2.32e-07 | 2.95e-06 |
| ENSMUSG00000032459 | Mrps22 | protein\_coding | 9:98588730-98601660 (-) |  | -0.3970 | 2.32e-07 | 2.96e-06 |
| ENSMUSG00000030747 | Dgat2 | protein\_coding | 7:99153658-99182719 (-) |  | -0.5990 | 2.34e-07 | 2.98e-06 |
| ENSMUSG00000031751 | Amfr | protein\_coding | 8:93971588-94012842 (-) |  | 0.1880 | 2.35e-07 | 2.99e-06 |
| ENSMUSG00000026083 | Eif5b | protein\_coding | 1:37998010-38055579 (+) |  | -0.2740 | 2.37e-07 | 3.01e-06 |
| ENSMUSG00000041362 | Shtn1 | protein\_coding | 19:58973356-59076100 (-) |  | -0.9550 | 2.38e-07 | 3.01e-06 |
| ENSMUSG00000022946 | Dop1b | protein\_coding | 16:93711904-93810590 (+) |  | 0.4320 | 2.40e-07 | 3.04e-06 |
| ENSMUSG00000027763 | Mbnl1 | protein\_coding | 3:60472830-60629750 (+) |  | 0.2370 | 2.43e-07 | 3.08e-06 |
| ENSMUSG00000075703 | Selenoi | protein\_coding | 5:30232581-30272427 (+) |  | -0.2650 | 2.45e-07 | 3.10e-06 |
| ENSMUSG00000000028 | Cdc45 | protein\_coding | 16:18780447-18811987 (-) |  | -0.4040 | 2.46e-07 | 3.11e-06 |
| ENSMUSG00000034570 | Inpp5j | protein\_coding | 11:3494375-3504821 (-) |  | -0.6740 | 2.49e-07 | 3.14e-06 |
| ENSMUSG00000056076 | Eif3b | protein\_coding | 5:140419328-140443360 (+) |  | -0.3280 | 2.50e-07 | 3.15e-06 |
| ENSMUSG00000030830 | Itgal | protein\_coding | 7:127296260-127335138 (+) |  | 0.2250 | 2.50e-07 | 3.15e-06 |
| ENSMUSG00000038416 | Cdc16 | protein\_coding | 8:13757676-13781938 (+) |  | -0.2560 | 2.51e-07 | 3.16e-06 |
| ENSMUSG00000057895 | Zfp105 | protein\_coding | 9:122923072-122931028 (+) |  | 1.3900 | 2.52e-07 | 3.17e-06 |
| ENSMUSG00000028639 | Ybx1 | protein\_coding | 4:119277981-119294604 (-) |  | -0.2860 | 2.55e-07 | 3.21e-06 |
| ENSMUSG00000025794 | Rpl14 | protein\_coding | 9:120571444-120574654 (+) |  | -0.3020 | 2.60e-07 | 3.27e-06 |
| ENSMUSG00000074781 | Ube2n | protein\_coding | 10:95515145-95545657 (+) |  | -0.3950 | 2.61e-07 | 3.28e-06 |
| ENSMUSG00000026632 | Tatdn3 | protein\_coding | 1:191045826-191062932 (-) |  | 0.5990 | 2.64e-07 | 3.31e-06 |
| ENSMUSG00000003604 | Aven | protein\_coding | 2:112492964-112634573 (+) |  | -0.4350 | 2.66e-07 | 3.33e-06 |
| ENSMUSG00000030079 | Ruvbl1 | protein\_coding | 6:88465409-88497572 (+) |  | -0.3310 | 2.66e-07 | 3.33e-06 |
| ENSMUSG00000024424 | Ttc39c | protein\_coding | 18:12599896-12738863 (+) |  | -0.3290 | 2.66e-07 | 3.33e-06 |
| ENSMUSG00000005981 | Trap1 | protein\_coding | 16:4039971-4077827 (-) |  | -0.3070 | 2.74e-07 | 3.43e-06 |
| ENSMUSG00000025968 | Ndufs1 | protein\_coding | 1:63143596-63176833 (-) |  | -0.2670 | 2.74e-07 | 3.43e-06 |
| ENSMUSG00000010663 | Fads1 | protein\_coding | 19:10182888-10196877 (+) |  | -0.7300 | 2.76e-07 | 3.44e-06 |
| ENSMUSG00000074088 | Snrnp40 | protein\_coding | 4:130360132-130390026 (+) |  | -0.2030 | 2.76e-07 | 3.44e-06 |
| ENSMUSG00000066571 | 4931406P16Rik | protein\_coding | 7:34236707-34313551 (-) |  | 0.3360 | 2.77e-07 | 3.45e-06 |
| ENSMUSG00000036323 | Srp72 | protein\_coding | 5:76974683-76999937 (+) |  | -0.1850 | 2.79e-07 | 3.48e-06 |
| ENSMUSG00000066037 | Hnrnpr | protein\_coding | 4:136310942-136359447 (+) |  | -0.1930 | 2.82e-07 | 3.51e-06 |
| ENSMUSG00000051695 | Pcbp1 | protein\_coding | 6:86524492-86526321 (-) |  | -0.2380 | 2.91e-07 | 3.61e-06 |
| ENSMUSG00000073386 | 9830107B12Rik | protein\_coding | 17:48125605-48146268 (-) |  | 1.1900 | 2.99e-07 | 3.71e-06 |
| ENSMUSG00000033845 | Mrpl15 | protein\_coding | 1:4773206-4785739 (-) |  | -0.3560 | 3.02e-07 | 3.75e-06 |
| ENSMUSG00000067203 | H2-K2 | transcribed\_unprocessed\_pseudogene | 17:33974659-33978827 (-) |  | 0.9140 | 3.06e-07 | 3.79e-06 |
| ENSMUSG00000060989 | Gm11847 | processed\_pseudogene | 4:12232833-12233951 (+) |  | -0.5140 | 3.06e-07 | 3.79e-06 |
| ENSMUSG00000089847 | Timm10b | protein\_coding | 7:105640056-105643637 (+) |  | -0.3990 | 3.10e-07 | 3.83e-06 |
| ENSMUSG00000053318 | Slamf8 | protein\_coding | 1:172581758-172590568 (-) |  | 0.8580 | 3.13e-07 | 3.87e-06 |
| ENSMUSG00000075014 | Gm10800 | protein\_coding | 2:98666547-98667301 (-) |  | 4.4600 | 3.13e-07 | 3.87e-06 |
| ENSMUSG00000059479 | B3gnt8 | protein\_coding | 7:25626654-25635100 (+) |  | 0.4250 | 3.14e-07 | 3.87e-06 |
| ENSMUSG00000062908 | Acadm | protein\_coding | 3:153922357-153944632 (-) |  | 0.2330 | 3.16e-07 | 3.90e-06 |
| ENSMUSG00000022489 | Pde1b | protein\_coding | 15:103503034-103530052 (+) |  | 0.6060 | 3.18e-07 | 3.92e-06 |
| ENSMUSG00000022889 | Mrpl39 | protein\_coding | 16:84717576-84735742 (-) |  | -0.2960 | 3.23e-07 | 3.97e-06 |
| ENSMUSG00000062794 | Zfp599 | protein\_coding | 9:22247430-22259895 (-) |  | -0.9600 | 3.28e-07 | 4.03e-06 |
| ENSMUSG00000031304 | Il2rg | protein\_coding | X:101264378-101268255 (-) |  | -0.5270 | 3.31e-07 | 4.07e-06 |
| ENSMUSG00000023034 | Nr4a1 | protein\_coding | 15:101254269-101274795 (+) |  | 1.6300 | 3.33e-07 | 4.09e-06 |
| ENSMUSG00000030062 | Rpn1 | protein\_coding | 6:88084482-88105304 (+) |  | -0.3370 | 3.33e-07 | 4.09e-06 |
| ENSMUSG00000032842 | Abcc10 | protein\_coding | 17:46303221-46328352 (-) |  | 0.6370 | 3.41e-07 | 4.18e-06 |
| ENSMUSG00000040084 | Bub1b | protein\_coding | 2:118598211-118641591 (+) |  | -0.3210 | 3.47e-07 | 4.24e-06 |
| ENSMUSG00000109865 | Hspa14 | protein\_coding | 2:3488850-3512814 (-) |  | -0.2410 | 3.47e-07 | 4.25e-06 |
| ENSMUSG00000015846 | Rxra | protein\_coding | 2:27676440-27762957 (+) |  | 0.5000 | 3.52e-07 | 4.31e-06 |
| ENSMUSG00000044864 | Ankrd50 | protein\_coding | 3:38449259-38484844 (-) |  | 0.3230 | 3.53e-07 | 4.31e-06 |
| ENSMUSG00000001056 | Nhp2 | protein\_coding | 11:51619735-51623714 (+) |  | -0.3730 | 3.56e-07 | 4.35e-06 |
| ENSMUSG00000040624 | Plekhg1 | protein\_coding | 10:3740364-3967303 (+) |  | 0.5110 | 3.58e-07 | 4.36e-06 |
| ENSMUSG00000011257 | Pabpc4 | protein\_coding | 4:123262351-123298925 (+) |  | -0.4490 | 3.65e-07 | 4.45e-06 |
| ENSMUSG00000042675 | Ypel3 | protein\_coding | 7:126776955-126780514 (+) |  | 0.5750 | 3.69e-07 | 4.48e-06 |
| ENSMUSG00000014551 | Mrps25 | protein\_coding | 6:92169525-92184033 (-) |  | -0.3360 | 3.69e-07 | 4.48e-06 |
| ENSMUSG00000061981 | Flot2 | protein\_coding | 11:78037931-78060434 (+) |  | -0.2710 | 3.74e-07 | 4.54e-06 |
| ENSMUSG00000026234 | Ncl | protein\_coding | 1:86344719-86359400 (-) |  | -0.3890 | 3.74e-07 | 4.54e-06 |
| ENSMUSG00000025870 | Arl10 | protein\_coding | 13:54575015-54581128 (+) |  | 0.4320 | 3.75e-07 | 4.55e-06 |
| ENSMUSG00000100801 | Gm15459 | processed\_pseudogene | 5:5781615-5783555 (-) |  | -0.3890 | 3.75e-07 | 4.55e-06 |
| ENSMUSG00000045087 | S1pr5 | protein\_coding | 9:21242912-21248443 (-) |  | 2.2000 | 3.77e-07 | 4.56e-06 |
| ENSMUSG00000020577 | Tspan13 | protein\_coding | 12:36014557-36042500 (-) |  | -0.7220 | 3.78e-07 | 4.57e-06 |
| ENSMUSG00000019849 | Prep | protein\_coding | 10:45067203-45167198 (+) |  | -0.2970 | 3.78e-07 | 4.57e-06 |
| ENSMUSG00000026646 | Suv39h2 | protein\_coding | 2:3455815-3475031 (-) |  | -0.5220 | 3.80e-07 | 4.59e-06 |
| ENSMUSG00000078941 | Ak6 | protein\_coding | 13:100650979-100666359 (+) |  | -0.4510 | 3.84e-07 | 4.64e-06 |
| ENSMUSG00000020474 | Polm | protein\_coding | 11:5827860-5838016 (-) |  | 0.5580 | 3.86e-07 | 4.65e-06 |
| ENSMUSG00000037251 | Pomk | protein\_coding | 8:25980604-25994133 (-) |  | 0.5940 | 3.86e-07 | 4.65e-06 |
| ENSMUSG00000021432 | Slc35b3 | protein\_coding | 13:38932136-38960875 (-) |  | 0.3840 | 3.86e-07 | 4.65e-06 |
| ENSMUSG00000017548 | Suz12 | protein\_coding | 11:79993106-80034123 (+) |  | -0.2450 | 3.89e-07 | 4.68e-06 |
| ENSMUSG00000045868 | Gvin1 | protein\_coding | 7:106156556-106215326 (-) |  | -0.5960 | 3.90e-07 | 4.69e-06 |
| ENSMUSG00000004393 | Ddx56 | protein\_coding | 11:6258919-6267772 (-) |  | -0.3710 | 3.95e-07 | 4.74e-06 |
| ENSMUSG00000020664 | Dld | protein\_coding | 12:31331277-31351453 (-) |  | -0.2980 | 4.03e-07 | 4.84e-06 |
| ENSMUSG00000030697 | Ppp4c | protein\_coding | 7:126785866-126792496 (-) |  | -0.2670 | 4.13e-07 | 4.96e-06 |
| ENSMUSG00000032122 | Slc37a2 | protein\_coding | 9:37227585-37255738 (-) |  | -0.5200 | 4.15e-07 | 4.97e-06 |
| ENSMUSG00000022673 | Mcm4 | protein\_coding | 16:15623897-15637400 (-) |  | -0.4570 | 4.27e-07 | 5.11e-06 |
| ENSMUSG00000072763 | 5430403G16Rik | protein\_coding | 5:109674545-109691041 (-) |  | 0.9350 | 4.27e-07 | 5.11e-06 |
| ENSMUSG00000007891 | Ctsd | protein\_coding | 7:142375911-142388038 (-) |  | -0.3240 | 4.33e-07 | 5.18e-06 |
| ENSMUSG00000040928 | S100pbp | protein\_coding | 4:129148005-129189727 (-) |  | 0.3480 | 4.37e-07 | 5.22e-06 |
| ENSMUSG00000023025 | Larp4 | protein\_coding | 15:99970065-100016358 (+) |  | -0.2730 | 4.38e-07 | 5.22e-06 |
| ENSMUSG00000032381 | Ciao2a | protein\_coding | 9:66126611-66138955 (+) |  | -0.3100 | 4.39e-07 | 5.23e-06 |
| ENSMUSG00000017176 | Nt5c3b | protein\_coding | 11:100422321-100441808 (-) |  | -0.6120 | 4.46e-07 | 5.31e-06 |
| ENSMUSG00000035021 | Baz1a | protein\_coding | 12:54892989-55014348 (-) |  | -0.2690 | 4.48e-07 | 5.33e-06 |
| ENSMUSG00000004567 | Mcoln1 | protein\_coding | 8:3500457-3515232 (+) |  | 0.3990 | 4.49e-07 | 5.33e-06 |
| ENSMUSG00000059824 | Dbp | protein\_coding | 7:45705088-45710203 (+) |  | 1.0300 | 4.49e-07 | 5.33e-06 |
| ENSMUSG00000027642 | Rpn2 | protein\_coding | 2:157279017-157326319 (+) |  | -0.3560 | 4.52e-07 | 5.36e-06 |
| ENSMUSG00000029455 | Aldh2 | protein\_coding | 5:121566027-121593824 (-) |  | 0.2990 | 4.62e-07 | 5.48e-06 |
| ENSMUSG00000027544 | Nfatc2 | protein\_coding | 2:168476410-168601657 (-) |  | 0.6370 | 4.66e-07 | 5.52e-06 |
| ENSMUSG00000029135 | Fosl2 | protein\_coding | 5:32135801-32157842 (+) |  | 0.3910 | 4.67e-07 | 5.54e-06 |
| ENSMUSG00000030729 | Pgm2l1 | protein\_coding | 7:100227394-100278868 (+) |  | 0.3090 | 4.68e-07 | 5.54e-06 |
| ENSMUSG00000031791 | Tmem38a | protein\_coding | 8:72572055-72587282 (+) |  | 0.8560 | 4.70e-07 | 5.56e-06 |
| ENSMUSG00000037185 | Krt80 | protein\_coding | 15:101347444-101370162 (-) |  | 0.6240 | 4.71e-07 | 5.57e-06 |
| ENSMUSG00000024399 | Ltb | protein\_coding | 17:35194439-35196320 (+) |  | 1.5700 | 4.73e-07 | 5.59e-06 |
| ENSMUSG00000064366 | mt-Tl2 | Mt\_tRNA | MT:11671-11741 (+) |  | -1.0300 | 4.75e-07 | 5.61e-06 |
| ENSMUSG00000028799 | Zfp362 | protein\_coding | 4:128773088-128806045 (-) |  | 0.4120 | 4.77e-07 | 5.63e-06 |
| ENSMUSG00000015087 | Rabl6 | protein\_coding | 2:25583018-25608521 (-) |  | -0.1980 | 4.86e-07 | 5.72e-06 |
| ENSMUSG00000100514 | Gm12960 | processed\_pseudogene | 4:111807358-111808423 (-) |  | -0.4280 | 4.89e-07 | 5.75e-06 |
| ENSMUSG00000068921 | Dap3 | protein\_coding | 3:88920803-88951181 (-) |  | -0.2560 | 4.90e-07 | 5.76e-06 |
| ENSMUSG00000107383 | Gm4366 | processed\_pseudogene | 7:116824510-116825851 (-) |  | -0.4900 | 4.90e-07 | 5.76e-06 |
| ENSMUSG00000047604 | Frat2 | protein\_coding | 19:41845972-41848132 (-) |  | 0.6300 | 4.91e-07 | 5.76e-06 |
| ENSMUSG00000079056 | Kcnip3 | protein\_coding | 2:127456498-127522094 (-) |  | 0.9880 | 4.92e-07 | 5.77e-06 |
| ENSMUSG00000020527 | Myo19 | protein\_coding | 11:84880148-84911226 (+) |  | -0.5570 | 4.95e-07 | 5.81e-06 |
| ENSMUSG00000022438 | Parvb | protein\_coding | 15:84232043-84315688 (+) |  | 0.4430 | 4.96e-07 | 5.81e-06 |
| ENSMUSG00000022742 | Cpox | protein\_coding | 16:58670292-58717636 (+) |  | -0.2870 | 4.96e-07 | 5.81e-06 |
| ENSMUSG00000039842 | Mcph1 | protein\_coding | 8:18595131-18803189 (+) |  | -0.3390 | 5.03e-07 | 5.88e-06 |
| ENSMUSG00000018189 | Uchl5 | protein\_coding | 1:143777272-143807466 (+) |  | -0.3530 | 5.06e-07 | 5.91e-06 |
| ENSMUSG00000002985 | Apoe | protein\_coding | 7:19696109-19699188 (-) |  | 1.6300 | 5.09e-07 | 5.94e-06 |
| ENSMUSG00000055633 | Zfp580 | protein\_coding | 7:5051538-5053723 (+) |  | 1.2200 | 5.17e-07 | 6.03e-06 |
| ENSMUSG00000043702 | Pde12 | protein\_coding | 14:26659958-26669883 (-) |  | -0.2450 | 5.19e-07 | 6.04e-06 |
| ENSMUSG00000020457 | Drg1 | protein\_coding | 11:3187360-3266415 (-) |  | -0.2560 | 5.23e-07 | 6.09e-06 |
| ENSMUSG00000006476 | Nsmf | protein\_coding | 2:25054355-25062881 (+) |  | 0.4520 | 5.30e-07 | 6.17e-06 |
| ENSMUSG00000035351 | Nup37 | protein\_coding | 10:88146992-88178390 (+) |  | -0.4240 | 5.33e-07 | 6.20e-06 |
| ENSMUSG00000070305 | Mpzl3 | protein\_coding | 9:45055186-45077436 (+) |  | -0.8060 | 5.37e-07 | 6.24e-06 |
| ENSMUSG00000013629 | Cad | protein\_coding | 5:31054780-31078479 (+) |  | -0.5780 | 5.37e-07 | 6.24e-06 |
| ENSMUSG00000062397 | Zfp706 | protein\_coding | 15:36997027-37007773 (-) |  | -0.2050 | 5.40e-07 | 6.26e-06 |
| ENSMUSG00000046668 | Cxxc5 | protein\_coding | 18:35829397-35861688 (+) |  | 0.9940 | 5.41e-07 | 6.26e-06 |
| ENSMUSG00000031605 | Klhl2 | protein\_coding | 8:64739675-64850017 (-) |  | -0.3740 | 5.43e-07 | 6.28e-06 |
| ENSMUSG00000072809 | 9330160F10Rik | lncRNA | 11:69057718-69060483 (-) |  | 0.8090 | 5.48e-07 | 6.34e-06 |
| ENSMUSG00000021996 | Esd | protein\_coding | 14:74732297-74750765 (+) |  | -0.3210 | 5.52e-07 | 6.38e-06 |
| ENSMUSG00000011267 | Zfp296 | protein\_coding | 7:19577287-19580656 (+) |  | 0.6540 | 5.52e-07 | 6.38e-06 |
| ENSMUSG00000064360 | mt-Nd3 | protein\_coding | MT:9459-9806 (+) |  | -0.6410 | 5.56e-07 | 6.42e-06 |
| ENSMUSG00000031984 | 2810004N23Rik | protein\_coding | 8:124836945-124863120 (-) |  | -0.2760 | 5.57e-07 | 6.42e-06 |
| ENSMUSG00000029203 | Ube2k | protein\_coding | 5:65537233-65598988 (+) |  | -0.1890 | 5.60e-07 | 6.45e-06 |
| ENSMUSG00000025393 | Atp5b | protein\_coding | 10:128083273-128090391 (+) |  | -0.3380 | 5.61e-07 | 6.46e-06 |
| ENSMUSG00000035227 | Spcs2 | protein\_coding | 7:99837569-99863462 (-) |  | -0.2700 | 5.62e-07 | 6.46e-06 |
| ENSMUSG00000032437 | Stt3b | protein\_coding | 9:115242581-115310421 (-) |  | -0.2460 | 5.67e-07 | 6.52e-06 |
| ENSMUSG00000039157 | Fam102a | protein\_coding | 2:32535332-32569756 (+) |  | 0.6760 | 5.67e-07 | 6.52e-06 |
| ENSMUSG00000022818 | Cyp2ab1 | protein\_coding | 16:20308387-20325404 (-) |  | 1.1600 | 5.69e-07 | 6.53e-06 |
| ENSMUSG00000041035 | Gm17018 | protein\_coding | 19:45560615-45579763 (+) |  | -0.3310 | 5.75e-07 | 6.59e-06 |
| ENSMUSG00000058392 | Rrp1b | protein\_coding | 17:32036100-32062865 (+) |  | -0.3500 | 5.76e-07 | 6.60e-06 |
| ENSMUSG00000014077 | Chp1 | protein\_coding | 2:119547697-119587027 (+) |  | -0.2110 | 5.83e-07 | 6.67e-06 |
| ENSMUSG00000028010 | Gar1 | protein\_coding | 3:129824912-129831396 (-) |  | -0.4010 | 5.85e-07 | 6.69e-06 |
| ENSMUSG00000029202 | Pds5a | protein\_coding | 5:65605721-65698273 (-) |  | -0.2400 | 5.85e-07 | 6.69e-06 |
| ENSMUSG00000039585 | Myo9a | protein\_coding | 9:59750896-59928866 (+) |  | 0.3800 | 5.86e-07 | 6.69e-06 |
| ENSMUSG00000034765 | Dusp5 | protein\_coding | 19:53529109-53542431 (+) |  | 1.2000 | 5.90e-07 | 6.74e-06 |
| ENSMUSG00000030695 | Aldoa | protein\_coding | 7:126795234-126800751 (-) |  | -0.3340 | 5.97e-07 | 6.81e-06 |
| ENSMUSG00000022814 | Umps | protein\_coding | 16:33954782-33967038 (-) |  | -0.2670 | 6.02e-07 | 6.86e-06 |
| ENSMUSG00000029467 | Atp2a2 | protein\_coding | 5:122453513-122502225 (-) |  | -0.2680 | 6.03e-07 | 6.86e-06 |
| ENSMUSG00000020522 | Mfap3 | protein\_coding | 11:57518664-57533815 (+) |  | 0.2590 | 6.03e-07 | 6.86e-06 |
| ENSMUSG00000020346 | Mgat1 | protein\_coding | 11:49244191-49263030 (+) |  | 0.2460 | 6.12e-07 | 6.96e-06 |
| ENSMUSG00000028630 | Dyrk2 | protein\_coding | 10:118855603-118870209 (-) |  | 0.3760 | 6.16e-07 | 7.00e-06 |
| ENSMUSG00000061024 | Rrs1 | protein\_coding | 1:9545408-9547455 (+) |  | -0.2710 | 6.19e-07 | 7.03e-06 |
| ENSMUSG00000035835 | Plppr3 | protein\_coding | 10:79860475-79874634 (-) |  | -0.5900 | 6.20e-07 | 7.03e-06 |
| ENSMUSG00000006998 | Psmd2 | protein\_coding | 16:20651652-20663414 (+) |  | -0.2850 | 6.22e-07 | 7.05e-06 |
| ENSMUSG00000064068 | Mtx1 | protein\_coding | 3:89209081-89227088 (-) |  | -0.2620 | 6.23e-07 | 7.06e-06 |
| ENSMUSG00000056116 | H2-T22 | protein\_coding | 17:36037128-36042747 (-) |  | -0.3270 | 6.23e-07 | 7.06e-06 |
| ENSMUSG00000029311 | Hsd17b11 | protein\_coding | 5:103989762-104021919 (-) |  | 0.2300 | 6.24e-07 | 7.06e-06 |
| ENSMUSG00000040204 | Pclaf | protein\_coding | 9:65890237-65903266 (+) |  | -0.3980 | 6.27e-07 | 7.08e-06 |
| ENSMUSG00000029036 | Atad3a | protein\_coding | 4:155740641-155761093 (-) |  | -0.3720 | 6.32e-07 | 7.14e-06 |
| ENSMUSG00000073131 | Vma21 | protein\_coding | X:71815924-71839757 (+) |  | -0.3050 | 6.34e-07 | 7.16e-06 |
| ENSMUSG00000019944 | Rhobtb1 | protein\_coding | 10:69151434-69291791 (+) |  | 1.3700 | 6.38e-07 | 7.20e-06 |
| ENSMUSG00000024493 | Lars | protein\_coding | 18:42202298-42262194 (-) |  | -0.2770 | 6.40e-07 | 7.20e-06 |
| ENSMUSG00000040667 | Nup88 | protein\_coding | 11:70943058-70969973 (-) |  | -0.2040 | 6.40e-07 | 7.20e-06 |
| ENSMUSG00000020974 | Pole2 | protein\_coding | 12:69201773-69228195 (-) |  | -0.3730 | 6.52e-07 | 7.34e-06 |
| ENSMUSG00000071866 | Ppia | protein\_coding | 11:6415443-6419817 (+) |  | -0.4000 | 6.58e-07 | 7.39e-06 |
| ENSMUSG00000032855 | Pkd1 | protein\_coding | 17:24549834-24596508 (+) |  | 0.3600 | 6.63e-07 | 7.45e-06 |
| ENSMUSG00000028107 | Tars2 | protein\_coding | 3:95739976-95760206 (-) |  | -0.3130 | 6.68e-07 | 7.49e-06 |
| ENSMUSG00000038683 | Pak1ip1 | protein\_coding | 13:41001023-41013015 (+) |  | -0.2400 | 6.70e-07 | 7.51e-06 |
| ENSMUSG00000022102 | Dok2 | protein\_coding | 14:70766036-70778495 (+) |  | -0.6370 | 6.73e-07 | 7.54e-06 |
| ENSMUSG00000049191 | Rtl5 | protein\_coding | X:102066544-102071304 (-) |  | 0.6950 | 6.79e-07 | 7.60e-06 |
| ENSMUSG00000028438 | Kif24 | protein\_coding | 4:41390745-41464887 (-) |  | -0.3840 | 6.81e-07 | 7.62e-06 |
| ENSMUSG00000028741 | Mrto4 | protein\_coding | 4:139347435-139352576 (-) |  | -0.3890 | 6.84e-07 | 7.65e-06 |
| ENSMUSG00000019132 | BC005537 | protein\_coding | 13:24801657-24816197 (+) |  | -0.2080 | 6.86e-07 | 7.66e-06 |
| ENSMUSG00000031095 | Cul4b | protein\_coding | X:38533274-38576196 (-) |  | -0.2480 | 6.87e-07 | 7.67e-06 |
| ENSMUSG00000025534 | Gusb | protein\_coding | 5:129989011-130003049 (-) |  | -0.1910 | 6.89e-07 | 7.68e-06 |
| ENSMUSG00000032563 | Mrpl3 | protein\_coding | 9:105053239-105079888 (+) |  | -0.3540 | 6.89e-07 | 7.68e-06 |
| ENSMUSG00000038295 | Atg9b | protein\_coding | 5:24384181-24392143 (-) |  | -0.7260 | 6.93e-07 | 7.71e-06 |
| ENSMUSG00000024037 | Wdr4 | protein\_coding | 17:31494322-31519980 (-) |  | -0.3600 | 6.94e-07 | 7.72e-06 |
| ENSMUSG00000017264 | Exosc10 | protein\_coding | 4:148558429-148582401 (+) |  | -0.2280 | 6.98e-07 | 7.76e-06 |
| ENSMUSG00000025130 | P4hb | protein\_coding | 11:120560298-120573253 (-) |  | -0.2950 | 7.02e-07 | 7.80e-06 |
| ENSMUSG00000028654 | Mycl | protein\_coding | 4:122995652-123002485 (+) |  | 1.3100 | 7.06e-07 | 7.83e-06 |
| ENSMUSG00000048779 | P2ry6 | protein\_coding | 7:100937630-100974649 (-) |  | 0.6380 | 7.09e-07 | 7.87e-06 |
| ENSMUSG00000015053 | Gata2 | protein\_coding | 6:88193891-88207032 (+) |  | 1.5200 | 7.17e-07 | 7.95e-06 |
| ENSMUSG00000003813 | Rad23a | protein\_coding | 8:84834019-84840665 (-) |  | -0.2860 | 7.24e-07 | 8.02e-06 |
| ENSMUSG00000079429 | Mroh2a | protein\_coding | 1:88226986-88262289 (+) |  | -0.4070 | 7.25e-07 | 8.02e-06 |
| ENSMUSG00000029432 | Nipsnap2 | protein\_coding | 5:129725063-129758327 (+) |  | 0.2880 | 7.26e-07 | 8.03e-06 |
| ENSMUSG00000043252 | Tmem64 | protein\_coding | 4:15265831-15286753 (+) |  | 0.4810 | 7.33e-07 | 8.09e-06 |
| ENSMUSG00000037966 | Ninj1 | protein\_coding | 13:49187485-49196244 (+) |  | 0.4590 | 7.33e-07 | 8.09e-06 |
| ENSMUSG00000061479 | Snrpa | protein\_coding | 7:27187005-27196271 (-) |  | -0.3360 | 7.39e-07 | 8.15e-06 |
| ENSMUSG00000001424 | Snd1 | protein\_coding | 6:28475139-28935162 (+) |  | -0.2850 | 7.39e-07 | 8.15e-06 |
| ENSMUSG00000006360 | Crip1 | protein\_coding | 12:113146316-113153879 (+) |  | 0.5060 | 7.41e-07 | 8.16e-06 |
| ENSMUSG00000056737 | Capg | protein\_coding | 6:72544391-72562983 (+) |  | 0.4400 | 7.50e-07 | 8.26e-06 |
| ENSMUSG00000018750 | Zbtb4 | protein\_coding | 11:69765912-69784023 (+) |  | 0.9280 | 7.52e-07 | 8.28e-06 |
| ENSMUSG00000052821 | Cysltr1 | protein\_coding | X:106574346-106603679 (-) |  | -0.5990 | 7.56e-07 | 8.31e-06 |
| ENSMUSG00000003581 | Rnf215 | protein\_coding | 11:4135202-4141172 (+) |  | 0.3790 | 7.57e-07 | 8.31e-06 |
| ENSMUSG00000019699 | Akt3 | protein\_coding | 1:177020073-177258203 (-) |  | 0.3150 | 7.58e-07 | 8.32e-06 |
| ENSMUSG00000030603 | Psmc4 | protein\_coding | 7:28041707-28050101 (-) |  | -0.2870 | 7.59e-07 | 8.32e-06 |
| ENSMUSG00000043257 | Pigv | protein\_coding | 4:133660387-133672647 (-) |  | 0.3740 | 7.67e-07 | 8.41e-06 |
| ENSMUSG00000025962 | Fastkd2 | protein\_coding | 1:63730614-63754655 (+) |  | -0.4310 | 7.75e-07 | 8.49e-06 |
| ENSMUSG00000031749 | St3gal2 | protein\_coding | 8:110919922-110972480 (+) |  | 0.4640 | 7.79e-07 | 8.52e-06 |
| ENSMUSG00000031467 | Agpat5 | protein\_coding | 8:18846277-18891361 (+) |  | -0.2950 | 7.79e-07 | 8.52e-06 |
| ENSMUSG00000038507 | Parp12 | protein\_coding | 6:39086410-39118349 (-) |  | -0.6030 | 7.83e-07 | 8.55e-06 |
| ENSMUSG00000042331 | Specc1 | protein\_coding | 11:61956763-62223013 (+) |  | 0.2770 | 7.86e-07 | 8.58e-06 |
| ENSMUSG00000064193 | Gm4735 | processed\_pseudogene | 2:80837141-80838442 (+) |  | -0.4440 | 7.88e-07 | 8.59e-06 |
| ENSMUSG00000000568 | Hnrnpd | protein\_coding | 5:99955935-99978938 (-) |  | -0.2410 | 7.92e-07 | 8.63e-06 |
| ENSMUSG00000031060 | Rbm10 | protein\_coding | X:20617503-20650901 (+) |  | -0.2200 | 7.95e-07 | 8.66e-06 |
| ENSMUSG00000069892 | 9930111J21Rik2 | protein\_coding | 11:49015874-49051242 (-) |  | 0.4120 | 8.05e-07 | 8.76e-06 |
| ENSMUSG00000021963 | Sap18 | protein\_coding | 14:57798180-57806638 (+) |  | -0.2310 | 8.12e-07 | 8.84e-06 |
| ENSMUSG00000027185 | Nat10 | protein\_coding | 2:103721256-103761270 (-) |  | -0.3970 | 8.21e-07 | 8.92e-06 |
| ENSMUSG00000060600 | Eno3 | protein\_coding | 11:70657202-70662513 (+) |  | 0.7570 | 8.28e-07 | 8.99e-06 |
| ENSMUSG00000038387 | Rras | protein\_coding | 7:45017961-45021647 (+) |  | 0.6560 | 8.28e-07 | 8.99e-06 |
| ENSMUSG00000039356 | Exosc2 | protein\_coding | 2:31670715-31681349 (+) |  | -0.3490 | 8.38e-07 | 9.08e-06 |
| ENSMUSG00000038179 | Slamf7 | protein\_coding | 1:171632403-171653035 (-) |  | 1.2700 | 8.42e-07 | 9.13e-06 |
| ENSMUSG00000069793 | Slfn9 | protein\_coding | 11:82978390-82991830 (-) |  | -0.3460 | 8.46e-07 | 9.16e-06 |
| ENSMUSG00000028756 | Pink1 | protein\_coding | 4:138313409-138326307 (-) |  | 0.5010 | 8.47e-07 | 9.16e-06 |
| ENSMUSG00000032067 | Pts | protein\_coding | 9:50521617-50528724 (-) |  | 0.3380 | 8.53e-07 | 9.22e-06 |
| ENSMUSG00000054364 | Rhob | protein\_coding | 12:8497661-8500009 (-) |  | 0.5810 | 8.55e-07 | 9.24e-06 |
| ENSMUSG00000035726 | Supt16 | protein\_coding | 14:52160414-52197416 (-) |  | -0.2310 | 8.57e-07 | 9.25e-06 |
| ENSMUSG00000033751 | Gadd45gip1 | protein\_coding | 8:84831522-84835482 (+) |  | -0.4690 | 8.58e-07 | 9.26e-06 |
| ENSMUSG00000042594 | Sh2b3 | protein\_coding | 5:121815488-121837646 (-) |  | 0.2780 | 8.60e-07 | 9.27e-06 |
| ENSMUSG00000022360 | Atad2 | protein\_coding | 15:58094044-58135082 (-) |  | -0.3690 | 8.65e-07 | 9.32e-06 |
| ENSMUSG00000034880 | Mrpl34 | protein\_coding | 8:71464959-71465747 (+) |  | -0.3330 | 8.72e-07 | 9.38e-06 |
| ENSMUSG00000041754 | Trem3 | protein\_coding | 17:48247777-48258841 (+) |  | -0.4760 | 8.76e-07 | 9.42e-06 |
| ENSMUSG00000031508 | Ankrd10 | protein\_coding | 8:11611583-11635757 (-) |  | 0.2120 | 8.79e-07 | 9.44e-06 |
| ENSMUSG00000034211 | Mrps17 | protein\_coding | 5:129715497-129722556 (+) |  | -0.2460 | 8.79e-07 | 9.44e-06 |
| ENSMUSG00000034266 | Batf | protein\_coding | 12:85686669-85709087 (+) |  | -0.4480 | 8.82e-07 | 9.46e-06 |
| ENSMUSG00000028760 | Eif4g3 | protein\_coding | 4:137993022-138208508 (+) |  | 0.2820 | 8.92e-07 | 9.56e-06 |
| ENSMUSG00000040247 | Tbc1d10c | protein\_coding | 19:4183411-4191284 (-) |  | 0.9450 | 8.93e-07 | 9.57e-06 |
| ENSMUSG00000031828 | Klhl36 | protein\_coding | 8:119862266-119876995 (+) |  | 0.3380 | 9.06e-07 | 9.70e-06 |
| ENSMUSG00000028385 | Snx30 | protein\_coding | 4:59805840-59904737 (+) |  | 0.3300 | 9.16e-07 | 9.80e-06 |
| ENSMUSG00000028961 | Pgd | protein\_coding | 4:149149991-149166771 (-) |  | -0.2170 | 9.17e-07 | 9.81e-06 |
| ENSMUSG00000030738 | Eif3c | protein\_coding | 7:126546455-126566411 (-) |  | -0.2460 | 9.35e-07 | 9.98e-06 |
| ENSMUSG00000026315 | Serpinb8 | protein\_coding | 1:107590006-107610484 (+) |  | -1.3300 | 9.40e-07 | 1.00e-05 |
| ENSMUSG00000035245 | Eogt | protein\_coding | 6:97110024-97149182 (-) |  | -0.5750 | 9.49e-07 | 1.01e-05 |
| ENSMUSG00000028426 | Rad23b | protein\_coding | 4:55350043-55392237 (+) |  | -0.2420 | 9.57e-07 | 1.02e-05 |
| ENSMUSG00000010142 | Tnfrsf13b | protein\_coding | 11:61126755-61149372 (+) |  | 0.2790 | 9.59e-07 | 1.02e-05 |
| ENSMUSG00000050410 | Tcf19 | protein\_coding | 17:35512734-35516824 (-) |  | -0.4400 | 9.62e-07 | 1.02e-05 |
| ENSMUSG00000040345 | Arhgap9 | protein\_coding | 10:127321964-127329943 (+) |  | 0.2870 | 9.63e-07 | 1.02e-05 |
| ENSMUSG00000039753 | Fbxl5 | protein\_coding | 5:43744615-43821638 (-) |  | -0.5360 | 9.68e-07 | 1.03e-05 |
| ENSMUSG00000107215 | Gm43197 | TEC | 6:3336772-3339354 (-) |  | 0.8940 | 9.76e-07 | 1.04e-05 |
| ENSMUSG00000020116 | Pno1 | protein\_coding | 11:17203198-17211568 (-) |  | -0.4940 | 9.84e-07 | 1.04e-05 |
| ENSMUSG00000041355 | Ssr2 | protein\_coding | 3:88575876-88588419 (+) |  | -0.3050 | 9.94e-07 | 1.05e-05 |
| ENSMUSG00000032397 | Tipin | protein\_coding | 9:64281581-64305424 (+) |  | -0.3350 | 9.97e-07 | 1.06e-05 |
| ENSMUSG00000060373 | Hnrnpc | protein\_coding | 14:52073377-52104028 (-) |  | -0.2920 | 9.97e-07 | 1.06e-05 |
| ENSMUSG00000058799 | Nap1l1 | protein\_coding | 10:111473223-111498150 (+) |  | -0.2110 | 1.00e-06 | 1.06e-05 |
| ENSMUSG00000028977 | Casz1 | protein\_coding | 4:148804429-148954889 (+) |  | 0.3850 | 1.01e-06 | 1.07e-05 |
| ENSMUSG00000064120 | Mocs1 | protein\_coding | 17:49428362-49455435 (+) |  | 0.2190 | 1.01e-06 | 1.07e-05 |
| ENSMUSG00000030541 | Idh2 | protein\_coding | 7:80094846-80115392 (-) |  | -0.3220 | 1.02e-06 | 1.08e-05 |
| ENSMUSG00000024613 | Tcof1 | protein\_coding | 18:60813755-60848971 (-) |  | -0.3190 | 1.03e-06 | 1.08e-05 |
| ENSMUSG00000053801 | Grwd1 | protein\_coding | 7:45825223-45830944 (-) |  | -0.5030 | 1.03e-06 | 1.08e-05 |
| ENSMUSG00000040463 | Mybbp1a | protein\_coding | 11:72441355-72451768 (+) |  | -0.4130 | 1.04e-06 | 1.09e-05 |
| ENSMUSG00000022295 | Atp6v1c1 | protein\_coding | 15:38661933-38692446 (+) |  | -0.2860 | 1.05e-06 | 1.10e-05 |
| ENSMUSG00000024640 | Psat1 | protein\_coding | 19:15904678-15947337 (-) |  | -0.3000 | 1.05e-06 | 1.10e-05 |
| ENSMUSG00000030718 | Ppme1 | protein\_coding | 7:100326737-100372307 (-) |  | -0.2590 | 1.05e-06 | 1.10e-05 |
| ENSMUSG00000026637 | Traf5 | protein\_coding | 1:191997205-192092559 (-) |  | 0.4320 | 1.05e-06 | 1.10e-05 |
| ENSMUSG00000060301 | 2610008E11Rik | protein\_coding | 10:79064374-79097600 (-) |  | 0.5330 | 1.05e-06 | 1.11e-05 |
| ENSMUSG00000015869 | Prpsap1 | protein\_coding | 11:116470845-116494202 (-) |  | -0.2500 | 1.05e-06 | 1.11e-05 |
| ENSMUSG00000034120 | Srsf2 | protein\_coding | 11:116849901-116853094 (-) |  | -0.1880 | 1.05e-06 | 1.11e-05 |
| ENSMUSG00000021998 | Lcp1 | protein\_coding | 14:75131101-75230842 (+) |  | -0.2410 | 1.06e-06 | 1.11e-05 |
| ENSMUSG00000035329 | Fbxo33 | protein\_coding | 12:59200655-59219725 (-) |  | -0.3510 | 1.06e-06 | 1.11e-05 |
| ENSMUSG00000061613 | U2af1 | protein\_coding | 17:31647081-31658892 (-) |  | -0.2920 | 1.08e-06 | 1.13e-05 |
| ENSMUSG00000057315 | Arhgap24 | protein\_coding | 5:102481391-102897937 (+) |  | -0.4650 | 1.09e-06 | 1.13e-05 |
| ENSMUSG00000032423 | Syncrip | protein\_coding | 9:88447009-88482574 (-) |  | -0.2680 | 1.09e-06 | 1.14e-05 |
| ENSMUSG00000030602 | Pak4 | protein\_coding | 7:28558819-28598185 (-) |  | 0.3570 | 1.09e-06 | 1.14e-05 |
| ENSMUSG00000022451 | Twf1 | protein\_coding | 15:94577951-94589889 (-) |  | -0.3860 | 1.11e-06 | 1.16e-05 |
| ENSMUSG00000001120 | Pcbp3 | protein\_coding | 10:76761857-76961887 (-) |  | 0.7200 | 1.12e-06 | 1.16e-05 |
| ENSMUSG00000042105 | Inpp5f | protein\_coding | 7:128611328-128696425 (+) |  | 0.3440 | 1.12e-06 | 1.17e-05 |
| ENSMUSG00000015501 | Hivep2 | protein\_coding | 10:13966075-14151374 (+) |  | -0.8040 | 1.13e-06 | 1.17e-05 |
| ENSMUSG00000030978 | Rrm1 | protein\_coding | 7:102441695-102469771 (+) |  | -0.3530 | 1.14e-06 | 1.19e-05 |
| ENSMUSG00000032116 | Stt3a | protein\_coding | 9:36729344-36767679 (-) |  | -0.2290 | 1.14e-06 | 1.19e-05 |
| ENSMUSG00000029610 | Aimp2 | protein\_coding | 5:143902704-143909847 (-) |  | -0.3890 | 1.15e-06 | 1.20e-05 |
| ENSMUSG00000028613 | Lrp8 | protein\_coding | 4:107801869-107876840 (+) |  | -0.4920 | 1.15e-06 | 1.20e-05 |
| ENSMUSG00000021952 | Xpo4 | protein\_coding | 14:57577521-57665430 (-) |  | -0.2940 | 1.16e-06 | 1.20e-05 |
| ENSMUSG00000003438 | Timm50 | protein\_coding | 7:28305516-28312072 (-) |  | -0.2670 | 1.16e-06 | 1.20e-05 |
| ENSMUSG00000028322 | Exosc3 | protein\_coding | 4:45316613-45342732 (-) |  | -0.2900 | 1.16e-06 | 1.20e-05 |
| ENSMUSG00000024740 | Ddb1 | protein\_coding | 19:10605327-10629819 (+) |  | -0.3100 | 1.16e-06 | 1.20e-05 |
| ENSMUSG00000023044 | Csad | protein\_coding | 15:102176999-102204724 (-) |  | 0.4050 | 1.16e-06 | 1.20e-05 |
| ENSMUSG00000022214 | Dcaf11 | protein\_coding | 14:55560006-55570065 (+) |  | 0.2510 | 1.18e-06 | 1.22e-05 |
| ENSMUSG00000021993 | Mipep | protein\_coding | 14:60784573-60905478 (+) |  | -0.4220 | 1.18e-06 | 1.22e-05 |
| ENSMUSG00000026932 | Nacc2 | protein\_coding | 2:26055535-26123220 (-) |  | 0.5810 | 1.19e-06 | 1.22e-05 |
| ENSMUSG00000024735 | Prpf19 | protein\_coding | 19:10895231-10909559 (+) |  | -0.2730 | 1.19e-06 | 1.23e-05 |
| ENSMUSG00000029672 | Fam3c | protein\_coding | 6:22306520-22356243 (-) |  | -0.2950 | 1.20e-06 | 1.23e-05 |
| ENSMUSG00000013236 | Ptprs | protein\_coding | 17:56412426-56476483 (-) |  | 0.4150 | 1.20e-06 | 1.24e-05 |
| ENSMUSG00000002944 | Cd36 | protein\_coding | 5:17781690-17888801 (-) |  | 2.8500 | 1.21e-06 | 1.25e-05 |
| ENSMUSG00000057236 | Rbbp4 | protein\_coding | 4:129307100-129335370 (-) |  | -0.2050 | 1.21e-06 | 1.25e-05 |
| ENSMUSG00000018882 | Mrpl45 | protein\_coding | 11:97315716-97329920 (+) |  | -0.2960 | 1.22e-06 | 1.25e-05 |
| ENSMUSG00000027349 | Fam98b | protein\_coding | 2:117249739-117271540 (+) |  | -0.2870 | 1.22e-06 | 1.25e-05 |
| ENSMUSG00000033033 | Calhm2 | protein\_coding | 19:47105353-47138294 (-) |  | 0.3060 | 1.23e-06 | 1.26e-05 |
| ENSMUSG00000106847 | Peg13 | lncRNA | 15:72805600-72810324 (-) |  | 0.6750 | 1.23e-06 | 1.26e-05 |
| ENSMUSG00000038780 | Smurf1 | protein\_coding | 5:144876495-144965847 (-) |  | 0.3790 | 1.23e-06 | 1.26e-05 |
| ENSMUSG00000025403 | Shmt2 | protein\_coding | 10:127517123-127522444 (-) |  | -0.5030 | 1.26e-06 | 1.29e-05 |
| ENSMUSG00000035783 | Acta2 | protein\_coding | 19:34241090-34255590 (-) |  | -0.7270 | 1.27e-06 | 1.30e-05 |
| ENSMUSG00000028467 | Gba2 | protein\_coding | 4:43566928-43578873 (-) |  | 0.2810 | 1.27e-06 | 1.30e-05 |
| ENSMUSG00000052299 | Ltn1 | protein\_coding | 16:87376651-87432612 (-) |  | -0.2840 | 1.29e-06 | 1.32e-05 |
| ENSMUSG00000024681 | Ms4a3 | protein\_coding | 19:11629496-11640851 (-) |  | -1.2600 | 1.29e-06 | 1.32e-05 |
| ENSMUSG00000024999 | Noc3l | protein\_coding | 19:38788128-38819237 (-) |  | -0.3280 | 1.29e-06 | 1.32e-05 |
| ENSMUSG00000000168 | Dlat | protein\_coding | 9:50634633-50659780 (-) |  | -0.3100 | 1.30e-06 | 1.33e-05 |
| ENSMUSG00000029528 | Pxn | protein\_coding | 5:115506676-115555987 (+) |  | 0.1960 | 1.31e-06 | 1.33e-05 |
| ENSMUSG00000029780 | Nt5c3 | protein\_coding | 6:56882400-56923932 (-) |  | -0.3930 | 1.31e-06 | 1.33e-05 |
| ENSMUSG00000027828 | Ssr3 | protein\_coding | 3:65379655-65392623 (-) |  | -0.2050 | 1.37e-06 | 1.40e-05 |
| ENSMUSG00000020224 | Llph | protein\_coding | 10:120227070-120232582 (+) |  | -0.3930 | 1.38e-06 | 1.40e-05 |
| ENSMUSG00000022018 | Rgcc | protein\_coding | 14:79288756-79301645 (-) |  | -0.9540 | 1.38e-06 | 1.40e-05 |
| ENSMUSG00000029994 | Anxa4 | protein\_coding | 6:86736840-86793584 (-) |  | 0.3430 | 1.38e-06 | 1.41e-05 |
| ENSMUSG00000026482 | Rgl1 | protein\_coding | 1:152516760-152766351 (-) |  | 1.2000 | 1.39e-06 | 1.41e-05 |
| ENSMUSG00000046207 | Pik3r6 | protein\_coding | 11:68503019-68552698 (+) |  | 0.2960 | 1.41e-06 | 1.42e-05 |
| ENSMUSG00000001525 | Tubb5 | protein\_coding | 17:35833921-35838306 (-) |  | -0.3030 | 1.41e-06 | 1.43e-05 |
| ENSMUSG00000045348 | Nyap1 | protein\_coding | 5:137729899-137741607 (-) |  | 1.3200 | 1.42e-06 | 1.44e-05 |
| ENSMUSG00000066975 | Cryba4 | protein\_coding | 5:112246493-112252518 (-) |  | 1.0500 | 1.45e-06 | 1.47e-05 |
| ENSMUSG00000020585 | Laptm4a | protein\_coding | 12:8921664-8938742 (+) |  | 0.3350 | 1.46e-06 | 1.47e-05 |
| ENSMUSG00000022186 | Oxct1 | protein\_coding | 15:4026383-4155344 (+) |  | -0.3290 | 1.48e-06 | 1.49e-05 |
| ENSMUSG00000024053 | Emilin2 | protein\_coding | 17:71252172-71311978 (-) |  | -0.4070 | 1.50e-06 | 1.51e-05 |
| ENSMUSG00000021143 | Pacs2 | protein\_coding | 12:113014508-113074401 (+) |  | 0.3180 | 1.50e-06 | 1.51e-05 |
| ENSMUSG00000002983 | Relb | protein\_coding | 7:19606217-19629438 (-) |  | 0.6300 | 1.50e-06 | 1.52e-05 |
| ENSMUSG00000029507 | Pus1 | protein\_coding | 5:110773667-110780659 (-) |  | -0.2900 | 1.51e-06 | 1.52e-05 |
| ENSMUSG00000045763 | Basp1 | protein\_coding | 15:25363277-25413764 (-) |  | -1.0500 | 1.51e-06 | 1.52e-05 |
| ENSMUSG00000030579 | Tyrobp | protein\_coding | 7:30413760-30417585 (+) |  | 0.4270 | 1.52e-06 | 1.53e-05 |
| ENSMUSG00000042121 | Ssh1 | protein\_coding | 5:113937094-113993894 (-) |  | 0.4070 | 1.52e-06 | 1.53e-05 |
| ENSMUSG00000081684 | Rps2-ps13 | processed\_pseudogene | X:52898588-52899469 (+) |  | -0.4900 | 1.52e-06 | 1.53e-05 |
| ENSMUSG00000026696 | Vamp4 | protein\_coding | 1:162570515-162599084 (+) |  | -0.4340 | 1.53e-06 | 1.54e-05 |
| ENSMUSG00000025584 | Pde8a | protein\_coding | 7:81213596-81334533 (+) |  | -0.3920 | 1.53e-06 | 1.54e-05 |
| ENSMUSG00000003402 | Prkcsh | protein\_coding | 9:22002806-22014222 (+) |  | -0.3050 | 1.53e-06 | 1.54e-05 |
| ENSMUSG00000030207 | Fam234b | protein\_coding | 6:135197977-135244955 (+) |  | -0.5100 | 1.54e-06 | 1.54e-05 |
| ENSMUSG00000027006 | Dnajc10 | protein\_coding | 2:80315466-80354043 (+) |  | -0.2260 | 1.56e-06 | 1.57e-05 |
| ENSMUSG00000028063 | Lmna | protein\_coding | 3:88480147-88509956 (-) |  | -1.0100 | 1.57e-06 | 1.57e-05 |
| ENSMUSG00000021707 | Dhfr | protein\_coding | 13:92354726-92389053 (+) |  | -0.3990 | 1.58e-06 | 1.58e-05 |
| ENSMUSG00000026174 | Cnot9 | protein\_coding | 1:74506058-74530842 (+) |  | -0.2500 | 1.59e-06 | 1.59e-05 |
| ENSMUSG00000004798 | Ulk2 | protein\_coding | 11:61775649-61855073 (-) |  | 0.3510 | 1.60e-06 | 1.60e-05 |
| ENSMUSG00000103367 | Gm38158 | processed\_pseudogene | 1:176835724-176836062 (+) |  | 0.7410 | 1.61e-06 | 1.61e-05 |
| ENSMUSG00000023341 | Mx2 | polymorphic\_pseudogene | 16:97535308-97560900 (+) |  | 0.7280 | 1.63e-06 | 1.63e-05 |
| ENSMUSG00000028647 | Mycbp | protein\_coding | 4:123904832-123912269 (+) |  | -0.4580 | 1.64e-06 | 1.63e-05 |
| ENSMUSG00000024121 | Atp6v0c | protein\_coding | 17:24163866-24169702 (-) |  | -0.2550 | 1.66e-06 | 1.65e-05 |
| ENSMUSG00000028577 | Plaa | protein\_coding | 4:94567514-94603244 (-) |  | -0.2390 | 1.66e-06 | 1.65e-05 |
| ENSMUSG00000032078 | Zpr1 | protein\_coding | 9:46273064-46282643 (+) |  | -0.2690 | 1.69e-06 | 1.68e-05 |
| ENSMUSG00000027454 | Gins1 | protein\_coding | 2:150905400-150931280 (+) |  | -0.3920 | 1.69e-06 | 1.68e-05 |
| ENSMUSG00000054770 | Kctd18 | protein\_coding | 1:57955101-58018956 (-) |  | 0.3850 | 1.71e-06 | 1.70e-05 |
| ENSMUSG00000027540 | Ptpn1 | protein\_coding | 2:167932057-167979385 (+) |  | -0.3180 | 1.72e-06 | 1.71e-05 |
| ENSMUSG00000029299 | Abcg3 | protein\_coding | 5:104935057-104982718 (-) |  | 1.1400 | 1.74e-06 | 1.73e-05 |
| ENSMUSG00000020042 | Btbd11 | protein\_coding | 10:85386814-85660292 (+) |  | -0.8900 | 1.75e-06 | 1.73e-05 |
| ENSMUSG00000037395 | Rcor3 | protein\_coding | 1:192098546-192138062 (-) |  | 0.3790 | 1.76e-06 | 1.74e-05 |
| ENSMUSG00000001707 | Eef1e1 | protein\_coding | 13:38644207-38659058 (-) |  | -0.4090 | 1.76e-06 | 1.74e-05 |
| ENSMUSG00000006728 | Cdk4 | protein\_coding | 10:127063534-127067920 (+) |  | -0.3270 | 1.77e-06 | 1.76e-05 |
| ENSMUSG00000050377 | Il31ra | protein\_coding | 13:112519898-112594360 (-) |  | 0.6450 | 1.78e-06 | 1.76e-05 |
| ENSMUSG00000053175 | Bcl3 | protein\_coding | 7:19808462-19822770 (-) |  | -0.4710 | 1.79e-06 | 1.77e-05 |
| ENSMUSG00000032959 | Pebp1 | protein\_coding | 5:117282654-117287625 (-) |  | -0.3510 | 1.79e-06 | 1.77e-05 |
| ENSMUSG00000023022 | Lima1 | protein\_coding | 15:99778470-99875456 (-) |  | 0.5210 | 1.82e-06 | 1.79e-05 |
| ENSMUSG00000017057 | Il13ra1 | protein\_coding | X:36112110-36171259 (+) |  | -0.8550 | 1.84e-06 | 1.81e-05 |
| ENSMUSG00000106019 | Gm43672 | lncRNA | 3:22074412-22076142 (-) |  | 0.3770 | 1.85e-06 | 1.83e-05 |
| ENSMUSG00000024921 | Smarca2 | protein\_coding | 19:26605050-26778322 (+) |  | 0.3290 | 1.86e-06 | 1.83e-05 |
| ENSMUSG00000021661 | Ankra2 | protein\_coding | 13:98263074-98274754 (+) |  | 0.3910 | 1.87e-06 | 1.84e-05 |
| ENSMUSG00000057706 | Mex3b | protein\_coding | 7:82867333-82871515 (+) |  | 0.6080 | 1.90e-06 | 1.87e-05 |
| ENSMUSG00000060441 | Trim5 | protein\_coding | 7:104263386-104288094 (-) |  | 0.4420 | 1.91e-06 | 1.88e-05 |
| ENSMUSG00000026627 | Pacc1 | protein\_coding | 1:191325912-191350914 (+) |  | 0.3600 | 1.92e-06 | 1.88e-05 |
| ENSMUSG00000022433 | Csnk1e | protein\_coding | 15:79417856-79455566 (-) |  | 0.2440 | 1.92e-06 | 1.89e-05 |
| ENSMUSG00000032265 | Tent5a | protein\_coding | 9:85320439-85327348 (-) |  | 0.3230 | 1.94e-06 | 1.90e-05 |
| ENSMUSG00000020180 | Snrpd3 | protein\_coding | 10:75517551-75537381 (+) |  | -0.3520 | 1.96e-06 | 1.92e-05 |
| ENSMUSG00000030835 | Nomo1 | protein\_coding | 7:46033698-46084212 (+) |  | -0.3870 | 1.97e-06 | 1.93e-05 |
| ENSMUSG00000003546 | Klc4 | protein\_coding | 17:46630624-46646022 (-) |  | 0.3900 | 1.98e-06 | 1.93e-05 |
| ENSMUSG00000031493 | Ggn | protein\_coding | 7:29170210-29173976 (+) |  | -0.9840 | 1.99e-06 | 1.94e-05 |
| ENSMUSG00000055897 | Ppp4r1l-ps | transcribed\_unprocessed\_pseudogene | 2:173579320-173659640 (-) |  | 0.5920 | 1.99e-06 | 1.94e-05 |
| ENSMUSG00000053560 | Ier2 | protein\_coding | 8:84661331-84662854 (-) |  | 0.4620 | 1.99e-06 | 1.95e-05 |
| ENSMUSG00000004846 | Plod3 | protein\_coding | 5:136987019-136996648 (+) |  | -0.2430 | 2.04e-06 | 1.99e-05 |
| ENSMUSG00000022108 | Itm2b | protein\_coding | 14:73362226-73385289 (-) |  | 0.2400 | 2.05e-06 | 2.00e-05 |
| ENSMUSG00000036781 | Rps27l | protein\_coding | 9:66946086-66949516 (+) |  | -0.5270 | 2.06e-06 | 2.01e-05 |
| ENSMUSG00000075702 | Selenom | protein\_coding | 11:3514684-3517351 (+) |  | -0.5600 | 2.10e-06 | 2.05e-05 |
| ENSMUSG00000108414 | Snhg1 | lncRNA | 19:8723475-8726443 (+) |  | -0.3160 | 2.11e-06 | 2.05e-05 |
| ENSMUSG00000074785 | Plxnc1 | protein\_coding | 10:94790866-94944835 (-) |  | 0.3490 | 2.11e-06 | 2.05e-05 |
| ENSMUSG00000031928 | Mre11a | protein\_coding | 9:14784654-14837123 (+) |  | -0.2320 | 2.11e-06 | 2.05e-05 |
| ENSMUSG00000024150 | Mcfd2 | protein\_coding | 17:87254443-87265935 (-) |  | -0.2950 | 2.12e-06 | 2.06e-05 |
| ENSMUSG00000032314 | Etfa | protein\_coding | 9:55454508-55512243 (-) |  | -0.2570 | 2.13e-06 | 2.07e-05 |
| ENSMUSG00000038127 | Ccdc50 | protein\_coding | 16:27388869-27452218 (+) |  | 0.2980 | 2.15e-06 | 2.08e-05 |
| ENSMUSG00000035944 | Ttc38 | protein\_coding | 15:85832306-85858822 (+) |  | 0.3580 | 2.15e-06 | 2.08e-05 |
| ENSMUSG00000006736 | Tspan31 | protein\_coding | 10:127067280-127070264 (-) |  | -0.2210 | 2.16e-06 | 2.09e-05 |
| ENSMUSG00000028932 | Psmc2 | protein\_coding | 5:21785283-21803787 (+) |  | -0.2350 | 2.17e-06 | 2.10e-05 |
| ENSMUSG00000002835 | Chaf1a | protein\_coding | 17:56040439-56072289 (+) |  | -0.3550 | 2.18e-06 | 2.11e-05 |
| ENSMUSG00000063787 | Chchd1 | protein\_coding | 14:20703006-20704425 (+) |  | -0.4530 | 2.18e-06 | 2.11e-05 |
| ENSMUSG00000022822 | Abcc5 | protein\_coding | 16:20331303-20426394 (-) |  | 0.3330 | 2.18e-06 | 2.11e-05 |
| ENSMUSG00000032560 | Dnajc13 | protein\_coding | 9:104151282-104262930 (-) |  | -0.3010 | 2.19e-06 | 2.11e-05 |
| ENSMUSG00000097754 | Ptgs2os2 | lncRNA | 1:150159043-150164948 (-) |  | -2.0100 | 2.20e-06 | 2.12e-05 |
| ENSMUSG00000023175 | Bsg | protein\_coding | 10:79704491-79711969 (+) |  | -0.2210 | 2.20e-06 | 2.12e-05 |
| ENSMUSG00000028671 | Gale | protein\_coding | 4:135963727-135968178 (+) |  | -0.3970 | 2.21e-06 | 2.13e-05 |
| ENSMUSG00000025422 | Agap2 | protein\_coding | 10:127075284-127093169 (+) |  | 0.3200 | 2.21e-06 | 2.13e-05 |
| ENSMUSG00000027067 | Ssrp1 | protein\_coding | 2:85037234-85047109 (+) |  | -0.2080 | 2.22e-06 | 2.13e-05 |
| ENSMUSG00000023505 | Cdca3 | protein\_coding | 6:124829547-124833701 (+) |  | -0.2570 | 2.22e-06 | 2.13e-05 |
| ENSMUSG00000016619 | Nup50 | protein\_coding | 15:84923411-84942963 (+) |  | -0.2220 | 2.22e-06 | 2.13e-05 |
| ENSMUSG00000029478 | Ncor2 | protein\_coding | 5:125017153-125179219 (-) |  | 0.3910 | 2.22e-06 | 2.14e-05 |
| ENSMUSG00000031843 | Mphosph6 | protein\_coding | 8:117791645-117801943 (-) |  | -0.3790 | 2.23e-06 | 2.14e-05 |
| ENSMUSG00000007739 | Cct4 | protein\_coding | 11:22990519-23003780 (+) |  | -0.2630 | 2.24e-06 | 2.15e-05 |
| ENSMUSG00000044749 | Abca6 | protein\_coding | 11:110176820-110251776 (-) |  | 1.1300 | 2.26e-06 | 2.16e-05 |
| ENSMUSG00000040712 | Camta2 | protein\_coding | 11:70669463-70688105 (-) |  | 0.3770 | 2.27e-06 | 2.17e-05 |
| ENSMUSG00000044734 | Serpinb1a | protein\_coding | 13:32842092-32851185 (-) |  | -0.4530 | 2.27e-06 | 2.17e-05 |
| ENSMUSG00000007721 | Ccdc124 | protein\_coding | 8:70868227-70873935 (-) |  | -0.2910 | 2.28e-06 | 2.18e-05 |
| ENSMUSG00000028455 | Stoml2 | protein\_coding | 4:43027690-43031710 (-) |  | -0.3030 | 2.28e-06 | 2.18e-05 |
| ENSMUSG00000045216 | Hs6st1 | protein\_coding | 1:36068400-36106446 (+) |  | 0.3580 | 2.28e-06 | 2.18e-05 |
| ENSMUSG00000027404 | Snrpb | protein\_coding | 2:130171414-130179403 (-) |  | -0.1730 | 2.29e-06 | 2.18e-05 |
| ENSMUSG00000030282 | Cmas | protein\_coding | 6:142756686-142775714 (+) |  | -0.2700 | 2.30e-06 | 2.19e-05 |
| ENSMUSG00000039747 | Orai2 | protein\_coding | 5:136147459-136170713 (-) |  | 0.2980 | 2.31e-06 | 2.20e-05 |
| ENSMUSG00000016534 | Lamp2 | protein\_coding | X:38401357-38456454 (-) |  | -0.3390 | 2.34e-06 | 2.23e-05 |
| ENSMUSG00000021338 | Carmil1 | protein\_coding | 13:24012344-24280795 (-) |  | 0.8060 | 2.36e-06 | 2.25e-05 |
| ENSMUSG00000062901 | Klhl24 | protein\_coding | 16:20097542-20129221 (+) |  | 0.3280 | 2.39e-06 | 2.27e-05 |
| ENSMUSG00000048578 | Mlec | protein\_coding | 5:115142981-115158179 (-) |  | -0.2380 | 2.40e-06 | 2.28e-05 |
| ENSMUSG00000026630 | Batf3 | protein\_coding | 1:191097847-191108945 (+) |  | 1.3300 | 2.40e-06 | 2.28e-05 |
| ENSMUSG00000046434 | Hnrnpa1 | protein\_coding | 15:103240432-103246692 (+) |  | -0.3230 | 2.41e-06 | 2.29e-05 |
| ENSMUSG00000004356 | Utp20 | protein\_coding | 10:88746607-88826804 (-) |  | -0.4730 | 2.44e-06 | 2.32e-05 |
| ENSMUSG00000024953 | Prdx5 | protein\_coding | 19:6906697-6910106 (-) |  | 0.4530 | 2.44e-06 | 2.32e-05 |
| ENSMUSG00000000088 | Cox5a | protein\_coding | 9:57521274-57532426 (+) |  | -0.3530 | 2.46e-06 | 2.33e-05 |
| ENSMUSG00000042747 | Krtcap2 | protein\_coding | 3:89245966-89249906 (+) |  | -0.3120 | 2.47e-06 | 2.34e-05 |
| ENSMUSG00000031242 | 2610002M06Rik | protein\_coding | X:107782751-107816334 (-) |  | -0.3220 | 2.48e-06 | 2.34e-05 |
| ENSMUSG00000035967 | Ints6l | protein\_coding | X:56454857-56507843 (+) |  | 0.4170 | 2.49e-06 | 2.35e-05 |
| ENSMUSG00000018427 | Ypel2 | protein\_coding | 11:86936425-86993707 (-) |  | 0.5430 | 2.51e-06 | 2.37e-05 |
| ENSMUSG00000021508 | Cxcl14 | protein\_coding | 13:56288643-56296551 (-) |  | 2.5200 | 2.51e-06 | 2.37e-05 |
| ENSMUSG00000041471 | Shld2 | protein\_coding | 14:34237033-34310493 (-) |  | 0.3950 | 2.52e-06 | 2.38e-05 |
| ENSMUSG00000010307 | Tmem86a | protein\_coding | 7:47050601-47054777 (+) |  | 0.5000 | 2.53e-06 | 2.39e-05 |
| ENSMUSG00000053965 | Pde5a | protein\_coding | 3:122728947-122859374 (+) |  | -0.7020 | 2.55e-06 | 2.40e-05 |
| ENSMUSG00000044229 | Nxpe4 | protein\_coding | 9:48162023-48400025 (+) |  | 0.8090 | 2.56e-06 | 2.41e-05 |
| ENSMUSG00000023852 | Chd1 | protein\_coding | 17:15704967-15772610 (+) |  | -0.2410 | 2.56e-06 | 2.41e-05 |
| ENSMUSG00000024829 | Mrpl21 | protein\_coding | 19:3282901-3292837 (+) |  | -0.2970 | 2.59e-06 | 2.43e-05 |
| ENSMUSG00000035539 | Ccdc180 | protein\_coding | 4:45890303-45950774 (+) |  | 0.6960 | 2.60e-06 | 2.44e-05 |
| ENSMUSG00000004633 | Chn2 | protein\_coding | 6:54039554-54301810 (+) |  | 0.4980 | 2.61e-06 | 2.45e-05 |
| ENSMUSG00000066682 | Pilrb2 | protein\_coding | 5:137865827-137871815 (-) |  | 0.8070 | 2.66e-06 | 2.50e-05 |
| ENSMUSG00000089844 | A530032D15Rik | protein\_coding | 1:85083069-85109853 (-) |  | -1.1500 | 2.68e-06 | 2.51e-05 |
| ENSMUSG00000027288 | Zfp106 | protein\_coding | 2:120506820-120563843 (-) |  | -0.2340 | 2.68e-06 | 2.51e-05 |
| ENSMUSG00000040658 | Dnph1 | protein\_coding | 17:46496711-46499624 (+) |  | -0.4690 | 2.71e-06 | 2.54e-05 |
| ENSMUSG00000034789 | Rab24 | protein\_coding | 13:55319743-55321946 (-) |  | -0.2730 | 2.71e-06 | 2.54e-05 |
| ENSMUSG00000087543 | Gm16576 | lncRNA | 15:79742698-79757394 (+) |  | 0.5430 | 2.72e-06 | 2.54e-05 |
| ENSMUSG00000022752 | Tomm70a | protein\_coding | 16:57121703-57156705 (+) |  | -0.2840 | 2.77e-06 | 2.59e-05 |
| ENSMUSG00000039509 | Nup133 | protein\_coding | 8:123897123-123949265 (-) |  | -0.2840 | 2.80e-06 | 2.62e-05 |
| ENSMUSG00000027087 | Itgav | protein\_coding | 2:83724397-83806916 (+) |  | 0.2890 | 2.82e-06 | 2.63e-05 |
| ENSMUSG00000070873 | Lilra5 | protein\_coding | 7:4237754-4243463 (+) |  | 3.9900 | 2.84e-06 | 2.66e-05 |
| ENSMUSG00000025225 | Nfkb2 | protein\_coding | 19:46304320-46312385 (+) |  | 0.3190 | 2.86e-06 | 2.67e-05 |
| ENSMUSG00000087281 | Gm16015 | lncRNA | 5:43909215-43909885 (-) |  | -4.5600 | 2.87e-06 | 2.67e-05 |
| ENSMUSG00000025872 | Thoc3 | protein\_coding | 13:54458837-54468849 (-) |  | -0.2200 | 2.87e-06 | 2.68e-05 |
| ENSMUSG00000026923 | Notch1 | protein\_coding | 2:26457903-26516663 (-) |  | 0.3670 | 2.88e-06 | 2.69e-05 |
| ENSMUSG00000021810 | Ecd | protein\_coding | 14:20319852-20348121 (-) |  | -0.2470 | 2.89e-06 | 2.69e-05 |
| ENSMUSG00000006127 | Inpp5k | protein\_coding | 11:75630988-75648871 (+) |  | 0.2730 | 2.90e-06 | 2.70e-05 |
| ENSMUSG00000055538 | Zcchc24 | protein\_coding | 14:25711642-25769039 (-) |  | 0.4760 | 2.91e-06 | 2.71e-05 |
| ENSMUSG00000031608 | Galnt7 | protein\_coding | 8:57523828-57653032 (-) |  | -0.2710 | 2.92e-06 | 2.71e-05 |
| ENSMUSG00000044149 | Nkrf | protein\_coding | X:36887540-36903513 (-) |  | -0.3730 | 2.93e-06 | 2.72e-05 |
| ENSMUSG00000032407 | U2surp | protein\_coding | 9:95456898-95511996 (-) |  | -0.2150 | 2.95e-06 | 2.74e-05 |
| ENSMUSG00000015474 | Ppt2 | protein\_coding | 17:34616662-34628510 (-) |  | 0.3590 | 2.95e-06 | 2.74e-05 |
| ENSMUSG00000002996 | Hbp1 | protein\_coding | 12:31926254-31950535 (-) |  | 0.3280 | 2.96e-06 | 2.74e-05 |
| ENSMUSG00000017677 | Wsb1 | protein\_coding | 11:79239372-79254671 (-) |  | 0.2620 | 3.01e-06 | 2.79e-05 |
| ENSMUSG00000028410 | Dnaja1 | protein\_coding | 4:40722150-40737149 (+) |  | -0.3230 | 3.02e-06 | 2.79e-05 |
| ENSMUSG00000033166 | Dis3 | protein\_coding | 14:99075206-99099770 (-) |  | -0.3100 | 3.02e-06 | 2.80e-05 |
| ENSMUSG00000057596 | Trim30d | protein\_coding | 7:104470014-104507849 (-) |  | 0.3790 | 3.03e-06 | 2.80e-05 |
| ENSMUSG00000028069 | Gpatch4 | protein\_coding | 3:88043108-88055993 (+) |  | -0.3910 | 3.03e-06 | 2.80e-05 |
| ENSMUSG00000027163 | Commd9 | protein\_coding | 2:101886247-101901646 (+) |  | -0.3290 | 3.03e-06 | 2.80e-05 |
| ENSMUSG00000040410 | Fbxl4 | protein\_coding | 4:22357543-22434091 (+) |  | 0.4070 | 3.05e-06 | 2.81e-05 |
| ENSMUSG00000030397 | Mark4 | protein\_coding | 7:19424775-19458821 (-) |  | 0.3090 | 3.09e-06 | 2.84e-05 |
| ENSMUSG00000005674 | Tomm40l | protein\_coding | 1:171216011-171222514 (-) |  | -0.4180 | 3.10e-06 | 2.86e-05 |
| ENSMUSG00000041057 | Wdr43 | protein\_coding | 17:71615895-71659031 (+) |  | -0.2630 | 3.11e-06 | 2.86e-05 |
| ENSMUSG00000029059 | Prxl2b | protein\_coding | 4:154895504-154899135 (-) |  | 0.6360 | 3.16e-06 | 2.91e-05 |
| ENSMUSG00000039470 | Zdhhc2 | protein\_coding | 8:40423815-40510268 (+) |  | -1.0900 | 3.17e-06 | 2.91e-05 |
| ENSMUSG00000028964 | Park7 | protein\_coding | 4:150897133-150914437 (-) |  | -0.3680 | 3.18e-06 | 2.92e-05 |
| ENSMUSG00000035049 | Rrp12 | protein\_coding | 19:41862851-41896173 (-) |  | -0.4300 | 3.20e-06 | 2.94e-05 |
| ENSMUSG00000019782 | Rwdd1 | protein\_coding | 10:33996555-34019624 (-) |  | -0.3650 | 3.21e-06 | 2.95e-05 |
| ENSMUSG00000022350 | Washc5 | protein\_coding | 15:59331997-59374167 (-) |  | -0.2430 | 3.26e-06 | 2.99e-05 |
| ENSMUSG00000005846 | Rsl1d1 | protein\_coding | 16:11192970-11203331 (-) |  | -0.3250 | 3.29e-06 | 3.02e-05 |
| ENSMUSG00000017760 | Ctsa | protein\_coding | 2:164832873-164841032 (+) |  | -0.2050 | 3.31e-06 | 3.03e-05 |
| ENSMUSG00000058818 | Pirb | protein\_coding | 7:3711409-3720391 (-) |  | -0.4930 | 3.32e-06 | 3.04e-05 |
| ENSMUSG00000033400 | Agl | protein\_coding | 3:116739999-116808166 (-) |  | 0.2430 | 3.33e-06 | 3.04e-05 |
| ENSMUSG00000073400 | Trim10 | protein\_coding | 17:36869574-36877833 (+) |  | 2.1300 | 3.33e-06 | 3.04e-05 |
| ENSMUSG00000021286 | Zfyve21 | protein\_coding | 12:111814170-111832192 (+) |  | 0.4020 | 3.33e-06 | 3.04e-05 |
| ENSMUSG00000027615 | Hps3 | protein\_coding | 3:19995945-20035315 (-) |  | 0.3530 | 3.38e-06 | 3.09e-05 |
| ENSMUSG00000023307 | March5 | protein\_coding | 19:37207543-37222151 (+) |  | -0.2400 | 3.40e-06 | 3.10e-05 |
| ENSMUSG00000032396 | Dis3l | protein\_coding | 9:64306756-64341288 (-) |  | -0.2540 | 3.41e-06 | 3.11e-05 |
| ENSMUSG00000027712 | Anxa5 | protein\_coding | 3:36448923-36475894 (-) |  | 0.3160 | 3.45e-06 | 3.15e-05 |
| ENSMUSG00000034353 | Ramp1 | protein\_coding | 1:91179822-91225196 (+) |  | 0.4610 | 3.46e-06 | 3.15e-05 |
| ENSMUSG00000116504 | I730030J21Rik | lncRNA | 15:100730481-100732737 (-) |  | 1.4900 | 3.47e-06 | 3.16e-05 |
| ENSMUSG00000063605 | Ccdc102a | protein\_coding | 8:94902869-94918098 (-) |  | 0.6380 | 3.48e-06 | 3.16e-05 |
| ENSMUSG00000039879 | Heca | protein\_coding | 10:17868612-17948067 (-) |  | 0.3080 | 3.49e-06 | 3.17e-05 |
| ENSMUSG00000026171 | Rnf25 | protein\_coding | 1:74593748-74601397 (-) |  | 0.2850 | 3.49e-06 | 3.17e-05 |
| ENSMUSG00000052331 | Ankrd44 | protein\_coding | 1:54645340-54926387 (-) |  | 0.2770 | 3.51e-06 | 3.18e-05 |
| ENSMUSG00000023952 | Gtpbp2 | protein\_coding | 17:46161032-46169370 (+) |  | 0.3020 | 3.51e-06 | 3.19e-05 |
| ENSMUSG00000028282 | Casp8ap2 | protein\_coding | 4:32615451-32653265 (+) |  | -0.2320 | 3.54e-06 | 3.21e-05 |
| ENSMUSG00000017405 | Nek8 | protein\_coding | 11:78166106-78176675 (-) |  | 0.5110 | 3.54e-06 | 3.21e-05 |
| ENSMUSG00000020349 | Ppp2ca | protein\_coding | 11:52098681-52127778 (+) |  | -0.3590 | 3.55e-06 | 3.21e-05 |
| ENSMUSG00000061371 | Zfp873 | protein\_coding | 10:82048123-82064745 (+) |  | 0.4880 | 3.56e-06 | 3.22e-05 |
| ENSMUSG00000029017 | Pmpcb | protein\_coding | 5:21737141-21757152 (+) |  | -0.2330 | 3.56e-06 | 3.22e-05 |
| ENSMUSG00000009575 | Cbx5 | protein\_coding | 15:103191544-103239816 (-) |  | -0.3500 | 3.60e-06 | 3.26e-05 |
| ENSMUSG00000057541 | Pus7 | protein\_coding | 5:23740648-23783711 (-) |  | -0.4680 | 3.66e-06 | 3.31e-05 |
| ENSMUSG00000026986 | Hnmt | protein\_coding | 2:24002910-24049394 (-) |  | -0.6270 | 3.67e-06 | 3.31e-05 |
| ENSMUSG00000029191 | Rfc1 | protein\_coding | 5:65261850-65335670 (-) |  | -0.2490 | 3.68e-06 | 3.32e-05 |
| ENSMUSG00000050821 | Fam131a | protein\_coding | 16:20693241-20703048 (+) |  | 0.9330 | 3.69e-06 | 3.32e-05 |
| ENSMUSG00000061904 | Slc25a3 | protein\_coding | 10:91116574-91124059 (-) |  | -0.2240 | 3.69e-06 | 3.33e-05 |
| ENSMUSG00000025223 | Ldb1 | protein\_coding | 19:46031570-46045214 (-) |  | 0.2410 | 3.73e-06 | 3.36e-05 |
| ENSMUSG00000040034 | Nup43 | protein\_coding | 10:7667503-7678881 (+) |  | -0.4440 | 3.73e-06 | 3.36e-05 |
| ENSMUSG00000038143 | Stox2 | protein\_coding | 8:47180048-47446362 (-) |  | 0.4820 | 3.74e-06 | 3.37e-05 |
| ENSMUSG00000029413 | Naaa | protein\_coding | 5:92257659-92278170 (-) |  | -0.4030 | 3.75e-06 | 3.37e-05 |
| ENSMUSG00000030220 | Arhgdib | protein\_coding | 6:136923655-136941899 (-) |  | -0.1620 | 3.76e-06 | 3.37e-05 |
| ENSMUSG00000027367 | Stard7 | protein\_coding | 2:127270218-127298932 (+) |  | -0.2060 | 3.76e-06 | 3.37e-05 |
| ENSMUSG00000020780 | Srp68 | protein\_coding | 11:116245166-116274217 (-) |  | -0.2550 | 3.76e-06 | 3.38e-05 |
| ENSMUSG00000021040 | Slirp | protein\_coding | 12:87443896-87452206 (+) |  | -0.5510 | 3.76e-06 | 3.38e-05 |
| ENSMUSG00000040565 | Btaf1 | protein\_coding | 19:36926079-37012752 (+) |  | -0.2130 | 3.77e-06 | 3.38e-05 |
| ENSMUSG00000001761 | Smo | protein\_coding | 6:29735503-29761365 (+) |  | -0.5760 | 3.79e-06 | 3.39e-05 |
| ENSMUSG00000024576 | Csnk1a1 | protein\_coding | 18:61555274-61590061 (+) |  | -0.1740 | 3.82e-06 | 3.42e-05 |
| ENSMUSG00000027282 | Mtch2 | protein\_coding | 2:90847155-90866810 (+) |  | -0.2480 | 3.83e-06 | 3.42e-05 |
| ENSMUSG00000011263 | Exoc3l2 | protein\_coding | 7:19463331-19496762 (+) |  | 1.5200 | 3.84e-06 | 3.43e-05 |
| ENSMUSG00000015112 | Slc25a13 | protein\_coding | 6:6041218-6217173 (-) |  | -0.2380 | 3.84e-06 | 3.43e-05 |
| ENSMUSG00000032652 | Crebl2 | protein\_coding | 6:134830154-134858931 (+) |  | 0.5710 | 3.84e-06 | 3.43e-05 |
| ENSMUSG00000036362 | P2ry13 | protein\_coding | 3:59207892-59210882 (-) |  | -1.0000 | 3.85e-06 | 3.44e-05 |
| ENSMUSG00000035697 | Arhgap45 | protein\_coding | 10:80016653-80031472 (+) |  | 0.2180 | 3.88e-06 | 3.46e-05 |
| ENSMUSG00000017999 | Ddx27 | protein\_coding | 2:167015193-167034947 (+) |  | -0.2570 | 3.92e-06 | 3.49e-05 |
| ENSMUSG00000035172 | Plekhh3 | protein\_coding | 11:101162679-101171351 (-) |  | 0.6650 | 3.92e-06 | 3.49e-05 |
| ENSMUSG00000020914 | Top2a | protein\_coding | 11:98992943-99024189 (-) |  | -0.2400 | 3.93e-06 | 3.50e-05 |
| ENSMUSG00000024952 | Rps6ka4 | protein\_coding | 19:6829085-6840636 (-) |  | -0.2540 | 3.93e-06 | 3.50e-05 |
| ENSMUSG00000090164 | BC035044 | protein\_coding | 6:128849090-128891126 (-) |  | 0.3770 | 3.96e-06 | 3.52e-05 |
| ENSMUSG00000022892 | App | protein\_coding | 16:84949685-85173766 (-) |  | -0.2540 | 3.97e-06 | 3.53e-05 |
| ENSMUSG00000041733 | Coq5 | protein\_coding | 5:115279666-115296972 (+) |  | -0.2830 | 3.98e-06 | 3.53e-05 |
| ENSMUSG00000070526 | Peg12 | protein\_coding | 7:62461871-62464510 (-) |  | 0.9650 | 4.04e-06 | 3.58e-05 |
| ENSMUSG00000004105 | Angptl2 | protein\_coding | 2:33216069-33247717 (+) |  | 1.1500 | 4.07e-06 | 3.61e-05 |
| ENSMUSG00000001604 | Tcea3 | protein\_coding | 4:136247729-136274898 (+) |  | 0.8770 | 4.08e-06 | 3.61e-05 |
| ENSMUSG00000021193 | Pitrm1 | protein\_coding | 13:6548149-6580515 (+) |  | -0.3010 | 4.08e-06 | 3.62e-05 |
| ENSMUSG00000078812 | Eif5a | protein\_coding | 11:69916714-69921958 (-) |  | -0.2860 | 4.09e-06 | 3.62e-05 |
| ENSMUSG00000022472 | Desi1 | protein\_coding | 15:81992523-82033862 (-) |  | -0.2970 | 4.09e-06 | 3.62e-05 |
| ENSMUSG00000006398 | Cdc20 | protein\_coding | 4:118432901-118437352 (-) |  | -0.2420 | 4.10e-06 | 3.63e-05 |
| ENSMUSG00000021102 | Glrx5 | protein\_coding | 12:105032688-105042906 (+) |  | -0.2810 | 4.12e-06 | 3.64e-05 |
| ENSMUSG00000052656 | Rnf103 | protein\_coding | 6:71493894-71510881 (+) |  | 0.3360 | 4.15e-06 | 3.66e-05 |
| ENSMUSG00000091575 | 2010016I18Rik | lncRNA | 3:106481982-106485913 (-) |  | -0.7970 | 4.15e-06 | 3.66e-05 |
| ENSMUSG00000024174 | Pot1b | protein\_coding | 17:55651951-55712628 (-) |  | 0.4480 | 4.18e-06 | 3.69e-05 |
| ENSMUSG00000024726 | Carnmt1 | protein\_coding | 19:18670764-18707200 (+) |  | -0.3890 | 4.23e-06 | 3.73e-05 |
| ENSMUSG00000035236 | Scai | protein\_coding | 2:39066214-39190734 (-) |  | 0.6280 | 4.23e-06 | 3.73e-05 |
| ENSMUSG00000036398 | Ppp1r11 | protein\_coding | 17:36948356-36951741 (-) |  | -0.3300 | 4.23e-06 | 3.73e-05 |
| ENSMUSG00000034932 | Mrpl54 | protein\_coding | 10:81264713-81266934 (-) |  | -0.3720 | 4.25e-06 | 3.74e-05 |
| ENSMUSG00000064210 | Ano6 | protein\_coding | 15:95790843-95974751 (+) |  | 0.2630 | 4.35e-06 | 3.82e-05 |
| ENSMUSG00000039086 | Ss18l1 | protein\_coding | 2:180042509-180070201 (+) |  | 0.5960 | 4.38e-06 | 3.85e-05 |
| ENSMUSG00000027952 | Pmvk | protein\_coding | 3:89454541-89469013 (+) |  | -0.4000 | 4.40e-06 | 3.86e-05 |
| ENSMUSG00000009894 | Snap47 | protein\_coding | 11:59407134-59451186 (-) |  | -0.8320 | 4.42e-06 | 3.88e-05 |
| ENSMUSG00000037946 | Fgd3 | protein\_coding | 13:49261554-49320311 (-) |  | 0.3250 | 4.43e-06 | 3.88e-05 |
| ENSMUSG00000034445 | Cyb561a3 | protein\_coding | 19:10577172-10595961 (+) |  | 0.3310 | 4.46e-06 | 3.91e-05 |
| ENSMUSG00000038279 | Nop2 | protein\_coding | 6:125131909-125144753 (+) |  | -0.3110 | 4.48e-06 | 3.93e-05 |
| ENSMUSG00000078521 | Aunip | protein\_coding | 4:134510999-134523927 (+) |  | -0.4340 | 4.49e-06 | 3.93e-05 |
| ENSMUSG00000026688 | Mgst3 | protein\_coding | 1:167371966-167393841 (-) |  | -0.4490 | 4.54e-06 | 3.97e-05 |
| ENSMUSG00000074342 | I830077J02Rik | protein\_coding | 3:105924358-105932664 (-) |  | 0.2670 | 4.54e-06 | 3.97e-05 |
| ENSMUSG00000023456 | Tpi1 | protein\_coding | 6:124810586-124814296 (-) |  | -0.3530 | 4.56e-06 | 3.99e-05 |
| ENSMUSG00000002897 | Il17ra | protein\_coding | 6:120463247-120487559 (+) |  | 0.2460 | 4.56e-06 | 3.99e-05 |
| ENSMUSG00000068923 | Syt11 | protein\_coding | 3:88744700-88775164 (-) |  | -0.5330 | 4.57e-06 | 3.99e-05 |
| ENSMUSG00000022551 | Cyc1 | protein\_coding | 15:76343523-76346260 (+) |  | -0.3020 | 4.59e-06 | 4.01e-05 |
| ENSMUSG00000021211 | Akr1c12 | protein\_coding | 13:4268176-4279433 (-) |  | 2.1100 | 4.62e-06 | 4.03e-05 |
| ENSMUSG00000038975 | Rabggtb | protein\_coding | 3:153907287-153913009 (-) |  | -0.2900 | 4.63e-06 | 4.03e-05 |
| ENSMUSG00000036533 | Cdc42ep3 | protein\_coding | 17:79333727-79355091 (-) |  | 0.2720 | 4.63e-06 | 4.03e-05 |
| ENSMUSG00000024422 | Dhx16 | protein\_coding | 17:35879819-35892670 (+) |  | -0.2120 | 4.65e-06 | 4.04e-05 |
| ENSMUSG00000014355 | Anapc1 | protein\_coding | 2:128610104-128687391 (-) |  | -0.2820 | 4.65e-06 | 4.04e-05 |
| ENSMUSG00000021108 | Prkch | protein\_coding | 12:73584796-73778185 (+) |  | 0.4160 | 4.68e-06 | 4.06e-05 |
| ENSMUSG00000002279 | Lmf1 | protein\_coding | 17:25579085-25662826 (+) |  | 0.4810 | 4.68e-06 | 4.06e-05 |
| ENSMUSG00000018379 | Srsf1 | protein\_coding | 11:88047373-88053755 (+) |  | -0.1860 | 4.70e-06 | 4.08e-05 |
| ENSMUSG00000024844 | Banf1 | protein\_coding | 19:5364638-5367168 (-) |  | -0.2950 | 4.71e-06 | 4.09e-05 |
| ENSMUSG00000029484 | Anxa3 | protein\_coding | 5:96793339-96845966 (+) |  | 0.2450 | 4.71e-06 | 4.09e-05 |
| ENSMUSG00000062075 | Lmnb2 | protein\_coding | 10:80901203-80918245 (-) |  | -0.2300 | 4.72e-06 | 4.09e-05 |
| ENSMUSG00000062580 | Timm17a | protein\_coding | 1:135295213-135313778 (-) |  | -0.3060 | 4.73e-06 | 4.10e-05 |
| ENSMUSG00000026110 | Mgat4a | protein\_coding | 1:37439340-37541016 (-) |  | -0.5390 | 4.74e-06 | 4.11e-05 |
| ENSMUSG00000028277 | Ube2j1 | protein\_coding | 4:33031416-33052363 (+) |  | -0.2070 | 4.75e-06 | 4.11e-05 |
| ENSMUSG00000029701 | Rbm28 | protein\_coding | 6:29123576-29165006 (-) |  | -0.2450 | 4.75e-06 | 4.11e-05 |
| ENSMUSG00000032458 | Copb2 | protein\_coding | 9:98563721-98588382 (+) |  | -0.1700 | 4.81e-06 | 4.15e-05 |
| ENSMUSG00000118164 | Gm20570 | processed\_pseudogene | 18:75767143-75767274 (+) |  | 0.8300 | 4.84e-06 | 4.18e-05 |
| ENSMUSG00000025357 | Dgka | protein\_coding | 10:128720134-128744855 (-) |  | 0.3340 | 4.87e-06 | 4.20e-05 |
| ENSMUSG00000058809 | Hspd1-ps3 | processed\_pseudogene | 11:41498737-41500458 (+) |  | -0.5660 | 4.89e-06 | 4.22e-05 |
| ENSMUSG00000047735 | Samd9l | protein\_coding | 6:3372257-3399572 (-) |  | 0.7820 | 4.90e-06 | 4.22e-05 |
| ENSMUSG00000020922 | Lsm12 | protein\_coding | 11:102162497-102185296 (-) |  | -0.2390 | 4.90e-06 | 4.23e-05 |
| ENSMUSG00000026558 | Uck2 | protein\_coding | 1:167222883-167285320 (-) |  | -0.3230 | 4.92e-06 | 4.24e-05 |
| ENSMUSG00000032096 | Arcn1 | protein\_coding | 9:44741564-44767845 (-) |  | -0.1810 | 4.98e-06 | 4.29e-05 |
| ENSMUSG00000070002 | Ell | protein\_coding | 8:70539457-70592858 (+) |  | 0.2790 | 4.99e-06 | 4.29e-05 |
| ENSMUSG00000001305 | Rrp15 | protein\_coding | 1:186720978-186749358 (-) |  | -0.3270 | 4.99e-06 | 4.29e-05 |
| ENSMUSG00000027215 | Cd82 | protein\_coding | 2:93419111-93463140 (-) |  | 0.3540 | 4.99e-06 | 4.29e-05 |
| ENSMUSG00000023004 | Tuba1b | protein\_coding | 15:98931425-98934565 (-) |  | -0.2950 | 5.01e-06 | 4.30e-05 |
| ENSMUSG00000000730 | Dnmt3l | protein\_coding | 10:78041947-78063622 (+) |  | -1.5400 | 5.01e-06 | 4.30e-05 |
| ENSMUSG00000021548 | Ccnh | protein\_coding | 13:85189408-85223469 (+) |  | -0.2490 | 5.05e-06 | 4.33e-05 |
| ENSMUSG00000057219 | Armc7 | protein\_coding | 11:115475667-115490467 (+) |  | 0.3280 | 5.08e-06 | 4.35e-05 |
| ENSMUSG00000029616 | Erp29 | protein\_coding | 5:121428590-121452506 (-) |  | -0.2100 | 5.10e-06 | 4.37e-05 |
| ENSMUSG00000028693 | Nasp | protein\_coding | 4:116601052-116627941 (-) |  | -0.2490 | 5.12e-06 | 4.38e-05 |
| ENSMUSG00000034833 | Tespa1 | protein\_coding | 10:130322870-130364111 (+) |  | 0.6820 | 5.15e-06 | 4.41e-05 |
| ENSMUSG00000029415 | Sdad1 | protein\_coding | 5:92284010-92310479 (-) |  | -0.2010 | 5.16e-06 | 4.41e-05 |
| ENSMUSG00000030733 | Sh2b1 | protein\_coding | 7:126466994-126475424 (-) |  | 0.3360 | 5.17e-06 | 4.42e-05 |
| ENSMUSG00000055172 | C1ra | protein\_coding | 6:124512405-124523443 (+) |  | 0.5120 | 5.17e-06 | 4.42e-05 |
| ENSMUSG00000041313 | Slc7a1 | protein\_coding | 5:148327410-148399904 (-) |  | -0.3660 | 5.20e-06 | 4.44e-05 |
| ENSMUSG00000022956 | Atp5o | protein\_coding | 16:91925214-91931687 (-) |  | -0.2100 | 5.20e-06 | 4.44e-05 |
| ENSMUSG00000038034 | Igsf8 | protein\_coding | 1:172261641-172319841 (+) |  | -0.3260 | 5.21e-06 | 4.45e-05 |
| ENSMUSG00000020137 | Thap2 | protein\_coding | 10:115368404-115384443 (-) |  | -0.3790 | 5.22e-06 | 4.45e-05 |
| ENSMUSG00000098055 | Gm26947 | lncRNA | 10:60931991-60940942 (+) |  | -0.7540 | 5.25e-06 | 4.47e-05 |
| ENSMUSG00000001270 | Ckb | protein\_coding | 12:111669361-111672338 (-) |  | 0.7200 | 5.31e-06 | 4.52e-05 |
| ENSMUSG00000028420 | Tmem38b | protein\_coding | 4:53826045-53862019 (+) |  | -0.3550 | 5.32e-06 | 4.52e-05 |
| ENSMUSG00000018341 | Il12rb2 | protein\_coding | 6:67291318-67376188 (-) |  | 1.5500 | 5.35e-06 | 4.54e-05 |
| ENSMUSG00000042628 | Zfyve1 | protein\_coding | 12:83546558-83597222 (-) |  | 0.3130 | 5.35e-06 | 4.54e-05 |
| ENSMUSG00000064326 | Siva1 | protein\_coding | 12:112644679-112649149 (+) |  | -0.2950 | 5.36e-06 | 4.55e-05 |
| ENSMUSG00000075592 | Nynrin | protein\_coding | 14:55854010-55874736 (+) |  | 0.5060 | 5.37e-06 | 4.56e-05 |
| ENSMUSG00000036932 | Aifm1 | protein\_coding | X:48474944-48513563 (-) |  | -0.2620 | 5.40e-06 | 4.58e-05 |
| ENSMUSG00000091478 | Gm10039 | processed\_pseudogene | 11:100361716-100362126 (-) |  | -0.3360 | 5.43e-06 | 4.60e-05 |
| ENSMUSG00000022594 | Lynx1 | protein\_coding | 15:74747852-74753046 (-) |  | 0.6120 | 5.46e-06 | 4.62e-05 |
| ENSMUSG00000025869 | Nop16 | protein\_coding | 13:54584185-54590090 (-) |  | -0.3590 | 5.48e-06 | 4.64e-05 |
| ENSMUSG00000029428 | Stx2 | protein\_coding | 5:128984557-129008574 (-) |  | 0.3440 | 5.52e-06 | 4.67e-05 |
| ENSMUSG00000038991 | Txndc5 | protein\_coding | 13:38500079-38528824 (-) |  | -0.2350 | 5.52e-06 | 4.67e-05 |
| ENSMUSG00000000826 | Dnajc5 | protein\_coding | 2:181520485-181555133 (+) |  | 0.1310 | 5.56e-06 | 4.70e-05 |
| ENSMUSG00000073412 | Lst1 | protein\_coding | 17:35185095-35188439 (-) |  | 0.5980 | 5.56e-06 | 4.70e-05 |
| ENSMUSG00000015143 | Actn1 | protein\_coding | 12:80167547-80260371 (-) |  | -0.7750 | 5.59e-06 | 4.72e-05 |
| ENSMUSG00000029394 | Cdk2ap1 | protein\_coding | 5:124345417-124363082 (-) |  | -0.2560 | 5.61e-06 | 4.73e-05 |
| ENSMUSG00000028798 | Eif3i | protein\_coding | 4:129591960-129600648 (-) |  | -0.2080 | 5.61e-06 | 4.73e-05 |
| ENSMUSG00000022234 | Cct5 | protein\_coding | 15:31590800-31601804 (-) |  | -0.2510 | 5.64e-06 | 4.75e-05 |
| ENSMUSG00000022488 | Nckap1l | protein\_coding | 15:103453794-103498810 (+) |  | 0.1830 | 5.68e-06 | 4.78e-05 |
| ENSMUSG00000036371 | Serbp1 | protein\_coding | 6:67238176-67297736 (+) |  | -0.2060 | 5.69e-06 | 4.78e-05 |
| ENSMUSG00000002102 | Psmc3 | protein\_coding | 2:91054009-91066369 (+) |  | -0.2470 | 5.69e-06 | 4.78e-05 |
| ENSMUSG00000005069 | Pex5 | protein\_coding | 6:124396816-124415067 (-) |  | 0.2090 | 5.69e-06 | 4.78e-05 |
| ENSMUSG00000047721 | Bola2 | protein\_coding | 7:126695401-126699798 (+) |  | -0.2550 | 5.69e-06 | 4.78e-05 |
| ENSMUSG00000001576 | Ergic1 | protein\_coding | 17:26561489-26656934 (+) |  | -0.3230 | 5.72e-06 | 4.80e-05 |
| ENSMUSG00000026393 | Nek7 | protein\_coding | 1:138482875-138620141 (-) |  | 0.2280 | 5.73e-06 | 4.81e-05 |
| ENSMUSG00000113769 | 5033406O09Rik | lncRNA | 12:111941991-111944484 (-) |  | 1.0100 | 5.78e-06 | 4.85e-05 |
| ENSMUSG00000040592 | Cd79b | protein\_coding | 11:106311341-106314762 (-) |  | 0.6210 | 5.80e-06 | 4.86e-05 |
| ENSMUSG00000041354 | Rgl2 | protein\_coding | 17:33929543-33937687 (+) |  | 0.2070 | 5.80e-06 | 4.86e-05 |
| ENSMUSG00000079019 | Insl3 | protein\_coding | 8:71689214-71690575 (+) |  | -1.5200 | 5.88e-06 | 4.92e-05 |
| ENSMUSG00000052776 | Oas1a | protein\_coding | 5:120896256-120907521 (-) |  | -0.6520 | 5.94e-06 | 4.97e-05 |
| ENSMUSG00000073988 | Ttpa | protein\_coding | 4:20007938-20030785 (+) |  | -0.6900 | 5.95e-06 | 4.98e-05 |
| ENSMUSG00000006732 | Mettl1 | protein\_coding | 10:127041414-127046365 (+) |  | -0.5290 | 5.97e-06 | 4.99e-05 |
| ENSMUSG00000034006 | Pqlc1 | protein\_coding | 18:80253292-80292725 (+) |  | 0.3210 | 6.01e-06 | 5.02e-05 |
| ENSMUSG00000021133 | Susd6 | protein\_coding | 12:80790510-80880835 (+) |  | -0.2150 | 6.03e-06 | 5.03e-05 |
| ENSMUSG00000041431 | Ccnb1 | protein\_coding | 13:100778650-100786570 (-) |  | -0.2500 | 6.09e-06 | 5.08e-05 |
| ENSMUSG00000035704 | Alg8 | protein\_coding | 7:97371606-97392185 (+) |  | -0.3650 | 6.17e-06 | 5.14e-05 |
| ENSMUSG00000005672 | Kit | protein\_coding | 5:75574916-75656722 (+) |  | 0.6820 | 6.18e-06 | 5.15e-05 |
| ENSMUSG00000027447 | Cst3 | protein\_coding | 2:148871722-148875692 (-) |  | 0.3430 | 6.25e-06 | 5.20e-05 |
| ENSMUSG00000083327 | Vcp-rs | processed\_pseudogene | X:104345829-104348234 (-) |  | -0.2980 | 6.29e-06 | 5.23e-05 |
| ENSMUSG00000026589 | Sec16b | protein\_coding | 1:157506728-157568425 (+) |  | 1.3500 | 6.29e-06 | 5.23e-05 |
| ENSMUSG00000051220 | Ercc6l | protein\_coding | X:102141716-102157091 (-) |  | -0.2770 | 6.33e-06 | 5.26e-05 |
| ENSMUSG00000026201 | Stk16 | protein\_coding | 1:75210838-75215606 (+) |  | -0.2120 | 6.40e-06 | 5.32e-05 |
| ENSMUSG00000062545 | Tlr12 | protein\_coding | 4:128615443-128618619 (-) |  | 1.0800 | 6.43e-06 | 5.34e-05 |
| ENSMUSG00000024862 | Klc2 | protein\_coding | 19:5107746-5118560 (-) |  | 0.4500 | 6.46e-06 | 5.36e-05 |
| ENSMUSG00000004980 | Hnrnpa2b1 | protein\_coding | 6:51460932-51469894 (-) |  | -0.2200 | 6.50e-06 | 5.40e-05 |
| ENSMUSG00000032422 | Snx14 | protein\_coding | 9:88376750-88438958 (-) |  | -0.3000 | 6.53e-06 | 5.42e-05 |
| ENSMUSG00000106099 | Gm42664 | lncRNA | 3:119743208-119746446 (-) |  | 0.6140 | 6.56e-06 | 5.43e-05 |
| ENSMUSG00000000876 | Pxmp4 | protein\_coding | 2:154585758-154603708 (-) |  | 0.2610 | 6.56e-06 | 5.43e-05 |
| ENSMUSG00000084106 | Gm6136 | processed\_pseudogene | 1:86908793-86909683 (-) |  | -0.2210 | 6.63e-06 | 5.49e-05 |
| ENSMUSG00000047592 | Nxpe5 | protein\_coding | 5:138225898-138253363 (+) |  | -0.5960 | 6.64e-06 | 5.49e-05 |
| ENSMUSG00000027522 | Stx16 | protein\_coding | 2:174076308-174099771 (+) |  | 0.3500 | 6.68e-06 | 5.52e-05 |
| ENSMUSG00000038383 | Pigu | protein\_coding | 2:155278243-155357430 (-) |  | -0.2830 | 6.70e-06 | 5.54e-05 |
| ENSMUSG00000003746 | Man1a | protein\_coding | 10:53904785-54076609 (-) |  | 0.3350 | 6.72e-06 | 5.55e-05 |
| ENSMUSG00000097779 | 4833407H14Rik | lncRNA | 19:53460622-53462723 (+) |  | 0.9480 | 6.76e-06 | 5.58e-05 |
| ENSMUSG00000071052 | Rpl7a-ps5 | processed\_pseudogene | 17:57838430-57839221 (-) |  | -0.5000 | 6.76e-06 | 5.58e-05 |
| ENSMUSG00000022515 | Anks3 | protein\_coding | 16:4941436-4964205 (-) |  | 0.3000 | 6.78e-06 | 5.59e-05 |
| ENSMUSG00000016382 | Pls3 | protein\_coding | X:75785654-75875182 (-) |  | -0.6310 | 6.83e-06 | 5.63e-05 |
| ENSMUSG00000058239 | Usf2 | protein\_coding | 7:30945248-30956803 (-) |  | 0.1920 | 6.84e-06 | 5.63e-05 |
| ENSMUSG00000071533 | Pcnp | protein\_coding | 16:56007245-56029739 (-) |  | -0.2800 | 7.04e-06 | 5.79e-05 |
| ENSMUSG00000054648 | Zfp869 | protein\_coding | 8:69702656-69716983 (-) |  | 0.2280 | 7.07e-06 | 5.81e-05 |
| ENSMUSG00000029571 | Tmem106b | protein\_coding | 6:13069759-13089269 (+) |  | 0.2990 | 7.08e-06 | 5.82e-05 |
| ENSMUSG00000021737 | Psmd6 | protein\_coding | 14:14112174-14120984 (-) |  | -0.2420 | 7.10e-06 | 5.84e-05 |
| ENSMUSG00000070738 | Dgkd | protein\_coding | 1:87853287-87945180 (+) |  | 0.2350 | 7.13e-06 | 5.86e-05 |
| ENSMUSG00000027018 | Hat1 | protein\_coding | 2:71388958-71441622 (+) |  | -0.3450 | 7.18e-06 | 5.89e-05 |
| ENSMUSG00000036944 | Tmem71 | protein\_coding | 15:66526212-66561103 (-) |  | 0.4070 | 7.23e-06 | 5.93e-05 |
| ENSMUSG00000036718 | Micall2 | protein\_coding | 5:139706696-139736336 (-) |  | 0.4230 | 7.25e-06 | 5.94e-05 |
| ENSMUSG00000031985 | Gnpat | protein\_coding | 8:124863033-124890057 (+) |  | -0.2410 | 7.26e-06 | 5.95e-05 |
| ENSMUSG00000021809 | Nudt13 | protein\_coding | 14:20294697-20317578 (+) |  | 0.3440 | 7.29e-06 | 5.97e-05 |
| ENSMUSG00000051391 | Ywhag | protein\_coding | 5:135908409-135934616 (-) |  | -0.1800 | 7.34e-06 | 6.00e-05 |
| ENSMUSG00000036908 | Unc93b1 | protein\_coding | 19:3935186-3949340 (+) |  | 0.2620 | 7.34e-06 | 6.01e-05 |
| ENSMUSG00000005204 | Senp3 | protein\_coding | 11:69673115-69682084 (-) |  | -0.2060 | 7.47e-06 | 6.11e-05 |
| ENSMUSG00000026622 | Nek2 | protein\_coding | 1:191821444-191833050 (+) |  | -0.2880 | 7.57e-06 | 6.18e-05 |
| ENSMUSG00000029366 | Dck | protein\_coding | 5:88764996-88783281 (+) |  | -0.2380 | 7.57e-06 | 6.18e-05 |
| ENSMUSG00000034652 | Cd300a | protein\_coding | 11:114890041-114904654 (+) |  | 0.3590 | 7.64e-06 | 6.24e-05 |
| ENSMUSG00000041132 | N4bp2l1 | protein\_coding | 5:150571644-150597188 (-) |  | 0.3450 | 7.65e-06 | 6.24e-05 |
| ENSMUSG00000003779 | Kif20a | protein\_coding | 18:34624613-34633277 (+) |  | -0.2480 | 7.68e-06 | 6.26e-05 |
| ENSMUSG00000029166 | Mapre3 | protein\_coding | 5:30814641-30866106 (+) |  | 1.5200 | 7.71e-06 | 6.28e-05 |
| ENSMUSG00000097636 | Mirt1 | lncRNA | 19:53443230-53464796 (-) |  | 0.6270 | 7.84e-06 | 6.38e-05 |
| ENSMUSG00000020340 | Cyfip2 | protein\_coding | 11:46193850-46312859 (-) |  | -0.4000 | 7.84e-06 | 6.38e-05 |
| ENSMUSG00000002957 | Ap2a2 | protein\_coding | 7:141562173-141633011 (+) |  | -0.2290 | 7.87e-06 | 6.40e-05 |
| ENSMUSG00000047617 | Paxx | protein\_coding | 2:25455141-25461094 (-) |  | 0.3740 | 7.95e-06 | 6.46e-05 |
| ENSMUSG00000021276 | Cinp | protein\_coding | 12:110872610-110889145 (-) |  | -0.2620 | 7.97e-06 | 6.47e-05 |
| ENSMUSG00000034484 | Snx2 | protein\_coding | 18:53176316-53220865 (+) |  | -0.2950 | 7.97e-06 | 6.47e-05 |
| ENSMUSG00000032477 | Cdc25a | protein\_coding | 9:109875579-109893895 (+) |  | -0.3520 | 7.98e-06 | 6.47e-05 |
| ENSMUSG00000035378 | Shq1 | protein\_coding | 6:100568256-100671157 (-) |  | -0.4230 | 7.98e-06 | 6.47e-05 |
| ENSMUSG00000021266 | Wars | protein\_coding | 12:108860030-108894174 (-) |  | -0.2690 | 8.03e-06 | 6.51e-05 |
| ENSMUSG00000102869 | 2900097C17Rik | lncRNA | 2:156388065-156392979 (-) |  | -0.2220 | 8.04e-06 | 6.51e-05 |
| ENSMUSG00000030793 | Pycard | protein\_coding | 7:127989708-127993867 (-) |  | -0.3940 | 8.05e-06 | 6.52e-05 |
| ENSMUSG00000029629 | Phf14 | protein\_coding | 6:11907809-12081205 (+) |  | 0.2160 | 8.05e-06 | 6.52e-05 |
| ENSMUSG00000058216 | Gstp3 | protein\_coding | 19:4057477-4059569 (-) |  | 1.7000 | 8.09e-06 | 6.54e-05 |
| ENSMUSG00000052456 | Asna1 | protein\_coding | 8:85017931-85025281 (-) |  | -0.2900 | 8.12e-06 | 6.57e-05 |
| ENSMUSG00000032860 | P2ry2 | protein\_coding | 7:100996568-101012866 (-) |  | -0.5580 | 8.13e-06 | 6.57e-05 |
| ENSMUSG00000004264 | Phb2 | protein\_coding | 6:124712336-124716950 (+) |  | -0.2410 | 8.13e-06 | 6.57e-05 |
| ENSMUSG00000031935 | Med17 | protein\_coding | 9:15260351-15279931 (-) |  | -0.2190 | 8.14e-06 | 6.57e-05 |
| ENSMUSG00000020307 | Cdc34 | protein\_coding | 10:79682195-79688398 (+) |  | -0.3540 | 8.15e-06 | 6.58e-05 |
| ENSMUSG00000004535 | Tax1bp1 | protein\_coding | 6:52713729-52766780 (+) |  | -0.2000 | 8.19e-06 | 6.61e-05 |
| ENSMUSG00000019302 | Atp6v0a1 | protein\_coding | 11:101009452-101063719 (+) |  | 0.4700 | 8.20e-06 | 6.61e-05 |
| ENSMUSG00000055067 | Smyd3 | protein\_coding | 1:178951960-179518041 (-) |  | -0.4980 | 8.22e-06 | 6.62e-05 |
| ENSMUSG00000045538 | Ddx28 | protein\_coding | 8:106009621-106011882 (-) |  | -0.3490 | 8.25e-06 | 6.64e-05 |
| ENSMUSG00000044068 | Zrsr1 | protein\_coding | 11:22972005-22976496 (+) |  | 0.4940 | 8.26e-06 | 6.64e-05 |
| ENSMUSG00000020576 | Nbas | protein\_coding | 12:13269133-13583811 (+) |  | -0.3670 | 8.26e-06 | 6.64e-05 |
| ENSMUSG00000059248 | Sept9 | protein\_coding | 11:117199661-117362325 (+) |  | 0.2250 | 8.31e-06 | 6.67e-05 |
| ENSMUSG00000113389 | Gm9512 | processed\_pseudogene | 13:15118632-15119128 (+) |  | 0.3910 | 8.33e-06 | 6.69e-05 |
| ENSMUSG00000020077 | Srgn | protein\_coding | 10:62493833-62527451 (-) |  | -0.4610 | 8.40e-06 | 6.74e-05 |
| ENSMUSG00000038388 | Mpp6 | protein\_coding | 6:50110241-50198939 (+) |  | -0.1930 | 8.41e-06 | 6.74e-05 |
| ENSMUSG00000028108 | Ecm1 | protein\_coding | 3:95734147-95739569 (-) |  | -0.7740 | 8.41e-06 | 6.74e-05 |
| ENSMUSG00000004849 | Ap1s1 | protein\_coding | 5:137034993-137046135 (-) |  | -0.3400 | 8.46e-06 | 6.78e-05 |
| ENSMUSG00000014867 | Surf4 | protein\_coding | 2:26920040-26933928 (-) |  | -0.1780 | 8.59e-06 | 6.88e-05 |
| ENSMUSG00000014226 | Cacybp | protein\_coding | 1:160202367-160212875 (-) |  | -0.4430 | 8.59e-06 | 6.88e-05 |
| ENSMUSG00000036452 | Arhgap26 | protein\_coding | 18:38993145-39376284 (+) |  | -0.3180 | 8.69e-06 | 6.95e-05 |
| ENSMUSG00000029722 | Agfg2 | protein\_coding | 5:137650463-137684726 (-) |  | 0.2620 | 8.82e-06 | 7.05e-05 |
| ENSMUSG00000039377 | Hlx | protein\_coding | 1:184727140-184732619 (-) |  | 0.2870 | 8.85e-06 | 7.07e-05 |
| ENSMUSG00000028803 | Nipal3 | protein\_coding | 4:135445420-135495038 (-) |  | 0.4380 | 8.89e-06 | 7.10e-05 |
| ENSMUSG00000032497 | Lrrfip2 | protein\_coding | 9:111117592-111225668 (+) |  | 0.2580 | 8.99e-06 | 7.17e-05 |
| ENSMUSG00000000399 | Ndufa9 | protein\_coding | 6:126821721-126849136 (-) |  | -0.2310 | 9.02e-06 | 7.20e-05 |
| ENSMUSG00000058470 | Gm8369 | protein\_coding | 19:11485938-11512577 (+) |  | 0.6010 | 9.09e-06 | 7.25e-05 |
| ENSMUSG00000016528 | Mapkapk2 | protein\_coding | 1:131053700-131097826 (-) |  | -0.1750 | 9.14e-06 | 7.28e-05 |
| ENSMUSG00000017639 | Rab11fip4 | protein\_coding | 11:79591212-79698023 (+) |  | 0.4350 | 9.18e-06 | 7.31e-05 |
| ENSMUSG00000034557 | Zfyve9 | protein\_coding | 4:108637466-108780798 (-) |  | 0.5240 | 9.22e-06 | 7.34e-05 |
| ENSMUSG00000030720 | Cln3 | protein\_coding | 7:126571207-126585817 (-) |  | 0.2340 | 9.25e-06 | 7.36e-05 |
| ENSMUSG00000033781 | Asb13 | protein\_coding | 13:3634032-3653822 (+) |  | 0.4450 | 9.28e-06 | 7.38e-05 |
| ENSMUSG00000035629 | Rubcn | protein\_coding | 16:32821703-32877766 (-) |  | 0.2660 | 9.30e-06 | 7.39e-05 |
| ENSMUSG00000018796 | Acsl1 | protein\_coding | 8:46471037-46536051 (+) |  | 0.2210 | 9.34e-06 | 7.42e-05 |
| ENSMUSG00000053470 | Kdm3a | protein\_coding | 6:71588972-71632990 (-) |  | 0.3050 | 9.35e-06 | 7.42e-05 |
| ENSMUSG00000032216 | Nedd4 | protein\_coding | 9:72662346-72749852 (+) |  | 1.5100 | 9.40e-06 | 7.46e-05 |
| ENSMUSG00000032199 | Polr2m | protein\_coding | 9:71478437-71485935 (-) |  | -0.2020 | 9.42e-06 | 7.47e-05 |
| ENSMUSG00000036285 | Noa1 | protein\_coding | 5:77294182-77310084 (-) |  | -0.2810 | 9.46e-06 | 7.49e-05 |
| ENSMUSG00000046223 | Plaur | protein\_coding | 7:24462484-24475968 (+) |  | 0.2990 | 9.48e-06 | 7.51e-05 |
| ENSMUSG00000013160 | Atp6v0d1 | protein\_coding | 8:105524465-105566047 (-) |  | -0.2080 | 9.55e-06 | 7.56e-05 |
| ENSMUSG00000031353 | Rbbp7 | protein\_coding | X:162760402-162779092 (+) |  | -0.2180 | 9.56e-06 | 7.57e-05 |
| ENSMUSG00000062093 | Gm10110 | transcribed\_processed\_pseudogene | 14:89896228-89899447 (-) |  | -0.5750 | 9.57e-06 | 7.57e-05 |
| ENSMUSG00000038545 | Cul7 | protein\_coding | 17:46650337-46664364 (+) |  | 0.6210 | 9.64e-06 | 7.61e-05 |
| ENSMUSG00000056724 | Nbeal2 | protein\_coding | 9:110624789-110654161 (-) |  | 0.3550 | 9.64e-06 | 7.62e-05 |
| ENSMUSG00000087412 | Gm15501 | transcribed\_processed\_pseudogene | 7:93178983-93184176 (-) |  | -0.4130 | 9.65e-06 | 7.62e-05 |
| ENSMUSG00000023118 | Sympk | protein\_coding | 7:19024377-19054618 (+) |  | -0.2370 | 9.66e-06 | 7.62e-05 |
| ENSMUSG00000042079 | Hnrnpf | protein\_coding | 6:117900324-117925622 (+) |  | -0.2350 | 9.78e-06 | 7.71e-05 |
| ENSMUSG00000022544 | Eef2kmt | protein\_coding | 16:5244152-5255983 (-) |  | -0.2780 | 9.79e-06 | 7.71e-05 |
| ENSMUSG00000017144 | Rnd3 | protein\_coding | 2:51130438-51149111 (-) |  | 0.4000 | 9.87e-06 | 7.78e-05 |
| ENSMUSG00000022221 | Ripk3 | protein\_coding | 14:55784995-55788865 (-) |  | -0.3590 | 9.89e-06 | 7.79e-05 |
| ENSMUSG00000027935 | Rab13 | protein\_coding | 3:90213695-90226385 (+) |  | -1.0900 | 9.93e-06 | 7.81e-05 |
| ENSMUSG00000030761 | Myo7a | protein\_coding | 7:98051060-98119524 (-) |  | 0.3440 | 9.94e-06 | 7.81e-05 |
| ENSMUSG00000011008 | Mcoln2 | protein\_coding | 3:146149833-146195513 (+) |  | -1.2300 | 9.98e-06 | 7.85e-05 |
| ENSMUSG00000030726 | Pold3 | protein\_coding | 7:100082111-100121565 (-) |  | -0.2360 | 1.00e-05 | 7.88e-05 |
| ENSMUSG00000031622 | Sin3b | protein\_coding | 8:72723285-72758201 (+) |  | 0.2060 | 1.01e-05 | 7.89e-05 |
| ENSMUSG00000029016 | Clcn6 | protein\_coding | 4:148004259-148038821 (-) |  | 0.3540 | 1.01e-05 | 7.90e-05 |
| ENSMUSG00000054931 | Zkscan4 | protein\_coding | 13:21478907-21485507 (+) |  | 1.7600 | 1.01e-05 | 7.92e-05 |
| ENSMUSG00000024665 | Fads2 | protein\_coding | 19:10062765-10101746 (-) |  | -1.2200 | 1.01e-05 | 7.92e-05 |
| ENSMUSG00000053398 | Phgdh | protein\_coding | 3:98313170-98339990 (-) |  | -0.7650 | 1.01e-05 | 7.94e-05 |
| ENSMUSG00000089762 | Ier5l | protein\_coding | 2:30471537-30474219 (-) |  | 1.0300 | 1.03e-05 | 8.03e-05 |
| ENSMUSG00000040648 | Ppip5k2 | protein\_coding | 1:97706048-97770411 (-) |  | -0.2000 | 1.03e-05 | 8.04e-05 |
| ENSMUSG00000061474 | Mrps36 | protein\_coding | 13:100735955-100744659 (-) |  | -0.4070 | 1.03e-05 | 8.05e-05 |
| ENSMUSG00000039748 | Exo1 | protein\_coding | 1:175880581-175913489 (+) |  | -0.4160 | 1.03e-05 | 8.08e-05 |
| ENSMUSG00000081405 | Gm13142 | processed\_pseudogene | 4:147102327-147103981 (+) |  | 0.7710 | 1.04e-05 | 8.09e-05 |
| ENSMUSG00000040731 | Eif4h | protein\_coding | 5:134619721-134639490 (-) |  | -0.1970 | 1.04e-05 | 8.14e-05 |
| ENSMUSG00000028104 | Polr3gl | protein\_coding | 3:96577872-96594181 (-) |  | 0.4100 | 1.05e-05 | 8.17e-05 |
| ENSMUSG00000074698 | Csnk2a1 | protein\_coding | 2:152226839-152281852 (+) |  | -0.2000 | 1.06e-05 | 8.23e-05 |
| ENSMUSG00000029486 | Mrpl1 | protein\_coding | 5:96209493-96266727 (+) |  | -0.3050 | 1.06e-05 | 8.23e-05 |
| ENSMUSG00000058728 | Cd300c | protein\_coding | 11:114956116-114969157 (-) |  | 1.1900 | 1.06e-05 | 8.26e-05 |
| ENSMUSG00000092086 | Gm6793 | processed\_pseudogene | 8:112013953-112015008 (-) |  | -0.2840 | 1.09e-05 | 8.50e-05 |
| ENSMUSG00000025571 | Tnrc6c | protein\_coding | 11:117654289-117763439 (+) |  | 0.2310 | 1.09e-05 | 8.52e-05 |
| ENSMUSG00000083899 | Gm12346 | transcribed\_processed\_pseudogene | 11:77293444-77295898 (+) |  | -0.5020 | 1.10e-05 | 8.53e-05 |
| ENSMUSG00000055541 | Lair1 | protein\_coding | 7:4003402-4063204 (-) |  | -0.5780 | 1.11e-05 | 8.62e-05 |
| ENSMUSG00000039041 | Adrm1 | protein\_coding | 2:180171485-180176286 (+) |  | -0.2510 | 1.12e-05 | 8.72e-05 |
| ENSMUSG00000032666 | 1700025G04Rik | protein\_coding | 1:151852403-152090125 (-) |  | -0.2560 | 1.12e-05 | 8.73e-05 |
| ENSMUSG00000081137 | BC022960 | transcribed\_processed\_pseudogene | X:169368189-169370003 (+) |  | 0.7130 | 1.13e-05 | 8.74e-05 |
| ENSMUSG00000051355 | Commd1 | protein\_coding | 11:22896136-22982382 (-) |  | -0.3200 | 1.13e-05 | 8.76e-05 |
| ENSMUSG00000031697 | Orc6 | protein\_coding | 8:85299632-85308278 (+) |  | -0.2830 | 1.13e-05 | 8.78e-05 |
| ENSMUSG00000033209 | Ttc28 | protein\_coding | 5:110879803-111289780 (+) |  | 0.7870 | 1.14e-05 | 8.84e-05 |
| ENSMUSG00000020330 | Hmmr | protein\_coding | 11:40701395-40733422 (-) |  | -0.3280 | 1.15e-05 | 8.88e-05 |
| ENSMUSG00000004268 | Emg1 | protein\_coding | 6:124704085-124712178 (-) |  | -0.2660 | 1.16e-05 | 8.98e-05 |
| ENSMUSG00000073910 | Mob3b | protein\_coding | 4:34949074-35157484 (-) |  | 0.3510 | 1.16e-05 | 8.99e-05 |
| ENSMUSG00000019066 | Rab3d | protein\_coding | 9:21907491-21918192 (-) |  | 0.2300 | 1.17e-05 | 9.02e-05 |
| ENSMUSG00000013622 | Atraid | protein\_coding | 5:31048312-31054623 (+) |  | 0.2860 | 1.17e-05 | 9.08e-05 |
| ENSMUSG00000061360 | Phf5a | protein\_coding | 15:81864520-81871911 (-) |  | -0.2540 | 1.18e-05 | 9.12e-05 |
| ENSMUSG00000101389 | Ms4a4a | protein\_coding | 19:11375523-11392790 (+) |  | -1.0500 | 1.19e-05 | 9.23e-05 |
| ENSMUSG00000004668 | Abca13 | protein\_coding | 11:9191942-9684259 (+) |  | -1.2100 | 1.20e-05 | 9.26e-05 |
| ENSMUSG00000041426 | Hibch | protein\_coding | 1:52844929-52920986 (+) |  | -0.2840 | 1.20e-05 | 9.26e-05 |
| ENSMUSG00000022982 | Sod1 | protein\_coding | 16:90220754-90226329 (+) |  | 0.2060 | 1.28e-05 | 9.84e-05 |
| ENSMUSG00000024566 | Atp9b | protein\_coding | 18:80734141-80934060 (-) |  | 0.2480 | 1.28e-05 | 9.84e-05 |
| ENSMUSG00000000555 | Itga5 | protein\_coding | 15:103344286-103366763 (-) |  | -0.3040 | 1.29e-05 | 9.96e-05 |
| ENSMUSG00000029752 | Asns | protein\_coding | 6:7675169-7693254 (-) |  | -1.3700 | 1.30e-05 | 9.98e-05 |
| ENSMUSG00000106743 | Gm42847 | TEC | 5:33990510-33993062 (-) |  | 1.1100 | 1.30e-05 | 9.99e-05 |
| ENSMUSG00000031109 | Enox2 | protein\_coding | X:49009707-49288259 (-) |  | 0.3030 | 1.30e-05 | 1.00e-04 |
| ENSMUSG00000040451 | Sgms1 | protein\_coding | 19:32122727-32389714 (-) |  | 0.3290 | 1.31e-05 | 1.00e-04 |
| ENSMUSG00000018900 | Slc22a5 | protein\_coding | 11:53864542-53891660 (-) |  | 0.3530 | 1.31e-05 | 1.01e-04 |
| ENSMUSG00000032046 | Abhd12 | protein\_coding | 2:150832493-150904741 (-) |  | 0.2250 | 1.32e-05 | 1.01e-04 |
| ENSMUSG00000030269 | Mtmr14 | protein\_coding | 6:113237843-113281392 (+) |  | 0.3290 | 1.32e-05 | 1.02e-04 |
| ENSMUSG00000020899 | Pfas | protein\_coding | 11:68985697-69008460 (-) |  | -0.3910 | 1.34e-05 | 1.03e-04 |
| ENSMUSG00000079139 | Gm4204 | processed\_pseudogene | 1:135231995-135233225 (+) |  | -0.2100 | 1.34e-05 | 1.03e-04 |
| ENSMUSG00000063694 | Cycs | protein\_coding | 6:50562563-50566538 (-) |  | -0.4020 | 1.35e-05 | 1.03e-04 |
| ENSMUSG00000026771 | Spopl | protein\_coding | 2:23506220-23572106 (-) |  | 0.3020 | 1.36e-05 | 1.04e-04 |
| ENSMUSG00000029635 | Cdk8 | protein\_coding | 5:146231230-146302874 (+) |  | -0.2550 | 1.36e-05 | 1.04e-04 |
| ENSMUSG00000063802 | Hspbp1 | protein\_coding | 7:4660521-4685068 (-) |  | -0.3620 | 1.36e-05 | 1.04e-04 |
| ENSMUSG00000025971 | Maip1 | protein\_coding | 1:57406328-57417953 (+) |  | -0.4010 | 1.36e-05 | 1.04e-04 |
| ENSMUSG00000027947 | Il6ra | protein\_coding | 3:89864059-89913196 (-) |  | 0.2130 | 1.38e-05 | 1.05e-04 |
| ENSMUSG00000030727 | Rabep2 | protein\_coding | 7:126428759-126449245 (+) |  | 0.2980 | 1.38e-05 | 1.06e-04 |
| ENSMUSG00000030165 | Klrd1 | protein\_coding | 6:129591782-129598775 (+) |  | 1.2900 | 1.39e-05 | 1.06e-04 |
| ENSMUSG00000003809 | Gcdh | protein\_coding | 8:84886393-84893921 (-) |  | -0.2960 | 1.40e-05 | 1.07e-04 |
| ENSMUSG00000027508 | Pag1 | protein\_coding | 3:9687479-9833679 (-) |  | -0.3360 | 1.40e-05 | 1.07e-04 |
| ENSMUSG00000029650 | Slc46a3 | protein\_coding | 5:147878437-147894815 (-) |  | 1.0400 | 1.41e-05 | 1.07e-04 |
| ENSMUSG00000023944 | Hsp90ab1 | protein\_coding | 17:45567775-45573271 (-) |  | -0.3670 | 1.41e-05 | 1.07e-04 |
| ENSMUSG00000104835 | Gm5547 | lncRNA | 3:105815540-105817357 (+) |  | 1.1100 | 1.41e-05 | 1.08e-04 |
| ENSMUSG00000105987 | AI506816 | lncRNA | 5:23698296-23712667 (-) |  | -0.8100 | 1.42e-05 | 1.08e-04 |
| ENSMUSG00000000976 | Heatr6 | protein\_coding | 11:83753696-83783754 (+) |  | -0.2810 | 1.42e-05 | 1.08e-04 |
| ENSMUSG00000066258 | Trim12a | protein\_coding | 7:104299894-104315466 (-) |  | 0.2770 | 1.43e-05 | 1.08e-04 |
| ENSMUSG00000005312 | Ubqln1 | protein\_coding | 13:58176156-58215653 (-) |  | -0.1870 | 1.43e-05 | 1.08e-04 |
| ENSMUSG00000030225 | Dera | protein\_coding | 6:137754546-137857340 (+) |  | -0.2480 | 1.43e-05 | 1.09e-04 |
| ENSMUSG00000030041 | M1ap | protein\_coding | 6:82946902-83030309 (+) |  | 0.5700 | 1.44e-05 | 1.09e-04 |
| ENSMUSG00000109118 | Gm32031 | lncRNA | 7:47008083-47009008 (+) |  | 0.9410 | 1.44e-05 | 1.09e-04 |
| ENSMUSG00000021079 | Timm9 | protein\_coding | 12:71123173-71136684 (-) |  | -0.4150 | 1.45e-05 | 1.10e-04 |
| ENSMUSG00000064037 | Gpn1 | protein\_coding | 5:31494741-31512904 (+) |  | -0.2820 | 1.46e-05 | 1.11e-04 |
| ENSMUSG00000040785 | Ttc3 | protein\_coding | 16:94370618-94469343 (+) |  | 0.3840 | 1.47e-05 | 1.11e-04 |
| ENSMUSG00000069833 | Ahnak | protein\_coding | 19:8989284-9076914 (+) |  | 0.4390 | 1.49e-05 | 1.13e-04 |
| ENSMUSG00000042099 | Kank3 | protein\_coding | 17:33810519-33822918 (+) |  | 0.3890 | 1.50e-05 | 1.14e-04 |
| ENSMUSG00000054733 | Msra | protein\_coding | 14:64122625-64455903 (-) |  | 0.2820 | 1.51e-05 | 1.14e-04 |
| ENSMUSG00000027422 | Rrbp1 | protein\_coding | 2:143947395-144011263 (-) |  | -0.2070 | 1.52e-05 | 1.15e-04 |
| ENSMUSG00000002948 | Map2k7 | protein\_coding | 8:4238740-4247897 (+) |  | 0.2940 | 1.54e-05 | 1.16e-04 |
| ENSMUSG00000058569 | Tmed9 | protein\_coding | 13:55573732-55597697 (+) |  | -0.1870 | 1.55e-05 | 1.17e-04 |
| ENSMUSG00000054752 | Fsd1l | protein\_coding | 4:53631471-53707009 (+) |  | -0.5510 | 1.57e-05 | 1.18e-04 |
| ENSMUSG00000004864 | Mapk13 | protein\_coding | 17:28769297-28780233 (+) |  | -0.5650 | 1.57e-05 | 1.18e-04 |
| ENSMUSG00000041598 | Cdc42ep4 | protein\_coding | 11:113726850-113751881 (-) |  | 0.3810 | 1.57e-05 | 1.18e-04 |
| ENSMUSG00000026193 | Fn1 | protein\_coding | 1:71585520-71653200 (-) |  | 0.7160 | 1.58e-05 | 1.19e-04 |
| ENSMUSG00000058318 | Phf21a | protein\_coding | 2:92093117-92364666 (+) |  | 0.3120 | 1.59e-05 | 1.20e-04 |
| ENSMUSG00000051457 | Spn | protein\_coding | 7:127132232-127137823 (-) |  | 0.2120 | 1.59e-05 | 1.20e-04 |
| ENSMUSG00000031443 | F7 | protein\_coding | 8:13026034-13035809 (+) |  | -0.7030 | 1.60e-05 | 1.20e-04 |
| ENSMUSG00000032561 | Acpp | protein\_coding | 9:104288251-104337748 (-) |  | -0.8330 | 1.61e-05 | 1.21e-04 |
| ENSMUSG00000040720 | Virma | protein\_coding | 4:11485958-11550684 (+) |  | -0.1700 | 1.63e-05 | 1.22e-04 |
| ENSMUSG00000039361 | Picalm | protein\_coding | 7:90130213-90213465 (+) |  | -0.3400 | 1.63e-05 | 1.22e-04 |
| ENSMUSG00000028403 | Zdhhc21 | protein\_coding | 4:82798738-82859958 (-) |  | -0.2100 | 1.63e-05 | 1.23e-04 |
| ENSMUSG00000032373 | Car12 | protein\_coding | 9:66713686-66766845 (+) |  | -1.2700 | 1.64e-05 | 1.23e-04 |
| ENSMUSG00000024055 | Cyp4f13 | protein\_coding | 17:32924688-32947402 (-) |  | 0.4380 | 1.64e-05 | 1.23e-04 |
| ENSMUSG00000036223 | Ska1 | protein\_coding | 18:74195299-74207818 (-) |  | -0.3930 | 1.65e-05 | 1.24e-04 |
| ENSMUSG00000019883 | Echdc1 | protein\_coding | 10:29313166-29347469 (+) |  | -0.6470 | 1.66e-05 | 1.24e-04 |
| ENSMUSG00000001062 | Vps9d1 | protein\_coding | 8:123242356-123254348 (-) |  | 0.3500 | 1.66e-05 | 1.24e-04 |
| ENSMUSG00000022757 | Tfg | protein\_coding | 16:56690332-56717450 (-) |  | -0.2520 | 1.66e-05 | 1.24e-04 |
| ENSMUSG00000036768 | Kif15 | protein\_coding | 9:122951046-123018733 (+) |  | -0.2970 | 1.67e-05 | 1.25e-04 |
| ENSMUSG00000049502 | Dtx3l | protein\_coding | 16:35926511-35939151 (-) |  | -0.2600 | 1.67e-05 | 1.25e-04 |
| ENSMUSG00000022788 | Fgd4 | protein\_coding | 16:16416917-16600549 (-) |  | 0.3340 | 1.68e-05 | 1.25e-04 |
| ENSMUSG00000026669 | Mcm10 | protein\_coding | 2:4989714-5012791 (-) |  | -0.3490 | 1.69e-05 | 1.26e-04 |
| ENSMUSG00000030474 | Siglece | protein\_coding | 7:43651070-43660161 (-) |  | -0.2870 | 1.70e-05 | 1.27e-04 |
| ENSMUSG00000041642 | Kif21b | protein\_coding | 1:136131389-136177998 (+) |  | 0.3590 | 1.70e-05 | 1.27e-04 |
| ENSMUSG00000003226 | Ranbp2 | protein\_coding | 10:58446920-58494356 (+) |  | -0.2020 | 1.71e-05 | 1.28e-04 |
| ENSMUSG00000033356 | Pus7l | protein\_coding | 15:94522688-94543547 (-) |  | -0.3260 | 1.72e-05 | 1.28e-04 |
| ENSMUSG00000070709 | Zfp974 | protein\_coding | 7:27907392-27929460 (-) |  | 0.6170 | 1.72e-05 | 1.28e-04 |
| ENSMUSG00000020623 | Map2k6 | protein\_coding | 11:110399122-110525522 (+) |  | -0.3210 | 1.73e-05 | 1.29e-04 |
| ENSMUSG00000024137 | E4f1 | protein\_coding | 17:24443778-24470313 (-) |  | 0.2910 | 1.73e-05 | 1.29e-04 |
| ENSMUSG00000024431 | Nr3c1 | protein\_coding | 18:39410545-39519421 (-) |  | 0.1760 | 1.73e-05 | 1.29e-04 |
| ENSMUSG00000022051 | Bnip3l | protein\_coding | 14:66985239-67008877 (-) |  | 0.2630 | 1.74e-05 | 1.30e-04 |
| ENSMUSG00000025786 | Zdhhc3 | protein\_coding | 9:123066160-123113205 (-) |  | -0.2000 | 1.75e-05 | 1.30e-04 |
| ENSMUSG00000045948 | Mrps12 | protein\_coding | 7:28739641-28741820 (-) |  | -0.3460 | 1.76e-05 | 1.30e-04 |
| ENSMUSG00000035722 | Abca7 | protein\_coding | 10:79996494-80015572 (+) |  | 0.2350 | 1.76e-05 | 1.31e-04 |
| ENSMUSG00000068184 | Ndufaf2 | protein\_coding | 13:108002715-108158623 (-) |  | -0.4120 | 1.77e-05 | 1.31e-04 |
| ENSMUSG00000021451 | Sema4d | protein\_coding | 13:51685529-51793747 (-) |  | 0.3650 | 1.77e-05 | 1.31e-04 |
| ENSMUSG00000099875 | Rbm3-ps | processed\_pseudogene | 1:150265535-150265999 (-) |  | 0.3690 | 1.77e-05 | 1.31e-04 |
| ENSMUSG00000041840 | Haus1 | protein\_coding | 18:77757567-77773886 (-) |  | -0.4050 | 1.77e-05 | 1.31e-04 |
| ENSMUSG00000051335 | Gfod1 | protein\_coding | 13:43195245-43304172 (-) |  | 0.3580 | 1.78e-05 | 1.32e-04 |
| ENSMUSG00000032596 | Uba7 | protein\_coding | 9:107975505-107984060 (+) |  | 0.3310 | 1.80e-05 | 1.33e-04 |
| ENSMUSG00000042410 | Agps | protein\_coding | 2:75832177-75931350 (+) |  | -0.1860 | 1.81e-05 | 1.34e-04 |
| ENSMUSG00000090100 | Ttbk2 | protein\_coding | 2:120732816-120850604 (-) |  | 0.3950 | 1.81e-05 | 1.34e-04 |
| ENSMUSG00000029538 | Srsf9 | protein\_coding | 5:115327177-115333080 (+) |  | -0.1840 | 1.81e-05 | 1.34e-04 |
| ENSMUSG00000029198 | Grpel1 | protein\_coding | 5:36464998-36474080 (+) |  | -0.2690 | 1.81e-05 | 1.34e-04 |
| ENSMUSG00000020647 | Ncoa1 | protein\_coding | 12:4247362-4477182 (-) |  | 0.3060 | 1.82e-05 | 1.34e-04 |
| ENSMUSG00000026223 | Itm2c | protein\_coding | 1:85894281-85908675 (+) |  | 0.2630 | 1.82e-05 | 1.34e-04 |
| ENSMUSG00000026895 | Ndufa8 | protein\_coding | 2:36036326-36049406 (-) |  | -0.2620 | 1.82e-05 | 1.34e-04 |
| ENSMUSG00000032425 | Zfp949 | protein\_coding | 9:88548020-88571061 (+) |  | 0.5090 | 1.83e-05 | 1.35e-04 |
| ENSMUSG00000025494 | Sigirr | protein\_coding | 7:141091175-141100572 (-) |  | 0.3800 | 1.83e-05 | 1.35e-04 |
| ENSMUSG00000036202 | Rif1 | protein\_coding | 2:52072832-52122383 (+) |  | -0.2110 | 1.84e-05 | 1.36e-04 |
| ENSMUSG00000026767 | Mindy3 | protein\_coding | 2:12347263-12419470 (-) |  | -0.2500 | 1.85e-05 | 1.36e-04 |
| ENSMUSG00000070354 | Evi2 | protein\_coding | 11:79513385-79530589 (-) |  | 0.4220 | 1.85e-05 | 1.36e-04 |
| ENSMUSG00000041859 | Mcm3 | protein\_coding | 1:20802968-20820312 (-) |  | -0.3660 | 1.88e-05 | 1.38e-04 |
| ENSMUSG00000020608 | Smc6 | protein\_coding | 12:11265886-11319785 (+) |  | -0.2110 | 1.88e-05 | 1.38e-04 |
| ENSMUSG00000063317 | Usp31 | protein\_coding | 7:121642021-121707253 (-) |  | -0.2910 | 1.90e-05 | 1.39e-04 |
| ENSMUSG00000009470 | Tnpo1 | protein\_coding | 13:98839019-98926384 (-) |  | -0.2350 | 1.91e-05 | 1.40e-04 |
| ENSMUSG00000003623 | Crot | protein\_coding | 5:8966033-8997324 (-) |  | 0.2920 | 1.92e-05 | 1.41e-04 |
| ENSMUSG00000047767 | Atg16l2 | protein\_coding | 7:101263034-101302251 (-) |  | 0.3680 | 1.92e-05 | 1.41e-04 |
| ENSMUSG00000052435 | Cebpe | protein\_coding | 14:54710360-54712174 (-) |  | 0.4380 | 1.93e-05 | 1.41e-04 |
| ENSMUSG00000048249 | Crebrf | protein\_coding | 17:26715650-26776635 (+) |  | 0.4180 | 1.93e-05 | 1.41e-04 |
| ENSMUSG00000033732 | Sf3b3 | protein\_coding | 8:110810239-110846787 (-) |  | -0.3040 | 1.93e-05 | 1.42e-04 |
| ENSMUSG00000040209 | Zfp704 | protein\_coding | 3:9427020-9610085 (-) |  | 0.3690 | 1.94e-05 | 1.42e-04 |
| ENSMUSG00000007613 | Tgfbr1 | protein\_coding | 4:47353222-47414931 (+) |  | 0.2270 | 1.94e-05 | 1.42e-04 |
| ENSMUSG00000028822 | Tmem50a | protein\_coding | 4:134897849-134915024 (-) |  | 0.2230 | 1.97e-05 | 1.44e-04 |
| ENSMUSG00000038967 | Pdk2 | protein\_coding | 11:95026258-95041354 (-) |  | 0.8600 | 1.97e-05 | 1.44e-04 |
| ENSMUSG00000086425 | F730016J06Rik | lncRNA | 2:28095477-28127731 (+) |  | 0.6920 | 1.97e-05 | 1.44e-04 |
| ENSMUSG00000031897 | Psmb10 | protein\_coding | 8:105935735-105938444 (-) |  | -0.2400 | 1.97e-05 | 1.44e-04 |
| ENSMUSG00000090602 | Gm5611 | processed\_pseudogene | 9:17030045-17030896 (+) |  | -0.4950 | 2.00e-05 | 1.46e-04 |
| ENSMUSG00000018677 | Slc25a39 | protein\_coding | 11:102402985-102407946 (-) |  | -0.2230 | 2.01e-05 | 1.47e-04 |
| ENSMUSG00000022246 | Rai14 | protein\_coding | 15:10568969-10714624 (-) |  | -0.4950 | 2.02e-05 | 1.47e-04 |
| ENSMUSG00000042766 | Trim46 | protein\_coding | 3:89234177-89246309 (-) |  | -0.8980 | 2.04e-05 | 1.48e-04 |
| ENSMUSG00000029209 | Gnpda2 | protein\_coding | 5:69573108-69592340 (-) |  | 0.4860 | 2.05e-05 | 1.49e-04 |
| ENSMUSG00000021807 | Rtraf | protein\_coding | 14:19811351-19823824 (-) |  | -0.3050 | 2.06e-05 | 1.50e-04 |
| ENSMUSG00000097194 | 9330175E14Rik | lncRNA | 8:94422898-94435103 (-) |  | -0.7130 | 2.06e-05 | 1.50e-04 |
| ENSMUSG00000090394 | 4930523C07Rik | protein\_coding | 1:160044382-160080208 (+) |  | 0.2390 | 2.07e-05 | 1.50e-04 |
| ENSMUSG00000030609 | Aen | protein\_coding | 7:78895854-78911209 (+) |  | -0.3180 | 2.08e-05 | 1.51e-04 |
| ENSMUSG00000028494 | Plin2 | protein\_coding | 4:86648386-86670060 (-) |  | -0.4960 | 2.08e-05 | 1.51e-04 |
| ENSMUSG00000046027 | Stard5 | protein\_coding | 7:83631959-83653127 (+) |  | -0.2840 | 2.10e-05 | 1.52e-04 |
| ENSMUSG00000005986 | Ankrd13d | protein\_coding | 19:4270180-4283137 (-) |  | 0.4110 | 2.10e-05 | 1.52e-04 |
| ENSMUSG00000038893 | Fam117a | protein\_coding | 11:95337018-95381872 (+) |  | 0.2370 | 2.10e-05 | 1.52e-04 |
| ENSMUSG00000030703 | Gdpd3 | protein\_coding | 7:126766334-126775649 (+) |  | 0.5720 | 2.11e-05 | 1.53e-04 |
| ENSMUSG00000018666 | Cbx1 | protein\_coding | 11:96789127-96808640 (+) |  | -0.1850 | 2.12e-05 | 1.54e-04 |
| ENSMUSG00000025578 | Cbx8 | protein\_coding | 11:119036305-119040969 (-) |  | 0.2800 | 2.14e-05 | 1.55e-04 |
| ENSMUSG00000063406 | Tmed5 | protein\_coding | 5:108106366-108132620 (-) |  | -0.3200 | 2.16e-05 | 1.57e-04 |
| ENSMUSG00000019797 | 1700021F05Rik | protein\_coding | 10:43525133-43540994 (-) |  | -0.3320 | 2.17e-05 | 1.57e-04 |
| ENSMUSG00000105632 | Gm43272 | TEC | 5:101810492-101812075 (+) |  | 1.1500 | 2.18e-05 | 1.58e-04 |
| ENSMUSG00000034610 | Tut4 | protein\_coding | 4:108459426-108559421 (+) |  | 0.2920 | 2.20e-05 | 1.59e-04 |
| ENSMUSG00000039813 | Tbc1d2 | protein\_coding | 4:46604390-46650209 (-) |  | 0.3000 | 2.22e-05 | 1.60e-04 |
| ENSMUSG00000041890 | Git2 | protein\_coding | 5:114727407-114775517 (-) |  | 0.2070 | 2.22e-05 | 1.60e-04 |
| ENSMUSG00000025037 | Maoa | protein\_coding | X:16619698-16687818 (+) |  | -0.4900 | 2.23e-05 | 1.61e-04 |
| ENSMUSG00000021591 | Glrx | protein\_coding | 13:75839868-75850154 (+) |  | -0.3460 | 2.23e-05 | 1.61e-04 |
| ENSMUSG00000020798 | Spns3 | protein\_coding | 11:72494919-72550506 (-) |  | 0.5530 | 2.23e-05 | 1.61e-04 |
| ENSMUSG00000015702 | Anxa9 | protein\_coding | 3:95296096-95307176 (-) |  | -0.5250 | 2.24e-05 | 1.61e-04 |
| ENSMUSG00000029064 | Gnb1 | protein\_coding | 4:155491361-155559269 (+) |  | -0.1750 | 2.24e-05 | 1.61e-04 |
| ENSMUSG00000031776 | Arl2bp | protein\_coding | 8:94666600-94674425 (+) |  | 0.2330 | 2.24e-05 | 1.62e-04 |
| ENSMUSG00000040236 | Trappc5 | protein\_coding | 8:3676299-3681255 (+) |  | 0.2730 | 2.26e-05 | 1.62e-04 |
| ENSMUSG00000017837 | Nkiras2 | protein\_coding | 11:100619244-100627607 (+) |  | 0.2390 | 2.26e-05 | 1.62e-04 |
| ENSMUSG00000051839 | Gypa | protein\_coding | 8:80493781-80510542 (+) |  | 1.8200 | 2.27e-05 | 1.63e-04 |
| ENSMUSG00000022881 | Rfc4 | protein\_coding | 16:23113943-23127737 (-) |  | -0.3350 | 2.28e-05 | 1.64e-04 |
| ENSMUSG00000098112 | Bin2 | protein\_coding | 15:100641077-100669553 (-) |  | -0.2210 | 2.28e-05 | 1.64e-04 |
| ENSMUSG00000031967 | Afg3l1 | protein\_coding | 8:123477903-123503916 (+) |  | -0.2510 | 2.30e-05 | 1.65e-04 |
| ENSMUSG00000061046 | Haghl | protein\_coding | 17:25779843-25785673 (-) |  | 0.3510 | 2.30e-05 | 1.65e-04 |
| ENSMUSG00000041645 | Ddx24 | protein\_coding | 12:103407982-103425830 (-) |  | -0.1640 | 2.30e-05 | 1.65e-04 |
| ENSMUSG00000031403 | Dkc1 | protein\_coding | X:75095854-75109777 (+) |  | -0.2850 | 2.31e-05 | 1.65e-04 |
| ENSMUSG00000054640 | Slc8a1 | protein\_coding | 17:81373105-81738377 (-) |  | -0.2330 | 2.31e-05 | 1.66e-04 |
| ENSMUSG00000024537 | Psmg2 | protein\_coding | 18:67641599-67654162 (+) |  | -0.2660 | 2.32e-05 | 1.66e-04 |
| ENSMUSG00000021360 | Gcnt2 | protein\_coding | 13:40859754-40960892 (+) |  | -0.9190 | 2.32e-05 | 1.66e-04 |
| ENSMUSG00000054408 | Spcs3 | protein\_coding | 8:54520433-54529998 (-) |  | -0.1480 | 2.32e-05 | 1.66e-04 |
| ENSMUSG00000007564 | Ppp2r1a | protein\_coding | 17:20945311-20965916 (+) |  | -0.1840 | 2.33e-05 | 1.66e-04 |
| ENSMUSG00000049848 | Ceacam19 | protein\_coding | 7:19875742-19887965 (-) |  | 1.0000 | 2.34e-05 | 1.67e-04 |
| ENSMUSG00000022427 | Tomm22 | protein\_coding | 15:79670861-79673400 (+) |  | -0.2690 | 2.35e-05 | 1.68e-04 |
| ENSMUSG00000040128 | Pnrc1 | protein\_coding | 4:33245423-33290163 (-) |  | 0.3660 | 2.36e-05 | 1.68e-04 |
| ENSMUSG00000035493 | Tgfbi | protein\_coding | 13:56609523-56639562 (+) |  | -0.3730 | 2.37e-05 | 1.69e-04 |
| ENSMUSG00000018167 | Stard3 | protein\_coding | 11:98358368-98381112 (+) |  | 0.2700 | 2.37e-05 | 1.69e-04 |
| ENSMUSG00000025375 | Aatk | protein\_coding | 11:120007313-120047167 (-) |  | 0.5120 | 2.39e-05 | 1.70e-04 |
| ENSMUSG00000020739 | Nup85 | protein\_coding | 11:115564434-115583985 (+) |  | -0.2550 | 2.41e-05 | 1.72e-04 |
| ENSMUSG00000044338 | Aplnr | protein\_coding | 2:85136225-85139923 (+) |  | 1.1300 | 2.43e-05 | 1.73e-04 |
| ENSMUSG00000024878 | Cbwd1 | protein\_coding | 19:24919916-24961610 (-) |  | -0.4770 | 2.43e-05 | 1.73e-04 |
| ENSMUSG00000028943 | Espn | protein\_coding | 4:152120331-152152371 (-) |  | 0.9690 | 2.44e-05 | 1.74e-04 |
| ENSMUSG00000025580 | Eif4a3 | protein\_coding | 11:119288363-119300089 (-) |  | -0.2100 | 2.44e-05 | 1.74e-04 |
| ENSMUSG00000034088 | Hdlbp | protein\_coding | 1:93405940-93478815 (-) |  | -0.3050 | 2.49e-05 | 1.77e-04 |
| ENSMUSG00000057497 | Fam136a | protein\_coding | 6:86365646-86370058 (+) |  | -0.4570 | 2.51e-05 | 1.78e-04 |
| ENSMUSG00000022789 | Dnm1l | protein\_coding | 16:16312230-16358959 (-) |  | -0.2360 | 2.51e-05 | 1.78e-04 |
| ENSMUSG00000031103 | Elf4 | protein\_coding | X:48411046-48463132 (-) |  | 0.2750 | 2.51e-05 | 1.78e-04 |
| ENSMUSG00000033467 | Crlf2 | protein\_coding | 5:109554709-109558993 (-) |  | 0.2650 | 2.52e-05 | 1.79e-04 |
| ENSMUSG00000004843 | Chmp2b | protein\_coding | 16:65539128-65562726 (-) |  | 0.2600 | 2.53e-05 | 1.79e-04 |
| ENSMUSG00000051984 | Sec31b | protein\_coding | 19:44516957-44545864 (-) |  | 0.8080 | 2.53e-05 | 1.80e-04 |
| ENSMUSG00000017697 | Ada | protein\_coding | 2:163726584-163750239 (-) |  | -0.5070 | 2.56e-05 | 1.81e-04 |
| ENSMUSG00000000149 | Gna12 | protein\_coding | 5:140758408-140830431 (-) |  | 0.1890 | 2.56e-05 | 1.81e-04 |
| ENSMUSG00000042699 | Dhx9 | protein\_coding | 1:153455758-153487660 (-) |  | -0.2660 | 2.59e-05 | 1.83e-04 |
| ENSMUSG00000025956 | Mettl21a | protein\_coding | 1:64606473-64617242 (-) |  | 0.3190 | 2.59e-05 | 1.83e-04 |
| ENSMUSG00000061175 | Fnip2 | protein\_coding | 3:79455974-79567796 (-) |  | -0.3980 | 2.60e-05 | 1.84e-04 |
| ENSMUSG00000041781 | Cpsf2 | protein\_coding | 12:101975988-102006424 (+) |  | -0.1820 | 2.61e-05 | 1.84e-04 |
| ENSMUSG00000041481 | Serpina3g | protein\_coding | 12:104236245-104241939 (+) |  | -1.2000 | 2.62e-05 | 1.85e-04 |
| ENSMUSG00000035620 | Ric8b | protein\_coding | 10:84917616-85018337 (+) |  | 0.2810 | 2.62e-05 | 1.85e-04 |
| ENSMUSG00000038145 | Snrk | protein\_coding | 9:122117266-122169702 (+) |  | 0.2190 | 2.63e-05 | 1.85e-04 |
| ENSMUSG00000054499 | Dedd2 | protein\_coding | 7:25199915-25220615 (-) |  | 0.2570 | 2.63e-05 | 1.85e-04 |
| ENSMUSG00000047409 | Ctdspl | protein\_coding | 9:118926453-119043998 (+) |  | -0.3030 | 2.64e-05 | 1.86e-04 |
| ENSMUSG00000060397 | Zfp128 | protein\_coding | 7:12881177-12893422 (+) |  | 0.4630 | 2.64e-05 | 1.86e-04 |
| ENSMUSG00000028680 | Plk3 | protein\_coding | 4:117128655-117133963 (-) |  | -0.9410 | 2.66e-05 | 1.87e-04 |
| ENSMUSG00000040339 | Fam102b | protein\_coding | 3:108970997-109027607 (-) |  | 0.2840 | 2.72e-05 | 1.91e-04 |
| ENSMUSG00000030844 | Rgs10 | protein\_coding | 7:128373621-128418758 (-) |  | 0.3020 | 2.73e-05 | 1.92e-04 |
| ENSMUSG00000006920 | Ezh1 | protein\_coding | 11:101191115-101226463 (-) |  | 0.2940 | 2.73e-05 | 1.92e-04 |
| ENSMUSG00000028795 | Ccdc28b | protein\_coding | 4:129619274-129623947 (-) |  | 0.2600 | 2.74e-05 | 1.92e-04 |
| ENSMUSG00000109715 | Gm45606 | lncRNA | 11:75530277-75533071 (-) |  | 0.5460 | 2.76e-05 | 1.94e-04 |
| ENSMUSG00000062867 | Impdh2 | protein\_coding | 9:108560286-108565584 (+) |  | -0.3380 | 2.77e-05 | 1.94e-04 |
| ENSMUSG00000029330 | Cds1 | protein\_coding | 5:101765130-101823858 (+) |  | 0.3090 | 2.77e-05 | 1.95e-04 |
| ENSMUSG00000051682 | Treml4 | protein\_coding | 17:48264295-48275360 (+) |  | 1.7900 | 2.78e-05 | 1.95e-04 |
| ENSMUSG00000053119 | Chmp3 | protein\_coding | 6:71543797-71582609 (+) |  | 0.2600 | 2.78e-05 | 1.95e-04 |
| ENSMUSG00000038520 | Tbc1d17 | protein\_coding | 7:44834623-44849079 (-) |  | 0.2490 | 2.79e-05 | 1.96e-04 |
| ENSMUSG00000060791 | Gmfg | protein\_coding | 7:28437447-28448233 (+) |  | 0.3250 | 2.79e-05 | 1.96e-04 |
| ENSMUSG00000070868 | Skint3 | protein\_coding | 4:112232245-112300468 (+) |  | 1.5000 | 2.80e-05 | 1.96e-04 |
| ENSMUSG00000028212 | Ccne2 | protein\_coding | 4:11191351-11204779 (+) |  | -0.4060 | 2.81e-05 | 1.97e-04 |
| ENSMUSG00000042215 | Bag2 | protein\_coding | 1:33745484-33757795 (-) |  | -0.6610 | 2.85e-05 | 1.99e-04 |
| ENSMUSG00000043866 | Taf10 | protein\_coding | 7:105739393-105744361 (-) |  | -0.2510 | 2.85e-05 | 1.99e-04 |
| ENSMUSG00000025132 | Arhgdia | protein\_coding | 11:120578104-120581624 (-) |  | -0.2070 | 2.86e-05 | 2.00e-04 |
| ENSMUSG00000004642 | Slbp | protein\_coding | 5:33634952-33652574 (-) |  | -0.2120 | 2.86e-05 | 2.00e-04 |
| ENSMUSG00000018921 | Pelp1 | protein\_coding | 11:70392883-70410031 (-) |  | -0.3080 | 2.88e-05 | 2.01e-04 |
| ENSMUSG00000050029 | Rap2c | protein\_coding | X:51003912-51018018 (-) |  | -0.2240 | 2.89e-05 | 2.02e-04 |
| ENSMUSG00000049091 | Sephs2 | protein\_coding | 7:127271879-127274055 (-) |  | -0.2270 | 2.92e-05 | 2.03e-04 |
| ENSMUSG00000078636 | Gm7336 | processed\_pseudogene | 7:51746647-51747878 (+) |  | -0.6910 | 2.92e-05 | 2.03e-04 |
| ENSMUSG00000054720 | Lrrc8c | protein\_coding | 5:105519388-105613018 (+) |  | 0.2190 | 2.94e-05 | 2.05e-04 |
| ENSMUSG00000028792 | Ak2 | protein\_coding | 4:128991958-129011529 (+) |  | -0.2290 | 2.94e-05 | 2.05e-04 |
| ENSMUSG00000055839 | Elob | protein\_coding | 17:23824738-23829136 (-) |  | -0.2740 | 2.95e-05 | 2.05e-04 |
| ENSMUSG00000034681 | Rnps1 | protein\_coding | 17:24414565-24425901 (+) |  | -0.1880 | 2.95e-05 | 2.05e-04 |
| ENSMUSG00000030263 | Lrmp | protein\_coding | 6:145115653-145174934 (+) |  | 0.2120 | 2.96e-05 | 2.06e-04 |
| ENSMUSG00000039787 | Cercam | protein\_coding | 2:29869164-29882840 (+) |  | 0.5330 | 2.97e-05 | 2.06e-04 |
| ENSMUSG00000028484 | Psip1 | protein\_coding | 4:83455680-83486459 (-) |  | -0.2120 | 2.98e-05 | 2.07e-04 |
| ENSMUSG00000001131 | Timp1 | protein\_coding | X:20870166-20874735 (+) |  | -3.2700 | 2.98e-05 | 2.07e-04 |
| ENSMUSG00000056305 | Usp39 | protein\_coding | 6:72318678-72345184 (-) |  | -0.2160 | 3.00e-05 | 2.08e-04 |
| ENSMUSG00000028784 | Spocd1 | protein\_coding | 4:129929249-129957115 (+) |  | 1.4500 | 3.04e-05 | 2.11e-04 |
| ENSMUSG00000029469 | Ift81 | protein\_coding | 5:122550204-122614518 (-) |  | 0.7350 | 3.05e-05 | 2.12e-04 |
| ENSMUSG00000029594 | Rbm19 | protein\_coding | 5:120116465-120198981 (+) |  | -0.3490 | 3.05e-05 | 2.12e-04 |
| ENSMUSG00000015747 | Vps45 | protein\_coding | 3:95999832-96058466 (-) |  | -0.3380 | 3.10e-05 | 2.15e-04 |
| ENSMUSG00000046658 | Zfp316 | protein\_coding | 5:143249697-143270022 (-) |  | 0.5620 | 3.11e-05 | 2.15e-04 |
| ENSMUSG00000053338 | Tarm1 | protein\_coding | 7:3486500-3502624 (-) |  | -0.5720 | 3.12e-05 | 2.16e-04 |
| ENSMUSG00000026034 | Clk1 | protein\_coding | 1:58410189-58424066 (-) |  | 0.4550 | 3.13e-05 | 2.16e-04 |
| ENSMUSG00000040945 | Rcc2 | protein\_coding | 4:140700541-140723220 (+) |  | -0.2750 | 3.14e-05 | 2.17e-04 |
| ENSMUSG00000039221 | Rpl22l1 | protein\_coding | 3:28805436-28807424 (+) |  | -0.4110 | 3.15e-05 | 2.17e-04 |
| ENSMUSG00000025147 | Mob2 | protein\_coding | 7:142008535-142061065 (-) |  | 0.2630 | 3.20e-05 | 2.21e-04 |
| ENSMUSG00000024776 | Stambpl1 | protein\_coding | 19:34192229-34240333 (+) |  | -0.2860 | 3.22e-05 | 2.22e-04 |
| ENSMUSG00000028973 | Abcb8 | protein\_coding | 5:24393663-24410054 (+) |  | -0.3210 | 3.23e-05 | 2.23e-04 |
| ENSMUSG00000022191 | Drosha | protein\_coding | 15:12824815-12935291 (+) |  | -0.2750 | 3.23e-05 | 2.23e-04 |
| ENSMUSG00000021559 | Dapk1 | protein\_coding | 13:60601947-60763191 (+) |  | 0.4840 | 3.24e-05 | 2.23e-04 |
| ENSMUSG00000062981 | Mrpl42 | protein\_coding | 10:95480805-95501940 (-) |  | -0.2610 | 3.25e-05 | 2.24e-04 |
| ENSMUSG00000067150 | Xpo5 | protein\_coding | 17:46202782-46243598 (+) |  | -0.2360 | 3.25e-05 | 2.24e-04 |
| ENSMUSG00000032050 | Rdx | protein\_coding | 9:52047173-52100463 (+) |  | -0.1650 | 3.26e-05 | 2.24e-04 |
| ENSMUSG00000026004 | Kansl1l | protein\_coding | 1:66719248-66817562 (-) |  | 0.4010 | 3.27e-05 | 2.25e-04 |
| ENSMUSG00000039478 | Micu3 | protein\_coding | 8:40307458-40386308 (+) |  | 0.8200 | 3.28e-05 | 2.25e-04 |
| ENSMUSG00000040383 | Aqr | protein\_coding | 2:114101170-114187024 (-) |  | -0.2680 | 3.29e-05 | 2.26e-04 |
| ENSMUSG00000079491 | H2-T10 | polymorphic\_pseudogene | 17:36115876-36121465 (-) |  | -0.3890 | 3.30e-05 | 2.27e-04 |
| ENSMUSG00000026005 | Rpe | protein\_coding | 1:66700831-66719805 (+) |  | -0.3210 | 3.31e-05 | 2.27e-04 |
| ENSMUSG00000045438 | Cox19 | protein\_coding | 5:139336189-139351418 (-) |  | -0.2890 | 3.33e-05 | 2.29e-04 |
| ENSMUSG00000054808 | Actn4 | protein\_coding | 7:28893248-28962340 (-) |  | -0.1940 | 3.34e-05 | 2.29e-04 |
| ENSMUSG00000010406 | Mrpl52 | protein\_coding | 14:54426909-54429756 (+) |  | -0.3330 | 3.34e-05 | 2.29e-04 |
| ENSMUSG00000024413 | Npc1 | protein\_coding | 18:12189692-12236400 (-) |  | 0.2540 | 3.35e-05 | 2.30e-04 |
| ENSMUSG00000038014 | Fam120a | protein\_coding | 13:48879219-48968017 (-) |  | -0.2100 | 3.35e-05 | 2.30e-04 |
| ENSMUSG00000028885 | Smpdl3b | protein\_coding | 4:132732966-132757252 (-) |  | 1.0800 | 3.36e-05 | 2.30e-04 |
| ENSMUSG00000051235 | Gen1 | protein\_coding | 12:11238920-11265801 (-) |  | -0.2280 | 3.36e-05 | 2.30e-04 |
| ENSMUSG00000006715 | Gmnn | protein\_coding | 13:24751845-24761923 (-) |  | -0.2500 | 3.38e-05 | 2.31e-04 |
| ENSMUSG00000003309 | Ap1m2 | protein\_coding | 9:21294275-21312337 (-) |  | -0.9030 | 3.41e-05 | 2.33e-04 |
| ENSMUSG00000024989 | Cep55 | protein\_coding | 19:38055011-38074423 (+) |  | -0.2830 | 3.41e-05 | 2.33e-04 |
| ENSMUSG00000039763 | Dnajc28 | protein\_coding | 16:91614254-91619026 (-) |  | 0.5300 | 3.42e-05 | 2.34e-04 |
| ENSMUSG00000021596 | Mctp1 | protein\_coding | 13:76384535-77031810 (+) |  | 0.2900 | 3.46e-05 | 2.36e-04 |
| ENSMUSG00000038510 | Rpf2 | protein\_coding | 10:40223246-40247036 (-) |  | -0.3460 | 3.48e-05 | 2.37e-04 |
| ENSMUSG00000019295 | Tmem129 | protein\_coding | 5:33653216-33658416 (-) |  | -0.2290 | 3.48e-05 | 2.37e-04 |
| ENSMUSG00000087701 | Gm13493 | processed\_pseudogene | 2:51871126-51871618 (-) |  | -0.3490 | 3.51e-05 | 2.39e-04 |
| ENSMUSG00000035198 | Tubg1 | protein\_coding | 11:101119938-101126419 (+) |  | -0.3160 | 3.51e-05 | 2.39e-04 |
| ENSMUSG00000026634 | Angel2 | protein\_coding | 1:190925112-190946962 (+) |  | 0.1630 | 3.51e-05 | 2.39e-04 |
| ENSMUSG00000035443 | Thyn1 | protein\_coding | 9:26999710-27007336 (+) |  | -0.2150 | 3.52e-05 | 2.40e-04 |
| ENSMUSG00000038637 | Lrrc56 | protein\_coding | 7:141194157-141210055 (+) |  | 0.5250 | 3.54e-05 | 2.41e-04 |
| ENSMUSG00000002870 | Mcm2 | protein\_coding | 6:88883474-88898780 (-) |  | -0.3220 | 3.55e-05 | 2.41e-04 |
| ENSMUSG00000000378 | Ccm2 | protein\_coding | 11:6546887-6596744 (+) |  | 0.2240 | 3.57e-05 | 2.42e-04 |
| ENSMUSG00000039013 | Siglecf | protein\_coding | 7:43351341-43359531 (+) |  | 0.5930 | 3.59e-05 | 2.44e-04 |
| ENSMUSG00000035754 | Wdr18 | protein\_coding | 10:79960152-79970203 (+) |  | -0.3380 | 3.61e-05 | 2.45e-04 |
| ENSMUSG00000021687 | Scamp1 | protein\_coding | 13:94201310-94285857 (-) |  | -0.3410 | 3.62e-05 | 2.46e-04 |
| ENSMUSG00000001228 | Uhrf1 | protein\_coding | 17:56303321-56323486 (+) |  | -0.3610 | 3.64e-05 | 2.47e-04 |
| ENSMUSG00000087175 | Gm15133 | lncRNA | 7:104315019-104324838 (+) |  | 1.0300 | 3.65e-05 | 2.47e-04 |
| ENSMUSG00000046908 | Ltb4r1 | protein\_coding | 14:55765962-55768494 (+) |  | 0.2800 | 3.66e-05 | 2.48e-04 |
| ENSMUSG00000019992 | Mtfr2 | protein\_coding | 10:20347770-20361304 (+) |  | -0.2480 | 3.67e-05 | 2.49e-04 |
| ENSMUSG00000032294 | Pkm | protein\_coding | 9:59656368-59679375 (+) |  | -0.2220 | 3.68e-05 | 2.49e-04 |
| ENSMUSG00000092572 | Serpinb10 | polymorphic\_pseudogene | 1:107529003-107549271 (+) |  | 0.6700 | 3.69e-05 | 2.50e-04 |
| ENSMUSG00000039989 | Cbx4 | protein\_coding | 11:119077573-119086221 (-) |  | 0.2030 | 3.72e-05 | 2.52e-04 |
| ENSMUSG00000111877 | Gm6477 | processed\_pseudogene | 10:39198538-39199301 (+) |  | -0.4990 | 3.73e-05 | 2.52e-04 |
| ENSMUSG00000015243 | Abca1 | protein\_coding | 4:53030787-53159895 (-) |  | 0.7580 | 3.73e-05 | 2.52e-04 |
| ENSMUSG00000001348 | Acp5 | protein\_coding | 9:22126727-22135711 (-) |  | -0.6050 | 3.75e-05 | 2.54e-04 |
| ENSMUSG00000073902 | Gm1966 | unprocessed\_pseudogene | 7:106596743-106604035 (-) |  | -0.4170 | 3.76e-05 | 2.54e-04 |
| ENSMUSG00000039987 | Phtf2 | protein\_coding | 5:20758663-20882124 (-) |  | -0.2770 | 3.76e-05 | 2.54e-04 |
| ENSMUSG00000068129 | Cst7 | protein\_coding | 2:150570415-150578944 (+) |  | -0.7840 | 3.77e-05 | 2.54e-04 |
| ENSMUSG00000038633 | Degs1 | protein\_coding | 1:182275772-182282804 (-) |  | 0.2730 | 3.77e-05 | 2.54e-04 |
| ENSMUSG00000032306 | Mpi | protein\_coding | 9:57544256-57552763 (-) |  | -0.2900 | 3.78e-05 | 2.55e-04 |
| ENSMUSG00000006057 | Atp5g1 | protein\_coding | 11:96068852-96075670 (-) |  | -0.3100 | 3.78e-05 | 2.55e-04 |
| ENSMUSG00000035575 | Utp6 | protein\_coding | 11:79932321-79962390 (-) |  | -0.1790 | 3.79e-05 | 2.56e-04 |
| ENSMUSG00000081344 | Gm14303 | processed\_pseudogene | 2:172509521-172509691 (-) |  | 0.4660 | 3.82e-05 | 2.57e-04 |
| ENSMUSG00000006392 | Med8 | protein\_coding | 4:118409337-118415782 (+) |  | -0.2070 | 3.83e-05 | 2.58e-04 |
| ENSMUSG00000035439 | Haus8 | protein\_coding | 8:71248561-71272934 (-) |  | -0.2270 | 3.83e-05 | 2.58e-04 |
| ENSMUSG00000020644 | Id2 | protein\_coding | 12:25093799-25097140 (-) |  | 0.6500 | 3.85e-05 | 2.59e-04 |
| ENSMUSG00000020573 | Pik3cg | protein\_coding | 12:32173473-32208659 (-) |  | 0.2070 | 3.85e-05 | 2.59e-04 |
| ENSMUSG00000020143 | Dock2 | protein\_coding | 11:34226815-34783892 (-) |  | 0.1770 | 3.86e-05 | 2.60e-04 |
| ENSMUSG00000042029 | Ncapg2 | protein\_coding | 12:116405402-116463731 (+) |  | -0.2850 | 3.94e-05 | 2.64e-04 |
| ENSMUSG00000026655 | Fam107b | protein\_coding | 2:3570488-3782142 (+) |  | 0.1600 | 3.95e-05 | 2.65e-04 |
| ENSMUSG00000013155 | Enkd1 | protein\_coding | 8:105703651-105708210 (-) |  | -0.3440 | 3.95e-05 | 2.65e-04 |
| ENSMUSG00000059179 | Gm8991 | processed\_pseudogene | 5:16729996-16731070 (-) |  | -0.3880 | 3.95e-05 | 2.65e-04 |
| ENSMUSG00000047747 | Rnf150 | protein\_coding | 8:82863356-83091268 (+) |  | 0.7140 | 3.95e-05 | 2.65e-04 |
| ENSMUSG00000056054 | S100a8 | protein\_coding | 3:90668978-90670035 (+) |  | -1.2600 | 3.99e-05 | 2.67e-04 |
| ENSMUSG00000011254 | Thg1l | protein\_coding | 11:45946843-45955494 (-) |  | -0.3000 | 3.99e-05 | 2.67e-04 |
| ENSMUSG00000026037 | Orc2 | protein\_coding | 1:58462771-58505109 (-) |  | -0.2740 | 4.02e-05 | 2.69e-04 |
| ENSMUSG00000034165 | Ccnd3 | protein\_coding | 17:47505051-47599691 (+) |  | -0.2230 | 4.02e-05 | 2.69e-04 |
| ENSMUSG00000029501 | Ankle2 | protein\_coding | 5:110231004-110256651 (+) |  | 0.1930 | 4.02e-05 | 2.69e-04 |
| ENSMUSG00000002871 | Tpra1 | protein\_coding | 6:88902251-88912238 (+) |  | 0.2750 | 4.03e-05 | 2.69e-04 |
| ENSMUSG00000009293 | Ube2g2 | protein\_coding | 10:77622275-77645993 (+) |  | -0.2150 | 4.08e-05 | 2.72e-04 |
| ENSMUSG00000015597 | Zfp318 | protein\_coding | 17:46383731-46420920 (+) |  | 0.3720 | 4.11e-05 | 2.74e-04 |
| ENSMUSG00000034424 | Gcsh | protein\_coding | 8:116981810-116993537 (-) |  | -0.3700 | 4.11e-05 | 2.74e-04 |
| ENSMUSG00000042570 | Mier2 | protein\_coding | 10:79540245-79555199 (-) |  | 0.2780 | 4.13e-05 | 2.75e-04 |
| ENSMUSG00000024527 | Afg3l2 | protein\_coding | 18:67404764-67449172 (-) |  | -0.1830 | 4.13e-05 | 2.75e-04 |
| ENSMUSG00000061143 | Maml3 | protein\_coding | 3:51685907-52105076 (-) |  | 0.3450 | 4.13e-05 | 2.75e-04 |
| ENSMUSG00000041538 | H2-Ob | protein\_coding | 17:34238903-34254414 (+) |  | 0.9490 | 4.14e-05 | 2.76e-04 |
| ENSMUSG00000024498 | Tcerg1 | protein\_coding | 18:42511487-42575793 (+) |  | -0.2310 | 4.15e-05 | 2.76e-04 |
| ENSMUSG00000040165 | Cd209c | protein\_coding | 8:3940222-3954746 (-) |  | 2.4300 | 4.19e-05 | 2.79e-04 |
| ENSMUSG00000025510 | Cd151 | protein\_coding | 7:141467392-141471473 (+) |  | 0.3480 | 4.19e-05 | 2.79e-04 |
| ENSMUSG00000005410 | Mcm5 | protein\_coding | 8:75109569-75128439 (+) |  | -0.3100 | 4.20e-05 | 2.79e-04 |
| ENSMUSG00000034724 | Cnot6l | protein\_coding | 5:96070333-96164171 (-) |  | 0.1870 | 4.20e-05 | 2.79e-04 |
| ENSMUSG00000090231 | Cfb | protein\_coding | 17:34856374-34862518 (-) |  | -1.3300 | 4.20e-05 | 2.79e-04 |
| ENSMUSG00000049625 | Tifab | protein\_coding | 13:56173704-56178885 (-) |  | 0.2380 | 4.20e-05 | 2.79e-04 |
| ENSMUSG00000031389 | Arhgap4 | protein\_coding | X:73891442-73921870 (-) |  | 0.2350 | 4.21e-05 | 2.79e-04 |
| ENSMUSG00000015467 | Egfl8 | protein\_coding | 17:34613349-34615971 (-) |  | 0.7200 | 4.25e-05 | 2.82e-04 |
| ENSMUSG00000031256 | Cstf2 | protein\_coding | X:134059187-134086819 (+) |  | -0.1950 | 4.25e-05 | 2.82e-04 |
| ENSMUSG00000046707 | Csnk2a2 | protein\_coding | 8:95446096-95490039 (-) |  | -0.2310 | 4.29e-05 | 2.84e-04 |
| ENSMUSG00000062949 | Atp11c | protein\_coding | X:60223290-60592698 (-) |  | 0.2550 | 4.34e-05 | 2.88e-04 |
| ENSMUSG00000056071 | S100a9 | protein\_coding | 3:90692632-90695721 (-) |  | -1.4200 | 4.36e-05 | 2.89e-04 |
| ENSMUSG00000055639 | Dach1 | protein\_coding | 14:97786853-98169765 (-) |  | 0.9380 | 4.36e-05 | 2.89e-04 |
| ENSMUSG00000038227 | Hoxa9 | protein\_coding | 6:52223100-52231089 (-) |  | -1.5100 | 4.37e-05 | 2.89e-04 |
| ENSMUSG00000001415 | Smg5 | protein\_coding | 3:88336260-88362338 (+) |  | -0.1940 | 4.38e-05 | 2.90e-04 |
| ENSMUSG00000054582 | Pabpc1l | protein\_coding | 2:164025450-164050538 (+) |  | 0.8060 | 4.38e-05 | 2.90e-04 |
| ENSMUSG00000071715 | Ncf4 | protein\_coding | 15:78244801-78262580 (+) |  | -0.3120 | 4.39e-05 | 2.90e-04 |
| ENSMUSG00000042606 | Hirip3 | protein\_coding | 7:126861972-126865377 (+) |  | -0.2590 | 4.40e-05 | 2.90e-04 |
| ENSMUSG00000027323 | Rad51 | protein\_coding | 2:119112793-119147445 (+) |  | -0.3480 | 4.40e-05 | 2.90e-04 |
| ENSMUSG00000041921 | Metap1d | protein\_coding | 2:71453276-71525194 (+) |  | 0.4060 | 4.40e-05 | 2.91e-04 |
| ENSMUSG00000057409 | Zfp53 | protein\_coding | 17:21488998-21510473 (+) |  | 0.2770 | 4.44e-05 | 2.93e-04 |
| ENSMUSG00000069792 | Wfdc17 | protein\_coding | 11:83703991-83706268 (+) |  | -0.8960 | 4.45e-05 | 2.93e-04 |
| ENSMUSG00000037337 | Map4k1 | protein\_coding | 7:28982050-29003279 (+) |  | -0.2300 | 4.45e-05 | 2.93e-04 |
| ENSMUSG00000029387 | Gtf2h3 | protein\_coding | 5:124579140-124597680 (+) |  | -0.3840 | 4.46e-05 | 2.93e-04 |
| ENSMUSG00000098274 | Rpl24 | protein\_coding | 16:55966275-55971435 (+) |  | -0.4420 | 4.47e-05 | 2.94e-04 |
| ENSMUSG00000022710 | Usp7 | protein\_coding | 16:8689595-8792308 (-) |  | -0.1810 | 4.48e-05 | 2.95e-04 |
| ENSMUSG00000027130 | Slc12a6 | protein\_coding | 2:112265825-112363163 (+) |  | 0.2810 | 4.49e-05 | 2.95e-04 |
| ENSMUSG00000021957 | Tkt | protein\_coding | 14:30548359-30574720 (+) |  | -0.2130 | 4.49e-05 | 2.95e-04 |
| ENSMUSG00000005034 | Prkacb | protein\_coding | 3:146729574-146812990 (-) |  | 0.1890 | 4.49e-05 | 2.95e-04 |
| ENSMUSG00000044452 | Zfp507 | protein\_coding | 7:35772343-35803003 (-) |  | 0.9120 | 4.50e-05 | 2.96e-04 |
| ENSMUSG00000038335 | Tsr1 | protein\_coding | 11:74898071-74909342 (+) |  | -0.3010 | 4.50e-05 | 2.96e-04 |
| ENSMUSG00000042997 | Nhlrc3 | protein\_coding | 3:53448583-53463332 (-) |  | 0.3120 | 4.55e-05 | 2.98e-04 |
| ENSMUSG00000020537 | Drg2 | protein\_coding | 11:60454591-60468754 (+) |  | -0.2480 | 4.56e-05 | 2.99e-04 |
| ENSMUSG00000025792 | Slc25a10 | protein\_coding | 11:120491840-120499187 (+) |  | -0.2460 | 4.62e-05 | 3.03e-04 |
| ENSMUSG00000008193 | Spib | protein\_coding | 7:44525993-44532071 (-) |  | 1.0900 | 4.63e-05 | 3.03e-04 |
| ENSMUSG00000033705 | Stard9 | protein\_coding | 2:120629121-120731895 (+) |  | 0.4320 | 4.64e-05 | 3.04e-04 |
| ENSMUSG00000089782 | Gm3531 | processed\_pseudogene | 1:97948901-97949389 (-) |  | -0.4430 | 4.65e-05 | 3.05e-04 |
| ENSMUSG00000081999 | Gm13461 | processed\_pseudogene | 2:41987473-41988829 (-) |  | -0.5280 | 4.67e-05 | 3.06e-04 |
| ENSMUSG00000109572 | Cfap99 | protein\_coding | 5:34288600-34327327 (+) |  | 1.5300 | 4.69e-05 | 3.07e-04 |
| ENSMUSG00000027227 | Sord | protein\_coding | 2:122234749-122265340 (+) |  | -0.2210 | 4.73e-05 | 3.09e-04 |
| ENSMUSG00000035776 | Cd99l2 | protein\_coding | X:71420060-71492849 (-) |  | 0.3650 | 4.76e-05 | 3.11e-04 |
| ENSMUSG00000030872 | Gga2 | protein\_coding | 7:121986722-122021222 (-) |  | -0.2840 | 4.80e-05 | 3.14e-04 |
| ENSMUSG00000026547 | Tagln2 | protein\_coding | 1:172500047-172507380 (+) |  | -0.3070 | 4.82e-05 | 3.15e-04 |
| ENSMUSG00000021930 | Spryd7 | protein\_coding | 14:61531993-61556886 (-) |  | -0.3770 | 4.91e-05 | 3.20e-04 |
| ENSMUSG00000038244 | Mical2 | protein\_coding | 7:112225856-112413106 (+) |  | 0.7590 | 4.97e-05 | 3.24e-04 |
| ENSMUSG00000069565 | Dazap1 | protein\_coding | 10:80261486-80288408 (+) |  | -0.1840 | 4.98e-05 | 3.25e-04 |
| ENSMUSG00000027479 | Mapre1 | protein\_coding | 2:153741274-153773310 (+) |  | -0.1610 | 5.05e-05 | 3.29e-04 |
| ENSMUSG00000056629 | Fkbp2 | protein\_coding | 19:6977741-6980501 (-) |  | -0.2410 | 5.06e-05 | 3.30e-04 |
| ENSMUSG00000025428 | Atp5a1 | protein\_coding | 18:77773729-77782869 (+) |  | -0.2030 | 5.08e-05 | 3.31e-04 |
| ENSMUSG00000063052 | Lrrc40 | protein\_coding | 3:158036662-158068487 (+) |  | -0.2610 | 5.09e-05 | 3.32e-04 |
| ENSMUSG00000034111 | Tmed8 | protein\_coding | 12:87166237-87200454 (-) |  | -0.2890 | 5.10e-05 | 3.32e-04 |
| ENSMUSG00000031820 | Babam1 | protein\_coding | 8:71396861-71404619 (+) |  | -0.2070 | 5.18e-05 | 3.37e-04 |
| ENSMUSG00000031527 | Eri1 | protein\_coding | 8:35465253-35496196 (-) |  | -0.1830 | 5.19e-05 | 3.37e-04 |
| ENSMUSG00000022999 | Lmbr1l | protein\_coding | 15:98903917-98918231 (-) |  | 0.3360 | 5.21e-05 | 3.38e-04 |
| ENSMUSG00000028737 | Aldh4a1 | protein\_coding | 4:139622866-139649690 (+) |  | -0.3200 | 5.21e-05 | 3.38e-04 |
| ENSMUSG00000004837 | Grap | protein\_coding | 11:61653265-61672784 (+) |  | 0.5680 | 5.22e-05 | 3.39e-04 |
| ENSMUSG00000000244 | Tspan32 | protein\_coding | 7:143005046-143019644 (+) |  | 0.2950 | 5.24e-05 | 3.40e-04 |
| ENSMUSG00000022707 | Gbe1 | protein\_coding | 16:70313949-70569716 (+) |  | 0.2480 | 5.24e-05 | 3.40e-04 |
| ENSMUSG00000022711 | Pmm2 | protein\_coding | 16:8637674-8662467 (+) |  | -0.2030 | 5.27e-05 | 3.42e-04 |
| ENSMUSG00000094257 | Ap3s1-ps2 | processed\_pseudogene | 8:94405211-94405789 (+) |  | -0.6570 | 5.33e-05 | 3.45e-04 |
| ENSMUSG00000005225 | Plekha8 | protein\_coding | 6:54595111-54645839 (+) |  | -0.6640 | 5.38e-05 | 3.48e-04 |
| ENSMUSG00000008036 | Ap2s1 | protein\_coding | 7:16738410-16749294 (+) |  | -0.2160 | 5.41e-05 | 3.50e-04 |
| ENSMUSG00000037710 | Cisd1 | protein\_coding | 10:71330480-71344954 (-) |  | -0.2750 | 5.41e-05 | 3.50e-04 |
| ENSMUSG00000034674 | Tdg | protein\_coding | 10:82629828-82650799 (+) |  | -0.2510 | 5.42e-05 | 3.50e-04 |
| ENSMUSG00000049038 | Mterf2 | protein\_coding | 10:85119433-85128027 (-) |  | -1.0100 | 5.47e-05 | 3.54e-04 |
| ENSMUSG00000015980 | Lrrc27 | protein\_coding | 7:139212988-139242979 (+) |  | -1.0700 | 5.52e-05 | 3.57e-04 |
| ENSMUSG00000070003 | Ssbp4 | protein\_coding | 8:70597490-70608872 (-) |  | -0.2330 | 5.57e-05 | 3.60e-04 |
| ENSMUSG00000074419 | Gm15448 | protein\_coding | 7:3816781-3825687 (-) |  | 0.4610 | 5.62e-05 | 3.63e-04 |
| ENSMUSG00000026811 | St6galnac6 | protein\_coding | 2:32599709-32620806 (+) |  | 0.2670 | 5.63e-05 | 3.64e-04 |
| ENSMUSG00000019970 | Sgk1 | protein\_coding | 10:21882184-21999903 (+) |  | 0.4600 | 5.65e-05 | 3.65e-04 |
| ENSMUSG00000117975 | Itprip | protein\_coding | 19:47894602-47919299 (-) |  | 0.1860 | 5.68e-05 | 3.66e-04 |
| ENSMUSG00000005609 | Ctr9 | protein\_coding | 7:111028951-111056377 (+) |  | -0.1760 | 5.69e-05 | 3.67e-04 |
| ENSMUSG00000035649 | Zcchc7 | protein\_coding | 4:44755877-44932215 (+) |  | 0.3100 | 5.75e-05 | 3.71e-04 |
| ENSMUSG00000045917 | Tmem268 | protein\_coding | 4:63558781-63586357 (+) |  | -0.2630 | 5.76e-05 | 3.71e-04 |
| ENSMUSG00000085442 | Gm3362 | processed\_pseudogene | 15:37943327-37943792 (-) |  | -0.4470 | 5.77e-05 | 3.71e-04 |
| ENSMUSG00000034764 | 1700006J14Rik | lncRNA | 10:120364157-120384336 (+) |  | 0.9640 | 5.78e-05 | 3.72e-04 |
| ENSMUSG00000075232 | Amd1 | protein\_coding | 10:40287458-40302188 (-) |  | -0.2000 | 5.84e-05 | 3.76e-04 |
| ENSMUSG00000024120 | Lrpprc | protein\_coding | 17:84705247-84790789 (-) |  | -0.2660 | 5.88e-05 | 3.78e-04 |
| ENSMUSG00000049916 | 2610318N02Rik | protein\_coding | 16:17113398-17125167 (-) |  | -0.5640 | 5.88e-05 | 3.78e-04 |
| ENSMUSG00000059013 | Sh2d3c | protein\_coding | 2:32721055-32755512 (+) |  | 0.3830 | 5.95e-05 | 3.82e-04 |
| ENSMUSG00000025857 | Dnaaf5 | protein\_coding | 5:139150223-139186510 (+) |  | -0.4180 | 5.96e-05 | 3.83e-04 |
| ENSMUSG00000029397 | Rchy1 | protein\_coding | 5:91948904-91963068 (-) |  | 0.2300 | 5.97e-05 | 3.83e-04 |
| ENSMUSG00000029161 | Cgref1 | protein\_coding | 5:30933143-30945591 (-) |  | 1.0400 | 6.04e-05 | 3.87e-04 |
| ENSMUSG00000036257 | Pnpla8 | protein\_coding | 12:44221370-44322532 (+) |  | 0.2130 | 6.04e-05 | 3.87e-04 |
| ENSMUSG00000036693 | Nop14 | protein\_coding | 5:34638536-34660148 (-) |  | -0.3080 | 6.06e-05 | 3.89e-04 |
| ENSMUSG00000041439 | Mfsd6 | protein\_coding | 1:52656286-52727462 (-) |  | -0.3160 | 6.08e-05 | 3.89e-04 |
| ENSMUSG00000024457 | Trim26 | protein\_coding | 17:36837134-36859398 (+) |  | 0.2590 | 6.10e-05 | 3.90e-04 |
| ENSMUSG00000035929 | H2-Q4 | protein\_coding | 17:35379617-35385290 (+) |  | -0.2550 | 6.12e-05 | 3.92e-04 |
| ENSMUSG00000002319 | Ipo4 | protein\_coding | 14:55625400-55635957 (-) |  | -0.3800 | 6.12e-05 | 3.92e-04 |
| ENSMUSG00000060429 | Sntb1 | protein\_coding | 15:55636388-55906949 (-) |  | 0.2540 | 6.16e-05 | 3.94e-04 |
| ENSMUSG00000026525 | Opn3 | protein\_coding | 1:175662421-175692776 (-) |  | -0.7010 | 6.16e-05 | 3.94e-04 |
| ENSMUSG00000019822 | Smpd2 | protein\_coding | 10:41485642-41490369 (-) |  | 0.3100 | 6.17e-05 | 3.94e-04 |
| ENSMUSG00000082016 | Pgam1-ps2 | processed\_pseudogene | 7:27469771-27470535 (-) |  | -0.4970 | 6.18e-05 | 3.95e-04 |
| ENSMUSG00000073542 | Cep76 | protein\_coding | 18:67617800-67641336 (-) |  | -0.2490 | 6.19e-05 | 3.95e-04 |
| ENSMUSG00000003814 | Calr | protein\_coding | 8:84841850-84846934 (-) |  | -0.4500 | 6.20e-05 | 3.95e-04 |
| ENSMUSG00000010453 | Kansl3 | protein\_coding | 1:36335730-36369181 (-) |  | 0.2000 | 6.20e-05 | 3.95e-04 |
| ENSMUSG00000030528 | Blm | protein\_coding | 7:80454733-80535119 (-) |  | -0.2410 | 6.20e-05 | 3.95e-04 |
| ENSMUSG00000017615 | Tnfaip1 | protein\_coding | 11:78522850-78536332 (-) |  | -0.2520 | 6.24e-05 | 3.98e-04 |
| ENSMUSG00000027936 | Crtc2 | protein\_coding | 3:90254163-90264125 (+) |  | 0.2510 | 6.26e-05 | 3.99e-04 |
| ENSMUSG00000039128 | Cdc123 | protein\_coding | 2:5794294-5845164 (-) |  | -0.1920 | 6.26e-05 | 3.99e-04 |
| ENSMUSG00000022142 | Nup155 | protein\_coding | 15:8109273-8161247 (+) |  | -0.2190 | 6.30e-05 | 4.01e-04 |
| ENSMUSG00000032041 | Tirap | protein\_coding | 9:35184551-35200291 (-) |  | -0.2460 | 6.32e-05 | 4.02e-04 |
| ENSMUSG00000049606 | Zfp644 | protein\_coding | 5:106616739-106697287 (-) |  | 0.2250 | 6.34e-05 | 4.03e-04 |
| ENSMUSG00000029810 | Tmem176b | protein\_coding | 6:48833818-48841496 (-) |  | -0.4370 | 6.36e-05 | 4.04e-04 |
| ENSMUSG00000000154 | Slc22a18 | protein\_coding | 7:143473736-143499334 (+) |  | 0.8660 | 6.37e-05 | 4.04e-04 |
| ENSMUSG00000004207 | Psap | protein\_coding | 10:60277627-60302597 (+) |  | 0.2300 | 6.39e-05 | 4.06e-04 |
| ENSMUSG00000031442 | Mcf2l | protein\_coding | 8:12873806-13020905 (+) |  | 0.2710 | 6.40e-05 | 4.06e-04 |
| ENSMUSG00000049687 | Pheta2 | protein\_coding | 15:82338942-82345743 (+) |  | 0.4390 | 6.43e-05 | 4.08e-04 |
| ENSMUSG00000033488 | Cryzl2 | protein\_coding | 1:157458577-157492638 (+) |  | 0.4390 | 6.46e-05 | 4.09e-04 |
| ENSMUSG00000063358 | Mapk1 | protein\_coding | 16:16983382-17047453 (+) |  | -0.1350 | 6.46e-05 | 4.09e-04 |
| ENSMUSG00000043445 | Pgp | protein\_coding | 17:24470392-24473110 (+) |  | -0.1940 | 6.48e-05 | 4.10e-04 |
| ENSMUSG00000028443 | Nudt2 | protein\_coding | 4:41465151-41480926 (+) |  | -0.3830 | 6.48e-05 | 4.11e-04 |
| ENSMUSG00000040652 | Oaz2 | protein\_coding | 9:65668001-65690300 (+) |  | 0.3200 | 6.50e-05 | 4.11e-04 |
| ENSMUSG00000001918 | Slc1a5 | protein\_coding | 7:16781340-16798274 (+) |  | -0.2050 | 6.51e-05 | 4.12e-04 |
| ENSMUSG00000030138 | Bms1 | protein\_coding | 6:118383381-118419474 (-) |  | -0.2300 | 6.62e-05 | 4.19e-04 |
| ENSMUSG00000017715 | Pgs1 | protein\_coding | 11:117986292-118024011 (+) |  | -0.1900 | 6.63e-05 | 4.19e-04 |
| ENSMUSG00000027131 | Emc4 | protein\_coding | 2:112363011-112368027 (-) |  | -0.2650 | 6.65e-05 | 4.20e-04 |
| ENSMUSG00000030881 | Arfip2 | protein\_coding | 7:105634203-105640416 (-) |  | -0.2010 | 6.66e-05 | 4.21e-04 |
| ENSMUSG00000028312 | Smc2 | protein\_coding | 4:52439243-52488260 (+) |  | -0.2350 | 6.68e-05 | 4.21e-04 |
| ENSMUSG00000107355 | AI839979 | lncRNA | 5:31569595-31571397 (-) |  | 0.4160 | 6.68e-05 | 4.21e-04 |
| ENSMUSG00000029028 | Lrrc47 | protein\_coding | 4:154011731-154021513 (+) |  | -0.1880 | 6.72e-05 | 4.24e-04 |
| ENSMUSG00000028163 | Nfkb1 | protein\_coding | 3:135584655-135691547 (-) |  | 0.1730 | 6.74e-05 | 4.25e-04 |
| ENSMUSG00000006395 | Hyi | polymorphic\_pseudogene | 4:118359990-118362744 (+) |  | 0.7240 | 6.74e-05 | 4.25e-04 |
| ENSMUSG00000038042 | Ptpdc1 | protein\_coding | 13:48577872-48625664 (-) |  | 0.7770 | 6.80e-05 | 4.28e-04 |
| ENSMUSG00000032540 | Abhd5 | protein\_coding | 9:122351608-122381524 (+) |  | -0.2810 | 6.81e-05 | 4.29e-04 |
| ENSMUSG00000053907 | Mat2a | protein\_coding | 6:72432799-72439558 (-) |  | -0.2420 | 6.81e-05 | 4.29e-04 |
| ENSMUSG00000068262 | Gm5879 | processed\_pseudogene | 6:87764996-87766204 (-) |  | -0.3530 | 6.85e-05 | 4.31e-04 |
| ENSMUSG00000045078 | Rnf216 | protein\_coding | 5:142990893-143112994 (-) |  | 0.1620 | 6.87e-05 | 4.32e-04 |
| ENSMUSG00000029322 | Plac8 | protein\_coding | 5:100553725-100572245 (-) |  | -0.3640 | 6.90e-05 | 4.34e-04 |
| ENSMUSG00000092060 | Bend4 | protein\_coding | 5:67392147-67428310 (-) |  | -0.6880 | 6.92e-05 | 4.35e-04 |
| ENSMUSG00000056019 | Zfp709 | protein\_coding | 8:71882019-71895727 (+) |  | 0.3030 | 6.92e-05 | 4.35e-04 |
| ENSMUSG00000032012 | Nectin1 | protein\_coding | 9:43743984-43832658 (+) |  | 0.5290 | 6.93e-05 | 4.35e-04 |
| ENSMUSG00000048498 | Cd300e | protein\_coding | 11:115051917-115062177 (-) |  | 4.0400 | 6.94e-05 | 4.35e-04 |
| ENSMUSG00000018474 | Chd3 | protein\_coding | 11:69343273-69369406 (-) |  | 0.3990 | 6.97e-05 | 4.37e-04 |
| ENSMUSG00000044252 | Osbpl1a | protein\_coding | 18:12755314-12941841 (-) |  | -0.3140 | 6.98e-05 | 4.37e-04 |
| ENSMUSG00000079297 | Gm2223 | processed\_pseudogene | X:33505661-33507010 (+) |  | -0.3980 | 6.98e-05 | 4.38e-04 |
| ENSMUSG00000089872 | Rps6kc1 | protein\_coding | 1:190700202-190911770 (-) |  | 0.3300 | 7.01e-05 | 4.39e-04 |
| ENSMUSG00000024737 | Slc15a3 | protein\_coding | 19:10839727-10859362 (+) |  | 0.3150 | 7.01e-05 | 4.39e-04 |
| ENSMUSG00000026798 | Coq4 | protein\_coding | 2:29787493-29797935 (+) |  | -0.5680 | 7.04e-05 | 4.40e-04 |
| ENSMUSG00000041571 | Selenow | protein\_coding | 7:15917208-15922402 (-) |  | 0.4420 | 7.04e-05 | 4.40e-04 |
| ENSMUSG00000021182 | Ccdc88c | protein\_coding | 12:100911523-101029056 (-) |  | 0.3000 | 7.04e-05 | 4.40e-04 |
| ENSMUSG00000001891 | Ugp2 | protein\_coding | 11:21321138-21371201 (-) |  | -0.3140 | 7.10e-05 | 4.44e-04 |
| ENSMUSG00000039703 | Nploc4 | protein\_coding | 11:120380370-120437708 (-) |  | -0.2060 | 7.11e-05 | 4.44e-04 |
| ENSMUSG00000055850 | Rnf181 | protein\_coding | 6:72359714-72366956 (-) |  | 0.1720 | 7.12e-05 | 4.45e-04 |
| ENSMUSG00000061833 | Gm6311 | processed\_pseudogene | 13:75954136-75955013 (+) |  | -0.6190 | 7.13e-05 | 4.45e-04 |
| ENSMUSG00000032217 | Rnf111 | protein\_coding | 9:70425424-70503725 (-) |  | 0.2170 | 7.14e-05 | 4.45e-04 |
| ENSMUSG00000056692 | Ilrun | protein\_coding | 17:27751235-27820648 (-) |  | 0.1460 | 7.15e-05 | 4.46e-04 |
| ENSMUSG00000022844 | Pdia5 | protein\_coding | 16:35397312-35490873 (-) |  | -0.6660 | 7.15e-05 | 4.46e-04 |
| ENSMUSG00000000552 | Zfp385a | protein\_coding | 15:103313895-103340093 (-) |  | 0.2730 | 7.16e-05 | 4.46e-04 |
| ENSMUSG00000041649 | Klf8 | protein\_coding | X:153237466-153396132 (+) |  | 0.4330 | 7.16e-05 | 4.46e-04 |
| ENSMUSG00000045664 | Cdc42ep2 | protein\_coding | 19:5915636-5924816 (-) |  | 0.7770 | 7.20e-05 | 4.48e-04 |
| ENSMUSG00000022440 | C1qtnf6 | protein\_coding | 15:78523346-78531416 (-) |  | -0.6720 | 7.21e-05 | 4.49e-04 |
| ENSMUSG00000032470 | Mras | protein\_coding | 9:99385420-99437381 (-) |  | -1.0300 | 7.27e-05 | 4.52e-04 |
| ENSMUSG00000030031 | Kbtbd8 | protein\_coding | 6:95117240-95129790 (+) |  | -0.4220 | 7.28e-05 | 4.53e-04 |
| ENSMUSG00000054792 | Klhl18 | protein\_coding | 9:110425926-110476694 (-) |  | 0.1790 | 7.35e-05 | 4.57e-04 |
| ENSMUSG00000022314 | Rad21 | protein\_coding | 15:51962240-51991747 (-) |  | -0.1770 | 7.38e-05 | 4.58e-04 |
| ENSMUSG00000029408 | Abcb9 | protein\_coding | 5:124061530-124095798 (-) |  | 0.9850 | 7.39e-05 | 4.59e-04 |
| ENSMUSG00000040990 | Sh3kbp1 | protein\_coding | X:159627272-159978069 (+) |  | 0.1710 | 7.42e-05 | 4.61e-04 |
| ENSMUSG00000100182 | 1810006J02Rik | lncRNA | 1:98131456-98144655 (+) |  | 1.5500 | 7.43e-05 | 4.61e-04 |
| ENSMUSG00000015092 | Edf1 | protein\_coding | 2:25557847-25562082 (+) |  | -0.2330 | 7.43e-05 | 4.61e-04 |
| ENSMUSG00000000605 | Clcn4 | protein\_coding | 7:7282309-7300851 (-) |  | 0.1660 | 7.54e-05 | 4.67e-04 |
| ENSMUSG00000020988 | L2hgdh | protein\_coding | 12:69690433-69724873 (-) |  | -0.4280 | 7.57e-05 | 4.69e-04 |
| ENSMUSG00000031976 | Urb2 | protein\_coding | 8:124021508-124048505 (+) |  | -0.2620 | 7.62e-05 | 4.72e-04 |
| ENSMUSG00000110768 | Gm18541 | processed\_pseudogene | 9:63898265-63898802 (+) |  | -0.4810 | 7.63e-05 | 4.72e-04 |
| ENSMUSG00000109015 | Gm31024 | TEC | 7:28167275-28169627 (-) |  | 0.8190 | 7.63e-05 | 4.72e-04 |
| ENSMUSG00000061032 | Rrp1 | protein\_coding | 10:78400384-78413043 (-) |  | -0.2110 | 7.65e-05 | 4.74e-04 |
| ENSMUSG00000004565 | Pnpla6 | protein\_coding | 8:3515384-3544267 (+) |  | 0.3540 | 7.68e-05 | 4.75e-04 |
| ENSMUSG00000060147 | Serpinb6a | protein\_coding | 13:33917918-34002794 (-) |  | 0.4850 | 7.73e-05 | 4.78e-04 |
| ENSMUSG00000074466 | Gm15417 | lncRNA | 3:89391850-89398779 (+) |  | 0.8850 | 7.73e-05 | 4.78e-04 |
| ENSMUSG00000027699 | Ect2 | protein\_coding | 3:27097222-27153878 (-) |  | -0.2170 | 7.74e-05 | 4.78e-04 |
| ENSMUSG00000031641 | Cbr4 | protein\_coding | 8:61487734-61506694 (+) |  | 0.3470 | 7.75e-05 | 4.79e-04 |
| ENSMUSG00000002058 | Unc119 | protein\_coding | 11:78343482-78349164 (+) |  | 0.4140 | 7.78e-05 | 4.80e-04 |
| ENSMUSG00000028343 | Erp44 | protein\_coding | 4:48193323-48279558 (-) |  | -0.1950 | 7.79e-05 | 4.80e-04 |
| ENSMUSG00000035769 | Xylb | protein\_coding | 9:119357381-119393797 (+) |  | -0.5450 | 7.79e-05 | 4.80e-04 |
| ENSMUSG00000026988 | Wdsub1 | protein\_coding | 2:59852364-59882591 (-) |  | 0.3780 | 7.82e-05 | 4.82e-04 |
| ENSMUSG00000028729 | Ebna1bp2 | protein\_coding | 4:118620799-118627776 (+) |  | -0.3020 | 7.84e-05 | 4.83e-04 |
| ENSMUSG00000002064 | Sdf2 | protein\_coding | 11:78245746-78255496 (+) |  | 0.2420 | 7.90e-05 | 4.86e-04 |
| ENSMUSG00000113204 | Gm46430 | lncRNA | 13:74579358-74617945 (-) |  | 0.3200 | 7.91e-05 | 4.87e-04 |
| ENSMUSG00000041515 | Irf8 | protein\_coding | 8:120736358-120756694 (+) |  | 0.3890 | 7.92e-05 | 4.87e-04 |
| ENSMUSG00000018001 | Cyth3 | protein\_coding | 5:143622447-143710250 (+) |  | 0.4600 | 7.93e-05 | 4.87e-04 |
| ENSMUSG00000031497 | Tnfsf13b | protein\_coding | 8:10006467-10039072 (+) |  | 0.7560 | 7.94e-05 | 4.88e-04 |
| ENSMUSG00000024845 | Tmem134 | protein\_coding | 19:4125934-4132307 (+) |  | 0.1940 | 8.00e-05 | 4.92e-04 |
| ENSMUSG00000061315 | Naca | protein\_coding | 10:128035575-128048637 (+) |  | -0.2390 | 8.02e-05 | 4.92e-04 |
| ENSMUSG00000039263 | Npepl1 | protein\_coding | 2:174110349-174123070 (+) |  | 0.2540 | 8.03e-05 | 4.93e-04 |
| ENSMUSG00000037730 | Mynn | protein\_coding | 3:30602065-30619873 (+) |  | 0.2420 | 8.04e-05 | 4.93e-04 |
| ENSMUSG00000038459 | Abhd17c | protein\_coding | 7:84109356-84151893 (-) |  | -0.2220 | 8.05e-05 | 4.94e-04 |
| ENSMUSG00000073468 | Sft2d1 | protein\_coding | 17:8311102-8327442 (+) |  | 0.2590 | 8.17e-05 | 5.01e-04 |
| ENSMUSG00000021061 | Sptb | protein\_coding | 12:76580488-76710547 (-) |  | 0.6410 | 8.17e-05 | 5.01e-04 |
| ENSMUSG00000027695 | Pld1 | protein\_coding | 3:27938695-28133362 (+) |  | -0.3020 | 8.20e-05 | 5.02e-04 |
| ENSMUSG00000042363 | Lgalsl | protein\_coding | 11:20823576-20831056 (-) |  | -0.5440 | 8.22e-05 | 5.03e-04 |
| ENSMUSG00000001627 | Ifrd1 | protein\_coding | 12:40201567-40248504 (-) |  | -0.3430 | 8.23e-05 | 5.04e-04 |
| ENSMUSG00000026003 | Acadl | protein\_coding | 1:66830839-66863277 (-) |  | -0.2810 | 8.24e-05 | 5.04e-04 |
| ENSMUSG00000021484 | Lman2 | protein\_coding | 13:55343833-55362783 (-) |  | -0.1820 | 8.28e-05 | 5.06e-04 |
| ENSMUSG00000029310 | Nudt9 | protein\_coding | 5:104046306-104065379 (+) |  | -0.2370 | 8.28e-05 | 5.06e-04 |
| ENSMUSG00000091625 | Lsm5 | protein\_coding | 6:56701063-56704710 (-) |  | -0.3440 | 8.29e-05 | 5.06e-04 |
| ENSMUSG00000042284 | Itga1 | protein\_coding | 13:114953096-115101964 (-) |  | 0.3950 | 8.29e-05 | 5.06e-04 |
| ENSMUSG00000070639 | Lrrc8b | protein\_coding | 5:105415775-105490074 (+) |  | -0.2550 | 8.31e-05 | 5.07e-04 |
| ENSMUSG00000037419 | Endod1 | protein\_coding | 9:14353990-14381507 (-) |  | -0.2560 | 8.33e-05 | 5.08e-04 |
| ENSMUSG00000016554 | Eif3d | protein\_coding | 15:77958998-77970813 (-) |  | -0.2170 | 8.35e-05 | 5.09e-04 |
| ENSMUSG00000007029 | Vars | protein\_coding | 17:35000987-35016322 (+) |  | -0.2770 | 8.37e-05 | 5.11e-04 |
| ENSMUSG00000041765 | Ubac2 | protein\_coding | 14:121878620-122021034 (+) |  | 0.1910 | 8.38e-05 | 5.11e-04 |
| ENSMUSG00000026355 | Mcm6 | protein\_coding | 1:128331590-128359664 (-) |  | -0.3210 | 8.48e-05 | 5.17e-04 |
| ENSMUSG00000024042 | Sik1 | protein\_coding | 17:31844250-31855804 (-) |  | 0.8380 | 8.50e-05 | 5.18e-04 |
| ENSMUSG00000023051 | Tarbp2 | protein\_coding | 15:102518192-102523676 (+) |  | -0.2570 | 8.52e-05 | 5.19e-04 |
| ENSMUSG00000042650 | Alkbh5 | protein\_coding | 11:60536381-60558512 (+) |  | -0.2070 | 8.52e-05 | 5.19e-04 |
| ENSMUSG00000079470 | Utp14b | protein\_coding | 1:78658038-78671512 (+) |  | -0.3540 | 8.57e-05 | 5.21e-04 |
| ENSMUSG00000030447 | Cyfip1 | protein\_coding | 7:55841745-55932602 (+) |  | -0.1910 | 8.59e-05 | 5.22e-04 |
| ENSMUSG00000025728 | Pigq | protein\_coding | 17:25926421-25944936 (-) |  | -0.2010 | 8.70e-05 | 5.29e-04 |
| ENSMUSG00000033697 | Arhgap39 | protein\_coding | 15:76723985-76818170 (-) |  | 0.3380 | 8.71e-05 | 5.29e-04 |
| ENSMUSG00000089942 | Pira2 | protein\_coding | 7:3836812-3845051 (-) |  | 0.5640 | 8.74e-05 | 5.31e-04 |
| ENSMUSG00000074102 | Rbm15b | protein\_coding | 9:106880918-106887428 (-) |  | -0.2340 | 8.74e-05 | 5.31e-04 |
| ENSMUSG00000071650 | Ganab | protein\_coding | 19:8898071-8916672 (+) |  | -0.2110 | 8.77e-05 | 5.32e-04 |
| ENSMUSG00000015476 | Prrt1 | protein\_coding | 17:34629533-34633126 (+) |  | 1.6100 | 8.77e-05 | 5.32e-04 |
| ENSMUSG00000021556 | Golm1 | protein\_coding | 13:59634626-59675811 (-) |  | -0.2640 | 8.89e-05 | 5.39e-04 |
| ENSMUSG00000029551 | Psmg3 | protein\_coding | 5:139823592-139826885 (-) |  | -0.3510 | 8.91e-05 | 5.40e-04 |
| ENSMUSG00000027562 | Car2 | protein\_coding | 3:14886273-14900770 (+) |  | 0.9590 | 8.93e-05 | 5.41e-04 |
| ENSMUSG00000024660 | Incenp | protein\_coding | 19:9872296-9899551 (-) |  | -0.1920 | 8.95e-05 | 5.42e-04 |
| ENSMUSG00000041168 | Lonp1 | protein\_coding | 17:56614297-56626887 (-) |  | -0.2880 | 8.98e-05 | 5.44e-04 |
| ENSMUSG00000003527 | Ess2 | protein\_coding | 16:17900709-17911348 (-) |  | 0.2390 | 9.03e-05 | 5.46e-04 |
| ENSMUSG00000028085 | Gatb | protein\_coding | 3:85574119-85655622 (+) |  | -0.3080 | 9.08e-05 | 5.49e-04 |
| ENSMUSG00000090021 | Gm6493 | processed\_pseudogene | 16:23014208-23017038 (-) |  | -0.3570 | 9.18e-05 | 5.55e-04 |
| ENSMUSG00000038517 | Tbkbp1 | protein\_coding | 11:97136171-97151495 (-) |  | 0.2990 | 9.21e-05 | 5.56e-04 |
| ENSMUSG00000037514 | Pank2 | protein\_coding | 2:131262495-131299188 (+) |  | 0.1580 | 9.26e-05 | 5.59e-04 |
| ENSMUSG00000062980 | Cped1 | protein\_coding | 6:21985916-22256404 (+) |  | -0.8320 | 9.27e-05 | 5.60e-04 |
| ENSMUSG00000079884 | Gm10698 | processed\_pseudogene | 9:33728247-33728850 (+) |  | -0.2850 | 9.30e-05 | 5.61e-04 |
| ENSMUSG00000111535 | Gm35154 | lncRNA | 10:44598720-44689081 (+) |  | 0.8910 | 9.32e-05 | 5.62e-04 |
| ENSMUSG00000020516 | Rps6kb1 | protein\_coding | 11:86498871-86544805 (-) |  | -0.2180 | 9.34e-05 | 5.63e-04 |
| ENSMUSG00000036580 | Spg20 | protein\_coding | 3:55112108-55137322 (+) |  | 0.4290 | 9.34e-05 | 5.63e-04 |
| ENSMUSG00000029066 | Mrpl20 | protein\_coding | 4:155802878-155809975 (+) |  | -0.3540 | 9.35e-05 | 5.63e-04 |
| ENSMUSG00000024754 | Cemip2 | protein\_coding | 19:21778342-21858360 (+) |  | -0.2640 | 9.38e-05 | 5.65e-04 |
| ENSMUSG00000081219 | Bambi-ps1 | processed\_pseudogene | 2:122466935-122467526 (-) |  | -0.7740 | 9.40e-05 | 5.66e-04 |
| ENSMUSG00000075704 | Txnrd2 | protein\_coding | 16:18426384-18479073 (+) |  | -0.3050 | 9.41e-05 | 5.67e-04 |
| ENSMUSG00000035299 | Mid1 | protein\_coding | X:169685199-170005736 (+) |  | -0.8650 | 9.43e-05 | 5.67e-04 |
| ENSMUSG00000063894 | Zkscan8 | protein\_coding | 13:21513222-21531120 (-) |  | 0.2890 | 9.48e-05 | 5.70e-04 |
| ENSMUSG00000024590 | Lmnb1 | protein\_coding | 18:56707813-56753424 (+) |  | -0.2620 | 9.52e-05 | 5.72e-04 |
| ENSMUSG00000090523 | Gypc | protein\_coding | 18:32528322-32560034 (-) |  | -0.2450 | 9.52e-05 | 5.72e-04 |
| ENSMUSG00000020496 | Rnf187 | protein\_coding | 11:58932288-58938916 (-) |  | -0.1660 | 9.54e-05 | 5.73e-04 |
| ENSMUSG00000002603 | Tgfb1 | protein\_coding | 7:25687002-25705077 (+) |  | 0.2060 | 9.55e-05 | 5.74e-04 |
| ENSMUSG00000027423 | Snx5 | protein\_coding | 2:144250123-144270906 (-) |  | -0.2650 | 9.57e-05 | 5.74e-04 |
| ENSMUSG00000021775 | Nr1d2 | protein\_coding | 14:18204054-18239127 (-) |  | 0.4730 | 9.59e-05 | 5.75e-04 |
| ENSMUSG00000070697 | Utp3 | protein\_coding | 5:88554462-88556090 (+) |  | -0.1950 | 9.61e-05 | 5.76e-04 |
| ENSMUSG00000031299 | Pdha1 | protein\_coding | X:160122209-160138413 (-) |  | -0.1670 | 9.66e-05 | 5.79e-04 |
| ENSMUSG00000030629 | Zfand6 | protein\_coding | 7:84613766-84689959 (-) |  | 0.2390 | 9.68e-05 | 5.80e-04 |
| ENSMUSG00000029101 | Rgs12 | protein\_coding | 5:34949445-35039644 (+) |  | 0.3740 | 9.68e-05 | 5.80e-04 |
| ENSMUSG00000000811 | Txnrd3 | protein\_coding | 6:89643988-89675529 (+) |  | 0.5730 | 9.72e-05 | 5.82e-04 |
| ENSMUSG00000086922 | Gm13835 | processed\_pseudogene | 6:31141979-31142663 (-) |  | -0.2850 | 9.76e-05 | 5.84e-04 |
| ENSMUSG00000057561 | Eif1a | protein\_coding | 18:46597701-46616456 (+) |  | -0.3650 | 9.77e-05 | 5.84e-04 |
| ENSMUSG00000035202 | Lars2 | protein\_coding | 9:123366927-123462666 (+) |  | -0.3320 | 9.78e-05 | 5.85e-04 |
| ENSMUSG00000019990 | Pde7b | protein\_coding | 10:20398004-20725078 (-) |  | 0.4920 | 9.79e-05 | 5.85e-04 |
| ENSMUSG00000029254 | Stap1 | protein\_coding | 5:86071746-86106125 (+) |  | 0.5000 | 9.79e-05 | 5.85e-04 |
| ENSMUSG00000025283 | Sat1 | protein\_coding | X:155213132-155216449 (-) |  | 0.3510 | 9.82e-05 | 5.86e-04 |
| ENSMUSG00000025236 | Adpgk | protein\_coding | 9:59291558-59324052 (+) |  | -0.1870 | 9.85e-05 | 5.88e-04 |
| ENSMUSG00000074671 | Tspyl3 | protein\_coding | 2:153222370-153225441 (-) |  | 0.4320 | 9.87e-05 | 5.89e-04 |
| ENSMUSG00000025355 | Mmp19 | protein\_coding | 10:128790910-128800824 (+) |  | -0.4430 | 9.93e-05 | 5.92e-04 |
| ENSMUSG00000025439 | Clns1a | protein\_coding | 7:97696634-97720796 (+) |  | -0.1810 | 9.96e-05 | 5.94e-04 |
| ENSMUSG00000022962 | Gart | protein\_coding | 16:91621186-91646952 (-) |  | -0.2580 | 9.97e-05 | 5.94e-04 |
| ENSMUSG00000028602 | Tnfrsf8 | protein\_coding | 4:145267137-145315164 (-) |  | 0.6170 | 1.00e-04 | 5.96e-04 |
| ENSMUSG00000050846 | Zfp623 | protein\_coding | 15:75940952-75949400 (+) |  | 0.3400 | 1.00e-04 | 5.98e-04 |
| ENSMUSG00000020661 | Dnmt3a | protein\_coding | 12:3806007-3914443 (+) |  | 0.3690 | 1.00e-04 | 5.98e-04 |
| ENSMUSG00000028796 | Phc2 | protein\_coding | 4:128654702-128752881 (+) |  | -0.1910 | 1.01e-04 | 5.99e-04 |
| ENSMUSG00000048163 | Selplg | protein\_coding | 5:113818536-113832644 (-) |  | 0.1690 | 1.01e-04 | 6.01e-04 |
| ENSMUSG00000041488 | Stx3 | protein\_coding | 19:11775118-11819403 (-) |  | 0.2990 | 1.02e-04 | 6.03e-04 |
| ENSMUSG00000025858 | Get4 | protein\_coding | 5:139252324-139270051 (+) |  | -0.2070 | 1.02e-04 | 6.03e-04 |
| ENSMUSG00000021666 | Gfm2 | protein\_coding | 13:97137937-97181195 (+) |  | -0.2730 | 1.02e-04 | 6.03e-04 |
| ENSMUSG00000024858 | Grk2 | protein\_coding | 19:4286001-4306222 (-) |  | 0.1140 | 1.02e-04 | 6.05e-04 |
| ENSMUSG00000024383 | Map3k2 | protein\_coding | 18:32163089-32236751 (+) |  | 0.1760 | 1.02e-04 | 6.05e-04 |
| ENSMUSG00000033762 | Recql4 | protein\_coding | 15:76703553-76710548 (-) |  | -0.3930 | 1.02e-04 | 6.05e-04 |
| ENSMUSG00000033701 | Acbd6 | protein\_coding | 1:155558120-155691330 (+) |  | -0.2450 | 1.03e-04 | 6.10e-04 |
| ENSMUSG00000027765 | P2ry1 | protein\_coding | 3:61002795-61008982 (+) |  | -0.3480 | 1.03e-04 | 6.10e-04 |
| ENSMUSG00000014503 | Pkd2l2 | protein\_coding | 18:34408489-34444116 (+) |  | 0.3420 | 1.03e-04 | 6.11e-04 |
| ENSMUSG00000064127 | Med14 | protein\_coding | X:12675369-12762073 (-) |  | -0.2040 | 1.03e-04 | 6.11e-04 |
| ENSMUSG00000044573 | Acp1 | protein\_coding | 12:30893326-30911589 (-) |  | -0.2400 | 1.04e-04 | 6.15e-04 |
| ENSMUSG00000033793 | Atp6v1h | protein\_coding | 1:5070018-5162529 (+) |  | -0.2760 | 1.04e-04 | 6.16e-04 |
| ENSMUSG00000024758 | Rtn3 | protein\_coding | 19:7425901-7483281 (-) |  | -0.1840 | 1.04e-04 | 6.17e-04 |
| ENSMUSG00000025076 | Casp7 | protein\_coding | 19:56397129-56442344 (+) |  | -0.2770 | 1.04e-04 | 6.18e-04 |
| ENSMUSG00000073792 | Alg6 | protein\_coding | 4:99715664-99763460 (+) |  | -0.3160 | 1.05e-04 | 6.19e-04 |
| ENSMUSG00000029364 | Wsb2 | protein\_coding | 5:117357304-117378601 (+) |  | -0.2290 | 1.06e-04 | 6.28e-04 |
| ENSMUSG00000002833 | Hdgfl2 | protein\_coding | 17:56079634-56100607 (+) |  | -0.1910 | 1.07e-04 | 6.33e-04 |
| ENSMUSG00000030805 | Stx4a | protein\_coding | 7:127824294-127849019 (+) |  | 0.2050 | 1.08e-04 | 6.35e-04 |
| ENSMUSG00000026750 | Psmb7 | protein\_coding | 2:38588036-38644087 (-) |  | -0.2730 | 1.08e-04 | 6.35e-04 |
| ENSMUSG00000020280 | Pus10 | protein\_coding | 11:23665674-23732876 (+) |  | -0.2180 | 1.08e-04 | 6.39e-04 |
| ENSMUSG00000043323 | Fbrsl1 | protein\_coding | 5:110361754-110448602 (-) |  | 0.2540 | 1.08e-04 | 6.40e-04 |
| ENSMUSG00000027555 | Car13 | protein\_coding | 3:14641727-14663002 (+) |  | -0.5700 | 1.09e-04 | 6.40e-04 |
| ENSMUSG00000025860 | Xiap | protein\_coding | X:42059679-42109656 (+) |  | 0.1740 | 1.10e-04 | 6.48e-04 |
| ENSMUSG00000041438 | Utp4 | protein\_coding | 8:106893636-106923088 (+) |  | -0.2920 | 1.10e-04 | 6.48e-04 |
| ENSMUSG00000023068 | Nus1 | protein\_coding | 10:52417547-52440183 (+) |  | -0.2360 | 1.11e-04 | 6.52e-04 |
| ENSMUSG00000032198 | Dock6 | protein\_coding | 9:21799860-21852635 (-) |  | 0.5030 | 1.11e-04 | 6.56e-04 |
| ENSMUSG00000096544 | Gm4617 | processed\_pseudogene | 3:124385667-124385997 (+) |  | -0.2940 | 1.12e-04 | 6.56e-04 |
| ENSMUSG00000096981 | Gm16845 | lncRNA | 9:22071002-22086122 (+) |  | 0.6620 | 1.12e-04 | 6.56e-04 |
| ENSMUSG00000052033 | Pfdn4 | protein\_coding | 2:170496428-170519123 (+) |  | -0.4550 | 1.12e-04 | 6.57e-04 |
| ENSMUSG00000015749 | Anp32e | protein\_coding | 3:95929246-95947390 (+) |  | -0.1890 | 1.12e-04 | 6.57e-04 |
| ENSMUSG00000001700 | Gramd3 | protein\_coding | 18:56400337-56503792 (+) |  | 0.3560 | 1.12e-04 | 6.57e-04 |
| ENSMUSG00000040312 | Cchcr1 | protein\_coding | 17:35517100-35531015 (+) |  | -0.4530 | 1.12e-04 | 6.58e-04 |
| ENSMUSG00000030884 | Uqcrc2 | protein\_coding | 7:120635176-120659524 (+) |  | -0.2220 | 1.12e-04 | 6.59e-04 |
| ENSMUSG00000029414 | Kntc1 | protein\_coding | 5:123749716-123821593 (+) |  | -0.3800 | 1.13e-04 | 6.61e-04 |
| ENSMUSG00000049307 | Fut4 | protein\_coding | 9:14748320-14752393 (-) |  | 0.3030 | 1.13e-04 | 6.63e-04 |
| ENSMUSG00000058076 | Sdhc | protein\_coding | 1:171127165-171150603 (-) |  | -0.1870 | 1.13e-04 | 6.63e-04 |
| ENSMUSG00000015882 | Lcorl | protein\_coding | 5:45697181-45857615 (-) |  | 0.2610 | 1.13e-04 | 6.64e-04 |
| ENSMUSG00000004937 | Sgta | protein\_coding | 10:81044075-81060181 (-) |  | -0.1880 | 1.15e-04 | 6.71e-04 |
| ENSMUSG00000074274 | D930028M14Rik | lncRNA | 7:25152457-25156627 (+) |  | 1.1800 | 1.15e-04 | 6.73e-04 |
| ENSMUSG00000036052 | Dnajb5 | protein\_coding | 4:42949814-42959425 (+) |  | 0.4020 | 1.15e-04 | 6.73e-04 |
| ENSMUSG00000027893 | Ahcyl1 | protein\_coding | 3:107663118-107696560 (-) |  | -0.1580 | 1.16e-04 | 6.76e-04 |
| ENSMUSG00000029363 | Rfc5 | protein\_coding | 5:117378103-117389047 (-) |  | -0.2160 | 1.16e-04 | 6.77e-04 |
| ENSMUSG00000071041 | Impdh2-ps | processed\_pseudogene | 8:100030558-100032102 (+) |  | -0.3100 | 1.17e-04 | 6.84e-04 |
| ENSMUSG00000019256 | Ahr | protein\_coding | 12:35497974-35535038 (-) |  | -0.3320 | 1.17e-04 | 6.85e-04 |
| ENSMUSG00000026127 | Imp4 | protein\_coding | 1:34439851-34449356 (+) |  | -0.1880 | 1.17e-04 | 6.85e-04 |
| ENSMUSG00000064043 | Trerf1 | lncRNA | 17:47140875-47361958 (+) |  | 0.3250 | 1.18e-04 | 6.90e-04 |
| ENSMUSG00000025026 | Add3 | protein\_coding | 19:53140443-53247399 (+) |  | 0.2560 | 1.18e-04 | 6.91e-04 |
| ENSMUSG00000028261 | Ndufaf4 | protein\_coding | 4:24898083-24905001 (+) |  | -0.3140 | 1.19e-04 | 6.95e-04 |
| ENSMUSG00000074476 | Spc24 | protein\_coding | 9:21755442-21760303 (-) |  | -0.2500 | 1.20e-04 | 7.02e-04 |
| ENSMUSG00000064359 | mt-Tg | Mt\_tRNA | MT:9391-9458 (+) |  | -0.9850 | 1.21e-04 | 7.03e-04 |
| ENSMUSG00000033706 | Smyd5 | protein\_coding | 6:85431989-85446435 (+) |  | -0.3140 | 1.21e-04 | 7.04e-04 |
| ENSMUSG00000016493 | Cd46 | protein\_coding | 1:195036826-195092249 (-) |  | 1.0600 | 1.21e-04 | 7.05e-04 |
| ENSMUSG00000023072 | Cep89 | protein\_coding | 7:35397035-35438689 (+) |  | -0.3090 | 1.21e-04 | 7.05e-04 |
| ENSMUSG00000022452 | Smdt1 | protein\_coding | 15:82338959-82349091 (+) |  | -0.2660 | 1.21e-04 | 7.06e-04 |
| ENSMUSG00000022358 | Fbxo32 | protein\_coding | 15:58175879-58214932 (-) |  | 0.8100 | 1.21e-04 | 7.07e-04 |
| ENSMUSG00000029145 | Eif2b4 | protein\_coding | 5:31187558-31193430 (-) |  | -0.2400 | 1.22e-04 | 7.08e-04 |
| ENSMUSG00000031816 | Mthfsd | protein\_coding | 8:121091628-121108392 (-) |  | -0.2350 | 1.23e-04 | 7.16e-04 |
| ENSMUSG00000022721 | Trmt2a | protein\_coding | 16:18248679-18254772 (+) |  | -0.1920 | 1.23e-04 | 7.17e-04 |
| ENSMUSG00000020057 | Dram1 | protein\_coding | 10:88322804-88379080 (-) |  | 0.2360 | 1.24e-04 | 7.20e-04 |
| ENSMUSG00000023286 | Ube2j2 | protein\_coding | 4:155943831-155959604 (+) |  | -0.1700 | 1.24e-04 | 7.21e-04 |
| ENSMUSG00000091955 | Gm9844 | protein\_coding | 7:24862213-24862697 (+) |  | -0.3510 | 1.24e-04 | 7.22e-04 |
| ENSMUSG00000066632 | Pgk1-rs7 | processed\_pseudogene | 12:10898986-10900240 (-) |  | -0.5630 | 1.24e-04 | 7.22e-04 |
| ENSMUSG00000062743 | Zfp677 | protein\_coding | 17:21383748-21399265 (+) |  | 0.4250 | 1.25e-04 | 7.22e-04 |
| ENSMUSG00000026853 | Crat | protein\_coding | 2:30400471-30415813 (-) |  | -0.2280 | 1.25e-04 | 7.23e-04 |
| ENSMUSG00000069301 | H2ac11 | protein\_coding | 13:22042460-22042944 (-) |  | 0.7490 | 1.26e-04 | 7.28e-04 |
| ENSMUSG00000020901 | Pik3r5 | protein\_coding | 11:68432121-68497849 (+) |  | -0.2960 | 1.26e-04 | 7.31e-04 |
| ENSMUSG00000043923 | Ccdc84 | protein\_coding | 9:44410159-44418569 (-) |  | 0.3410 | 1.27e-04 | 7.33e-04 |
| ENSMUSG00000034957 | Cebpa | protein\_coding | 7:35119293-35121928 (+) |  | 0.3450 | 1.27e-04 | 7.37e-04 |
| ENSMUSG00000001029 | Icam2 | protein\_coding | 11:106377656-106388075 (-) |  | -0.2540 | 1.28e-04 | 7.39e-04 |
| ENSMUSG00000021177 | Tdp1 | protein\_coding | 12:99884517-99955219 (+) |  | -0.2610 | 1.28e-04 | 7.42e-04 |
| ENSMUSG00000054079 | Utp18 | protein\_coding | 11:93859243-93885766 (-) |  | -0.2580 | 1.29e-04 | 7.45e-04 |
| ENSMUSG00000073771 | Btbd19 | protein\_coding | 4:117119219-117125725 (-) |  | -0.4410 | 1.29e-04 | 7.46e-04 |
| ENSMUSG00000089838 | Gm2962 | processed\_pseudogene | 1:170925309-170925644 (-) |  | 0.4400 | 1.29e-04 | 7.47e-04 |
| ENSMUSG00000024369 | Nelfe | protein\_coding | 17:34850391-34856372 (+) |  | -0.2610 | 1.30e-04 | 7.50e-04 |
| ENSMUSG00000079557 | March2 | protein\_coding | 17:33685692-33718670 (-) |  | 0.2160 | 1.30e-04 | 7.52e-04 |
| ENSMUSG00000022100 | Xpo7 | protein\_coding | 14:70654246-70766628 (-) |  | -0.1890 | 1.31e-04 | 7.55e-04 |
| ENSMUSG00000021134 | Srsf5 | protein\_coding | 12:80945504-80950507 (+) |  | 0.1910 | 1.31e-04 | 7.55e-04 |
| ENSMUSG00000056258 | Kcnq3 | protein\_coding | 15:65986387-66286642 (-) |  | 0.5140 | 1.32e-04 | 7.59e-04 |
| ENSMUSG00000041936 | Agrn | protein\_coding | 4:156165290-156197488 (-) |  | 0.5660 | 1.32e-04 | 7.59e-04 |
| ENSMUSG00000079197 | Psme2 | protein\_coding | 14:55587441-55591113 (-) |  | -0.2490 | 1.32e-04 | 7.59e-04 |
| ENSMUSG00000022529 | Zfp263 | protein\_coding | 16:3744093-3750790 (+) |  | 0.1600 | 1.33e-04 | 7.64e-04 |
| ENSMUSG00000107476 | Zfp862-ps | transcribed\_unitary\_pseudogene | 6:48504337-48534832 (+) |  | 0.4780 | 1.34e-04 | 7.69e-04 |
| ENSMUSG00000040675 | Mthfd1l | protein\_coding | 10:3973118-4167081 (+) |  | -0.1980 | 1.34e-04 | 7.69e-04 |
| ENSMUSG00000081792 | Anp32b-ps1 | processed\_pseudogene | 4:143389659-143390416 (+) |  | -0.3490 | 1.34e-04 | 7.71e-04 |
| ENSMUSG00000038650 | Rnh1 | protein\_coding | 7:141160326-141172857 (-) |  | -0.2810 | 1.35e-04 | 7.73e-04 |
| ENSMUSG00000093904 | Tomm20 | protein\_coding | 8:126930667-126945844 (-) |  | -0.2690 | 1.36e-04 | 7.80e-04 |
| ENSMUSG00000033099 | Nol12 | protein\_coding | 15:78934933-78943638 (+) |  | -0.2570 | 1.36e-04 | 7.82e-04 |
| ENSMUSG00000035478 | Mbd3 | protein\_coding | 10:80392539-80399550 (-) |  | -0.1940 | 1.37e-04 | 7.86e-04 |
| ENSMUSG00000015966 | Il17rb | protein\_coding | 14:29996135-30008896 (-) |  | 0.8090 | 1.37e-04 | 7.87e-04 |
| ENSMUSG00000024563 | Smad2 | protein\_coding | 18:76241580-76310963 (+) |  | 0.1610 | 1.37e-04 | 7.88e-04 |
| ENSMUSG00000072082 | Ccnf | protein\_coding | 17:24222198-24251484 (-) |  | -0.2190 | 1.37e-04 | 7.88e-04 |
| ENSMUSG00000004221 | Ikbkg | protein\_coding | X:74393290-74453854 (+) |  | 0.2070 | 1.38e-04 | 7.89e-04 |
| ENSMUSG00000075420 | Smim6 | protein\_coding | 11:115912017-115913920 (+) |  | 0.9190 | 1.38e-04 | 7.90e-04 |
| ENSMUSG00000047675 | Rps8 | protein\_coding | 4:117153827-117156243 (-) |  | -0.2050 | 1.38e-04 | 7.92e-04 |
| ENSMUSG00000072596 | Ear2 | protein\_coding | 14:44102654-44103534 (+) |  | 1.0500 | 1.40e-04 | 7.99e-04 |
| ENSMUSG00000032601 | Prkar2a | protein\_coding | 9:108689314-108750436 (+) |  | -0.1880 | 1.40e-04 | 7.99e-04 |
| ENSMUSG00000056515 | Rab31 | protein\_coding | 17:65651729-65772752 (-) |  | 0.1960 | 1.40e-04 | 8.00e-04 |
| ENSMUSG00000051344 | Plekhm3 | protein\_coding | 1:64785983-64956824 (-) |  | 0.3290 | 1.41e-04 | 8.05e-04 |
| ENSMUSG00000047368 | Abhd17b | protein\_coding | 19:21653185-21685637 (+) |  | -0.2540 | 1.41e-04 | 8.08e-04 |
| ENSMUSG00000036526 | Card11 | protein\_coding | 5:140872990-141000582 (-) |  | 0.7380 | 1.42e-04 | 8.10e-04 |
| ENSMUSG00000043483 | Gm6863 | processed\_pseudogene | 12:96922423-96923058 (-) |  | -0.2730 | 1.42e-04 | 8.11e-04 |
| ENSMUSG00000050910 | Cdr2l | protein\_coding | 11:115381916-115396132 (+) |  | 1.2100 | 1.42e-04 | 8.13e-04 |
| ENSMUSG00000022407 | Adsl | protein\_coding | 15:80948490-80970946 (+) |  | -0.2620 | 1.43e-04 | 8.14e-04 |
| ENSMUSG00000028452 | Vcp | protein\_coding | 4:42979963-43000507 (-) |  | -0.2430 | 1.43e-04 | 8.15e-04 |
| ENSMUSG00000023903 | Mmp25 | protein\_coding | 17:23628311-23645277 (-) |  | -0.9870 | 1.44e-04 | 8.20e-04 |
| ENSMUSG00000007038 | Neu1 | protein\_coding | 17:34931253-34935953 (+) |  | -0.2340 | 1.44e-04 | 8.20e-04 |
| ENSMUSG00000024962 | Vegfb | protein\_coding | 19:6982473-6987651 (-) |  | -0.3740 | 1.44e-04 | 8.21e-04 |
| ENSMUSG00000025141 | Myadml2 | protein\_coding | 11:120646031-120648158 (-) |  | 1.0600 | 1.44e-04 | 8.21e-04 |
| ENSMUSG00000055044 | Pdlim1 | protein\_coding | 19:40221173-40271842 (-) |  | 0.1830 | 1.45e-04 | 8.23e-04 |
| ENSMUSG00000024556 | Me2 | protein\_coding | 18:73769903-73815449 (-) |  | -0.2240 | 1.45e-04 | 8.26e-04 |
| ENSMUSG00000040365 | Trim41 | protein\_coding | 11:48806404-48817353 (-) |  | 0.1510 | 1.45e-04 | 8.26e-04 |
| ENSMUSG00000070705 | Eid2b | protein\_coding | 7:28277739-28279489 (+) |  | 0.4140 | 1.46e-04 | 8.28e-04 |
| ENSMUSG00000062526 | Mppe1 | protein\_coding | 18:67225043-67245830 (-) |  | 0.1920 | 1.46e-04 | 8.28e-04 |
| ENSMUSG00000020949 | Fkbp3 | protein\_coding | 12:65062424-65074007 (-) |  | -0.3090 | 1.46e-04 | 8.30e-04 |
| ENSMUSG00000030600 | Lrfn1 | protein\_coding | 7:28451980-28468242 (+) |  | 0.5590 | 1.46e-04 | 8.32e-04 |
| ENSMUSG00000087403 | Kantr | protein\_coding | X:152294828-152327495 (-) |  | 0.3390 | 1.47e-04 | 8.32e-04 |
| ENSMUSG00000040687 | Madd | protein\_coding | 2:91137360-91183837 (-) |  | 0.2560 | 1.47e-04 | 8.35e-04 |
| ENSMUSG00000048755 | Mcat | protein\_coding | 15:83546797-83563787 (-) |  | -0.3230 | 1.47e-04 | 8.35e-04 |
| ENSMUSG00000001847 | Rac1 | protein\_coding | 5:143503634-143528036 (-) |  | -0.1810 | 1.47e-04 | 8.35e-04 |
| ENSMUSG00000081723 | Gm15931 | unprocessed\_pseudogene | 7:4274189-4282645 (+) |  | 1.2800 | 1.48e-04 | 8.37e-04 |
| ENSMUSG00000012443 | Kif11 | protein\_coding | 19:37376403-37421859 (+) |  | -0.2260 | 1.48e-04 | 8.39e-04 |
| ENSMUSG00000021577 | Sdha | protein\_coding | 13:74322254-74350280 (-) |  | -0.1930 | 1.49e-04 | 8.42e-04 |
| ENSMUSG00000038965 | Ube2l3 | protein\_coding | 16:17152013-17202649 (-) |  | -0.1850 | 1.49e-04 | 8.42e-04 |
| ENSMUSG00000040997 | Abhd4 | protein\_coding | 14:54254188-54270637 (+) |  | 0.4350 | 1.49e-04 | 8.45e-04 |
| ENSMUSG00000000171 | Sdhd | protein\_coding | 9:50596357-50603812 (-) |  | -0.2230 | 1.50e-04 | 8.46e-04 |
| ENSMUSG00000022370 | Mrpl13 | protein\_coding | 15:55534094-55557748 (-) |  | -0.2730 | 1.50e-04 | 8.46e-04 |
| ENSMUSG00000061111 | Mcrip1 | protein\_coding | 11:120542888-120549727 (-) |  | 0.2820 | 1.50e-04 | 8.49e-04 |
| ENSMUSG00000024521 | Pmaip1 | protein\_coding | 18:66458533-66465565 (+) |  | -0.7270 | 1.50e-04 | 8.49e-04 |
| ENSMUSG00000028645 | Slc2a1 | protein\_coding | 4:119108711-119137983 (+) |  | -0.2820 | 1.50e-04 | 8.49e-04 |
| ENSMUSG00000030286 | Emc3 | protein\_coding | 6:113514874-113531652 (-) |  | -0.1530 | 1.50e-04 | 8.49e-04 |
| ENSMUSG00000024696 | Lpxn | protein\_coding | 19:12796193-12833807 (+) |  | -0.2610 | 1.51e-04 | 8.50e-04 |
| ENSMUSG00000023967 | Mrps18a | protein\_coding | 17:46110986-46128910 (+) |  | -0.2470 | 1.53e-04 | 8.65e-04 |
| ENSMUSG00000024772 | Ehd1 | protein\_coding | 19:6276725-6300096 (+) |  | 0.7050 | 1.54e-04 | 8.70e-04 |
| ENSMUSG00000039242 | B3galnt2 | protein\_coding | 13:13954469-13999103 (+) |  | -0.2090 | 1.55e-04 | 8.73e-04 |
| ENSMUSG00000006005 | Tpr | protein\_coding | 1:150392838-150449935 (+) |  | -0.1970 | 1.55e-04 | 8.75e-04 |
| ENSMUSG00000009079 | Ewsr1 | protein\_coding | 11:5069689-5099266 (-) |  | -0.1500 | 1.56e-04 | 8.76e-04 |
| ENSMUSG00000030122 | Ptms | protein\_coding | 6:124913681-124920103 (-) |  | 0.3380 | 1.56e-04 | 8.79e-04 |
| ENSMUSG00000050812 | Ecpas | protein\_coding | 4:58798911-58912749 (-) |  | -0.2010 | 1.57e-04 | 8.81e-04 |
| ENSMUSG00000033159 | Cnppd1 | protein\_coding | 1:75134554-75142711 (-) |  | 0.1660 | 1.57e-04 | 8.82e-04 |
| ENSMUSG00000013701 | Timm23 | protein\_coding | 14:32180162-32201898 (-) |  | -0.2900 | 1.57e-04 | 8.82e-04 |
| ENSMUSG00000038866 | Zcchc2 | protein\_coding | 1:105990406-106034074 (+) |  | 0.2460 | 1.57e-04 | 8.83e-04 |
| ENSMUSG00000022561 | Gpaa1 | protein\_coding | 15:76331231-76334907 (+) |  | -0.2040 | 1.57e-04 | 8.84e-04 |
| ENSMUSG00000000563 | Atp5pb | protein\_coding | 3:105942698-105960099 (-) |  | -0.2490 | 1.57e-04 | 8.84e-04 |
| ENSMUSG00000026698 | Pigc | protein\_coding | 1:161969186-161973435 (+) |  | 0.2570 | 1.59e-04 | 8.92e-04 |
| ENSMUSG00000026639 | Lamb3 | protein\_coding | 1:193207699-193343878 (+) |  | 0.8720 | 1.59e-04 | 8.95e-04 |
| ENSMUSG00000017561 | Crlf3 | protein\_coding | 11:80046493-80080991 (-) |  | 0.1750 | 1.60e-04 | 8.95e-04 |
| ENSMUSG00000020827 | Mink1 | protein\_coding | 11:70562881-70614483 (+) |  | 0.3420 | 1.60e-04 | 8.95e-04 |
| ENSMUSG00000035671 | Zswim4 | protein\_coding | 8:84210678-84237055 (-) |  | 0.2860 | 1.60e-04 | 8.96e-04 |
| ENSMUSG00000006050 | Sra1 | protein\_coding | 18:36666681-36670815 (-) |  | -0.2060 | 1.60e-04 | 8.97e-04 |
| ENSMUSG00000036093 | Arl5a | protein\_coding | 2:52397951-52424901 (-) |  | -0.2430 | 1.60e-04 | 8.97e-04 |
| ENSMUSG00000085882 | 2610507I01Rik | lncRNA | 11:59199836-59202385 (-) |  | 0.3160 | 1.61e-04 | 9.04e-04 |
| ENSMUSG00000044141 | E130201H02Rik | processed\_pseudogene | 7:120597625-120598475 (+) |  | -1.1200 | 1.62e-04 | 9.05e-04 |
| ENSMUSG00000111964 | Gm8942 | processed\_pseudogene | 10:115269978-115270377 (+) |  | 0.4360 | 1.62e-04 | 9.05e-04 |
| ENSMUSG00000021495 | Fam193b | protein\_coding | 13:55539316-55571120 (-) |  | 0.2620 | 1.62e-04 | 9.06e-04 |
| ENSMUSG00000020010 | Vnn3 | protein\_coding | 10:23851462-23869843 (+) |  | 1.1300 | 1.62e-04 | 9.08e-04 |
| ENSMUSG00000106895 | Gm4754 | processed\_pseudogene | 5:41342123-41342931 (+) |  | -0.7380 | 1.63e-04 | 9.11e-04 |
| ENSMUSG00000040123 | Zmym5 | protein\_coding | 14:56790585-56811716 (-) |  | 0.2480 | 1.64e-04 | 9.13e-04 |
| ENSMUSG00000032187 | Smarca4 | protein\_coding | 9:21616169-21704230 (+) |  | -0.2470 | 1.64e-04 | 9.16e-04 |
| ENSMUSG00000031601 | Cnot7 | protein\_coding | 8:40492540-40515847 (-) |  | -0.1980 | 1.65e-04 | 9.19e-04 |
| ENSMUSG00000031851 | Ntpcr | protein\_coding | 8:125729963-125748235 (+) |  | -0.2340 | 1.66e-04 | 9.24e-04 |
| ENSMUSG00000001018 | Snapin | protein\_coding | 3:90488026-90491033 (-) |  | 0.2530 | 1.66e-04 | 9.28e-04 |
| ENSMUSG00000020230 | Prmt2 | protein\_coding | 10:76207222-76237865 (-) |  | 0.7650 | 1.67e-04 | 9.30e-04 |
| ENSMUSG00000107352 | Gm43660 | lncRNA | 5:31560812-31569072 (-) |  | 0.4880 | 1.68e-04 | 9.34e-04 |
| ENSMUSG00000043953 | Ccrl2 | protein\_coding | 9:111054486-111057519 (-) |  | 0.8780 | 1.68e-04 | 9.34e-04 |
| ENSMUSG00000005886 | Ncoa2 | protein\_coding | 1:13139105-13374083 (-) |  | 0.2220 | 1.68e-04 | 9.34e-04 |
| ENSMUSG00000004748 | Mtfp1 | protein\_coding | 11:4091480-4095445 (-) |  | -0.6360 | 1.68e-04 | 9.36e-04 |
| ENSMUSG00000016194 | Hsd11b1 | protein\_coding | 1:193221634-193264075 (-) |  | -0.6530 | 1.69e-04 | 9.39e-04 |
| ENSMUSG00000037070 | Rbmxl1 | protein\_coding | 8:78505269-78508898 (-) |  | -0.1980 | 1.69e-04 | 9.41e-04 |
| ENSMUSG00000025821 | Zfp282 | protein\_coding | 6:47877204-47908485 (+) |  | 0.2170 | 1.69e-04 | 9.41e-04 |
| ENSMUSG00000050107 | Haspin | protein\_coding | 11:73135485-73138294 (-) |  | -0.2160 | 1.70e-04 | 9.45e-04 |
| ENSMUSG00000084964 | Gm15503 | lncRNA | 7:128408968-128412341 (-) |  | 0.8540 | 1.70e-04 | 9.46e-04 |
| ENSMUSG00000030265 | Kras | protein\_coding | 6:145216699-145250239 (-) |  | -0.1680 | 1.71e-04 | 9.47e-04 |
| ENSMUSG00000028567 | Txndc12 | protein\_coding | 4:108834601-108862127 (+) |  | -0.2480 | 1.71e-04 | 9.47e-04 |
| ENSMUSG00000029642 | Polr1d | protein\_coding | 5:147077050-147111597 (+) |  | -0.3020 | 1.71e-04 | 9.47e-04 |
| ENSMUSG00000007338 | Mrpl49 | protein\_coding | 19:6053622-6057785 (-) |  | -0.2350 | 1.71e-04 | 9.47e-04 |
| ENSMUSG00000023990 | Tfeb | protein\_coding | 17:47737030-47792419 (+) |  | 0.2900 | 1.71e-04 | 9.47e-04 |
| ENSMUSG00000046169 | Adamts6 | protein\_coding | 13:104287835-104496695 (+) |  | 0.8300 | 1.71e-04 | 9.47e-04 |
| ENSMUSG00000022503 | Nubp1 | protein\_coding | 16:10411948-10424428 (+) |  | -0.1800 | 1.72e-04 | 9.52e-04 |
| ENSMUSG00000028266 | Lmo4 | protein\_coding | 3:144188530-144205220 (-) |  | 0.2490 | 1.72e-04 | 9.52e-04 |
| ENSMUSG00000036305 | Rpl39-ps | processed\_pseudogene | 15:102635038-102635188 (+) |  | 0.4030 | 1.73e-04 | 9.58e-04 |
| ENSMUSG00000009292 | Trpm2 | protein\_coding | 10:77907722-77970563 (-) |  | 0.4290 | 1.73e-04 | 9.59e-04 |
| ENSMUSG00000049811 | Fam161a | protein\_coding | 11:23007531-23030788 (+) |  | 0.6700 | 1.74e-04 | 9.65e-04 |
| ENSMUSG00000036833 | Pnpla7 | protein\_coding | 2:24976033-25054057 (+) |  | 0.2880 | 1.74e-04 | 9.65e-04 |
| ENSMUSG00000030126 | Tmcc1 | protein\_coding | 6:116018611-116193486 (-) |  | 0.2640 | 1.75e-04 | 9.66e-04 |
| ENSMUSG00000008845 | Cd163 | protein\_coding | 6:124304656-124330527 (+) |  | 0.4890 | 1.75e-04 | 9.67e-04 |
| ENSMUSG00000079593 | Gm14597 | protein\_coding | X:53577087-53608979 (-) |  | 2.6800 | 1.75e-04 | 9.68e-04 |
| ENSMUSG00000041638 | Gcn1 | protein\_coding | 5:115565254-115622654 (+) |  | -0.2250 | 1.76e-04 | 9.73e-04 |
| ENSMUSG00000025499 | Hras | protein\_coding | 7:141189105-141194005 (-) |  | -0.2710 | 1.77e-04 | 9.77e-04 |
| ENSMUSG00000024902 | Mrpl11 | protein\_coding | 19:4962147-4966999 (+) |  | -0.2690 | 1.79e-04 | 9.86e-04 |
| ENSMUSG00000028634 | Hivep3 | protein\_coding | 4:119733784-120138045 (+) |  | 0.4730 | 1.79e-04 | 9.88e-04 |
| ENSMUSG00000024054 | Smchd1 | protein\_coding | 17:71344489-71475343 (-) |  | -0.2110 | 1.79e-04 | 9.88e-04 |
| ENSMUSG00000031400 | G6pdx | protein\_coding | X:74409483-74429194 (-) |  | -0.1600 | 1.80e-04 | 9.91e-04 |
| ENSMUSG00000032582 | Rbm6 | protein\_coding | 9:107773559-107873237 (-) |  | -0.1580 | 1.81e-04 | 9.98e-04 |
| ENSMUSG00000061136 | Prpf40a | protein\_coding | 2:53134704-53191702 (-) |  | -0.1670 | 1.83e-04 | 1.01e-03 |
| ENSMUSG00000116757 | Gm4786 | processed\_pseudogene | 17:16557246-16558043 (+) |  | -0.3100 | 1.83e-04 | 1.01e-03 |
| ENSMUSG00000028247 | Coq3 | protein\_coding | 4:21879673-21912162 (+) |  | -0.3470 | 1.83e-04 | 1.01e-03 |
| ENSMUSG00000047126 | Cltc | protein\_coding | 11:86694351-86757565 (-) |  | -0.1640 | 1.83e-04 | 1.01e-03 |
| ENSMUSG00000026600 | Soat1 | protein\_coding | 1:156424525-156474331 (-) |  | -0.1800 | 1.84e-04 | 1.01e-03 |
| ENSMUSG00000020572 | Nampt | protein\_coding | 12:32819545-32853349 (+) |  | -0.2430 | 1.84e-04 | 1.01e-03 |
| ENSMUSG00000062825 | Actg1 | protein\_coding | 11:120345690-120348542 (-) |  | -0.1500 | 1.85e-04 | 1.02e-03 |
| ENSMUSG00000059288 | Cdyl | protein\_coding | 13:35659833-35874063 (+) |  | 0.2460 | 1.85e-04 | 1.02e-03 |
| ENSMUSG00000026796 | Fam129b | protein\_coding | 2:32876114-32925254 (+) |  | -0.2320 | 1.85e-04 | 1.02e-03 |
| ENSMUSG00000031731 | Ap1g1 | protein\_coding | 8:109778554-109864204 (+) |  | -0.1990 | 1.86e-04 | 1.02e-03 |
| ENSMUSG00000028041 | Adam15 | protein\_coding | 3:89338542-89349996 (-) |  | -0.1870 | 1.86e-04 | 1.02e-03 |
| ENSMUSG00000028207 | Asph | protein\_coding | 4:9448069-9669344 (-) |  | -0.3050 | 1.86e-04 | 1.02e-03 |
| ENSMUSG00000028434 | Epb41l4b | protein\_coding | 4:56991972-57143437 (-) |  | 0.7650 | 1.87e-04 | 1.02e-03 |
| ENSMUSG00000100755 | Rps23-ps1 | processed\_pseudogene | 3:37714740-37715164 (+) |  | 0.7630 | 1.87e-04 | 1.02e-03 |
| ENSMUSG00000051007 | Gatd1 | protein\_coding | 7:141407794-141414136 (-) |  | 0.2400 | 1.87e-04 | 1.03e-03 |
| ENSMUSG00000035941 | Ibtk | protein\_coding | 9:85687360-85749334 (-) |  | -0.2770 | 1.88e-04 | 1.03e-03 |
| ENSMUSG00000027195 | Hsd17b12 | protein\_coding | 2:94032689-94157964 (-) |  | -0.2330 | 1.88e-04 | 1.03e-03 |
| ENSMUSG00000001403 | Ube2c | protein\_coding | 2:164769898-164778822 (+) |  | -0.2060 | 1.89e-04 | 1.03e-03 |
| ENSMUSG00000031226 | Pbdc1 | protein\_coding | X:105079756-105117090 (+) |  | -0.2890 | 1.89e-04 | 1.03e-03 |
| ENSMUSG00000056204 | Pgpep1 | protein\_coding | 8:70646435-70660388 (-) |  | 0.3870 | 1.89e-04 | 1.04e-03 |
| ENSMUSG00000019088 | Dnase1l1 | protein\_coding | X:74273217-74282337 (-) |  | 0.3000 | 1.90e-04 | 1.04e-03 |
| ENSMUSG00000056749 | Nfil3 | protein\_coding | 13:52967209-52981073 (-) |  | -0.2850 | 1.93e-04 | 1.05e-03 |
| ENSMUSG00000032329 | Hmg20a | protein\_coding | 9:56418609-56496936 (+) |  | 0.2140 | 1.93e-04 | 1.05e-03 |
| ENSMUSG00000021149 | Gtpbp4 | protein\_coding | 13:8966331-8996083 (-) |  | -0.2280 | 1.94e-04 | 1.06e-03 |
| ENSMUSG00000024091 | Vapa | protein\_coding | 17:65578327-65613555 (-) |  | -0.1770 | 1.95e-04 | 1.06e-03 |
| ENSMUSG00000018008 | Cyth4 | protein\_coding | 15:78597047-78622019 (+) |  | 0.1880 | 1.96e-04 | 1.07e-03 |
| ENSMUSG00000033902 | Mapkbp1 | protein\_coding | 2:119972699-120027408 (+) |  | 0.3330 | 1.96e-04 | 1.07e-03 |
| ENSMUSG00000024610 | Cd74 | protein\_coding | 18:60803848-60812652 (+) |  | 0.7350 | 1.96e-04 | 1.07e-03 |
| ENSMUSG00000064061 | Dzip3 | protein\_coding | 16:48924232-48994165 (-) |  | -0.3610 | 1.96e-04 | 1.07e-03 |
| ENSMUSG00000020697 | Lig3 | protein\_coding | 11:82781108-82804274 (+) |  | -0.2260 | 1.97e-04 | 1.07e-03 |
| ENSMUSG00000031214 | Ophn1 | protein\_coding | X:98554277-98891025 (-) |  | 0.5670 | 1.97e-04 | 1.07e-03 |
| ENSMUSG00000038150 | Ormdl3 | protein\_coding | 11:98581256-98587368 (-) |  | 0.3410 | 1.98e-04 | 1.08e-03 |
| ENSMUSG00000111792 | Gm33858 | lncRNA | 9:120068996-120070622 (+) |  | 0.9070 | 1.98e-04 | 1.08e-03 |
| ENSMUSG00000030792 | Dkkl1 | protein\_coding | 7:45207519-45212147 (-) |  | -1.3500 | 1.98e-04 | 1.08e-03 |
| ENSMUSG00000025785 | Exosc7 | protein\_coding | 9:123113215-123136129 (+) |  | -0.2140 | 1.98e-04 | 1.08e-03 |
| ENSMUSG00000086688 | Gm11560 | processed\_pseudogene | 11:99932162-99933109 (+) |  | -0.2860 | 1.99e-04 | 1.08e-03 |
| ENSMUSG00000038811 | Gngt2 | protein\_coding | 11:95837216-95845734 (+) |  | -0.7700 | 1.99e-04 | 1.08e-03 |
| ENSMUSG00000004071 | Cdip1 | protein\_coding | 16:4750348-4790292 (-) |  | 0.2120 | 2.00e-04 | 1.08e-03 |
| ENSMUSG00000030674 | Qprt | protein\_coding | 7:127107114-127122226 (-) |  | 0.7980 | 2.00e-04 | 1.08e-03 |
| ENSMUSG00000114003 | Gm9616 | processed\_pseudogene | 13:3298887-3299667 (+) |  | 0.2810 | 2.00e-04 | 1.08e-03 |
| ENSMUSG00000002205 | Vrk3 | protein\_coding | 7:44748413-44777515 (+) |  | -0.2140 | 2.01e-04 | 1.09e-03 |
| ENSMUSG00000026516 | Nvl | protein\_coding | 1:181087138-181144204 (-) |  | -0.1800 | 2.01e-04 | 1.09e-03 |
| ENSMUSG00000031627 | Irf2 | protein\_coding | 8:46739732-46847458 (+) |  | 0.1800 | 2.01e-04 | 1.09e-03 |
| ENSMUSG00000071291 | Zfp58 | protein\_coding | 13:67490167-67500555 (-) |  | 0.5090 | 2.02e-04 | 1.10e-03 |
| ENSMUSG00000032171 | Pin1 | protein\_coding | 9:20652095-20666584 (+) |  | -0.2880 | 2.02e-04 | 1.10e-03 |
| ENSMUSG00000053040 | Aph1c | protein\_coding | 9:66814994-66834726 (-) |  | 0.3200 | 2.02e-04 | 1.10e-03 |
| ENSMUSG00000030652 | Coq7 | protein\_coding | 7:118509659-118533356 (-) |  | -0.3850 | 2.04e-04 | 1.11e-03 |
| ENSMUSG00000037366 | Pafah2 | protein\_coding | 4:134396320-134427413 (+) |  | -0.4350 | 2.05e-04 | 1.11e-03 |
| ENSMUSG00000053897 | Slc39a8 | protein\_coding | 3:135825279-135888572 (+) |  | -0.4480 | 2.05e-04 | 1.11e-03 |
| ENSMUSG00000024231 | Cul2 | protein\_coding | 18:3382988-3436377 (+) |  | -0.2490 | 2.05e-04 | 1.11e-03 |
| ENSMUSG00000039783 | Kmo | protein\_coding | 1:175620381-175662116 (+) |  | -0.3430 | 2.06e-04 | 1.12e-03 |
| ENSMUSG00000028433 | Ubap2 | protein\_coding | 4:41194313-41275144 (-) |  | -0.2760 | 2.08e-04 | 1.12e-03 |
| ENSMUSG00000028292 | Rars2 | protein\_coding | 4:34614957-34660167 (+) |  | -0.2910 | 2.08e-04 | 1.13e-03 |
| ENSMUSG00000085175 | Gm11423 | lncRNA | 11:82779601-82781042 (-) |  | 0.7420 | 2.09e-04 | 1.13e-03 |
| ENSMUSG00000032869 | Psmf1 | protein\_coding | 2:151715812-151744186 (-) |  | -0.2180 | 2.09e-04 | 1.13e-03 |
| ENSMUSG00000021792 | Prxl2a | protein\_coding | 14:40993740-41013788 (-) |  | 0.8950 | 2.10e-04 | 1.13e-03 |
| ENSMUSG00000041560 | Nop53 | protein\_coding | 7:15936183-15946074 (-) |  | 0.2570 | 2.10e-04 | 1.13e-03 |
| ENSMUSG00000021895 | Arhgef3 | protein\_coding | 14:27114899-27403911 (+) |  | 0.4000 | 2.10e-04 | 1.13e-03 |
| ENSMUSG00000036661 | Dennd3 | protein\_coding | 15:73512560-73572242 (+) |  | 0.3930 | 2.11e-04 | 1.14e-03 |
| ENSMUSG00000029407 | Uso1 | protein\_coding | 5:92137938-92202798 (+) |  | -0.1710 | 2.11e-04 | 1.14e-03 |
| ENSMUSG00000021690 | Jmy | protein\_coding | 13:93430101-93499808 (-) |  | 0.3610 | 2.11e-04 | 1.14e-03 |
| ENSMUSG00000029580 | Actb | protein\_coding | 5:142903115-142906754 (-) |  | -0.1930 | 2.12e-04 | 1.14e-03 |
| ENSMUSG00000006310 | Zbtb32 | protein\_coding | 7:30589681-30598909 (-) |  | -0.8130 | 2.12e-04 | 1.14e-03 |
| ENSMUSG00000021392 | Nol8 | protein\_coding | 13:49653078-49679016 (+) |  | -0.1900 | 2.13e-04 | 1.14e-03 |
| ENSMUSG00000029233 | Srd5a3 | protein\_coding | 5:76140271-76155504 (+) |  | 0.1710 | 2.13e-04 | 1.14e-03 |
| ENSMUSG00000063550 | Nup98 | protein\_coding | 7:102119398-102210176 (-) |  | -0.2350 | 2.14e-04 | 1.15e-03 |
| ENSMUSG00000026999 | Nup35 | protein\_coding | 2:80617236-80658906 (+) |  | -0.3470 | 2.14e-04 | 1.15e-03 |
| ENSMUSG00000041632 | Mrps27 | protein\_coding | 13:99344786-99415562 (+) |  | -0.3050 | 2.14e-04 | 1.15e-03 |
| ENSMUSG00000032002 | Dcun1d5 | protein\_coding | 9:7184520-7208205 (+) |  | -0.3210 | 2.15e-04 | 1.15e-03 |
| ENSMUSG00000040554 | Aipl1 | protein\_coding | 11:72027963-72037509 (-) |  | 0.7840 | 2.15e-04 | 1.15e-03 |
| ENSMUSG00000042292 | Mrtfa | protein\_coding | 15:81012281-81190757 (-) |  | 0.2710 | 2.15e-04 | 1.15e-03 |
| ENSMUSG00000042535 | Gtpbp1 | protein\_coding | 15:79690845-79721479 (+) |  | 0.1820 | 2.16e-04 | 1.16e-03 |
| ENSMUSG00000073633 | Fbxo36 | protein\_coding | 1:84839841-84900487 (+) |  | -1.0400 | 2.16e-04 | 1.16e-03 |
| ENSMUSG00000021806 | Nid2 | protein\_coding | 14:19751265-19811787 (+) |  | -0.8140 | 2.17e-04 | 1.16e-03 |
| ENSMUSG00000022945 | Chaf1b | protein\_coding | 16:93883901-93906115 (+) |  | -0.2990 | 2.17e-04 | 1.16e-03 |
| ENSMUSG00000020806 | Rhbdf2 | protein\_coding | 11:116598165-116627019 (-) |  | -0.2780 | 2.19e-04 | 1.18e-03 |
| ENSMUSG00000038214 | Bend3 | protein\_coding | 10:43478831-43515396 (+) |  | -0.4430 | 2.20e-04 | 1.18e-03 |
| ENSMUSG00000028538 | St3gal3 | protein\_coding | 4:117932154-118134914 (-) |  | 0.3100 | 2.20e-04 | 1.18e-03 |
| ENSMUSG00000059540 | Tcea2 | protein\_coding | 2:181680310-181688071 (+) |  | 0.4050 | 2.20e-04 | 1.18e-03 |
| ENSMUSG00000040857 | Erf | protein\_coding | 7:25242561-25250761 (-) |  | -0.2520 | 2.21e-04 | 1.18e-03 |
| ENSMUSG00000019564 | Arid3a | protein\_coding | 10:79927043-79955018 (+) |  | 0.1890 | 2.21e-04 | 1.18e-03 |
| ENSMUSG00000029270 | Dipk1a | protein\_coding | 5:107908053-107987085 (-) |  | 0.2340 | 2.21e-04 | 1.18e-03 |
| ENSMUSG00000022553 | Maf1 | protein\_coding | 15:76351294-76354380 (+) |  | 0.2200 | 2.22e-04 | 1.18e-03 |
| ENSMUSG00000022193 | Psmb5 | protein\_coding | 14:54614119-54618022 (-) |  | -0.2480 | 2.22e-04 | 1.19e-03 |
| ENSMUSG00000021322 | Aoah | protein\_coding | 13:20794113-21036617 (+) |  | 0.2190 | 2.23e-04 | 1.19e-03 |
| ENSMUSG00000026357 | Rgs18 | protein\_coding | 1:144752683-144775435 (-) |  | 0.3830 | 2.25e-04 | 1.20e-03 |
| ENSMUSG00000015149 | Sirt2 | protein\_coding | 7:28766735-28788661 (+) |  | 0.1820 | 2.25e-04 | 1.20e-03 |
| ENSMUSG00000044857 | Lemd2 | protein\_coding | 17:27189600-27204469 (-) |  | 0.1890 | 2.25e-04 | 1.20e-03 |
| ENSMUSG00000056130 | Ticam2 | protein\_coding | 18:46557291-46574533 (-) |  | 0.1810 | 2.25e-04 | 1.20e-03 |
| ENSMUSG00000051397 | Tacstd2 | protein\_coding | 6:67534062-67535796 (-) |  | -1.4100 | 2.26e-04 | 1.20e-03 |
| ENSMUSG00000053333 | Dis3l2 | protein\_coding | 1:86703808-87050095 (+) |  | 0.2760 | 2.26e-04 | 1.20e-03 |
| ENSMUSG00000027519 | Rab22a | protein\_coding | 2:173659760-173707343 (+) |  | 0.1610 | 2.26e-04 | 1.21e-03 |
| ENSMUSG00000037032 | Apbb1 | protein\_coding | 7:105558483-105581653 (-) |  | 0.8430 | 2.27e-04 | 1.21e-03 |
| ENSMUSG00000087687 | Pet100 | protein\_coding | 8:3621548-3625848 (+) |  | 0.3650 | 2.27e-04 | 1.21e-03 |
| ENSMUSG00000098176 | Ccdc166 | protein\_coding | 15:75979872-75982455 (-) |  | 0.4620 | 2.27e-04 | 1.21e-03 |
| ENSMUSG00000022070 | Bora | protein\_coding | 14:99046222-99074540 (+) |  | -0.2470 | 2.29e-04 | 1.21e-03 |
| ENSMUSG00000063659 | Zbtb18 | protein\_coding | 1:177442351-177450764 (+) |  | 0.1660 | 2.29e-04 | 1.21e-03 |
| ENSMUSG00000000751 | Rpa1 | protein\_coding | 11:75298166-75348324 (-) |  | -0.2430 | 2.29e-04 | 1.22e-03 |
| ENSMUSG00000057367 | Birc2 | protein\_coding | 9:7818227-7837064 (-) |  | 0.2000 | 2.29e-04 | 1.22e-03 |
| ENSMUSG00000062421 | Arf2 | protein\_coding | 11:103966739-103985337 (+) |  | 0.2050 | 2.30e-04 | 1.22e-03 |
| ENSMUSG00000023963 | Cyp39a1 | protein\_coding | 17:43667425-43751431 (+) |  | -0.3760 | 2.31e-04 | 1.22e-03 |
| ENSMUSG00000033991 | Ttc37 | protein\_coding | 13:76098734-76190316 (+) |  | -0.2160 | 2.31e-04 | 1.22e-03 |
| ENSMUSG00000021607 | Mrpl36 | protein\_coding | 13:73328513-73332178 (+) |  | -0.2910 | 2.31e-04 | 1.22e-03 |
| ENSMUSG00000030967 | Zranb1 | protein\_coding | 7:132931142-132986391 (+) |  | 0.1660 | 2.32e-04 | 1.23e-03 |
| ENSMUSG00000102608 | Gm37267 | lncRNA | 1:180508778-180519727 (-) |  | 3.0500 | 2.32e-04 | 1.23e-03 |
| ENSMUSG00000053841 | Txlna | protein\_coding | 4:129626078-129641065 (-) |  | -0.1770 | 2.36e-04 | 1.25e-03 |
| ENSMUSG00000048120 | Entpd1 | protein\_coding | 19:40612366-40741602 (+) |  | 0.4350 | 2.37e-04 | 1.25e-03 |
| ENSMUSG00000048922 | Cdca2 | protein\_coding | 14:67676331-67715841 (-) |  | -0.2510 | 2.37e-04 | 1.25e-03 |
| ENSMUSG00000027746 | Ufm1 | protein\_coding | 3:53853376-53863830 (-) |  | -0.2610 | 2.38e-04 | 1.26e-03 |
| ENSMUSG00000026153 | Fam135a | protein\_coding | 1:24011093-24100341 (-) |  | 0.5870 | 2.39e-04 | 1.26e-03 |
| ENSMUSG00000048039 | Isg20l2 | protein\_coding | 3:87930314-87940686 (+) |  | -0.1580 | 2.39e-04 | 1.26e-03 |
| ENSMUSG00000002660 | Clpp | protein\_coding | 17:56990305-56996188 (+) |  | -0.2570 | 2.39e-04 | 1.26e-03 |
| ENSMUSG00000028018 | Gstcd | protein\_coding | 3:132981752-133092033 (-) |  | -0.3520 | 2.39e-04 | 1.26e-03 |
| ENSMUSG00000058922 | Gm10052 | processed\_pseudogene | 9:123689233-123690192 (-) |  | -0.3040 | 2.40e-04 | 1.27e-03 |
| ENSMUSG00000025612 | Bach1 | protein\_coding | 16:87698945-87733346 (+) |  | 0.2230 | 2.40e-04 | 1.27e-03 |
| ENSMUSG00000026113 | Inpp4a | protein\_coding | 1:37299865-37410736 (+) |  | 0.2410 | 2.40e-04 | 1.27e-03 |
| ENSMUSG00000002748 | Baz1b | protein\_coding | 5:135187264-135246129 (+) |  | -0.1720 | 2.41e-04 | 1.27e-03 |
| ENSMUSG00000029071 | Dvl1 | protein\_coding | 4:155847402-155859303 (+) |  | 0.2240 | 2.41e-04 | 1.27e-03 |
| ENSMUSG00000028015 | Ctso | protein\_coding | 3:81932601-81956725 (+) |  | 0.3230 | 2.41e-04 | 1.27e-03 |
| ENSMUSG00000059713 | Rcan3 | protein\_coding | 4:135412308-135433853 (-) |  | 0.2800 | 2.41e-04 | 1.27e-03 |
| ENSMUSG00000030142 | Clec4e | protein\_coding | 6:123281789-123289870 (-) |  | -0.8280 | 2.43e-04 | 1.28e-03 |
| ENSMUSG00000042106 | Inka1 | protein\_coding | 9:107984223-107985879 (-) |  | 0.6280 | 2.43e-04 | 1.28e-03 |
| ENSMUSG00000001473 | Tubb6 | protein\_coding | 18:67390717-67402749 (+) |  | -0.3290 | 2.44e-04 | 1.28e-03 |
| ENSMUSG00000037405 | Icam1 | protein\_coding | 9:21015985-21028817 (+) |  | -0.2450 | 2.45e-04 | 1.29e-03 |
| ENSMUSG00000042423 | Fbrs | protein\_coding | 7:127479199-127491711 (+) |  | 0.2350 | 2.45e-04 | 1.29e-03 |
| ENSMUSG00000037204 | Atg101 | protein\_coding | 15:101284272-101290945 (+) |  | -0.2270 | 2.46e-04 | 1.29e-03 |
| ENSMUSG00000018932 | Map2k3 | protein\_coding | 11:60932033-60952811 (+) |  | -0.1770 | 2.46e-04 | 1.29e-03 |
| ENSMUSG00000037993 | Dhx38 | protein\_coding | 8:109548011-109565861 (-) |  | -0.1900 | 2.48e-04 | 1.30e-03 |
| ENSMUSG00000024236 | Svil | protein\_coding | 18:4920540-5119299 (+) |  | -0.2530 | 2.48e-04 | 1.30e-03 |
| ENSMUSG00000093798 | Gm8355 | processed\_pseudogene | 10:14745362-14747302 (-) |  | -0.3760 | 2.49e-04 | 1.31e-03 |
| ENSMUSG00000003099 | Ppp5c | protein\_coding | 7:17004640-17027924 (-) |  | -0.1860 | 2.50e-04 | 1.31e-03 |
| ENSMUSG00000021384 | Susd3 | protein\_coding | 13:49230690-49248706 (-) |  | 0.3450 | 2.50e-04 | 1.31e-03 |
| ENSMUSG00000022247 | Brix1 | protein\_coding | 15:10474779-10485947 (-) |  | -0.2850 | 2.51e-04 | 1.31e-03 |
| ENSMUSG00000051517 | Arhgef39 | protein\_coding | 4:43496142-43499695 (-) |  | 0.2240 | 2.51e-04 | 1.31e-03 |
| ENSMUSG00000060703 | Cd302 | protein\_coding | 2:60251993-60284488 (-) |  | -0.4300 | 2.51e-04 | 1.31e-03 |
| ENSMUSG00000070167 | Snora57 | snoRNA | 19:8888538-8888685 (-) |  | 0.6490 | 2.51e-04 | 1.31e-03 |
| ENSMUSG00000029723 | Tsc22d4 | protein\_coding | 5:137745730-137768450 (+) |  | 0.1720 | 2.51e-04 | 1.31e-03 |
| ENSMUSG00000020152 | Actr2 | protein\_coding | 11:20062304-20112913 (-) |  | -0.1780 | 2.51e-04 | 1.31e-03 |
| ENSMUSG00000029771 | Irf5 | protein\_coding | 6:29526625-29541871 (+) |  | 0.2670 | 2.52e-04 | 1.32e-03 |
| ENSMUSG00000023923 | Tbc1d5 | protein\_coding | 17:50733124-51179352 (-) |  | 0.2160 | 2.53e-04 | 1.32e-03 |
| ENSMUSG00000117458 | Gm6552 | processed\_pseudogene | 17:79933512-79934572 (-) |  | 0.4030 | 2.55e-04 | 1.33e-03 |
| ENSMUSG00000026260 | Ndufa10 | protein\_coding | 1:92439010-92473860 (-) |  | -0.2610 | 2.55e-04 | 1.33e-03 |
| ENSMUSG00000043885 | Slc36a4 | protein\_coding | 9:15709738-15743361 (+) |  | 0.2020 | 2.57e-04 | 1.34e-03 |
| ENSMUSG00000055200 | Sertad3 | protein\_coding | 7:27473768-27477364 (+) |  | 0.3900 | 2.57e-04 | 1.35e-03 |
| ENSMUSG00000038046 | Mrm3 | protein\_coding | 11:76243715-76250619 (+) |  | -0.3570 | 2.58e-04 | 1.35e-03 |
| ENSMUSG00000052428 | Tmco1 | protein\_coding | 1:167308378-167333978 (+) |  | -0.1950 | 2.58e-04 | 1.35e-03 |
| ENSMUSG00000043243 | Fam129c | protein\_coding | 8:71597648-71607936 (+) |  | 0.7070 | 2.58e-04 | 1.35e-03 |
| ENSMUSG00000021281 | Tnfaip2 | protein\_coding | 12:111442469-111455018 (+) |  | 0.2310 | 2.60e-04 | 1.36e-03 |
| ENSMUSG00000050490 | Gm8394 | processed\_pseudogene | 10:85313488-85314439 (+) |  | -0.3520 | 2.61e-04 | 1.36e-03 |
| ENSMUSG00000059791 | Nrm | protein\_coding | 17:35861318-35865402 (+) |  | 0.2870 | 2.62e-04 | 1.37e-03 |
| ENSMUSG00000021500 | Ddx46 | protein\_coding | 13:55635027-55681256 (+) |  | -0.1920 | 2.64e-04 | 1.38e-03 |
| ENSMUSG00000040883 | Tmem205 | protein\_coding | 9:21921008-21927556 (-) |  | 0.3230 | 2.66e-04 | 1.39e-03 |
| ENSMUSG00000019826 | Zbtb24 | protein\_coding | 10:41450383-41465574 (+) |  | 0.2370 | 2.68e-04 | 1.39e-03 |
| ENSMUSG00000001441 | Npepps | protein\_coding | 11:97205842-97280638 (-) |  | -0.2030 | 2.68e-04 | 1.40e-03 |
| ENSMUSG00000022940 | Pigp | protein\_coding | 16:94358763-94371842 (-) |  | 0.5490 | 2.69e-04 | 1.40e-03 |
| ENSMUSG00000000346 | Dazap2 | protein\_coding | 15:100615349-100620761 (+) |  | 0.1220 | 2.69e-04 | 1.40e-03 |
| ENSMUSG00000028873 | Cdca8 | protein\_coding | 4:124918465-124939311 (-) |  | -0.1970 | 2.71e-04 | 1.41e-03 |
| ENSMUSG00000001774 | Chordc1 | protein\_coding | 9:18292125-18317442 (+) |  | -0.2800 | 2.72e-04 | 1.41e-03 |
| ENSMUSG00000020020 | Usp44 | protein\_coding | 10:93831555-93858088 (+) |  | -0.9800 | 2.72e-04 | 1.41e-03 |
| ENSMUSG00000062031 | Pgghg | protein\_coding | 7:140941391-140947664 (+) |  | -0.2310 | 2.73e-04 | 1.42e-03 |
| ENSMUSG00000021238 | Aldh6a1 | protein\_coding | 12:84430717-84451004 (-) |  | 0.4350 | 2.74e-04 | 1.42e-03 |
| ENSMUSG00000075415 | Fnbp1 | protein\_coding | 2:31026206-31142008 (-) |  | 0.1740 | 2.75e-04 | 1.42e-03 |
| ENSMUSG00000036764 | Dnajc12 | protein\_coding | 10:63382443-63410576 (+) |  | -0.6060 | 2.76e-04 | 1.43e-03 |
| ENSMUSG00000041912 | Tdrkh | protein\_coding | 3:94413273-94434668 (+) |  | 0.4960 | 2.77e-04 | 1.44e-03 |
| ENSMUSG00000025887 | Casp12 | protein\_coding | 9:5345430-5373032 (+) |  | -1.3400 | 2.77e-04 | 1.44e-03 |
| ENSMUSG00000038290 | Smg6 | protein\_coding | 11:74925823-75164448 (+) |  | 0.2140 | 2.78e-04 | 1.44e-03 |
| ENSMUSG00000057193 | Slc44a2 | protein\_coding | 9:21320698-21355028 (+) |  | -0.1910 | 2.78e-04 | 1.44e-03 |
| ENSMUSG00000032897 | Nfyc | protein\_coding | 4:120757438-120831572 (-) |  | -0.2450 | 2.80e-04 | 1.45e-03 |
| ENSMUSG00000026887 | Mrrf | protein\_coding | 2:36136389-36190647 (+) |  | -0.3040 | 2.80e-04 | 1.45e-03 |
| ENSMUSG00000062761 | Zfp512 | protein\_coding | 5:31452431-31481754 (+) |  | 0.2620 | 2.80e-04 | 1.45e-03 |
| ENSMUSG00000046805 | Mpeg1 | protein\_coding | 19:12460779-12465283 (+) |  | 0.2430 | 2.80e-04 | 1.45e-03 |
| ENSMUSG00000020882 | Cacnb1 | protein\_coding | 11:98001508-98023034 (-) |  | 0.8050 | 2.81e-04 | 1.45e-03 |
| ENSMUSG00000020925 | Ccdc43 | protein\_coding | 11:102684686-102697782 (-) |  | -0.2130 | 2.81e-04 | 1.45e-03 |
| ENSMUSG00000006024 | Napa | protein\_coding | 7:16098458-16117975 (+) |  | -0.1590 | 2.82e-04 | 1.46e-03 |
| ENSMUSG00000032112 | Trappc4 | protein\_coding | 9:44403698-44407600 (-) |  | -0.2010 | 2.82e-04 | 1.46e-03 |
| ENSMUSG00000057406 | Nsd2 | protein\_coding | 5:33820725-33897975 (+) |  | -0.2240 | 2.84e-04 | 1.46e-03 |
| ENSMUSG00000031166 | Wdr13 | protein\_coding | X:8123301-8132892 (-) |  | 0.2380 | 2.85e-04 | 1.47e-03 |
| ENSMUSG00000029674 | Limk1 | protein\_coding | 5:134656039-134688598 (-) |  | 0.3290 | 2.86e-04 | 1.47e-03 |
| ENSMUSG00000109324 | Prmt1 | protein\_coding | 7:44975989-44986568 (-) |  | -0.2290 | 2.86e-04 | 1.48e-03 |
| ENSMUSG00000096472 | Cdkn2d | protein\_coding | 9:21288410-21291407 (-) |  | -0.3010 | 2.87e-04 | 1.48e-03 |
| ENSMUSG00000028702 | Rad54l | protein\_coding | 4:116094264-116123690 (-) |  | -0.3480 | 2.87e-04 | 1.48e-03 |
| ENSMUSG00000063884 | Ptcd3 | protein\_coding | 6:71880638-71908750 (-) |  | -0.2740 | 2.89e-04 | 1.49e-03 |
| ENSMUSG00000047832 | Cdca4 | protein\_coding | 12:112820229-112829423 (-) |  | -0.1770 | 2.91e-04 | 1.50e-03 |
| ENSMUSG00000090952 | Gm17251 | lncRNA | 17:33759992-33761870 (+) |  | 0.7650 | 2.94e-04 | 1.51e-03 |
| ENSMUSG00000025104 | Hdgfl3 | protein\_coding | 7:81881251-81934473 (-) |  | 0.4620 | 2.96e-04 | 1.52e-03 |
| ENSMUSG00000028248 | Pnisr | protein\_coding | 4:21847583-21876475 (+) |  | 0.3140 | 2.96e-04 | 1.52e-03 |
| ENSMUSG00000003355 | Fkbp11 | protein\_coding | 15:98724366-98728198 (-) |  | -0.8910 | 2.96e-04 | 1.52e-03 |
| ENSMUSG00000034274 | Thoc5 | protein\_coding | 11:4895320-4928867 (+) |  | -0.2190 | 2.96e-04 | 1.52e-03 |
| ENSMUSG00000047757 | Fancb | protein\_coding | X:164980592-164997272 (+) |  | -0.3200 | 2.97e-04 | 1.53e-03 |
| ENSMUSG00000053950 | Adnp2 | protein\_coding | 18:80126311-80151482 (-) |  | -0.2130 | 2.97e-04 | 1.53e-03 |
| ENSMUSG00000006281 | Tep1 | protein\_coding | 14:50824059-50870560 (-) |  | 0.2220 | 2.97e-04 | 1.53e-03 |
| ENSMUSG00000034522 | Zfp395 | protein\_coding | 14:65358389-65398930 (+) |  | 0.4420 | 2.98e-04 | 1.53e-03 |
| ENSMUSG00000004933 | Matk | protein\_coding | 10:81252935-81263365 (+) |  | 0.2550 | 2.98e-04 | 1.53e-03 |
| ENSMUSG00000106825 | 2510016D11Rik | TEC | 5:120552055-120553039 (-) |  | -0.3690 | 2.99e-04 | 1.54e-03 |
| ENSMUSG00000028407 | Smim27 | protein\_coding | 4:40269579-40270940 (+) |  | 0.5730 | 2.99e-04 | 1.54e-03 |
| ENSMUSG00000108732 | 2310043P16Rik | TEC | 7:34389732-34393612 (+) |  | 0.7460 | 3.00e-04 | 1.54e-03 |
| ENSMUSG00000114295 | Gm2534 | processed\_pseudogene | 13:101348752-101349181 (+) |  | 3.3100 | 3.00e-04 | 1.54e-03 |
| ENSMUSG00000016477 | E2f3 | protein\_coding | 13:29906575-29986063 (-) |  | -0.2490 | 3.00e-04 | 1.54e-03 |
| ENSMUSG00000029815 | Malsu1 | protein\_coding | 6:49073795-49086751 (+) |  | -0.2750 | 3.03e-04 | 1.55e-03 |
| ENSMUSG00000002032 | Tmem25 | protein\_coding | 9:44793769-44799307 (-) |  | 1.1000 | 3.03e-04 | 1.55e-03 |
| ENSMUSG00000038312 | Edem2 | protein\_coding | 2:155701677-155729475 (-) |  | -0.2240 | 3.04e-04 | 1.56e-03 |
| ENSMUSG00000000902 | Smarcb1 | protein\_coding | 10:75896769-75921617 (-) |  | -0.2620 | 3.05e-04 | 1.56e-03 |
| ENSMUSG00000031826 | Usp10 | protein\_coding | 8:119910360-119957560 (+) |  | -0.2310 | 3.06e-04 | 1.56e-03 |
| ENSMUSG00000035232 | Pdk3 | protein\_coding | X:93764607-93832201 (-) |  | -0.2370 | 3.07e-04 | 1.57e-03 |
| ENSMUSG00000018500 | Adora2b | protein\_coding | 11:62248984-62266453 (+) |  | -0.2960 | 3.07e-04 | 1.57e-03 |
| ENSMUSG00000020017 | Hal | protein\_coding | 10:93488768-93519304 (+) |  | 0.6440 | 3.08e-04 | 1.58e-03 |
| ENSMUSG00000036867 | Smad6 | protein\_coding | 9:63953076-64022059 (-) |  | 0.5160 | 3.11e-04 | 1.59e-03 |
| ENSMUSG00000004455 | Ppp1cc | protein\_coding | 5:122158278-122175273 (+) |  | -0.2540 | 3.11e-04 | 1.59e-03 |
| ENSMUSG00000029627 | Zkscan14 | protein\_coding | 5:145194946-145201868 (-) |  | 0.2890 | 3.11e-04 | 1.59e-03 |
| ENSMUSG00000070284 | Gmppb | protein\_coding | 9:108049242-108052801 (+) |  | -0.2430 | 3.12e-04 | 1.59e-03 |
| ENSMUSG00000032308 | Ulk3 | protein\_coding | 9:57589452-57596233 (+) |  | 0.3960 | 3.13e-04 | 1.60e-03 |
| ENSMUSG00000050240 | Hic2 | protein\_coding | 16:17233572-17263430 (+) |  | 0.4260 | 3.13e-04 | 1.60e-03 |
| ENSMUSG00000030619 | Eed | protein\_coding | 7:89954654-89980983 (-) |  | -0.2720 | 3.13e-04 | 1.60e-03 |
| ENSMUSG00000053749 | Gm9920 | lncRNA | 15:55099917-55113677 (-) |  | 0.5840 | 3.13e-04 | 1.60e-03 |
| ENSMUSG00000049588 | Ccdc69 | protein\_coding | 11:55049731-55078131 (-) |  | 0.2940 | 3.14e-04 | 1.60e-03 |
| ENSMUSG00000058446 | Znrf2 | protein\_coding | 6:54816916-54893500 (+) |  | 0.1620 | 3.17e-04 | 1.61e-03 |
| ENSMUSG00000017499 | Cdc6 | protein\_coding | 11:98907801-98923940 (+) |  | -0.4300 | 3.18e-04 | 1.62e-03 |
| ENSMUSG00000037740 | Mrps26 | protein\_coding | 2:130563742-130568695 (+) |  | -0.1980 | 3.18e-04 | 1.62e-03 |
| ENSMUSG00000093577 | Gm20632 | lncRNA | 3:96239393-96241819 (+) |  | 1.1600 | 3.19e-04 | 1.62e-03 |
| ENSMUSG00000029833 | Trim24 | protein\_coding | 6:37870811-37966296 (+) |  | 0.2740 | 3.20e-04 | 1.63e-03 |
| ENSMUSG00000034471 | Caskin2 | protein\_coding | 11:115799183-115813639 (-) |  | -0.9230 | 3.20e-04 | 1.63e-03 |
| ENSMUSG00000087213 | 2810408I11Rik | lncRNA | 1:64679871-64690659 (-) |  | -0.4470 | 3.20e-04 | 1.63e-03 |
| ENSMUSG00000032322 | Pstpip1 | protein\_coding | 9:56089962-56128888 (+) |  | 0.1890 | 3.21e-04 | 1.63e-03 |
| ENSMUSG00000038828 | Tmem214 | protein\_coding | 5:30868012-30879180 (+) |  | -0.1800 | 3.21e-04 | 1.63e-03 |
| ENSMUSG00000028957 | Per3 | protein\_coding | 4:151003652-151044665 (-) |  | 0.6910 | 3.23e-04 | 1.64e-03 |
| ENSMUSG00000060992 | Copz1 | protein\_coding | 15:103272714-103299868 (+) |  | -0.1440 | 3.27e-04 | 1.66e-03 |
| ENSMUSG00000091623 | Gm17092 | lncRNA | 5:99977686-99980171 (+) |  | -0.4820 | 3.27e-04 | 1.66e-03 |
| ENSMUSG00000034931 | Dhx8 | protein\_coding | 11:101732919-101767358 (+) |  | -0.2060 | 3.27e-04 | 1.66e-03 |
| ENSMUSG00000030156 | Cd69 | protein\_coding | 6:129267325-129275436 (-) |  | -0.5140 | 3.29e-04 | 1.67e-03 |
| ENSMUSG00000020101 | Vsir | protein\_coding | 10:60346851-60372684 (+) |  | 0.2450 | 3.29e-04 | 1.67e-03 |
| ENSMUSG00000020034 | Tcp11l2 | protein\_coding | 10:84576626-84614359 (+) |  | 0.2990 | 3.31e-04 | 1.68e-03 |
| ENSMUSG00000059409 | Ppp2r5d | protein\_coding | 17:46682991-46705111 (-) |  | -0.1750 | 3.31e-04 | 1.68e-03 |
| ENSMUSG00000027956 | Tmem144 | protein\_coding | 3:79812564-79852773 (-) |  | -0.5520 | 3.31e-04 | 1.68e-03 |
| ENSMUSG00000030188 | Magohb | protein\_coding | 6:131284388-131293244 (-) |  | -0.3710 | 3.33e-04 | 1.68e-03 |
| ENSMUSG00000001924 | Uba1 | protein\_coding | X:20658326-20683179 (+) |  | -0.1590 | 3.33e-04 | 1.68e-03 |
| ENSMUSG00000038299 | Wdr36 | protein\_coding | 18:32837225-32867594 (+) |  | -0.2340 | 3.33e-04 | 1.69e-03 |
| ENSMUSG00000038646 | Ramac | protein\_coding | 7:81762925-81769491 (+) |  | 0.2420 | 3.34e-04 | 1.69e-03 |
| ENSMUSG00000017754 | Pltp | protein\_coding | 2:164839518-164857711 (-) |  | 1.6000 | 3.35e-04 | 1.69e-03 |
| ENSMUSG00000027326 | Knl1 | protein\_coding | 2:119047119-119105501 (+) |  | -0.2110 | 3.36e-04 | 1.70e-03 |
| ENSMUSG00000016257 | Prelid3b | protein\_coding | 2:174465067-174473081 (-) |  | -0.2340 | 3.38e-04 | 1.71e-03 |
| ENSMUSG00000020219 | Timm13 | protein\_coding | 10:80899450-80900969 (-) |  | -0.2460 | 3.39e-04 | 1.71e-03 |
| ENSMUSG00000040105 | Plpp6 | protein\_coding | 19:28963953-28966811 (+) |  | 0.2780 | 3.39e-04 | 1.71e-03 |
| ENSMUSG00000035248 | Tut7 | protein\_coding | 13:59771561-59823147 (-) |  | 0.1410 | 3.40e-04 | 1.72e-03 |
| ENSMUSG00000089929 | Bcl2a1b | protein\_coding | 9:89199209-89207827 (+) |  | 1.7600 | 3.42e-04 | 1.72e-03 |
| ENSMUSG00000039656 | Rxrb | protein\_coding | 17:34031812-34038399 (+) |  | 0.1980 | 3.42e-04 | 1.72e-03 |
| ENSMUSG00000034427 | Myo15b | protein\_coding | 11:115858406-115892603 (+) |  | 0.8980 | 3.44e-04 | 1.73e-03 |
| ENSMUSG00000078485 | Plekhn1 | protein\_coding | 4:156221456-156234857 (-) |  | 0.3020 | 3.44e-04 | 1.74e-03 |
| ENSMUSG00000045767 | B230219D22Rik | protein\_coding | 13:55693124-55703500 (+) |  | 0.1260 | 3.45e-04 | 1.74e-03 |
| ENSMUSG00000027514 | Zbp1 | protein\_coding | 2:173206612-173218923 (-) |  | -0.6390 | 3.45e-04 | 1.74e-03 |
| ENSMUSG00000058704 | Memo1 | protein\_coding | 17:74199036-74295521 (-) |  | -0.2170 | 3.47e-04 | 1.75e-03 |
| ENSMUSG00000026791 | Slc2a8 | protein\_coding | 2:32972990-32982083 (-) |  | 0.3710 | 3.47e-04 | 1.75e-03 |
| ENSMUSG00000048538 | Gm9826 | processed\_pseudogene | 1:7177739-7179037 (+) |  | -0.4990 | 3.48e-04 | 1.75e-03 |
| ENSMUSG00000114277 | Gm48583 | processed\_pseudogene | 13:60641790-60642009 (+) |  | 0.2570 | 3.51e-04 | 1.77e-03 |
| ENSMUSG00000045251 | Zfp688 | protein\_coding | 7:127418967-127422068 (-) |  | 0.3500 | 3.55e-04 | 1.78e-03 |
| ENSMUSG00000055760 | Gemin6 | protein\_coding | 17:80224441-80228497 (+) |  | -0.3360 | 3.55e-04 | 1.78e-03 |
| ENSMUSG00000078427 | Sarnp | protein\_coding | 10:128817333-128877629 (+) |  | -0.3790 | 3.58e-04 | 1.80e-03 |
| ENSMUSG00000063235 | Ptpmt1 | protein\_coding | 2:90908716-90918258 (-) |  | -0.2950 | 3.60e-04 | 1.80e-03 |
| ENSMUSG00000031568 | Rwdd4a | protein\_coding | 8:47533664-47552955 (+) |  | -0.1910 | 3.60e-04 | 1.80e-03 |
| ENSMUSG00000038764 | Ptpn3 | protein\_coding | 4:57190841-57301837 (-) |  | -0.8170 | 3.61e-04 | 1.81e-03 |
| ENSMUSG00000061544 | Zfp229 | protein\_coding | 17:21730795-21769342 (+) |  | 0.3990 | 3.61e-04 | 1.81e-03 |
| ENSMUSG00000049106 | Dcaf5 | protein\_coding | 12:80335848-80436601 (-) |  | 0.2010 | 3.62e-04 | 1.81e-03 |
| ENSMUSG00000039814 | Xkr5 | protein\_coding | 8:18932729-18950975 (-) |  | 0.2860 | 3.63e-04 | 1.82e-03 |
| ENSMUSG00000021493 | Pdlim7 | protein\_coding | 13:55495795-55513676 (-) |  | 0.3790 | 3.63e-04 | 1.82e-03 |
| ENSMUSG00000038170 | Pde4dip | protein\_coding | 3:97689824-97888707 (-) |  | 0.3070 | 3.64e-04 | 1.82e-03 |
| ENSMUSG00000034064 | Poglut1 | protein\_coding | 16:38525137-38550258 (-) |  | -0.2250 | 3.66e-04 | 1.83e-03 |
| ENSMUSG00000096740 | Lbhd1 | protein\_coding | 19:8883732-8892627 (+) |  | -0.3690 | 3.69e-04 | 1.85e-03 |
| ENSMUSG00000037487 | Ubr5 | protein\_coding | 15:37967328-38078854 (-) |  | -0.2050 | 3.71e-04 | 1.85e-03 |
| ENSMUSG00000031352 | Hccs | protein\_coding | X:169250193-169320372 (-) |  | -0.2260 | 3.71e-04 | 1.86e-03 |
| ENSMUSG00000079083 | Jrkl | protein\_coding | 9:13242607-13245829 (-) |  | 0.2970 | 3.71e-04 | 1.86e-03 |
| ENSMUSG00000057637 | Prdm2 | protein\_coding | 4:143107391-143212995 (-) |  | 0.1800 | 3.73e-04 | 1.87e-03 |
| ENSMUSG00000005800 | Mmp8 | protein\_coding | 9:7558456-7568485 (+) |  | -0.5340 | 3.74e-04 | 1.87e-03 |
| ENSMUSG00000027552 | E2f5 | protein\_coding | 3:14578641-14606309 (+) |  | -0.6670 | 3.74e-04 | 1.87e-03 |
| ENSMUSG00000045730 | Adrb2 | protein\_coding | 18:62177816-62179959 (-) |  | 0.7830 | 3.75e-04 | 1.87e-03 |
| ENSMUSG00000033222 | Ttf2 | protein\_coding | 3:100938860-100969663 (-) |  | -0.2150 | 3.77e-04 | 1.88e-03 |
| ENSMUSG00000005823 | Gpr108 | protein\_coding | 17:57234635-57248446 (-) |  | 0.1530 | 3.77e-04 | 1.88e-03 |
| ENSMUSG00000049709 | Nlrp10 | protein\_coding | 7:108921852-108930178 (-) |  | 0.7830 | 3.77e-04 | 1.88e-03 |
| ENSMUSG00000038178 | Slc43a2 | protein\_coding | 11:75531694-75577575 (+) |  | 0.2070 | 3.78e-04 | 1.89e-03 |
| ENSMUSG00000004070 | Hmox2 | protein\_coding | 16:4726361-4766742 (+) |  | -0.2130 | 3.79e-04 | 1.89e-03 |
| ENSMUSG00000035623 | Rsf1 | protein\_coding | 7:97579889-97692778 (+) |  | 0.2220 | 3.82e-04 | 1.90e-03 |
| ENSMUSG00000037355 | Uvssa | protein\_coding | 5:33378549-33419754 (+) |  | 0.3320 | 3.83e-04 | 1.91e-03 |
| ENSMUSG00000035181 | Heatr5a | protein\_coding | 12:51875871-51971321 (-) |  | 0.1750 | 3.84e-04 | 1.91e-03 |
| ENSMUSG00000001020 | S100a4 | protein\_coding | 3:90603771-90606045 (+) |  | 0.3970 | 3.85e-04 | 1.92e-03 |
| ENSMUSG00000022377 | Asap1 | protein\_coding | 15:64086857-64382919 (-) |  | -0.1830 | 3.86e-04 | 1.92e-03 |
| ENSMUSG00000106205 | C230096K16Rik | TEC | 5:64804014-64805735 (+) |  | 0.9630 | 3.87e-04 | 1.93e-03 |
| ENSMUSG00000024425 | Ndfip1 | protein\_coding | 18:38410396-38465303 (+) |  | 0.2630 | 3.87e-04 | 1.93e-03 |
| ENSMUSG00000069255 | Dusp22 | protein\_coding | 13:30659999-30711231 (+) |  | 0.3430 | 3.88e-04 | 1.93e-03 |
| ENSMUSG00000110935 | Gm8834 | processed\_pseudogene | 10:34898256-34898820 (+) |  | 0.8330 | 3.89e-04 | 1.93e-03 |
| ENSMUSG00000018821 | Avpi1 | protein\_coding | 19:42123273-42129059 (-) |  | -0.5900 | 3.91e-04 | 1.94e-03 |
| ENSMUSG00000089917 | Uckl1 | protein\_coding | 2:181569149-181584892 (-) |  | 0.2260 | 3.92e-04 | 1.95e-03 |
| ENSMUSG00000047996 | Prrg1 | protein\_coding | X:78449613-78583896 (-) |  | 0.4350 | 3.93e-04 | 1.95e-03 |
| ENSMUSG00000090031 | 4732440D04Rik | lncRNA | 1:6209866-6215293 (-) |  | 0.6700 | 3.94e-04 | 1.95e-03 |
| ENSMUSG00000037531 | Mrpl47 | protein\_coding | 3:32725397-32737605 (-) |  | -0.3450 | 3.94e-04 | 1.96e-03 |
| ENSMUSG00000025959 | Klf7 | protein\_coding | 1:64029447-64122282 (-) |  | -0.3310 | 3.97e-04 | 1.97e-03 |
| ENSMUSG00000025795 | Rassf3 | protein\_coding | 10:121410350-121476347 (-) |  | 0.1760 | 3.98e-04 | 1.97e-03 |
| ENSMUSG00000021822 | Plau | protein\_coding | 14:20836660-20843385 (+) |  | 0.3960 | 3.99e-04 | 1.98e-03 |
| ENSMUSG00000025544 | Tm9sf2 | protein\_coding | 14:122107038-122159604 (+) |  | -0.1380 | 4.00e-04 | 1.98e-03 |
| ENSMUSG00000044201 | Cdc25c | protein\_coding | 18:34732993-34751533 (-) |  | -0.2560 | 4.01e-04 | 1.98e-03 |
| ENSMUSG00000039474 | Wfs1 | protein\_coding | 5:36966104-36989205 (-) |  | 0.4440 | 4.01e-04 | 1.98e-03 |
| ENSMUSG00000083678 | Gm12989 | processed\_pseudogene | 4:135715868-135716598 (-) |  | -0.3140 | 4.03e-04 | 1.99e-03 |
| ENSMUSG00000020741 | Cluh | protein\_coding | 11:74649495-74670847 (+) |  | -0.2510 | 4.05e-04 | 2.00e-03 |
| ENSMUSG00000042351 | Grap2 | protein\_coding | 15:80572594-80652854 (+) |  | 0.4360 | 4.06e-04 | 2.01e-03 |
| ENSMUSG00000018509 | Cenpv | protein\_coding | 11:62524946-62539261 (-) |  | -0.4940 | 4.06e-04 | 2.01e-03 |
| ENSMUSG00000113749 | Mrto4-ps1 | processed\_pseudogene | 12:20417722-20418442 (+) |  | -0.6720 | 4.07e-04 | 2.01e-03 |
| ENSMUSG00000085531 | Slc36a3os | lncRNA | 11:55137044-55140193 (+) |  | 1.0900 | 4.07e-04 | 2.01e-03 |
| ENSMUSG00000025736 | Jmjd8 | protein\_coding | 17:25828867-25831843 (+) |  | -0.2990 | 4.08e-04 | 2.02e-03 |
| ENSMUSG00000004788 | Eif2b2 | protein\_coding | 12:85219481-85226628 (+) |  | -0.1910 | 4.08e-04 | 2.02e-03 |
| ENSMUSG00000018570 | 2810408A11Rik | protein\_coding | 11:69897352-69900987 (-) |  | 0.8060 | 4.09e-04 | 2.02e-03 |
| ENSMUSG00000023087 | Noct | protein\_coding | 3:51224447-51251644 (+) |  | -0.3040 | 4.12e-04 | 2.03e-03 |
| ENSMUSG00000025153 | Fasn | protein\_coding | 11:120805846-120824547 (-) |  | -0.3730 | 4.13e-04 | 2.03e-03 |
| ENSMUSG00000068243 | Gm7079 | processed\_pseudogene | X:8047229-8047399 (+) |  | 1.1400 | 4.14e-04 | 2.04e-03 |
| ENSMUSG00000034126 | Pomt2 | protein\_coding | 12:87106861-87147968 (-) |  | -0.3050 | 4.17e-04 | 2.05e-03 |
| ENSMUSG00000059981 | Taok2 | protein\_coding | 7:126865678-126884703 (-) |  | 0.1710 | 4.19e-04 | 2.06e-03 |
| ENSMUSG00000002222 | Rmnd5a | protein\_coding | 6:71388634-71440637 (-) |  | 0.2350 | 4.19e-04 | 2.06e-03 |
| ENSMUSG00000027014 | Cwc22 | protein\_coding | 2:77881159-77946375 (-) |  | -0.1620 | 4.20e-04 | 2.07e-03 |
| ENSMUSG00000025871 | 4833439L19Rik | protein\_coding | 13:54551218-54565435 (-) |  | -0.1310 | 4.20e-04 | 2.07e-03 |
| ENSMUSG00000020668 | Kif3c | protein\_coding | 12:3365132-3406494 (+) |  | 0.4950 | 4.20e-04 | 2.07e-03 |
| ENSMUSG00000021546 | Hnrnpk | protein\_coding | 13:58391142-58403343 (-) |  | -0.1240 | 4.21e-04 | 2.07e-03 |
| ENSMUSG00000019977 | Hbs1l | protein\_coding | 10:21295979-21368898 (+) |  | -0.1680 | 4.21e-04 | 2.07e-03 |
| ENSMUSG00000047139 | Cd24a | protein\_coding | 10:43578284-43584265 (+) |  | 0.1590 | 4.21e-04 | 2.07e-03 |
| ENSMUSG00000021832 | Psmc6 | protein\_coding | 14:45329788-45349705 (+) |  | -0.3100 | 4.23e-04 | 2.08e-03 |
| ENSMUSG00000026177 | Slc11a1 | protein\_coding | 1:74375195-74386062 (+) |  | 0.7220 | 4.23e-04 | 2.08e-03 |
| ENSMUSG00000035266 | Helq | protein\_coding | 5:100762145-100798598 (-) |  | -0.3290 | 4.23e-04 | 2.08e-03 |
| ENSMUSG00000039914 | Coq10a | protein\_coding | 10:128363100-128368997 (-) |  | 0.2770 | 4.24e-04 | 2.08e-03 |
| ENSMUSG00000044328 | Trp53i13 | protein\_coding | 11:77508099-77515980 (-) |  | 0.3290 | 4.27e-04 | 2.09e-03 |
| ENSMUSG00000058492 | Scp2-ps2 | transcribed\_unprocessed\_pseudogene | 9:123306600-123317998 (-) |  | 0.2590 | 4.28e-04 | 2.10e-03 |
| ENSMUSG00000038250 | Usp38 | protein\_coding | 8:80980733-81014928 (-) |  | -0.1680 | 4.28e-04 | 2.10e-03 |
| ENSMUSG00000048200 | Cracr2b | protein\_coding | 7:141461094-141466613 (+) |  | 0.7920 | 4.28e-04 | 2.10e-03 |
| ENSMUSG00000038374 | Rbm8a | protein\_coding | 3:96629933-96633791 (+) |  | -0.1820 | 4.31e-04 | 2.11e-03 |
| ENSMUSG00000022536 | Glyr1 | protein\_coding | 16:5013909-5049863 (-) |  | -0.1160 | 4.32e-04 | 2.11e-03 |
| ENSMUSG00000051285 | Pcmtd1 | protein\_coding | 1:7088920-7173628 (+) |  | 0.2550 | 4.33e-04 | 2.12e-03 |
| ENSMUSG00000074358 | Ccdc61 | protein\_coding | 7:18890883-18910415 (-) |  | 0.2960 | 4.33e-04 | 2.12e-03 |
| ENSMUSG00000025066 | Sfr1 | protein\_coding | 19:47731682-47735588 (+) |  | -0.1640 | 4.35e-04 | 2.13e-03 |
| ENSMUSG00000040112 | Mrps35 | protein\_coding | 6:147042764-147073991 (+) |  | -0.2320 | 4.36e-04 | 2.13e-03 |
| ENSMUSG00000017802 | Retreg3 | protein\_coding | 11:101096322-101119893 (-) |  | 0.1410 | 4.36e-04 | 2.13e-03 |
| ENSMUSG00000042978 | Sbk1 | protein\_coding | 7:126248862-126295016 (+) |  | 0.3710 | 4.36e-04 | 2.13e-03 |
| ENSMUSG00000097835 | Gm26910 | lncRNA | 18:67773712-67774857 (-) |  | 1.1600 | 4.37e-04 | 2.14e-03 |
| ENSMUSG00000110545 | Gm7730 | processed\_pseudogene | 8:70010549-70011002 (-) |  | -0.3420 | 4.40e-04 | 2.15e-03 |
| ENSMUSG00000076621 | Ighj1 | IG\_J\_gene | 12:113429781-113429833 (-) |  | 0.7910 | 4.42e-04 | 2.16e-03 |
| ENSMUSG00000055401 | Fbxo6 | protein\_coding | 4:148145716-148152140 (-) |  | 0.2800 | 4.42e-04 | 2.16e-03 |
| ENSMUSG00000039183 | Nubp2 | protein\_coding | 17:24882611-24886349 (-) |  | -0.1880 | 4.43e-04 | 2.16e-03 |
| ENSMUSG00000032370 | Lactb | protein\_coding | 9:66955388-66975484 (-) |  | -0.2100 | 4.43e-04 | 2.16e-03 |
| ENSMUSG00000037935 | Smarce1 | protein\_coding | 11:99209047-99231017 (-) |  | -0.1660 | 4.46e-04 | 2.17e-03 |
| ENSMUSG00000018362 | Kpna2 | protein\_coding | 11:106988629-106999541 (-) |  | -0.1770 | 4.47e-04 | 2.18e-03 |
| ENSMUSG00000031821 | Gins2 | protein\_coding | 8:120578633-120589304 (-) |  | -0.3460 | 4.48e-04 | 2.19e-03 |
| ENSMUSG00000081871 | Gm11488 | processed\_pseudogene | 4:73497615-73498477 (-) |  | 0.6710 | 4.53e-04 | 2.21e-03 |
| ENSMUSG00000015127 | Unkl | protein\_coding | 17:25188397-25234443 (+) |  | 0.5020 | 4.54e-04 | 2.21e-03 |
| ENSMUSG00000024781 | Lipa | protein\_coding | 19:34492318-34527474 (-) |  | 0.1890 | 4.54e-04 | 2.21e-03 |
| ENSMUSG00000038827 | Abitram | protein\_coding | 4:56802345-56809601 (+) |  | -0.2130 | 4.55e-04 | 2.21e-03 |
| ENSMUSG00000006678 | Pola1 | protein\_coding | X:93304767-93632155 (-) |  | -0.1980 | 4.55e-04 | 2.21e-03 |
| ENSMUSG00000036959 | Bcorl1 | protein\_coding | X:48341358-48408049 (+) |  | 0.3760 | 4.56e-04 | 2.22e-03 |
| ENSMUSG00000036061 | Smug1 | protein\_coding | 15:103153290-103167092 (-) |  | 0.2750 | 4.57e-04 | 2.22e-03 |
| ENSMUSG00000080845 | Gm9115 | processed\_pseudogene | X:102724402-102725749 (-) |  | -0.8480 | 4.58e-04 | 2.23e-03 |
| ENSMUSG00000020840 | Blmh | protein\_coding | 11:76924809-76987379 (+) |  | -0.1900 | 4.59e-04 | 2.23e-03 |
| ENSMUSG00000117284 | Gm7072 | protein\_coding | 17:22285871-22314837 (-) |  | 0.2760 | 4.60e-04 | 2.23e-03 |
| ENSMUSG00000001986 | Gria3 | protein\_coding | X:41400854-41678601 (+) |  | -0.3720 | 4.61e-04 | 2.24e-03 |
| ENSMUSG00000024181 | Mrpl28 | protein\_coding | 17:26123500-26126613 (+) |  | -0.1840 | 4.61e-04 | 2.24e-03 |
| ENSMUSG00000045594 | Glb1 | protein\_coding | 9:114401076-114474898 (+) |  | -0.2140 | 4.62e-04 | 2.24e-03 |
| ENSMUSG00000030303 | Far2 | protein\_coding | 6:148047259-148182758 (+) |  | 0.4450 | 4.63e-04 | 2.25e-03 |
| ENSMUSG00000054693 | Adam10 | protein\_coding | 9:70678997-70780229 (+) |  | -0.1660 | 4.64e-04 | 2.25e-03 |
| ENSMUSG00000020376 | Rnf130 | protein\_coding | 11:50025346-50125719 (+) |  | 0.1940 | 4.68e-04 | 2.27e-03 |
| ENSMUSG00000015536 | Mocs2 | protein\_coding | 13:114818236-114832275 (+) |  | 0.2580 | 4.68e-04 | 2.27e-03 |
| ENSMUSG00000021375 | Kif13a | protein\_coding | 13:46749087-46929867 (-) |  | 0.2170 | 4.69e-04 | 2.27e-03 |
| ENSMUSG00000031506 | Ptpn7 | protein\_coding | 1:135132700-135145317 (+) |  | -0.1520 | 4.70e-04 | 2.27e-03 |
| ENSMUSG00000037907 | Ankrd13b | protein\_coding | 11:77470485-77489678 (-) |  | 0.4290 | 4.70e-04 | 2.27e-03 |
| ENSMUSG00000029648 | Flt1 | protein\_coding | 5:147561604-147726011 (-) |  | 0.9310 | 4.71e-04 | 2.28e-03 |
| ENSMUSG00000050192 | Eif5a2 | protein\_coding | 3:28781276-28798846 (+) |  | 0.3640 | 4.73e-04 | 2.29e-03 |
| ENSMUSG00000034663 | Bmp2k | protein\_coding | 5:96997689-97091867 (+) |  | -0.2400 | 4.73e-04 | 2.29e-03 |
| ENSMUSG00000031864 | Ints10 | protein\_coding | 8:68793929-68831667 (+) |  | -0.1590 | 4.74e-04 | 2.29e-03 |
| ENSMUSG00000022216 | Psme1 | protein\_coding | 14:55578123-55581529 (+) |  | -0.2170 | 4.76e-04 | 2.30e-03 |
| ENSMUSG00000037058 | Paip2 | protein\_coding | 18:35598617-35617187 (+) |  | 0.2310 | 4.78e-04 | 2.31e-03 |
| ENSMUSG00000041220 | Elovl6 | protein\_coding | 3:129532355-129638495 (+) |  | -0.2680 | 4.78e-04 | 2.31e-03 |
| ENSMUSG00000021767 | Kat6b | protein\_coding | 14:21481434-21672478 (+) |  | 0.2700 | 4.78e-04 | 2.31e-03 |
| ENSMUSG00000059316 | Slc27a4 | protein\_coding | 2:29802634-29817522 (+) |  | 0.1830 | 4.78e-04 | 2.31e-03 |
| ENSMUSG00000060073 | Psma3 | protein\_coding | 12:70974621-70996347 (+) |  | -0.2410 | 4.80e-04 | 2.32e-03 |
| ENSMUSG00000019802 | Sec63 | protein\_coding | 10:42761496-42832514 (+) |  | -0.1610 | 4.81e-04 | 2.32e-03 |
| ENSMUSG00000022601 | Zbtb11 | protein\_coding | 16:55973883-56008913 (+) |  | -0.2150 | 4.82e-04 | 2.32e-03 |
| ENSMUSG00000028211 | Trp53inp1 | protein\_coding | 4:11156431-11174379 (+) |  | 0.3700 | 4.84e-04 | 2.33e-03 |
| ENSMUSG00000020464 | Pnpt1 | protein\_coding | 11:29130744-29161828 (+) |  | -0.2210 | 4.84e-04 | 2.33e-03 |
| ENSMUSG00000022114 | Spry2 | protein\_coding | 14:105891947-105896819 (-) |  | -0.8900 | 4.84e-04 | 2.33e-03 |
| ENSMUSG00000027752 | Exosc8 | protein\_coding | 3:54728678-54735393 (-) |  | -0.1700 | 4.85e-04 | 2.34e-03 |
| ENSMUSG00000008604 | Ubqln4 | protein\_coding | 3:88553758-88569725 (+) |  | -0.2110 | 4.85e-04 | 2.34e-03 |
| ENSMUSG00000030347 | D6Wsu163e | protein\_coding | 6:126939962-126975967 (+) |  | -0.1840 | 4.87e-04 | 2.35e-03 |
| ENSMUSG00000029714 | Gigyf1 | protein\_coding | 5:137518548-137527935 (+) |  | 0.3120 | 4.88e-04 | 2.35e-03 |
| ENSMUSG00000028470 | Hint2 | protein\_coding | 4:43654227-43656466 (-) |  | 0.3760 | 4.90e-04 | 2.36e-03 |
| ENSMUSG00000034343 | Ube2f | protein\_coding | 1:91250304-91290337 (+) |  | -0.1960 | 4.91e-04 | 2.36e-03 |
| ENSMUSG00000072618 | Gm10384 | lncRNA | 15:36870501-36879816 (-) |  | 0.4730 | 4.93e-04 | 2.37e-03 |
| ENSMUSG00000048118 | Arid4a | protein\_coding | 12:71015990-71098592 (+) |  | 0.2010 | 4.93e-04 | 2.37e-03 |
| ENSMUSG00000017830 | Dhx58 | protein\_coding | 11:100694884-100704271 (-) |  | 0.2770 | 4.93e-04 | 2.37e-03 |
| ENSMUSG00000040007 | Bahd1 | protein\_coding | 2:118900377-118924528 (+) |  | -0.1850 | 4.93e-04 | 2.37e-03 |
| ENSMUSG00000075595 | Zfp652 | protein\_coding | 11:95712673-95835115 (+) |  | 0.2610 | 4.95e-04 | 2.38e-03 |
| ENSMUSG00000024312 | Wdr46 | protein\_coding | 17:33940660-33949697 (+) |  | -0.2480 | 4.95e-04 | 2.38e-03 |
| ENSMUSG00000017716 | Birc5 | protein\_coding | 11:117849251-117855743 (+) |  | -0.1910 | 4.97e-04 | 2.38e-03 |
| ENSMUSG00000039621 | Prex1 | protein\_coding | 2:166566342-166713832 (-) |  | 0.2190 | 4.97e-04 | 2.38e-03 |
| ENSMUSG00000066721 | Zfp575 | protein\_coding | 7:24583838-24587641 (-) |  | 1.0800 | 5.02e-04 | 2.41e-03 |
| ENSMUSG00000118264 | Rps15-ps3 | transcribed\_processed\_pseudogene | 18:64288135-64291820 (+) |  | 0.6690 | 5.04e-04 | 2.41e-03 |
| ENSMUSG00000037921 | Ddx60 | protein\_coding | 8:61928087-62038244 (+) |  | 0.6910 | 5.05e-04 | 2.42e-03 |
| ENSMUSG00000022092 | Ppp3cc | protein\_coding | 14:70217865-70289471 (-) |  | -0.6870 | 5.08e-04 | 2.43e-03 |
| ENSMUSG00000024589 | Nedd4l | protein\_coding | 18:64887705-65217828 (+) |  | 0.2590 | 5.08e-04 | 2.43e-03 |
| ENSMUSG00000040940 | Arhgef1 | protein\_coding | 7:24902912-24926594 (+) |  | 0.2030 | 5.12e-04 | 2.45e-03 |
| ENSMUSG00000038692 | Hoxb4 | protein\_coding | 11:96318267-96321638 (+) |  | -0.2790 | 5.12e-04 | 2.45e-03 |
| ENSMUSG00000039997 | Ifi203 | protein\_coding | 1:173920407-173942672 (-) |  | 0.3040 | 5.12e-04 | 2.45e-03 |
| ENSMUSG00000038976 | Ppp1r9b | protein\_coding | 11:94991035-95006899 (+) |  | 0.1700 | 5.13e-04 | 2.45e-03 |
| ENSMUSG00000027613 | Eif6 | protein\_coding | 2:155819832-155826925 (-) |  | -0.1950 | 5.15e-04 | 2.46e-03 |
| ENSMUSG00000027509 | Rae1 | protein\_coding | 2:173000117-173015739 (+) |  | -0.1910 | 5.15e-04 | 2.46e-03 |
| ENSMUSG00000067121 | Gm7027 | processed\_pseudogene | 7:101573460-101574321 (-) |  | -0.3070 | 5.16e-04 | 2.47e-03 |
| ENSMUSG00000040272 | Accs | protein\_coding | 2:93833467-93849943 (-) |  | 0.3690 | 5.18e-04 | 2.47e-03 |
| ENSMUSG00000046841 | Ckap4 | protein\_coding | 10:84526305-84534062 (-) |  | -0.2150 | 5.19e-04 | 2.48e-03 |
| ENSMUSG00000020605 | Hs1bp3 | protein\_coding | 12:8313432-8343824 (+) |  | 0.3810 | 5.20e-04 | 2.48e-03 |
| ENSMUSG00000078713 | Tomm5 | protein\_coding | 4:45105208-45108114 (-) |  | -0.3430 | 5.20e-04 | 2.48e-03 |
| ENSMUSG00000015668 | Pdzd11 | protein\_coding | X:100622883-100626568 (-) |  | -0.2190 | 5.21e-04 | 2.48e-03 |
| ENSMUSG00000040560 | Wdr7 | protein\_coding | 18:63708685-63989776 (+) |  | -0.2430 | 5.24e-04 | 2.50e-03 |
| ENSMUSG00000007610 | Gtpbp3 | protein\_coding | 8:71488103-71499583 (+) |  | -0.2060 | 5.26e-04 | 2.50e-03 |
| ENSMUSG00000006411 | Nectin4 | protein\_coding | 1:171370099-171388598 (+) |  | 0.4060 | 5.26e-04 | 2.50e-03 |
| ENSMUSG00000087060 | Eldr | lncRNA | 11:16935154-16951282 (-) |  | 0.5970 | 5.26e-04 | 2.51e-03 |
| ENSMUSG00000028668 | Eloa | protein\_coding | 4:136003368-136021763 (-) |  | -0.1510 | 5.27e-04 | 2.51e-03 |
| ENSMUSG00000061482 | H4c4 | protein\_coding | 13:23581598-23582735 (+) |  | 0.6980 | 5.29e-04 | 2.52e-03 |
| ENSMUSG00000009630 | Ppp2cb | protein\_coding | 8:33599625-33619441 (+) |  | -0.2630 | 5.29e-04 | 2.52e-03 |
| ENSMUSG00000046324 | Ermp1 | protein\_coding | 19:29608214-29648415 (-) |  | -0.1570 | 5.30e-04 | 2.52e-03 |
| ENSMUSG00000069270 | H2ac6 | protein\_coding | 13:23681467-23683948 (-) |  | 1.2900 | 5.31e-04 | 2.53e-03 |
| ENSMUSG00000052724 | Gm9888 | lncRNA | 9:114780100-114782257 (+) |  | 0.5350 | 5.32e-04 | 2.53e-03 |
| ENSMUSG00000048285 | Frmd6 | protein\_coding | 12:70825514-70902234 (+) |  | -0.6980 | 5.38e-04 | 2.56e-03 |
| ENSMUSG00000023800 | Tiam2 | protein\_coding | 17:3326573-3531344 (+) |  | 0.2210 | 5.39e-04 | 2.56e-03 |
| ENSMUSG00000095280 | Gm21738 | protein\_coding | 14:19415857-19418930 (-) |  | 5.0000 | 5.39e-04 | 2.56e-03 |
| ENSMUSG00000058927 | Gm10053 | protein\_coding | 19:24875686-24876631 (+) |  | -0.3570 | 5.39e-04 | 2.56e-03 |
| ENSMUSG00000006519 | Cyba | protein\_coding | 8:122424776-122432930 (-) |  | 0.2450 | 5.40e-04 | 2.56e-03 |
| ENSMUSG00000044709 | Gemin7 | protein\_coding | 7:19564946-19577595 (-) |  | 0.2290 | 5.41e-04 | 2.57e-03 |
| ENSMUSG00000021326 | Trim27 | protein\_coding | 13:21179445-21194724 (+) |  | -0.1790 | 5.42e-04 | 2.57e-03 |
| ENSMUSG00000098702 | 1500015A07Rik | lncRNA | 18:61726390-61735917 (+) |  | 0.5100 | 5.43e-04 | 2.58e-03 |
| ENSMUSG00000050921 | P2ry10 | protein\_coding | X:107088492-107104974 (+) |  | 0.6910 | 5.45e-04 | 2.58e-03 |
| ENSMUSG00000024213 | Nudt3 | protein\_coding | 17:27579382-27623495 (-) |  | -0.2160 | 5.45e-04 | 2.58e-03 |
| ENSMUSG00000030707 | Coro1a | protein\_coding | 7:126699773-126707787 (-) |  | 0.2220 | 5.46e-04 | 2.58e-03 |
| ENSMUSG00000021124 | Vti1b | protein\_coding | 12:79156017-79172667 (-) |  | -0.2130 | 5.48e-04 | 2.59e-03 |
| ENSMUSG00000032194 | Kank2 | protein\_coding | 9:21766784-21798744 (-) |  | 0.6190 | 5.48e-04 | 2.59e-03 |
| ENSMUSG00000043384 | Gprasp1 | protein\_coding | X:135742733-135803474 (+) |  | 0.2470 | 5.49e-04 | 2.60e-03 |
| ENSMUSG00000052144 | Ppp4r2 | protein\_coding | 6:100833622-100869934 (+) |  | -0.2280 | 5.49e-04 | 2.60e-03 |
| ENSMUSG00000001942 | Siae | protein\_coding | 9:37555698-37649655 (+) |  | 0.2630 | 5.50e-04 | 2.60e-03 |
| ENSMUSG00000027469 | Tpx2 | protein\_coding | 2:152847964-152895321 (+) |  | -0.2080 | 5.53e-04 | 2.61e-03 |
| ENSMUSG00000038489 | Polr2l | protein\_coding | 7:141471860-141475132 (-) |  | -0.3260 | 5.53e-04 | 2.61e-03 |
| ENSMUSG00000042148 | Cox10 | protein\_coding | 11:63962627-64079468 (-) |  | -0.2530 | 5.54e-04 | 2.61e-03 |
| ENSMUSG00000005687 | Bcas2 | protein\_coding | 3:103171655-103179166 (+) |  | -0.1630 | 5.54e-04 | 2.62e-03 |
| ENSMUSG00000110129 | Gm45453 | TEC | 8:22410457-22411309 (+) |  | 1.0600 | 5.54e-04 | 2.62e-03 |
| ENSMUSG00000024083 | Pja2 | protein\_coding | 17:64281005-64331916 (-) |  | 0.1770 | 5.55e-04 | 2.62e-03 |
| ENSMUSG00000031753 | Cog4 | protein\_coding | 8:110846600-110882227 (+) |  | -0.1600 | 5.57e-04 | 2.63e-03 |
| ENSMUSG00000020225 | Tmbim4 | protein\_coding | 10:120201590-120224917 (+) |  | 0.1920 | 5.60e-04 | 2.64e-03 |
| ENSMUSG00000031154 | Otud5 | protein\_coding | X:7841364-7876626 (+) |  | 0.1380 | 5.64e-04 | 2.66e-03 |
| ENSMUSG00000026000 | Lancl1 | protein\_coding | 1:67000517-67038872 (-) |  | 0.3090 | 5.64e-04 | 2.66e-03 |
| ENSMUSG00000056665 | Them6 | protein\_coding | 15:74721204-74724639 (+) |  | -0.4100 | 5.64e-04 | 2.66e-03 |
| ENSMUSG00000040151 | Hs2st1 | protein\_coding | 3:144429706-144570181 (-) |  | -0.1890 | 5.66e-04 | 2.66e-03 |
| ENSMUSG00000072940 | Gm10443 | processed\_pseudogene | 6:86334031-86334240 (+) |  | 0.2440 | 5.67e-04 | 2.67e-03 |
| ENSMUSG00000021271 | Zfp839 | protein\_coding | 12:110850253-110869996 (+) |  | 0.2980 | 5.72e-04 | 2.69e-03 |
| ENSMUSG00000022545 | Ercc4 | protein\_coding | 16:13109684-13150617 (+) |  | 0.1980 | 5.72e-04 | 2.69e-03 |
| ENSMUSG00000072872 | Rybp | protein\_coding | 6:100228565-100287485 (-) |  | -0.2320 | 5.72e-04 | 2.69e-03 |
| ENSMUSG00000067629 | Syngap1 | protein\_coding | 17:26941253-26972434 (+) |  | 0.7710 | 5.73e-04 | 2.69e-03 |
| ENSMUSG00000041319 | Thoc6 | protein\_coding | 17:23668614-23673882 (-) |  | -0.2140 | 5.75e-04 | 2.70e-03 |
| ENSMUSG00000039458 | Mtmr12 | protein\_coding | 15:12205028-12274496 (+) |  | 0.1610 | 5.79e-04 | 2.72e-03 |
| ENSMUSG00000017707 | Serinc3 | protein\_coding | 2:163623272-163645131 (-) |  | 0.1560 | 5.81e-04 | 2.73e-03 |
| ENSMUSG00000039686 | Zer1 | protein\_coding | 2:30097283-30124585 (-) |  | 0.3440 | 5.82e-04 | 2.73e-03 |
| ENSMUSG00000023805 | Synj2 | protein\_coding | 17:5941280-6044290 (+) |  | -0.4860 | 5.83e-04 | 2.74e-03 |
| ENSMUSG00000002625 | Akap8l | protein\_coding | 17:32321424-32350607 (-) |  | 0.2810 | 5.87e-04 | 2.76e-03 |
| ENSMUSG00000034158 | Lrrc58 | protein\_coding | 16:37868389-37888858 (+) |  | -0.1710 | 5.89e-04 | 2.76e-03 |
| ENSMUSG00000021918 | Nek4 | protein\_coding | 14:30951377-30988821 (+) |  | -0.2820 | 5.91e-04 | 2.77e-03 |
| ENSMUSG00000035770 | Dync1li2 | protein\_coding | 8:104417680-104443047 (-) |  | 0.2260 | 5.92e-04 | 2.77e-03 |
| ENSMUSG00000024319 | Vps52 | protein\_coding | 17:33955812-33967035 (+) |  | -0.1850 | 5.93e-04 | 2.78e-03 |
| ENSMUSG00000040414 | Slc25a28 | protein\_coding | 19:43663801-43674881 (-) |  | 0.1970 | 5.95e-04 | 2.79e-03 |
| ENSMUSG00000021981 | Cab39l | protein\_coding | 14:59440972-59585764 (+) |  | -0.2080 | 5.98e-04 | 2.80e-03 |
| ENSMUSG00000022035 | Ccdc25 | protein\_coding | 14:65837302-65866607 (+) |  | -0.2240 | 6.00e-04 | 2.81e-03 |
| ENSMUSG00000022824 | Muc13 | protein\_coding | 16:33794037-33819934 (+) |  | -0.6420 | 6.04e-04 | 2.83e-03 |
| ENSMUSG00000021959 | Lats2 | protein\_coding | 14:57689662-57758388 (-) |  | 0.1960 | 6.06e-04 | 2.84e-03 |
| ENSMUSG00000098320 | Vis1 | misc\_RNA | 2:45280724-45280924 (+) |  | 0.9220 | 6.07e-04 | 2.84e-03 |
| ENSMUSG00000045757 | Zfp764 | protein\_coding | 7:127403668-127406822 (-) |  | 0.3020 | 6.07e-04 | 2.84e-03 |
| ENSMUSG00000021816 | Ppp3cb | protein\_coding | 14:20499364-20546573 (-) |  | -0.1170 | 6.10e-04 | 2.85e-03 |
| ENSMUSG00000022090 | Pdlim2 | protein\_coding | 14:70164218-70177681 (-) |  | 0.3360 | 6.10e-04 | 2.85e-03 |
| ENSMUSG00000038286 | Bphl | protein\_coding | 13:34037597-34074074 (+) |  | 0.2690 | 6.11e-04 | 2.85e-03 |
| ENSMUSG00000032114 | Slc37a4 | protein\_coding | 9:44396852-44402968 (+) |  | 0.2090 | 6.12e-04 | 2.86e-03 |
| ENSMUSG00000032777 | Gtf3c1 | protein\_coding | 7:125640954-125707780 (-) |  | -0.2030 | 6.13e-04 | 2.86e-03 |
| ENSMUSG00000031365 | Zfp275 | protein\_coding | X:73342621-73359080 (+) |  | 0.3180 | 6.21e-04 | 2.90e-03 |
| ENSMUSG00000100629 | Gm28192 | lncRNA | 11:120963844-120964764 (+) |  | 0.8260 | 6.25e-04 | 2.92e-03 |
| ENSMUSG00000031311 | Nono | protein\_coding | X:101429318-101448591 (+) |  | -0.1010 | 6.25e-04 | 2.92e-03 |
| ENSMUSG00000026623 | Lpgat1 | protein\_coding | 1:191717834-191784255 (+) |  | -0.1860 | 6.26e-04 | 2.92e-03 |
| ENSMUSG00000044018 | Mrpl50 | protein\_coding | 4:49512596-49521093 (-) |  | -0.2140 | 6.28e-04 | 2.93e-03 |
| ENSMUSG00000037313 | Tacc3 | protein\_coding | 5:33658128-33678995 (+) |  | -0.1940 | 6.30e-04 | 2.93e-03 |
| ENSMUSG00000104484 | Gm33142 | TEC | 8:120685399-120686265 (+) |  | 0.9830 | 6.33e-04 | 2.95e-03 |
| ENSMUSG00000095115 | Itpripl2 | protein\_coding | 7:118485111-118491975 (-) |  | 0.1480 | 6.34e-04 | 2.95e-03 |
| ENSMUSG00000051341 | Zfp52 | protein\_coding | 17:21535539-21562601 (+) |  | 0.2840 | 6.36e-04 | 2.96e-03 |
| ENSMUSG00000045160 | Bola3 | protein\_coding | 6:83349147-83360136 (+) |  | -0.3580 | 6.37e-04 | 2.96e-03 |
| ENSMUSG00000025920 | Stau2 | protein\_coding | 1:16228674-16520112 (-) |  | -0.6430 | 6.39e-04 | 2.97e-03 |
| ENSMUSG00000116632 | Magef1 | transcribed\_processed\_pseudogene | 16:21331902-21333356 (-) |  | 0.4890 | 6.39e-04 | 2.97e-03 |
| ENSMUSG00000028034 | Fubp1 | protein\_coding | 3:152210422-152236826 (+) |  | -0.1680 | 6.43e-04 | 2.99e-03 |
| ENSMUSG00000004931 | Apba3 | protein\_coding | 10:81266960-81273246 (+) |  | -0.2160 | 6.44e-04 | 2.99e-03 |
| ENSMUSG00000020074 | Ccar1 | protein\_coding | 10:62743928-62792286 (-) |  | -0.1530 | 6.46e-04 | 3.00e-03 |
| ENSMUSG00000020263 | Appl2 | protein\_coding | 10:83600033-83648738 (-) |  | 0.3620 | 6.46e-04 | 3.00e-03 |
| ENSMUSG00000026238 | Ptma | protein\_coding | 1:86526726-86530712 (+) |  | -0.1820 | 6.49e-04 | 3.01e-03 |
| ENSMUSG00000107792 | Gm43914 | lncRNA | 6:129418179-129418772 (+) |  | 1.5300 | 6.50e-04 | 3.02e-03 |
| ENSMUSG00000002395 | Use1 | protein\_coding | 8:71366848-71369732 (+) |  | 0.3350 | 6.51e-04 | 3.02e-03 |
| ENSMUSG00000079426 | Arpc4 | protein\_coding | 6:113378115-113390448 (+) |  | -0.1870 | 6.51e-04 | 3.02e-03 |
| ENSMUSG00000066278 | Vps37b | protein\_coding | 5:124004641-124032270 (-) |  | 0.2680 | 6.51e-04 | 3.02e-03 |
| ENSMUSG00000017861 | Mybl2 | protein\_coding | 2:163054687-163084688 (+) |  | -0.3140 | 6.54e-04 | 3.03e-03 |
| ENSMUSG00000052337 | Immt | protein\_coding | 6:71831331-71877388 (+) |  | -0.1400 | 6.54e-04 | 3.03e-03 |
| ENSMUSG00000000290 | Itgb2 | protein\_coding | 10:77530252-77565708 (+) |  | 0.3030 | 6.57e-04 | 3.04e-03 |
| ENSMUSG00000026980 | Ly75 | protein\_coding | 2:60292103-60383303 (-) |  | -0.4370 | 6.59e-04 | 3.05e-03 |
| ENSMUSG00000037001 | Zfp39 | protein\_coding | 11:58888153-58904225 (-) |  | 0.3410 | 6.60e-04 | 3.05e-03 |
| ENSMUSG00000055866 | Per2 | protein\_coding | 1:91415982-91459324 (-) |  | 0.6560 | 6.64e-04 | 3.07e-03 |
| ENSMUSG00000066270 | Gm10157 | processed\_pseudogene | 9:118837613-118837955 (-) |  | -0.4970 | 6.65e-04 | 3.08e-03 |
| ENSMUSG00000028581 | Laptm5 | protein\_coding | 4:130913125-130936141 (+) |  | 0.1910 | 6.66e-04 | 3.08e-03 |
| ENSMUSG00000015312 | Gadd45b | protein\_coding | 10:80930073-80932204 (+) |  | -0.2770 | 6.66e-04 | 3.08e-03 |
| ENSMUSG00000023262 | Acy1 | protein\_coding | 9:106432981-106438319 (-) |  | -0.3630 | 6.67e-04 | 3.08e-03 |
| ENSMUSG00000022507 | 1810013L24Rik | protein\_coding | 16:8830100-8858922 (+) |  | -0.2060 | 6.70e-04 | 3.09e-03 |
| ENSMUSG00000037108 | Zcwpw1 | protein\_coding | 5:137787798-137822621 (+) |  | 0.4900 | 6.70e-04 | 3.10e-03 |
| ENSMUSG00000027193 | Api5 | protein\_coding | 2:94411682-94438136 (-) |  | -0.1400 | 6.70e-04 | 3.10e-03 |
| ENSMUSG00000021144 | Mta1 | protein\_coding | 12:113098278-113137206 (+) |  | -0.2200 | 6.73e-04 | 3.11e-03 |
| ENSMUSG00000028757 | Ddost | protein\_coding | 4:138304730-138312628 (+) |  | -0.2090 | 6.73e-04 | 3.11e-03 |
| ENSMUSG00000021728 | Emb | protein\_coding | 13:117208536-117274415 (+) |  | -0.1730 | 6.77e-04 | 3.12e-03 |
| ENSMUSG00000022706 | Mrpl40 | protein\_coding | 16:18872018-18876862 (-) |  | -0.2610 | 6.77e-04 | 3.12e-03 |
| ENSMUSG00000040738 | Ints8 | protein\_coding | 4:11199158-11254258 (-) |  | -0.1780 | 6.80e-04 | 3.13e-03 |
| ENSMUSG00000026730 | Pter | protein\_coding | 2:12924041-13003455 (+) |  | -0.3880 | 6.82e-04 | 3.14e-03 |
| ENSMUSG00000061607 | Mdc1 | protein\_coding | 17:35841515-35859670 (+) |  | -0.2410 | 6.82e-04 | 3.14e-03 |
| ENSMUSG00000024974 | Smc3 | protein\_coding | 19:53600398-53645833 (+) |  | -0.1520 | 6.82e-04 | 3.14e-03 |
| ENSMUSG00000027933 | Ints3 | protein\_coding | 3:90391388-90433622 (-) |  | -0.2450 | 6.83e-04 | 3.15e-03 |
| ENSMUSG00000004730 | Adgre1 | protein\_coding | 17:57358691-57483527 (+) |  | 0.3540 | 6.85e-04 | 3.15e-03 |
| ENSMUSG00000028910 | Mecr | protein\_coding | 4:131843470-131867786 (+) |  | -0.3070 | 6.85e-04 | 3.15e-03 |
| ENSMUSG00000031328 | Flna | protein\_coding | X:74223461-74249820 (-) |  | 0.1880 | 6.87e-04 | 3.16e-03 |
| ENSMUSG00000002949 | Timm44 | protein\_coding | 8:4259731-4275913 (-) |  | -0.1560 | 6.89e-04 | 3.17e-03 |
| ENSMUSG00000003731 | Kpna6 | protein\_coding | 4:129643980-129672767 (-) |  | -0.1980 | 6.90e-04 | 3.17e-03 |
| ENSMUSG00000114822 | Gm4813 | processed\_pseudogene | 13:81029103-81029901 (-) |  | 2.3400 | 6.91e-04 | 3.18e-03 |
| ENSMUSG00000079508 | Apoo | protein\_coding | X:94367117-94417093 (+) |  | -0.3820 | 6.92e-04 | 3.18e-03 |
| ENSMUSG00000021610 | Clptm1l | protein\_coding | 13:73604006-73620605 (+) |  | -0.1680 | 6.96e-04 | 3.20e-03 |
| ENSMUSG00000010095 | Slc3a2 | protein\_coding | 19:8706882-8723369 (-) |  | -0.2370 | 6.97e-04 | 3.20e-03 |
| ENSMUSG00000036246 | Gmip | protein\_coding | 8:69808679-69821870 (+) |  | 0.1890 | 6.99e-04 | 3.21e-03 |
| ENSMUSG00000032913 | Lrig2 | protein\_coding | 3:104396418-104511918 (-) |  | 0.2450 | 7.00e-04 | 3.21e-03 |
| ENSMUSG00000083220 | Gm12919 | processed\_pseudogene | 4:10853843-10855436 (+) |  | -0.9320 | 7.00e-04 | 3.21e-03 |
| ENSMUSG00000026094 | Stk17b | protein\_coding | 1:53755506-53785224 (-) |  | 0.3080 | 7.00e-04 | 3.21e-03 |
| ENSMUSG00000036002 | Fam214b | protein\_coding | 4:43032414-43046220 (-) |  | 0.2790 | 7.01e-04 | 3.21e-03 |
| ENSMUSG00000028790 | Khdrbs1 | protein\_coding | 4:129703164-129742303 (-) |  | -0.1310 | 7.03e-04 | 3.22e-03 |
| ENSMUSG00000024811 | Tnks2 | protein\_coding | 19:36834232-36893477 (+) |  | -0.1460 | 7.05e-04 | 3.23e-03 |
| ENSMUSG00000009013 | Dynll1 | protein\_coding | 5:115297110-115300999 (-) |  | -0.1970 | 7.06e-04 | 3.23e-03 |
| ENSMUSG00000022401 | Xpnpep3 | protein\_coding | 15:81400138-81457482 (+) |  | -0.2590 | 7.06e-04 | 3.23e-03 |
| ENSMUSG00000026925 | Inpp5e | protein\_coding | 2:26396249-26409203 (-) |  | 0.2480 | 7.08e-04 | 3.24e-03 |
| ENSMUSG00000028896 | Rcc1 | protein\_coding | 4:132331919-132353605 (-) |  | -0.2180 | 7.08e-04 | 3.24e-03 |
| ENSMUSG00000022369 | Mtbp | protein\_coding | 15:55557408-55626423 (+) |  | -0.2990 | 7.09e-04 | 3.24e-03 |
| ENSMUSG00000095762 | Gm16378 | processed\_pseudogene | 8:122841731-122842072 (+) |  | -0.6860 | 7.10e-04 | 3.25e-03 |
| ENSMUSG00000112537 | 4930543I11Rik | TEC | 10:67785735-67786693 (+) |  | 1.2000 | 7.12e-04 | 3.26e-03 |
| ENSMUSG00000022475 | Hdac7 | protein\_coding | 15:97792664-97844502 (-) |  | 0.3370 | 7.16e-04 | 3.27e-03 |
| ENSMUSG00000032690 | Oas2 | protein\_coding | 5:120730333-120749853 (-) |  | -0.9340 | 7.17e-04 | 3.28e-03 |
| ENSMUSG00000001569 | Nom1 | protein\_coding | 5:29434664-29457843 (+) |  | -0.1460 | 7.17e-04 | 3.28e-03 |
| ENSMUSG00000020733 | Slc9a3r1 | protein\_coding | 11:115163341-115181181 (+) |  | 0.1590 | 7.18e-04 | 3.28e-03 |
| ENSMUSG00000048574 | Ccnb1-ps | processed\_pseudogene | 7:42105899-42107185 (-) |  | -0.4390 | 7.19e-04 | 3.28e-03 |
| ENSMUSG00000004865 | Srpk1 | protein\_coding | 17:28587648-28622709 (-) |  | -0.2020 | 7.21e-04 | 3.29e-03 |
| ENSMUSG00000041528 | Rnf123 | protein\_coding | 9:108051534-108083346 (-) |  | 0.2220 | 7.23e-04 | 3.30e-03 |
| ENSMUSG00000026869 | Psmd5 | protein\_coding | 2:34849734-34874968 (-) |  | -0.2040 | 7.24e-04 | 3.30e-03 |
| ENSMUSG00000032580 | Rbm5 | protein\_coding | 9:107740371-107770996 (-) |  | 0.1760 | 7.24e-04 | 3.30e-03 |
| ENSMUSG00000028890 | Mtf1 | protein\_coding | 4:124802104-124849800 (+) |  | -0.2280 | 7.24e-04 | 3.30e-03 |
| ENSMUSG00000024387 | Csnk2b | protein\_coding | 17:35116196-35122053 (-) |  | -0.1850 | 7.25e-04 | 3.30e-03 |
| ENSMUSG00000023150 | Ivns1abp | protein\_coding | 1:151344477-151364422 (+) |  | 0.2220 | 7.27e-04 | 3.31e-03 |
| ENSMUSG00000011114 | Tbrg1 | protein\_coding | 9:37648763-37657312 (-) |  | -0.2810 | 7.29e-04 | 3.32e-03 |
| ENSMUSG00000015095 | Fbxw5 | protein\_coding | 2:25500750-25505471 (+) |  | 0.1850 | 7.33e-04 | 3.34e-03 |
| ENSMUSG00000032712 | Resf1 | protein\_coding | 6:149309414-149335663 (+) |  | -0.2020 | 7.33e-04 | 3.34e-03 |
| ENSMUSG00000037343 | Taf2 | protein\_coding | 15:55015131-55072152 (-) |  | -0.1950 | 7.34e-04 | 3.34e-03 |
| ENSMUSG00000028857 | Tmem222 | protein\_coding | 4:133266045-133277792 (-) |  | 0.1760 | 7.43e-04 | 3.38e-03 |
| ENSMUSG00000037544 | Dlgap5 | protein\_coding | 14:47387779-47418407 (-) |  | -0.2330 | 7.43e-04 | 3.38e-03 |
| ENSMUSG00000081888 | Spcs2-ps | unprocessed\_pseudogene | 2:129378271-129379396 (-) |  | 0.7160 | 7.43e-04 | 3.38e-03 |
| ENSMUSG00000058126 | Tpm3-rs7 | protein\_coding | 14:113314608-113316754 (+) |  | -0.1860 | 7.43e-04 | 3.38e-03 |
| ENSMUSG00000021712 | Trim23 | protein\_coding | 13:104178797-104203372 (+) |  | 0.2160 | 7.46e-04 | 3.39e-03 |
| ENSMUSG00000019054 | Fis1 | protein\_coding | 5:136953275-136966234 (+) |  | 0.2120 | 7.48e-04 | 3.39e-03 |
| ENSMUSG00000057672 | Pkn1 | protein\_coding | 8:83666536-83699179 (-) |  | 0.1640 | 7.48e-04 | 3.39e-03 |
| ENSMUSG00000003847 | Nfat5 | protein\_coding | 8:107293470-107379517 (+) |  | 0.2890 | 7.58e-04 | 3.44e-03 |
| ENSMUSG00000066735 | Vkorc1l1 | protein\_coding | 5:129941970-129986692 (+) |  | -0.1500 | 7.59e-04 | 3.44e-03 |
| ENSMUSG00000028641 | P3h1 | protein\_coding | 4:119232915-119248975 (+) |  | -0.3350 | 7.61e-04 | 3.45e-03 |
| ENSMUSG00000037936 | Scarb1 | protein\_coding | 5:125277087-125341094 (-) |  | -0.1530 | 7.62e-04 | 3.45e-03 |
| ENSMUSG00000020255 | D10Wsu102e | protein\_coding | 10:83360221-83488505 (+) |  | -0.1740 | 7.66e-04 | 3.47e-03 |
| ENSMUSG00000026784 | Pdss1 | protein\_coding | 2:22895522-22940266 (+) |  | -0.2240 | 7.67e-04 | 3.47e-03 |
| ENSMUSG00000026603 | Smyd2 | protein\_coding | 1:189880492-189922363 (-) |  | -0.3270 | 7.75e-04 | 3.51e-03 |
| ENSMUSG00000029647 | Pan3 | protein\_coding | 5:147430161-147548502 (+) |  | 0.2260 | 7.78e-04 | 3.52e-03 |
| ENSMUSG00000049686 | Orai1 | protein\_coding | 5:123015074-123030456 (+) |  | 0.1680 | 7.79e-04 | 3.52e-03 |
| ENSMUSG00000024165 | Jpt2 | protein\_coding | 17:24937419-24960689 (-) |  | -0.2270 | 7.82e-04 | 3.54e-03 |
| ENSMUSG00000034203 | Chchd4 | protein\_coding | 6:91462172-91473546 (-) |  | -0.3130 | 7.89e-04 | 3.57e-03 |
| ENSMUSG00000025027 | Xpnpep1 | protein\_coding | 19:52931926-53040214 (-) |  | -0.1530 | 7.95e-04 | 3.59e-03 |
| ENSMUSG00000058006 | Mdn1 | protein\_coding | 4:32657119-32775217 (+) |  | -0.3270 | 7.95e-04 | 3.59e-03 |
| ENSMUSG00000043391 | 2510009E07Rik | protein\_coding | 16:21649045-21694665 (-) |  | -0.6450 | 7.95e-04 | 3.59e-03 |
| ENSMUSG00000062488 | Ifit3b | protein\_coding | 19:34607970-34613401 (+) |  | 1.5000 | 7.95e-04 | 3.59e-03 |
| ENSMUSG00000028559 | Osbpl9 | protein\_coding | 4:109061145-109202272 (-) |  | -0.1780 | 7.96e-04 | 3.59e-03 |
| ENSMUSG00000024875 | Yif1a | protein\_coding | 19:5088538-5092881 (+) |  | -0.2360 | 7.97e-04 | 3.60e-03 |
| ENSMUSG00000029720 | Gm20605 | protein\_coding | 5:137629175-137642899 (+) |  | 0.4620 | 8.03e-04 | 3.62e-03 |
| ENSMUSG00000021973 | Micu2 | protein\_coding | 14:57916261-57999262 (-) |  | 0.1630 | 8.04e-04 | 3.63e-03 |
| ENSMUSG00000032462 | Pik3cb | protein\_coding | 9:99036654-99140621 (-) |  | -0.1760 | 8.04e-04 | 3.63e-03 |
| ENSMUSG00000049411 | Tmem241 | protein\_coding | 18:11964450-12121537 (-) |  | 0.3300 | 8.11e-04 | 3.66e-03 |
| ENSMUSG00000020018 | Snrpf | protein\_coding | 10:93583029-93589706 (-) |  | -0.2990 | 8.11e-04 | 3.66e-03 |
| ENSMUSG00000025747 | Tyms | protein\_coding | 5:30058202-30073617 (-) |  | -0.3040 | 8.13e-04 | 3.66e-03 |
| ENSMUSG00000001100 | Poldip2 | protein\_coding | 11:78512193-78522736 (+) |  | -0.1880 | 8.15e-04 | 3.67e-03 |
| ENSMUSG00000028670 | Lypla2 | protein\_coding | 4:135968224-135972626 (-) |  | 0.1400 | 8.17e-04 | 3.68e-03 |
| ENSMUSG00000001285 | Myg1 | protein\_coding | 15:102331709-102338139 (+) |  | -0.1830 | 8.18e-04 | 3.68e-03 |
| ENSMUSG00000100865 | Gm9320 | transcribed\_processed\_pseudogene | 17:70853767-70876312 (+) |  | -0.5080 | 8.20e-04 | 3.69e-03 |
| ENSMUSG00000024899 | Papss2 | protein\_coding | 19:32595790-32667187 (+) |  | -0.2840 | 8.25e-04 | 3.71e-03 |
| ENSMUSG00000029776 | Hibadh | protein\_coding | 6:52546228-52640389 (-) |  | -0.2170 | 8.25e-04 | 3.71e-03 |
| ENSMUSG00000048897 | Zfp710 | protein\_coding | 7:80024814-80094173 (+) |  | 0.1900 | 8.25e-04 | 3.71e-03 |
| ENSMUSG00000032640 | Chsy1 | protein\_coding | 7:66109515-66173798 (+) |  | -0.1920 | 8.28e-04 | 3.72e-03 |
| ENSMUSG00000024011 | Pi16 | protein\_coding | 17:29317680-29330593 (+) |  | 0.2770 | 8.36e-04 | 3.76e-03 |
| ENSMUSG00000032867 | Fbxw8 | protein\_coding | 5:118064965-118155464 (-) |  | -0.1830 | 8.38e-04 | 3.76e-03 |
| ENSMUSG00000030064 | Frmd4b | protein\_coding | 6:97286867-97617541 (-) |  | 0.3320 | 8.38e-04 | 3.76e-03 |
| ENSMUSG00000031803 | B3gnt3 | protein\_coding | 8:71690756-71701789 (-) |  | -0.8770 | 8.38e-04 | 3.76e-03 |
| ENSMUSG00000024277 | Mapre2 | protein\_coding | 18:23752333-23893861 (+) |  | 0.1950 | 8.38e-04 | 3.76e-03 |
| ENSMUSG00000066839 | Ecsit | protein\_coding | 9:22072246-22085438 (-) |  | -0.3050 | 8.41e-04 | 3.77e-03 |
| ENSMUSG00000051212 | Gpr183 | protein\_coding | 14:121952551-121965195 (-) |  | 0.2830 | 8.43e-04 | 3.78e-03 |
| ENSMUSG00000040269 | Mrps28 | protein\_coding | 3:8802146-8923918 (-) |  | -0.2600 | 8.43e-04 | 3.78e-03 |
| ENSMUSG00000020420 | Zfp607a | protein\_coding | 7:27857527-27880825 (+) |  | 0.3330 | 8.44e-04 | 3.78e-03 |
| ENSMUSG00000031387 | Renbp | protein\_coding | X:73922121-73930850 (-) |  | 0.3180 | 8.45e-04 | 3.79e-03 |
| ENSMUSG00000014554 | Dguok | protein\_coding | 6:83480217-83506969 (-) |  | 0.2840 | 8.55e-04 | 3.83e-03 |
| ENSMUSG00000055322 | Tns1 | protein\_coding | 1:73910231-74124449 (-) |  | 0.5800 | 8.57e-04 | 3.84e-03 |
| ENSMUSG00000037152 | Ndufc1 | protein\_coding | 3:51404677-51408988 (-) |  | -0.2450 | 8.60e-04 | 3.85e-03 |
| ENSMUSG00000066487 | Gm5786 | processed\_pseudogene | 12:59081019-59081900 (+) |  | -0.4170 | 8.60e-04 | 3.85e-03 |
| ENSMUSG00000073609 | D2hgdh | protein\_coding | 1:93824909-93852348 (+) |  | 0.3190 | 8.61e-04 | 3.85e-03 |
| ENSMUSG00000020415 | Pttg1 | protein\_coding | 11:43420250-43426251 (-) |  | 0.2810 | 8.63e-04 | 3.86e-03 |
| ENSMUSG00000115049 | Gm49172 | lncRNA | 15:95972739-95994129 (-) |  | 0.2610 | 8.65e-04 | 3.87e-03 |
| ENSMUSG00000016940 | Kctd2 | protein\_coding | 11:115420128-115431274 (+) |  | 0.2250 | 8.68e-04 | 3.88e-03 |
| ENSMUSG00000040711 | Sh3pxd2b | protein\_coding | 11:32347820-32428173 (+) |  | -0.3260 | 8.69e-04 | 3.88e-03 |
| ENSMUSG00000023027 | Atf1 | protein\_coding | 15:100227819-100261244 (+) |  | -0.1510 | 8.72e-04 | 3.89e-03 |
| ENSMUSG00000032330 | Cox7a2 | protein\_coding | 9:79755361-79759878 (-) |  | -0.3190 | 8.74e-04 | 3.90e-03 |
| ENSMUSG00000039449 | Prpf18 | protein\_coding | 2:4622058-4652113 (-) |  | -0.1350 | 8.76e-04 | 3.91e-03 |
| ENSMUSG00000055485 | Soga1 | protein\_coding | 2:157015799-157079254 (-) |  | 0.4280 | 8.78e-04 | 3.92e-03 |
| ENSMUSG00000019852 | Arfgef3 | protein\_coding | 10:18581839-18743949 (-) |  | -0.5710 | 8.79e-04 | 3.92e-03 |
| ENSMUSG00000037461 | Ints7 | protein\_coding | 1:191575636-191623688 (+) |  | -0.2690 | 8.81e-04 | 3.93e-03 |
| ENSMUSG00000037720 | Tmem33 | protein\_coding | 5:67260565-67291461 (+) |  | -0.2090 | 8.83e-04 | 3.94e-03 |
| ENSMUSG00000036751 | Cox6b1 | protein\_coding | 7:30616861-30626151 (-) |  | -0.1850 | 8.87e-04 | 3.95e-03 |
| ENSMUSG00000051550 | Zfp579 | protein\_coding | 7:4983483-4996158 (-) |  | 0.7080 | 8.88e-04 | 3.96e-03 |
| ENSMUSG00000021279 | Cdc42bpb | protein\_coding | 12:111292976-111377718 (-) |  | 0.3050 | 8.89e-04 | 3.96e-03 |
| ENSMUSG00000051147 | Nat2 | protein\_coding | 8:67494858-67502584 (+) |  | 0.2910 | 8.90e-04 | 3.96e-03 |
| ENSMUSG00000032633 | Flcn | protein\_coding | 11:59791408-59810016 (-) |  | 0.1970 | 8.92e-04 | 3.97e-03 |
| ENSMUSG00000016255 | Tubb1 | protein\_coding | 2:174450695-174457882 (+) |  | 1.5100 | 8.93e-04 | 3.97e-03 |
| ENSMUSG00000039206 | Daglb | protein\_coding | 5:143464584-143505942 (+) |  | 0.1780 | 8.94e-04 | 3.98e-03 |
| ENSMUSG00000060152 | Pop5 | protein\_coding | 5:115235836-115245351 (+) |  | -0.2760 | 8.97e-04 | 3.99e-03 |
| ENSMUSG00000078716 | Tmem8b | protein\_coding | 4:43668971-43692668 (+) |  | 0.9290 | 8.97e-04 | 3.99e-03 |
| ENSMUSG00000086914 | Gm16124 | lncRNA | 9:53667246-53681265 (+) |  | 1.1300 | 8.97e-04 | 3.99e-03 |
| ENSMUSG00000024668 | Sdhaf2 | protein\_coding | 19:10500534-10526142 (-) |  | -0.1570 | 8.98e-04 | 3.99e-03 |
| ENSMUSG00000034453 | Polr3b | protein\_coding | 10:84622292-84727178 (+) |  | 0.1680 | 8.99e-04 | 3.99e-03 |
| ENSMUSG00000031575 | Ash2l | protein\_coding | 8:25815996-25847694 (-) |  | -0.1400 | 9.00e-04 | 4.00e-03 |
| ENSMUSG00000048310 | Pskh1 | protein\_coding | 8:105900441-105931778 (+) |  | 0.2380 | 9.02e-04 | 4.01e-03 |
| ENSMUSG00000031696 | Vps35 | protein\_coding | 8:85260392-85299802 (-) |  | -0.2080 | 9.04e-04 | 4.01e-03 |
| ENSMUSG00000048406 | B330016D10Rik | pseudogene | 4:141546189-141547892 (+) |  | 0.5170 | 9.04e-04 | 4.01e-03 |
| ENSMUSG00000061650 | Med9 | protein\_coding | 11:59948206-59962205 (+) |  | -0.2510 | 9.06e-04 | 4.02e-03 |
| ENSMUSG00000067851 | Arfgef1 | protein\_coding | 1:10137571-10232670 (-) |  | -0.1410 | 9.08e-04 | 4.03e-03 |
| ENSMUSG00000092564 | BC051226 | lncRNA | 17:33908044-33909317 (-) |  | 0.6110 | 9.10e-04 | 4.03e-03 |
| ENSMUSG00000028869 | Gnl2 | protein\_coding | 4:125016585-125055380 (+) |  | -0.1500 | 9.11e-04 | 4.04e-03 |
| ENSMUSG00000022124 | Fbxl3 | protein\_coding | 14:103080239-103099566 (-) |  | 0.1670 | 9.14e-04 | 4.05e-03 |
| ENSMUSG00000010277 | 2610507B11Rik | protein\_coding | 11:78261752-78290623 (+) |  | -0.1640 | 9.16e-04 | 4.06e-03 |
| ENSMUSG00000033434 | Gtpbp6 | protein\_coding | 5:110103975-110108197 (-) |  | 0.2900 | 9.17e-04 | 4.06e-03 |
| ENSMUSG00000031312 | Itgb1bp2 | protein\_coding | X:101449088-101453541 (+) |  | -1.1600 | 9.19e-04 | 4.07e-03 |
| ENSMUSG00000058258 | Idi1 | protein\_coding | 13:8885501-8892451 (+) |  | -0.3320 | 9.20e-04 | 4.07e-03 |
| ENSMUSG00000022437 | Samm50 | protein\_coding | 15:84192241-84217267 (+) |  | -0.1780 | 9.20e-04 | 4.07e-03 |
| ENSMUSG00000013698 | Pea15a | protein\_coding | 1:172196728-172206804 (-) |  | 0.2510 | 9.20e-04 | 4.07e-03 |
| ENSMUSG00000043795 | Prr33 | protein\_coding | 7:142491074-142506771 (-) |  | 0.6040 | 9.22e-04 | 4.07e-03 |
| ENSMUSG00000031783 | Polr2c | protein\_coding | 8:94857450-94873535 (+) |  | -0.2370 | 9.22e-04 | 4.08e-03 |
| ENSMUSG00000024597 | Slc12a2 | protein\_coding | 18:57878678-57946821 (+) |  | 0.5300 | 9.24e-04 | 4.08e-03 |
| ENSMUSG00000053617 | Sh3pxd2a | protein\_coding | 19:47260174-47464401 (-) |  | 0.4430 | 9.24e-04 | 4.08e-03 |
| ENSMUSG00000018965 | Ywhah | protein\_coding | 5:33018816-33027966 (+) |  | -0.1570 | 9.27e-04 | 4.09e-03 |
| ENSMUSG00000080727 | C920021L13Rik | lncRNA | 3:95871522-95889093 (+) |  | 0.6010 | 9.27e-04 | 4.09e-03 |
| ENSMUSG00000101567 | Txn-ps1 | processed\_pseudogene | 1:44463923-44464231 (-) |  | 1.8400 | 9.28e-04 | 4.10e-03 |
| ENSMUSG00000038623 | Tm6sf1 | protein\_coding | 7:81859001-81884434 (+) |  | 0.1940 | 9.33e-04 | 4.12e-03 |
| ENSMUSG00000080877 | Rpl22-ps1 | processed\_pseudogene | X:8038611-8038996 (-) |  | 0.6860 | 9.37e-04 | 4.13e-03 |
| ENSMUSG00000031840 | Rab3a | protein\_coding | 8:70754679-70758677 (+) |  | 0.4200 | 9.45e-04 | 4.17e-03 |
| ENSMUSG00000058756 | Thra | protein\_coding | 11:98740638-98769006 (+) |  | 0.2650 | 9.46e-04 | 4.17e-03 |
| ENSMUSG00000031754 | Nudt21 | protein\_coding | 8:94015496-94037031 (-) |  | -0.1930 | 9.49e-04 | 4.18e-03 |
| ENSMUSG00000081113 | Gm7308 | processed\_pseudogene | 6:127282063-127282509 (-) |  | 0.8440 | 9.52e-04 | 4.19e-03 |
| ENSMUSG00000101939 | Gm28438 | unprocessed\_pseudogene | 1:24612775-24613119 (-) |  | -0.5840 | 9.54e-04 | 4.20e-03 |
| ENSMUSG00000022906 | Parp9 | protein\_coding | 16:35938470-35972605 (+) |  | -0.2340 | 9.63e-04 | 4.24e-03 |
| ENSMUSG00000072980 | Oip5 | protein\_coding | 2:119609512-119618469 (-) |  | -0.2830 | 9.63e-04 | 4.24e-03 |
| ENSMUSG00000085917 | Gm15899 | processed\_pseudogene | 8:122683602-122683839 (-) |  | 0.3200 | 9.66e-04 | 4.25e-03 |
| ENSMUSG00000111118 | Gm6545 | processed\_pseudogene | 19:12528755-12530540 (-) |  | 0.5800 | 9.66e-04 | 4.25e-03 |
| ENSMUSG00000031101 | Sash3 | protein\_coding | X:48146436-48161565 (+) |  | 0.1410 | 9.67e-04 | 4.25e-03 |
| ENSMUSG00000031901 | Dus2 | protein\_coding | 8:105991337-106053840 (+) |  | -0.3510 | 9.71e-04 | 4.27e-03 |
| ENSMUSG00000024012 | Mtch1 | protein\_coding | 17:29332072-29347934 (-) |  | -0.1540 | 9.72e-04 | 4.27e-03 |
| ENSMUSG00000026842 | Abl1 | protein\_coding | 2:31688376-31804227 (+) |  | 0.1630 | 9.72e-04 | 4.27e-03 |
| ENSMUSG00000026096 | Osgepl1 | protein\_coding | 1:53313624-53326343 (+) |  | -0.3870 | 9.74e-04 | 4.28e-03 |
| ENSMUSG00000065126 | Snord104 | snoRNA | 11:106500993-106501062 (+) |  | 0.5040 | 9.76e-04 | 4.28e-03 |
| ENSMUSG00000040268 | Plekha1 | protein\_coding | 7:130865756-130913312 (+) |  | 0.2180 | 9.76e-04 | 4.28e-03 |
| ENSMUSG00000025591 | Tma16 | protein\_coding | 8:66473118-66486530 (-) |  | -0.2880 | 9.77e-04 | 4.29e-03 |
| ENSMUSG00000117621 | Hspe1-rs1 | protein\_coding | 18:47112139-47112653 (+) |  | -0.4410 | 9.77e-04 | 4.29e-03 |
| ENSMUSG00000056602 | Fry | protein\_coding | 5:150118645-150497753 (+) |  | 0.3040 | 9.78e-04 | 4.29e-03 |
| ENSMUSG00000021877 | Arf4 | protein\_coding | 14:26638074-26665084 (+) |  | -0.1460 | 9.78e-04 | 4.29e-03 |
| ENSMUSG00000052397 | Ezr | protein\_coding | 17:6738041-6782784 (-) |  | -0.1750 | 9.84e-04 | 4.31e-03 |
| ENSMUSG00000114253 | Gm47798 | lncRNA | 14:16304511-16308325 (+) |  | 0.6330 | 9.89e-04 | 4.33e-03 |
| ENSMUSG00000007670 | Khsrp | protein\_coding | 17:57021051-57031522 (-) |  | -0.2360 | 9.90e-04 | 4.34e-03 |
| ENSMUSG00000039450 | Dcxr | protein\_coding | 11:120725399-120727281 (-) |  | 0.2950 | 9.91e-04 | 4.34e-03 |
| ENSMUSG00000030878 | Cdr2 | protein\_coding | 7:120957036-120982312 (-) |  | -0.5700 | 9.91e-04 | 4.34e-03 |
| ENSMUSG00000025140 | Pycr1 | protein\_coding | 11:120635712-120643769 (-) |  | 0.4010 | 9.92e-04 | 4.34e-03 |
| ENSMUSG00000078816 | Prkcg | protein\_coding | 7:3289179-3331099 (+) |  | 0.6930 | 9.94e-04 | 4.35e-03 |
| ENSMUSG00000031303 | Map3k15 | protein\_coding | X:159988433-160123351 (+) |  | 0.3170 | 9.97e-04 | 4.36e-03 |
| ENSMUSG00000034007 | Scaper | protein\_coding | 9:55549879-55938119 (-) |  | 0.3590 | 9.97e-04 | 4.36e-03 |
| ENSMUSG00000014504 | Srp19 | protein\_coding | 18:34330847-34336599 (+) |  | -0.2320 | 9.99e-04 | 4.36e-03 |
| ENSMUSG00000061414 | Cracr2a | protein\_coding | 6:127561338-127674248 (+) |  | 0.3070 | 1.00e-03 | 4.37e-03 |
| ENSMUSG00000075269 | Bex6 | protein\_coding | 16:32179823-32186972 (+) |  | 0.2850 | 1.00e-03 | 4.38e-03 |
| ENSMUSG00000074807 | Gm10762 | lncRNA | 2:128965505-128968044 (-) |  | 0.4700 | 1.01e-03 | 4.42e-03 |
| ENSMUSG00000043964 | Orai3 | protein\_coding | 7:127769815-127775150 (+) |  | 0.2490 | 1.01e-03 | 4.42e-03 |
| ENSMUSG00000074748 | Atxn7l3b | protein\_coding | 10:112925430-112929001 (-) |  | 0.1290 | 1.02e-03 | 4.43e-03 |
| ENSMUSG00000085129 | 5031425F14Rik | lncRNA | 2:166447451-166459232 (+) |  | 0.8680 | 1.02e-03 | 4.43e-03 |
| ENSMUSG00000005625 | Psmd4 | protein\_coding | 3:95032694-95042614 (-) |  | -0.1510 | 1.02e-03 | 4.45e-03 |
| ENSMUSG00000083061 | Gm12191 | processed\_pseudogene | 11:49678710-49679057 (-) |  | 0.3670 | 1.02e-03 | 4.45e-03 |
| ENSMUSG00000068742 | Cry2 | protein\_coding | 2:92403646-92434043 (-) |  | 0.2950 | 1.02e-03 | 4.45e-03 |
| ENSMUSG00000091086 | Rpl6l | processed\_pseudogene | 10:111125851-111126721 (+) |  | -0.2610 | 1.02e-03 | 4.45e-03 |
| ENSMUSG00000034987 | Hrh2 | protein\_coding | 13:54192129-54236180 (+) |  | -0.4300 | 1.03e-03 | 4.48e-03 |
| ENSMUSG00000038047 | Haus6 | protein\_coding | 4:86578855-86612055 (-) |  | -0.2410 | 1.03e-03 | 4.48e-03 |
| ENSMUSG00000041115 | Iqsec2 | protein\_coding | X:152144268-152225236 (+) |  | 0.2860 | 1.04e-03 | 4.51e-03 |
| ENSMUSG00000015217 | Hmgb3 | protein\_coding | X:71555918-71560676 (+) |  | -0.2110 | 1.04e-03 | 4.54e-03 |
| ENSMUSG00000100017 | 2410022M11Rik | lncRNA | 14:56811989-56813899 (+) |  | 0.5600 | 1.05e-03 | 4.55e-03 |
| ENSMUSG00000057130 | Txnl4a | protein\_coding | 18:80206795-80225855 (+) |  | -0.2550 | 1.05e-03 | 4.55e-03 |
| ENSMUSG00000030346 | Rad51ap1 | protein\_coding | 6:126923050-126939587 (-) |  | -0.2550 | 1.05e-03 | 4.55e-03 |
| ENSMUSG00000024782 | Ak3 | protein\_coding | 19:29020833-29047961 (-) |  | 0.2280 | 1.05e-03 | 4.56e-03 |
| ENSMUSG00000031007 | Atp6ap2 | protein\_coding | X:12587801-12617049 (+) |  | -0.1540 | 1.05e-03 | 4.56e-03 |
| ENSMUSG00000063849 | Ppcdc | protein\_coding | 9:57385095-57440124 (-) |  | 0.2590 | 1.05e-03 | 4.56e-03 |
| ENSMUSG00000035390 | Brsk1 | protein\_coding | 7:4690604-4715997 (+) |  | 0.4950 | 1.05e-03 | 4.57e-03 |
| ENSMUSG00000019471 | Cdc37 | protein\_coding | 9:21133222-21149982 (-) |  | -0.1430 | 1.05e-03 | 4.57e-03 |
| ENSMUSG00000034729 | Mrps10 | protein\_coding | 17:47368887-47381417 (+) |  | -0.2390 | 1.05e-03 | 4.57e-03 |
| ENSMUSG00000052056 | Zfp217 | protein\_coding | 2:170108643-170148103 (-) |  | 0.1540 | 1.05e-03 | 4.57e-03 |
| ENSMUSG00000027189 | Trim44 | protein\_coding | 2:102300119-102407828 (-) |  | 0.1610 | 1.06e-03 | 4.59e-03 |
| ENSMUSG00000026683 | Nuf2 | protein\_coding | 1:169497934-169531464 (-) |  | -0.1770 | 1.06e-03 | 4.59e-03 |
| ENSMUSG00000031532 | Saraf | protein\_coding | 8:34154575-34170821 (+) |  | 0.1510 | 1.06e-03 | 4.60e-03 |
| ENSMUSG00000032643 | Fhl3 | protein\_coding | 4:124700701-124708611 (+) |  | -0.4530 | 1.06e-03 | 4.60e-03 |
| ENSMUSG00000001910 | Nacc1 | protein\_coding | 8:84670479-84687902 (-) |  | -0.1760 | 1.06e-03 | 4.60e-03 |
| ENSMUSG00000114055 | Gm32089 | lncRNA | 13:94289448-94295006 (-) |  | -1.1900 | 1.07e-03 | 4.63e-03 |
| ENSMUSG00000030750 | Nsmce1 | protein\_coding | 7:125467640-125491596 (-) |  | -0.2650 | 1.07e-03 | 4.63e-03 |
| ENSMUSG00000037971 | 1110032A03Rik | protein\_coding | 9:50762828-50775520 (-) |  | 0.3190 | 1.07e-03 | 4.63e-03 |
| ENSMUSG00000024300 | Myo1f | protein\_coding | 17:33555707-33607764 (+) |  | 0.1740 | 1.08e-03 | 4.65e-03 |
| ENSMUSG00000006288 | Ttc5 | protein\_coding | 14:50765415-50785519 (-) |  | 0.1560 | 1.08e-03 | 4.68e-03 |
| ENSMUSG00000074039 | 4930520O04Rik | lncRNA | 9:114368303-114377229 (+) |  | 0.6050 | 1.08e-03 | 4.69e-03 |
| ENSMUSG00000059475 | Zfp426 | protein\_coding | 9:20468549-20492746 (-) |  | 0.2050 | 1.09e-03 | 4.70e-03 |
| ENSMUSG00000079962 | Gm12643 | processed\_pseudogene | 4:91037900-91038273 (+) |  | 0.8250 | 1.09e-03 | 4.70e-03 |
| ENSMUSG00000031924 | Cyb5b | protein\_coding | 8:107150640-107187471 (+) |  | -0.1850 | 1.09e-03 | 4.70e-03 |
| ENSMUSG00000021684 | Pde8b | protein\_coding | 13:95024454-95250336 (-) |  | 0.6750 | 1.09e-03 | 4.70e-03 |
| ENSMUSG00000021096 | Ppm1a | protein\_coding | 12:72757455-72799819 (+) |  | 0.1610 | 1.09e-03 | 4.71e-03 |
| ENSMUSG00000094872 | Igkv9-120 | IG\_V\_gene | 6:68049983-68050456 (+) |  | 5.5600 | 1.09e-03 | 4.71e-03 |
| ENSMUSG00000022280 | Rnf19a | protein\_coding | 15:36239933-36283147 (-) |  | 0.2230 | 1.09e-03 | 4.71e-03 |
| ENSMUSG00000032977 | Fam207a | protein\_coding | 10:77486661-77515785 (-) |  | -0.1700 | 1.09e-03 | 4.71e-03 |
| ENSMUSG00000035561 | Aldh1b1 | protein\_coding | 4:45799022-45804604 (+) |  | -0.2070 | 1.09e-03 | 4.72e-03 |
| ENSMUSG00000028800 | Hdac1 | protein\_coding | 4:129516104-129542713 (-) |  | -0.1280 | 1.09e-03 | 4.72e-03 |
| ENSMUSG00000059456 | Ptk2b | protein\_coding | 14:66153257-66281052 (-) |  | 0.1700 | 1.10e-03 | 4.73e-03 |
| ENSMUSG00000027313 | Chac1 | protein\_coding | 2:119351229-119354381 (+) |  | -1.1600 | 1.10e-03 | 4.73e-03 |
| ENSMUSG00000019505 | Ubb | protein\_coding | 11:62551171-62553213 (+) |  | 0.2370 | 1.10e-03 | 4.74e-03 |
| ENSMUSG00000024960 | Plcb3 | protein\_coding | 19:6952325-6976470 (-) |  | -0.1860 | 1.10e-03 | 4.74e-03 |
| ENSMUSG00000027167 | Elp4 | protein\_coding | 2:105701027-105904564 (-) |  | -0.3780 | 1.10e-03 | 4.75e-03 |
| ENSMUSG00000055553 | Kxd1 | protein\_coding | 8:70508272-70527956 (-) |  | -0.1670 | 1.10e-03 | 4.75e-03 |
| ENSMUSG00000049739 | Zfp646 | protein\_coding | 7:127876221-127885996 (+) |  | 0.1860 | 1.10e-03 | 4.75e-03 |
| ENSMUSG00000029312 | Klhl8 | protein\_coding | 5:103861973-103911259 (-) |  | 0.7280 | 1.11e-03 | 4.76e-03 |
| ENSMUSG00000031848 | Lsm4 | protein\_coding | 8:70673248-70678752 (+) |  | -0.2170 | 1.11e-03 | 4.76e-03 |
| ENSMUSG00000021391 | Cenpp | protein\_coding | 13:49464023-49652785 (-) |  | -0.4250 | 1.11e-03 | 4.77e-03 |
| ENSMUSG00000024818 | Slc25a45 | protein\_coding | 19:5877808-5885878 (+) |  | 0.2000 | 1.11e-03 | 4.77e-03 |
| ENSMUSG00000024491 | Rbm27 | protein\_coding | 18:42275353-42341542 (+) |  | -0.1390 | 1.11e-03 | 4.78e-03 |
| ENSMUSG00000052298 | Cdc42se2 | protein\_coding | 11:54717456-54787675 (-) |  | 0.1390 | 1.11e-03 | 4.78e-03 |
| ENSMUSG00000035621 | Midn | protein\_coding | 10:80148272-80158368 (+) |  | -0.1920 | 1.11e-03 | 4.79e-03 |
| ENSMUSG00000047669 | Msl3l2 | protein\_coding | 10:56106917-56116880 (+) |  | 0.3770 | 1.12e-03 | 4.79e-03 |
| ENSMUSG00000029004 | Kmt2e | protein\_coding | 5:23434441-23504235 (+) |  | 0.1900 | 1.12e-03 | 4.79e-03 |
| ENSMUSG00000020493 | Prr11 | protein\_coding | 11:87089153-87108708 (-) |  | -0.1970 | 1.12e-03 | 4.80e-03 |
| ENSMUSG00000030315 | Vgll4 | protein\_coding | 6:114860628-114969994 (-) |  | 0.2350 | 1.12e-03 | 4.80e-03 |
| ENSMUSG00000060470 | Adgrg3 | protein\_coding | 8:95017692-95045250 (+) |  | 0.4130 | 1.12e-03 | 4.80e-03 |
| ENSMUSG00000020386 | Sar1b | protein\_coding | 11:51763687-51791925 (+) |  | -0.2600 | 1.12e-03 | 4.81e-03 |
| ENSMUSG00000005299 | Letm1 | protein\_coding | 5:33739673-33782817 (-) |  | -0.1670 | 1.12e-03 | 4.82e-03 |
| ENSMUSG00000039007 | Cpq | protein\_coding | 15:33083129-33594552 (+) |  | 0.2990 | 1.13e-03 | 4.82e-03 |
| ENSMUSG00000032215 | Rsl24d1 | protein\_coding | 9:73113426-73123333 (+) |  | -0.1710 | 1.13e-03 | 4.82e-03 |
| ENSMUSG00000032097 | Ddx6 | protein\_coding | 9:44604892-44640731 (+) |  | -0.1460 | 1.13e-03 | 4.82e-03 |
| ENSMUSG00000021218 | Gdi2 | protein\_coding | 13:3538063-3567871 (+) |  | -0.1390 | 1.13e-03 | 4.83e-03 |
| ENSMUSG00000039275 | Foxk2 | protein\_coding | 11:121259990-121309896 (+) |  | -0.1490 | 1.13e-03 | 4.83e-03 |
| ENSMUSG00000028931 | Kcnab2 | protein\_coding | 4:152390742-152477910 (-) |  | -0.1300 | 1.13e-03 | 4.83e-03 |
| ENSMUSG00000018040 | Rrp7a | protein\_coding | 15:83113433-83122801 (-) |  | -0.2000 | 1.14e-03 | 4.85e-03 |
| ENSMUSG00000035376 | Hacd2 | protein\_coding | 16:35022428-35109177 (+) |  | -0.2830 | 1.14e-03 | 4.88e-03 |
| ENSMUSG00000027088 | Phospho2 | protein\_coding | 2:69789623-69800005 (+) |  | 0.2520 | 1.15e-03 | 4.90e-03 |
| ENSMUSG00000018169 | Mfng | protein\_coding | 15:78755882-78773475 (-) |  | 0.4310 | 1.15e-03 | 4.90e-03 |
| ENSMUSG00000055239 | Kcmf1 | protein\_coding | 6:72841114-72899979 (-) |  | -0.1560 | 1.15e-03 | 4.91e-03 |
| ENSMUSG00000078452 | Raet1d | protein\_coding | 10:22360552-22374139 (+) |  | -1.9900 | 1.15e-03 | 4.92e-03 |
| ENSMUSG00000090247 | Bloc1s1 | protein\_coding | 10:128917882-128924035 (-) |  | 0.3450 | 1.15e-03 | 4.92e-03 |
| ENSMUSG00000031388 | Naa10 | protein\_coding | X:73916873-73921944 (-) |  | -0.2290 | 1.16e-03 | 4.93e-03 |
| ENSMUSG00000079562 | Maea | protein\_coding | 5:33335509-33373296 (+) |  | -0.1530 | 1.16e-03 | 4.93e-03 |
| ENSMUSG00000020687 | Cdc27 | protein\_coding | 11:104502745-104550620 (-) |  | -0.1990 | 1.16e-03 | 4.93e-03 |
| ENSMUSG00000025486 | Sirt3 | protein\_coding | 7:140863666-140882309 (-) |  | 0.2570 | 1.16e-03 | 4.94e-03 |
| ENSMUSG00000099871 | Gm21742 | unprocessed\_pseudogene | Y:90837413-90844040 (+) |  | -0.8550 | 1.16e-03 | 4.94e-03 |
| ENSMUSG00000079641 | Rpl39 | protein\_coding | X:37082520-37085402 (-) |  | 0.3260 | 1.16e-03 | 4.94e-03 |
| ENSMUSG00000027194 | Ttc17 | protein\_coding | 2:94300767-94406689 (-) |  | 0.1910 | 1.16e-03 | 4.94e-03 |
| ENSMUSG00000101585 | 1600010M07Rik | lncRNA | 7:109998376-110151202 (-) |  | 0.6440 | 1.16e-03 | 4.94e-03 |
| ENSMUSG00000073775 | Kti12 | protein\_coding | 4:108847785-108849413 (+) |  | -0.1860 | 1.16e-03 | 4.96e-03 |
| ENSMUSG00000039629 | Strip2 | protein\_coding | 6:29917012-29959681 (+) |  | -0.6400 | 1.16e-03 | 4.96e-03 |
| ENSMUSG00000025138 | Sirt7 | protein\_coding | 11:120618372-120625240 (-) |  | 0.1400 | 1.17e-03 | 4.96e-03 |
| ENSMUSG00000024907 | Gal | protein\_coding | 19:3409915-3414544 (-) |  | 2.0100 | 1.17e-03 | 4.97e-03 |
| ENSMUSG00000062028 | Irgc1 | protein\_coding | 7:24431922-24452297 (-) |  | 0.8190 | 1.17e-03 | 4.98e-03 |
| ENSMUSG00000048307 | Ankrd46 | protein\_coding | 15:36477668-36496820 (-) |  | 0.1760 | 1.17e-03 | 4.98e-03 |
| ENSMUSG00000073422 | H2-Ke6 | protein\_coding | 17:34026033-34028060 (-) |  | 0.8010 | 1.18e-03 | 4.99e-03 |
| ENSMUSG00000055493 | Epm2a | protein\_coding | 10:11343404-11459644 (+) |  | 0.6660 | 1.18e-03 | 4.99e-03 |
| ENSMUSG00000087004 | Gm14154 | lncRNA | 2:151947273-151950641 (-) |  | 1.7700 | 1.18e-03 | 5.02e-03 |
| ENSMUSG00000015837 | Sqstm1 | protein\_coding | 11:50199366-50210827 (-) |  | 0.1250 | 1.18e-03 | 5.02e-03 |
| ENSMUSG00000086695 | Gm15247 | lncRNA | X:169985801-169986939 (-) |  | -0.7710 | 1.19e-03 | 5.04e-03 |
| ENSMUSG00000028960 | Ube4b | protein\_coding | 4:149328416-149426749 (-) |  | -0.2230 | 1.19e-03 | 5.05e-03 |
| ENSMUSG00000035298 | Klhl35 | protein\_coding | 7:99466004-99474022 (+) |  | 0.9290 | 1.19e-03 | 5.05e-03 |
| ENSMUSG00000027506 | Tpd52 | protein\_coding | 3:8925593-9004723 (-) |  | -0.2130 | 1.19e-03 | 5.05e-03 |
| ENSMUSG00000072235 | Tuba1a | protein\_coding | 15:98949837-98953703 (-) |  | -0.3520 | 1.19e-03 | 5.06e-03 |
| ENSMUSG00000020289 | Nprl3 | protein\_coding | 11:32225628-32267707 (-) |  | 0.3000 | 1.20e-03 | 5.07e-03 |
| ENSMUSG00000036106 | Prr5 | protein\_coding | 15:84669620-84703673 (+) |  | 0.3460 | 1.20e-03 | 5.08e-03 |
| ENSMUSG00000020362 | Cnot6 | protein\_coding | 11:49671503-49712723 (-) |  | -0.1530 | 1.20e-03 | 5.10e-03 |
| ENSMUSG00000022559 | Fbxl6 | protein\_coding | 15:76535721-76538746 (-) |  | 0.2040 | 1.21e-03 | 5.14e-03 |
| ENSMUSG00000027357 | Crls1 | protein\_coding | 2:132846666-132866785 (+) |  | -0.3120 | 1.21e-03 | 5.14e-03 |
| ENSMUSG00000015202 | Cnksr3 | protein\_coding | 10:7119063-7212237 (-) |  | 0.2640 | 1.22e-03 | 5.17e-03 |
| ENSMUSG00000001419 | Mef2d | protein\_coding | 3:88142372-88172086 (+) |  | 0.2510 | 1.22e-03 | 5.17e-03 |
| ENSMUSG00000049422 | Chchd10 | protein\_coding | 10:75933130-75937747 (+) |  | -0.5170 | 1.22e-03 | 5.17e-03 |
| ENSMUSG00000024101 | Washc1 | protein\_coding | 17:66111545-66120503 (+) |  | 0.1570 | 1.22e-03 | 5.18e-03 |
| ENSMUSG00000038806 | Sde2 | protein\_coding | 1:180851127-180868113 (+) |  | -0.1570 | 1.23e-03 | 5.18e-03 |
| ENSMUSG00000043004 | Gng2 | protein\_coding | 14:19872559-19977627 (-) |  | 0.1690 | 1.23e-03 | 5.18e-03 |
| ENSMUSG00000038236 | Hoxa7 | protein\_coding | 6:52214491-52221854 (-) |  | -0.8220 | 1.23e-03 | 5.19e-03 |
| ENSMUSG00000036986 | Pml | protein\_coding | 9:58218076-58249786 (-) |  | 0.2390 | 1.23e-03 | 5.19e-03 |
| ENSMUSG00000024164 | C3 | protein\_coding | 17:57203970-57228136 (-) |  | 0.2510 | 1.23e-03 | 5.19e-03 |
| ENSMUSG00000001524 | Gtf2h4 | protein\_coding | 17:35667730-35673739 (-) |  | -0.2390 | 1.23e-03 | 5.20e-03 |
| ENSMUSG00000060904 | Arl1 | protein\_coding | 10:88730858-88744094 (+) |  | -0.2320 | 1.23e-03 | 5.21e-03 |
| ENSMUSG00000078435 | AU041133 | protein\_coding | 10:82128013-82153065 (+) |  | 0.5210 | 1.23e-03 | 5.21e-03 |
| ENSMUSG00000060802 | B2m | protein\_coding | 2:122147686-122153083 (+) |  | -0.2160 | 1.24e-03 | 5.24e-03 |
| ENSMUSG00000031660 | Brd7 | protein\_coding | 8:88331039-88362194 (-) |  | -0.1420 | 1.24e-03 | 5.24e-03 |
| ENSMUSG00000024069 | Slc30a6 | protein\_coding | 17:74395608-74424221 (+) |  | -0.2560 | 1.25e-03 | 5.26e-03 |
| ENSMUSG00000019864 | Rtn4ip1 | protein\_coding | 10:43901807-43957201 (+) |  | -0.2390 | 1.25e-03 | 5.26e-03 |
| ENSMUSG00000060862 | Zbtb40 | protein\_coding | 4:136979732-137048801 (-) |  | -0.2240 | 1.25e-03 | 5.27e-03 |
| ENSMUSG00000000127 | Fer | protein\_coding | 17:63863062-64139496 (+) |  | 0.8430 | 1.25e-03 | 5.28e-03 |
| ENSMUSG00000020650 | Bcap29 | protein\_coding | 12:31590967-31634658 (-) |  | -0.2460 | 1.26e-03 | 5.29e-03 |
| ENSMUSG00000048796 | Cyb561d1 | protein\_coding | 3:108195687-108201212 (-) |  | 0.2400 | 1.26e-03 | 5.29e-03 |
| ENSMUSG00000054203 | Ifi205 | protein\_coding | 1:174011998-174031810 (-) |  | 0.7480 | 1.26e-03 | 5.29e-03 |
| ENSMUSG00000033904 | Ccp110 | protein\_coding | 7:118712552-118737024 (+) |  | -0.2200 | 1.26e-03 | 5.31e-03 |
| ENSMUSG00000059474 | Mbtd1 | protein\_coding | 11:93885852-93946985 (+) |  | 0.1350 | 1.26e-03 | 5.32e-03 |
| ENSMUSG00000019857 | Asf1a | protein\_coding | 10:53596757-53609225 (+) |  | -0.3210 | 1.27e-03 | 5.32e-03 |
| ENSMUSG00000032834 | Pwp2 | protein\_coding | 10:78170909-78185149 (-) |  | -0.2350 | 1.27e-03 | 5.32e-03 |
| ENSMUSG00000020100 | Slc29a3 | protein\_coding | 10:60712072-60752794 (-) |  | 0.2110 | 1.27e-03 | 5.32e-03 |
| ENSMUSG00000033721 | Vav3 | protein\_coding | 3:109340653-109685698 (+) |  | 0.1490 | 1.27e-03 | 5.34e-03 |
| ENSMUSG00000031642 | Sh3rf1 | protein\_coding | 8:61223872-61396071 (+) |  | 0.3990 | 1.27e-03 | 5.34e-03 |
| ENSMUSG00000081400 | Gm13680 | processed\_pseudogene | 2:83353157-83353949 (-) |  | 0.2700 | 1.28e-03 | 5.35e-03 |
| ENSMUSG00000022971 | Ifnar2 | protein\_coding | 16:91372783-91405589 (+) |  | 0.1710 | 1.28e-03 | 5.36e-03 |
| ENSMUSG00000113536 | Gm49327 | transcribed\_unprocessed\_pseudogene | 12:17933164-17939628 (+) |  | 0.9560 | 1.28e-03 | 5.36e-03 |
| ENSMUSG00000014633 | Cmc2 | protein\_coding | 8:116888685-116921455 (-) |  | -0.2630 | 1.28e-03 | 5.36e-03 |
| ENSMUSG00000027582 | Zgpat | protein\_coding | 2:181364928-181383628 (+) |  | 0.1870 | 1.28e-03 | 5.37e-03 |
| ENSMUSG00000089911 | Mfsd14a | protein\_coding | 3:116631164-116662677 (-) |  | 0.1440 | 1.29e-03 | 5.39e-03 |
| ENSMUSG00000031537 | Ikbkb | protein\_coding | 8:22659212-22706589 (-) |  | 0.1920 | 1.29e-03 | 5.39e-03 |
| ENSMUSG00000097141 | Gm10524 | TEC | 18:82692284-82694176 (+) |  | 0.4260 | 1.30e-03 | 5.43e-03 |
| ENSMUSG00000021870 | Slmap | protein\_coding | 14:26413168-26534931 (-) |  | -0.1220 | 1.30e-03 | 5.43e-03 |
| ENSMUSG00000083626 | Gm4459 | processed\_pseudogene | 7:139659688-139660155 (-) |  | -0.4240 | 1.30e-03 | 5.44e-03 |
| ENSMUSG00000036053 | Fmnl2 | protein\_coding | 2:52857860-53133804 (+) |  | 0.6890 | 1.30e-03 | 5.44e-03 |
| ENSMUSG00000026335 | Pam | protein\_coding | 1:97795114-98095646 (-) |  | -0.3460 | 1.30e-03 | 5.46e-03 |
| ENSMUSG00000022570 | Tsta3 | protein\_coding | 15:75924676-75929832 (-) |  | -0.2340 | 1.31e-03 | 5.46e-03 |
| ENSMUSG00000102418 | Sh2d1b1 | protein\_coding | 1:170277320-170286769 (+) |  | 0.3100 | 1.31e-03 | 5.46e-03 |
| ENSMUSG00000026430 | Rassf5 | protein\_coding | 1:131176410-131245258 (-) |  | 0.1670 | 1.31e-03 | 5.48e-03 |
| ENSMUSG00000094441 | Zfp955a | protein\_coding | 17:33239718-33255145 (-) |  | 0.2570 | 1.34e-03 | 5.60e-03 |
| ENSMUSG00000053768 | Chchd3 | protein\_coding | 6:32790976-33060260 (-) |  | -0.2300 | 1.34e-03 | 5.61e-03 |
| ENSMUSG00000026915 | Strbp | protein\_coding | 2:37483228-37703859 (-) |  | -0.1950 | 1.34e-03 | 5.61e-03 |
| ENSMUSG00000043644 | 0610009L18Rik | lncRNA | 11:120348678-120351190 (+) |  | 1.1600 | 1.35e-03 | 5.62e-03 |
| ENSMUSG00000046822 | Slc39a3 | protein\_coding | 10:81028538-81037426 (-) |  | 0.2020 | 1.35e-03 | 5.62e-03 |
| ENSMUSG00000085887 | Arhgap27os3 | lncRNA | 11:103344753-103350124 (+) |  | -0.6880 | 1.35e-03 | 5.64e-03 |
| ENSMUSG00000031161 | Hdac6 | protein\_coding | X:7930120-7947889 (-) |  | -0.2310 | 1.35e-03 | 5.65e-03 |
| ENSMUSG00000012117 | Dhdds | protein\_coding | 4:133969028-134000918 (-) |  | -0.1480 | 1.36e-03 | 5.66e-03 |
| ENSMUSG00000055612 | Cdca7 | protein\_coding | 2:72476159-72486893 (+) |  | -0.2590 | 1.36e-03 | 5.67e-03 |
| ENSMUSG00000009406 | Elk1 | protein\_coding | X:20933395-20950608 (-) |  | 0.2290 | 1.36e-03 | 5.69e-03 |
| ENSMUSG00000044148 | 1810030O07Rik | protein\_coding | X:12654879-12673668 (-) |  | -0.1290 | 1.36e-03 | 5.69e-03 |
| ENSMUSG00000035517 | Tdrd7 | protein\_coding | 4:45965334-46034761 (+) |  | 0.1930 | 1.37e-03 | 5.69e-03 |
| ENSMUSG00000062758 | Gm16477 | processed\_pseudogene | 7:131614188-131614988 (-) |  | -0.3040 | 1.37e-03 | 5.69e-03 |
| ENSMUSG00000060063 | Alox5ap | protein\_coding | 5:149264767-149288153 (+) |  | 0.1760 | 1.37e-03 | 5.69e-03 |
| ENSMUSG00000029094 | Afap1 | protein\_coding | 5:35893319-36003923 (+) |  | 0.4450 | 1.37e-03 | 5.70e-03 |
| ENSMUSG00000035683 | Melk | protein\_coding | 4:44300876-44364675 (+) |  | -0.1910 | 1.37e-03 | 5.70e-03 |
| ENSMUSG00000046721 | Rpl14-ps1 | processed\_pseudogene | 7:45324965-45325617 (+) |  | -0.2020 | 1.37e-03 | 5.70e-03 |
| ENSMUSG00000059852 | Kcng2 | protein\_coding | 18:80294546-80364254 (-) |  | 0.9590 | 1.37e-03 | 5.70e-03 |
| ENSMUSG00000019373 | Cops3 | protein\_coding | 11:59817795-59839838 (-) |  | -0.2090 | 1.38e-03 | 5.73e-03 |
| ENSMUSG00000039831 | Arhgap29 | protein\_coding | 3:121952541-122016753 (+) |  | 1.3200 | 1.38e-03 | 5.73e-03 |
| ENSMUSG00000025743 | Sdc3 | protein\_coding | 4:130792537-130826319 (+) |  | 0.3720 | 1.38e-03 | 5.73e-03 |
| ENSMUSG00000020534 | Shmt1 | protein\_coding | 11:60788104-60811718 (-) |  | -0.2750 | 1.38e-03 | 5.75e-03 |
| ENSMUSG00000033538 | Casp4 | protein\_coding | 9:5308828-5336783 (+) |  | -0.4400 | 1.39e-03 | 5.76e-03 |
| ENSMUSG00000114784 | Gm47754 | lncRNA | 13:37581077-37589313 (+) |  | 0.6120 | 1.39e-03 | 5.76e-03 |
| ENSMUSG00000026566 | Mpzl1 | protein\_coding | 1:165592240-165634538 (-) |  | -0.8060 | 1.39e-03 | 5.76e-03 |
| ENSMUSG00000030521 | Mphosph10 | protein\_coding | 7:64376527-64392268 (-) |  | -0.2570 | 1.39e-03 | 5.76e-03 |
| ENSMUSG00000097180 | 2700038G22Rik | lncRNA | 5:23850597-23855038 (+) |  | -0.6120 | 1.39e-03 | 5.76e-03 |
| ENSMUSG00000060261 | Gtf2i | protein\_coding | 5:134237834-134314760 (-) |  | -0.1870 | 1.39e-03 | 5.76e-03 |
| ENSMUSG00000005267 | Zfp287 | protein\_coding | 11:62700356-62731905 (-) |  | -0.5160 | 1.39e-03 | 5.77e-03 |
| ENSMUSG00000046230 | Vps13a | protein\_coding | 19:16615366-16780933 (-) |  | -0.2360 | 1.39e-03 | 5.77e-03 |
| ENSMUSG00000100441 | Gm7266 | processed\_pseudogene | 1:144312032-144312436 (-) |  | 1.3900 | 1.39e-03 | 5.77e-03 |
| ENSMUSG00000025217 | Btrc | protein\_coding | 19:45363734-45530013 (+) |  | 0.2870 | 1.40e-03 | 5.82e-03 |
| ENSMUSG00000018425 | Dhx40 | protein\_coding | 11:86768846-86807746 (-) |  | 0.1890 | 1.40e-03 | 5.82e-03 |
| ENSMUSG00000027368 | Dusp2 | protein\_coding | 2:127336159-127338376 (+) |  | 0.5300 | 1.41e-03 | 5.82e-03 |
| ENSMUSG00000047909 | Ankrd16 | protein\_coding | 2:11777876-11790329 (+) |  | 0.3220 | 1.41e-03 | 5.83e-03 |
| ENSMUSG00000022385 | Gtse1 | protein\_coding | 15:85859745-85876573 (+) |  | -0.2010 | 1.41e-03 | 5.83e-03 |
| ENSMUSG00000028821 | Syf2 | protein\_coding | 4:134930898-134937548 (+) |  | 0.2050 | 1.42e-03 | 5.85e-03 |
| ENSMUSG00000020538 | Srebf1 | protein\_coding | 11:60199089-60222581 (-) |  | -0.1720 | 1.42e-03 | 5.85e-03 |
| ENSMUSG00000002326 | Gmpr2 | protein\_coding | 14:55671941-55679200 (+) |  | -0.2230 | 1.42e-03 | 5.87e-03 |
| ENSMUSG00000054520 | Sh3bp2 | protein\_coding | 5:34525838-34563641 (+) |  | 0.1900 | 1.42e-03 | 5.88e-03 |
| ENSMUSG00000021715 | Cwc27 | protein\_coding | 13:104631140-104817142 (-) |  | -0.1960 | 1.43e-03 | 5.90e-03 |
| ENSMUSG00000021285 | Ppp1r13b | protein\_coding | 12:111828457-111908110 (-) |  | 0.3890 | 1.43e-03 | 5.90e-03 |
| ENSMUSG00000096403 | Gm9825 | processed\_pseudogene | 6:7982541-7983383 (-) |  | -0.6790 | 1.43e-03 | 5.92e-03 |
| ENSMUSG00000004661 | Arid3b | protein\_coding | 9:57790353-57836793 (-) |  | 0.3190 | 1.43e-03 | 5.92e-03 |
| ENSMUSG00000102780 | Gm38253 | TEC | 3:106725599-106730276 (+) |  | 0.5080 | 1.43e-03 | 5.92e-03 |
| ENSMUSG00000026520 | Pycr2 | protein\_coding | 1:180904293-180908088 (+) |  | -0.1870 | 1.43e-03 | 5.92e-03 |
| ENSMUSG00000032504 | Pdcd6ip | protein\_coding | 9:113651744-113708259 (-) |  | -0.1500 | 1.44e-03 | 5.95e-03 |
| ENSMUSG00000006818 | Sod2 | protein\_coding | 17:13006846-13040063 (+) |  | -0.1760 | 1.44e-03 | 5.95e-03 |
| ENSMUSG00000033192 | Lpcat2 | protein\_coding | 8:92855339-92919279 (+) |  | 0.4810 | 1.44e-03 | 5.96e-03 |
| ENSMUSG00000030342 | Cd9 | protein\_coding | 6:125460266-125494791 (-) |  | 0.4210 | 1.45e-03 | 5.96e-03 |
| ENSMUSG00000038845 | Phb | protein\_coding | 11:95666957-95680773 (+) |  | -0.2660 | 1.45e-03 | 5.96e-03 |
| ENSMUSG00000028064 | Sema4a | protein\_coding | 3:88435959-88461182 (-) |  | 0.1510 | 1.45e-03 | 5.96e-03 |
| ENSMUSG00000004054 | Map3k11 | protein\_coding | 19:5688742-5702865 (+) |  | 0.1340 | 1.45e-03 | 5.96e-03 |
| ENSMUSG00000005510 | Ndufs3 | protein\_coding | 2:90894634-90904827 (-) |  | -0.1440 | 1.46e-03 | 5.99e-03 |
| ENSMUSG00000043587 | Pxylp1 | protein\_coding | 9:96823336-96892669 (-) |  | 0.1700 | 1.46e-03 | 6.00e-03 |
| ENSMUSG00000118087 | 4833438C02Rik | lncRNA | 19:46303591-46305611 (-) |  | 0.3760 | 1.46e-03 | 6.01e-03 |
| ENSMUSG00000050106 | Tmc8 | protein\_coding | 11:117782076-117793110 (+) |  | 0.3250 | 1.46e-03 | 6.02e-03 |
| ENSMUSG00000049744 | Arhgap15 | protein\_coding | 2:43748824-44395953 (+) |  | 0.3190 | 1.46e-03 | 6.02e-03 |
| ENSMUSG00000007836 | Hnrnpa0 | protein\_coding | 13:58125879-58128556 (-) |  | -0.1640 | 1.46e-03 | 6.02e-03 |
| ENSMUSG00000022255 | Mtdh | protein\_coding | 15:34082694-34145624 (+) |  | 0.2410 | 1.47e-03 | 6.03e-03 |
| ENSMUSG00000008855 | Hdac5 | protein\_coding | 11:102194432-102230166 (-) |  | 0.1870 | 1.47e-03 | 6.05e-03 |
| ENSMUSG00000029490 | Mfsd7a | protein\_coding | 5:108441054-108449100 (-) |  | 0.2800 | 1.48e-03 | 6.06e-03 |
| ENSMUSG00000037470 | Uggt1 | protein\_coding | 1:36140027-36244720 (-) |  | -0.1740 | 1.48e-03 | 6.06e-03 |
| ENSMUSG00000020253 | Ppm1m | protein\_coding | 9:106194947-106199746 (-) |  | -0.1570 | 1.48e-03 | 6.08e-03 |
| ENSMUSG00000070034 | Sp110 | protein\_coding | 1:85576899-85598817 (-) |  | -0.1660 | 1.49e-03 | 6.10e-03 |
| ENSMUSG00000029634 | Rnf6 | protein\_coding | 5:146209192-146221555 (-) |  | 0.1550 | 1.49e-03 | 6.12e-03 |
| ENSMUSG00000024975 | Pdcd4 | protein\_coding | 19:53892231-53929860 (+) |  | 0.2940 | 1.51e-03 | 6.18e-03 |
| ENSMUSG00000029781 | Fkbp9 | protein\_coding | 6:56832059-56879358 (+) |  | -0.3970 | 1.51e-03 | 6.18e-03 |
| ENSMUSG00000061665 | Cd2ap | protein\_coding | 17:42792951-42876665 (-) |  | -0.2300 | 1.51e-03 | 6.18e-03 |
| ENSMUSG00000038679 | Trps1 | protein\_coding | 15:50654752-50890463 (-) |  | -0.2660 | 1.51e-03 | 6.19e-03 |
| ENSMUSG00000021460 | Auh | protein\_coding | 13:52835119-52929681 (-) |  | -0.1930 | 1.51e-03 | 6.19e-03 |
| ENSMUSG00000035212 | Leprot | protein\_coding | 4:101647718-101659364 (+) |  | 0.1920 | 1.52e-03 | 6.22e-03 |
| ENSMUSG00000031610 | Scrg1 | protein\_coding | 8:57455923-57477585 (+) |  | 2.6100 | 1.52e-03 | 6.23e-03 |
| ENSMUSG00000030917 | Tmem159 | protein\_coding | 7:120102353-120120992 (+) |  | 0.3110 | 1.52e-03 | 6.23e-03 |
| ENSMUSG00000031767 | Nudt7 | protein\_coding | 8:114133557-114154739 (+) |  | 0.3430 | 1.52e-03 | 6.23e-03 |
| ENSMUSG00000039768 | Dnajc11 | protein\_coding | 4:151933691-151982137 (+) |  | -0.1630 | 1.52e-03 | 6.23e-03 |
| ENSMUSG00000115205 | Gm16374 | processed\_pseudogene | 15:4885120-4886118 (+) |  | -0.7790 | 1.52e-03 | 6.23e-03 |
| ENSMUSG00000016018 | Mtrex | protein\_coding | 13:112867418-112927398 (-) |  | -0.2040 | 1.53e-03 | 6.25e-03 |
| ENSMUSG00000014846 | Tppp3 | protein\_coding | 8:105467493-105471526 (-) |  | 0.6340 | 1.53e-03 | 6.26e-03 |
| ENSMUSG00000062729 | Ppox | protein\_coding | 1:171275990-171281186 (-) |  | 0.2070 | 1.53e-03 | 6.26e-03 |
| ENSMUSG00000027620 | Rbm39 | protein\_coding | 2:156147239-156180238 (-) |  | 0.1760 | 1.53e-03 | 6.27e-03 |
| ENSMUSG00000032400 | Zwilch | protein\_coding | 9:64137144-64173104 (-) |  | -0.2020 | 1.54e-03 | 6.30e-03 |
| ENSMUSG00000019731 | Slc35e1 | protein\_coding | 8:72480641-72492614 (-) |  | -0.1440 | 1.54e-03 | 6.30e-03 |
| ENSMUSG00000031068 | Glrx3 | protein\_coding | 7:137437614-137468594 (+) |  | -0.2170 | 1.54e-03 | 6.31e-03 |
| ENSMUSG00000000339 | Rtca | protein\_coding | 3:116488963-116508208 (-) |  | -0.1510 | 1.55e-03 | 6.32e-03 |
| ENSMUSG00000020114 | Cand1 | protein\_coding | 10:119199255-119240055 (-) |  | -0.1810 | 1.55e-03 | 6.33e-03 |
| ENSMUSG00000040061 | Plcb2 | protein\_coding | 2:118707517-118728438 (-) |  | 0.2440 | 1.55e-03 | 6.33e-03 |
| ENSMUSG00000065954 | Tacc1 | protein\_coding | 8:25154552-25256588 (-) |  | 0.1330 | 1.56e-03 | 6.35e-03 |
| ENSMUSG00000104126 | Gm37486 | processed\_pseudogene | 1:178299396-178299800 (+) |  | -0.3520 | 1.56e-03 | 6.37e-03 |
| ENSMUSG00000028028 | Alpk1 | protein\_coding | 3:127670310-127780527 (-) |  | 0.2180 | 1.56e-03 | 6.37e-03 |
| ENSMUSG00000051890 | Klhdc1 | protein\_coding | 12:69241176-69284632 (+) |  | 0.4610 | 1.57e-03 | 6.41e-03 |
| ENSMUSG00000024309 | Pfdn6 | protein\_coding | 17:33938821-33940343 (-) |  | -0.1540 | 1.58e-03 | 6.42e-03 |
| ENSMUSG00000060288 | Ppih | protein\_coding | 4:119300010-119320546 (-) |  | -0.2570 | 1.58e-03 | 6.43e-03 |
| ENSMUSG00000008429 | Herpud2 | protein\_coding | 9:25108132-25151820 (-) |  | 0.1600 | 1.58e-03 | 6.45e-03 |
| ENSMUSG00000057421 | Las1l | protein\_coding | X:95935335-95956962 (-) |  | -0.1990 | 1.58e-03 | 6.45e-03 |
| ENSMUSG00000028394 | Pole3 | protein\_coding | 4:62522649-62525068 (-) |  | -0.2010 | 1.59e-03 | 6.45e-03 |
| ENSMUSG00000074170 | Plekhf1 | protein\_coding | 7:38216972-38228016 (-) |  | -0.3820 | 1.59e-03 | 6.49e-03 |
| ENSMUSG00000028788 | Ptp4a2 | protein\_coding | 4:129811219-129850003 (+) |  | 0.1570 | 1.60e-03 | 6.49e-03 |
| ENSMUSG00000003153 | Slc2a3 | protein\_coding | 6:122727809-122801640 (-) |  | 0.3990 | 1.60e-03 | 6.49e-03 |
| ENSMUSG00000017776 | Crk | protein\_coding | 11:75679259-75706908 (+) |  | 0.1900 | 1.60e-03 | 6.49e-03 |
| ENSMUSG00000112226 | Gm48786 | processed\_pseudogene | 10:51543035-51546848 (+) |  | -1.0300 | 1.60e-03 | 6.50e-03 |
| ENSMUSG00000055884 | Fancm | protein\_coding | 12:65075603-65132058 (+) |  | -0.2850 | 1.60e-03 | 6.50e-03 |
| ENSMUSG00000036594 | H2-Aa | protein\_coding | 17:34282744-34287823 (-) |  | 0.8860 | 1.60e-03 | 6.50e-03 |
| ENSMUSG00000020783 | Ncbp3 | protein\_coding | 11:73047783-73089317 (+) |  | -0.1440 | 1.60e-03 | 6.51e-03 |
| ENSMUSG00000008976 | Gabpa | protein\_coding | 16:84834925-84863779 (+) |  | -0.1370 | 1.61e-03 | 6.51e-03 |
| ENSMUSG00000019874 | Fabp7 | protein\_coding | 10:57784881-57788450 (+) |  | -1.2000 | 1.61e-03 | 6.51e-03 |
| ENSMUSG00000066315 | Gm12918 | processed\_pseudogene | 4:10848420-10849055 (+) |  | -0.3690 | 1.61e-03 | 6.53e-03 |
| ENSMUSG00000115584 | Gm5854 | processed\_pseudogene | 14:101981991-101982763 (-) |  | -0.5040 | 1.62e-03 | 6.55e-03 |
| ENSMUSG00000038542 | Pcid2 | protein\_coding | 8:13077189-13105459 (-) |  | -0.1890 | 1.62e-03 | 6.56e-03 |
| ENSMUSG00000117404 | Gm50035 | lncRNA | 18:15201112-15207791 (-) |  | 0.5300 | 1.62e-03 | 6.57e-03 |
| ENSMUSG00000032688 | Malt1 | protein\_coding | 18:65430938-65479067 (+) |  | 0.1990 | 1.62e-03 | 6.58e-03 |
| ENSMUSG00000046985 | Tapt1 | protein\_coding | 5:44175154-44226626 (-) |  | -0.1950 | 1.63e-03 | 6.59e-03 |
| ENSMUSG00000026743 | Mllt10 | protein\_coding | 2:18055237-18212388 (+) |  | 0.1390 | 1.63e-03 | 6.60e-03 |
| ENSMUSG00000047153 | Khnyn | protein\_coding | 14:55884947-55898775 (+) |  | 0.2390 | 1.63e-03 | 6.61e-03 |
| ENSMUSG00000029186 | Pi4k2b | protein\_coding | 5:52741574-52769340 (+) |  | -0.2570 | 1.63e-03 | 6.61e-03 |
| ENSMUSG00000002015 | Bcap31 | protein\_coding | X:73686178-73716175 (-) |  | -0.1410 | 1.64e-03 | 6.64e-03 |
| ENSMUSG00000030861 | Acadsb | protein\_coding | 7:131410601-131448944 (+) |  | -0.1720 | 1.64e-03 | 6.65e-03 |
| ENSMUSG00000058173 | Smco4 | protein\_coding | 9:15493432-15545260 (+) |  | -0.3840 | 1.64e-03 | 6.65e-03 |
| ENSMUSG00000007035 | Msh5 | protein\_coding | 17:35028605-35046745 (-) |  | 0.4220 | 1.65e-03 | 6.67e-03 |
| ENSMUSG00000026596 | Abl2 | protein\_coding | 1:156558786-156649568 (+) |  | -0.2340 | 1.65e-03 | 6.67e-03 |
| ENSMUSG00000032673 | Prorsd1 | protein\_coding | 11:29511757-29515033 (-) |  | -0.2770 | 1.65e-03 | 6.67e-03 |
| ENSMUSG00000029038 | Ssu72 | protein\_coding | 4:155704800-155733879 (+) |  | -0.2210 | 1.66e-03 | 6.69e-03 |
| ENSMUSG00000031833 | Mast3 | protein\_coding | 8:70778117-70805054 (-) |  | 0.2720 | 1.66e-03 | 6.70e-03 |
| ENSMUSG00000033610 | Pank1 | protein\_coding | 19:34806940-34879455 (-) |  | 0.2810 | 1.67e-03 | 6.74e-03 |
| ENSMUSG00000035372 | 1810055G02Rik | protein\_coding | 19:3708293-3717881 (+) |  | -0.3330 | 1.67e-03 | 6.75e-03 |
| ENSMUSG00000054855 | Rnd1 | protein\_coding | 15:98663421-98677461 (-) |  | -0.8490 | 1.67e-03 | 6.76e-03 |
| ENSMUSG00000026213 | Stk11ip | protein\_coding | 1:75521529-75537335 (+) |  | 0.2250 | 1.68e-03 | 6.79e-03 |
| ENSMUSG00000006638 | Abhd1 | polymorphic\_pseudogene | 5:30950066-30955091 (+) |  | 1.6800 | 1.70e-03 | 6.84e-03 |
| ENSMUSG00000023206 | Il15ra | protein\_coding | 2:11705290-11734317 (+) |  | -0.3460 | 1.70e-03 | 6.85e-03 |
[truncated: 24,583 more chars]
