# Supplementary material for: Transcriptome profiling of osteoclast subsets associated with arthritis: A pathogenic role of CCR2hi osteoclast progenitors
Source: Front Immunol. 2022 Dec 15;13:994035. doi: 10.3389/fimmu.2022.994035 (PMC9797520; doi:10.3389/fimmu.2022.994035)
Supplement: Supplementary file 12 [file DataSheet_4.zip › Supplementary data 4 DGE CTRL vs CIA all samples/Supplementary data 4 legend.docx]

Supplementary data 4. Differential gene expression analysis of control and collagen-induced arthritis (CIA) mice samples, regardless of osteoclast progenitor subset. Control mice samples (DG9-16) were compared to collagen-induced arthritis (CIA) mice samples (DG1-8) using DESeq 2 and a total of 3818 genes with a Benjamini-Hochberg (BH) correction adjusted p value lower than 0.01 were listed.

First column denotes Ensembl gene ID, followed by gene name in second column, gene type in third column and chromosomal location in the fourth column. Comparison graphs in the fifth column were ommited as to meet the file size constraints.

Sixth column denoted the difference in expression of genes based on intervention using log_2_ fold change comparison of control mice gene counts compared to CIA mice gene counts (more negative numbers equal higher expression in CIA samples and vice-versa).

p values obtained using Wald test are noted, as well as BH-correction adjusted p values in the last column. The columns are sortable by clicking on column heading, and all results are searchable by inputting text into the search box in the upper right corner.

The data is accessed using the provided .html file, while the folders contain accessory files needed for .html functionality.
