# Supplementary material for: Transcriptome profiling of osteoclast subsets associated with arthritis: A pathogenic role of CCR2hi osteoclast progenitors
Source: Front Immunol. 2022 Dec 15;13:994035. doi: 10.3389/fimmu.2022.994035 (PMC9797520; doi:10.3389/fimmu.2022.994035)
Supplement: Supplementary file 13 [file DataSheet_5.zip › Supplementary data 5 DGE CTRL vs CIA in CCR2hi/RNAseq_analysis_with_DESeq2_p0.01_extended.html]

RNA-seq analysis of differential expression using DESeq2, P value cutoff 0.01


## RNA-seq analysis of differential expression using DESeq2, P value cutoff 0.01

| ID | Name | Type | Position | Image | logFC | p-Value | Adjusted p-Value |
| --- | --- | --- | --- | --- | --- | --- | --- |
| ID | Name | Type | Position | Image | logFC | p-Value | Adjusted p-Value |
| ENSMUSG00000048612 | Myof | protein\_coding | 19:37899036-38043577 (-) |  | -2.150 | 3.20e-47 | 4.15e-43 |
| ENSMUSG00000034168 | Irf2bpl | protein\_coding | 12:86880701-86884798 (-) |  | -1.490 | 9.22e-29 | 5.98e-25 |
| ENSMUSG00000044103 | Il1f9 | protein\_coding | 2:24186476-24193568 (+) |  | -5.440 | 5.02e-28 | 2.05e-24 |
| ENSMUSG00000079227 | Ccr5 | protein\_coding | 9:124121543-124147699 (+) |  | -1.840 | 6.34e-28 | 2.05e-24 |
| ENSMUSG00000069917 | Hba-a2 | protein\_coding | 11:32296489-32297298 (+) |  | 3.200 | 7.90e-28 | 2.05e-24 |
| ENSMUSG00000052212 | Cd177 | protein\_coding | 7:24743983-24760311 (-) |  | 1.610 | 1.91e-26 | 4.13e-23 |
| ENSMUSG00000116114 | Gm35853 | lncRNA | 15:101322888-101405834 (+) |  | 1.950 | 9.09e-26 | 1.68e-22 |
| ENSMUSG00000024679 | Ms4a6d | protein\_coding | 19:11586604-11604849 (-) |  | -1.700 | 1.05e-25 | 1.69e-22 |
| ENSMUSG00000006800 | Sulf2 | protein\_coding | 2:166073089-166155663 (-) |  | 1.260 | 5.24e-25 | 7.55e-22 |
| ENSMUSG00000056643 | Chst13 | protein\_coding | 6:90308349-90325185 (-) |  | 1.930 | 4.37e-24 | 5.66e-21 |
| ENSMUSG00000039145 | Camk1d | protein\_coding | 2:5293457-5714515 (-) |  | 0.964 | 4.00e-23 | 4.72e-20 |
| ENSMUSG00000028927 | Padi2 | protein\_coding | 4:140906344-140952586 (+) |  | 1.490 | 3.04e-22 | 3.29e-19 |
| ENSMUSG00000048142 | Nat8l | protein\_coding | 5:33995984-34005916 (+) |  | 1.350 | 3.55e-22 | 3.54e-19 |
| ENSMUSG00000037235 | Mxd4 | protein\_coding | 5:34173883-34187720 (-) |  | 0.788 | 4.83e-22 | 4.47e-19 |
| ENSMUSG00000027199 | Gatm | protein\_coding | 2:122594467-122611303 (-) |  | -1.420 | 7.14e-22 | 6.00e-19 |
| ENSMUSG00000043939 | A530064D06Rik | protein\_coding | 17:48149126-48167275 (-) |  | 1.230 | 7.40e-22 | 6.00e-19 |
| ENSMUSG00000033170 | Card10 | protein\_coding | 15:78775138-78803042 (-) |  | 3.410 | 1.93e-21 | 1.47e-18 |
| ENSMUSG00000030560 | Ctsc | protein\_coding | 7:88278085-88310888 (+) |  | -1.200 | 2.28e-21 | 1.64e-18 |
| ENSMUSG00000046314 | Stxbp6 | protein\_coding | 12:44852484-45074709 (-) |  | 1.380 | 2.42e-21 | 1.65e-18 |
| ENSMUSG00000035847 | Ids | protein\_coding | X:70343069-70365084 (-) |  | 0.859 | 4.95e-21 | 3.21e-18 |
| ENSMUSG00000030427 | Lilra6 | protein\_coding | 7:3908280-3915503 (-) |  | 0.864 | 1.15e-20 | 7.11e-18 |
| ENSMUSG00000021097 | Clmn | protein\_coding | 12:104763117-104865076 (-) |  | -2.190 | 1.62e-20 | 9.54e-18 |
| ENSMUSG00000038970 | Lmtk2 | protein\_coding | 5:144100436-144188204 (+) |  | 0.998 | 6.04e-20 | 3.41e-17 |
| ENSMUSG00000059336 | Slc14a1 | protein\_coding | 18:78100091-78142119 (-) |  | 2.370 | 6.80e-20 | 3.68e-17 |
| ENSMUSG00000042082 | Arsb | protein\_coding | 13:93771630-93943016 (+) |  | 0.857 | 1.13e-19 | 5.87e-17 |
| ENSMUSG00000048442 | Smim5 | protein\_coding | 11:115899966-115906269 (+) |  | 1.410 | 1.70e-19 | 8.49e-17 |
| ENSMUSG00000048058 | Ldlrad3 | protein\_coding | 2:101950203-102186385 (-) |  | 0.899 | 2.70e-19 | 1.29e-16 |
| ENSMUSG00000021477 | Ctsl | protein\_coding | 13:64359337-64370890 (-) |  | -1.110 | 4.46e-19 | 2.07e-16 |
| ENSMUSG00000027864 | Ptgfrn | protein\_coding | 3:101040232-101110278 (-) |  | -1.350 | 7.40e-19 | 3.31e-16 |
| ENSMUSG00000022912 | Pros1 | protein\_coding | 16:62854307-62929346 (+) |  | -0.859 | 1.06e-18 | 4.47e-16 |
| ENSMUSG00000026170 | Cyp27a1 | protein\_coding | 1:74713574-74737892 (+) |  | 1.260 | 1.07e-18 | 4.47e-16 |
| ENSMUSG00000001227 | Sema6b | protein\_coding | 17:56123085-56140343 (-) |  | -1.520 | 1.23e-18 | 4.99e-16 |
| ENSMUSG00000060512 | 0610040J01Rik | protein\_coding | 5:63812363-63899625 (+) |  | -1.190 | 1.55e-18 | 6.08e-16 |
| ENSMUSG00000020593 | Lpin1 | protein\_coding | 12:16535669-16646966 (-) |  | 1.860 | 2.50e-18 | 9.33e-16 |
| ENSMUSG00000021356 | Irf4 | protein\_coding | 13:30749226-30766976 (+) |  | 1.650 | 2.52e-18 | 9.33e-16 |
| ENSMUSG00000037685 | Atp8a1 | protein\_coding | 5:67618140-67847434 (-) |  | -0.789 | 2.62e-18 | 9.45e-16 |
| ENSMUSG00000037224 | Zfyve28 | protein\_coding | 5:34194893-34288449 (-) |  | 1.890 | 6.97e-18 | 2.44e-15 |
| ENSMUSG00000045658 | Pid1 | protein\_coding | 1:84036296-84364180 (-) |  | 0.935 | 7.66e-18 | 2.61e-15 |
| ENSMUSG00000032369 | Plscr1 | protein\_coding | 9:92249750-92272278 (+) |  | -1.330 | 1.95e-17 | 6.48e-15 |
| ENSMUSG00000023045 | Soat2 | protein\_coding | 15:102150526-102163469 (+) |  | -2.160 | 2.61e-17 | 8.12e-15 |
| ENSMUSG00000025270 | Alas2 | protein\_coding | X:150547375-150570638 (+) |  | 2.410 | 2.63e-17 | 8.12e-15 |
| ENSMUSG00000055541 | Lair1 | protein\_coding | 7:4003402-4063204 (-) |  | -1.070 | 2.63e-17 | 8.12e-15 |
| ENSMUSG00000075010 | AW112010 | lncRNA | 19:11047612-11055808 (-) |  | -1.270 | 3.08e-17 | 9.28e-15 |
| ENSMUSG00000025330 | Padi4 | protein\_coding | 4:140745865-140774236 (-) |  | 0.833 | 4.24e-17 | 1.24e-14 |
| ENSMUSG00000009585 | Apobec3 | protein\_coding | 15:79891659-79915906 (+) |  | -0.591 | 4.31e-17 | 1.24e-14 |
| ENSMUSG00000020641 | Rsad2 | protein\_coding | 12:26442746-26456452 (-) |  | 1.450 | 4.86e-17 | 1.37e-14 |
| ENSMUSG00000038393 | Txnip | protein\_coding | 3:96557957-96561883 (+) |  | 0.886 | 1.13e-16 | 3.11e-14 |
| ENSMUSG00000056091 | St3gal5 | protein\_coding | 6:72097592-72154571 (+) |  | 1.530 | 1.59e-16 | 4.29e-14 |
| ENSMUSG00000038352 | Arl5c | protein\_coding | 11:97989578-97996181 (-) |  | 1.110 | 1.70e-16 | 4.51e-14 |
| ENSMUSG00000016552 | Foxred2 | protein\_coding | 15:77940522-77956722 (-) |  | 1.040 | 2.22e-16 | 5.73e-14 |
| ENSMUSG00000045038 | Prkce | protein\_coding | 17:86167785-86657919 (+) |  | 0.881 | 2.25e-16 | 5.73e-14 |
| ENSMUSG00000021127 | Zfp36l1 | protein\_coding | 12:80107754-80113013 (-) |  | -2.750 | 2.43e-16 | 6.05e-14 |
| ENSMUSG00000062127 | Cttnbp2nl | protein\_coding | 3:105001915-105053146 (-) |  | -1.810 | 2.67e-16 | 6.54e-14 |
| ENSMUSG00000017466 | Timp2 | protein\_coding | 11:118301069-118355740 (-) |  | 1.350 | 2.87e-16 | 6.90e-14 |
| ENSMUSG00000042719 | Naa25 | protein\_coding | 5:121397936-121444378 (+) |  | -0.706 | 3.93e-16 | 9.26e-14 |
| ENSMUSG00000026721 | Rabgap1l | protein\_coding | 1:160219174-160793211 (-) |  | 1.070 | 4.02e-16 | 9.30e-14 |
| ENSMUSG00000074151 | Nlrc5 | protein\_coding | 8:94434356-94527272 (+) |  | -0.859 | 5.05e-16 | 1.13e-13 |
| ENSMUSG00000041633 | Kctd12b | protein\_coding | X:153685154-153696391 (-) |  | 1.260 | 5.05e-16 | 1.13e-13 |
| ENSMUSG00000061353 | Cxcl12 | protein\_coding | 6:117168535-117181367 (+) |  | 2.490 | 6.70e-16 | 1.47e-13 |
| ENSMUSG00000025491 | Ifitm1 | protein\_coding | 7:140967221-140969825 (+) |  | -1.890 | 7.29e-16 | 1.58e-13 |
| ENSMUSG00000003420 | Fcgrt | protein\_coding | 7:45092990-45103851 (-) |  | 1.330 | 8.91e-16 | 1.89e-13 |
| ENSMUSG00000031805 | Jak3 | protein\_coding | 8:71676296-71690575 (+) |  | -1.100 | 9.38e-16 | 1.96e-13 |
| ENSMUSG00000038437 | Mllt6 | protein\_coding | 11:97663414-97685463 (+) |  | 0.923 | 1.04e-15 | 2.14e-13 |
| ENSMUSG00000024238 | Zeb1 | protein\_coding | 18:5591860-5775467 (+) |  | 1.580 | 1.41e-15 | 2.83e-13 |
| ENSMUSG00000040253 | Gbp7 | protein\_coding | 3:142530342-142550149 (+) |  | -1.110 | 1.42e-15 | 2.83e-13 |
| ENSMUSG00000030246 | Ldhb | protein\_coding | 6:142490249-142507957 (-) |  | 1.300 | 1.46e-15 | 2.88e-13 |
| ENSMUSG00000019806 | Aig1 | protein\_coding | 10:13647054-13868980 (-) |  | -2.180 | 1.75e-15 | 3.39e-13 |
| ENSMUSG00000038235 | F11r | protein\_coding | 1:171437535-171464603 (+) |  | -4.020 | 1.84e-15 | 3.52e-13 |
| ENSMUSG00000045087 | S1pr5 | protein\_coding | 9:21242912-21248443 (-) |  | 1.850 | 2.44e-15 | 4.59e-13 |
| ENSMUSG00000016024 | Lbp | protein\_coding | 2:158306493-158332852 (+) |  | -1.220 | 2.69e-15 | 4.98e-13 |
| ENSMUSG00000029082 | Bst1 | protein\_coding | 5:43818885-43843986 (+) |  | -1.400 | 2.90e-15 | 5.29e-13 |
| ENSMUSG00000032902 | Slc16a1 | protein\_coding | 3:104638668-104658462 (+) |  | -1.410 | 3.48e-15 | 6.18e-13 |
| ENSMUSG00000033720 | Sfxn5 | protein\_coding | 6:85213049-85333422 (-) |  | -1.060 | 3.48e-15 | 6.18e-13 |
| ENSMUSG00000066952 | Myo1h | protein\_coding | 5:114289166-114365357 (+) |  | 1.550 | 4.12e-15 | 7.22e-13 |
| ENSMUSG00000047180 | Neurl3 | protein\_coding | 1:36264597-36274679 (-) |  | 0.992 | 4.22e-15 | 7.29e-13 |
| ENSMUSG00000026768 | Itga8 | protein\_coding | 2:12106632-12301922 (-) |  | 3.430 | 4.50e-15 | 7.63e-13 |
| ENSMUSG00000022218 | Tgm1 | protein\_coding | 14:55700009-55713926 (-) |  | -1.640 | 4.53e-15 | 7.63e-13 |
| ENSMUSG00000015766 | Eps8 | protein\_coding | 6:137477245-137654876 (-) |  | -0.948 | 7.06e-15 | 1.17e-12 |
| ENSMUSG00000019528 | Gyg | protein\_coding | 3:20122084-20155317 (-) |  | -0.705 | 7.72e-15 | 1.27e-12 |
| ENSMUSG00000051748 | Wfdc21 | protein\_coding | 11:83746940-83752642 (+) |  | -4.320 | 1.08e-14 | 1.76e-12 |
| ENSMUSG00000027463 | Slc52a3 | protein\_coding | 2:151996511-152009258 (+) |  | -1.910 | 1.10e-14 | 1.76e-12 |
| ENSMUSG00000032192 | Gnb5 | protein\_coding | 9:75306288-75345876 (+) |  | -2.730 | 1.19e-14 | 1.88e-12 |
| ENSMUSG00000022010 | Tsc22d1 | protein\_coding | 14:76414961-76507765 (+) |  | 1.000 | 1.74e-14 | 2.72e-12 |
| ENSMUSG00000050379 | Sept6 | protein\_coding | X:36911326-36991794 (-) |  | 0.757 | 2.36e-14 | 3.64e-12 |
| ENSMUSG00000031494 | Cd209a | protein\_coding | 8:3743397-3748984 (-) |  | 3.320 | 2.43e-14 | 3.70e-12 |
| ENSMUSG00000051832 | E230016K23Rik | lncRNA | 11:83582056-83623693 (+) |  | 1.450 | 3.93e-14 | 5.93e-12 |
| ENSMUSG00000025701 | Alox5 | protein\_coding | 6:116410077-116461178 (-) |  | 1.380 | 4.05e-14 | 6.03e-12 |
| ENSMUSG00000028124 | Gclm | protein\_coding | 3:122245557-122270732 (+) |  | -1.150 | 4.72e-14 | 6.96e-12 |
| ENSMUSG00000032554 | Trf | protein\_coding | 9:103204001-103230444 (-) |  | 1.080 | 7.04e-14 | 1.03e-11 |
| ENSMUSG00000022094 | Slc39a14 | protein\_coding | 14:70303469-70351425 (-) |  | -2.320 | 7.52e-14 | 1.08e-11 |
| ENSMUSG00000026315 | Serpinb8 | protein\_coding | 1:107590006-107610484 (+) |  | -1.720 | 9.92e-14 | 1.41e-11 |
| ENSMUSG00000030083 | Abtb1 | protein\_coding | 6:88835914-88841984 (-) |  | 0.711 | 1.08e-13 | 1.53e-11 |
| ENSMUSG00000020021 | Fgd6 | protein\_coding | 10:94036001-94145339 (+) |  | -1.650 | 2.90e-13 | 4.04e-11 |
| ENSMUSG00000021948 | Prkcd | protein\_coding | 14:30595354-30626210 (-) |  | 0.567 | 3.76e-13 | 5.19e-11 |
| ENSMUSG00000023039 | Krt7 | protein\_coding | 15:101411043-101430313 (+) |  | 2.440 | 3.99e-13 | 5.45e-11 |
| ENSMUSG00000019820 | Utrn | protein\_coding | 10:12382188-12869365 (-) |  | 0.747 | 4.92e-13 | 6.64e-11 |
| ENSMUSG00000052534 | Pbx1 | protein\_coding | 1:168119364-168432270 (-) |  | -1.320 | 5.50e-13 | 7.36e-11 |
| ENSMUSG00000027219 | Slc28a2 | protein\_coding | 2:122426477-122461137 (+) |  | -0.986 | 6.20e-13 | 8.20e-11 |
| ENSMUSG00000049191 | Rtl5 | protein\_coding | X:102066544-102071304 (-) |  | 1.150 | 6.43e-13 | 8.42e-11 |
| ENSMUSG00000027452 | Acss1 | protein\_coding | 2:150618105-150668500 (-) |  | 1.290 | 7.06e-13 | 9.16e-11 |
| ENSMUSG00000024855 | Pacs1 | protein\_coding | 19:5133158-5273119 (-) |  | 0.635 | 7.48e-13 | 9.61e-11 |
| ENSMUSG00000024014 | Pim1 | protein\_coding | 17:29490753-29496112 (+) |  | -1.040 | 7.79e-13 | 9.90e-11 |
| ENSMUSG00000031441 | Atp11a | protein\_coding | 8:12757014-12868728 (+) |  | -2.100 | 8.22e-13 | 1.03e-10 |
| ENSMUSG00000021708 | Rasgrf2 | protein\_coding | 13:91880400-92131656 (-) |  | 1.360 | 8.40e-13 | 1.04e-10 |
| ENSMUSG00000062866 | Phactr2 | protein\_coding | 10:13207717-13474412 (-) |  | 0.812 | 8.48e-13 | 1.04e-10 |
| ENSMUSG00000052305 | Hbb-bs | protein\_coding | 7:103826534-103828096 (-) |  | 2.480 | 8.49e-13 | 1.04e-10 |
| ENSMUSG00000040204 | Pclaf | protein\_coding | 9:65890237-65903266 (+) |  | -0.620 | 1.01e-12 | 1.23e-10 |
| ENSMUSG00000040820 | Hlcs | protein\_coding | 16:94128882-94313571 (-) |  | 0.772 | 1.23e-12 | 1.48e-10 |
| ENSMUSG00000020787 | P2rx1 | protein\_coding | 11:72999103-73015200 (+) |  | 1.310 | 1.25e-12 | 1.48e-10 |
| ENSMUSG00000029471 | Camkk2 | protein\_coding | 5:122731170-122779409 (-) |  | 0.774 | 1.27e-12 | 1.49e-10 |
| ENSMUSG00000074203 | G430095P16Rik | protein\_coding | 8:84723007-84726844 (+) |  | 1.100 | 1.30e-12 | 1.52e-10 |
| ENSMUSG00000037989 | Wnk2 | protein\_coding | 13:49036303-49148014 (-) |  | 2.420 | 1.32e-12 | 1.53e-10 |
| ENSMUSG00000024247 | Pkdcc | protein\_coding | 17:83215292-83225070 (+) |  | 1.460 | 1.36e-12 | 1.56e-10 |
| ENSMUSG00000036353 | P2ry12 | protein\_coding | 3:59216272-59262871 (-) |  | -1.580 | 1.44e-12 | 1.63e-10 |
| ENSMUSG00000041607 | Mbp | protein\_coding | 18:82475146-82585637 (+) |  | 0.820 | 1.76e-12 | 1.98e-10 |
| ENSMUSG00000030147 | Clec4b1 | protein\_coding | 6:123049962-123071555 (+) |  | 1.420 | 1.79e-12 | 2.00e-10 |
| ENSMUSG00000020844 | Nxn | protein\_coding | 11:76257198-76399140 (-) |  | -0.920 | 2.21e-12 | 2.45e-10 |
| ENSMUSG00000001270 | Ckb | protein\_coding | 12:111669361-111672338 (-) |  | 1.150 | 2.27e-12 | 2.49e-10 |
| ENSMUSG00000022564 | Grina | protein\_coding | 15:76246764-76249904 (+) |  | -0.635 | 2.34e-12 | 2.55e-10 |
| ENSMUSG00000018381 | Abi3 | protein\_coding | 11:95830074-95842476 (-) |  | -1.060 | 2.43e-12 | 2.62e-10 |
| ENSMUSG00000039713 | Plekhg5 | protein\_coding | 4:152072498-152115400 (+) |  | 2.000 | 3.03e-12 | 3.25e-10 |
| ENSMUSG00000021037 | Ahsa1 | protein\_coding | 12:87266479-87273998 (+) |  | -0.520 | 3.30e-12 | 3.51e-10 |
| ENSMUSG00000037966 | Ninj1 | protein\_coding | 13:49187485-49196244 (+) |  | 0.748 | 3.41e-12 | 3.58e-10 |
| ENSMUSG00000027175 | Tcp11l1 | protein\_coding | 2:104657288-104712169 (-) |  | -0.892 | 3.42e-12 | 3.58e-10 |
| ENSMUSG00000022389 | Tef | protein\_coding | 15:81802421-81826863 (+) |  | 1.490 | 3.50e-12 | 3.62e-10 |
| ENSMUSG00000023947 | Nfkbie | protein\_coding | 17:45555703-45563169 (+) |  | 0.966 | 3.52e-12 | 3.62e-10 |
| ENSMUSG00000042659 | Arrdc4 | protein\_coding | 7:68736995-68749241 (-) |  | 0.710 | 4.21e-12 | 4.29e-10 |
| ENSMUSG00000026893 | Gca | protein\_coding | 2:62664285-62694109 (+) |  | -1.540 | 6.15e-12 | 6.23e-10 |
| ENSMUSG00000029925 | Tbxas1 | protein\_coding | 6:38875404-39084585 (+) |  | 0.780 | 6.35e-12 | 6.38e-10 |
| ENSMUSG00000026177 | Slc11a1 | protein\_coding | 1:74375195-74386062 (+) |  | 0.687 | 7.61e-12 | 7.53e-10 |
| ENSMUSG00000105504 | Gbp5 | protein\_coding | 3:142493978-142522344 (+) |  | -1.210 | 7.61e-12 | 7.53e-10 |
| ENSMUSG00000052698 | Tln2 | protein\_coding | 9:67217087-67559703 (-) |  | -2.420 | 9.93e-12 | 9.76e-10 |
| ENSMUSG00000015947 | Fcgr1 | protein\_coding | 3:96282909-96293969 (-) |  | -0.990 | 1.09e-11 | 1.06e-09 |
| ENSMUSG00000020644 | Id2 | protein\_coding | 12:25093799-25097140 (-) |  | 0.888 | 1.41e-11 | 1.36e-09 |
| ENSMUSG00000032508 | Myd88 | protein\_coding | 9:119335934-119341411 (-) |  | -0.513 | 1.42e-11 | 1.36e-09 |
| ENSMUSG00000032011 | Thy1 | protein\_coding | 9:44043384-44048579 (+) |  | 2.040 | 1.43e-11 | 1.37e-09 |
| ENSMUSG00000024180 | Tmem8 | protein\_coding | 17:26113299-26123254 (+) |  | 0.762 | 1.46e-11 | 1.38e-09 |
| ENSMUSG00000031391 | L1cam | protein\_coding | X:73853778-73896105 (-) |  | 0.809 | 1.61e-11 | 1.51e-09 |
| ENSMUSG00000021458 | Aopep | protein\_coding | 13:62964893-63326096 (+) |  | -0.812 | 1.65e-11 | 1.54e-09 |
| ENSMUSG00000054675 | Tmem119 | protein\_coding | 5:113793729-113800516 (-) |  | 1.210 | 1.81e-11 | 1.68e-09 |
| ENSMUSG00000066687 | Zbtb16 | protein\_coding | 9:48654297-48836222 (-) |  | 1.980 | 1.83e-11 | 1.68e-09 |
| ENSMUSG00000025150 | Cbr2 | protein\_coding | 11:120729489-120732114 (-) |  | 1.620 | 1.91e-11 | 1.74e-09 |
| ENSMUSG00000054676 | 1600014C10Rik | protein\_coding | 7:38183217-38197568 (+) |  | -0.593 | 2.14e-11 | 1.94e-09 |
| ENSMUSG00000038831 | Ralgps1 | protein\_coding | 2:33133417-33371486 (-) |  | 1.260 | 2.28e-11 | 2.05e-09 |
| ENSMUSG00000019873 | Reep3 | protein\_coding | 10:67009189-67096945 (-) |  | 0.549 | 2.56e-11 | 2.29e-09 |
| ENSMUSG00000025648 | Pfkfb4 | protein\_coding | 9:108991778-109032228 (+) |  | 0.671 | 2.60e-11 | 2.31e-09 |
| ENSMUSG00000028523 | Tctex1d1 | protein\_coding | 4:102978606-103005594 (+) |  | -4.050 | 2.68e-11 | 2.37e-09 |
| ENSMUSG00000014158 | Trpv4 | protein\_coding | 5:114622152-114658421 (-) |  | 1.970 | 2.83e-11 | 2.48e-09 |
| ENSMUSG00000021270 | Hsp90aa1 | protein\_coding | 12:110690605-110702728 (-) |  | -0.663 | 2.93e-11 | 2.55e-09 |
| ENSMUSG00000031555 | Adam9 | protein\_coding | 8:24949611-25016927 (-) |  | -0.738 | 3.25e-11 | 2.81e-09 |
| ENSMUSG00000039831 | Arhgap29 | protein\_coding | 3:121952541-122016753 (+) |  | 2.470 | 3.60e-11 | 3.09e-09 |
| ENSMUSG00000003206 | Ebi3 | protein\_coding | 17:55952640-55957022 (+) |  | 1.300 | 3.92e-11 | 3.34e-09 |
| ENSMUSG00000034226 | Rhov | protein\_coding | 2:119269201-119271272 (-) |  | -3.980 | 3.97e-11 | 3.34e-09 |
| ENSMUSG00000020869 | Lrrc59 | protein\_coding | 11:94629767-94645216 (+) |  | -0.680 | 3.98e-11 | 3.34e-09 |
| ENSMUSG00000031444 | F10 | protein\_coding | 8:13037308-13056676 (+) |  | -0.639 | 3.99e-11 | 3.34e-09 |
| ENSMUSG00000060962 | Dmkn | protein\_coding | 7:30763756-30781063 (+) |  | -2.990 | 4.60e-11 | 3.82e-09 |
| ENSMUSG00000019850 | Tnfaip3 | protein\_coding | 10:19000910-19015657 (-) |  | 0.883 | 4.72e-11 | 3.90e-09 |
| ENSMUSG00000045917 | Tmem268 | protein\_coding | 4:63558781-63586357 (+) |  | -0.447 | 4.80e-11 | 3.93e-09 |
| ENSMUSG00000029816 | Gpnmb | protein\_coding | 6:49036546-49070929 (+) |  | -1.110 | 5.86e-11 | 4.78e-09 |
| ENSMUSG00000024349 | Tmem173 | protein\_coding | 18:35733679-35740554 (-) |  | -0.441 | 6.70e-11 | 5.43e-09 |
| ENSMUSG00000020846 | Rflnb | protein\_coding | 11:76019194-76027782 (-) |  | 0.591 | 7.01e-11 | 5.65e-09 |
| ENSMUSG00000030468 | Siglecg | protein\_coding | 7:43408204-43418358 (+) |  | 0.975 | 7.20e-11 | 5.77e-09 |
| ENSMUSG00000038633 | Degs1 | protein\_coding | 1:182275772-182282804 (-) |  | 0.395 | 7.44e-11 | 5.91e-09 |
| ENSMUSG00000040078 | Ptges3-ps | processed\_pseudogene | 6:85843980-85844459 (+) |  | -0.733 | 8.02e-11 | 6.34e-09 |
| ENSMUSG00000032089 | Il10ra | protein\_coding | 9:45253837-45269149 (-) |  | 0.528 | 8.10e-11 | 6.36e-09 |
| ENSMUSG00000025980 | Hspd1 | protein\_coding | 1:55077835-55088243 (-) |  | -0.650 | 8.14e-11 | 6.36e-09 |
| ENSMUSG00000032531 | Amotl2 | protein\_coding | 9:102716672-102733418 (+) |  | -1.380 | 1.01e-10 | 7.87e-09 |
| ENSMUSG00000047866 | Lonp2 | protein\_coding | 8:86624043-86723873 (+) |  | -0.546 | 1.11e-10 | 8.56e-09 |
| ENSMUSG00000021486 | Prelid1 | protein\_coding | 13:55320500-55325272 (+) |  | -0.542 | 1.14e-10 | 8.74e-09 |
| ENSMUSG00000044229 | Nxpe4 | protein\_coding | 9:48162023-48400025 (+) |  | 1.070 | 1.18e-10 | 8.99e-09 |
| ENSMUSG00000055538 | Zcchc24 | protein\_coding | 14:25711642-25769039 (-) |  | 0.535 | 1.19e-10 | 9.03e-09 |
| ENSMUSG00000017309 | Cd300lg | protein\_coding | 11:102041509-102055620 (+) |  | 0.495 | 1.25e-10 | 9.45e-09 |
| ENSMUSG00000050022 | Amz1 | protein\_coding | 5:140724127-140761439 (+) |  | 1.160 | 1.27e-10 | 9.54e-09 |
| ENSMUSG00000045817 | Zfp36l2 | protein\_coding | 17:84183931-84187947 (-) |  | 0.637 | 1.32e-10 | 9.86e-09 |
| ENSMUSG00000044788 | Fads6 | protein\_coding | 11:115279622-115297663 (-) |  | 1.140 | 1.36e-10 | 1.00e-08 |
| ENSMUSG00000038456 | Dennd2a | protein\_coding | 6:39462378-39557867 (-) |  | 0.931 | 1.36e-10 | 1.00e-08 |
| ENSMUSG00000000686 | Abhd15 | protein\_coding | 11:77515121-77538607 (+) |  | 0.642 | 1.43e-10 | 1.04e-08 |
| ENSMUSG00000073678 | Pgap1 | protein\_coding | 1:54472994-54557684 (-) |  | 0.801 | 1.46e-10 | 1.06e-08 |
| ENSMUSG00000002983 | Relb | protein\_coding | 7:19606217-19629438 (-) |  | 0.835 | 1.47e-10 | 1.06e-08 |
| ENSMUSG00000042688 | Mapk6 | protein\_coding | 9:75369062-75410005 (-) |  | -0.635 | 1.66e-10 | 1.20e-08 |
| ENSMUSG00000021707 | Dhfr | protein\_coding | 13:92354726-92389053 (+) |  | -0.646 | 1.70e-10 | 1.22e-08 |
| ENSMUSG00000001229 | Dpp9 | protein\_coding | 17:56186807-56218905 (-) |  | -0.704 | 1.74e-10 | 1.24e-08 |
| ENSMUSG00000039542 | Ncam1 | protein\_coding | 9:49502136-49798925 (-) |  | 1.040 | 1.76e-10 | 1.24e-08 |
| ENSMUSG00000029304 | Spp1 | protein\_coding | 5:104435118-104441050 (+) |  | -1.260 | 1.83e-10 | 1.29e-08 |
| ENSMUSG00000027454 | Gins1 | protein\_coding | 2:150905400-150931280 (+) |  | -0.636 | 2.13e-10 | 1.50e-08 |
| ENSMUSG00000038147 | Cd84 | protein\_coding | 1:171839697-171890718 (+) |  | 0.721 | 2.16e-10 | 1.50e-08 |
| ENSMUSG00000030214 | Plbd1 | protein\_coding | 6:136612070-136661928 (-) |  | 0.680 | 2.19e-10 | 1.52e-08 |
| ENSMUSG00000099775 | Gm5960 | unprocessed\_pseudogene | 15:75071112-75074638 (+) |  | 1.060 | 2.21e-10 | 1.52e-08 |
| ENSMUSG00000041936 | Agrn | protein\_coding | 4:156165290-156197488 (-) |  | 1.020 | 2.35e-10 | 1.61e-08 |
| ENSMUSG00000042105 | Inpp5f | protein\_coding | 7:128611328-128696425 (+) |  | 0.534 | 2.41e-10 | 1.64e-08 |
| ENSMUSG00000032802 | Srxn1 | protein\_coding | 2:152105516-152111376 (+) |  | -1.100 | 3.19e-10 | 2.17e-08 |
| ENSMUSG00000030605 | Mfge8 | protein\_coding | 7:79133768-79149060 (-) |  | 0.817 | 3.24e-10 | 2.18e-08 |
| ENSMUSG00000025743 | Sdc3 | protein\_coding | 4:130792537-130826319 (+) |  | 0.634 | 3.26e-10 | 2.18e-08 |
| ENSMUSG00000040084 | Bub1b | protein\_coding | 2:118598211-118641591 (+) |  | -0.490 | 3.26e-10 | 2.18e-08 |
| ENSMUSG00000037280 | Galnt6 | protein\_coding | 15:100691813-100729376 (-) |  | -0.973 | 3.28e-10 | 2.18e-08 |
| ENSMUSG00000028811 | Yars | protein\_coding | 4:129189760-129219607 (+) |  | -0.643 | 3.46e-10 | 2.29e-08 |
| ENSMUSG00000103865 | Gm37416 | TEC | 2:16023108-16023425 (-) |  | -0.760 | 3.61e-10 | 2.38e-08 |
| ENSMUSG00000071547 | Nt5dc2 | protein\_coding | 14:31131053-31139124 (+) |  | -0.925 | 3.74e-10 | 2.45e-08 |
| ENSMUSG00000024579 | Pcyox1l | protein\_coding | 18:61696837-61707635 (-) |  | -0.925 | 3.86e-10 | 2.52e-08 |
| ENSMUSG00000023571 | C1qtnf12 | protein\_coding | 4:155962318-155966629 (+) |  | 0.877 | 3.95e-10 | 2.56e-08 |
| ENSMUSG00000006574 | Slc4a1 | protein\_coding | 11:102348824-102366203 (-) |  | 1.750 | 4.42e-10 | 2.84e-08 |
| ENSMUSG00000029430 | Ran | protein\_coding | 5:129020069-129024323 (+) |  | -0.609 | 4.42e-10 | 2.84e-08 |
| ENSMUSG00000098557 | Kctd12 | protein\_coding | 14:102976581-102982637 (-) |  | 0.469 | 4.69e-10 | 2.99e-08 |
| ENSMUSG00000074896 | Ifit3 | protein\_coding | 19:34583531-34588731 (+) |  | 1.450 | 4.75e-10 | 3.02e-08 |
| ENSMUSG00000017756 | Slc12a7 | protein\_coding | 13:73733094-73816754 (+) |  | -0.947 | 5.09e-10 | 3.22e-08 |
| ENSMUSG00000041926 | Rnpep | protein\_coding | 1:135262712-135284084 (-) |  | -0.567 | 5.51e-10 | 3.47e-08 |
| ENSMUSG00000038872 | Zfhx3 | protein\_coding | 8:107942644-108961630 (+) |  | 0.857 | 5.65e-10 | 3.54e-08 |
| ENSMUSG00000058624 | Gda | protein\_coding | 19:21391307-21473445 (-) |  | -0.700 | 6.33e-10 | 3.93e-08 |
| ENSMUSG00000035824 | Tk2 | protein\_coding | 8:104226685-104248558 (-) |  | 0.648 | 6.34e-10 | 3.93e-08 |
| ENSMUSG00000030123 | Plxnd1 | protein\_coding | 6:115954811-115995005 (-) |  | 0.559 | 6.38e-10 | 3.94e-08 |
| ENSMUSG00000038127 | Ccdc50 | protein\_coding | 16:27388869-27452218 (+) |  | 0.475 | 6.43e-10 | 3.95e-08 |
| ENSMUSG00000025817 | Nudt5 | protein\_coding | 2:5845019-5871895 (+) |  | -0.559 | 6.49e-10 | 3.95e-08 |
| ENSMUSG00000030867 | Plk1 | protein\_coding | 7:122159439-122169873 (+) |  | -0.552 | 6.51e-10 | 3.95e-08 |
| ENSMUSG00000031765 | Mt1 | protein\_coding | 8:94179082-94180327 (+) |  | -1.320 | 6.52e-10 | 3.95e-08 |
| ENSMUSG00000028268 | Gbp3 | protein\_coding | 3:142560026-142573209 (+) |  | -0.799 | 6.71e-10 | 4.05e-08 |
| ENSMUSG00000074794 | Arrdc3 | protein\_coding | 13:80883384-80896042 (+) |  | 0.560 | 6.95e-10 | 4.17e-08 |
| ENSMUSG00000064267 | Hvcn1 | protein\_coding | 5:122206804-122242297 (+) |  | 0.834 | 7.23e-10 | 4.32e-08 |
| ENSMUSG00000032724 | Abtb2 | protein\_coding | 2:103566310-103718423 (+) |  | -1.010 | 7.64e-10 | 4.54e-08 |
| ENSMUSG00000047798 | Cd300lf | protein\_coding | 11:115116214-115133992 (-) |  | -0.846 | 7.91e-10 | 4.68e-08 |
| ENSMUSG00000040624 | Plekhg1 | protein\_coding | 10:3740364-3967303 (+) |  | 0.614 | 7.96e-10 | 4.69e-08 |
| ENSMUSG00000043017 | Ptgir | protein\_coding | 7:16906490-16910905 (+) |  | 0.849 | 8.40e-10 | 4.93e-08 |
| ENSMUSG00000035891 | Cerk | protein\_coding | 15:86139128-86186141 (-) |  | 0.506 | 8.44e-10 | 4.93e-08 |
| ENSMUSG00000041707 | Tmem273 | protein\_coding | 14:32785963-32817984 (+) |  | -1.470 | 8.64e-10 | 5.02e-08 |
| ENSMUSG00000035673 | Sbno2 | protein\_coding | 10:80056992-80105571 (-) |  | -0.513 | 8.75e-10 | 5.07e-08 |
| ENSMUSG00000045404 | Kcnk13 | protein\_coding | 12:99964499-100062682 (+) |  | -2.550 | 8.94e-10 | 5.15e-08 |
| ENSMUSG00000039899 | Fgl2 | protein\_coding | 5:21372642-21378374 (+) |  | -0.673 | 9.10e-10 | 5.22e-08 |
| ENSMUSG00000014470 | Rnf166 | protein\_coding | 8:122466147-122476064 (-) |  | 0.555 | 9.52e-10 | 5.44e-08 |
| ENSMUSG00000070031 | Sp140 | protein\_coding | 1:85600378-85645037 (+) |  | -0.542 | 9.68e-10 | 5.50e-08 |
| ENSMUSG00000035725 | Prkx | protein\_coding | X:77761411-77796278 (-) |  | -0.372 | 1.01e-09 | 5.74e-08 |
| ENSMUSG00000052459 | Atp6v1a | protein\_coding | 16:44085402-44139705 (-) |  | -0.476 | 1.02e-09 | 5.76e-08 |
| ENSMUSG00000030748 | Il4ra | protein\_coding | 7:125552120-125579474 (+) |  | -0.853 | 1.04e-09 | 5.85e-08 |
| ENSMUSG00000027315 | Spint1 | protein\_coding | 2:119237362-119249527 (+) |  | -5.210 | 1.06e-09 | 5.93e-08 |
| ENSMUSG00000042249 | Grk3 | protein\_coding | 5:112910482-113015791 (-) |  | 0.847 | 1.10e-09 | 6.10e-08 |
| ENSMUSG00000032271 | Nnmt | protein\_coding | 9:48591877-48605153 (-) |  | -3.020 | 1.15e-09 | 6.37e-08 |
| ENSMUSG00000004609 | Cd33 | protein\_coding | 7:43524216-43544428 (-) |  | 1.050 | 1.18e-09 | 6.49e-08 |
| ENSMUSG00000001288 | Rarg | protein\_coding | 15:102234938-102257517 (-) |  | 0.622 | 1.26e-09 | 6.91e-08 |
| ENSMUSG00000020089 | Ppa1 | protein\_coding | 10:61648552-61674168 (+) |  | -0.976 | 1.28e-09 | 7.00e-08 |
| ENSMUSG00000085611 | Ap3s1-ps1 | processed\_pseudogene | X:38685592-38686170 (-) |  | -0.885 | 1.30e-09 | 7.06e-08 |
| ENSMUSG00000002279 | Lmf1 | protein\_coding | 17:25579085-25662826 (+) |  | 0.779 | 1.30e-09 | 7.06e-08 |
| ENSMUSG00000020250 | Txnrd1 | protein\_coding | 10:82833951-82897712 (+) |  | -0.616 | 1.36e-09 | 7.33e-08 |
| ENSMUSG00000025007 | Aldh18a1 | protein\_coding | 19:40550257-40588463 (-) |  | -0.883 | 1.36e-09 | 7.33e-08 |
| ENSMUSG00000079057 | Cyp4v3 | protein\_coding | 8:45304944-45333216 (-) |  | 0.720 | 1.42e-09 | 7.59e-08 |
| ENSMUSG00000047735 | Samd9l | protein\_coding | 6:3372257-3399572 (-) |  | 0.867 | 1.55e-09 | 8.28e-08 |
| ENSMUSG00000005802 | Slc30a4 | protein\_coding | 2:122681233-122702663 (-) |  | -1.520 | 1.60e-09 | 8.48e-08 |
| ENSMUSG00000032575 | Manf | protein\_coding | 9:106838312-106891979 (-) |  | -0.591 | 1.62e-09 | 8.55e-08 |
| ENSMUSG00000037260 | Hgsnat | protein\_coding | 8:25944453-25976753 (-) |  | 0.560 | 1.62e-09 | 8.56e-08 |
| ENSMUSG00000005237 | Dnah2 | protein\_coding | 11:69420809-69549110 (-) |  | 0.999 | 1.78e-09 | 9.36e-08 |
| ENSMUSG00000029674 | Limk1 | protein\_coding | 5:134656039-134688598 (-) |  | 0.624 | 1.80e-09 | 9.39e-08 |
| ENSMUSG00000005732 | Ranbp1 | protein\_coding | 16:18239784-18248732 (-) |  | -0.610 | 1.80e-09 | 9.39e-08 |
| ENSMUSG00000033307 | Mif | protein\_coding | 10:75859353-75860240 (-) |  | -1.010 | 1.82e-09 | 9.46e-08 |
| ENSMUSG00000028599 | Tnfrsf1b | protein\_coding | 4:145213463-145246870 (-) |  | 0.471 | 1.99e-09 | 1.03e-07 |
| ENSMUSG00000035165 | Kcne3 | protein\_coding | 7:100176502-100184869 (+) |  | 1.150 | 2.06e-09 | 1.06e-07 |
| ENSMUSG00000028228 | Cpne3 | protein\_coding | 4:19519254-19570108 (-) |  | -0.470 | 2.14e-09 | 1.09e-07 |
| ENSMUSG00000001054 | Rmnd5b | protein\_coding | 11:51623671-51635896 (-) |  | 0.539 | 2.17e-09 | 1.11e-07 |
| ENSMUSG00000040699 | Limd2 | protein\_coding | 11:106156256-106160860 (-) |  | 0.554 | 2.19e-09 | 1.11e-07 |
| ENSMUSG00000027379 | Bub1 | protein\_coding | 2:127801122-127831865 (-) |  | -0.514 | 2.19e-09 | 1.11e-07 |
| ENSMUSG00000011256 | Adam19 | protein\_coding | 11:46055992-46147343 (+) |  | 2.060 | 2.26e-09 | 1.14e-07 |
| ENSMUSG00000029247 | Paics | protein\_coding | 5:76951307-76967509 (+) |  | -0.439 | 2.37e-09 | 1.19e-07 |
| ENSMUSG00000000028 | Cdc45 | protein\_coding | 16:18780447-18811987 (-) |  | -0.592 | 2.42e-09 | 1.21e-07 |
| ENSMUSG00000036587 | Fut7 | protein\_coding | 2:25423267-25426374 (+) |  | 1.130 | 2.45e-09 | 1.22e-07 |
| ENSMUSG00000030978 | Rrm1 | protein\_coding | 7:102441695-102469771 (+) |  | -0.550 | 2.46e-09 | 1.22e-07 |
| ENSMUSG00000028524 | Sgip1 | protein\_coding | 4:102741297-102973628 (+) |  | -2.290 | 2.58e-09 | 1.28e-07 |
| ENSMUSG00000025001 | Hells | protein\_coding | 19:38930915-38971051 (+) |  | -0.659 | 2.75e-09 | 1.36e-07 |
| ENSMUSG00000022360 | Atad2 | protein\_coding | 15:58094044-58135082 (-) |  | -0.554 | 2.98e-09 | 1.46e-07 |
| ENSMUSG00000066682 | Pilrb2 | protein\_coding | 5:137865827-137871815 (-) |  | 0.837 | 2.99e-09 | 1.46e-07 |
| ENSMUSG00000008206 | Cers4 | protein\_coding | 8:4493026-4531680 (+) |  | 1.840 | 3.20e-09 | 1.56e-07 |
| ENSMUSG00000041324 | Inhba | protein\_coding | 13:16011851-16031621 (+) |  | -2.740 | 3.25e-09 | 1.58e-07 |
| ENSMUSG00000071644 | Eef1g | protein\_coding | 19:8967041-8978479 (+) |  | -0.572 | 3.40e-09 | 1.65e-07 |
| ENSMUSG00000041571 | Selenow | protein\_coding | 7:15917208-15922402 (-) |  | 0.790 | 3.45e-09 | 1.66e-07 |
| ENSMUSG00000063193 | Cd300lb | protein\_coding | 11:114922781-114934386 (-) |  | 0.596 | 3.55e-09 | 1.71e-07 |
| ENSMUSG00000024732 | Ccdc86 | protein\_coding | 19:10941481-10949266 (-) |  | -0.684 | 3.57e-09 | 1.71e-07 |
| ENSMUSG00000037544 | Dlgap5 | protein\_coding | 14:47387779-47418407 (-) |  | -0.412 | 3.71e-09 | 1.77e-07 |
| ENSMUSG00000021190 | Lgmn | protein\_coding | 12:102394084-102439813 (-) |  | -0.610 | 3.94e-09 | 1.87e-07 |
| ENSMUSG00000030223 | Ptpro | protein\_coding | 6:137252319-137463233 (+) |  | 0.678 | 4.24e-09 | 2.01e-07 |
| ENSMUSG00000034041 | Lyl1 | protein\_coding | 8:84701449-84704940 (+) |  | 0.615 | 4.40e-09 | 2.07e-07 |
| ENSMUSG00000005824 | Tnfsf14 | protein\_coding | 17:57189492-57194177 (-) |  | -0.712 | 4.60e-09 | 2.16e-07 |
| ENSMUSG00000026193 | Fn1 | protein\_coding | 1:71585520-71653200 (-) |  | 0.679 | 4.78e-09 | 2.24e-07 |
| ENSMUSG00000086291 | Gm15513 | lncRNA | 5:34211810-34213802 (-) |  | 1.540 | 4.83e-09 | 2.25e-07 |
| ENSMUSG00000023805 | Synj2 | protein\_coding | 17:5941280-6044290 (+) |  | -0.998 | 4.87e-09 | 2.26e-07 |
| ENSMUSG00000039005 | Tlr4 | protein\_coding | 4:66827584-66930284 (+) |  | -0.809 | 5.05e-09 | 2.34e-07 |
| ENSMUSG00000029314 | Gpat3 | protein\_coding | 5:100845713-100899102 (+) |  | -1.680 | 5.26e-09 | 2.43e-07 |
| ENSMUSG00000056529 | Ptafr | protein\_coding | 4:132564067-132582683 (+) |  | 0.652 | 5.40e-09 | 2.48e-07 |
| ENSMUSG00000030272 | Camk1 | protein\_coding | 6:113334124-113343984 (-) |  | 0.832 | 5.68e-09 | 2.60e-07 |
| ENSMUSG00000040247 | Tbc1d10c | protein\_coding | 19:4183411-4191284 (-) |  | 1.590 | 5.69e-09 | 2.60e-07 |
| ENSMUSG00000052833 | Sae1 | protein\_coding | 7:16320234-16387806 (-) |  | -0.505 | 5.75e-09 | 2.62e-07 |
| ENSMUSG00000028063 | Lmna | protein\_coding | 3:88480147-88509956 (-) |  | -1.230 | 5.84e-09 | 2.65e-07 |
| ENSMUSG00000036777 | Anln | protein\_coding | 9:22332012-22389188 (-) |  | -0.444 | 5.91e-09 | 2.67e-07 |
| ENSMUSG00000042066 | Tmcc2 | protein\_coding | 1:132356315-132391281 (-) |  | 0.727 | 6.00e-09 | 2.70e-07 |
| ENSMUSG00000052336 | Cx3cr1 | protein\_coding | 9:119901616-120069879 (-) |  | 1.630 | 6.02e-09 | 2.70e-07 |
| ENSMUSG00000036528 | Ppfibp2 | protein\_coding | 7:107595207-107748583 (+) |  | 0.898 | 6.11e-09 | 2.73e-07 |
| ENSMUSG00000022636 | Alcam | protein\_coding | 16:52248996-52454074 (-) |  | 0.589 | 6.15e-09 | 2.74e-07 |
| ENSMUSG00000040327 | Cul9 | protein\_coding | 17:46500572-46546388 (-) |  | 0.832 | 6.36e-09 | 2.83e-07 |
| ENSMUSG00000022221 | Ripk3 | protein\_coding | 14:55784995-55788865 (-) |  | -0.594 | 6.47e-09 | 2.86e-07 |
| ENSMUSG00000074093 | Svip | protein\_coding | 7:51997171-52006018 (-) |  | 0.610 | 6.48e-09 | 2.86e-07 |
| ENSMUSG00000021830 | Txndc16 | protein\_coding | 14:45133465-45220328 (-) |  | 0.652 | 6.59e-09 | 2.90e-07 |
| ENSMUSG00000022574 | Naprt | protein\_coding | 15:75890956-75894481 (-) |  | -1.670 | 6.95e-09 | 3.04e-07 |
| ENSMUSG00000047963 | Stbd1 | protein\_coding | 5:92603041-92606579 (+) |  | 1.510 | 7.06e-09 | 3.08e-07 |
| ENSMUSG00000026245 | Farsb | protein\_coding | 1:78417975-78488897 (-) |  | -0.600 | 7.25e-09 | 3.15e-07 |
| ENSMUSG00000037331 | Larp1 | protein\_coding | 11:58009064-58062034 (+) |  | -0.549 | 7.48e-09 | 3.24e-07 |
| ENSMUSG00000038780 | Smurf1 | protein\_coding | 5:144876495-144965847 (-) |  | 0.525 | 7.52e-09 | 3.25e-07 |
| ENSMUSG00000027778 | Ift80 | protein\_coding | 3:68892499-69004570 (-) |  | -0.722 | 7.54e-09 | 3.25e-07 |
| ENSMUSG00000040592 | Cd79b | protein\_coding | 11:106311341-106314762 (-) |  | 1.080 | 7.60e-09 | 3.26e-07 |
| ENSMUSG00000071713 | Csf2rb | protein\_coding | 15:78325752-78353847 (+) |  | -0.496 | 7.89e-09 | 3.38e-07 |
| ENSMUSG00000028378 | Ptgr1 | protein\_coding | 4:58965439-58987119 (-) |  | -0.500 | 8.20e-09 | 3.49e-07 |
| ENSMUSG00000025151 | Maged1 | protein\_coding | X:94535474-94542143 (-) |  | -2.280 | 8.21e-09 | 3.49e-07 |
| ENSMUSG00000035835 | Plppr3 | protein\_coding | 10:79860475-79874634 (-) |  | -0.906 | 8.29e-09 | 3.51e-07 |
| ENSMUSG00000020423 | Btg2 | protein\_coding | 1:134075170-134079120 (-) |  | 0.805 | 8.69e-09 | 3.67e-07 |
| ENSMUSG00000041695 | Kcnj2 | protein\_coding | 11:111066164-111076821 (+) |  | -1.190 | 9.11e-09 | 3.83e-07 |
| ENSMUSG00000038807 | Rap1gap2 | protein\_coding | 11:74383356-74610915 (-) |  | 0.542 | 9.20e-09 | 3.86e-07 |
| ENSMUSG00000036362 | P2ry13 | protein\_coding | 3:59207892-59210882 (-) |  | -1.320 | 9.23e-09 | 3.86e-07 |
| ENSMUSG00000041797 | Abca9 | protein\_coding | 11:110100749-110168196 (-) |  | 0.952 | 9.67e-09 | 4.03e-07 |
| ENSMUSG00000039263 | Npepl1 | protein\_coding | 2:174110349-174123070 (+) |  | 0.463 | 9.73e-09 | 4.04e-07 |
| ENSMUSG00000051166 | Eml5 | protein\_coding | 12:98786805-98901484 (-) |  | 1.350 | 9.79e-09 | 4.05e-07 |
| ENSMUSG00000039234 | Sec24d | protein\_coding | 3:123267455-123365641 (+) |  | -0.694 | 1.00e-08 | 4.13e-07 |
| ENSMUSG00000021213 | Akr1c13 | protein\_coding | 13:4191150-4205596 (+) |  | 0.918 | 1.03e-08 | 4.23e-07 |
| ENSMUSG00000029650 | Slc46a3 | protein\_coding | 5:147878437-147894815 (-) |  | 1.200 | 1.07e-08 | 4.41e-07 |
| ENSMUSG00000038387 | Rras | protein\_coding | 7:45017961-45021647 (+) |  | 0.806 | 1.08e-08 | 4.43e-07 |
| ENSMUSG00000041736 | Tspo | protein\_coding | 15:83563592-83574203 (+) |  | -0.636 | 1.09e-08 | 4.45e-07 |
| ENSMUSG00000029084 | Cd38 | protein\_coding | 5:43868553-43912375 (+) |  | -5.530 | 1.11e-08 | 4.49e-07 |
| ENSMUSG00000003955 | Fam162a | protein\_coding | 16:36043761-36071594 (-) |  | -0.707 | 1.11e-08 | 4.50e-07 |
| ENSMUSG00000097077 | Gm16712 | lncRNA | 17:55954771-55959381 (-) |  | 1.580 | 1.13e-08 | 4.57e-07 |
| ENSMUSG00000028630 | Dyrk2 | protein\_coding | 10:118855603-118870209 (-) |  | 0.564 | 1.21e-08 | 4.87e-07 |
| ENSMUSG00000022114 | Spry2 | protein\_coding | 14:105891947-105896819 (-) |  | -1.970 | 1.21e-08 | 4.88e-07 |
| ENSMUSG00000044197 | Gpr146 | protein\_coding | 5:139377697-139396415 (+) |  | -0.757 | 1.23e-08 | 4.93e-07 |
| ENSMUSG00000062488 | Ifit3b | protein\_coding | 19:34607970-34613401 (+) |  | 2.150 | 1.30e-08 | 5.17e-07 |
| ENSMUSG00000049999 | Ppp1r3d | protein\_coding | 2:178411206-178414472 (-) |  | -0.871 | 1.32e-08 | 5.23e-07 |
| ENSMUSG00000020455 | Trim11 | protein\_coding | 11:58978093-58991458 (+) |  | -0.443 | 1.36e-08 | 5.37e-07 |
| ENSMUSG00000021116 | Eif2s1 | protein\_coding | 12:78861819-78887010 (+) |  | -0.478 | 1.36e-08 | 5.37e-07 |
| ENSMUSG00000031129 | Slc9a9 | protein\_coding | 9:94669909-95230445 (+) |  | 0.782 | 1.38e-08 | 5.45e-07 |
| ENSMUSG00000026003 | Acadl | protein\_coding | 1:66830839-66863277 (-) |  | -0.481 | 1.42e-08 | 5.58e-07 |
| ENSMUSG00000028587 | Orc1 | protein\_coding | 4:108579423-108614833 (+) |  | -0.911 | 1.43e-08 | 5.60e-07 |
| ENSMUSG00000031838 | Ifi30 | lncRNA | 8:70762774-70766663 (-) |  | -0.572 | 1.44e-08 | 5.61e-07 |
| ENSMUSG00000027597 | Ahcy | protein\_coding | 2:155059310-155074497 (-) |  | -0.659 | 1.48e-08 | 5.77e-07 |
| ENSMUSG00000030413 | Pglyrp1 | protein\_coding | 7:18871331-18890459 (+) |  | 1.150 | 1.50e-08 | 5.83e-07 |
| ENSMUSG00000017144 | Rnd3 | protein\_coding | 2:51130438-51149111 (-) |  | 0.560 | 1.52e-08 | 5.87e-07 |
| ENSMUSG00000026068 | Il18rap | protein\_coding | 1:40515362-40551705 (+) |  | -2.900 | 1.56e-08 | 6.02e-07 |
| ENSMUSG00000038732 | Mboat1 | protein\_coding | 13:30136489-30246717 (+) |  | -0.956 | 1.58e-08 | 6.06e-07 |
| ENSMUSG00000000673 | Haao | protein\_coding | 17:83831156-83847963 (-) |  | 0.747 | 1.59e-08 | 6.08e-07 |
| ENSMUSG00000044533 | Rps2 | protein\_coding | 17:24718116-24721929 (+) |  | -0.588 | 1.62e-08 | 6.20e-07 |
| ENSMUSG00000024905 | Tesmin | protein\_coding | 19:3388857-3407823 (+) |  | 1.150 | 1.63e-08 | 6.23e-07 |
| ENSMUSG00000020493 | Prr11 | protein\_coding | 11:87089153-87108708 (-) |  | -0.361 | 1.65e-08 | 6.26e-07 |
| ENSMUSG00000031907 | Zfp90 | protein\_coding | 8:106415327-106426598 (+) |  | 0.798 | 1.72e-08 | 6.52e-07 |
| ENSMUSG00000020272 | Stk10 | protein\_coding | 11:32533305-32624587 (+) |  | 0.481 | 1.73e-08 | 6.53e-07 |
| ENSMUSG00000033294 | Noc4l | protein\_coding | 5:110648418-110653417 (-) |  | -0.587 | 1.74e-08 | 6.55e-07 |
| ENSMUSG00000029447 | Cct6a | protein\_coding | 5:129786998-129846371 (+) |  | -0.498 | 1.76e-08 | 6.60e-07 |
| ENSMUSG00000028270 | Gbp2 | protein\_coding | 3:142620602-142638008 (+) |  | -1.050 | 1.91e-08 | 7.16e-07 |
| ENSMUSG00000047747 | Rnf150 | protein\_coding | 8:82863356-83091268 (+) |  | 1.270 | 1.92e-08 | 7.16e-07 |
| ENSMUSG00000019960 | Dusp6 | protein\_coding | 10:99263231-99267489 (+) |  | 1.550 | 1.96e-08 | 7.30e-07 |
| ENSMUSG00000028614 | Ndc1 | protein\_coding | 4:107367784-107416346 (+) |  | -0.543 | 2.04e-08 | 7.57e-07 |
| ENSMUSG00000050410 | Tcf19 | protein\_coding | 17:35512734-35516824 (-) |  | -0.659 | 2.08e-08 | 7.72e-07 |
| ENSMUSG00000024070 | Prkd3 | protein\_coding | 17:78949405-79020816 (-) |  | 0.352 | 2.10e-08 | 7.75e-07 |
| ENSMUSG00000024480 | Ap3s1 | protein\_coding | 18:46741876-46790826 (+) |  | -0.663 | 2.12e-08 | 7.83e-07 |
| ENSMUSG00000018167 | Stard3 | protein\_coding | 11:98358368-98381112 (+) |  | 0.429 | 2.22e-08 | 8.17e-07 |
| ENSMUSG00000037601 | Nme1 | protein\_coding | 11:93956979-93968521 (-) |  | -0.596 | 2.25e-08 | 8.24e-07 |
| ENSMUSG00000023046 | Igfbp6 | protein\_coding | 15:102144362-102149511 (+) |  | -2.510 | 2.28e-08 | 8.34e-07 |
| ENSMUSG00000011179 | Odc1 | protein\_coding | 12:17544794-17551505 (+) |  | -0.735 | 2.32e-08 | 8.43e-07 |
| ENSMUSG00000037149 | Ddx1 | protein\_coding | 12:13216973-13249213 (-) |  | -0.554 | 2.40e-08 | 8.72e-07 |
| ENSMUSG00000027322 | Siglec1 | protein\_coding | 2:131069220-131086765 (-) |  | 1.210 | 2.43e-08 | 8.82e-07 |
| ENSMUSG00000031539 | Ap3m2 | protein\_coding | 8:22787354-22805622 (-) |  | 0.782 | 2.54e-08 | 9.17e-07 |
| ENSMUSG00000037465 | Klf10 | protein\_coding | 15:38291463-38300706 (-) |  | 0.666 | 2.59e-08 | 9.33e-07 |
| ENSMUSG00000028851 | Nudc | protein\_coding | 4:133532542-133545996 (-) |  | -0.469 | 2.65e-08 | 9.52e-07 |
| ENSMUSG00000029162 | Khk | protein\_coding | 5:30921431-30931248 (+) |  | 0.781 | 2.67e-08 | 9.56e-07 |
| ENSMUSG00000058818 | Pirb | protein\_coding | 7:3711409-3720391 (-) |  | -0.486 | 2.71e-08 | 9.67e-07 |
| ENSMUSG00000000759 | Tubgcp3 | protein\_coding | 8:12614277-12672248 (-) |  | -0.415 | 2.74e-08 | 9.75e-07 |
| ENSMUSG00000042675 | Ypel3 | protein\_coding | 7:126776955-126780514 (+) |  | 0.605 | 2.79e-08 | 9.90e-07 |
| ENSMUSG00000025059 | Gk | protein\_coding | X:85701937-85776819 (-) |  | -0.606 | 2.82e-08 | 1.00e-06 |
| ENSMUSG00000038463 | Olfml2b | protein\_coding | 1:170644532-170682789 (+) |  | -2.570 | 2.84e-08 | 1.00e-06 |
| ENSMUSG00000020661 | Dnmt3a | protein\_coding | 12:3806007-3914443 (+) |  | 0.595 | 2.85e-08 | 1.00e-06 |
| ENSMUSG00000028212 | Ccne2 | protein\_coding | 4:11191351-11204779 (+) |  | -0.583 | 2.86e-08 | 1.00e-06 |
| ENSMUSG00000071714 | Csf2rb2 | protein\_coding | 15:78282507-78305721 (-) |  | -0.576 | 2.91e-08 | 1.02e-06 |
| ENSMUSG00000000359 | Rem1 | protein\_coding | 2:152626951-152635198 (+) |  | 0.846 | 2.94e-08 | 1.03e-06 |
| ENSMUSG00000016757 | Ttll12 | protein\_coding | 15:83575090-83595157 (-) |  | -0.886 | 3.02e-08 | 1.05e-06 |
| ENSMUSG00000067203 | H2-K2 | transcribed\_unprocessed\_pseudogene | 17:33974659-33978827 (-) |  | 1.150 | 3.06e-08 | 1.06e-06 |
| ENSMUSG00000062070 | Pgk1 | protein\_coding | X:106187100-106203699 (+) |  | -0.578 | 3.34e-08 | 1.16e-06 |
| ENSMUSG00000018363 | Smurf2 | protein\_coding | 11:106820066-106920715 (-) |  | 0.439 | 3.40e-08 | 1.18e-06 |
| ENSMUSG00000103367 | Gm38158 | processed\_pseudogene | 1:176835724-176836062 (+) |  | 0.915 | 3.43e-08 | 1.18e-06 |
| ENSMUSG00000037095 | Lrg1 | protein\_coding | 17:56119678-56122001 (-) |  | -5.650 | 3.45e-08 | 1.19e-06 |
| ENSMUSG00000021892 | Sh3bp5 | protein\_coding | 14:31359880-31436078 (-) |  | 0.528 | 3.48e-08 | 1.19e-06 |
| ENSMUSG00000025794 | Rpl14 | protein\_coding | 9:120571444-120574654 (+) |  | -0.431 | 3.51e-08 | 1.20e-06 |
| ENSMUSG00000030231 | Plekha5 | protein\_coding | 6:140424054-140597110 (+) |  | -1.260 | 3.51e-08 | 1.20e-06 |
| ENSMUSG00000042042 | Csgalnact2 | protein\_coding | 6:118107452-118139140 (-) |  | 0.478 | 3.56e-08 | 1.21e-06 |
| ENSMUSG00000021087 | Rtn1 | protein\_coding | 12:72211752-72409054 (-) |  | 1.550 | 3.62e-08 | 1.23e-06 |
| ENSMUSG00000039126 | Prune2 | protein\_coding | 19:16956118-17223932 (+) |  | -1.230 | 3.63e-08 | 1.23e-06 |
| ENSMUSG00000011752 | Pgam1 | protein\_coding | 19:41911923-41918660 (+) |  | -0.525 | 3.69e-08 | 1.25e-06 |
| ENSMUSG00000073940 | Hbb-bt | protein\_coding | 7:103812524-103813996 (-) |  | 2.860 | 3.79e-08 | 1.28e-06 |
| ENSMUSG00000053113 | Socs3 | protein\_coding | 11:117966079-117970047 (-) |  | -2.820 | 3.97e-08 | 1.33e-06 |
| ENSMUSG00000028108 | Ecm1 | protein\_coding | 3:95734147-95739569 (-) |  | -0.966 | 4.05e-08 | 1.36e-06 |
| ENSMUSG00000051811 | Cox6b2 | protein\_coding | 7:4751792-4753094 (-) |  | 0.954 | 4.14e-08 | 1.38e-06 |
| ENSMUSG00000002083 | Bbc3 | protein\_coding | 7:16308393-16318205 (+) |  | 0.952 | 4.15e-08 | 1.38e-06 |
| ENSMUSG00000056153 | Socs6 | protein\_coding | 18:88665224-88927481 (-) |  | 0.525 | 4.16e-08 | 1.38e-06 |
| ENSMUSG00000030512 | Snrpa1 | protein\_coding | 7:66059003-66074587 (+) |  | -0.464 | 4.31e-08 | 1.43e-06 |
| ENSMUSG00000022075 | Rhobtb2 | protein\_coding | 14:69784990-69805636 (-) |  | -0.730 | 4.51e-08 | 1.49e-06 |
| ENSMUSG00000034652 | Cd300a | protein\_coding | 11:114890041-114904654 (+) |  | 0.514 | 4.52e-08 | 1.49e-06 |
| ENSMUSG00000021340 | Gpld1 | protein\_coding | 13:24943152-24992501 (+) |  | -1.300 | 4.53e-08 | 1.49e-06 |
| ENSMUSG00000032740 | Ccdc88a | protein\_coding | 11:29373658-29510808 (+) |  | 0.526 | 4.65e-08 | 1.53e-06 |
| ENSMUSG00000079547 | H2-DMb1 | protein\_coding | 17:34153072-34160230 (+) |  | 0.669 | 4.69e-08 | 1.54e-06 |
| ENSMUSG00000023963 | Cyp39a1 | protein\_coding | 17:43667425-43751431 (+) |  | -0.571 | 4.72e-08 | 1.54e-06 |
| ENSMUSG00000002068 | Ccne1 | protein\_coding | 7:38097984-38107534 (-) |  | -0.765 | 4.76e-08 | 1.55e-06 |
| ENSMUSG00000011832 | Evi5l | protein\_coding | 8:4166567-4211257 (+) |  | 0.896 | 4.77e-08 | 1.55e-06 |
| ENSMUSG00000004633 | Chn2 | protein\_coding | 6:54039554-54301810 (+) |  | 0.800 | 4.78e-08 | 1.55e-06 |
| ENSMUSG00000024299 | Adamts10 | protein\_coding | 17:33524204-33553782 (+) |  | 0.659 | 4.80e-08 | 1.55e-06 |
| ENSMUSG00000053219 | Raet1e | protein\_coding | 10:22158569-22374139 (+) |  | -1.540 | 4.83e-08 | 1.55e-06 |
| ENSMUSG00000067038 | Rps12-ps3 | processed\_pseudogene | 19:59322371-59322766 (+) |  | -0.694 | 4.83e-08 | 1.55e-06 |
| ENSMUSG00000032232 | Cgnl1 | protein\_coding | 9:71626509-71771602 (-) |  | 1.430 | 4.83e-08 | 1.55e-06 |
| ENSMUSG00000050350 | Gpr18 | protein\_coding | 14:121911253-121915781 (-) |  | 1.300 | 4.88e-08 | 1.56e-06 |
| ENSMUSG00000041920 | Slc16a6 | protein\_coding | 11:109450855-109473598 (-) |  | -0.583 | 5.18e-08 | 1.65e-06 |
| ENSMUSG00000062991 | Nrg1 | protein\_coding | 8:31814551-32884797 (-) |  | -0.669 | 5.38e-08 | 1.71e-06 |
| ENSMUSG00000032946 | Rasgrp2 | protein\_coding | 19:6399340-6415216 (+) |  | 0.453 | 5.44e-08 | 1.73e-06 |
| ENSMUSG00000016194 | Hsd11b1 | protein\_coding | 1:193221634-193264075 (-) |  | -0.929 | 5.50e-08 | 1.74e-06 |
| ENSMUSG00000024966 | Stip1 | protein\_coding | 19:7020702-7039967 (-) |  | -0.493 | 5.57e-08 | 1.76e-06 |
| ENSMUSG00000030109 | Slc6a12 | protein\_coding | 6:121343076-121365775 (+) |  | 6.540 | 5.63e-08 | 1.78e-06 |
| ENSMUSG00000004105 | Angptl2 | protein\_coding | 2:33216069-33247717 (+) |  | 1.820 | 5.76e-08 | 1.81e-06 |
| ENSMUSG00000029922 | Mkrn1 | protein\_coding | 6:39397804-39420462 (-) |  | 0.408 | 5.85e-08 | 1.83e-06 |
| ENSMUSG00000021969 | Zdhhc20 | protein\_coding | 14:57832703-57890276 (-) |  | 0.506 | 5.94e-08 | 1.86e-06 |
| ENSMUSG00000030207 | Fam234b | protein\_coding | 6:135197977-135244955 (+) |  | -0.811 | 6.08e-08 | 1.90e-06 |
| ENSMUSG00000079111 | Kdelr2 | protein\_coding | 5:143403838-143421901 (+) |  | -0.479 | 6.34e-08 | 1.98e-06 |
| ENSMUSG00000037509 | Arhgef4 | protein\_coding | 1:34678188-34813309 (+) |  | 1.040 | 6.59e-08 | 2.05e-06 |
| ENSMUSG00000025355 | Mmp19 | protein\_coding | 10:128790910-128800824 (+) |  | -0.758 | 6.64e-08 | 2.06e-06 |
| ENSMUSG00000063605 | Ccdc102a | protein\_coding | 8:94902869-94918098 (-) |  | 0.945 | 6.66e-08 | 2.06e-06 |
| ENSMUSG00000062585 | Cnr2 | protein\_coding | 4:135895394-135920207 (+) |  | 0.446 | 6.82e-08 | 2.11e-06 |
| ENSMUSG00000038179 | Slamf7 | protein\_coding | 1:171632403-171653035 (-) |  | 1.780 | 6.96e-08 | 2.14e-06 |
| ENSMUSG00000068129 | Cst7 | protein\_coding | 2:150570415-150578944 (+) |  | -1.240 | 7.03e-08 | 2.16e-06 |
| ENSMUSG00000029414 | Kntc1 | protein\_coding | 5:123749716-123821593 (+) |  | -0.674 | 7.07e-08 | 2.17e-06 |
| ENSMUSG00000020277 | Pfkl | protein\_coding | 10:77986947-78010083 (-) |  | -0.636 | 7.12e-08 | 2.18e-06 |
| ENSMUSG00000030541 | Idh2 | protein\_coding | 7:80094846-80115392 (-) |  | -0.458 | 7.14e-08 | 2.18e-06 |
| ENSMUSG00000026275 | Ppp1r7 | protein\_coding | 1:93342854-93373489 (+) |  | -0.443 | 7.16e-08 | 2.18e-06 |
| ENSMUSG00000069255 | Dusp22 | protein\_coding | 13:30659999-30711231 (+) |  | 0.544 | 7.24e-08 | 2.20e-06 |
| ENSMUSG00000051444 | Bbs12 | protein\_coding | 3:37312554-37321453 (+) |  | -1.150 | 7.77e-08 | 2.35e-06 |
| ENSMUSG00000021178 | Psmc1 | protein\_coding | 12:100110154-100123405 (+) |  | -0.463 | 7.81e-08 | 2.36e-06 |
| ENSMUSG00000005142 | Man2b1 | protein\_coding | 8:85083270-85098282 (+) |  | 0.403 | 7.98e-08 | 2.39e-06 |
| ENSMUSG00000022587 | Ly6e | protein\_coding | 15:74955051-74959905 (+) |  | -0.443 | 7.99e-08 | 2.39e-06 |
| ENSMUSG00000023055 | Calcoco1 | protein\_coding | 15:102706777-102722178 (-) |  | 0.786 | 8.00e-08 | 2.39e-06 |
| ENSMUSG00000062980 | Cped1 | protein\_coding | 6:21985916-22256404 (+) |  | -1.290 | 8.00e-08 | 2.39e-06 |
| ENSMUSG00000054766 | Set | protein\_coding | 2:30057378-30072577 (+) |  | -0.429 | 8.03e-08 | 2.39e-06 |
| ENSMUSG00000024063 | Lbh | protein\_coding | 17:72918305-72941947 (+) |  | 0.505 | 8.03e-08 | 2.39e-06 |
| ENSMUSG00000020689 | Itgb3 | protein\_coding | 11:104608000-104670476 (+) |  | 1.930 | 8.17e-08 | 2.43e-06 |
| ENSMUSG00000009291 | Pttg1ip | protein\_coding | 10:77581720-77598732 (+) |  | 0.368 | 8.21e-08 | 2.44e-06 |
| ENSMUSG00000073412 | Lst1 | protein\_coding | 17:35185095-35188439 (-) |  | 0.760 | 8.44e-08 | 2.50e-06 |
| ENSMUSG00000031486 | Adgra2 | protein\_coding | 8:27085583-27123436 (+) |  | 1.720 | 8.49e-08 | 2.51e-06 |
| ENSMUSG00000067714 | Lpar5 | protein\_coding | 6:125067920-125082472 (+) |  | 0.655 | 8.73e-08 | 2.57e-06 |
| ENSMUSG00000040532 | Abhd11 | protein\_coding | 5:135009152-135012175 (+) |  | -0.584 | 8.92e-08 | 2.62e-06 |
| ENSMUSG00000027203 | Dut | protein\_coding | 2:125247190-125258608 (+) |  | -0.475 | 9.22e-08 | 2.70e-06 |
| ENSMUSG00000033444 | Specc1l | protein\_coding | 10:75212073-75312743 (+) |  | 0.414 | 9.28e-08 | 2.71e-06 |
| ENSMUSG00000073418 | C4b | protein\_coding | 17:34728380-34743882 (-) |  | -2.480 | 9.51e-08 | 2.78e-06 |
| ENSMUSG00000032594 | Ip6k1 | protein\_coding | 9:108002501-108048782 (+) |  | 0.386 | 9.63e-08 | 2.81e-06 |
| ENSMUSG00000024610 | Cd74 | protein\_coding | 18:60803848-60812652 (+) |  | 1.260 | 9.65e-08 | 2.81e-06 |
| ENSMUSG00000038871 | Bpgm | protein\_coding | 6:34476207-34505613 (+) |  | 0.803 | 9.97e-08 | 2.89e-06 |
| ENSMUSG00000048922 | Cdca2 | protein\_coding | 14:67676331-67715841 (-) |  | -0.445 | 1.03e-07 | 2.98e-06 |
| ENSMUSG00000018169 | Mfng | protein\_coding | 15:78755882-78773475 (-) |  | 0.835 | 1.04e-07 | 3.02e-06 |
| ENSMUSG00000004929 | Thop1 | protein\_coding | 10:81070035-81082559 (+) |  | -0.773 | 1.05e-07 | 3.02e-06 |
| ENSMUSG00000086425 | F730016J06Rik | lncRNA | 2:28095477-28127731 (+) |  | 0.894 | 1.06e-07 | 3.04e-06 |
| ENSMUSG00000062906 | Hdac10 | protein\_coding | 15:89123307-89128700 (-) |  | 0.717 | 1.07e-07 | 3.06e-06 |
| ENSMUSG00000026192 | Atic | protein\_coding | 1:71557150-71579631 (+) |  | -0.562 | 1.07e-07 | 3.07e-06 |
| ENSMUSG00000003309 | Ap1m2 | protein\_coding | 9:21294275-21312337 (-) |  | -1.490 | 1.10e-07 | 3.14e-06 |
| ENSMUSG00000025512 | Chid1 | protein\_coding | 7:141493136-141539857 (-) |  | -0.508 | 1.10e-07 | 3.14e-06 |
| ENSMUSG00000044037 | Als2cl | protein\_coding | 9:110879870-110900530 (+) |  | 1.240 | 1.11e-07 | 3.15e-06 |
| ENSMUSG00000015846 | Rxra | protein\_coding | 2:27676440-27762957 (+) |  | 0.553 | 1.11e-07 | 3.16e-06 |
| ENSMUSG00000031586 | Rbpms | protein\_coding | 8:33782643-33929863 (-) |  | 1.330 | 1.14e-07 | 3.24e-06 |
| ENSMUSG00000075595 | Zfp652 | protein\_coding | 11:95712673-95835115 (+) |  | 0.459 | 1.21e-07 | 3.41e-06 |
| ENSMUSG00000030094 | Xpc | protein\_coding | 6:91489305-91515888 (-) |  | 0.564 | 1.22e-07 | 3.45e-06 |
| ENSMUSG00000015396 | Cd83 | protein\_coding | 13:43784775-43803132 (+) |  | 6.100 | 1.30e-07 | 3.65e-06 |
| ENSMUSG00000057729 | Prtn3 | protein\_coding | 10:79874476-79883174 (+) |  | -1.450 | 1.31e-07 | 3.68e-06 |
| ENSMUSG00000018909 | Arrb1 | protein\_coding | 7:99535466-99606771 (+) |  | 0.467 | 1.32e-07 | 3.69e-06 |
| ENSMUSG00000020649 | Rrm2 | protein\_coding | 12:24708241-24714146 (+) |  | -0.616 | 1.33e-07 | 3.71e-06 |
| ENSMUSG00000054364 | Rhob | protein\_coding | 12:8497661-8500009 (-) |  | 0.728 | 1.34e-07 | 3.74e-06 |
| ENSMUSG00000022512 | Cldn1 | protein\_coding | 16:26356642-26371841 (-) |  | 2.030 | 1.35e-07 | 3.76e-06 |
| ENSMUSG00000026478 | Lamc1 | protein\_coding | 1:153218922-153332786 (-) |  | -3.110 | 1.35e-07 | 3.76e-06 |
| ENSMUSG00000038524 | Fchsd1 | protein\_coding | 18:37957431-37969774 (-) |  | 0.998 | 1.37e-07 | 3.81e-06 |
| ENSMUSG00000055067 | Smyd3 | protein\_coding | 1:178951960-179518041 (-) |  | -0.807 | 1.38e-07 | 3.81e-06 |
| ENSMUSG00000044468 | Tent5c | protein\_coding | 3:100451628-100489324 (-) |  | 1.260 | 1.43e-07 | 3.95e-06 |
| ENSMUSG00000040026 | Saa3 | protein\_coding | 7:46711998-46715700 (-) |  | -3.780 | 1.44e-07 | 3.96e-06 |
| ENSMUSG00000034858 | Fam214a | protein\_coding | 9:74952884-75032468 (+) |  | 1.270 | 1.44e-07 | 3.96e-06 |
| ENSMUSG00000091811 | Inafm1 | protein\_coding | 7:16272013-16273617 (-) |  | 0.712 | 1.46e-07 | 3.99e-06 |
| ENSMUSG00000095742 | CAAA01147332.1 | protein\_coding | JH584295.1:66-1479 (-) |  | 0.770 | 1.47e-07 | 4.01e-06 |
| ENSMUSG00000031613 | Hpgd | protein\_coding | 8:56294585-56321043 (+) |  | 2.350 | 1.50e-07 | 4.10e-06 |
| ENSMUSG00000044734 | Serpinb1a | protein\_coding | 13:32842092-32851185 (-) |  | -0.675 | 1.52e-07 | 4.13e-06 |
| ENSMUSG00000068039 | Tcp1 | protein\_coding | 17:12915701-12925067 (+) |  | -0.398 | 1.55e-07 | 4.22e-06 |
| ENSMUSG00000033287 | Kctd17 | protein\_coding | 15:78428564-78439303 (+) |  | -0.630 | 1.57e-07 | 4.25e-06 |
| ENSMUSG00000015968 | Cacna1d | protein\_coding | 14:30039939-30491455 (-) |  | 0.929 | 1.60e-07 | 4.33e-06 |
| ENSMUSG00000031672 | Got2 | protein\_coding | 8:95864134-95888547 (-) |  | -0.547 | 1.61e-07 | 4.34e-06 |
| ENSMUSG00000106734 | Gm20559 | lncRNA | 6:3333194-3346128 (-) |  | 0.883 | 1.62e-07 | 4.36e-06 |
| ENSMUSG00000017639 | Rab11fip4 | protein\_coding | 11:79591212-79698023 (+) |  | 0.688 | 1.66e-07 | 4.45e-06 |
| ENSMUSG00000053819 | Camk2d | protein\_coding | 3:126596302-126846326 (+) |  | -1.290 | 1.67e-07 | 4.48e-06 |
| ENSMUSG00000000489 | Pdgfb | protein\_coding | 15:79995874-80014977 (-) |  | 6.010 | 1.72e-07 | 4.60e-06 |
| ENSMUSG00000026365 | Cfh | protein\_coding | 1:140084708-140183764 (-) |  | -0.572 | 1.80e-07 | 4.81e-06 |
| ENSMUSG00000041608 | Entpd3 | protein\_coding | 9:120539818-120568327 (+) |  | -5.690 | 1.81e-07 | 4.83e-06 |
| ENSMUSG00000040213 | Kyat3 | protein\_coding | 3:142701051-142746870 (+) |  | -0.880 | 1.83e-07 | 4.86e-06 |
| ENSMUSG00000003541 | Ier3 | protein\_coding | 17:35821684-35822923 (+) |  | -1.270 | 1.87e-07 | 4.97e-06 |
| ENSMUSG00000071072 | Ptges3 | protein\_coding | 10:128058954-128077272 (+) |  | -0.522 | 1.88e-07 | 4.99e-06 |
| ENSMUSG00000109244 | Gm44751 | lncRNA | 7:88311478-88315864 (+) |  | -0.875 | 1.91e-07 | 5.04e-06 |
| ENSMUSG00000022021 | Diaph3 | protein\_coding | 14:86655367-87141235 (-) |  | -0.451 | 1.91e-07 | 5.04e-06 |
| ENSMUSG00000070305 | Mpzl3 | protein\_coding | 9:45055186-45077436 (+) |  | -1.160 | 1.92e-07 | 5.05e-06 |
| ENSMUSG00000023367 | Tmem176a | protein\_coding | 6:48840919-48847071 (+) |  | -0.735 | 1.93e-07 | 5.07e-06 |
| ENSMUSG00000070476 | Fam217b | protein\_coding | 2:178414524-178424428 (+) |  | -0.564 | 1.97e-07 | 5.18e-06 |
| ENSMUSG00000000386 | Mx1 | polymorphic\_pseudogene | 16:97447035-97462907 (-) |  | 0.768 | 2.01e-07 | 5.26e-06 |
| ENSMUSG00000057948 | Unc13d | protein\_coding | 11:116062095-116077961 (-) |  | 0.449 | 2.03e-07 | 5.30e-06 |
| ENSMUSG00000040848 | Sft2d2 | protein\_coding | 1:165174337-165194438 (-) |  | 0.421 | 2.08e-07 | 5.43e-06 |
| ENSMUSG00000024681 | Ms4a3 | protein\_coding | 19:11629496-11640851 (-) |  | -1.900 | 2.17e-07 | 5.65e-06 |
| ENSMUSG00000036138 | Acaa1a | protein\_coding | 9:119339676-119350299 (+) |  | -0.483 | 2.18e-07 | 5.65e-06 |
| ENSMUSG00000030126 | Tmcc1 | protein\_coding | 6:116018611-116193486 (-) |  | 0.385 | 2.18e-07 | 5.65e-06 |
| ENSMUSG00000018770 | Atp5g3 | protein\_coding | 2:73908447-73911326 (-) |  | -0.501 | 2.25e-07 | 5.83e-06 |
| ENSMUSG00000047250 | Ptgs1 | protein\_coding | 2:36230426-36252272 (+) |  | 1.060 | 2.27e-07 | 5.86e-06 |
| ENSMUSG00000027248 | Pdia3 | protein\_coding | 2:121413775-121438687 (+) |  | -0.490 | 2.34e-07 | 6.03e-06 |
| ENSMUSG00000020834 | Dhrs13 | protein\_coding | 11:78032280-78037866 (+) |  | -0.978 | 2.37e-07 | 6.10e-06 |
| ENSMUSG00000025225 | Nfkb2 | protein\_coding | 19:46304320-46312385 (+) |  | 0.461 | 2.38e-07 | 6.11e-06 |
| ENSMUSG00000051727 | Kctd14 | protein\_coding | 7:97451323-97459557 (+) |  | 1.330 | 2.40e-07 | 6.14e-06 |
| ENSMUSG00000035901 | Dennd5a | protein\_coding | 7:109893780-109960470 (-) |  | 0.322 | 2.47e-07 | 6.32e-06 |
| ENSMUSG00000034557 | Zfyve9 | protein\_coding | 4:108637466-108780798 (-) |  | 0.611 | 2.50e-07 | 6.37e-06 |
| ENSMUSG00000030089 | Slc41a3 | protein\_coding | 6:90604725-90646412 (+) |  | 0.742 | 2.55e-07 | 6.50e-06 |
| ENSMUSG00000020178 | Adora2a | protein\_coding | 10:75316877-75334784 (+) |  | 1.670 | 2.62e-07 | 6.66e-06 |
| ENSMUSG00000034471 | Caskin2 | protein\_coding | 11:115799183-115813639 (-) |  | -1.500 | 2.68e-07 | 6.79e-06 |
| ENSMUSG00000030148 | Clec4a2 | protein\_coding | 6:123106428-123143999 (+) |  | 0.627 | 2.83e-07 | 7.16e-06 |
| ENSMUSG00000022818 | Cyp2ab1 | protein\_coding | 16:20308387-20325404 (-) |  | 1.100 | 2.84e-07 | 7.17e-06 |
| ENSMUSG00000032279 | Idh3a | protein\_coding | 9:54586334-54604661 (+) |  | -0.615 | 2.88e-07 | 7.27e-06 |
| ENSMUSG00000026134 | Prim2 | protein\_coding | 1:33453810-33669795 (-) |  | -0.482 | 2.89e-07 | 7.28e-06 |
| ENSMUSG00000027533 | Fabp5 | protein\_coding | 3:10012548-10016607 (+) |  | 0.650 | 2.93e-07 | 7.37e-06 |
| ENSMUSG00000115338 | Pnp | protein\_coding | 14:50931082-50965237 (+) |  | -0.550 | 2.98e-07 | 7.48e-06 |
| ENSMUSG00000030287 | Itpr2 | protein\_coding | 6:146108299-146502223 (-) |  | 0.599 | 3.07e-07 | 7.67e-06 |
| ENSMUSG00000022540 | Rogdi | protein\_coding | 16:5008730-5013553 (-) |  | 0.714 | 3.08e-07 | 7.70e-06 |
| ENSMUSG00000027496 | Aurka | protein\_coding | 2:172356190-172370535 (-) |  | -0.405 | 3.11e-07 | 7.77e-06 |
| ENSMUSG00000004709 | Cd244a | protein\_coding | 1:171559193-171609746 (+) |  | -0.851 | 3.14e-07 | 7.82e-06 |
| ENSMUSG00000021831 | Ero1l | protein\_coding | 14:45283087-45318771 (-) |  | -0.529 | 3.26e-07 | 8.10e-06 |
| ENSMUSG00000025498 | Irf7 | protein\_coding | 7:141262706-141266481 (-) |  | 0.544 | 3.35e-07 | 8.31e-06 |
| ENSMUSG00000000530 | Acvrl1 | protein\_coding | 15:101128522-101145336 (+) |  | 0.522 | 3.38e-07 | 8.34e-06 |
| ENSMUSG00000011884 | Gltp | protein\_coding | 5:114669398-114690984 (-) |  | 0.398 | 3.38e-07 | 8.34e-06 |
| ENSMUSG00000048924 | Ccdc125 | protein\_coding | 13:100669717-100697240 (+) |  | 0.521 | 3.41e-07 | 8.41e-06 |
| ENSMUSG00000026547 | Tagln2 | protein\_coding | 1:172500047-172507380 (+) |  | -0.492 | 3.44e-07 | 8.47e-06 |
| ENSMUSG00000045193 | Cirbp | protein\_coding | 10:80165985-80172786 (+) |  | 0.557 | 3.47e-07 | 8.51e-06 |
| ENSMUSG00000029413 | Naaa | protein\_coding | 5:92257659-92278170 (-) |  | -0.367 | 3.61e-07 | 8.84e-06 |
| ENSMUSG00000037251 | Pomk | protein\_coding | 8:25980604-25994133 (-) |  | 0.797 | 3.62e-07 | 8.85e-06 |
| ENSMUSG00000021360 | Gcnt2 | protein\_coding | 13:40859754-40960892 (+) |  | -1.460 | 3.65e-07 | 8.92e-06 |
| ENSMUSG00000042817 | Flt3 | protein\_coding | 5:147330741-147400489 (-) |  | 1.070 | 3.67e-07 | 8.95e-06 |
| ENSMUSG00000003746 | Man1a | protein\_coding | 10:53904785-54076609 (-) |  | 0.522 | 3.72e-07 | 9.05e-06 |
| ENSMUSG00000006378 | Gcat | protein\_coding | 15:79030874-79038353 (+) |  | -1.350 | 3.85e-07 | 9.33e-06 |
| ENSMUSG00000010663 | Fads1 | protein\_coding | 19:10182888-10196877 (+) |  | -1.040 | 3.85e-07 | 9.33e-06 |
| ENSMUSG00000027715 | Ccna2 | protein\_coding | 3:36564865-36572150 (-) |  | -0.415 | 3.86e-07 | 9.33e-06 |
| ENSMUSG00000092572 | Serpinb10 | polymorphic\_pseudogene | 1:107529003-107549271 (+) |  | 0.834 | 3.95e-07 | 9.54e-06 |
| ENSMUSG00000039159 | Ube2h | protein\_coding | 6:30211289-30304539 (-) |  | -0.617 | 4.07e-07 | 9.80e-06 |
| ENSMUSG00000003072 | Atp5d | protein\_coding | 10:80138632-80145818 (+) |  | -0.423 | 4.15e-07 | 9.97e-06 |
| ENSMUSG00000024587 | Nars | protein\_coding | 18:64499647-64516652 (-) |  | -0.359 | 4.16e-07 | 9.99e-06 |
| ENSMUSG00000025875 | Tspan17 | protein\_coding | 13:54789377-54796776 (+) |  | 0.689 | 4.20e-07 | 1.01e-05 |
| ENSMUSG00000049971 | Glt1d1 | protein\_coding | 5:127632262-127709374 (+) |  | 1.810 | 4.25e-07 | 1.02e-05 |
| ENSMUSG00000028633 | Ctps | protein\_coding | 4:120539868-120570276 (-) |  | -0.656 | 4.27e-07 | 1.02e-05 |
| ENSMUSG00000058881 | Zfp516 | protein\_coding | 18:82910663-83005314 (+) |  | 0.482 | 4.27e-07 | 1.02e-05 |
| ENSMUSG00000066621 | Tecpr1 | protein\_coding | 5:144194442-144223615 (-) |  | 0.600 | 4.29e-07 | 1.02e-05 |
| ENSMUSG00000029217 | Tec | protein\_coding | 5:72755716-72868483 (-) |  | 0.559 | 4.33e-07 | 1.03e-05 |
| ENSMUSG00000037185 | Krt80 | protein\_coding | 15:101347444-101370162 (-) |  | 0.465 | 4.37e-07 | 1.04e-05 |
| ENSMUSG00000038175 | Mylip | protein\_coding | 13:45389742-45412022 (+) |  | 0.674 | 4.52e-07 | 1.07e-05 |
| ENSMUSG00000037242 | Clic4 | protein\_coding | 4:135213969-135272814 (-) |  | -0.367 | 4.53e-07 | 1.07e-05 |
| ENSMUSG00000018446 | C1qbp | protein\_coding | 11:70977836-70983026 (-) |  | -0.649 | 4.58e-07 | 1.08e-05 |
| ENSMUSG00000020914 | Top2a | protein\_coding | 11:98992943-99024189 (-) |  | -0.350 | 4.62e-07 | 1.09e-05 |
| ENSMUSG00000031821 | Gins2 | protein\_coding | 8:120578633-120589304 (-) |  | -0.634 | 4.63e-07 | 1.09e-05 |
| ENSMUSG00000022186 | Oxct1 | protein\_coding | 15:4026383-4155344 (+) |  | -0.474 | 4.63e-07 | 1.09e-05 |
| ENSMUSG00000056592 | Zfp658 | protein\_coding | 7:43562256-43575461 (+) |  | 0.827 | 4.69e-07 | 1.10e-05 |
| ENSMUSG00000034024 | Cct2 | protein\_coding | 10:117051001-117063814 (-) |  | -0.397 | 4.79e-07 | 1.12e-05 |
| ENSMUSG00000041362 | Shtn1 | protein\_coding | 19:58973356-59076100 (-) |  | -1.290 | 4.84e-07 | 1.13e-05 |
| ENSMUSG00000046808 | Atp10d | polymorphic\_pseudogene | 5:72203329-72298775 (+) |  | 0.632 | 4.97e-07 | 1.16e-05 |
| ENSMUSG00000036112 | Metap2 | protein\_coding | 10:93858489-93897093 (-) |  | -0.445 | 4.98e-07 | 1.16e-05 |
| ENSMUSG00000053411 | Cbx7 | protein\_coding | 15:79915807-79971119 (-) |  | 1.020 | 5.08e-07 | 1.18e-05 |
| ENSMUSG00000031373 | Car5b | protein\_coding | X:163976822-164027997 (-) |  | -1.800 | 5.09e-07 | 1.18e-05 |
| ENSMUSG00000036817 | Sun1 | protein\_coding | 5:139200637-139249840 (+) |  | 0.419 | 5.11e-07 | 1.18e-05 |
| ENSMUSG00000027698 | Nceh1 | protein\_coding | 3:27182965-27284608 (+) |  | 0.433 | 5.19e-07 | 1.20e-05 |
| ENSMUSG00000027323 | Rad51 | protein\_coding | 2:119112793-119147445 (+) |  | -0.576 | 5.27e-07 | 1.21e-05 |
| ENSMUSG00000020682 | Mmp28 | protein\_coding | 11:83440768-83463071 (-) |  | 1.030 | 5.27e-07 | 1.21e-05 |
| ENSMUSG00000030216 | Wbp11 | protein\_coding | 6:136813654-136828233 (-) |  | -0.451 | 5.28e-07 | 1.21e-05 |
| ENSMUSG00000027544 | Nfatc2 | protein\_coding | 2:168476410-168601657 (-) |  | 0.804 | 5.32e-07 | 1.22e-05 |
| ENSMUSG00000039770 | Ypel5 | protein\_coding | 17:72836453-72851195 (+) |  | 0.410 | 5.60e-07 | 1.28e-05 |
| ENSMUSG00000015745 | Plekho1 | protein\_coding | 3:95988429-95996001 (-) |  | 0.510 | 5.71e-07 | 1.30e-05 |
| ENSMUSG00000001440 | Kpnb1 | protein\_coding | 11:97159714-97187881 (-) |  | -0.405 | 5.72e-07 | 1.30e-05 |
| ENSMUSG00000109572 | Cfap99 | protein\_coding | 5:34288600-34327327 (+) |  | 2.060 | 5.76e-07 | 1.31e-05 |
| ENSMUSG00000052560 | Cpne8 | protein\_coding | 15:90487482-90679432 (-) |  | -4.200 | 5.83e-07 | 1.32e-05 |
| ENSMUSG00000022003 | Slc25a30 | protein\_coding | 14:75760117-75787037 (-) |  | -0.456 | 5.97e-07 | 1.35e-05 |
| ENSMUSG00000020638 | Cmpk2 | protein\_coding | 12:26469204-26479837 (+) |  | 0.473 | 6.00e-07 | 1.36e-05 |
| ENSMUSG00000007050 | Lsm2 | protein\_coding | 17:34981862-34985891 (+) |  | -0.564 | 6.08e-07 | 1.37e-05 |
| ENSMUSG00000001380 | Hars | protein\_coding | 18:36766528-36783205 (-) |  | -0.418 | 6.34e-07 | 1.43e-05 |
| ENSMUSG00000071203 | Naip5 | protein\_coding | 13:100211739-100246323 (-) |  | 0.475 | 6.50e-07 | 1.46e-05 |
| ENSMUSG00000026121 | Sema4c | protein\_coding | 1:36548639-36558349 (-) |  | 1.630 | 6.58e-07 | 1.48e-05 |
| ENSMUSG00000051329 | Nup160 | protein\_coding | 2:90677215-90736328 (+) |  | -0.439 | 6.60e-07 | 1.48e-05 |
| ENSMUSG00000017754 | Pltp | protein\_coding | 2:164839518-164857711 (-) |  | 1.350 | 6.63e-07 | 1.48e-05 |
| ENSMUSG00000019823 | Mical1 | protein\_coding | 10:41476314-41487032 (+) |  | 0.486 | 6.64e-07 | 1.48e-05 |
| ENSMUSG00000070407 | Hs3st3b1 | protein\_coding | 11:63885792-63922290 (-) |  | -2.070 | 6.71e-07 | 1.50e-05 |
| ENSMUSG00000029648 | Flt1 | protein\_coding | 5:147561604-147726011 (-) |  | 1.650 | 6.85e-07 | 1.53e-05 |
| ENSMUSG00000034667 | Xpot | protein\_coding | 10:121587380-121626332 (-) |  | -0.451 | 6.88e-07 | 1.53e-05 |
| ENSMUSG00000021591 | Glrx | protein\_coding | 13:75839868-75850154 (+) |  | -0.513 | 6.97e-07 | 1.55e-05 |
| ENSMUSG00000048779 | P2ry6 | protein\_coding | 7:100937630-100974649 (-) |  | 0.589 | 6.98e-07 | 1.55e-05 |
| ENSMUSG00000027435 | Cd93 | protein\_coding | 2:148436640-148443563 (-) |  | -0.721 | 7.18e-07 | 1.59e-05 |
| ENSMUSG00000021298 | Gpr132 | protein\_coding | 12:112850873-112868228 (-) |  | 0.491 | 7.36e-07 | 1.63e-05 |
| ENSMUSG00000030930 | Chst15 | protein\_coding | 7:132235780-132317228 (-) |  | 1.370 | 7.42e-07 | 1.63e-05 |
| ENSMUSG00000022391 | Rangap1 | protein\_coding | 15:81704248-81745530 (-) |  | -0.384 | 7.50e-07 | 1.65e-05 |
| ENSMUSG00000028657 | Ppt1 | protein\_coding | 4:122836242-122859175 (+) |  | -0.352 | 7.54e-07 | 1.66e-05 |
| ENSMUSG00000025612 | Bach1 | protein\_coding | 16:87698945-87733346 (+) |  | 0.350 | 8.32e-07 | 1.82e-05 |
| ENSMUSG00000027495 | Fam210b | protein\_coding | 2:172345565-172355749 (+) |  | 0.830 | 8.42e-07 | 1.84e-05 |
| ENSMUSG00000070868 | Skint3 | protein\_coding | 4:112232245-112300468 (+) |  | 1.660 | 8.88e-07 | 1.94e-05 |
| ENSMUSG00000054400 | Cklf | protein\_coding | 8:104250861-104264938 (+) |  | 0.559 | 8.91e-07 | 1.94e-05 |
| ENSMUSG00000050244 | Heatr1 | protein\_coding | 13:12395027-12440289 (+) |  | -0.472 | 9.00e-07 | 1.96e-05 |
| ENSMUSG00000062372 | Otof | protein\_coding | 5:30367062-30461932 (-) |  | 1.320 | 9.02e-07 | 1.96e-05 |
| ENSMUSG00000029657 | Hsph1 | protein\_coding | 5:149614287-149636376 (-) |  | -0.696 | 9.13e-07 | 1.98e-05 |
| ENSMUSG00000063229 | Ldha | protein\_coding | 7:46841475-46855627 (+) |  | -0.574 | 9.14e-07 | 1.98e-05 |
| ENSMUSG00000023067 | Cdkn1a | protein\_coding | 17:29090976-29100727 (+) |  | -0.591 | 9.26e-07 | 2.00e-05 |
| ENSMUSG00000032932 | Hspa13 | protein\_coding | 16:75745431-75767104 (-) |  | -0.384 | 9.27e-07 | 2.00e-05 |
| ENSMUSG00000068959 | Zfp619 | protein\_coding | 7:39517766-39540420 (+) |  | 0.804 | 9.47e-07 | 2.04e-05 |
| ENSMUSG00000045322 | Tlr9 | protein\_coding | 9:106222598-106226883 (+) |  | 0.536 | 9.49e-07 | 2.04e-05 |
| ENSMUSG00000025026 | Add3 | protein\_coding | 19:53140443-53247399 (+) |  | 0.307 | 9.60e-07 | 2.06e-05 |
| ENSMUSG00000089762 | Ier5l | protein\_coding | 2:30471537-30474219 (-) |  | 1.480 | 9.66e-07 | 2.07e-05 |
| ENSMUSG00000035704 | Alg8 | protein\_coding | 7:97371606-97392185 (+) |  | -0.558 | 9.99e-07 | 2.14e-05 |
| ENSMUSG00000002997 | Prkar2b | protein\_coding | 12:31958476-32061296 (-) |  | 0.391 | 1.01e-06 | 2.15e-05 |
| ENSMUSG00000041235 | Chd7 | protein\_coding | 4:8690406-8867659 (+) |  | -0.662 | 1.02e-06 | 2.17e-05 |
| ENSMUSG00000057789 | Bak1 | protein\_coding | 17:27019810-27029009 (-) |  | -0.461 | 1.02e-06 | 2.17e-05 |
| ENSMUSG00000038866 | Zcchc2 | protein\_coding | 1:105990406-106034074 (+) |  | 0.400 | 1.08e-06 | 2.29e-05 |
| ENSMUSG00000045094 | Arhgef37 | protein\_coding | 18:61491657-61536594 (-) |  | 0.378 | 1.08e-06 | 2.29e-05 |
| ENSMUSG00000049866 | Arl4c | protein\_coding | 1:88673125-88702221 (-) |  | 0.618 | 1.09e-06 | 2.31e-05 |
| ENSMUSG00000020547 | Bzw2 | protein\_coding | 12:36091835-36158080 (-) |  | -0.569 | 1.10e-06 | 2.33e-05 |
| ENSMUSG00000055013 | Agap1 | protein\_coding | 1:89454806-89897617 (+) |  | 0.739 | 1.10e-06 | 2.34e-05 |
| ENSMUSG00000049295 | Zfp219 | protein\_coding | 14:52006077-52020733 (-) |  | 0.776 | 1.12e-06 | 2.36e-05 |
| ENSMUSG00000036768 | Kif15 | protein\_coding | 9:122951046-123018733 (+) |  | -0.434 | 1.13e-06 | 2.38e-05 |
| ENSMUSG00000036764 | Dnajc12 | protein\_coding | 10:63382443-63410576 (+) |  | -1.140 | 1.14e-06 | 2.40e-05 |
| ENSMUSG00000048087 | Gm4737 | protein\_coding | 16:46152985-46155077 (-) |  | -0.865 | 1.18e-06 | 2.49e-05 |
| ENSMUSG00000006715 | Gmnn | protein\_coding | 13:24751845-24761923 (-) |  | -0.370 | 1.24e-06 | 2.60e-05 |
| ENSMUSG00000024948 | Map4k2 | protein\_coding | 19:6341135-6355615 (+) |  | 0.530 | 1.27e-06 | 2.65e-05 |
| ENSMUSG00000036304 | Zdhhc23 | protein\_coding | 16:43965033-43979791 (-) |  | -0.745 | 1.30e-06 | 2.72e-05 |
| ENSMUSG00000042029 | Ncapg2 | protein\_coding | 12:116405402-116463731 (+) |  | -0.440 | 1.30e-06 | 2.72e-05 |
| ENSMUSG00000059824 | Dbp | protein\_coding | 7:45705088-45710203 (+) |  | 1.410 | 1.31e-06 | 2.72e-05 |
| ENSMUSG00000029135 | Fosl2 | protein\_coding | 5:32135801-32157842 (+) |  | 0.442 | 1.32e-06 | 2.75e-05 |
| ENSMUSG00000028101 | Pias3 | protein\_coding | 3:96696384-96706070 (+) |  | 0.482 | 1.32e-06 | 2.75e-05 |
| ENSMUSG00000006442 | Srm | protein\_coding | 4:148591503-148594993 (+) |  | -0.640 | 1.33e-06 | 2.75e-05 |
| ENSMUSG00000038205 | Prkab2 | protein\_coding | 3:97658193-97673812 (+) |  | 0.668 | 1.33e-06 | 2.76e-05 |
| ENSMUSG00000025747 | Tyms | protein\_coding | 5:30058202-30073617 (-) |  | -0.531 | 1.34e-06 | 2.76e-05 |
| ENSMUSG00000026972 | Arrdc1 | protein\_coding | 2:24925352-24935252 (-) |  | 0.416 | 1.36e-06 | 2.80e-05 |
| ENSMUSG00000070730 | Rmdn3 | protein\_coding | 2:119137001-119157034 (-) |  | -0.481 | 1.37e-06 | 2.82e-05 |
| ENSMUSG00000020941 | Map3k14 | protein\_coding | 11:103219762-103267472 (-) |  | 0.468 | 1.42e-06 | 2.92e-05 |
| ENSMUSG00000028161 | Ppp3ca | protein\_coding | 3:136670124-136937727 (+) |  | 0.302 | 1.44e-06 | 2.97e-05 |
| ENSMUSG00000045176 | Borcs6 | protein\_coding | 11:69059717-69061578 (+) |  | 0.599 | 1.46e-06 | 3.00e-05 |
| ENSMUSG00000022673 | Mcm4 | protein\_coding | 16:15623897-15637400 (-) |  | -0.634 | 1.46e-06 | 3.00e-05 |
| ENSMUSG00000038271 | Iffo1 | protein\_coding | 6:125145241-125161782 (+) |  | 0.509 | 1.47e-06 | 3.01e-05 |
| ENSMUSG00000039697 | Ncoa7 | protein\_coding | 10:30628999-30803326 (-) |  | 0.436 | 1.49e-06 | 3.03e-05 |
| ENSMUSG00000106847 | Peg13 | lncRNA | 15:72805600-72810324 (-) |  | 0.883 | 1.51e-06 | 3.09e-05 |
| ENSMUSG00000033545 | Znrf1 | protein\_coding | 8:111536097-111626030 (+) |  | 0.388 | 1.52e-06 | 3.09e-05 |
| ENSMUSG00000034349 | Smc4 | protein\_coding | 3:69004738-69034623 (+) |  | -0.303 | 1.53e-06 | 3.11e-05 |
| ENSMUSG00000047879 | Usp14 | protein\_coding | 18:9993066-10045119 (-) |  | -0.434 | 1.54e-06 | 3.12e-05 |
| ENSMUSG00000016494 | Cd34 | protein\_coding | 1:194938819-194961279 (+) |  | 1.060 | 1.55e-06 | 3.13e-05 |
| ENSMUSG00000027615 | Hps3 | protein\_coding | 3:19995945-20035315 (-) |  | 0.462 | 1.55e-06 | 3.14e-05 |
| ENSMUSG00000054752 | Fsd1l | protein\_coding | 4:53631471-53707009 (+) |  | -0.826 | 1.58e-06 | 3.19e-05 |
| ENSMUSG00000019992 | Mtfr2 | protein\_coding | 10:20347770-20361304 (+) |  | -0.382 | 1.59e-06 | 3.20e-05 |
| ENSMUSG00000032915 | Adgre4 | protein\_coding | 17:55749984-55853662 (+) |  | 1.970 | 1.59e-06 | 3.20e-05 |
| ENSMUSG00000033047 | Eif3l | protein\_coding | 15:79075179-79094405 (+) |  | -0.363 | 1.61e-06 | 3.23e-05 |
| ENSMUSG00000021998 | Lcp1 | protein\_coding | 14:75131101-75230842 (+) |  | -0.335 | 1.61e-06 | 3.23e-05 |
| ENSMUSG00000024544 | Ldlrad4 | protein\_coding | 18:67933257-68268630 (+) |  | 1.430 | 1.61e-06 | 3.23e-05 |
| ENSMUSG00000046006 | Gapt | protein\_coding | 13:110352616-110357199 (-) |  | 0.401 | 1.63e-06 | 3.26e-05 |
| ENSMUSG00000039997 | Ifi203 | protein\_coding | 1:173920407-173942672 (-) |  | 0.518 | 1.65e-06 | 3.30e-05 |
| ENSMUSG00000006476 | Nsmf | protein\_coding | 2:25054355-25062881 (+) |  | 0.604 | 1.67e-06 | 3.34e-05 |
| ENSMUSG00000050377 | Il31ra | protein\_coding | 13:112519898-112594360 (-) |  | 1.140 | 1.68e-06 | 3.34e-05 |
| ENSMUSG00000113701 | B230303A05Rik | transcribed\_processed\_pseudogene | 13:15813548-16023423 (-) |  | -3.880 | 1.70e-06 | 3.37e-05 |
| ENSMUSG00000031493 | Ggn | protein\_coding | 7:29170210-29173976 (+) |  | -1.330 | 1.70e-06 | 3.37e-05 |
| ENSMUSG00000030493 | Faap24 | protein\_coding | 7:35392152-35396836 (-) |  | -0.670 | 1.73e-06 | 3.43e-05 |
| ENSMUSG00000029030 | Tprgl | protein\_coding | 4:154157485-154160666 (-) |  | 0.519 | 1.73e-06 | 3.43e-05 |
| ENSMUSG00000002668 | Dennd1c | protein\_coding | 17:57065905-57078514 (-) |  | 0.675 | 1.77e-06 | 3.51e-05 |
| ENSMUSG00000052798 | Nup107 | protein\_coding | 10:117750621-117792705 (-) |  | -0.368 | 1.79e-06 | 3.53e-05 |
| ENSMUSG00000033697 | Arhgap39 | protein\_coding | 15:76723985-76818170 (-) |  | 0.551 | 1.81e-06 | 3.57e-05 |
| ENSMUSG00000026180 | Cxcr2 | protein\_coding | 1:74153989-74161246 (+) |  | -1.900 | 1.82e-06 | 3.58e-05 |
| ENSMUSG00000004655 | Aqp1 | protein\_coding | 6:55336432-55348555 (+) |  | 2.360 | 1.83e-06 | 3.59e-05 |
| ENSMUSG00000022122 | Ednrb | protein\_coding | 14:103814625-103844402 (-) |  | -1.190 | 1.86e-06 | 3.64e-05 |
| ENSMUSG00000032123 | Dpagt1 | protein\_coding | 9:44326019-44333900 (+) |  | -0.438 | 1.86e-06 | 3.64e-05 |
| ENSMUSG00000047592 | Nxpe5 | protein\_coding | 5:138225898-138253363 (+) |  | -0.882 | 1.93e-06 | 3.77e-05 |
| ENSMUSG00000024187 | Fam234a | protein\_coding | 17:26211822-26244242 (-) |  | 0.546 | 1.97e-06 | 3.85e-05 |
| ENSMUSG00000001089 | Luzp1 | protein\_coding | 4:136469761-136554780 (+) |  | -0.535 | 2.00e-06 | 3.90e-05 |
| ENSMUSG00000053931 | Cnn3 | protein\_coding | 3:121426497-121458207 (+) |  | 1.140 | 2.03e-06 | 3.95e-05 |
| ENSMUSG00000047604 | Frat2 | protein\_coding | 19:41845972-41848132 (-) |  | 0.901 | 2.04e-06 | 3.97e-05 |
| ENSMUSG00000030745 | Il21r | protein\_coding | 7:125603429-125633570 (+) |  | -1.660 | 2.06e-06 | 4.00e-05 |
| ENSMUSG00000026434 | Nucks1 | protein\_coding | 1:131910534-131936321 (+) |  | -0.496 | 2.06e-06 | 4.00e-05 |
| ENSMUSG00000078942 | Naip6 | protein\_coding | 13:100281121-100317674 (-) |  | 0.560 | 2.08e-06 | 4.02e-05 |
| ENSMUSG00000030662 | Ipo5 | protein\_coding | 14:120911224-120947999 (+) |  | -0.486 | 2.12e-06 | 4.09e-05 |
| ENSMUSG00000039747 | Orai2 | protein\_coding | 5:136147459-136170713 (-) |  | 0.393 | 2.14e-06 | 4.12e-05 |
| ENSMUSG00000069793 | Slfn9 | protein\_coding | 11:82978390-82991830 (-) |  | -0.457 | 2.16e-06 | 4.17e-05 |
| ENSMUSG00000098112 | Bin2 | protein\_coding | 15:100641077-100669553 (-) |  | -0.285 | 2.23e-06 | 4.29e-05 |
| ENSMUSG00000046805 | Mpeg1 | protein\_coding | 19:12460779-12465283 (+) |  | 0.320 | 2.24e-06 | 4.30e-05 |
| ENSMUSG00000026594 | Ralgps2 | protein\_coding | 1:156804166-156939626 (-) |  | 0.786 | 2.27e-06 | 4.35e-05 |
| ENSMUSG00000022106 | Rcbtb2 | protein\_coding | 14:73123037-73207843 (+) |  | 0.481 | 2.27e-06 | 4.35e-05 |
| ENSMUSG00000074129 | Rpl13a | protein\_coding | 7:45125558-45128761 (-) |  | -0.608 | 2.28e-06 | 4.36e-05 |
| ENSMUSG00000036580 | Spg20 | protein\_coding | 3:55112108-55137322 (+) |  | 0.704 | 2.31e-06 | 4.42e-05 |
| ENSMUSG00000017146 | Brca1 | protein\_coding | 11:101488764-101551955 (-) |  | -0.576 | 2.40e-06 | 4.57e-05 |
| ENSMUSG00000005410 | Mcm5 | protein\_coding | 8:75109569-75128439 (+) |  | -0.492 | 2.43e-06 | 4.63e-05 |
| ENSMUSG00000056124 | B4galt6 | protein\_coding | 18:20684599-20746404 (-) |  | -1.450 | 2.45e-06 | 4.65e-05 |
| ENSMUSG00000021987 | Mtmr6 | protein\_coding | 14:60265228-60302370 (+) |  | 0.357 | 2.47e-06 | 4.69e-05 |
| ENSMUSG00000020680 | Taf15 | protein\_coding | 11:83473086-83506743 (+) |  | -0.440 | 2.51e-06 | 4.76e-05 |
| ENSMUSG00000045273 | Cenph | protein\_coding | 13:100759674-100775899 (-) |  | -0.523 | 2.52e-06 | 4.76e-05 |
| ENSMUSG00000022945 | Chaf1b | protein\_coding | 16:93883901-93906115 (+) |  | -0.533 | 2.55e-06 | 4.81e-05 |
| ENSMUSG00000026622 | Nek2 | protein\_coding | 1:191821444-191833050 (+) |  | -0.401 | 2.55e-06 | 4.81e-05 |
| ENSMUSG00000056069 | Otulinl | protein\_coding | 15:27655069-27681579 (-) |  | 0.407 | 2.55e-06 | 4.81e-05 |
| ENSMUSG00000043252 | Tmem64 | protein\_coding | 4:15265831-15286753 (+) |  | 0.760 | 2.56e-06 | 4.82e-05 |
| ENSMUSG00000015291 | Gdi1 | protein\_coding | X:74304998-74311862 (+) |  | 0.429 | 2.58e-06 | 4.85e-05 |
| ENSMUSG00000073147 | 5031425E22Rik | lncRNA | 5:23382308-23434269 (-) |  | 0.572 | 2.61e-06 | 4.89e-05 |
| ENSMUSG00000021048 | Mthfd1 | protein\_coding | 12:76255298-76319803 (+) |  | -0.633 | 2.62e-06 | 4.92e-05 |
| ENSMUSG00000000088 | Cox5a | protein\_coding | 9:57521274-57532426 (+) |  | -0.493 | 2.63e-06 | 4.92e-05 |
| ENSMUSG00000020402 | Vdac1 | protein\_coding | 11:52360860-52389397 (+) |  | -0.366 | 2.66e-06 | 4.96e-05 |
| ENSMUSG00000001986 | Gria3 | protein\_coding | X:41400854-41678601 (+) |  | -0.660 | 2.66e-06 | 4.97e-05 |
| ENSMUSG00000030729 | Pgm2l1 | protein\_coding | 7:100227394-100278868 (+) |  | 0.421 | 2.69e-06 | 5.00e-05 |
| ENSMUSG00000020785 | Camkk1 | protein\_coding | 11:73019008-73042073 (+) |  | -2.100 | 2.69e-06 | 5.00e-05 |
| ENSMUSG00000001525 | Tubb5 | protein\_coding | 17:35833921-35838306 (-) |  | -0.429 | 2.70e-06 | 5.02e-05 |
| ENSMUSG00000031960 | Aars | protein\_coding | 8:111033144-111057664 (+) |  | -0.428 | 2.78e-06 | 5.16e-05 |
| ENSMUSG00000071711 | Mpst | protein\_coding | 15:78406416-78414013 (+) |  | -0.456 | 2.83e-06 | 5.24e-05 |
| ENSMUSG00000041859 | Mcm3 | protein\_coding | 1:20802968-20820312 (-) |  | -0.565 | 2.84e-06 | 5.25e-05 |
| ENSMUSG00000028550 | Atg4c | protein\_coding | 4:99193934-99259787 (+) |  | 0.486 | 2.85e-06 | 5.27e-05 |
| ENSMUSG00000002910 | Arrdc2 | protein\_coding | 8:70835129-70839720 (-) |  | 0.628 | 2.89e-06 | 5.33e-05 |
| ENSMUSG00000030789 | Itgax | protein\_coding | 7:128129547-128150657 (+) |  | 1.360 | 2.93e-06 | 5.40e-05 |
| ENSMUSG00000082099 | Gm12013 | processed\_pseudogene | 11:17095490-17095644 (-) |  | 1.480 | 2.95e-06 | 5.43e-05 |
| ENSMUSG00000035493 | Tgfbi | protein\_coding | 13:56609523-56639562 (+) |  | -0.468 | 2.96e-06 | 5.43e-05 |
| ENSMUSG00000024663 | Rab3il1 | protein\_coding | 19:10001669-10038380 (+) |  | -0.920 | 2.96e-06 | 5.43e-05 |
| ENSMUSG00000030254 | Rad18 | protein\_coding | 6:112619850-112696686 (-) |  | -0.503 | 2.96e-06 | 5.43e-05 |
| ENSMUSG00000015176 | Nolc1 | protein\_coding | 19:46075863-46085530 (+) |  | -0.485 | 2.98e-06 | 5.45e-05 |
| ENSMUSG00000034334 | Fam151b | protein\_coding | 13:92449625-92484015 (-) |  | 0.941 | 3.02e-06 | 5.52e-05 |
| ENSMUSG00000028654 | Mycl | protein\_coding | 4:122995652-123002485 (+) |  | 2.420 | 3.03e-06 | 5.53e-05 |
| ENSMUSG00000028702 | Rad54l | protein\_coding | 4:116094264-116123690 (-) |  | -0.618 | 3.08e-06 | 5.61e-05 |
| ENSMUSG00000021131 | Erh | protein\_coding | 12:80634022-80644341 (-) |  | -0.521 | 3.09e-06 | 5.62e-05 |
| ENSMUSG00000070858 | Gm1673 | protein\_coding | 5:33983433-33985013 (+) |  | 2.310 | 3.17e-06 | 5.76e-05 |
| ENSMUSG00000026335 | Pam | protein\_coding | 1:97795114-98095646 (-) |  | -0.664 | 3.20e-06 | 5.80e-05 |
| ENSMUSG00000106743 | Gm42847 | TEC | 5:33990510-33993062 (-) |  | 1.350 | 3.22e-06 | 5.84e-05 |
| ENSMUSG00000075602 | Ly6a | protein\_coding | 15:74994877-74998031 (-) |  | -1.920 | 3.29e-06 | 5.95e-05 |
| ENSMUSG00000074656 | Eif2s2 | protein\_coding | 2:154871410-154892935 (-) |  | -0.433 | 3.31e-06 | 5.97e-05 |
| ENSMUSG00000059714 | Flot1 | protein\_coding | 17:35823230-35832791 (+) |  | -0.477 | 3.32e-06 | 5.99e-05 |
| ENSMUSG00000057666 | Gapdh | protein\_coding | 6:125161715-125166467 (-) |  | -0.523 | 3.47e-06 | 6.25e-05 |
| ENSMUSG00000022471 | Xrcc6 | protein\_coding | 15:81987835-82040085 (+) |  | -0.412 | 3.49e-06 | 6.27e-05 |
| ENSMUSG00000041840 | Haus1 | protein\_coding | 18:77757567-77773886 (-) |  | -0.633 | 3.50e-06 | 6.28e-05 |
| ENSMUSG00000027775 | Mfsd1 | protein\_coding | 3:67582741-67604237 (+) |  | -0.325 | 3.58e-06 | 6.41e-05 |
| ENSMUSG00000030681 | Mvp | protein\_coding | 7:126986860-127014621 (-) |  | -0.421 | 3.61e-06 | 6.46e-05 |
| ENSMUSG00000031875 | Cmtm3 | protein\_coding | 8:104339410-104347672 (+) |  | 0.514 | 3.63e-06 | 6.48e-05 |
| ENSMUSG00000024359 | Hspa9 | protein\_coding | 18:34937414-34954357 (-) |  | -0.485 | 3.63e-06 | 6.48e-05 |
| ENSMUSG00000027006 | Dnajc10 | protein\_coding | 2:80315466-80354043 (+) |  | -0.303 | 3.75e-06 | 6.68e-05 |
| ENSMUSG00000031497 | Tnfsf13b | protein\_coding | 8:10006467-10039072 (+) |  | 1.010 | 3.80e-06 | 6.77e-05 |
| ENSMUSG00000025223 | Ldb1 | protein\_coding | 19:46031570-46045214 (-) |  | 0.364 | 3.84e-06 | 6.84e-05 |
| ENSMUSG00000037321 | Tap1 | protein\_coding | 17:34187553-34197225 (+) |  | -0.408 | 3.88e-06 | 6.88e-05 |
| ENSMUSG00000029771 | Irf5 | protein\_coding | 6:29526625-29541871 (+) |  | 0.313 | 3.94e-06 | 6.99e-05 |
| ENSMUSG00000028943 | Espn | protein\_coding | 4:152120331-152152371 (-) |  | 1.480 | 3.95e-06 | 6.99e-05 |
| ENSMUSG00000046434 | Hnrnpa1 | protein\_coding | 15:103240432-103246692 (+) |  | -0.422 | 3.99e-06 | 7.06e-05 |
| ENSMUSG00000051235 | Gen1 | protein\_coding | 12:11238920-11265801 (-) |  | -0.365 | 4.00e-06 | 7.07e-05 |
| ENSMUSG00000018341 | Il12rb2 | protein\_coding | 6:67291318-67376188 (-) |  | 2.380 | 4.09e-06 | 7.21e-05 |
| ENSMUSG00000071068 | Treml2 | protein\_coding | 17:48299498-48312533 (+) |  | -0.506 | 4.11e-06 | 7.24e-05 |
| ENSMUSG00000018583 | G3bp1 | protein\_coding | 11:55469685-55504838 (+) |  | -0.346 | 4.12e-06 | 7.24e-05 |
| ENSMUSG00000046688 | Tifa | protein\_coding | 3:127789805-127832164 (+) |  | 0.466 | 4.13e-06 | 7.25e-05 |
| ENSMUSG00000020681 | Ace | protein\_coding | 11:105967945-105989964 (+) |  | 2.600 | 4.13e-06 | 7.25e-05 |
| ENSMUSG00000026458 | Ppfia4 | protein\_coding | 1:134296783-134332928 (-) |  | 0.491 | 4.26e-06 | 7.46e-05 |
| ENSMUSG00000028832 | Stmn1 | protein\_coding | 4:134468320-134473843 (+) |  | -0.382 | 4.28e-06 | 7.48e-05 |
| ENSMUSG00000056888 | Glipr1 | protein\_coding | 10:111985448-112002631 (-) |  | 0.521 | 4.30e-06 | 7.52e-05 |
| ENSMUSG00000096727 | Psmb9 | protein\_coding | 17:34181987-34187764 (-) |  | -0.475 | 4.33e-06 | 7.56e-05 |
| ENSMUSG00000067367 | Lyar | protein\_coding | 5:38220470-38234306 (+) |  | -0.500 | 4.34e-06 | 7.56e-05 |
| ENSMUSG00000020238 | Ncln | protein\_coding | 10:81486249-81496392 (-) |  | -0.356 | 4.44e-06 | 7.72e-05 |
| ENSMUSG00000042851 | Zc3h6 | protein\_coding | 2:128967402-129018563 (+) |  | 1.020 | 4.58e-06 | 7.96e-05 |
| ENSMUSG00000073400 | Trim10 | protein\_coding | 17:36869574-36877833 (+) |  | 2.680 | 4.59e-06 | 7.96e-05 |
| ENSMUSG00000024812 | Tjp2 | protein\_coding | 19:24094505-24225030 (-) |  | -0.780 | 4.62e-06 | 8.02e-05 |
| ENSMUSG00000053560 | Ier2 | protein\_coding | 8:84661331-84662854 (-) |  | 0.594 | 4.64e-06 | 8.04e-05 |
| ENSMUSG00000035683 | Melk | protein\_coding | 4:44300876-44364675 (+) |  | -0.345 | 4.67e-06 | 8.08e-05 |
| ENSMUSG00000072694 | 1500011B03Rik | protein\_coding | 5:114808196-114823468 (-) |  | 0.515 | 4.81e-06 | 8.30e-05 |
| ENSMUSG00000073421 | H2-Ab1 | protein\_coding | 17:34257689-34269419 (+) |  | 1.470 | 4.87e-06 | 8.39e-05 |
| ENSMUSG00000108621 | Gm37494 | lncRNA | 7:39544402-39580589 (+) |  | 0.509 | 4.95e-06 | 8.51e-05 |
| ENSMUSG00000052997 | Uba2 | protein\_coding | 7:34140688-34169599 (-) |  | -0.294 | 4.96e-06 | 8.53e-05 |
| ENSMUSG00000020328 | Nudcd2 | protein\_coding | 11:40733667-40740046 (+) |  | -0.457 | 5.02e-06 | 8.61e-05 |
| ENSMUSG00000053338 | Tarm1 | protein\_coding | 7:3486500-3502624 (-) |  | -0.618 | 5.18e-06 | 8.88e-05 |
| ENSMUSG00000060044 | Tmem26 | protein\_coding | 10:68723646-68782650 (+) |  | 2.100 | 5.19e-06 | 8.88e-05 |
| ENSMUSG00000029071 | Dvl1 | protein\_coding | 4:155847402-155859303 (+) |  | 0.397 | 5.22e-06 | 8.94e-05 |
| ENSMUSG00000026914 | Psmd14 | protein\_coding | 2:61711694-61800376 (+) |  | -0.379 | 5.29e-06 | 9.04e-05 |
| ENSMUSG00000117613 | Gm2629 | lncRNA | 18:15194782-15214696 (+) |  | 0.896 | 5.30e-06 | 9.04e-05 |
| ENSMUSG00000070354 | Evi2 | protein\_coding | 11:79513385-79530589 (-) |  | 0.550 | 5.40e-06 | 9.20e-05 |
| ENSMUSG00000024590 | Lmnb1 | protein\_coding | 18:56707813-56753424 (+) |  | -0.421 | 5.44e-06 | 9.25e-05 |
| ENSMUSG00000030761 | Myo7a | protein\_coding | 7:98051060-98119524 (-) |  | 0.469 | 5.48e-06 | 9.31e-05 |
| ENSMUSG00000104350 | Gm38244 | TEC | 3:106478283-106480868 (+) |  | -1.120 | 5.50e-06 | 9.34e-05 |
| ENSMUSG00000038650 | Rnh1 | protein\_coding | 7:141160326-141172857 (-) |  | -0.435 | 5.53e-06 | 9.37e-05 |
| ENSMUSG00000063160 | Numbl | protein\_coding | 7:27258433-27282144 (+) |  | 0.645 | 5.54e-06 | 9.38e-05 |
| ENSMUSG00000018819 | Lsp1 | protein\_coding | 7:142460809-142494867 (+) |  | 0.402 | 5.58e-06 | 9.43e-05 |
| ENSMUSG00000000753 | Serpinf1 | protein\_coding | 11:75409769-75422701 (-) |  | 0.827 | 5.60e-06 | 9.44e-05 |
| ENSMUSG00000035150 | Eif2s3x | protein\_coding | X:94188707-94212862 (-) |  | -0.456 | 5.60e-06 | 9.44e-05 |
| ENSMUSG00000019579 | Mydgf | protein\_coding | 17:56175744-56183920 (-) |  | -0.470 | 5.61e-06 | 9.45e-05 |
| ENSMUSG00000000628 | Hk2 | protein\_coding | 6:82725025-82774454 (-) |  | 0.451 | 5.67e-06 | 9.54e-05 |
| ENSMUSG00000038482 | Tfdp1 | protein\_coding | 8:13338751-13378448 (+) |  | -0.349 | 5.71e-06 | 9.59e-05 |
| ENSMUSG00000040711 | Sh3pxd2b | protein\_coding | 11:32347820-32428173 (+) |  | -0.575 | 5.73e-06 | 9.61e-05 |
| ENSMUSG00000048758 | Rpl29 | protein\_coding | 9:106429454-106431568 (+) |  | -0.371 | 5.79e-06 | 9.70e-05 |
| ENSMUSG00000064147 | Rab44 | protein\_coding | 17:29114145-29148980 (+) |  | 0.361 | 5.81e-06 | 9.73e-05 |
| ENSMUSG00000030750 | Nsmce1 | protein\_coding | 7:125467640-125491596 (-) |  | -0.472 | 5.90e-06 | 9.86e-05 |
| ENSMUSG00000025364 | Pa2g4 | protein\_coding | 10:128557766-128565987 (-) |  | -0.497 | 5.92e-06 | 9.88e-05 |
| ENSMUSG00000024769 | Cdc42bpg | protein\_coding | 19:6306456-6325652 (+) |  | 1.130 | 5.97e-06 | 9.94e-05 |
| ENSMUSG00000053063 | Clec12a | protein\_coding | 6:129342691-129365303 (+) |  | 0.603 | 6.00e-06 | 9.98e-05 |
| ENSMUSG00000027642 | Rpn2 | protein\_coding | 2:157279017-157326319 (+) |  | -0.477 | 6.15e-06 | 1.02e-04 |
| ENSMUSG00000026630 | Batf3 | protein\_coding | 1:191097847-191108945 (+) |  | 1.520 | 6.22e-06 | 1.03e-04 |
| ENSMUSG00000021365 | Nedd9 | protein\_coding | 13:41309581-41487362 (-) |  | 0.290 | 6.24e-06 | 1.04e-04 |
| ENSMUSG00000034023 | Fancd2 | protein\_coding | 6:113531682-113597017 (+) |  | -0.449 | 6.26e-06 | 1.04e-04 |
| ENSMUSG00000026281 | Dtymk | protein\_coding | 1:93792576-93801934 (-) |  | -0.362 | 6.33e-06 | 1.05e-04 |
| ENSMUSG00000049307 | Fut4 | protein\_coding | 9:14748320-14752393 (-) |  | 0.469 | 6.36e-06 | 1.05e-04 |
| ENSMUSG00000000303 | Cdh1 | protein\_coding | 8:106603351-106670246 (+) |  | -2.130 | 6.44e-06 | 1.06e-04 |
| ENSMUSG00000002870 | Mcm2 | protein\_coding | 6:88883474-88898780 (-) |  | -0.508 | 6.48e-06 | 1.07e-04 |
| ENSMUSG00000022967 | Ifnar1 | protein\_coding | 16:91485238-91507441 (+) |  | 0.306 | 6.73e-06 | 1.11e-04 |
| ENSMUSG00000055675 | Kbtbd11 | protein\_coding | 8:15011025-15033333 (+) |  | 0.522 | 6.73e-06 | 1.11e-04 |
| ENSMUSG00000031712 | Il15 | protein\_coding | 8:82331632-82403222 (-) |  | 0.711 | 6.74e-06 | 1.11e-04 |
| ENSMUSG00000035199 | Arl6ip5 | protein\_coding | 6:97210689-97233315 (+) |  | 0.342 | 6.86e-06 | 1.12e-04 |
| ENSMUSG00000035776 | Cd99l2 | protein\_coding | X:71420060-71492849 (-) |  | 0.530 | 6.97e-06 | 1.14e-04 |
| ENSMUSG00000002477 | Snrpd1 | protein\_coding | 18:10617775-10642079 (+) |  | -0.466 | 6.98e-06 | 1.14e-04 |
| ENSMUSG00000037706 | Cd81 | protein\_coding | 7:143052739-143067934 (+) |  | -1.230 | 7.08e-06 | 1.16e-04 |
| ENSMUSG00000024851 | Pitpnm1 | protein\_coding | 19:4099998-4113965 (+) |  | 0.358 | 7.09e-06 | 1.16e-04 |
| ENSMUSG00000019179 | Mdh2 | protein\_coding | 5:135778480-135790398 (+) |  | -0.512 | 7.10e-06 | 1.16e-04 |
| ENSMUSG00000040663 | Clcf1 | protein\_coding | 19:4214238-4223490 (+) |  | 1.630 | 7.10e-06 | 1.16e-04 |
| ENSMUSG00000041471 | Shld2 | protein\_coding | 14:34237033-34310493 (-) |  | 0.531 | 7.14e-06 | 1.16e-04 |
| ENSMUSG00000066975 | Cryba4 | protein\_coding | 5:112246493-112252518 (-) |  | 0.951 | 7.15e-06 | 1.16e-04 |
| ENSMUSG00000032691 | Nlrp3 | protein\_coding | 11:59541568-59566956 (+) |  | 0.448 | 7.21e-06 | 1.17e-04 |
| ENSMUSG00000058922 | Gm10052 | processed\_pseudogene | 9:123689233-123690192 (-) |  | -0.478 | 7.42e-06 | 1.20e-04 |
| ENSMUSG00000107761 | 2010008C14Rik | TEC | 6:125083437-125084029 (+) |  | 0.776 | 7.52e-06 | 1.22e-04 |
| ENSMUSG00000015501 | Hivep2 | protein\_coding | 10:13966075-14151374 (+) |  | -0.975 | 7.54e-06 | 1.22e-04 |
| ENSMUSG00000081534 | Slc48a1 | protein\_coding | 15:97778520-97792692 (+) |  | 0.387 | 7.58e-06 | 1.22e-04 |
| ENSMUSG00000039157 | Fam102a | protein\_coding | 2:32535332-32569756 (+) |  | 0.817 | 7.59e-06 | 1.22e-04 |
| ENSMUSG00000036594 | H2-Aa | protein\_coding | 17:34282744-34287823 (-) |  | 1.630 | 7.80e-06 | 1.25e-04 |
| ENSMUSG00000037104 | Socs5 | protein\_coding | 17:87107679-87137839 (+) |  | -0.676 | 7.81e-06 | 1.25e-04 |
| ENSMUSG00000028756 | Pink1 | protein\_coding | 4:138313409-138326307 (-) |  | 0.563 | 7.98e-06 | 1.28e-04 |
| ENSMUSG00000032417 | Rwdd2a | protein\_coding | 9:86571991-86574899 (+) |  | 0.767 | 8.04e-06 | 1.29e-04 |
| ENSMUSG00000031722 | Hp | protein\_coding | 8:109575128-109579172 (-) |  | -0.473 | 8.05e-06 | 1.29e-04 |
| ENSMUSG00000027469 | Tpx2 | protein\_coding | 2:152847964-152895321 (+) |  | -0.351 | 8.06e-06 | 1.29e-04 |
| ENSMUSG00000035953 | Pip4p1 | protein\_coding | 14:50926068-50930856 (-) |  | 0.392 | 8.20e-06 | 1.31e-04 |
| ENSMUSG00000022822 | Abcc5 | protein\_coding | 16:20331303-20426394 (-) |  | 0.417 | 8.21e-06 | 1.31e-04 |
| ENSMUSG00000028438 | Kif24 | protein\_coding | 4:41390745-41464887 (-) |  | -0.546 | 8.38e-06 | 1.33e-04 |
| ENSMUSG00000036825 | Ssx2ip | protein\_coding | 3:146404642-146440144 (+) |  | -0.583 | 8.57e-06 | 1.36e-04 |
| ENSMUSG00000022353 | Mtss1 | protein\_coding | 15:58941234-59082005 (-) |  | 1.490 | 8.59e-06 | 1.37e-04 |
| ENSMUSG00000034377 | Tulp4 | protein\_coding | 17:6106437-6251128 (+) |  | -0.686 | 8.90e-06 | 1.41e-04 |
| ENSMUSG00000006728 | Cdk4 | protein\_coding | 10:127063534-127067920 (+) |  | -0.459 | 8.98e-06 | 1.42e-04 |
| ENSMUSG00000021775 | Nr1d2 | protein\_coding | 14:18204054-18239127 (-) |  | 0.739 | 9.13e-06 | 1.44e-04 |
| ENSMUSG00000090164 | BC035044 | protein\_coding | 6:128849090-128891126 (-) |  | 0.540 | 9.28e-06 | 1.47e-04 |
| ENSMUSG00000020010 | Vnn3 | protein\_coding | 10:23851462-23869843 (+) |  | 1.240 | 9.39e-06 | 1.48e-04 |
| ENSMUSG00000025823 | Pdia4 | protein\_coding | 6:47796141-47813430 (-) |  | -0.468 | 9.41e-06 | 1.48e-04 |
| ENSMUSG00000017499 | Cdc6 | protein\_coding | 11:98907801-98923940 (+) |  | -0.737 | 9.52e-06 | 1.50e-04 |
| ENSMUSG00000024084 | Qpct | protein\_coding | 17:79051906-79090378 (+) |  | 0.663 | 9.61e-06 | 1.51e-04 |
| ENSMUSG00000018377 | Vezf1 | protein\_coding | 11:88068279-88084729 (+) |  | 0.388 | 9.66e-06 | 1.52e-04 |
| ENSMUSG00000020475 | Pgam2 | protein\_coding | 11:5801640-5803733 (-) |  | 0.559 | 9.72e-06 | 1.53e-04 |
| ENSMUSG00000020571 | Pdia6 | protein\_coding | 12:17266545-17284770 (+) |  | -0.451 | 9.81e-06 | 1.54e-04 |
| ENSMUSG00000040010 | Slc7a5 | protein\_coding | 8:121881150-121907694 (-) |  | -0.784 | 9.83e-06 | 1.54e-04 |
| ENSMUSG00000056999 | Ide | protein\_coding | 19:37268743-37337852 (-) |  | -0.590 | 9.84e-06 | 1.54e-04 |
| ENSMUSG00000045348 | Nyap1 | protein\_coding | 5:137729899-137741607 (-) |  | 1.640 | 9.84e-06 | 1.54e-04 |
| ENSMUSG00000035711 | Dok3 | protein\_coding | 13:55523231-55529296 (-) |  | 0.261 | 9.93e-06 | 1.55e-04 |
| ENSMUSG00000034987 | Hrh2 | protein\_coding | 13:54192129-54236180 (+) |  | -0.746 | 9.93e-06 | 1.55e-04 |
| ENSMUSG00000022844 | Pdia5 | protein\_coding | 16:35397312-35490873 (-) |  | -1.150 | 9.99e-06 | 1.55e-04 |
| ENSMUSG00000024399 | Ltb | protein\_coding | 17:35194439-35196320 (+) |  | 1.640 | 1.00e-05 | 1.55e-04 |
| ENSMUSG00000025868 | Higd2a | protein\_coding | 13:54590207-54591158 (+) |  | 0.473 | 1.00e-05 | 1.56e-04 |
| ENSMUSG00000031443 | F7 | protein\_coding | 8:13026034-13035809 (+) |  | -1.000 | 1.00e-05 | 1.56e-04 |
| ENSMUSG00000001588 | Acap1 | protein\_coding | 11:69881567-69895539 (-) |  | 0.497 | 1.01e-05 | 1.56e-04 |
| ENSMUSG00000053318 | Slamf8 | protein\_coding | 1:172581758-172590568 (-) |  | 0.981 | 1.01e-05 | 1.56e-04 |
| ENSMUSG00000062203 | Gspt1 | protein\_coding | 16:11219292-11254325 (-) |  | -0.339 | 1.03e-05 | 1.59e-04 |
| ENSMUSG00000021709 | Erbin | protein\_coding | 13:103818787-103920514 (-) |  | 0.311 | 1.03e-05 | 1.59e-04 |
| ENSMUSG00000031948 | Kars | protein\_coding | 8:111993443-112011323 (-) |  | -0.346 | 1.05e-05 | 1.62e-04 |
| ENSMUSG00000042784 | Muc1 | protein\_coding | 3:89229057-89233381 (+) |  | -2.540 | 1.06e-05 | 1.63e-04 |
| ENSMUSG00000008450 | Nutf2 | protein\_coding | 8:105860580-105879330 (+) |  | -0.506 | 1.07e-05 | 1.64e-04 |
| ENSMUSG00000023072 | Cep89 | protein\_coding | 7:35397035-35438689 (+) |  | -0.493 | 1.08e-05 | 1.65e-04 |
| ENSMUSG00000029587 | Zfp12 | protein\_coding | 5:143235163-143248834 (+) |  | 0.535 | 1.08e-05 | 1.66e-04 |
| ENSMUSG00000020023 | Tmcc3 | protein\_coding | 10:94311949-94590956 (+) |  | 1.210 | 1.09e-05 | 1.67e-04 |
| ENSMUSG00000110397 | Gm45540 | TEC | 8:56311304-56314909 (+) |  | 3.220 | 1.09e-05 | 1.67e-04 |
| ENSMUSG00000003581 | Rnf215 | protein\_coding | 11:4135202-4141172 (+) |  | 0.453 | 1.09e-05 | 1.67e-04 |
| ENSMUSG00000030695 | Aldoa | protein\_coding | 7:126795234-126800751 (-) |  | -0.446 | 1.11e-05 | 1.69e-04 |
| ENSMUSG00000001763 | Tspan33 | protein\_coding | 6:29694222-29718559 (+) |  | 1.220 | 1.11e-05 | 1.70e-04 |
| ENSMUSG00000023903 | Mmp25 | protein\_coding | 17:23628311-23645277 (-) |  | -1.040 | 1.13e-05 | 1.73e-04 |
| ENSMUSG00000060860 | Ube2s | protein\_coding | 7:4794546-4812590 (-) |  | -0.444 | 1.16e-05 | 1.76e-04 |
| ENSMUSG00000055491 | Pprc1 | protein\_coding | 19:46044886-46072915 (+) |  | -0.583 | 1.19e-05 | 1.82e-04 |
| ENSMUSG00000023572 | Ccndbp1 | protein\_coding | 2:121008403-121016904 (+) |  | 0.391 | 1.20e-05 | 1.82e-04 |
| ENSMUSG00000015340 | Cybb | protein\_coding | X:9435252-9487771 (-) |  | 0.337 | 1.20e-05 | 1.82e-04 |
| ENSMUSG00000041754 | Trem3 | protein\_coding | 17:48247777-48258841 (+) |  | -0.653 | 1.23e-05 | 1.86e-04 |
| ENSMUSG00000025134 | Alyref | protein\_coding | 11:120592121-120598365 (-) |  | -0.440 | 1.23e-05 | 1.86e-04 |
| ENSMUSG00000022667 | Cd200r1 | protein\_coding | 16:44765736-44794978 (+) |  | -0.654 | 1.24e-05 | 1.88e-04 |
| ENSMUSG00000063354 | Slc39a4 | protein\_coding | 15:76612383-76617384 (-) |  | 3.150 | 1.26e-05 | 1.90e-04 |
| ENSMUSG00000069539 | Scyl2 | protein\_coding | 10:89638721-89686285 (-) |  | -0.393 | 1.28e-05 | 1.94e-04 |
| ENSMUSG00000078515 | Ddi2 | protein\_coding | 4:141677549-141723419 (-) |  | -0.554 | 1.29e-05 | 1.94e-04 |
| ENSMUSG00000052310 | Slc39a1 | protein\_coding | 3:90248172-90253612 (+) |  | -0.382 | 1.30e-05 | 1.95e-04 |
| ENSMUSG00000039055 | Eme1 | protein\_coding | 11:94644996-94653964 (-) |  | -0.474 | 1.30e-05 | 1.95e-04 |
| ENSMUSG00000028634 | Hivep3 | protein\_coding | 4:119733784-120138045 (+) |  | 0.624 | 1.31e-05 | 1.96e-04 |
| ENSMUSG00000003134 | Tbc1d8 | protein\_coding | 1:39371492-39478755 (-) |  | 0.537 | 1.32e-05 | 1.98e-04 |
| ENSMUSG00000001228 | Uhrf1 | protein\_coding | 17:56303321-56323486 (+) |  | -0.551 | 1.32e-05 | 1.98e-04 |
| ENSMUSG00000021038 | Vipas39 | protein\_coding | 12:87238868-87266256 (-) |  | -0.418 | 1.33e-05 | 1.99e-04 |
| ENSMUSG00000015937 | Macroh2a1 | protein\_coding | 13:56073619-56136361 (-) |  | -0.350 | 1.34e-05 | 2.00e-04 |
| ENSMUSG00000032397 | Tipin | protein\_coding | 9:64281581-64305424 (+) |  | -0.421 | 1.34e-05 | 2.00e-04 |
| ENSMUSG00000033446 | Lpar6 | protein\_coding | 14:73237895-73243294 (+) |  | 0.621 | 1.35e-05 | 2.02e-04 |
| ENSMUSG00000026110 | Mgat4a | protein\_coding | 1:37439340-37541016 (-) |  | -0.802 | 1.40e-05 | 2.09e-04 |
| ENSMUSG00000037572 | Wdhd1 | protein\_coding | 14:47240944-47276857 (-) |  | -0.418 | 1.41e-05 | 2.09e-04 |
| ENSMUSG00000007891 | Ctsd | protein\_coding | 7:142375911-142388038 (-) |  | -0.432 | 1.42e-05 | 2.10e-04 |
| ENSMUSG00000024240 | Epc1 | protein\_coding | 18:6435951-6516108 (-) |  | 0.357 | 1.42e-05 | 2.10e-04 |
| ENSMUSG00000022769 | Sdf2l1 | protein\_coding | 16:17130138-17132383 (-) |  | -0.463 | 1.42e-05 | 2.10e-04 |
| ENSMUSG00000066278 | Vps37b | protein\_coding | 5:124004641-124032270 (-) |  | 0.448 | 1.42e-05 | 2.10e-04 |
| ENSMUSG00000052749 | Trim30b | protein\_coding | 7:104355382-104369884 (-) |  | -0.721 | 1.43e-05 | 2.12e-04 |
| ENSMUSG00000030088 | Aldh1l1 | protein\_coding | 6:90486427-90600203 (+) |  | -1.980 | 1.44e-05 | 2.12e-04 |
| ENSMUSG00000041921 | Metap1d | protein\_coding | 2:71453276-71525194 (+) |  | 0.605 | 1.45e-05 | 2.13e-04 |
| ENSMUSG00000015702 | Anxa9 | protein\_coding | 3:95296096-95307176 (-) |  | -0.789 | 1.47e-05 | 2.17e-04 |
| ENSMUSG00000026974 | Zmynd19 | protein\_coding | 2:24949792-24962075 (+) |  | -0.548 | 1.47e-05 | 2.17e-04 |
| ENSMUSG00000070639 | Lrrc8b | protein\_coding | 5:105415775-105490074 (+) |  | -0.385 | 1.48e-05 | 2.18e-04 |
| ENSMUSG00000066491 | Cox6c2 | processed\_pseudogene | 12:56373593-56373823 (-) |  | 1.370 | 1.49e-05 | 2.19e-04 |
| ENSMUSG00000022314 | Rad21 | protein\_coding | 15:51962240-51991747 (-) |  | -0.256 | 1.52e-05 | 2.23e-04 |
| ENSMUSG00000029994 | Anxa4 | protein\_coding | 6:86736840-86793584 (-) |  | 0.373 | 1.54e-05 | 2.25e-04 |
| ENSMUSG00000026669 | Mcm10 | protein\_coding | 2:4989714-5012791 (-) |  | -0.528 | 1.56e-05 | 2.28e-04 |
| ENSMUSG00000093622 | Gm20703 | lncRNA | 17:70834664-70836044 (+) |  | 1.770 | 1.56e-05 | 2.28e-04 |
| ENSMUSG00000011257 | Pabpc4 | protein\_coding | 4:123262351-123298925 (+) |  | -0.524 | 1.56e-05 | 2.28e-04 |
| ENSMUSG00000039682 | Lap3 | protein\_coding | 5:45493374-45512691 (+) |  | -0.507 | 1.62e-05 | 2.37e-04 |
| ENSMUSG00000017417 | Plxdc1 | protein\_coding | 11:97923238-97986444 (-) |  | 0.600 | 1.64e-05 | 2.38e-04 |
| ENSMUSG00000044456 | Rin3 | protein\_coding | 12:102283048-102390855 (+) |  | 0.454 | 1.64e-05 | 2.38e-04 |
| ENSMUSG00000021182 | Ccdc88c | protein\_coding | 12:100911523-101029056 (-) |  | 0.410 | 1.65e-05 | 2.40e-04 |
| ENSMUSG00000033703 | Fcsk | protein\_coding | 8:110882456-110902488 (-) |  | 0.609 | 1.66e-05 | 2.41e-04 |
| ENSMUSG00000101567 | Txn-ps1 | processed\_pseudogene | 1:44463923-44464231 (-) |  | 3.130 | 1.68e-05 | 2.43e-04 |
| ENSMUSG00000003154 | Foxj2 | protein\_coding | 6:122819914-122845366 (+) |  | 0.461 | 1.70e-05 | 2.46e-04 |
| ENSMUSG00000006058 | Snf8 | protein\_coding | 11:96034885-96047430 (+) |  | -0.296 | 1.70e-05 | 2.47e-04 |
| ENSMUSG00000001741 | Il16 | protein\_coding | 7:83642825-83745726 (-) |  | 0.345 | 1.71e-05 | 2.47e-04 |
| ENSMUSG00000037922 | Bank1 | protein\_coding | 3:136053363-136326066 (-) |  | 0.721 | 1.72e-05 | 2.48e-04 |
| ENSMUSG00000030108 | Slc6a13 | protein\_coding | 6:121300227-121337733 (+) |  | 2.120 | 1.72e-05 | 2.48e-04 |
| ENSMUSG00000042121 | Ssh1 | protein\_coding | 5:113937094-113993894 (-) |  | 0.513 | 1.76e-05 | 2.53e-04 |
| ENSMUSG00000025324 | Atp10a | protein\_coding | 7:58656166-58829420 (+) |  | -0.411 | 1.78e-05 | 2.56e-04 |
| ENSMUSG00000031432 | Prps1 | protein\_coding | X:140456613-140476140 (+) |  | -0.477 | 1.78e-05 | 2.56e-04 |
| ENSMUSG00000025403 | Shmt2 | protein\_coding | 10:127517123-127522444 (-) |  | -0.654 | 1.81e-05 | 2.59e-04 |
| ENSMUSG00000078606 | Gm4070 | protein\_coding | 7:105895139-105953967 (-) |  | -0.611 | 1.81e-05 | 2.59e-04 |
| ENSMUSG00000024899 | Papss2 | protein\_coding | 19:32595790-32667187 (+) |  | -0.497 | 1.81e-05 | 2.60e-04 |
| ENSMUSG00000032966 | Fkbp1a | protein\_coding | 2:151542483-151561692 (+) |  | -0.378 | 1.82e-05 | 2.60e-04 |
| ENSMUSG00000085465 | Gm15347 | lncRNA | 8:12860358-12877344 (-) |  | -1.870 | 1.84e-05 | 2.63e-04 |
| ENSMUSG00000041836 | Ptpre | protein\_coding | 7:135537481-135686293 (+) |  | 0.335 | 1.86e-05 | 2.66e-04 |
| ENSMUSG00000020130 | Tbc1d15 | protein\_coding | 10:115197872-115251467 (-) |  | -0.347 | 1.87e-05 | 2.67e-04 |
| ENSMUSG00000025130 | P4hb | protein\_coding | 11:120560298-120573253 (-) |  | -0.384 | 1.88e-05 | 2.68e-04 |
| ENSMUSG00000057133 | Chd6 | protein\_coding | 2:160946978-161109075 (-) |  | 0.529 | 1.90e-05 | 2.71e-04 |
| ENSMUSG00000025076 | Casp7 | protein\_coding | 19:56397129-56442344 (+) |  | -0.436 | 1.90e-05 | 2.71e-04 |
| ENSMUSG00000003814 | Calr | protein\_coding | 8:84841850-84846934 (-) |  | -0.618 | 1.91e-05 | 2.72e-04 |
| ENSMUSG00000036752 | Tubb4b | protein\_coding | 2:25222160-25224702 (-) |  | -0.365 | 1.94e-05 | 2.75e-04 |
| ENSMUSG00000038539 | Atf5 | protein\_coding | 7:44812256-44816658 (-) |  | -0.660 | 1.97e-05 | 2.80e-04 |
| ENSMUSG00000024921 | Smarca2 | protein\_coding | 19:26605050-26778322 (+) |  | 0.419 | 1.98e-05 | 2.80e-04 |
| ENSMUSG00000042978 | Sbk1 | protein\_coding | 7:126248862-126295016 (+) |  | 0.499 | 1.98e-05 | 2.80e-04 |
| ENSMUSG00000033949 | Trim36 | protein\_coding | 18:46165300-46212607 (-) |  | 0.578 | 1.98e-05 | 2.80e-04 |
| ENSMUSG00000023022 | Lima1 | protein\_coding | 15:99778470-99875456 (-) |  | 0.490 | 1.99e-05 | 2.81e-04 |
| ENSMUSG00000032375 | Aph1b | protein\_coding | 9:66775202-66795490 (-) |  | 0.694 | 2.00e-05 | 2.82e-04 |
| ENSMUSG00000022708 | Zbtb20 | protein\_coding | 16:42875881-43642602 (+) |  | 0.855 | 2.00e-05 | 2.82e-04 |
| ENSMUSG00000020642 | Rnf144a | protein\_coding | 12:26300964-26415254 (-) |  | 0.322 | 2.01e-05 | 2.82e-04 |
| ENSMUSG00000025227 | Mfsd13a | protein\_coding | 19:46341121-46375252 (+) |  | -0.597 | 2.03e-05 | 2.85e-04 |
| ENSMUSG00000042524 | Sun2 | protein\_coding | 15:79724070-79742536 (-) |  | 0.314 | 2.04e-05 | 2.87e-04 |
| ENSMUSG00000040521 | Tsfm | protein\_coding | 10:127011572-127030840 (-) |  | -0.468 | 2.06e-05 | 2.89e-04 |
| ENSMUSG00000022014 | Epsti1 | protein\_coding | 14:77904239-78002657 (+) |  | 0.490 | 2.08e-05 | 2.91e-04 |
| ENSMUSG00000021156 | Zmynd11 | protein\_coding | 13:9684833-9765330 (-) |  | 0.373 | 2.08e-05 | 2.91e-04 |
| ENSMUSG00000038545 | Cul7 | protein\_coding | 17:46650337-46664364 (+) |  | 0.793 | 2.08e-05 | 2.91e-04 |
| ENSMUSG00000027763 | Mbnl1 | protein\_coding | 3:60472830-60629750 (+) |  | 0.307 | 2.10e-05 | 2.94e-04 |
| ENSMUSG00000028010 | Gar1 | protein\_coding | 3:129824912-129831396 (-) |  | -0.523 | 2.11e-05 | 2.94e-04 |
| ENSMUSG00000027968 | Larp7 | protein\_coding | 3:127536714-127553349 (-) |  | -0.318 | 2.12e-05 | 2.95e-04 |
| ENSMUSG00000053835 | H2-T24 | protein\_coding | 17:36005695-36020560 (-) |  | 0.598 | 2.13e-05 | 2.96e-04 |
| ENSMUSG00000030602 | Pak4 | protein\_coding | 7:28558819-28598185 (-) |  | 0.431 | 2.17e-05 | 3.01e-04 |
| ENSMUSG00000032218 | Ccnb2 | protein\_coding | 9:70407692-70421547 (-) |  | -0.301 | 2.17e-05 | 3.02e-04 |
| ENSMUSG00000002658 | Gtf2f1 | protein\_coding | 17:57003405-57011288 (-) |  | -0.356 | 2.18e-05 | 3.03e-04 |
| ENSMUSG00000111171 | Gm47815 | lncRNA | 10:43034993-43050219 (+) |  | 0.993 | 2.23e-05 | 3.09e-04 |
| ENSMUSG00000020585 | Laptm4a | protein\_coding | 12:8921664-8938742 (+) |  | 0.415 | 2.23e-05 | 3.09e-04 |
| ENSMUSG00000026355 | Mcm6 | protein\_coding | 1:128331590-128359664 (-) |  | -0.501 | 2.23e-05 | 3.09e-04 |
| ENSMUSG00000031149 | Praf2 | protein\_coding | X:7728439-7731064 (+) |  | -1.030 | 2.26e-05 | 3.12e-04 |
| ENSMUSG00000029191 | Rfc1 | protein\_coding | 5:65261850-65335670 (-) |  | -0.351 | 2.28e-05 | 3.14e-04 |
| ENSMUSG00000114608 | Gm36161 | lncRNA | 13:120010390-120018931 (+) |  | 0.550 | 2.31e-05 | 3.18e-04 |
| ENSMUSG00000002835 | Chaf1a | protein\_coding | 17:56040439-56072289 (+) |  | -0.502 | 2.32e-05 | 3.19e-04 |
| ENSMUSG00000076441 | Ass1 | protein\_coding | 2:31470207-31520672 (+) |  | -0.509 | 2.32e-05 | 3.19e-04 |
| ENSMUSG00000023944 | Hsp90ab1 | protein\_coding | 17:45567775-45573271 (-) |  | -0.489 | 2.34e-05 | 3.21e-04 |
| ENSMUSG00000020368 | Canx | protein\_coding | 11:50293961-50325673 (-) |  | -0.378 | 2.34e-05 | 3.21e-04 |
| ENSMUSG00000032939 | Nup93 | protein\_coding | 8:94214564-94317227 (+) |  | -0.419 | 2.35e-05 | 3.22e-04 |
| ENSMUSG00000039478 | Micu3 | protein\_coding | 8:40307458-40386308 (+) |  | 1.310 | 2.36e-05 | 3.23e-04 |
| ENSMUSG00000028333 | Anp32b | protein\_coding | 4:46450902-46472657 (+) |  | -0.426 | 2.42e-05 | 3.31e-04 |
| ENSMUSG00000025393 | Atp5b | protein\_coding | 10:128083273-128090391 (+) |  | -0.410 | 2.44e-05 | 3.34e-04 |
| ENSMUSG00000032477 | Cdc25a | protein\_coding | 9:109875579-109893895 (+) |  | -0.497 | 2.47e-05 | 3.38e-04 |
| ENSMUSG00000003779 | Kif20a | protein\_coding | 18:34624613-34633277 (+) |  | -0.346 | 2.48e-05 | 3.38e-04 |
| ENSMUSG00000024007 | Ppil1 | protein\_coding | 17:29250803-29264186 (-) |  | -0.526 | 2.49e-05 | 3.38e-04 |
| ENSMUSG00000040263 | Klhdc4 | protein\_coding | 8:121796313-121829569 (-) |  | -0.364 | 2.50e-05 | 3.40e-04 |
| ENSMUSG00000051682 | Treml4 | protein\_coding | 17:48264295-48275360 (+) |  | 1.170 | 2.51e-05 | 3.42e-04 |
| ENSMUSG00000032010 | Usp2 | protein\_coding | 9:44067021-44095627 (+) |  | -0.848 | 2.52e-05 | 3.42e-04 |
| ENSMUSG00000026688 | Mgst3 | protein\_coding | 1:167371966-167393841 (-) |  | -0.597 | 2.53e-05 | 3.44e-04 |
| ENSMUSG00000029804 | Herc3 | protein\_coding | 6:58831465-58920398 (+) |  | 0.480 | 2.55e-05 | 3.46e-04 |
| ENSMUSG00000022246 | Rai14 | protein\_coding | 15:10568969-10714624 (-) |  | -0.822 | 2.59e-05 | 3.50e-04 |
| ENSMUSG00000029299 | Abcg3 | protein\_coding | 5:104935057-104982718 (-) |  | 1.230 | 2.60e-05 | 3.51e-04 |
| ENSMUSG00000078521 | Aunip | protein\_coding | 4:134510999-134523927 (+) |  | -0.651 | 2.61e-05 | 3.53e-04 |
| ENSMUSG00000070544 | Top1 | protein\_coding | 2:160645888-160722764 (+) |  | -0.297 | 2.62e-05 | 3.54e-04 |
| ENSMUSG00000027854 | Sike1 | protein\_coding | 3:102995708-103008459 (+) |  | 0.310 | 2.65e-05 | 3.56e-04 |
| ENSMUSG00000040746 | Rnf167 | protein\_coding | 11:70647235-70651421 (+) |  | 0.411 | 2.66e-05 | 3.58e-04 |
| ENSMUSG00000034570 | Inpp5j | protein\_coding | 11:3494375-3504821 (-) |  | -0.712 | 2.67e-05 | 3.60e-04 |
| ENSMUSG00000004864 | Mapk13 | protein\_coding | 17:28769297-28780233 (+) |  | -0.787 | 2.68e-05 | 3.60e-04 |
| ENSMUSG00000026646 | Suv39h2 | protein\_coding | 2:3455815-3475031 (-) |  | -0.657 | 2.68e-05 | 3.60e-04 |
| ENSMUSG00000037822 | Smim14 | protein\_coding | 5:65446844-65537184 (-) |  | 0.367 | 2.71e-05 | 3.63e-04 |
| ENSMUSG00000004552 | Ctse | protein\_coding | 1:131638306-131675505 (+) |  | 0.481 | 2.71e-05 | 3.63e-04 |
| ENSMUSG00000087129 | Gm16316 | lncRNA | 2:163685004-163692279 (-) |  | 1.050 | 2.71e-05 | 3.63e-04 |
| ENSMUSG00000030357 | Fkbp4 | protein\_coding | 6:128429735-128438677 (-) |  | -0.480 | 2.72e-05 | 3.64e-04 |
| ENSMUSG00000024640 | Psat1 | protein\_coding | 19:15904678-15947337 (-) |  | -0.396 | 2.73e-05 | 3.65e-04 |
| ENSMUSG00000091575 | 2010016I18Rik | lncRNA | 3:106481982-106485913 (-) |  | -0.930 | 2.77e-05 | 3.69e-04 |
| ENSMUSG00000040907 | Atp1a3 | protein\_coding | 7:24978167-25005958 (-) |  | 0.433 | 2.78e-05 | 3.71e-04 |
| ENSMUSG00000022817 | Itgb5 | protein\_coding | 16:33829665-33949338 (+) |  | -0.444 | 2.81e-05 | 3.74e-04 |
| ENSMUSG00000028613 | Lrp8 | protein\_coding | 4:107801869-107876840 (+) |  | -0.621 | 2.82e-05 | 3.75e-04 |
| ENSMUSG00000081723 | Gm15931 | unprocessed\_pseudogene | 7:4274189-4282645 (+) |  | 0.873 | 2.83e-05 | 3.76e-04 |
| ENSMUSG00000028671 | Gale | protein\_coding | 4:135963727-135968178 (+) |  | -0.511 | 2.84e-05 | 3.77e-04 |
| ENSMUSG00000042747 | Krtcap2 | protein\_coding | 3:89245966-89249906 (+) |  | -0.410 | 2.88e-05 | 3.81e-04 |
| ENSMUSG00000005034 | Prkacb | protein\_coding | 3:146729574-146812990 (-) |  | 0.300 | 2.91e-05 | 3.85e-04 |
| ENSMUSG00000031749 | St3gal2 | protein\_coding | 8:110919922-110972480 (+) |  | 0.496 | 2.91e-05 | 3.85e-04 |
| ENSMUSG00000036928 | Stag3 | protein\_coding | 5:138280240-138312393 (+) |  | 0.674 | 2.93e-05 | 3.87e-04 |
| ENSMUSG00000017221 | Psmd3 | protein\_coding | 11:98682554-98695979 (+) |  | -0.406 | 3.02e-05 | 3.99e-04 |
| ENSMUSG00000053716 | Dusp7 | protein\_coding | 9:106368632-106375724 (+) |  | 0.527 | 3.03e-05 | 3.99e-04 |
| ENSMUSG00000066861 | Oas1g | protein\_coding | 5:120876142-120887613 (-) |  | -1.110 | 3.03e-05 | 4.00e-04 |
| ENSMUSG00000000751 | Rpa1 | protein\_coding | 11:75298166-75348324 (-) |  | -0.387 | 3.05e-05 | 4.01e-04 |
| ENSMUSG00000020474 | Polm | protein\_coding | 11:5827860-5838016 (-) |  | 0.560 | 3.06e-05 | 4.02e-04 |
| ENSMUSG00000006585 | Cdt1 | protein\_coding | 8:122568015-122573554 (+) |  | -0.472 | 3.06e-05 | 4.02e-04 |
| ENSMUSG00000044338 | Aplnr | protein\_coding | 2:85136225-85139923 (+) |  | 1.230 | 3.08e-05 | 4.04e-04 |
| ENSMUSG00000002017 | Fam98a | protein\_coding | 17:75537086-75551946 (-) |  | -0.409 | 3.11e-05 | 4.07e-04 |
| ENSMUSG00000028885 | Smpdl3b | protein\_coding | 4:132732966-132757252 (-) |  | 0.966 | 3.14e-05 | 4.11e-04 |
| ENSMUSG00000037151 | Lrrc20 | protein\_coding | 10:61475801-61582791 (+) |  | 0.609 | 3.14e-05 | 4.11e-04 |
| ENSMUSG00000018848 | Rars | protein\_coding | 11:35808381-35834506 (-) |  | -0.350 | 3.16e-05 | 4.13e-04 |
| ENSMUSG00000021474 | Sfxn1 | protein\_coding | 13:54071869-54108342 (+) |  | -0.566 | 3.18e-05 | 4.15e-04 |
| ENSMUSG00000038070 | Cntln | protein\_coding | 4:84884309-85131921 (+) |  | 0.415 | 3.21e-05 | 4.19e-04 |
| ENSMUSG00000022070 | Bora | protein\_coding | 14:99046222-99074540 (+) |  | -0.414 | 3.22e-05 | 4.20e-04 |
| ENSMUSG00000024986 | Hhex | protein\_coding | 19:37434810-37440731 (+) |  | 0.359 | 3.23e-05 | 4.20e-04 |
| ENSMUSG00000042046 | Dstyk | protein\_coding | 1:132417555-132466958 (+) |  | 0.459 | 3.24e-05 | 4.21e-04 |
| ENSMUSG00000033705 | Stard9 | protein\_coding | 2:120629121-120731895 (+) |  | 0.522 | 3.27e-05 | 4.24e-04 |
| ENSMUSG00000018340 | Anxa6 | protein\_coding | 11:54979108-55033445 (-) |  | 0.322 | 3.31e-05 | 4.30e-04 |
| ENSMUSG00000060550 | H2-Q7 | protein\_coding | 17:35439155-35443773 (+) |  | -0.934 | 3.32e-05 | 4.31e-04 |
| ENSMUSG00000020798 | Spns3 | protein\_coding | 11:72494919-72550506 (-) |  | 0.918 | 3.33e-05 | 4.32e-04 |
| ENSMUSG00000031176 | Dynlt3 | protein\_coding | X:9654267-9663003 (-) |  | 0.611 | 3.34e-05 | 4.32e-04 |
| ENSMUSG00000002058 | Unc119 | protein\_coding | 11:78343482-78349164 (+) |  | 0.544 | 3.34e-05 | 4.32e-04 |
| ENSMUSG00000026020 | Nop58 | protein\_coding | 1:59684971-59719044 (+) |  | -0.459 | 3.35e-05 | 4.32e-04 |
| ENSMUSG00000016206 | H2-M3 | protein\_coding | 17:37270220-37274484 (+) |  | -0.387 | 3.37e-05 | 4.34e-04 |
| ENSMUSG00000030007 | Cct7 | protein\_coding | 6:85451514-85468475 (+) |  | -0.292 | 3.37e-05 | 4.34e-04 |
| ENSMUSG00000027997 | Casp6 | protein\_coding | 3:129901425-129914103 (+) |  | -0.570 | 3.39e-05 | 4.36e-04 |
| ENSMUSG00000044072 | Eml6 | protein\_coding | 11:29743048-30026033 (-) |  | 1.160 | 3.41e-05 | 4.38e-04 |
| ENSMUSG00000024193 | Phf1 | protein\_coding | 17:26933052-26937908 (+) |  | 0.510 | 3.42e-05 | 4.40e-04 |
| ENSMUSG00000033933 | Vhl | protein\_coding | 6:113623959-113631633 (+) |  | 0.394 | 3.43e-05 | 4.41e-04 |
| ENSMUSG00000029166 | Mapre3 | protein\_coding | 5:30814641-30866106 (+) |  | 1.540 | 3.44e-05 | 4.41e-04 |
| ENSMUSG00000111118 | Gm6545 | processed\_pseudogene | 19:12528755-12530540 (-) |  | 0.866 | 3.52e-05 | 4.50e-04 |
| ENSMUSG00000040658 | Dnph1 | protein\_coding | 17:46496711-46499624 (+) |  | -0.739 | 3.55e-05 | 4.54e-04 |
| ENSMUSG00000000290 | Itgb2 | protein\_coding | 10:77530252-77565708 (+) |  | 0.315 | 3.59e-05 | 4.59e-04 |
| ENSMUSG00000030149 | Klrk1 | protein\_coding | 6:129610323-129623864 (-) |  | 1.080 | 3.59e-05 | 4.59e-04 |
| ENSMUSG00000036898 | Zfp157 | protein\_coding | 5:138441468-138460694 (+) |  | 0.582 | 3.64e-05 | 4.64e-04 |
| ENSMUSG00000042369 | Rbm45 | protein\_coding | 2:76369984-76383768 (+) |  | -0.383 | 3.64e-05 | 4.64e-04 |
| ENSMUSG00000035198 | Tubg1 | protein\_coding | 11:101119938-101126419 (+) |  | -0.482 | 3.66e-05 | 4.66e-04 |
| ENSMUSG00000046687 | Gm5424 | processed\_pseudogene | 10:62071123-62072362 (+) |  | -0.478 | 3.67e-05 | 4.66e-04 |
| ENSMUSG00000043740 | B430306N03Rik | protein\_coding | 17:48316141-48327024 (+) |  | 0.433 | 3.72e-05 | 4.73e-04 |
| ENSMUSG00000025889 | Snca | protein\_coding | 6:60731575-60829855 (-) |  | 2.040 | 3.73e-05 | 4.74e-04 |
| ENSMUSG00000025792 | Slc25a10 | protein\_coding | 11:120491840-120499187 (+) |  | -0.377 | 3.76e-05 | 4.76e-04 |
| ENSMUSG00000029063 | Nadk | protein\_coding | 4:155562378-155591001 (+) |  | 0.245 | 3.76e-05 | 4.76e-04 |
| ENSMUSG00000028018 | Gstcd | protein\_coding | 3:132981752-133092033 (-) |  | -0.609 | 3.78e-05 | 4.79e-04 |
| ENSMUSG00000002205 | Vrk3 | protein\_coding | 7:44748413-44777515 (+) |  | -0.329 | 3.79e-05 | 4.79e-04 |
| ENSMUSG00000037148 | Arhgap10 | protein\_coding | 8:77250366-77517953 (-) |  | -0.747 | 3.79e-05 | 4.80e-04 |
| ENSMUSG00000020048 | Hsp90b1 | protein\_coding | 10:86690209-86705509 (-) |  | -0.381 | 3.81e-05 | 4.81e-04 |
| ENSMUSG00000040354 | Mars | protein\_coding | 10:127296221-127311786 (-) |  | -0.367 | 3.84e-05 | 4.84e-04 |
| ENSMUSG00000015747 | Vps45 | protein\_coding | 3:95999832-96058466 (-) |  | -0.465 | 3.85e-05 | 4.84e-04 |
| ENSMUSG00000056612 | Ppp1r14b | protein\_coding | 19:6974968-6977324 (+) |  | -0.427 | 3.86e-05 | 4.86e-04 |
| ENSMUSG00000024982 | Zdhhc6 | protein\_coding | 19:55271291-55316032 (-) |  | -0.377 | 3.88e-05 | 4.87e-04 |
| ENSMUSG00000026864 | Hspa5 | protein\_coding | 2:34771970-34777547 (+) |  | -0.441 | 3.90e-05 | 4.90e-04 |
| ENSMUSG00000056394 | Lig1 | protein\_coding | 7:13277283-13311433 (+) |  | -0.514 | 3.91e-05 | 4.90e-04 |
| ENSMUSG00000027555 | Car13 | protein\_coding | 3:14641727-14663002 (+) |  | -0.964 | 3.93e-05 | 4.93e-04 |
| ENSMUSG00000035783 | Acta2 | protein\_coding | 19:34241090-34255590 (-) |  | -0.635 | 3.94e-05 | 4.94e-04 |
| ENSMUSG00000043207 | Zmpste24 | protein\_coding | 4:121059237-121098241 (-) |  | -0.388 | 3.95e-05 | 4.94e-04 |
| ENSMUSG00000028873 | Cdca8 | protein\_coding | 4:124918465-124939311 (-) |  | -0.312 | 3.96e-05 | 4.95e-04 |
| ENSMUSG00000015889 | Lta4h | protein\_coding | 10:93453411-93484875 (+) |  | -0.361 | 3.97e-05 | 4.95e-04 |
| ENSMUSG00000026683 | Nuf2 | protein\_coding | 1:169497934-169531464 (-) |  | -0.297 | 3.98e-05 | 4.97e-04 |
| ENSMUSG00000049848 | Ceacam19 | protein\_coding | 7:19875742-19887965 (-) |  | 1.140 | 3.99e-05 | 4.97e-04 |
| ENSMUSG00000020653 | Klf11 | protein\_coding | 12:24651274-24662789 (+) |  | 0.620 | 4.02e-05 | 5.01e-04 |
| ENSMUSG00000022102 | Dok2 | protein\_coding | 14:70766036-70778495 (+) |  | -0.758 | 4.03e-05 | 5.02e-04 |
| ENSMUSG00000030711 | Sult1a1 | protein\_coding | 7:126672865-126676432 (-) |  | 1.540 | 4.07e-05 | 5.07e-04 |
| ENSMUSG00000039236 | Isg20 | protein\_coding | 7:78913424-78920396 (+) |  | 0.881 | 4.11e-05 | 5.10e-04 |
| ENSMUSG00000019970 | Sgk1 | protein\_coding | 10:21882184-21999903 (+) |  | 0.477 | 4.12e-05 | 5.10e-04 |
| ENSMUSG00000046668 | Cxxc5 | protein\_coding | 18:35829397-35861688 (+) |  | 1.530 | 4.12e-05 | 5.10e-04 |
| ENSMUSG00000052609 | Plekhg3 | protein\_coding | 12:76530891-76580488 (+) |  | 0.341 | 4.12e-05 | 5.10e-04 |
| ENSMUSG00000042595 | Fam199x | protein\_coding | X:137049594-137082503 (+) |  | 0.598 | 4.13e-05 | 5.11e-04 |
| ENSMUSG00000032507 | Fbxl2 | protein\_coding | 9:113963637-114046191 (-) |  | -0.525 | 4.13e-05 | 5.11e-04 |
| ENSMUSG00000069892 | 9930111J21Rik2 | protein\_coding | 11:49015874-49051242 (-) |  | 0.431 | 4.19e-05 | 5.18e-04 |
| ENSMUSG00000020358 | Hnrnpab | protein\_coding | 11:51600100-51606847 (-) |  | -0.340 | 4.24e-05 | 5.23e-04 |
| ENSMUSG00000005575 | Ube2m | protein\_coding | 7:13035120-13038275 (-) |  | -0.295 | 4.27e-05 | 5.26e-04 |
| ENSMUSG00000021814 | Anxa7 | protein\_coding | 14:20455260-20480133 (-) |  | -0.300 | 4.30e-05 | 5.29e-04 |
| ENSMUSG00000021687 | Scamp1 | protein\_coding | 13:94201310-94285857 (-) |  | -0.505 | 4.31e-05 | 5.30e-04 |
| ENSMUSG00000041406 | BC055324 | protein\_coding | 1:163945993-163994796 (-) |  | -0.554 | 4.32e-05 | 5.30e-04 |
| ENSMUSG00000030805 | Stx4a | protein\_coding | 7:127824294-127849019 (+) |  | 0.332 | 4.32e-05 | 5.31e-04 |
| ENSMUSG00000030189 | Ybx3 | protein\_coding | 6:131364855-131388476 (-) |  | -0.398 | 4.35e-05 | 5.33e-04 |
| ENSMUSG00000032333 | Stoml1 | protein\_coding | 9:58253164-58262520 (+) |  | 0.572 | 4.40e-05 | 5.39e-04 |
| ENSMUSG00000030168 | Adipor2 | protein\_coding | 6:119353150-119417704 (-) |  | 0.309 | 4.42e-05 | 5.41e-04 |
| ENSMUSG00000011267 | Zfp296 | protein\_coding | 7:19577287-19580656 (+) |  | 0.711 | 4.43e-05 | 5.42e-04 |
| ENSMUSG00000017830 | Dhx58 | protein\_coding | 11:100694884-100704271 (-) |  | 0.411 | 4.45e-05 | 5.44e-04 |
| ENSMUSG00000028525 | Pde4b | protein\_coding | 4:102087543-102607259 (+) |  | 0.532 | 4.47e-05 | 5.45e-04 |
| ENSMUSG00000036561 | Ppp6r2 | protein\_coding | 15:89211553-89287010 (+) |  | 0.477 | 4.47e-05 | 5.45e-04 |
| ENSMUSG00000053617 | Sh3pxd2a | protein\_coding | 19:47260174-47464401 (-) |  | 0.819 | 4.50e-05 | 5.48e-04 |
| ENSMUSG00000052299 | Ltn1 | protein\_coding | 16:87376651-87432612 (-) |  | -0.357 | 4.50e-05 | 5.48e-04 |
| ENSMUSG00000047547 | Cltb | protein\_coding | 13:54592401-54611344 (-) |  | -0.466 | 4.53e-05 | 5.51e-04 |
| ENSMUSG00000020413 | Hus1 | protein\_coding | 11:8993137-9011191 (-) |  | -0.433 | 4.58e-05 | 5.56e-04 |
| ENSMUSG00000031266 | Gla | protein\_coding | X:134588149-134601125 (-) |  | -0.470 | 4.61e-05 | 5.59e-04 |
| ENSMUSG00000036533 | Cdc42ep3 | protein\_coding | 17:79333727-79355091 (-) |  | 0.369 | 4.61e-05 | 5.59e-04 |
| ENSMUSG00000028973 | Abcb8 | protein\_coding | 5:24393663-24410054 (+) |  | -0.450 | 4.62e-05 | 5.60e-04 |
| ENSMUSG00000039633 | Lonrf1 | protein\_coding | 8:36216064-36249516 (-) |  | 0.467 | 4.63e-05 | 5.60e-04 |
| ENSMUSG00000047454 | Gphn | protein\_coding | 12:78226379-78684772 (+) |  | -0.521 | 4.67e-05 | 5.65e-04 |
| ENSMUSG00000032381 | Ciao2a | protein\_coding | 9:66126611-66138955 (+) |  | -0.354 | 4.67e-05 | 5.65e-04 |
| ENSMUSG00000035539 | Ccdc180 | protein\_coding | 4:45890303-45950774 (+) |  | 0.818 | 4.73e-05 | 5.71e-04 |
| ENSMUSG00000003402 | Prkcsh | protein\_coding | 9:22002806-22014222 (+) |  | -0.391 | 4.76e-05 | 5.74e-04 |
| ENSMUSG00000089942 | Pira2 | protein\_coding | 7:3836812-3845051 (-) |  | 0.515 | 4.79e-05 | 5.77e-04 |
| ENSMUSG00000031422 | Morf4l2 | protein\_coding | X:136732942-136743690 (-) |  | -0.354 | 4.84e-05 | 5.82e-04 |
| ENSMUSG00000046223 | Plaur | protein\_coding | 7:24462484-24475968 (+) |  | 0.370 | 4.86e-05 | 5.85e-04 |
| ENSMUSG00000037337 | Map4k1 | protein\_coding | 7:28982050-29003279 (+) |  | -0.340 | 4.88e-05 | 5.87e-04 |
| ENSMUSG00000021040 | Slirp | protein\_coding | 12:87443896-87452206 (+) |  | -0.654 | 4.96e-05 | 5.95e-04 |
| ENSMUSG00000013629 | Cad | protein\_coding | 5:31054780-31078479 (+) |  | -0.694 | 5.00e-05 | 5.99e-04 |
| ENSMUSG00000039585 | Myo9a | protein\_coding | 9:59750896-59928866 (+) |  | 0.405 | 5.00e-05 | 5.99e-04 |
| ENSMUSG00000022419 | Deptor | protein\_coding | 15:55112317-55259271 (+) |  | 0.377 | 5.02e-05 | 6.01e-04 |
| ENSMUSG00000002733 | Plekha3 | protein\_coding | 2:76675281-76696828 (+) |  | -0.499 | 5.03e-05 | 6.01e-04 |
| ENSMUSG00000021451 | Sema4d | protein\_coding | 13:51685529-51793747 (-) |  | 0.460 | 5.17e-05 | 6.17e-04 |
| ENSMUSG00000105987 | AI506816 | lncRNA | 5:23698296-23712667 (-) |  | -1.180 | 5.19e-05 | 6.20e-04 |
| ENSMUSG00000022881 | Rfc4 | protein\_coding | 16:23113943-23127737 (-) |  | -0.467 | 5.25e-05 | 6.26e-04 |
| ENSMUSG00000071866 | Ppia | protein\_coding | 11:6415443-6419817 (+) |  | -0.412 | 5.26e-05 | 6.27e-04 |
| ENSMUSG00000004642 | Slbp | protein\_coding | 5:33634952-33652574 (-) |  | -0.295 | 5.27e-05 | 6.28e-04 |
| ENSMUSG00000010755 | Cars | protein\_coding | 7:143557230-143600090 (-) |  | -0.397 | 5.32e-05 | 6.32e-04 |
| ENSMUSG00000062075 | Lmnb2 | protein\_coding | 10:80901203-80918245 (-) |  | -0.331 | 5.34e-05 | 6.35e-04 |
| ENSMUSG00000023452 | Pisd | protein\_coding | 5:32736301-32785646 (-) |  | 0.336 | 5.36e-05 | 6.36e-04 |
| ENSMUSG00000018750 | Zbtb4 | protein\_coding | 11:69765912-69784023 (+) |  | 0.994 | 5.38e-05 | 6.38e-04 |
| ENSMUSG00000003348 | Mob3a | protein\_coding | 10:80685253-80701977 (-) |  | 0.296 | 5.38e-05 | 6.38e-04 |
| ENSMUSG00000031634 | Ufsp2 | protein\_coding | 8:45975528-45996958 (+) |  | -0.385 | 5.39e-05 | 6.38e-04 |
| ENSMUSG00000032336 | Nptn | protein\_coding | 9:58582240-58657955 (+) |  | 0.270 | 5.42e-05 | 6.41e-04 |
| ENSMUSG00000040451 | Sgms1 | protein\_coding | 19:32122727-32389714 (-) |  | 0.400 | 5.42e-05 | 6.41e-04 |
| ENSMUSG00000024665 | Fads2 | protein\_coding | 19:10062765-10101746 (-) |  | -1.540 | 5.54e-05 | 6.55e-04 |
| ENSMUSG00000063952 | Brpf3 | protein\_coding | 17:28801090-28839949 (+) |  | 0.557 | 5.57e-05 | 6.57e-04 |
| ENSMUSG00000045838 | Ccdc9b | protein\_coding | 2:118754158-118762661 (-) |  | 2.010 | 5.61e-05 | 6.61e-04 |
| ENSMUSG00000018068 | Ints2 | protein\_coding | 11:86210681-86257575 (-) |  | -0.451 | 5.66e-05 | 6.67e-04 |
| ENSMUSG00000024066 | Xdh | protein\_coding | 17:73883895-73950196 (-) |  | -0.342 | 5.73e-05 | 6.74e-04 |
| ENSMUSG00000022913 | Psmg1 | protein\_coding | 16:95979933-95990960 (-) |  | -0.552 | 5.73e-05 | 6.74e-04 |
| ENSMUSG00000069919 | Hba-a1 | protein\_coding | 11:32283511-32284465 (+) |  | 2.040 | 5.80e-05 | 6.81e-04 |
| ENSMUSG00000081684 | Rps2-ps13 | processed\_pseudogene | X:52898588-52899469 (+) |  | -0.600 | 5.88e-05 | 6.90e-04 |
| ENSMUSG00000049090 | Zadh2 | protein\_coding | 18:84088063-84097528 (+) |  | 0.373 | 6.01e-05 | 7.04e-04 |
| ENSMUSG00000024424 | Ttc39c | protein\_coding | 18:12599896-12738863 (+) |  | -0.399 | 6.05e-05 | 7.08e-04 |
| ENSMUSG00000047264 | Zfp358 | protein\_coding | 8:3493138-3497208 (+) |  | 0.698 | 6.09e-05 | 7.13e-04 |
| ENSMUSG00000037548 | H2-DMb2 | protein\_coding | 17:34143307-34151555 (+) |  | 0.686 | 6.10e-05 | 7.13e-04 |
| ENSMUSG00000004668 | Abca13 | protein\_coding | 11:9191942-9684259 (+) |  | -1.800 | 6.11e-05 | 7.13e-04 |
| ENSMUSG00000006498 | Ptbp1 | protein\_coding | 10:79854427-79864771 (+) |  | -0.332 | 6.17e-05 | 7.20e-04 |
| ENSMUSG00000058216 | Gstp3 | protein\_coding | 19:4057477-4059569 (-) |  | 2.100 | 6.19e-05 | 7.21e-04 |
| ENSMUSG00000015305 | Sash1 | protein\_coding | 10:8722219-8886070 (-) |  | 0.620 | 6.27e-05 | 7.30e-04 |
| ENSMUSG00000062867 | Impdh2 | protein\_coding | 9:108560286-108565584 (+) |  | -0.459 | 6.28e-05 | 7.31e-04 |
| ENSMUSG00000017716 | Birc5 | protein\_coding | 11:117849251-117855743 (+) |  | -0.311 | 6.28e-05 | 7.31e-04 |
| ENSMUSG00000027699 | Ect2 | protein\_coding | 3:27097222-27153878 (-) |  | -0.297 | 6.29e-05 | 7.31e-04 |
| ENSMUSG00000027108 | Ola1 | protein\_coding | 2:73092801-73218924 (-) |  | -0.334 | 6.43e-05 | 7.46e-04 |
| ENSMUSG00000008373 | Prpf31 | protein\_coding | 7:3629985-3642486 (+) |  | -0.345 | 6.44e-05 | 7.47e-04 |
| ENSMUSG00000048329 | Mfsd6l | protein\_coding | 11:68556186-68558245 (+) |  | 0.918 | 6.46e-05 | 7.48e-04 |
| ENSMUSG00000028134 | Ptbp2 | protein\_coding | 3:119718742-119784466 (-) |  | 0.505 | 6.48e-05 | 7.50e-04 |
| ENSMUSG00000026034 | Clk1 | protein\_coding | 1:58410189-58424066 (-) |  | 0.601 | 6.53e-05 | 7.55e-04 |
| ENSMUSG00000021508 | Cxcl14 | protein\_coding | 13:56288643-56296551 (-) |  | 2.890 | 6.58e-05 | 7.60e-04 |
| ENSMUSG00000042606 | Hirip3 | protein\_coding | 7:126861972-126865377 (+) |  | -0.374 | 6.59e-05 | 7.60e-04 |
| ENSMUSG00000020125 | Elane | protein\_coding | 10:79886247-79888215 (+) |  | -0.988 | 6.60e-05 | 7.61e-04 |
| ENSMUSG00000029730 | Mcm7 | protein\_coding | 5:138164583-138172422 (-) |  | -0.414 | 6.64e-05 | 7.66e-04 |
| ENSMUSG00000040430 | Pitpnc1 | protein\_coding | 11:107207892-107470699 (-) |  | 0.469 | 6.81e-05 | 7.84e-04 |
| ENSMUSG00000053965 | Pde5a | protein\_coding | 3:122728947-122859374 (+) |  | -0.791 | 6.82e-05 | 7.84e-04 |
| ENSMUSG00000061175 | Fnip2 | protein\_coding | 3:79455974-79567796 (-) |  | -0.511 | 6.92e-05 | 7.95e-04 |
| ENSMUSG00000041132 | N4bp2l1 | protein\_coding | 5:150571644-150597188 (-) |  | 0.437 | 7.03e-05 | 8.07e-04 |
| ENSMUSG00000020577 | Tspan13 | protein\_coding | 12:36014557-36042500 (-) |  | -0.583 | 7.06e-05 | 8.10e-04 |
| ENSMUSG00000025613 | Cct8 | protein\_coding | 16:87483326-87495873 (-) |  | -0.324 | 7.07e-05 | 8.11e-04 |
| ENSMUSG00000005667 | Mthfd2 | protein\_coding | 6:83305691-83325908 (-) |  | -0.377 | 7.13e-05 | 8.17e-04 |
| ENSMUSG00000030397 | Mark4 | protein\_coding | 7:19424775-19458821 (-) |  | 0.360 | 7.18e-05 | 8.21e-04 |
| ENSMUSG00000029390 | Tmed2 | protein\_coding | 5:124540695-124550506 (+) |  | -0.322 | 7.20e-05 | 8.23e-04 |
| ENSMUSG00000052656 | Rnf103 | protein\_coding | 6:71493894-71510881 (+) |  | 0.401 | 7.27e-05 | 8.30e-04 |
| ENSMUSG00000073910 | Mob3b | protein\_coding | 4:34949074-35157484 (-) |  | 0.432 | 7.31e-05 | 8.34e-04 |
| ENSMUSG00000039086 | Ss18l1 | protein\_coding | 2:180042509-180070201 (+) |  | 0.781 | 7.31e-05 | 8.34e-04 |
| ENSMUSG00000006360 | Crip1 | protein\_coding | 12:113146316-113153879 (+) |  | 0.466 | 7.34e-05 | 8.36e-04 |
| ENSMUSG00000009575 | Cbx5 | protein\_coding | 15:103191544-103239816 (-) |  | -0.449 | 7.35e-05 | 8.37e-04 |
| ENSMUSG00000002985 | Apoe | protein\_coding | 7:19696109-19699188 (-) |  | 1.570 | 7.41e-05 | 8.43e-04 |
| ENSMUSG00000107215 | Gm43197 | TEC | 6:3336772-3339354 (-) |  | 0.946 | 7.42e-05 | 8.43e-04 |
| ENSMUSG00000022214 | Dcaf11 | protein\_coding | 14:55560006-55570065 (+) |  | 0.320 | 7.44e-05 | 8.44e-04 |
| ENSMUSG00000045664 | Cdc42ep2 | protein\_coding | 19:5915636-5924816 (-) |  | 0.845 | 7.44e-05 | 8.44e-04 |
| ENSMUSG00000027589 | Pcmtd2 | protein\_coding | 2:181837854-181857461 (+) |  | 0.434 | 7.54e-05 | 8.54e-04 |
| ENSMUSG00000025212 | Sfxn3 | protein\_coding | 19:45047503-45056383 (+) |  | 0.415 | 7.60e-05 | 8.61e-04 |
| ENSMUSG00000058355 | Abce1 | protein\_coding | 8:79683462-79711740 (-) |  | -0.344 | 7.62e-05 | 8.62e-04 |
| ENSMUSG00000005981 | Trap1 | protein\_coding | 16:4039971-4077827 (-) |  | -0.328 | 7.68e-05 | 8.67e-04 |
| ENSMUSG00000026811 | St6galnac6 | protein\_coding | 2:32599709-32620806 (+) |  | 0.384 | 7.68e-05 | 8.67e-04 |
| ENSMUSG00000039231 | Suv39h1 | protein\_coding | X:8061171-8074760 (-) |  | -0.348 | 7.70e-05 | 8.69e-04 |
| ENSMUSG00000032959 | Pebp1 | protein\_coding | 5:117282654-117287625 (-) |  | -0.441 | 7.76e-05 | 8.74e-04 |
| ENSMUSG00000029571 | Tmem106b | protein\_coding | 6:13069759-13089269 (+) |  | 0.418 | 7.85e-05 | 8.84e-04 |
| ENSMUSG00000060586 | H2-Eb1 | protein\_coding | 17:34305867-34316674 (+) |  | 1.580 | 7.87e-05 | 8.85e-04 |
| ENSMUSG00000051839 | Gypa | protein\_coding | 8:80493781-80510542 (+) |  | 2.280 | 7.88e-05 | 8.86e-04 |
| ENSMUSG00000037295 | Ldlrap1 | protein\_coding | 4:134741554-134768024 (-) |  | 0.409 | 8.01e-05 | 9.00e-04 |
| ENSMUSG00000029478 | Ncor2 | protein\_coding | 5:125017153-125179219 (-) |  | 0.429 | 8.02e-05 | 9.00e-04 |
| ENSMUSG00000028784 | Spocd1 | protein\_coding | 4:129929249-129957115 (+) |  | 2.210 | 8.10e-05 | 9.08e-04 |
| ENSMUSG00000107352 | Gm43660 | lncRNA | 5:31560812-31569072 (-) |  | 0.663 | 8.16e-05 | 9.14e-04 |
| ENSMUSG00000073705 | Cenps | protein\_coding | 4:149127121-149137629 (-) |  | -0.554 | 8.17e-05 | 9.15e-04 |
| ENSMUSG00000038759 | Nup205 | protein\_coding | 6:35177421-35247596 (+) |  | -0.365 | 8.19e-05 | 9.17e-04 |
| ENSMUSG00000042500 | Ago4 | protein\_coding | 4:126489541-126533472 (-) |  | 0.597 | 8.33e-05 | 9.31e-04 |
| ENSMUSG00000029836 | Cbx3 | protein\_coding | 6:51470360-51483704 (+) |  | -0.308 | 8.33e-05 | 9.31e-04 |
| ENSMUSG00000000594 | Gm2a | protein\_coding | 11:55098115-55113029 (+) |  | 0.257 | 8.40e-05 | 9.38e-04 |
| ENSMUSG00000024909 | Efemp2 | protein\_coding | 19:5473954-5482517 (+) |  | -1.350 | 8.43e-05 | 9.39e-04 |
| ENSMUSG00000051397 | Tacstd2 | protein\_coding | 6:67534062-67535796 (-) |  | -2.040 | 8.46e-05 | 9.42e-04 |
| ENSMUSG00000018189 | Uchl5 | protein\_coding | 1:143777272-143807466 (+) |  | -0.400 | 8.51e-05 | 9.47e-04 |
| ENSMUSG00000022018 | Rgcc | protein\_coding | 14:79288756-79301645 (-) |  | -1.600 | 8.52e-05 | 9.47e-04 |
| ENSMUSG00000106825 | 2510016D11Rik | TEC | 5:120552055-120553039 (-) |  | -0.561 | 8.54e-05 | 9.49e-04 |
| ENSMUSG00000026229 | Psmd1 | protein\_coding | 1:86064387-86139151 (+) |  | -0.365 | 8.57e-05 | 9.51e-04 |
| ENSMUSG00000114822 | Gm4813 | processed\_pseudogene | 13:81029103-81029901 (-) |  | 3.550 | 8.59e-05 | 9.53e-04 |
| ENSMUSG00000022270 | Retreg1 | protein\_coding | 15:25843180-25973687 (+) |  | -0.611 | 8.66e-05 | 9.60e-04 |
| ENSMUSG00000027562 | Car2 | protein\_coding | 3:14886273-14900770 (+) |  | 1.490 | 8.72e-05 | 9.66e-04 |
| ENSMUSG00000073676 | Hspe1 | protein\_coding | 1:55088132-55091307 (+) |  | -0.489 | 8.74e-05 | 9.67e-04 |
| ENSMUSG00000026094 | Stk17b | protein\_coding | 1:53755506-53785224 (-) |  | 0.465 | 8.75e-05 | 9.68e-04 |
| ENSMUSG00000058794 | Nfe2 | protein\_coding | 15:103248212-103258403 (-) |  | 0.355 | 8.80e-05 | 9.71e-04 |
| ENSMUSG00000037313 | Tacc3 | protein\_coding | 5:33658128-33678995 (+) |  | -0.322 | 8.85e-05 | 9.76e-04 |
| ENSMUSG00000072082 | Ccnf | protein\_coding | 17:24222198-24251484 (-) |  | -0.335 | 8.86e-05 | 9.76e-04 |
| ENSMUSG00000023272 | Creld2 | protein\_coding | 15:88819646-88826683 (+) |  | -0.454 | 8.86e-05 | 9.76e-04 |
| ENSMUSG00000022255 | Mtdh | protein\_coding | 15:34082694-34145624 (+) |  | 0.429 | 8.98e-05 | 9.88e-04 |
| ENSMUSG00000074570 | Cass4 | protein\_coding | 2:172393794-172433757 (+) |  | 0.805 | 9.03e-05 | 9.92e-04 |
| ENSMUSG00000003235 | Eif2b5 | protein\_coding | 16:20498817-20509323 (+) |  | -0.291 | 9.20e-05 | 1.01e-03 |
| ENSMUSG00000017677 | Wsb1 | protein\_coding | 11:79239372-79254671 (-) |  | 0.349 | 9.20e-05 | 1.01e-03 |
| ENSMUSG00000030677 | Kif22 | protein\_coding | 7:127027729-127042471 (-) |  | -0.304 | 9.21e-05 | 1.01e-03 |
| ENSMUSG00000063779 | Chil4 | protein\_coding | 3:106201490-106219507 (-) |  | 2.650 | 9.30e-05 | 1.02e-03 |
| ENSMUSG00000107383 | Gm4366 | processed\_pseudogene | 7:116824510-116825851 (-) |  | -0.618 | 9.38e-05 | 1.03e-03 |
| ENSMUSG00000029777 | Gars | protein\_coding | 6:55038007-55079500 (+) |  | -0.370 | 9.40e-05 | 1.03e-03 |
| ENSMUSG00000020077 | Srgn | protein\_coding | 10:62493833-62527451 (-) |  | -0.599 | 9.40e-05 | 1.03e-03 |
| ENSMUSG00000005800 | Mmp8 | protein\_coding | 9:7558456-7568485 (+) |  | -0.463 | 9.47e-05 | 1.03e-03 |
| ENSMUSG00000028494 | Plin2 | protein\_coding | 4:86648386-86670060 (-) |  | -0.572 | 9.52e-05 | 1.04e-03 |
| ENSMUSG00000029614 | Rpl6 | protein\_coding | 5:121204481-121209241 (+) |  | -0.236 | 9.53e-05 | 1.04e-03 |
| ENSMUSG00000018427 | Ypel2 | protein\_coding | 11:86936425-86993707 (-) |  | 0.625 | 9.56e-05 | 1.04e-03 |
| ENSMUSG00000040712 | Camta2 | protein\_coding | 11:70669463-70688105 (-) |  | 0.417 | 9.62e-05 | 1.05e-03 |
| ENSMUSG00000031502 | Col4a1 | protein\_coding | 8:11198423-11312826 (-) |  | 1.560 | 9.91e-05 | 1.08e-03 |
| ENSMUSG00000061371 | Zfp873 | protein\_coding | 10:82048123-82064745 (+) |  | 0.658 | 9.92e-05 | 1.08e-03 |
| ENSMUSG00000027522 | Stx16 | protein\_coding | 2:174076308-174099771 (+) |  | 0.400 | 9.95e-05 | 1.08e-03 |
| ENSMUSG00000032382 | Snx1 | protein\_coding | 9:66088133-66126587 (-) |  | -0.330 | 1.00e-04 | 1.09e-03 |
| ENSMUSG00000022285 | Ywhaz | protein\_coding | 15:36770770-36796929 (-) |  | -0.238 | 1.01e-04 | 1.09e-03 |
| ENSMUSG00000033713 | Foxn3 | protein\_coding | 12:99190078-99563582 (-) |  | 0.533 | 1.01e-04 | 1.09e-03 |
| ENSMUSG00000104835 | Gm5547 | lncRNA | 3:105815540-105817357 (+) |  | 1.200 | 1.01e-04 | 1.09e-03 |
| ENSMUSG00000028693 | Nasp | protein\_coding | 4:116601052-116627941 (-) |  | -0.306 | 1.03e-04 | 1.11e-03 |
| ENSMUSG00000018927 | Ccl6 | protein\_coding | 11:83587882-83593087 (-) |  | 0.977 | 1.03e-04 | 1.11e-03 |
| ENSMUSG00000000730 | Dnmt3l | protein\_coding | 10:78041947-78063622 (+) |  | -2.040 | 1.04e-04 | 1.12e-03 |
| ENSMUSG00000046456 | Tmem150b | protein\_coding | 7:4706832-4725249 (-) |  | 0.443 | 1.04e-04 | 1.12e-03 |
| ENSMUSG00000055762 | Eef1d | protein\_coding | 15:75894205-75909556 (-) |  | -0.327 | 1.04e-04 | 1.12e-03 |
| ENSMUSG00000005161 | Prdx2 | protein\_coding | 8:84969587-84974834 (+) |  | -0.336 | 1.04e-04 | 1.12e-03 |
| ENSMUSG00000079037 | Prnp | protein\_coding | 2:131909928-131938429 (+) |  | -2.690 | 1.05e-04 | 1.12e-03 |
| ENSMUSG00000017776 | Crk | protein\_coding | 11:75679259-75706908 (+) |  | 0.322 | 1.05e-04 | 1.12e-03 |
| ENSMUSG00000039748 | Exo1 | protein\_coding | 1:175880581-175913489 (+) |  | -0.612 | 1.05e-04 | 1.13e-03 |
| ENSMUSG00000033161 | Atp1a1 | protein\_coding | 3:101576219-101604684 (-) |  | -0.354 | 1.05e-04 | 1.13e-03 |
| ENSMUSG00000024660 | Incenp | protein\_coding | 19:9872296-9899551 (-) |  | -0.286 | 1.05e-04 | 1.13e-03 |
| ENSMUSG00000043091 | Tuba1c | protein\_coding | 15:99029891-99038110 (+) |  | -0.364 | 1.05e-04 | 1.13e-03 |
| ENSMUSG00000068749 | Psma5 | protein\_coding | 3:108256926-108279974 (+) |  | -0.338 | 1.05e-04 | 1.13e-03 |
| ENSMUSG00000040314 | Ctsg | protein\_coding | 14:56099881-56102574 (-) |  | -0.655 | 1.06e-04 | 1.13e-03 |
| ENSMUSG00000024981 | Acsl5 | protein\_coding | 19:55251938-55297720 (+) |  | -0.325 | 1.06e-04 | 1.13e-03 |
| ENSMUSG00000001416 | Cct3 | protein\_coding | 3:88297116-88321767 (+) |  | -0.389 | 1.06e-04 | 1.14e-03 |
| ENSMUSG00000015656 | Hspa8 | protein\_coding | 9:40800984-40810087 (+) |  | -0.321 | 1.08e-04 | 1.15e-03 |
| ENSMUSG00000047676 | Rpsa-ps10 | processed\_pseudogene | 3:150072658-150073542 (-) |  | -0.379 | 1.08e-04 | 1.15e-03 |
| ENSMUSG00000020120 | Plek | protein\_coding | 11:16971206-17052381 (-) |  | 0.585 | 1.08e-04 | 1.15e-03 |
| ENSMUSG00000014633 | Cmc2 | protein\_coding | 8:116888685-116921455 (-) |  | -0.454 | 1.08e-04 | 1.15e-03 |
| ENSMUSG00000038582 | Pptc7 | protein\_coding | 5:122284365-122324281 (+) |  | 0.368 | 1.09e-04 | 1.16e-03 |
| ENSMUSG00000039787 | Cercam | protein\_coding | 2:29869164-29882840 (+) |  | 0.717 | 1.09e-04 | 1.16e-03 |
| ENSMUSG00000056071 | S100a9 | protein\_coding | 3:90692632-90695721 (-) |  | -1.750 | 1.09e-04 | 1.16e-03 |
| ENSMUSG00000039753 | Fbxl5 | protein\_coding | 5:43744615-43821638 (-) |  | -0.618 | 1.09e-04 | 1.16e-03 |
| ENSMUSG00000034353 | Ramp1 | protein\_coding | 1:91179822-91225196 (+) |  | 0.561 | 1.09e-04 | 1.16e-03 |
| ENSMUSG00000022438 | Parvb | protein\_coding | 15:84232043-84315688 (+) |  | 0.383 | 1.10e-04 | 1.16e-03 |
| ENSMUSG00000024991 | Eif3a | protein\_coding | 19:60761117-60790658 (-) |  | -0.298 | 1.10e-04 | 1.16e-03 |
| ENSMUSG00000029623 | Pdap1 | protein\_coding | 5:145128769-145140238 (-) |  | -0.364 | 1.10e-04 | 1.16e-03 |
| ENSMUSG00000032565 | Nudt16 | protein\_coding | 9:105128903-105131824 (-) |  | 0.542 | 1.10e-04 | 1.16e-03 |
| ENSMUSG00000046295 | Ankle1 | protein\_coding | 8:71406010-71409904 (+) |  | -0.390 | 1.11e-04 | 1.17e-03 |
| ENSMUSG00000034329 | Brip1 | protein\_coding | 11:86058138-86201193 (-) |  | -0.317 | 1.12e-04 | 1.18e-03 |
| ENSMUSG00000004843 | Chmp2b | protein\_coding | 16:65539128-65562726 (-) |  | 0.385 | 1.12e-04 | 1.18e-03 |
| ENSMUSG00000063524 | Eno1 | protein\_coding | 4:150236721-150248879 (+) |  | -0.365 | 1.12e-04 | 1.18e-03 |
| ENSMUSG00000037649 | H2-DMa | protein\_coding | 17:34119541-34139101 (+) |  | 0.401 | 1.13e-04 | 1.19e-03 |
| ENSMUSG00000060594 | Layn | protein\_coding | 9:51054640-51077094 (-) |  | 0.910 | 1.14e-04 | 1.20e-03 |
| ENSMUSG00000042099 | Kank3 | protein\_coding | 17:33810519-33822918 (+) |  | 0.468 | 1.14e-04 | 1.20e-03 |
| ENSMUSG00000004849 | Ap1s1 | protein\_coding | 5:137034993-137046135 (-) |  | -0.444 | 1.14e-04 | 1.20e-03 |
| ENSMUSG00000020849 | Ywhae | protein\_coding | 11:75732869-75765845 (+) |  | -0.283 | 1.15e-04 | 1.21e-03 |
| ENSMUSG00000031659 | Adcy7 | protein\_coding | 8:88272403-88329962 (+) |  | 0.325 | 1.16e-04 | 1.22e-03 |
| ENSMUSG00000028678 | Kif2c | protein\_coding | 4:117159639-117182639 (-) |  | -0.318 | 1.17e-04 | 1.22e-03 |
| ENSMUSG00000002984 | Tomm40 | protein\_coding | 7:19701313-19715438 (-) |  | -0.401 | 1.17e-04 | 1.22e-03 |
| ENSMUSG00000061577 | Adgrg5 | protein\_coding | 8:94923694-94943280 (+) |  | 1.730 | 1.17e-04 | 1.22e-03 |
| ENSMUSG00000053398 | Phgdh | protein\_coding | 3:98313170-98339990 (-) |  | -1.070 | 1.18e-04 | 1.23e-03 |
| ENSMUSG00000109556 | Gm38843 | lncRNA | 6:82803769-82805083 (-) |  | 1.260 | 1.18e-04 | 1.23e-03 |
| ENSMUSG00000052087 | Rgs14 | protein\_coding | 13:55369732-55384687 (+) |  | 0.281 | 1.18e-04 | 1.23e-03 |
| ENSMUSG00000034850 | Tmem127 | protein\_coding | 2:127247908-127261107 (+) |  | 0.237 | 1.19e-04 | 1.24e-03 |
| ENSMUSG00000029923 | Rab19 | protein\_coding | 6:39381175-39390380 (+) |  | 0.763 | 1.20e-04 | 1.25e-03 |
| ENSMUSG00000003534 | Ddr1 | protein\_coding | 17:35681567-35704621 (-) |  | 1.330 | 1.20e-04 | 1.25e-03 |
| ENSMUSG00000020883 | Fbxl20 | protein\_coding | 11:98082556-98150403 (-) |  | 0.562 | 1.20e-04 | 1.25e-03 |
| ENSMUSG00000070056 | Mfhas1 | protein\_coding | 8:35587798-35679449 (+) |  | 0.557 | 1.20e-04 | 1.25e-03 |
| ENSMUSG00000031365 | Zfp275 | protein\_coding | X:73342621-73359080 (+) |  | 0.572 | 1.20e-04 | 1.25e-03 |
| ENSMUSG00000031508 | Ankrd10 | protein\_coding | 8:11611583-11635757 (-) |  | 0.289 | 1.21e-04 | 1.25e-03 |
| ENSMUSG00000024360 | Etf1 | protein\_coding | 18:34902785-34932007 (-) |  | -0.326 | 1.22e-04 | 1.26e-03 |
| ENSMUSG00000063273 | Naa15 | protein\_coding | 3:51415148-51476507 (+) |  | -0.287 | 1.22e-04 | 1.26e-03 |
| ENSMUSG00000112980 | D430020J02Rik | lncRNA | 12:116401947-116405210 (-) |  | -0.711 | 1.22e-04 | 1.26e-03 |
| ENSMUSG00000019297 | Nop9 | protein\_coding | 14:55745693-55755500 (+) |  | -0.329 | 1.22e-04 | 1.26e-03 |
| ENSMUSG00000024121 | Atp6v0c | protein\_coding | 17:24163866-24169702 (-) |  | -0.316 | 1.23e-04 | 1.27e-03 |
| ENSMUSG00000037992 | Rara | protein\_coding | 11:98927818-98974942 (+) |  | 0.380 | 1.23e-04 | 1.27e-03 |
| ENSMUSG00000038005 | Hpf1 | protein\_coding | 8:60890418-60908671 (+) |  | -0.308 | 1.23e-04 | 1.27e-03 |
| ENSMUSG00000027901 | Dennd2d | protein\_coding | 3:106482405-106503030 (+) |  | -0.462 | 1.23e-04 | 1.27e-03 |
| ENSMUSG00000023044 | Csad | protein\_coding | 15:102176999-102204724 (-) |  | 0.487 | 1.24e-04 | 1.28e-03 |
| ENSMUSG00000021133 | Susd6 | protein\_coding | 12:80790510-80880835 (+) |  | -0.255 | 1.24e-04 | 1.28e-03 |
| ENSMUSG00000052738 | Suclg1 | protein\_coding | 6:73248382-73276911 (+) |  | -0.310 | 1.25e-04 | 1.29e-03 |
| ENSMUSG00000057113 | Npm1 | protein\_coding | 11:33152287-33163206 (-) |  | -0.309 | 1.26e-04 | 1.29e-03 |
| ENSMUSG00000018548 | Trim37 | protein\_coding | 11:87127077-87220683 (+) |  | -0.390 | 1.27e-04 | 1.30e-03 |
| ENSMUSG00000030579 | Tyrobp | protein\_coding | 7:30413760-30417585 (+) |  | 0.459 | 1.27e-04 | 1.30e-03 |
| ENSMUSG00000016319 | Slc25a5 | protein\_coding | X:36795651-36798807 (+) |  | -0.258 | 1.28e-04 | 1.31e-03 |
| ENSMUSG00000027184 | Caprin1 | protein\_coding | 2:103762941-103797649 (-) |  | -0.213 | 1.29e-04 | 1.32e-03 |
| ENSMUSG00000032314 | Etfa | protein\_coding | 9:55454508-55512243 (-) |  | -0.306 | 1.30e-04 | 1.33e-03 |
| ENSMUSG00000026637 | Traf5 | protein\_coding | 1:191997205-192092559 (-) |  | 0.426 | 1.30e-04 | 1.33e-03 |
| ENSMUSG00000006678 | Pola1 | protein\_coding | X:93304767-93632155 (-) |  | -0.322 | 1.30e-04 | 1.33e-03 |
| ENSMUSG00000061479 | Snrpa | protein\_coding | 7:27187005-27196271 (-) |  | -0.394 | 1.30e-04 | 1.33e-03 |
| ENSMUSG00000046207 | Pik3r6 | protein\_coding | 11:68503019-68552698 (+) |  | 0.322 | 1.30e-04 | 1.33e-03 |
| ENSMUSG00000057596 | Trim30d | protein\_coding | 7:104470014-104507849 (-) |  | 0.446 | 1.31e-04 | 1.33e-03 |
| ENSMUSG00000102336 | Gm37233 | lncRNA | 1:34427002-34433264 (-) |  | -0.653 | 1.31e-04 | 1.33e-03 |
| ENSMUSG00000041355 | Ssr2 | protein\_coding | 3:88575876-88588419 (+) |  | -0.369 | 1.31e-04 | 1.33e-03 |
| ENSMUSG00000024737 | Slc15a3 | protein\_coding | 19:10839727-10859362 (+) |  | 0.289 | 1.32e-04 | 1.34e-03 |
| ENSMUSG00000022387 | Brd1 | protein\_coding | 15:88687034-88734233 (-) |  | 0.262 | 1.32e-04 | 1.34e-03 |
| ENSMUSG00000048458 | Inka2 | protein\_coding | 3:105704599-105720842 (+) |  | 1.880 | 1.32e-04 | 1.34e-03 |
| ENSMUSG00000036223 | Ska1 | protein\_coding | 18:74195299-74207818 (-) |  | -0.427 | 1.32e-04 | 1.34e-03 |
| ENSMUSG00000030674 | Qprt | protein\_coding | 7:127107114-127122226 (-) |  | 1.270 | 1.34e-04 | 1.36e-03 |
| ENSMUSG00000058470 | Gm8369 | protein\_coding | 19:11485938-11512577 (+) |  | 0.694 | 1.35e-04 | 1.37e-03 |
| ENSMUSG00000046721 | Rpl14-ps1 | processed\_pseudogene | 7:45324965-45325617 (+) |  | -0.342 | 1.36e-04 | 1.37e-03 |
| ENSMUSG00000056536 | Pign | protein\_coding | 1:105518422-105663677 (-) |  | -0.325 | 1.38e-04 | 1.40e-03 |
| ENSMUSG00000021322 | Aoah | protein\_coding | 13:20794113-21036617 (+) |  | 0.294 | 1.38e-04 | 1.40e-03 |
| ENSMUSG00000032786 | Alas1 | protein\_coding | 9:106233455-106248654 (-) |  | -0.348 | 1.38e-04 | 1.40e-03 |
| ENSMUSG00000074274 | D930028M14Rik | lncRNA | 7:25152457-25156627 (+) |  | 1.130 | 1.40e-04 | 1.42e-03 |
| ENSMUSG00000055485 | Soga1 | protein\_coding | 2:157015799-157079254 (-) |  | 0.691 | 1.41e-04 | 1.42e-03 |
| ENSMUSG00000023341 | Mx2 | polymorphic\_pseudogene | 16:97535308-97560900 (+) |  | 0.808 | 1.41e-04 | 1.42e-03 |
| ENSMUSG00000048120 | Entpd1 | protein\_coding | 19:40612366-40741602 (+) |  | 0.593 | 1.43e-04 | 1.44e-03 |
| ENSMUSG00000040312 | Cchcr1 | protein\_coding | 17:35517100-35531015 (+) |  | -0.675 | 1.43e-04 | 1.44e-03 |
| ENSMUSG00000089844 | A530032D15Rik | protein\_coding | 1:85083069-85109853 (-) |  | -1.290 | 1.43e-04 | 1.44e-03 |
| ENSMUSG00000001018 | Snapin | protein\_coding | 3:90488026-90491033 (-) |  | 0.384 | 1.44e-04 | 1.45e-03 |
| ENSMUSG00000005610 | Eif4g2 | protein\_coding | 7:111067750-111083030 (-) |  | -0.293 | 1.44e-04 | 1.45e-03 |
| ENSMUSG00000079019 | Insl3 | protein\_coding | 8:71689214-71690575 (+) |  | -2.250 | 1.45e-04 | 1.45e-03 |
| ENSMUSG00000061981 | Flot2 | protein\_coding | 11:78037931-78060434 (+) |  | -0.300 | 1.46e-04 | 1.46e-03 |
| ENSMUSG00000032727 | Mier3 | protein\_coding | 13:111680979-111718596 (+) |  | 0.358 | 1.47e-04 | 1.47e-03 |
| ENSMUSG00000108084 | Gm7932 | lncRNA | 6:48860329-48866083 (+) |  | -2.900 | 1.47e-04 | 1.47e-03 |
| ENSMUSG00000092086 | Gm6793 | processed\_pseudogene | 8:112013953-112015008 (-) |  | -0.360 | 1.49e-04 | 1.49e-03 |
| ENSMUSG00000098055 | Gm26947 | lncRNA | 10:60931991-60940942 (+) |  | -1.150 | 1.50e-04 | 1.49e-03 |
| ENSMUSG00000073386 | 9830107B12Rik | protein\_coding | 17:48125605-48146268 (-) |  | 1.180 | 1.52e-04 | 1.51e-03 |
| ENSMUSG00000009563 | Tor2a | protein\_coding | 2:32757234-32762244 (+) |  | -0.383 | 1.52e-04 | 1.51e-03 |
| ENSMUSG00000025104 | Hdgfl3 | protein\_coding | 7:81881251-81934473 (-) |  | 0.621 | 1.52e-04 | 1.52e-03 |
| ENSMUSG00000020706 | Ftsj3 | protein\_coding | 11:106249142-106256079 (-) |  | -0.379 | 1.52e-04 | 1.52e-03 |
| ENSMUSG00000001700 | Gramd3 | protein\_coding | 18:56400337-56503792 (+) |  | 0.519 | 1.53e-04 | 1.52e-03 |
| ENSMUSG00000027087 | Itgav | protein\_coding | 2:83724397-83806916 (+) |  | 0.377 | 1.54e-04 | 1.53e-03 |
| ENSMUSG00000026142 | Rhbdd1 | protein\_coding | 1:82316452-82445366 (+) |  | 0.339 | 1.55e-04 | 1.54e-03 |
| ENSMUSG00000060600 | Eno3 | protein\_coding | 11:70657202-70662513 (+) |  | 0.866 | 1.55e-04 | 1.54e-03 |
| ENSMUSG00000066150 | Slc31a1 | protein\_coding | 4:62360727-62391769 (+) |  | -0.323 | 1.58e-04 | 1.56e-03 |
| ENSMUSG00000027242 | Wdr76 | protein\_coding | 2:121506723-121544860 (+) |  | -0.370 | 1.58e-04 | 1.56e-03 |
| ENSMUSG00000026771 | Spopl | protein\_coding | 2:23506220-23572106 (-) |  | 0.435 | 1.59e-04 | 1.58e-03 |
| ENSMUSG00000031928 | Mre11a | protein\_coding | 9:14784654-14837123 (+) |  | -0.306 | 1.59e-04 | 1.58e-03 |
| ENSMUSG00000067071 | Hes6 | protein\_coding | 1:91411483-91414038 (-) |  | 0.364 | 1.61e-04 | 1.59e-03 |
| ENSMUSG00000046562 | Unc119b | protein\_coding | 5:115122550-115134975 (-) |  | 0.334 | 1.61e-04 | 1.59e-03 |
| ENSMUSG00000066487 | Gm5786 | processed\_pseudogene | 12:59081019-59081900 (+) |  | -0.773 | 1.62e-04 | 1.60e-03 |
| ENSMUSG00000041360 | Pum3 | protein\_coding | 19:27388698-27429825 (-) |  | -0.298 | 1.62e-04 | 1.60e-03 |
| ENSMUSG00000100514 | Gm12960 | processed\_pseudogene | 4:111807358-111808423 (-) |  | -0.414 | 1.62e-04 | 1.60e-03 |
| ENSMUSG00000021963 | Sap18 | protein\_coding | 14:57798180-57806638 (+) |  | -0.290 | 1.63e-04 | 1.61e-03 |
| ENSMUSG00000030793 | Pycard | protein\_coding | 7:127989708-127993867 (-) |  | -0.457 | 1.65e-04 | 1.62e-03 |
| ENSMUSG00000014859 | E2f4 | protein\_coding | 8:105297663-105305370 (+) |  | -0.330 | 1.65e-04 | 1.62e-03 |
| ENSMUSG00000049916 | 2610318N02Rik | protein\_coding | 16:17113398-17125167 (-) |  | -0.939 | 1.66e-04 | 1.63e-03 |
| ENSMUSG00000021483 | Cdk20 | protein\_coding | 13:64432314-64441773 (+) |  | -0.510 | 1.66e-04 | 1.63e-03 |
| ENSMUSG00000031791 | Tmem38a | protein\_coding | 8:72572055-72587282 (+) |  | 0.836 | 1.67e-04 | 1.64e-03 |
| ENSMUSG00000006398 | Cdc20 | protein\_coding | 4:118432901-118437352 (-) |  | -0.315 | 1.67e-04 | 1.64e-03 |
| ENSMUSG00000021738 | Atxn7 | protein\_coding | 14:13961440-14107302 (+) |  | 0.403 | 1.70e-04 | 1.66e-03 |
| ENSMUSG00000033033 | Calhm2 | protein\_coding | 19:47105353-47138294 (-) |  | 0.360 | 1.71e-04 | 1.67e-03 |
| ENSMUSG00000071715 | Ncf4 | protein\_coding | 15:78244801-78262580 (+) |  | -0.345 | 1.71e-04 | 1.68e-03 |
| ENSMUSG00000015749 | Anp32e | protein\_coding | 3:95929246-95947390 (+) |  | -0.266 | 1.72e-04 | 1.68e-03 |
| ENSMUSG00000087543 | Gm16576 | lncRNA | 15:79742698-79757394 (+) |  | 0.580 | 1.72e-04 | 1.68e-03 |
| ENSMUSG00000029501 | Ankle2 | protein\_coding | 5:110231004-110256651 (+) |  | 0.285 | 1.73e-04 | 1.69e-03 |
| ENSMUSG00000039168 | Dap | protein\_coding | 15:31224314-31274341 (+) |  | -0.310 | 1.73e-04 | 1.69e-03 |
| ENSMUSG00000026313 | Hdac4 | protein\_coding | 1:91928779-92195699 (-) |  | -0.399 | 1.74e-04 | 1.69e-03 |
| ENSMUSG00000021577 | Sdha | protein\_coding | 13:74322254-74350280 (-) |  | -0.267 | 1.74e-04 | 1.69e-03 |
| ENSMUSG00000011831 | Evi5 | protein\_coding | 5:107744795-107875107 (-) |  | 0.327 | 1.74e-04 | 1.69e-03 |
| ENSMUSG00000026709 | Dars2 | protein\_coding | 1:161040601-161070658 (-) |  | -0.433 | 1.74e-04 | 1.69e-03 |
| ENSMUSG00000060477 | Irak2 | protein\_coding | 6:113638467-113695026 (+) |  | 0.456 | 1.75e-04 | 1.70e-03 |
| ENSMUSG00000049625 | Tifab | protein\_coding | 13:56173704-56178885 (-) |  | 0.326 | 1.75e-04 | 1.70e-03 |
| ENSMUSG00000041147 | Brca2 | protein\_coding | 5:150522630-150570329 (+) |  | -0.508 | 1.75e-04 | 1.70e-03 |
| ENSMUSG00000045312 | Lhfpl2 | protein\_coding | 13:94057796-94195409 (+) |  | -0.998 | 1.77e-04 | 1.72e-03 |
| ENSMUSG00000042770 | Hebp1 | protein\_coding | 6:135137522-135198022 (-) |  | -0.960 | 1.77e-04 | 1.72e-03 |
| ENSMUSG00000005683 | Cs | protein\_coding | 10:128337734-128362479 (+) |  | -0.322 | 1.78e-04 | 1.72e-03 |
| ENSMUSG00000001424 | Snd1 | protein\_coding | 6:28475139-28935162 (+) |  | -0.319 | 1.78e-04 | 1.72e-03 |
| ENSMUSG00000031355 | Arhgap6 | protein\_coding | X:168795099-169304435 (+) |  | 0.940 | 1.79e-04 | 1.73e-03 |
| ENSMUSG00000019768 | Esr1 | protein\_coding | 10:4611593-5005614 (+) |  | 0.616 | 1.80e-04 | 1.73e-03 |
| ENSMUSG00000032040 | Dcps | protein\_coding | 9:35124408-35176061 (-) |  | -0.329 | 1.80e-04 | 1.73e-03 |
| ENSMUSG00000096917 | 2500002B13Rik | lncRNA | 8:57488054-57508877 (+) |  | -0.508 | 1.80e-04 | 1.73e-03 |
| ENSMUSG00000021792 | Prxl2a | protein\_coding | 14:40993740-41013788 (-) |  | 1.510 | 1.81e-04 | 1.74e-03 |
| ENSMUSG00000032126 | Hmbs | protein\_coding | 9:44336339-44344228 (-) |  | -0.387 | 1.81e-04 | 1.74e-03 |
| ENSMUSG00000028788 | Ptp4a2 | protein\_coding | 4:129811219-129850003 (+) |  | 0.269 | 1.81e-04 | 1.74e-03 |
| ENSMUSG00000026866 | Kynu | protein\_coding | 2:43555329-43682715 (+) |  | -0.657 | 1.81e-04 | 1.74e-03 |
| ENSMUSG00000057406 | Nsd2 | protein\_coding | 5:33820725-33897975 (+) |  | -0.346 | 1.82e-04 | 1.75e-03 |
| ENSMUSG00000028964 | Park7 | protein\_coding | 4:150897133-150914437 (-) |  | -0.437 | 1.82e-04 | 1.75e-03 |
| ENSMUSG00000004099 | Dnmt1 | protein\_coding | 9:20907209-20959888 (-) |  | -0.404 | 1.83e-04 | 1.76e-03 |
| ENSMUSG00000087177 | E130307A14Rik | lncRNA | 10:39621412-39732007 (-) |  | 0.686 | 1.84e-04 | 1.76e-03 |
| ENSMUSG00000023004 | Tuba1b | protein\_coding | 15:98931425-98934565 (-) |  | -0.330 | 1.85e-04 | 1.77e-03 |
| ENSMUSG00000027018 | Hat1 | protein\_coding | 2:71388958-71441622 (+) |  | -0.370 | 1.85e-04 | 1.77e-03 |
| ENSMUSG00000031353 | Rbbp7 | protein\_coding | X:162760402-162779092 (+) |  | -0.290 | 1.85e-04 | 1.77e-03 |
| ENSMUSG00000039067 | Psmd7 | protein\_coding | 8:107580381-107588464 (-) |  | -0.340 | 1.85e-04 | 1.77e-03 |
| ENSMUSG00000031214 | Ophn1 | protein\_coding | X:98554277-98891025 (-) |  | 0.641 | 1.86e-04 | 1.78e-03 |
| ENSMUSG00000062949 | Atp11c | protein\_coding | X:60223290-60592698 (-) |  | 0.353 | 1.87e-04 | 1.78e-03 |
| ENSMUSG00000041431 | Ccnb1 | protein\_coding | 13:100778650-100786570 (-) |  | -0.312 | 1.87e-04 | 1.79e-03 |
| ENSMUSG00000024583 | Txnl1 | protein\_coding | 18:63661094-63708801 (-) |  | -0.325 | 1.88e-04 | 1.79e-03 |
| ENSMUSG00000028433 | Ubap2 | protein\_coding | 4:41194313-41275144 (-) |  | -0.413 | 1.89e-04 | 1.80e-03 |
| ENSMUSG00000002329 | Mdp1 | protein\_coding | 14:55657879-55660508 (-) |  | 0.346 | 1.90e-04 | 1.81e-03 |
| ENSMUSG00000000318 | Clec10a | protein\_coding | 11:70156197-70170834 (+) |  | 0.446 | 1.92e-04 | 1.83e-03 |
| ENSMUSG00000032425 | Zfp949 | protein\_coding | 9:88548020-88571061 (+) |  | 0.593 | 1.93e-04 | 1.83e-03 |
| ENSMUSG00000071041 | Impdh2-ps | processed\_pseudogene | 8:100030558-100032102 (+) |  | -0.446 | 1.93e-04 | 1.83e-03 |
| ENSMUSG00000026932 | Nacc2 | protein\_coding | 2:26055535-26123220 (-) |  | 0.591 | 1.95e-04 | 1.85e-03 |
| ENSMUSG00000026019 | Wdr12 | protein\_coding | 1:60069785-60098645 (-) |  | -0.501 | 1.96e-04 | 1.86e-03 |
| ENSMUSG00000032411 | Tfdp2 | protein\_coding | 9:96196275-96323646 (+) |  | 0.414 | 1.97e-04 | 1.86e-03 |
| ENSMUSG00000020330 | Hmmr | protein\_coding | 11:40701395-40733422 (-) |  | -0.377 | 1.97e-04 | 1.87e-03 |
| ENSMUSG00000041491 | Cep78 | protein\_coding | 19:15955773-15984989 (-) |  | -0.352 | 1.99e-04 | 1.88e-03 |
| ENSMUSG00000037366 | Pafah2 | protein\_coding | 4:134396320-134427413 (+) |  | -0.692 | 1.99e-04 | 1.88e-03 |
| ENSMUSG00000002409 | Dyrk1b | protein\_coding | 7:28179469-28187294 (+) |  | 0.593 | 2.00e-04 | 1.89e-03 |
| ENSMUSG00000029408 | Abcb9 | protein\_coding | 5:124061530-124095798 (-) |  | 1.110 | 2.01e-04 | 1.90e-03 |
| ENSMUSG00000068551 | Zfp467 | protein\_coding | 6:48427697-48445825 (-) |  | 0.564 | 2.02e-04 | 1.90e-03 |
| ENSMUSG00000040339 | Fam102b | protein\_coding | 3:108970997-109027607 (-) |  | 0.392 | 2.02e-04 | 1.90e-03 |
| ENSMUSG00000021254 | Gpatch2l | protein\_coding | 12:86241858-86291784 (+) |  | 0.292 | 2.03e-04 | 1.91e-03 |
| ENSMUSG00000047409 | Ctdspl | protein\_coding | 9:118926453-119043998 (+) |  | -0.409 | 2.03e-04 | 1.91e-03 |
| ENSMUSG00000004460 | Dnajb11 | protein\_coding | 16:22857845-22879634 (+) |  | -0.274 | 2.03e-04 | 1.91e-03 |
| ENSMUSG00000072235 | Tuba1a | protein\_coding | 15:98949837-98953703 (-) |  | -0.436 | 2.04e-04 | 1.92e-03 |
| ENSMUSG00000048249 | Crebrf | protein\_coding | 17:26715650-26776635 (+) |  | 0.488 | 2.05e-04 | 1.93e-03 |
| ENSMUSG00000021432 | Slc35b3 | protein\_coding | 13:38932136-38960875 (-) |  | 0.456 | 2.06e-04 | 1.94e-03 |
| ENSMUSG00000013160 | Atp6v0d1 | protein\_coding | 8:105524465-105566047 (-) |  | -0.269 | 2.08e-04 | 1.95e-03 |
| ENSMUSG00000031060 | Rbm10 | protein\_coding | X:20617503-20650901 (+) |  | -0.271 | 2.09e-04 | 1.95e-03 |
| ENSMUSG00000039842 | Mcph1 | protein\_coding | 8:18595131-18803189 (+) |  | -0.415 | 2.09e-04 | 1.96e-03 |
| ENSMUSG00000020532 | Acaca | protein\_coding | 11:84129672-84401651 (+) |  | -0.414 | 2.09e-04 | 1.96e-03 |
| ENSMUSG00000029521 | Chek2 | protein\_coding | 5:110839979-110874145 (+) |  | -0.355 | 2.10e-04 | 1.96e-03 |
| ENSMUSG00000045216 | Hs6st1 | protein\_coding | 1:36068400-36106446 (+) |  | 0.419 | 2.10e-04 | 1.96e-03 |
| ENSMUSG00000040997 | Abhd4 | protein\_coding | 14:54254188-54270637 (+) |  | 0.494 | 2.10e-04 | 1.96e-03 |
| ENSMUSG00000012535 | Tnpo3 | protein\_coding | 6:29540827-29609887 (-) |  | -0.271 | 2.10e-04 | 1.96e-03 |
| ENSMUSG00000020788 | Atp2a3 | protein\_coding | 11:72961169-72993044 (+) |  | 0.333 | 2.11e-04 | 1.97e-03 |
| ENSMUSG00000056220 | Pla2g4a | protein\_coding | 1:149829618-149961290 (-) |  | -0.456 | 2.11e-04 | 1.97e-03 |
| ENSMUSG00000021629 | Slc30a5 | protein\_coding | 13:100802648-100833427 (-) |  | -0.239 | 2.12e-04 | 1.97e-03 |
| ENSMUSG00000032855 | Pkd1 | protein\_coding | 17:24549834-24596508 (+) |  | 0.366 | 2.12e-04 | 1.97e-03 |
| ENSMUSG00000030726 | Pold3 | protein\_coding | 7:100082111-100121565 (-) |  | -0.329 | 2.12e-04 | 1.98e-03 |
| ENSMUSG00000026632 | Tatdn3 | protein\_coding | 1:191045826-191062932 (-) |  | 0.616 | 2.13e-04 | 1.98e-03 |
| ENSMUSG00000056724 | Nbeal2 | protein\_coding | 9:110624789-110654161 (-) |  | 0.426 | 2.13e-04 | 1.98e-03 |
| ENSMUSG00000020604 | Arsg | protein\_coding | 11:109473374-109573330 (+) |  | -0.618 | 2.15e-04 | 2.00e-03 |
| ENSMUSG00000060568 | Fam78b | protein\_coding | 1:167001417-167091302 (+) |  | 1.140 | 2.15e-04 | 2.00e-03 |
| ENSMUSG00000031633 | Slc25a4 | protein\_coding | 8:46206797-46211284 (-) |  | -0.258 | 2.16e-04 | 2.00e-03 |
| ENSMUSG00000030036 | Mogs | protein\_coding | 6:83115496-83118898 (+) |  | -0.345 | 2.16e-04 | 2.00e-03 |
| ENSMUSG00000031828 | Klhl36 | protein\_coding | 8:119862266-119876995 (+) |  | 0.401 | 2.17e-04 | 2.01e-03 |
| ENSMUSG00000035967 | Ints6l | protein\_coding | X:56454857-56507843 (+) |  | 0.434 | 2.18e-04 | 2.01e-03 |
| ENSMUSG00000111535 | Gm35154 | lncRNA | 10:44598720-44689081 (+) |  | 0.957 | 2.19e-04 | 2.02e-03 |
| ENSMUSG00000038047 | Haus6 | protein\_coding | 4:86578855-86612055 (-) |  | -0.395 | 2.20e-04 | 2.03e-03 |
| ENSMUSG00000027944 | Hax1 | protein\_coding | 3:89995446-89998780 (-) |  | -0.360 | 2.20e-04 | 2.03e-03 |
| ENSMUSG00000075415 | Fnbp1 | protein\_coding | 2:31026206-31142008 (-) |  | 0.242 | 2.20e-04 | 2.03e-03 |
| ENSMUSG00000074781 | Ube2n | protein\_coding | 10:95515145-95545657 (+) |  | -0.331 | 2.22e-04 | 2.04e-03 |
| ENSMUSG00000039763 | Dnajc28 | protein\_coding | 16:91614254-91619026 (-) |  | 0.767 | 2.23e-04 | 2.06e-03 |
| ENSMUSG00000024164 | C3 | protein\_coding | 17:57203970-57228136 (-) |  | 0.246 | 2.26e-04 | 2.08e-03 |
| ENSMUSG00000020635 | Fkbp1b | protein\_coding | 12:4833174-4841591 (-) |  | 0.479 | 2.26e-04 | 2.08e-03 |
| ENSMUSG00000034075 | Zdhhc5 | protein\_coding | 2:84687970-84715180 (-) |  | -0.258 | 2.26e-04 | 2.08e-03 |
| ENSMUSG00000002948 | Map2k7 | protein\_coding | 8:4238740-4247897 (+) |  | 0.381 | 2.26e-04 | 2.08e-03 |
| ENSMUSG00000092203 | 1110038B12Rik | lncRNA | 17:34950238-34952471 (-) |  | -0.530 | 2.26e-04 | 2.08e-03 |
| ENSMUSG00000029330 | Cds1 | protein\_coding | 5:101765130-101823858 (+) |  | 0.358 | 2.29e-04 | 2.10e-03 |
| ENSMUSG00000030107 | Usp18 | protein\_coding | 6:121245906-121270917 (+) |  | 0.514 | 2.31e-04 | 2.11e-03 |
| ENSMUSG00000057219 | Armc7 | protein\_coding | 11:115475667-115490467 (+) |  | 0.354 | 2.32e-04 | 2.13e-03 |
| ENSMUSG00000052713 | Zfp608 | protein\_coding | 18:54888048-54992555 (-) |  | -0.374 | 2.33e-04 | 2.13e-03 |
| ENSMUSG00000003380 | Rabac1 | protein\_coding | 7:24969752-24972754 (-) |  | 0.475 | 2.33e-04 | 2.13e-03 |
| ENSMUSG00000067121 | Gm7027 | processed\_pseudogene | 7:101573460-101574321 (-) |  | -0.450 | 2.34e-04 | 2.14e-03 |
| ENSMUSG00000022797 | Tfrc | protein\_coding | 16:32608920-32632794 (+) |  | -0.451 | 2.35e-04 | 2.14e-03 |
| ENSMUSG00000059179 | Gm8991 | processed\_pseudogene | 5:16729996-16731070 (-) |  | -0.523 | 2.35e-04 | 2.14e-03 |
| ENSMUSG00000038481 | Cdk19 | protein\_coding | 10:40339564-40483818 (+) |  | 0.226 | 2.36e-04 | 2.15e-03 |
| ENSMUSG00000036412 | Arsi | protein\_coding | 18:60911780-60918561 (+) |  | 0.868 | 2.36e-04 | 2.15e-03 |
| ENSMUSG00000067869 | Tcea1-ps1 | processed\_pseudogene | 15:90882615-90883518 (+) |  | 0.421 | 2.37e-04 | 2.15e-03 |
| ENSMUSG00000108732 | 2310043P16Rik | TEC | 7:34389732-34393612 (+) |  | 1.190 | 2.38e-04 | 2.16e-03 |
| ENSMUSG00000029416 | Slc15a4 | protein\_coding | 5:127595664-127632897 (-) |  | 0.326 | 2.38e-04 | 2.17e-03 |
| ENSMUSG00000057706 | Mex3b | protein\_coding | 7:82867333-82871515 (+) |  | 0.724 | 2.38e-04 | 2.17e-03 |
| ENSMUSG00000032115 | Hyou1 | protein\_coding | 9:44379490-44392369 (+) |  | -0.399 | 2.39e-04 | 2.17e-03 |
| ENSMUSG00000015467 | Egfl8 | protein\_coding | 17:34613349-34615971 (-) |  | 0.859 | 2.39e-04 | 2.17e-03 |
| ENSMUSG00000027331 | Knstrn | protein\_coding | 2:118814003-118853957 (+) |  | -0.299 | 2.40e-04 | 2.17e-03 |
| ENSMUSG00000030652 | Coq7 | protein\_coding | 7:118509659-118533356 (-) |  | -0.558 | 2.42e-04 | 2.19e-03 |
| ENSMUSG00000020859 | Spag9 | protein\_coding | 11:93996091-94126085 (+) |  | 0.285 | 2.42e-04 | 2.19e-03 |
| ENSMUSG00000029752 | Asns | protein\_coding | 6:7675169-7693254 (-) |  | -1.540 | 2.43e-04 | 2.20e-03 |
| ENSMUSG00000001016 | Ilf2 | protein\_coding | 3:90476126-90488379 (+) |  | -0.271 | 2.44e-04 | 2.21e-03 |
| ENSMUSG00000001604 | Tcea3 | protein\_coding | 4:136247729-136274898 (+) |  | 0.848 | 2.46e-04 | 2.23e-03 |
| ENSMUSG00000042766 | Trim46 | protein\_coding | 3:89234177-89246309 (-) |  | -1.510 | 2.48e-04 | 2.24e-03 |
| ENSMUSG00000044811 | Cd300c2 | protein\_coding | 11:114996721-115001880 (-) |  | 0.646 | 2.48e-04 | 2.24e-03 |
| ENSMUSG00000029490 | Mfsd7a | protein\_coding | 5:108441054-108449100 (-) |  | 0.404 | 2.49e-04 | 2.24e-03 |
| ENSMUSG00000028932 | Psmc2 | protein\_coding | 5:21785283-21803787 (+) |  | -0.268 | 2.51e-04 | 2.26e-03 |
| ENSMUSG00000031983 | 2310022B05Rik | protein\_coding | 8:124635756-124663369 (-) |  | -0.467 | 2.52e-04 | 2.26e-03 |
| ENSMUSG00000030224 | Strap | protein\_coding | 6:137735078-137751932 (+) |  | -0.354 | 2.53e-04 | 2.28e-03 |
| ENSMUSG00000092564 | BC051226 | lncRNA | 17:33908044-33909317 (-) |  | 0.941 | 2.55e-04 | 2.29e-03 |
| ENSMUSG00000018932 | Map2k3 | protein\_coding | 11:60932033-60952811 (+) |  | -0.277 | 2.55e-04 | 2.29e-03 |
| ENSMUSG00000026615 | Eprs | protein\_coding | 1:185363044-185428360 (+) |  | -0.393 | 2.55e-04 | 2.29e-03 |
| ENSMUSG00000026641 | Usf1 | protein\_coding | 1:171411313-171419142 (+) |  | -0.228 | 2.55e-04 | 2.29e-03 |
| ENSMUSG00000045551 | Fpr1 | protein\_coding | 17:17876471-17883940 (-) |  | 1.000 | 2.55e-04 | 2.29e-03 |
| ENSMUSG00000063052 | Lrrc40 | protein\_coding | 3:158036662-158068487 (+) |  | -0.357 | 2.56e-04 | 2.29e-03 |
| ENSMUSG00000004980 | Hnrnpa2b1 | protein\_coding | 6:51460932-51469894 (-) |  | -0.281 | 2.60e-04 | 2.33e-03 |
| ENSMUSG00000032265 | Tent5a | protein\_coding | 9:85320439-85327348 (-) |  | 0.389 | 2.60e-04 | 2.33e-03 |
| ENSMUSG00000024782 | Ak3 | protein\_coding | 19:29020833-29047961 (-) |  | 0.394 | 2.62e-04 | 2.34e-03 |
| ENSMUSG00000071379 | Hpcal1 | protein\_coding | 12:17690856-17791933 (+) |  | -0.272 | 2.63e-04 | 2.35e-03 |
| ENSMUSG00000030047 | Arhgap25 | protein\_coding | 6:87458545-87533259 (-) |  | 0.352 | 2.63e-04 | 2.35e-03 |
| ENSMUSG00000031232 | Magt1 | protein\_coding | X:105968084-106011906 (-) |  | -0.295 | 2.64e-04 | 2.35e-03 |
| ENSMUSG00000007987 | Ift22 | protein\_coding | 5:136908150-136915404 (+) |  | -0.529 | 2.66e-04 | 2.37e-03 |
| ENSMUSG00000099413 | Gm17767 | lncRNA | 1:51507086-51596718 (-) |  | -0.969 | 2.66e-04 | 2.37e-03 |
| ENSMUSG00000026696 | Vamp4 | protein\_coding | 1:162570515-162599084 (+) |  | -0.422 | 2.67e-04 | 2.38e-03 |
| ENSMUSG00000045328 | Cenpe | protein\_coding | 3:135212537-135273611 (+) |  | -0.312 | 2.69e-04 | 2.39e-03 |
| ENSMUSG00000016256 | Ctsz | protein\_coding | 2:174427493-174439039 (-) |  | -0.313 | 2.69e-04 | 2.39e-03 |
| ENSMUSG00000022774 | Ncbp2 | protein\_coding | 16:31948513-31961781 (+) |  | -0.354 | 2.70e-04 | 2.40e-03 |
| ENSMUSG00000021196 | Pfkp | protein\_coding | 13:6579768-6648777 (-) |  | -0.326 | 2.71e-04 | 2.40e-03 |
| ENSMUSG00000023034 | Nr4a1 | protein\_coding | 15:101254269-101274795 (+) |  | 1.550 | 2.71e-04 | 2.40e-03 |
| ENSMUSG00000027508 | Pag1 | protein\_coding | 3:9687479-9833679 (-) |  | -0.430 | 2.71e-04 | 2.40e-03 |
| ENSMUSG00000078812 | Eif5a | protein\_coding | 11:69916714-69921958 (-) |  | -0.320 | 2.71e-04 | 2.40e-03 |
| ENSMUSG00000044042 | Fmn1 | protein\_coding | 2:113327736-113716767 (+) |  | -1.020 | 2.73e-04 | 2.42e-03 |
| ENSMUSG00000026174 | Cnot9 | protein\_coding | 1:74506058-74530842 (+) |  | -0.307 | 2.73e-04 | 2.42e-03 |
| ENSMUSG00000021583 | Erap1 | protein\_coding | 13:74639568-74693201 (+) |  | -0.256 | 2.76e-04 | 2.44e-03 |
| ENSMUSG00000024397 | Aif1 | protein\_coding | 17:35170991-35176068 (-) |  | 0.561 | 2.78e-04 | 2.45e-03 |
| ENSMUSG00000024735 | Prpf19 | protein\_coding | 19:10895231-10909559 (+) |  | -0.293 | 2.79e-04 | 2.46e-03 |
| ENSMUSG00000001281 | Itgb7 | protein\_coding | 15:102215995-102231944 (-) |  | 0.259 | 2.79e-04 | 2.46e-03 |
| ENSMUSG00000031171 | Ftsj1 | protein\_coding | X:8238668-8252406 (-) |  | -0.391 | 2.80e-04 | 2.47e-03 |
| ENSMUSG00000046062 | Ppp1r15b | protein\_coding | 1:133131143-133139783 (+) |  | -0.273 | 2.80e-04 | 2.47e-03 |
| ENSMUSG00000026489 | Coq8a | protein\_coding | 1:180165238-180199602 (-) |  | 0.878 | 2.83e-04 | 2.49e-03 |
| ENSMUSG00000068921 | Dap3 | protein\_coding | 3:88920803-88951181 (-) |  | -0.287 | 2.83e-04 | 2.49e-03 |
| ENSMUSG00000023505 | Cdca3 | protein\_coding | 6:124829547-124833701 (+) |  | -0.305 | 2.84e-04 | 2.50e-03 |
| ENSMUSG00000041763 | Tpp2 | protein\_coding | 1:43933647-44003000 (+) |  | -0.279 | 2.87e-04 | 2.52e-03 |
| ENSMUSG00000006057 | Atp5g1 | protein\_coding | 11:96068852-96075670 (-) |  | -0.382 | 2.87e-04 | 2.52e-03 |
| ENSMUSG00000028312 | Smc2 | protein\_coding | 4:52439243-52488260 (+) |  | -0.303 | 2.87e-04 | 2.52e-03 |
| ENSMUSG00000048327 | Ckap2l | protein\_coding | 2:129268210-129297212 (-) |  | -0.302 | 2.88e-04 | 2.53e-03 |
| ENSMUSG00000079499 | 6530402F18Rik | lncRNA | 2:29245107-29253006 (-) |  | -0.482 | 2.88e-04 | 2.53e-03 |
| ENSMUSG00000017428 | Psmd11 | protein\_coding | 11:80428615-80473248 (+) |  | -0.309 | 2.88e-04 | 2.53e-03 |
| ENSMUSG00000079038 | D130040H23Rik | protein\_coding | 8:69271080-69314207 (+) |  | 1.410 | 2.89e-04 | 2.53e-03 |
| ENSMUSG00000034653 | Ythdc2 | protein\_coding | 18:44827746-44889724 (+) |  | -0.291 | 2.89e-04 | 2.53e-03 |
| ENSMUSG00000022698 | Naa50 | protein\_coding | 16:44139830-44163366 (+) |  | -0.291 | 2.89e-04 | 2.53e-03 |
| ENSMUSG00000029992 | Gfpt1 | protein\_coding | 6:87042846-87092197 (+) |  | 0.308 | 2.93e-04 | 2.56e-03 |
| ENSMUSG00000028617 | Lrrc42 | protein\_coding | 4:107233514-107253532 (-) |  | -0.479 | 2.94e-04 | 2.57e-03 |
| ENSMUSG00000028088 | Fmo5 | protein\_coding | 3:97628804-97655282 (+) |  | 0.656 | 2.98e-04 | 2.60e-03 |
| ENSMUSG00000074476 | Spc24 | protein\_coding | 9:21755442-21760303 (-) |  | -0.359 | 2.99e-04 | 2.61e-03 |
| ENSMUSG00000107355 | AI839979 | lncRNA | 5:31569595-31571397 (-) |  | 0.510 | 3.00e-04 | 2.62e-03 |
| ENSMUSG00000072763 | 5430403G16Rik | protein\_coding | 5:109674545-109691041 (-) |  | 1.110 | 3.02e-04 | 2.63e-03 |
| ENSMUSG00000042997 | Nhlrc3 | protein\_coding | 3:53448583-53463332 (-) |  | 0.370 | 3.02e-04 | 2.63e-03 |
| ENSMUSG00000064193 | Gm4735 | processed\_pseudogene | 2:80837141-80838442 (+) |  | -0.459 | 3.02e-04 | 2.63e-03 |
| ENSMUSG00000034484 | Snx2 | protein\_coding | 18:53176316-53220865 (+) |  | -0.316 | 3.06e-04 | 2.66e-03 |
| ENSMUSG00000020696 | Rffl | protein\_coding | 11:82802449-82871210 (-) |  | 0.397 | 3.07e-04 | 2.67e-03 |
| ENSMUSG00000061458 | Nol10 | protein\_coding | 12:17348458-17430095 (+) |  | -0.374 | 3.08e-04 | 2.67e-03 |
| ENSMUSG00000043157 | Arl11 | protein\_coding | 14:61309753-61311936 (+) |  | 0.419 | 3.11e-04 | 2.70e-03 |
| ENSMUSG00000022142 | Nup155 | protein\_coding | 15:8109273-8161247 (+) |  | -0.303 | 3.12e-04 | 2.70e-03 |
| ENSMUSG00000024338 | Psmb8 | protein\_coding | 17:34197721-34201454 (+) |  | -0.335 | 3.12e-04 | 2.70e-03 |
| ENSMUSG00000020608 | Smc6 | protein\_coding | 12:11265886-11319785 (+) |  | -0.265 | 3.14e-04 | 2.72e-03 |
| ENSMUSG00000015143 | Actn1 | protein\_coding | 12:80167547-80260371 (-) |  | -1.000 | 3.14e-04 | 2.72e-03 |
| ENSMUSG00000013155 | Enkd1 | protein\_coding | 8:105703651-105708210 (-) |  | -0.517 | 3.15e-04 | 2.72e-03 |
| ENSMUSG00000062929 | Cfl2 | protein\_coding | 12:54858809-54862877 (-) |  | 0.557 | 3.16e-04 | 2.73e-03 |
| ENSMUSG00000045983 | Eif4g1 | protein\_coding | 16:20668313-20692884 (+) |  | -0.319 | 3.18e-04 | 2.75e-03 |
| ENSMUSG00000027620 | Rbm39 | protein\_coding | 2:156147239-156180238 (-) |  | 0.296 | 3.19e-04 | 2.75e-03 |
| ENSMUSG00000026944 | Abca2 | protein\_coding | 2:25428703-25448540 (+) |  | 0.412 | 3.21e-04 | 2.77e-03 |
| ENSMUSG00000017548 | Suz12 | protein\_coding | 11:79993106-80034123 (+) |  | -0.270 | 3.21e-04 | 2.77e-03 |
| ENSMUSG00000042331 | Specc1 | protein\_coding | 11:61956763-62223013 (+) |  | 0.312 | 3.22e-04 | 2.77e-03 |
| ENSMUSG00000045039 | Megf8 | protein\_coding | 7:25317164-25365917 (+) |  | -0.505 | 3.24e-04 | 2.79e-03 |
| ENSMUSG00000008036 | Ap2s1 | protein\_coding | 7:16738410-16749294 (+) |  | -0.283 | 3.25e-04 | 2.80e-03 |
| ENSMUSG00000003438 | Timm50 | protein\_coding | 7:28305516-28312072 (-) |  | -0.290 | 3.26e-04 | 2.80e-03 |
| ENSMUSG00000026833 | Olfm1 | protein\_coding | 2:28192992-28230736 (+) |  | -0.337 | 3.29e-04 | 2.83e-03 |
| ENSMUSG00000034006 | Pqlc1 | protein\_coding | 18:80253292-80292725 (+) |  | 0.375 | 3.30e-04 | 2.83e-03 |
| ENSMUSG00000020075 | Ddx21 | protein\_coding | 10:62580251-62602281 (-) |  | -0.289 | 3.30e-04 | 2.83e-03 |
| ENSMUSG00000003992 | Ssbp2 | protein\_coding | 13:91460283-91703429 (+) |  | 0.538 | 3.31e-04 | 2.83e-03 |
| ENSMUSG00000022292 | Rrm2b | protein\_coding | 15:37923952-37961318 (-) |  | 0.482 | 3.32e-04 | 2.84e-03 |
| ENSMUSG00000000384 | Tbrg4 | protein\_coding | 11:6615598-6626067 (-) |  | -0.387 | 3.33e-04 | 2.85e-03 |
| ENSMUSG00000064366 | mt-Tl2 | Mt\_tRNA | MT:11671-11741 (+) |  | -1.220 | 3.34e-04 | 2.86e-03 |
| ENSMUSG00000030041 | M1ap | protein\_coding | 6:82946902-83030309 (+) |  | 0.723 | 3.35e-04 | 2.87e-03 |
| ENSMUSG00000073838 | Tufm | protein\_coding | 7:126487361-126490731 (+) |  | -0.356 | 3.36e-04 | 2.87e-03 |
| ENSMUSG00000028538 | St3gal3 | protein\_coding | 4:117932154-118134914 (-) |  | 0.453 | 3.36e-04 | 2.87e-03 |
| ENSMUSG00000038332 | Sesn1 | protein\_coding | 10:41809935-41908424 (+) |  | 0.450 | 3.42e-04 | 2.91e-03 |
| ENSMUSG00000034906 | Ncaph | protein\_coding | 2:127103809-127133954 (-) |  | -0.286 | 3.42e-04 | 2.91e-03 |
| ENSMUSG00000030062 | Rpn1 | protein\_coding | 6:88084482-88105304 (+) |  | -0.375 | 3.42e-04 | 2.91e-03 |
| ENSMUSG00000028467 | Gba2 | protein\_coding | 4:43566928-43578873 (-) |  | 0.310 | 3.45e-04 | 2.93e-03 |
| ENSMUSG00000004798 | Ulk2 | protein\_coding | 11:61775649-61855073 (-) |  | 0.398 | 3.45e-04 | 2.93e-03 |
| ENSMUSG00000039208 | Metrnl | protein\_coding | 11:121701544-121716306 (+) |  | 0.570 | 3.45e-04 | 2.93e-03 |
| ENSMUSG00000029254 | Stap1 | protein\_coding | 5:86071746-86106125 (+) |  | 0.689 | 3.45e-04 | 2.93e-03 |
| ENSMUSG00000029163 | Emilin1 | protein\_coding | 5:30913402-30921277 (+) |  | -0.342 | 3.45e-04 | 2.93e-03 |
| ENSMUSG00000031095 | Cul4b | protein\_coding | X:38533274-38576196 (-) |  | -0.256 | 3.46e-04 | 2.94e-03 |
| ENSMUSG00000029060 | Mib2 | protein\_coding | 4:155654677-155669198 (-) |  | 0.508 | 3.49e-04 | 2.96e-03 |
| ENSMUSG00000042487 | Leo1 | protein\_coding | 9:75441524-75466432 (+) |  | -0.406 | 3.50e-04 | 2.96e-03 |
| ENSMUSG00000017057 | Il13ra1 | protein\_coding | X:36112110-36171259 (+) |  | -0.535 | 3.52e-04 | 2.98e-03 |
| ENSMUSG00000009621 | Vav2 | protein\_coding | 2:27262104-27427033 (-) |  | 0.408 | 3.52e-04 | 2.98e-03 |
| ENSMUSG00000038312 | Edem2 | protein\_coding | 2:155701677-155729475 (-) |  | -0.328 | 3.55e-04 | 3.00e-03 |
| ENSMUSG00000078429 | Ctdsp2 | protein\_coding | 10:126978717-126999975 (+) |  | 0.234 | 3.55e-04 | 3.00e-03 |
| ENSMUSG00000026988 | Wdsub1 | protein\_coding | 2:59852364-59882591 (-) |  | 0.464 | 3.56e-04 | 3.00e-03 |
| ENSMUSG00000021996 | Esd | protein\_coding | 14:74732297-74750765 (+) |  | -0.349 | 3.56e-04 | 3.00e-03 |
| ENSMUSG00000054770 | Kctd18 | protein\_coding | 1:57955101-58018956 (-) |  | 0.426 | 3.62e-04 | 3.05e-03 |
| ENSMUSG00000003813 | Rad23a | protein\_coding | 8:84834019-84840665 (-) |  | -0.344 | 3.62e-04 | 3.05e-03 |
| ENSMUSG00000029363 | Rfc5 | protein\_coding | 5:117378103-117389047 (-) |  | -0.327 | 3.63e-04 | 3.06e-03 |
| ENSMUSG00000031925 | Maml2 | protein\_coding | 9:13297957-13709388 (+) |  | -1.120 | 3.63e-04 | 3.06e-03 |
| ENSMUSG00000022433 | Csnk1e | protein\_coding | 15:79417856-79455566 (-) |  | 0.311 | 3.64e-04 | 3.06e-03 |
| ENSMUSG00000025155 | Dus1l | protein\_coding | 11:120789201-120796403 (-) |  | -0.272 | 3.66e-04 | 3.08e-03 |
| ENSMUSG00000003868 | Ruvbl2 | protein\_coding | 7:45421760-45438096 (-) |  | -0.358 | 3.66e-04 | 3.08e-03 |
| ENSMUSG00000022561 | Gpaa1 | protein\_coding | 15:76331231-76334907 (+) |  | -0.301 | 3.68e-04 | 3.09e-03 |
| ENSMUSG00000056629 | Fkbp2 | protein\_coding | 19:6977741-6980501 (-) |  | -0.322 | 3.70e-04 | 3.11e-03 |
| ENSMUSG00000059108 | Ifitm6 | protein\_coding | 7:141015699-141017924 (-) |  | 0.513 | 3.71e-04 | 3.11e-03 |
| ENSMUSG00000002996 | Hbp1 | protein\_coding | 12:31926254-31950535 (-) |  | 0.330 | 3.73e-04 | 3.13e-03 |
| ENSMUSG00000035329 | Fbxo33 | protein\_coding | 12:59200655-59219725 (-) |  | -0.309 | 3.76e-04 | 3.15e-03 |
| ENSMUSG00000055612 | Cdca7 | protein\_coding | 2:72476159-72486893 (+) |  | -0.431 | 3.76e-04 | 3.15e-03 |
| ENSMUSG00000052926 | Rnaseh2a | protein\_coding | 8:84956610-84969767 (-) |  | -0.361 | 3.78e-04 | 3.16e-03 |
| ENSMUSG00000004730 | Adgre1 | protein\_coding | 17:57358691-57483527 (+) |  | 0.381 | 3.78e-04 | 3.16e-03 |
| ENSMUSG00000078485 | Plekhn1 | protein\_coding | 4:156221456-156234857 (-) |  | 0.447 | 3.78e-04 | 3.16e-03 |
| ENSMUSG00000037474 | Dtl | protein\_coding | 1:191537356-191575544 (-) |  | -0.412 | 3.79e-04 | 3.16e-03 |
| ENSMUSG00000037991 | Rmi2 | protein\_coding | 16:10835059-10892966 (+) |  | -0.692 | 3.79e-04 | 3.17e-03 |
| ENSMUSG00000038736 | Nudcd1 | protein\_coding | 15:44373163-44428307 (-) |  | -0.466 | 3.80e-04 | 3.17e-03 |
| ENSMUSG00000069516 | Lyz2 | protein\_coding | 10:117277331-117282321 (-) |  | 0.494 | 3.86e-04 | 3.21e-03 |
| ENSMUSG00000031985 | Gnpat | protein\_coding | 8:124863033-124890057 (+) |  | -0.312 | 3.86e-04 | 3.21e-03 |
| ENSMUSG00000029687 | Ezh2 | protein\_coding | 6:47530139-47595341 (-) |  | -0.297 | 3.88e-04 | 3.23e-03 |
| ENSMUSG00000055884 | Fancm | protein\_coding | 12:65075603-65132058 (+) |  | -0.477 | 3.89e-04 | 3.23e-03 |
| ENSMUSG00000026526 | Fh1 | protein\_coding | 1:175600374-175625635 (-) |  | -0.313 | 3.89e-04 | 3.23e-03 |
| ENSMUSG00000050821 | Fam131a | protein\_coding | 16:20693241-20703048 (+) |  | 0.938 | 3.89e-04 | 3.23e-03 |
| ENSMUSG00000036099 | Vezt | protein\_coding | 10:93939165-94035817 (-) |  | -0.535 | 3.90e-04 | 3.24e-03 |
| ENSMUSG00000037395 | Rcor3 | protein\_coding | 1:192098546-192138062 (-) |  | 0.453 | 3.92e-04 | 3.25e-03 |
| ENSMUSG00000020929 | Eftud2 | protein\_coding | 11:102838473-102880985 (-) |  | -0.317 | 3.94e-04 | 3.26e-03 |
| ENSMUSG00000028211 | Trp53inp1 | protein\_coding | 4:11156431-11174379 (+) |  | 0.471 | 3.94e-04 | 3.27e-03 |
| ENSMUSG00000024896 | Minpp1 | protein\_coding | 19:32485769-32515364 (+) |  | -0.309 | 3.95e-04 | 3.27e-03 |
| ENSMUSG00000006732 | Mettl1 | protein\_coding | 10:127041414-127046365 (+) |  | -0.714 | 3.99e-04 | 3.30e-03 |
| ENSMUSG00000038967 | Pdk2 | protein\_coding | 11:95026258-95041354 (-) |  | 0.768 | 3.99e-04 | 3.30e-03 |
| ENSMUSG00000024844 | Banf1 | protein\_coding | 19:5364638-5367168 (-) |  | -0.353 | 4.00e-04 | 3.30e-03 |
| ENSMUSG00000031256 | Cstf2 | protein\_coding | X:134059187-134086819 (+) |  | -0.282 | 4.00e-04 | 3.30e-03 |
| ENSMUSG00000061833 | Gm6311 | processed\_pseudogene | 13:75954136-75955013 (+) |  | -0.904 | 4.01e-04 | 3.31e-03 |
| ENSMUSG00000033781 | Asb13 | protein\_coding | 13:3634032-3653822 (+) |  | 0.521 | 4.03e-04 | 3.32e-03 |
| ENSMUSG00000025044 | Msr1 | protein\_coding | 8:39581685-39642673 (-) |  | 0.434 | 4.05e-04 | 3.34e-03 |
| ENSMUSG00000022295 | Atp6v1c1 | protein\_coding | 15:38661933-38692446 (+) |  | -0.309 | 4.07e-04 | 3.35e-03 |
| ENSMUSG00000037458 | Azin1 | protein\_coding | 15:38487427-38519266 (-) |  | -0.248 | 4.09e-04 | 3.37e-03 |
| ENSMUSG00000054582 | Pabpc1l | protein\_coding | 2:164025450-164050538 (+) |  | 0.785 | 4.09e-04 | 3.37e-03 |
| ENSMUSG00000058799 | Nap1l1 | protein\_coding | 10:111473223-111498150 (+) |  | -0.232 | 4.13e-04 | 3.40e-03 |
| ENSMUSG00000035245 | Eogt | protein\_coding | 6:97110024-97149182 (-) |  | -0.599 | 4.15e-04 | 3.41e-03 |
| ENSMUSG00000028639 | Ybx1 | protein\_coding | 4:119277981-119294604 (-) |  | -0.291 | 4.15e-04 | 3.41e-03 |
| ENSMUSG00000074419 | Gm15448 | protein\_coding | 7:3816781-3825687 (-) |  | 0.504 | 4.16e-04 | 3.42e-03 |
| ENSMUSG00000019990 | Pde7b | protein\_coding | 10:20398004-20725078 (-) |  | 0.548 | 4.17e-04 | 3.42e-03 |
| ENSMUSG00000029468 | P2rx7 | protein\_coding | 5:122643911-122691432 (+) |  | 0.301 | 4.18e-04 | 3.42e-03 |
| ENSMUSG00000034880 | Mrpl34 | protein\_coding | 8:71464959-71465747 (+) |  | -0.393 | 4.20e-04 | 3.44e-03 |
| ENSMUSG00000066632 | Pgk1-rs7 | processed\_pseudogene | 12:10898986-10900240 (-) |  | -0.843 | 4.20e-04 | 3.44e-03 |
| ENSMUSG00000026171 | Rnf25 | protein\_coding | 1:74593748-74601397 (-) |  | 0.338 | 4.21e-04 | 3.44e-03 |
| ENSMUSG00000034932 | Mrpl54 | protein\_coding | 10:81264713-81266934 (-) |  | -0.404 | 4.21e-04 | 3.44e-03 |
| ENSMUSG00000075014 | Gm10800 | protein\_coding | 2:98666547-98667301 (-) |  | 4.670 | 4.22e-04 | 3.45e-03 |
| ENSMUSG00000016619 | Nup50 | protein\_coding | 15:84923411-84942963 (+) |  | -0.284 | 4.22e-04 | 3.45e-03 |
| ENSMUSG00000015112 | Slc25a13 | protein\_coding | 6:6041218-6217173 (-) |  | -0.312 | 4.23e-04 | 3.45e-03 |
| ENSMUSG00000024740 | Ddb1 | protein\_coding | 19:10605327-10629819 (+) |  | -0.335 | 4.24e-04 | 3.46e-03 |
| ENSMUSG00000021177 | Tdp1 | protein\_coding | 12:99884517-99955219 (+) |  | -0.347 | 4.25e-04 | 3.47e-03 |
| ENSMUSG00000038280 | Ostm1 | protein\_coding | 10:42583822-42702459 (+) |  | 0.290 | 4.27e-04 | 3.47e-03 |
| ENSMUSG00000040811 | Eml2 | protein\_coding | 7:19176421-19206482 (+) |  | 0.381 | 4.29e-04 | 3.49e-03 |
| ENSMUSG00000030786 | Itgam | protein\_coding | 7:128062640-128118491 (+) |  | 0.244 | 4.31e-04 | 3.50e-03 |
| ENSMUSG00000022241 | Tars | protein\_coding | 15:11382301-11399665 (-) |  | -0.308 | 4.31e-04 | 3.50e-03 |
| ENSMUSG00000101585 | 1600010M07Rik | lncRNA | 7:109998376-110151202 (-) |  | 0.763 | 4.31e-04 | 3.50e-03 |
| ENSMUSG00000000555 | Itga5 | protein\_coding | 15:103344286-103366763 (-) |  | -0.380 | 4.33e-04 | 3.51e-03 |
| ENSMUSG00000030165 | Klrd1 | protein\_coding | 6:129591782-129598775 (+) |  | 2.370 | 4.33e-04 | 3.52e-03 |
| ENSMUSG00000046841 | Ckap4 | protein\_coding | 10:84526305-84534062 (-) |  | -0.325 | 4.37e-04 | 3.54e-03 |
| ENSMUSG00000031495 | Cd209d | protein\_coding | 8:3871824-3878555 (-) |  | 2.700 | 4.37e-04 | 3.54e-03 |
| ENSMUSG00000060441 | Trim5 | protein\_coding | 7:104263386-104288094 (-) |  | 0.491 | 4.38e-04 | 3.55e-03 |
| ENSMUSG00000029705 | Cux1 | protein\_coding | 5:136248135-136567490 (-) |  | -0.309 | 4.39e-04 | 3.55e-03 |
| ENSMUSG00000019822 | Smpd2 | protein\_coding | 10:41485642-41490369 (-) |  | 0.411 | 4.39e-04 | 3.55e-03 |
| ENSMUSG00000089872 | Rps6kc1 | protein\_coding | 1:190700202-190911770 (-) |  | 0.384 | 4.40e-04 | 3.56e-03 |
| ENSMUSG00000022489 | Pde1b | protein\_coding | 15:103503034-103530052 (+) |  | 0.585 | 4.40e-04 | 3.56e-03 |
| ENSMUSG00000032842 | Abcc10 | protein\_coding | 17:46303221-46328352 (-) |  | 0.612 | 4.42e-04 | 3.57e-03 |
| ENSMUSG00000025584 | Pde8a | protein\_coding | 7:81213596-81334533 (+) |  | -0.436 | 4.45e-04 | 3.59e-03 |
| ENSMUSG00000032349 | Elovl5 | protein\_coding | 9:77917364-77984519 (+) |  | -0.272 | 4.45e-04 | 3.59e-03 |
| ENSMUSG00000022555 | Dgat1 | protein\_coding | 15:76502015-76511953 (-) |  | 0.380 | 4.50e-04 | 3.63e-03 |
| ENSMUSG00000032560 | Dnajc13 | protein\_coding | 9:104151282-104262930 (-) |  | -0.361 | 4.51e-04 | 3.63e-03 |
| ENSMUSG00000020739 | Nup85 | protein\_coding | 11:115564434-115583985 (+) |  | -0.344 | 4.51e-04 | 3.63e-03 |
| ENSMUSG00000025959 | Klf7 | protein\_coding | 1:64029447-64122282 (-) |  | -0.478 | 4.55e-04 | 3.66e-03 |
| ENSMUSG00000066232 | Ipo7 | protein\_coding | 7:110018274-110056609 (+) |  | -0.234 | 4.60e-04 | 3.70e-03 |
| ENSMUSG00000021427 | Ssr1 | protein\_coding | 13:37966605-37994217 (-) |  | -0.277 | 4.61e-04 | 3.70e-03 |
| ENSMUSG00000025860 | Xiap | protein\_coding | X:42059679-42109656 (+) |  | 0.230 | 4.62e-04 | 3.71e-03 |
| ENSMUSG00000001020 | S100a4 | protein\_coding | 3:90603771-90606045 (+) |  | 0.474 | 4.62e-04 | 3.71e-03 |
| ENSMUSG00000026393 | Nek7 | protein\_coding | 1:138482875-138620141 (-) |  | 0.265 | 4.63e-04 | 3.71e-03 |
| ENSMUSG00000029246 | Ppat | protein\_coding | 5:76913249-76951578 (-) |  | -0.414 | 4.64e-04 | 3.72e-03 |
| ENSMUSG00000037447 | Arid5a | protein\_coding | 1:36307733-36324029 (+) |  | -0.321 | 4.65e-04 | 3.72e-03 |
| ENSMUSG00000048534 | Jaml | protein\_coding | 9:45079183-45108534 (+) |  | -0.538 | 4.65e-04 | 3.72e-03 |
| ENSMUSG00000030498 | Gas2 | protein\_coding | 7:51862015-51994975 (+) |  | 0.638 | 4.68e-04 | 3.74e-03 |
| ENSMUSG00000018042 | Cyb5r3 | protein\_coding | 15:83153494-83172592 (-) |  | 0.319 | 4.69e-04 | 3.75e-03 |
| ENSMUSG00000042489 | Clspn | protein\_coding | 4:126556935-126593903 (+) |  | -0.425 | 4.71e-04 | 3.76e-03 |
| ENSMUSG00000023992 | Trem2 | protein\_coding | 17:48346401-48354147 (+) |  | -0.393 | 4.72e-04 | 3.77e-03 |
| ENSMUSG00000033970 | Rfc3 | protein\_coding | 5:151642756-151651242 (-) |  | -0.381 | 4.72e-04 | 3.77e-03 |
| ENSMUSG00000040410 | Fbxl4 | protein\_coding | 4:22357543-22434091 (+) |  | 0.469 | 4.72e-04 | 3.77e-03 |
| ENSMUSG00000039187 | Fanci | protein\_coding | 7:79391929-79450264 (+) |  | -0.438 | 4.76e-04 | 3.79e-03 |
| ENSMUSG00000022884 | Eif4a2 | protein\_coding | 16:23107444-23114136 (+) |  | 0.387 | 4.78e-04 | 3.81e-03 |
| ENSMUSG00000026627 | Pacc1 | protein\_coding | 1:191325912-191350914 (+) |  | 0.472 | 4.79e-04 | 3.81e-03 |
| ENSMUSG00000041538 | H2-Ob | protein\_coding | 17:34238903-34254414 (+) |  | 1.360 | 4.81e-04 | 3.83e-03 |
| ENSMUSG00000027774 | Gfm1 | protein\_coding | 3:67430096-67476529 (+) |  | -0.354 | 4.84e-04 | 3.85e-03 |
| ENSMUSG00000022594 | Lynx1 | protein\_coding | 15:74747852-74753046 (-) |  | 0.724 | 4.85e-04 | 3.85e-03 |
| ENSMUSG00000004665 | Cnn2 | protein\_coding | 10:79988584-79996062 (+) |  | 0.355 | 4.92e-04 | 3.91e-03 |
| ENSMUSG00000035227 | Spcs2 | protein\_coding | 7:99837569-99863462 (-) |  | -0.286 | 4.95e-04 | 3.93e-03 |
| ENSMUSG00000026766 | Mmadhc | protein\_coding | 2:50279881-50296801 (-) |  | -0.331 | 4.95e-04 | 3.93e-03 |
| ENSMUSG00000024174 | Pot1b | protein\_coding | 17:55651951-55712628 (-) |  | 0.483 | 4.96e-04 | 3.93e-03 |
| ENSMUSG00000019699 | Akt3 | protein\_coding | 1:177020073-177258203 (-) |  | 0.326 | 4.99e-04 | 3.95e-03 |
| ENSMUSG00000046756 | Mrps7 | protein\_coding | 11:115603925-115608036 (+) |  | -0.366 | 5.00e-04 | 3.96e-03 |
| ENSMUSG00000021384 | Susd3 | protein\_coding | 13:49230690-49248706 (-) |  | 0.361 | 5.02e-04 | 3.97e-03 |
| ENSMUSG00000030423 | Pop4 | protein\_coding | 7:38261996-38271423 (-) |  | -0.381 | 5.02e-04 | 3.97e-03 |
| ENSMUSG00000032497 | Lrrfip2 | protein\_coding | 9:111117592-111225668 (+) |  | 0.261 | 5.03e-04 | 3.97e-03 |
| ENSMUSG00000036959 | Bcorl1 | protein\_coding | X:48341358-48408049 (+) |  | 0.546 | 5.04e-04 | 3.97e-03 |
| ENSMUSG00000070319 | Eif3g | protein\_coding | 9:20894349-20898623 (-) |  | -0.240 | 5.04e-04 | 3.97e-03 |
| ENSMUSG00000095609 | Gm21188 | protein\_coding | 13:120034605-120052194 (-) |  | 0.325 | 5.04e-04 | 3.98e-03 |
| ENSMUSG00000070873 | Lilra5 | protein\_coding | 7:4237754-4243463 (+) |  | 3.630 | 5.13e-04 | 4.05e-03 |
| ENSMUSG00000028902 | Sf3a3 | protein\_coding | 4:124714776-124732460 (+) |  | -0.294 | 5.17e-04 | 4.07e-03 |
| ENSMUSG00000003546 | Klc4 | protein\_coding | 17:46630624-46646022 (-) |  | 0.426 | 5.18e-04 | 4.08e-03 |
| ENSMUSG00000026798 | Coq4 | protein\_coding | 2:29787493-29797935 (+) |  | -0.883 | 5.21e-04 | 4.10e-03 |
| ENSMUSG00000032042 | Srpr | protein\_coding | 9:35200175-35247973 (+) |  | -0.265 | 5.21e-04 | 4.10e-03 |
| ENSMUSG00000045751 | Mms22l | protein\_coding | 4:24496451-24602950 (+) |  | -0.434 | 5.25e-04 | 4.12e-03 |
| ENSMUSG00000022789 | Dnm1l | protein\_coding | 16:16312230-16358959 (-) |  | -0.290 | 5.26e-04 | 4.13e-03 |
| ENSMUSG00000034765 | Dusp5 | protein\_coding | 19:53529109-53542431 (+) |  | 1.490 | 5.26e-04 | 4.13e-03 |
| ENSMUSG00000118164 | Gm20570 | processed\_pseudogene | 18:75767143-75767274 (+) |  | 0.861 | 5.26e-04 | 4.13e-03 |
| ENSMUSG00000026385 | Dbi | protein\_coding | 1:120113280-120121078 (-) |  | -0.371 | 5.27e-04 | 4.13e-03 |
| ENSMUSG00000026873 | Phf19 | protein\_coding | 2:34893757-34914026 (-) |  | -0.387 | 5.28e-04 | 4.14e-03 |
| ENSMUSG00000006920 | Ezh1 | protein\_coding | 11:101191115-101226463 (-) |  | 0.336 | 5.33e-04 | 4.17e-03 |
| ENSMUSG00000020780 | Srp68 | protein\_coding | 11:116245166-116274217 (-) |  | -0.262 | 5.34e-04 | 4.18e-03 |
| ENSMUSG00000001120 | Pcbp3 | protein\_coding | 10:76761857-76961887 (-) |  | 0.858 | 5.34e-04 | 4.18e-03 |
| ENSMUSG00000018425 | Dhx40 | protein\_coding | 11:86768846-86807746 (-) |  | 0.312 | 5.39e-04 | 4.21e-03 |
| ENSMUSG00000035828 | Pim3 | protein\_coding | 15:88862186-88865726 (+) |  | 0.492 | 5.42e-04 | 4.23e-03 |
| ENSMUSG00000052331 | Ankrd44 | protein\_coding | 1:54645340-54926387 (-) |  | 0.291 | 5.46e-04 | 4.26e-03 |
| ENSMUSG00000024989 | Cep55 | protein\_coding | 19:38055011-38074423 (+) |  | -0.323 | 5.47e-04 | 4.27e-03 |
| ENSMUSG00000026822 | Lcn2 | protein\_coding | 2:32384633-32388252 (-) |  | -1.660 | 5.47e-04 | 4.27e-03 |
| ENSMUSG00000039629 | Strip2 | protein\_coding | 6:29917012-29959681 (+) |  | -0.941 | 5.48e-04 | 4.27e-03 |
| ENSMUSG00000079429 | Mroh2a | protein\_coding | 1:88226986-88262289 (+) |  | -0.432 | 5.50e-04 | 4.28e-03 |
| ENSMUSG00000057315 | Arhgap24 | protein\_coding | 5:102481391-102897937 (+) |  | -0.489 | 5.50e-04 | 4.28e-03 |
| ENSMUSG00000021876 | Rnase4 | protein\_coding | 14:51091077-51106151 (+) |  | 0.511 | 5.50e-04 | 4.28e-03 |
| ENSMUSG00000031657 | Heatr3 | protein\_coding | 8:88137855-88172027 (+) |  | -0.389 | 5.52e-04 | 4.29e-03 |
| ENSMUSG00000006310 | Zbtb32 | protein\_coding | 7:30589681-30598909 (-) |  | -1.090 | 5.54e-04 | 4.30e-03 |
| ENSMUSG00000040325 | Dcaf1 | protein\_coding | 9:106821874-106880992 (+) |  | -0.486 | 5.55e-04 | 4.31e-03 |
| ENSMUSG00000028156 | Eif4e | protein\_coding | 3:138526179-138559696 (+) |  | -0.305 | 5.56e-04 | 4.32e-03 |
| ENSMUSG00000020185 | E2f7 | protein\_coding | 10:110745439-110787384 (+) |  | -0.502 | 5.57e-04 | 4.32e-03 |
| ENSMUSG00000044749 | Abca6 | protein\_coding | 11:110176820-110251776 (-) |  | 1.190 | 5.58e-04 | 4.32e-03 |
| ENSMUSG00000039879 | Heca | protein\_coding | 10:17868612-17948067 (-) |  | 0.355 | 5.59e-04 | 4.33e-03 |
| ENSMUSG00000056116 | H2-T22 | protein\_coding | 17:36037128-36042747 (-) |  | -0.345 | 5.60e-04 | 4.33e-03 |
| ENSMUSG00000097328 | Tnfsf12 | protein\_coding | 11:69686250-69695849 (-) |  | 0.627 | 5.60e-04 | 4.33e-03 |
| ENSMUSG00000002944 | Cd36 | protein\_coding | 5:17781690-17888801 (-) |  | 2.530 | 5.62e-04 | 4.34e-03 |
| ENSMUSG00000021660 | Btf3 | protein\_coding | 13:98309896-98317006 (-) |  | -0.281 | 5.64e-04 | 4.35e-03 |
| ENSMUSG00000022892 | App | protein\_coding | 16:84949685-85173766 (-) |  | -0.295 | 5.64e-04 | 4.36e-03 |
| ENSMUSG00000098188 | Sowahc | protein\_coding | 10:59221953-59226434 (+) |  | 0.358 | 5.65e-04 | 4.36e-03 |
| ENSMUSG00000027067 | Ssrp1 | protein\_coding | 2:85037234-85047109 (+) |  | -0.257 | 5.65e-04 | 4.36e-03 |
| ENSMUSG00000020936 | Nmt1 | protein\_coding | 11:103028190-103068912 (+) |  | -0.216 | 5.65e-04 | 4.36e-03 |
| ENSMUSG00000072809 | 9330160F10Rik | lncRNA | 11:69057718-69060483 (-) |  | 0.779 | 5.66e-04 | 4.36e-03 |
| ENSMUSG00000053040 | Aph1c | protein\_coding | 9:66814994-66834726 (-) |  | 0.388 | 5.66e-04 | 4.36e-03 |
| ENSMUSG00000027823 | Gmps | protein\_coding | 3:63976106-64022579 (+) |  | -0.264 | 5.68e-04 | 4.37e-03 |
| ENSMUSG00000038145 | Snrk | protein\_coding | 9:122117266-122169702 (+) |  | 0.280 | 5.70e-04 | 4.38e-03 |
| ENSMUSG00000038252 | Ncapd2 | protein\_coding | 6:125168007-125191701 (-) |  | -0.370 | 5.70e-04 | 4.38e-03 |
| ENSMUSG00000000711 | Rab5b | protein\_coding | 10:128677175-128696264 (-) |  | 0.242 | 5.71e-04 | 4.39e-03 |
| ENSMUSG00000044827 | Tlr1 | protein\_coding | 5:64924679-64933563 (-) |  | -0.444 | 5.72e-04 | 4.39e-03 |
| ENSMUSG00000034764 | 1700006J14Rik | lncRNA | 10:120364157-120384336 (+) |  | 1.150 | 5.72e-04 | 4.39e-03 |
| ENSMUSG00000020522 | Mfap3 | protein\_coding | 11:57518664-57533815 (+) |  | 0.285 | 5.74e-04 | 4.40e-03 |
| ENSMUSG00000064326 | Siva1 | protein\_coding | 12:112644679-112649149 (+) |  | -0.366 | 5.78e-04 | 4.43e-03 |
| ENSMUSG00000048578 | Mlec | protein\_coding | 5:115142981-115158179 (-) |  | -0.272 | 5.78e-04 | 4.43e-03 |
| ENSMUSG00000066037 | Hnrnpr | protein\_coding | 4:136310942-136359447 (+) |  | -0.231 | 5.80e-04 | 4.44e-03 |
| ENSMUSG00000032434 | Cmtm6 | protein\_coding | 9:114731116-114749344 (+) |  | 0.226 | 5.80e-04 | 4.44e-03 |
| ENSMUSG00000051220 | Ercc6l | protein\_coding | X:102141716-102157091 (-) |  | -0.352 | 5.81e-04 | 4.44e-03 |
| ENSMUSG00000015776 | Med22 | protein\_coding | 2:26905262-26910677 (-) |  | 0.306 | 5.83e-04 | 4.45e-03 |
| ENSMUSG00000028159 | Dapp1 | protein\_coding | 3:137931007-137981545 (-) |  | 0.307 | 5.84e-04 | 4.46e-03 |
| ENSMUSG00000034111 | Tmed8 | protein\_coding | 12:87166237-87200454 (-) |  | -0.380 | 5.85e-04 | 4.46e-03 |
| ENSMUSG00000025545 | Clybl | protein\_coding | 14:122181704-122402234 (+) |  | -0.613 | 5.92e-04 | 4.51e-03 |
| ENSMUSG00000057388 | Mrpl18 | protein\_coding | 17:12911349-12916345 (-) |  | -0.324 | 5.93e-04 | 4.52e-03 |
| ENSMUSG00000021806 | Nid2 | protein\_coding | 14:19751265-19811787 (+) |  | -1.060 | 5.95e-04 | 4.53e-03 |
| ENSMUSG00000030830 | Itgal | protein\_coding | 7:127296260-127335138 (+) |  | 0.203 | 5.99e-04 | 4.56e-03 |
| ENSMUSG00000029171 | Pgm2 | protein\_coding | 5:64092950-64128351 (+) |  | -0.306 | 6.00e-04 | 4.57e-03 |
| ENSMUSG00000035202 | Lars2 | protein\_coding | 9:123366927-123462666 (+) |  | -0.475 | 6.02e-04 | 4.58e-03 |
| ENSMUSG00000028937 | Acot7 | protein\_coding | 4:152178134-152271855 (+) |  | -0.309 | 6.06e-04 | 4.61e-03 |
| ENSMUSG00000001739 | Cldn15 | protein\_coding | 5:136966616-136975858 (+) |  | -0.460 | 6.08e-04 | 4.62e-03 |
| ENSMUSG00000043953 | Ccrl2 | protein\_coding | 9:111054486-111057519 (-) |  | 1.110 | 6.10e-04 | 4.63e-03 |
| ENSMUSG00000024011 | Pi16 | protein\_coding | 17:29317680-29330593 (+) |  | 0.256 | 6.11e-04 | 4.64e-03 |
| ENSMUSG00000017176 | Nt5c3b | protein\_coding | 11:100422321-100441808 (-) |  | -0.879 | 6.12e-04 | 4.64e-03 |
| ENSMUSG00000005107 | Slc2a9 | protein\_coding | 5:38349273-38503143 (-) |  | 0.465 | 6.12e-04 | 4.64e-03 |
| ENSMUSG00000030603 | Psmc4 | protein\_coding | 7:28041707-28050101 (-) |  | -0.301 | 6.13e-04 | 4.64e-03 |
| ENSMUSG00000023915 | Tnfrsf21 | protein\_coding | 17:43016555-43089189 (+) |  | 0.269 | 6.16e-04 | 4.66e-03 |
| ENSMUSG00000081219 | Bambi-ps1 | processed\_pseudogene | 2:122466935-122467526 (-) |  | -1.000 | 6.16e-04 | 4.66e-03 |
| ENSMUSG00000024286 | Ccny | protein\_coding | 18:9312304-9450154 (-) |  | 0.265 | 6.20e-04 | 4.68e-03 |
| ENSMUSG00000027956 | Tmem144 | protein\_coding | 3:79812564-79852773 (-) |  | -0.883 | 6.21e-04 | 4.69e-03 |
| ENSMUSG00000087403 | Kantr | protein\_coding | X:152294828-152327495 (-) |  | 0.479 | 6.21e-04 | 4.69e-03 |
| ENSMUSG00000037907 | Ankrd13b | protein\_coding | 11:77470485-77489678 (-) |  | 0.720 | 6.22e-04 | 4.69e-03 |
| ENSMUSG00000057236 | Rbbp4 | protein\_coding | 4:129307100-129335370 (-) |  | -0.242 | 6.22e-04 | 4.69e-03 |
| ENSMUSG00000024758 | Rtn3 | protein\_coding | 19:7425901-7483281 (-) |  | -0.262 | 6.24e-04 | 4.70e-03 |
| ENSMUSG00000054203 | Ifi205 | protein\_coding | 1:174011998-174031810 (-) |  | 1.070 | 6.26e-04 | 4.72e-03 |
| ENSMUSG00000025969 | Nrp2 | protein\_coding | 1:62703285-62818695 (+) |  | -0.366 | 6.30e-04 | 4.74e-03 |
| ENSMUSG00000049744 | Arhgap15 | protein\_coding | 2:43748824-44395953 (+) |  | 0.476 | 6.31e-04 | 4.75e-03 |
| ENSMUSG00000030335 | Mrpl51 | protein\_coding | 6:125191801-125196269 (+) |  | -0.363 | 6.39e-04 | 4.80e-03 |
| ENSMUSG00000014077 | Chp1 | protein\_coding | 2:119547697-119587027 (+) |  | -0.247 | 6.40e-04 | 4.81e-03 |
| ENSMUSG00000071337 | Tia1 | protein\_coding | 6:86404219-86433405 (+) |  | 0.304 | 6.41e-04 | 4.82e-03 |
| ENSMUSG00000033762 | Recql4 | protein\_coding | 15:76703553-76710548 (-) |  | -0.577 | 6.43e-04 | 4.83e-03 |
| ENSMUSG00000059248 | Sept9 | protein\_coding | 11:117199661-117362325 (+) |  | 0.283 | 6.44e-04 | 4.83e-03 |
| ENSMUSG00000063480 | Snu13 | protein\_coding | 15:82040525-82047598 (-) |  | -0.348 | 6.44e-04 | 4.83e-03 |
| ENSMUSG00000049037 | Clec4a1 | protein\_coding | 6:122921848-122934619 (+) |  | 0.405 | 6.48e-04 | 4.86e-03 |
| ENSMUSG00000103041 | Gm37305 | lncRNA | 3:65957758-65962036 (+) |  | 0.491 | 6.49e-04 | 4.86e-03 |
| ENSMUSG00000054693 | Adam10 | protein\_coding | 9:70678997-70780229 (+) |  | -0.258 | 6.52e-04 | 4.88e-03 |
| ENSMUSG00000110331 | Nudc-ps1 | processed\_pseudogene | 8:29286431-29286981 (-) |  | -0.518 | 6.53e-04 | 4.88e-03 |
| ENSMUSG00000054808 | Actn4 | protein\_coding | 7:28893248-28962340 (-) |  | -0.263 | 6.53e-04 | 4.89e-03 |
| ENSMUSG00000027374 | Mrps5 | protein\_coding | 2:127587222-127606829 (+) |  | -0.415 | 6.54e-04 | 4.89e-03 |
| ENSMUSG00000032437 | Stt3b | protein\_coding | 9:115242581-115310421 (-) |  | -0.283 | 6.59e-04 | 4.92e-03 |
| ENSMUSG00000020840 | Blmh | protein\_coding | 11:76924809-76987379 (+) |  | -0.269 | 6.60e-04 | 4.92e-03 |
| ENSMUSG00000003299 | Mrpl4 | protein\_coding | 9:21002738-21008839 (+) |  | -0.265 | 6.63e-04 | 4.95e-03 |
| ENSMUSG00000042207 | Kdm5b | protein\_coding | 1:134560171-134635285 (+) |  | 0.637 | 6.64e-04 | 4.95e-03 |
| ENSMUSG00000030629 | Zfand6 | protein\_coding | 7:84613766-84689959 (-) |  | 0.321 | 6.66e-04 | 4.96e-03 |
| ENSMUSG00000022946 | Dop1b | protein\_coding | 16:93711904-93810590 (+) |  | 0.390 | 6.67e-04 | 4.97e-03 |
| ENSMUSG00000021993 | Mipep | protein\_coding | 14:60784573-60905478 (+) |  | -0.493 | 6.67e-04 | 4.97e-03 |
| ENSMUSG00000108414 | Snhg1 | lncRNA | 19:8723475-8726443 (+) |  | -0.367 | 6.68e-04 | 4.97e-03 |
| ENSMUSG00000021108 | Prkch | protein\_coding | 12:73584796-73778185 (+) |  | 0.372 | 6.70e-04 | 4.98e-03 |
| ENSMUSG00000024856 | Cdk2ap2 | protein\_coding | 19:4097182-4099019 (+) |  | 0.315 | 6.71e-04 | 4.98e-03 |
| ENSMUSG00000041459 | Tardbp | protein\_coding | 4:148612382-148627019 (-) |  | -0.250 | 6.72e-04 | 4.99e-03 |
| ENSMUSG00000040028 | Elavl1 | protein\_coding | 8:4285382-4325413 (-) |  | -0.285 | 6.80e-04 | 5.04e-03 |
| ENSMUSG00000079164 | Tlr5 | protein\_coding | 1:182954788-182976044 (+) |  | -0.557 | 6.82e-04 | 5.05e-03 |
| ENSMUSG00000085882 | 2610507I01Rik | lncRNA | 11:59199836-59202385 (-) |  | 0.451 | 6.82e-04 | 5.06e-03 |
| ENSMUSG00000067878 | Map7d3 | protein\_coding | X:56797858-56822326 (-) |  | 0.562 | 6.88e-04 | 5.09e-03 |
| ENSMUSG00000024833 | Pola2 | protein\_coding | 19:5940542-5964202 (-) |  | -0.338 | 6.96e-04 | 5.15e-03 |
| ENSMUSG00000000149 | Gna12 | protein\_coding | 5:140758408-140830431 (-) |  | 0.280 | 6.97e-04 | 5.15e-03 |
| ENSMUSG00000028458 | Tesk1 | protein\_coding | 4:43441939-43448064 (+) |  | 0.468 | 6.97e-04 | 5.16e-03 |
| ENSMUSG00000021109 | Hif1a | protein\_coding | 12:73901375-73947530 (+) |  | -0.426 | 6.99e-04 | 5.17e-03 |
| ENSMUSG00000028041 | Adam15 | protein\_coding | 3:89338542-89349996 (-) |  | -0.273 | 7.00e-04 | 5.17e-03 |
| ENSMUSG00000012443 | Kif11 | protein\_coding | 19:37376403-37421859 (+) |  | -0.264 | 7.04e-04 | 5.20e-03 |
| ENSMUSG00000032633 | Flcn | protein\_coding | 11:59791408-59810016 (-) |  | 0.295 | 7.08e-04 | 5.22e-03 |
| ENSMUSG00000041891 | Lman1 | protein\_coding | 18:65980738-66022580 (-) |  | -0.295 | 7.08e-04 | 5.22e-03 |
| ENSMUSG00000110018 | 5430437J10Rik | transcribed\_processed\_pseudogene | 15:5496266-5594984 (+) |  | 1.100 | 7.09e-04 | 5.22e-03 |
| ENSMUSG00000033192 | Lpcat2 | protein\_coding | 8:92855339-92919279 (+) |  | 0.516 | 7.09e-04 | 5.22e-03 |
| ENSMUSG00000028107 | Tars2 | protein\_coding | 3:95739976-95760206 (-) |  | -0.357 | 7.11e-04 | 5.23e-03 |
| ENSMUSG00000056054 | S100a8 | protein\_coding | 3:90668978-90670035 (+) |  | -1.470 | 7.12e-04 | 5.23e-03 |
| ENSMUSG00000061904 | Slc25a3 | protein\_coding | 10:91116574-91124059 (-) |  | -0.252 | 7.12e-04 | 5.23e-03 |
| ENSMUSG00000030346 | Rad51ap1 | protein\_coding | 6:126923050-126939587 (-) |  | -0.393 | 7.18e-04 | 5.28e-03 |
| ENSMUSG00000024953 | Prdx5 | protein\_coding | 19:6906697-6910106 (-) |  | 0.471 | 7.22e-04 | 5.30e-03 |
| ENSMUSG00000026321 | Tnfrsf11a | protein\_coding | 1:105780718-105847981 (+) |  | 0.500 | 7.23e-04 | 5.31e-03 |
| ENSMUSG00000049588 | Ccdc69 | protein\_coding | 11:55049731-55078131 (-) |  | 0.361 | 7.24e-04 | 5.31e-03 |
| ENSMUSG00000021288 | Klc1 | protein\_coding | 12:111758849-111807844 (+) |  | 0.258 | 7.26e-04 | 5.32e-03 |
| ENSMUSG00000026807 | Ak8 | protein\_coding | 2:28700164-28813165 (+) |  | 0.678 | 7.29e-04 | 5.34e-03 |
| ENSMUSG00000055044 | Pdlim1 | protein\_coding | 19:40221173-40271842 (-) |  | 0.254 | 7.34e-04 | 5.37e-03 |
| ENSMUSG00000031848 | Lsm4 | protein\_coding | 8:70673248-70678752 (+) |  | -0.331 | 7.37e-04 | 5.39e-03 |
| ENSMUSG00000117284 | Gm7072 | protein\_coding | 17:22285871-22314837 (-) |  | 0.400 | 7.43e-04 | 5.43e-03 |
| ENSMUSG00000034773 | BC030867 | protein\_coding | 11:102248882-102265187 (+) |  | -0.590 | 7.45e-04 | 5.45e-03 |
| ENSMUSG00000036948 | Map11 | protein\_coding | 5:138259656-138264033 (-) |  | 0.245 | 7.46e-04 | 5.45e-03 |
| ENSMUSG00000028757 | Ddost | protein\_coding | 4:138304730-138312628 (+) |  | -0.312 | 7.47e-04 | 5.45e-03 |
| ENSMUSG00000026712 | Mrc1 | protein\_coding | 2:14229392-14332057 (+) |  | 0.829 | 7.51e-04 | 5.48e-03 |
| ENSMUSG00000034663 | Bmp2k | protein\_coding | 5:96997689-97091867 (+) |  | -0.345 | 7.55e-04 | 5.51e-03 |
| ENSMUSG00000018500 | Adora2b | protein\_coding | 11:62248984-62266453 (+) |  | -0.326 | 7.55e-04 | 5.51e-03 |
| ENSMUSG00000078636 | Gm7336 | processed\_pseudogene | 7:51746647-51747878 (+) |  | -0.838 | 7.59e-04 | 5.53e-03 |
| ENSMUSG00000024962 | Vegfb | protein\_coding | 19:6982473-6987651 (-) |  | -0.682 | 7.62e-04 | 5.55e-03 |
| ENSMUSG00000036106 | Prr5 | protein\_coding | 15:84669620-84703673 (+) |  | 0.673 | 7.62e-04 | 5.55e-03 |
| ENSMUSG00000045636 | Mtus1 | protein\_coding | 8:40990914-41133726 (-) |  | -0.245 | 7.64e-04 | 5.56e-03 |
| ENSMUSG00000031715 | Smarca5 | protein\_coding | 8:80698507-80739497 (-) |  | -0.241 | 7.65e-04 | 5.56e-03 |
| ENSMUSG00000052821 | Cysltr1 | protein\_coding | X:106574346-106603679 (-) |  | -0.482 | 7.65e-04 | 5.56e-03 |
| ENSMUSG00000020101 | Vsir | protein\_coding | 10:60346851-60372684 (+) |  | 0.240 | 7.67e-04 | 5.57e-03 |
| ENSMUSG00000056076 | Eif3b | protein\_coding | 5:140419328-140443360 (+) |  | -0.319 | 7.74e-04 | 5.62e-03 |
| ENSMUSG00000025428 | Atp5a1 | protein\_coding | 18:77773729-77782869 (+) |  | -0.258 | 7.74e-04 | 5.62e-03 |
| ENSMUSG00000030082 | Sec61a1 | protein\_coding | 6:88503579-88518905 (-) |  | -0.299 | 7.79e-04 | 5.65e-03 |
| ENSMUSG00000038683 | Pak1ip1 | protein\_coding | 13:41001023-41013015 (+) |  | -0.260 | 7.80e-04 | 5.65e-03 |
| ENSMUSG00000053119 | Chmp3 | protein\_coding | 6:71543797-71582609 (+) |  | 0.332 | 7.81e-04 | 5.65e-03 |
| ENSMUSG00000072596 | Ear2 | protein\_coding | 14:44102654-44103534 (+) |  | 0.951 | 7.84e-04 | 5.68e-03 |
| ENSMUSG00000035941 | Ibtk | protein\_coding | 9:85687360-85749334 (-) |  | -0.376 | 7.85e-04 | 5.68e-03 |
| ENSMUSG00000034187 | Nsf | protein\_coding | 11:103821782-103954056 (-) |  | -0.297 | 7.87e-04 | 5.69e-03 |
| ENSMUSG00000016534 | Lamp2 | protein\_coding | X:38401357-38456454 (-) |  | -0.347 | 7.88e-04 | 5.70e-03 |
| ENSMUSG00000034361 | Cpne2 | protein\_coding | 8:94532990-94570531 (+) |  | -0.408 | 7.92e-04 | 5.72e-03 |
| ENSMUSG00000015536 | Mocs2 | protein\_coding | 13:114818236-114832275 (+) |  | 0.380 | 7.94e-04 | 5.73e-03 |
| ENSMUSG00000034206 | Polq | protein\_coding | 16:37011786-37095417 (+) |  | -0.433 | 7.97e-04 | 5.75e-03 |
| ENSMUSG00000036078 | Sigmar1 | protein\_coding | 4:41738493-41756157 (-) |  | -0.334 | 8.01e-04 | 5.77e-03 |
| ENSMUSG00000000568 | Hnrnpd | protein\_coding | 5:99955935-99978938 (-) |  | -0.280 | 8.03e-04 | 5.79e-03 |
| ENSMUSG00000017861 | Mybl2 | protein\_coding | 2:163054687-163084688 (+) |  | -0.484 | 8.04e-04 | 5.79e-03 |
| ENSMUSG00000035172 | Plekhh3 | protein\_coding | 11:101162679-101171351 (-) |  | 0.573 | 8.07e-04 | 5.81e-03 |
| ENSMUSG00000024165 | Jpt2 | protein\_coding | 17:24937419-24960689 (-) |  | -0.360 | 8.07e-04 | 5.81e-03 |
| ENSMUSG00000023456 | Tpi1 | protein\_coding | 6:124810586-124814296 (-) |  | -0.385 | 8.08e-04 | 5.81e-03 |
| ENSMUSG00000030528 | Blm | protein\_coding | 7:80454733-80535119 (-) |  | -0.333 | 8.10e-04 | 5.82e-03 |
| ENSMUSG00000036944 | Tmem71 | protein\_coding | 15:66526212-66561103 (-) |  | 0.315 | 8.10e-04 | 5.82e-03 |
| ENSMUSG00000029388 | Eif2b1 | protein\_coding | 5:124570213-124579131 (-) |  | -0.342 | 8.12e-04 | 5.83e-03 |
| ENSMUSG00000066595 | Flvcr1 | protein\_coding | 1:191005847-191026158 (-) |  | 0.279 | 8.12e-04 | 5.83e-03 |
| ENSMUSG00000032596 | Uba7 | protein\_coding | 9:107975505-107984060 (+) |  | 0.384 | 8.12e-04 | 5.83e-03 |
| ENSMUSG00000075420 | Smim6 | protein\_coding | 11:115912017-115913920 (+) |  | 1.040 | 8.27e-04 | 5.93e-03 |
| ENSMUSG00000044068 | Zrsr1 | protein\_coding | 11:22972005-22976496 (+) |  | 0.535 | 8.29e-04 | 5.94e-03 |
| ENSMUSG00000045078 | Rnf216 | protein\_coding | 5:142990893-143112994 (-) |  | 0.228 | 8.29e-04 | 5.94e-03 |
| ENSMUSG00000078861 | Zfp931 | protein\_coding | 2:178067695-178078476 (-) |  | 0.919 | 8.30e-04 | 5.94e-03 |
| ENSMUSG00000016382 | Pls3 | protein\_coding | X:75785654-75875182 (-) |  | -0.778 | 8.31e-04 | 5.94e-03 |
| ENSMUSG00000069270 | H2ac6 | protein\_coding | 13:23681467-23683948 (-) |  | 1.460 | 8.31e-04 | 5.94e-03 |
| ENSMUSG00000031605 | Klhl2 | protein\_coding | 8:64739675-64850017 (-) |  | -0.386 | 8.32e-04 | 5.95e-03 |
| ENSMUSG00000025190 | Got1 | protein\_coding | 19:43499752-43524605 (-) |  | -0.542 | 8.36e-04 | 5.97e-03 |
| ENSMUSG00000024056 | Ndc80 | protein\_coding | 17:71496100-71526857 (-) |  | -0.289 | 8.40e-04 | 6.00e-03 |
| ENSMUSG00000045538 | Ddx28 | protein\_coding | 8:106009621-106011882 (-) |  | -0.431 | 8.45e-04 | 6.03e-03 |
| ENSMUSG00000029147 | Ppm1g | protein\_coding | 5:31202664-31220687 (-) |  | -0.277 | 8.46e-04 | 6.03e-03 |
| ENSMUSG00000017837 | Nkiras2 | protein\_coding | 11:100619244-100627607 (+) |  | 0.303 | 8.47e-04 | 6.03e-03 |
| ENSMUSG00000034430 | Zxdc | protein\_coding | 6:90369492-90403490 (+) |  | 0.360 | 8.51e-04 | 6.06e-03 |
| ENSMUSG00000051355 | Commd1 | protein\_coding | 11:22896136-22982382 (-) |  | -0.368 | 8.54e-04 | 6.07e-03 |
| ENSMUSG00000092528 | Nlrp1c-ps | transcribed\_unprocessed\_pseudogene | 11:71242430-71285232 (-) |  | 0.963 | 8.57e-04 | 6.10e-03 |
| ENSMUSG00000026925 | Inpp5e | protein\_coding | 2:26396249-26409203 (-) |  | 0.352 | 8.60e-04 | 6.11e-03 |
| ENSMUSG00000025870 | Arl10 | protein\_coding | 13:54575015-54581128 (+) |  | 0.442 | 8.63e-04 | 6.13e-03 |
| ENSMUSG00000000440 | Pparg | protein\_coding | 6:115360951-115490399 (+) |  | 1.410 | 8.68e-04 | 6.17e-03 |
| ENSMUSG00000017561 | Crlf3 | protein\_coding | 11:80046493-80080991 (-) |  | 0.256 | 8.71e-04 | 6.18e-03 |
| ENSMUSG00000027952 | Pmvk | protein\_coding | 3:89454541-89469013 (+) |  | -0.483 | 8.79e-04 | 6.23e-03 |
| ENSMUSG00000029810 | Tmem176b | protein\_coding | 6:48833818-48841496 (-) |  | -0.523 | 8.79e-04 | 6.23e-03 |
| ENSMUSG00000029455 | Aldh2 | protein\_coding | 5:121566027-121593824 (-) |  | 0.311 | 8.80e-04 | 6.23e-03 |
| ENSMUSG00000060098 | Prmt7 | protein\_coding | 8:106210936-106252794 (+) |  | -0.353 | 8.80e-04 | 6.23e-03 |
| ENSMUSG00000052688 | Rab7b | protein\_coding | 1:131688695-131715439 (+) |  | -0.376 | 8.86e-04 | 6.27e-03 |
| ENSMUSG00000032116 | Stt3a | protein\_coding | 9:36729344-36767679 (-) |  | -0.251 | 8.96e-04 | 6.33e-03 |
| ENSMUSG00000049709 | Nlrp10 | protein\_coding | 7:108921852-108930178 (-) |  | 1.110 | 8.99e-04 | 6.35e-03 |
| ENSMUSG00000029553 | Tfec | protein\_coding | 6:16833373-16898441 (-) |  | -0.371 | 9.01e-04 | 6.36e-03 |
| ENSMUSG00000003824 | Syce2 | protein\_coding | 8:84872111-84888221 (+) |  | -0.418 | 9.01e-04 | 6.36e-03 |
| ENSMUSG00000056737 | Capg | protein\_coding | 6:72544391-72562983 (+) |  | 0.310 | 9.09e-04 | 6.41e-03 |
| ENSMUSG00000074657 | Kif5a | protein\_coding | 10:127225696-127263348 (-) |  | 1.130 | 9.11e-04 | 6.43e-03 |
| ENSMUSG00000030105 | Arl8b | protein\_coding | 6:108783099-108825278 (+) |  | -0.249 | 9.15e-04 | 6.45e-03 |
| ENSMUSG00000060143 | Gm10076 | lncRNA | 14:105681828-105682211 (+) |  | -0.409 | 9.16e-04 | 6.45e-03 |
| ENSMUSG00000032763 | Ilvbl | protein\_coding | 10:78574346-78584502 (+) |  | 0.339 | 9.28e-04 | 6.53e-03 |
| ENSMUSG00000002897 | Il17ra | protein\_coding | 6:120463247-120487559 (+) |  | 0.241 | 9.28e-04 | 6.53e-03 |
| ENSMUSG00000003623 | Crot | protein\_coding | 5:8966033-8997324 (-) |  | 0.361 | 9.35e-04 | 6.57e-03 |
| ENSMUSG00000020143 | Dock2 | protein\_coding | 11:34226815-34783892 (-) |  | 0.227 | 9.35e-04 | 6.57e-03 |
| ENSMUSG00000045752 | Tssc4 | protein\_coding | 7:143069249-143071093 (+) |  | -0.386 | 9.38e-04 | 6.59e-03 |
| ENSMUSG00000015837 | Sqstm1 | protein\_coding | 11:50199366-50210827 (-) |  | 0.212 | 9.38e-04 | 6.59e-03 |
| ENSMUSG00000029629 | Phf14 | protein\_coding | 6:11907809-12081205 (+) |  | 0.260 | 9.44e-04 | 6.63e-03 |
| ENSMUSG00000087150 | BC064078 | transcribed\_unprocessed\_pseudogene | 6:128992952-129008040 (+) |  | 0.605 | 9.45e-04 | 6.63e-03 |
| ENSMUSG00000014503 | Pkd2l2 | protein\_coding | 18:34408489-34444116 (+) |  | 0.456 | 9.46e-04 | 6.63e-03 |
| ENSMUSG00000018921 | Pelp1 | protein\_coding | 11:70392883-70410031 (-) |  | -0.370 | 9.46e-04 | 6.63e-03 |
| ENSMUSG00000035439 | Haus8 | protein\_coding | 8:71248561-71272934 (-) |  | -0.289 | 9.50e-04 | 6.65e-03 |
| ENSMUSG00000000085 | Scmh1 | protein\_coding | 4:120405281-120530186 (+) |  | 0.439 | 9.54e-04 | 6.68e-03 |
| ENSMUSG00000027405 | Nop56 | protein\_coding | 2:130274430-130279313 (+) |  | -0.301 | 9.55e-04 | 6.68e-03 |
| ENSMUSG00000024764 | Naa40 | protein\_coding | 19:7225668-7241183 (-) |  | -0.290 | 9.61e-04 | 6.72e-03 |
| ENSMUSG00000030156 | Cd69 | protein\_coding | 6:129267325-129275436 (-) |  | -0.937 | 9.69e-04 | 6.77e-03 |
| ENSMUSG00000079293 | Clec7a | polymorphic\_pseudogene | 6:129461591-129472779 (-) |  | 0.495 | 9.71e-04 | 6.78e-03 |
| ENSMUSG00000022500 | Litaf | protein\_coding | 16:10959275-11066157 (-) |  | -0.219 | 9.81e-04 | 6.85e-03 |
| ENSMUSG00000002732 | Fkbp7 | protein\_coding | 2:76663044-76673116 (-) |  | 0.891 | 9.82e-04 | 6.85e-03 |
| ENSMUSG00000022136 | Dnajc3 | protein\_coding | 14:118937976-118981697 (+) |  | -0.241 | 9.82e-04 | 6.85e-03 |
| ENSMUSG00000032803 | Cdv3 | protein\_coding | 9:103353094-103365840 (-) |  | -0.252 | 9.83e-04 | 6.85e-03 |
| ENSMUSG00000030263 | Lrmp | protein\_coding | 6:145115653-145174934 (+) |  | 0.273 | 9.83e-04 | 6.85e-03 |
| ENSMUSG00000033685 | Ucp2 | protein\_coding | 7:100493337-100502020 (+) |  | 0.266 | 9.84e-04 | 6.85e-03 |
| ENSMUSG00000047617 | Paxx | protein\_coding | 2:25455141-25461094 (-) |  | 0.421 | 9.91e-04 | 6.89e-03 |
| ENSMUSG00000090394 | 4930523C07Rik | protein\_coding | 1:160044382-160080208 (+) |  | 0.280 | 9.93e-04 | 6.90e-03 |
| ENSMUSG00000028803 | Nipal3 | protein\_coding | 4:135445420-135495038 (-) |  | 0.349 | 9.99e-04 | 6.94e-03 |
| ENSMUSG00000042079 | Hnrnpf | protein\_coding | 6:117900324-117925622 (+) |  | -0.223 | 1.00e-03 | 6.97e-03 |
| ENSMUSG00000032440 | Tgfbr2 | protein\_coding | 9:116084293-116175360 (-) |  | 0.302 | 1.00e-03 | 6.97e-03 |
| ENSMUSG00000032122 | Slc37a2 | protein\_coding | 9:37227585-37255738 (-) |  | -0.436 | 1.01e-03 | 6.99e-03 |
| ENSMUSG00000097754 | Ptgs2os2 | lncRNA | 1:150159043-150164948 (-) |  | -2.190 | 1.01e-03 | 7.00e-03 |
| ENSMUSG00000037278 | Tmem97 | protein\_coding | 11:78541817-78550777 (-) |  | -0.378 | 1.01e-03 | 7.00e-03 |
| ENSMUSG00000033222 | Ttf2 | protein\_coding | 3:100938860-100969663 (-) |  | -0.303 | 1.01e-03 | 7.00e-03 |
| ENSMUSG00000015127 | Unkl | protein\_coding | 17:25188397-25234443 (+) |  | 0.642 | 1.01e-03 | 7.01e-03 |
| ENSMUSG00000028266 | Lmo4 | protein\_coding | 3:144188530-144205220 (-) |  | 0.347 | 1.02e-03 | 7.03e-03 |
| ENSMUSG00000021895 | Arhgef3 | protein\_coding | 14:27114899-27403911 (+) |  | 0.482 | 1.02e-03 | 7.06e-03 |
| ENSMUSG00000087701 | Gm13493 | processed\_pseudogene | 2:51871126-51871618 (-) |  | -0.396 | 1.03e-03 | 7.09e-03 |
| ENSMUSG00000027349 | Fam98b | protein\_coding | 2:117249739-117271540 (+) |  | -0.331 | 1.03e-03 | 7.09e-03 |
| ENSMUSG00000027189 | Trim44 | protein\_coding | 2:102300119-102407828 (-) |  | 0.243 | 1.03e-03 | 7.12e-03 |
| ENSMUSG00000040219 | Ttc12 | protein\_coding | 9:49436963-49486225 (-) |  | 0.967 | 1.03e-03 | 7.13e-03 |
| ENSMUSG00000075592 | Nynrin | protein\_coding | 14:55854010-55874736 (+) |  | 0.558 | 1.03e-03 | 7.13e-03 |
| ENSMUSG00000039630 | Hnrnpu | protein\_coding | 1:178321108-178337797 (-) |  | -0.214 | 1.04e-03 | 7.16e-03 |
| ENSMUSG00000029009 | Mthfr | protein\_coding | 4:148039077-148059551 (+) |  | 0.313 | 1.04e-03 | 7.18e-03 |
| ENSMUSG00000023988 | Bysl | protein\_coding | 17:47599331-47611492 (-) |  | -0.322 | 1.04e-03 | 7.19e-03 |
| ENSMUSG00000037020 | Wdr62 | protein\_coding | 7:30240138-30280419 (-) |  | -0.487 | 1.05e-03 | 7.20e-03 |
| ENSMUSG00000047115 | Fam221a | protein\_coding | 6:49367739-49390539 (+) |  | -0.795 | 1.05e-03 | 7.20e-03 |
| ENSMUSG00000021706 | Zfyve16 | protein\_coding | 13:92487108-92530868 (-) |  | 0.334 | 1.05e-03 | 7.21e-03 |
| ENSMUSG00000042594 | Sh2b3 | protein\_coding | 5:121815488-121837646 (-) |  | 0.283 | 1.05e-03 | 7.22e-03 |
| ENSMUSG00000002102 | Psmc3 | protein\_coding | 2:91054009-91066369 (+) |  | -0.256 | 1.07e-03 | 7.32e-03 |
| ENSMUSG00000013236 | Ptprs | protein\_coding | 17:56412426-56476483 (-) |  | 0.418 | 1.07e-03 | 7.34e-03 |
| ENSMUSG00000033427 | Upb1 | protein\_coding | 10:75401115-75441679 (+) |  | 0.886 | 1.07e-03 | 7.36e-03 |
| ENSMUSG00000016477 | E2f3 | protein\_coding | 13:29906575-29986063 (-) |  | -0.379 | 1.08e-03 | 7.38e-03 |
| ENSMUSG00000046897 | Zfp740 | protein\_coding | 15:102203249-102215606 (+) |  | 0.238 | 1.08e-03 | 7.42e-03 |
| ENSMUSG00000022051 | Bnip3l | protein\_coding | 14:66985239-67008877 (-) |  | 0.304 | 1.08e-03 | 7.43e-03 |
| ENSMUSG00000073725 | Lmbrd1 | protein\_coding | 1:24678630-24766301 (+) |  | 0.320 | 1.09e-03 | 7.49e-03 |
| ENSMUSG00000040111 | Gramd1b | protein\_coding | 9:40293233-40531383 (-) |  | 0.298 | 1.09e-03 | 7.49e-03 |
| ENSMUSG00000079225 | Gm9531 | transcribed\_processed\_pseudogene | 9:81677598-81678677 (+) |  | -0.381 | 1.10e-03 | 7.50e-03 |
| ENSMUSG00000020700 | Map3k3 | protein\_coding | 11:106084613-106155446 (+) |  | 0.261 | 1.10e-03 | 7.54e-03 |
| ENSMUSG00000029202 | Pds5a | protein\_coding | 5:65605721-65698273 (-) |  | -0.257 | 1.10e-03 | 7.54e-03 |
| ENSMUSG00000015474 | Ppt2 | protein\_coding | 17:34616662-34628510 (-) |  | 0.380 | 1.10e-03 | 7.54e-03 |
| ENSMUSG00000010517 | Faf1 | protein\_coding | 4:109676588-109963960 (+) |  | -0.296 | 1.11e-03 | 7.56e-03 |
| ENSMUSG00000024493 | Lars | protein\_coding | 18:42202298-42262194 (-) |  | -0.264 | 1.11e-03 | 7.58e-03 |
| ENSMUSG00000064120 | Mocs1 | protein\_coding | 17:49428362-49455435 (+) |  | 0.237 | 1.11e-03 | 7.58e-03 |
| ENSMUSG00000035697 | Arhgap45 | protein\_coding | 10:80016653-80031472 (+) |  | 0.244 | 1.11e-03 | 7.58e-03 |
| ENSMUSG00000040209 | Zfp704 | protein\_coding | 3:9427020-9610085 (-) |  | 0.438 | 1.12e-03 | 7.60e-03 |
| ENSMUSG00000039754 | Alkbh4 | protein\_coding | 5:136136146-136141615 (+) |  | 0.366 | 1.12e-03 | 7.62e-03 |
| ENSMUSG00000024597 | Slc12a2 | protein\_coding | 18:57878678-57946821 (+) |  | 0.962 | 1.12e-03 | 7.62e-03 |
| ENSMUSG00000026260 | Ndufa10 | protein\_coding | 1:92439010-92473860 (-) |  | -0.344 | 1.12e-03 | 7.62e-03 |
| ENSMUSG00000027714 | Exosc9 | protein\_coding | 3:36552606-36565727 (+) |  | -0.343 | 1.12e-03 | 7.62e-03 |
| ENSMUSG00000002718 | Cse1l | protein\_coding | 2:166906040-166946389 (+) |  | -0.249 | 1.12e-03 | 7.63e-03 |
| ENSMUSG00000021880 | Rnase6 | protein\_coding | 14:51123908-51132187 (+) |  | 0.747 | 1.12e-03 | 7.63e-03 |
| ENSMUSG00000001847 | Rac1 | protein\_coding | 5:143503634-143528036 (-) |  | -0.228 | 1.14e-03 | 7.72e-03 |
| ENSMUSG00000025037 | Maoa | protein\_coding | X:16619698-16687818 (+) |  | -0.760 | 1.15e-03 | 7.77e-03 |
| ENSMUSG00000020407 | Upp1 | protein\_coding | 11:9118103-9136170 (+) |  | -2.690 | 1.15e-03 | 7.79e-03 |
| ENSMUSG00000090523 | Gypc | protein\_coding | 18:32528322-32560034 (-) |  | -0.342 | 1.15e-03 | 7.79e-03 |
| ENSMUSG00000055897 | Ppp4r1l-ps | transcribed\_unprocessed\_pseudogene | 2:173579320-173659640 (-) |  | 0.573 | 1.15e-03 | 7.80e-03 |
| ENSMUSG00000010307 | Tmem86a | protein\_coding | 7:47050601-47054777 (+) |  | 0.469 | 1.15e-03 | 7.80e-03 |
| ENSMUSG00000087052 | Gm8093 | transcribed\_processed\_pseudogene | 9:78427507-78430805 (+) |  | 1.680 | 1.15e-03 | 7.80e-03 |
| ENSMUSG00000085531 | Slc36a3os | lncRNA | 11:55137044-55140193 (+) |  | 1.450 | 1.15e-03 | 7.80e-03 |
| ENSMUSG00000067194 | Eif1ax | protein\_coding | X:159372178-159389928 (+) |  | -0.352 | 1.16e-03 | 7.85e-03 |
| ENSMUSG00000109118 | Gm32031 | lncRNA | 7:47008083-47009008 (+) |  | 1.110 | 1.16e-03 | 7.85e-03 |
| ENSMUSG00000031167 | Rbm3 | protein\_coding | X:8138975-8145880 (-) |  | 0.329 | 1.16e-03 | 7.86e-03 |
| ENSMUSG00000006998 | Psmd2 | protein\_coding | 16:20651652-20663414 (+) |  | -0.283 | 1.16e-03 | 7.86e-03 |
| ENSMUSG00000023150 | Ivns1abp | protein\_coding | 1:151344477-151364422 (+) |  | 0.331 | 1.17e-03 | 7.92e-03 |
| ENSMUSG00000027660 | Skil | protein\_coding | 3:31095058-31122577 (+) |  | 0.335 | 1.18e-03 | 7.97e-03 |
| ENSMUSG00000053687 | Dpep2 | protein\_coding | 8:105984944-105991759 (-) |  | -0.942 | 1.19e-03 | 8.00e-03 |
| ENSMUSG00000039470 | Zdhhc2 | protein\_coding | 8:40423815-40510268 (+) |  | -1.240 | 1.19e-03 | 8.00e-03 |
| ENSMUSG00000031776 | Arl2bp | protein\_coding | 8:94666600-94674425 (+) |  | 0.300 | 1.20e-03 | 8.07e-03 |
| ENSMUSG00000038811 | Gngt2 | protein\_coding | 11:95837216-95845734 (+) |  | -0.595 | 1.21e-03 | 8.12e-03 |
| ENSMUSG00000027569 | Mrgbp | protein\_coding | 2:180581304-180586304 (+) |  | 0.380 | 1.21e-03 | 8.12e-03 |
| ENSMUSG00000117458 | Gm6552 | processed\_pseudogene | 17:79933512-79934572 (-) |  | 0.553 | 1.21e-03 | 8.14e-03 |
| ENSMUSG00000022477 | Aco2 | protein\_coding | 15:81872309-81915133 (+) |  | -0.258 | 1.21e-03 | 8.14e-03 |
| ENSMUSG00000054855 | Rnd1 | protein\_coding | 15:98663421-98677461 (-) |  | -1.420 | 1.22e-03 | 8.16e-03 |
| ENSMUSG00000070526 | Peg12 | protein\_coding | 7:62461871-62464510 (-) |  | 1.260 | 1.22e-03 | 8.17e-03 |
| ENSMUSG00000021495 | Fam193b | protein\_coding | 13:55539316-55571120 (-) |  | 0.335 | 1.22e-03 | 8.18e-03 |
| ENSMUSG00000020899 | Pfas | protein\_coding | 11:68985697-69008460 (-) |  | -0.469 | 1.23e-03 | 8.22e-03 |
| ENSMUSG00000039128 | Cdc123 | protein\_coding | 2:5794294-5845164 (-) |  | -0.252 | 1.23e-03 | 8.22e-03 |
| ENSMUSG00000019494 | Cops6 | protein\_coding | 5:138161071-138164646 (+) |  | -0.271 | 1.24e-03 | 8.33e-03 |
| ENSMUSG00000030055 | Rab43 | protein\_coding | 6:87788853-87812164 (-) |  | 0.462 | 1.25e-03 | 8.33e-03 |
| ENSMUSG00000034636 | Zyg11b | protein\_coding | 4:108229724-108301096 (-) |  | 0.229 | 1.25e-03 | 8.33e-03 |
| ENSMUSG00000024556 | Me2 | protein\_coding | 18:73769903-73815449 (-) |  | -0.243 | 1.25e-03 | 8.36e-03 |
| ENSMUSG00000031527 | Eri1 | protein\_coding | 8:35465253-35496196 (-) |  | -0.191 | 1.25e-03 | 8.37e-03 |
| ENSMUSG00000004567 | Mcoln1 | protein\_coding | 8:3500457-3515232 (+) |  | 0.378 | 1.25e-03 | 8.38e-03 |
| ENSMUSG00000029528 | Pxn | protein\_coding | 5:115506676-115555987 (+) |  | 0.188 | 1.26e-03 | 8.41e-03 |
| ENSMUSG00000034116 | Vav1 | protein\_coding | 17:57279100-57328031 (+) |  | -0.189 | 1.26e-03 | 8.43e-03 |
| ENSMUSG00000037058 | Paip2 | protein\_coding | 18:35598617-35617187 (+) |  | 0.339 | 1.27e-03 | 8.46e-03 |
| ENSMUSG00000052298 | Cdc42se2 | protein\_coding | 11:54717456-54787675 (-) |  | 0.211 | 1.27e-03 | 8.46e-03 |
| ENSMUSG00000002055 | Spag5 | protein\_coding | 11:78301529-78322457 (+) |  | -0.216 | 1.27e-03 | 8.47e-03 |
| ENSMUSG00000060373 | Hnrnpc | protein\_coding | 14:52073377-52104028 (-) |  | -0.254 | 1.27e-03 | 8.47e-03 |
| ENSMUSG00000031751 | Amfr | protein\_coding | 8:93971588-94012842 (-) |  | 0.228 | 1.27e-03 | 8.47e-03 |
| ENSMUSG00000058099 | Nfam1 | protein\_coding | 15:82992973-83033306 (-) |  | 0.189 | 1.29e-03 | 8.55e-03 |
| ENSMUSG00000043923 | Ccdc84 | protein\_coding | 9:44410159-44418569 (-) |  | 0.440 | 1.29e-03 | 8.56e-03 |
| ENSMUSG00000030365 | Clec2i | protein\_coding | 6:128887588-128898167 (+) |  | 0.878 | 1.29e-03 | 8.57e-03 |
| ENSMUSG00000024621 | Csf1r | protein\_coding | 18:61100598-61132149 (+) |  | 0.197 | 1.29e-03 | 8.57e-03 |
| ENSMUSG00000073968 | Trim68 | protein\_coding | 7:102677582-102687327 (-) |  | 0.743 | 1.29e-03 | 8.57e-03 |
| ENSMUSG00000015568 | Lpl | protein\_coding | 8:68880491-68907448 (+) |  | 1.110 | 1.29e-03 | 8.59e-03 |
| ENSMUSG00000037851 | Iars | protein\_coding | 13:49682100-49734267 (+) |  | -0.376 | 1.30e-03 | 8.59e-03 |
| ENSMUSG00000036167 | Pphln1 | protein\_coding | 15:93398350-93491510 (+) |  | -0.305 | 1.31e-03 | 8.65e-03 |
| ENSMUSG00000042700 | Sipa1l1 | protein\_coding | 12:82169320-82451786 (+) |  | -0.530 | 1.31e-03 | 8.68e-03 |
| ENSMUSG00000039456 | Morc3 | protein\_coding | 16:93832121-93876073 (+) |  | 0.281 | 1.32e-03 | 8.73e-03 |
| ENSMUSG00000027804 | Ppid | protein\_coding | 3:79591342-79603650 (+) |  | -0.284 | 1.32e-03 | 8.74e-03 |
| ENSMUSG00000034674 | Tdg | protein\_coding | 10:82629828-82650799 (+) |  | -0.308 | 1.33e-03 | 8.77e-03 |
| ENSMUSG00000026112 | Coa5 | protein\_coding | 1:37417084-37430103 (-) |  | -0.241 | 1.33e-03 | 8.77e-03 |
| ENSMUSG00000002395 | Use1 | protein\_coding | 8:71366848-71369732 (+) |  | 0.470 | 1.33e-03 | 8.79e-03 |
| ENSMUSG00000021277 | Traf3 | protein\_coding | 12:111166370-111267153 (+) |  | 0.333 | 1.33e-03 | 8.79e-03 |
| ENSMUSG00000039656 | Rxrb | protein\_coding | 17:34031812-34038399 (+) |  | 0.267 | 1.33e-03 | 8.81e-03 |
| ENSMUSG00000023110 | Prmt5 | protein\_coding | 14:54507187-54517525 (-) |  | -0.361 | 1.34e-03 | 8.86e-03 |
| ENSMUSG00000027330 | Cdc25b | protein\_coding | 2:131186949-131198497 (+) |  | -0.235 | 1.35e-03 | 8.87e-03 |
| ENSMUSG00000023262 | Acy1 | protein\_coding | 9:106432981-106438319 (-) |  | -0.674 | 1.35e-03 | 8.87e-03 |
| ENSMUSG00000038507 | Parp12 | protein\_coding | 6:39086410-39118349 (-) |  | -0.378 | 1.35e-03 | 8.87e-03 |
| ENSMUSG00000000399 | Ndufa9 | protein\_coding | 6:126821721-126849136 (-) |  | -0.280 | 1.35e-03 | 8.88e-03 |
| ENSMUSG00000022265 | Ank | protein\_coding | 15:27466677-27594909 (+) |  | 0.290 | 1.35e-03 | 8.89e-03 |
| ENSMUSG00000031161 | Hdac6 | protein\_coding | X:7930120-7947889 (-) |  | -0.346 | 1.35e-03 | 8.89e-03 |
| ENSMUSG00000003809 | Gcdh | protein\_coding | 8:84886393-84893921 (-) |  | -0.361 | 1.35e-03 | 8.89e-03 |
| ENSMUSG00000028648 | Ndufs5 | protein\_coding | 4:123712710-123718202 (-) |  | -0.370 | 1.35e-03 | 8.89e-03 |
| ENSMUSG00000074785 | Plxnc1 | protein\_coding | 10:94790866-94944835 (-) |  | 0.388 | 1.36e-03 | 8.91e-03 |
| ENSMUSG00000021069 | Pygl | protein\_coding | 12:70190811-70231488 (-) |  | 0.277 | 1.37e-03 | 8.98e-03 |
| ENSMUSG00000002797 | Ggct | protein\_coding | 6:54982580-54992950 (-) |  | -0.616 | 1.37e-03 | 9.02e-03 |
| ENSMUSG00000009894 | Snap47 | protein\_coding | 11:59407134-59451186 (-) |  | -0.988 | 1.38e-03 | 9.06e-03 |
| ENSMUSG00000024436 | Mrps18b | protein\_coding | 17:35910379-35916389 (-) |  | -0.602 | 1.38e-03 | 9.06e-03 |
| ENSMUSG00000045679 | Pqlc3 | protein\_coding | 12:16988648-17000408 (-) |  | 0.251 | 1.38e-03 | 9.06e-03 |
| ENSMUSG00000030557 | Mef2a | protein\_coding | 7:67231163-67372858 (-) |  | 0.255 | 1.39e-03 | 9.08e-03 |
| ENSMUSG00000105632 | Gm43272 | TEC | 5:101810492-101812075 (+) |  | 1.330 | 1.39e-03 | 9.10e-03 |
| ENSMUSG00000035378 | Shq1 | protein\_coding | 6:100568256-100671157 (-) |  | -0.553 | 1.39e-03 | 9.10e-03 |
| ENSMUSG00000034957 | Cebpa | protein\_coding | 7:35119293-35121928 (+) |  | 0.431 | 1.39e-03 | 9.11e-03 |
| ENSMUSG00000035310 | Lin54 | protein\_coding | 5:100441918-100500639 (-) |  | -0.256 | 1.40e-03 | 9.15e-03 |
| ENSMUSG00000036781 | Rps27l | protein\_coding | 9:66946086-66949516 (+) |  | -0.486 | 1.40e-03 | 9.15e-03 |
| ENSMUSG00000037032 | Apbb1 | protein\_coding | 7:105558483-105581653 (-) |  | 0.960 | 1.41e-03 | 9.22e-03 |
| ENSMUSG00000045594 | Glb1 | protein\_coding | 9:114401076-114474898 (+) |  | -0.327 | 1.42e-03 | 9.26e-03 |
| ENSMUSG00000032883 | Acsl3 | protein\_coding | 1:78657825-78707743 (+) |  | -0.501 | 1.42e-03 | 9.29e-03 |
| ENSMUSG00000097194 | 9330175E14Rik | lncRNA | 8:94422898-94435103 (-) |  | -0.618 | 1.43e-03 | 9.30e-03 |
| ENSMUSG00000024878 | Cbwd1 | protein\_coding | 19:24919916-24961610 (-) |  | -0.598 | 1.43e-03 | 9.30e-03 |
| ENSMUSG00000028884 | Rpa2 | protein\_coding | 4:132768332-132778752 (+) |  | -0.385 | 1.43e-03 | 9.31e-03 |
| ENSMUSG00000054000 | Tusc1 | protein\_coding | 4:93334138-93335511 (-) |  | 0.390 | 1.43e-03 | 9.33e-03 |
| ENSMUSG00000056749 | Nfil3 | protein\_coding | 13:52967209-52981073 (-) |  | -0.339 | 1.44e-03 | 9.35e-03 |
| ENSMUSG00000094257 | Ap3s1-ps2 | processed\_pseudogene | 8:94405211-94405789 (+) |  | -0.898 | 1.44e-03 | 9.38e-03 |
| ENSMUSG00000000378 | Ccm2 | protein\_coding | 11:6546887-6596744 (+) |  | 0.294 | 1.45e-03 | 9.40e-03 |
| ENSMUSG00000036678 | Aaas | protein\_coding | 15:102338252-102350771 (-) |  | -0.340 | 1.45e-03 | 9.44e-03 |
| ENSMUSG00000021945 | Zmym2 | protein\_coding | 14:56886653-56962701 (+) |  | 0.271 | 1.45e-03 | 9.44e-03 |
| ENSMUSG00000025510 | Cd151 | protein\_coding | 7:141467392-141471473 (+) |  | 0.411 | 1.45e-03 | 9.44e-03 |
| ENSMUSG00000002032 | Tmem25 | protein\_coding | 9:44793769-44799307 (-) |  | 1.500 | 1.46e-03 | 9.44e-03 |
| ENSMUSG00000021929 | Kpna3 | protein\_coding | 14:61365211-61439874 (-) |  | -0.231 | 1.46e-03 | 9.44e-03 |
| ENSMUSG00000054648 | Zfp869 | protein\_coding | 8:69702656-69716983 (-) |  | 0.267 | 1.47e-03 | 9.51e-03 |
| ENSMUSG00000024431 | Nr3c1 | protein\_coding | 18:39410545-39519421 (-) |  | 0.206 | 1.48e-03 | 9.55e-03 |
| ENSMUSG00000115946 | Mirt2 | lncRNA | 15:76266461-76269839 (-) |  | -2.240 | 1.48e-03 | 9.55e-03 |
| ENSMUSG00000035455 | Fignl1 | protein\_coding | 11:11787431-11808962 (-) |  | -0.269 | 1.48e-03 | 9.55e-03 |
| ENSMUSG00000032459 | Mrps22 | protein\_coding | 9:98588730-98601660 (-) |  | -0.373 | 1.48e-03 | 9.55e-03 |
| ENSMUSG00000029177 | Cenpa | protein\_coding | 5:30666777-30674830 (+) |  | -0.227 | 1.48e-03 | 9.55e-03 |
| ENSMUSG00000020034 | Tcp11l2 | protein\_coding | 10:84576626-84614359 (+) |  | 0.382 | 1.48e-03 | 9.55e-03 |
| ENSMUSG00000038775 | Vill | protein\_coding | 9:119052778-119071525 (+) |  | 0.353 | 1.48e-03 | 9.55e-03 |
| ENSMUSG00000026737 | Pip4k2a | protein\_coding | 2:18842255-18998126 (-) |  | 0.258 | 1.48e-03 | 9.55e-03 |
| ENSMUSG00000053950 | Adnp2 | protein\_coding | 18:80126311-80151482 (-) |  | -0.317 | 1.49e-03 | 9.60e-03 |
| ENSMUSG00000015355 | Cd48 | protein\_coding | 1:171682009-171705258 (+) |  | 0.347 | 1.49e-03 | 9.60e-03 |
| ENSMUSG00000018659 | Pnpo | protein\_coding | 11:96937825-96943986 (-) |  | 0.282 | 1.49e-03 | 9.61e-03 |
| ENSMUSG00000039519 | Cyp7b1 | protein\_coding | 3:18071950-18243338 (-) |  | -1.880 | 1.49e-03 | 9.61e-03 |
| ENSMUSG00000030835 | Nomo1 | protein\_coding | 7:46033698-46084212 (+) |  | -0.376 | 1.49e-03 | 9.62e-03 |
| ENSMUSG00000024952 | Rps6ka4 | protein\_coding | 19:6829085-6840636 (-) |  | -0.255 | 1.50e-03 | 9.64e-03 |
| ENSMUSG00000015755 | Tab2 | protein\_coding | 10:7905653-7956230 (-) |  | 0.226 | 1.50e-03 | 9.64e-03 |
| ENSMUSG00000030982 | Vps35l | protein\_coding | 7:118740226-118842966 (+) |  | -0.238 | 1.50e-03 | 9.64e-03 |
| ENSMUSG00000031262 | Cenpi | protein\_coding | X:134308084-134362639 (+) |  | -0.349 | 1.50e-03 | 9.66e-03 |
| ENSMUSG00000009292 | Trpm2 | protein\_coding | 10:77907722-77970563 (-) |  | 0.416 | 1.51e-03 | 9.69e-03 |
| ENSMUSG00000027309 | 4930402H24Rik | protein\_coding | 2:130706200-130906406 (-) |  | 0.430 | 1.52e-03 | 9.74e-03 |
| ENSMUSG00000059796 | Eif4a1 | protein\_coding | 11:69666936-69672423 (-) |  | -0.226 | 1.52e-03 | 9.74e-03 |
| ENSMUSG00000020974 | Pole2 | protein\_coding | 12:69201773-69228195 (-) |  | -0.421 | 1.52e-03 | 9.74e-03 |
| ENSMUSG00000010048 | Ifrd2 | protein\_coding | 9:107587642-107593385 (+) |  | -0.415 | 1.53e-03 | 9.78e-03 |
| ENSMUSG00000038150 | Ormdl3 | protein\_coding | 11:98581256-98587368 (-) |  | 0.429 | 1.53e-03 | 9.78e-03 |
| ENSMUSG00000081603 | Gm14681 | processed\_pseudogene | X:66778442-66778852 (-) |  | -0.385 | 1.53e-03 | 9.80e-03 |
| ENSMUSG00000043964 | Orai3 | protein\_coding | 7:127769815-127775150 (+) |  | 0.295 | 1.54e-03 | 9.88e-03 |
| ENSMUSG00000062093 | Gm10110 | transcribed\_processed\_pseudogene | 14:89896228-89899447 (-) |  | -0.773 | 1.55e-03 | 9.88e-03 |
| ENSMUSG00000034210 | Efcab14 | protein\_coding | 4:115737744-115777327 (+) |  | -0.325 | 1.55e-03 | 9.88e-03 |
| ENSMUSG00000022721 | Trmt2a | protein\_coding | 16:18248679-18254772 (+) |  | -0.250 | 1.55e-03 | 9.88e-03 |
| ENSMUSG00000060429 | Sntb1 | protein\_coding | 15:55636388-55906949 (-) |  | 0.339 | 1.55e-03 | 9.91e-03 |
| ENSMUSG00000046432 | Bex3 | protein\_coding | X:136270253-136271978 (+) |  | -0.447 | 1.55e-03 | 9.91e-03 |
| ENSMUSG00000028385 | Snx30 | protein\_coding | 4:59805840-59904737 (+) |  | 0.351 | 1.55e-03 | 9.92e-03 |
| ENSMUSG00000026986 | Hnmt | protein\_coding | 2:24002910-24049394 (-) |  | -0.727 | 1.57e-03 | 9.99e-03 |

| ID | Name | Type | Position | Image | logFC | p-Value | Adjusted p-Value |
| --- | --- | --- | --- | --- | --- | --- | --- |

(Page generated on Wed Aug 11 10:23:06 2021 by ReportingTools 2.28.0 and hwriter )
