# Supplementary material for: Transcriptome profiling of osteoclast subsets associated with arthritis: A pathogenic role of CCR2hi osteoclast progenitors
Source: Front Immunol. 2022 Dec 15;13:994035. doi: 10.3389/fimmu.2022.994035 (PMC9797520; doi:10.3389/fimmu.2022.994035)
Supplement: Supplementary file 13 [file DataSheet_5.zip › Supplementary data 5 DGE CTRL vs CIA in CCR2lo/RNAseq_analysis_with_DESeq2_p0.01_extended.html]

RNA-seq analysis of differential expression using DESeq2, P value cutoff 0.01


## RNA-seq analysis of differential expression using DESeq2, P value cutoff 0.01

| ID | Name | Type | Position | Image | logFC | p-Value | Adjusted p-Value |
| --- | --- | --- | --- | --- | --- | --- | --- |
| ID | Name | Type | Position | Image | logFC | p-Value | Adjusted p-Value |
| ENSMUSG00000031441 | Atp11a | protein\_coding | 8:12757014-12868728 (+) |  | -1.890 | 1.34e-73 | 1.74e-69 |
| ENSMUSG00000026478 | Lamc1 | protein\_coding | 1:153218922-153332786 (-) |  | -1.880 | 9.50e-36 | 6.17e-32 |
| ENSMUSG00000017002 | Slpi | protein\_coding | 2:164354070-164389095 (-) |  | 1.020 | 8.37e-26 | 3.62e-22 |
| ENSMUSG00000042082 | Arsb | protein\_coding | 13:93771630-93943016 (+) |  | 0.928 | 6.79e-25 | 2.20e-21 |
| ENSMUSG00000026768 | Itga8 | protein\_coding | 2:12106632-12301922 (-) |  | 3.040 | 2.43e-24 | 6.16e-21 |
| ENSMUSG00000024679 | Ms4a6d | protein\_coding | 19:11586604-11604849 (-) |  | -1.690 | 2.85e-24 | 6.16e-21 |
| ENSMUSG00000060962 | Dmkn | protein\_coding | 7:30763756-30781063 (+) |  | -1.540 | 4.89e-23 | 7.76e-20 |
| ENSMUSG00000052698 | Tln2 | protein\_coding | 9:67217087-67559703 (-) |  | -1.760 | 5.13e-23 | 7.76e-20 |
| ENSMUSG00000030246 | Ldhb | protein\_coding | 6:142490249-142507957 (-) |  | 1.350 | 5.38e-23 | 7.76e-20 |
| ENSMUSG00000048612 | Myof | protein\_coding | 19:37899036-38043577 (-) |  | -2.170 | 5.75e-22 | 7.47e-19 |
| ENSMUSG00000073940 | Hbb-bt | protein\_coding | 7:103812524-103813996 (-) |  | 2.960 | 1.10e-20 | 1.29e-17 |
| ENSMUSG00000021477 | Ctsl | protein\_coding | 13:64359337-64370890 (-) |  | -0.882 | 3.65e-20 | 3.95e-17 |
| ENSMUSG00000056124 | B4galt6 | protein\_coding | 18:20684599-20746404 (-) |  | -1.160 | 7.10e-20 | 7.10e-17 |
| ENSMUSG00000074151 | Nlrc5 | protein\_coding | 8:94434356-94527272 (+) |  | -0.799 | 5.26e-19 | 4.88e-16 |
| ENSMUSG00000022912 | Pros1 | protein\_coding | 16:62854307-62929346 (+) |  | -0.875 | 5.63e-19 | 4.88e-16 |
| ENSMUSG00000030748 | Il4ra | protein\_coding | 7:125552120-125579474 (+) |  | -0.824 | 8.90e-19 | 7.23e-16 |
| ENSMUSG00000032216 | Nedd4 | protein\_coding | 9:72662346-72749852 (+) |  | 1.240 | 2.63e-18 | 2.01e-15 |
| ENSMUSG00000037685 | Atp8a1 | protein\_coding | 5:67618140-67847434 (-) |  | -0.850 | 3.77e-18 | 2.72e-15 |
| ENSMUSG00000034168 | Irf2bpl | protein\_coding | 12:86880701-86884798 (-) |  | -1.360 | 6.21e-18 | 4.24e-15 |
| ENSMUSG00000039713 | Plekhg5 | protein\_coding | 4:152072498-152115400 (+) |  | 1.810 | 7.99e-18 | 5.19e-15 |
| ENSMUSG00000028523 | Tctex1d1 | protein\_coding | 4:102978606-103005594 (+) |  | -1.890 | 1.26e-17 | 7.80e-15 |
| ENSMUSG00000062127 | Cttnbp2nl | protein\_coding | 3:105001915-105053146 (-) |  | -1.810 | 2.92e-17 | 1.73e-14 |
| ENSMUSG00000032508 | Myd88 | protein\_coding | 9:119335934-119341411 (-) |  | -0.670 | 4.04e-17 | 2.28e-14 |
| ENSMUSG00000027864 | Ptgfrn | protein\_coding | 3:101040232-101110278 (-) |  | -2.310 | 4.72e-17 | 2.56e-14 |
| ENSMUSG00000024014 | Pim1 | protein\_coding | 17:29490753-29496112 (+) |  | -1.590 | 5.91e-17 | 3.05e-14 |
| ENSMUSG00000051166 | Eml5 | protein\_coding | 12:98786805-98901484 (-) |  | 1.540 | 6.11e-17 | 3.05e-14 |
| ENSMUSG00000037095 | Lrg1 | protein\_coding | 17:56119678-56122001 (-) |  | -4.940 | 9.34e-17 | 4.49e-14 |
| ENSMUSG00000058624 | Gda | protein\_coding | 19:21391307-21473445 (-) |  | -0.764 | 1.68e-16 | 7.80e-14 |
| ENSMUSG00000030560 | Ctsc | protein\_coding | 7:88278085-88310888 (+) |  | -1.410 | 2.42e-16 | 1.08e-13 |
| ENSMUSG00000042719 | Naa25 | protein\_coding | 5:121397936-121444378 (+) |  | -0.674 | 3.34e-16 | 1.44e-13 |
| ENSMUSG00000024349 | Tmem173 | protein\_coding | 18:35733679-35740554 (-) |  | -0.508 | 3.83e-16 | 1.61e-13 |
| ENSMUSG00000040253 | Gbp7 | protein\_coding | 3:142530342-142550149 (+) |  | -0.704 | 5.07e-16 | 2.06e-13 |
| ENSMUSG00000005802 | Slc30a4 | protein\_coding | 2:122681233-122702663 (-) |  | -1.130 | 6.63e-16 | 2.61e-13 |
| ENSMUSG00000069917 | Hba-a2 | protein\_coding | 11:32296489-32297298 (+) |  | 3.180 | 8.41e-16 | 3.21e-13 |
| ENSMUSG00000019528 | Gyg | protein\_coding | 3:20122084-20155317 (-) |  | -0.721 | 9.73e-16 | 3.61e-13 |
| ENSMUSG00000071713 | Csf2rb | protein\_coding | 15:78325752-78353847 (+) |  | -0.462 | 1.69e-15 | 6.09e-13 |
| ENSMUSG00000059336 | Slc14a1 | protein\_coding | 18:78100091-78142119 (-) |  | 1.850 | 2.38e-15 | 8.36e-13 |
| ENSMUSG00000028124 | Gclm | protein\_coding | 3:122245557-122270732 (+) |  | -0.882 | 2.93e-15 | 1.00e-12 |
| ENSMUSG00000027199 | Gatm | protein\_coding | 2:122594467-122611303 (-) |  | -0.858 | 4.49e-15 | 1.50e-12 |
| ENSMUSG00000018381 | Abi3 | protein\_coding | 11:95830074-95842476 (-) |  | -1.850 | 7.99e-15 | 2.59e-12 |
| ENSMUSG00000029162 | Khk | protein\_coding | 5:30921431-30931248 (+) |  | 0.796 | 8.17e-15 | 2.59e-12 |
| ENSMUSG00000038070 | Cntln | protein\_coding | 4:84884309-85131921 (+) |  | 0.750 | 1.43e-14 | 4.43e-12 |
| ENSMUSG00000031494 | Cd209a | protein\_coding | 8:3743397-3748984 (-) |  | 1.690 | 2.32e-14 | 7.01e-12 |
| ENSMUSG00000046314 | Stxbp6 | protein\_coding | 12:44852484-45074709 (-) |  | 1.040 | 2.39e-14 | 7.05e-12 |
| ENSMUSG00000032902 | Slc16a1 | protein\_coding | 3:104638668-104658462 (+) |  | -0.673 | 2.86e-14 | 8.25e-12 |
| ENSMUSG00000022429 | Dmc1 | protein\_coding | 15:79561497-79605109 (-) |  | 1.420 | 3.23e-14 | 9.11e-12 |
| ENSMUSG00000021458 | Aopep | protein\_coding | 13:62964893-63326096 (+) |  | -0.627 | 5.85e-14 | 1.62e-11 |
| ENSMUSG00000031391 | L1cam | protein\_coding | X:73853778-73896105 (-) |  | 0.990 | 7.37e-14 | 1.99e-11 |
| ENSMUSG00000046562 | Unc119b | protein\_coding | 5:115122550-115134975 (-) |  | 0.600 | 7.75e-14 | 2.05e-11 |
| ENSMUSG00000032411 | Tfdp2 | protein\_coding | 9:96196275-96323646 (+) |  | 0.649 | 8.43e-14 | 2.16e-11 |
| ENSMUSG00000021830 | Txndc16 | protein\_coding | 14:45133465-45220328 (-) |  | 0.648 | 8.49e-14 | 2.16e-11 |
| ENSMUSG00000030083 | Abtb1 | protein\_coding | 6:88835914-88841984 (-) |  | 0.694 | 8.86e-14 | 2.21e-11 |
| ENSMUSG00000053063 | Clec12a | protein\_coding | 6:129342691-129365303 (+) |  | 0.652 | 1.41e-13 | 3.47e-11 |
| ENSMUSG00000105504 | Gbp5 | protein\_coding | 3:142493978-142522344 (+) |  | -0.908 | 1.50e-13 | 3.62e-11 |
| ENSMUSG00000022636 | Alcam | protein\_coding | 16:52248996-52454074 (-) |  | 0.637 | 2.07e-13 | 4.89e-11 |
| ENSMUSG00000015176 | Nolc1 | protein\_coding | 19:46075863-46085530 (+) |  | -0.586 | 2.27e-13 | 5.27e-11 |
| ENSMUSG00000057497 | Fam136a | protein\_coding | 6:86365646-86370058 (+) |  | -0.717 | 2.74e-13 | 6.25e-11 |
| ENSMUSG00000002006 | Pdzd4 | protein\_coding | X:73793359-73824969 (-) |  | 1.110 | 2.85e-13 | 6.39e-11 |
| ENSMUSG00000022218 | Tgm1 | protein\_coding | 14:55700009-55713926 (-) |  | -1.370 | 3.21e-13 | 7.08e-11 |
| ENSMUSG00000028228 | Cpne3 | protein\_coding | 4:19519254-19570108 (-) |  | -0.527 | 3.48e-13 | 7.54e-11 |
| ENSMUSG00000018848 | Rars | protein\_coding | 11:35808381-35834506 (-) |  | -0.482 | 4.69e-13 | 9.99e-11 |
| ENSMUSG00000039542 | Ncam1 | protein\_coding | 9:49502136-49798925 (-) |  | 1.110 | 4.99e-13 | 1.04e-10 |
| ENSMUSG00000079227 | Ccr5 | protein\_coding | 9:124121543-124147699 (+) |  | -1.530 | 5.52e-13 | 1.14e-10 |
| ENSMUSG00000024905 | Tesmin | protein\_coding | 19:3388857-3407823 (+) |  | 1.660 | 5.77e-13 | 1.17e-10 |
| ENSMUSG00000020593 | Lpin1 | protein\_coding | 12:16535669-16646966 (-) |  | 1.320 | 5.88e-13 | 1.18e-10 |
| ENSMUSG00000049971 | Glt1d1 | protein\_coding | 5:127632262-127709374 (+) |  | 0.941 | 6.75e-13 | 1.32e-10 |
| ENSMUSG00000029657 | Hsph1 | protein\_coding | 5:149614287-149636376 (-) |  | -0.958 | 6.79e-13 | 1.32e-10 |
| ENSMUSG00000025491 | Ifitm1 | protein\_coding | 7:140967221-140969825 (+) |  | -3.040 | 7.87e-13 | 1.50e-10 |
| ENSMUSG00000052534 | Pbx1 | protein\_coding | 1:168119364-168432270 (-) |  | -1.260 | 8.76e-13 | 1.65e-10 |
| ENSMUSG00000031805 | Jak3 | protein\_coding | 8:71676296-71690575 (+) |  | -0.897 | 1.27e-12 | 2.36e-10 |
| ENSMUSG00000074794 | Arrdc3 | protein\_coding | 13:80883384-80896042 (+) |  | 0.743 | 1.42e-12 | 2.59e-10 |
| ENSMUSG00000022094 | Slc39a14 | protein\_coding | 14:70303469-70351425 (-) |  | -1.320 | 1.57e-12 | 2.84e-10 |
| ENSMUSG00000048442 | Smim5 | protein\_coding | 11:115899966-115906269 (+) |  | 1.300 | 1.61e-12 | 2.86e-10 |
| ENSMUSG00000069805 | Fbp1 | protein\_coding | 13:62864753-62888282 (-) |  | 1.980 | 1.88e-12 | 3.30e-10 |
| ENSMUSG00000028524 | Sgip1 | protein\_coding | 4:102741297-102973628 (+) |  | -1.030 | 2.13e-12 | 3.69e-10 |
| ENSMUSG00000067714 | Lpar5 | protein\_coding | 6:125067920-125082472 (+) |  | 0.782 | 2.74e-12 | 4.68e-10 |
| ENSMUSG00000006442 | Srm | protein\_coding | 4:148591503-148594993 (+) |  | -0.694 | 3.37e-12 | 5.69e-10 |
| ENSMUSG00000041926 | Rnpep | protein\_coding | 1:135262712-135284084 (-) |  | -0.471 | 3.56e-12 | 5.93e-10 |
| ENSMUSG00000029060 | Mib2 | protein\_coding | 4:155654677-155669198 (-) |  | 0.666 | 3.65e-12 | 6.00e-10 |
| ENSMUSG00000037331 | Larp1 | protein\_coding | 11:58009064-58062034 (+) |  | -0.482 | 3.97e-12 | 6.45e-10 |
| ENSMUSG00000117613 | Gm2629 | lncRNA | 18:15194782-15214696 (+) |  | 0.833 | 4.19e-12 | 6.66e-10 |
| ENSMUSG00000038481 | Cdk19 | protein\_coding | 10:40339564-40483818 (+) |  | 0.392 | 4.20e-12 | 6.66e-10 |
| ENSMUSG00000037235 | Mxd4 | protein\_coding | 5:34173883-34187720 (-) |  | 0.916 | 4.40e-12 | 6.88e-10 |
| ENSMUSG00000031722 | Hp | protein\_coding | 8:109575128-109579172 (-) |  | -0.615 | 4.72e-12 | 7.31e-10 |
| ENSMUSG00000029082 | Bst1 | protein\_coding | 5:43818885-43843986 (+) |  | -1.810 | 7.28e-12 | 1.11e-09 |
| ENSMUSG00000045193 | Cirbp | protein\_coding | 10:80165985-80172786 (+) |  | 0.630 | 8.94e-12 | 1.35e-09 |
| ENSMUSG00000116114 | Gm35853 | lncRNA | 15:101322888-101405834 (+) |  | 2.380 | 1.05e-11 | 1.57e-09 |
| ENSMUSG00000056091 | St3gal5 | protein\_coding | 6:72097592-72154571 (+) |  | 1.550 | 1.33e-11 | 1.96e-09 |
| ENSMUSG00000026121 | Sema4c | protein\_coding | 1:36548639-36558349 (-) |  | 1.450 | 1.37e-11 | 2.00e-09 |
| ENSMUSG00000000753 | Serpinf1 | protein\_coding | 11:75409769-75422701 (-) |  | 0.737 | 1.63e-11 | 2.35e-09 |
| ENSMUSG00000032803 | Cdv3 | protein\_coding | 9:103353094-103365840 (-) |  | -0.461 | 1.65e-11 | 2.36e-09 |
| ENSMUSG00000024042 | Sik1 | protein\_coding | 17:31844250-31855804 (-) |  | 0.948 | 1.71e-11 | 2.42e-09 |
| ENSMUSG00000038352 | Arl5c | protein\_coding | 11:97989578-97996181 (-) |  | 0.874 | 2.13e-11 | 2.97e-09 |
| ENSMUSG00000062937 | Mtap | protein\_coding | 4:89137122-89181081 (+) |  | -0.537 | 2.17e-11 | 2.98e-09 |
| ENSMUSG00000038271 | Iffo1 | protein\_coding | 6:125145241-125161782 (+) |  | 0.745 | 2.18e-11 | 2.98e-09 |
| ENSMUSG00000026580 | Selp | protein\_coding | 1:164115264-164150026 (+) |  | -2.280 | 2.53e-11 | 3.43e-09 |
| ENSMUSG00000053819 | Camk2d | protein\_coding | 3:126596302-126846326 (+) |  | -1.740 | 2.74e-11 | 3.67e-09 |
| ENSMUSG00000042524 | Sun2 | protein\_coding | 15:79724070-79742536 (-) |  | 0.477 | 3.03e-11 | 4.01e-09 |
| ENSMUSG00000097164 | Cep83os | lncRNA | 10:94671025-94688576 (-) |  | 0.906 | 4.07e-11 | 5.34e-09 |
| ENSMUSG00000036928 | Stag3 | protein\_coding | 5:138280240-138312393 (+) |  | 1.250 | 5.33e-11 | 6.92e-09 |
| ENSMUSG00000039899 | Fgl2 | protein\_coding | 5:21372642-21378374 (+) |  | -0.601 | 5.42e-11 | 6.97e-09 |
| ENSMUSG00000020682 | Mmp28 | protein\_coding | 11:83440768-83463071 (-) |  | 1.080 | 7.00e-11 | 8.86e-09 |
| ENSMUSG00000034667 | Xpot | protein\_coding | 10:121587380-121626332 (-) |  | -0.494 | 7.02e-11 | 8.86e-09 |
| ENSMUSG00000110647 | Gm17745 | lncRNA | 10:93335393-93348877 (+) |  | 1.450 | 7.77e-11 | 9.70e-09 |
| ENSMUSG00000001229 | Dpp9 | protein\_coding | 17:56186807-56218905 (-) |  | -0.435 | 8.56e-11 | 1.06e-08 |
| ENSMUSG00000030427 | Lilra6 | protein\_coding | 7:3908280-3915503 (-) |  | 1.090 | 9.11e-11 | 1.12e-08 |
| ENSMUSG00000054676 | 1600014C10Rik | protein\_coding | 7:38183217-38197568 (+) |  | -0.499 | 9.29e-11 | 1.13e-08 |
| ENSMUSG00000029447 | Cct6a | protein\_coding | 5:129786998-129846371 (+) |  | -0.639 | 9.70e-11 | 1.17e-08 |
| ENSMUSG00000073418 | C4b | protein\_coding | 17:34728380-34743882 (-) |  | -2.360 | 1.20e-10 | 1.43e-08 |
| ENSMUSG00000024180 | Tmem8 | protein\_coding | 17:26113299-26123254 (+) |  | 0.784 | 1.38e-10 | 1.63e-08 |
| ENSMUSG00000050379 | Sept6 | protein\_coding | X:36911326-36991794 (-) |  | 0.537 | 1.40e-10 | 1.64e-08 |
| ENSMUSG00000001674 | Ddx18 | protein\_coding | 1:121553835-121567989 (-) |  | -0.517 | 1.43e-10 | 1.65e-08 |
| ENSMUSG00000025190 | Got1 | protein\_coding | 19:43499752-43524605 (-) |  | -0.701 | 1.44e-10 | 1.65e-08 |
| ENSMUSG00000026245 | Farsb | protein\_coding | 1:78417975-78488897 (-) |  | -0.573 | 1.61e-10 | 1.83e-08 |
| ENSMUSG00000030257 | Srgap3 | protein\_coding | 6:112717971-112947266 (-) |  | 0.901 | 2.43e-10 | 2.74e-08 |
| ENSMUSG00000021270 | Hsp90aa1 | protein\_coding | 12:110690605-110702728 (-) |  | -0.680 | 2.69e-10 | 3.01e-08 |
| ENSMUSG00000045817 | Zfp36l2 | protein\_coding | 17:84183931-84187947 (-) |  | 0.599 | 2.89e-10 | 3.20e-08 |
| ENSMUSG00000031555 | Adam9 | protein\_coding | 8:24949611-25016927 (-) |  | -0.603 | 3.12e-10 | 3.44e-08 |
| ENSMUSG00000029084 | Cd38 | protein\_coding | 5:43868553-43912375 (+) |  | -4.940 | 3.28e-10 | 3.56e-08 |
| ENSMUSG00000040078 | Ptges3-ps | processed\_pseudogene | 6:85843980-85844459 (+) |  | -0.573 | 3.29e-10 | 3.56e-08 |
| ENSMUSG00000033427 | Upb1 | protein\_coding | 10:75401115-75441679 (+) |  | 1.240 | 3.42e-10 | 3.67e-08 |
| ENSMUSG00000026893 | Gca | protein\_coding | 2:62664285-62694109 (+) |  | -0.833 | 3.72e-10 | 3.96e-08 |
| ENSMUSG00000094872 | Igkv9-120 | IG\_V\_gene | 6:68049983-68050456 (+) |  | 7.000 | 3.76e-10 | 3.97e-08 |
| ENSMUSG00000018340 | Anxa6 | protein\_coding | 11:54979108-55033445 (-) |  | 0.432 | 4.21e-10 | 4.41e-08 |
| ENSMUSG00000019823 | Mical1 | protein\_coding | 10:41476314-41487032 (+) |  | 0.494 | 4.24e-10 | 4.41e-08 |
| ENSMUSG00000015947 | Fcgr1 | protein\_coding | 3:96282909-96293969 (-) |  | -1.750 | 4.51e-10 | 4.65e-08 |
| ENSMUSG00000024521 | Pmaip1 | protein\_coding | 18:66458533-66465565 (+) |  | -1.240 | 4.60e-10 | 4.71e-08 |
| ENSMUSG00000019960 | Dusp6 | protein\_coding | 10:99263231-99267489 (+) |  | 2.280 | 4.70e-10 | 4.77e-08 |
| ENSMUSG00000058355 | Abce1 | protein\_coding | 8:79683462-79711740 (-) |  | -0.502 | 5.21e-10 | 5.24e-08 |
| ENSMUSG00000032369 | Plscr1 | protein\_coding | 9:92249750-92272278 (+) |  | -1.000 | 5.27e-10 | 5.26e-08 |
| ENSMUSG00000031995 | St14 | protein\_coding | 9:31089402-31131853 (-) |  | 0.725 | 5.30e-10 | 5.26e-08 |
| ENSMUSG00000016552 | Foxred2 | protein\_coding | 15:77940522-77956722 (-) |  | 0.594 | 5.66e-10 | 5.57e-08 |
| ENSMUSG00000073902 | Gm1966 | unprocessed\_pseudogene | 7:106596743-106604035 (-) |  | -0.653 | 6.06e-10 | 5.91e-08 |
| ENSMUSG00000046080 | Clec9a | protein\_coding | 6:129408862-129424763 (+) |  | 0.961 | 6.45e-10 | 6.26e-08 |
| ENSMUSG00000028741 | Mrto4 | protein\_coding | 4:139347435-139352576 (-) |  | -0.547 | 6.65e-10 | 6.40e-08 |
| ENSMUSG00000028156 | Eif4e | protein\_coding | 3:138526179-138559696 (+) |  | -0.450 | 7.08e-10 | 6.72e-08 |
| ENSMUSG00000035847 | Ids | protein\_coding | X:70343069-70365084 (-) |  | 0.690 | 7.09e-10 | 6.72e-08 |
| ENSMUSG00000054766 | Set | protein\_coding | 2:30057378-30072577 (+) |  | -0.525 | 7.37e-10 | 6.92e-08 |
| ENSMUSG00000052305 | Hbb-bs | protein\_coding | 7:103826534-103828096 (-) |  | 2.530 | 7.40e-10 | 6.92e-08 |
| ENSMUSG00000020075 | Ddx21 | protein\_coding | 10:62580251-62602281 (-) |  | -0.436 | 7.51e-10 | 6.96e-08 |
| ENSMUSG00000003206 | Ebi3 | protein\_coding | 17:55952640-55957022 (+) |  | 1.110 | 8.58e-10 | 7.91e-08 |
| ENSMUSG00000026923 | Notch1 | protein\_coding | 2:26457903-26516663 (-) |  | 0.371 | 8.74e-10 | 8.00e-08 |
| ENSMUSG00000024053 | Emilin2 | protein\_coding | 17:71252172-71311978 (-) |  | -0.620 | 9.23e-10 | 8.33e-08 |
| ENSMUSG00000042757 | Tmem108 | protein\_coding | 9:103482947-103761837 (-) |  | -2.000 | 9.24e-10 | 8.33e-08 |
| ENSMUSG00000021109 | Hif1a | protein\_coding | 12:73901375-73947530 (+) |  | -0.568 | 9.60e-10 | 8.60e-08 |
| ENSMUSG00000030123 | Plxnd1 | protein\_coding | 6:115954811-115995005 (-) |  | 0.622 | 9.76e-10 | 8.65e-08 |
| ENSMUSG00000017057 | Il13ra1 | protein\_coding | X:36112110-36171259 (+) |  | -1.180 | 9.83e-10 | 8.65e-08 |
| ENSMUSG00000026721 | Rabgap1l | protein\_coding | 1:160219174-160793211 (-) |  | 0.609 | 9.86e-10 | 8.65e-08 |
| ENSMUSG00000031495 | Cd209d | protein\_coding | 8:3871824-3878555 (-) |  | 2.390 | 1.08e-09 | 9.44e-08 |
| ENSMUSG00000041268 | Dmxl2 | protein\_coding | 9:54365158-54501626 (-) |  | -0.564 | 1.14e-09 | 9.84e-08 |
| ENSMUSG00000000673 | Haao | protein\_coding | 17:83831156-83847963 (-) |  | 0.619 | 1.21e-09 | 1.04e-07 |
| ENSMUSG00000027333 | Smox | protein\_coding | 2:131491496-131525922 (+) |  | 0.862 | 1.23e-09 | 1.05e-07 |
| ENSMUSG00000022241 | Tars | protein\_coding | 15:11382301-11399665 (-) |  | -0.487 | 1.26e-09 | 1.07e-07 |
| ENSMUSG00000028811 | Yars | protein\_coding | 4:129189760-129219607 (+) |  | -0.513 | 1.28e-09 | 1.08e-07 |
| ENSMUSG00000003420 | Fcgrt | protein\_coding | 7:45092990-45103851 (-) |  | 0.791 | 1.37e-09 | 1.15e-07 |
| ENSMUSG00000025701 | Alox5 | protein\_coding | 6:116410077-116461178 (-) |  | 1.940 | 1.44e-09 | 1.20e-07 |
| ENSMUSG00000022265 | Ank | protein\_coding | 15:27466677-27594909 (+) |  | 0.583 | 1.67e-09 | 1.38e-07 |
| ENSMUSG00000069792 | Wfdc17 | protein\_coding | 11:83703991-83706268 (+) |  | -1.370 | 1.72e-09 | 1.41e-07 |
| ENSMUSG00000040283 | Btnl9 | protein\_coding | 11:49165585-49187159 (-) |  | 1.350 | 1.79e-09 | 1.46e-07 |
| ENSMUSG00000020689 | Itgb3 | protein\_coding | 11:104608000-104670476 (+) |  | 1.910 | 1.80e-09 | 1.46e-07 |
| ENSMUSG00000054263 | Lifr | protein\_coding | 15:7090614-7197489 (+) |  | -1.360 | 1.87e-09 | 1.50e-07 |
| ENSMUSG00000037275 | Gemin5 | protein\_coding | 11:58120002-58168539 (-) |  | -0.503 | 1.89e-09 | 1.51e-07 |
| ENSMUSG00000025007 | Aldh18a1 | protein\_coding | 19:40550257-40588463 (-) |  | -0.635 | 1.97e-09 | 1.57e-07 |
| ENSMUSG00000074656 | Eif2s2 | protein\_coding | 2:154871410-154892935 (-) |  | -0.590 | 2.03e-09 | 1.61e-07 |
| ENSMUSG00000004665 | Cnn2 | protein\_coding | 10:79988584-79996062 (+) |  | 0.571 | 2.39e-09 | 1.88e-07 |
| ENSMUSG00000047879 | Usp14 | protein\_coding | 18:9993066-10045119 (-) |  | -0.411 | 2.52e-09 | 1.97e-07 |
| ENSMUSG00000027236 | Eif3j1 | protein\_coding | 2:122028546-122056598 (+) |  | -0.531 | 2.65e-09 | 2.06e-07 |
| ENSMUSG00000001227 | Sema6b | protein\_coding | 17:56123085-56140343 (-) |  | -0.723 | 2.74e-09 | 2.12e-07 |
| ENSMUSG00000006574 | Slc4a1 | protein\_coding | 11:102348824-102366203 (-) |  | 2.180 | 2.79e-09 | 2.14e-07 |
| ENSMUSG00000005667 | Mthfd2 | protein\_coding | 6:83305691-83325908 (-) |  | -0.526 | 2.80e-09 | 2.14e-07 |
| ENSMUSG00000042660 | Wdr55 | protein\_coding | 18:36760220-36763810 (+) |  | -0.548 | 2.97e-09 | 2.25e-07 |
| ENSMUSG00000062585 | Cnr2 | protein\_coding | 4:135895394-135920207 (+) |  | 0.740 | 3.01e-09 | 2.27e-07 |
| ENSMUSG00000097077 | Gm16712 | lncRNA | 17:55954771-55959381 (-) |  | 1.550 | 3.02e-09 | 2.27e-07 |
| ENSMUSG00000004929 | Thop1 | protein\_coding | 10:81070035-81082559 (+) |  | -0.655 | 3.10e-09 | 2.31e-07 |
| ENSMUSG00000060512 | 0610040J01Rik | protein\_coding | 5:63812363-63899625 (+) |  | -0.811 | 3.31e-09 | 2.45e-07 |
| ENSMUSG00000021048 | Mthfd1 | protein\_coding | 12:76255298-76319803 (+) |  | -0.505 | 3.56e-09 | 2.63e-07 |
| ENSMUSG00000021196 | Pfkp | protein\_coding | 13:6579768-6648777 (-) |  | -0.441 | 3.60e-09 | 2.64e-07 |
| ENSMUSG00000041506 | Rrp9 | protein\_coding | 9:106475963-106485424 (+) |  | -0.445 | 3.70e-09 | 2.69e-07 |
| ENSMUSG00000026020 | Nop58 | protein\_coding | 1:59684971-59719044 (+) |  | -0.542 | 3.71e-09 | 2.69e-07 |
| ENSMUSG00000002984 | Tomm40 | protein\_coding | 7:19701313-19715438 (-) |  | -0.490 | 3.84e-09 | 2.77e-07 |
| ENSMUSG00000082292 | Gm12250 | processed\_pseudogene | 11:58187739-58189012 (+) |  | -0.821 | 3.99e-09 | 2.86e-07 |
| ENSMUSG00000039682 | Lap3 | protein\_coding | 5:45493374-45512691 (+) |  | -0.610 | 4.65e-09 | 3.32e-07 |
| ENSMUSG00000067367 | Lyar | protein\_coding | 5:38220470-38234306 (+) |  | -0.562 | 4.76e-09 | 3.38e-07 |
| ENSMUSG00000001128 | Cfp | protein\_coding | X:20925454-20931555 (-) |  | 0.477 | 4.86e-09 | 3.43e-07 |
| ENSMUSG00000026615 | Eprs | protein\_coding | 1:185363044-185428360 (+) |  | -0.515 | 5.18e-09 | 3.63e-07 |
| ENSMUSG00000071714 | Csf2rb2 | protein\_coding | 15:78282507-78305721 (-) |  | -0.496 | 5.52e-09 | 3.85e-07 |
| ENSMUSG00000038463 | Olfml2b | protein\_coding | 1:170644532-170682789 (+) |  | -2.490 | 5.57e-09 | 3.87e-07 |
| ENSMUSG00000050244 | Heatr1 | protein\_coding | 13:12395027-12440289 (+) |  | -0.479 | 5.81e-09 | 4.01e-07 |
| ENSMUSG00000025151 | Maged1 | protein\_coding | X:94535474-94542143 (-) |  | -1.160 | 6.13e-09 | 4.21e-07 |
| ENSMUSG00000020788 | Atp2a3 | protein\_coding | 11:72961169-72993044 (+) |  | 0.502 | 6.50e-09 | 4.43e-07 |
| ENSMUSG00000032946 | Rasgrp2 | protein\_coding | 19:6399340-6415216 (+) |  | 0.336 | 6.54e-09 | 4.43e-07 |
| ENSMUSG00000021947 | Cryl1 | protein\_coding | 14:57274993-57398529 (-) |  | 0.577 | 6.56e-09 | 4.43e-07 |
| ENSMUSG00000020592 | Sdc1 | protein\_coding | 12:8771323-8793715 (+) |  | -1.440 | 6.61e-09 | 4.43e-07 |
| ENSMUSG00000011884 | Gltp | protein\_coding | 5:114669398-114690984 (-) |  | 0.438 | 6.61e-09 | 4.43e-07 |
| ENSMUSG00000062991 | Nrg1 | protein\_coding | 8:31814551-32884797 (-) |  | -0.385 | 7.07e-09 | 4.71e-07 |
| ENSMUSG00000020869 | Lrrc59 | protein\_coding | 11:94629767-94645216 (+) |  | -0.540 | 7.42e-09 | 4.92e-07 |
| ENSMUSG00000030149 | Klrk1 | protein\_coding | 6:129610323-129623864 (-) |  | 0.827 | 7.84e-09 | 5.17e-07 |
| ENSMUSG00000023143 | Nagpa | protein\_coding | 16:5195289-5204012 (-) |  | -0.413 | 8.41e-09 | 5.52e-07 |
| ENSMUSG00000042066 | Tmcc2 | protein\_coding | 1:132356315-132391281 (-) |  | 0.588 | 9.05e-09 | 5.91e-07 |
| ENSMUSG00000030147 | Clec4b1 | protein\_coding | 6:123049962-123071555 (+) |  | 1.380 | 9.44e-09 | 6.13e-07 |
| ENSMUSG00000020532 | Acaca | protein\_coding | 11:84129672-84401651 (+) |  | -0.411 | 9.88e-09 | 6.39e-07 |
| ENSMUSG00000026068 | Il18rap | protein\_coding | 1:40515362-40551705 (+) |  | -0.968 | 1.01e-08 | 6.49e-07 |
| ENSMUSG00000020250 | Txnrd1 | protein\_coding | 10:82833951-82897712 (+) |  | -0.391 | 1.04e-08 | 6.65e-07 |
| ENSMUSG00000051727 | Kctd14 | protein\_coding | 7:97451323-97459557 (+) |  | 0.652 | 1.04e-08 | 6.65e-07 |
| ENSMUSG00000005846 | Rsl1d1 | protein\_coding | 16:11192970-11203331 (-) |  | -0.477 | 1.05e-08 | 6.67e-07 |
| ENSMUSG00000112825 | Gm9118 | processed\_pseudogene | 10:56497341-56498094 (+) |  | -0.625 | 1.06e-08 | 6.69e-07 |
| ENSMUSG00000004040 | Stat3 | protein\_coding | 11:100885098-100939540 (-) |  | -0.446 | 1.07e-08 | 6.69e-07 |
| ENSMUSG00000029925 | Tbxas1 | protein\_coding | 6:38875404-39084585 (+) |  | 0.930 | 1.09e-08 | 6.83e-07 |
| ENSMUSG00000020844 | Nxn | protein\_coding | 11:76257198-76399140 (-) |  | -0.583 | 1.12e-08 | 6.95e-07 |
| ENSMUSG00000022353 | Mtss1 | protein\_coding | 15:58941234-59082005 (-) |  | 0.998 | 1.14e-08 | 7.00e-07 |
| ENSMUSG00000020238 | Ncln | protein\_coding | 10:81486249-81496392 (-) |  | -0.316 | 1.14e-08 | 7.00e-07 |
| ENSMUSG00000056643 | Chst13 | protein\_coding | 6:90308349-90325185 (-) |  | 0.904 | 1.15e-08 | 7.00e-07 |
| ENSMUSG00000029623 | Pdap1 | protein\_coding | 5:145128769-145140238 (-) |  | -0.511 | 1.15e-08 | 7.00e-07 |
| ENSMUSG00000004356 | Utp20 | protein\_coding | 10:88746607-88826804 (-) |  | -0.514 | 1.15e-08 | 7.00e-07 |
| ENSMUSG00000020785 | Camkk1 | protein\_coding | 11:73019008-73042073 (+) |  | -1.640 | 1.17e-08 | 7.06e-07 |
| ENSMUSG00000081603 | Gm14681 | processed\_pseudogene | X:66778442-66778852 (-) |  | -0.562 | 1.20e-08 | 7.22e-07 |
| ENSMUSG00000020116 | Pno1 | protein\_coding | 11:17203198-17211568 (-) |  | -0.665 | 1.23e-08 | 7.37e-07 |
| ENSMUSG00000027804 | Ppid | protein\_coding | 3:79591342-79603650 (+) |  | -0.523 | 1.27e-08 | 7.59e-07 |
| ENSMUSG00000021474 | Sfxn1 | protein\_coding | 13:54071869-54108342 (+) |  | -0.399 | 1.31e-08 | 7.78e-07 |
| ENSMUSG00000063952 | Brpf3 | protein\_coding | 17:28801090-28839949 (+) |  | 0.508 | 1.35e-08 | 8.00e-07 |
| ENSMUSG00000063193 | Cd300lb | protein\_coding | 11:114922781-114934386 (-) |  | 0.630 | 1.41e-08 | 8.29e-07 |
| ENSMUSG00000020361 | Hspa4 | protein\_coding | 11:53259814-53300457 (-) |  | -0.409 | 1.43e-08 | 8.39e-07 |
| ENSMUSG00000059791 | Nrm | protein\_coding | 17:35861318-35865402 (+) |  | 0.453 | 1.44e-08 | 8.39e-07 |
| ENSMUSG00000028337 | Coro2a | protein\_coding | 4:46536937-46602202 (-) |  | 0.370 | 1.46e-08 | 8.46e-07 |
| ENSMUSG00000031216 | Stard8 | protein\_coding | X:99003248-99074728 (+) |  | 0.619 | 1.60e-08 | 9.27e-07 |
| ENSMUSG00000039005 | Tlr4 | protein\_coding | 4:66827584-66930284 (+) |  | -0.749 | 1.64e-08 | 9.44e-07 |
| ENSMUSG00000036112 | Metap2 | protein\_coding | 10:93858489-93897093 (-) |  | -0.417 | 1.74e-08 | 9.94e-07 |
| ENSMUSG00000017132 | Cyth1 | protein\_coding | 11:118132019-118248592 (-) |  | 0.380 | 1.77e-08 | 1.01e-06 |
| ENSMUSG00000028683 | Eif2b3 | protein\_coding | 4:117019402-117087306 (+) |  | -0.597 | 1.79e-08 | 1.02e-06 |
| ENSMUSG00000031657 | Heatr3 | protein\_coding | 8:88137855-88172027 (+) |  | -0.552 | 1.83e-08 | 1.03e-06 |
| ENSMUSG00000098557 | Kctd12 | protein\_coding | 14:102976581-102982637 (-) |  | 0.425 | 1.84e-08 | 1.04e-06 |
| ENSMUSG00000028268 | Gbp3 | protein\_coding | 3:142560026-142573209 (+) |  | -0.425 | 1.89e-08 | 1.06e-06 |
| ENSMUSG00000057133 | Chd6 | protein\_coding | 2:160946978-161109075 (-) |  | 0.496 | 1.90e-08 | 1.06e-06 |
| ENSMUSG00000039159 | Ube2h | protein\_coding | 6:30211289-30304539 (-) |  | -0.542 | 1.91e-08 | 1.06e-06 |
| ENSMUSG00000051984 | Sec31b | protein\_coding | 19:44516957-44545864 (-) |  | 1.290 | 2.06e-08 | 1.14e-06 |
| ENSMUSG00000023952 | Gtpbp2 | protein\_coding | 17:46161032-46169370 (+) |  | 0.462 | 2.08e-08 | 1.14e-06 |
| ENSMUSG00000011179 | Odc1 | protein\_coding | 12:17544794-17551505 (+) |  | -0.637 | 2.15e-08 | 1.18e-06 |
| ENSMUSG00000023025 | Larp4 | protein\_coding | 15:99970065-100016358 (+) |  | -0.390 | 2.23e-08 | 1.22e-06 |
| ENSMUSG00000030148 | Clec4a2 | protein\_coding | 6:123106428-123143999 (+) |  | 0.822 | 2.37e-08 | 1.29e-06 |
| ENSMUSG00000034792 | Gna15 | protein\_coding | 10:81502306-81524225 (-) |  | 0.512 | 2.40e-08 | 1.30e-06 |
| ENSMUSG00000010048 | Ifrd2 | protein\_coding | 9:107587642-107593385 (+) |  | -0.595 | 2.45e-08 | 1.32e-06 |
| ENSMUSG00000029922 | Mkrn1 | protein\_coding | 6:39397804-39420462 (-) |  | 0.439 | 2.68e-08 | 1.43e-06 |
| ENSMUSG00000057729 | Prtn3 | protein\_coding | 10:79874476-79883174 (+) |  | -0.545 | 2.68e-08 | 1.43e-06 |
| ENSMUSG00000030711 | Sult1a1 | protein\_coding | 7:126672865-126676432 (-) |  | 1.450 | 2.72e-08 | 1.45e-06 |
| ENSMUSG00000041324 | Inhba | protein\_coding | 13:16011851-16031621 (+) |  | -3.450 | 2.74e-08 | 1.45e-06 |
| ENSMUSG00000025375 | Aatk | protein\_coding | 11:120007313-120047167 (-) |  | 0.795 | 2.77e-08 | 1.46e-06 |
| ENSMUSG00000021948 | Prkcd | protein\_coding | 14:30595354-30626210 (-) |  | 0.461 | 2.79e-08 | 1.46e-06 |
| ENSMUSG00000024948 | Map4k2 | protein\_coding | 19:6341135-6355615 (+) |  | 0.540 | 2.80e-08 | 1.46e-06 |
| ENSMUSG00000052533 | Nup188 | protein\_coding | 2:30286397-30344266 (+) |  | -0.353 | 2.80e-08 | 1.46e-06 |
| ENSMUSG00000070319 | Eif3g | protein\_coding | 9:20894349-20898623 (-) |  | -0.389 | 2.90e-08 | 1.51e-06 |
| ENSMUSG00000045427 | Hnrnph2 | protein\_coding | X:134601179-134607060 (+) |  | -0.383 | 2.94e-08 | 1.52e-06 |
| ENSMUSG00000001305 | Rrp15 | protein\_coding | 1:186720978-186749358 (-) |  | -0.455 | 2.99e-08 | 1.54e-06 |
| ENSMUSG00000001380 | Hars | protein\_coding | 18:36766528-36783205 (-) |  | -0.373 | 3.10e-08 | 1.59e-06 |
| ENSMUSG00000037278 | Tmem97 | protein\_coding | 11:78541817-78550777 (-) |  | -0.602 | 3.16e-08 | 1.62e-06 |
| ENSMUSG00000024063 | Lbh | protein\_coding | 17:72918305-72941947 (+) |  | 0.501 | 3.31e-08 | 1.69e-06 |
| ENSMUSG00000054619 | Mettl7a1 | protein\_coding | 15:100304140-100328662 (+) |  | 0.554 | 3.46e-08 | 1.76e-06 |
| ENSMUSG00000020720 | Psmd12 | protein\_coding | 11:107479484-107504362 (+) |  | -0.465 | 3.57e-08 | 1.81e-06 |
| ENSMUSG00000021614 | Vcan | protein\_coding | 13:89655312-89742509 (-) |  | -3.180 | 3.63e-08 | 1.82e-06 |
| ENSMUSG00000057789 | Bak1 | protein\_coding | 17:27019810-27029009 (-) |  | -0.369 | 3.63e-08 | 1.82e-06 |
| ENSMUSG00000057113 | Npm1 | protein\_coding | 11:33152287-33163206 (-) |  | -0.553 | 3.67e-08 | 1.83e-06 |
| ENSMUSG00000052825 | Gm9892 | processed\_pseudogene | 8:52196065-52197056 (-) |  | -0.655 | 3.77e-08 | 1.88e-06 |
| ENSMUSG00000031304 | Il2rg | protein\_coding | X:101264378-101268255 (-) |  | -0.783 | 3.79e-08 | 1.88e-06 |
| ENSMUSG00000068329 | Htra2 | protein\_coding | 6:83051266-83055273 (-) |  | -0.384 | 3.90e-08 | 1.93e-06 |
| ENSMUSG00000029617 | Ccz1 | protein\_coding | 5:143987909-144014877 (-) |  | -0.379 | 3.95e-08 | 1.95e-06 |
| ENSMUSG00000020883 | Fbxl20 | protein\_coding | 11:98082556-98150403 (-) |  | 0.627 | 4.12e-08 | 2.02e-06 |
| ENSMUSG00000019850 | Tnfaip3 | protein\_coding | 10:19000910-19015657 (-) |  | 0.814 | 4.28e-08 | 2.09e-06 |
| ENSMUSG00000021037 | Ahsa1 | protein\_coding | 12:87266479-87273998 (+) |  | -0.467 | 4.50e-08 | 2.19e-06 |
| ENSMUSG00000016256 | Ctsz | protein\_coding | 2:174427493-174439039 (-) |  | -0.341 | 4.57e-08 | 2.22e-06 |
| ENSMUSG00000087150 | BC064078 | transcribed\_unprocessed\_pseudogene | 6:128992952-129008040 (+) |  | 0.743 | 4.59e-08 | 2.22e-06 |
| ENSMUSG00000020635 | Fkbp1b | protein\_coding | 12:4833174-4841591 (-) |  | 0.655 | 4.62e-08 | 2.22e-06 |
| ENSMUSG00000055053 | Nfic | protein\_coding | 10:81396186-81455635 (-) |  | 0.436 | 4.70e-08 | 2.25e-06 |
| ENSMUSG00000024360 | Etf1 | protein\_coding | 18:34902785-34932007 (-) |  | -0.444 | 4.82e-08 | 2.30e-06 |
| ENSMUSG00000029014 | Dnajc2 | protein\_coding | 5:21757267-21785251 (-) |  | -0.385 | 4.86e-08 | 2.31e-06 |
| ENSMUSG00000028270 | Gbp2 | protein\_coding | 3:142620602-142638008 (+) |  | -0.756 | 4.92e-08 | 2.33e-06 |
| ENSMUSG00000042354 | Gnl3 | protein\_coding | 14:31012433-31019152 (-) |  | -0.433 | 4.98e-08 | 2.35e-06 |
| ENSMUSG00000042688 | Mapk6 | protein\_coding | 9:75369062-75410005 (-) |  | -0.456 | 5.25e-08 | 2.47e-06 |
| ENSMUSG00000034041 | Lyl1 | protein\_coding | 8:84701449-84704940 (+) |  | 0.328 | 5.62e-08 | 2.64e-06 |
| ENSMUSG00000024966 | Stip1 | protein\_coding | 19:7020702-7039967 (-) |  | -0.447 | 5.74e-08 | 2.68e-06 |
| ENSMUSG00000042284 | Itga1 | protein\_coding | 13:114953096-115101964 (-) |  | 0.626 | 5.84e-08 | 2.72e-06 |
| ENSMUSG00000028420 | Tmem38b | protein\_coding | 4:53826045-53862019 (+) |  | -0.511 | 5.85e-08 | 2.72e-06 |
| ENSMUSG00000036167 | Pphln1 | protein\_coding | 15:93398350-93491510 (+) |  | -0.394 | 6.01e-08 | 2.78e-06 |
| ENSMUSG00000008450 | Nutf2 | protein\_coding | 8:105860580-105879330 (+) |  | -0.616 | 6.06e-08 | 2.79e-06 |
| ENSMUSG00000028069 | Gpatch4 | protein\_coding | 3:88043108-88055993 (+) |  | -0.479 | 6.51e-08 | 2.99e-06 |
| ENSMUSG00000062203 | Gspt1 | protein\_coding | 16:11219292-11254325 (-) |  | -0.437 | 6.82e-08 | 3.11e-06 |
| ENSMUSG00000010142 | Tnfrsf13b | protein\_coding | 11:61126755-61149372 (+) |  | 0.367 | 6.83e-08 | 3.11e-06 |
| ENSMUSG00000024841 | Eif1ad | protein\_coding | 19:5366741-5371526 (+) |  | -0.362 | 6.96e-08 | 3.16e-06 |
| ENSMUSG00000051811 | Cox6b2 | protein\_coding | 7:4751792-4753094 (-) |  | 0.839 | 6.98e-08 | 3.16e-06 |
| ENSMUSG00000041057 | Wdr43 | protein\_coding | 17:71615895-71659031 (+) |  | -0.367 | 7.03e-08 | 3.17e-06 |
| ENSMUSG00000020224 | Llph | protein\_coding | 10:120227070-120232582 (+) |  | -0.537 | 7.08e-08 | 3.18e-06 |
| ENSMUSG00000000787 | Ddx3x | protein\_coding | X:13280970-13294052 (+) |  | -0.375 | 7.46e-08 | 3.34e-06 |
| ENSMUSG00000044468 | Tent5c | protein\_coding | 3:100451628-100489324 (-) |  | 1.520 | 7.51e-08 | 3.35e-06 |
| ENSMUSG00000005142 | Man2b1 | protein\_coding | 8:85083270-85098282 (+) |  | 0.434 | 7.56e-08 | 3.36e-06 |
| ENSMUSG00000020706 | Ftsj3 | protein\_coding | 11:106249142-106256079 (-) |  | -0.415 | 7.63e-08 | 3.38e-06 |
| ENSMUSG00000022557 | Bop1 | protein\_coding | 15:76452989-76477277 (-) |  | -0.446 | 7.67e-08 | 3.38e-06 |
| ENSMUSG00000036528 | Ppfibp2 | protein\_coding | 7:107595207-107748583 (+) |  | 0.817 | 7.68e-08 | 3.38e-06 |
| ENSMUSG00000033294 | Noc4l | protein\_coding | 5:110648418-110653417 (-) |  | -0.521 | 8.16e-08 | 3.58e-06 |
| ENSMUSG00000031834 | Pik3r2 | protein\_coding | 8:70768176-70776713 (-) |  | -0.402 | 8.23e-08 | 3.60e-06 |
| ENSMUSG00000048924 | Ccdc125 | protein\_coding | 13:100669717-100697240 (+) |  | 0.503 | 8.27e-08 | 3.60e-06 |
| ENSMUSG00000079547 | H2-DMb1 | protein\_coding | 17:34153072-34160230 (+) |  | 0.609 | 8.64e-08 | 3.75e-06 |
| ENSMUSG00000020368 | Canx | protein\_coding | 11:50293961-50325673 (-) |  | -0.441 | 8.73e-08 | 3.78e-06 |
| ENSMUSG00000031838 | Ifi30 | lncRNA | 8:70762774-70766663 (-) |  | -0.495 | 8.80e-08 | 3.80e-06 |
| ENSMUSG00000030413 | Pglyrp1 | protein\_coding | 7:18871331-18890459 (+) |  | 1.090 | 9.02e-08 | 3.88e-06 |
| ENSMUSG00000031167 | Rbm3 | protein\_coding | X:8138975-8145880 (-) |  | 0.428 | 9.09e-08 | 3.90e-06 |
| ENSMUSG00000021116 | Eif2s1 | protein\_coding | 12:78861819-78887010 (+) |  | -0.449 | 9.16e-08 | 3.91e-06 |
| ENSMUSG00000002083 | Bbc3 | protein\_coding | 7:16308393-16318205 (+) |  | 1.080 | 9.37e-08 | 3.99e-06 |
| ENSMUSG00000021831 | Ero1l | protein\_coding | 14:45283087-45318771 (-) |  | -0.437 | 9.46e-08 | 4.01e-06 |
| ENSMUSG00000021880 | Rnase6 | protein\_coding | 14:51123908-51132187 (+) |  | 0.808 | 1.02e-07 | 4.31e-06 |
| ENSMUSG00000074129 | Rpl13a | protein\_coding | 7:45125558-45128761 (-) |  | -0.483 | 1.06e-07 | 4.47e-06 |
| ENSMUSG00000018433 | Nol11 | protein\_coding | 11:107166663-107189381 (-) |  | -0.402 | 1.06e-07 | 4.47e-06 |
| ENSMUSG00000042500 | Ago4 | protein\_coding | 4:126489541-126533472 (-) |  | 0.761 | 1.09e-07 | 4.57e-06 |
| ENSMUSG00000029430 | Ran | protein\_coding | 5:129020069-129024323 (+) |  | -0.454 | 1.15e-07 | 4.80e-06 |
| ENSMUSG00000024247 | Pkdcc | protein\_coding | 17:83215292-83225070 (+) |  | 1.140 | 1.17e-07 | 4.85e-06 |
| ENSMUSG00000068856 | Sf3b4 | protein\_coding | 3:96172332-96177564 (+) |  | -0.377 | 1.18e-07 | 4.92e-06 |
| ENSMUSG00000024981 | Acsl5 | protein\_coding | 19:55251938-55297720 (+) |  | -0.400 | 1.20e-07 | 4.98e-06 |
| ENSMUSG00000062901 | Klhl24 | protein\_coding | 16:20097542-20129221 (+) |  | 0.471 | 1.26e-07 | 5.20e-06 |
| ENSMUSG00000028851 | Nudc | protein\_coding | 4:133532542-133545996 (-) |  | -0.391 | 1.27e-07 | 5.21e-06 |
| ENSMUSG00000005610 | Eif4g2 | protein\_coding | 7:111067750-111083030 (-) |  | -0.381 | 1.27e-07 | 5.21e-06 |
| ENSMUSG00000000440 | Pparg | protein\_coding | 6:115360951-115490399 (+) |  | 1.810 | 1.35e-07 | 5.52e-06 |
| ENSMUSG00000026234 | Ncl | protein\_coding | 1:86344719-86359400 (-) |  | -0.525 | 1.36e-07 | 5.53e-06 |
| ENSMUSG00000030747 | Dgat2 | protein\_coding | 7:99153658-99182719 (-) |  | -0.769 | 1.38e-07 | 5.62e-06 |
| ENSMUSG00000022403 | St13 | protein\_coding | 15:81363669-81400077 (-) |  | -0.411 | 1.40e-07 | 5.65e-06 |
| ENSMUSG00000030609 | Aen | protein\_coding | 7:78895854-78911209 (+) |  | -0.461 | 1.44e-07 | 5.79e-06 |
| ENSMUSG00000082099 | Gm12013 | processed\_pseudogene | 11:17095490-17095644 (-) |  | 1.390 | 1.45e-07 | 5.84e-06 |
| ENSMUSG00000079225 | Gm9531 | transcribed\_processed\_pseudogene | 9:81677598-81678677 (+) |  | -0.458 | 1.50e-07 | 6.00e-06 |
| ENSMUSG00000041313 | Slc7a1 | protein\_coding | 5:148327410-148399904 (-) |  | -0.416 | 1.50e-07 | 6.00e-06 |
| ENSMUSG00000030681 | Mvp | protein\_coding | 7:126986860-127014621 (-) |  | -0.443 | 1.51e-07 | 6.02e-06 |
| ENSMUSG00000027405 | Nop56 | protein\_coding | 2:130274430-130279313 (+) |  | -0.309 | 1.52e-07 | 6.02e-06 |
| ENSMUSG00000072694 | 1500011B03Rik | protein\_coding | 5:114808196-114823468 (-) |  | 0.594 | 1.55e-07 | 6.13e-06 |
| ENSMUSG00000000686 | Abhd15 | protein\_coding | 11:77515121-77538607 (+) |  | 0.689 | 1.56e-07 | 6.13e-06 |
| ENSMUSG00000078515 | Ddi2 | protein\_coding | 4:141677549-141723419 (-) |  | -0.407 | 1.56e-07 | 6.13e-06 |
| ENSMUSG00000019179 | Mdh2 | protein\_coding | 5:135778480-135790398 (+) |  | -0.434 | 1.59e-07 | 6.23e-06 |
| ENSMUSG00000040811 | Eml2 | protein\_coding | 7:19176421-19206482 (+) |  | 0.436 | 1.59e-07 | 6.23e-06 |
| ENSMUSG00000000561 | Wdr77 | protein\_coding | 3:105959369-105970037 (+) |  | -0.412 | 1.60e-07 | 6.25e-06 |
| ENSMUSG00000020272 | Stk10 | protein\_coding | 11:32533305-32624587 (+) |  | 0.563 | 1.63e-07 | 6.33e-06 |
| ENSMUSG00000018446 | C1qbp | protein\_coding | 11:70977836-70983026 (-) |  | -0.561 | 1.68e-07 | 6.53e-06 |
| ENSMUSG00000063273 | Naa15 | protein\_coding | 3:51415148-51476507 (+) |  | -0.375 | 1.69e-07 | 6.55e-06 |
| ENSMUSG00000020021 | Fgd6 | protein\_coding | 10:94036001-94145339 (+) |  | -1.060 | 1.71e-07 | 6.61e-06 |
| ENSMUSG00000020120 | Plek | protein\_coding | 11:16971206-17052381 (-) |  | 0.608 | 1.72e-07 | 6.61e-06 |
| ENSMUSG00000037151 | Lrrc20 | protein\_coding | 10:61475801-61582791 (+) |  | 0.530 | 1.73e-07 | 6.61e-06 |
| ENSMUSG00000036718 | Micall2 | protein\_coding | 5:139706696-139736336 (-) |  | 0.534 | 1.80e-07 | 6.87e-06 |
| ENSMUSG00000002718 | Cse1l | protein\_coding | 2:166906040-166946389 (+) |  | -0.363 | 1.86e-07 | 7.10e-06 |
| ENSMUSG00000014470 | Rnf166 | protein\_coding | 8:122466147-122476064 (-) |  | 0.501 | 1.87e-07 | 7.10e-06 |
| ENSMUSG00000037242 | Clic4 | protein\_coding | 4:135213969-135272814 (-) |  | -0.319 | 1.88e-07 | 7.14e-06 |
| ENSMUSG00000027253 | Lrp4 | protein\_coding | 2:91457511-91513779 (+) |  | 0.744 | 1.91e-07 | 7.22e-06 |
| ENSMUSG00000021595 | Nsun2 | protein\_coding | 13:69533746-69635780 (+) |  | -0.462 | 1.96e-07 | 7.39e-06 |
| ENSMUSG00000051314 | Ffar2 | protein\_coding | 7:30818348-30823775 (-) |  | -1.140 | 1.97e-07 | 7.40e-06 |
| ENSMUSG00000020349 | Ppp2ca | protein\_coding | 11:52098681-52127778 (+) |  | -0.450 | 1.98e-07 | 7.40e-06 |
| ENSMUSG00000115338 | Pnp | protein\_coding | 14:50931082-50965237 (+) |  | -0.498 | 2.01e-07 | 7.50e-06 |
| ENSMUSG00000019494 | Cops6 | protein\_coding | 5:138161071-138164646 (+) |  | -0.341 | 2.04e-07 | 7.58e-06 |
| ENSMUSG00000040430 | Pitpnc1 | protein\_coding | 11:107207892-107470699 (-) |  | 0.592 | 2.10e-07 | 7.81e-06 |
| ENSMUSG00000053470 | Kdm3a | protein\_coding | 6:71588972-71632990 (-) |  | 0.406 | 2.15e-07 | 7.95e-06 |
| ENSMUSG00000002733 | Plekha3 | protein\_coding | 2:76675281-76696828 (+) |  | -0.628 | 2.17e-07 | 8.02e-06 |
| ENSMUSG00000056999 | Ide | protein\_coding | 19:37268743-37337852 (-) |  | -0.412 | 2.19e-07 | 8.08e-06 |
| ENSMUSG00000002068 | Ccne1 | protein\_coding | 7:38097984-38107534 (-) |  | -0.467 | 2.23e-07 | 8.17e-06 |
| ENSMUSG00000022555 | Dgat1 | protein\_coding | 15:76502015-76511953 (-) |  | 0.546 | 2.24e-07 | 8.19e-06 |
| ENSMUSG00000001440 | Kpnb1 | protein\_coding | 11:97159714-97187881 (-) |  | -0.403 | 2.24e-07 | 8.19e-06 |
| ENSMUSG00000031486 | Adgra2 | protein\_coding | 8:27085583-27123436 (+) |  | 1.280 | 2.30e-07 | 8.35e-06 |
| ENSMUSG00000017221 | Psmd3 | protein\_coding | 11:98682554-98695979 (+) |  | -0.414 | 2.31e-07 | 8.39e-06 |
| ENSMUSG00000019806 | Aig1 | protein\_coding | 10:13647054-13868980 (-) |  | -0.747 | 2.33e-07 | 8.44e-06 |
| ENSMUSG00000026864 | Hspa5 | protein\_coding | 2:34771970-34777547 (+) |  | -0.548 | 2.39e-07 | 8.61e-06 |
| ENSMUSG00000079685 | Ulbp1 | protein\_coding | 10:7440362-7473638 (-) |  | 0.470 | 2.44e-07 | 8.79e-06 |
| ENSMUSG00000036986 | Pml | protein\_coding | 9:58218076-58249786 (-) |  | 0.385 | 2.46e-07 | 8.82e-06 |
| ENSMUSG00000032271 | Nnmt | protein\_coding | 9:48591877-48605153 (-) |  | -2.860 | 2.48e-07 | 8.86e-06 |
| ENSMUSG00000045322 | Tlr9 | protein\_coding | 9:106222598-106226883 (+) |  | 0.576 | 2.48e-07 | 8.86e-06 |
| ENSMUSG00000028614 | Ndc1 | protein\_coding | 4:107367784-107416346 (+) |  | -0.337 | 2.56e-07 | 9.11e-06 |
| ENSMUSG00000008373 | Prpf31 | protein\_coding | 7:3629985-3642486 (+) |  | -0.406 | 2.67e-07 | 9.47e-06 |
| ENSMUSG00000004709 | Cd244a | protein\_coding | 1:171559193-171609746 (+) |  | -0.607 | 2.69e-07 | 9.51e-06 |
| ENSMUSG00000057561 | Eif1a | protein\_coding | 18:46597701-46616456 (+) |  | -0.413 | 2.72e-07 | 9.61e-06 |
| ENSMUSG00000004846 | Plod3 | protein\_coding | 5:136987019-136996648 (+) |  | -0.345 | 2.76e-07 | 9.72e-06 |
| ENSMUSG00000004609 | Cd33 | protein\_coding | 7:43524216-43544428 (-) |  | 0.857 | 2.87e-07 | 1.00e-05 |
| ENSMUSG00000040820 | Hlcs | protein\_coding | 16:94128882-94313571 (-) |  | 0.607 | 2.87e-07 | 1.00e-05 |
| ENSMUSG00000030036 | Mogs | protein\_coding | 6:83115496-83118898 (+) |  | -0.386 | 2.87e-07 | 1.00e-05 |
| ENSMUSG00000055762 | Eef1d | protein\_coding | 15:75894205-75909556 (-) |  | -0.452 | 2.88e-07 | 1.00e-05 |
| ENSMUSG00000070348 | Ccnd1 | protein\_coding | 7:144929931-144939925 (-) |  | 0.796 | 2.91e-07 | 1.01e-05 |
| ENSMUSG00000030224 | Strap | protein\_coding | 6:137735078-137751932 (+) |  | -0.426 | 2.97e-07 | 1.03e-05 |
| ENSMUSG00000020089 | Ppa1 | protein\_coding | 10:61648552-61674168 (+) |  | -0.662 | 2.99e-07 | 1.03e-05 |
| ENSMUSG00000053113 | Socs3 | protein\_coding | 11:117966079-117970047 (-) |  | -3.100 | 2.99e-07 | 1.03e-05 |
| ENSMUSG00000025823 | Pdia4 | protein\_coding | 6:47796141-47813430 (-) |  | -0.514 | 3.01e-07 | 1.03e-05 |
| ENSMUSG00000034445 | Cyb561a3 | protein\_coding | 19:10577172-10595961 (+) |  | 0.425 | 3.03e-07 | 1.04e-05 |
| ENSMUSG00000016757 | Ttll12 | protein\_coding | 15:83575090-83595157 (-) |  | -0.547 | 3.04e-07 | 1.04e-05 |
| ENSMUSG00000021952 | Xpo4 | protein\_coding | 14:57577521-57665430 (-) |  | -0.381 | 3.07e-07 | 1.05e-05 |
| ENSMUSG00000021738 | Atxn7 | protein\_coding | 14:13961440-14107302 (+) |  | 0.435 | 3.11e-07 | 1.06e-05 |
| ENSMUSG00000020549 | Elac2 | protein\_coding | 11:64979038-65002069 (+) |  | -0.407 | 3.13e-07 | 1.06e-05 |
| ENSMUSG00000045038 | Prkce | protein\_coding | 17:86167785-86657919 (+) |  | 0.642 | 3.15e-07 | 1.06e-05 |
| ENSMUSG00000024613 | Tcof1 | protein\_coding | 18:60813755-60848971 (-) |  | -0.342 | 3.16e-07 | 1.06e-05 |
| ENSMUSG00000020307 | Cdc34 | protein\_coding | 10:79682195-79688398 (+) |  | -0.420 | 3.37e-07 | 1.13e-05 |
| ENSMUSG00000027175 | Tcp11l1 | protein\_coding | 2:104657288-104712169 (-) |  | -0.689 | 3.44e-07 | 1.15e-05 |
| ENSMUSG00000040327 | Cul9 | protein\_coding | 17:46500572-46546388 (-) |  | 0.684 | 3.53e-07 | 1.18e-05 |
| ENSMUSG00000058006 | Mdn1 | protein\_coding | 4:32657119-32775217 (+) |  | -0.458 | 3.54e-07 | 1.18e-05 |
| ENSMUSG00000052459 | Atp6v1a | protein\_coding | 16:44085402-44139705 (-) |  | -0.323 | 3.61e-07 | 1.20e-05 |
| ENSMUSG00000032575 | Manf | protein\_coding | 9:106838312-106891979 (-) |  | -0.452 | 3.64e-07 | 1.21e-05 |
| ENSMUSG00000023272 | Creld2 | protein\_coding | 15:88819646-88826683 (+) |  | -0.466 | 3.72e-07 | 1.23e-05 |
| ENSMUSG00000020572 | Nampt | protein\_coding | 12:32819545-32853349 (+) |  | -0.373 | 3.77e-07 | 1.24e-05 |
| ENSMUSG00000059479 | B3gnt8 | protein\_coding | 7:25626654-25635100 (+) |  | 0.485 | 3.78e-07 | 1.25e-05 |
| ENSMUSG00000068551 | Zfp467 | protein\_coding | 6:48427697-48445825 (-) |  | 0.810 | 3.93e-07 | 1.29e-05 |
| ENSMUSG00000018583 | G3bp1 | protein\_coding | 11:55469685-55504838 (+) |  | -0.335 | 4.08e-07 | 1.34e-05 |
| ENSMUSG00000001707 | Eef1e1 | protein\_coding | 13:38644207-38659058 (-) |  | -0.530 | 4.19e-07 | 1.37e-05 |
| ENSMUSG00000022427 | Tomm22 | protein\_coding | 15:79670861-79673400 (+) |  | -0.390 | 4.24e-07 | 1.38e-05 |
| ENSMUSG00000041890 | Git2 | protein\_coding | 5:114727407-114775517 (-) |  | 0.288 | 4.25e-07 | 1.38e-05 |
| ENSMUSG00000024999 | Noc3l | protein\_coding | 19:38788128-38819237 (-) |  | -0.426 | 4.67e-07 | 1.52e-05 |
| ENSMUSG00000022389 | Tef | protein\_coding | 15:81802421-81826863 (+) |  | 0.916 | 4.72e-07 | 1.53e-05 |
| ENSMUSG00000015656 | Hspa8 | protein\_coding | 9:40800984-40810087 (+) |  | -0.370 | 4.95e-07 | 1.60e-05 |
| ENSMUSG00000022453 | Naga | protein\_coding | 15:82329532-82338925 (-) |  | 0.385 | 5.17e-07 | 1.66e-05 |
| ENSMUSG00000024986 | Hhex | protein\_coding | 19:37434810-37440731 (+) |  | 0.415 | 5.25e-07 | 1.69e-05 |
| ENSMUSG00000034610 | Tut4 | protein\_coding | 4:108459426-108559421 (+) |  | 0.408 | 5.29e-07 | 1.70e-05 |
| ENSMUSG00000036304 | Zdhhc23 | protein\_coding | 16:43965033-43979791 (-) |  | -0.912 | 5.30e-07 | 1.70e-05 |
| ENSMUSG00000057541 | Pus7 | protein\_coding | 5:23740648-23783711 (-) |  | -0.488 | 5.36e-07 | 1.71e-05 |
| ENSMUSG00000025868 | Higd2a | protein\_coding | 13:54590207-54591158 (+) |  | 0.431 | 5.50e-07 | 1.75e-05 |
| ENSMUSG00000022769 | Sdf2l1 | protein\_coding | 16:17130138-17132383 (-) |  | -0.468 | 5.50e-07 | 1.75e-05 |
| ENSMUSG00000019944 | Rhobtb1 | protein\_coding | 10:69151434-69291791 (+) |  | 1.450 | 5.52e-07 | 1.75e-05 |
| ENSMUSG00000021365 | Nedd9 | protein\_coding | 13:41309581-41487362 (-) |  | 0.501 | 5.56e-07 | 1.76e-05 |
| ENSMUSG00000005107 | Slc2a9 | protein\_coding | 5:38349273-38503143 (-) |  | 0.659 | 5.70e-07 | 1.80e-05 |
| ENSMUSG00000033720 | Sfxn5 | protein\_coding | 6:85213049-85333422 (-) |  | -0.920 | 5.77e-07 | 1.81e-05 |
| ENSMUSG00000031613 | Hpgd | protein\_coding | 8:56294585-56321043 (+) |  | 1.290 | 5.78e-07 | 1.81e-05 |
| ENSMUSG00000063229 | Ldha | protein\_coding | 7:46841475-46855627 (+) |  | -0.509 | 5.91e-07 | 1.85e-05 |
| ENSMUSG00000030605 | Mfge8 | protein\_coding | 7:79133768-79149060 (-) |  | 0.505 | 5.91e-07 | 1.85e-05 |
| ENSMUSG00000010755 | Cars | protein\_coding | 7:143557230-143600090 (-) |  | -0.490 | 6.00e-07 | 1.87e-05 |
| ENSMUSG00000044103 | Il1f9 | protein\_coding | 2:24186476-24193568 (+) |  | -2.210 | 6.08e-07 | 1.89e-05 |
| ENSMUSG00000030269 | Mtmr14 | protein\_coding | 6:113237843-113281392 (+) |  | 0.435 | 6.13e-07 | 1.90e-05 |
| ENSMUSG00000056069 | Otulinl | protein\_coding | 15:27655069-27681579 (-) |  | 0.342 | 6.23e-07 | 1.93e-05 |
| ENSMUSG00000018377 | Vezf1 | protein\_coding | 11:88068279-88084729 (+) |  | 0.401 | 6.38e-07 | 1.97e-05 |
| ENSMUSG00000025995 | Wdr75 | protein\_coding | 1:45795166-45823619 (+) |  | -0.446 | 6.44e-07 | 1.98e-05 |
| ENSMUSG00000001436 | Slc19a1 | protein\_coding | 10:77032241-77061002 (+) |  | -0.589 | 6.62e-07 | 2.03e-05 |
| ENSMUSG00000056737 | Capg | protein\_coding | 6:72544391-72562983 (+) |  | 0.591 | 6.62e-07 | 2.03e-05 |
| ENSMUSG00000023055 | Calcoco1 | protein\_coding | 15:102706777-102722178 (-) |  | 0.880 | 6.66e-07 | 2.03e-05 |
| ENSMUSG00000004069 | Dnaja3 | protein\_coding | 16:4639989-4707695 (+) |  | -0.382 | 6.74e-07 | 2.06e-05 |
| ENSMUSG00000023367 | Tmem176a | protein\_coding | 6:48840919-48847071 (+) |  | -0.491 | 6.79e-07 | 2.06e-05 |
| ENSMUSG00000004268 | Emg1 | protein\_coding | 6:124704085-124712178 (-) |  | -0.374 | 6.80e-07 | 2.06e-05 |
| ENSMUSG00000002997 | Prkar2b | protein\_coding | 12:31958476-32061296 (-) |  | 0.311 | 6.85e-07 | 2.07e-05 |
| ENSMUSG00000024150 | Mcfd2 | protein\_coding | 17:87254443-87265935 (-) |  | -0.393 | 6.95e-07 | 2.10e-05 |
| ENSMUSG00000021069 | Pygl | protein\_coding | 12:70190811-70231488 (-) |  | 0.418 | 7.00e-07 | 2.11e-05 |
| ENSMUSG00000091811 | Inafm1 | protein\_coding | 7:16272013-16273617 (-) |  | 0.695 | 7.03e-07 | 2.11e-05 |
| ENSMUSG00000010554 | Mettl16 | protein\_coding | 11:74770830-74828525 (+) |  | -0.461 | 7.26e-07 | 2.18e-05 |
| ENSMUSG00000038970 | Lmtk2 | protein\_coding | 5:144100436-144188204 (+) |  | 0.477 | 7.30e-07 | 2.19e-05 |
| ENSMUSG00000029467 | Atp2a2 | protein\_coding | 5:122453513-122502225 (-) |  | -0.315 | 7.48e-07 | 2.23e-05 |
| ENSMUSG00000047293 | Gpr15 | protein\_coding | 16:58717433-58719070 (-) |  | -1.280 | 7.54e-07 | 2.25e-05 |
| ENSMUSG00000008206 | Cers4 | protein\_coding | 8:4493026-4531680 (+) |  | 0.988 | 7.61e-07 | 2.26e-05 |
| ENSMUSG00000031432 | Prps1 | protein\_coding | X:140456613-140476140 (+) |  | -0.380 | 7.63e-07 | 2.26e-05 |
| ENSMUSG00000035150 | Eif2s3x | protein\_coding | X:94188707-94212862 (-) |  | -0.454 | 7.70e-07 | 2.27e-05 |
| ENSMUSG00000087177 | E130307A14Rik | lncRNA | 10:39621412-39732007 (-) |  | 0.746 | 7.71e-07 | 2.27e-05 |
| ENSMUSG00000040463 | Mybbp1a | protein\_coding | 11:72441355-72451768 (+) |  | -0.511 | 7.72e-07 | 2.27e-05 |
| ENSMUSG00000025270 | Alas2 | protein\_coding | X:150547375-150570638 (+) |  | 2.230 | 7.74e-07 | 2.27e-05 |
| ENSMUSG00000050064 | Zfp697 | protein\_coding | 3:98382461-98753812 (+) |  | 1.650 | 8.02e-07 | 2.35e-05 |
| ENSMUSG00000061458 | Nol10 | protein\_coding | 12:17348458-17430095 (+) |  | -0.434 | 8.30e-07 | 2.43e-05 |
| ENSMUSG00000037321 | Tap1 | protein\_coding | 17:34187553-34197225 (+) |  | -0.318 | 8.33e-07 | 2.43e-05 |
| ENSMUSG00000017417 | Plxdc1 | protein\_coding | 11:97923238-97986444 (-) |  | 0.662 | 8.36e-07 | 2.43e-05 |
| ENSMUSG00000027540 | Ptpn1 | protein\_coding | 2:167932057-167979385 (+) |  | -0.415 | 8.47e-07 | 2.46e-05 |
| ENSMUSG00000014226 | Cacybp | protein\_coding | 1:160202367-160212875 (-) |  | -0.578 | 8.59e-07 | 2.49e-05 |
| ENSMUSG00000003868 | Ruvbl2 | protein\_coding | 7:45421760-45438096 (-) |  | -0.459 | 8.87e-07 | 2.57e-05 |
| ENSMUSG00000026458 | Ppfia4 | protein\_coding | 1:134296783-134332928 (-) |  | 0.531 | 9.08e-07 | 2.62e-05 |
| ENSMUSG00000060550 | H2-Q7 | protein\_coding | 17:35439155-35443773 (+) |  | -0.834 | 9.24e-07 | 2.66e-05 |
| ENSMUSG00000045983 | Eif4g1 | protein\_coding | 16:20668313-20692884 (+) |  | -0.356 | 9.27e-07 | 2.66e-05 |
| ENSMUSG00000017760 | Ctsa | protein\_coding | 2:164832873-164841032 (+) |  | -0.295 | 9.47e-07 | 2.72e-05 |
| ENSMUSG00000015766 | Eps8 | protein\_coding | 6:137477245-137654876 (-) |  | -0.668 | 9.51e-07 | 2.72e-05 |
| ENSMUSG00000036138 | Acaa1a | protein\_coding | 9:119339676-119350299 (+) |  | -0.357 | 9.55e-07 | 2.73e-05 |
| ENSMUSG00000040197 | Cd209e | protein\_coding | 8:3847965-3854309 (-) |  | 3.230 | 9.61e-07 | 2.74e-05 |
| ENSMUSG00000030108 | Slc6a13 | protein\_coding | 6:121300227-121337733 (+) |  | 1.150 | 9.67e-07 | 2.75e-05 |
| ENSMUSG00000025155 | Dus1l | protein\_coding | 11:120789201-120796403 (-) |  | -0.309 | 9.85e-07 | 2.79e-05 |
| ENSMUSG00000024785 | Rcl1 | protein\_coding | 19:29101375-29143929 (+) |  | -0.433 | 9.90e-07 | 2.80e-05 |
| ENSMUSG00000021149 | Gtpbp4 | protein\_coding | 13:8966331-8996083 (-) |  | -0.351 | 9.92e-07 | 2.80e-05 |
| ENSMUSG00000063354 | Slc39a4 | protein\_coding | 15:76612383-76617384 (-) |  | 2.290 | 1.00e-06 | 2.82e-05 |
| ENSMUSG00000032554 | Trf | protein\_coding | 9:103204001-103230444 (-) |  | 0.555 | 1.00e-06 | 2.82e-05 |
| ENSMUSG00000073676 | Hspe1 | protein\_coding | 1:55088132-55091307 (+) |  | -0.572 | 1.01e-06 | 2.83e-05 |
| ENSMUSG00000024587 | Nars | protein\_coding | 18:64499647-64516652 (-) |  | -0.448 | 1.02e-06 | 2.87e-05 |
| ENSMUSG00000024991 | Eif3a | protein\_coding | 19:60761117-60790658 (-) |  | -0.287 | 1.04e-06 | 2.89e-05 |
| ENSMUSG00000001416 | Cct3 | protein\_coding | 3:88297116-88321767 (+) |  | -0.460 | 1.05e-06 | 2.93e-05 |
| ENSMUSG00000027248 | Pdia3 | protein\_coding | 2:121413775-121438687 (+) |  | -0.400 | 1.06e-06 | 2.95e-05 |
| ENSMUSG00000017756 | Slc12a7 | protein\_coding | 13:73733094-73816754 (+) |  | -0.719 | 1.09e-06 | 3.03e-05 |
| ENSMUSG00000020653 | Klf11 | protein\_coding | 12:24651274-24662789 (+) |  | 0.589 | 1.09e-06 | 3.03e-05 |
| ENSMUSG00000079499 | 6530402F18Rik | lncRNA | 2:29245107-29253006 (-) |  | -0.399 | 1.11e-06 | 3.06e-05 |
| ENSMUSG00000027712 | Anxa5 | protein\_coding | 3:36448923-36475894 (-) |  | 0.446 | 1.13e-06 | 3.11e-05 |
| ENSMUSG00000035901 | Dennd5a | protein\_coding | 7:109893780-109960470 (-) |  | 0.308 | 1.15e-06 | 3.17e-05 |
| ENSMUSG00000035299 | Mid1 | protein\_coding | X:169685199-170005736 (+) |  | -0.851 | 1.18e-06 | 3.23e-05 |
| ENSMUSG00000039756 | Dnttip2 | protein\_coding | 3:122274388-122285271 (+) |  | -0.393 | 1.18e-06 | 3.23e-05 |
| ENSMUSG00000039640 | Mrpl12 | protein\_coding | 11:120484613-120489065 (+) |  | -0.539 | 1.19e-06 | 3.25e-05 |
| ENSMUSG00000026594 | Ralgps2 | protein\_coding | 1:156804166-156939626 (-) |  | 0.632 | 1.19e-06 | 3.25e-05 |
| ENSMUSG00000031765 | Mt1 | protein\_coding | 8:94179082-94180327 (+) |  | -0.670 | 1.20e-06 | 3.25e-05 |
| ENSMUSG00000038872 | Zfhx3 | protein\_coding | 8:107942644-108961630 (+) |  | 0.963 | 1.20e-06 | 3.25e-05 |
| ENSMUSG00000020048 | Hsp90b1 | protein\_coding | 10:86690209-86705509 (-) |  | -0.449 | 1.21e-06 | 3.27e-05 |
| ENSMUSG00000035673 | Sbno2 | protein\_coding | 10:80056992-80105571 (-) |  | -0.383 | 1.21e-06 | 3.28e-05 |
| ENSMUSG00000034795 | Ccdc122 | protein\_coding | 14:77036772-77112257 (+) |  | 0.879 | 1.23e-06 | 3.32e-05 |
| ENSMUSG00000029551 | Psmg3 | protein\_coding | 5:139823592-139826885 (-) |  | -0.549 | 1.26e-06 | 3.39e-05 |
| ENSMUSG00000034203 | Chchd4 | protein\_coding | 6:91462172-91473546 (-) |  | -0.502 | 1.26e-06 | 3.40e-05 |
| ENSMUSG00000046456 | Tmem150b | protein\_coding | 7:4706832-4725249 (-) |  | 0.435 | 1.27e-06 | 3.41e-05 |
| ENSMUSG00000027775 | Mfsd1 | protein\_coding | 3:67582741-67604237 (+) |  | -0.278 | 1.29e-06 | 3.46e-05 |
| ENSMUSG00000032691 | Nlrp3 | protein\_coding | 11:59541568-59566956 (+) |  | 0.692 | 1.30e-06 | 3.47e-05 |
| ENSMUSG00000041695 | Kcnj2 | protein\_coding | 11:111066164-111076821 (+) |  | -0.661 | 1.34e-06 | 3.56e-05 |
| ENSMUSG00000040016 | Ptger3 | protein\_coding | 3:157566892-157645888 (+) |  | -1.820 | 1.36e-06 | 3.62e-05 |
| ENSMUSG00000018819 | Lsp1 | protein\_coding | 7:142460809-142494867 (+) |  | 0.566 | 1.37e-06 | 3.63e-05 |
| ENSMUSG00000036908 | Unc93b1 | protein\_coding | 19:3935186-3949340 (+) |  | 0.357 | 1.39e-06 | 3.68e-05 |
| ENSMUSG00000039067 | Psmd7 | protein\_coding | 8:107580381-107588464 (-) |  | -0.382 | 1.43e-06 | 3.78e-05 |
| ENSMUSG00000060477 | Irak2 | protein\_coding | 6:113638467-113695026 (+) |  | 0.606 | 1.44e-06 | 3.80e-05 |
| ENSMUSG00000004952 | Rasa4 | protein\_coding | 5:136083916-136111860 (+) |  | 0.329 | 1.45e-06 | 3.82e-05 |
| ENSMUSG00000026833 | Olfm1 | protein\_coding | 2:28192992-28230736 (+) |  | -0.613 | 1.48e-06 | 3.88e-05 |
| ENSMUSG00000070730 | Rmdn3 | protein\_coding | 2:119137001-119157034 (-) |  | -0.386 | 1.48e-06 | 3.88e-05 |
| ENSMUSG00000032279 | Idh3a | protein\_coding | 9:54586334-54604661 (+) |  | -0.425 | 1.50e-06 | 3.93e-05 |
| ENSMUSG00000032740 | Ccdc88a | protein\_coding | 11:29373658-29510808 (+) |  | 0.455 | 1.51e-06 | 3.96e-05 |
| ENSMUSG00000032254 | Kif23 | protein\_coding | 9:61915905-61946774 (-) |  | 0.312 | 1.56e-06 | 4.07e-05 |
| ENSMUSG00000041515 | Irf8 | protein\_coding | 8:120736358-120756694 (+) |  | 0.472 | 1.57e-06 | 4.08e-05 |
| ENSMUSG00000030082 | Sec61a1 | protein\_coding | 6:88503579-88518905 (-) |  | -0.272 | 1.59e-06 | 4.13e-05 |
| ENSMUSG00000039646 | Vasn | protein\_coding | 16:4639941-4650802 (+) |  | 0.783 | 1.60e-06 | 4.13e-05 |
| ENSMUSG00000053175 | Bcl3 | protein\_coding | 7:19808462-19822770 (-) |  | -0.617 | 1.60e-06 | 4.13e-05 |
| ENSMUSG00000042851 | Zc3h6 | protein\_coding | 2:128967402-129018563 (+) |  | 1.090 | 1.61e-06 | 4.16e-05 |
| ENSMUSG00000040028 | Elavl1 | protein\_coding | 8:4285382-4325413 (-) |  | -0.351 | 1.61e-06 | 4.16e-05 |
| ENSMUSG00000024359 | Hspa9 | protein\_coding | 18:34937414-34954357 (-) |  | -0.491 | 1.62e-06 | 4.17e-05 |
| ENSMUSG00000095567 | Noc2l | protein\_coding | 4:156235919-156247616 (+) |  | -0.391 | 1.67e-06 | 4.29e-05 |
| ENSMUSG00000040010 | Slc7a5 | protein\_coding | 8:121881150-121907694 (-) |  | -0.555 | 1.70e-06 | 4.35e-05 |
| ENSMUSG00000071068 | Treml2 | protein\_coding | 17:48299498-48312533 (+) |  | -0.307 | 1.79e-06 | 4.58e-05 |
| ENSMUSG00000101389 | Ms4a4a | protein\_coding | 19:11375523-11392790 (+) |  | -1.390 | 1.81e-06 | 4.61e-05 |
| ENSMUSG00000029086 | Prom1 | protein\_coding | 5:43993620-44102032 (-) |  | -1.190 | 1.82e-06 | 4.65e-05 |
| ENSMUSG00000021338 | Carmil1 | protein\_coding | 13:24012344-24280795 (-) |  | 0.762 | 1.84e-06 | 4.67e-05 |
| ENSMUSG00000028617 | Lrrc42 | protein\_coding | 4:107233514-107253532 (-) |  | -0.651 | 1.84e-06 | 4.67e-05 |
| ENSMUSG00000027422 | Rrbp1 | protein\_coding | 2:143947395-144011263 (-) |  | -0.250 | 1.86e-06 | 4.70e-05 |
| ENSMUSG00000052949 | Rnf157 | protein\_coding | 11:116336353-116413032 (-) |  | 0.447 | 1.86e-06 | 4.70e-05 |
| ENSMUSG00000034413 | Neurl1b | protein\_coding | 17:26414829-26446349 (+) |  | 0.647 | 1.87e-06 | 4.73e-05 |
| ENSMUSG00000057948 | Unc13d | protein\_coding | 11:116062095-116077961 (-) |  | 0.363 | 1.89e-06 | 4.77e-05 |
| ENSMUSG00000052212 | Cd177 | protein\_coding | 7:24743983-24760311 (-) |  | 1.430 | 1.90e-06 | 4.77e-05 |
| ENSMUSG00000024299 | Adamts10 | protein\_coding | 17:33524204-33553782 (+) |  | 0.539 | 1.92e-06 | 4.83e-05 |
| ENSMUSG00000033287 | Kctd17 | protein\_coding | 15:78428564-78439303 (+) |  | -0.574 | 1.93e-06 | 4.83e-05 |
| ENSMUSG00000032382 | Snx1 | protein\_coding | 9:66088133-66126587 (-) |  | -0.383 | 1.94e-06 | 4.83e-05 |
| ENSMUSG00000024665 | Fads2 | protein\_coding | 19:10062765-10101746 (-) |  | -0.928 | 2.00e-06 | 4.99e-05 |
| ENSMUSG00000030223 | Ptpro | protein\_coding | 6:137252319-137463233 (+) |  | 0.564 | 2.01e-06 | 5.00e-05 |
| ENSMUSG00000025869 | Nop16 | protein\_coding | 13:54584185-54590090 (-) |  | -0.451 | 2.02e-06 | 5.01e-05 |
| ENSMUSG00000079056 | Kcnip3 | protein\_coding | 2:127456498-127522094 (-) |  | 0.925 | 2.02e-06 | 5.01e-05 |
| ENSMUSG00000024120 | Lrpprc | protein\_coding | 17:84705247-84790789 (-) |  | -0.356 | 2.03e-06 | 5.02e-05 |
| ENSMUSG00000030007 | Cct7 | protein\_coding | 6:85451514-85468475 (+) |  | -0.383 | 2.04e-06 | 5.03e-05 |
| ENSMUSG00000109556 | Gm38843 | lncRNA | 6:82803769-82805083 (-) |  | 1.900 | 2.07e-06 | 5.11e-05 |
| ENSMUSG00000058392 | Rrp1b | protein\_coding | 17:32036100-32062865 (+) |  | -0.403 | 2.08e-06 | 5.11e-05 |
| ENSMUSG00000020787 | P2rx1 | protein\_coding | 11:72999103-73015200 (+) |  | 0.887 | 2.08e-06 | 5.11e-05 |
| ENSMUSG00000020149 | Rab1a | protein\_coding | 11:20201432-20226856 (+) |  | -0.296 | 2.11e-06 | 5.17e-05 |
| ENSMUSG00000034187 | Nsf | protein\_coding | 11:103821782-103954056 (-) |  | -0.359 | 2.12e-06 | 5.18e-05 |
| ENSMUSG00000036752 | Tubb4b | protein\_coding | 2:25222160-25224702 (-) |  | -0.355 | 2.18e-06 | 5.31e-05 |
| ENSMUSG00000043939 | A530064D06Rik | protein\_coding | 17:48149126-48167275 (-) |  | 1.100 | 2.24e-06 | 5.45e-05 |
| ENSMUSG00000062906 | Hdac10 | protein\_coding | 15:89123307-89128700 (-) |  | 0.675 | 2.24e-06 | 5.46e-05 |
| ENSMUSG00000024855 | Pacs1 | protein\_coding | 19:5133158-5273119 (-) |  | 0.467 | 2.27e-06 | 5.51e-05 |
| ENSMUSG00000036587 | Fut7 | protein\_coding | 2:25423267-25426374 (+) |  | 0.584 | 2.28e-06 | 5.54e-05 |
| ENSMUSG00000031948 | Kars | protein\_coding | 8:111993443-112011323 (-) |  | -0.330 | 2.29e-06 | 5.54e-05 |
| ENSMUSG00000017999 | Ddx27 | protein\_coding | 2:167015193-167034947 (+) |  | -0.332 | 2.30e-06 | 5.54e-05 |
| ENSMUSG00000031875 | Cmtm3 | protein\_coding | 8:104339410-104347672 (+) |  | 0.374 | 2.32e-06 | 5.59e-05 |
| ENSMUSG00000079641 | Rpl39 | protein\_coding | X:37082520-37085402 (-) |  | 0.440 | 2.41e-06 | 5.79e-05 |
| ENSMUSG00000028134 | Ptbp2 | protein\_coding | 3:119718742-119784466 (-) |  | 0.632 | 2.42e-06 | 5.81e-05 |
| ENSMUSG00000030869 | Ndufab1 | protein\_coding | 7:122085403-122101886 (-) |  | -0.412 | 2.43e-06 | 5.82e-05 |
| ENSMUSG00000053411 | Cbx7 | protein\_coding | 15:79915807-79971119 (-) |  | 0.815 | 2.44e-06 | 5.84e-05 |
| ENSMUSG00000021178 | Psmc1 | protein\_coding | 12:100110154-100123405 (+) |  | -0.395 | 2.48e-06 | 5.91e-05 |
| ENSMUSG00000031960 | Aars | protein\_coding | 8:111033144-111057664 (+) |  | -0.351 | 2.48e-06 | 5.91e-05 |
| ENSMUSG00000029388 | Eif2b1 | protein\_coding | 5:124570213-124579131 (-) |  | -0.477 | 2.48e-06 | 5.91e-05 |
| ENSMUSG00000031586 | Rbpms | protein\_coding | 8:33782643-33929863 (-) |  | 1.170 | 2.51e-06 | 5.96e-05 |
| ENSMUSG00000015053 | Gata2 | protein\_coding | 6:88193891-88207032 (+) |  | 1.600 | 2.53e-06 | 6.01e-05 |
| ENSMUSG00000028577 | Plaa | protein\_coding | 4:94567514-94603244 (-) |  | -0.305 | 2.56e-06 | 6.04e-05 |
| ENSMUSG00000026083 | Eif5b | protein\_coding | 1:37998010-38055579 (+) |  | -0.321 | 2.56e-06 | 6.04e-05 |
| ENSMUSG00000101188 | Eif4a-ps4 | processed\_pseudogene | 1:60703932-60705149 (+) |  | -0.394 | 2.56e-06 | 6.04e-05 |
| ENSMUSG00000003541 | Ier3 | protein\_coding | 17:35821684-35822923 (+) |  | -1.010 | 2.58e-06 | 6.06e-05 |
| ENSMUSG00000001281 | Itgb7 | protein\_coding | 15:102215995-102231944 (-) |  | 0.414 | 2.58e-06 | 6.07e-05 |
| ENSMUSG00000004460 | Dnajb11 | protein\_coding | 16:22857845-22879634 (+) |  | -0.360 | 2.60e-06 | 6.11e-05 |
| ENSMUSG00000041360 | Pum3 | protein\_coding | 19:27388698-27429825 (-) |  | -0.355 | 2.63e-06 | 6.16e-05 |
| ENSMUSG00000068039 | Tcp1 | protein\_coding | 17:12915701-12925067 (+) |  | -0.328 | 2.74e-06 | 6.41e-05 |
| ENSMUSG00000029777 | Gars | protein\_coding | 6:55038007-55079500 (+) |  | -0.374 | 2.76e-06 | 6.42e-05 |
| ENSMUSG00000034485 | Uaca | protein\_coding | 9:60794542-60880370 (+) |  | -1.570 | 2.76e-06 | 6.42e-05 |
| ENSMUSG00000021087 | Rtn1 | protein\_coding | 12:72211752-72409054 (-) |  | 1.190 | 2.77e-06 | 6.44e-05 |
| ENSMUSG00000055013 | Agap1 | protein\_coding | 1:89454806-89897617 (+) |  | 0.632 | 2.80e-06 | 6.50e-05 |
| ENSMUSG00000028159 | Dapp1 | protein\_coding | 3:137931007-137981545 (-) |  | 0.332 | 2.88e-06 | 6.67e-05 |
| ENSMUSG00000007038 | Neu1 | protein\_coding | 17:34931253-34935953 (+) |  | -0.376 | 2.90e-06 | 6.71e-05 |
| ENSMUSG00000060044 | Tmem26 | protein\_coding | 10:68723646-68782650 (+) |  | 1.140 | 2.94e-06 | 6.78e-05 |
| ENSMUSG00000021486 | Prelid1 | protein\_coding | 13:55320500-55325272 (+) |  | -0.366 | 3.01e-06 | 6.94e-05 |
| ENSMUSG00000042628 | Zfyve1 | protein\_coding | 12:83546558-83597222 (-) |  | 0.479 | 3.02e-06 | 6.95e-05 |
| ENSMUSG00000029101 | Rgs12 | protein\_coding | 5:34949445-35039644 (+) |  | 0.566 | 3.03e-06 | 6.96e-05 |
| ENSMUSG00000000168 | Dlat | protein\_coding | 9:50634633-50659780 (-) |  | -0.418 | 3.05e-06 | 6.99e-05 |
| ENSMUSG00000078616 | Trim30c | protein\_coding | 7:104382065-104400837 (-) |  | -1.440 | 3.06e-06 | 6.99e-05 |
| ENSMUSG00000092203 | 1110038B12Rik | lncRNA | 17:34950238-34952471 (-) |  | -0.492 | 3.12e-06 | 7.12e-05 |
| ENSMUSG00000029484 | Anxa3 | protein\_coding | 5:96793339-96845966 (+) |  | 0.311 | 3.13e-06 | 7.13e-05 |
| ENSMUSG00000004668 | Abca13 | protein\_coding | 11:9191942-9684259 (+) |  | -0.596 | 3.21e-06 | 7.30e-05 |
| ENSMUSG00000011256 | Adam19 | protein\_coding | 11:46055992-46147343 (+) |  | 1.860 | 3.27e-06 | 7.41e-05 |
| ENSMUSG00000024732 | Ccdc86 | protein\_coding | 19:10941481-10949266 (-) |  | -0.529 | 3.27e-06 | 7.41e-05 |
| ENSMUSG00000025194 | Abcc2 | protein\_coding | 19:43782192-43840740 (+) |  | 0.843 | 3.28e-06 | 7.41e-05 |
| ENSMUSG00000029066 | Mrpl20 | protein\_coding | 4:155802878-155809975 (+) |  | -0.496 | 3.29e-06 | 7.43e-05 |
| ENSMUSG00000016528 | Mapkapk2 | protein\_coding | 1:131053700-131097826 (-) |  | -0.227 | 3.32e-06 | 7.48e-05 |
| ENSMUSG00000037405 | Icam1 | protein\_coding | 9:21015985-21028817 (+) |  | -0.409 | 3.35e-06 | 7.53e-05 |
| ENSMUSG00000071723 | Gspt2 | protein\_coding | X:94636069-94643244 (+) |  | 0.477 | 3.38e-06 | 7.60e-05 |
| ENSMUSG00000083899 | Gm12346 | transcribed\_processed\_pseudogene | 11:77293444-77295898 (+) |  | -0.629 | 3.43e-06 | 7.69e-05 |
| ENSMUSG00000021807 | Rtraf | protein\_coding | 14:19811351-19823824 (-) |  | -0.363 | 3.45e-06 | 7.73e-05 |
| ENSMUSG00000025613 | Cct8 | protein\_coding | 16:87483326-87495873 (-) |  | -0.414 | 3.49e-06 | 7.80e-05 |
| ENSMUSG00000071072 | Ptges3 | protein\_coding | 10:128058954-128077272 (+) |  | -0.510 | 3.50e-06 | 7.81e-05 |
| ENSMUSG00000054079 | Utp18 | protein\_coding | 11:93859243-93885766 (-) |  | -0.390 | 3.53e-06 | 7.87e-05 |
| ENSMUSG00000037851 | Iars | protein\_coding | 13:49682100-49734267 (+) |  | -0.380 | 3.54e-06 | 7.87e-05 |
| ENSMUSG00000097636 | Mirt1 | lncRNA | 19:53443230-53464796 (-) |  | 0.868 | 3.61e-06 | 8.02e-05 |
| ENSMUSG00000035891 | Cerk | protein\_coding | 15:86139128-86186141 (-) |  | 0.398 | 3.64e-06 | 8.07e-05 |
| ENSMUSG00000031467 | Agpat5 | protein\_coding | 8:18846277-18891361 (+) |  | -0.354 | 3.65e-06 | 8.08e-05 |
| ENSMUSG00000045730 | Adrb2 | protein\_coding | 18:62177816-62179959 (-) |  | 0.994 | 3.70e-06 | 8.17e-05 |
| ENSMUSG00000027774 | Gfm1 | protein\_coding | 3:67430096-67476529 (+) |  | -0.398 | 3.74e-06 | 8.24e-05 |
| ENSMUSG00000034858 | Fam214a | protein\_coding | 9:74952884-75032468 (+) |  | 0.997 | 3.76e-06 | 8.27e-05 |
| ENSMUSG00000031712 | Il15 | protein\_coding | 8:82331632-82403222 (-) |  | 0.939 | 3.83e-06 | 8.41e-05 |
| ENSMUSG00000001763 | Tspan33 | protein\_coding | 6:29694222-29718559 (+) |  | 0.843 | 3.85e-06 | 8.44e-05 |
| ENSMUSG00000004655 | Aqp1 | protein\_coding | 6:55336432-55348555 (+) |  | 1.900 | 3.88e-06 | 8.46e-05 |
| ENSMUSG00000011752 | Pgam1 | protein\_coding | 19:41911923-41918660 (+) |  | -0.360 | 3.88e-06 | 8.46e-05 |
| ENSMUSG00000030468 | Siglecg | protein\_coding | 7:43408204-43418358 (+) |  | 0.627 | 3.88e-06 | 8.46e-05 |
| ENSMUSG00000002668 | Dennd1c | protein\_coding | 17:57065905-57078514 (-) |  | 0.791 | 3.88e-06 | 8.46e-05 |
| ENSMUSG00000042046 | Dstyk | protein\_coding | 1:132417555-132466958 (+) |  | 0.372 | 3.89e-06 | 8.46e-05 |
| ENSMUSG00000070056 | Mfhas1 | protein\_coding | 8:35587798-35679449 (+) |  | 0.674 | 3.90e-06 | 8.47e-05 |
| ENSMUSG00000029468 | P2rx7 | protein\_coding | 5:122643911-122691432 (+) |  | 0.418 | 3.92e-06 | 8.48e-05 |
| ENSMUSG00000028927 | Padi2 | protein\_coding | 4:140906344-140952586 (+) |  | 1.140 | 3.92e-06 | 8.48e-05 |
| ENSMUSG00000074203 | G430095P16Rik | protein\_coding | 8:84723007-84726844 (+) |  | 0.872 | 3.93e-06 | 8.48e-05 |
| ENSMUSG00000035711 | Dok3 | protein\_coding | 13:55523231-55529296 (-) |  | 0.394 | 3.95e-06 | 8.53e-05 |
| ENSMUSG00000028385 | Snx30 | protein\_coding | 4:59805840-59904737 (+) |  | 0.327 | 4.01e-06 | 8.63e-05 |
| ENSMUSG00000025364 | Pa2g4 | protein\_coding | 10:128557766-128565987 (-) |  | -0.450 | 4.02e-06 | 8.63e-05 |
| ENSMUSG00000061613 | U2af1 | protein\_coding | 17:31647081-31658892 (-) |  | -0.327 | 4.02e-06 | 8.63e-05 |
| ENSMUSG00000003348 | Mob3a | protein\_coding | 10:80685253-80701977 (-) |  | 0.323 | 4.05e-06 | 8.68e-05 |
| ENSMUSG00000040532 | Abhd11 | protein\_coding | 5:135009152-135012175 (+) |  | -0.506 | 4.15e-06 | 8.88e-05 |
| ENSMUSG00000066232 | Ipo7 | protein\_coding | 7:110018274-110056609 (+) |  | -0.304 | 4.16e-06 | 8.90e-05 |
| ENSMUSG00000030172 | Erc1 | protein\_coding | 6:119570796-119848167 (-) |  | -0.997 | 4.24e-06 | 9.05e-05 |
| ENSMUSG00000032002 | Dcun1d5 | protein\_coding | 9:7184520-7208205 (+) |  | -0.478 | 4.26e-06 | 9.06e-05 |
| ENSMUSG00000024397 | Aif1 | protein\_coding | 17:35170991-35176068 (-) |  | 0.604 | 4.30e-06 | 9.15e-05 |
| ENSMUSG00000030493 | Faap24 | protein\_coding | 7:35392152-35396836 (-) |  | -0.478 | 4.33e-06 | 9.19e-05 |
| ENSMUSG00000064360 | mt-Nd3 | protein\_coding | MT:9459-9806 (+) |  | -0.812 | 4.34e-06 | 9.19e-05 |
| ENSMUSG00000003235 | Eif2b5 | protein\_coding | 16:20498817-20509323 (+) |  | -0.367 | 4.38e-06 | 9.26e-05 |
| ENSMUSG00000024037 | Wdr4 | protein\_coding | 17:31494322-31519980 (-) |  | -0.439 | 4.38e-06 | 9.26e-05 |
| ENSMUSG00000018042 | Cyb5r3 | protein\_coding | 15:83153494-83172592 (-) |  | 0.401 | 4.50e-06 | 9.48e-05 |
| ENSMUSG00000027423 | Snx5 | protein\_coding | 2:144250123-144270906 (-) |  | -0.374 | 4.50e-06 | 9.48e-05 |
| ENSMUSG00000037905 | Bri3bp | protein\_coding | 5:125441568-125460370 (+) |  | -0.286 | 4.54e-06 | 9.55e-05 |
| ENSMUSG00000070003 | Ssbp4 | protein\_coding | 8:70597490-70608872 (-) |  | -0.337 | 4.57e-06 | 9.59e-05 |
| ENSMUSG00000002017 | Fam98a | protein\_coding | 17:75537086-75551946 (-) |  | -0.375 | 4.59e-06 | 9.62e-05 |
| ENSMUSG00000032802 | Srxn1 | protein\_coding | 2:152105516-152111376 (+) |  | -0.918 | 4.60e-06 | 9.63e-05 |
| ENSMUSG00000078713 | Tomm5 | protein\_coding | 4:45105208-45108114 (-) |  | -0.508 | 4.62e-06 | 9.64e-05 |
| ENSMUSG00000016206 | H2-M3 | protein\_coding | 17:37270220-37274484 (+) |  | -0.443 | 4.69e-06 | 9.77e-05 |
| ENSMUSG00000022489 | Pde1b | protein\_coding | 15:103503034-103530052 (+) |  | 0.645 | 4.69e-06 | 9.77e-05 |
| ENSMUSG00000046808 | Atp10d | polymorphic\_pseudogene | 5:72203329-72298775 (+) |  | 0.502 | 4.71e-06 | 9.80e-05 |
| ENSMUSG00000033845 | Mrpl15 | protein\_coding | 1:4773206-4785739 (-) |  | -0.440 | 4.80e-06 | 9.96e-05 |
| ENSMUSG00000000244 | Tspan32 | protein\_coding | 7:143005046-143019644 (+) |  | 0.435 | 4.92e-06 | 1.02e-04 |
| ENSMUSG00000025153 | Fasn | protein\_coding | 11:120805846-120824547 (-) |  | -0.394 | 5.05e-06 | 1.04e-04 |
| ENSMUSG00000042590 | Ipo11 | protein\_coding | 13:106794439-106936958 (-) |  | -0.416 | 5.06e-06 | 1.04e-04 |
| ENSMUSG00000078429 | Ctdsp2 | protein\_coding | 10:126978717-126999975 (+) |  | 0.379 | 5.13e-06 | 1.06e-04 |
| ENSMUSG00000028633 | Ctps | protein\_coding | 4:120539868-120570276 (-) |  | -0.378 | 5.14e-06 | 1.06e-04 |
| ENSMUSG00000020936 | Nmt1 | protein\_coding | 11:103028190-103068912 (+) |  | -0.293 | 5.15e-06 | 1.06e-04 |
| ENSMUSG00000059796 | Eif4a1 | protein\_coding | 11:69666936-69672423 (-) |  | -0.347 | 5.17e-06 | 1.06e-04 |
| ENSMUSG00000040026 | Saa3 | protein\_coding | 7:46711998-46715700 (-) |  | -2.340 | 5.18e-06 | 1.06e-04 |
| ENSMUSG00000067071 | Hes6 | protein\_coding | 1:91411483-91414038 (-) |  | 0.401 | 5.18e-06 | 1.06e-04 |
| ENSMUSG00000100801 | Gm15459 | processed\_pseudogene | 5:5781615-5783555 (-) |  | -0.467 | 5.22e-06 | 1.06e-04 |
| ENSMUSG00000030045 | Mrpl19 | protein\_coding | 6:81957851-81965958 (-) |  | -0.360 | 5.23e-06 | 1.06e-04 |
| ENSMUSG00000028953 | Abcf2 | protein\_coding | 5:24565345-24577467 (-) |  | -0.301 | 5.23e-06 | 1.06e-04 |
| ENSMUSG00000116504 | I730030J21Rik | lncRNA | 15:100730481-100732737 (-) |  | 1.310 | 5.24e-06 | 1.06e-04 |
| ENSMUSG00000039013 | Siglecf | protein\_coding | 7:43351341-43359531 (+) |  | 0.823 | 5.25e-06 | 1.06e-04 |
| ENSMUSG00000043157 | Arl11 | protein\_coding | 14:61309753-61311936 (+) |  | 0.448 | 5.33e-06 | 1.08e-04 |
| ENSMUSG00000048234 | Rnf149 | protein\_coding | 1:39551296-39577405 (-) |  | -0.357 | 5.39e-06 | 1.09e-04 |
| ENSMUSG00000053289 | Ddx10 | protein\_coding | 9:53098635-53248053 (-) |  | -0.320 | 5.49e-06 | 1.11e-04 |
| ENSMUSG00000038332 | Sesn1 | protein\_coding | 10:41809935-41908424 (+) |  | 0.433 | 5.55e-06 | 1.12e-04 |
| ENSMUSG00000026005 | Rpe | protein\_coding | 1:66700831-66719805 (+) |  | -0.401 | 5.55e-06 | 1.12e-04 |
| ENSMUSG00000040688 | Tbl3 | protein\_coding | 17:24697949-24707660 (-) |  | -0.407 | 5.60e-06 | 1.13e-04 |
| ENSMUSG00000025980 | Hspd1 | protein\_coding | 1:55077835-55088243 (-) |  | -0.808 | 5.63e-06 | 1.13e-04 |
| ENSMUSG00000062761 | Zfp512 | protein\_coding | 5:31452431-31481754 (+) |  | 0.404 | 5.72e-06 | 1.15e-04 |
| ENSMUSG00000016024 | Lbp | protein\_coding | 2:158306493-158332852 (+) |  | -0.377 | 5.78e-06 | 1.16e-04 |
| ENSMUSG00000038679 | Trps1 | protein\_coding | 15:50654752-50890463 (-) |  | -0.439 | 5.80e-06 | 1.16e-04 |
| ENSMUSG00000062515 | Fabp4 | protein\_coding | 3:10204088-10208576 (-) |  | 3.670 | 5.86e-06 | 1.17e-04 |
| ENSMUSG00000026489 | Coq8a | protein\_coding | 1:180165238-180199602 (-) |  | 0.874 | 5.89e-06 | 1.17e-04 |
| ENSMUSG00000079084 | Ccdc82 | protein\_coding | 9:13246955-13292520 (+) |  | 0.353 | 5.90e-06 | 1.17e-04 |
| ENSMUSG00000025969 | Nrp2 | protein\_coding | 1:62703285-62818695 (+) |  | -0.516 | 5.99e-06 | 1.19e-04 |
| ENSMUSG00000038507 | Parp12 | protein\_coding | 6:39086410-39118349 (-) |  | -0.848 | 6.01e-06 | 1.19e-04 |
| ENSMUSG00000030109 | Slc6a12 | protein\_coding | 6:121343076-121365775 (+) |  | 3.760 | 6.02e-06 | 1.19e-04 |
| ENSMUSG00000036737 | Oxsr1 | protein\_coding | 9:119238432-119322427 (-) |  | -0.332 | 6.06e-06 | 1.20e-04 |
| ENSMUSG00000038335 | Tsr1 | protein\_coding | 11:74898071-74909342 (+) |  | -0.374 | 6.06e-06 | 1.20e-04 |
| ENSMUSG00000038736 | Nudcd1 | protein\_coding | 15:44373163-44428307 (-) |  | -0.491 | 6.11e-06 | 1.20e-04 |
| ENSMUSG00000040928 | S100pbp | protein\_coding | 4:129148005-129189727 (-) |  | 0.420 | 6.15e-06 | 1.21e-04 |
| ENSMUSG00000037108 | Zcwpw1 | protein\_coding | 5:137787798-137822621 (+) |  | 0.848 | 6.16e-06 | 1.21e-04 |
| ENSMUSG00000023988 | Bysl | protein\_coding | 17:47599331-47611492 (-) |  | -0.415 | 6.16e-06 | 1.21e-04 |
| ENSMUSG00000026170 | Cyp27a1 | protein\_coding | 1:74713574-74737892 (+) |  | 0.738 | 6.17e-06 | 1.21e-04 |
| ENSMUSG00000028221 | Pip4p2 | protein\_coding | 4:14864076-14915176 (+) |  | -0.438 | 6.23e-06 | 1.22e-04 |
| ENSMUSG00000052776 | Oas1a | protein\_coding | 5:120896256-120907521 (-) |  | -0.938 | 6.31e-06 | 1.23e-04 |
| ENSMUSG00000022682 | Rrn3 | protein\_coding | 16:13780708-13814839 (+) |  | -0.343 | 6.38e-06 | 1.25e-04 |
| ENSMUSG00000032763 | Ilvbl | protein\_coding | 10:78574346-78584502 (+) |  | 0.435 | 6.40e-06 | 1.25e-04 |
| ENSMUSG00000024772 | Ehd1 | protein\_coding | 19:6276725-6300096 (+) |  | 0.707 | 6.42e-06 | 1.25e-04 |
| ENSMUSG00000027447 | Cst3 | protein\_coding | 2:148871722-148875692 (-) |  | 0.377 | 6.44e-06 | 1.25e-04 |
| ENSMUSG00000005481 | Ddx39 | protein\_coding | 8:83715177-83726892 (+) |  | -0.391 | 6.51e-06 | 1.26e-04 |
| ENSMUSG00000063884 | Ptcd3 | protein\_coding | 6:71880638-71908750 (-) |  | -0.409 | 6.52e-06 | 1.26e-04 |
| ENSMUSG00000033538 | Casp4 | protein\_coding | 9:5308828-5336783 (+) |  | -0.674 | 6.57e-06 | 1.27e-04 |
| ENSMUSG00000022913 | Psmg1 | protein\_coding | 16:95979933-95990960 (-) |  | -0.465 | 6.68e-06 | 1.29e-04 |
| ENSMUSG00000040354 | Mars | protein\_coding | 10:127296221-127311786 (-) |  | -0.389 | 6.81e-06 | 1.31e-04 |
| ENSMUSG00000046006 | Gapt | protein\_coding | 13:110352616-110357199 (-) |  | 0.479 | 6.82e-06 | 1.31e-04 |
| ENSMUSG00000029246 | Ppat | protein\_coding | 5:76913249-76951578 (-) |  | -0.350 | 6.92e-06 | 1.33e-04 |
| ENSMUSG00000005204 | Senp3 | protein\_coding | 11:69673115-69682084 (-) |  | -0.284 | 6.98e-06 | 1.34e-04 |
| ENSMUSG00000038416 | Cdc16 | protein\_coding | 8:13757676-13781938 (+) |  | -0.314 | 7.05e-06 | 1.35e-04 |
| ENSMUSG00000027583 | Zbtb46 | protein\_coding | 2:181387762-181459426 (-) |  | 0.998 | 7.25e-06 | 1.39e-04 |
| ENSMUSG00000028163 | Nfkb1 | protein\_coding | 3:135584655-135691547 (-) |  | 0.240 | 7.36e-06 | 1.41e-04 |
| ENSMUSG00000078606 | Gm4070 | protein\_coding | 7:105895139-105953967 (-) |  | -0.938 | 7.38e-06 | 1.41e-04 |
| ENSMUSG00000024436 | Mrps18b | protein\_coding | 17:35910379-35916389 (-) |  | -0.589 | 7.54e-06 | 1.44e-04 |
| ENSMUSG00000030168 | Adipor2 | protein\_coding | 6:119353150-119417704 (-) |  | 0.254 | 7.57e-06 | 1.44e-04 |
| ENSMUSG00000020277 | Pfkl | protein\_coding | 10:77986947-78010083 (-) |  | -0.387 | 7.58e-06 | 1.44e-04 |
| ENSMUSG00000074342 | I830077J02Rik | protein\_coding | 3:105924358-105932664 (-) |  | 0.326 | 7.96e-06 | 1.51e-04 |
| ENSMUSG00000024800 | Rpp30 | protein\_coding | 19:36083716-36104777 (+) |  | -0.487 | 7.97e-06 | 1.51e-04 |
| ENSMUSG00000024683 | Mrpl16 | protein\_coding | 19:11770391-11774960 (+) |  | -0.383 | 8.07e-06 | 1.52e-04 |
| ENSMUSG00000038831 | Ralgps1 | protein\_coding | 2:33133417-33371486 (-) |  | 0.823 | 8.07e-06 | 1.52e-04 |
| ENSMUSG00000060791 | Gmfg | protein\_coding | 7:28437447-28448233 (+) |  | 0.366 | 8.12e-06 | 1.53e-04 |
| ENSMUSG00000068749 | Psma5 | protein\_coding | 3:108256926-108279974 (+) |  | -0.388 | 8.31e-06 | 1.56e-04 |
| ENSMUSG00000022014 | Epsti1 | protein\_coding | 14:77904239-78002657 (+) |  | 0.535 | 8.33e-06 | 1.57e-04 |
| ENSMUSG00000029415 | Sdad1 | protein\_coding | 5:92284010-92310479 (-) |  | -0.278 | 8.36e-06 | 1.57e-04 |
| ENSMUSG00000058881 | Zfp516 | protein\_coding | 18:82910663-83005314 (+) |  | 0.352 | 8.37e-06 | 1.57e-04 |
| ENSMUSG00000026037 | Orc2 | protein\_coding | 1:58462771-58505109 (-) |  | -0.351 | 8.39e-06 | 1.57e-04 |
| ENSMUSG00000056612 | Ppp1r14b | protein\_coding | 19:6974968-6977324 (+) |  | -0.484 | 8.41e-06 | 1.57e-04 |
| ENSMUSG00000032028 | Nxpe2 | protein\_coding | 9:48318006-48353454 (-) |  | -2.140 | 8.41e-06 | 1.57e-04 |
| ENSMUSG00000057895 | Zfp105 | protein\_coding | 9:122923072-122931028 (+) |  | 1.500 | 8.49e-06 | 1.58e-04 |
| ENSMUSG00000039356 | Exosc2 | protein\_coding | 2:31670715-31681349 (+) |  | -0.367 | 8.63e-06 | 1.61e-04 |
| ENSMUSG00000015305 | Sash1 | protein\_coding | 10:8722219-8886070 (-) |  | 0.627 | 8.66e-06 | 1.61e-04 |
| ENSMUSG00000039813 | Tbc1d2 | protein\_coding | 4:46604390-46650209 (-) |  | 0.369 | 8.71e-06 | 1.61e-04 |
| ENSMUSG00000027215 | Cd82 | protein\_coding | 2:93419111-93463140 (-) |  | 0.473 | 8.72e-06 | 1.61e-04 |
| ENSMUSG00000045868 | Gvin1 | protein\_coding | 7:106156556-106215326 (-) |  | -0.811 | 8.72e-06 | 1.61e-04 |
| ENSMUSG00000038227 | Hoxa9 | protein\_coding | 6:52223100-52231089 (-) |  | -0.880 | 8.96e-06 | 1.66e-04 |
| ENSMUSG00000034708 | Grn | protein\_coding | 11:102430315-102437048 (+) |  | 0.377 | 9.04e-06 | 1.67e-04 |
| ENSMUSG00000099775 | Gm5960 | unprocessed\_pseudogene | 15:75071112-75074638 (+) |  | 0.608 | 9.21e-06 | 1.70e-04 |
| ENSMUSG00000026275 | Ppp1r7 | protein\_coding | 1:93342854-93373489 (+) |  | -0.294 | 9.30e-06 | 1.71e-04 |
| ENSMUSG00000033685 | Ucp2 | protein\_coding | 7:100493337-100502020 (+) |  | 0.304 | 9.46e-06 | 1.74e-04 |
| ENSMUSG00000024663 | Rab3il1 | protein\_coding | 19:10001669-10038380 (+) |  | -0.612 | 9.51e-06 | 1.74e-04 |
| ENSMUSG00000032067 | Pts | protein\_coding | 9:50521617-50528724 (-) |  | 0.356 | 9.52e-06 | 1.74e-04 |
| ENSMUSG00000043740 | B430306N03Rik | protein\_coding | 17:48316141-48327024 (+) |  | 0.637 | 9.61e-06 | 1.76e-04 |
| ENSMUSG00000113389 | Gm9512 | processed\_pseudogene | 13:15118632-15119128 (+) |  | 0.495 | 9.62e-06 | 1.76e-04 |
| ENSMUSG00000033706 | Smyd5 | protein\_coding | 6:85431989-85446435 (+) |  | -0.450 | 9.67e-06 | 1.76e-04 |
| ENSMUSG00000055172 | C1ra | protein\_coding | 6:124512405-124523443 (+) |  | 0.764 | 9.74e-06 | 1.77e-04 |
| ENSMUSG00000029804 | Herc3 | protein\_coding | 6:58831465-58920398 (+) |  | 0.606 | 9.77e-06 | 1.78e-04 |
| ENSMUSG00000019849 | Prep | protein\_coding | 10:45067203-45167198 (+) |  | -0.365 | 9.81e-06 | 1.78e-04 |
| ENSMUSG00000037197 | Rbm17 | protein\_coding | 2:11585437-11604153 (-) |  | -0.297 | 1.02e-05 | 1.86e-04 |
| ENSMUSG00000020023 | Tmcc3 | protein\_coding | 10:94311949-94590956 (+) |  | 1.570 | 1.02e-05 | 1.86e-04 |
| ENSMUSG00000058886 | Deaf1 | protein\_coding | 7:141297180-141327690 (-) |  | 0.433 | 1.06e-05 | 1.91e-04 |
| ENSMUSG00000032690 | Oas2 | protein\_coding | 5:120730333-120749853 (-) |  | -1.470 | 1.06e-05 | 1.92e-04 |
| ENSMUSG00000038482 | Tfdp1 | protein\_coding | 8:13338751-13378448 (+) |  | -0.312 | 1.07e-05 | 1.93e-04 |
| ENSMUSG00000034343 | Ube2f | protein\_coding | 1:91250304-91290337 (+) |  | -0.313 | 1.07e-05 | 1.93e-04 |
| ENSMUSG00000032565 | Nudt16 | protein\_coding | 9:105128903-105131824 (-) |  | 0.569 | 1.07e-05 | 1.93e-04 |
| ENSMUSG00000032089 | Il10ra | protein\_coding | 9:45253837-45269149 (-) |  | 0.448 | 1.08e-05 | 1.94e-04 |
| ENSMUSG00000070031 | Sp140 | protein\_coding | 1:85600378-85645037 (+) |  | -0.441 | 1.09e-05 | 1.95e-04 |
| ENSMUSG00000003380 | Rabac1 | protein\_coding | 7:24969752-24972754 (-) |  | 0.472 | 1.09e-05 | 1.95e-04 |
| ENSMUSG00000041481 | Serpina3g | protein\_coding | 12:104236245-104241939 (+) |  | -1.370 | 1.10e-05 | 1.96e-04 |
| ENSMUSG00000001588 | Acap1 | protein\_coding | 11:69881567-69895539 (-) |  | 0.406 | 1.10e-05 | 1.97e-04 |
| ENSMUSG00000026113 | Inpp4a | protein\_coding | 1:37299865-37410736 (+) |  | 0.353 | 1.11e-05 | 1.97e-04 |
| ENSMUSG00000015568 | Lpl | protein\_coding | 8:68880491-68907448 (+) |  | 1.170 | 1.12e-05 | 1.99e-04 |
| ENSMUSG00000040111 | Gramd1b | protein\_coding | 9:40293233-40531383 (-) |  | 0.409 | 1.12e-05 | 1.99e-04 |
| ENSMUSG00000024953 | Prdx5 | protein\_coding | 19:6906697-6910106 (-) |  | 0.456 | 1.13e-05 | 2.00e-04 |
| ENSMUSG00000035165 | Kcne3 | protein\_coding | 7:100176502-100184869 (+) |  | 1.150 | 1.13e-05 | 2.00e-04 |
| ENSMUSG00000021190 | Lgmn | protein\_coding | 12:102394084-102439813 (-) |  | -0.519 | 1.13e-05 | 2.00e-04 |
| ENSMUSG00000021143 | Pacs2 | protein\_coding | 12:113014508-113074401 (+) |  | 0.372 | 1.13e-05 | 2.00e-04 |
| ENSMUSG00000029036 | Atad3a | protein\_coding | 4:155740641-155761093 (-) |  | -0.399 | 1.15e-05 | 2.02e-04 |
| ENSMUSG00000022108 | Itm2b | protein\_coding | 14:73362226-73385289 (-) |  | 0.294 | 1.15e-05 | 2.02e-04 |
| ENSMUSG00000035629 | Rubcn | protein\_coding | 16:32821703-32877766 (-) |  | 0.335 | 1.16e-05 | 2.04e-04 |
| ENSMUSG00000007739 | Cct4 | protein\_coding | 11:22990519-23003780 (+) |  | -0.350 | 1.16e-05 | 2.05e-04 |
| ENSMUSG00000047798 | Cd300lf | protein\_coding | 11:115116214-115133992 (-) |  | -0.800 | 1.18e-05 | 2.07e-04 |
| ENSMUSG00000051329 | Nup160 | protein\_coding | 2:90677215-90736328 (+) |  | -0.307 | 1.18e-05 | 2.07e-04 |
| ENSMUSG00000074800 | Gm4149 | processed\_pseudogene | 13:75644896-75645171 (+) |  | 0.306 | 1.19e-05 | 2.08e-04 |
| ENSMUSG00000046707 | Csnk2a2 | protein\_coding | 8:95446096-95490039 (-) |  | -0.315 | 1.19e-05 | 2.08e-04 |
| ENSMUSG00000032232 | Cgnl1 | protein\_coding | 9:71626509-71771602 (-) |  | 1.350 | 1.21e-05 | 2.11e-04 |
| ENSMUSG00000036693 | Nop14 | protein\_coding | 5:34638536-34660148 (-) |  | -0.396 | 1.23e-05 | 2.14e-04 |
| ENSMUSG00000040699 | Limd2 | protein\_coding | 11:106156256-106160860 (-) |  | 0.278 | 1.23e-05 | 2.14e-04 |
| ENSMUSG00000034024 | Cct2 | protein\_coding | 10:117051001-117063814 (-) |  | -0.331 | 1.23e-05 | 2.14e-04 |
| ENSMUSG00000096727 | Psmb9 | protein\_coding | 17:34181987-34187764 (-) |  | -0.338 | 1.23e-05 | 2.15e-04 |
| ENSMUSG00000040263 | Klhdc4 | protein\_coding | 8:121796313-121829569 (-) |  | -0.370 | 1.24e-05 | 2.15e-04 |
| ENSMUSG00000033751 | Gadd45gip1 | protein\_coding | 8:84831522-84835482 (+) |  | -0.561 | 1.24e-05 | 2.16e-04 |
| ENSMUSG00000003134 | Tbc1d8 | protein\_coding | 1:39371492-39478755 (-) |  | 0.495 | 1.25e-05 | 2.17e-04 |
| ENSMUSG00000045763 | Basp1 | protein\_coding | 15:25363277-25413764 (-) |  | -1.120 | 1.27e-05 | 2.19e-04 |
| ENSMUSG00000028140 | Mrpl9 | protein\_coding | 3:94443318-94451130 (+) |  | -0.334 | 1.27e-05 | 2.20e-04 |
| ENSMUSG00000029534 | St7 | protein\_coding | 6:17692933-17943025 (+) |  | -0.569 | 1.28e-05 | 2.21e-04 |
| ENSMUSG00000038147 | Cd84 | protein\_coding | 1:171839697-171890718 (+) |  | 0.569 | 1.29e-05 | 2.21e-04 |
| ENSMUSG00000027854 | Sike1 | protein\_coding | 3:102995708-103008459 (+) |  | 0.359 | 1.29e-05 | 2.21e-04 |
| ENSMUSG00000030789 | Itgax | protein\_coding | 7:128129547-128150657 (+) |  | 0.743 | 1.29e-05 | 2.22e-04 |
| ENSMUSG00000026623 | Lpgat1 | protein\_coding | 1:191717834-191784255 (+) |  | -0.272 | 1.29e-05 | 2.22e-04 |
| ENSMUSG00000022752 | Tomm70a | protein\_coding | 16:57121703-57156705 (+) |  | -0.375 | 1.30e-05 | 2.24e-04 |
| ENSMUSG00000029390 | Tmed2 | protein\_coding | 5:124540695-124550506 (+) |  | -0.339 | 1.31e-05 | 2.24e-04 |
| ENSMUSG00000042042 | Csgalnact2 | protein\_coding | 6:118107452-118139140 (-) |  | 0.257 | 1.31e-05 | 2.24e-04 |
| ENSMUSG00000004393 | Ddx56 | protein\_coding | 11:6258919-6267772 (-) |  | -0.414 | 1.32e-05 | 2.24e-04 |
| ENSMUSG00000027184 | Caprin1 | protein\_coding | 2:103762941-103797649 (-) |  | -0.245 | 1.32e-05 | 2.26e-04 |
| ENSMUSG00000042249 | Grk3 | protein\_coding | 5:112910482-113015791 (-) |  | 0.489 | 1.34e-05 | 2.27e-04 |
| ENSMUSG00000031442 | Mcf2l | protein\_coding | 8:12873806-13020905 (+) |  | 0.421 | 1.35e-05 | 2.29e-04 |
| ENSMUSG00000045636 | Mtus1 | protein\_coding | 8:40990914-41133726 (-) |  | -0.227 | 1.36e-05 | 2.32e-04 |
| ENSMUSG00000032115 | Hyou1 | protein\_coding | 9:44379490-44392369 (+) |  | -0.442 | 1.38e-05 | 2.33e-04 |
| ENSMUSG00000024007 | Ppil1 | protein\_coding | 17:29250803-29264186 (-) |  | -0.298 | 1.38e-05 | 2.33e-04 |
| ENSMUSG00000005732 | Ranbp1 | protein\_coding | 16:18239784-18248732 (-) |  | -0.405 | 1.38e-05 | 2.34e-04 |
| ENSMUSG00000051748 | Wfdc21 | protein\_coding | 11:83746940-83752642 (+) |  | -2.610 | 1.38e-05 | 2.34e-04 |
| ENSMUSG00000025140 | Pycr1 | protein\_coding | 11:120635712-120643769 (-) |  | 0.573 | 1.41e-05 | 2.38e-04 |
| ENSMUSG00000040345 | Arhgap9 | protein\_coding | 10:127321964-127329943 (+) |  | 0.337 | 1.45e-05 | 2.44e-04 |
| ENSMUSG00000064037 | Gpn1 | protein\_coding | 5:31494741-31512904 (+) |  | -0.389 | 1.46e-05 | 2.45e-04 |
| ENSMUSG00000028410 | Dnaja1 | protein\_coding | 4:40722150-40737149 (+) |  | -0.396 | 1.46e-05 | 2.45e-04 |
| ENSMUSG00000059013 | Sh2d3c | protein\_coding | 2:32721055-32755512 (+) |  | 0.496 | 1.47e-05 | 2.46e-04 |
| ENSMUSG00000029599 | Ddx54 | protein\_coding | 5:120612739-120628592 (+) |  | -0.292 | 1.48e-05 | 2.48e-04 |
| ENSMUSG00000053801 | Grwd1 | protein\_coding | 7:45825223-45830944 (-) |  | -0.447 | 1.48e-05 | 2.48e-04 |
| ENSMUSG00000025962 | Fastkd2 | protein\_coding | 1:63730614-63754655 (+) |  | -0.500 | 1.50e-05 | 2.51e-04 |
| ENSMUSG00000036561 | Ppp6r2 | protein\_coding | 15:89211553-89287010 (+) |  | 0.545 | 1.51e-05 | 2.51e-04 |
| ENSMUSG00000028789 | Azin2 | protein\_coding | 4:128930233-128962442 (-) |  | 1.480 | 1.53e-05 | 2.55e-04 |
| ENSMUSG00000030094 | Xpc | protein\_coding | 6:91489305-91515888 (-) |  | 0.459 | 1.53e-05 | 2.55e-04 |
| ENSMUSG00000028729 | Ebna1bp2 | protein\_coding | 4:118620799-118627776 (+) |  | -0.423 | 1.54e-05 | 2.55e-04 |
| ENSMUSG00000074657 | Kif5a | protein\_coding | 10:127225696-127263348 (-) |  | 1.080 | 1.54e-05 | 2.55e-04 |
| ENSMUSG00000040521 | Tsfm | protein\_coding | 10:127011572-127030840 (-) |  | -0.376 | 1.55e-05 | 2.56e-04 |
| ENSMUSG00000078652 | Psme3 | protein\_coding | 11:101316213-101323537 (+) |  | -0.299 | 1.58e-05 | 2.62e-04 |
| ENSMUSG00000052738 | Suclg1 | protein\_coding | 6:73248382-73276911 (+) |  | -0.340 | 1.58e-05 | 2.62e-04 |
| ENSMUSG00000019820 | Utrn | protein\_coding | 10:12382188-12869365 (-) |  | 0.513 | 1.58e-05 | 2.62e-04 |
| ENSMUSG00000056592 | Zfp658 | protein\_coding | 7:43562256-43575461 (+) |  | 0.897 | 1.59e-05 | 2.62e-04 |
| ENSMUSG00000022698 | Naa50 | protein\_coding | 16:44139830-44163366 (+) |  | -0.383 | 1.60e-05 | 2.63e-04 |
| ENSMUSG00000044456 | Rin3 | protein\_coding | 12:102283048-102390855 (+) |  | 0.332 | 1.60e-05 | 2.64e-04 |
| ENSMUSG00000020681 | Ace | protein\_coding | 11:105967945-105989964 (+) |  | 3.920 | 1.61e-05 | 2.65e-04 |
| ENSMUSG00000034427 | Myo15b | protein\_coding | 11:115858406-115892603 (+) |  | 1.130 | 1.65e-05 | 2.71e-04 |
| ENSMUSG00000038034 | Igsf8 | protein\_coding | 1:172261641-172319841 (+) |  | -0.250 | 1.65e-05 | 2.71e-04 |
| ENSMUSG00000037149 | Ddx1 | protein\_coding | 12:13216973-13249213 (-) |  | -0.426 | 1.67e-05 | 2.73e-04 |
| ENSMUSG00000032423 | Syncrip | protein\_coding | 9:88447009-88482574 (-) |  | -0.330 | 1.67e-05 | 2.74e-04 |
| ENSMUSG00000022774 | Ncbp2 | protein\_coding | 16:31948513-31961781 (+) |  | -0.286 | 1.69e-05 | 2.76e-04 |
| ENSMUSG00000071669 | Snx29 | protein\_coding | 16:11322908-11755472 (+) |  | 0.767 | 1.70e-05 | 2.78e-04 |
| ENSMUSG00000032417 | Rwdd2a | protein\_coding | 9:86571991-86574899 (+) |  | 0.725 | 1.72e-05 | 2.80e-04 |
| ENSMUSG00000041763 | Tpp2 | protein\_coding | 1:43933647-44003000 (+) |  | -0.331 | 1.73e-05 | 2.82e-04 |
| ENSMUSG00000019302 | Atp6v0a1 | protein\_coding | 11:101009452-101063719 (+) |  | 0.651 | 1.74e-05 | 2.84e-04 |
| ENSMUSG00000073838 | Tufm | protein\_coding | 7:126487361-126490731 (+) |  | -0.379 | 1.76e-05 | 2.86e-04 |
| ENSMUSG00000015291 | Gdi1 | protein\_coding | X:74304998-74311862 (+) |  | 0.373 | 1.76e-05 | 2.86e-04 |
| ENSMUSG00000000384 | Tbrg4 | protein\_coding | 11:6615598-6626067 (-) |  | -0.387 | 1.76e-05 | 2.86e-04 |
| ENSMUSG00000020708 | Psmc5 | protein\_coding | 11:106256154-106263120 (+) |  | -0.342 | 1.77e-05 | 2.86e-04 |
| ENSMUSG00000071547 | Nt5dc2 | protein\_coding | 14:31131053-31139124 (+) |  | -0.406 | 1.78e-05 | 2.87e-04 |
| ENSMUSG00000038143 | Stox2 | protein\_coding | 8:47180048-47446362 (-) |  | 0.631 | 1.79e-05 | 2.88e-04 |
| ENSMUSG00000051695 | Pcbp1 | protein\_coding | 6:86524492-86526321 (-) |  | -0.273 | 1.79e-05 | 2.89e-04 |
| ENSMUSG00000027944 | Hax1 | protein\_coding | 3:89995446-89998780 (-) |  | -0.456 | 1.80e-05 | 2.89e-04 |
| ENSMUSG00000030697 | Ppp4c | protein\_coding | 7:126785866-126792496 (-) |  | -0.278 | 1.81e-05 | 2.91e-04 |
| ENSMUSG00000060098 | Prmt7 | protein\_coding | 8:106210936-106252794 (+) |  | -0.420 | 1.84e-05 | 2.95e-04 |
| ENSMUSG00000032563 | Mrpl3 | protein\_coding | 9:105053239-105079888 (+) |  | -0.412 | 1.85e-05 | 2.97e-04 |
| ENSMUSG00000020692 | Nle1 | protein\_coding | 11:82900768-82908411 (-) |  | -0.481 | 1.85e-05 | 2.97e-04 |
| ENSMUSG00000021375 | Kif13a | protein\_coding | 13:46749087-46929867 (-) |  | 0.269 | 1.87e-05 | 2.99e-04 |
| ENSMUSG00000031389 | Arhgap4 | protein\_coding | X:73891442-73921870 (-) |  | 0.357 | 1.87e-05 | 2.99e-04 |
| ENSMUSG00000010517 | Faf1 | protein\_coding | 4:109676588-109963960 (+) |  | -0.345 | 1.89e-05 | 3.02e-04 |
| ENSMUSG00000020650 | Bcap29 | protein\_coding | 12:31590967-31634658 (-) |  | -0.374 | 1.95e-05 | 3.10e-04 |
| ENSMUSG00000049295 | Zfp219 | protein\_coding | 14:52006077-52020733 (-) |  | 0.460 | 1.98e-05 | 3.15e-04 |
| ENSMUSG00000044072 | Eml6 | protein\_coding | 11:29743048-30026033 (-) |  | 1.160 | 1.98e-05 | 3.15e-04 |
| ENSMUSG00000030498 | Gas2 | protein\_coding | 7:51862015-51994975 (+) |  | 0.567 | 1.99e-05 | 3.16e-04 |
| ENSMUSG00000053907 | Mat2a | protein\_coding | 6:72432799-72439558 (-) |  | -0.333 | 1.99e-05 | 3.16e-04 |
| ENSMUSG00000032966 | Fkbp1a | protein\_coding | 2:151542483-151561692 (+) |  | -0.292 | 2.00e-05 | 3.17e-04 |
| ENSMUSG00000042215 | Bag2 | protein\_coding | 1:33745484-33757795 (-) |  | -0.678 | 2.01e-05 | 3.17e-04 |
| ENSMUSG00000019916 | P4ha1 | protein\_coding | 10:59323296-59373304 (+) |  | -0.360 | 2.01e-05 | 3.18e-04 |
| ENSMUSG00000051517 | Arhgef39 | protein\_coding | 4:43496142-43499695 (-) |  | 0.333 | 2.03e-05 | 3.21e-04 |
| ENSMUSG00000037601 | Nme1 | protein\_coding | 11:93956979-93968521 (-) |  | -0.476 | 2.04e-05 | 3.21e-04 |
| ENSMUSG00000071203 | Naip5 | protein\_coding | 13:100211739-100246323 (-) |  | 0.506 | 2.04e-05 | 3.21e-04 |
| ENSMUSG00000046688 | Tifa | protein\_coding | 3:127789805-127832164 (+) |  | 0.347 | 2.04e-05 | 3.21e-04 |
| ENSMUSG00000036452 | Arhgap26 | protein\_coding | 18:38993145-39376284 (+) |  | -0.350 | 2.06e-05 | 3.23e-04 |
| ENSMUSG00000028977 | Casz1 | protein\_coding | 4:148804429-148954889 (+) |  | 0.485 | 2.06e-05 | 3.23e-04 |
| ENSMUSG00000030980 | Knop1 | protein\_coding | 7:118842222-118856254 (-) |  | -0.352 | 2.06e-05 | 3.23e-04 |
| ENSMUSG00000027288 | Zfp106 | protein\_coding | 2:120506820-120563843 (-) |  | -0.269 | 2.07e-05 | 3.23e-04 |
| ENSMUSG00000030689 | Ino80e | protein\_coding | 7:126850960-126862377 (-) |  | -0.291 | 2.07e-05 | 3.24e-04 |
| ENSMUSG00000020464 | Pnpt1 | protein\_coding | 11:29130744-29161828 (+) |  | -0.335 | 2.08e-05 | 3.25e-04 |
| ENSMUSG00000035890 | Rnf126 | protein\_coding | 10:79758515-79766952 (-) |  | -0.302 | 2.09e-05 | 3.25e-04 |
| ENSMUSG00000023571 | C1qtnf12 | protein\_coding | 4:155962318-155966629 (+) |  | 0.527 | 2.10e-05 | 3.27e-04 |
| ENSMUSG00000029455 | Aldh2 | protein\_coding | 5:121566027-121593824 (-) |  | 0.307 | 2.10e-05 | 3.27e-04 |
| ENSMUSG00000043483 | Gm6863 | processed\_pseudogene | 12:96922423-96923058 (-) |  | -0.365 | 2.11e-05 | 3.27e-04 |
| ENSMUSG00000041891 | Lman1 | protein\_coding | 18:65980738-66022580 (-) |  | -0.326 | 2.11e-05 | 3.27e-04 |
| ENSMUSG00000014907 | Naf1 | protein\_coding | 8:66860217-66890564 (+) |  | -0.391 | 2.13e-05 | 3.30e-04 |
| ENSMUSG00000031103 | Elf4 | protein\_coding | X:48411046-48463132 (-) |  | 0.325 | 2.13e-05 | 3.30e-04 |
| ENSMUSG00000087129 | Gm16316 | lncRNA | 2:163685004-163692279 (-) |  | 1.530 | 2.14e-05 | 3.30e-04 |
| ENSMUSG00000095742 | CAAA01147332.1 | protein\_coding | JH584295.1:66-1479 (-) |  | 0.896 | 2.14e-05 | 3.30e-04 |
| ENSMUSG00000053716 | Dusp7 | protein\_coding | 9:106368632-106375724 (+) |  | 0.514 | 2.15e-05 | 3.31e-04 |
| ENSMUSG00000022792 | Yars2 | protein\_coding | 16:16302965-16309640 (+) |  | -0.485 | 2.17e-05 | 3.35e-04 |
| ENSMUSG00000071337 | Tia1 | protein\_coding | 6:86404219-86433405 (+) |  | 0.322 | 2.18e-05 | 3.35e-04 |
| ENSMUSG00000038510 | Rpf2 | protein\_coding | 10:40223246-40247036 (-) |  | -0.483 | 2.22e-05 | 3.41e-04 |
| ENSMUSG00000028861 | Mrps15 | protein\_coding | 4:126046925-126055532 (+) |  | -0.419 | 2.23e-05 | 3.42e-04 |
| ENSMUSG00000022234 | Cct5 | protein\_coding | 15:31590800-31601804 (-) |  | -0.321 | 2.25e-05 | 3.45e-04 |
| ENSMUSG00000041633 | Kctd12b | protein\_coding | X:153685154-153696391 (-) |  | 0.802 | 2.28e-05 | 3.49e-04 |
| ENSMUSG00000020664 | Dld | protein\_coding | 12:31331277-31351453 (-) |  | -0.359 | 2.29e-05 | 3.51e-04 |
| ENSMUSG00000036781 | Rps27l | protein\_coding | 9:66946086-66949516 (+) |  | -0.546 | 2.30e-05 | 3.51e-04 |
| ENSMUSG00000046756 | Mrps7 | protein\_coding | 11:115603925-115608036 (+) |  | -0.384 | 2.35e-05 | 3.59e-04 |
| ENSMUSG00000063480 | Snu13 | protein\_coding | 15:82040525-82047598 (-) |  | -0.401 | 2.36e-05 | 3.60e-04 |
| ENSMUSG00000029432 | Nipsnap2 | protein\_coding | 5:129725063-129758327 (+) |  | 0.382 | 2.38e-05 | 3.63e-04 |
| ENSMUSG00000035021 | Baz1a | protein\_coding | 12:54892989-55014348 (-) |  | -0.310 | 2.40e-05 | 3.66e-04 |
| ENSMUSG00000034653 | Ythdc2 | protein\_coding | 18:44827746-44889724 (+) |  | -0.336 | 2.42e-05 | 3.68e-04 |
| ENSMUSG00000059713 | Rcan3 | protein\_coding | 4:135412308-135433853 (-) |  | 0.417 | 2.44e-05 | 3.70e-04 |
| ENSMUSG00000043257 | Pigv | protein\_coding | 4:133660387-133672647 (-) |  | 0.439 | 2.45e-05 | 3.71e-04 |
| ENSMUSG00000027185 | Nat10 | protein\_coding | 2:103721256-103761270 (-) |  | -0.420 | 2.45e-05 | 3.71e-04 |
| ENSMUSG00000028907 | Utp11 | protein\_coding | 4:124678160-124693600 (-) |  | -0.314 | 2.45e-05 | 3.71e-04 |
| ENSMUSG00000023852 | Chd1 | protein\_coding | 17:15704967-15772610 (+) |  | -0.247 | 2.46e-05 | 3.72e-04 |
| ENSMUSG00000005069 | Pex5 | protein\_coding | 6:124396816-124415067 (-) |  | 0.281 | 2.47e-05 | 3.72e-04 |
| ENSMUSG00000067787 | Blcap | protein\_coding | 2:157556362-157571274 (-) |  | 0.516 | 2.48e-05 | 3.74e-04 |
| ENSMUSG00000025212 | Sfxn3 | protein\_coding | 19:45047503-45056383 (+) |  | 0.496 | 2.50e-05 | 3.76e-04 |
| ENSMUSG00000019066 | Rab3d | protein\_coding | 9:21907491-21918192 (-) |  | 0.309 | 2.51e-05 | 3.77e-04 |
| ENSMUSG00000045176 | Borcs6 | protein\_coding | 11:69059717-69061578 (+) |  | 0.528 | 2.53e-05 | 3.79e-04 |
| ENSMUSG00000001054 | Rmnd5b | protein\_coding | 11:51623671-51635896 (-) |  | 0.340 | 2.53e-05 | 3.79e-04 |
| ENSMUSG00000026558 | Uck2 | protein\_coding | 1:167222883-167285320 (-) |  | -0.270 | 2.53e-05 | 3.79e-04 |
| ENSMUSG00000085611 | Ap3s1-ps1 | processed\_pseudogene | X:38685592-38686170 (-) |  | -0.464 | 2.53e-05 | 3.79e-04 |
| ENSMUSG00000052310 | Slc39a1 | protein\_coding | 3:90248172-90253612 (+) |  | -0.303 | 2.54e-05 | 3.79e-04 |
| ENSMUSG00000030189 | Ybx3 | protein\_coding | 6:131364855-131388476 (-) |  | -0.395 | 2.54e-05 | 3.79e-04 |
| ENSMUSG00000027463 | Slc52a3 | protein\_coding | 2:151996511-152009258 (+) |  | -2.150 | 2.55e-05 | 3.80e-04 |
| ENSMUSG00000068141 | Gm10232 | processed\_pseudogene | 17:3044014-3044733 (-) |  | -0.573 | 2.60e-05 | 3.87e-04 |
| ENSMUSG00000006932 | Ctnnb1 | protein\_coding | 9:120929216-120960507 (+) |  | -0.220 | 2.60e-05 | 3.87e-04 |
| ENSMUSG00000019297 | Nop9 | protein\_coding | 14:55745693-55755500 (+) |  | -0.287 | 2.61e-05 | 3.88e-04 |
| ENSMUSG00000003153 | Slc2a3 | protein\_coding | 6:122727809-122801640 (-) |  | 0.538 | 2.62e-05 | 3.89e-04 |
| ENSMUSG00000037649 | H2-DMa | protein\_coding | 17:34119541-34139101 (+) |  | 0.422 | 2.63e-05 | 3.91e-04 |
| ENSMUSG00000056665 | Them6 | protein\_coding | 15:74721204-74724639 (+) |  | -0.622 | 2.65e-05 | 3.93e-04 |
| ENSMUSG00000028799 | Zfp362 | protein\_coding | 4:128773088-128806045 (-) |  | 0.437 | 2.67e-05 | 3.95e-04 |
| ENSMUSG00000078942 | Naip6 | protein\_coding | 13:100281121-100317674 (-) |  | 0.503 | 2.68e-05 | 3.96e-04 |
| ENSMUSG00000079037 | Prnp | protein\_coding | 2:131909928-131938429 (+) |  | -1.530 | 2.69e-05 | 3.97e-04 |
| ENSMUSG00000026566 | Mpzl1 | protein\_coding | 1:165592240-165634538 (-) |  | -0.955 | 2.70e-05 | 3.98e-04 |
| ENSMUSG00000020649 | Rrm2 | protein\_coding | 12:24708241-24714146 (+) |  | -0.289 | 2.79e-05 | 4.11e-04 |
| ENSMUSG00000021427 | Ssr1 | protein\_coding | 13:37966605-37994217 (-) |  | -0.291 | 2.81e-05 | 4.13e-04 |
| ENSMUSG00000052336 | Cx3cr1 | protein\_coding | 9:119901616-120069879 (-) |  | 0.996 | 2.83e-05 | 4.16e-04 |
| ENSMUSG00000029304 | Spp1 | protein\_coding | 5:104435118-104441050 (+) |  | -0.627 | 2.84e-05 | 4.16e-04 |
| ENSMUSG00000026766 | Mmadhc | protein\_coding | 2:50279881-50296801 (-) |  | -0.365 | 2.85e-05 | 4.17e-04 |
| ENSMUSG00000024070 | Prkd3 | protein\_coding | 17:78949405-79020816 (-) |  | 0.266 | 2.90e-05 | 4.25e-04 |
| ENSMUSG00000062866 | Phactr2 | protein\_coding | 10:13207717-13474412 (-) |  | 0.376 | 2.92e-05 | 4.27e-04 |
| ENSMUSG00000026896 | Ifih1 | protein\_coding | 2:62595798-62646255 (-) |  | -0.531 | 2.93e-05 | 4.28e-04 |
| ENSMUSG00000044149 | Nkrf | protein\_coding | X:36887540-36903513 (-) |  | -0.395 | 2.93e-05 | 4.28e-04 |
| ENSMUSG00000033545 | Znrf1 | protein\_coding | 8:111536097-111626030 (+) |  | 0.342 | 2.94e-05 | 4.28e-04 |
| ENSMUSG00000018697 | Aatf | protein\_coding | 11:84422855-84513522 (-) |  | -0.369 | 2.95e-05 | 4.30e-04 |
| ENSMUSG00000063787 | Chchd1 | protein\_coding | 14:20703006-20704425 (+) |  | -0.486 | 3.01e-05 | 4.37e-04 |
| ENSMUSG00000046687 | Gm5424 | processed\_pseudogene | 10:62071123-62072362 (+) |  | -0.288 | 3.01e-05 | 4.37e-04 |
| ENSMUSG00000020642 | Rnf144a | protein\_coding | 12:26300964-26415254 (-) |  | 0.351 | 3.02e-05 | 4.38e-04 |
| ENSMUSG00000030930 | Chst15 | protein\_coding | 7:132235780-132317228 (-) |  | 0.774 | 3.02e-05 | 4.38e-04 |
| ENSMUSG00000020834 | Dhrs13 | protein\_coding | 11:78032280-78037866 (+) |  | -0.580 | 3.04e-05 | 4.40e-04 |
| ENSMUSG00000001415 | Smg5 | protein\_coding | 3:88336260-88362338 (+) |  | -0.273 | 3.07e-05 | 4.44e-04 |
| ENSMUSG00000032185 | Carm1 | protein\_coding | 9:21546894-21592623 (+) |  | -0.336 | 3.07e-05 | 4.44e-04 |
| ENSMUSG00000022451 | Twf1 | protein\_coding | 15:94577951-94589889 (-) |  | -0.456 | 3.12e-05 | 4.50e-04 |
| ENSMUSG00000040907 | Atp1a3 | protein\_coding | 7:24978167-25005958 (-) |  | 0.398 | 3.13e-05 | 4.52e-04 |
| ENSMUSG00000030047 | Arhgap25 | protein\_coding | 6:87458545-87533259 (-) |  | 0.479 | 3.14e-05 | 4.52e-04 |
| ENSMUSG00000039145 | Camk1d | protein\_coding | 2:5293457-5714515 (-) |  | 1.070 | 3.16e-05 | 4.54e-04 |
| ENSMUSG00000037822 | Smim14 | protein\_coding | 5:65446844-65537184 (-) |  | 0.466 | 3.16e-05 | 4.54e-04 |
| ENSMUSG00000021111 | Papola | protein\_coding | 12:105784694-105838944 (+) |  | -0.265 | 3.17e-05 | 4.54e-04 |
| ENSMUSG00000020680 | Taf15 | protein\_coding | 11:83473086-83506743 (+) |  | -0.297 | 3.17e-05 | 4.55e-04 |
| ENSMUSG00000021660 | Btf3 | protein\_coding | 13:98309896-98317006 (-) |  | -0.321 | 3.22e-05 | 4.61e-04 |
| ENSMUSG00000024078 | Ttc27 | protein\_coding | 17:74717732-74863570 (+) |  | -0.394 | 3.25e-05 | 4.65e-04 |
| ENSMUSG00000025571 | Tnrc6c | protein\_coding | 11:117654289-117763439 (+) |  | 0.296 | 3.27e-05 | 4.66e-04 |
| ENSMUSG00000030298 | Sec13 | protein\_coding | 6:113728062-113740743 (-) |  | -0.253 | 3.27e-05 | 4.66e-04 |
| ENSMUSG00000028759 | Hp1bp3 | protein\_coding | 4:138216296-138244683 (+) |  | 0.212 | 3.28e-05 | 4.66e-04 |
| ENSMUSG00000055897 | Ppp4r1l-ps | transcribed\_unprocessed\_pseudogene | 2:173579320-173659640 (-) |  | 0.631 | 3.28e-05 | 4.66e-04 |
| ENSMUSG00000027597 | Ahcy | protein\_coding | 2:155059310-155074497 (-) |  | -0.402 | 3.28e-05 | 4.66e-04 |
| ENSMUSG00000041235 | Chd7 | protein\_coding | 4:8690406-8867659 (+) |  | -0.437 | 3.32e-05 | 4.71e-04 |
| ENSMUSG00000036078 | Sigmar1 | protein\_coding | 4:41738493-41756157 (-) |  | -0.275 | 3.33e-05 | 4.73e-04 |
| ENSMUSG00000001774 | Chordc1 | protein\_coding | 9:18292125-18317442 (+) |  | -0.398 | 3.35e-05 | 4.75e-04 |
| ENSMUSG00000025875 | Tspan17 | protein\_coding | 13:54789377-54796776 (+) |  | 0.825 | 3.36e-05 | 4.75e-04 |
| ENSMUSG00000023068 | Nus1 | protein\_coding | 10:52417547-52440183 (+) |  | -0.293 | 3.37e-05 | 4.77e-04 |
| ENSMUSG00000021810 | Ecd | protein\_coding | 14:20319852-20348121 (-) |  | -0.319 | 3.38e-05 | 4.78e-04 |
| ENSMUSG00000020280 | Pus10 | protein\_coding | 11:23665674-23732876 (+) |  | -0.315 | 3.39e-05 | 4.78e-04 |
| ENSMUSG00000063524 | Eno1 | protein\_coding | 4:150236721-150248879 (+) |  | -0.356 | 3.40e-05 | 4.80e-04 |
| ENSMUSG00000076617 | Ighm | IG\_C\_gene | 12:113418558-113422730 (-) |  | 0.343 | 3.41e-05 | 4.80e-04 |
| ENSMUSG00000038732 | Mboat1 | protein\_coding | 13:30136489-30246717 (+) |  | -0.386 | 3.41e-05 | 4.80e-04 |
| ENSMUSG00000033307 | Mif | protein\_coding | 10:75859353-75860240 (-) |  | -0.521 | 3.42e-05 | 4.80e-04 |
| ENSMUSG00000033793 | Atp6v1h | protein\_coding | 1:5070018-5162529 (+) |  | -0.361 | 3.44e-05 | 4.83e-04 |
| ENSMUSG00000013629 | Cad | protein\_coding | 5:31054780-31078479 (+) |  | -0.447 | 3.45e-05 | 4.84e-04 |
| ENSMUSG00000035049 | Rrp12 | protein\_coding | 19:41862851-41896173 (-) |  | -0.464 | 3.48e-05 | 4.87e-04 |
| ENSMUSG00000015968 | Cacna1d | protein\_coding | 14:30039939-30491455 (-) |  | 0.799 | 3.49e-05 | 4.88e-04 |
| ENSMUSG00000052013 | Btla | protein\_coding | 16:45224315-45257670 (+) |  | -0.451 | 3.53e-05 | 4.93e-04 |
| ENSMUSG00000060703 | Cd302 | protein\_coding | 2:60251993-60284488 (-) |  | -0.664 | 3.53e-05 | 4.93e-04 |
| ENSMUSG00000040565 | Btaf1 | protein\_coding | 19:36926079-37012752 (+) |  | -0.227 | 3.58e-05 | 5.00e-04 |
| ENSMUSG00000022377 | Asap1 | protein\_coding | 15:64086857-64382919 (-) |  | -0.272 | 3.60e-05 | 5.01e-04 |
| ENSMUSG00000005378 | Bud23 | protein\_coding | 5:135052957-135064959 (-) |  | -0.324 | 3.62e-05 | 5.04e-04 |
| ENSMUSG00000000359 | Rem1 | protein\_coding | 2:152626951-152635198 (+) |  | 0.909 | 3.63e-05 | 5.04e-04 |
| ENSMUSG00000020696 | Rffl | protein\_coding | 11:82802449-82871210 (-) |  | 0.388 | 3.63e-05 | 5.04e-04 |
| ENSMUSG00000009470 | Tnpo1 | protein\_coding | 13:98839019-98926384 (-) |  | -0.305 | 3.64e-05 | 5.05e-04 |
| ENSMUSG00000049999 | Ppp1r3d | protein\_coding | 2:178411206-178414472 (-) |  | -0.794 | 3.67e-05 | 5.08e-04 |
| ENSMUSG00000040746 | Rnf167 | protein\_coding | 11:70647235-70651421 (+) |  | 0.409 | 3.67e-05 | 5.09e-04 |
| ENSMUSG00000074093 | Svip | protein\_coding | 7:51997171-52006018 (-) |  | 0.307 | 3.69e-05 | 5.11e-04 |
| ENSMUSG00000001056 | Nhp2 | protein\_coding | 11:51619735-51623714 (+) |  | -0.378 | 3.76e-05 | 5.19e-04 |
| ENSMUSG00000028760 | Eif4g3 | protein\_coding | 4:137993022-138208508 (+) |  | 0.311 | 3.83e-05 | 5.28e-04 |
| ENSMUSG00000001473 | Tubb6 | protein\_coding | 18:67390717-67402749 (+) |  | -0.466 | 3.92e-05 | 5.40e-04 |
| ENSMUSG00000038762 | Abcf1 | protein\_coding | 17:35956819-35969761 (-) |  | -0.285 | 3.95e-05 | 5.44e-04 |
| ENSMUSG00000004567 | Mcoln1 | protein\_coding | 8:3500457-3515232 (+) |  | 0.441 | 3.96e-05 | 5.45e-04 |
| ENSMUSG00000060568 | Fam78b | protein\_coding | 1:167001417-167091302 (+) |  | 1.760 | 3.99e-05 | 5.49e-04 |
| ENSMUSG00000081344 | Gm14303 | processed\_pseudogene | 2:172509521-172509691 (-) |  | 0.544 | 4.01e-05 | 5.50e-04 |
| ENSMUSG00000002871 | Tpra1 | protein\_coding | 6:88902251-88912238 (+) |  | 0.348 | 4.02e-05 | 5.51e-04 |
| ENSMUSG00000035944 | Ttc38 | protein\_coding | 15:85832306-85858822 (+) |  | 0.484 | 4.03e-05 | 5.52e-04 |
| ENSMUSG00000017707 | Serinc3 | protein\_coding | 2:163623272-163645131 (-) |  | 0.227 | 4.09e-05 | 5.59e-04 |
| ENSMUSG00000061414 | Cracr2a | protein\_coding | 6:127561338-127674248 (+) |  | 0.459 | 4.09e-05 | 5.59e-04 |
| ENSMUSG00000031422 | Morf4l2 | protein\_coding | X:136732942-136743690 (-) |  | -0.301 | 4.10e-05 | 5.60e-04 |
| ENSMUSG00000069833 | Ahnak | protein\_coding | 19:8989284-9076914 (+) |  | 0.495 | 4.11e-05 | 5.60e-04 |
| ENSMUSG00000047676 | Rpsa-ps10 | processed\_pseudogene | 3:150072658-150073542 (-) |  | -0.393 | 4.11e-05 | 5.60e-04 |
| ENSMUSG00000019782 | Rwdd1 | protein\_coding | 10:33996555-34019624 (-) |  | -0.443 | 4.14e-05 | 5.64e-04 |
| ENSMUSG00000037992 | Rara | protein\_coding | 11:98927818-98974942 (+) |  | 0.453 | 4.16e-05 | 5.66e-04 |
| ENSMUSG00000024812 | Tjp2 | protein\_coding | 19:24094505-24225030 (-) |  | -0.588 | 4.17e-05 | 5.67e-04 |
| ENSMUSG00000020741 | Cluh | protein\_coding | 11:74649495-74670847 (+) |  | -0.321 | 4.20e-05 | 5.70e-04 |
| ENSMUSG00000027108 | Ola1 | protein\_coding | 2:73092801-73218924 (-) |  | -0.363 | 4.21e-05 | 5.70e-04 |
| ENSMUSG00000024238 | Zeb1 | protein\_coding | 18:5591860-5775467 (+) |  | 0.831 | 4.21e-05 | 5.70e-04 |
| ENSMUSG00000074604 | Mgst2 | protein\_coding | 3:51660360-51682677 (+) |  | 0.586 | 4.21e-05 | 5.70e-04 |
| ENSMUSG00000027997 | Casp6 | protein\_coding | 3:129901425-129914103 (+) |  | -0.557 | 4.22e-05 | 5.70e-04 |
| ENSMUSG00000030231 | Plekha5 | protein\_coding | 6:140424054-140597110 (+) |  | -0.684 | 4.30e-05 | 5.80e-04 |
| ENSMUSG00000017309 | Cd300lg | protein\_coding | 11:102041509-102055620 (+) |  | 0.536 | 4.30e-05 | 5.80e-04 |
| ENSMUSG00000027435 | Cd93 | protein\_coding | 2:148436640-148443563 (-) |  | 0.438 | 4.35e-05 | 5.87e-04 |
| ENSMUSG00000038279 | Nop2 | protein\_coding | 6:125131909-125144753 (+) |  | -0.380 | 4.41e-05 | 5.94e-04 |
| ENSMUSG00000052609 | Plekhg3 | protein\_coding | 12:76530891-76580488 (+) |  | 0.325 | 4.42e-05 | 5.95e-04 |
| ENSMUSG00000033166 | Dis3 | protein\_coding | 14:99075206-99099770 (-) |  | -0.336 | 4.48e-05 | 6.01e-04 |
| ENSMUSG00000028961 | Pgd | protein\_coding | 4:149149991-149166771 (-) |  | -0.231 | 4.48e-05 | 6.01e-04 |
| ENSMUSG00000022010 | Tsc22d1 | protein\_coding | 14:76414961-76507765 (+) |  | 0.454 | 4.48e-05 | 6.01e-04 |
| ENSMUSG00000022247 | Brix1 | protein\_coding | 15:10474779-10485947 (-) |  | -0.405 | 4.51e-05 | 6.04e-04 |
| ENSMUSG00000060989 | Gm11847 | processed\_pseudogene | 4:12232833-12233951 (+) |  | -0.540 | 4.53e-05 | 6.05e-04 |
| ENSMUSG00000030733 | Sh2b1 | protein\_coding | 7:126466994-126475424 (-) |  | 0.377 | 4.63e-05 | 6.18e-04 |
| ENSMUSG00000056220 | Pla2g4a | protein\_coding | 1:149829618-149961290 (-) |  | -0.386 | 4.67e-05 | 6.23e-04 |
| ENSMUSG00000035351 | Nup37 | protein\_coding | 10:88146992-88178390 (+) |  | -0.434 | 4.68e-05 | 6.24e-04 |
| ENSMUSG00000020547 | Bzw2 | protein\_coding | 12:36091835-36158080 (-) |  | -0.402 | 4.69e-05 | 6.25e-04 |
| ENSMUSG00000021583 | Erap1 | protein\_coding | 13:74639568-74693201 (+) |  | -0.279 | 4.70e-05 | 6.25e-04 |
| ENSMUSG00000034156 | Tspoap1 | protein\_coding | 11:87760541-87785928 (+) |  | 0.395 | 4.73e-05 | 6.29e-04 |
| ENSMUSG00000006378 | Gcat | protein\_coding | 15:79030874-79038353 (+) |  | -1.100 | 4.75e-05 | 6.31e-04 |
| ENSMUSG00000058794 | Nfe2 | protein\_coding | 15:103248212-103258403 (-) |  | 0.287 | 4.77e-05 | 6.33e-04 |
| ENSMUSG00000032123 | Dpagt1 | protein\_coding | 9:44326019-44333900 (+) |  | -0.282 | 4.79e-05 | 6.35e-04 |
| ENSMUSG00000030983 | Bccip | protein\_coding | 7:133709333-133721145 (+) |  | -0.316 | 4.82e-05 | 6.39e-04 |
| ENSMUSG00000003154 | Foxj2 | protein\_coding | 6:122819914-122845366 (+) |  | 0.580 | 4.84e-05 | 6.40e-04 |
| ENSMUSG00000041707 | Tmem273 | protein\_coding | 14:32785963-32817984 (+) |  | -0.746 | 4.88e-05 | 6.45e-04 |
| ENSMUSG00000031701 | Dnaja2 | protein\_coding | 8:85537633-85555344 (-) |  | -0.266 | 4.89e-05 | 6.46e-04 |
| ENSMUSG00000030521 | Mphosph10 | protein\_coding | 7:64376527-64392268 (-) |  | -0.357 | 4.91e-05 | 6.48e-04 |
| ENSMUSG00000030867 | Plk1 | protein\_coding | 7:122159439-122169873 (+) |  | -0.312 | 4.92e-05 | 6.48e-04 |
| ENSMUSG00000032531 | Amotl2 | protein\_coding | 9:102716672-102733418 (+) |  | -0.923 | 4.92e-05 | 6.48e-04 |
| ENSMUSG00000041126 | H2az2 | protein\_coding | 11:6427229-6444443 (-) |  | 0.373 | 4.97e-05 | 6.52e-04 |
| ENSMUSG00000037295 | Ldlrap1 | protein\_coding | 4:134741554-134768024 (-) |  | 0.335 | 4.97e-05 | 6.52e-04 |
| ENSMUSG00000020571 | Pdia6 | protein\_coding | 12:17266545-17284770 (+) |  | -0.372 | 5.00e-05 | 6.56e-04 |
| ENSMUSG00000055633 | Zfp580 | protein\_coding | 7:5051538-5053723 (+) |  | 1.320 | 5.01e-05 | 6.57e-04 |
| ENSMUSG00000067194 | Eif1ax | protein\_coding | X:159372178-159389928 (+) |  | -0.357 | 5.02e-05 | 6.58e-04 |
| ENSMUSG00000058809 | Hspd1-ps3 | processed\_pseudogene | 11:41498737-41500458 (+) |  | -0.648 | 5.07e-05 | 6.62e-04 |
| ENSMUSG00000075702 | Selenom | protein\_coding | 11:3514684-3517351 (+) |  | -0.622 | 5.07e-05 | 6.62e-04 |
| ENSMUSG00000052821 | Cysltr1 | protein\_coding | X:106574346-106603679 (-) |  | -0.709 | 5.08e-05 | 6.62e-04 |
| ENSMUSG00000033713 | Foxn3 | protein\_coding | 12:99190078-99563582 (-) |  | 0.423 | 5.08e-05 | 6.62e-04 |
| ENSMUSG00000056888 | Glipr1 | protein\_coding | 10:111985448-112002631 (-) |  | 0.317 | 5.13e-05 | 6.69e-04 |
| ENSMUSG00000028330 | Ncbp1 | protein\_coding | 4:46138613-46172403 (+) |  | -0.274 | 5.17e-05 | 6.73e-04 |
| ENSMUSG00000016018 | Mtrex | protein\_coding | 13:112867418-112927398 (-) |  | -0.318 | 5.18e-05 | 6.73e-04 |
| ENSMUSG00000040723 | Rcsd1 | protein\_coding | 1:165646516-165709757 (-) |  | 0.220 | 5.22e-05 | 6.78e-04 |
| ENSMUSG00000013236 | Ptprs | protein\_coding | 17:56412426-56476483 (-) |  | 0.431 | 5.23e-05 | 6.79e-04 |
| ENSMUSG00000029217 | Tec | protein\_coding | 5:72755716-72868483 (-) |  | 0.503 | 5.29e-05 | 6.86e-04 |
| ENSMUSG00000019579 | Mydgf | protein\_coding | 17:56175744-56183920 (-) |  | -0.388 | 5.30e-05 | 6.86e-04 |
| ENSMUSG00000044018 | Mrpl50 | protein\_coding | 4:49512596-49521093 (-) |  | -0.319 | 5.33e-05 | 6.90e-04 |
| ENSMUSG00000042292 | Mrtfa | protein\_coding | 15:81012281-81190757 (-) |  | 0.331 | 5.35e-05 | 6.92e-04 |
| ENSMUSG00000026655 | Fam107b | protein\_coding | 2:3570488-3782142 (+) |  | 0.217 | 5.37e-05 | 6.93e-04 |
| ENSMUSG00000075232 | Amd1 | protein\_coding | 10:40287458-40302188 (-) |  | -0.281 | 5.38e-05 | 6.94e-04 |
| ENSMUSG00000039630 | Hnrnpu | protein\_coding | 1:178321108-178337797 (-) |  | -0.219 | 5.39e-05 | 6.94e-04 |
| ENSMUSG00000027778 | Ift80 | protein\_coding | 3:68892499-69004570 (-) |  | -0.456 | 5.39e-05 | 6.94e-04 |
| ENSMUSG00000074785 | Plxnc1 | protein\_coding | 10:94790866-94944835 (-) |  | 0.328 | 5.41e-05 | 6.96e-04 |
| ENSMUSG00000059714 | Flot1 | protein\_coding | 17:35823230-35832791 (+) |  | -0.324 | 5.47e-05 | 7.02e-04 |
| ENSMUSG00000009585 | Apobec3 | protein\_coding | 15:79891659-79915906 (+) |  | -0.264 | 5.48e-05 | 7.03e-04 |
| ENSMUSG00000005824 | Tnfsf14 | protein\_coding | 17:57189492-57194177 (-) |  | -0.523 | 5.63e-05 | 7.21e-04 |
| ENSMUSG00000060470 | Adgrg3 | protein\_coding | 8:95017692-95045250 (+) |  | 0.515 | 5.64e-05 | 7.22e-04 |
| ENSMUSG00000023307 | March5 | protein\_coding | 19:37207543-37222151 (+) |  | -0.304 | 5.65e-05 | 7.23e-04 |
| ENSMUSG00000062647 | Rpl7a | protein\_coding | 2:26910764-26913318 (+) |  | -0.304 | 5.71e-05 | 7.31e-04 |
| ENSMUSG00000048163 | Selplg | protein\_coding | 5:113818536-113832644 (-) |  | 0.231 | 5.72e-05 | 7.31e-04 |
| ENSMUSG00000034522 | Zfp395 | protein\_coding | 14:65358389-65398930 (+) |  | 0.558 | 5.79e-05 | 7.39e-04 |
| ENSMUSG00000031403 | Dkc1 | protein\_coding | X:75095854-75109777 (+) |  | -0.314 | 5.82e-05 | 7.42e-04 |
| ENSMUSG00000060032 | H2aj | protein\_coding | 6:136808244-136810074 (+) |  | 0.355 | 5.84e-05 | 7.43e-04 |
| ENSMUSG00000029059 | Prxl2b | protein\_coding | 4:154895504-154899135 (-) |  | 0.797 | 5.86e-05 | 7.45e-04 |
| ENSMUSG00000031129 | Slc9a9 | protein\_coding | 9:94669909-95230445 (+) |  | 0.625 | 5.89e-05 | 7.49e-04 |
| ENSMUSG00000021645 | Smn1 | protein\_coding | 13:100124852-100137690 (+) |  | -0.342 | 5.94e-05 | 7.54e-04 |
| ENSMUSG00000022391 | Rangap1 | protein\_coding | 15:81704248-81745530 (-) |  | -0.257 | 5.95e-05 | 7.55e-04 |
| ENSMUSG00000018008 | Cyth4 | protein\_coding | 15:78597047-78622019 (+) |  | 0.281 | 5.96e-05 | 7.55e-04 |
| ENSMUSG00000020775 | Mrpl38 | protein\_coding | 11:116131817-116138868 (-) |  | -0.412 | 6.02e-05 | 7.62e-04 |
| ENSMUSG00000034075 | Zdhhc5 | protein\_coding | 2:84687970-84715180 (-) |  | -0.248 | 6.10e-05 | 7.71e-04 |
| ENSMUSG00000028647 | Mycbp | protein\_coding | 4:123904832-123912269 (+) |  | -0.533 | 6.11e-05 | 7.71e-04 |
| ENSMUSG00000030079 | Ruvbl1 | protein\_coding | 6:88465409-88497572 (+) |  | -0.376 | 6.11e-05 | 7.71e-04 |
| ENSMUSG00000024081 | Cebpz | protein\_coding | 17:78919006-78937066 (-) |  | -0.282 | 6.13e-05 | 7.73e-04 |
| ENSMUSG00000029416 | Slc15a4 | protein\_coding | 5:127595664-127632897 (-) |  | 0.342 | 6.15e-05 | 7.75e-04 |
| ENSMUSG00000006412 | Pfdn2 | protein\_coding | 1:171345670-171359254 (+) |  | -0.372 | 6.24e-05 | 7.86e-04 |
| ENSMUSG00000003604 | Aven | protein\_coding | 2:112492964-112634573 (+) |  | -0.430 | 6.26e-05 | 7.87e-04 |
| ENSMUSG00000085181 | Gm12709 | lncRNA | 4:102967266-102989755 (-) |  | -1.580 | 6.28e-05 | 7.88e-04 |
| ENSMUSG00000034266 | Batf | protein\_coding | 12:85686669-85709087 (+) |  | -0.507 | 6.29e-05 | 7.88e-04 |
| ENSMUSG00000035297 | Cops4 | protein\_coding | 5:100518309-100547803 (+) |  | -0.287 | 6.29e-05 | 7.88e-04 |
| ENSMUSG00000004264 | Phb2 | protein\_coding | 6:124712336-124716950 (+) |  | -0.304 | 6.29e-05 | 7.88e-04 |
| ENSMUSG00000047866 | Lonp2 | protein\_coding | 8:86624043-86723873 (+) |  | -0.330 | 6.32e-05 | 7.90e-04 |
| ENSMUSG00000025870 | Arl10 | protein\_coding | 13:54575015-54581128 (+) |  | 0.443 | 6.40e-05 | 8.00e-04 |
| ENSMUSG00000007721 | Ccdc124 | protein\_coding | 8:70868227-70873935 (-) |  | -0.317 | 6.41e-05 | 8.01e-04 |
| ENSMUSG00000032915 | Adgre4 | protein\_coding | 17:55749984-55853662 (+) |  | 2.550 | 6.46e-05 | 8.06e-04 |
| ENSMUSG00000073968 | Trim68 | protein\_coding | 7:102677582-102687327 (-) |  | 0.669 | 6.52e-05 | 8.10e-04 |
| ENSMUSG00000033906 | Zdhhc15 | protein\_coding | X:104536969-104671064 (-) |  | 0.718 | 6.52e-05 | 8.10e-04 |
| ENSMUSG00000006498 | Ptbp1 | protein\_coding | 10:79854427-79864771 (+) |  | -0.267 | 6.52e-05 | 8.10e-04 |
| ENSMUSG00000078941 | Ak6 | protein\_coding | 13:100650979-100666359 (+) |  | -0.513 | 6.52e-05 | 8.10e-04 |
| ENSMUSG00000041607 | Mbp | protein\_coding | 18:82475146-82585637 (+) |  | 0.483 | 6.53e-05 | 8.10e-04 |
| ENSMUSG00000032336 | Nptn | protein\_coding | 9:58582240-58657955 (+) |  | 0.233 | 6.53e-05 | 8.10e-04 |
| ENSMUSG00000024579 | Pcyox1l | protein\_coding | 18:61696837-61707635 (-) |  | -0.341 | 6.54e-05 | 8.10e-04 |
| ENSMUSG00000039753 | Fbxl5 | protein\_coding | 5:43744615-43821638 (-) |  | -0.434 | 6.56e-05 | 8.12e-04 |
| ENSMUSG00000022710 | Usp7 | protein\_coding | 16:8689595-8792308 (-) |  | -0.233 | 6.57e-05 | 8.12e-04 |
| ENSMUSG00000074802 | Gas2l3 | protein\_coding | 10:89408823-89443967 (-) |  | 0.311 | 6.59e-05 | 8.14e-04 |
| ENSMUSG00000030335 | Mrpl51 | protein\_coding | 6:125191801-125196269 (+) |  | -0.286 | 6.60e-05 | 8.14e-04 |
| ENSMUSG00000048007 | Timm8a1 | protein\_coding | X:134537256-134541865 (-) |  | -0.441 | 6.63e-05 | 8.17e-04 |
| ENSMUSG00000020423 | Btg2 | protein\_coding | 1:134075170-134079120 (-) |  | 1.010 | 6.63e-05 | 8.17e-04 |
| ENSMUSG00000046598 | Bdh1 | protein\_coding | 16:31422280-31458901 (+) |  | -1.350 | 6.70e-05 | 8.25e-04 |
| ENSMUSG00000041736 | Tspo | protein\_coding | 15:83563592-83574203 (+) |  | -0.384 | 6.72e-05 | 8.26e-04 |
| ENSMUSG00000087060 | Eldr | lncRNA | 11:16935154-16951282 (-) |  | 0.953 | 6.73e-05 | 8.26e-04 |
| ENSMUSG00000030423 | Pop4 | protein\_coding | 7:38261996-38271423 (-) |  | -0.345 | 6.76e-05 | 8.30e-04 |
| ENSMUSG00000020859 | Spag9 | protein\_coding | 11:93996091-94126085 (+) |  | 0.311 | 6.84e-05 | 8.39e-04 |
| ENSMUSG00000042487 | Leo1 | protein\_coding | 9:75441524-75466432 (+) |  | -0.340 | 6.90e-05 | 8.46e-04 |
| ENSMUSG00000029570 | Lfng | protein\_coding | 5:140607320-140615545 (+) |  | -0.540 | 6.93e-05 | 8.48e-04 |
| ENSMUSG00000044452 | Zfp507 | protein\_coding | 7:35772343-35803003 (-) |  | 0.872 | 6.94e-05 | 8.49e-04 |
| ENSMUSG00000003072 | Atp5d | protein\_coding | 10:80138632-80145818 (+) |  | -0.292 | 6.96e-05 | 8.50e-04 |
| ENSMUSG00000031843 | Mphosph6 | protein\_coding | 8:117791645-117801943 (-) |  | -0.393 | 7.01e-05 | 8.55e-04 |
| ENSMUSG00000019122 | Ccl9 | protein\_coding | 11:83572919-83578636 (-) |  | -0.324 | 7.03e-05 | 8.58e-04 |
| ENSMUSG00000030089 | Slc41a3 | protein\_coding | 6:90604725-90646412 (+) |  | 0.793 | 7.08e-05 | 8.62e-04 |
| ENSMUSG00000020573 | Pik3cg | protein\_coding | 12:32173473-32208659 (-) |  | 0.283 | 7.11e-05 | 8.65e-04 |
| ENSMUSG00000069301 | H2ac11 | protein\_coding | 13:22042460-22042944 (-) |  | 0.906 | 7.12e-05 | 8.66e-04 |
| ENSMUSG00000032078 | Zpr1 | protein\_coding | 9:46273064-46282643 (+) |  | -0.325 | 7.18e-05 | 8.72e-04 |
| ENSMUSG00000070476 | Fam217b | protein\_coding | 2:178414524-178424428 (+) |  | -0.493 | 7.19e-05 | 8.73e-04 |
| ENSMUSG00000001288 | Rarg | protein\_coding | 15:102234938-102257517 (-) |  | 0.427 | 7.42e-05 | 9.00e-04 |
| ENSMUSG00000036898 | Zfp157 | protein\_coding | 5:138441468-138460694 (+) |  | 0.453 | 7.46e-05 | 9.04e-04 |
| ENSMUSG00000020901 | Pik3r5 | protein\_coding | 11:68432121-68497849 (+) |  | -0.349 | 7.48e-05 | 9.05e-04 |
| ENSMUSG00000020340 | Cyfip2 | protein\_coding | 11:46193850-46312859 (-) |  | -0.438 | 7.55e-05 | 9.13e-04 |
| ENSMUSG00000036825 | Ssx2ip | protein\_coding | 3:146404642-146440144 (+) |  | -0.453 | 7.71e-05 | 9.32e-04 |
| ENSMUSG00000017405 | Nek8 | protein\_coding | 11:78166106-78176675 (-) |  | 0.684 | 7.73e-05 | 9.34e-04 |
| ENSMUSG00000035236 | Scai | protein\_coding | 2:39066214-39190734 (-) |  | 0.607 | 7.77e-05 | 9.37e-04 |
| ENSMUSG00000022003 | Slc25a30 | protein\_coding | 14:75760117-75787037 (-) |  | -0.352 | 7.78e-05 | 9.38e-04 |
| ENSMUSG00000026229 | Psmd1 | protein\_coding | 1:86064387-86139151 (+) |  | -0.305 | 7.79e-05 | 9.38e-04 |
| ENSMUSG00000017428 | Psmd11 | protein\_coding | 11:80428615-80473248 (+) |  | -0.314 | 7.81e-05 | 9.39e-04 |
| ENSMUSG00000066721 | Zfp575 | protein\_coding | 7:24583838-24587641 (-) |  | 1.650 | 7.85e-05 | 9.43e-04 |
| ENSMUSG00000036817 | Sun1 | protein\_coding | 5:139200637-139249840 (+) |  | 0.305 | 7.92e-05 | 9.51e-04 |
| ENSMUSG00000047250 | Ptgs1 | protein\_coding | 2:36230426-36252272 (+) |  | 0.567 | 7.94e-05 | 9.52e-04 |
| ENSMUSG00000058728 | Cd300c | protein\_coding | 11:114956116-114969157 (-) |  | 1.040 | 7.98e-05 | 9.56e-04 |
| ENSMUSG00000042594 | Sh2b3 | protein\_coding | 5:121815488-121837646 (-) |  | 0.292 | 7.99e-05 | 9.56e-04 |
| ENSMUSG00000033446 | Lpar6 | protein\_coding | 14:73237895-73243294 (+) |  | 0.415 | 8.01e-05 | 9.58e-04 |
| ENSMUSG00000099875 | Rbm3-ps | processed\_pseudogene | 1:150265535-150265999 (-) |  | 0.414 | 8.04e-05 | 9.61e-04 |
| ENSMUSG00000041598 | Cdc42ep4 | protein\_coding | 11:113726850-113751881 (-) |  | 0.421 | 8.07e-05 | 9.62e-04 |
| ENSMUSG00000105987 | AI506816 | lncRNA | 5:23698296-23712667 (-) |  | -0.424 | 8.07e-05 | 9.62e-04 |
| ENSMUSG00000045404 | Kcnk13 | protein\_coding | 12:99964499-100062682 (+) |  | -1.300 | 8.12e-05 | 9.67e-04 |
| ENSMUSG00000061315 | Naca | protein\_coding | 10:128035575-128048637 (+) |  | -0.286 | 8.14e-05 | 9.69e-04 |
| ENSMUSG00000014859 | E2f4 | protein\_coding | 8:105297663-105305370 (+) |  | -0.263 | 8.26e-05 | 9.83e-04 |
| ENSMUSG00000040848 | Sft2d2 | protein\_coding | 1:165174337-165194438 (-) |  | 0.339 | 8.32e-05 | 9.89e-04 |
| ENSMUSG00000030357 | Fkbp4 | protein\_coding | 6:128429735-128438677 (-) |  | -0.427 | 8.36e-05 | 9.93e-04 |
| ENSMUSG00000029701 | Rbm28 | protein\_coding | 6:29123576-29165006 (-) |  | -0.290 | 8.49e-05 | 1.01e-03 |
| ENSMUSG00000023947 | Nfkbie | protein\_coding | 17:45555703-45563169 (+) |  | 0.717 | 8.51e-05 | 1.01e-03 |
| ENSMUSG00000042570 | Mier2 | protein\_coding | 10:79540245-79555199 (-) |  | 0.359 | 8.61e-05 | 1.02e-03 |
| ENSMUSG00000032373 | Car12 | protein\_coding | 9:66713686-66766845 (+) |  | -1.130 | 8.62e-05 | 1.02e-03 |
| ENSMUSG00000021771 | Vdac2 | protein\_coding | 14:21825238-21845879 (+) |  | -0.262 | 8.65e-05 | 1.02e-03 |
| ENSMUSG00000030474 | Siglece | protein\_coding | 7:43651070-43660161 (-) |  | -0.365 | 8.66e-05 | 1.02e-03 |
| ENSMUSG00000033933 | Vhl | protein\_coding | 6:113623959-113631633 (+) |  | 0.337 | 8.67e-05 | 1.02e-03 |
| ENSMUSG00000031826 | Usp10 | protein\_coding | 8:119910360-119957560 (+) |  | -0.298 | 8.69e-05 | 1.02e-03 |
| ENSMUSG00000091021 | Gm17300 | lncRNA | 4:132351772-132353382 (+) |  | -0.643 | 8.72e-05 | 1.03e-03 |
| ENSMUSG00000018770 | Atp5g3 | protein\_coding | 2:73908447-73911326 (-) |  | -0.318 | 8.76e-05 | 1.03e-03 |
| ENSMUSG00000021708 | Rasgrf2 | protein\_coding | 13:91880400-92131656 (-) |  | 0.913 | 8.87e-05 | 1.04e-03 |
| ENSMUSG00000001627 | Ifrd1 | protein\_coding | 12:40201567-40248504 (-) |  | -0.402 | 8.90e-05 | 1.05e-03 |
| ENSMUSG00000066491 | Cox6c2 | processed\_pseudogene | 12:56373593-56373823 (-) |  | 1.200 | 8.98e-05 | 1.05e-03 |
| ENSMUSG00000021079 | Timm9 | protein\_coding | 12:71123173-71136684 (-) |  | -0.453 | 9.11e-05 | 1.07e-03 |
| ENSMUSG00000029610 | Aimp2 | protein\_coding | 5:143902704-143909847 (-) |  | -0.408 | 9.14e-05 | 1.07e-03 |
| ENSMUSG00000064147 | Rab44 | protein\_coding | 17:29114145-29148980 (+) |  | 0.343 | 9.17e-05 | 1.07e-03 |
| ENSMUSG00000109715 | Gm45606 | lncRNA | 11:75530277-75533071 (-) |  | 0.757 | 9.17e-05 | 1.07e-03 |
| ENSMUSG00000021213 | Akr1c13 | protein\_coding | 13:4191150-4205596 (+) |  | 0.612 | 9.32e-05 | 1.09e-03 |
| ENSMUSG00000025001 | Hells | protein\_coding | 19:38930915-38971051 (+) |  | -0.346 | 9.41e-05 | 1.10e-03 |
| ENSMUSG00000029642 | Polr1d | protein\_coding | 5:147077050-147111597 (+) |  | -0.381 | 9.42e-05 | 1.10e-03 |
| ENSMUSG00000019873 | Reep3 | protein\_coding | 10:67009189-67096945 (-) |  | 0.404 | 9.51e-05 | 1.11e-03 |
| ENSMUSG00000041642 | Kif21b | protein\_coding | 1:136131389-136177998 (+) |  | 0.373 | 9.62e-05 | 1.12e-03 |
| ENSMUSG00000071533 | Pcnp | protein\_coding | 16:56007245-56029739 (-) |  | -0.342 | 9.86e-05 | 1.15e-03 |
| ENSMUSG00000019814 | Ltv1 | protein\_coding | 10:13178140-13193168 (-) |  | -0.280 | 9.98e-05 | 1.16e-03 |
| ENSMUSG00000021728 | Emb | protein\_coding | 13:117208536-117274415 (+) |  | -0.241 | 9.98e-05 | 1.16e-03 |
| ENSMUSG00000028322 | Exosc3 | protein\_coding | 4:45316613-45342732 (-) |  | -0.296 | 9.99e-05 | 1.16e-03 |
| ENSMUSG00000016257 | Prelid3b | protein\_coding | 2:174465067-174473081 (-) |  | -0.330 | 1.01e-04 | 1.17e-03 |
| ENSMUSG00000029471 | Camkk2 | protein\_coding | 5:122731170-122779409 (-) |  | 0.417 | 1.01e-04 | 1.17e-03 |
| ENSMUSG00000039361 | Picalm | protein\_coding | 7:90130213-90213465 (+) |  | -0.364 | 1.02e-04 | 1.17e-03 |
| ENSMUSG00000024997 | Prdx3 | protein\_coding | 19:60864051-60874556 (-) |  | -0.321 | 1.02e-04 | 1.18e-03 |
| ENSMUSG00000056076 | Eif3b | protein\_coding | 5:140419328-140443360 (+) |  | -0.317 | 1.03e-04 | 1.18e-03 |
| ENSMUSG00000052456 | Asna1 | protein\_coding | 8:85017931-85025281 (-) |  | -0.350 | 1.03e-04 | 1.18e-03 |
| ENSMUSG00000031659 | Adcy7 | protein\_coding | 8:88272403-88329962 (+) |  | 0.241 | 1.03e-04 | 1.18e-03 |
| ENSMUSG00000044864 | Ankrd50 | protein\_coding | 3:38449259-38484844 (-) |  | 0.383 | 1.03e-04 | 1.19e-03 |
| ENSMUSG00000021238 | Aldh6a1 | protein\_coding | 12:84430717-84451004 (-) |  | 0.569 | 1.04e-04 | 1.19e-03 |
| ENSMUSG00000023909 | Paqr4 | protein\_coding | 17:23736186-23740867 (-) |  | 0.537 | 1.05e-04 | 1.20e-03 |
| ENSMUSG00000052997 | Uba2 | protein\_coding | 7:34140688-34169599 (-) |  | -0.277 | 1.05e-04 | 1.21e-03 |
| ENSMUSG00000050138 | Kcnk12 | protein\_coding | 17:87745801-87797994 (-) |  | 0.445 | 1.05e-04 | 1.21e-03 |
| ENSMUSG00000027130 | Slc12a6 | protein\_coding | 2:112265825-112363163 (+) |  | 0.326 | 1.06e-04 | 1.22e-03 |
| ENSMUSG00000003955 | Fam162a | protein\_coding | 16:36043761-36071594 (-) |  | -0.477 | 1.07e-04 | 1.22e-03 |
| ENSMUSG00000053398 | Phgdh | protein\_coding | 3:98313170-98339990 (-) |  | -0.452 | 1.07e-04 | 1.23e-03 |
| ENSMUSG00000020346 | Mgat1 | protein\_coding | 11:49244191-49263030 (+) |  | 0.278 | 1.08e-04 | 1.23e-03 |
| ENSMUSG00000060802 | B2m | protein\_coding | 2:122147686-122153083 (+) |  | -0.250 | 1.08e-04 | 1.24e-03 |
| ENSMUSG00000029428 | Stx2 | protein\_coding | 5:128984557-129008574 (-) |  | 0.418 | 1.09e-04 | 1.24e-03 |
| ENSMUSG00000028453 | Fancg | protein\_coding | 4:43002343-43010506 (-) |  | 0.330 | 1.09e-04 | 1.25e-03 |
| ENSMUSG00000024067 | Dpy30 | protein\_coding | 17:74299474-74323944 (-) |  | -0.315 | 1.09e-04 | 1.25e-03 |
| ENSMUSG00000005683 | Cs | protein\_coding | 10:128337734-128362479 (+) |  | -0.238 | 1.09e-04 | 1.25e-03 |
| ENSMUSG00000063694 | Cycs | protein\_coding | 6:50562563-50566538 (-) |  | -0.436 | 1.10e-04 | 1.25e-03 |
| ENSMUSG00000026822 | Lcn2 | protein\_coding | 2:32384633-32388252 (-) |  | -1.340 | 1.10e-04 | 1.25e-03 |
| ENSMUSG00000032407 | U2surp | protein\_coding | 9:95456898-95511996 (-) |  | -0.257 | 1.11e-04 | 1.26e-03 |
| ENSMUSG00000016496 | Cd274 | protein\_coding | 19:29367455-29388095 (+) |  | 0.902 | 1.11e-04 | 1.26e-03 |
| ENSMUSG00000051391 | Ywhag | protein\_coding | 5:135908409-135934616 (-) |  | -0.220 | 1.11e-04 | 1.26e-03 |
| ENSMUSG00000028261 | Ndufaf4 | protein\_coding | 4:24898083-24905001 (+) |  | -0.416 | 1.12e-04 | 1.26e-03 |
| ENSMUSG00000027589 | Pcmtd2 | protein\_coding | 2:181837854-181857461 (+) |  | 0.389 | 1.12e-04 | 1.27e-03 |
| ENSMUSG00000022906 | Parp9 | protein\_coding | 16:35938470-35972605 (+) |  | -0.341 | 1.14e-04 | 1.28e-03 |
| ENSMUSG00000026520 | Pycr2 | protein\_coding | 1:180904293-180908088 (+) |  | -0.276 | 1.14e-04 | 1.29e-03 |
| ENSMUSG00000038871 | Bpgm | protein\_coding | 6:34476207-34505613 (+) |  | 0.457 | 1.16e-04 | 1.31e-03 |
| ENSMUSG00000024338 | Psmb8 | protein\_coding | 17:34197721-34201454 (+) |  | -0.268 | 1.17e-04 | 1.32e-03 |
| ENSMUSG00000015597 | Zfp318 | protein\_coding | 17:46383731-46420920 (+) |  | 0.464 | 1.19e-04 | 1.34e-03 |
| ENSMUSG00000062545 | Tlr12 | protein\_coding | 4:128615443-128618619 (-) |  | 0.918 | 1.19e-04 | 1.34e-03 |
| ENSMUSG00000030830 | Itgal | protein\_coding | 7:127296260-127335138 (+) |  | 0.267 | 1.20e-04 | 1.35e-03 |
| ENSMUSG00000045658 | Pid1 | protein\_coding | 1:84036296-84364180 (-) |  | 0.732 | 1.20e-04 | 1.35e-03 |
| ENSMUSG00000022946 | Dop1b | protein\_coding | 16:93711904-93810590 (+) |  | 0.503 | 1.21e-04 | 1.36e-03 |
| ENSMUSG00000030738 | Eif3c | protein\_coding | 7:126546455-126566411 (-) |  | -0.245 | 1.21e-04 | 1.36e-03 |
| ENSMUSG00000003099 | Ppp5c | protein\_coding | 7:17004640-17027924 (-) |  | -0.267 | 1.22e-04 | 1.37e-03 |
| ENSMUSG00000054400 | Cklf | protein\_coding | 8:104250861-104264938 (+) |  | 0.449 | 1.23e-04 | 1.37e-03 |
| ENSMUSG00000015745 | Plekho1 | protein\_coding | 3:95988429-95996001 (-) |  | 0.325 | 1.23e-04 | 1.37e-03 |
| ENSMUSG00000066553 | Gm6969 | processed\_pseudogene | 12:24638669-24639010 (-) |  | 1.100 | 1.24e-04 | 1.38e-03 |
| ENSMUSG00000028333 | Anp32b | protein\_coding | 4:46450902-46472657 (+) |  | -0.250 | 1.24e-04 | 1.38e-03 |
| ENSMUSG00000057388 | Mrpl18 | protein\_coding | 17:12911349-12916345 (-) |  | -0.382 | 1.24e-04 | 1.39e-03 |
| ENSMUSG00000041836 | Ptpre | protein\_coding | 7:135537481-135686293 (+) |  | 0.345 | 1.25e-04 | 1.39e-03 |
| ENSMUSG00000000318 | Clec10a | protein\_coding | 11:70156197-70170834 (+) |  | 0.412 | 1.25e-04 | 1.39e-03 |
| ENSMUSG00000030884 | Uqcrc2 | protein\_coding | 7:120635176-120659524 (+) |  | -0.294 | 1.26e-04 | 1.40e-03 |
| ENSMUSG00000023908 | Pkmyt1 | protein\_coding | 17:23726336-23736735 (+) |  | 0.308 | 1.26e-04 | 1.40e-03 |
| ENSMUSG00000034833 | Tespa1 | protein\_coding | 10:130322870-130364111 (+) |  | 0.685 | 1.28e-04 | 1.42e-03 |
| ENSMUSG00000029147 | Ppm1g | protein\_coding | 5:31202664-31220687 (-) |  | -0.264 | 1.28e-04 | 1.42e-03 |
| ENSMUSG00000023110 | Prmt5 | protein\_coding | 14:54507187-54517525 (-) |  | -0.319 | 1.29e-04 | 1.43e-03 |
| ENSMUSG00000026112 | Coa5 | protein\_coding | 1:37417084-37430103 (-) |  | -0.241 | 1.30e-04 | 1.44e-03 |
| ENSMUSG00000027765 | P2ry1 | protein\_coding | 3:61002795-61008982 (+) |  | -0.482 | 1.30e-04 | 1.44e-03 |
| ENSMUSG00000009291 | Pttg1ip | protein\_coding | 10:77581720-77598732 (+) |  | 0.282 | 1.30e-04 | 1.44e-03 |
| ENSMUSG00000027496 | Aurka | protein\_coding | 2:172356190-172370535 (-) |  | -0.245 | 1.30e-04 | 1.44e-03 |
| ENSMUSG00000066861 | Oas1g | protein\_coding | 5:120876142-120887613 (-) |  | -1.580 | 1.31e-04 | 1.44e-03 |
| ENSMUSG00000031242 | 2610002M06Rik | protein\_coding | X:107782751-107816334 (-) |  | -0.375 | 1.31e-04 | 1.44e-03 |
| ENSMUSG00000069919 | Hba-a1 | protein\_coding | 11:32283511-32284465 (+) |  | 2.530 | 1.32e-04 | 1.45e-03 |
| ENSMUSG00000030579 | Tyrobp | protein\_coding | 7:30413760-30417585 (+) |  | 0.415 | 1.32e-04 | 1.45e-03 |
| ENSMUSG00000030062 | Rpn1 | protein\_coding | 6:88084482-88105304 (+) |  | -0.279 | 1.33e-04 | 1.47e-03 |
| ENSMUSG00000064210 | Ano6 | protein\_coding | 15:95790843-95974751 (+) |  | 0.294 | 1.34e-04 | 1.47e-03 |
| ENSMUSG00000032215 | Rsl24d1 | protein\_coding | 9:73113426-73123333 (+) |  | -0.268 | 1.34e-04 | 1.47e-03 |
| ENSMUSG00000036526 | Card11 | protein\_coding | 5:140872990-141000582 (-) |  | 0.933 | 1.35e-04 | 1.48e-03 |
| ENSMUSG00000061979 | Rcc1l | protein\_coding | 5:134148054-134176774 (-) |  | -0.477 | 1.35e-04 | 1.48e-03 |
| ENSMUSG00000033632 | AW554918 | protein\_coding | 18:25168999-25467321 (+) |  | 0.493 | 1.36e-04 | 1.49e-03 |
| ENSMUSG00000006281 | Tep1 | protein\_coding | 14:50824059-50870560 (-) |  | 0.306 | 1.36e-04 | 1.49e-03 |
| ENSMUSG00000035953 | Pip4p1 | protein\_coding | 14:50926068-50930856 (-) |  | 0.338 | 1.36e-04 | 1.49e-03 |
| ENSMUSG00000022111 | Uchl3 | protein\_coding | 14:101653967-101696125 (+) |  | -0.409 | 1.37e-04 | 1.50e-03 |
| ENSMUSG00000039126 | Prune2 | protein\_coding | 19:16956118-17223932 (+) |  | -0.761 | 1.38e-04 | 1.50e-03 |
| ENSMUSG00000047767 | Atg16l2 | protein\_coding | 7:101263034-101302251 (-) |  | 0.469 | 1.38e-04 | 1.51e-03 |
| ENSMUSG00000022438 | Parvb | protein\_coding | 15:84232043-84315688 (+) |  | 0.532 | 1.38e-04 | 1.51e-03 |
| ENSMUSG00000032333 | Stoml1 | protein\_coding | 9:58253164-58262520 (+) |  | 0.510 | 1.39e-04 | 1.52e-03 |
| ENSMUSG00000030203 | Dusp16 | protein\_coding | 6:134715468-134792625 (-) |  | 1.730 | 1.39e-04 | 1.52e-03 |
| ENSMUSG00000030835 | Nomo1 | protein\_coding | 7:46033698-46084212 (+) |  | -0.378 | 1.39e-04 | 1.52e-03 |
| ENSMUSG00000040950 | Mgl2 | protein\_coding | 11:70130329-70137550 (+) |  | 0.623 | 1.40e-04 | 1.52e-03 |
| ENSMUSG00000086291 | Gm15513 | lncRNA | 5:34211810-34213802 (-) |  | 2.000 | 1.40e-04 | 1.52e-03 |
| ENSMUSG00000006127 | Inpp5k | protein\_coding | 11:75630988-75648871 (+) |  | 0.292 | 1.40e-04 | 1.52e-03 |
| ENSMUSG00000029836 | Cbx3 | protein\_coding | 6:51470360-51483704 (+) |  | -0.284 | 1.42e-04 | 1.54e-03 |
| ENSMUSG00000061353 | Cxcl12 | protein\_coding | 6:117168535-117181367 (+) |  | 1.700 | 1.43e-04 | 1.55e-03 |
| ENSMUSG00000043702 | Pde12 | protein\_coding | 14:26659958-26669883 (-) |  | -0.287 | 1.44e-04 | 1.55e-03 |
| ENSMUSG00000060301 | 2610008E11Rik | protein\_coding | 10:79064374-79097600 (-) |  | 0.555 | 1.44e-04 | 1.56e-03 |
| ENSMUSG00000021127 | Zfp36l1 | protein\_coding | 12:80107754-80113013 (-) |  | -1.140 | 1.44e-04 | 1.56e-03 |
| ENSMUSG00000000628 | Hk2 | protein\_coding | 6:82725025-82774454 (-) |  | 0.259 | 1.46e-04 | 1.57e-03 |
| ENSMUSG00000037548 | H2-DMb2 | protein\_coding | 17:34143307-34151555 (+) |  | 0.575 | 1.46e-04 | 1.57e-03 |
| ENSMUSG00000052749 | Trim30b | protein\_coding | 7:104355382-104369884 (-) |  | -1.020 | 1.48e-04 | 1.59e-03 |
| ENSMUSG00000027599 | Armc1 | protein\_coding | 3:19131402-19163065 (-) |  | -0.288 | 1.49e-04 | 1.61e-03 |
| ENSMUSG00000028902 | Sf3a3 | protein\_coding | 4:124714776-124732460 (+) |  | -0.253 | 1.50e-04 | 1.62e-03 |
| ENSMUSG00000052033 | Pfdn4 | protein\_coding | 2:170496428-170519123 (+) |  | -0.566 | 1.52e-04 | 1.64e-03 |
| ENSMUSG00000086922 | Gm13835 | processed\_pseudogene | 6:31141979-31142663 (-) |  | -0.361 | 1.53e-04 | 1.64e-03 |
| ENSMUSG00000038366 | Lasp1 | protein\_coding | 11:97799000-97838764 (+) |  | 0.256 | 1.53e-04 | 1.65e-03 |
| ENSMUSG00000034765 | Dusp5 | protein\_coding | 19:53529109-53542431 (+) |  | 0.998 | 1.54e-04 | 1.65e-03 |
| ENSMUSG00000029311 | Hsd17b11 | protein\_coding | 5:103989762-104021919 (-) |  | 0.231 | 1.55e-04 | 1.66e-03 |
| ENSMUSG00000040663 | Clcf1 | protein\_coding | 19:4214238-4223490 (+) |  | 1.080 | 1.56e-04 | 1.67e-03 |
| ENSMUSG00000032652 | Crebl2 | protein\_coding | 6:134830154-134858931 (+) |  | 0.704 | 1.57e-04 | 1.68e-03 |
| ENSMUSG00000055491 | Pprc1 | protein\_coding | 19:46044886-46072915 (+) |  | -0.281 | 1.58e-04 | 1.69e-03 |
| ENSMUSG00000022673 | Mcm4 | protein\_coding | 16:15623897-15637400 (-) |  | -0.264 | 1.58e-04 | 1.69e-03 |
| ENSMUSG00000027206 | Cops2 | protein\_coding | 2:125830304-125859139 (-) |  | -0.331 | 1.60e-04 | 1.70e-03 |
| ENSMUSG00000026385 | Dbi | protein\_coding | 1:120113280-120121078 (-) |  | -0.318 | 1.60e-04 | 1.70e-03 |
| ENSMUSG00000074781 | Ube2n | protein\_coding | 10:95515145-95545657 (+) |  | -0.439 | 1.60e-04 | 1.70e-03 |
| ENSMUSG00000040383 | Aqr | protein\_coding | 2:114101170-114187024 (-) |  | -0.253 | 1.60e-04 | 1.71e-03 |
| ENSMUSG00000063785 | Utp14a | protein\_coding | X:48256862-48282453 (+) |  | -0.301 | 1.61e-04 | 1.71e-03 |
| ENSMUSG00000035722 | Abca7 | protein\_coding | 10:79996494-80015572 (+) |  | 0.261 | 1.61e-04 | 1.71e-03 |
| ENSMUSG00000044224 | Dnajc21 | protein\_coding | 15:10446756-10470516 (-) |  | -0.348 | 1.61e-04 | 1.71e-03 |
| ENSMUSG00000028378 | Ptgr1 | protein\_coding | 4:58965439-58987119 (-) |  | -0.258 | 1.62e-04 | 1.72e-03 |
| ENSMUSG00000025422 | Agap2 | protein\_coding | 10:127075284-127093169 (+) |  | 0.344 | 1.62e-04 | 1.72e-03 |
| ENSMUSG00000063406 | Tmed5 | protein\_coding | 5:108106366-108132620 (-) |  | -0.355 | 1.63e-04 | 1.72e-03 |
| ENSMUSG00000032265 | Tent5a | protein\_coding | 9:85320439-85327348 (-) |  | 0.274 | 1.64e-04 | 1.73e-03 |
| ENSMUSG00000003873 | Bax | protein\_coding | 7:45461697-45466898 (-) |  | -0.311 | 1.64e-04 | 1.73e-03 |
| ENSMUSG00000027374 | Mrps5 | protein\_coding | 2:127587222-127606829 (+) |  | -0.373 | 1.64e-04 | 1.73e-03 |
| ENSMUSG00000037465 | Klf10 | protein\_coding | 15:38291463-38300706 (-) |  | 0.381 | 1.64e-04 | 1.73e-03 |
| ENSMUSG00000000759 | Tubgcp3 | protein\_coding | 8:12614277-12672248 (-) |  | -0.238 | 1.64e-04 | 1.73e-03 |
| ENSMUSG00000026526 | Fh1 | protein\_coding | 1:175600374-175625635 (-) |  | -0.286 | 1.65e-04 | 1.74e-03 |
| ENSMUSG00000010663 | Fads1 | protein\_coding | 19:10182888-10196877 (+) |  | -0.418 | 1.67e-04 | 1.76e-03 |
| ENSMUSG00000024413 | Npc1 | protein\_coding | 18:12189692-12236400 (-) |  | 0.290 | 1.68e-04 | 1.77e-03 |
| ENSMUSG00000022708 | Zbtb20 | protein\_coding | 16:42875881-43642602 (+) |  | 0.871 | 1.70e-04 | 1.79e-03 |
| ENSMUSG00000079884 | Gm10698 | processed\_pseudogene | 9:33728247-33728850 (+) |  | -0.401 | 1.70e-04 | 1.79e-03 |
| ENSMUSG00000033444 | Specc1l | protein\_coding | 10:75212073-75312743 (+) |  | 0.314 | 1.70e-04 | 1.79e-03 |
| ENSMUSG00000076441 | Ass1 | protein\_coding | 2:31470207-31520672 (+) |  | -0.322 | 1.71e-04 | 1.80e-03 |
| ENSMUSG00000060073 | Psma3 | protein\_coding | 12:70974621-70996347 (+) |  | -0.332 | 1.72e-04 | 1.80e-03 |
| ENSMUSG00000033161 | Atp1a1 | protein\_coding | 3:101576219-101604684 (-) |  | -0.254 | 1.73e-04 | 1.81e-03 |
| ENSMUSG00000031066 | Usp11 | protein\_coding | X:20703906-20720539 (+) |  | 0.539 | 1.73e-04 | 1.81e-03 |
| ENSMUSG00000036305 | Rpl39-ps | processed\_pseudogene | 15:102635038-102635188 (+) |  | 0.430 | 1.73e-04 | 1.81e-03 |
| ENSMUSG00000018363 | Smurf2 | protein\_coding | 11:106820066-106920715 (-) |  | 0.250 | 1.74e-04 | 1.82e-03 |
| ENSMUSG00000097328 | Tnfsf12 | protein\_coding | 11:69686250-69695849 (-) |  | 0.879 | 1.74e-04 | 1.82e-03 |
| ENSMUSG00000024583 | Txnl1 | protein\_coding | 18:63661094-63708801 (-) |  | -0.373 | 1.75e-04 | 1.82e-03 |
| ENSMUSG00000004105 | Angptl2 | protein\_coding | 2:33216069-33247717 (+) |  | 0.511 | 1.75e-04 | 1.82e-03 |
| ENSMUSG00000032855 | Pkd1 | protein\_coding | 17:24549834-24596508 (+) |  | 0.375 | 1.76e-04 | 1.83e-03 |
| ENSMUSG00000002797 | Ggct | protein\_coding | 6:54982580-54992950 (-) |  | -0.599 | 1.76e-04 | 1.84e-03 |
| ENSMUSG00000038893 | Fam117a | protein\_coding | 11:95337018-95381872 (+) |  | 0.270 | 1.77e-04 | 1.84e-03 |
| ENSMUSG00000018548 | Trim37 | protein\_coding | 11:87127077-87220683 (+) |  | -0.266 | 1.79e-04 | 1.86e-03 |
| ENSMUSG00000025817 | Nudt5 | protein\_coding | 2:5845019-5871895 (+) |  | -0.363 | 1.81e-04 | 1.88e-03 |
| ENSMUSG00000062070 | Pgk1 | protein\_coding | X:106187100-106203699 (+) |  | -0.291 | 1.83e-04 | 1.90e-03 |
| ENSMUSG00000038486 | Sv2a | protein\_coding | 3:96181151-96195521 (+) |  | 1.080 | 1.84e-04 | 1.91e-03 |
| ENSMUSG00000033629 | Hacd3 | protein\_coding | 9:64986983-65021693 (-) |  | -0.380 | 1.84e-04 | 1.91e-03 |
| ENSMUSG00000001016 | Ilf2 | protein\_coding | 3:90476126-90488379 (+) |  | -0.263 | 1.84e-04 | 1.91e-03 |
| ENSMUSG00000060860 | Ube2s | protein\_coding | 7:4794546-4812590 (-) |  | -0.342 | 1.85e-04 | 1.92e-03 |
| ENSMUSG00000026019 | Wdr12 | protein\_coding | 1:60069785-60098645 (-) |  | -0.312 | 1.88e-04 | 1.94e-03 |
| ENSMUSG00000035967 | Ints6l | protein\_coding | X:56454857-56507843 (+) |  | 0.419 | 1.88e-04 | 1.94e-03 |
| ENSMUSG00000026974 | Zmynd19 | protein\_coding | 2:24949792-24962075 (+) |  | -0.278 | 1.90e-04 | 1.96e-03 |
| ENSMUSG00000020576 | Nbas | protein\_coding | 12:13269133-13583811 (+) |  | -0.284 | 1.91e-04 | 1.97e-03 |
| ENSMUSG00000041560 | Nop53 | protein\_coding | 7:15936183-15946074 (-) |  | 0.286 | 1.92e-04 | 1.97e-03 |
| ENSMUSG00000028434 | Epb41l4b | protein\_coding | 4:56991972-57143437 (-) |  | 0.648 | 1.92e-04 | 1.97e-03 |
| ENSMUSG00000020537 | Drg2 | protein\_coding | 11:60454591-60468754 (+) |  | -0.304 | 1.92e-04 | 1.98e-03 |
| ENSMUSG00000046897 | Zfp740 | protein\_coding | 15:102203249-102215606 (+) |  | 0.241 | 1.93e-04 | 1.98e-03 |
| ENSMUSG00000001761 | Smo | protein\_coding | 6:29735503-29761365 (+) |  | -0.545 | 1.94e-04 | 1.99e-03 |
| ENSMUSG00000046718 | Bst2 | protein\_coding | 8:71534255-71537456 (-) |  | -0.408 | 1.95e-04 | 2.00e-03 |
| ENSMUSG00000020413 | Hus1 | protein\_coding | 11:8993137-9011191 (-) |  | -0.327 | 1.97e-04 | 2.02e-03 |
| ENSMUSG00000038759 | Nup205 | protein\_coding | 6:35177421-35247596 (+) |  | -0.241 | 1.97e-04 | 2.02e-03 |
| ENSMUSG00000070544 | Top1 | protein\_coding | 2:160645888-160722764 (+) |  | -0.289 | 1.97e-04 | 2.02e-03 |
| ENSMUSG00000114003 | Gm9616 | processed\_pseudogene | 13:3298887-3299667 (+) |  | 0.307 | 1.98e-04 | 2.03e-03 |
| ENSMUSG00000032594 | Ip6k1 | protein\_coding | 9:108002501-108048782 (+) |  | 0.281 | 2.01e-04 | 2.05e-03 |
| ENSMUSG00000043207 | Zmpste24 | protein\_coding | 4:121059237-121098241 (-) |  | -0.247 | 2.01e-04 | 2.05e-03 |
| ENSMUSG00000026807 | Ak8 | protein\_coding | 2:28700164-28813165 (+) |  | 0.999 | 2.03e-04 | 2.07e-03 |
| ENSMUSG00000025968 | Ndufs1 | protein\_coding | 1:63143596-63176833 (-) |  | -0.286 | 2.04e-04 | 2.08e-03 |
| ENSMUSG00000022477 | Aco2 | protein\_coding | 15:81872309-81915133 (+) |  | -0.294 | 2.04e-04 | 2.08e-03 |
| ENSMUSG00000025047 | Pdcd11 | protein\_coding | 19:47090768-47131865 (+) |  | -0.274 | 2.06e-04 | 2.09e-03 |
| ENSMUSG00000018001 | Cyth3 | protein\_coding | 5:143622447-143710250 (+) |  | 0.612 | 2.07e-04 | 2.10e-03 |
| ENSMUSG00000029780 | Nt5c3 | protein\_coding | 6:56882400-56923932 (-) |  | -0.410 | 2.07e-04 | 2.10e-03 |
| ENSMUSG00000042784 | Muc1 | protein\_coding | 3:89229057-89233381 (+) |  | -2.640 | 2.08e-04 | 2.11e-03 |
| ENSMUSG00000028581 | Laptm5 | protein\_coding | 4:130913125-130936141 (+) |  | 0.250 | 2.10e-04 | 2.13e-03 |
| ENSMUSG00000024769 | Cdc42bpg | protein\_coding | 19:6306456-6325652 (+) |  | 0.643 | 2.10e-04 | 2.13e-03 |
| ENSMUSG00000050953 | Gja1 | protein\_coding | 10:56377330-56402513 (+) |  | -1.430 | 2.11e-04 | 2.14e-03 |
| ENSMUSG00000041438 | Utp4 | protein\_coding | 8:106893636-106923088 (+) |  | -0.379 | 2.13e-04 | 2.15e-03 |
| ENSMUSG00000106895 | Gm4754 | processed\_pseudogene | 5:41342123-41342931 (+) |  | -0.899 | 2.13e-04 | 2.16e-03 |
| ENSMUSG00000041912 | Tdrkh | protein\_coding | 3:94413273-94434668 (+) |  | 0.651 | 2.16e-04 | 2.18e-03 |
| ENSMUSG00000074358 | Ccdc61 | protein\_coding | 7:18890883-18910415 (-) |  | 0.415 | 2.17e-04 | 2.19e-03 |
| ENSMUSG00000029594 | Rbm19 | protein\_coding | 5:120116465-120198981 (+) |  | -0.387 | 2.17e-04 | 2.19e-03 |
| ENSMUSG00000024084 | Qpct | protein\_coding | 17:79051906-79090378 (+) |  | 0.695 | 2.17e-04 | 2.19e-03 |
| ENSMUSG00000062794 | Zfp599 | protein\_coding | 9:22247430-22259895 (-) |  | -0.923 | 2.17e-04 | 2.19e-03 |
| ENSMUSG00000055447 | Cd47 | protein\_coding | 16:49800533-49915010 (+) |  | -0.230 | 2.17e-04 | 2.19e-03 |
| ENSMUSG00000056529 | Ptafr | protein\_coding | 4:132564067-132582683 (+) |  | 0.526 | 2.18e-04 | 2.19e-03 |
| ENSMUSG00000023992 | Trem2 | protein\_coding | 17:48346401-48354147 (+) |  | 0.458 | 2.18e-04 | 2.19e-03 |
| ENSMUSG00000031633 | Slc25a4 | protein\_coding | 8:46206797-46211284 (-) |  | -0.285 | 2.18e-04 | 2.19e-03 |
| ENSMUSG00000001376 | Vps50 | protein\_coding | 6:3498382-3603531 (+) |  | -0.321 | 2.20e-04 | 2.21e-03 |
| ENSMUSG00000002957 | Ap2a2 | protein\_coding | 7:141562173-141633011 (+) |  | -0.225 | 2.20e-04 | 2.21e-03 |
| ENSMUSG00000001741 | Il16 | protein\_coding | 7:83642825-83745726 (-) |  | 0.254 | 2.23e-04 | 2.24e-03 |
| ENSMUSG00000071644 | Eef1g | protein\_coding | 19:8967041-8978479 (+) |  | -0.334 | 2.23e-04 | 2.24e-03 |
| ENSMUSG00000025134 | Alyref | protein\_coding | 11:120592121-120598365 (-) |  | -0.339 | 2.24e-04 | 2.24e-03 |
| ENSMUSG00000061024 | Rrs1 | protein\_coding | 1:9545408-9547455 (+) |  | -0.290 | 2.24e-04 | 2.24e-03 |
| ENSMUSG00000066687 | Zbtb16 | protein\_coding | 9:48654297-48836222 (-) |  | 1.550 | 2.28e-04 | 2.28e-03 |
| ENSMUSG00000041220 | Elovl6 | protein\_coding | 3:129532355-129638495 (+) |  | -0.343 | 2.29e-04 | 2.28e-03 |
| ENSMUSG00000028455 | Stoml2 | protein\_coding | 4:43027690-43031710 (-) |  | -0.331 | 2.29e-04 | 2.29e-03 |
| ENSMUSG00000021266 | Wars | protein\_coding | 12:108860030-108894174 (-) |  | -0.309 | 2.30e-04 | 2.30e-03 |
| ENSMUSG00000038374 | Rbm8a | protein\_coding | 3:96629933-96633791 (+) |  | -0.245 | 2.31e-04 | 2.30e-03 |
| ENSMUSG00000057421 | Las1l | protein\_coding | X:95935335-95956962 (-) |  | -0.319 | 2.32e-04 | 2.31e-03 |
| ENSMUSG00000027309 | 4930402H24Rik | protein\_coding | 2:130706200-130906406 (-) |  | 0.434 | 2.32e-04 | 2.31e-03 |
| ENSMUSG00000035772 | Mrps2 | protein\_coding | 2:28468066-28471178 (+) |  | -0.271 | 2.33e-04 | 2.32e-03 |
| ENSMUSG00000109244 | Gm44751 | lncRNA | 7:88311478-88315864 (+) |  | -0.893 | 2.36e-04 | 2.35e-03 |
| ENSMUSG00000002477 | Snrpd1 | protein\_coding | 18:10617775-10642079 (+) |  | -0.317 | 2.40e-04 | 2.38e-03 |
| ENSMUSG00000037946 | Fgd3 | protein\_coding | 13:49261554-49320311 (-) |  | 0.411 | 2.40e-04 | 2.38e-03 |
| ENSMUSG00000117621 | Hspe1-rs1 | protein\_coding | 18:47112139-47112653 (+) |  | -0.624 | 2.41e-04 | 2.39e-03 |
| ENSMUSG00000032329 | Hmg20a | protein\_coding | 9:56418609-56496936 (+) |  | 0.317 | 2.42e-04 | 2.40e-03 |
| ENSMUSG00000045948 | Mrps12 | protein\_coding | 7:28739641-28741820 (-) |  | -0.377 | 2.45e-04 | 2.42e-03 |
| ENSMUSG00000043587 | Pxylp1 | protein\_coding | 9:96823336-96892669 (-) |  | 0.259 | 2.49e-04 | 2.47e-03 |
| ENSMUSG00000046027 | Stard5 | protein\_coding | 7:83631959-83653127 (+) |  | -0.340 | 2.50e-04 | 2.47e-03 |
| ENSMUSG00000070738 | Dgkd | protein\_coding | 1:87853287-87945180 (+) |  | 0.243 | 2.52e-04 | 2.49e-03 |
| ENSMUSG00000020402 | Vdac1 | protein\_coding | 11:52360860-52389397 (+) |  | -0.295 | 2.53e-04 | 2.49e-03 |
| ENSMUSG00000020923 | Ubtf | protein\_coding | 11:102304560-102319742 (-) |  | -0.209 | 2.54e-04 | 2.50e-03 |
| ENSMUSG00000032842 | Abcc10 | protein\_coding | 17:46303221-46328352 (-) |  | 0.683 | 2.56e-04 | 2.52e-03 |
| ENSMUSG00000024174 | Pot1b | protein\_coding | 17:55651951-55712628 (-) |  | 0.430 | 2.58e-04 | 2.53e-03 |
| ENSMUSG00000023452 | Pisd | protein\_coding | 5:32736301-32785646 (-) |  | 0.283 | 2.59e-04 | 2.54e-03 |
| ENSMUSG00000036086 | Zranb3 | protein\_coding | 1:127954184-128103047 (-) |  | 0.419 | 2.59e-04 | 2.55e-03 |
| ENSMUSG00000027514 | Zbp1 | protein\_coding | 2:173206612-173218923 (-) |  | -0.807 | 2.60e-04 | 2.56e-03 |
| ENSMUSG00000036661 | Dennd3 | protein\_coding | 15:73512560-73572242 (+) |  | 0.551 | 2.61e-04 | 2.56e-03 |
| ENSMUSG00000036398 | Ppp1r11 | protein\_coding | 17:36948356-36951741 (-) |  | -0.370 | 2.61e-04 | 2.56e-03 |
| ENSMUSG00000031232 | Magt1 | protein\_coding | X:105968084-106011906 (-) |  | -0.219 | 2.64e-04 | 2.59e-03 |
| ENSMUSG00000034353 | Ramp1 | protein\_coding | 1:91179822-91225196 (+) |  | 0.384 | 2.64e-04 | 2.59e-03 |
| ENSMUSG00000036948 | Map11 | protein\_coding | 5:138259656-138264033 (-) |  | 0.265 | 2.67e-04 | 2.61e-03 |
| ENSMUSG00000020180 | Snrpd3 | protein\_coding | 10:75517551-75537381 (+) |  | -0.329 | 2.68e-04 | 2.62e-03 |
| ENSMUSG00000052889 | Prkcb | protein\_coding | 7:122288751-122634402 (+) |  | 0.649 | 2.70e-04 | 2.64e-03 |
| ENSMUSG00000069114 | Zbtb10 | protein\_coding | 3:9250602-9285333 (+) |  | 1.060 | 2.72e-04 | 2.66e-03 |
| ENSMUSG00000032096 | Arcn1 | protein\_coding | 9:44741564-44767845 (-) |  | -0.223 | 2.73e-04 | 2.66e-03 |
| ENSMUSG00000030282 | Cmas | protein\_coding | 6:142756686-142775714 (+) |  | -0.306 | 2.74e-04 | 2.67e-03 |
| ENSMUSG00000021929 | Kpna3 | protein\_coding | 14:61365211-61439874 (-) |  | -0.309 | 2.74e-04 | 2.67e-03 |
| ENSMUSG00000025786 | Zdhhc3 | protein\_coding | 9:123066160-123113205 (-) |  | -0.213 | 2.75e-04 | 2.68e-03 |
| ENSMUSG00000059554 | Ccdc28a | protein\_coding | 10:18213676-18234998 (-) |  | 0.857 | 2.75e-04 | 2.68e-03 |
| ENSMUSG00000047264 | Zfp358 | protein\_coding | 8:3493138-3497208 (+) |  | 0.561 | 2.77e-04 | 2.69e-03 |
| ENSMUSG00000046658 | Zfp316 | protein\_coding | 5:143249697-143270022 (-) |  | 0.666 | 2.78e-04 | 2.70e-03 |
| ENSMUSG00000041362 | Shtn1 | protein\_coding | 19:58973356-59076100 (-) |  | -0.585 | 2.78e-04 | 2.70e-03 |
| ENSMUSG00000022419 | Deptor | protein\_coding | 15:55112317-55259271 (+) |  | 0.331 | 2.81e-04 | 2.72e-03 |
| ENSMUSG00000032883 | Acsl3 | protein\_coding | 1:78657825-78707743 (+) |  | -0.431 | 2.82e-04 | 2.73e-03 |
| ENSMUSG00000036944 | Tmem71 | protein\_coding | 15:66526212-66561103 (-) |  | 0.536 | 2.82e-04 | 2.73e-03 |
| ENSMUSG00000027035 | Cers6 | protein\_coding | 2:68861441-69114282 (+) |  | -0.310 | 2.83e-04 | 2.74e-03 |
| ENSMUSG00000026000 | Lancl1 | protein\_coding | 1:67000517-67038872 (-) |  | 0.404 | 2.83e-04 | 2.74e-03 |
| ENSMUSG00000117875 | Gm6789 | processed\_pseudogene | 18:63543751-63545135 (-) |  | 0.318 | 2.84e-04 | 2.74e-03 |
| ENSMUSG00000033400 | Agl | protein\_coding | 3:116739999-116808166 (-) |  | 0.298 | 2.85e-04 | 2.75e-03 |
| ENSMUSG00000059851 | Kmt5c | protein\_coding | 7:4740115-4747514 (+) |  | 0.327 | 2.86e-04 | 2.75e-03 |
| ENSMUSG00000038205 | Prkab2 | protein\_coding | 3:97658193-97673812 (+) |  | 0.476 | 2.86e-04 | 2.76e-03 |
| ENSMUSG00000052435 | Cebpe | protein\_coding | 14:54710360-54712174 (-) |  | 0.486 | 2.87e-04 | 2.76e-03 |
| ENSMUSG00000049866 | Arl4c | protein\_coding | 1:88673125-88702221 (-) |  | 0.501 | 2.90e-04 | 2.79e-03 |
| ENSMUSG00000036777 | Anln | protein\_coding | 9:22332012-22389188 (-) |  | -0.267 | 2.92e-04 | 2.81e-03 |
| ENSMUSG00000059981 | Taok2 | protein\_coding | 7:126865678-126884703 (-) |  | 0.221 | 2.97e-04 | 2.86e-03 |
| ENSMUSG00000103865 | Gm37416 | TEC | 2:16023108-16023425 (-) |  | -0.512 | 3.00e-04 | 2.88e-03 |
| ENSMUSG00000004535 | Tax1bp1 | protein\_coding | 6:52713729-52766780 (+) |  | -0.203 | 3.01e-04 | 2.89e-03 |
| ENSMUSG00000026357 | Rgs18 | protein\_coding | 1:144752683-144775435 (-) |  | 0.387 | 3.02e-04 | 2.89e-03 |
| ENSMUSG00000030512 | Snrpa1 | protein\_coding | 7:66059003-66074587 (+) |  | -0.327 | 3.02e-04 | 2.90e-03 |
| ENSMUSG00000030055 | Rab43 | protein\_coding | 6:87788853-87812164 (-) |  | 0.636 | 3.03e-04 | 2.90e-03 |
| ENSMUSG00000024270 | Slc39a6 | protein\_coding | 18:24579881-24603817 (-) |  | -0.255 | 3.03e-04 | 2.90e-03 |
| ENSMUSG00000031897 | Psmb10 | protein\_coding | 8:105935735-105938444 (-) |  | -0.221 | 3.04e-04 | 2.90e-03 |
| ENSMUSG00000053931 | Cnn3 | protein\_coding | 3:121426497-121458207 (+) |  | 0.539 | 3.09e-04 | 2.96e-03 |
| ENSMUSG00000021814 | Anxa7 | protein\_coding | 14:20455260-20480133 (-) |  | -0.280 | 3.10e-04 | 2.96e-03 |
| ENSMUSG00000054509 | Parp4 | protein\_coding | 14:56575619-56659794 (+) |  | 0.334 | 3.14e-04 | 2.99e-03 |
| ENSMUSG00000035824 | Tk2 | protein\_coding | 8:104226685-104248558 (-) |  | 0.440 | 3.14e-04 | 2.99e-03 |
| ENSMUSG00000064267 | Hvcn1 | protein\_coding | 5:122206804-122242297 (+) |  | 0.386 | 3.15e-04 | 3.00e-03 |
| ENSMUSG00000028587 | Orc1 | protein\_coding | 4:108579423-108614833 (+) |  | -0.396 | 3.18e-04 | 3.02e-03 |
| ENSMUSG00000026434 | Nucks1 | protein\_coding | 1:131910534-131936321 (+) |  | -0.264 | 3.18e-04 | 3.03e-03 |
| ENSMUSG00000060594 | Layn | protein\_coding | 9:51054640-51077094 (-) |  | 0.620 | 3.19e-04 | 3.03e-03 |
| ENSMUSG00000089917 | Uckl1 | protein\_coding | 2:181569149-181584892 (-) |  | 0.324 | 3.21e-04 | 3.05e-03 |
| ENSMUSG00000038295 | Atg9b | protein\_coding | 5:24384181-24392143 (-) |  | -0.627 | 3.21e-04 | 3.05e-03 |
| ENSMUSG00000035726 | Supt16 | protein\_coding | 14:52160414-52197416 (-) |  | -0.210 | 3.22e-04 | 3.05e-03 |
| ENSMUSG00000022106 | Rcbtb2 | protein\_coding | 14:73123037-73207843 (+) |  | 0.338 | 3.22e-04 | 3.05e-03 |
| ENSMUSG00000022565 | Plec | protein\_coding | 15:76170974-76232574 (-) |  | 0.307 | 3.22e-04 | 3.05e-03 |
| ENSMUSG00000019699 | Akt3 | protein\_coding | 1:177020073-177258203 (-) |  | 0.323 | 3.23e-04 | 3.06e-03 |
| ENSMUSG00000031749 | St3gal2 | protein\_coding | 8:110919922-110972480 (+) |  | 0.450 | 3.25e-04 | 3.07e-03 |
| ENSMUSG00000023286 | Ube2j2 | protein\_coding | 4:155943831-155959604 (+) |  | -0.241 | 3.27e-04 | 3.09e-03 |
| ENSMUSG00000076621 | Ighj1 | IG\_J\_gene | 12:113429781-113429833 (-) |  | 0.946 | 3.27e-04 | 3.09e-03 |
| ENSMUSG00000030122 | Ptms | protein\_coding | 6:124913681-124920103 (-) |  | 0.440 | 3.28e-04 | 3.10e-03 |
| ENSMUSG00000028896 | Rcc1 | protein\_coding | 4:132331919-132353605 (-) |  | -0.267 | 3.29e-04 | 3.11e-03 |
| ENSMUSG00000078238 | Gm12854 | processed\_pseudogene | 4:116067268-116067564 (+) |  | 0.460 | 3.31e-04 | 3.12e-03 |
| ENSMUSG00000002329 | Mdp1 | protein\_coding | 14:55657879-55660508 (-) |  | 0.348 | 3.32e-04 | 3.12e-03 |
| ENSMUSG00000017176 | Nt5c3b | protein\_coding | 11:100422321-100441808 (-) |  | -0.446 | 3.32e-04 | 3.12e-03 |
| ENSMUSG00000054675 | Tmem119 | protein\_coding | 5:113793729-113800516 (-) |  | 0.544 | 3.34e-04 | 3.14e-03 |
| ENSMUSG00000066150 | Slc31a1 | protein\_coding | 4:62360727-62391769 (+) |  | -0.248 | 3.35e-04 | 3.15e-03 |
| ENSMUSG00000030619 | Eed | protein\_coding | 7:89954654-89980983 (-) |  | -0.372 | 3.38e-04 | 3.18e-03 |
| ENSMUSG00000043419 | Rnf227 | protein\_coding | 11:69340461-69342641 (+) |  | 0.525 | 3.41e-04 | 3.20e-03 |
| ENSMUSG00000073147 | 5031425E22Rik | lncRNA | 5:23382308-23434269 (-) |  | 0.547 | 3.42e-04 | 3.21e-03 |
| ENSMUSG00000060950 | Trmt61a | protein\_coding | 12:111678105-111683902 (+) |  | -0.414 | 3.48e-04 | 3.26e-03 |
| ENSMUSG00000036882 | Arhgap33 | protein\_coding | 7:30522226-30535060 (-) |  | 0.574 | 3.52e-04 | 3.29e-03 |
| ENSMUSG00000067150 | Xpo5 | protein\_coding | 17:46202782-46243598 (+) |  | -0.294 | 3.52e-04 | 3.29e-03 |
| ENSMUSG00000044788 | Fads6 | protein\_coding | 11:115279622-115297663 (-) |  | 0.654 | 3.53e-04 | 3.29e-03 |
| ENSMUSG00000079111 | Kdelr2 | protein\_coding | 5:143403838-143421901 (+) |  | -0.269 | 3.53e-04 | 3.30e-03 |
| ENSMUSG00000021987 | Mtmr6 | protein\_coding | 14:60265228-60302370 (+) |  | 0.276 | 3.54e-04 | 3.30e-03 |
| ENSMUSG00000079057 | Cyp4v3 | protein\_coding | 8:45304944-45333216 (-) |  | 0.514 | 3.57e-04 | 3.33e-03 |
| ENSMUSG00000075014 | Gm10800 | protein\_coding | 2:98666547-98667301 (-) |  | 4.260 | 3.59e-04 | 3.35e-03 |
| ENSMUSG00000020697 | Lig3 | protein\_coding | 11:82781108-82804274 (+) |  | -0.303 | 3.63e-04 | 3.38e-03 |
| ENSMUSG00000029752 | Asns | protein\_coding | 6:7675169-7693254 (-) |  | -1.200 | 3.63e-04 | 3.38e-03 |
| ENSMUSG00000022788 | Fgd4 | protein\_coding | 16:16416917-16600549 (-) |  | 0.386 | 3.64e-04 | 3.38e-03 |
| ENSMUSG00000026926 | Pmpca | protein\_coding | 2:26389339-26397122 (+) |  | -0.242 | 3.66e-04 | 3.40e-03 |
| ENSMUSG00000050490 | Gm8394 | processed\_pseudogene | 10:85313488-85314439 (+) |  | -0.497 | 3.67e-04 | 3.41e-03 |
| ENSMUSG00000028015 | Ctso | protein\_coding | 3:81932601-81956725 (+) |  | 0.454 | 3.67e-04 | 3.41e-03 |
| ENSMUSG00000103041 | Gm37305 | lncRNA | 3:65957758-65962036 (+) |  | 0.605 | 3.67e-04 | 3.41e-03 |
| ENSMUSG00000031605 | Klhl2 | protein\_coding | 8:64739675-64850017 (-) |  | -0.341 | 3.68e-04 | 3.41e-03 |
| ENSMUSG00000050108 | Bpifc | protein\_coding | 10:85959340-86011895 (-) |  | 2.120 | 3.71e-04 | 3.43e-03 |
| ENSMUSG00000024726 | Carnmt1 | protein\_coding | 19:18670764-18707200 (+) |  | -0.433 | 3.71e-04 | 3.43e-03 |
| ENSMUSG00000016494 | Cd34 | protein\_coding | 1:194938819-194961279 (+) |  | 0.708 | 3.73e-04 | 3.45e-03 |
| ENSMUSG00000020577 | Tspan13 | protein\_coding | 12:36014557-36042500 (-) |  | -0.862 | 3.74e-04 | 3.46e-03 |
| ENSMUSG00000021710 | Nln | protein\_coding | 13:104023057-104109614 (-) |  | -0.281 | 3.79e-04 | 3.50e-03 |
| ENSMUSG00000025358 | Cdk2 | protein\_coding | 10:128697939-128705031 (-) |  | 0.224 | 3.81e-04 | 3.52e-03 |
| ENSMUSG00000022018 | Rgcc | protein\_coding | 14:79288756-79301645 (-) |  | -0.601 | 3.82e-04 | 3.52e-03 |
| ENSMUSG00000011832 | Evi5l | protein\_coding | 8:4166567-4211257 (+) |  | 0.710 | 3.83e-04 | 3.53e-03 |
| ENSMUSG00000043866 | Taf10 | protein\_coding | 7:105739393-105744361 (-) |  | -0.305 | 3.83e-04 | 3.53e-03 |
| ENSMUSG00000093904 | Tomm20 | protein\_coding | 8:126930667-126945844 (-) |  | -0.318 | 3.89e-04 | 3.58e-03 |
| ENSMUSG00000032834 | Pwp2 | protein\_coding | 10:78170909-78185149 (-) |  | -0.293 | 3.92e-04 | 3.60e-03 |
| ENSMUSG00000060600 | Eno3 | protein\_coding | 11:70657202-70662513 (+) |  | 0.672 | 3.94e-04 | 3.62e-03 |
| ENSMUSG00000062580 | Timm17a | protein\_coding | 1:135295213-135313778 (-) |  | -0.359 | 3.96e-04 | 3.64e-03 |
| ENSMUSG00000030105 | Arl8b | protein\_coding | 6:108783099-108825278 (+) |  | -0.209 | 3.98e-04 | 3.65e-03 |
| ENSMUSG00000030662 | Ipo5 | protein\_coding | 14:120911224-120947999 (+) |  | -0.237 | 3.99e-04 | 3.66e-03 |
| ENSMUSG00000005161 | Prdx2 | protein\_coding | 8:84969587-84974834 (+) |  | -0.295 | 4.00e-04 | 3.66e-03 |
| ENSMUSG00000027823 | Gmps | protein\_coding | 3:63976106-64022579 (+) |  | -0.264 | 4.01e-04 | 3.67e-03 |
| ENSMUSG00000039007 | Cpq | protein\_coding | 15:33083129-33594552 (+) |  | 0.397 | 4.01e-04 | 3.67e-03 |
| ENSMUSG00000029478 | Ncor2 | protein\_coding | 5:125017153-125179219 (-) |  | 0.371 | 4.03e-04 | 3.68e-03 |
| ENSMUSG00000014867 | Surf4 | protein\_coding | 2:26920040-26933928 (-) |  | -0.180 | 4.04e-04 | 3.69e-03 |
| ENSMUSG00000048329 | Mfsd6l | protein\_coding | 11:68556186-68558245 (+) |  | 0.891 | 4.05e-04 | 3.69e-03 |
| ENSMUSG00000038539 | Atf5 | protein\_coding | 7:44812256-44816658 (-) |  | -0.522 | 4.05e-04 | 3.69e-03 |
| ENSMUSG00000061111 | Mcrip1 | protein\_coding | 11:120542888-120549727 (-) |  | 0.331 | 4.07e-04 | 3.71e-03 |
| ENSMUSG00000026361 | Cdc73 | protein\_coding | 1:143598800-143702893 (-) |  | -0.241 | 4.08e-04 | 3.71e-03 |
| ENSMUSG00000006998 | Psmd2 | protein\_coding | 16:20651652-20663414 (+) |  | -0.267 | 4.08e-04 | 3.71e-03 |
| ENSMUSG00000020358 | Hnrnpab | protein\_coding | 11:51600100-51606847 (-) |  | -0.261 | 4.08e-04 | 3.71e-03 |
| ENSMUSG00000028088 | Fmo5 | protein\_coding | 3:97628804-97655282 (+) |  | 0.913 | 4.10e-04 | 3.72e-03 |
| ENSMUSG00000025403 | Shmt2 | protein\_coding | 10:127517123-127522444 (-) |  | -0.337 | 4.10e-04 | 3.73e-03 |
| ENSMUSG00000052724 | Gm9888 | lncRNA | 9:114780100-114782257 (+) |  | 0.600 | 4.12e-04 | 3.74e-03 |
| ENSMUSG00000028837 | Psmb2 | protein\_coding | 4:126677630-126709714 (+) |  | -0.264 | 4.13e-04 | 3.74e-03 |
| ENSMUSG00000045928 | 4933440M02Rik | lncRNA | 7:125284025-125349788 (-) |  | 0.959 | 4.16e-04 | 3.77e-03 |
| ENSMUSG00000060373 | Hnrnpc | protein\_coding | 14:52073377-52104028 (-) |  | -0.310 | 4.17e-04 | 3.78e-03 |
| ENSMUSG00000029486 | Mrpl1 | protein\_coding | 5:96209493-96266727 (+) |  | -0.356 | 4.17e-04 | 3.78e-03 |
| ENSMUSG00000078427 | Sarnp | protein\_coding | 10:128817333-128877629 (+) |  | -0.481 | 4.17e-04 | 3.78e-03 |
| ENSMUSG00000034892 | Rps29 | protein\_coding | 12:69157722-69159186 (-) |  | 0.272 | 4.19e-04 | 3.78e-03 |
| ENSMUSG00000021737 | Psmd6 | protein\_coding | 14:14112174-14120984 (-) |  | -0.274 | 4.20e-04 | 3.80e-03 |
| ENSMUSG00000004933 | Matk | protein\_coding | 10:81252935-81263365 (+) |  | 0.330 | 4.21e-04 | 3.80e-03 |
| ENSMUSG00000040272 | Accs | protein\_coding | 2:93833467-93849943 (-) |  | 0.485 | 4.21e-04 | 3.80e-03 |
| ENSMUSG00000027714 | Exosc9 | protein\_coding | 3:36552606-36565727 (+) |  | -0.342 | 4.22e-04 | 3.80e-03 |
| ENSMUSG00000028426 | Rad23b | protein\_coding | 4:55350043-55392237 (+) |  | -0.272 | 4.23e-04 | 3.81e-03 |
| ENSMUSG00000022387 | Brd1 | protein\_coding | 15:88687034-88734233 (-) |  | 0.221 | 4.24e-04 | 3.81e-03 |
| ENSMUSG00000097848 | Gm807 | lncRNA | 13:99100706-99107580 (+) |  | 1.050 | 4.24e-04 | 3.81e-03 |
| ENSMUSG00000025217 | Btrc | protein\_coding | 19:45363734-45530013 (+) |  | 0.433 | 4.24e-04 | 3.81e-03 |
| ENSMUSG00000015966 | Il17rb | protein\_coding | 14:29996135-30008896 (-) |  | 0.843 | 4.25e-04 | 3.82e-03 |
| ENSMUSG00000034210 | Efcab14 | protein\_coding | 4:115737744-115777327 (+) |  | -0.333 | 4.31e-04 | 3.87e-03 |
| ENSMUSG00000107383 | Gm4366 | processed\_pseudogene | 7:116824510-116825851 (-) |  | -0.358 | 4.31e-04 | 3.87e-03 |
| ENSMUSG00000033032 | Afap1l1 | protein\_coding | 18:61730261-61786702 (-) |  | 0.492 | 4.32e-04 | 3.87e-03 |
| ENSMUSG00000028648 | Ndufs5 | protein\_coding | 4:123712710-123718202 (-) |  | -0.338 | 4.33e-04 | 3.88e-03 |
| ENSMUSG00000020527 | Myo19 | protein\_coding | 11:84880148-84911226 (+) |  | -0.452 | 4.34e-04 | 3.88e-03 |
| ENSMUSG00000027452 | Acss1 | protein\_coding | 2:150618105-150668500 (-) |  | 0.401 | 4.34e-04 | 3.89e-03 |
| ENSMUSG00000026914 | Psmd14 | protein\_coding | 2:61711694-61800376 (+) |  | -0.291 | 4.35e-04 | 3.89e-03 |
| ENSMUSG00000057666 | Gapdh | protein\_coding | 6:125161715-125166467 (-) |  | -0.364 | 4.36e-04 | 3.89e-03 |
| ENSMUSG00000030138 | Bms1 | protein\_coding | 6:118383381-118419474 (-) |  | -0.277 | 4.38e-04 | 3.91e-03 |
| ENSMUSG00000018417 | Myo1b | protein\_coding | 1:51749765-51916071 (-) |  | -0.778 | 4.38e-04 | 3.91e-03 |
| ENSMUSG00000038299 | Wdr36 | protein\_coding | 18:32837225-32867594 (+) |  | -0.321 | 4.39e-04 | 3.92e-03 |
| ENSMUSG00000022136 | Dnajc3 | protein\_coding | 14:118937976-118981697 (+) |  | -0.238 | 4.40e-04 | 3.92e-03 |
| ENSMUSG00000020522 | Mfap3 | protein\_coding | 11:57518664-57533815 (+) |  | 0.252 | 4.40e-04 | 3.92e-03 |
| ENSMUSG00000039770 | Ypel5 | protein\_coding | 17:72836453-72851195 (+) |  | 0.343 | 4.40e-04 | 3.92e-03 |
| ENSMUSG00000034088 | Hdlbp | protein\_coding | 1:93405940-93478815 (-) |  | -0.250 | 4.43e-04 | 3.93e-03 |
| ENSMUSG00000020017 | Hal | protein\_coding | 10:93488768-93519304 (+) |  | 0.996 | 4.43e-04 | 3.94e-03 |
| ENSMUSG00000106099 | Gm42664 | lncRNA | 3:119743208-119746446 (-) |  | 0.737 | 4.43e-04 | 3.94e-03 |
| ENSMUSG00000042426 | Dhx29 | protein\_coding | 13:112927454-112969432 (+) |  | -0.280 | 4.45e-04 | 3.95e-03 |
| ENSMUSG00000016534 | Lamp2 | protein\_coding | X:38401357-38456454 (-) |  | -0.310 | 4.46e-04 | 3.95e-03 |
| ENSMUSG00000081137 | BC022960 | transcribed\_processed\_pseudogene | X:169368189-169370003 (+) |  | 0.956 | 4.48e-04 | 3.97e-03 |
| ENSMUSG00000041733 | Coq5 | protein\_coding | 5:115279666-115296972 (+) |  | -0.302 | 4.48e-04 | 3.97e-03 |
| ENSMUSG00000037355 | Uvssa | protein\_coding | 5:33378549-33419754 (+) |  | 0.426 | 4.49e-04 | 3.97e-03 |
| ENSMUSG00000018068 | Ints2 | protein\_coding | 11:86210681-86257575 (-) |  | -0.298 | 4.50e-04 | 3.98e-03 |
| ENSMUSG00000005312 | Ubqln1 | protein\_coding | 13:58176156-58215653 (-) |  | -0.215 | 4.51e-04 | 3.98e-03 |
| ENSMUSG00000063802 | Hspbp1 | protein\_coding | 7:4660521-4685068 (-) |  | -0.377 | 4.51e-04 | 3.99e-03 |
| ENSMUSG00000031924 | Cyb5b | protein\_coding | 8:107150640-107187471 (+) |  | -0.247 | 4.54e-04 | 4.01e-03 |
| ENSMUSG00000004698 | Hdac9 | protein\_coding | 12:34047580-34917095 (-) |  | 0.596 | 4.55e-04 | 4.01e-03 |
| ENSMUSG00000109324 | Prmt1 | protein\_coding | 7:44975989-44986568 (-) |  | -0.290 | 4.55e-04 | 4.01e-03 |
| ENSMUSG00000064043 | Trerf1 | lncRNA | 17:47140875-47361958 (+) |  | 0.345 | 4.55e-04 | 4.01e-03 |
| ENSMUSG00000032560 | Dnajc13 | protein\_coding | 9:104151282-104262930 (-) |  | -0.221 | 4.55e-04 | 4.01e-03 |
| ENSMUSG00000056536 | Pign | protein\_coding | 1:105518422-105663677 (-) |  | -0.272 | 4.61e-04 | 4.06e-03 |
| ENSMUSG00000027131 | Emc4 | protein\_coding | 2:112363011-112368027 (-) |  | -0.327 | 4.63e-04 | 4.06e-03 |
| ENSMUSG00000099413 | Gm17767 | lncRNA | 1:51507086-51596718 (-) |  | -1.380 | 4.63e-04 | 4.06e-03 |
| ENSMUSG00000054871 | Tmem158 | protein\_coding | 9:123259053-123260764 (-) |  | -1.690 | 4.63e-04 | 4.07e-03 |
| ENSMUSG00000029030 | Tprgl | protein\_coding | 4:154157485-154160666 (-) |  | 0.310 | 4.66e-04 | 4.08e-03 |
| ENSMUSG00000043671 | Dpy19l3 | protein\_coding | 7:35685165-35754454 (-) |  | 0.665 | 4.67e-04 | 4.09e-03 |
| ENSMUSG00000049090 | Zadh2 | protein\_coding | 18:84088063-84097528 (+) |  | 0.305 | 4.68e-04 | 4.10e-03 |
| ENSMUSG00000032741 | Tpcn1 | protein\_coding | 5:120534153-120588673 (-) |  | 0.274 | 4.69e-04 | 4.11e-03 |
| ENSMUSG00000028822 | Tmem50a | protein\_coding | 4:134897849-134915024 (-) |  | 0.210 | 4.70e-04 | 4.11e-03 |
| ENSMUSG00000048498 | Cd300e | protein\_coding | 11:115051917-115062177 (-) |  | 4.250 | 4.74e-04 | 4.14e-03 |
| ENSMUSG00000032198 | Dock6 | protein\_coding | 9:21799860-21852635 (-) |  | 0.632 | 4.75e-04 | 4.15e-03 |
| ENSMUSG00000040620 | Dhx33 | protein\_coding | 11:70984091-71004437 (-) |  | -0.257 | 4.79e-04 | 4.18e-03 |
| ENSMUSG00000100182 | 1810006J02Rik | lncRNA | 1:98131456-98144655 (+) |  | 1.540 | 4.79e-04 | 4.18e-03 |
| ENSMUSG00000028803 | Nipal3 | protein\_coding | 4:135445420-135495038 (-) |  | 0.551 | 4.80e-04 | 4.19e-03 |
| ENSMUSG00000023022 | Lima1 | protein\_coding | 15:99778470-99875456 (-) |  | 0.574 | 4.83e-04 | 4.21e-03 |
| ENSMUSG00000031907 | Zfp90 | protein\_coding | 8:106415327-106426598 (+) |  | 0.404 | 4.83e-04 | 4.21e-03 |
| ENSMUSG00000047909 | Ankrd16 | protein\_coding | 2:11777876-11790329 (+) |  | 0.348 | 4.86e-04 | 4.23e-03 |
| ENSMUSG00000039686 | Zer1 | protein\_coding | 2:30097283-30124585 (-) |  | 0.466 | 4.87e-04 | 4.24e-03 |
| ENSMUSG00000051335 | Gfod1 | protein\_coding | 13:43195245-43304172 (-) |  | 0.306 | 4.88e-04 | 4.24e-03 |
| ENSMUSG00000000085 | Scmh1 | protein\_coding | 4:120405281-120530186 (+) |  | 0.358 | 4.88e-04 | 4.24e-03 |
| ENSMUSG00000028514 | Usp24 | protein\_coding | 4:106316213-106441322 (+) |  | -0.293 | 4.90e-04 | 4.25e-03 |
| ENSMUSG00000033909 | Usp36 | protein\_coding | 11:118259651-118290244 (-) |  | -0.354 | 4.90e-04 | 4.25e-03 |
| ENSMUSG00000025357 | Dgka | protein\_coding | 10:128720134-128744855 (-) |  | 0.411 | 4.91e-04 | 4.26e-03 |
| ENSMUSG00000005674 | Tomm40l | protein\_coding | 1:171216011-171222514 (-) |  | -0.502 | 4.94e-04 | 4.28e-03 |
| ENSMUSG00000021254 | Gpatch2l | protein\_coding | 12:86241858-86291784 (+) |  | 0.312 | 4.96e-04 | 4.29e-03 |
| ENSMUSG00000002835 | Chaf1a | protein\_coding | 17:56040439-56072289 (+) |  | -0.195 | 4.96e-04 | 4.29e-03 |
| ENSMUSG00000029145 | Eif2b4 | protein\_coding | 5:31187558-31193430 (-) |  | -0.314 | 4.99e-04 | 4.31e-03 |
| ENSMUSG00000022471 | Xrcc6 | protein\_coding | 15:81987835-82040085 (+) |  | -0.285 | 5.00e-04 | 4.32e-03 |
| ENSMUSG00000039985 | Sinhcaf | protein\_coding | 6:148921035-148946467 (-) |  | -0.342 | 5.04e-04 | 4.35e-03 |
| ENSMUSG00000022587 | Ly6e | protein\_coding | 15:74955051-74959905 (+) |  | -0.161 | 5.05e-04 | 4.35e-03 |
| ENSMUSG00000098274 | Rpl24 | protein\_coding | 16:55966275-55971435 (+) |  | -0.483 | 5.05e-04 | 4.35e-03 |
| ENSMUSG00000022982 | Sod1 | protein\_coding | 16:90220754-90226329 (+) |  | 0.196 | 5.05e-04 | 4.35e-03 |
| ENSMUSG00000031642 | Sh3rf1 | protein\_coding | 8:61223872-61396071 (+) |  | 0.602 | 5.06e-04 | 4.36e-03 |
| ENSMUSG00000018909 | Arrb1 | protein\_coding | 7:99535466-99606771 (+) |  | 0.331 | 5.07e-04 | 4.36e-03 |
| ENSMUSG00000034853 | Acot11 | protein\_coding | 4:106744555-106804998 (-) |  | 0.346 | 5.08e-04 | 4.37e-03 |
| ENSMUSG00000029507 | Pus1 | protein\_coding | 5:110773667-110780659 (-) |  | -0.275 | 5.09e-04 | 4.37e-03 |
| ENSMUSG00000029405 | G3bp2 | protein\_coding | 5:92052146-92083719 (-) |  | -0.222 | 5.11e-04 | 4.39e-03 |
| ENSMUSG00000046668 | Cxxc5 | protein\_coding | 18:35829397-35861688 (+) |  | 0.694 | 5.11e-04 | 4.39e-03 |
| ENSMUSG00000039585 | Myo9a | protein\_coding | 9:59750896-59928866 (+) |  | 0.374 | 5.13e-04 | 4.40e-03 |
| ENSMUSG00000004552 | Ctse | protein\_coding | 1:131638306-131675505 (+) |  | 0.513 | 5.14e-04 | 4.41e-03 |
| ENSMUSG00000020328 | Nudcd2 | protein\_coding | 11:40733667-40740046 (+) |  | -0.348 | 5.15e-04 | 4.41e-03 |
| ENSMUSG00000052833 | Sae1 | protein\_coding | 7:16320234-16387806 (-) |  | -0.234 | 5.16e-04 | 4.41e-03 |
| ENSMUSG00000041491 | Cep78 | protein\_coding | 19:15955773-15984989 (-) |  | -0.283 | 5.17e-04 | 4.42e-03 |
| ENSMUSG00000026127 | Imp4 | protein\_coding | 1:34439851-34449356 (+) |  | -0.251 | 5.18e-04 | 4.43e-03 |
| ENSMUSG00000037487 | Ubr5 | protein\_coding | 15:37967328-38078854 (-) |  | -0.209 | 5.20e-04 | 4.44e-03 |
| ENSMUSG00000032458 | Copb2 | protein\_coding | 9:98563721-98588382 (+) |  | -0.195 | 5.23e-04 | 4.47e-03 |
| ENSMUSG00000097365 | C030034L19Rik | lncRNA | 3:9403064-9437233 (+) |  | 0.874 | 5.29e-04 | 4.51e-03 |
| ENSMUSG00000022122 | Ednrb | protein\_coding | 14:103814625-103844402 (-) |  | -0.766 | 5.29e-04 | 4.51e-03 |
| ENSMUSG00000021661 | Ankra2 | protein\_coding | 13:98263074-98274754 (+) |  | 0.448 | 5.29e-04 | 4.51e-03 |
| ENSMUSG00000018796 | Acsl1 | protein\_coding | 8:46471037-46536051 (+) |  | 0.260 | 5.30e-04 | 4.51e-03 |
| ENSMUSG00000020647 | Ncoa1 | protein\_coding | 12:4247362-4477182 (-) |  | 0.344 | 5.33e-04 | 4.53e-03 |
| ENSMUSG00000047368 | Abhd17b | protein\_coding | 19:21653185-21685637 (+) |  | -0.313 | 5.34e-04 | 4.54e-03 |
| ENSMUSG00000055639 | Dach1 | protein\_coding | 14:97786853-98169765 (-) |  | 1.010 | 5.35e-04 | 4.55e-03 |
| ENSMUSG00000034220 | Gpc1 | protein\_coding | 1:92831645-92860779 (+) |  | 0.342 | 5.36e-04 | 4.55e-03 |
| ENSMUSG00000030057 | Cnbp | protein\_coding | 6:87842615-87851106 (-) |  | -0.270 | 5.37e-04 | 4.55e-03 |
| ENSMUSG00000024776 | Stambpl1 | protein\_coding | 19:34192229-34240333 (+) |  | -0.398 | 5.40e-04 | 4.58e-03 |
| ENSMUSG00000038975 | Rabggtb | protein\_coding | 3:153907287-153913009 (-) |  | -0.302 | 5.43e-04 | 4.60e-03 |
| ENSMUSG00000028101 | Pias3 | protein\_coding | 3:96696384-96706070 (+) |  | 0.408 | 5.44e-04 | 4.60e-03 |
| ENSMUSG00000047180 | Neurl3 | protein\_coding | 1:36264597-36274679 (-) |  | 0.645 | 5.44e-04 | 4.60e-03 |
| ENSMUSG00000097141 | Gm10524 | TEC | 18:82692284-82694176 (+) |  | 0.731 | 5.50e-04 | 4.65e-03 |
| ENSMUSG00000002897 | Il17ra | protein\_coding | 6:120463247-120487559 (+) |  | 0.270 | 5.51e-04 | 4.66e-03 |
| ENSMUSG00000046330 | Rpl37a | protein\_coding | 1:72711290-72713813 (+) |  | 0.283 | 5.53e-04 | 4.67e-03 |
| ENSMUSG00000019768 | Esr1 | protein\_coding | 10:4611593-5005614 (+) |  | 0.456 | 5.55e-04 | 4.68e-03 |
| ENSMUSG00000032012 | Nectin1 | protein\_coding | 9:43743984-43832658 (+) |  | 0.621 | 5.59e-04 | 4.71e-03 |
| ENSMUSG00000015882 | Lcorl | protein\_coding | 5:45697181-45857615 (-) |  | 0.282 | 5.63e-04 | 4.75e-03 |
| ENSMUSG00000030162 | Olr1 | protein\_coding | 6:129485244-129507165 (-) |  | 0.584 | 5.65e-04 | 4.76e-03 |
| ENSMUSG00000085442 | Gm3362 | processed\_pseudogene | 15:37943327-37943792 (-) |  | -0.539 | 5.69e-04 | 4.79e-03 |
| ENSMUSG00000020899 | Pfas | protein\_coding | 11:68985697-69008460 (-) |  | -0.301 | 5.69e-04 | 4.79e-03 |
| ENSMUSG00000028796 | Phc2 | protein\_coding | 4:128654702-128752881 (+) |  | -0.211 | 5.70e-04 | 4.79e-03 |
| ENSMUSG00000035754 | Wdr18 | protein\_coding | 10:79960152-79970203 (+) |  | -0.380 | 5.70e-04 | 4.79e-03 |
| ENSMUSG00000009293 | Ube2g2 | protein\_coding | 10:77622275-77645993 (+) |  | -0.276 | 5.77e-04 | 4.85e-03 |
| ENSMUSG00000016554 | Eif3d | protein\_coding | 15:77958998-77970813 (-) |  | -0.275 | 5.78e-04 | 4.85e-03 |
| ENSMUSG00000022488 | Nckap1l | protein\_coding | 15:103453794-103498810 (+) |  | 0.210 | 5.79e-04 | 4.85e-03 |
| ENSMUSG00000032860 | P2ry2 | protein\_coding | 7:100996568-101012866 (-) |  | -0.496 | 5.81e-04 | 4.87e-03 |
| ENSMUSG00000031715 | Smarca5 | protein\_coding | 8:80698507-80739497 (-) |  | -0.269 | 5.81e-04 | 4.87e-03 |
| ENSMUSG00000021493 | Pdlim7 | protein\_coding | 13:55495795-55513676 (-) |  | 0.508 | 5.81e-04 | 4.87e-03 |
| ENSMUSG00000020178 | Adora2a | protein\_coding | 10:75316877-75334784 (+) |  | 1.440 | 5.84e-04 | 4.88e-03 |
| ENSMUSG00000027787 | Nmd3 | protein\_coding | 3:69721985-69756373 (+) |  | -0.318 | 5.84e-04 | 4.88e-03 |
| ENSMUSG00000110331 | Nudc-ps1 | processed\_pseudogene | 8:29286431-29286981 (-) |  | -0.351 | 5.84e-04 | 4.88e-03 |
| ENSMUSG00000034007 | Scaper | protein\_coding | 9:55549879-55938119 (-) |  | 0.548 | 5.85e-04 | 4.89e-03 |
| ENSMUSG00000041459 | Tardbp | protein\_coding | 4:148612382-148627019 (-) |  | -0.222 | 5.86e-04 | 4.89e-03 |
| ENSMUSG00000032666 | 1700025G04Rik | protein\_coding | 1:151852403-152090125 (-) |  | -0.298 | 5.91e-04 | 4.93e-03 |
| ENSMUSG00000045438 | Cox19 | protein\_coding | 5:139336189-139351418 (-) |  | -0.358 | 5.94e-04 | 4.95e-03 |
| ENSMUSG00000000386 | Mx1 | polymorphic\_pseudogene | 16:97447035-97462907 (-) |  | 0.555 | 5.96e-04 | 4.96e-03 |
| ENSMUSG00000001089 | Luzp1 | protein\_coding | 4:136469761-136554780 (+) |  | -0.378 | 5.97e-04 | 4.96e-03 |
| ENSMUSG00000027901 | Dennd2d | protein\_coding | 3:106482405-106503030 (+) |  | -0.381 | 5.98e-04 | 4.97e-03 |
| ENSMUSG00000059436 | Max | protein\_coding | 12:76937269-76962201 (-) |  | -0.225 | 6.00e-04 | 4.98e-03 |
| ENSMUSG00000018171 | Vmp1 | protein\_coding | 11:86583865-86683836 (-) |  | -0.349 | 6.00e-04 | 4.98e-03 |
| ENSMUSG00000024231 | Cul2 | protein\_coding | 18:3382988-3436377 (+) |  | -0.306 | 6.03e-04 | 5.00e-03 |
| ENSMUSG00000024480 | Ap3s1 | protein\_coding | 18:46741876-46790826 (+) |  | -0.393 | 6.03e-04 | 5.00e-03 |
| ENSMUSG00000029913 | Prdm5 | protein\_coding | 6:65778988-65937010 (+) |  | -0.942 | 6.03e-04 | 5.00e-03 |
| ENSMUSG00000003355 | Fkbp11 | protein\_coding | 15:98724366-98728198 (-) |  | -0.818 | 6.04e-04 | 5.00e-03 |
| ENSMUSG00000066621 | Tecpr1 | protein\_coding | 5:144194442-144223615 (-) |  | 0.482 | 6.04e-04 | 5.00e-03 |
| ENSMUSG00000114277 | Gm48583 | processed\_pseudogene | 13:60641790-60642009 (+) |  | 0.316 | 6.05e-04 | 5.01e-03 |
| ENSMUSG00000084964 | Gm15503 | lncRNA | 7:128408968-128412341 (-) |  | 1.050 | 6.11e-04 | 5.05e-03 |
| ENSMUSG00000003299 | Mrpl4 | protein\_coding | 9:21002738-21008839 (+) |  | -0.236 | 6.14e-04 | 5.07e-03 |
| ENSMUSG00000040687 | Madd | protein\_coding | 2:91137360-91183837 (-) |  | 0.298 | 6.16e-04 | 5.08e-03 |
| ENSMUSG00000017264 | Exosc10 | protein\_coding | 4:148558429-148582401 (+) |  | -0.229 | 6.16e-04 | 5.08e-03 |
| ENSMUSG00000026421 | Csrp1 | protein\_coding | 1:135720061-135752232 (+) |  | 0.299 | 6.17e-04 | 5.08e-03 |
| ENSMUSG00000025889 | Snca | protein\_coding | 6:60731575-60829855 (-) |  | 2.310 | 6.17e-04 | 5.08e-03 |
| ENSMUSG00000023456 | Tpi1 | protein\_coding | 6:124810586-124814296 (-) |  | -0.301 | 6.17e-04 | 5.08e-03 |
| ENSMUSG00000029171 | Pgm2 | protein\_coding | 5:64092950-64128351 (+) |  | -0.255 | 6.18e-04 | 5.08e-03 |
| ENSMUSG00000023034 | Nr4a1 | protein\_coding | 15:101254269-101274795 (+) |  | 1.720 | 6.18e-04 | 5.08e-03 |
| ENSMUSG00000032965 | Ift57 | protein\_coding | 16:49699233-49765126 (+) |  | -0.405 | 6.18e-04 | 5.08e-03 |
| ENSMUSG00000022564 | Grina | protein\_coding | 15:76246764-76249904 (+) |  | -0.334 | 6.22e-04 | 5.11e-03 |
| ENSMUSG00000040990 | Sh3kbp1 | protein\_coding | X:159627272-159978069 (+) |  | 0.241 | 6.26e-04 | 5.14e-03 |
| ENSMUSG00000033047 | Eif3l | protein\_coding | 15:79075179-79094405 (+) |  | -0.225 | 6.27e-04 | 5.14e-03 |
| ENSMUSG00000014504 | Srp19 | protein\_coding | 18:34330847-34336599 (+) |  | -0.281 | 6.28e-04 | 5.15e-03 |
| ENSMUSG00000035835 | Plppr3 | protein\_coding | 10:79860475-79874634 (-) |  | -0.263 | 6.29e-04 | 5.16e-03 |
| ENSMUSG00000030766 | Arhgap17 | protein\_coding | 7:123279218-123369915 (-) |  | 0.269 | 6.31e-04 | 5.17e-03 |
| ENSMUSG00000000876 | Pxmp4 | protein\_coding | 2:154585758-154603708 (-) |  | 0.332 | 6.32e-04 | 5.18e-03 |
| ENSMUSG00000020111 | Micu1 | protein\_coding | 10:59702477-59864132 (+) |  | 0.269 | 6.36e-04 | 5.20e-03 |
| ENSMUSG00000023572 | Ccndbp1 | protein\_coding | 2:121008403-121016904 (+) |  | 0.361 | 6.39e-04 | 5.23e-03 |
| ENSMUSG00000040652 | Oaz2 | protein\_coding | 9:65668001-65690300 (+) |  | 0.324 | 6.44e-04 | 5.26e-03 |
| ENSMUSG00000013622 | Atraid | protein\_coding | 5:31048312-31054623 (+) |  | 0.304 | 6.44e-04 | 5.26e-03 |
| ENSMUSG00000024681 | Ms4a3 | protein\_coding | 19:11629496-11640851 (-) |  | -0.632 | 6.50e-04 | 5.30e-03 |
| ENSMUSG00000029923 | Rab19 | protein\_coding | 6:39381175-39390380 (+) |  | 1.240 | 6.56e-04 | 5.35e-03 |
| ENSMUSG00000022724 | Riox2 | protein\_coding | 16:59471775-59492461 (+) |  | -0.304 | 6.58e-04 | 5.36e-03 |
| ENSMUSG00000060904 | Arl1 | protein\_coding | 10:88730858-88744094 (+) |  | -0.309 | 6.59e-04 | 5.36e-03 |
| ENSMUSG00000075269 | Bex6 | protein\_coding | 16:32179823-32186972 (+) |  | 0.305 | 6.62e-04 | 5.39e-03 |
| ENSMUSG00000063564 | Col23a1 | protein\_coding | 11:51289920-51583918 (+) |  | 1.340 | 6.63e-04 | 5.39e-03 |
| ENSMUSG00000071866 | Ppia | protein\_coding | 11:6415443-6419817 (+) |  | -0.368 | 6.64e-04 | 5.40e-03 |
| ENSMUSG00000031917 | Nip7 | protein\_coding | 8:107056877-107060931 (+) |  | -0.345 | 6.66e-04 | 5.41e-03 |
| ENSMUSG00000051043 | Gprc5c | protein\_coding | 11:114851152-114872617 (+) |  | -1.070 | 6.68e-04 | 5.43e-03 |
| ENSMUSG00000021340 | Gpld1 | protein\_coding | 13:24943152-24992501 (+) |  | -1.620 | 6.73e-04 | 5.46e-03 |
| ENSMUSG00000050106 | Tmc8 | protein\_coding | 11:117782076-117793110 (+) |  | 0.544 | 6.73e-04 | 5.46e-03 |
| ENSMUSG00000021156 | Zmynd11 | protein\_coding | 13:9684833-9765330 (-) |  | 0.267 | 6.78e-04 | 5.49e-03 |
| ENSMUSG00000081723 | Gm15931 | unprocessed\_pseudogene | 7:4274189-4282645 (+) |  | 1.830 | 6.78e-04 | 5.49e-03 |
| ENSMUSG00000000326 | Comt | protein\_coding | 16:18406886-18426852 (-) |  | -0.321 | 6.79e-04 | 5.50e-03 |
| ENSMUSG00000073131 | Vma21 | protein\_coding | X:71815924-71839757 (+) |  | -0.308 | 6.81e-04 | 5.51e-03 |
| ENSMUSG00000022814 | Umps | protein\_coding | 16:33954782-33967038 (-) |  | -0.229 | 6.82e-04 | 5.51e-03 |
| ENSMUSG00000061046 | Haghl | protein\_coding | 17:25779843-25785673 (-) |  | 0.425 | 6.86e-04 | 5.54e-03 |
| ENSMUSG00000025648 | Pfkfb4 | protein\_coding | 9:108991778-109032228 (+) |  | 0.404 | 6.86e-04 | 5.54e-03 |
| ENSMUSG00000116971 | Gm49766 | lncRNA | 10:67785749-67836401 (+) |  | 1.900 | 6.87e-04 | 5.54e-03 |
| ENSMUSG00000027642 | Rpn2 | protein\_coding | 2:157279017-157326319 (+) |  | -0.217 | 6.89e-04 | 5.56e-03 |
| ENSMUSG00000032470 | Mras | protein\_coding | 9:99385420-99437381 (-) |  | -1.050 | 6.90e-04 | 5.56e-03 |
| ENSMUSG00000031608 | Galnt7 | protein\_coding | 8:57523828-57653032 (-) |  | -0.294 | 6.92e-04 | 5.57e-03 |
| ENSMUSG00000052331 | Ankrd44 | protein\_coding | 1:54645340-54926387 (-) |  | 0.282 | 6.92e-04 | 5.57e-03 |
| ENSMUSG00000018677 | Slc25a39 | protein\_coding | 11:102402985-102407946 (-) |  | -0.243 | 6.94e-04 | 5.58e-03 |
| ENSMUSG00000038286 | Bphl | protein\_coding | 13:34037597-34074074 (+) |  | 0.375 | 6.97e-04 | 5.60e-03 |
| ENSMUSG00000032939 | Nup93 | protein\_coding | 8:94214564-94317227 (+) |  | -0.231 | 6.97e-04 | 5.60e-03 |
| ENSMUSG00000006005 | Tpr | protein\_coding | 1:150392838-150449935 (+) |  | -0.206 | 6.98e-04 | 5.60e-03 |
| ENSMUSG00000042423 | Fbrs | protein\_coding | 7:127479199-127491711 (+) |  | 0.281 | 7.05e-04 | 5.66e-03 |
| ENSMUSG00000041187 | Prkd2 | protein\_coding | 7:16842902-16870464 (+) |  | 0.502 | 7.09e-04 | 5.69e-03 |
| ENSMUSG00000033703 | Fcsk | protein\_coding | 8:110882456-110902488 (-) |  | 0.414 | 7.10e-04 | 5.69e-03 |
| ENSMUSG00000049502 | Dtx3l | protein\_coding | 16:35926511-35939151 (-) |  | -0.255 | 7.13e-04 | 5.71e-03 |
| ENSMUSG00000033880 | Lgals3bp | protein\_coding | 11:118392751-118402092 (-) |  | -0.289 | 7.14e-04 | 5.71e-03 |
| ENSMUSG00000040234 | Tm7sf3 | protein\_coding | 6:146602352-146642824 (-) |  | 0.225 | 7.15e-04 | 5.72e-03 |
| ENSMUSG00000069892 | 9930111J21Rik2 | protein\_coding | 11:49015874-49051242 (-) |  | 0.412 | 7.20e-04 | 5.76e-03 |
| ENSMUSG00000028639 | Ybx1 | protein\_coding | 4:119277981-119294604 (-) |  | -0.260 | 7.21e-04 | 5.76e-03 |
| ENSMUSG00000051457 | Spn | protein\_coding | 7:127132232-127137823 (-) |  | 0.202 | 7.22e-04 | 5.76e-03 |
| ENSMUSG00000036093 | Arl5a | protein\_coding | 2:52397951-52424901 (-) |  | -0.292 | 7.23e-04 | 5.77e-03 |
| ENSMUSG00000021823 | Vcl | protein\_coding | 14:20929398-21033676 (+) |  | 0.346 | 7.25e-04 | 5.78e-03 |
| ENSMUSG00000074519 | Zfp971 | protein\_coding | 2:178023284-178034022 (+) |  | -0.548 | 7.27e-04 | 5.79e-03 |
| ENSMUSG00000023118 | Sympk | protein\_coding | 7:19024377-19054618 (+) |  | -0.213 | 7.30e-04 | 5.81e-03 |
| ENSMUSG00000065968 | Ifitm7 | protein\_coding | 16:13981699-13986888 (-) |  | 1.460 | 7.34e-04 | 5.84e-03 |
| ENSMUSG00000048039 | Isg20l2 | protein\_coding | 3:87930314-87940686 (+) |  | -0.218 | 7.36e-04 | 5.85e-03 |
| ENSMUSG00000035561 | Aldh1b1 | protein\_coding | 4:45799022-45804604 (+) |  | -0.293 | 7.37e-04 | 5.86e-03 |
| ENSMUSG00000052415 | Tchh | protein\_coding | 3:93442330-93449077 (+) |  | 0.750 | 7.37e-04 | 5.86e-03 |
| ENSMUSG00000025877 | Hk3 | protein\_coding | 13:55005985-55021385 (-) |  | 0.290 | 7.39e-04 | 5.87e-03 |
| ENSMUSG00000035697 | Arhgap45 | protein\_coding | 10:80016653-80031472 (+) |  | 0.211 | 7.40e-04 | 5.87e-03 |
| ENSMUSG00000020929 | Eftud2 | protein\_coding | 11:102838473-102880985 (-) |  | -0.249 | 7.40e-04 | 5.87e-03 |
| ENSMUSG00000035595 | 1600002K03Rik | protein\_coding | 10:80172944-80175146 (+) |  | -0.602 | 7.44e-04 | 5.89e-03 |
| ENSMUSG00000027698 | Nceh1 | protein\_coding | 3:27182965-27284608 (+) |  | 0.329 | 7.44e-04 | 5.89e-03 |
| ENSMUSG00000021215 | Net1 | protein\_coding | 13:3882018-3918220 (-) |  | 0.250 | 7.44e-04 | 5.89e-03 |
| ENSMUSG00000066258 | Trim12a | protein\_coding | 7:104299894-104315466 (-) |  | 0.321 | 7.45e-04 | 5.89e-03 |
| ENSMUSG00000041638 | Gcn1 | protein\_coding | 5:115565254-115622654 (+) |  | -0.254 | 7.47e-04 | 5.90e-03 |
| ENSMUSG00000025324 | Atp10a | protein\_coding | 7:58656166-58829420 (+) |  | -0.221 | 7.50e-04 | 5.92e-03 |
| ENSMUSG00000022551 | Cyc1 | protein\_coding | 15:76343523-76346260 (+) |  | -0.319 | 7.50e-04 | 5.92e-03 |
| ENSMUSG00000017801 | Mlx | protein\_coding | 11:101087277-101092207 (+) |  | -0.277 | 7.51e-04 | 5.92e-03 |
| ENSMUSG00000047604 | Frat2 | protein\_coding | 19:41845972-41848132 (-) |  | 0.404 | 7.52e-04 | 5.93e-03 |
| ENSMUSG00000044197 | Gpr146 | protein\_coding | 5:139377697-139396415 (+) |  | -0.335 | 7.52e-04 | 5.93e-03 |
| ENSMUSG00000032459 | Mrps22 | protein\_coding | 9:98588730-98601660 (-) |  | -0.399 | 7.53e-04 | 5.93e-03 |
| ENSMUSG00000024740 | Ddb1 | protein\_coding | 19:10605327-10629819 (+) |  | -0.264 | 7.53e-04 | 5.93e-03 |
| ENSMUSG00000021485 | Mxd3 | protein\_coding | 13:55325168-55329823 (-) |  | 0.367 | 7.55e-04 | 5.94e-03 |
| ENSMUSG00000005575 | Ube2m | protein\_coding | 7:13035120-13038275 (-) |  | -0.282 | 7.57e-04 | 5.95e-03 |
| ENSMUSG00000038388 | Mpp6 | protein\_coding | 6:50110241-50198939 (+) |  | -0.233 | 7.70e-04 | 6.05e-03 |
| ENSMUSG00000030064 | Frmd4b | protein\_coding | 6:97286867-97617541 (-) |  | 0.424 | 7.72e-04 | 6.06e-03 |
| ENSMUSG00000002944 | Cd36 | protein\_coding | 5:17781690-17888801 (-) |  | 3.190 | 7.76e-04 | 6.09e-03 |
| ENSMUSG00000036285 | Noa1 | protein\_coding | 5:77294182-77310084 (-) |  | -0.326 | 7.76e-04 | 6.09e-03 |
| ENSMUSG00000030844 | Rgs10 | protein\_coding | 7:128373621-128418758 (-) |  | 0.326 | 7.78e-04 | 6.10e-03 |
| ENSMUSG00000010110 | Stx5a | protein\_coding | 19:8741413-8756069 (+) |  | 0.246 | 7.82e-04 | 6.13e-03 |
| ENSMUSG00000028756 | Pink1 | protein\_coding | 4:138313409-138326307 (-) |  | 0.458 | 7.86e-04 | 6.15e-03 |
| ENSMUSG00000018040 | Rrp7a | protein\_coding | 15:83113433-83122801 (-) |  | -0.271 | 7.87e-04 | 6.16e-03 |
| ENSMUSG00000058258 | Idi1 | protein\_coding | 13:8885501-8892451 (+) |  | -0.452 | 7.88e-04 | 6.16e-03 |
| ENSMUSG00000034724 | Cnot6l | protein\_coding | 5:96070333-96164171 (-) |  | 0.229 | 7.94e-04 | 6.20e-03 |
| ENSMUSG00000041354 | Rgl2 | protein\_coding | 17:33929543-33937687 (+) |  | 0.217 | 7.95e-04 | 6.21e-03 |
| ENSMUSG00000048142 | Nat8l | protein\_coding | 5:33995984-34005916 (+) |  | 0.581 | 7.96e-04 | 6.21e-03 |
| ENSMUSG00000051682 | Treml4 | protein\_coding | 17:48264295-48275360 (+) |  | 2.470 | 7.97e-04 | 6.21e-03 |
| ENSMUSG00000014355 | Anapc1 | protein\_coding | 2:128610104-128687391 (-) |  | -0.253 | 8.00e-04 | 6.24e-03 |
| ENSMUSG00000104802 | Gm5869 | processed\_pseudogene | 5:86997159-86998415 (+) |  | 0.511 | 8.02e-04 | 6.25e-03 |
| ENSMUSG00000075703 | Selenoi | protein\_coding | 5:30232581-30272427 (+) |  | -0.267 | 8.05e-04 | 6.27e-03 |
| ENSMUSG00000029860 | Zyx | protein\_coding | 6:42349630-42360213 (+) |  | 0.271 | 8.08e-04 | 6.29e-03 |
| ENSMUSG00000066571 | 4931406P16Rik | protein\_coding | 7:34236707-34313551 (-) |  | 0.358 | 8.10e-04 | 6.30e-03 |
| ENSMUSG00000017386 | Traf4 | protein\_coding | 11:78158499-78165589 (-) |  | -1.080 | 8.10e-04 | 6.30e-03 |
| ENSMUSG00000031444 | F10 | protein\_coding | 8:13037308-13056676 (+) |  | -0.322 | 8.11e-04 | 6.30e-03 |
| ENSMUSG00000034789 | Rab24 | protein\_coding | 13:55319743-55321946 (-) |  | -0.320 | 8.11e-04 | 6.30e-03 |
| ENSMUSG00000089782 | Gm3531 | processed\_pseudogene | 1:97948901-97949389 (-) |  | -0.481 | 8.12e-04 | 6.30e-03 |
| ENSMUSG00000038517 | Tbkbp1 | protein\_coding | 11:97136171-97151495 (-) |  | 0.333 | 8.14e-04 | 6.31e-03 |
| ENSMUSG00000026972 | Arrdc1 | protein\_coding | 2:24925352-24935252 (-) |  | 0.267 | 8.24e-04 | 6.38e-03 |
| ENSMUSG00000020057 | Dram1 | protein\_coding | 10:88322804-88379080 (-) |  | 0.286 | 8.25e-04 | 6.39e-03 |
| ENSMUSG00000028680 | Plk3 | protein\_coding | 4:117128655-117133963 (-) |  | -1.310 | 8.26e-04 | 6.39e-03 |
| ENSMUSG00000006395 | Hyi | polymorphic\_pseudogene | 4:118359990-118362744 (+) |  | 0.763 | 8.27e-04 | 6.39e-03 |
| ENSMUSG00000026835 | Fcnb | protein\_coding | 2:28076378-28084885 (-) |  | 0.408 | 8.27e-04 | 6.39e-03 |
| ENSMUSG00000020849 | Ywhae | protein\_coding | 11:75732869-75765845 (+) |  | -0.226 | 8.28e-04 | 6.40e-03 |
| ENSMUSG00000100514 | Gm12960 | processed\_pseudogene | 4:111807358-111808423 (-) |  | -0.422 | 8.31e-04 | 6.42e-03 |
| ENSMUSG00000085465 | Gm15347 | lncRNA | 8:12860358-12877344 (-) |  | -1.540 | 8.32e-04 | 6.42e-03 |
| ENSMUSG00000046546 | Fam43a | protein\_coding | 16:30599723-30602797 (+) |  | 0.513 | 8.34e-04 | 6.43e-03 |
| ENSMUSG00000111792 | Gm33858 | lncRNA | 9:120068996-120070622 (+) |  | 1.370 | 8.40e-04 | 6.47e-03 |
| ENSMUSG00000037458 | Azin1 | protein\_coding | 15:38487427-38519266 (-) |  | -0.196 | 8.40e-04 | 6.47e-03 |
| ENSMUSG00000038524 | Fchsd1 | protein\_coding | 18:37957431-37969774 (-) |  | 0.698 | 8.52e-04 | 6.56e-03 |
| ENSMUSG00000022962 | Gart | protein\_coding | 16:91621186-91646952 (-) |  | -0.276 | 8.59e-04 | 6.61e-03 |
| ENSMUSG00000032724 | Abtb2 | protein\_coding | 2:103566310-103718423 (+) |  | -1.070 | 8.60e-04 | 6.62e-03 |
| ENSMUSG00000027947 | Il6ra | protein\_coding | 3:89864059-89913196 (-) |  | 0.215 | 8.62e-04 | 6.63e-03 |
| ENSMUSG00000025289 | Prdx4 | protein\_coding | X:155323918-155340754 (-) |  | 0.305 | 8.65e-04 | 6.64e-03 |
| ENSMUSG00000000594 | Gm2a | protein\_coding | 11:55098115-55113029 (+) |  | 0.215 | 8.65e-04 | 6.64e-03 |
| ENSMUSG00000017697 | Ada | protein\_coding | 2:163726584-163750239 (-) |  | -0.438 | 8.66e-04 | 6.64e-03 |
| ENSMUSG00000000568 | Hnrnpd | protein\_coding | 5:99955935-99978938 (-) |  | -0.183 | 8.72e-04 | 6.69e-03 |
| ENSMUSG00000112537 | 4930543I11Rik | TEC | 10:67785735-67786693 (+) |  | 1.570 | 8.73e-04 | 6.69e-03 |
| ENSMUSG00000068854 | H2bc21 | protein\_coding | 3:96221119-96223738 (+) |  | 0.578 | 8.78e-04 | 6.72e-03 |
| ENSMUSG00000063286 | Gm8995 | transcribed\_unprocessed\_pseudogene | 7:106504239-106511671 (-) |  | -0.264 | 8.79e-04 | 6.73e-03 |
| ENSMUSG00000055850 | Rnf181 | protein\_coding | 6:72359714-72366956 (-) |  | 0.242 | 8.84e-04 | 6.76e-03 |
| ENSMUSG00000049950 | Rpp38 | protein\_coding | 2:3328949-3332643 (-) |  | -0.711 | 8.91e-04 | 6.81e-03 |
| ENSMUSG00000021996 | Esd | protein\_coding | 14:74732297-74750765 (+) |  | -0.272 | 8.97e-04 | 6.85e-03 |
| ENSMUSG00000030695 | Aldoa | protein\_coding | 7:126795234-126800751 (-) |  | -0.202 | 9.02e-04 | 6.88e-03 |
| ENSMUSG00000021678 | F2rl1 | protein\_coding | 13:95511732-95525227 (-) |  | 1.630 | 9.02e-04 | 6.88e-03 |
| ENSMUSG00000004207 | Psap | protein\_coding | 10:60277627-60302597 (+) |  | 0.275 | 9.02e-04 | 6.88e-03 |
| ENSMUSG00000011257 | Pabpc4 | protein\_coding | 4:123262351-123298925 (+) |  | -0.358 | 9.05e-04 | 6.90e-03 |
| ENSMUSG00000043091 | Tuba1c | protein\_coding | 15:99029891-99038110 (+) |  | -0.262 | 9.06e-04 | 6.90e-03 |
| ENSMUSG00000034430 | Zxdc | protein\_coding | 6:90369492-90403490 (+) |  | 0.377 | 9.11e-04 | 6.93e-03 |
| ENSMUSG00000022779 | Top3b | protein\_coding | 16:16870736-16892990 (+) |  | 0.304 | 9.11e-04 | 6.94e-03 |
| ENSMUSG00000037148 | Arhgap10 | protein\_coding | 8:77250366-77517953 (-) |  | -0.617 | 9.13e-04 | 6.94e-03 |
| ENSMUSG00000029385 | Ccng2 | protein\_coding | 5:93267257-93276231 (+) |  | 0.319 | 9.13e-04 | 6.94e-03 |
| ENSMUSG00000021572 | Cep72 | protein\_coding | 13:74036500-74062299 (-) |  | 0.338 | 9.19e-04 | 6.98e-03 |
| ENSMUSG00000037509 | Arhgef4 | protein\_coding | 1:34678188-34813309 (+) |  | 0.829 | 9.23e-04 | 7.01e-03 |
| ENSMUSG00000049225 | Pdp1 | protein\_coding | 4:11958184-11966452 (-) |  | 0.364 | 9.29e-04 | 7.05e-03 |
| ENSMUSG00000113769 | 5033406O09Rik | lncRNA | 12:111941991-111944484 (-) |  | 0.833 | 9.29e-04 | 7.05e-03 |
| ENSMUSG00000028028 | Alpk1 | protein\_coding | 3:127670310-127780527 (-) |  | 0.295 | 9.32e-04 | 7.06e-03 |
| ENSMUSG00000073988 | Ttpa | protein\_coding | 4:20007938-20030785 (+) |  | -0.664 | 9.37e-04 | 7.10e-03 |
| ENSMUSG00000022325 | Pop1 | protein\_coding | 15:34495304-34530648 (+) |  | -0.336 | 9.38e-04 | 7.10e-03 |
| ENSMUSG00000024982 | Zdhhc6 | protein\_coding | 19:55271291-55316032 (-) |  | -0.218 | 9.39e-04 | 7.11e-03 |
| ENSMUSG00000035575 | Utp6 | protein\_coding | 11:79932321-79962390 (-) |  | -0.221 | 9.44e-04 | 7.14e-03 |
| ENSMUSG00000028792 | Ak2 | protein\_coding | 4:128991958-129011529 (+) |  | -0.256 | 9.47e-04 | 7.15e-03 |
| ENSMUSG00000062908 | Acadm | protein\_coding | 3:153922357-153944632 (-) |  | 0.237 | 9.48e-04 | 7.16e-03 |
| ENSMUSG00000036371 | Serbp1 | protein\_coding | 6:67238176-67297736 (+) |  | -0.237 | 9.55e-04 | 7.21e-03 |
| ENSMUSG00000028550 | Atg4c | protein\_coding | 4:99193934-99259787 (+) |  | 0.387 | 9.57e-04 | 7.22e-03 |
| ENSMUSG00000031904 | Slc7a6 | protein\_coding | 8:106168857-106198706 (+) |  | -0.277 | 9.61e-04 | 7.24e-03 |
| ENSMUSG00000001576 | Ergic1 | protein\_coding | 17:26561489-26656934 (+) |  | -0.331 | 9.62e-04 | 7.25e-03 |
| ENSMUSG00000025236 | Adpgk | protein\_coding | 9:59291558-59324052 (+) |  | -0.222 | 9.65e-04 | 7.27e-03 |
| ENSMUSG00000067878 | Map7d3 | protein\_coding | X:56797858-56822326 (-) |  | 0.443 | 9.67e-04 | 7.28e-03 |
| ENSMUSG00000031672 | Got2 | protein\_coding | 8:95864134-95888547 (-) |  | -0.300 | 9.71e-04 | 7.30e-03 |
| ENSMUSG00000105397 | Gm43471 | processed\_pseudogene | 3:53401731-53402197 (-) |  | -0.728 | 9.73e-04 | 7.31e-03 |
| ENSMUSG00000033467 | Crlf2 | protein\_coding | 5:109554709-109558993 (-) |  | 0.317 | 9.79e-04 | 7.35e-03 |
| ENSMUSG00000037260 | Hgsnat | protein\_coding | 8:25944453-25976753 (-) |  | 0.334 | 9.81e-04 | 7.37e-03 |
| ENSMUSG00000040785 | Ttc3 | protein\_coding | 16:94370618-94469343 (+) |  | 0.318 | 9.82e-04 | 7.37e-03 |
| ENSMUSG00000028431 | Elp1 | protein\_coding | 4:56749680-56802331 (-) |  | -0.284 | 9.82e-04 | 7.37e-03 |
| ENSMUSG00000068882 | Ssb | protein\_coding | 2:69861562-69871846 (+) |  | -0.241 | 9.84e-04 | 7.38e-03 |
| ENSMUSG00000020733 | Slc9a3r1 | protein\_coding | 11:115163341-115181181 (+) |  | 0.218 | 9.87e-04 | 7.39e-03 |
| ENSMUSG00000107792 | Gm43914 | lncRNA | 6:129418179-129418772 (+) |  | 1.530 | 9.89e-04 | 7.40e-03 |
| ENSMUSG00000025492 | Ifitm3 | protein\_coding | 7:141009586-141010770 (-) |  | -0.610 | 9.90e-04 | 7.40e-03 |
| ENSMUSG00000029135 | Fosl2 | protein\_coding | 5:32135801-32157842 (+) |  | 0.357 | 9.93e-04 | 7.43e-03 |
| ENSMUSG00000029863 | Casp2 | protein\_coding | 6:42264985-42282508 (+) |  | 0.196 | 9.98e-04 | 7.46e-03 |
| ENSMUSG00000019132 | BC005537 | protein\_coding | 13:24801657-24816197 (+) |  | -0.182 | 1.00e-03 | 7.47e-03 |
| ENSMUSG00000024687 | Osbp | protein\_coding | 19:11965941-11994112 (+) |  | 0.191 | 1.00e-03 | 7.48e-03 |
| ENSMUSG00000000204 | Slfn4 | protein\_coding | 11:83175186-83190216 (+) |  | -0.989 | 1.01e-03 | 7.55e-03 |
| ENSMUSG00000002409 | Dyrk1b | protein\_coding | 7:28179469-28187294 (+) |  | 0.773 | 1.01e-03 | 7.55e-03 |
| ENSMUSG00000056724 | Nbeal2 | protein\_coding | 9:110624789-110654161 (-) |  | 0.303 | 1.02e-03 | 7.60e-03 |
| ENSMUSG00000029672 | Fam3c | protein\_coding | 6:22306520-22356243 (-) |  | -0.307 | 1.02e-03 | 7.60e-03 |
| ENSMUSG00000027404 | Snrpb | protein\_coding | 2:130171414-130179403 (-) |  | -0.183 | 1.02e-03 | 7.60e-03 |
| ENSMUSG00000010095 | Slc3a2 | protein\_coding | 19:8706882-8723369 (-) |  | -0.309 | 1.03e-03 | 7.64e-03 |
| ENSMUSG00000034729 | Mrps10 | protein\_coding | 17:47368887-47381417 (+) |  | -0.331 | 1.03e-03 | 7.69e-03 |
| ENSMUSG00000026223 | Itm2c | protein\_coding | 1:85894281-85908675 (+) |  | 0.260 | 1.03e-03 | 7.69e-03 |
| ENSMUSG00000037300 | Ttc13 | protein\_coding | 8:124671332-124721983 (-) |  | -0.207 | 1.04e-03 | 7.73e-03 |
| ENSMUSG00000040940 | Arhgef1 | protein\_coding | 7:24902912-24926594 (+) |  | 0.247 | 1.04e-03 | 7.75e-03 |
| ENSMUSG00000035329 | Fbxo33 | protein\_coding | 12:59200655-59219725 (-) |  | -0.375 | 1.04e-03 | 7.75e-03 |
| ENSMUSG00000029528 | Pxn | protein\_coding | 5:115506676-115555987 (+) |  | 0.224 | 1.05e-03 | 7.75e-03 |
| ENSMUSG00000028654 | Mycl | protein\_coding | 4:122995652-123002485 (+) |  | 0.808 | 1.05e-03 | 7.76e-03 |
| ENSMUSG00000034341 | Wbp2 | protein\_coding | 11:116078573-116086995 (-) |  | 0.268 | 1.05e-03 | 7.79e-03 |
| ENSMUSG00000053219 | Raet1e | protein\_coding | 10:22158569-22374139 (+) |  | -1.470 | 1.05e-03 | 7.79e-03 |
| ENSMUSG00000111877 | Gm6477 | processed\_pseudogene | 10:39198538-39199301 (+) |  | -0.559 | 1.05e-03 | 7.79e-03 |
| ENSMUSG00000018882 | Mrpl45 | protein\_coding | 11:97315716-97329920 (+) |  | -0.297 | 1.06e-03 | 7.82e-03 |
| ENSMUSG00000039768 | Dnajc11 | protein\_coding | 4:151933691-151982137 (+) |  | -0.236 | 1.07e-03 | 7.89e-03 |
| ENSMUSG00000034768 | Asb16 | protein\_coding | 11:102268743-102279462 (+) |  | -1.770 | 1.07e-03 | 7.91e-03 |
| ENSMUSG00000027968 | Larp7 | protein\_coding | 3:127536714-127553349 (-) |  | -0.234 | 1.07e-03 | 7.91e-03 |
| ENSMUSG00000039234 | Sec24d | protein\_coding | 3:123267455-123365641 (+) |  | -0.286 | 1.07e-03 | 7.91e-03 |
| ENSMUSG00000019883 | Echdc1 | protein\_coding | 10:29313166-29347469 (+) |  | -0.686 | 1.07e-03 | 7.92e-03 |
| ENSMUSG00000004865 | Srpk1 | protein\_coding | 17:28587648-28622709 (-) |  | -0.202 | 1.07e-03 | 7.92e-03 |
| ENSMUSG00000028381 | Ugcg | protein\_coding | 4:59189257-59222833 (+) |  | -0.263 | 1.08e-03 | 7.94e-03 |
| ENSMUSG00000024493 | Lars | protein\_coding | 18:42202298-42262194 (-) |  | -0.270 | 1.08e-03 | 7.96e-03 |
| ENSMUSG00000039041 | Adrm1 | protein\_coding | 2:180171485-180176286 (+) |  | -0.283 | 1.09e-03 | 8.01e-03 |
| ENSMUSG00000021607 | Mrpl36 | protein\_coding | 13:73328513-73332178 (+) |  | -0.325 | 1.09e-03 | 8.02e-03 |
| ENSMUSG00000024312 | Wdr46 | protein\_coding | 17:33940660-33949697 (+) |  | -0.323 | 1.09e-03 | 8.02e-03 |
| ENSMUSG00000028599 | Tnfrsf1b | protein\_coding | 4:145213463-145246870 (-) |  | 0.308 | 1.09e-03 | 8.04e-03 |
| ENSMUSG00000020100 | Slc29a3 | protein\_coding | 10:60712072-60752794 (-) |  | 0.242 | 1.10e-03 | 8.06e-03 |
| ENSMUSG00000040219 | Ttc12 | protein\_coding | 9:49436963-49486225 (-) |  | 1.030 | 1.10e-03 | 8.08e-03 |
| ENSMUSG00000054720 | Lrrc8c | protein\_coding | 5:105519388-105613018 (+) |  | 0.242 | 1.10e-03 | 8.10e-03 |
| ENSMUSG00000073678 | Pgap1 | protein\_coding | 1:54472994-54557684 (-) |  | 0.728 | 1.11e-03 | 8.10e-03 |
| ENSMUSG00000023067 | Cdkn1a | protein\_coding | 17:29090976-29100727 (+) |  | -0.386 | 1.11e-03 | 8.11e-03 |
| ENSMUSG00000024896 | Minpp1 | protein\_coding | 19:32485769-32515364 (+) |  | -0.266 | 1.11e-03 | 8.16e-03 |
| ENSMUSG00000039530 | Tusc3 | protein\_coding | 8:39005845-39165114 (+) |  | 0.329 | 1.12e-03 | 8.16e-03 |
| ENSMUSG00000033732 | Sf3b3 | protein\_coding | 8:110810239-110846787 (-) |  | -0.238 | 1.12e-03 | 8.16e-03 |
| ENSMUSG00000004568 | Arhgef18 | protein\_coding | 8:3353415-3456601 (+) |  | 0.246 | 1.12e-03 | 8.16e-03 |
| ENSMUSG00000029202 | Pds5a | protein\_coding | 5:65605721-65698273 (-) |  | -0.204 | 1.12e-03 | 8.16e-03 |
| ENSMUSG00000039697 | Ncoa7 | protein\_coding | 10:30628999-30803326 (-) |  | 0.317 | 1.12e-03 | 8.17e-03 |
| ENSMUSG00000068874 | Selenbp1 | protein\_coding | 3:94933056-94944758 (+) |  | 0.328 | 1.12e-03 | 8.18e-03 |
| ENSMUSG00000002985 | Apoe | protein\_coding | 7:19696109-19699188 (-) |  | 1.710 | 1.13e-03 | 8.20e-03 |
| ENSMUSG00000041797 | Abca9 | protein\_coding | 11:110100749-110168196 (-) |  | 0.916 | 1.13e-03 | 8.20e-03 |
| ENSMUSG00000036833 | Pnpla7 | protein\_coding | 2:24976033-25054057 (+) |  | 0.338 | 1.13e-03 | 8.23e-03 |
| ENSMUSG00000034377 | Tulp4 | protein\_coding | 17:6106437-6251128 (+) |  | -0.649 | 1.13e-03 | 8.23e-03 |
| ENSMUSG00000049103 | Ccr2 | protein\_coding | 9:124101950-124113557 (+) |  | -0.360 | 1.13e-03 | 8.24e-03 |
| ENSMUSG00000041426 | Hibch | protein\_coding | 1:52844929-52920986 (+) |  | -0.293 | 1.14e-03 | 8.26e-03 |
| ENSMUSG00000026589 | Sec16b | protein\_coding | 1:157506728-157568425 (+) |  | 1.280 | 1.14e-03 | 8.29e-03 |
| ENSMUSG00000002222 | Rmnd5a | protein\_coding | 6:71388634-71440637 (-) |  | 0.279 | 1.14e-03 | 8.31e-03 |
| ENSMUSG00000039879 | Heca | protein\_coding | 10:17868612-17948067 (-) |  | 0.279 | 1.15e-03 | 8.31e-03 |
| ENSMUSG00000096544 | Gm4617 | processed\_pseudogene | 3:124385667-124385997 (+) |  | -0.339 | 1.16e-03 | 8.38e-03 |
| ENSMUSG00000033826 | Dnah8 | protein\_coding | 17:30624354-30877365 (+) |  | 0.464 | 1.16e-03 | 8.39e-03 |
| ENSMUSG00000083097 | Gm14494 | processed\_pseudogene | 2:153826171-153826605 (+) |  | -0.464 | 1.16e-03 | 8.40e-03 |
| ENSMUSG00000025059 | Gk | protein\_coding | X:85701937-85776819 (-) |  | -0.413 | 1.16e-03 | 8.40e-03 |
| ENSMUSG00000020922 | Lsm12 | protein\_coding | 11:102162497-102185296 (-) |  | -0.249 | 1.16e-03 | 8.42e-03 |
| ENSMUSG00000007891 | Ctsd | protein\_coding | 7:142375911-142388038 (-) |  | -0.194 | 1.16e-03 | 8.42e-03 |
| ENSMUSG00000048087 | Gm4737 | protein\_coding | 16:46152985-46155077 (-) |  | -0.389 | 1.16e-03 | 8.42e-03 |
| ENSMUSG00000046908 | Ltb4r1 | protein\_coding | 14:55765962-55768494 (+) |  | 0.357 | 1.17e-03 | 8.42e-03 |
| ENSMUSG00000007817 | Zmiz1 | protein\_coding | 14:25459185-25666743 (+) |  | 0.262 | 1.17e-03 | 8.42e-03 |
| ENSMUSG00000034064 | Poglut1 | protein\_coding | 16:38525137-38550258 (-) |  | -0.265 | 1.17e-03 | 8.47e-03 |
| ENSMUSG00000022475 | Hdac7 | protein\_coding | 15:97792664-97844502 (-) |  | 0.412 | 1.17e-03 | 8.47e-03 |
| ENSMUSG00000039637 | Coro7 | protein\_coding | 16:4626133-4679777 (-) |  | 0.240 | 1.18e-03 | 8.48e-03 |
| ENSMUSG00000037447 | Arid5a | protein\_coding | 1:36307733-36324029 (+) |  | -0.244 | 1.18e-03 | 8.48e-03 |
| ENSMUSG00000038214 | Bend3 | protein\_coding | 10:43478831-43515396 (+) |  | -0.384 | 1.18e-03 | 8.50e-03 |
| ENSMUSG00000049739 | Zfp646 | protein\_coding | 7:127876221-127885996 (+) |  | 0.237 | 1.18e-03 | 8.53e-03 |
| ENSMUSG00000030727 | Rabep2 | protein\_coding | 7:126428759-126449245 (+) |  | 0.337 | 1.19e-03 | 8.53e-03 |
| ENSMUSG00000055866 | Per2 | protein\_coding | 1:91415982-91459324 (-) |  | 0.681 | 1.19e-03 | 8.57e-03 |
| ENSMUSG00000030465 | Psd3 | protein\_coding | 8:67689082-68212027 (-) |  | 0.427 | 1.19e-03 | 8.57e-03 |
| ENSMUSG00000028104 | Polr3gl | protein\_coding | 3:96577872-96594181 (-) |  | 0.451 | 1.20e-03 | 8.61e-03 |
| ENSMUSG00000038393 | Txnip | protein\_coding | 3:96557957-96561883 (+) |  | 0.431 | 1.20e-03 | 8.61e-03 |
| ENSMUSG00000035273 | Hpse | protein\_coding | 5:100679484-100719716 (-) |  | 0.274 | 1.20e-03 | 8.61e-03 |
| ENSMUSG00000024240 | Epc1 | protein\_coding | 18:6435951-6516108 (-) |  | 0.264 | 1.20e-03 | 8.62e-03 |
| ENSMUSG00000028937 | Acot7 | protein\_coding | 4:152178134-152271855 (+) |  | -0.242 | 1.20e-03 | 8.62e-03 |
| ENSMUSG00000025512 | Chid1 | protein\_coding | 7:141493136-141539857 (-) |  | -0.272 | 1.20e-03 | 8.62e-03 |
| ENSMUSG00000027763 | Mbnl1 | protein\_coding | 3:60472830-60629750 (+) |  | 0.187 | 1.21e-03 | 8.64e-03 |
| ENSMUSG00000118264 | Rps15-ps3 | transcribed\_processed\_pseudogene | 18:64288135-64291820 (+) |  | 0.816 | 1.21e-03 | 8.64e-03 |
| ENSMUSG00000011254 | Thg1l | protein\_coding | 11:45946843-45955494 (-) |  | -0.333 | 1.21e-03 | 8.66e-03 |
| ENSMUSG00000109865 | Hspa14 | protein\_coding | 2:3488850-3512814 (-) |  | -0.235 | 1.22e-03 | 8.75e-03 |
| ENSMUSG00000048758 | Rpl29 | protein\_coding | 9:106429454-106431568 (+) |  | -0.262 | 1.23e-03 | 8.76e-03 |
| ENSMUSG00000026696 | Vamp4 | protein\_coding | 1:162570515-162599084 (+) |  | -0.426 | 1.23e-03 | 8.80e-03 |
| ENSMUSG00000047547 | Cltb | protein\_coding | 13:54592401-54611344 (-) |  | -0.291 | 1.24e-03 | 8.81e-03 |
| ENSMUSG00000030703 | Gdpd3 | protein\_coding | 7:126766334-126775649 (+) |  | 0.701 | 1.24e-03 | 8.82e-03 |
| ENSMUSG00000049091 | Sephs2 | protein\_coding | 7:127271879-127274055 (-) |  | -0.191 | 1.24e-03 | 8.87e-03 |
| ENSMUSG00000032561 | Acpp | protein\_coding | 9:104288251-104337748 (-) |  | -0.888 | 1.25e-03 | 8.88e-03 |
| ENSMUSG00000022614 | Lmf2 | protein\_coding | 15:89351004-89355659 (-) |  | 0.229 | 1.26e-03 | 8.97e-03 |
| ENSMUSG00000021102 | Glrx5 | protein\_coding | 12:105032688-105042906 (+) |  | -0.300 | 1.27e-03 | 9.01e-03 |
| ENSMUSG00000083327 | Vcp-rs | processed\_pseudogene | X:104345829-104348234 (-) |  | -0.261 | 1.27e-03 | 9.01e-03 |
| ENSMUSG00000032126 | Hmbs | protein\_coding | 9:44336339-44344228 (-) |  | -0.278 | 1.27e-03 | 9.02e-03 |
| ENSMUSG00000039183 | Nubp2 | protein\_coding | 17:24882611-24886349 (-) |  | -0.234 | 1.27e-03 | 9.05e-03 |
| ENSMUSG00000032507 | Fbxl2 | protein\_coding | 9:113963637-114046191 (-) |  | -0.566 | 1.28e-03 | 9.09e-03 |
| ENSMUSG00000090290 | Tarbp1 | protein\_coding | 8:126425329-126475065 (-) |  | -0.302 | 1.28e-03 | 9.09e-03 |
| ENSMUSG00000010307 | Tmem86a | protein\_coding | 7:47050601-47054777 (+) |  | 0.561 | 1.29e-03 | 9.12e-03 |
| ENSMUSG00000025494 | Sigirr | protein\_coding | 7:141091175-141100572 (-) |  | 0.394 | 1.29e-03 | 9.13e-03 |
| ENSMUSG00000031751 | Amfr | protein\_coding | 8:93971588-94012842 (-) |  | 0.169 | 1.29e-03 | 9.13e-03 |
| ENSMUSG00000021610 | Clptm1l | protein\_coding | 13:73604006-73620605 (+) |  | -0.231 | 1.29e-03 | 9.14e-03 |
| ENSMUSG00000013698 | Pea15a | protein\_coding | 1:172196728-172206804 (-) |  | 0.363 | 1.29e-03 | 9.14e-03 |
| ENSMUSG00000021733 | Slc4a7 | protein\_coding | 14:14702279-14799940 (+) |  | -0.233 | 1.29e-03 | 9.14e-03 |
| ENSMUSG00000032727 | Mier3 | protein\_coding | 13:111680979-111718596 (+) |  | 0.247 | 1.29e-03 | 9.14e-03 |
| ENSMUSG00000027506 | Tpd52 | protein\_coding | 3:8925593-9004723 (-) |  | -0.274 | 1.30e-03 | 9.15e-03 |
| ENSMUSG00000029094 | Afap1 | protein\_coding | 5:35893319-36003923 (+) |  | 0.571 | 1.30e-03 | 9.15e-03 |
| ENSMUSG00000024187 | Fam234a | protein\_coding | 17:26211822-26244242 (-) |  | 0.358 | 1.30e-03 | 9.17e-03 |
| ENSMUSG00000041781 | Cpsf2 | protein\_coding | 12:101975988-102006424 (+) |  | -0.212 | 1.30e-03 | 9.18e-03 |
| ENSMUSG00000001062 | Vps9d1 | protein\_coding | 8:123242356-123254348 (-) |  | 0.370 | 1.31e-03 | 9.21e-03 |
| ENSMUSG00000032661 | Oas3 | protein\_coding | 5:120753098-120777661 (-) |  | -0.556 | 1.31e-03 | 9.21e-03 |
| ENSMUSG00000046062 | Ppp1r15b | protein\_coding | 1:133131143-133139783 (+) |  | -0.221 | 1.31e-03 | 9.25e-03 |
| ENSMUSG00000022824 | Muc13 | protein\_coding | 16:33794037-33819934 (+) |  | -0.543 | 1.32e-03 | 9.25e-03 |
| ENSMUSG00000031833 | Mast3 | protein\_coding | 8:70778117-70805054 (-) |  | 0.295 | 1.32e-03 | 9.25e-03 |
| ENSMUSG00000040466 | Blvrb | protein\_coding | 7:27447978-27466144 (+) |  | 0.625 | 1.32e-03 | 9.27e-03 |
| ENSMUSG00000024193 | Phf1 | protein\_coding | 17:26933052-26937908 (+) |  | 0.519 | 1.32e-03 | 9.29e-03 |
| ENSMUSG00000030718 | Ppme1 | protein\_coding | 7:100326737-100372307 (-) |  | -0.274 | 1.33e-03 | 9.34e-03 |
| ENSMUSG00000060152 | Pop5 | protein\_coding | 5:115235836-115245351 (+) |  | -0.387 | 1.33e-03 | 9.34e-03 |
| ENSMUSG00000035725 | Prkx | protein\_coding | X:77761411-77796278 (-) |  | -0.351 | 1.34e-03 | 9.37e-03 |
| ENSMUSG00000040363 | Bcor | protein\_coding | X:12036740-12160355 (-) |  | 0.232 | 1.34e-03 | 9.39e-03 |
| ENSMUSG00000020077 | Srgn | protein\_coding | 10:62493833-62527451 (-) |  | -0.306 | 1.35e-03 | 9.46e-03 |
| ENSMUSG00000055302 | Mrfap1 | protein\_coding | 5:36794867-36796772 (-) |  | -0.200 | 1.36e-03 | 9.51e-03 |
| ENSMUSG00000031328 | Flna | protein\_coding | X:74223461-74249820 (-) |  | 0.233 | 1.36e-03 | 9.51e-03 |
| ENSMUSG00000102051 | Ly6a2 | transcribed\_unprocessed\_pseudogene | 15:75131377-75135128 (-) |  | 0.333 | 1.36e-03 | 9.52e-03 |
| ENSMUSG00000037815 | Ctnna1 | protein\_coding | 18:35118859-35254779 (+) |  | 0.258 | 1.36e-03 | 9.52e-03 |
| ENSMUSG00000072889 | Nfxl1 | protein\_coding | 5:72513301-72559684 (-) |  | 0.324 | 1.36e-03 | 9.53e-03 |
| ENSMUSG00000022216 | Psme1 | protein\_coding | 14:55578123-55581529 (+) |  | -0.224 | 1.36e-03 | 9.53e-03 |
| ENSMUSG00000061360 | Phf5a | protein\_coding | 15:81864520-81871911 (-) |  | -0.263 | 1.37e-03 | 9.55e-03 |
| ENSMUSG00000039221 | Rpl22l1 | protein\_coding | 3:28805436-28807424 (+) |  | -0.389 | 1.37e-03 | 9.55e-03 |
| ENSMUSG00000113204 | Gm46430 | lncRNA | 13:74579358-74617945 (-) |  | 0.370 | 1.37e-03 | 9.58e-03 |
| ENSMUSG00000097180 | 2700038G22Rik | lncRNA | 5:23850597-23855038 (+) |  | -0.718 | 1.38e-03 | 9.62e-03 |
| ENSMUSG00000024566 | Atp9b | protein\_coding | 18:80734141-80934060 (-) |  | 0.253 | 1.38e-03 | 9.63e-03 |
| ENSMUSG00000025499 | Hras | protein\_coding | 7:141189105-141194005 (-) |  | -0.312 | 1.38e-03 | 9.63e-03 |
| ENSMUSG00000041754 | Trem3 | protein\_coding | 17:48247777-48258841 (+) |  | -0.289 | 1.38e-03 | 9.63e-03 |
| ENSMUSG00000008855 | Hdac5 | protein\_coding | 11:102194432-102230166 (-) |  | 0.256 | 1.39e-03 | 9.65e-03 |
| ENSMUSG00000027715 | Ccna2 | protein\_coding | 3:36564865-36572150 (-) |  | -0.270 | 1.39e-03 | 9.67e-03 |
| ENSMUSG00000020638 | Cmpk2 | protein\_coding | 12:26469204-26479837 (+) |  | 0.301 | 1.39e-03 | 9.68e-03 |
| ENSMUSG00000022540 | Rogdi | protein\_coding | 16:5008730-5013553 (-) |  | 0.309 | 1.40e-03 | 9.68e-03 |
| ENSMUSG00000022574 | Naprt | protein\_coding | 15:75890956-75894481 (-) |  | -1.530 | 1.40e-03 | 9.72e-03 |
| ENSMUSG00000020864 | Ankrd40 | protein\_coding | 11:94328001-94341841 (+) |  | -0.208 | 1.40e-03 | 9.72e-03 |
| ENSMUSG00000040712 | Camta2 | protein\_coding | 11:70669463-70688105 (-) |  | 0.355 | 1.40e-03 | 9.72e-03 |
| ENSMUSG00000014551 | Mrps25 | protein\_coding | 6:92169525-92184033 (-) |  | -0.312 | 1.41e-03 | 9.76e-03 |
| ENSMUSG00000045216 | Hs6st1 | protein\_coding | 1:36068400-36106446 (+) |  | 0.314 | 1.41e-03 | 9.79e-03 |
| ENSMUSG00000021262 | Evl | protein\_coding | 12:108554720-108688516 (+) |  | 0.278 | 1.42e-03 | 9.80e-03 |
| ENSMUSG00000040591 | 1110051M20Rik | protein\_coding | 2:91275068-91444704 (-) |  | 0.594 | 1.42e-03 | 9.83e-03 |
| ENSMUSG00000039621 | Prex1 | protein\_coding | 2:166566342-166713832 (-) |  | 0.264 | 1.43e-03 | 9.90e-03 |
| ENSMUSG00000006818 | Sod2 | protein\_coding | 17:13006846-13040063 (+) |  | -0.260 | 1.45e-03 | 1.00e-02 |

| ID | Name | Type | Position | Image | logFC | p-Value | Adjusted p-Value |
| --- | --- | --- | --- | --- | --- | --- | --- |

(Page generated on Tue Aug 10 15:54:25 2021 by ReportingTools 2.28.0 and hwriter )
