# Supplementary material for: Transcriptome profiling of osteoclast subsets associated with arthritis: A pathogenic role of CCR2hi osteoclast progenitors
Source: Front Immunol. 2022 Dec 15;13:994035. doi: 10.3389/fimmu.2022.994035 (PMC9797520; doi:10.3389/fimmu.2022.994035)
Supplement: Supplementary file 14 [file DataSheet_6.zip › Supplementary data 6 GSEA CCR2lo vs CCR2hi all samples/Supplementary data 6 legend.docx]

Supplementary data 6. Downstream pathway analysis comparison of CCR2^lo^ with CCR2^hi^ osteoclast progenitor subset across all samples, regardless of intervention (both CIA and control mice).

To obtain possible functional insight from the high number of differentially expressed genes, gene set enrichment (GSE) analysis (GSEA) was performed by WEB-based GEne SeT AnaLysis Toolkit (WebGestalt) for an overrepresentation analysis on all genes of the pathways in the Kyoto Encyclopedia of Genes and Genomes (KEGG) database, with False Discovery Rate (FDR) threshold for pathways set at <0.05.

Upper part of the WebGestalt report displays a bar chart of the most differentially expressed gene sets between CCR2^lo^ and CCR2^hi^ osteoclast progenitor subsets. The bars visually denote normalized enrichment scores for significantly enriched downstream pathways in CCR2^hi^ (orange) or CCR2^lo^ (blue) OCP subsets in comparison with gene expression in all samples. Lower, or more negative, normalized enrichment scores indicate higher gene set expression in CCR2^hi^ osteoclast progenitor subset and vice-versa.

Middle part feature a gene set or pathway selection box, from which one of detected significantly changed pathways can be selected. GSE plot for the selected significant pathway is displayed. The line in the top plot represents the running enrichment score for a given pathway as the analysis goes down the ranked list. The value at the peak denotes the final enrichment score. The middle plot shows where the genes related to the pathway are located in the ranking. Genes that appear before the positive enrichment score, or after the negative enrichment score, represent the leading edge subset. The lower plot shows the distribution of the ranking metric along the list of the ranked genes. Genes in the leading edge with highest enrichment scores that fit the proposed criteria are listed in curly brackets.

This section also notes basic data for the selected pathway – p and FDR values of the pathway's difference in expression, total number of genes in the patway (size), as well as number of genes in the leading edge, and pathway's enrichment score.

The bottom part feature a table of all the genes included in the pathway, denoting their Ensembl ID (User ID), gene name and symbol, as well as gene's respective enrichment score. The table can be sorted by click a column's heading.

WebGestalt report is accessed using the provided .html file, while the project folder contains accessory files needed for .html functionality.
