# Supplementary figures and images for: Transcriptome profiling of osteoclast subsets associated with arthritis: A pathogenic role of CCR2hi osteoclast progenitors (part 2 of 2)
Source: Front Immunol. 2022 Dec 15;13:994035. doi: 10.3389/fimmu.2022.994035 (PMC9797520; doi:10.3389/fimmu.2022.994035)

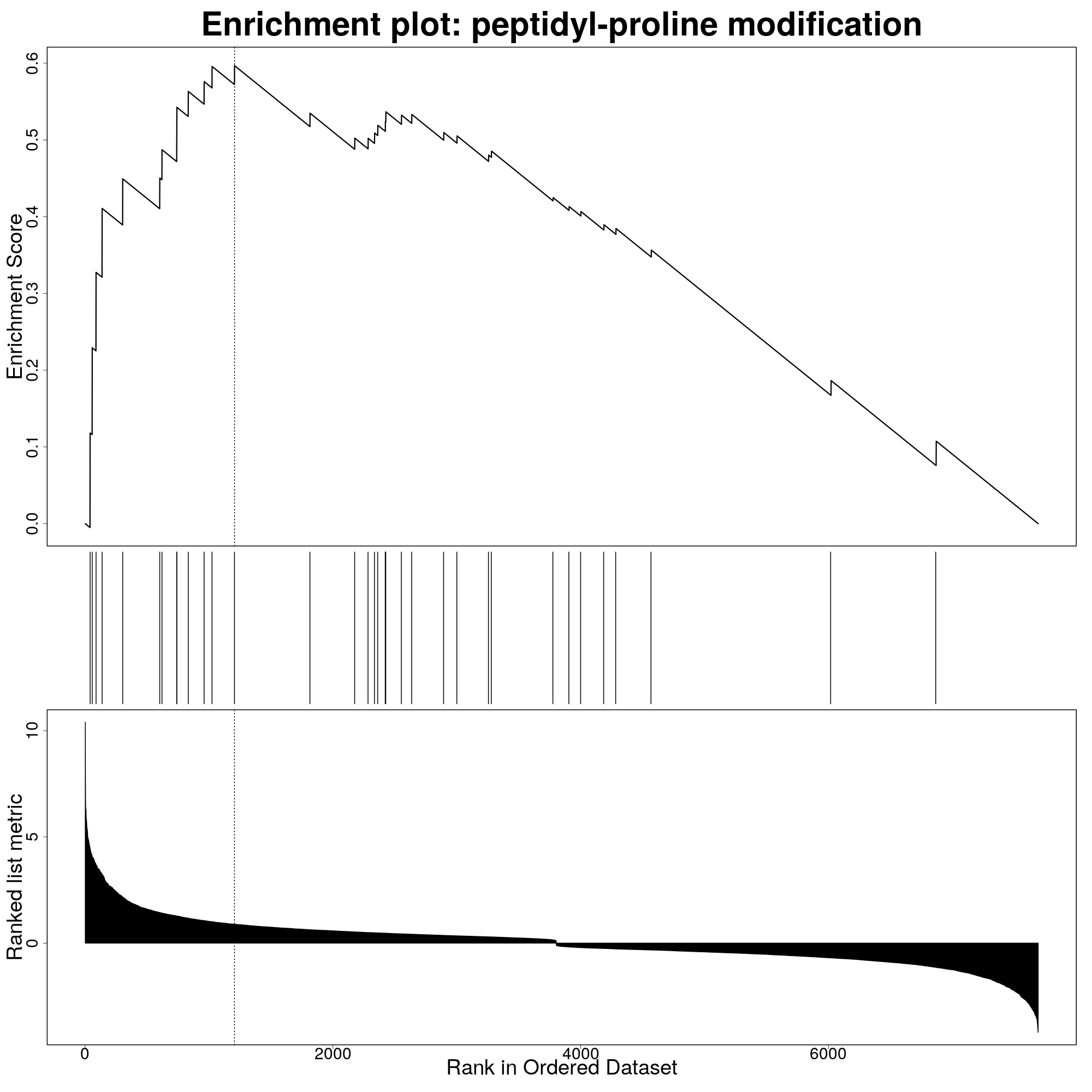

Supplement: Supplementary file 15 [file DataSheet_7.zip › Supplementary data 7 GSEA CCR2lo vs CCR2hi in CIA/Project_high_vs_low_GSEA/GO_0018208.png]

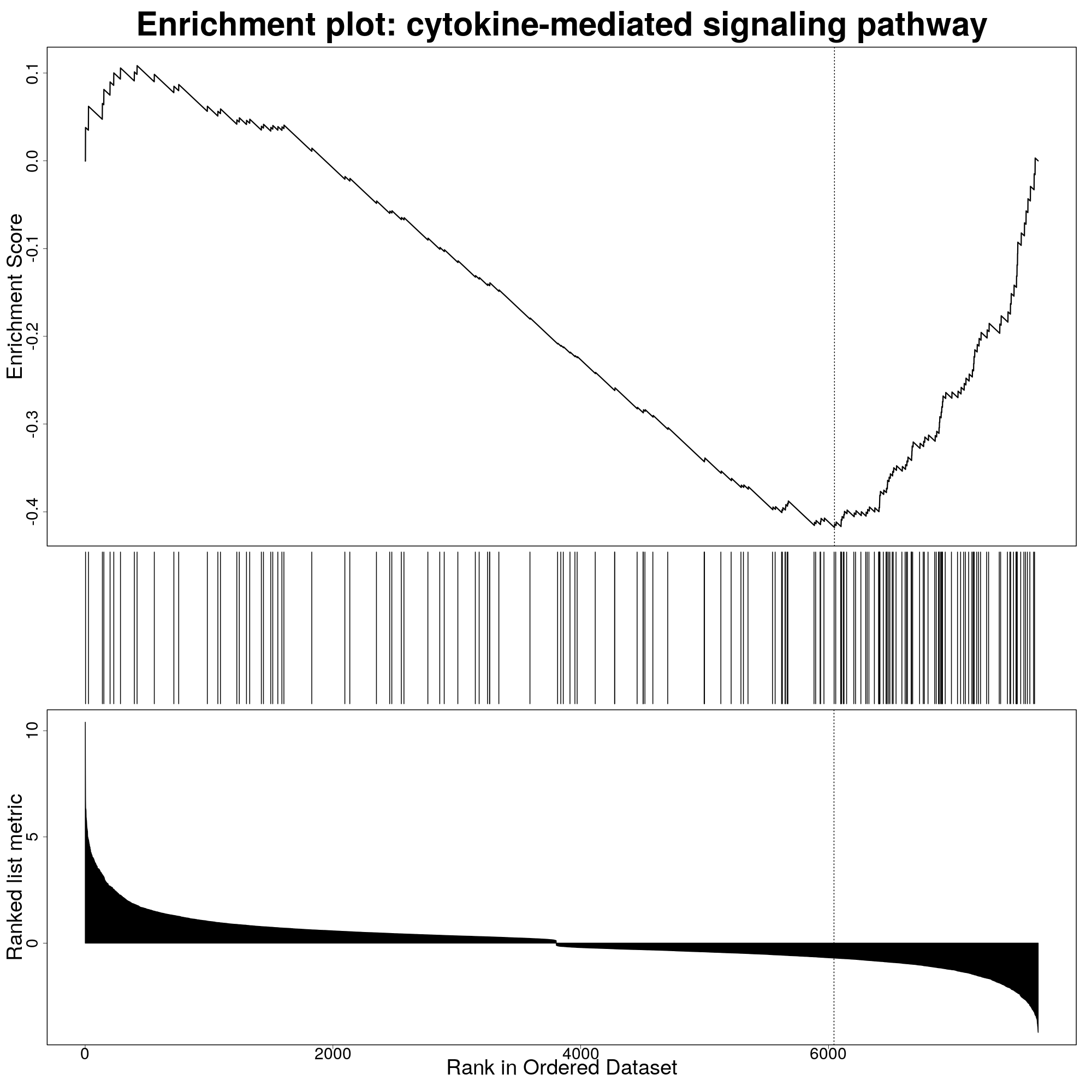

Supplement: Supplementary file 15 [file DataSheet_7.zip › Supplementary data 7 GSEA CCR2lo vs CCR2hi in CIA/Project_high_vs_low_GSEA/GO_0019221.png]

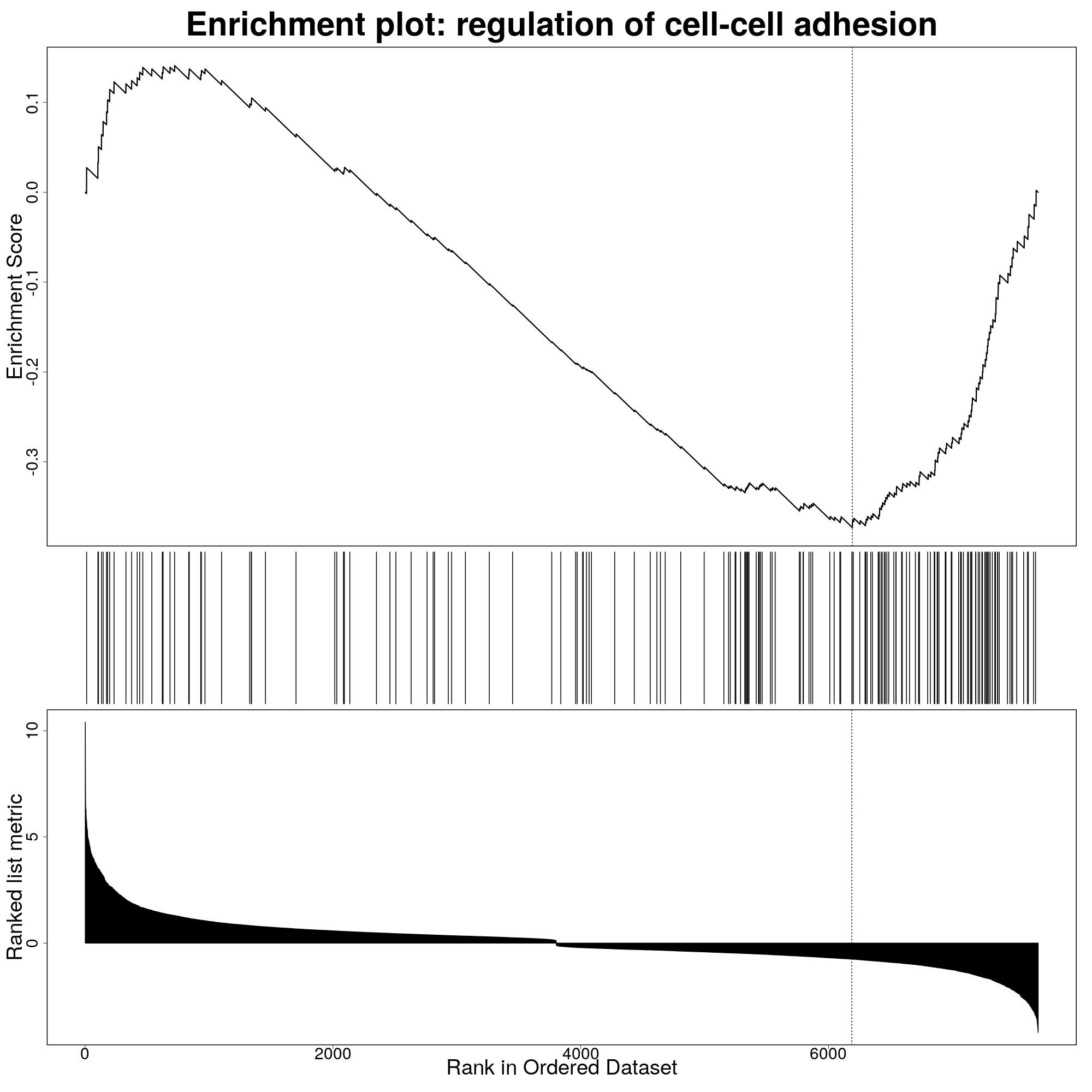

Supplement: Supplementary file 15 [file DataSheet_7.zip › Supplementary data 7 GSEA CCR2lo vs CCR2hi in CIA/Project_high_vs_low_GSEA/GO_0022407.png]

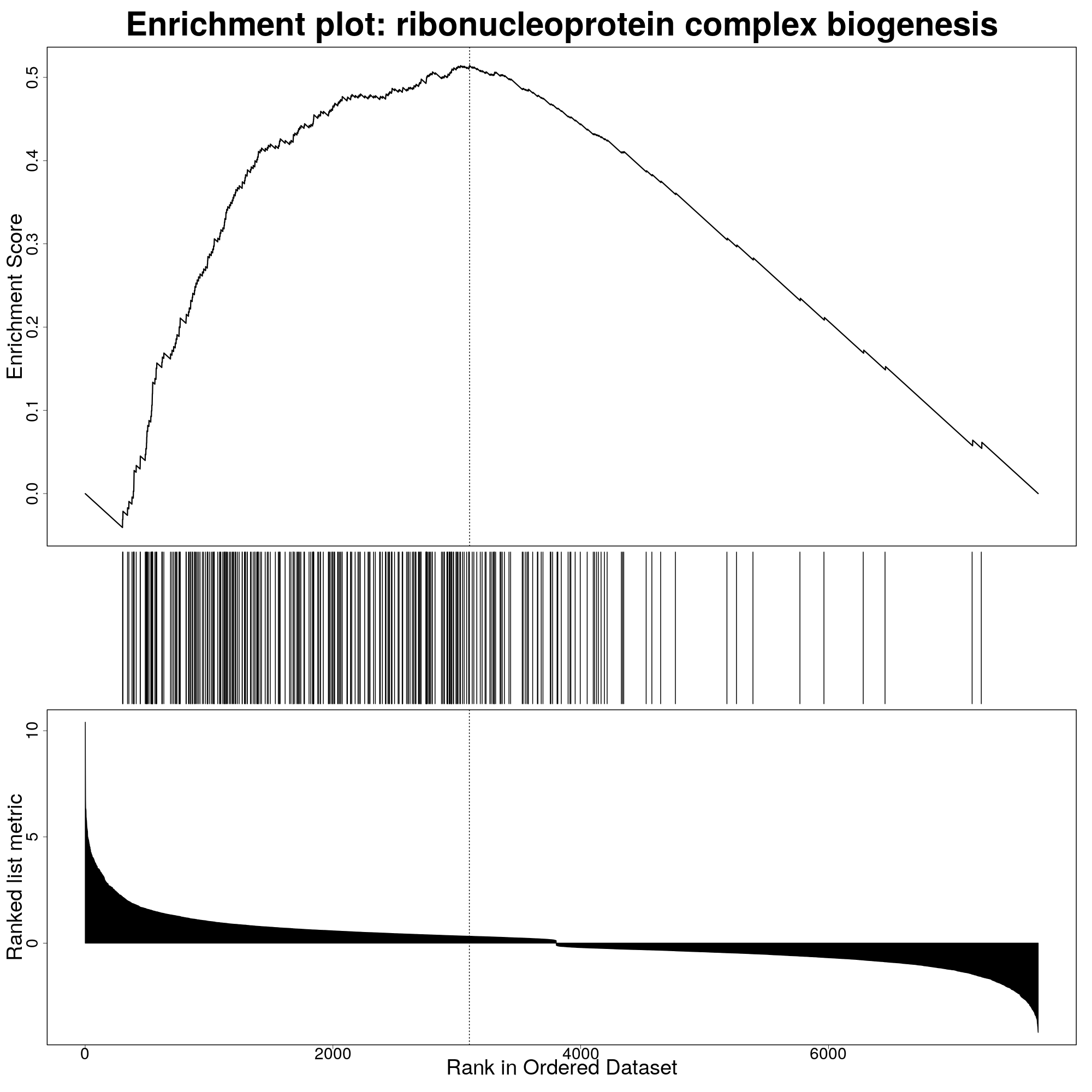

Supplement: Supplementary file 15 [file DataSheet_7.zip › Supplementary data 7 GSEA CCR2lo vs CCR2hi in CIA/Project_high_vs_low_GSEA/GO_0022613.png]

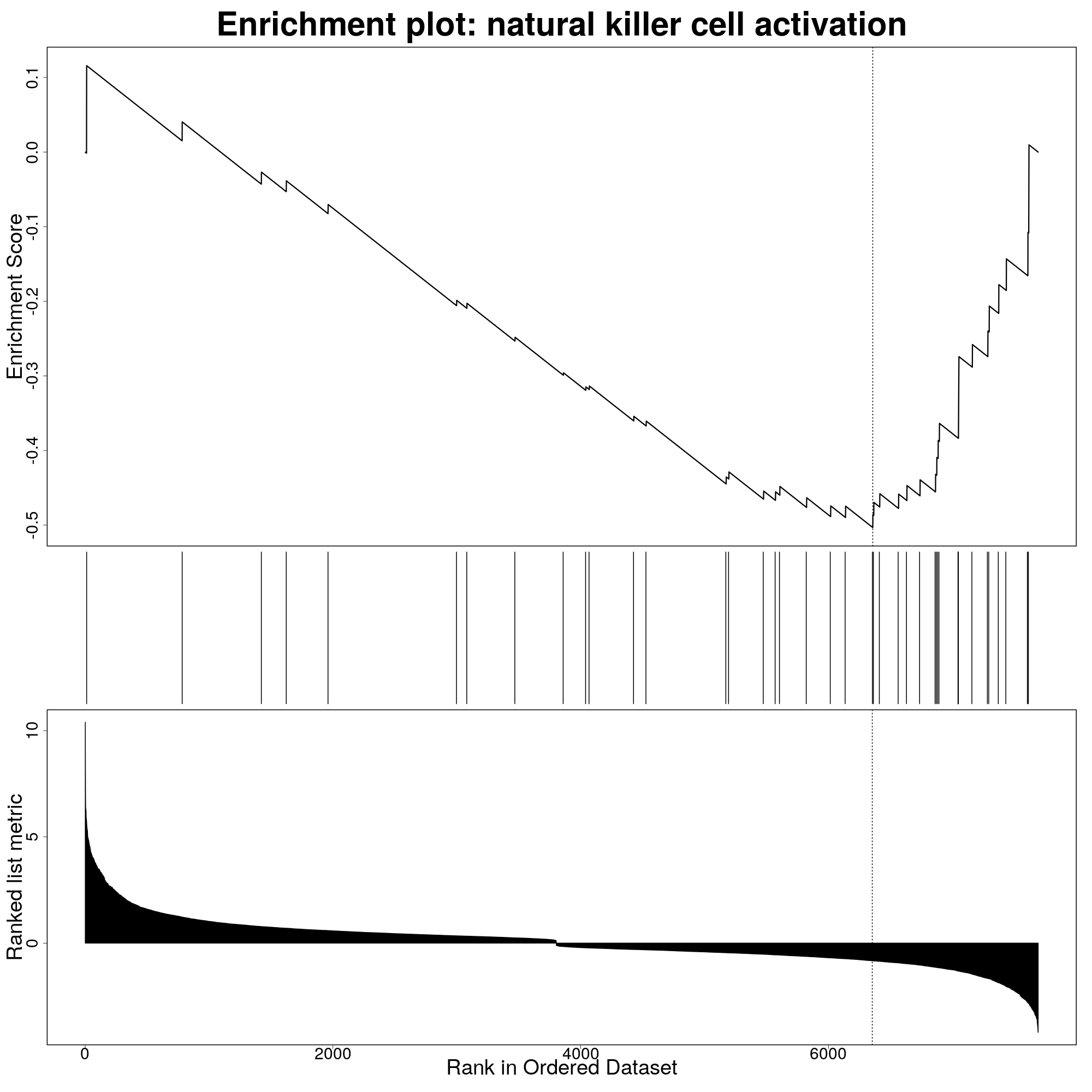

Supplement: Supplementary file 15 [file DataSheet_7.zip › Supplementary data 7 GSEA CCR2lo vs CCR2hi in CIA/Project_high_vs_low_GSEA/GO_0030101.png]

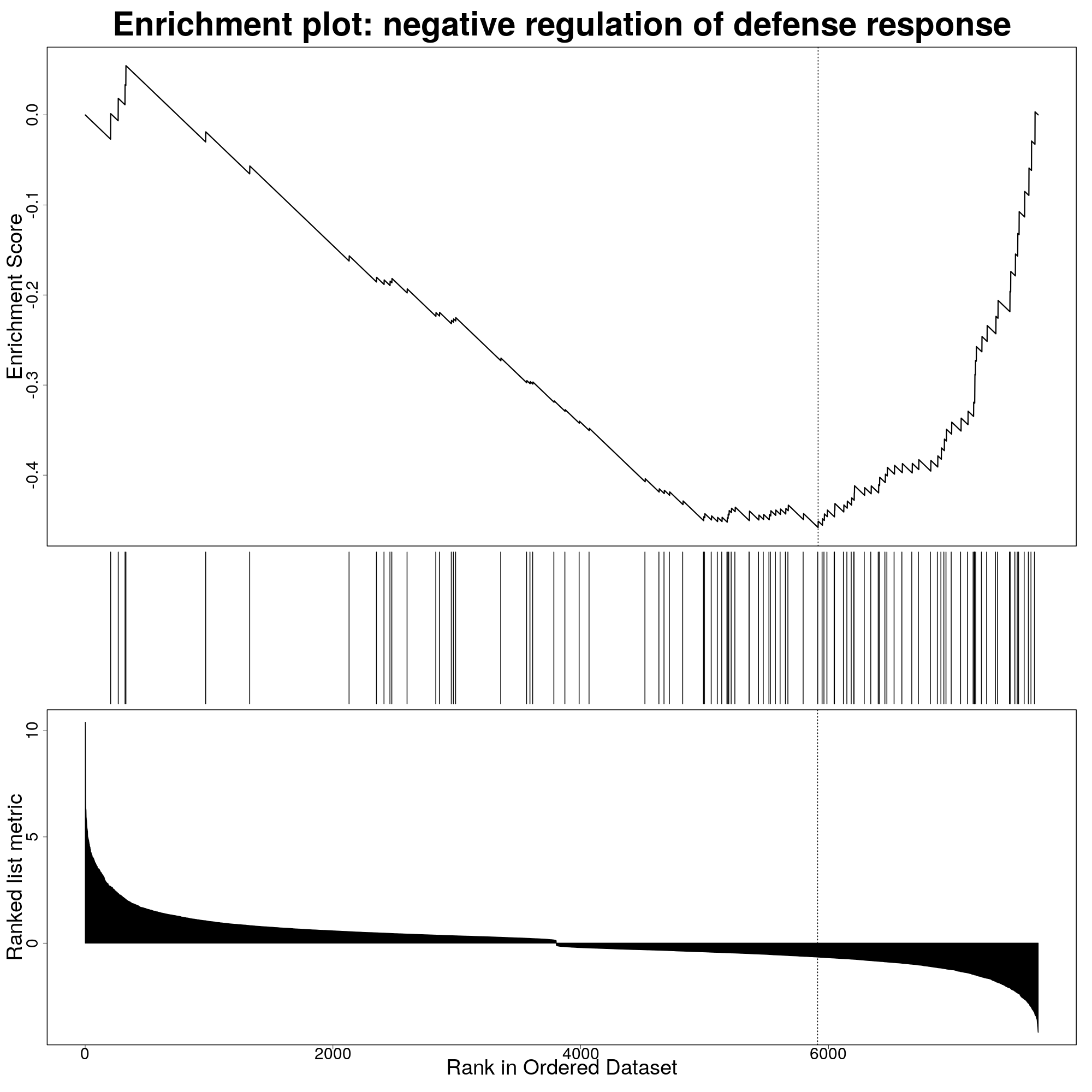

Supplement: Supplementary file 15 [file DataSheet_7.zip › Supplementary data 7 GSEA CCR2lo vs CCR2hi in CIA/Project_high_vs_low_GSEA/GO_0031348.png]

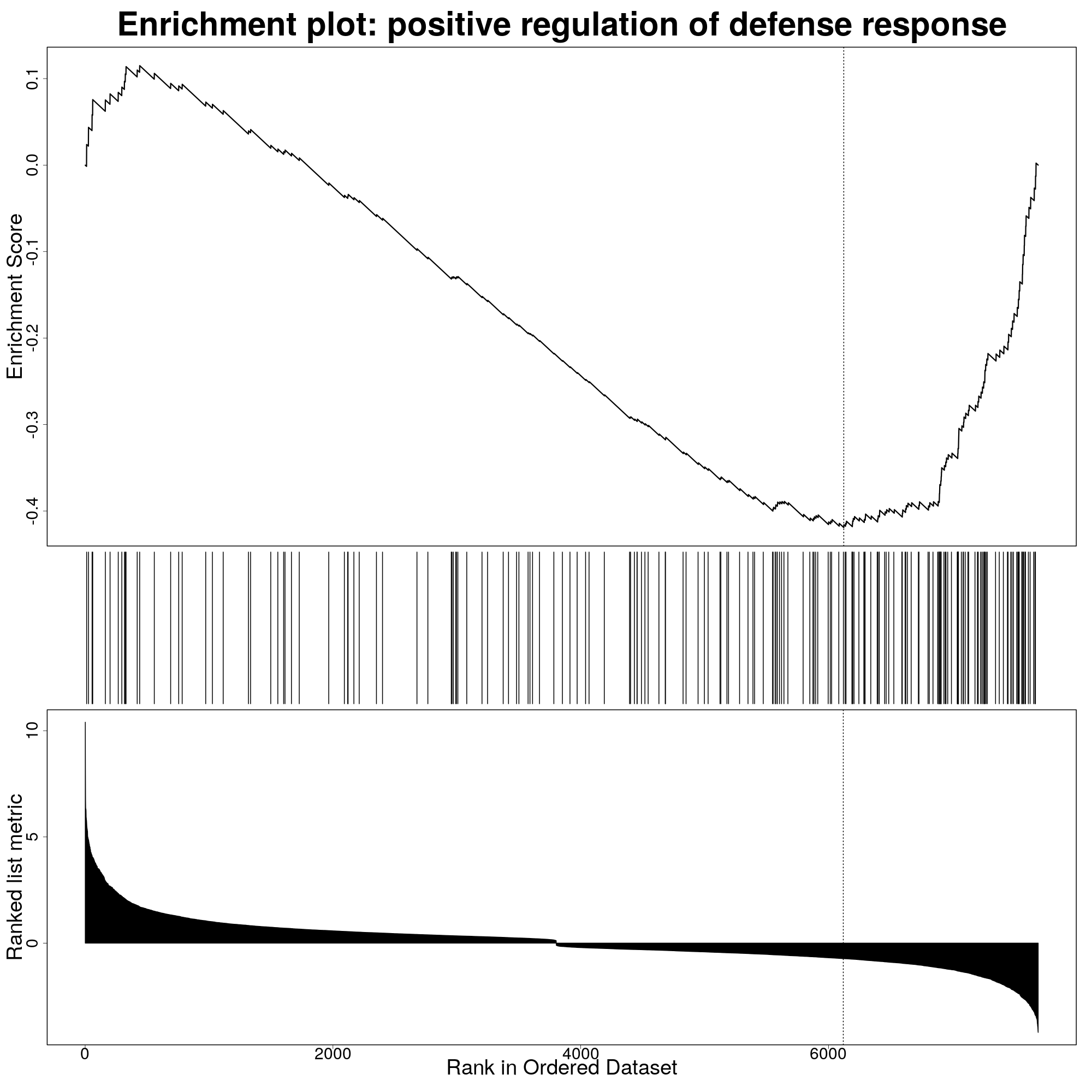

Supplement: Supplementary file 15 [file DataSheet_7.zip › Supplementary data 7 GSEA CCR2lo vs CCR2hi in CIA/Project_high_vs_low_GSEA/GO_0031349.png]

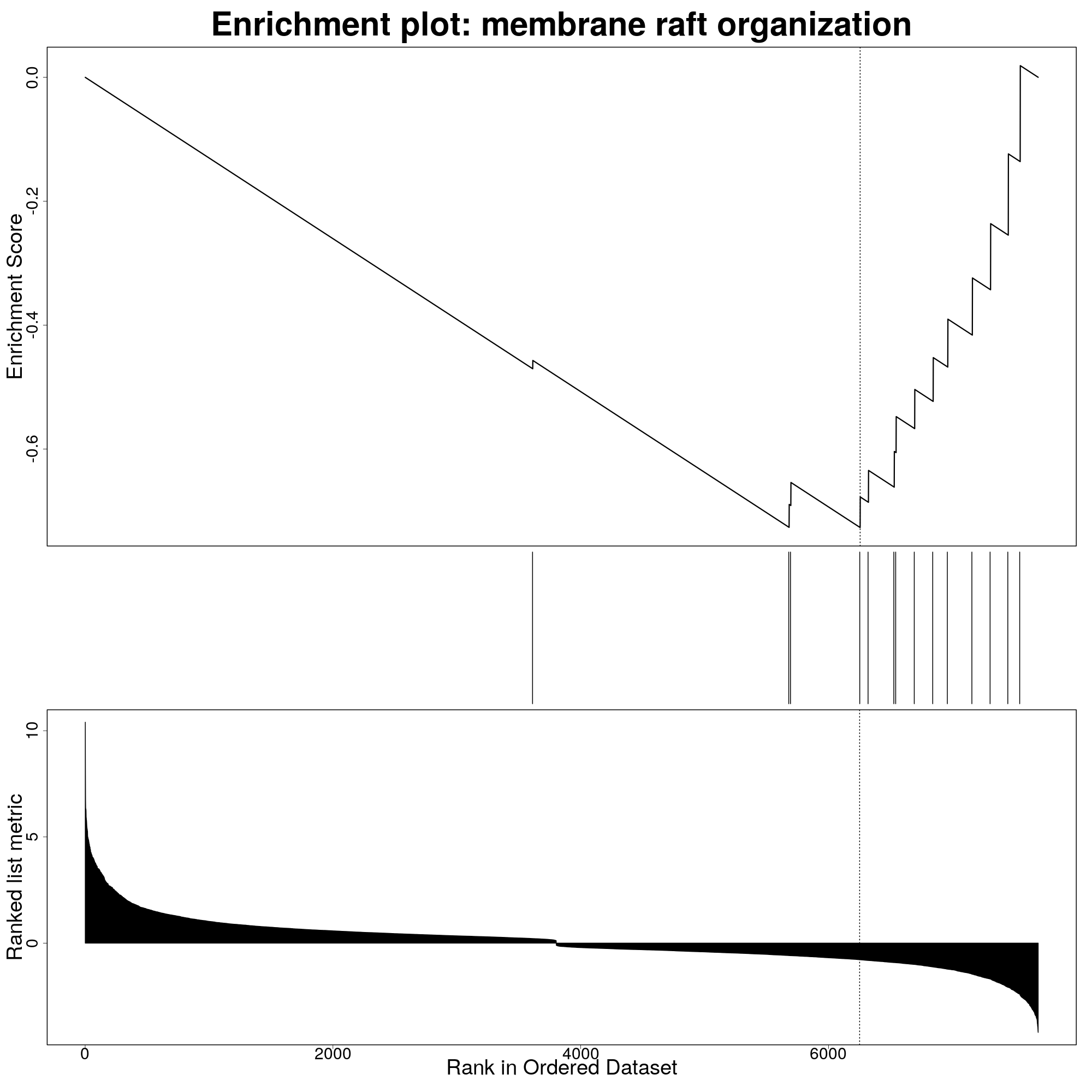

Supplement: Supplementary file 15 [file DataSheet_7.zip › Supplementary data 7 GSEA CCR2lo vs CCR2hi in CIA/Project_high_vs_low_GSEA/GO_0031579.png]

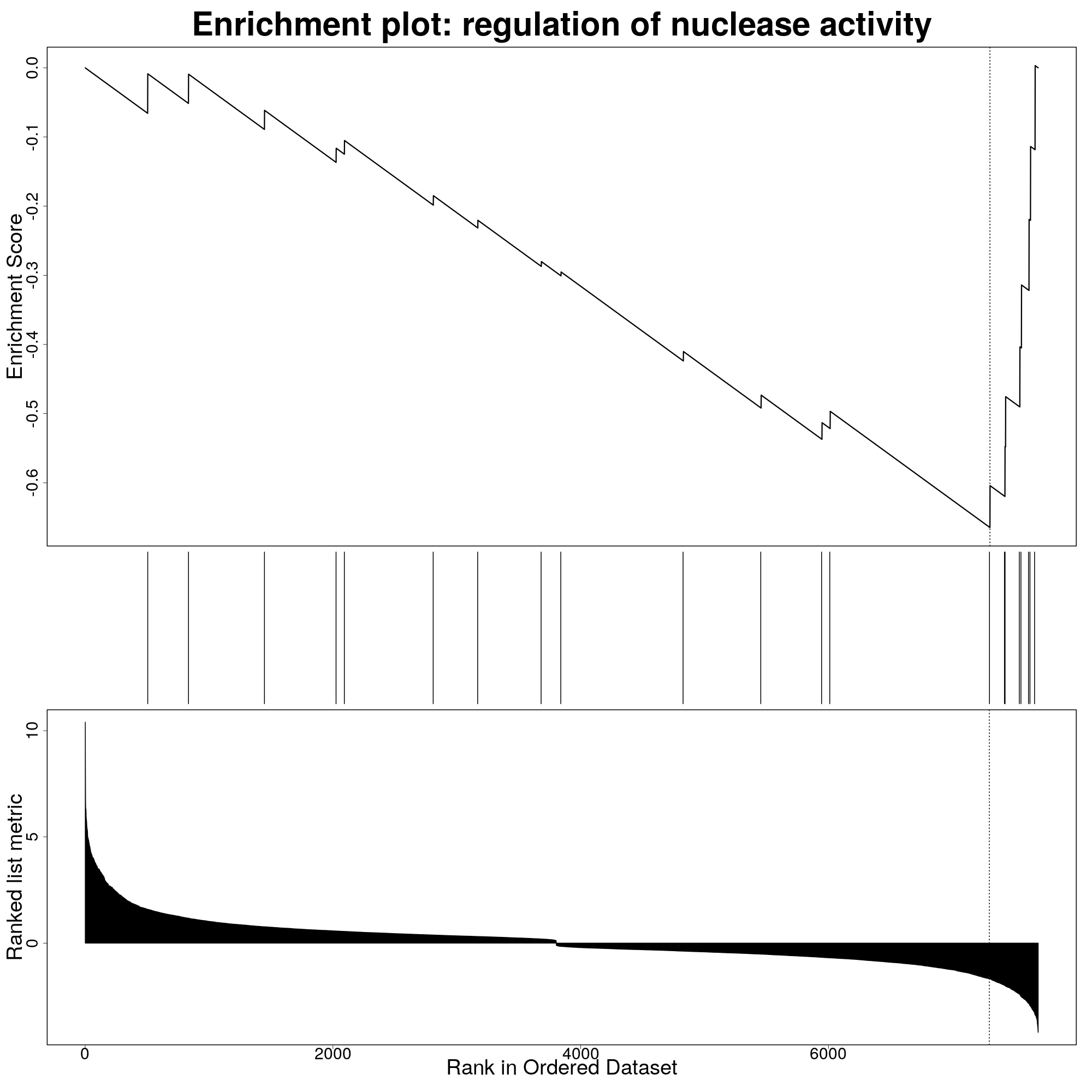

Supplement: Supplementary file 15 [file DataSheet_7.zip › Supplementary data 7 GSEA CCR2lo vs CCR2hi in CIA/Project_high_vs_low_GSEA/GO_0032069.png]

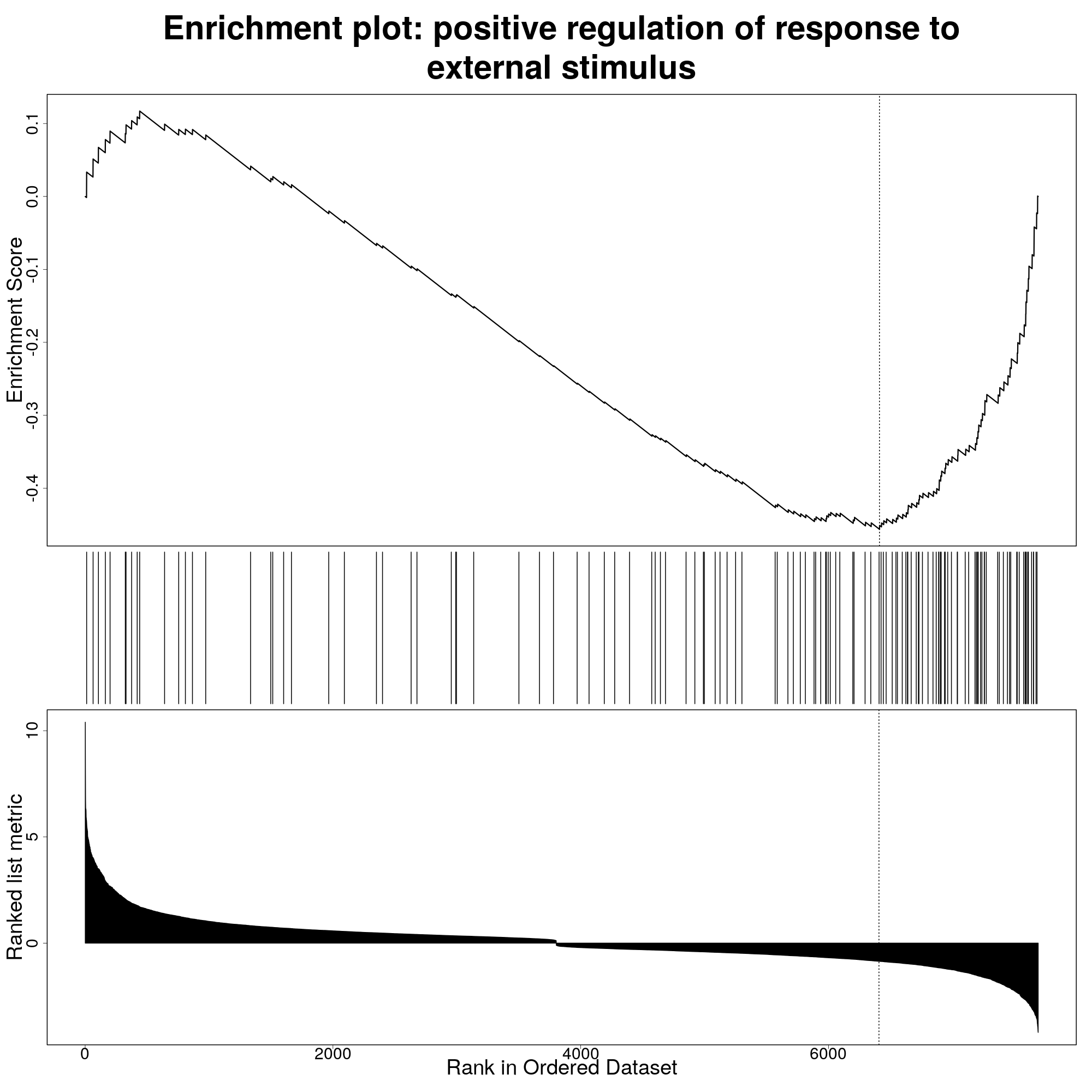

Supplement: Supplementary file 15 [file DataSheet_7.zip › Supplementary data 7 GSEA CCR2lo vs CCR2hi in CIA/Project_high_vs_low_GSEA/GO_0032103.png]

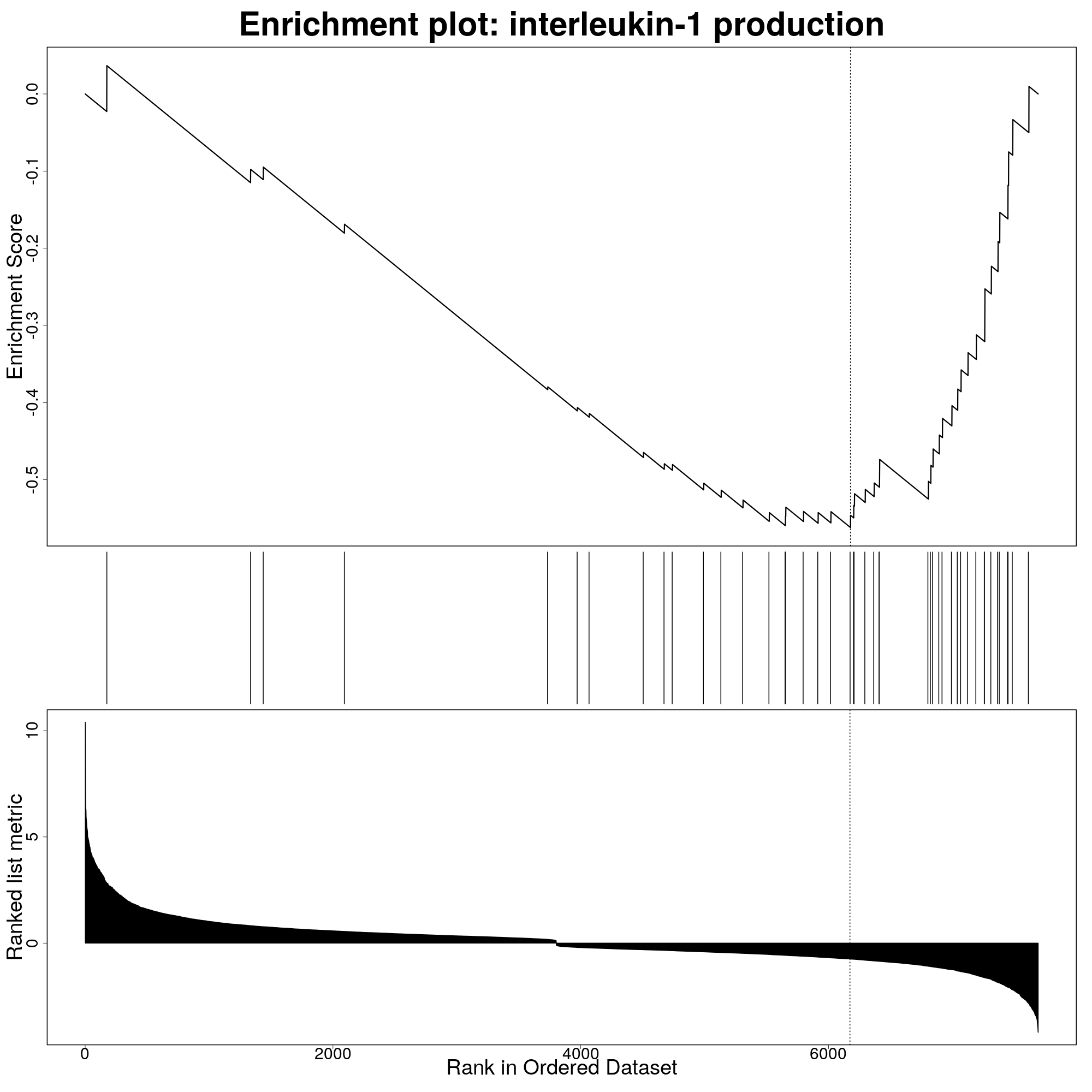

Supplement: Supplementary file 15 [file DataSheet_7.zip › Supplementary data 7 GSEA CCR2lo vs CCR2hi in CIA/Project_high_vs_low_GSEA/GO_0032612.png]

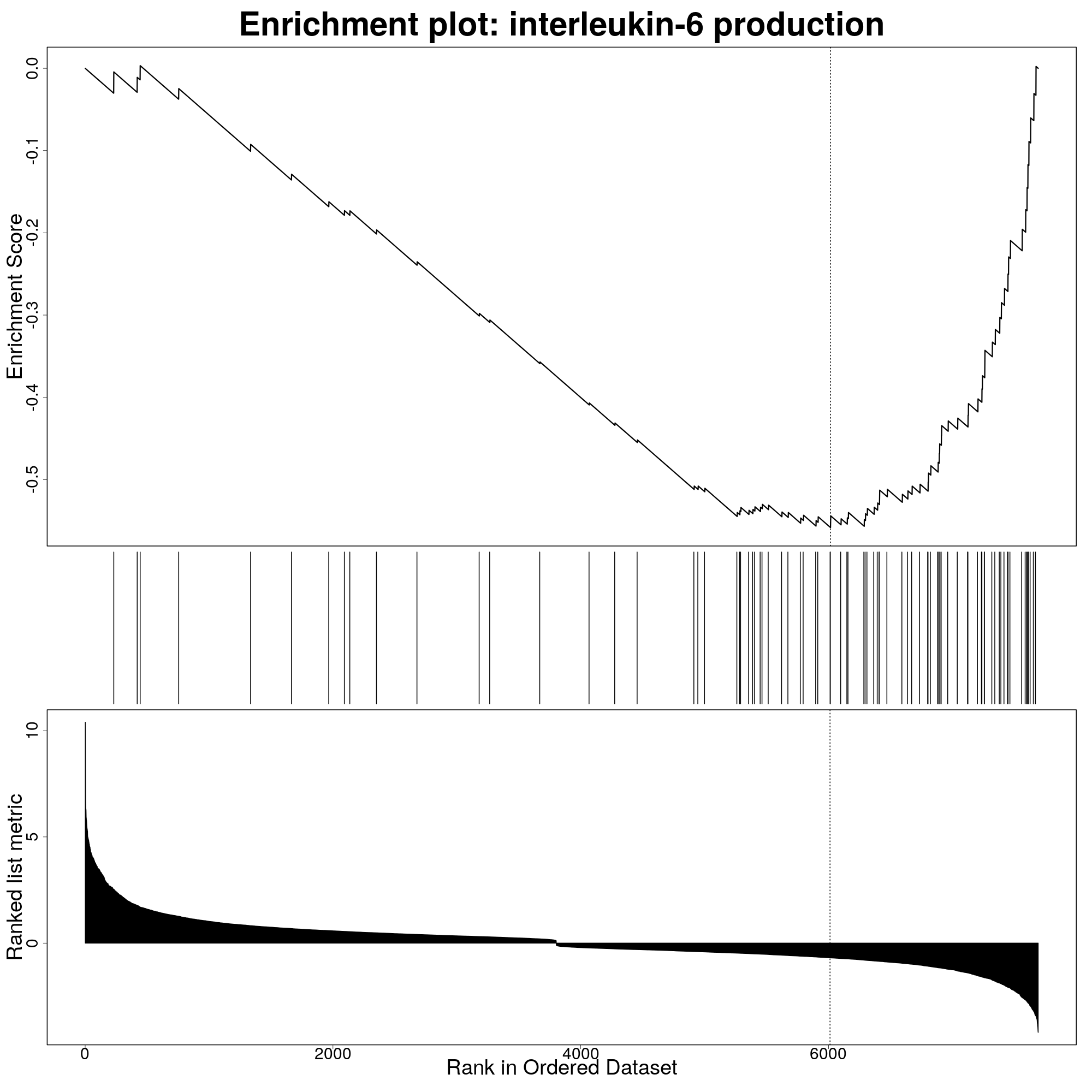

Supplement: Supplementary file 15 [file DataSheet_7.zip › Supplementary data 7 GSEA CCR2lo vs CCR2hi in CIA/Project_high_vs_low_GSEA/GO_0032635.png]

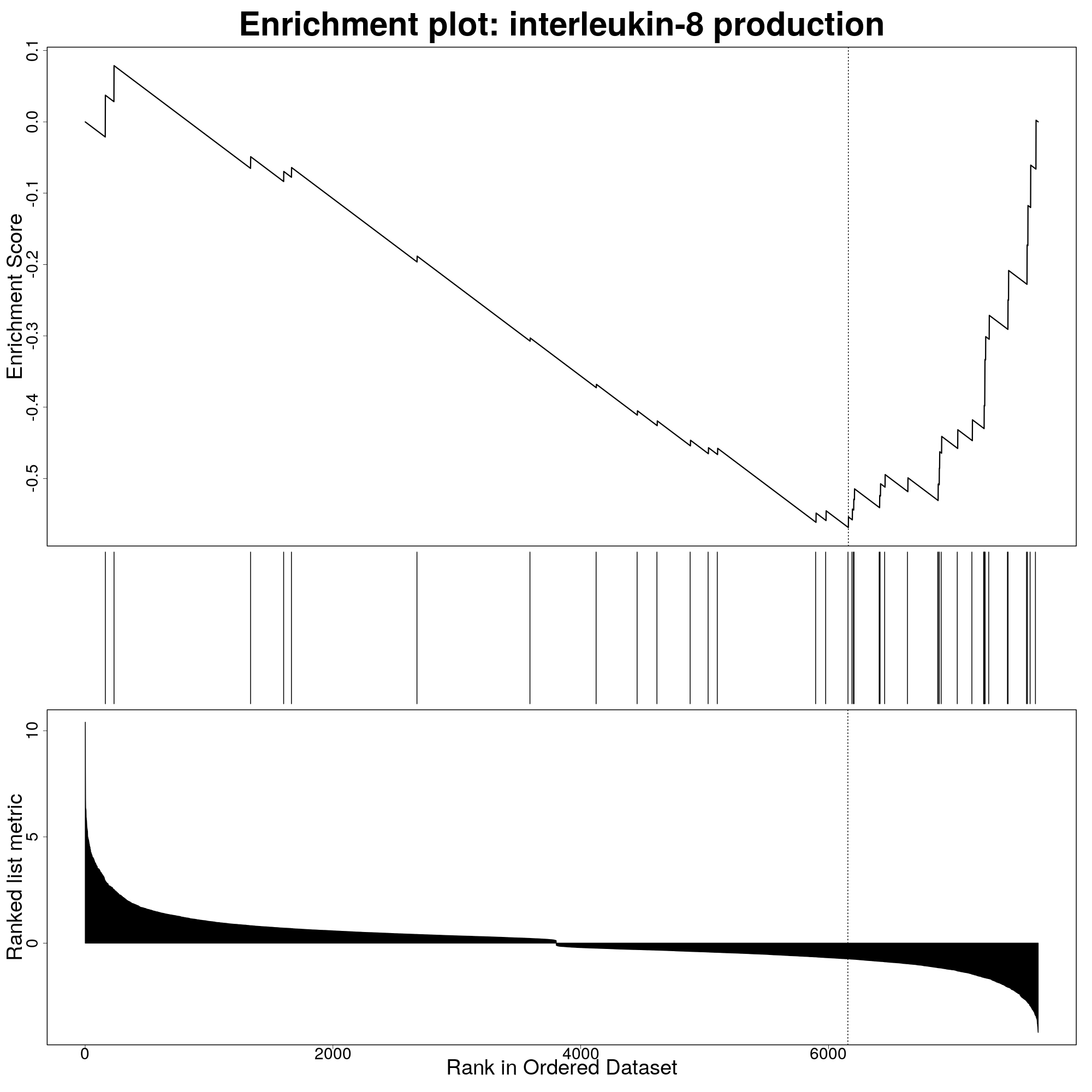

Supplement: Supplementary file 15 [file DataSheet_7.zip › Supplementary data 7 GSEA CCR2lo vs CCR2hi in CIA/Project_high_vs_low_GSEA/GO_0032637.png]

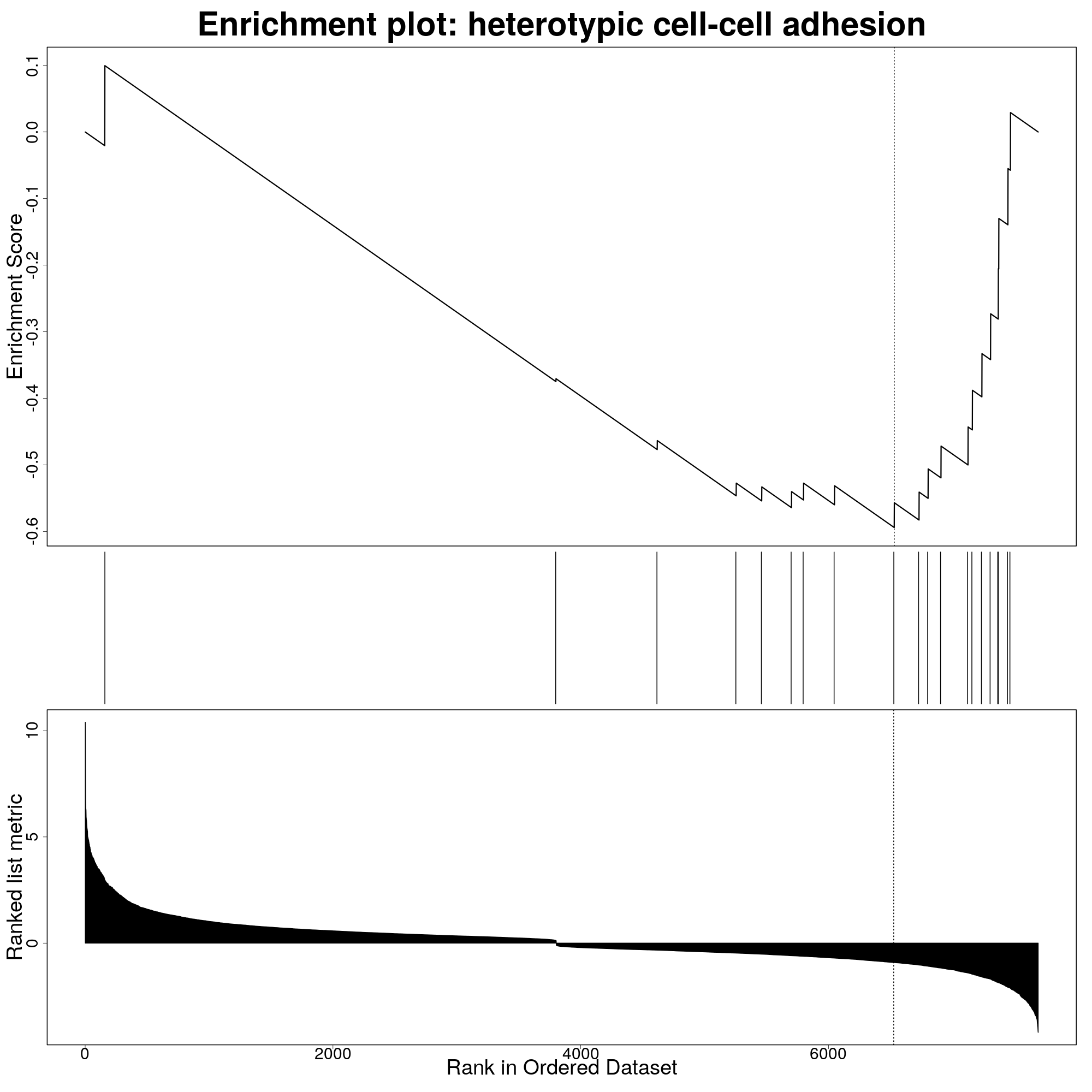

Supplement: Supplementary file 15 [file DataSheet_7.zip › Supplementary data 7 GSEA CCR2lo vs CCR2hi in CIA/Project_high_vs_low_GSEA/GO_0034113.png]

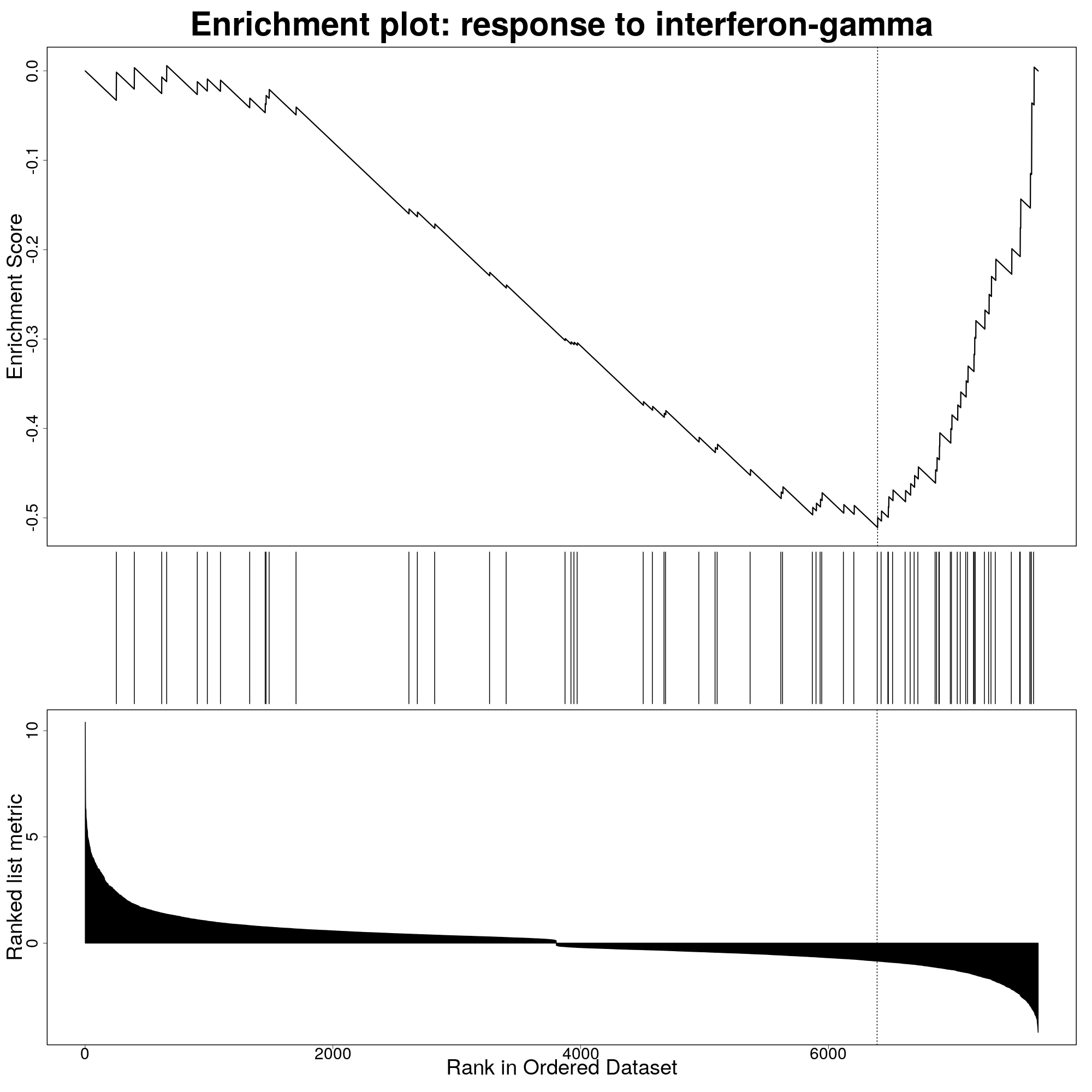

Supplement: Supplementary file 15 [file DataSheet_7.zip › Supplementary data 7 GSEA CCR2lo vs CCR2hi in CIA/Project_high_vs_low_GSEA/GO_0034341.png]

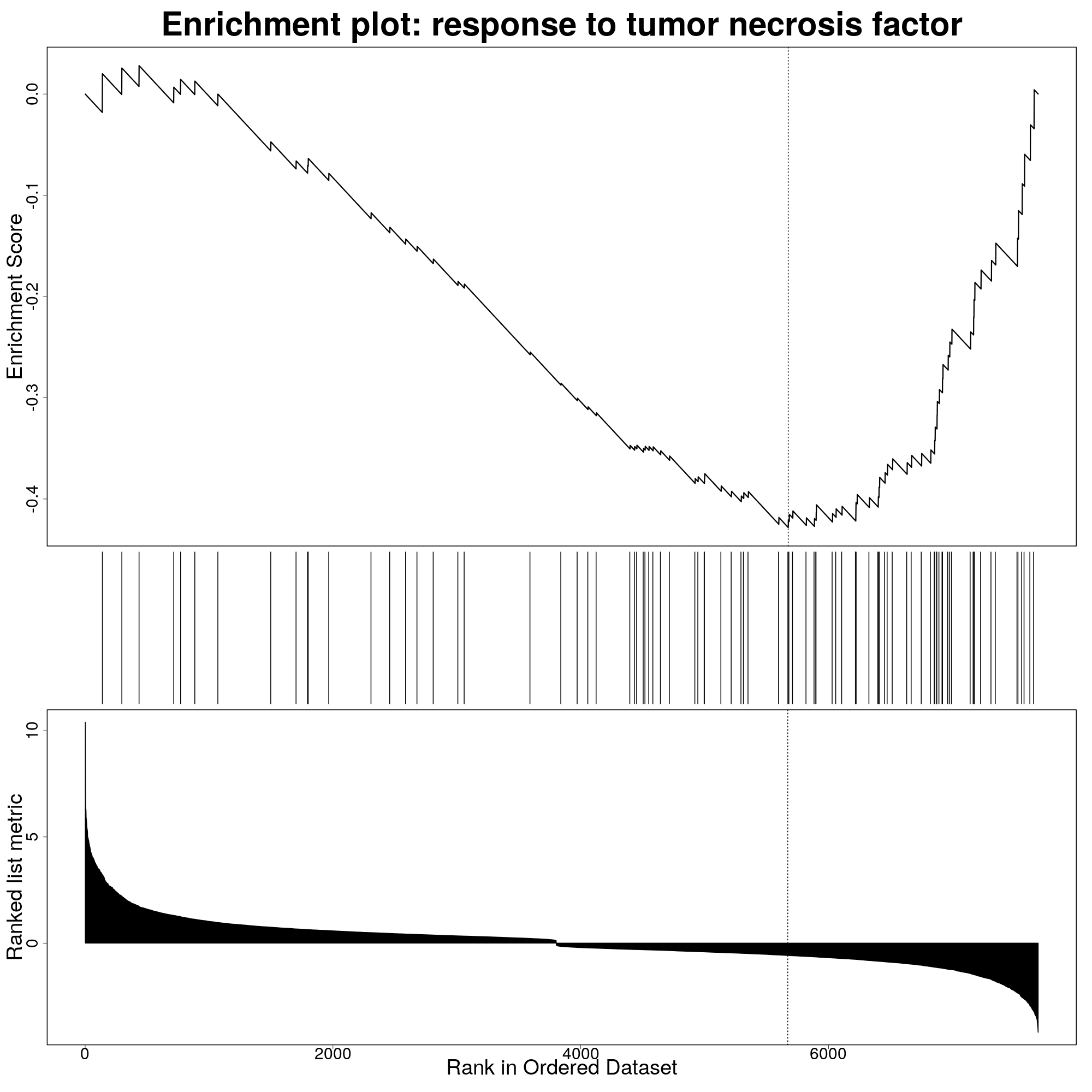

Supplement: Supplementary file 15 [file DataSheet_7.zip › Supplementary data 7 GSEA CCR2lo vs CCR2hi in CIA/Project_high_vs_low_GSEA/GO_0034612.png]

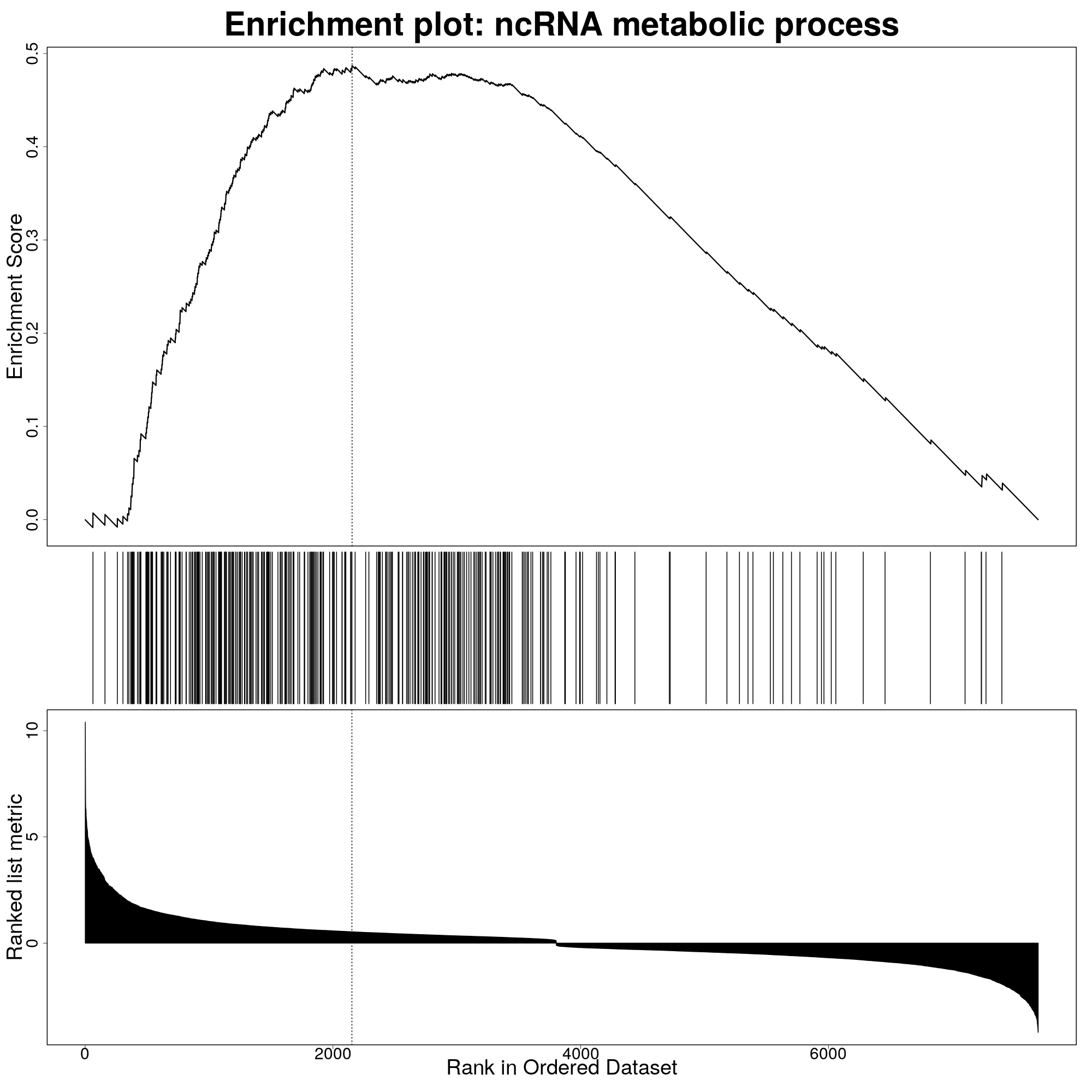

Supplement: Supplementary file 15 [file DataSheet_7.zip › Supplementary data 7 GSEA CCR2lo vs CCR2hi in CIA/Project_high_vs_low_GSEA/GO_0034660.png]

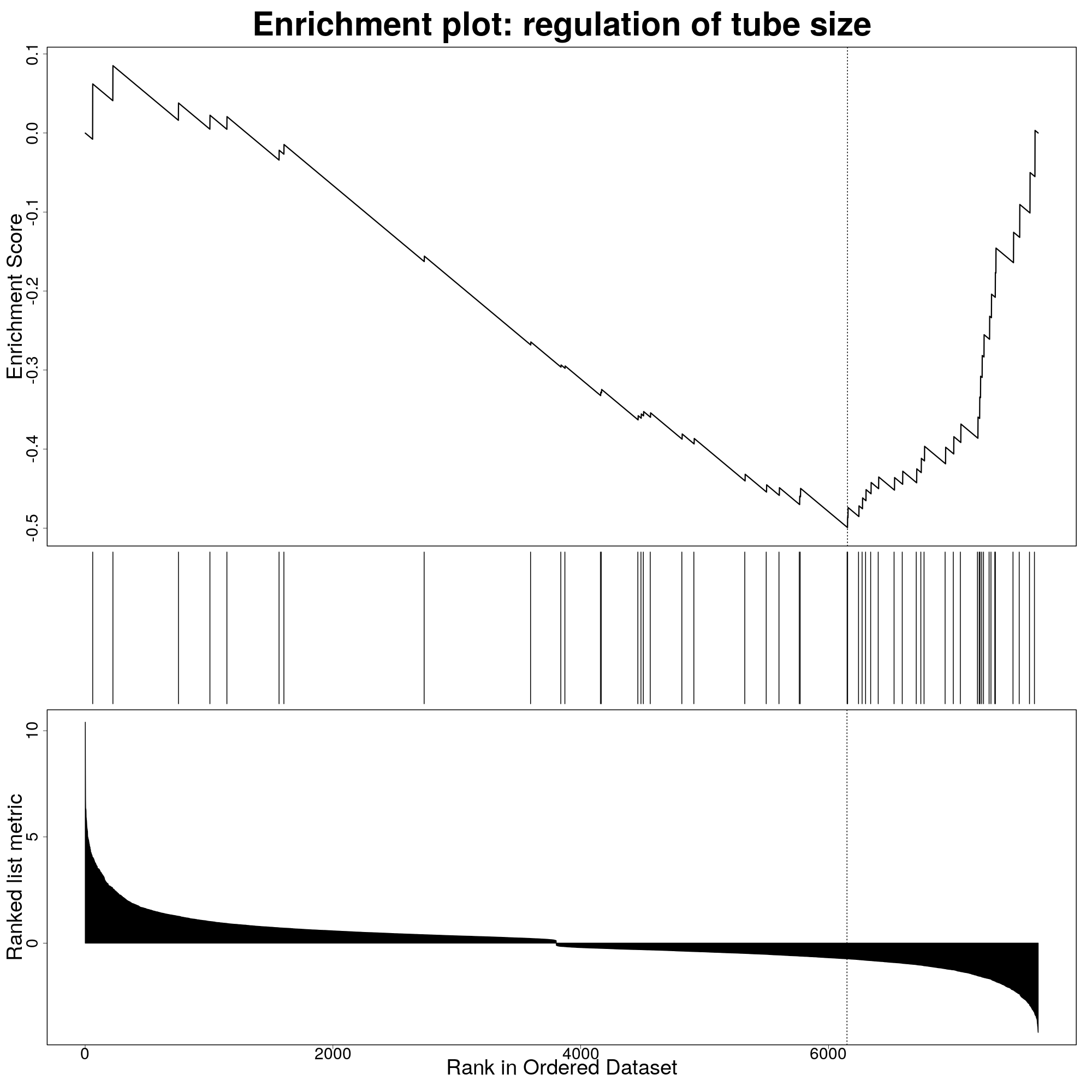

Supplement: Supplementary file 15 [file DataSheet_7.zip › Supplementary data 7 GSEA CCR2lo vs CCR2hi in CIA/Project_high_vs_low_GSEA/GO_0035150.png]

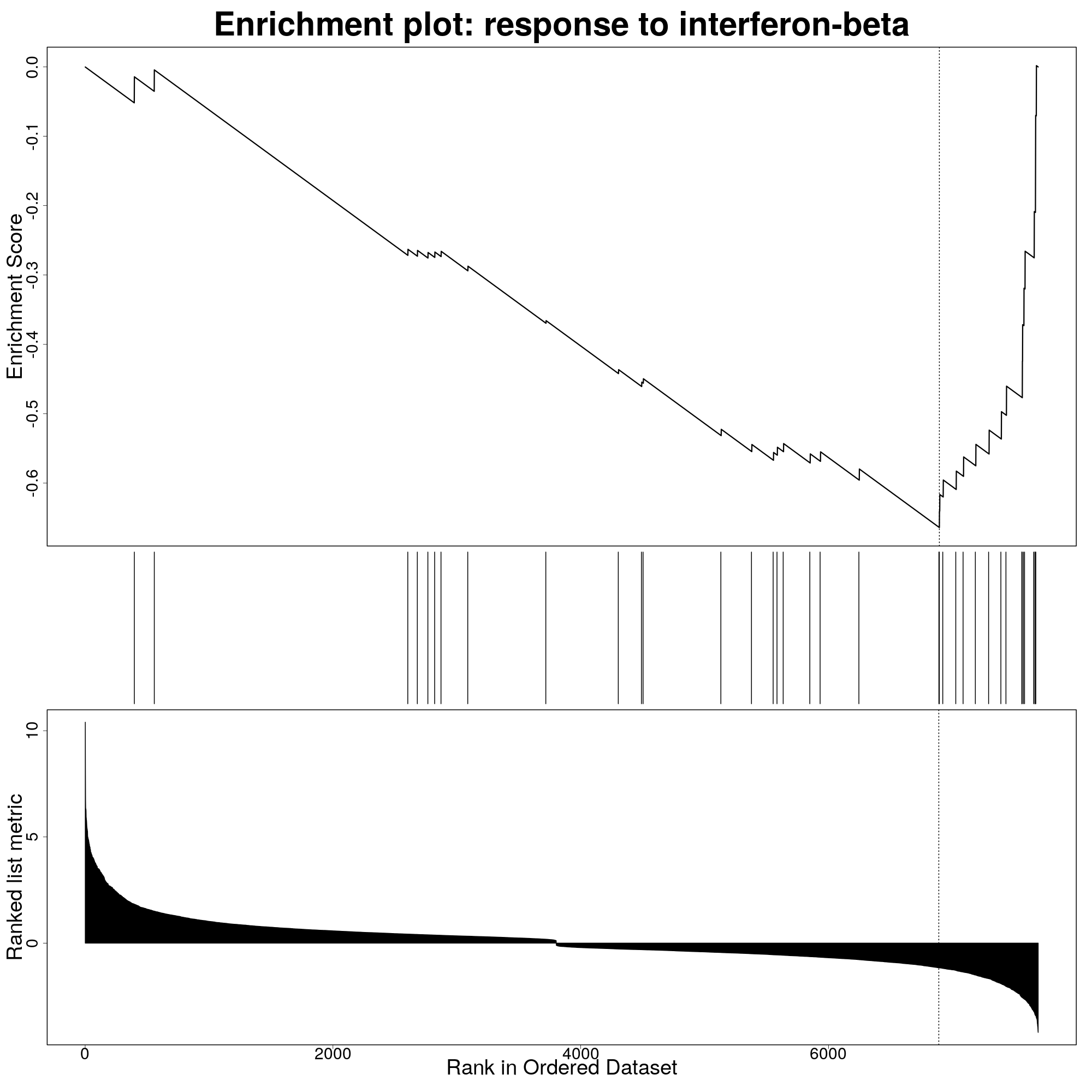

Supplement: Supplementary file 15 [file DataSheet_7.zip › Supplementary data 7 GSEA CCR2lo vs CCR2hi in CIA/Project_high_vs_low_GSEA/GO_0035456.png]

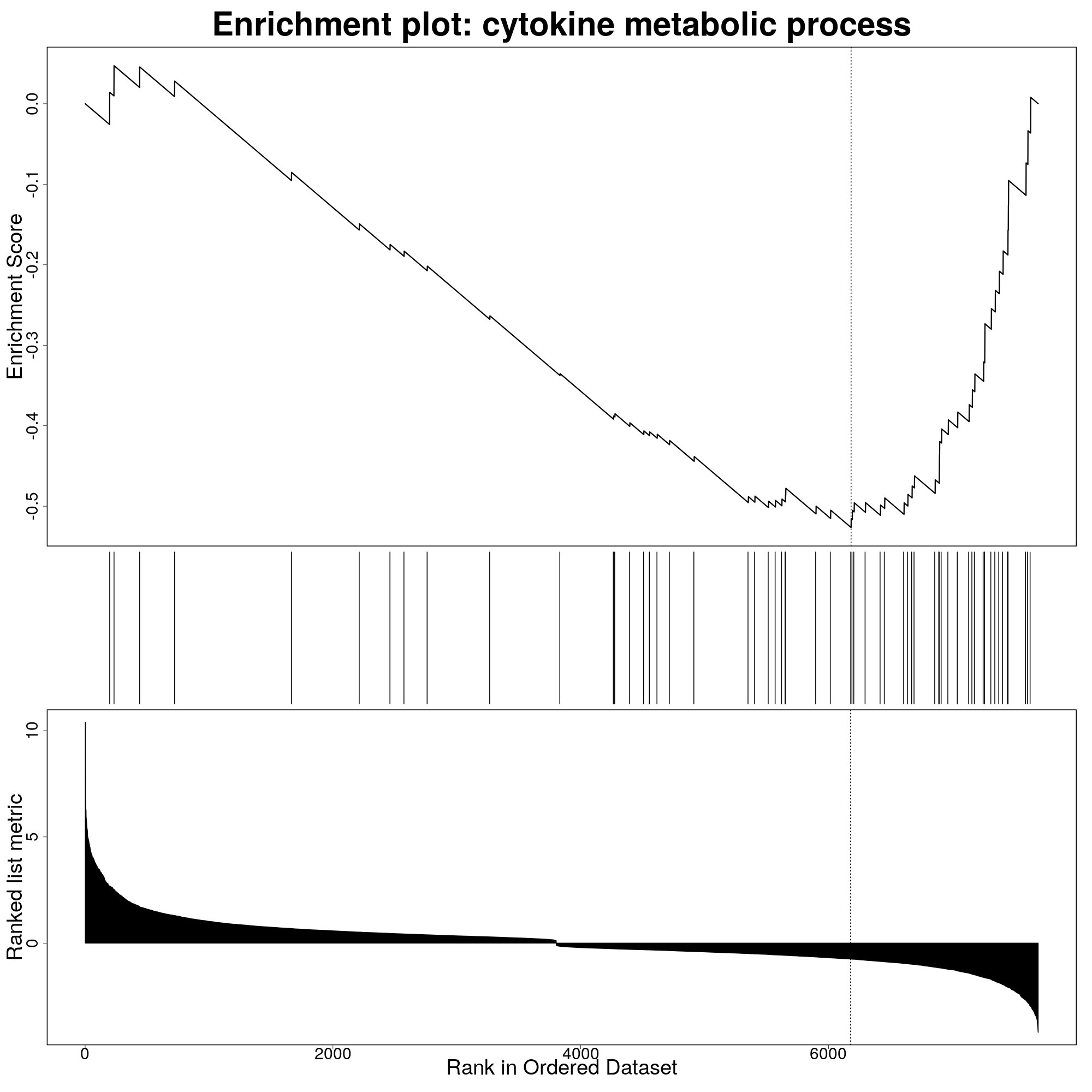

Supplement: Supplementary file 15 [file DataSheet_7.zip › Supplementary data 7 GSEA CCR2lo vs CCR2hi in CIA/Project_high_vs_low_GSEA/GO_0042107.png]

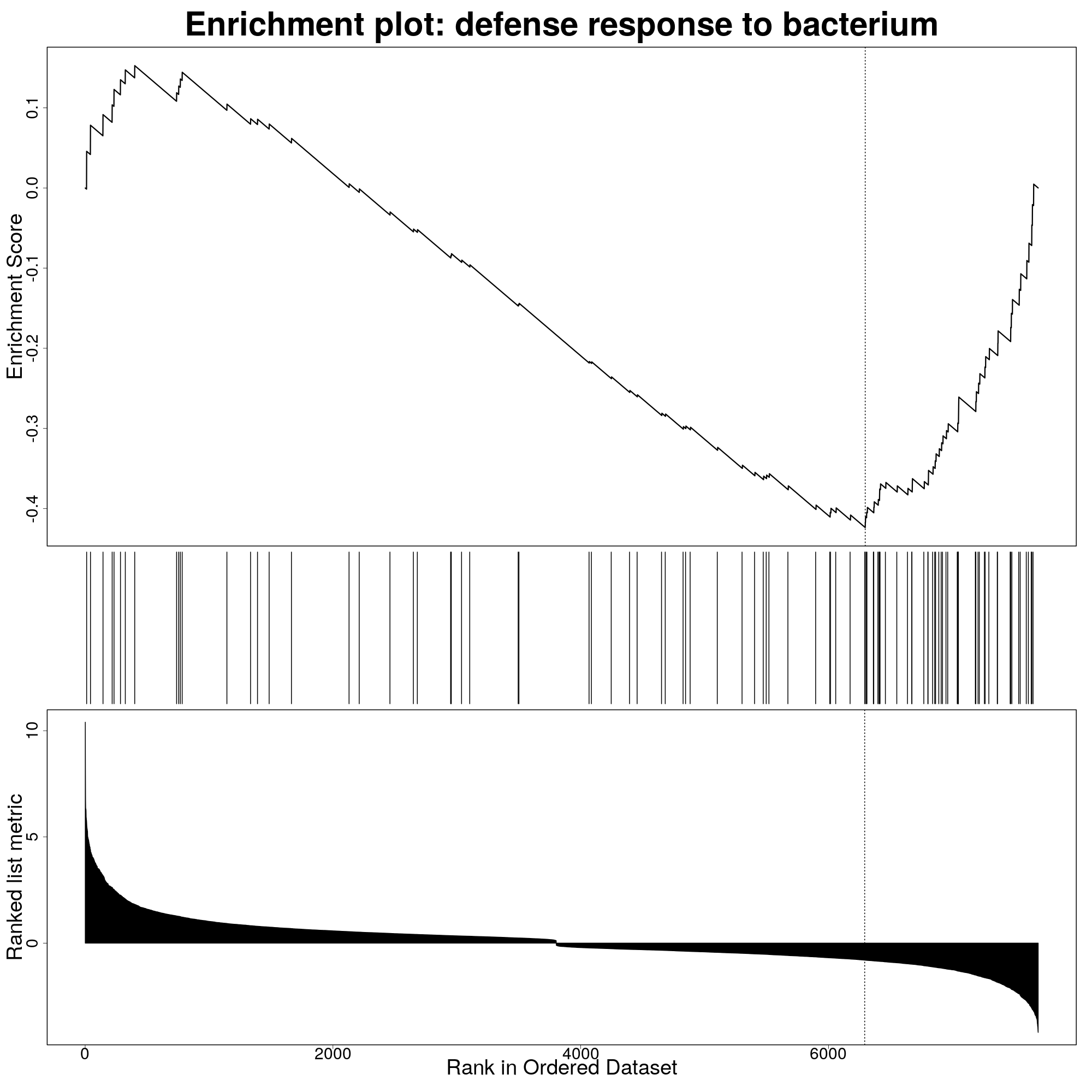

Supplement: Supplementary file 15 [file DataSheet_7.zip › Supplementary data 7 GSEA CCR2lo vs CCR2hi in CIA/Project_high_vs_low_GSEA/GO_0042742.png]

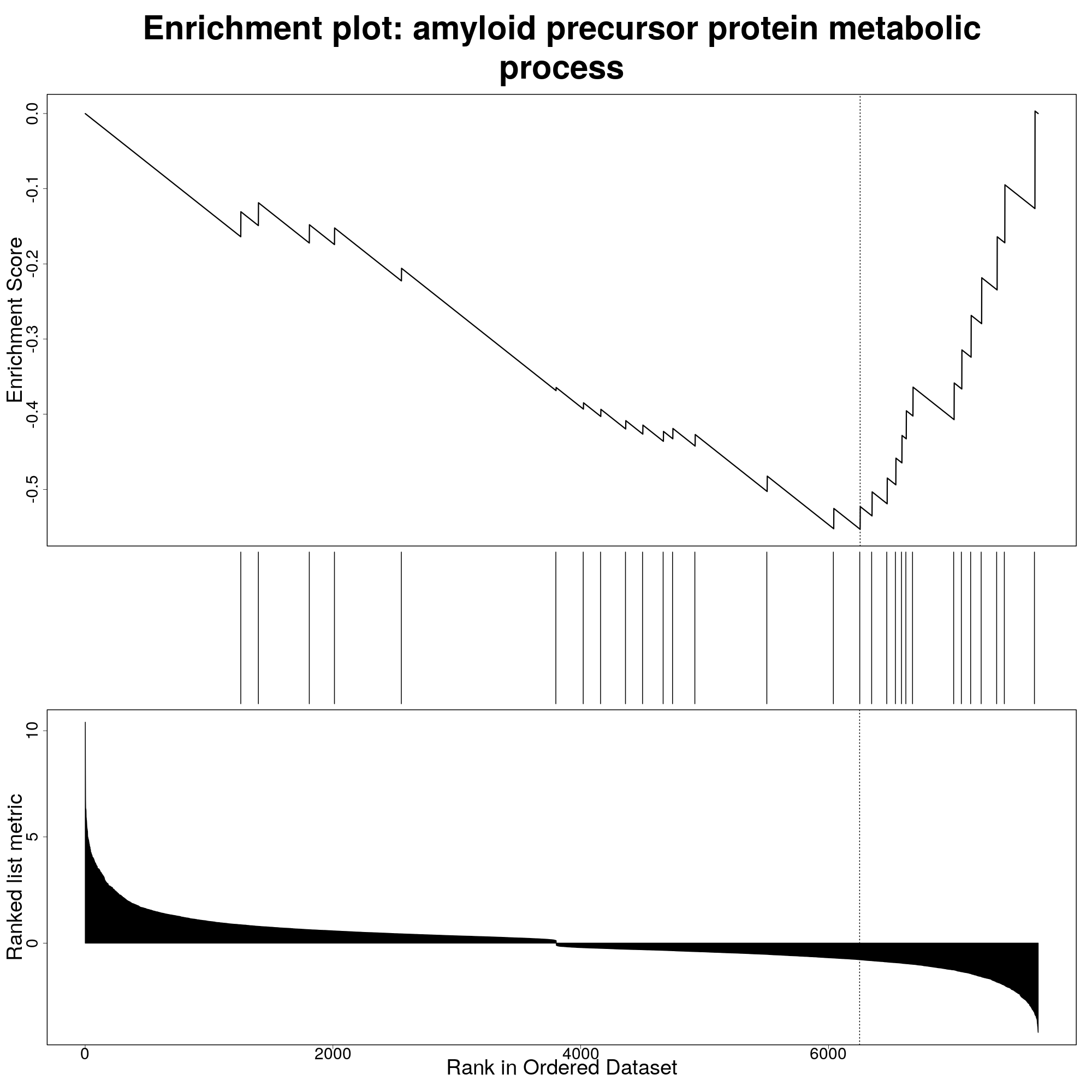

Supplement: Supplementary file 15 [file DataSheet_7.zip › Supplementary data 7 GSEA CCR2lo vs CCR2hi in CIA/Project_high_vs_low_GSEA/GO_0042982.png]

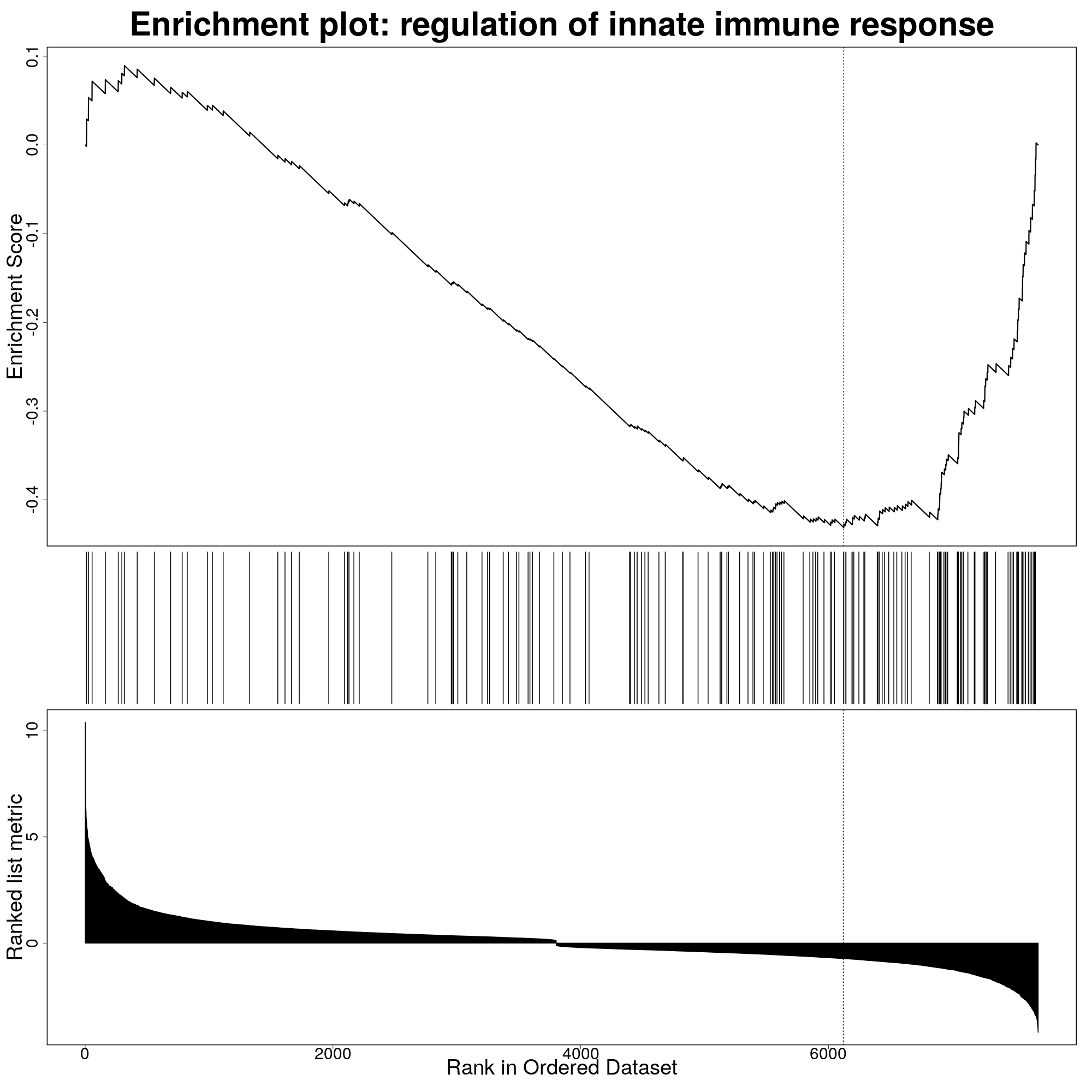

Supplement: Supplementary file 15 [file DataSheet_7.zip › Supplementary data 7 GSEA CCR2lo vs CCR2hi in CIA/Project_high_vs_low_GSEA/GO_0045088.png]

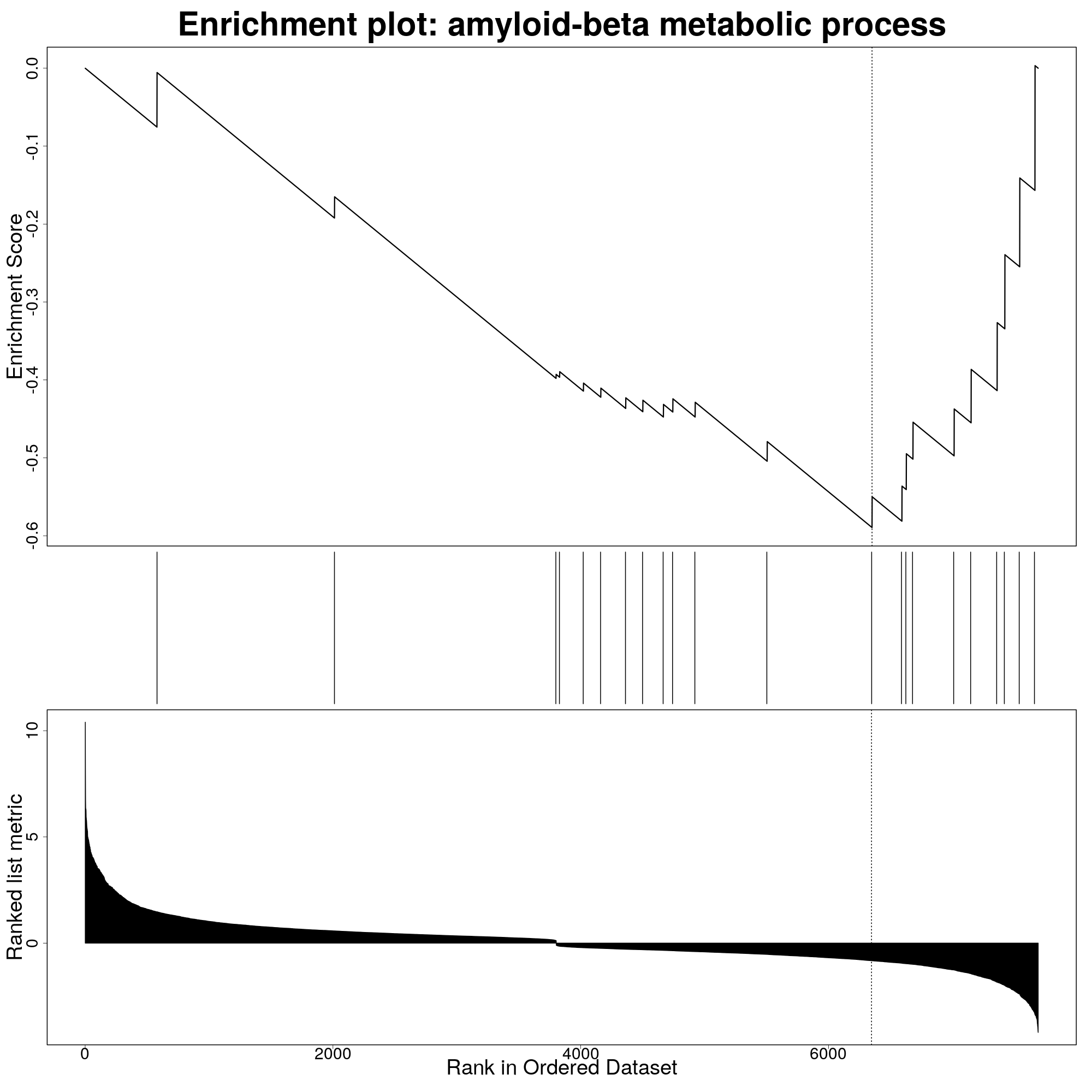

Supplement: Supplementary file 15 [file DataSheet_7.zip › Supplementary data 7 GSEA CCR2lo vs CCR2hi in CIA/Project_high_vs_low_GSEA/GO_0050435.png]

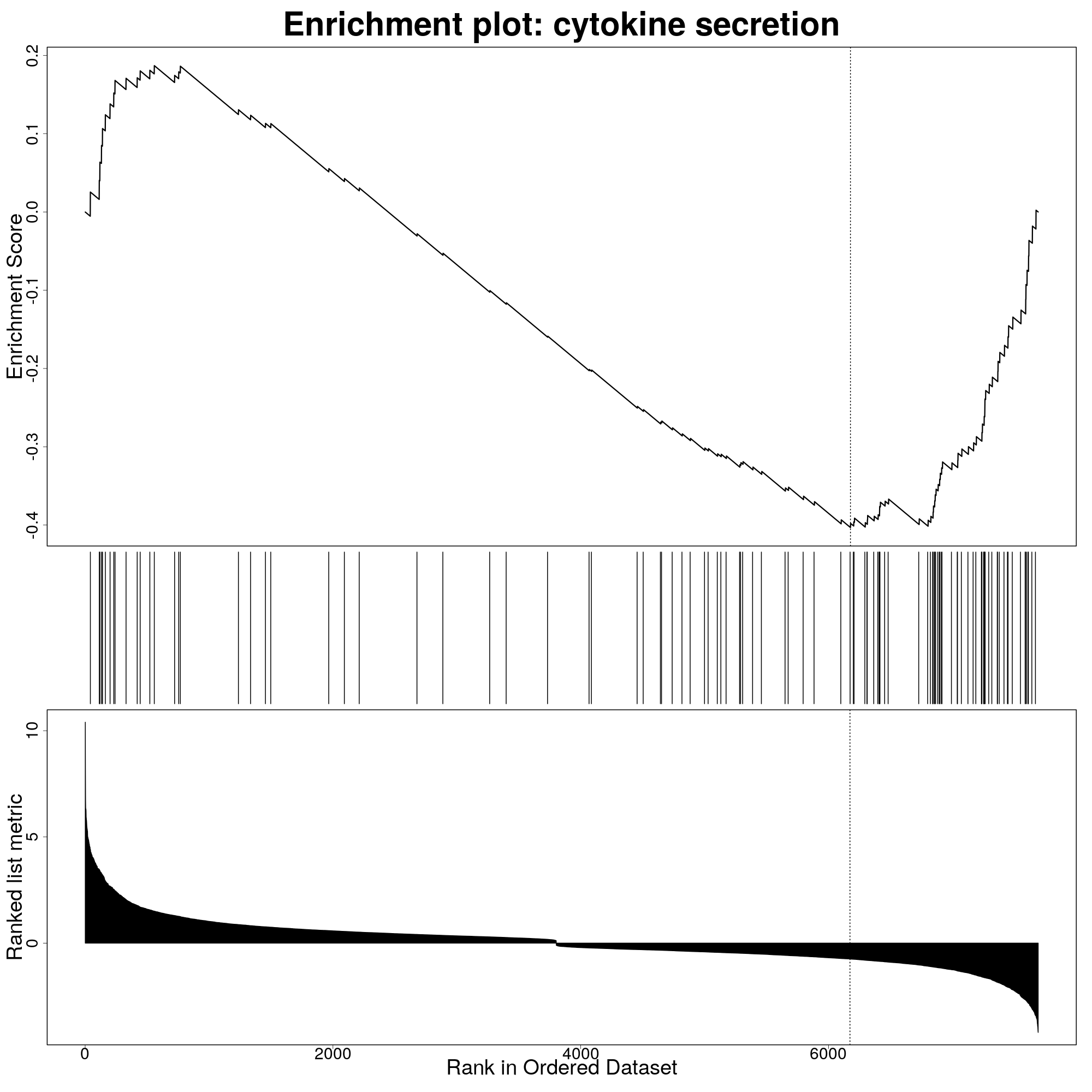

Supplement: Supplementary file 15 [file DataSheet_7.zip › Supplementary data 7 GSEA CCR2lo vs CCR2hi in CIA/Project_high_vs_low_GSEA/GO_0050663.png]

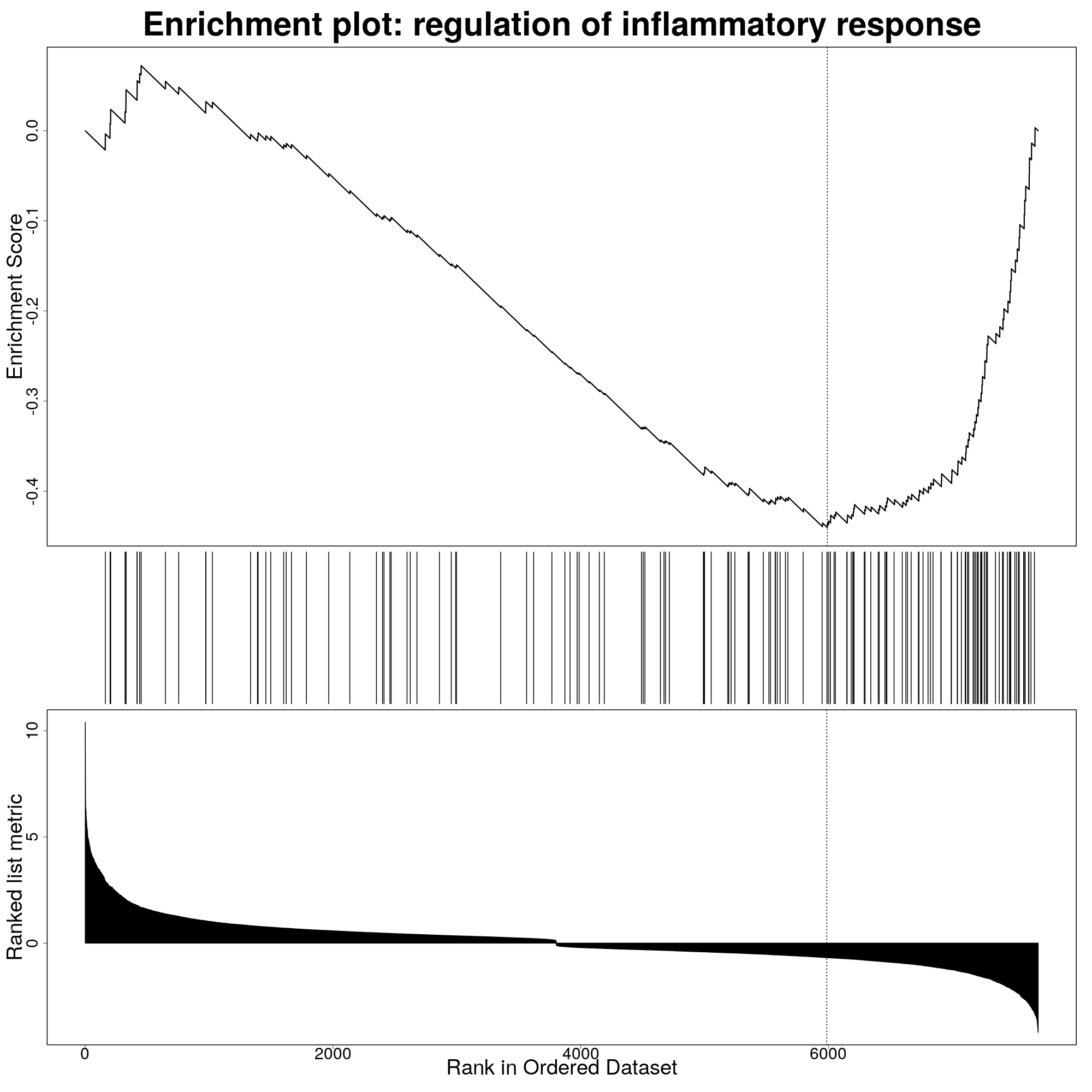

Supplement: Supplementary file 15 [file DataSheet_7.zip › Supplementary data 7 GSEA CCR2lo vs CCR2hi in CIA/Project_high_vs_low_GSEA/GO_0050727.png]

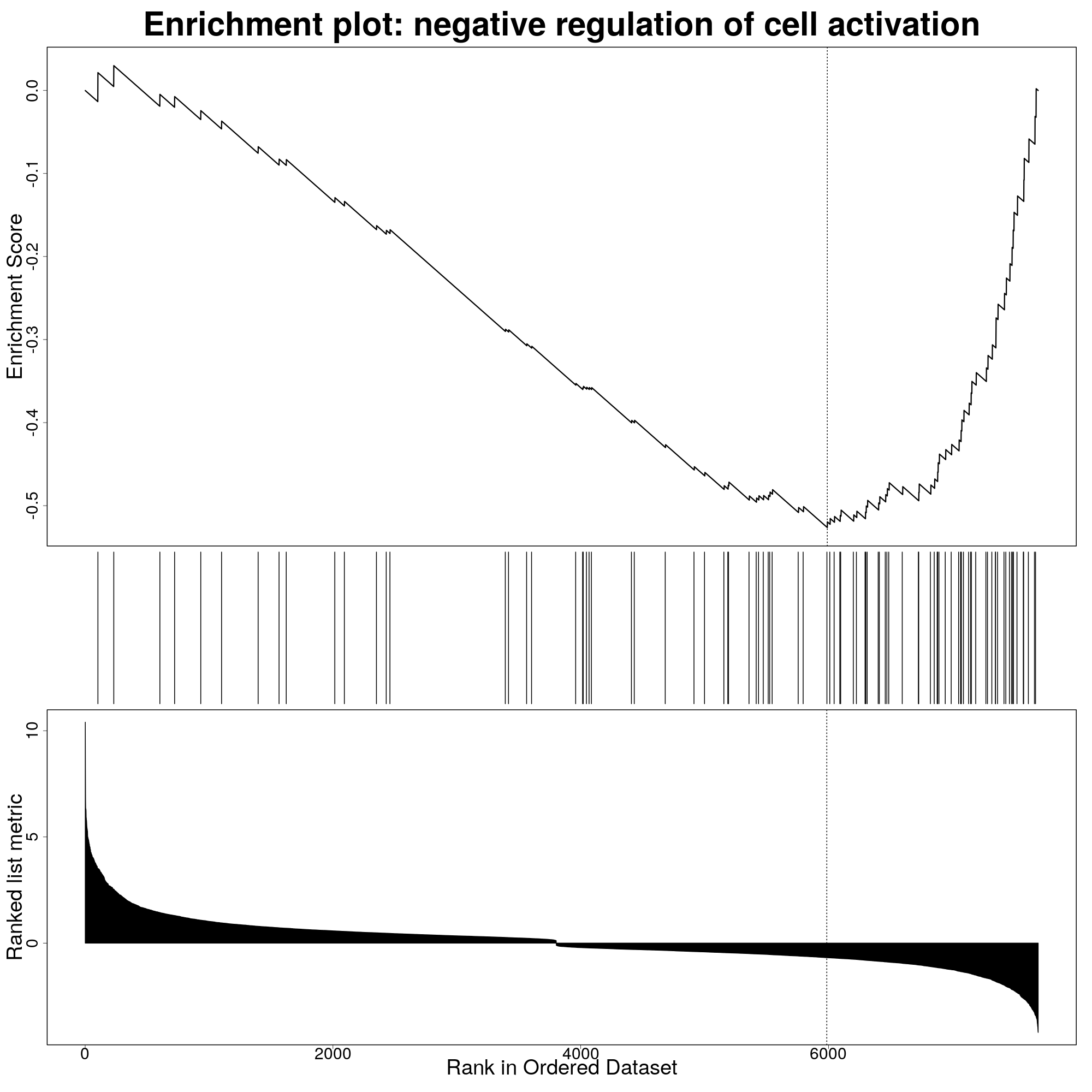

Supplement: Supplementary file 15 [file DataSheet_7.zip › Supplementary data 7 GSEA CCR2lo vs CCR2hi in CIA/Project_high_vs_low_GSEA/GO_0050866.png]

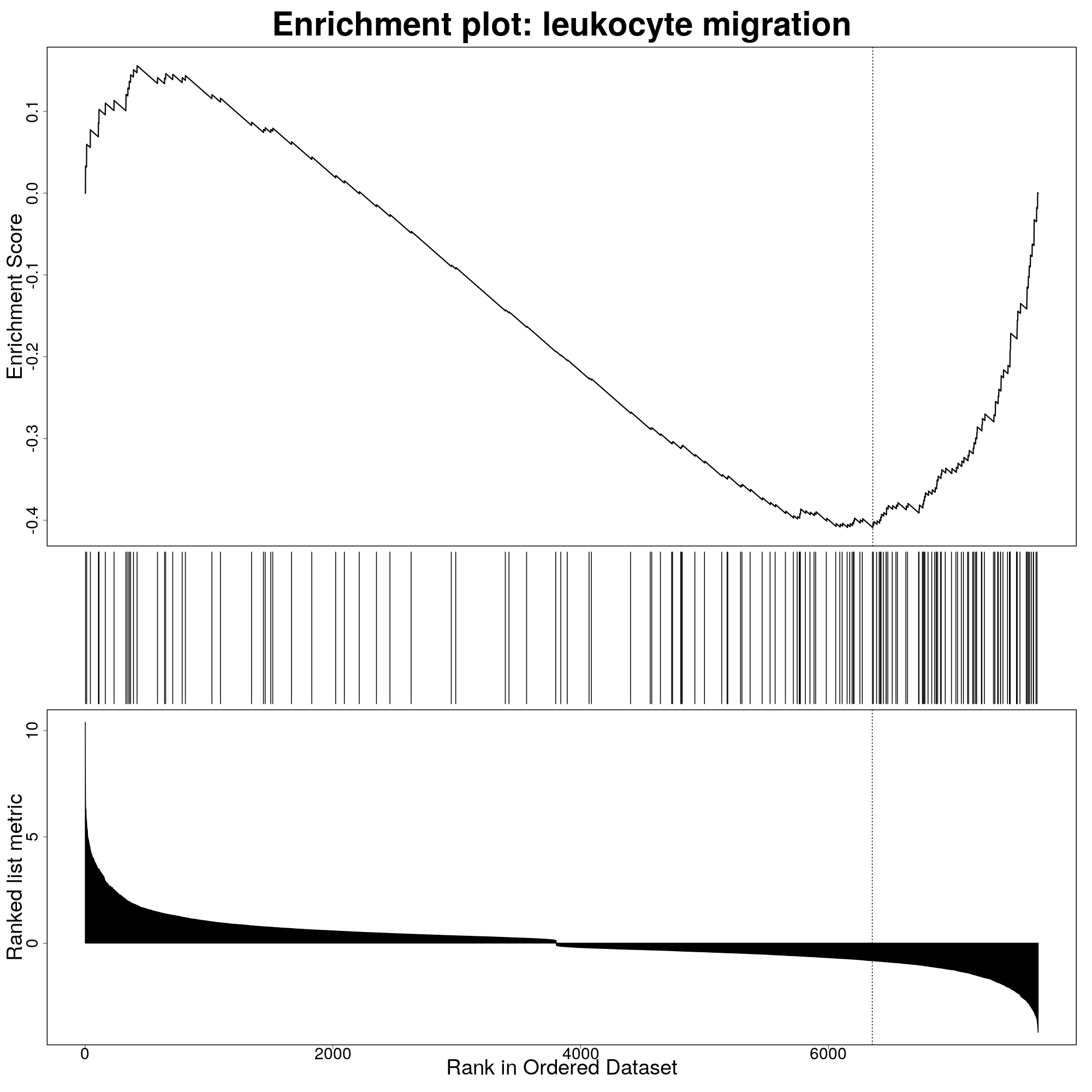

Supplement: Supplementary file 15 [file DataSheet_7.zip › Supplementary data 7 GSEA CCR2lo vs CCR2hi in CIA/Project_high_vs_low_GSEA/GO_0050900.png]

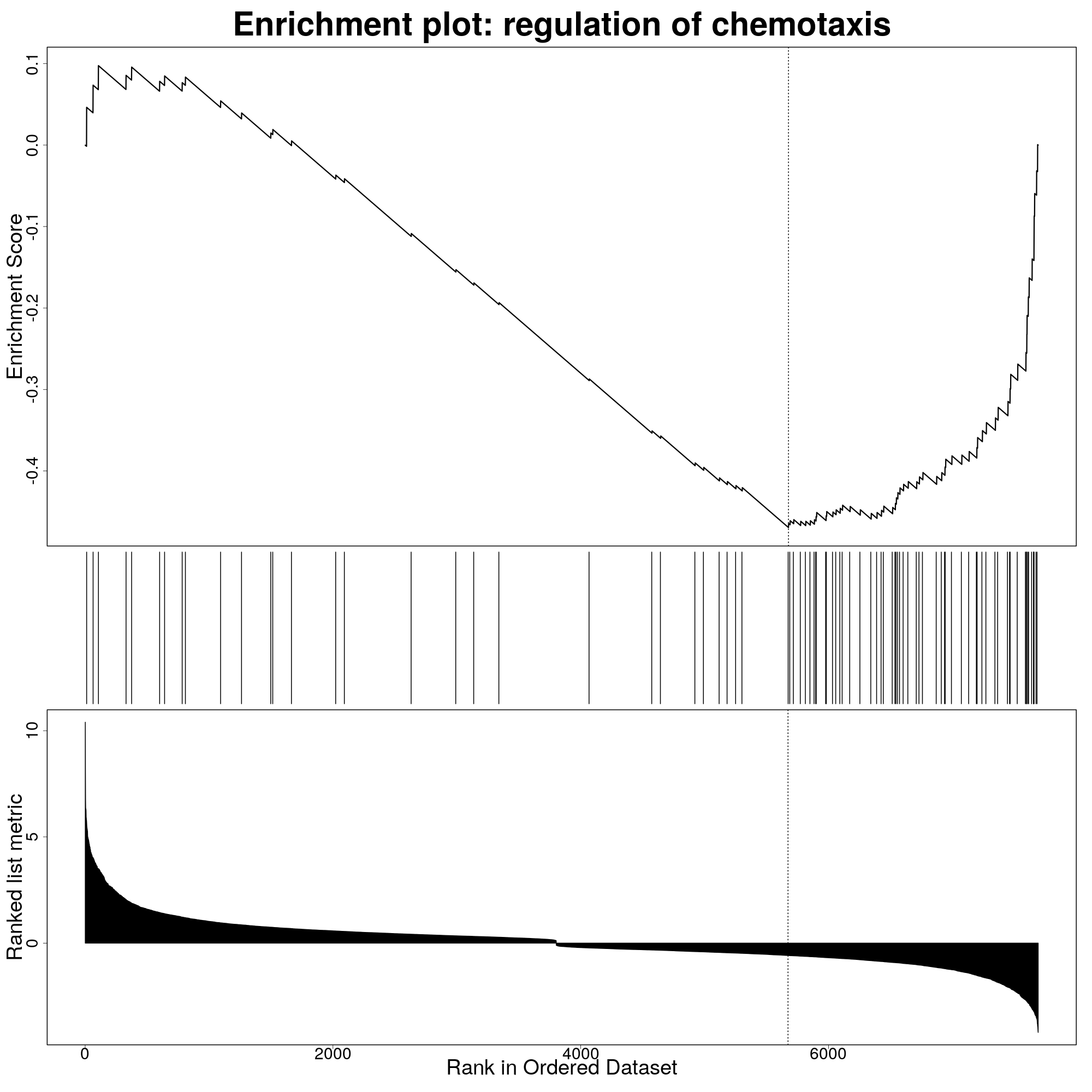

Supplement: Supplementary file 15 [file DataSheet_7.zip › Supplementary data 7 GSEA CCR2lo vs CCR2hi in CIA/Project_high_vs_low_GSEA/GO_0050920.png]

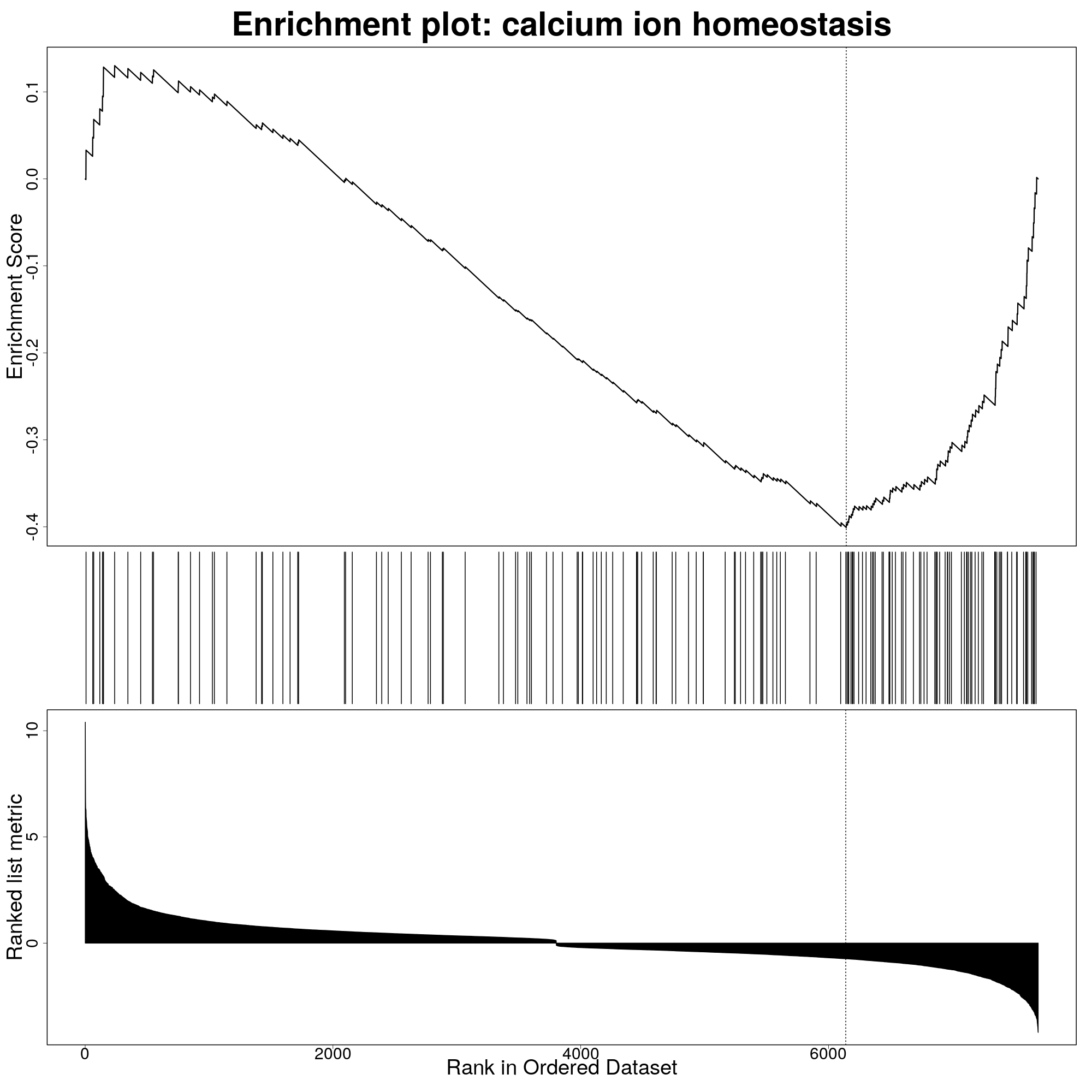

Supplement: Supplementary file 15 [file DataSheet_7.zip › Supplementary data 7 GSEA CCR2lo vs CCR2hi in CIA/Project_high_vs_low_GSEA/GO_0055074.png]

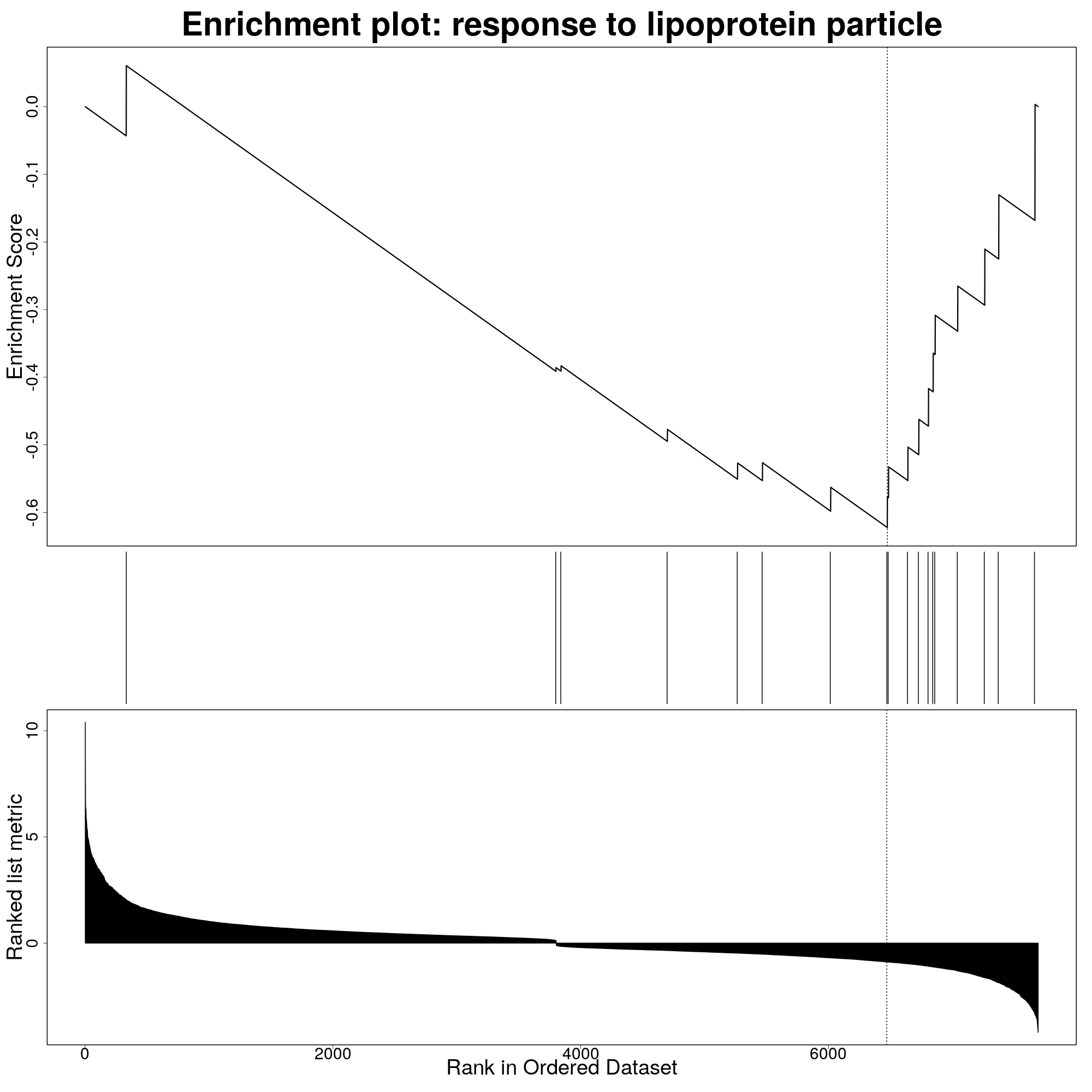

Supplement: Supplementary file 15 [file DataSheet_7.zip › Supplementary data 7 GSEA CCR2lo vs CCR2hi in CIA/Project_high_vs_low_GSEA/GO_0055094.png]

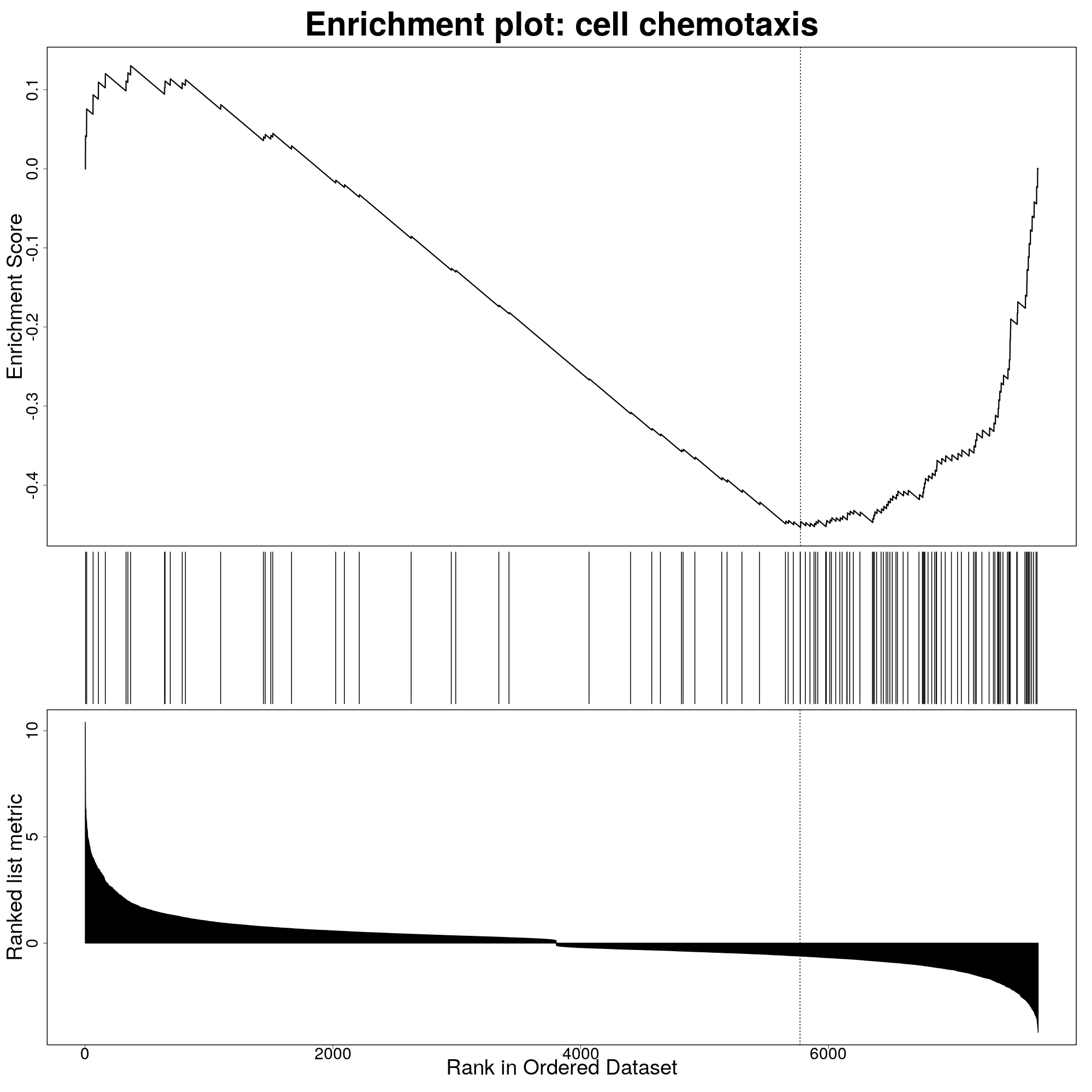

Supplement: Supplementary file 15 [file DataSheet_7.zip › Supplementary data 7 GSEA CCR2lo vs CCR2hi in CIA/Project_high_vs_low_GSEA/GO_0060326.png]

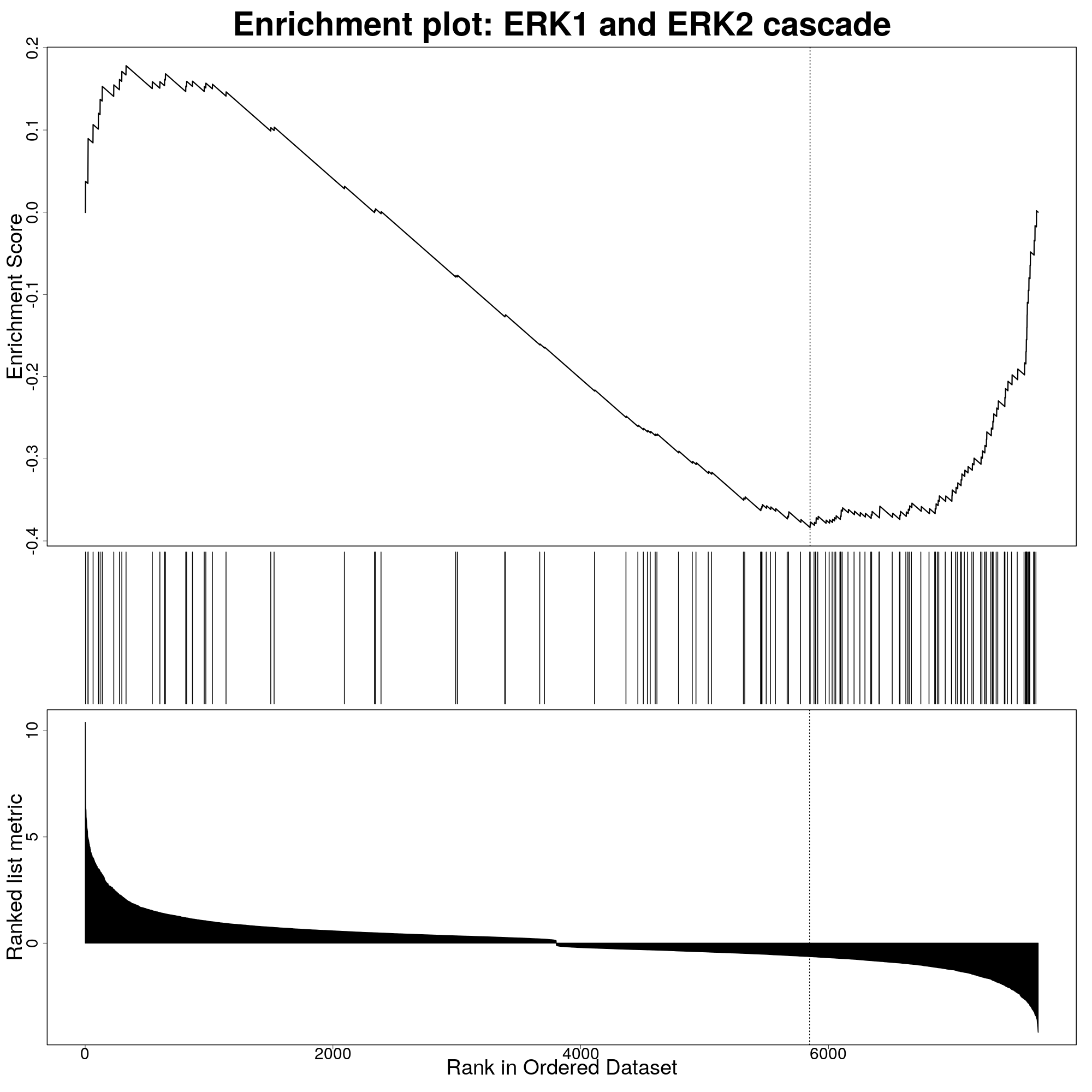

Supplement: Supplementary file 15 [file DataSheet_7.zip › Supplementary data 7 GSEA CCR2lo vs CCR2hi in CIA/Project_high_vs_low_GSEA/GO_0070371.png]

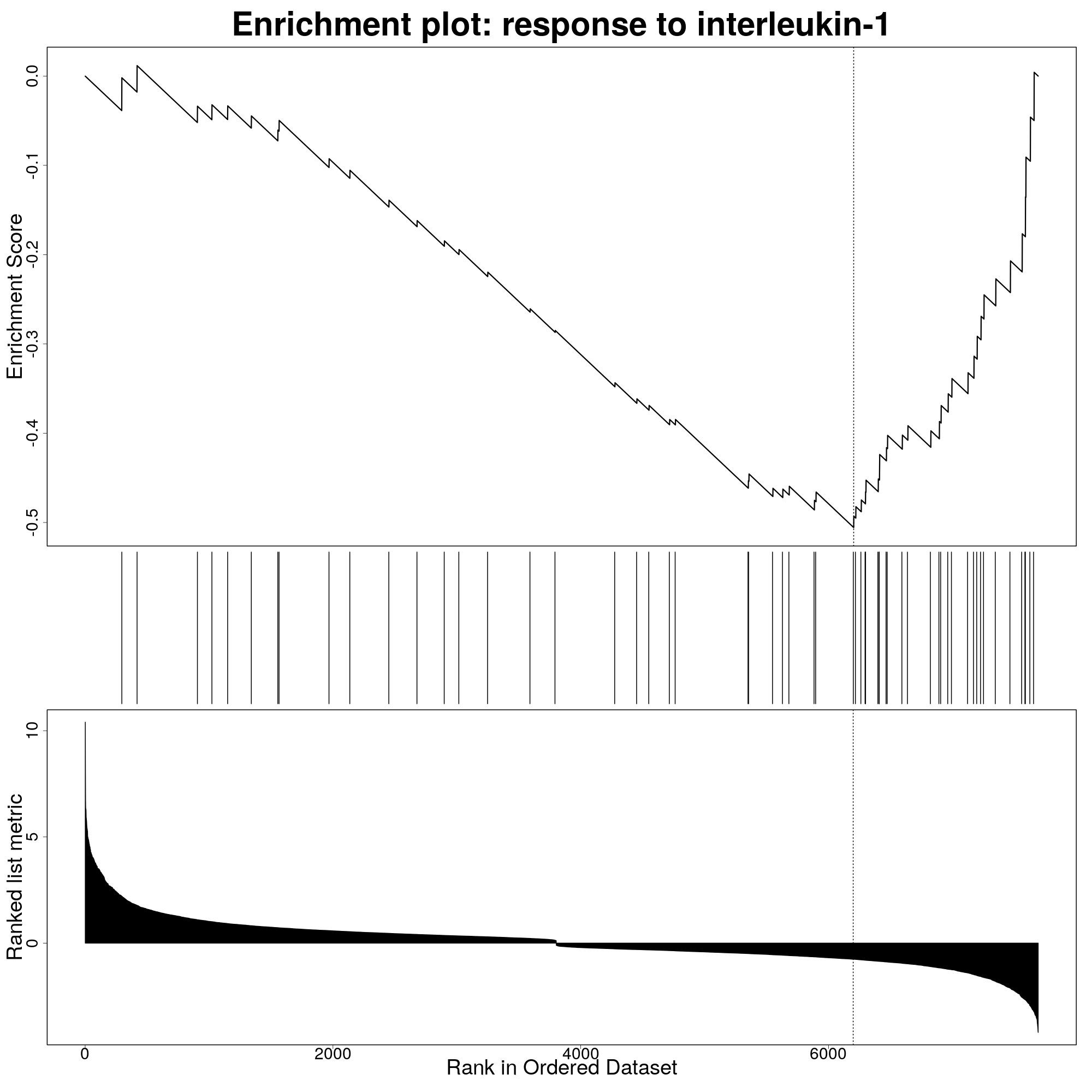

Supplement: Supplementary file 15 [file DataSheet_7.zip › Supplementary data 7 GSEA CCR2lo vs CCR2hi in CIA/Project_high_vs_low_GSEA/GO_0070555.png]

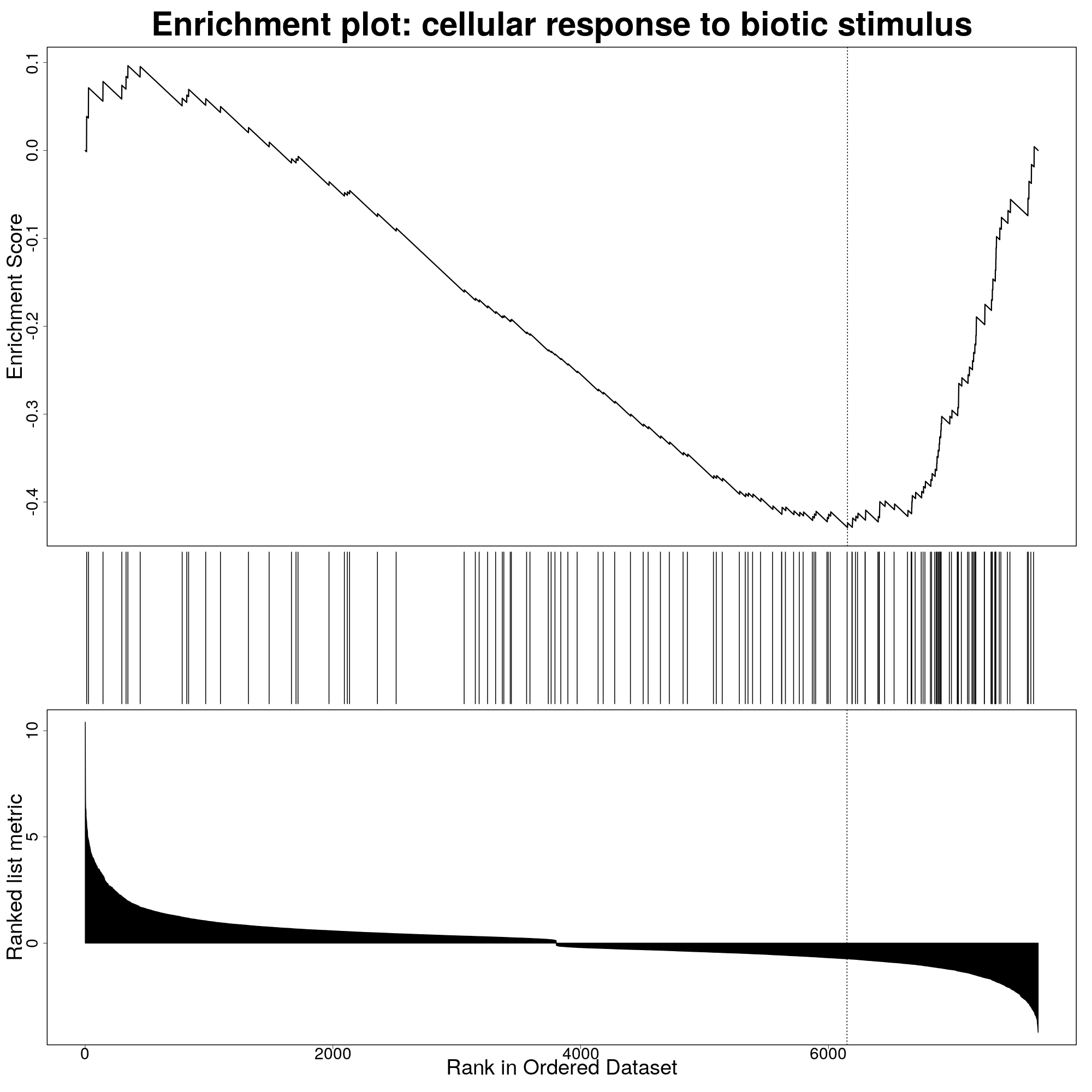

Supplement: Supplementary file 15 [file DataSheet_7.zip › Supplementary data 7 GSEA CCR2lo vs CCR2hi in CIA/Project_high_vs_low_GSEA/GO_0071216.png]

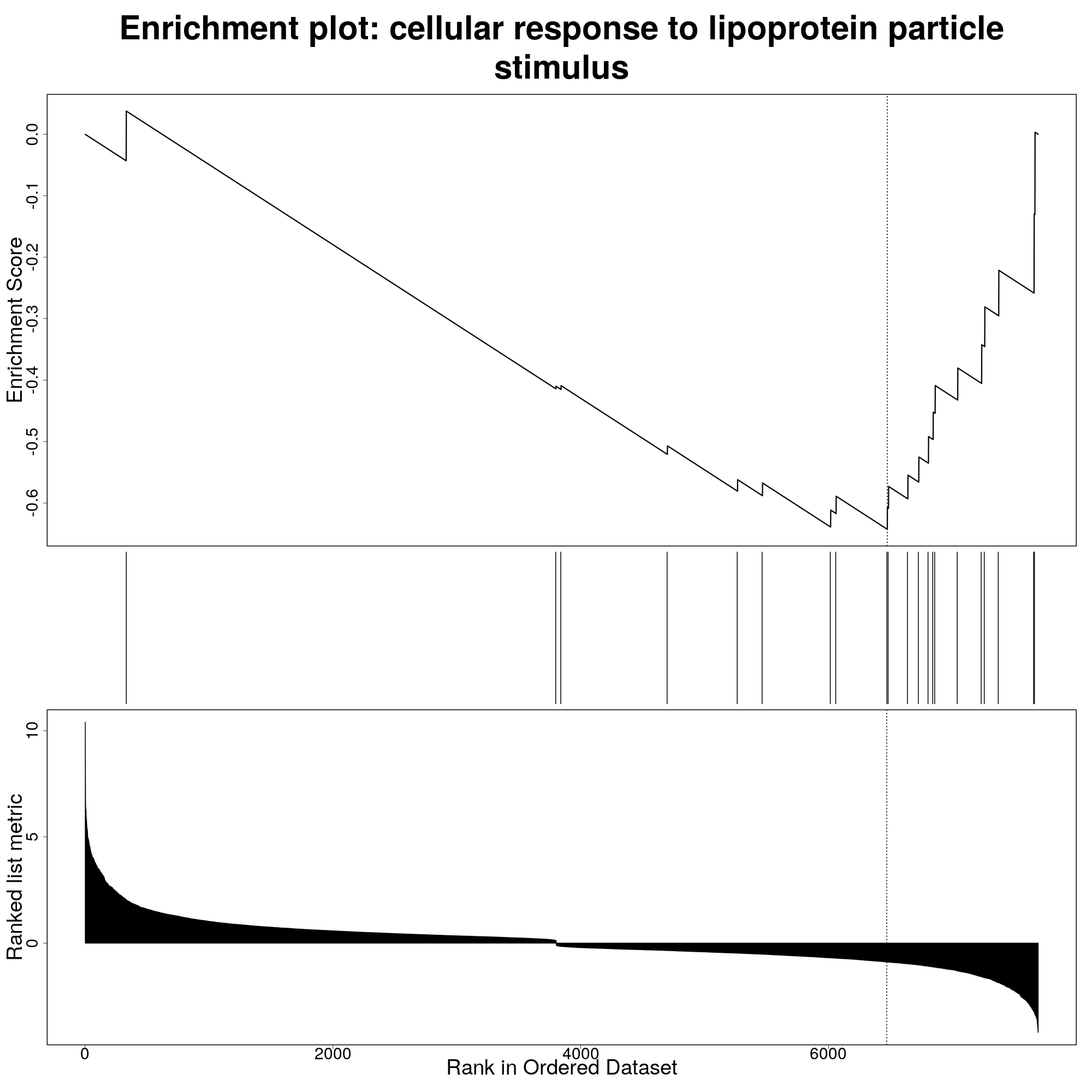

Supplement: Supplementary file 15 [file DataSheet_7.zip › Supplementary data 7 GSEA CCR2lo vs CCR2hi in CIA/Project_high_vs_low_GSEA/GO_0071402.png]

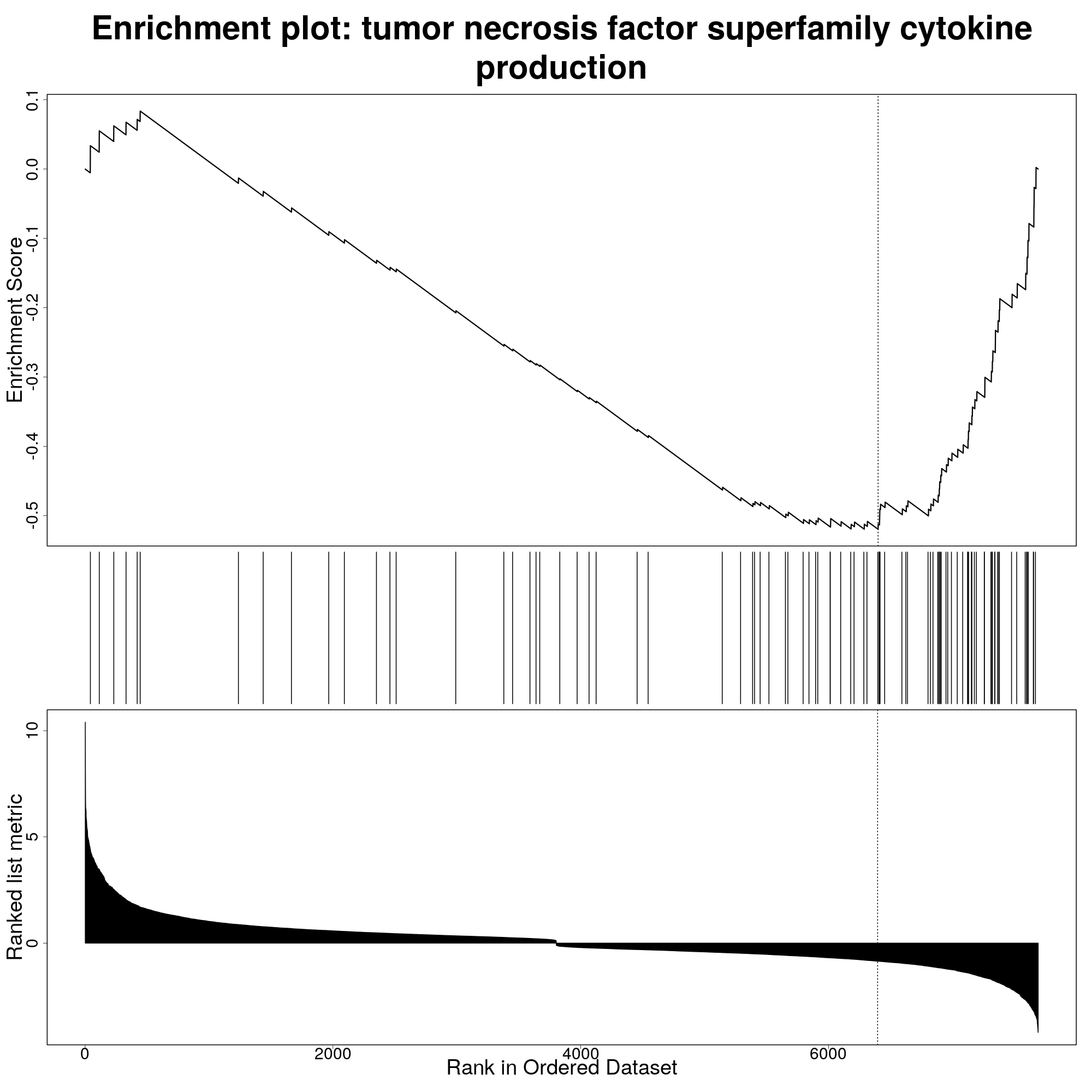

Supplement: Supplementary file 15 [file DataSheet_7.zip › Supplementary data 7 GSEA CCR2lo vs CCR2hi in CIA/Project_high_vs_low_GSEA/GO_0071706.png]

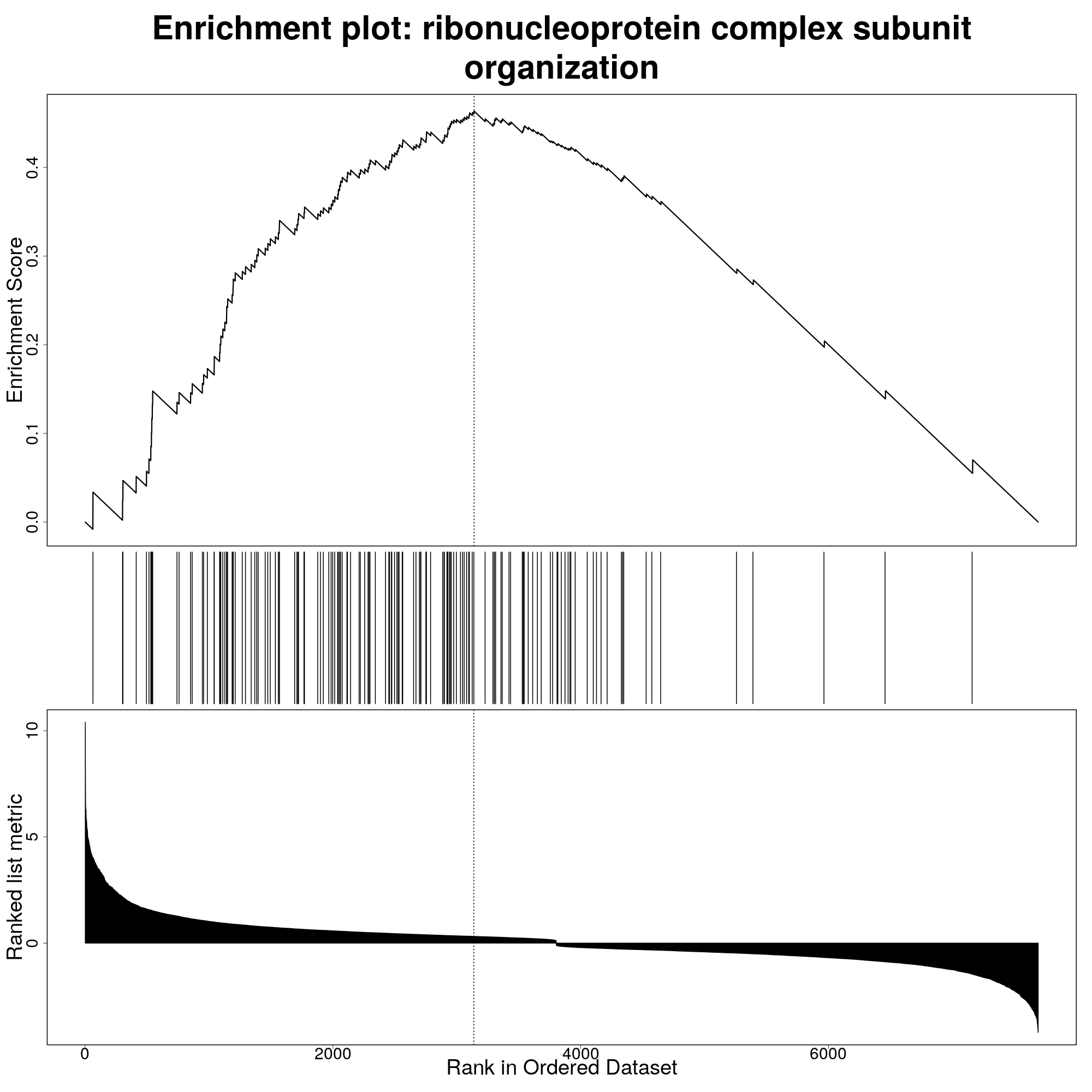

Supplement: Supplementary file 15 [file DataSheet_7.zip › Supplementary data 7 GSEA CCR2lo vs CCR2hi in CIA/Project_high_vs_low_GSEA/GO_0071826.png]

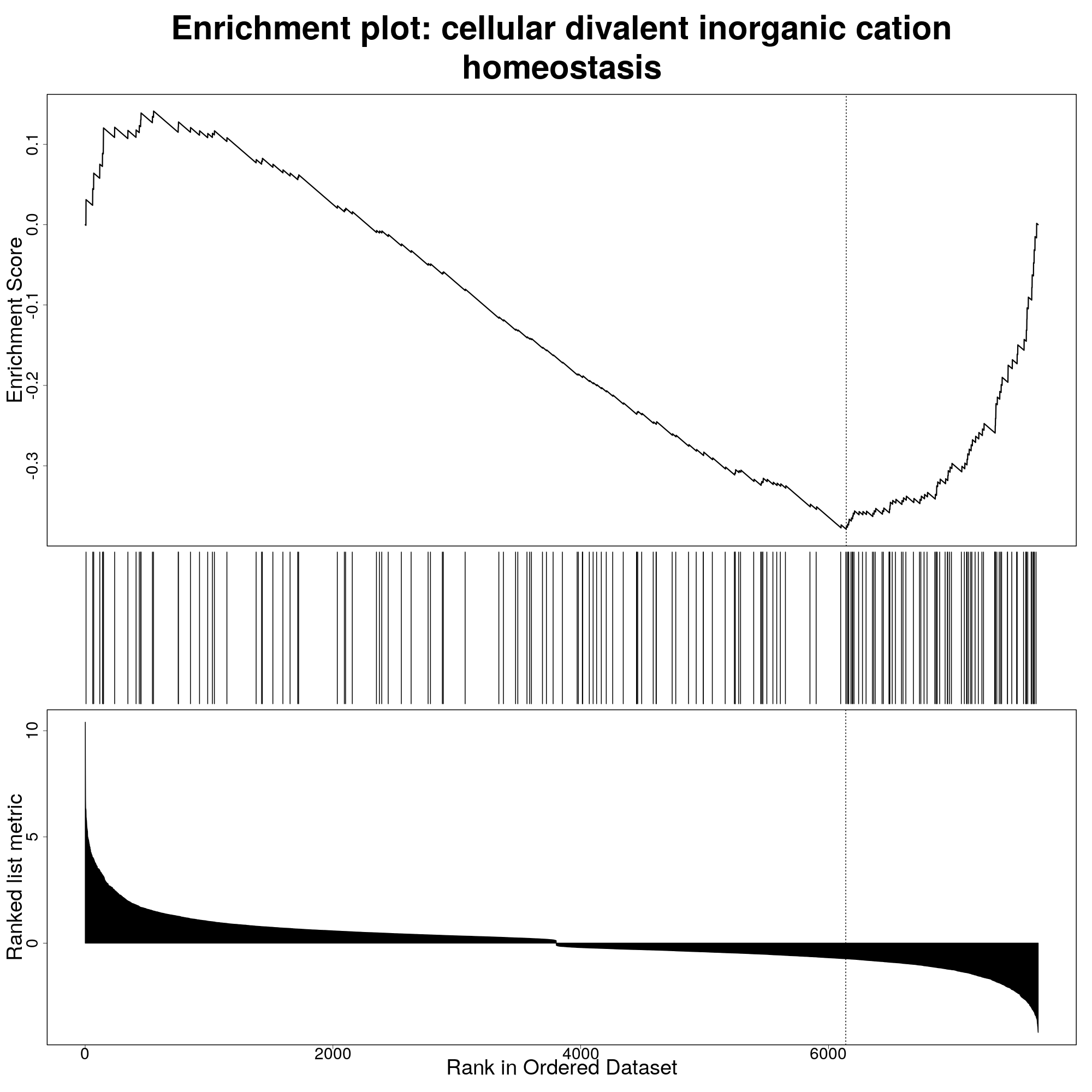

Supplement: Supplementary file 15 [file DataSheet_7.zip › Supplementary data 7 GSEA CCR2lo vs CCR2hi in CIA/Project_high_vs_low_GSEA/GO_0072503.png]

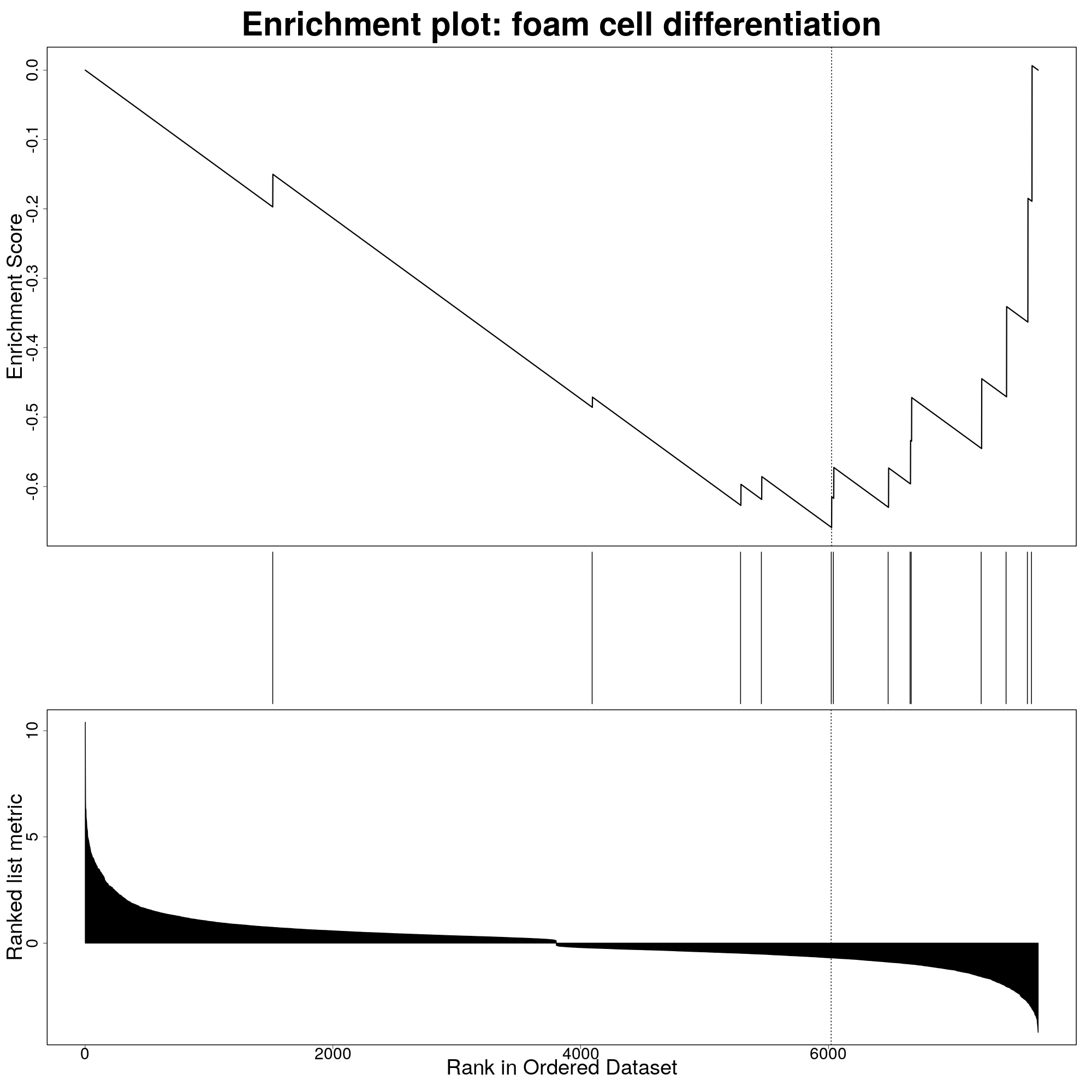

Supplement: Supplementary file 15 [file DataSheet_7.zip › Supplementary data 7 GSEA CCR2lo vs CCR2hi in CIA/Project_high_vs_low_GSEA/GO_0090077.png]

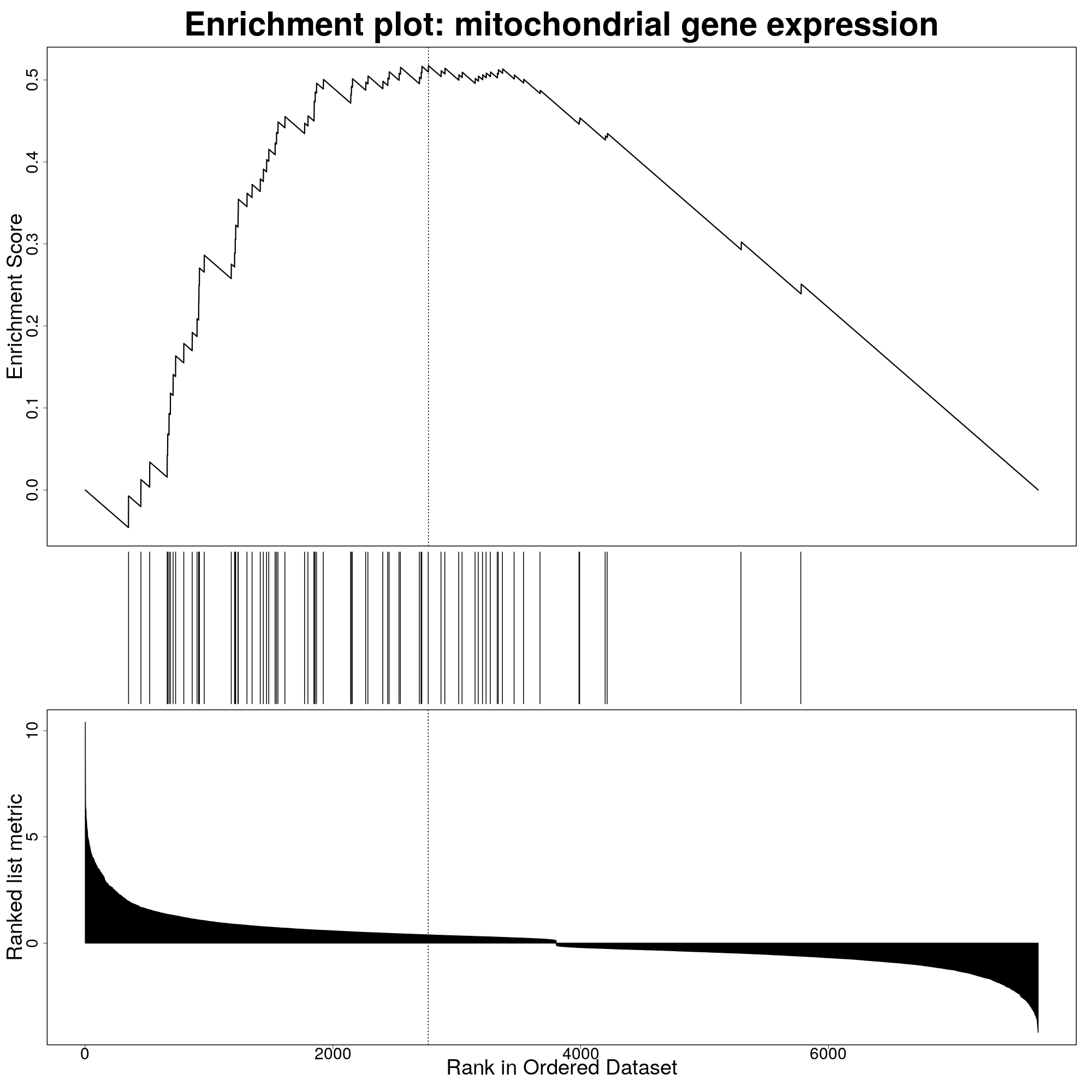

Supplement: Supplementary file 15 [file DataSheet_7.zip › Supplementary data 7 GSEA CCR2lo vs CCR2hi in CIA/Project_high_vs_low_GSEA/GO_0140053.png]

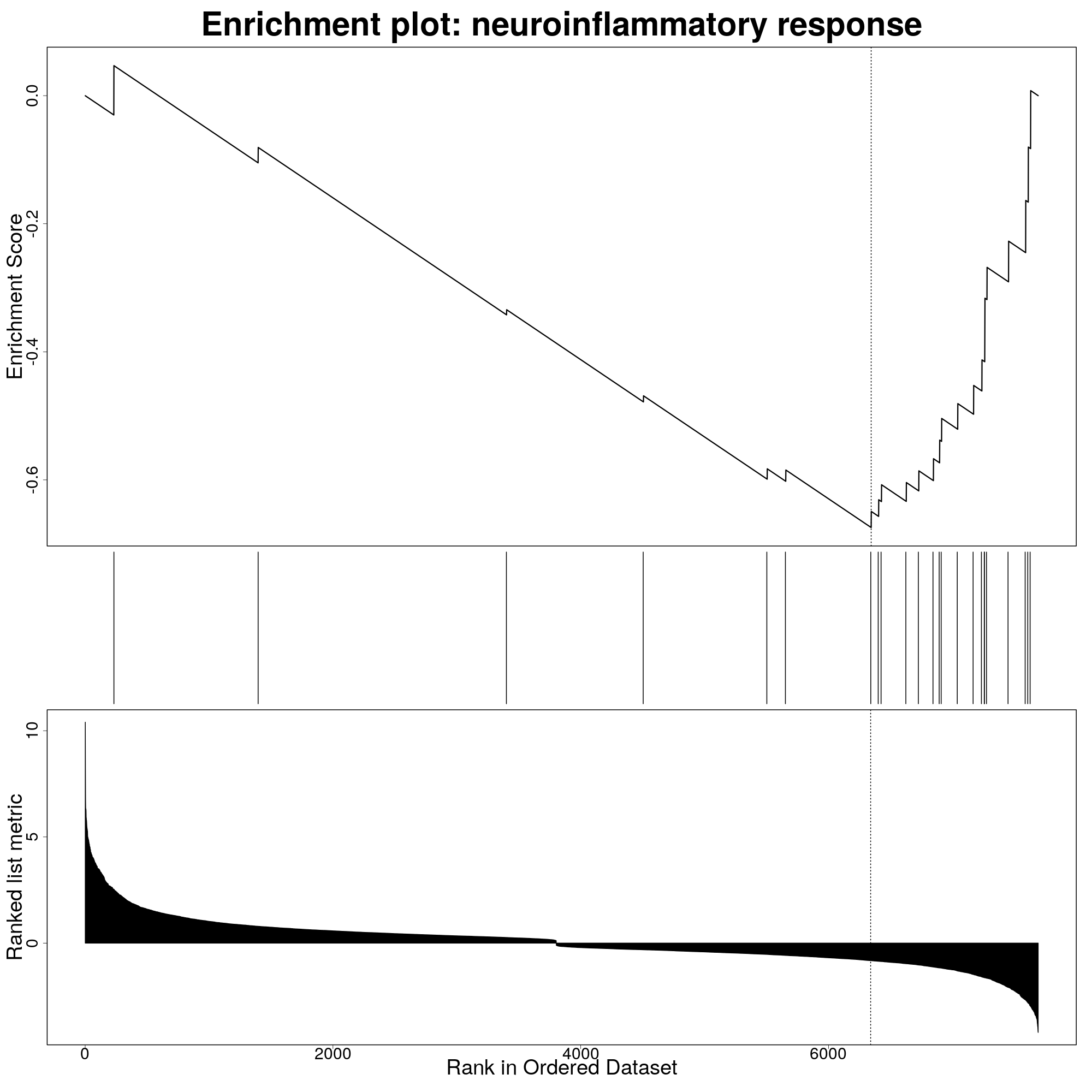

Supplement: Supplementary file 15 [file DataSheet_7.zip › Supplementary data 7 GSEA CCR2lo vs CCR2hi in CIA/Project_high_vs_low_GSEA/GO_0150076.png]

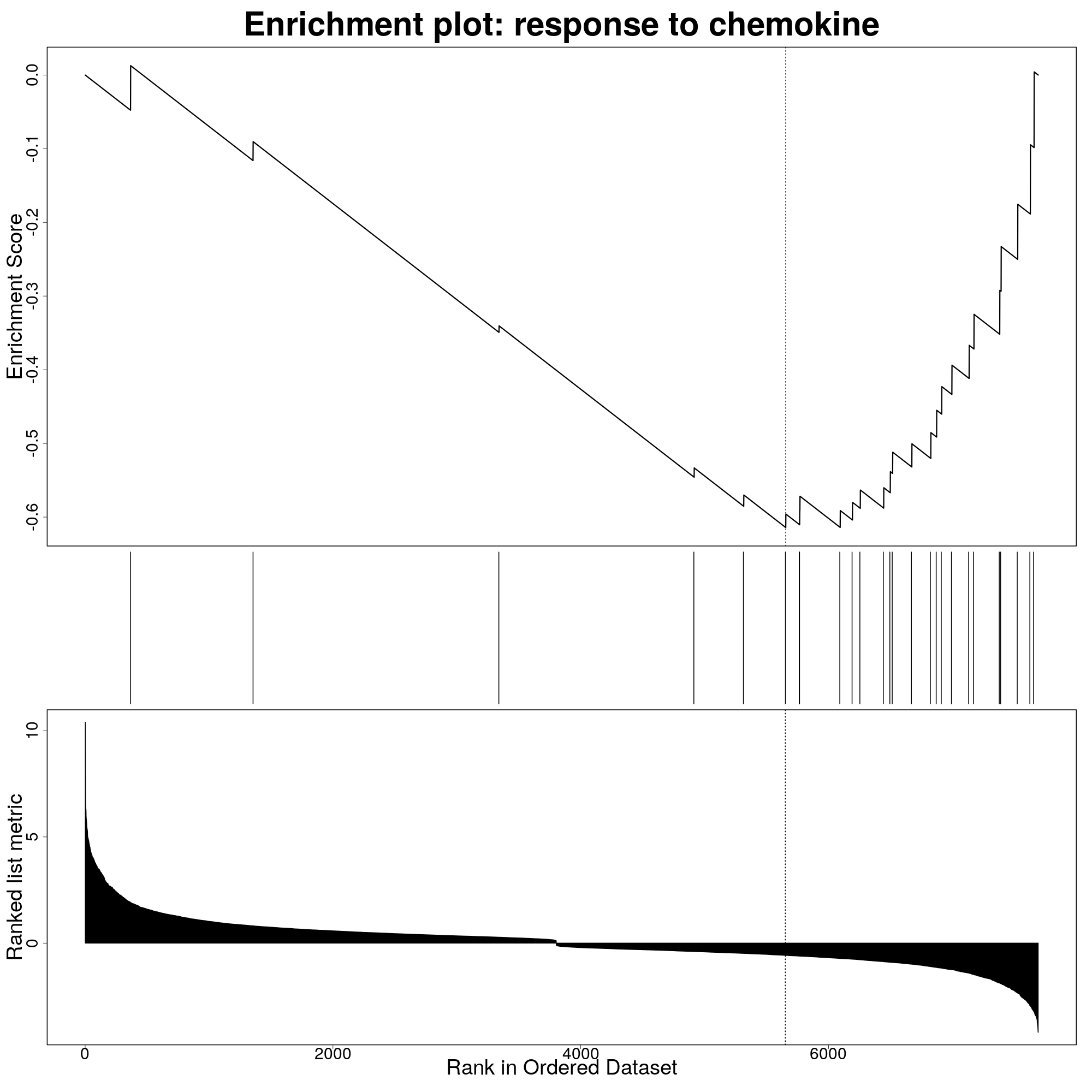

Supplement: Supplementary file 15 [file DataSheet_7.zip › Supplementary data 7 GSEA CCR2lo vs CCR2hi in CIA/Project_high_vs_low_GSEA/GO_1990868.png]

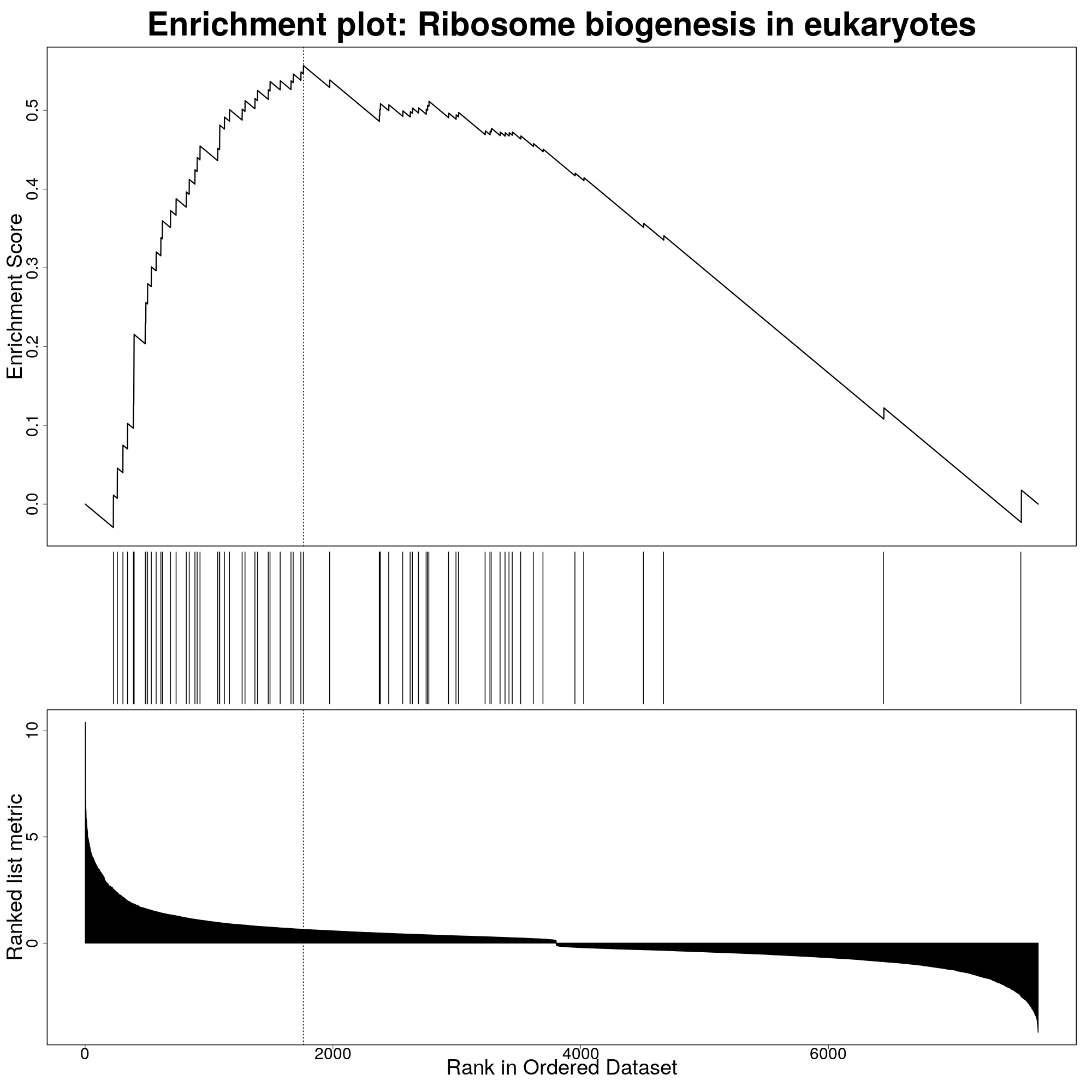

Supplement: Supplementary file 15 [file DataSheet_7.zip › Supplementary data 7 GSEA CCR2lo vs CCR2hi in CIA/Project_high_vs_low_GSEA/mmu03008.png]

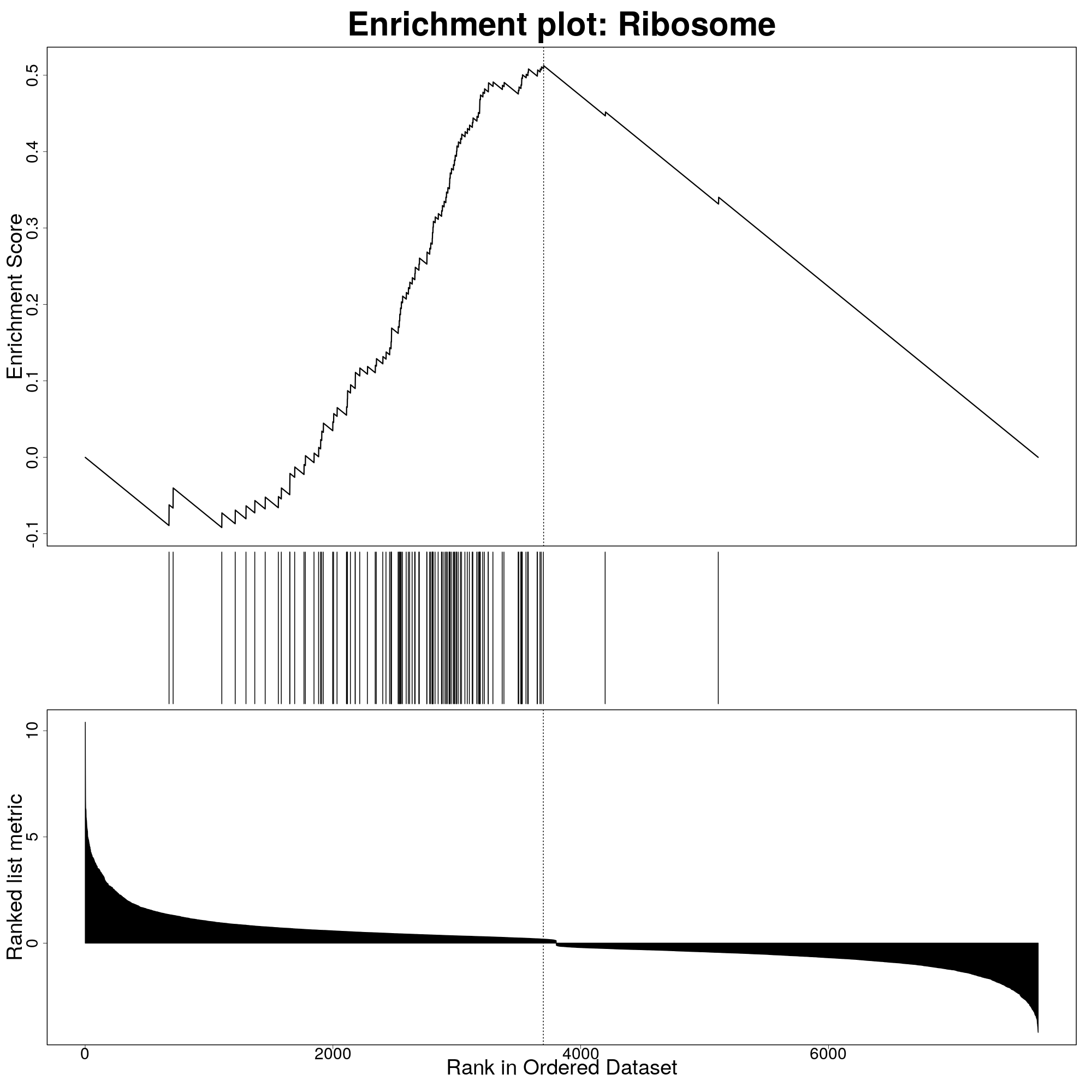

Supplement: Supplementary file 15 [file DataSheet_7.zip › Supplementary data 7 GSEA CCR2lo vs CCR2hi in CIA/Project_high_vs_low_GSEA/mmu03010.png]

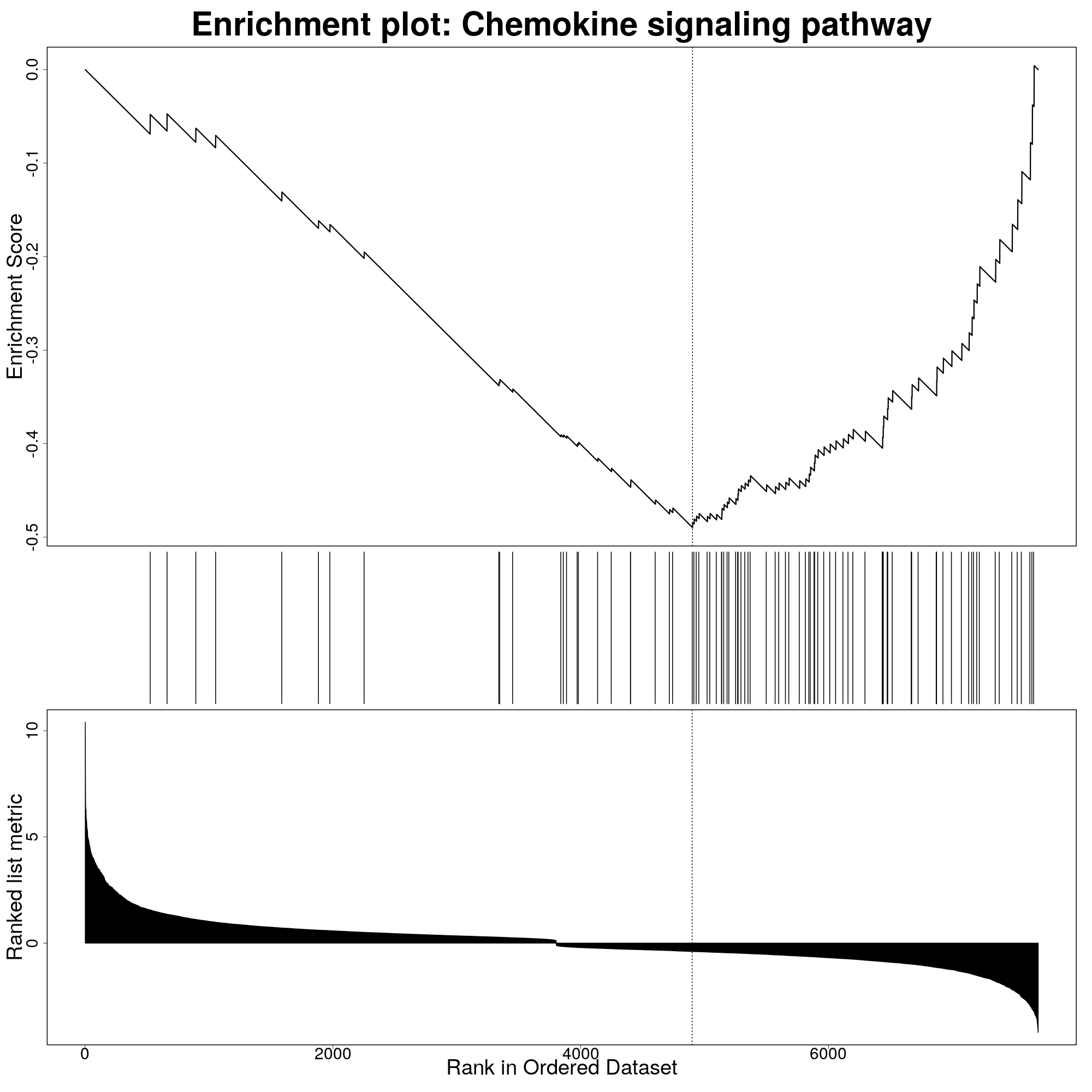

Supplement: Supplementary file 15 [file DataSheet_7.zip › Supplementary data 7 GSEA CCR2lo vs CCR2hi in CIA/Project_high_vs_low_GSEA/mmu04062.png]

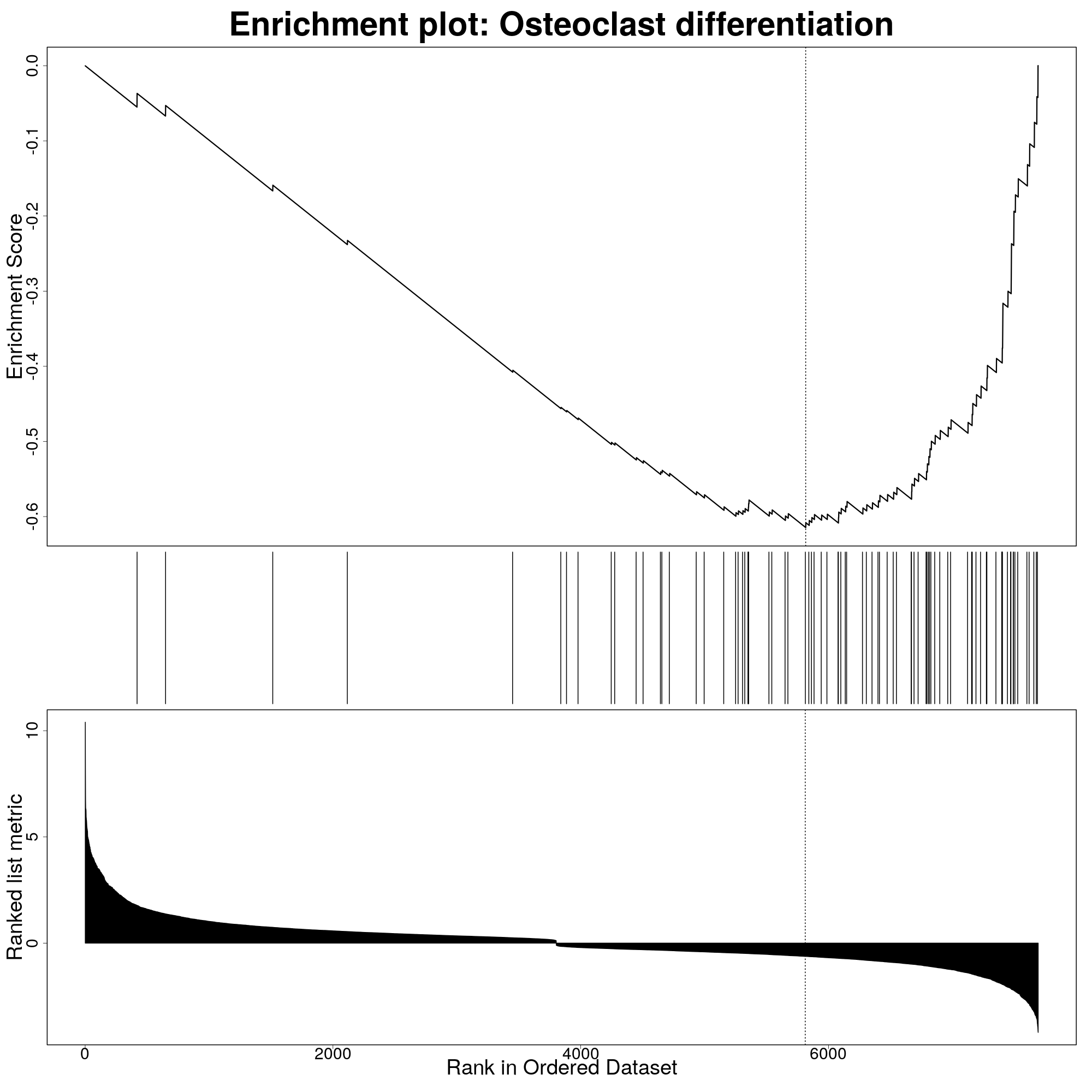

Supplement: Supplementary file 15 [file DataSheet_7.zip › Supplementary data 7 GSEA CCR2lo vs CCR2hi in CIA/Project_high_vs_low_GSEA/mmu04380.png]

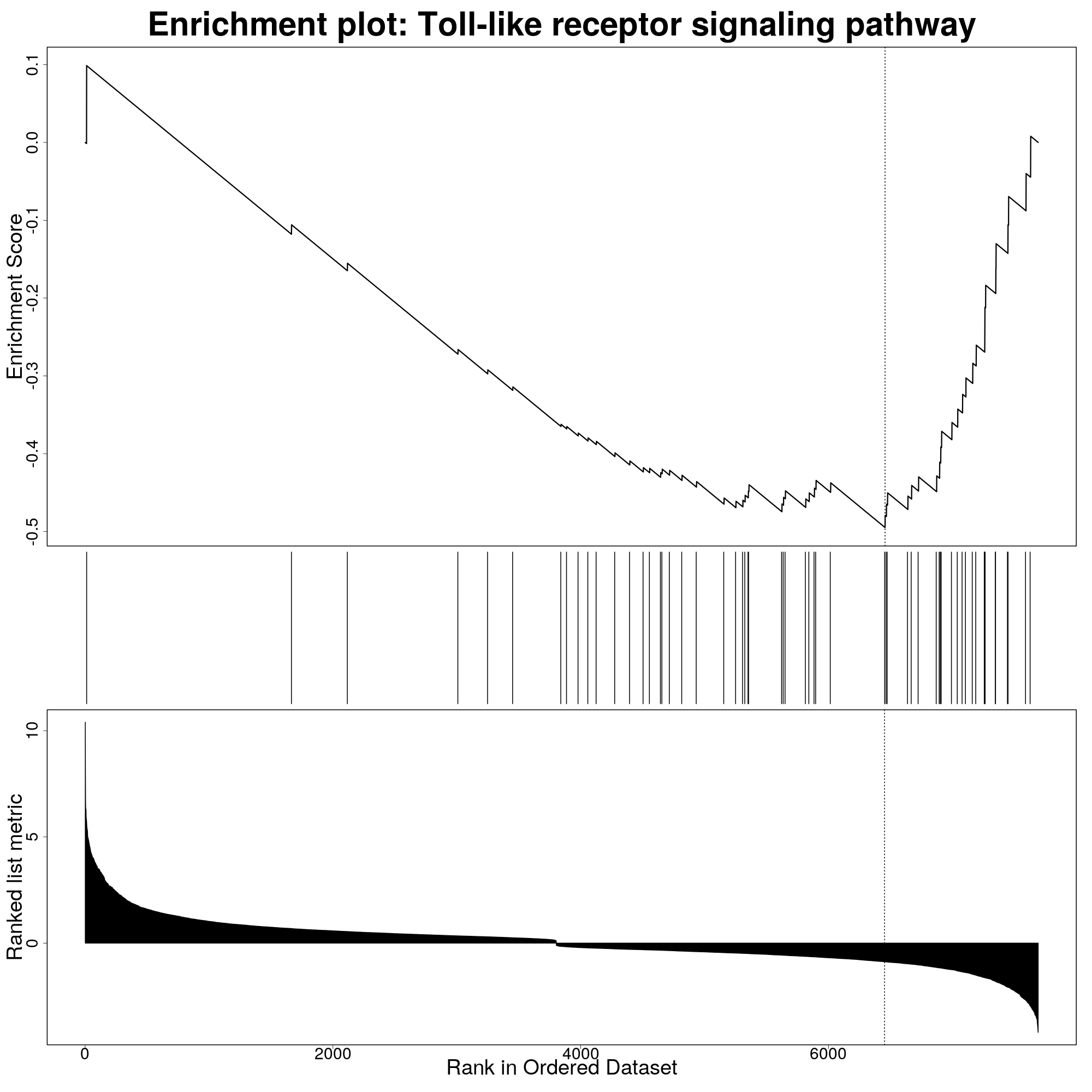

Supplement: Supplementary file 15 [file DataSheet_7.zip › Supplementary data 7 GSEA CCR2lo vs CCR2hi in CIA/Project_high_vs_low_GSEA/mmu04620.png]

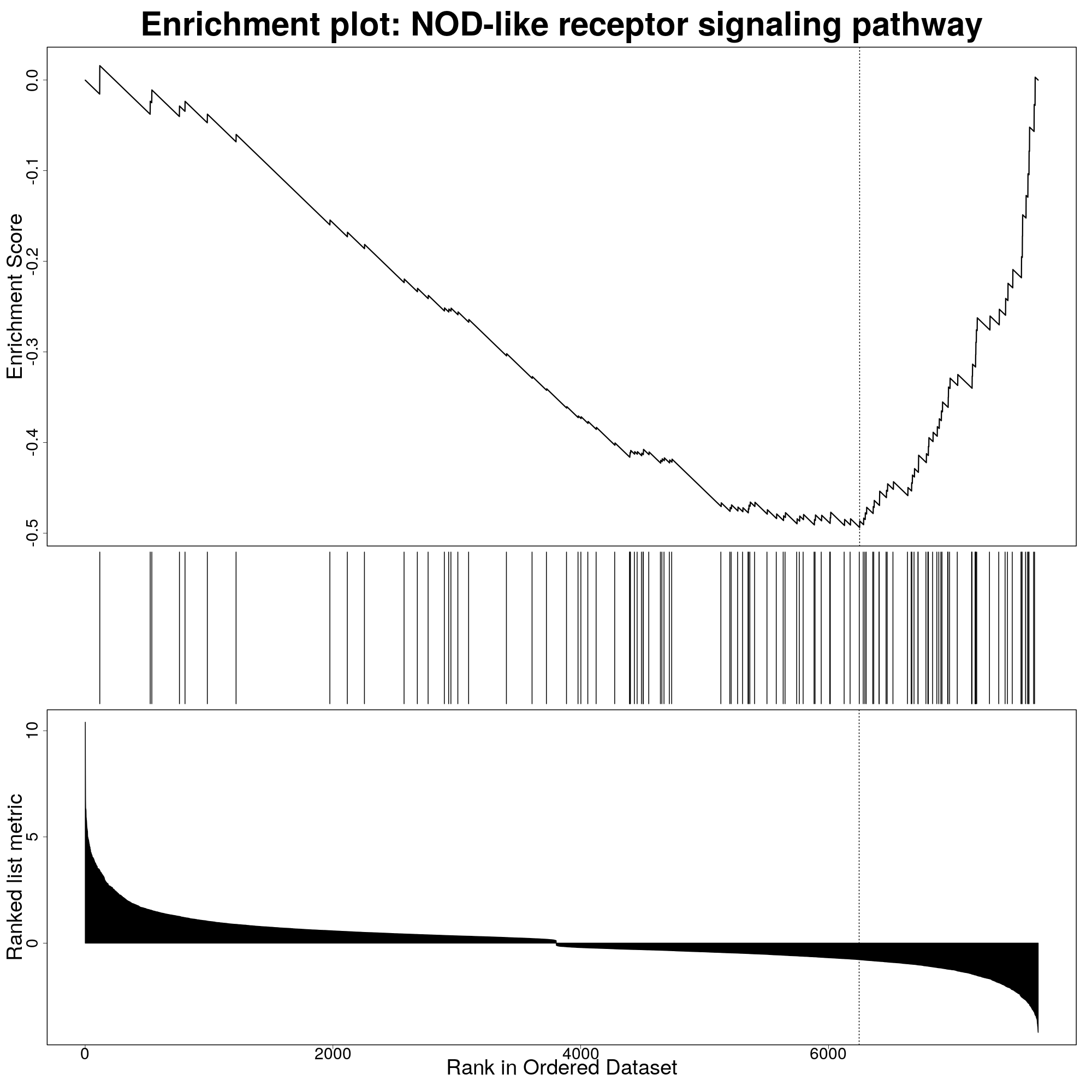

Supplement: Supplementary file 15 [file DataSheet_7.zip › Supplementary data 7 GSEA CCR2lo vs CCR2hi in CIA/Project_high_vs_low_GSEA/mmu04621.png]

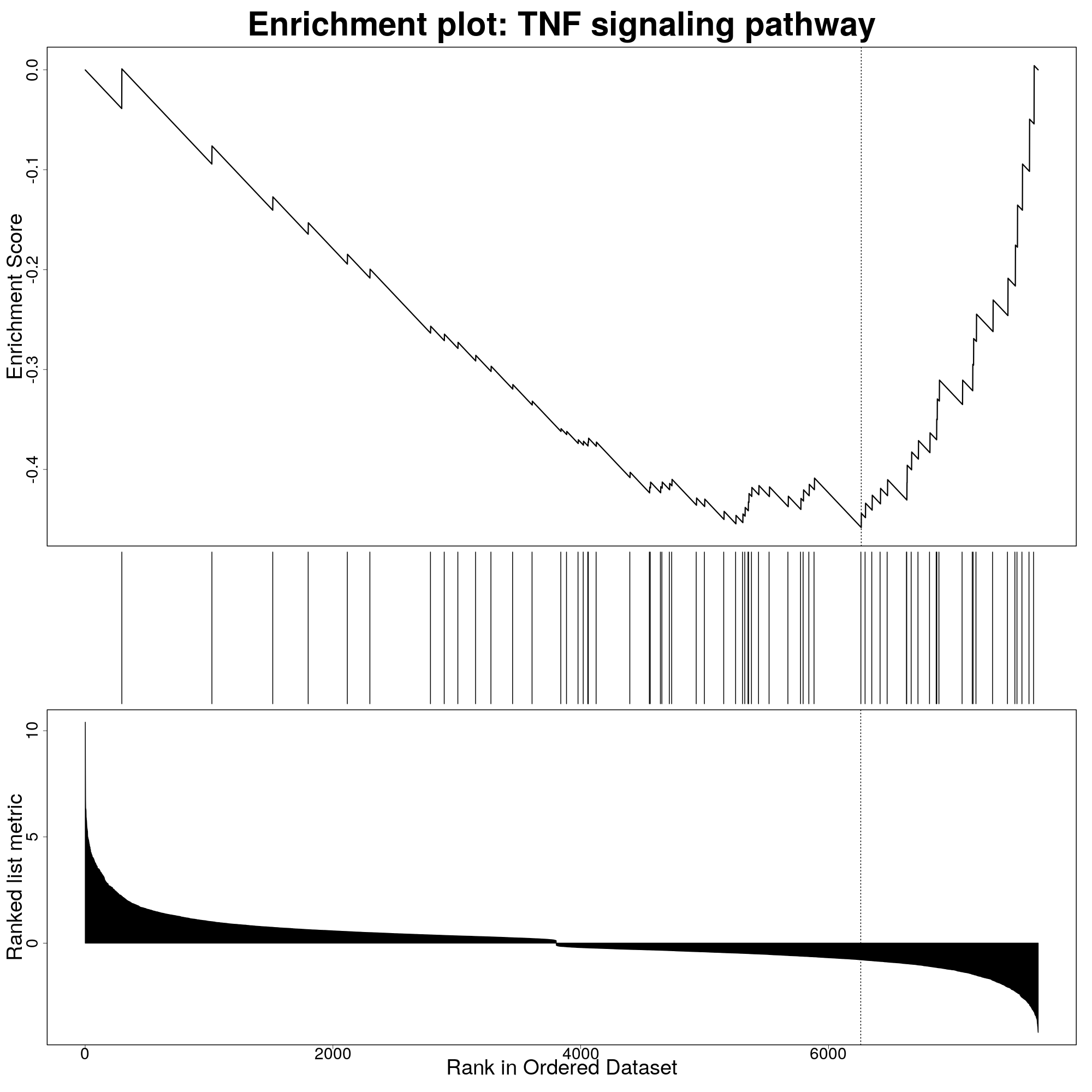

Supplement: Supplementary file 15 [file DataSheet_7.zip › Supplementary data 7 GSEA CCR2lo vs CCR2hi in CIA/Project_high_vs_low_GSEA/mmu04668.png]

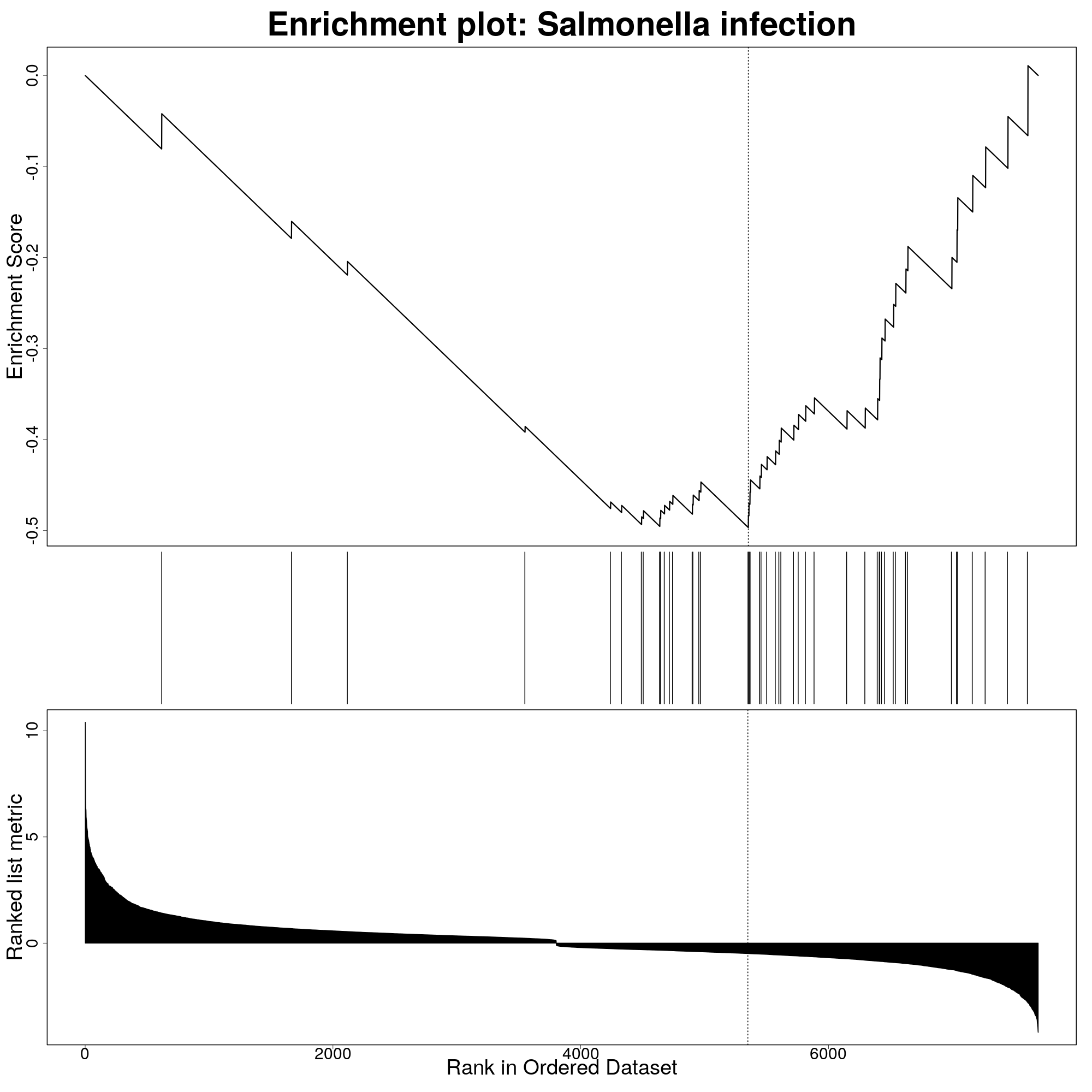

Supplement: Supplementary file 15 [file DataSheet_7.zip › Supplementary data 7 GSEA CCR2lo vs CCR2hi in CIA/Project_high_vs_low_GSEA/mmu05132.png]

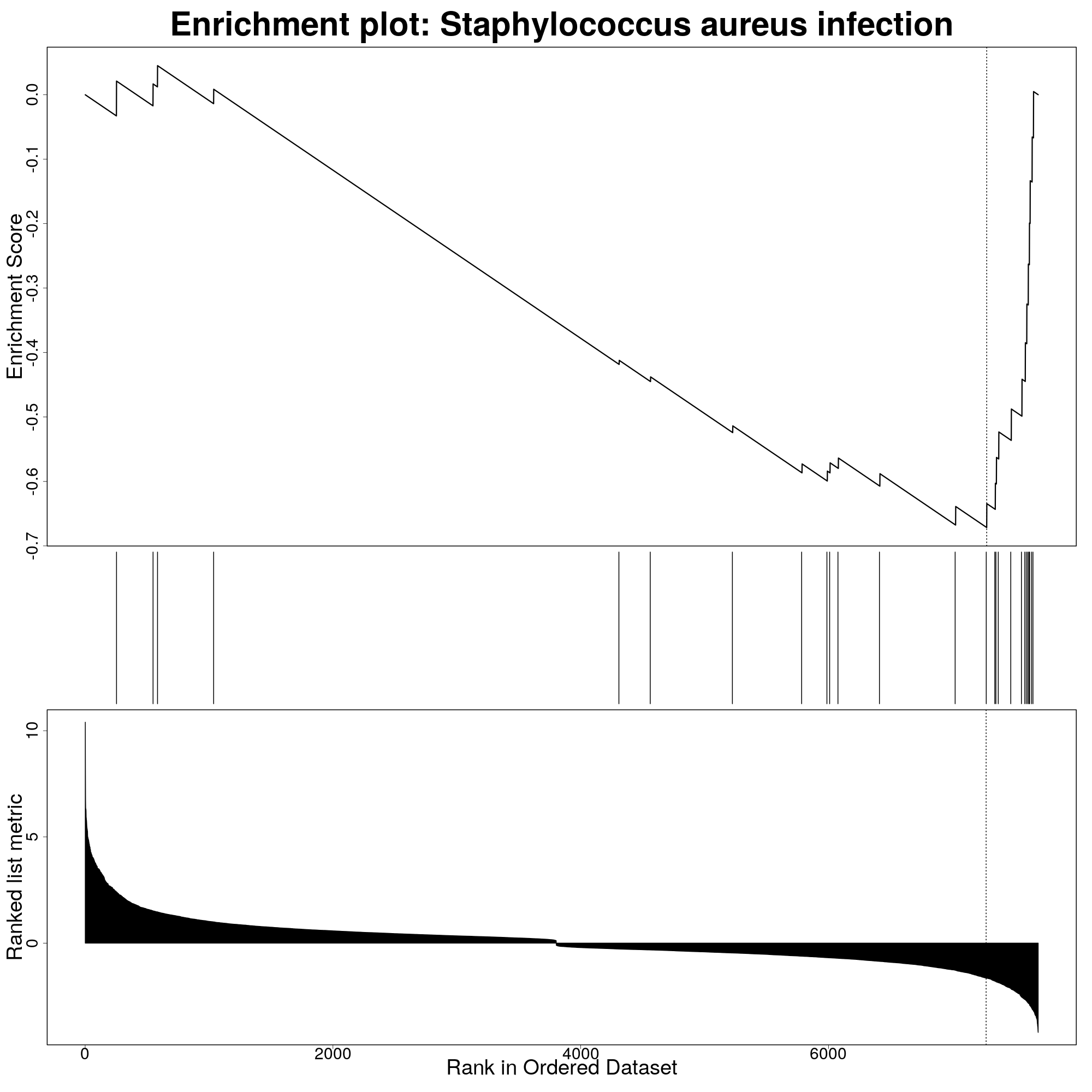

Supplement: Supplementary file 15 [file DataSheet_7.zip › Supplementary data 7 GSEA CCR2lo vs CCR2hi in CIA/Project_high_vs_low_GSEA/mmu05150.png]

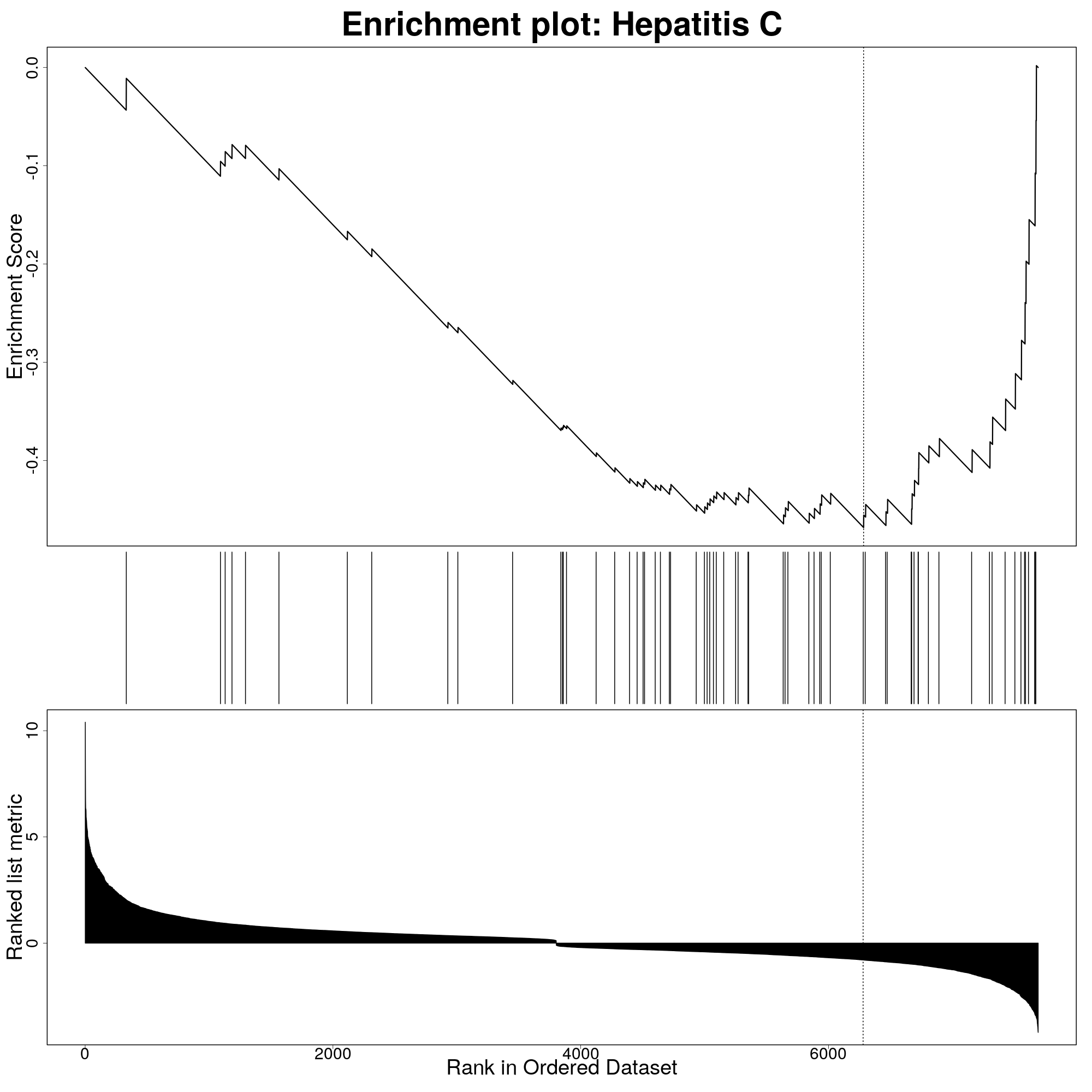

Supplement: Supplementary file 15 [file DataSheet_7.zip › Supplementary data 7 GSEA CCR2lo vs CCR2hi in CIA/Project_high_vs_low_GSEA/mmu05160.png]

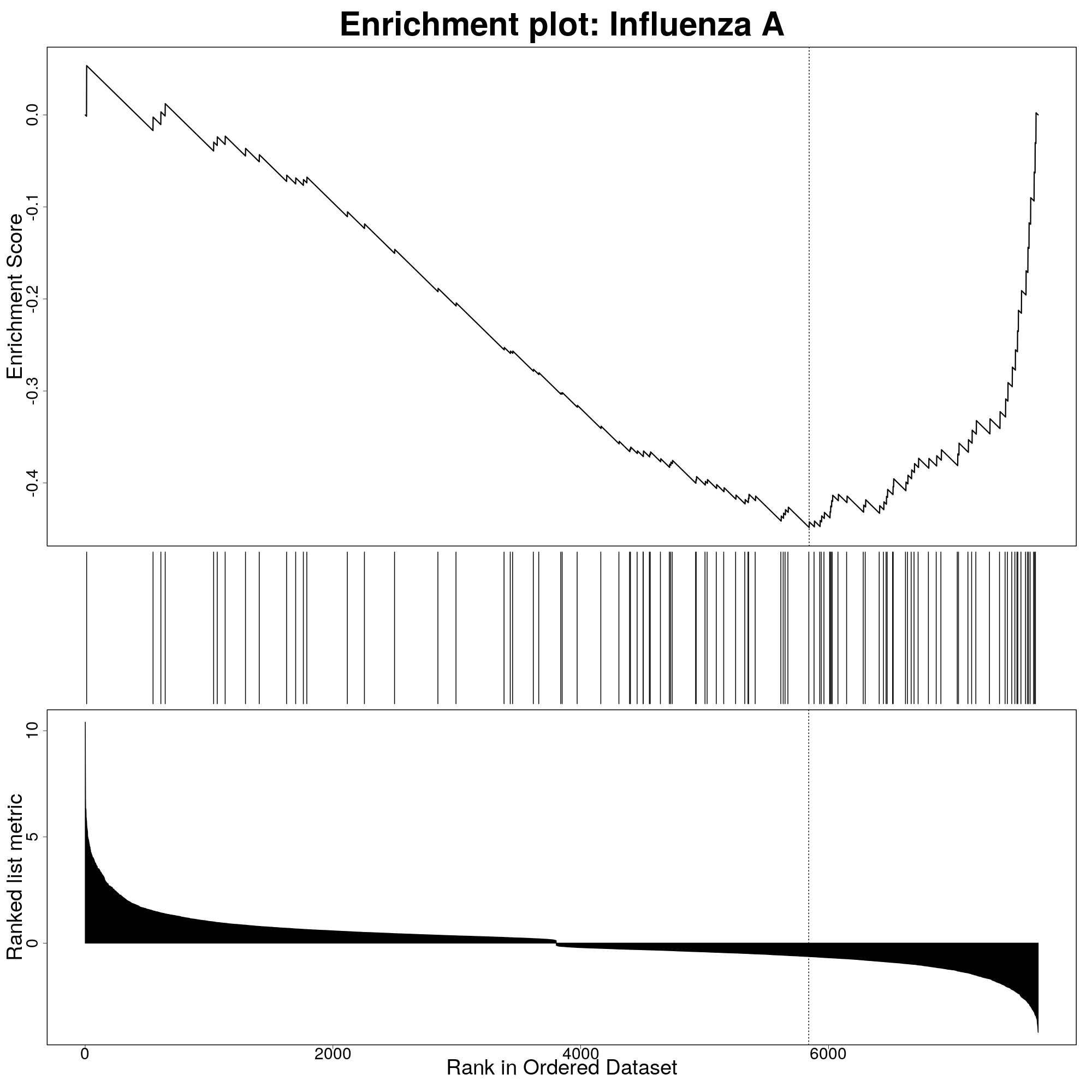

Supplement: Supplementary file 15 [file DataSheet_7.zip › Supplementary data 7 GSEA CCR2lo vs CCR2hi in CIA/Project_high_vs_low_GSEA/mmu05164.png]

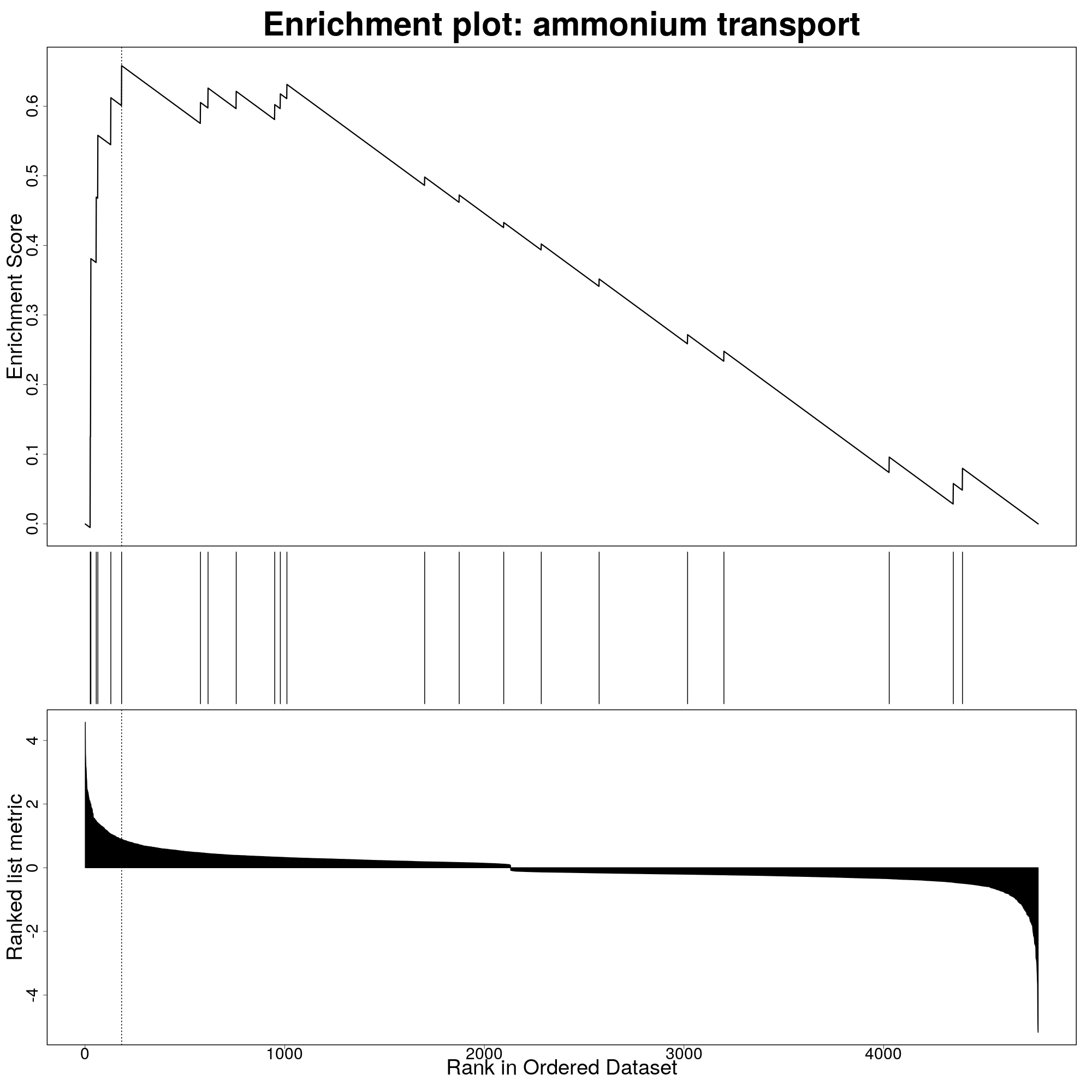

Supplement: Supplementary file 16 [file DataSheet_8.zip › Supplementary data 8 GSEA CIA vs CTRL all samples/Project_CIA_vs_control_GSEA/GO_0015696.png]

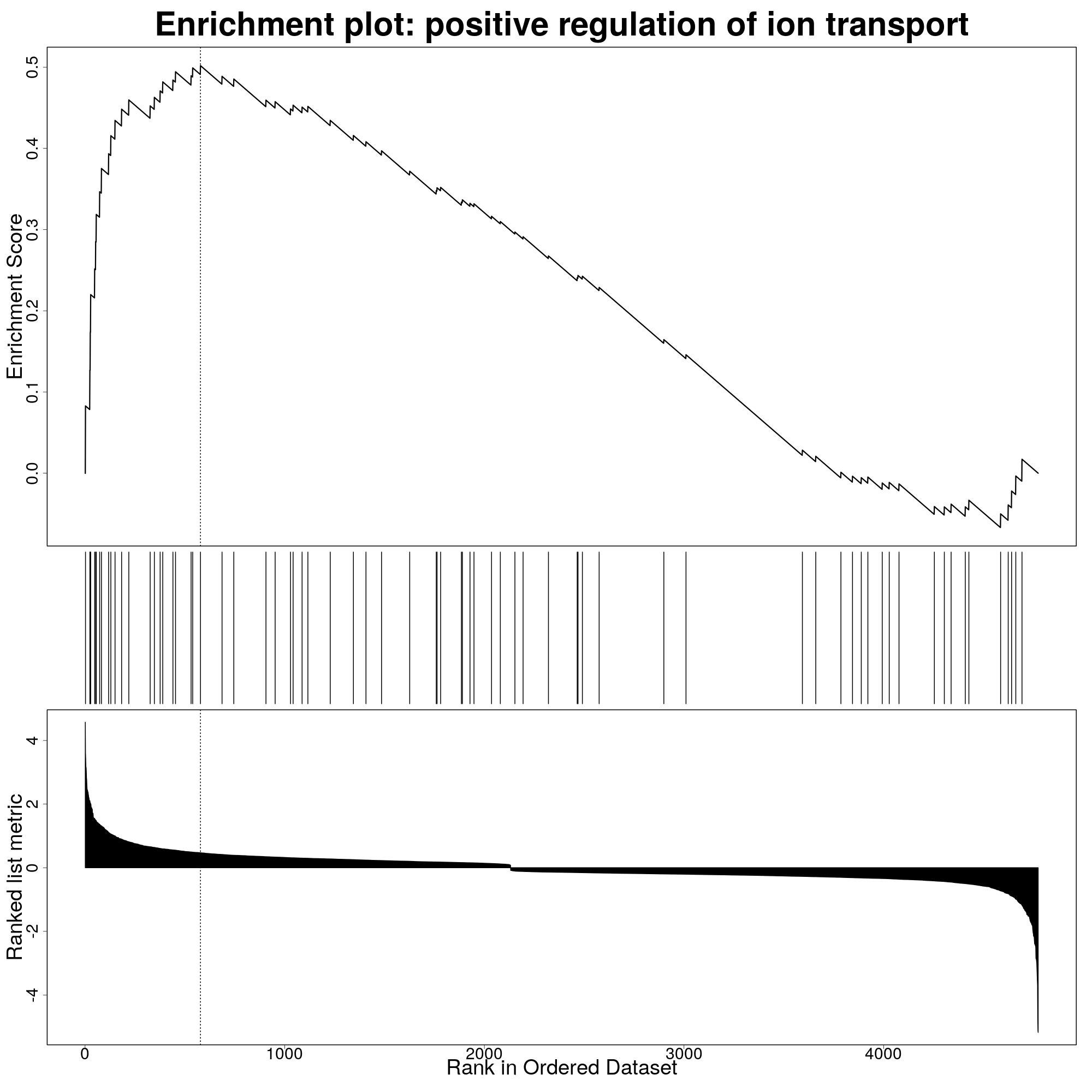

Supplement: Supplementary file 16 [file DataSheet_8.zip › Supplementary data 8 GSEA CIA vs CTRL all samples/Project_CIA_vs_control_GSEA/GO_0043270.png]

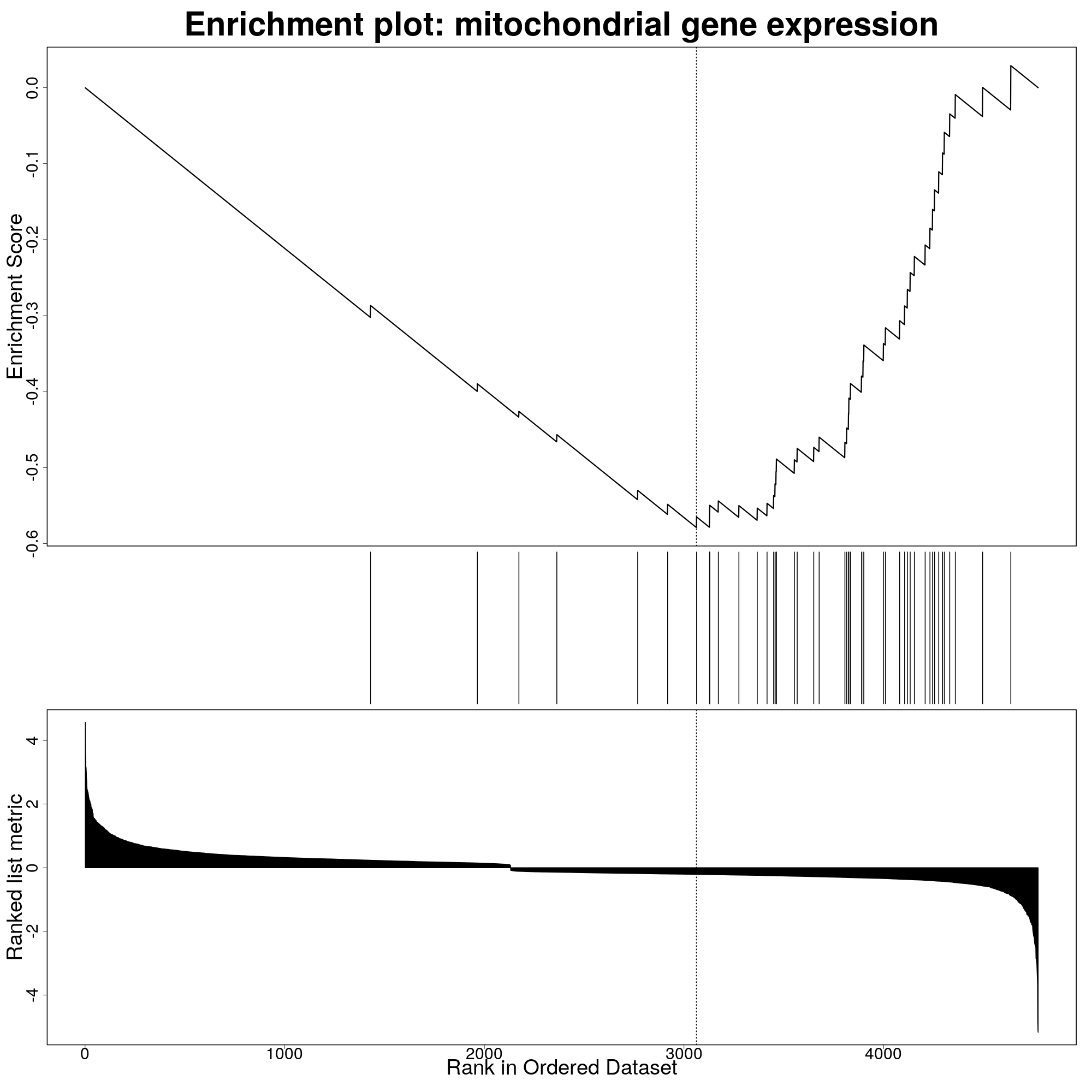

Supplement: Supplementary file 16 [file DataSheet_8.zip › Supplementary data 8 GSEA CIA vs CTRL all samples/Project_CIA_vs_control_GSEA/GO_0140053.png]

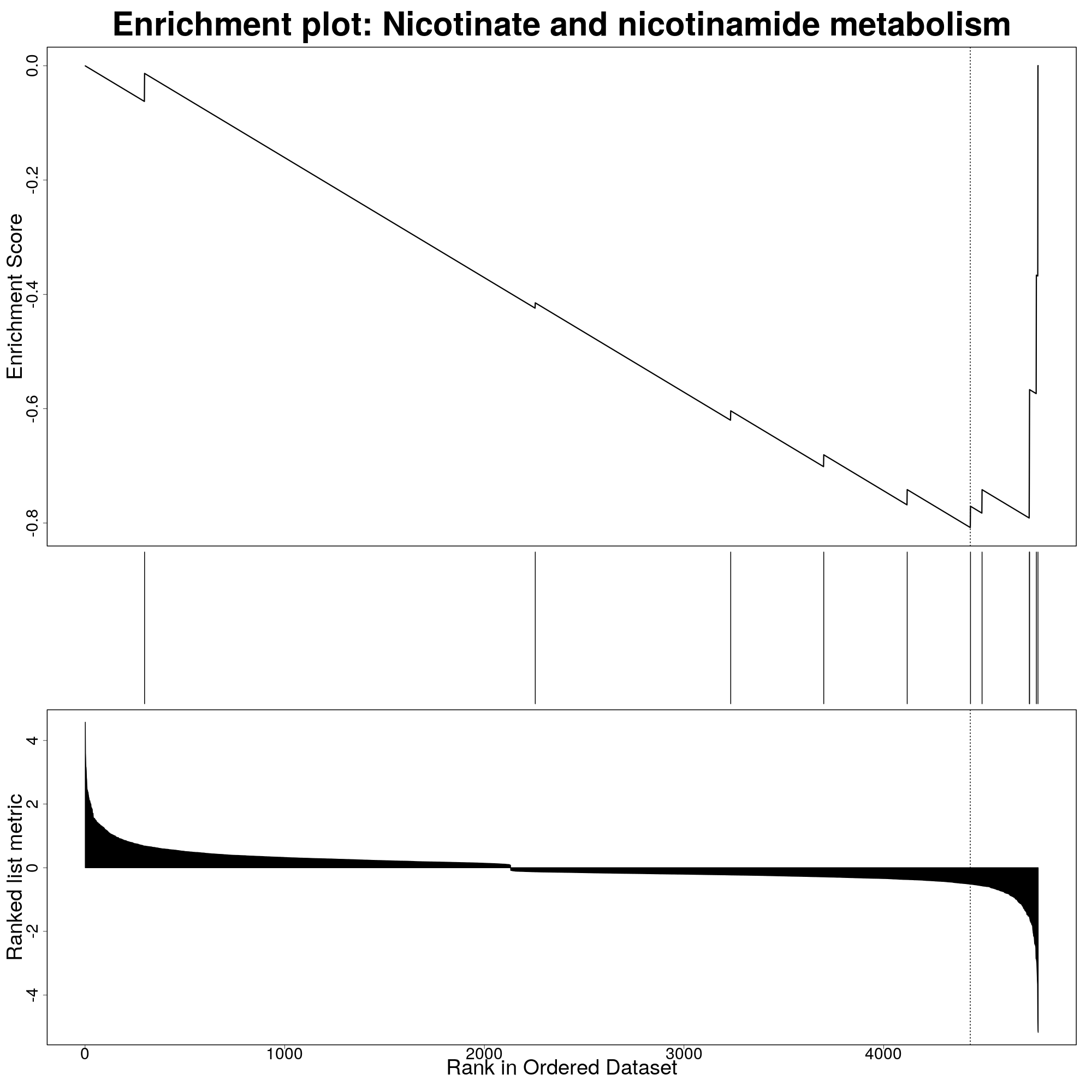

Supplement: Supplementary file 16 [file DataSheet_8.zip › Supplementary data 8 GSEA CIA vs CTRL all samples/Project_CIA_vs_control_GSEA/mmu00760.png]

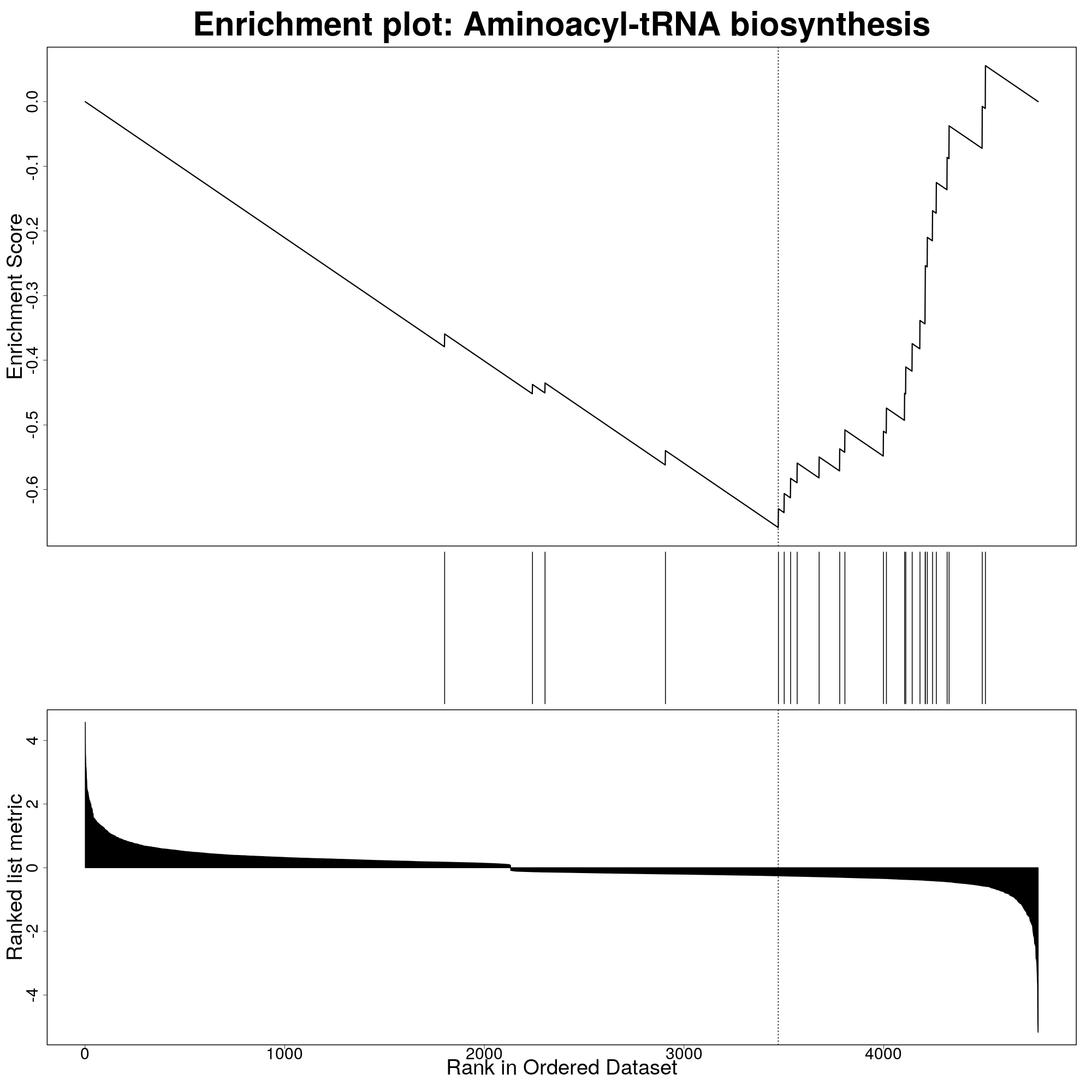

Supplement: Supplementary file 16 [file DataSheet_8.zip › Supplementary data 8 GSEA CIA vs CTRL all samples/Project_CIA_vs_control_GSEA/mmu00970.png]

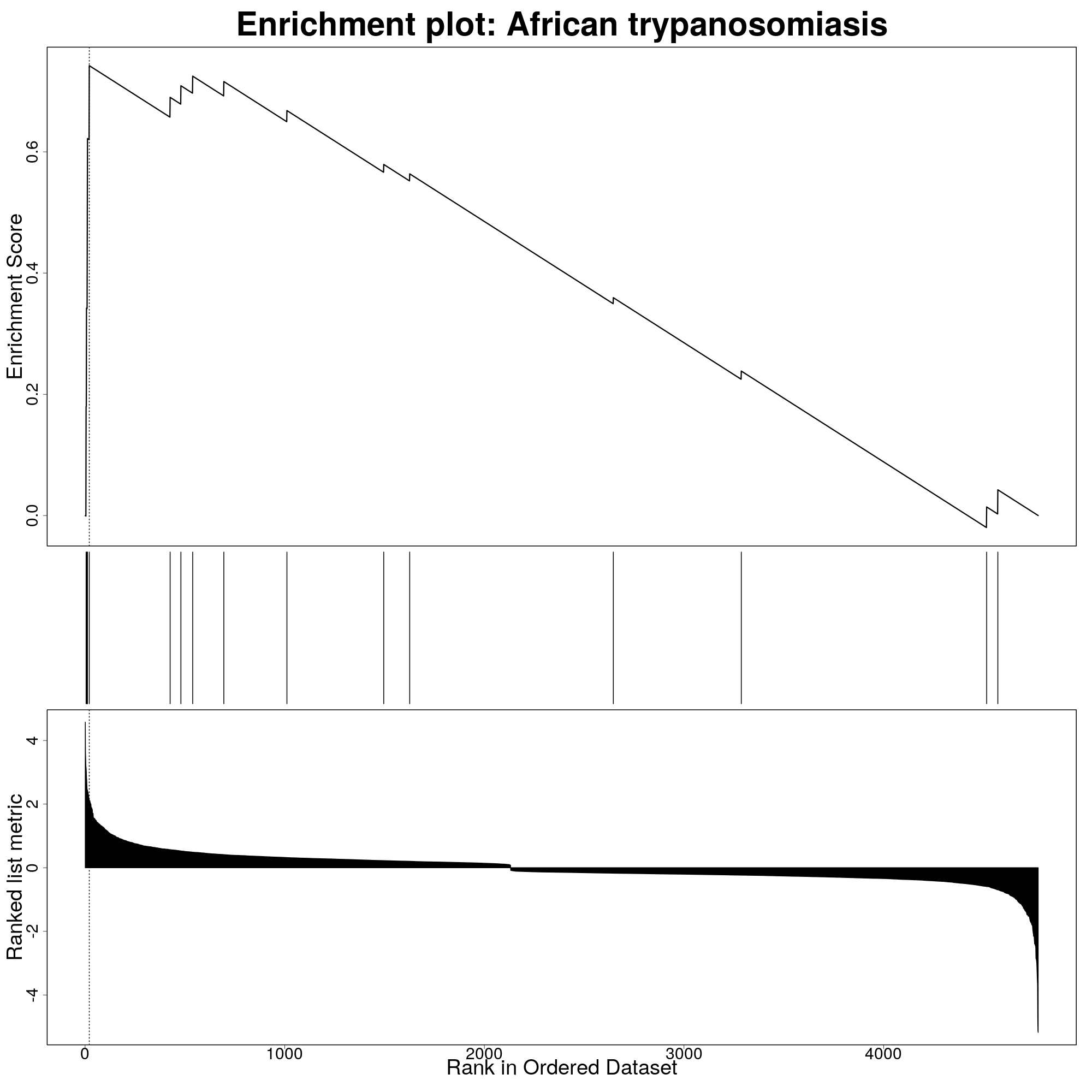

Supplement: Supplementary file 16 [file DataSheet_8.zip › Supplementary data 8 GSEA CIA vs CTRL all samples/Project_CIA_vs_control_GSEA/mmu05143.png]

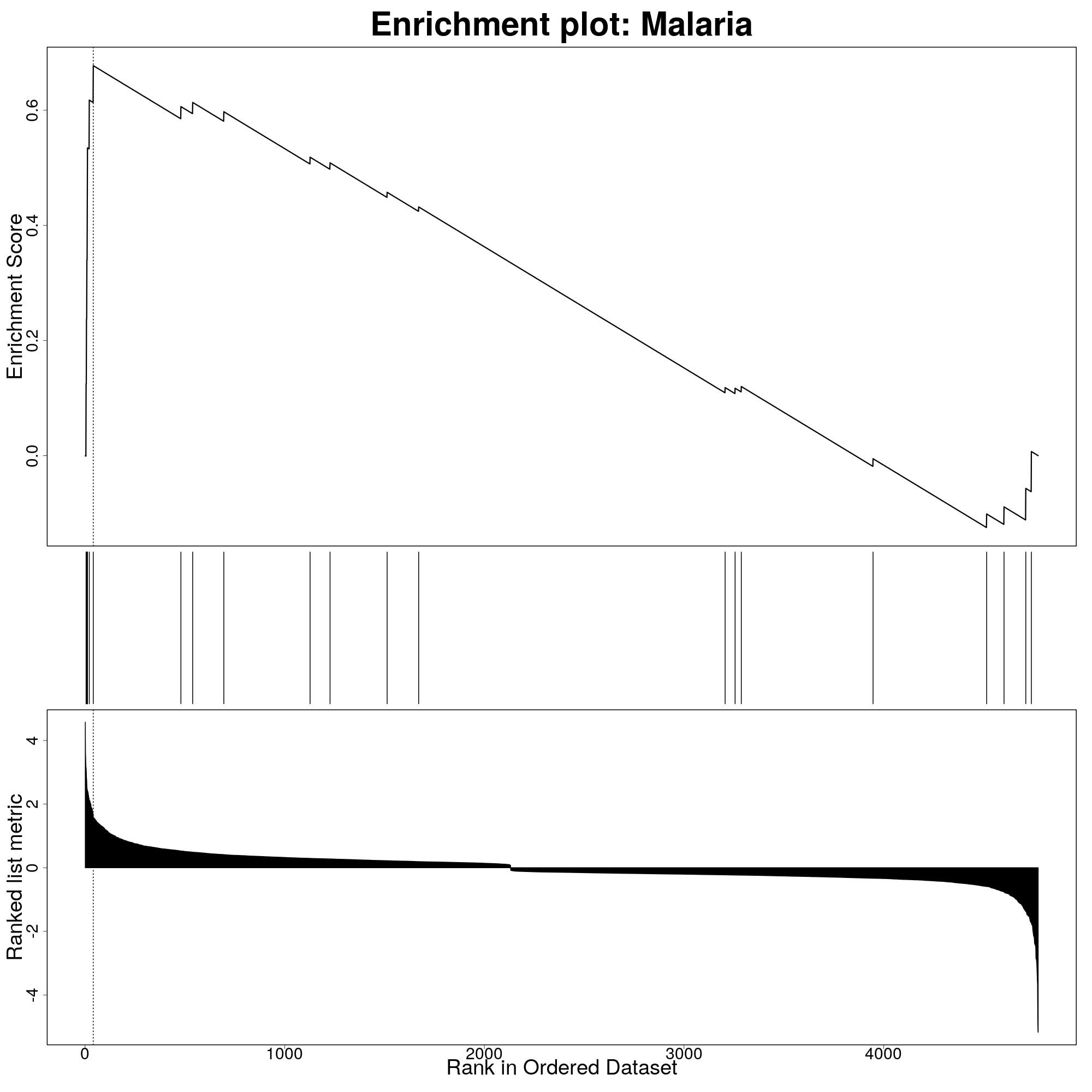

Supplement: Supplementary file 16 [file DataSheet_8.zip › Supplementary data 8 GSEA CIA vs CTRL all samples/Project_CIA_vs_control_GSEA/mmu05144.png]
